# Assessment of adaptive evolution between wheat and rice as deduced from full-length common wheat cDNA sequence data and expression patterns

**DOI:** 10.1186/1471-2164-10-271

**Published:** 2009-06-18

**Authors:** Kanako Kawaura, Keiichi Mochida, Akiko Enju, Yasushi Totoki, Atsushi Toyoda, Yoshiyuki Sakaki, Chikatoshi Kai, Jun Kawai, Yoshihide Hayashizaki, Motoaki Seki, Kazuo Shinozaki, Yasunari Ogihara

**Affiliations:** 1 Kihara Institute for Biological Research, Yokohama City University, Maioka-cho 641-12, Yokohama 244-0813, Japan; 2 RIKEN Plant Science Center, 1-7-22, Suehiro-cho, Yokohama 230-0045, Japan; 3 RIKEN Genomic Science Center, 1-7-22, Suehiro-cho, Yokohama 230-0045, Japan

## Abstract

**Background:**

Wheat is an allopolyploid plant that harbors a huge, complex genome. Therefore, accumulation of expressed sequence tags (ESTs) for wheat is becoming particularly important for functional genomics and molecular breeding. We prepared a comprehensive collection of ESTs from the various tissues that develop during the wheat life cycle and from tissues subjected to stress. We also examined their expression profiles *in silico*. As full-length cDNAs are indispensable to certify the collected ESTs and annotate the genes in the wheat genome, we performed a systematic survey and sequencing of the full-length cDNA clones. This sequence information is a valuable genetic resource for functional genomics and will enable carrying out comparative genomics in cereals.

**Results:**

As part of the functional genomics and development of genomic wheat resources, we have generated a collection of full-length cDNAs from common wheat. By grouping the ESTs of recombinant clones randomly selected from the full-length cDNA library, we were able to sequence 6,162 independent clones with high accuracy. About 10% of the clones were wheat-unique genes, without any counterparts within the DNA database. Wheat clones that showed high homology to those of rice were selected in order to investigate their expression patterns in various tissues throughout the wheat life cycle and in response to abiotic-stress treatments. To assess the variability of genes that have evolved differently in wheat and rice, we calculated the substitution rate (*Ka/Ks*) of the counterparts in wheat and rice. Genes that were preferentially expressed in certain tissues or treatments had higher *Ka/Ks *values than those in other tissues and treatments, which suggests that the genes with the higher variability expressed in these tissues is under adaptive selection.

**Conclusion:**

We have generated a high-quality full-length cDNA resource for common wheat, which is essential for continuation of the ongoing curation and annotation of the wheat genome. The data for each clone's expression in various tissues and stress treatments and its variability in wheat and rice as a result of their diversification are valuable tools for functional genomics in wheat and for comparative genomics in cereals.

## Background

Wheat is mainly cultivated in temperate zones and is one of the world's main staple foods. Wheat is polyploid and common wheat is an allohexaploid that has three homoeologous genomes. These genomes have been designated as A, B and D, with the coding regions of the homoeologous genes sharing more than 90% homology. Due to the huge genome size of the hexaploid (17 Gbp) [1] along with the high content of repeat sequences, it is very difficult to carry out complete sequencing of the entire genome or to perform forward genetics in polyploid wheat. Although common wheat genetic maps with molecular markers and cytological maps with deletion mutants of the chromosome segments have been constructed [2,3], the number of mapped DNA markers for both map-based cloning and anchoring of the genome positions remains restricted. Comparison of wheat and rice genomic sequences to corresponding full-length cDNAs can provide information on exon-intron boundaries. With this information, it should be possible to set up primers for PCR-based markers such as the simple-sequence repeats [4]. An international effort has been made to determine the DNA sequence of the entire chromosome of group 3 in bread wheat and in its ancestor, *Aegilops tauschii *(the D genome donor) [5]. However, transformation of wheat is still difficult, and tagged lines with transposable elements or T-DNA are not yet available. While tools for forward genetics have proven insufficient for wheat, a recent report [6] suggested that RNA interference can suppress the action of three homoeologous genes, even in polyploid wheat. Therefore, reverse genetic approaches based on functional genomics might be quite useful. We thus have been collecting expressed sequence tags (ESTs) from single limited strains of common wheat [7,8]. Simultaneous efforts from both our own and other laboratories have generated data on more than one million wheat ESTs . With the recent innovations that have been developed for the systematic collection and *in silico *display of comprehensive ESTs from a number of tissues, including abiotic-stressed tissues [8], it has become possible to characterize the expression profiles of target genes in these particular tissues and treatments.

Sequence-verified full-length cDNA clones with high accuracy that harbor protein coding sequences are critical for advances in structural, functional and comparative genomic studies. Using full-length cDNA sequence data, the protein-coding regions in the genome can be precisely predicted. In the wheat genome, gene annotation using full-length cDNA sequence data is essential, because there are many repetitive sequences and retrotransposons that can cause confusion when trying to predict the gene regions in genomic sequences [see examples in reference [9]]. In addition, functional annotation should be more reliable for converted amino acid sequence (coding sequence or CDS) predicted from full-length cDNA sequence data. Employing CDS data should enable inference of functional roles based on gene ontology from model plants such as rice and *Arabidopsis *(InterPro: ) [10].

CDS data can also be used to search for counterparts in related plant species. Wheat and rice both belong to the grass family, the Gramineae, and CDS data can reveal characteristic breeding behavior and the ability to adapt to environmental conditions that led to their diversification more than 50 million years ago [11]. Wheat, which originated in temperate zones, grows on dry land, is a long-day plant, has a shortened rachis, accumulates gluten-rich flour, and its germination is promoted in response to cold temperatures [12]. In contrast, rice, which is cultivated in tropical or subtropical areas, grows in water-rich conditions, is a short-day plant, has an elongated rachis, accumulates storage proteins via two types of protein bodies, and its germination is promoted in response to high temperatures [13]. Although it has long been theorized that selection pressure controls the distinct growth habits of wheat and rice, there has been no systematic investigation on the evolutionary rates of change for a genome-wide set of growth habit-related genes for these plants. The main reason for this is that DNA sequence information for the wheat genome is still limited.

Here we report on the construction of a full-length cDNA library for Chinese Spring wheat and its resulting quality as a starting resource for the complete sequencing of 6,162 independent full-length cDNA clones. We describe the overall characteristics of these full-length cDNA clones and their annotation compared to other model plants. From these clones, we selected 3,487 genes for which expression patterns could be traced with EST data in 28 tissues that are either observed during the wheat life cycle or were also stress-treated [7,8]. Based on hierarchical cluster classification of expression pattern, the substitution rates (*Ka/Ks*) of genes common to wheat and rice were calculated in order to assess the variability of the gene grouping in each cluster. Genes preferentially expressed in certain tissues or stress treatments showed higher *Ka/Ks *values, suggesting that molecular selection occurred during the diversification of wheat and rice.

## Results and Discussion

### Sequence determination and functional annotation of full-length wheat cDNAs

**Figure 1:**
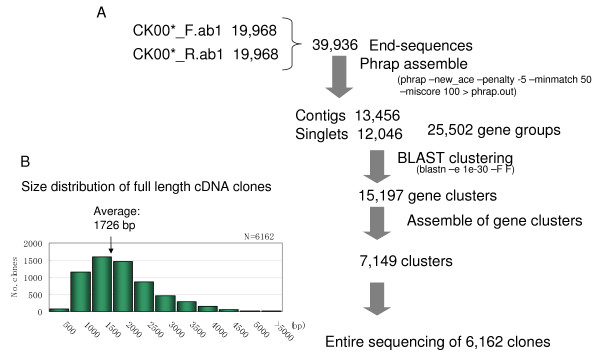
**Selection and sequence determination of cDNA clones from full-length cDNA library**. A total of 19,968 full-length cDNA clones of common wheat were sequenced from both ends. End sequences were assembled with the Phrap method. The assembled sequences were then clustered with the blastn method. These contigs might correspond to homoeologues. Subsequently, these contigs were grouped into independent gene clusters with the blastn method. Finally, the longest sequences from each gene cluster were selected for complete sequencing.

**Figure 2:**
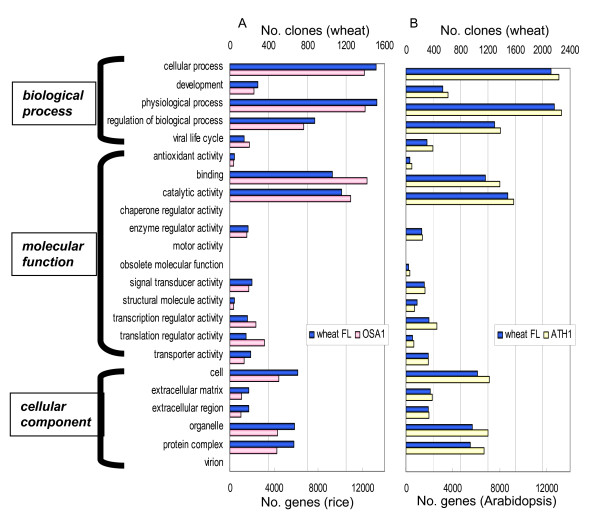
**Gene ontology of full-length wheat cDNAs**. Functions of the 6,162 full-length wheat cDNAs were estimated using gene ontology [10]. Distribution patterns of genes classified into each category were compared for wheat and rice (A), and for wheat and *Arabidopsis *(B). FL, full-length.

The CAP-trapper method [14,15] was used to construct a full-length cDNA library from pooled RNAs derived from the common wheat (*Triticum aestivum *cv. Chinese Spring). The library included 17 tissues that are either formed during the wheat life cycle or were subjected to an abiotic stress (Table 1). A total of 19,968 randomly selected recombinant clones from the full-length cDNA library have been sequenced from both ends. By grouping these one-path sequences, we obtained 7,149 independent gene clusters, which made it possible to group the homoeogenes expressed from each of the three common wheat genomes (A, B and D genomes) [16]. The largest clones were representative of each gene cluster and harbored the CDSs. After they were rearrayed, we were able to determine the full-length sequences of 6,162 clones. Figure 1 presents a detailed description of the sequence data for the full-length cDNA clones and the size distribution of the inserted DNAs. The full-length wheat cDNA data were used to carry out a blast homology search versus the public DNA database. About 10% of the clones were determined to be wheat-specific genes without any counterparts in the DNA database. Molecular functions of these sequenced cDNAs were predicted using the InterPro database [10]. The distribution of the wheat genes that were classified into various categories of the InterPro database was similar to that for rice and *Arabidopsis*. These results indicate that the selection of full-length wheat cDNAs from the pooled RNAs reflects their expression within the tissues (Figure 2).

**Table 1:** Wheat tissues used for construction of the full-length cDNA library

No. cDNA library	Tissue	Stage or treatment
1	Young spikelet	Early flowering stage
	Young spikelet	Late flowering stage
	Young spikelet	Booting stage
	Whole grains	5 DPA*
	Whole grains	10 DPA
	Whole grains	15 DPA
	Whole grains	20 DPA
	Whole grains	30 DPA
	Whole grains	50 DPA
	Spike	Heading date
	Spike	Flowering date
	Seedling	Heat shocked**
	Seedling	Salt-stressed***
	Seedling	Dehydrated^+^
	Seedling	-
	Seedling	Vernalized^++^
	Whole plant	Booting stage

*DPA: Days post-anthesis.

**Wheat seedlings were grown for 14 days after germination. 14-day-old wheat seedlings were incubated at 42°C for 1 h before RNA extraction.

***14-day-old wheat seedlings were treated with 150 mM NaCl solution for 12 h.

^+^14-day-old wheat seedlings were dehydrated on filter paper for 2 days.

^++^14-day-old wheat seedlings were grown at 4°C for 16 days.

### Comparison of gene nucleotide substitution rates in coding regions in wheat and rice

**Figure 3:**
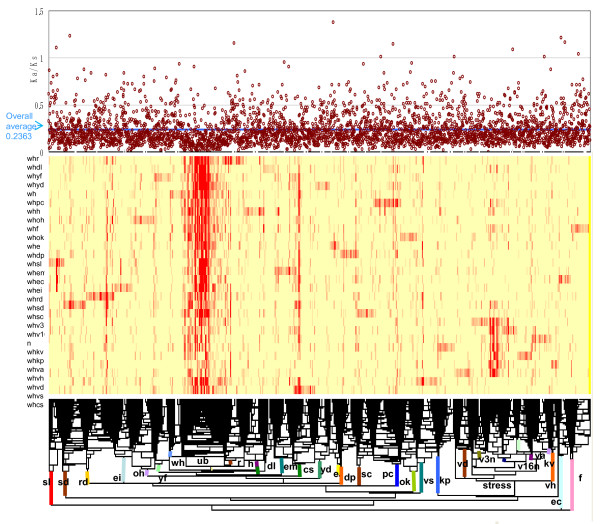
**Expression profiles of the 3,487 full-length wheat cDNA genes in 28 tissues or treatments and their *Ka/Ks *values**. Relative gene expression is indicated by color intensity, which depends upon the number of EST constituents. The contigs orthologous to the 3,487 full-length cDNAs were selected from the wheat cDNA libraries constructed by the 28 tissues or treatments. Similarities of gene expression patterns among cDNAs and tissues or treatments were estimated using Pearson's correlation coefficient. Hierarchical clustering [25] was applied in order to compare EST expression profiles among the 28 tissues and treatments. Color scale ranges from 0 members to 414 members in EST contstituents. The calculated *Ka/Ks *values for the wheat and rice homologues are plotted above the expression profile for each gene. For abbreviations of tissues and treatments, see Table 2.

To compare the gene nucleotide substitution rates of wheat and rice, we selected 4,321 wheat genes that had deduced amino acid sequences with more than 80% overlapping homology as determined by the blastx method. In contrast, when the homology search was carried out against the wheat EST database using selected rice genes as the query, the corresponding wheat genes had the highest ranking. In order to examine the expression profiles in various tissues of wheat, including abiotic-stressed tissues, we used blastn (E < 1e-60) to search the wheat EST database (MUGEST: ) for corresponding ESTs of the 4,321 wheat cDNAs described in references 7 and 8. We selected 3,487 wheat genes to compare their amino acid sequences in wheat and rice (Additional file 1). In addition, we were also able to trace their expression profiles in 28 normal or stress-treated tissues of common wheat (Table 2). Since the most common way to estimate selection constraints on protein evolution is using the ratio of the nonsynonymous (*Ka*) and synonymous (*Ks*) substitution rates [17], we calculated the *Ka/Ks *ratio for each gene to compare the values for the wheat and rice counterparts. The *Ka/Ks *value varied from 1.3 to 0, with the average being 0.2363 (Figure 3). The likely occurrence of adaptive evolution for a given gene is indicated by a *Ka/Ks *value greater than 1 [17]. Out of the 3,487 wheat genes, 12 had *Ka/Ks *values greater than 1. Although the identity of most of these 12 genes is yet unknown, five have been annotated, including one gene as involved in lipid transfer, one with homology to heat shock protein HSP20, one to proteinase inhibitor, one to thionin and one to an RNA binding protein. Genes for lipid transfer proteins and HSP20 have been demonstrated to be involved in stress responses, such as plant defense [18] and heat shock [19]. A proteinase inhibitor gene has been reported to regulate plant development [20] and drought tolerance [21]. Thionin genes are specifically expressed in the seed and control plant defense against *Fusarium *head blight [22,23]. RNA binding protein also has a role in both the development of flowers and seeds [24]. Taken together, this evidence suggests that positive selection may have a role for beneficial functions of these genes. Therefore, to some extent these genes characterize the adaptation that must occur in order for growth to continue within the specific habitats for wheat and rice.

**Table 2:** Wheat tissues and treatments selected to evaluate expression patterns of full-length cDNAs using ESTs homologous to their gene counterparts

Abbreviation for tissue or treatment	Tissue or treatment with which EST analysis was carried out
r	Root at 14 days old
dl	Crown of 14-day-old seedling
yf	Spikelet at early flowering stage (3–5 mm)
yd	Spikelet at late flowering stage (5–10 mm)
o	Spike at meiosis (booting stage)
pc	Anther at meiosis
h	Spike at heading date
oh	Pistil at heading date
f	Spike at flowering date
ok	Developing seed 5 DPA*
e	Developing seed 10 DPA
dp	Developing seed 20 DPA
sl	Developing seed 30 DPA
em	Dormant seed after water absorption
ec	Dormant seed with cold treatment after water absorption
ei	Dormant seed with water absorption after wounding
rd	Root of desiccated 14-day-old seedling
sd	Shoot of desiccated 14-day-old seedling
sc	14-day-old seedling with 24 h cold treatment at 4°C at day 13
v3n	14-day-old seedling grown for 3 days at 4°C at day 11
v16n	14-day-old seedling grown for 16 days at 4°C
kv	14-day-old seedling grown under continuous light after 24 h cold treatment at 4°C
kp	14-day-old seedling grown under continuous light
va	14-day-old seedling treated with abscisic acid
vh	14-day-old seedling treated with heat shock
vd	Shoot grown with desiccation
vs	Liquid cultured tissue
cs	Callus

*DPA: Days post-anthesis.

### Comparative and functional genomic analysis of full-length wheat cDNAs

In order to examine nucleotide substitution rates of the genes in relation to their expression patterns for the various tissues and stress treatments, global expression profiles of the 3,487 full-length cDNA genes in the 28 tissues and treatments were estimated using the full-length cDNAs as scaffolds to cluster ESTs. Expression patterns of the clustered wheat ESTs (contigs) have been previously reported [7,8]. The number of EST constituents assigned to the 28 cDNA libraries was scored for each contig, which produced a two-way expression profile, i.e., contig vs. library. As seen in Figure 3, hierarchical clustering was constructed based on the EST constituent matrix [25]. To determine the counterparts of the 3,487 full-length cDNAs in the wheat ESTs, we performed a search with blastn (E < 1e-60) against the contigs [7,8]. Based on the expression patterns of their counterpart contigs after hierarchical clustering, the 3,487 genes were classified into 30 major groups. In addition to the genes that were ubiquitously or nearly ubiquitously expressed in the 28 tissues and treatments (designated as ubiquitous or "ub"), we also classified the genes that were dominantly expressed in each of these 28 tissues and treatments (Figure 3 and Table 2). Out of the 28 cDNA libraries, 13 were assigned to a life cycle group, while 15 were assigned to the stressed-tissue group (Figure 3) [8]. During the clustering, a group was found that showed dominant expression in all of the stress-treated tissues (Figure 3). These genes were designated stress constant or "strc". The number of genes classified into each category ranged from 44 (heat shock) to 182 (dried roots), with a mean of 109.9.

**Figure 4:**
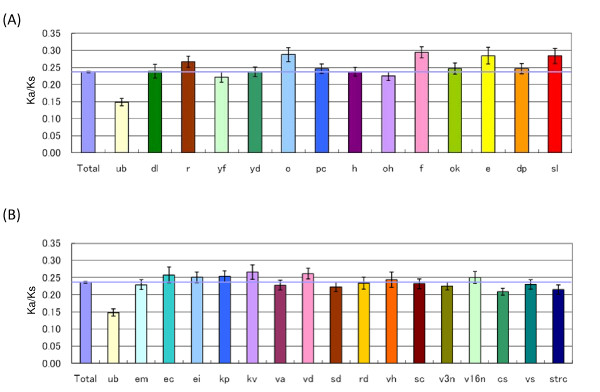
**Mean *Ka/Ks *values in various wheat tissues either untreated or subjected to stress treatment**. Mean *Ka/Ks *values were calculated for each of 28 wheat tissues formed during the wheat life cycle (A) or subjected to stress treatment (B). For abbreviations of tissues and treatments, see Table 2. The blue horizontal line indicates the overall average of the mean *Ka/Ks *values from tissues.

In order to assess the relationship between the variability of the genes and their expression profiles, the *Ka/Ks *value of each gene was plotted against its expression pattern (listed above the expression profile of each contig in Figure 3). The mean *Ka/Ks *values for the genes classified in each group are shown in Figure 4. In addition, the *Ka/Ks *values of the life cycle and stressed-tissue groups are respectively displayed in Figure 4A and 4B. Analysis of variance indicated a significant difference in the *Ka/Ks *value between the groups. Notably, the *Ka/Ks *values of the "ub" and "strc" genes were significantly lower than the average of the 3,487 genes, which suggests a selective constraint on these subsets of the genes [26]. Genes preferentially expressed in the callus (Figure 4B) also had significantly lower *Ka/Ks *values than the average. On the other hand, the 35 genes expressed in six tissues and in the stressed tissues, namely, root (r), the spike at booting (o), the spike at flowering (f), the developing seed 30 days post-anthesis (DPA) (sl), seedlings subjected to a cold treatment (kv) and desiccated seedlings (vd), all had significantly higher mean *Ka/Ks *values compared to the overall average (Figure 4A, B). This shows that there was a loose selection constraint against these genes during the evolutionary divergence of wheat and rice. Table 3 lists the annotated genes that displayed higher *Ka/Ks *values (greater than 0.5) for the genes expressed in these six tissues and treatments (for further explanation, see Additional file 2). These six tissues and treatments characterize the growth habit or speciation requirements of wheat and rice. For example, rice roots are usually covered by water, which creates anaerobic conditions around the roots [27]. In contrast, since wheat plants prefer dry land, anaerobic conditions do not occur around the wheat roots [28]. Among the genes that display higher *Ka/Ks *values, genes encoding transcription factors [29,30], beta-glucanase [31] and nodulin [32], which are regulated by abiotic as well as biotic stresses, were found. Additionally, radial oxygen loss is a critical marker for adaptation of roots to anaerobic conditions [33,34]. Two genes related to radial oxygen loss had higher *Ka/Ks *values (Table 1 and Additional file 2). The evidence accumulating from these observations suggests that genes expressed in the roots that are related to signal perception or transduction, transcription regulation, or stress responses have all been exposed, to some extent, to the adaptive selection that occurred during the diversification of wheat and rice.

**Table 3:** Tissues and genes that showed higher variation as a result of wheat and rice diversification

Tissue or treatment	No. of genes*	Function controlled by gene
Root	16 (6)	Signal response related to environmental stress (2 genes)
		Redox in response to stimuli (2 genes)
		Transcription factors regulated by stress (2 genes)
Spike at booting stage	12 (4)	Lipid-related proteins modulated in developing seeds (2 genes)
		Redox in photosynthesis and respiration (2 genes)
Spike at flowering	15 (11)	Stress related (4 genes)
		Nucleic acid binding protein (2 genes)
		Signal transduction (1 gene)
		Epigenetic regulation (1 gene)
		Tissue or stage specific proteins (2 genes)
		Proteinase inhibitor (1 gene)
Seed at 30 DPA**	16 (10)	Plant stress defense proteins (6 genes)
		Stress-response proteins expressed during seed maturation (3 genes)
		Tissue or stage specific protein (1 gene)
Seedling with cold treatment	9 (4)	Stress related (2 genes)
		Polysaccharide-related protein (1 gene)
		Lipid-related protein (1 gene)
Desiccated seedling	9 (4)	Chaperon (1 gene)
		Stress related (1 gene)
		Senescence related (1 gene)
		Photosystem II related (1 gene)

Ubiquitous***	25 (25)	Ubiquitin, tubulin subunits, proteasome subunits, histones, translation-related proteins, signal transduction-related protein ATPase

*Number of genes showing *Ka/Ks *ratio higher than 0.5 are listed. In the case of ubiquitous genes, only those with a ratio less than 0.05 are listed. The number of genes annotated are given in parentheses.

**DPA: Days post-anthesis.

*** Ubiquitous genes are also listed for reference.

The spike at the booting stage of both wheat and rice, during which the critical process of meiosis occurs, generates the gametes [35,36]. Among the four annotated genes in this tissue, two genes are related to lipid metabolism for cellular activity [37,38] and two genes are associated with redox reactions in photosynthesis [39] and respiration [40]. All of these had relatively higher *Ka/Ks *values (Table 3 and Additional file 2), suggesting that the spike at the booting stage is under highly stressed conditions for dry (wheat) and moist (rice) habitats.

The spike that occurs at flowering supplies the field for pollination. Thus, genes that control compatibility for fertilization need to work at this particular stage [41]. Among the genes that were preferentially expressed at this stage, the RNA binding protein showed *Ka/Ks *values that were higher than 1, which suggests a key role in the pollination by the spikes during flowering [24]. The DNA binding histone-like protein that is specifically expressed in this tissue has a higher *Ka/Ks *value. Additionally, a methyltransferase involved in epigenetic regulation has highly diverged. Certain stress-related proteins such as glutathione S-transferase [42], osmotin [43,44], pectate lyase [45] and glucan endo-1,3-beta-D-glucosidase [46] were also characteristically expressed in this tissue. Likewise, a gene encoding a receptor-like kinase is also uniquely expressed in this tissue. In addition, genes that encode pollen allergen-related protein [47,48] and proteinase inhibitor [49] were expressed in the spike at flowering. All of these data suggest that selection for recognition between male and female gametes, modulation of nucleic acids, stress responses and signal transduction in the field during pollination might operate on these genes.

In response to internal and external environments, developing seeds at 30 DPA enter into dormancy [50,51]. Genes for thionin 1 and 2, polysaccharide-related proteins, lipid transfer protein and ankyrins are all involved in controlling plant defense at this stage (Additional file 2). Stage-specific proteins that play substantial roles in the seeds, such as late embryo abundant proteins I and II [52], stress-related protein [53] and α-amylase [54], all showed higher *Ka/Ks *values during this part of the life cycle. This suggests that genes for signal perception during seed dormancy, along with those for stage-specific and stress-related conditions, might characterize the growth habits of wheat and rice in situations where they are adapting to environmental conditions.

Genes expressed in cold-treated and desiccated seedlings showed higher *Ka/Ks *values than the others. Genes found to be involved after these treatments are normally associated with cell membranes, cell walls, stress and senescence.

It is assumed that adaptive selection works on a large number of genes, some of which show rapid evolution. Unfortunately, in genes that have rapidly evolved, there is great divergence in the plants that have resulted from genetic divergence, which makes it difficult to trace their orthology. In the current study, after deducing the complete sequence of full-length cDNA clones, we compared coding sequences of common wheat genes to their rice counterparts. The expression patterns in various tissues during the wheat life cycle as well as in stress-treated tissues can be systematically monitored [8]. Thus, based on mean *Ka/Ks *values, it may be possible to infer the variability of genes in their sequences that will be expressed in their respective tissues or treatments to the plants. Genes ubiquitously expressed throughout the majority of the tissues or stress treatments exhibited lower *Ka/Ks *values, whereas significantly higher *Ka/Ks *values were noted both for the genes that were characteristically expressed in four tissues of the wheat life cycle (the root, the spike at the booting stage, the spike at flowering and the seed at 30 DPA), and for the genes expressed in response to the two stress treatments (seedlings undergoing cold treatment and desiccated seedlings). These findings of gene expression patterns in response to stresses are also reported for mammalian genes [26]. Therefore, to some extent, positive selection might play a role in the gene expression that occurs in response to environmental changes.

## Conclusion

We have developed a resource of a large number of sequenced full-length cDNAs of common wheat that covers the majority of the functional annotations deduced from gene ontology of rice and *Arabidopsis*. This full-length wheat cDNA resource is indispensable for gene annotations in the wheat genome for which sequencing is still ongoing, and for the functional analysis of these genes and their products. The information presented here on the full-length wheat cDNA sequences, their variability during the evolution of cereals and their expression profiles in various tissues during both the life cycle and in response to stress treatments should facilitate functional genomics and genome breeding of wheat and other cereals.

## Methods

### Construction of full-length wheat cDNA library, DNA sequencing, and selection of independent cDNA clones

The CAP-trapper method [14,15] was used to construct a full-length cDNA library from pooled RNAs derived from 17 samples of common wheat (*Triticum aestivum *cv. Chinese Spring) tissues, a combination of those formed during the wheat life cycle and those subjected to abiotic stresses (Table 1). Subsequently, 39,936 cDNA clones were randomly selected from the library and sequenced by a one-path method from both ends of the inserts. These DNA sequences were assembled with the Phrap method (University of Washington Genome Center; ) using the program new_ace-penalty-5 – mismatch 50 – minscore 100. To construct the gene clusters (E < 1e-30), the resultant contigs were clustered using the blastn method [55]. Representative clones from each gene cluster that was predicted to harbor CDSs were then rearrayed. Subsequently, 6,162 full-length cDNA clones were selected, and their inserts were completely sequenced using the primer walking method.

### Data mining and characterization of full-length wheat cDNA clones

The wheat cDNAs were annotated using the blast method against the public database (E < 1e-5). Rice counterparts for each of the wheat cDNAs were selected from the public database when a blastx search determined that there was more than 80% overlapping homology in the deduced amino acids. The CDSs of the counterparts were automatically aligned. The alignments were needed to be corrected manually. Then, the *Ka/Ks *values [17] were calculated for the codons that corresponded between the wheat and rice homologues.

cDNA expression patterns were monitored using a tissue expression map of wheat, which included 28 cDNA libraries (Table 2). The blastn program (with E < 1e-60) was then used to search the full-length cDNAs for their correct counterparts in the wheat tissue expression map, with the full-length cDNAs used as scaffolds to cluster ESTs. Similarities between the resultant 3,487 cDNAs and the libraries were estimated using Pearson's correlation coefficient [25]. Hierarchical clustering  was applied in order to compare these cDNAs expression profiles among the 28 wheat libraries. Expression profiles were displayed based on the number of constituents in the cDNAs along with their *Ka/Ks *value.

### Full-length cDNA data

The full-length cDNA data have been deposited in the DDBJ under accession nos. AK330135 to AK336296, and are available at .

## Competing interests

The authors declare that they have no competing interests.

## Authors' contributions

This study was conceived and directed by YO, MS and KS. The full-length cDNA library was constructed by AE, YT and CK under the guidance of JK and YH. Selection of cDNA clones and DNA sequencing were conducted by AE and YT with the help of KM, AT and YS. Data were analyzed by KK and KM. AT, YS, JK, YH, MS, KS and YO participated in the design and coordination of the study. All authors read and approved the final manuscript.

## Supplementary Material

Additional file 1 **Annotation of the 3407 full-length wheat cDNA genes and their *Ka/Ks *values**. The 3407 full-length wheat cDNA genes are annotated and their *Ka/Ks *values are calculated. Click here for file

Additional file 2 **Adaptational genes showing relatively faster evolution during diversification between wheat and rice**. Adaptational genes showing relatively faster evolution are listed and their annotation is given with their *Ka/Ks *values. Click here for file
