# Supplementary material for: Assessment of adaptive evolution between wheat and rice as deduced from full-length common wheat cDNA sequence data and expression patterns
Source: BMC Genomics. 2009 Jun 18;10:271. doi: 10.1186/1471-2164-10-271 (PMC2703658; doi:10.1186/1471-2164-10-271)
Supplement: Additional file 1 — Annotation of the 3407 full-length wheat cDNA genes and their Ka/Ks values. The 3407 full-length wheat cDNA genes are annotated and their Ka/Ks values are calculated. [file 1471-2164-10-271-S1.pdf]

PDF Complete
Your complimentary use period has ended. Thank you for using PDF Complete.
Click Here to upgrade to Unlimited Pages and Expanded Features

<table>
<tr><th></th><th>NAME</th><th>nr</th><th>rice_ver3</th><th>Ka/Ks</th><th>Ka,Ks</th><th>Ka</th><th>Ks</th></tr>
<tr><td>1</td><td>SET5_N14_CLUSTER_25419</td><td>gi|50908619|ref|XP_465798.1| unknown protein [Oryza sativa (japonica cultivar-group)] >gi|48716537|dbj|BAD23141.1| unknown protein [Oryza sativa (japonica cultivar-group)] e_value:6.87276e-58</td><td>11668.m02780|LOC_Os02g29320|protein expressed protein e_value:4.15478e-56</td><td>0.272</td><td>(0.1027 0.3780)</td><td>0.1027</td><td>0.378</td></tr>
<tr><td>2</td><td>WT008_I09_CLUSTER_33832</td><td>gi|50940751|ref|XP_479903.1| putative histidine amino acid transporter [Oryza sativa (japonica cultivar-group)] >gnl|BL_ORD_ID|1601531 gi|51964650|ref|XP_507109.1| PREDICTED OJ1163_G08.25 gene product [Oryza sativa (japonica cultivar-group)] >gnl|BL</td><td>11674.m00247|LOC_Os08g03350|protein Transmembrane amino acid transporter protein e_value:0</td><td>0.121</td><td>(0.0888 0.7358)</td><td>0.0888</td><td>0.7358</td></tr>
<tr><td>3</td><td>SET1_O04_CLUSTER_15404</td><td>gi|51090497|dbj|BAD35699.1| putative transmembrane protein Tmp21 precursor [Oryza sativa (japonica cultivar-group)] e_value:4.95791e-99</td><td>11680.m01046|LOC_Os06g10760|protein emp24/gp25L/p24 family e_value:1.13859e-100</td><td>0.182</td><td>(0.0654 0.3603)</td><td>0.0654</td><td>0.3603</td></tr>
<tr><td>4</td><td>WT003_L24_CLUSTER_34910</td><td>gi|51964432|ref|XP_507001.1| PREDICTED OJ1353_F08.16-1 gene product [Oryza sativa (japonica cultivar-group)] >gnl|BL_ORD_ID|1583134 gi|50913219|ref|XP_468017.1| 1-aminocyclopropane-1-carboxylate oxidase [Oryza sativa (japonica cultivar-group)] >gnl|</td><td>11668.m05217|LOC_Os02g53180|protein oxidoreductase, 2OG-Fe(II) oxygenase family, putative e_value:5.68689e-157</td><td>0.232</td><td>(0.0768 0.3317)</td><td>0.0768</td><td>0.3317</td></tr>
<tr><td>5</td><td>WT004__C24_CLUSTER_11834</td><td>gi|31430769|gb|AAP52635.1| nodulin-like protein [Oryza sativa (japonica cultivar-group)] >gnl|BL_ORD_ID|2009294 gi|37532092|ref|NP_920348.1| nodulin-like protein [Oryza sativa (japonica cultivar-group)] e_value:5.80994e-85</td><td>11686.m01886|LOC_Os12g18960|protein Integral membrane protein, putative e_value:3.107e-149</td><td>0.282</td><td>(0.2112 0.7503)</td><td>0.2112</td><td>0.7503</td></tr>
<tr><td>6</td><td>SET2_L08_CLUSTER_2537</td><td>gi|50943261|ref|XP_481158.1| unknown protein [Oryza sativa (japonica cultivar-group)] >gi|37805880|dbj|BAC99608.1| unknown protein [Oryza sativa (japonica cultivar-group)] e_value:4.63681e-23</td><td>11674.m01651|LOC_Os08g16810|protein expressed protein e_value:1.26712e-25</td><td>0.451</td><td>(0.3152 0.6981)</td><td>0.3152</td><td>0.6981</td></tr>
<tr><td>7</td><td>WT012_J21_CLUSTER_30927</td><td>gi|56202085|dbj|BAD73614.1| no apical meristem (NAM) protein-like [Oryza sativa (japonica cultivar-group)] >gnl|BL_ORD_ID|542467 gi|56201848|dbj|BAD73298.1| no apical meristem (NAM) protein-like [Oryza sativa (japonica cultivar-group)] e_value:7.77232e</td><td>11667.m04665|LOC_Os01g48130|protein No apical meristem (NAM) protein, putative e_value:4.76659e-151</td><td>0.185</td><td>(0.0937 0.5074)</td><td>0.0937</td><td>0.5074</td></tr>
<tr><td>8</td><td>WT005_A15_CLUSTER_6435</td><td>gi|50919199|ref|XP_469996.1| putative protease inhibitor [Oryza sativa (japonica cultivar-group)] >gnl|BL_ORD_ID|1587472 gi|41469392|gb|AAS07215.1| putative protease inhibitor [Oryza sativa (japonica cultivar-group)] e_value:1.96439e-58</td><td>11669.m05980|LOC_Os03g58940|protein putative protease inhibitor e_value:3.47335e-60</td><td>0.615</td><td>(0.2640 0.4295)</td><td>0.264</td><td>0.4295</td></tr>
<tr><td>9</td><td>WT012_F15_CLUSTER_17217</td><td>gi|50912019|ref|XP_467417.1| putative nuclear matrix constituent protein 1 [Oryza sativa (japonica cultivar-group)] >gnl|BL_ORD_ID|1580560 gi|32352206|dbj|BAC78596.1| hypothetical protein [Oryza sativa (japonica cultivar-group)] >gnl|BL_ORD_ID|15805</td><td>11668.m04659|LOC_Os02g48010|protein expressed protein e_value:0</td><td>0.214</td><td>(0.1460 0.6833)</td><td>0.146</td><td>0.6833</td></tr>
<tr><td>10</td><td>SET3_K13_CLUSTER_19614</td><td>gi|50918431|ref|XP_469612.1| putative ACT domain containing protein [Oryza sativa (japonica cultivar-group)] >gi|28273389|gb|AAO38475.1| putative ACT domain containing protein [Oryza sativa (japonica cultivar-group)] e_value:0</td><td>11669.m05250|LOC_Os03g51990|protein putative ACT domain containing protein e_value:0</td><td>0.264</td><td>(0.0860 0.3260)</td><td>0.086</td><td>0.326</td></tr>
<tr><td>11</td><td>SET2_E12_CLUSTER_32513</td><td>gi|50946513|ref|XP_482784.1| putative 12-oxophytodienoate reductase [Oryza sativa (japonica cultivar-group)] >gi|51964946|ref|XP_507257.1| PREDICTED P0493A04.35 gene product [Oryza sativa (japonica cultivar-group)] >gi|42408416|dbj|BAD09599.1| putat</td><td>11674.m03547|LOC_Os08g35740|protein oxidoreductase, FAD/FMN-binding e_value:0</td><td>0.088</td><td>(0.0529 0.5985)</td><td>0.0529</td><td>0.5985</td></tr>
<tr><td>12</td><td>WT010_M11_CLUSTER_26626</td><td>gi|50725393|dbj|BAD32867.1| putative UDP-galactose/UDP-glucose transporter [Oryza sativa (japonica cultivar-group)] e_value:0</td><td>11680.m03824|LOC_Os06g39260|protein expressed protein</td><td>0.112</td><td>(0.0484 0.4302)</td><td>0.0484</td><td>0.4302</td></tr>
<tr><td>13</td><td>SET6_M20_CLUSTER_29414</td><td>gi|50940045|ref|XP_479550.1| putative receptor-like protein kinase [Oryza sativa (japonica cultivar-group)] >gi|51979072|ref|XP_507413.1| PREDICTED OSJNBa0008J01.18 gene product [Oryza sativa (japonica cultivar-group)]</td><td>11673.m04790|LOC_Os07g48310|protein Protein kinase domain, putative e_value:0</td><td>0.122</td><td>(0.0925 0.7558)</td><td>0.0925</td><td>0.7558</td></tr>
<tr><td>14</td><td>WT006_O13_CLUSTER_13085</td><td>gi|38112707|gb|AAR11387.1| cytochrome P450 [Triticum aestivum] e_value:6.88366e-136</td><td>11667.m02416|LOC_Os01g24780|protein Cytochrome P450 e_value:3.09455e-122</td><td>0.225</td><td>(0.3922 1.7438)</td><td>0.3922</td><td>1.7438</td></tr>
<tr><td>15</td><td>WT008_D09_CLUSTER_9078</td><td>gi|50941377|ref|XP_480216.1| unknown protein [Oryza sativa (japonica cultivar-group)] >gnl|BL_ORD_ID|1602390 gi|51964716|ref|XP_507142.1| PREDICTED OJ9990_A01.113 gene product [Oryza sativa (japonica cultivar-group)] >gnl|BL_ORD_ID|1602390 gi|378064</td><td>11674.m00592|LOC_Os08g06600|protein Nuf2 family, putative e_value:6.31652e-145</td><td>0.497</td><td>(0.2867 0.5772)</td><td>0.2867</td><td>0.5772</td></tr>
<tr><td>16</td><td>WT013_G05_CLUSTER_28763</td><td>gi|50932723|ref|XP_475889.1| unknown protein [Oryza sativa (japonica cultivar-group)] >gnl|BL_ORD_ID|1599470 gi|49328004|gb|AAT58705.1| unknown protein [Oryza sativa (japonica cultivar-group)] e_value:0</td><td>11682.m04611|LOC_Os05g48230|protein expressed protein e_value:0</td><td>0.113</td><td>(0.0596 0.5296)</td><td>0.0596</td><td>0.5296</td></tr>
<tr><td>17</td><td>SET1_P17_CLUSTER_26879</td><td>gi|31433569|gb|AAP55067.1| putative chloroplast chaperonin [Oryza sativa (japonica cultivar-group)] >gi|37536956|ref|NP_922780.1| putative chloroplast chaperonin [Oryza sativa (japonica cultivar-group)] >gi|18855008|gb|AAL79700.1| putative chloropla</td><td>11676.m03844|LOC_Os10g41710|protein putative chloroplast chaperonin e_value:1.49598e-56</td><td>0.151</td><td>(0.0540 0.3565)</td><td>0.054</td><td>0.3565</td></tr>
<tr><td>18</td><td>WT003_H13_CLUSTER_24095</td><td>gi|38347168|emb|CAE05158.2| OSJNBa0039C07.14 [Oryza sativa (japonica cultivar-group)] >gnl|BL_ORD_ID|1536017 gi|50923969|ref|XP_472345.1| OSJNBa0039C07.14 [Oryza sativa (japonica cultivar-group)] e_value:2.61712e-66</td><td>11670.m03126|LOC_Os04g32680|protein Pollen proteins Ole e I family, putative e_value:4.45407e-68</td><td>0.239</td><td>(0.1771 0.7413)</td><td>0.1771</td><td>0.7413</td></tr>
<tr><td>19</td><td>WT013_L13_CLUSTER_29818</td><td>gi|50900022|ref|XP_450799.1| putative cysteine proteinase [Oryza sativa (japonica cultivar-group)] >gnl|BL_ORD_ID|1564107 gi|49388888|dbj|BAD26098.1| putative cysteine proteinase [Oryza sativa (japonica cultivar-group)] >gnl|BL_ORD_ID|1564107 gi|493</td><td>11681.m01937|LOC_Os09g21370|protein cysteine proteinase (EC 3.4.22.-) - barley e_value:1.24321e-128</td><td>0.432</td><td>(0.2747 0.6361)</td><td>0.2747</td><td>0.6361</td></tr>
<tr><td>20</td><td>WT004_E09_CLUSTER_29818</td><td>gi|50900022|ref|XP_450799.1| putative cysteine proteinase [Oryza sativa (japonica cultivar-group)] >gnl|BL_ORD_ID|1564107 gi|49388888|dbj|BAD26098.1| putative cysteine proteinase [Oryza sativa (japonica cultivar-group)] >gnl|BL_ORD_ID|1564107 gi|493</td><td>11681.m01937|LOC_Os09g21370|protein cysteine proteinase (EC 3.4.22.-) - barley e_value:5.58051e-129</td><td>0.429</td><td>(0.2729 0.6366)</td><td>0.2729</td><td>0.6366</td></tr>
<tr><td>21</td><td>WT002__C06_CLUSTER_5</td><td>gi|50928789|ref|XP_473922.1| OSJNBa0058K23.20 [Oryza sativa (japonica cultivar-group)] >gnl|BL_ORD_ID|1592022 gi|38344338|emb|CAE02154.2| OSJNBa0058K23.20 [Oryza sativa (japonica cultivar-group)] e_value:1.09869e-19</td><td>11670.m05188|LOC_Os04g52950|protein Nitrate-induced NOI protein e_value:6.77742e-22</td><td>0.519</td><td>(0.2251 0.4333)</td><td>0.2251</td><td>0.4333</td></tr>
<tr><td>22</td><td>SET1_K01_CLUSTER_21783</td><td>gi|51963244|ref|XP_506139.1| PREDICTED OJ1567_G09.101 gene product [Oryza sativa (japonica cultivar-group)] >gi|50933715|ref|XP_476385.1| zinc finger and C2 domain protein-like [Oryza sativa (japonica cultivar-group)] >gi|33146442|dbj|BAC79550.1| zi</td><td>11673.m00081|LOC_Os07g01770|protein C2 domain, putative e_value:2.71722e-67</td><td>0.157</td><td>(0.1813 1.1544)</td><td>0.1813</td><td>1.1544</td></tr>
<tr><td>23</td><td>SET1_G01_CLUSTER_21783</td><td>gi|51963244|ref|XP_506139.1| PREDICTED OJ1567_G09.101 gene product [Oryza sativa (japonica cultivar-group)] >gi|50933715|ref|XP_476385.1| zinc finger and C2 domain protein-like [Oryza sativa (japonica cultivar-group)] >gi|33146442|dbj|BAC79550.1| zi</td><td>11673.m00081|LOC_Os07g01770|protein C2 domain, putative e_value:2.71722e-67</td><td>0.157</td><td>(0.1813 1.1544)</td><td>0.1813</td><td>1.1544</td></tr>
<tr><td>24</td><td>SET6__C17_CLUSTER_6119</td><td>gi|34902432|ref|NP_912562.1| Unknown protein [Oryza sativa (japonica cultivar-group)] >gi|24756881|gb|AAN64145.1| Unknown protein [Oryza sativa (japonica cultivar-group)] e_value:4.78994e-90</td><td>11669.m00875|LOC_Os03g08960|protein Homeobox domain, putative e_value:1.09733e-101</td><td>0.579</td><td>(0.2024 0.3496)</td><td>0.2024</td><td>0.3496</td></tr>
<tr><td>25</td><td>SET5_P01_CLUSTER_28586</td><td>gi|50931431|ref|XP_475243.1| unknown protein [Oryza sativa (japonica cultivar-group)] >gi|53980845|gb|AAV24766.1| unknown protein [Oryza sativa (japonica cultivar-group)] >gi|48475186|gb|AAT44255.1| unknown protein [Oryza sativa (japonica cultivar-g</td><td>11682.m03290|LOC_Os05g34880|protein SecA-type chloroplast transport factor protein, putative e_value:0</td><td>0.394</td><td>(0.2128 0.5397)</td><td>0.2128</td><td>0.5397</td></tr>
<tr><td>26</td><td>SET5_L14_CLUSTER_20040</td><td>gi|50947413|ref|XP_483234.1| hypothetical protein [Oryza sativa (japonica cultivar-group)] >gi|42408910|dbj|BAD10167.1| hypothetical protein [Oryza sativa (japonica cultivar-group)] >gi|42407681|dbj|BAD08830.1| hypothetical protein [Oryza sativa (ja</td><td>11681.m02888|LOC_Os09g31260|protein hypothetical protein e_value:2.4501e-53</td><td>0.327</td><td>(0.0928 0.2841)</td><td>0.0928</td><td>0.2841</td></tr>
<tr><td>27</td><td>SET5_K18_CLUSTER_25878</td><td>gi|50912211|ref|XP_467513.1| putative 41 kD chloroplast nucleoid DNA binding protein (CND41) [Oryza sativa (japonica cultivar-group)] >gi|51964318|ref|XP_506944.1| PREDICTED OJ1008_D06.19 gene product [Oryza sativa (japonica cultivar-group)] >gi|457</td><td>11668.m04748|LOC_Os02g48870|protein Eukaryotic aspartyl protease, putative e_value:1.96963e-148</td><td>0.565</td><td>(0.2815 0.4980)</td><td>0.2815</td><td>0.498</td></tr>
<tr><td>28</td><td>SET5_I08_CLUSTER_25492</td><td>gi|50911877|ref|XP_467346.1| putative syntaxin [Oryza sativa (japonica cultivar-group)] >gi|41053124|dbj|BAD08067.1| putative syntaxin [Oryza sativa (japonica cultivar-group)] >gi|41052700|dbj|BAD07558.1| putative syntaxin [Oryza sativa (japonica cu</td><td>11667.m01508|LOC_Os01g15110|protein SNARE domain, putative e_value:1.31012e-78</td><td>0.156</td><td>(0.3186 2.0443)</td><td>0.3186</td><td>2.0443</td></tr>
<tr><td>29</td><td>SET5_G09_CLUSTER_2624</td><td>gi|3986693|gb|AAC84135.1| cytochrome [Cichorium intybus] e_value:8.90019e-54</td><td>11682.m03278|LOC_Os05g34770|protein Cytochrome c e_value:5.03226e-54</td><td>0.09</td><td>(0.1073 1.1934)</td><td>0.1073</td><td>1.1934</td></tr>
<tr><td>30</td><td>SET4_P04_CLUSTER_10152</td><td>gi|54291486|dbj|BAD62307.1| putative dual specificity phosphatase [Oryza sativa (japonica cultivar-group)] >gi|54290616|dbj|BAD62187.1| putative dual specificity phosphatase [Oryza sativa (japonica cultivar-group)] e_value:2.82083e-133</td><td>11680.m01982|LOC_Os06g20340|protein Dual specificity phosphatase, catalytic domain, putative e_value:7.51032e-135</td><td>0.124</td><td>(0.0551 0.4449)</td><td>0.0551</td><td>0.4449</td></tr>
<tr><td>31</td><td>SET4_N11_CLUSTER_1975</td><td>gi|57900387|dbj|BAD87597.1| putative Serine/threonine Kinase [Oryza sativa (japonica cultivar-group)] e_value:0</td><td>11667.m05469|LOC_Os01g55440|protein Protein kinase domain, putative e_value:0</td><td>0.402</td><td>(0.1392 0.3459)</td><td>0.1392</td><td>0.3459</td></tr>
<tr><td>32</td><td>SET3_M14_CLUSTER_7507</td><td>gi|50948279|ref|XP_483667.1| putative glycosyl hydrolase family 35 (beta-galactosidase) [Oryza sativa (japonica cultivar-group)] >gi|42407808|dbj|BAD08952.1| putative glycosyl hydrolase family 35 (beta-galactosidase) [Oryza sativa (japonica cultivar-gr</td><td>11674.m04398|LOC_Os08g43570|protein Glycosyl hydrolases family 35, putative e_value:0</td><td>0.282</td><td>(0.1545 0.5485)</td><td>0.1545</td><td>0.5485</td></tr>
<tr><td>33</td><td>SET3_L06_CLUSTER_2632</td><td>gi|60678627|gb|AAX33674.1| plus agglutinin [Chlamydomonas incerta] e_value:1.16308e-10</td><td>11670.m05658|LOC_Os04g57260|protein hypothetical protein e_value:4.61557e-08</td><td>0.406</td><td>(0.5236 1.2898)</td><td>0.5236</td><td>1.2898</td></tr>
<tr><td>34</td><td>SET3_G04_CLUSTER_13434</td><td>gi|55733810|gb|AAV59317.1| putative pectin methylesterase [Oryza sativa (japonica cultivar-group)] e_value:5.98857e-72</td><td>11687.m04326|LOC_Os11g45720|protein Pectinesterase, putative e_value:1.0728e-167</td><td>0.275</td><td>(0.2514 0.9141)</td><td>0.2514</td><td>0.9141</td></tr>
<tr><td>35</td><td>SET3__C19_CLUSTER_4224</td><td>gi|50915670|ref|XP_468299.1| zinc finger-like [Oryza sativa (japonica cultivar-group)] >gi|47497349|dbj|BAD19389.1| zinc finger-like [Oryza sativa (japonica cultivar-group)] e_value:0</td><td>11668.m05555|LOC_Os02g56280|protein von Willebrand factor type A domain, putative e_value:0</td><td>0.1</td><td>(0.0600 0.6017)</td><td>0.06</td><td>0.6017</td></tr>
<tr><td>36</td><td>SET2_O11_CLUSTER_29829</td><td>gi|50929581|ref|XP_474318.1| OSJNBb0004A17.16 [Oryza sativa (japonica cultivar-group)] >gi|38345634|emb|CAE54556.1| OSJNBb0004A17.16 [Oryza sativa (japonica cultivar-group)] >gi|32488056|emb|CAE03230.1| OSJNBa0018M05.5 [Oryza sativa (japonica cultiv</td><td>11670.m05711|LOC_Os04g57750|protein Similar to NtEPc e_value:3.98775e-53</td><td>0.663</td><td>(0.2550 0.3845)</td><td>0.255</td><td>0.3845</td></tr>
<tr><td>37</td><td>SET2__C19_CLUSTER_15140</td><td>gi|50927895|ref|XP_473475.1| OSJNBa0065O17.12 [Oryza sativa (japonica cultivar-group)] >gi|38345759|emb|CAE03487.2| OSJNBa0065O17.12 [Oryza sativa (japonica cultivar-group)] e_value:0</td><td>11670.m04600|LOC_Os04g47150|protein PA domain, putative e_value:0</td><td>0.522</td><td>(0.2019 0.3868)</td><td>0.2019</td><td>0.3868</td></tr>
<tr><td>38</td><td>SET1_P19_CLUSTER_9033</td><td>gi|50915920|ref|XP_468424.1| zinc finger-like [Oryza sativa (japonica cultivar-group)] >gi|48716354|dbj|BAD22965.1| zinc finger-like [Oryza sativa (japonica cultivar-group)] e_value:6.58442e-137</td><td>11668.m05677|LOC_Os02g57370|protein AT3g09320/F3L24_19 e_value:1.55316e-138</td><td>0.15</td><td>(0.0884 0.5876)</td><td>0.0884</td><td>0.5876</td></tr>
<tr><td>39</td><td>SET1_H12_CLUSTER_15510</td><td>gi|20197320|gb|AAM15021.1| putative inorganic pyrophosphatase [Arabidopsis thaliana] >gi|3522960|gb|AAC34242.1| putative inorganic pyrophosphatase [Arabidopsis thaliana] >gi|15226453|ref|NP_182209.1| inorganic pyrophosphatase, putative [soluble] / p</td><td>11676.m02281|LOC_Os10g26600|protein inorganic pyrophosphatase, putative e_value:8.42333e-103</td><td>0.067</td><td>(0.0633 0.9458)</td><td>0.0633</td><td>0.9458</td></tr>
<tr><td>40</td><td>WT013_M09_CLUSTER_16886</td><td>gi|50933667|ref|XP_476361.1| putative RNA-binding protein homolog [Oryza sativa (japonica cultivar-group)] >gnl|BL_ORD_ID|1517692 gi|51963238|ref|XP_506132.1| PREDICTED B1026C12.35 gene product [Oryza sativa (japonica cultivar-group)] >gnl|BL_ORD_ID</td><td>11673.m00029|LOC_Os07g01260|protein La domain, putative e_value:1.45847e-164</td><td>0.209</td><td>(0.1748 0.8368)</td><td>0.1748</td><td>0.8368</td></tr>
<tr><td>41</td><td>WT012_P16_CLUSTER_21887</td><td>gi|34907386|ref|NP_915040.1| P0471B04.27 [Oryza sativa (japonica cultivar-group)] e_value:1.60731e-53</td><td>11681.m03383|LOC_Os09g37850|protein retrotransposon protein, putative, Ty1-copia sub-class e_value:1.26882e-56</td><td>0.284</td><td>(0.6041 2.1301)</td><td>0.6041</td><td>2.1301</td></tr>
</table>

PDF Complete
Your complimentary use period has ended. Thank you for using PDF Complete.
Click Here to upgrade to Unlimited Pages and Expanded Features

<table>
<tr><td></td><td></td><td></td><td></td><td></td><td></td><td></td><td></td></tr>
<tr><td>42</td><td>WT012_I13_CLUSTER_5356</td><td>gi|50918953|ref|XP_469873.1| putative proteophosphoglycan [Oryza sativa (japonica cultivar-group)] >gnl|BL_ORD_ID|1586256 gi|17027266|gb|AAL34120.1| putative proteophosphoglycan [Oryza sativa (japonica cultivar-group)] e_value:0</td><td>11669.m05582|LOC_Os03g55470|protein putative proteophosphoglycan e_value:0</td><td>0.202</td><td>(0.0949 0.4699)</td><td>0.0949</td><td>0.4699</td></tr>
<tr><td>43</td><td>WT011_O24_CLUSTER_21272</td><td>gi|52076576|dbj|BAD45478.1| putative transcription factor GAMyb [Oryza sativa (japonica cultivar-group)] e_value:2.43251e-142</td><td>11680.m04594|LOC_Os06g46560|protein Similar to myb protein e_value:5.52952e-144</td><td>0.492</td><td>(0.2070 0.4207)</td><td>0.207</td><td>0.4207</td></tr>
<tr><td>44</td><td>WT011_M18_CLUSTER_20152</td><td>gi|50725353|dbj|BAD34425.1| putative calcium-dependent protein kinase [Oryza sativa (japonica cultivar-group)] e_value:0</td><td>11681.m03072|LOC_Os09g33910|protein Protein kinase domain, putative e_value:0</td><td>0.281</td><td>(0.1460 0.5193)</td><td>0.146</td><td>0.5193</td></tr>
<tr><td>45</td><td>WT011_K10_CLUSTER_11027</td><td>gi|51964412|ref|XP_506991.1| PREDICTED P0539D10.38 gene product [Oryza sativa (japonica cultivar-group)] >gnl|BL_ORD_ID|1583078 gi|50913107|ref|XP_467961.1| unknown protein [Oryza sativa (japonica cultivar-group)] >gnl|BL_ORD_ID|1583078 gi|46805742|</td><td>11668.m05170|LOC_Os02g52770|protein expressed protein e_value:1.29279e-53</td><td>0.765</td><td>(0.2982 0.3898)</td><td>0.2982</td><td>0.3898</td></tr>
<tr><td>46</td><td>WT011_F21_CLUSTER_2979</td><td>gi|50946097|ref|XP_482576.1| putative ectonucleoside triphosphate diphosphohydrolase [Oryza sativa (japonica cultivar-group)] >gnl|BL_ORD_ID|1607587 gi|42408882|dbj|BAD10140.1| putative ectonucleoside triphosphate diphosphohydrolase [Oryza sativa (japo</td><td>11674.m03351|LOC_Os08g33850|protein GDA1/CD39 (nucleoside phosphatase) family, putative e_value:0</td><td>0.323</td><td>(0.1941 0.6011)</td><td>0.1941</td><td>0.6011</td></tr>
<tr><td>47</td><td>WT011_D18_CLUSTER_9105</td><td>gi|50939321|ref|XP_479188.1| hydrolase-like protein [Oryza sativa (japonica cultivar-group)] >gnl|BL_ORD_ID|1530857 gi|33146617|dbj|BAC79905.1| hydrolase-like protein [Oryza sativa (japonica cultivar-group)] e_value:7.83077e-142</td><td>11669.m02758|LOC_Os03g27110|protein putative hydrolase e_value:1.34091e-121</td><td>0.298</td><td>(0.2185 0.7322)</td><td>0.2185</td><td>0.7322</td></tr>
<tr><td>48</td><td>WT010_N03_CLUSTER_27133</td><td>gi|23452821|gb|AAN33181.1| major facilitator superfamily antiporter [Oryza sativa (japonica cultivar-group)] >gnl|BL_ORD_ID|2091773 gi|56783672|dbj|BAD81084.1| major facilitator superfamily antiporter [Oryza sativa (japonica cultivar-group)] e_value:0</td><td>11667.m01732|LOC_Os01g17200|protein major facilitator superfamily protein e_value:0</td><td>0.186</td><td>(0.1008 0.5413)</td><td>0.1008</td><td>0.5413</td></tr>
<tr><td>49</td><td>WT010_K11_CLUSTER_29910</td><td>gi|34902456|ref|NP_912574.1| Unknown protein [Oryza sativa (japonica cultivar-group)] >gnl|BL_ORD_ID|1588582 gi|22748325|gb|AAN05327.1| Unknown protein [Oryza sativa (japonica cultivar-group)] e_value:0</td><td>11669.m01173|LOC_Os03g12160|protein Leucine Rich Repeat, putative e_value:0</td><td>0.293</td><td>(0.1539 0.5250)</td><td>0.1539</td><td>0.525</td></tr>
<tr><td>50</td><td>WT010_J17_CLUSTER_12966</td><td>gi|34901732|ref|NP_912212.1| putative xyloglucan endo-transglycosylase [Oryza sativa (japonica cultivar-group)] >gnl|BL_ORD_ID|1526834 gi|27261015|dbj|BAC45131.1| putative xyloglucan endo-transglycosylase [Oryza sativa (japonica cultivar-</td><td>11669.m00186|LOC_Os03g02610|protein Glycosyl hydrolases family 16, putative e_value:2.33682e-103</td><td>0.327</td><td>(0.1533 0.4692)</td><td>0.1533</td><td>0.4692</td></tr>
<tr><td>51</td><td>WT010_I20_CLUSTER_7571</td><td>gi|50946935|ref|XP_482995.1| putative Avr9/Cf-9 rapidly elicited protein [Oryza sativa (japonica cultivar-group)] >gnl|BL_ORD_ID|1609230 gi|42409028|dbj|BAD10281.1| putative Avr9/Cf-9 rapidly elicited protein [Oryza sativa (japonica cultivar-group)]</td><td>11674.m03740|LOC_Os08g37570|protein U-box domain, putative e_value:0</td><td>0.233</td><td>(0.0990 0.4241)</td><td>0.099</td><td>0.4241</td></tr>
<tr><td>52</td><td>WT010_E15_CLUSTER_30667</td><td>gi|50931813|ref|XP_475434.1| unknown protein [Oryza sativa (japonica cultivar-group)] >gnl|BL_ORD_ID|1596272 gi|46576017|gb|AAT01378.1| unknown protein [Oryza sativa (japonica cultivar-group)] >gnl|BL_ORD_ID|1596272 gi|57863811|gb|AAW56864.1| unknow</td><td>11682.m03826|LOC_Os05g40220|protein retrotransposon protein, putative, unclassified e_value:0</td><td>0.255</td><td>(0.1096 0.4292)</td><td>0.1096</td><td>0.4292</td></tr>
<tr><td>53</td><td>WT010_B09_CLUSTER_24583</td><td>gi|53793373|dbj|BAD52954.1| IFA-binding protein-like [Oryza sativa (japonica cultivar-group)] e_value:0</td><td>11667.m05620|LOC_Os01g56790|protein expressed protein</td><td>0.401</td><td>(0.2571 0.6405)</td><td>0.2571</td><td>0.6405</td></tr>
<tr><td>54</td><td>WT008_P21_CLUSTER_9385</td><td>gi|53792627|dbj|BAD53641.1| unknown protein [Oryza sativa (japonica cultivar-group)] e_value:2.19638e-96</td><td>11680.m04933|LOC_Os06g49640|protein AT4g19390/T5K18_170 e_value:5.482e-98</td><td>0.294</td><td>(0.1690 0.5752)</td><td>0.169</td><td>0.5752</td></tr>
<tr><td>55</td><td>WT008_L21_CLUSTER_15604</td><td>gi|50929119|ref|XP_474087.1| OSJNBa0033G05.10 [Oryza sativa (japonica cultivar-group)] >gnl|BL_ORD_ID|1594455 gi|38344886|emb|CAD41909.2| OSJNBa0033G05.10 [Oryza sativa (japonica cultivar-group)] e_value:0</td><td>11670.m05420|LOC_Os04g55110|protein expressed protein e_value:0</td><td>0.48</td><td>(0.2664 0.5552)</td><td>0.2664</td><td>0.5552</td></tr>
<tr><td>56</td><td>WT008_L03_CLUSTER_13367</td><td>gi|38174813|emb|CAD42650.1| putative heparanase [Hordeum vulgare subsp. vulgare] e_value:0</td><td>11669.m01082|LOC_Os03g11320|protein Glycosyl hydrolase family 79, N-terminal domain, putative e_value:0</td><td>0.146</td><td>(0.0879 0.6022)</td><td>0.0879</td><td>0.6022</td></tr>
<tr><td>57</td><td>WT008_E20_CLUSTER_25300</td><td>gi|50934927|ref|XP_476991.1| unknown protein [Oryza sativa (japonica cultivar-group)] >gnl|BL_ORD_ID|1522525 gi|50508384|dbj|BAD30384.1| unknown protein [Oryza sativa (japonica cultivar-group)] >gnl|BL_ORD_ID|1522525 gi|34393890|dbj|BAC83584.1| unkn</td><td>11673.m00924|LOC_Os07g09810|protein F-box domain, putative e_value:4.11777e-65</td><td>0.637</td><td>(0.5449 0.8550)</td><td>0.5449</td><td>0.855</td></tr>
<tr><td>58</td><td>WT007_M11_CLUSTER_7548</td><td>gi|55775286|ref|XP_550569.1| putative receptor-like protein kinase [Oryza sativa (japonica cultivar-group)] >gnl|BL_ORD_ID|1752722 gi|25553554|dbj|BAC24825.1| putative receptor-like protein kinase [Oryza sativa (japonica cultivar-group)] >gnl|BL_ORD</td><td>11680.m00273|LOC_Os06g03610|protein Protein kinase domain, putative e_value:0</td><td>0.589</td><td>(0.1803 0.3064)</td><td>0.1803</td><td>0.3064</td></tr>
<tr><td>59</td><td>WT007_G24_CLUSTER_2342</td><td>gi|50940595|ref|XP_479825.1| putative DNA polymerase III like protein [Oryza sativa (japonica cultivar-group)] >gnl|BL_ORD_ID|1601362 gi|42407921|dbj|BAD09061.1| putative DNA polymerase III like protein [Oryza sativa (japonica cultivar-group)] >gnl|</td><td>11674.m00176|LOC_Os08g02670|protein hypothetical protein e_value:0</td><td>0.154</td><td>(0.0863 0.5618)</td><td>0.0863</td><td>0.5618</td></tr>
<tr><td>60</td><td>WT006_L09_CLUSTER_26846</td><td>gi|31432071|gb|AAP53756.1| putative RNA-binding protein [Oryza sativa (japonica cultivar-group)] >gnl|BL_ORD_ID|2011201 gi|37534334|ref|NP_921469.1| putative RNA-binding protein [Oryza sativa (japonica cultivar-group)] e_value:0</td><td>11676.m02371|LOC_Os10g27480|protein expressed protein e_value:0</td><td>0.275</td><td>(0.2018 0.7337)</td><td>0.2018</td><td>0.7337</td></tr>
<tr><td>61</td><td>WT006_H20_CLUSTER_29302</td><td>gi|21436281|gb|AAM51279.1| putative casein kinase [Arabidopsis thaliana] >gnl|BL_ORD_ID|1208657 gi|19310581|gb|AAL85021.1| putative casein kinase [Arabidopsis thaliana] >gnl|BL_ORD_ID|1208657 gi|9294011|dbj|BAB01914.1| casein kinase-like protein [Ar</td><td>11682.m01047|LOC_Os05g11140|protein Protein kinase domain, putative e_value:0</td><td>0.067</td><td>(0.1727 2.5945)</td><td>0.1727</td><td>2.5945</td></tr>
<tr><td>62</td><td>WT006_F23_CLUSTER_11541</td><td>gi|13784989|gb|AAK38344.1| seven transmembrane protein Mlo8 [Zea mays] e_value:0</td><td>11682.m03255|LOC_Os05g34550|protein seven transmembrane protein Mlo8 e_value:0</td><td>0.15</td><td>(0.1006 0.6701)</td><td>0.1006</td><td>0.6701</td></tr>
<tr><td>63</td><td>WT006_D05_CLUSTER_16709</td><td>gi|11385505|gb|AAG34835.1| glutathione S-transferase GST 27 [Zea mays] e_value:1.68002e-82</td><td>11676.m03478|LOC_Os10g38340|protein putative glutathione S-transferase e_value:1.5617e-80</td><td>0.608</td><td>(0.3117 0.5123)</td><td>0.3117</td><td>0.5123</td></tr>
<tr><td>64</td><td>WT006_A06_CLUSTER_10059</td><td>gi|38344076|emb|CAE02751.2| OSJNBa0006B20.20 [Oryza sativa (japonica cultivar-group)] >gnl|BL_ORD_ID|1520869 gi|50924474|ref|XP_472597.1| OSJNBa0006B20.20 [Oryza sativa (japonica cultivar-group)] e_value:1.10236e-169</td><td>11670.m03461|LOC_Os04g35780|protein expressed protein e_value:2.48392e-171</td><td>0.161</td><td>(0.0865 0.5364)</td><td>0.0865</td><td>0.5364</td></tr>
<tr><td>65</td><td>WT005_N11_CLUSTER_12811</td><td>gi|34914992|ref|NP_918843.1| OSJNBb0093M23.16 [Oryza sativa (japonica cultivar-group)] >gnl|BL_ORD_ID|2369613 gi|21743361|dbj|BAC03353.1| C4-dicarboxylate transporter-like protein [Oryza sativa (japonica cultivar-group)] >gnl|BL_ORD_ID|2369613 gi|21</td><td>11667.m02788|LOC_Os01g28840|protein C4-dicarboxylate transporter/malic acid transport protein, putative e_value:0</td><td>0.49</td><td>(0.2039 0.4158)</td><td>0.2039</td><td>0.4158</td></tr>
<tr><td>66</td><td>WT005_L04_CLUSTER_14383</td><td>gi|62733228|gb|AAX95345.1| osmotin protein homolog - rice (fragment) [Oryza sativa (japonica cultivar-group)] e_value:3.83035e-48</td><td>11686.m03758|LOC_Os12g38150|protein thaumatin/PR5-like protein e_value:4.24375e-83</td><td>0.595</td><td>(0.2509 0.4219)</td><td>0.2509</td><td>0.4219</td></tr>
<tr><td>67</td><td>WT005_B10_CLUSTER_20943</td><td>gi|50906377|ref|XP_464677.1| putative beta-galactosidase precursor [Oryza sativa (japonica cultivar-group)] >gnl|BL_ORD_ID|1567098 gi|46805855|dbj|BAD17189.1| putative beta-galactosidase precursor [Oryza sativa (japonica cultivar-group)] e_value:0</td><td>11668.m01169|LOC_Os02g12730|protein beta-galactosidase e_value:0</td><td>0.117</td><td>(0.0603 0.5134)</td><td>0.0603</td><td>0.5134</td></tr>
<tr><td>68</td><td>WT005_A16_CLUSTER_8864</td><td>gi|50929061|ref|XP_474058.1| OSJNBb0034I13.25 [Oryza sativa (japonica cultivar-group)] >gnl|BL_ORD_ID|1593152 gi|21741231|emb|CAD41715.1| OSJNBb0034I13.25 [Oryza sativa (japonica cultivar-group)] >gnl|BL_ORD_ID|1593152 gi|32488677|emb|CAE03614.1| OS</td><td>11670.m05368|LOC_Os04g54600|protein uncharacterized plant-specific domain TIGR01570, putative e_value:2.82708e-86</td><td>0.713</td><td>(0.3406 0.4776)</td><td>0.3406</td><td>0.4776</td></tr>
<tr><td>69</td><td>WT004_N21_CLUSTER_6963</td><td>gi|34903372|ref|NP_913033.1| unnamed protein product [Oryza sativa (japonica cultivar-group)] >gnl|BL_ORD_ID|1752524 gi|11041564|dbj|BAB00648.2| unnamed protein product [Oryza sativa (japonica cultivar-group)] >gnl|BL_ORD_ID|1752524 gi|13873014|dbj|</td><td>11667.m01751|LOC_Os01g17390|protein F-box domain, putative e_value:0</td><td>0.209</td><td>(0.1057 0.5048)</td><td>0.1057</td><td>0.5048</td></tr>
<tr><td>70</td><td>WT004_H21_CLUSTER_17305</td><td>gi|34903186|ref|NP_912940.1| unnamed protein product [Oryza sativa (japonica cultivar-group)] >gnl|BL_ORD_ID|1752426 gi|6815082|dbj|BAA90368.1| unknown protein [Oryza sativa (japonica cultivar-group)] >gnl|BL_ORD_ID|1752426</td><td>11667.m01655|LOC_Os01g16470|protein Phosphatidylinositol 3- and 4-kinase, putative e_value:1.18641e-157</td><td>0.437</td><td>(0.1493 0.3414)</td><td>0.1493</td><td>0.3414</td></tr>
<tr><td>71</td><td>WT004_H15_CLUSTER_10153</td><td>gi|52077266|dbj|BAD46308.1| putative endo-1,4-beta-glucanase precursor [Oryza sativa (japonica cultivar-group)] e_value:0</td><td>11680.m01439|LOC_Os06g14540|protein cellulase (EC 3.2.1.4) F19H22.110 - Arabidopsis thaliana e_value:0</td><td>0.302</td><td>(0.0734 0.2434)</td><td>0.0734</td><td>0.2434</td></tr>
<tr><td>72</td><td>WT004_E24_CLUSTER_3211</td><td>gi|34906964|ref|NP_914829.1| putative gamma-adaptin 1 [Oryza sativa (japonica cultivar-group)] >gnl|BL_ORD_ID|1560006 gi|19386749|dbj|BAB86130.1| putative adapter-related protein complex 4 epsilon 1 subunit [Oryza sativa (japonica cultivar-group)] ></td><td>11667.m06931|LOC_Os01g68750|protein Adaptin N terminal region, putative e_value:0</td><td>0.117</td><td>(0.0544 0.4631)</td><td>0.0544</td><td>0.4631</td></tr>
<tr><td>73</td><td>WT004_D04_CLUSTER_1423</td><td>gi|50935471|ref|XP_477263.1| mucin-like protein [Oryza sativa (japonica cultivar-group)] >gnl|BL_ORD_ID|1523759 gi|51963342|ref|XP_506240.1| PREDICTED OSJNBa0003K21.13 gene product [Oryza sativa (japonica cultivar-group)] >gnl|BL_ORD_ID|1523759 gi|3</td><td>11673.m01397|LOC_Os07g14340|protein Plant invertase/pectin methylesterase inhibitor, putative e_value:7.71768e-91</td><td>0.498</td><td>(0.3994 0.8015)</td><td>0.3994</td><td>0.8015</td></tr>
<tr><td>74</td><td>WT003_L19_CLUSTER_20152</td><td>gi|50725353|dbj|BAD34425.1| putative calcium-dependent protein kinase [Oryza sativa (japonica cultivar-group)] e_value:0</td><td>11681.m03072|LOC_Os09g33910|protein Protein kinase domain, putative e_value:0</td><td>0.303</td><td>(0.1561 0.5157)</td><td>0.1561</td><td>0.5157</td></tr>
<tr><td>75</td><td>WT003_K21_CLUSTER_18774</td><td>gi|51854288|gb|AAU10669.1| putative integral membrane protein [Oryza sativa (japonica cultivar-group)] e_value:0</td><td>11682.m04730|LOC_Os05g49270|protein Sugar transporter</td><td>0.136</td><td>(0.0894 0.6576)</td><td>0.0894</td><td>0.6576</td></tr>
<tr><td>76</td><td>WT003_E12_CLUSTER_15203</td><td>gi|5821153|dbj|BAA83718.1| RNA binding protein [Homo sapiens] >gnl|BL_ORD_ID|2322248 gi|19923466|ref|NP_057417.2| splicing coactivator subunit SRm300 [Homo sapiens] e_value:1.76345e-22</td><td>11668.m00510|LOC_Os02g05670|protein expressed protein e_value:2.31217e-53</td><td>1.042</td><td>(0.5025 0.4825)</td><td>0.5025</td><td>0.4825</td></tr>
<tr><td>77</td><td>WT003_C08_CLUSTER_20687</td><td>gi|34911076|ref|NP_916885.1| P0485G01.25 [Oryza sativa (japonica cultivar-group)] e_value:0</td><td>11667.m04695|LOC_Os01g48410|protein hypothetical protein e_value:0</td><td>0.2</td><td>(0.1702 0.8529)</td><td>0.1702</td><td>0.8529</td></tr>
<tr><td>78</td><td>WT002_N07_CLUSTER_4788</td><td>gi|31415946|gb|AAP50967.1| putative Myb-like DNA-binding protein [Oryza sativa (japonica cultivar-group)] >gnl|BL_ORD_ID|2005451 gi|50918981|ref|XP_469887.1| putative Myb-like DNA-binding protein [Oryza sativa (japonica cultivar-group)] e_value:1.98454</td><td>11669.m05595|LOC_Os03g55590|protein putative Myb-like DNA-binding protein e_value:4.69098e-106</td><td>0.374</td><td>(0.2318 0.6196)</td><td>0.2318</td><td>0.6196</td></tr>
<tr><td>79</td><td>WT002_F17_CLUSTER_15415</td><td>gi|50905791|ref|XP_464384.1| glycerophosphoryl diester phosphodiesterase 2 precursor-like protein [Oryza sativa (japonica cultivar-group)] >gnl|BL_ORD_ID|1566543 gi|46390048|dbj|BAD15424.1| glycerophosphoryl diester phosphodiesterase 2 precursor-like p</td><td>11668.m00868|LOC_Os02g09450|protein Glycerophosphoryl diester phosphodiesterase family, putative e_value:0</td><td>0.253</td><td>(0.2423 0.9587)</td><td>0.2423</td><td>0.9587</td></tr>
<tr><td>80</td><td>WT005_L22_CLUSTER_33908</td><td>gi|50922341|ref|XP_471531.1| OSJNBa0071G03.3 [Oryza sativa (japonica cultivar-group)] >gnl|BL_ORD_ID|1590249 gi|38346599|emb|CAD39790.2| OSJNBa0071G03.3 [Oryza sativa (japonica cultivar-group)] e_value:2.51382e-73</td><td>11670.m01965|LOC_Os04g21340|protein expressed protein e_value:4.85895e-75</td><td>0.181</td><td>(0.1639 0.9054)</td><td>0.1639</td><td>0.9054</td></tr>
<tr><td>81</td><td>SET5_E19_CLUSTER_1741</td><td>gi|50924778|ref|XP_472740.1| OSJNBa0036B21.20 [Oryza sativa (japonica cultivar-group)] >gi|21740906|emb|CAD40902.1| OSJNBa0036B21.20 [Oryza sativa (japonica cultivar-group)] e_value:0</td><td>11670.m03655|LOC_Os04g38560|protein pectin methylesterase-like protein - maize e_value:0</td><td>0.249</td><td>(0.0852 0.3428)</td><td>0.0852</td><td>0.3428</td></tr>
<tr><td>82</td><td>WT006_N24_CLUSTER_24701</td><td>gi|31432667|gb|AAP54272.1| putative esterase [Oryza sativa (japonica cultivar-group)] >gnl|BL_ORD_ID|2012158 gi|37535366|ref|NP_921985.1| putative esterase [Oryza sativa (japonica cultivar-group)] >gnl|BL_ORD_ID|2012158 gi|13129506|gb|AAK13160.1| pu</td><td>11676.m02969|LOC_Os10g33690|protein putative esterase e_value:3.74476e-129</td><td>0.18</td><td>(0.1058 0.5866)</td><td>0.1058</td><td>0.5866</td></tr>
<tr><td>83</td><td>WT010_D11_CLUSTER_17617</td><td>gi|55296520|dbj|BAD68734.1| putative pectate lyase [Oryza sativa (japonica cultivar-group)] e_value:0</td><td>11680.m03749|LOC_Os06g38510|protein probable pectate lyase (EC 4.2.2.2) - alfalfa e_value:0</td><td>0.542</td><td>(0.1717 0.3170)</td><td>0.1717</td><td>0.317</td></tr>
</table>

PDF Complete
Your complimentary use period has ended. Thank you for using PDF Complete.
Click Here to upgrade to Unlimited Pages and Expanded Features

<table>
<tr><td>84</td><td>WT004_N08_CLUSTER_16971</td><td>gi|82449|pir||A34291 pollen allergen Lol p IIA - perennial ryegrass >gnl|BL_ORD_ID|2336092 gi|126386|sp|P14947|MPAL2_LOLPR Pollen allergen Lol p 2-A (Lol p II-A) e_value:1.29835e-31</td><td>11670.m02463|LOC_Os04g26230|protein Pollen allergen, putative e_value:9.67079e-18</td><td>0.64</td><td>(0.5582 0.8726)</td><td>0.5582</td><td>0.8726</td></tr>
<tr><td>85</td><td>SET2_H10_CLUSTER_33496</td><td>gi|2415702|emb|CAA70610.1| profilin 4 [Phleum pratense] >gi|2415698|emb|CAA70608.1| profilin 2 [Phleum pratense] >gi|3914432|sp|O24650|PROF2_PHLPR Profilin-2/4 (Pollen allergen Phl p 12) (Phl p 11) e_value:4.19106e-69</td><td>11676.m01466|LOC_Os10g17680|protein profilin a e_value:2.20474e-64</td><td>0.259</td><td>(0.0879 0.3393)</td><td>0.0879</td><td>0.3393</td></tr>
<tr><td>86</td><td>SET4_I19_CLUSTER_17912</td><td>gi|972513|emb|CAA90746.1| pollen allergen-like protein [Triticum aestivum] >gi|7489676|pir||T06550 pollen allergen homolog - wheat e_value:1.33057e-55</td><td>11670.m02358|LOC_Os04g25190|protein Pollen allergen, putative e_value:4.22798e-13</td><td>0.712</td><td>(0.7115 0.9998)</td><td>0.7115</td><td>0.9998</td></tr>
<tr><td>87</td><td>SET4_A08_CLUSTER_3948</td><td>gi|50252129|dbj|BAD28125.1| tetracycline transporter protein-like [Oryza sativa (japonica cultivar-group)] >gi|50251859|dbj|BAD27788.1| tetracycline transporter protein-like [Oryza sativa (japonica cultivar-group)] e_value:1.41275e-99</td><td>11668.m04314|LOC_Os02g44730|protein At2g16980 e_value:0</td><td>0.145</td><td>(0.0851 0.5855)</td><td>0.0851</td><td>0.5855</td></tr>
<tr><td>88</td><td>WT004_D06_CLUSTER_32528</td><td>gi|32489824|emb|CAE04568.1| OSJNBb0039L24.7 [Oryza sativa (japonica cultivar-group)] >gnl|BL_ORD_ID|1535909 gi|38346775|emb|CAE54551.1| OSJNBa0081C01.26 [Oryza sativa (japonica cultivar-group)] >gnl|BL_ORD_ID|1535909 gi|50926686|ref|XP_473290.1| OSJ</td><td>11670.m04367|LOC_Os04g45010|protein Y11002 LIM-domain SF3 protein e_value:2.77713e-98</td><td>0.2</td><td>(0.1033 0.5172)</td><td>0.1033</td><td>0.5172</td></tr>
<tr><td>89</td><td>WT007_I23_CLUSTER_7401</td><td>gi|3372236|gb|AAC28254.1| eukaryotic translation initiation factor 4B [Triticum aestivum] e_value:0</td><td>11670.m03861|LOC_Os04g40400|protein eukaryotic initiation factor 4B e_value:0</td><td>0.213</td><td>(0.1203 0.5654)</td><td>0.1203</td><td>0.5654</td></tr>
<tr><td>90</td><td>WT003_I15_CLUSTER_32435</td><td>gi|50945223|ref|XP_482139.1| unknown protein [Oryza sativa (japonica cultivar-group)] >gnl|BL_ORD_ID|1605981 gi|40253891|dbj|BAD05825.1| unknown protein [Oryza sativa (japonica cultivar-group)] e_value:5.26665e-55</td><td>11674.m02900|LOC_Os08g29530|protein Double-stranded RNA binding motif, putative e_value:1.19914e-56</td><td>0.605</td><td>(0.4164 0.6879)</td><td>0.4164</td><td>0.6879</td></tr>
<tr><td>91</td><td>WT008_N19_CLUSTER_28978</td><td>gi|18855037|gb|AAL79729.1| putative zinc finger protein [Oryza sativa] >gnl|BL_ORD_ID|867113 gi|54291044|dbj|BAD61721.1| putative NEP1-interacting protein 2 [Oryza sativa (japonica cultivar-group)] e_value:7.98246e-109</td><td>11680.m05019|LOC_Os06g50370|protein expressed protein e_value:1.45871e-110</td><td>0.173</td><td>(0.1015 0.5853)</td><td>0.1015</td><td>0.5853</td></tr>
<tr><td>92</td><td>WT012_I01_CLUSTER_16962</td><td>gi|34906916|ref|NP_914805.1| putative Rho GDP-dissociation inhibitor [Oryza sativa (japonica cultivar-group)] >gnl|BL_ORD_ID|1559933 gi|20161386|dbj|BAB90310.1| putative Rac GDP-dissociation inhibitor 1 [Oryza sativa (japonica cultivar-group)] e_value:</td><td>11667.m06910|LOC_Os01g68540|protein RHO protein GDP dissociation inhibitor e_value:1.4368e-102</td><td>0.238</td><td>(0.0770 0.3237)</td><td>0.077</td><td>0.3237</td></tr>
<tr><td>93</td><td>WT013_F23_CLUSTER_32771</td><td>gi|62701682|gb|AAX92755.1| phosphoglycerate mutase family, putative [Oryza sativa (japonica cultivar-group)] e_value:0</td><td>11687.m00445|LOC_Os11g05260|protein phosphoglycerate mutase family, putative e_value:0</td><td>0.196</td><td>(0.1186 0.6039)</td><td>0.1186</td><td>0.6039</td></tr>
<tr><td>94</td><td>SET5_E21_CLUSTER_5434</td><td>gi|21555186|gb|AAM63799.1| unknown [Arabidopsis thaliana] e_value:1.13934e-51</td><td>11686.m01029|LOC_Os12g10650|protein expressed protein e_value:2.87701e-94</td><td>0.15</td><td>(0.0802 0.5363)</td><td>0.0802</td><td>0.5363</td></tr>
<tr><td>95</td><td>WT010_G18_CLUSTER_33752</td><td>gi|50901526|ref|XP_463196.1| putative vesicle transport-related protein [Oryza sativa (japonica cultivar-group)] >gnl|BL_ORD_ID|1565224 gi|28201563|gb|AAO34501.1| putative vesicle transport-related protein [Oryza sativa (japonica cultivar-group)] e_val</td><td>11669.m04200|LOC_Os03g42320|protein putative vesicle transport-related protein e_value:0</td><td>0.146</td><td>(0.0721 0.4927)</td><td>0.0721</td><td>0.4927</td></tr>
<tr><td>96</td><td>WT008_F23_CLUSTER_14126</td><td>gi|50938801|ref|XP_478928.1| arm repeat-containing protein-like protein [Oryza sativa (japonica cultivar-group)] >gnl|BL_ORD_ID|1529899 gi|50509368|dbj|BAD30923.1| arm repeat-containing protein-like protein [Oryza sativa (japonica cultivar-group)]</td><td>11673.m03890|LOC_Os07g39880|protein Armadillo/beta-catenin-like repeat, putative e_value:0</td><td>0.099</td><td>(0.0398 0.4018)</td><td>0.0398</td><td>0.4018</td></tr>
<tr><td>97</td><td>WT010_M12_CLUSTER_27081</td><td>gi|50939883|ref|XP_479469.1| putative MSP1(mitochondrial sorting of proteins) protein [Oryza sativa (japonica cultivar-group)] >gnl|BL_ORD_ID|1534184 gi|33146850|dbj|BAC79845.1| putative MSP1(mitochondrial sorting of proteins) protein [Oryza sativa (ja</td><td>11669.m02307|LOC_Os03g22420|protein ATPase, AAA family, putative e_value:0</td><td>0.239</td><td>(0.1274 0.5323)</td><td>0.1274</td><td>0.5323</td></tr>
<tr><td>98</td><td>WT009_J12_CLUSTER_16388</td><td>gi|50904895|ref|XP_463936.1| putative NADPH-dependent mannose 6-phosphate reductase [Oryza sativa (japonica cultivar-group)] >gnl|BL_ORD_ID|1565888 gi|41053022|dbj|BAD07953.1| putative NADPH-dependent mannose 6-phosphate reductase [Oryza sativa</td><td>11668.m00237|LOC_Os02g03100|protein oxidoreductase, aldo/keto reductase family, putative e_value:7.23383e-167</td><td>0.079</td><td>(0.0443 0.5636)</td><td>0.0443</td><td>0.5636</td></tr>
<tr><td>99</td><td>SET4_N10_CLUSTER_9642</td><td>gi|21700871|gb|AAM70559.1| AT3g27050/MOJ10_14 [Arabidopsis thaliana] >gi|13937133|gb|AAK50060.1| AT3g27050/MOJ10_14 [Arabidopsis thaliana] >gi|18405214|ref|NP_566807.1| expressed protein [Arabidopsis thaliana] e_value:1.56723e-48</td><td>11687.m02708|LOC_Os11g29700|protein AT3g27050/MOJ10_14 e_value:8.33695e-76</td><td>0.216</td><td>(0.0855 0.3950)</td><td>0.0855</td><td>0.395</td></tr>
<tr><td>100</td><td>WT005_K23_CLUSTER_11056</td><td>gi|29569880|gb|AAO85268.1| glucan endo-1,3-beta-D-glucosidase [Hordeum vulgare subsp. vulgare] e_value:2.91881e-132</td><td>11667.m07188|LOC_Os01g71670|protein Glycosyl hydrolases family 17 e_value:2.39878e-102</td><td>0.512</td><td>(0.3278 0.6407)</td><td>0.3278</td><td>0.6407</td></tr>
<tr><td>101</td><td>SET6_I02_CLUSTER_24801</td><td>gi|50945329|ref|XP_482192.1| putative 2-oxoglutarate-dependent oxygenase [Oryza sativa (japonica cultivar-group)] >gi|40253423|dbj|BAD05352.1| putative 2-oxoglutarate-dependent oxygenase [Oryza sativa (japonica cultivar-group)] e_value:7.13177e-</td><td>11674.m02960|LOC_Os08g30100|protein oxidoreductase, 2OG-Fe(II) oxygenase family, putative e_value:2.13894e-112</td><td>0.497</td><td>(0.3108 0.6258)</td><td>0.3108</td><td>0.6258</td></tr>
<tr><td>102</td><td>WT006_A04_CLUSTER_886</td><td>gi|50508504|dbj|BAD30749.1| GTPase activating protein-like [Oryza sativa (japonica cultivar-group)] e_value:0</td><td>11687.m03478|LOC_Os11g37690|protein TBC domain, putative e_value:0</td><td>0.164</td><td>(0.1456 0.8888)</td><td>0.1456</td><td>0.8888</td></tr>
<tr><td>103</td><td>WT006_N10_CLUSTER_34707</td><td>gi|1617036|emb|CAA69914.1| Ted2 [Vigna unguiculata] >gnl|BL_ORD_ID|1714854 gi|7430955|pir||T11672 quinone oxidoreductase homolog - cowpea e_value:2.72699e-140</td><td>11669.m00020|LOC_Os03g01190|protein Ted2 e_value:2.59541e-156</td><td>0.189</td><td>(0.1579 0.8337)</td><td>0.1579</td><td>0.8337</td></tr>
<tr><td>104</td><td>WT006_K17_CLUSTER_7267</td><td>gi|55770301|ref|XP_550140.1| putative transcription factor X1 [Oryza sativa (japonica cultivar-group)] >gnl|BL_ORD_ID|1753536 gi|54290399|dbj|BAD61269.1| putative transcription factor X1 [Oryza sativa (japonica cultivar-group)] >gnl|BL_ORD_ID|175353</td><td>11686.m03788|LOC_Os12g38440|protein XH domain, putative e_value:2.26844e-46</td><td>0.35</td><td>(0.5090 1.4538)</td><td>0.509</td><td>1.4538</td></tr>
<tr><td>105</td><td>SET2_H06_CLUSTER_31528</td><td>gi|15228659|ref|NP_189574.1| expressed protein [Arabidopsis thaliana] e_value:1.85212e-56</td><td>11669.m01513|LOC_Os03g15190|protein hypothetical protein e_value:1.58234e-84</td><td>0.148</td><td>(0.0810 0.5488)</td><td>0.081</td><td>0.5488</td></tr>
<tr><td>106</td><td>WT012_J01_CLUSTER_32980</td><td>gi|31432681|gb|AAP54286.1| hypothetical protein [Oryza sativa (japonica cultivar-group)] >gnl|BL_ORD_ID|2012172 gi|37535394|ref|NP_921999.1| hypothetical protein [Oryza sativa (japonica cultivar-group)] >gnl|BL_ORD_ID|2012172 gi|10140746|gb|AAG13578</td><td>11676.m02985|LOC_Os10g33830|protein hypothetical protein e_value:2.51717e-26</td><td>0.444</td><td>(0.7709 1.7362)</td><td>0.7709</td><td>1.7362</td></tr>
<tr><td>107</td><td>WT006_N07_CLUSTER_31545</td><td>gi|34902400|ref|NP_912546.1| Unknown protein [Oryza sativa (japonica cultivar-group)] >gnl|BL_ORD_ID|1584098 gi|24476043|gb|AAN62785.1| Unknown protein [Oryza sativa (japonica cultivar-group)] e_value:1.40303e-105</td><td>11669.m00095|LOC_Os03g01810|protein SNF7 family, putative e_value:4.62979e-111</td><td>0.105</td><td>(0.0534 0.5068)</td><td>0.0534</td><td>0.5068</td></tr>
<tr><td>108</td><td>SET4_P16_CLUSTER_8891</td><td>gi|50932157|ref|XP_475606.1| putative RNA polymerase II fifth largest subunit [Oryza sativa (japonica cultivar-group)] >gi|45267865|gb|AAS55764.1| putative RNA polymerase II [Oryza sativa (japonica cultivar-group)] e_value:2.90926e-82</td><td>11682.m00600|LOC_Os05g06490|protein probable RNA polymerase II fifth largest subunit - Arabidopsis thaliana</td><td>0.134</td><td>(0.0732 0.5462)</td><td>0.0732</td><td>0.5462</td></tr>
<tr><td>109</td><td>WT009_N23_CLUSTER_10411</td><td>gi|50940207|ref|XP_479631.1| putative protein serine/threonine kinase BNK1 [Oryza sativa (japonica cultivar-group)] >gnl|BL_ORD_ID|1534921 gi|34394036|dbj|BAC84067.1| putative protein serine/threonine kinase BNK1 [Oryza sativa (japonica cultivar-group)</td><td>11673.m04924|LOC_Os07g49470|protein Protein kinase domain, putative e_value:5.11242e-84</td><td>0.211</td><td>(0.3163 1.5014)</td><td>0.3163</td><td>1.5014</td></tr>
<tr><td>110</td><td>WT007_L16_CLUSTER_7563</td><td>gi|42733516|dbj|BAD11355.1| BRI1-KD interacting protein 127 [Oryza sativa (japonica cultivar-group)] e_value:7.90435e-123</td><td>11686.m04175|LOC_Os12g41920|protein DNA-binding protein PcMYB1, putative e_value:6.58675e-128</td><td>0.182</td><td>(0.1106 0.6071)</td><td>0.1106</td><td>0.6071</td></tr>
<tr><td>111</td><td>SET5_N20_CLUSTER_2072</td><td>gi|18391139|ref|NP_563866.1| short-chain dehydrogenase/reductase (SDR) family protein [Arabidopsis thaliana] >gi|4914333|gb|AAD32881.1| F14N23.19 [Arabidopsis thaliana] >gi|51971537|dbj|BAD44433.1| unknown protein [Arabidopsis thaliana] >gi|25283</td><td>11668.m03335|LOC_Os02g35170|protein oxidoreductase, short chain dehydrogenase/reductase family e_value:1.50418e-126</td><td>0.115</td><td>(0.0490 0.4279)</td><td>0.049</td><td>0.4279</td></tr>
<tr><td>112</td><td>WT003_A21_CLUSTER_34325</td><td>gi|2429087|gb|AAB70865.1| lipoxygenase 2 [Hordeum vulgare subsp. vulgare] >gnl|BL_ORD_ID|2285595 gi|7433148|pir||T05945 lipoxygenase (EC 1.13.11.12) 2 - barley e_value:0</td><td>11669.m05345|LOC_Os03g52860|protein lipoxygenase L-2; lipoxygenase e_value:0</td><td>0.487</td><td>(0.1495 0.3066)</td><td>0.1495</td><td>0.3066</td></tr>
<tr><td>113</td><td>WT007_E20_CLUSTER_34806</td><td>gi|31433708|gb|AAP55187.1| putative ADP-ribosylation factor [Oryza sativa (japonica cultivar-group)] >gnl|BL_ORD_ID|2013200 gi|37537196|ref|NP_922901.1| putative ADP-ribosylation factor [Oryza sativa (japonica cultivar-group)] >gnl|BL_ORD_ID|2013200</td><td>11668.m04563|LOC_Os02g47110|protein ADP-ribosylation factor family e_value:4.62096e-85</td><td>0.056</td><td>(0.0221 0.3967)</td><td>0.0221</td><td>0.3967</td></tr>
<tr><td>114</td><td>SET1_A09_CLUSTER_4706</td><td>gi|34906220|ref|NP_914457.1| putative small basic membrane integral protein [Oryza sativa (japonica cultivar-group)] >gi|13122433|dbj|BAB32914.1| putative small basic membrane integral protein [Oryza sativa (japonica cultivar-group)] e_value:1.87124e-1</td><td>11667.m00815|LOC_Os01g08660|protein Major intrinsic protein e_value:3.63617e-117</td><td>0.144</td><td>(0.0758 0.5249)</td><td>0.0758</td><td>0.5249</td></tr>
<tr><td>115</td><td>WT005_J14_CLUSTER_30659</td><td>gi|55297348|dbj|BAD69189.1| putative o-methyltransferase [Oryza sativa (japonica cultivar-group)] >gnl|BL_ORD_ID|269832 gi|55297295|dbj|BAD69125.1| putative o-methyltransferase [Oryza sativa (japonica cultivar-group)] e_value:5.75755e-95</td><td>11687.m03074|LOC_Os11g33300|protein O-methyltransferase, putative e_value:1.41541e-98</td><td>0.712</td><td>(0.3607 0.5064)</td><td>0.3607</td><td>0.5064</td></tr>
<tr><td>116</td><td>SET4_L18_CLUSTER_7948</td><td>gi|50912501|ref|XP_467658.1| putative calcium binding protein [Oryza sativa (japonica cultivar-group)] >gi|46390425|dbj|BAD15887.1| putative calcium binding protein [Oryza sativa (japonica cultivar-group)] >gi|46390681|dbj|BAD16163.1| putative calci</td><td>11668.m04887|LOC_Os02g50190|protein ABA-induced protein, putative e_value:6.49181e-75</td><td>0.301</td><td>(0.2196 0.7294)</td><td>0.2196</td><td>0.7294</td></tr>
<tr><td>117</td><td>WT002_L17_CLUSTER_34253</td><td>gi|50915926|ref|XP_468427.1| OTU-like cysteine protease-like [Oryza sativa (japonica cultivar-group)] >gnl|BL_ORD_ID|1583560 gi|51964534|ref|XP_507052.1| PREDICTED OJ1202_E07.21-2 gene product [Oryza sativa (japonica cultivar-group)] >gnl|BL_ORD_ID|</td><td>11668.m05682|LOC_Os02g57410|protein At3g02070 e_value:4.73827e-152</td><td>0.239</td><td>(0.0903 0.3774)</td><td>0.0903</td><td>0.3774</td></tr>
<tr><td>118</td><td>WT005_P04_CLUSTER_13790</td><td>gi|18483249|gb|AAL73984.1| type IIB calcium ATPase [Medicago truncatula] e_value:0</td><td>11686.m00347|LOC_Os12g04220|protein calcium-translocating P-type ATPase, PMCA-type e_value:0</td><td>0.166</td><td>(0.0841 0.5070)</td><td>0.0841</td><td>0.507</td></tr>
<tr><td>119</td><td>WT012_H23_CLUSTER_27704</td><td>gi|50934811|ref|XP_476933.1| putative nodule-specific protein [Oryza sativa (japonica cultivar-group)] >gnl|BL_ORD_ID|1522309 gi|34394606|dbj|BAC83908.1| putative nodule-specific protein [Oryza sativa (japonica cultivar-group)] >gnl|BL_ORD_ID|152230</td><td>11673.m00843|LOC_Os07g09010|protein Nodulin, putative e_value:0</td><td>0.298</td><td>(0.1055 0.3533)</td><td>0.1055</td><td>0.3533</td></tr>
<tr><td>120</td><td>SET3_E19_CLUSTER_4843</td><td>gi|55908885|gb|AAV67828.1| unknown protein [Oryza sativa (japonica cultivar-group)] e_value:9.05457e-10</td><td>11667.m04971|LOC_Os01g50920|protein hypothetical protein e_value:1.0285e-23</td><td>0.461</td><td>(0.1579 0.3423)</td><td>0.1579</td><td>0.3423</td></tr>
<tr><td>121</td><td>WT004_F24_CLUSTER_12470</td><td>gi|20562915|gb|AAM22751.1| one helix protein [Deschampsia antarctica] e_value:6.52872e-37</td><td>11686.m02867|LOC_Os12g29570|protein one helix protein e_value:6.44742e-33</td><td>0.373</td><td>(0.2731 0.7316)</td><td>0.2731</td><td>0.7316</td></tr>
<tr><td>122</td><td>SET4_L17_CLUSTER_20971</td><td>gi|50909841|ref|XP_466409.1| putative glucosyltransferase-10 [Oryza sativa (japonica cultivar-group)] >gi|50725260|dbj|BAD34262.1| putative glucosyltransferase-10 [Oryza sativa (japonica cultivar-group)] e_value:0</td><td>11668.m03507|LOC_Os02g36830|protein expressed protein e_value:0</td><td>0.393</td><td>(0.1690 0.4298)</td><td>0.169</td><td>0.4298</td></tr>
<tr><td>123</td><td>WT009_P05_CLUSTER_28752</td><td>gi|50934283|ref|XP_476669.1| putative glycolate oxidase [Oryza sativa (japonica cultivar-group)] >gnl|BL_ORD_ID|1521592 gi|51963268|ref|XP_506164.1| PREDICTED B1364A02.11-1 gene product [Oryza sativa (japonica cultivar-group)]</td><td>11669.m05782|LOC_Os03g57220|protein glycolate oxidase e_value:0</td><td>0.121</td><td>(0.0699 0.5784)</td><td>0.0699</td><td>0.5784</td></tr>
<tr><td>124</td><td>WT005_C13_CLUSTER_28752</td><td>gi|50934283|ref|XP_476669.1| putative glycolate oxidase [Oryza sativa (japonica cultivar-group)] >gnl|BL_ORD_ID|1521592 gi|51963268|ref|XP_506164.1| PREDICTED B1364A02.11-1 gene product [Oryza sativa (japonica cultivar-group)]</td><td>11669.m05782|LOC_Os03g57220|protein glycolate oxidase e_value:0</td><td>0.108</td><td>(0.0625 0.5790)</td><td>0.0625</td><td>0.579</td></tr>
<tr><td>125</td><td>WT004_B06_CLUSTER_13179</td><td>gi|50934989|ref|XP_477022.1| unknown protein [Oryza sativa (japonica cultivar-group)] >gnl|BL_ORD_ID|1522556 gi|34394796|dbj|BAC84209.1| unknown protein [Oryza sativa (japonica cultivar-group)] e_value:0</td><td>11673.m00954|LOC_Os07g10110|protein AT5g49830/K21G20_4 e_value:0</td><td>0.072</td><td>(0.0379 0.5285)</td><td>0.0379</td><td>0.5285</td></tr>
</table>

PDF Complete
Your complimentary use period has ended. Thank you for using PDF Complete.
Click Here to upgrade to Unlimited Pages and Expanded Features

126	SET5_J10_CLUSTER_17472	gi|50882170|ref|NP_911149.2| aldo/keto reductase family-like protein [Oryza sativa (japonica cultivar-group)] >gi|38175438|dbj|BAC21406.2| aldo/keto reductase family-like protein [Oryza sativa (japonica cultivar-group)] e_value:2.02068e-178	11673.m00410|LOC_Os07g04990|protein oxidoreductase, aldo/keto reductase family e_value:5.65712e-180	0.088	(0.0950 1.0745)	0.095	1.0745
127	WT003_J15_CLUSTER_18301	gi|51964664|ref|XP_507116.1| PREDICTED P0582D05.120 gene product [Oryza sativa (japonica cultivar-group)] >gnl|BL_ORD_ID|1601557 gi|27529690|dbj|BAC53786.1| UDP-galactose 4-epimerase-like protein [Oryza sativa (japonica cultivar-group)] e_value:0	11674.m00270|LOC_Os08g03570|protein UDP-glucose 4-epimerase e_value:0	0.089	(0.0395 0.4425)	0.0395	0.4425
128	WT007_A16_CLUSTER_34191	gi|34900026|ref|NP_911359.1| putative brassinosteroid LRR receptor kinase prrotein [Oryza sativa (japonica cultivar-group)] >gnl|BL_ORD_ID|1521344 gi|50509961|dbj|BAD30371.1| putative brassinosteroid LRR receptor kinase protein [Oryza sativa (japonica	11673.m00646|LOC_Os07g07230|protein Leucine Rich Repeat, putative e_value:0	0.235	(0.1446 0.6161)	0.1446	0.6161
129	WT004_H01_CLUSTER_18368	gi|51090927|dbj|BAD35531.1| Phospholipase D alpha 2 [Oryza sativa (japonica cultivar-group)] e_value:0	11680.m03919|LOC_Os06g40190|protein phospholipase d alpha 2 (ec 3.1.4.4) (pld alpha 2) (cholinephosphatase 2) (phosphatidylcholine-hydrolyzing phospholipase d 2) e_value:0	0.245	(0.1036 0.4233)	0.1036	0.4233
130	WT003_H17_CLUSTER_28609	gi|51979000|ref|XP_507377.1| PREDICTED OJ1699_E05.22-1 gene product [Oryza sativa (japonica cultivar-group)] >gnl|BL_ORD_ID|1529588 gi|50938549|ref|XP_478802.1| putative root border cell-specific protein [Oryza sativa (japonica cultivar-group)] >gnl	11673.m03755|LOC_Os07g38620|protein Similar to root border cell-specific protein e_value:3.05051e-153	0.157	(0.0834 0.5305)	0.0834	0.5305
131	WT002_J13_CLUSTER_11654	gi|34896998|ref|NP_909845.1| unknown protein [Oryza sativa (japonica cultivar-group)] >gnl|BL_ORD_ID|1564840 gi|28269467|gb|AAO38010.1| unknown protein [Oryza sativa (japonica cultivar-group)] e_value:7.87887e-53	11669.m03942|LOC_Os03g39820|protein expressed protein e_value:1.11012e-54	0.313	(0.1779 0.5686)	0.1779	0.5686
132	SET2_N17_CLUSTER_25780	gi|50933061|ref|XP_476058.1| putative zinc finger protein [Oryza sativa (japonica cultivar-group)] >gi|47777443|gb|AAT38076.1| putative zinc finger protein [Oryza sativa (japonica cultivar-group)] e_value:0	11682.m00833|LOC_Os05g08610|protein probable RING zinc finger protein, 53384-54880 [imported] - Arabidopsis thaliana	0.205	(0.1166 0.5687)	0.1166	0.5687
133	WT009_G24_CLUSTER_33397	gi|55770613|ref|XP_550297.1| putative cytochrome b5 reductase [Oryza sativa (japonica cultivar-group)] >gnl|BL_ORD_ID|1753359 gi|55296793|dbj|BAD68119.1| putative cytochrome b5 reductase [Oryza sativa (japonica cultivar-group)] e_value:3.1447e-159	11667.m00733|LOC_Os01g07910|protein Oxidoreductase NAD-binding domain, putative e_value:7.74664e-161	0.119	(0.0568 0.4756)	0.0568	0.4756
134	WT006_L18_CLUSTER_16220	gi|56784222|dbj|BAD81717.1| putative lung alpha/beta hydrolase protein 1 [Oryza sativa (japonica cultivar-group)] e_value:0	11667.m06533|LOC_Os01g65070|protein expressed protein	0.24	(0.1239 0.5162)	0.1239	0.5162
135	WT012_D22_CLUSTER_13375	gi|51535088|dbj|BAD37677.1| nucleolar protein-like [Oryza sativa (japonica cultivar-group)] e_value:3.54395e-156	11680.m01149|LOC_Os06g11790|protein TLD, putative e_value:1.12586e-132	0.254	(0.1152 0.4540)	0.1152	0.454
136	WT007_F17_CLUSTER_21224	gi|52077175|dbj|BAD46220.1| F1F0-ATPase inhibitor protein [Oryza sativa (japonica cultivar-group)] >gnl|BL_ORD_ID|252055 gi|5821432|dbj|BAA81662.2| F1F0-ATPase inhibitor protein [Oryza sativa (japonica cultivar-group)] e_value:9.38074e-37	11681.m03560|LOC_Os09g39550|protein expressed protein e_value:5.74545e-39	0.242	(0.1930 0.7979)	0.193	0.7979
137	SET1_E07_CLUSTER_21224	gi|52077175|dbj|BAD46220.1| F1F0-ATPase inhibitor protein [Oryza sativa (japonica cultivar-group)] >gi|5821432|dbj|BAA81662.2| F1F0-ATPase inhibitor protein [Oryza sativa (japonica cultivar-group)] e_value:5.26212e-39	11681.m03560|LOC_Os09g39550|protein expressed protein e_value:5.84245e-41	0.201	(0.1602 0.7989)	0.1602	0.7989
138	WT012_D08_CLUSTER_33202	gi|50913117|ref|XP_467966.1| unknown protein [Oryza sativa (japonica cultivar-group)] >gnl|BL_ORD_ID|1583083 gi|51964414|ref|XP_506992.1| PREDICTED OJ1004_A11.24 gene product [Oryza sativa (japonica cultivar-group)] >gnl|BL_ORD_ID|1583083 gi|4680607	11668.m05176|LOC_Os02g52820|protein expressed protein e_value:0	0.202	(0.0993 0.4908)	0.0993	0.4908
139	WT009_A13_CLUSTER_16466	gi|6469117|emb|CAB61740.1| putative enoyl CoA hydratase [Cicer arietinum] e_value:1.49467e-94	11669.m02001|LOC_Os03g19680|protein enoyl CoA hydratase, putative e_value:3.79679e-134	0.135	(0.0635 0.4717)	0.0635	0.4717
140	WT004_I08_CLUSTER_33568	gi|55168342|gb|AAV44207.1| unknown protein [Oryza sativa (japonica cultivar-group)] e_value:0	11682.m00187|LOC_Os05g02650|protein expressed protein	0.15	(0.0634 0.4223)	0.0634	0.4223
141	WT012_K06_CLUSTER_28002	gi|32489531|emb|CAE04734.1| OSJNBa0043L24.22 [Oryza sativa (japonica cultivar-group)] >gnl|BL_ORD_ID|1523561 gi|50926352|ref|XP_473123.1| OSJNBa0043L24.22 [Oryza sativa (japonica cultivar-group)] e_value:0	11670.m04149|LOC_Os04g42980|protein von Willebrand factor type A domain, putative e_value:0	0.192	(0.1131 0.5885)	0.1131	0.5885
142	WT007_M07_CLUSTER_15022	gi|21436231|gb|AAM51254.1| putative alpha-N-acetylglucosaminidase [Arabidopsis thaliana] >gnl|BL_ORD_ID|1208641 gi|19423948|gb|AAL87291.1| putative alpha-N-acetylglucosaminidase [Arabidopsis thaliana] >gnl|BL_ORD_ID|1208641 gi|9758035|dbj|BAB08696.1	11670.m05490|LOC_Os04g55730|protein Alpha-N-acetylglucosaminidase (NAGLU) e_value:0	0.393	(0.3564 -1.0000)	0.3564	-1
143	WT006_D13_CLUSTER_3518	gi|51963836|ref|XP_506701.1| PREDICTED P0482F12.28-1 gene product [Oryza sativa (japonica cultivar-group)] >gnl|BL_ORD_ID|1566218 gi|50904977|ref|XP_463977.1| putative phosphoribosyl pyrophosphate synthase [Oryza sativa (japonica cultivar-group)] >g	11680.m04046|LOC_Os06g41360|protein ribose-phosphate diphosphokinase (EC 2.7.6.1) isoform II [validated] - Arabidopsis thaliana (fragment) e_value:1.96003e-141	0.117	(0.2756 2.3528)	0.2756	2.3528
144	WT005_K07_CLUSTER_34186	gi|50918567|ref|XP_469680.1| putative CorA-like Mg2+ transporter protein [Oryza sativa (japonica cultivar-group)] >gnl|BL_ORD_ID|1538732 gi|40539050|gb|AAR87307.1| putative CorA-like Mg2+ transporter protein [Oryza sativa (japonica cultivar-	11669.m05372|LOC_Os03g53110|protein putative CorA-like Mg2+ transporter protein e_value:0	0.076	(0.0333 0.4362)	0.0333	0.4362
145	WT003_O16_CLUSTER_32531	gi|50251453|dbj|BAD28518.1| putative tetratricoredoxin [Oryza sativa (japonica cultivar-group)] e_value:4.58858e-141	11681.m02120|LOC_Os09g23650|protein tetratricoredoxin e_value:1.28734e-142	0.201	(0.1137 0.5670)	0.1137	0.567
146	WT008_N14_CLUSTER_34501	gi|31432529|gb|AAP54151.1| hypothetical protein [Oryza sativa (japonica cultivar-group)] >gnl|BL_ORD_ID|2012038 gi|37535124|ref|NP_921864.1| hypothetical protein [Oryza sativa (japonica cultivar-group)] >gnl|BL_ORD_ID|2012038 gi|22165093|gb|AAM93710	11686.m03168|LOC_Os12g32480|protein hypothetical protein e_value:2.78329e-93	0.403	(0.7866 1.9507)	0.7866	1.9507
147	WT007_A05_CLUSTER_1908	gi|21742080|emb|CAD41191.1| OSJNBa0074L08.3 [Oryza sativa (japonica cultivar-group)] >gnl|BL_ORD_ID|1535063 gi|50926616|ref|XP_473255.1| OSJNBa0074L08.3 [Oryza sativa (japonica cultivar-group)] e_value:0	11670.m04324|LOC_Os04g44610|protein ABC transporter, putative e_value:0	0.077	(0.0342 0.4425)	0.0342	0.4425
148	SET3_H03_CLUSTER_50	gi|62733391|gb|AAX95508.1| Putative Phosphatidylinositol-4-phosphate 5-Kinase [Oryza sativa (japonica cultivar-group)] >gi|50470594|emb|CAD67588.1| phosphatidylinositol 4-phosphate 5-kinase [Oryza sativa (japonica cultivar-group)] e_value:0	11669.m04977|LOC_Os03g49510|protein phosphatidylinositol-4-phosphate 5-Kinase domain containing protein e_value:0	0.167	(0.0936 0.5619)	0.0936	0.5619
149	WT007_E03_CLUSTER_8621	gi|31433256|gb|AAP54794.1| hypothetical protein [Oryza sativa (japonica cultivar-group)] >gnl|BL_ORD_ID|2013714 gi|37536410|ref|NP_922507.1| hypothetical protein [Oryza sativa (japonica cultivar-group)] >gnl|BL_ORD_ID|2013714 gi|22002159|gb|AAM88643	11676.m03544|LOC_Os10g38980|protein expressed protein e_value:0	0.12	(0.0658 0.5504)	0.0658	0.5504
150	SET6_I22_CLUSTER_31673	gi|37695546|gb|AAR00318.1| pyridoxal kinase [Triticum aestivum] e_value:1.21974e-173	11686.m04050|LOC_Os12g40830|protein pyridoxal kinase e_value:8.24283e-165	0.09	(0.0420 0.4663)	0.042	0.4663
151	WT011_A16_CLUSTER_5048	gi|38347446|emb|CAE02488.2| OSJNBa0076N16.10 [Oryza sativa (japonica cultivar-group)] >gnl|BL_ORD_ID|1536872 gi|38345234|emb|CAE01663.2| OSJNBa0084K20.12 [Oryza sativa (japonica cultivar-group)] >gnl|BL_ORD_ID|1536872 gi|50925601|ref|XP_472985.1| OS	11670.m03966|LOC_Os04g41320|protein Nucleotide-sugar transporter, putative e_value:0	0.169	(0.0688 0.4064)	0.0688	0.4064
152	WT012_D02_CLUSTER_9757	gi|10177025|dbj|BAB10263.1| beta-1,3-glucanase-like [Arabidopsis thaliana] >gnl|BL_ORD_ID|1096465 gi|28827612|gb|AAO50650.1| putative glycosyl hydrolase family 17 protein [Arabidopsis thaliana] >gnl|BL_ORD_ID|1096465 gi|28393035|gb|AAO41952.1| putat	11669.m01878|LOC_Os03g18520|protein Similar to beta-1,3-glucanase e_value:0	0.273	(0.1294 0.4744)	0.1294	0.4744
153	WT009_J16_CLUSTER_29238	gi|50934873|ref|XP_476964.1| putative N-amidino-scyllo-inosamine-4-phosphate phosphatase [Oryza sativa (japonica cultivar-group)] >gnl|BL_ORD_ID|1522343 gi|51963310|ref|XP_506210.1| PREDICTED OSJNBb0003E08.17 gene product [Oryza sativa (japonica cultiv	11673.m00875|LOC_Os07g09330|protein putative, inositol monophosphatase family e_value:1.72817e-156	0.181	(0.1108 0.6109)	0.1108	0.6109
154	WT009_P04_CLUSTER_32484	gi|2462911|emb|CAB06081.1| UDP-glucose:sterol glucosyltransferase [Avena sativa] e_value:0	11667.m06492|LOC_Os01g64750|protein probable UDP-glucose,sterol glucosyltransferase [imported] - Arabidopsis thaliana e_value:9.71524e-178	0.193	(0.4618 2.3936)	0.4618	2.3936
155	WT008_E01_CLUSTER_6293	gi|34905012|ref|NP_913853.1| unknown protein [Oryza sativa (japonica cultivar-group)] >gnl|BL_ORD_ID|1606409 gi|27817985|dbj|BAC55749.1| unknown protein [Oryza sativa (japonica cultivar-group)] e_value:4.27393e-87	11674.m03257|LOC_Os08g32980|protein expressed protein e_value:8.76189e-89	0.223	(0.1212 0.5434)	0.1212	0.5434
156	WT009_C15_CLUSTER_15284	gi|50939831|ref|XP_479443.1| putative protein serine/threonine kinase [Oryza sativa (japonica cultivar-group)] >gnl|BL_ORD_ID|1533473 gi|34395193|dbj|BAC83593.1| putative protein serine/threonine kinase [Oryza sativa (japonica cultivar-group)] e_value:	11673.m04675|LOC_Os07g47270|protein Protein kinase domain, putative e_value:0	0.148	(0.0870 0.5885)	0.087	0.5885
157	WT007_H12_CLUSTER_4522	gi|50920813|ref|XP_470767.1| expressed protein [Oryza sativa (japonica cultivar-group)] >gnl|BL_ORD_ID|1511194 gi|40882691|gb|AAR96232.1| expressed protein [Oryza sativa (japonica cultivar-group)] e_value:5.81455e-40	11669.m02691|LOC_Os03g26490|protein expressed protein e_value:1.27177e-41	0.461	(0.2786 0.6039)	0.2786	0.6039
158	WT011_D09_CLUSTER_20224	gi|28209488|gb|AAO37506.1| expressed protein [Oryza sativa (japonica cultivar-group)] >gnl|BL_ORD_ID|2058520 gi|50916517|ref|XP_468655.1| unknown protein [Oryza sativa (japonica cultivar-group)] e_value:3.55562e-56	11669.m03014|LOC_Os03g29920|protein expressed protein e_value:1.1476e-57	0.435	(0.2471 0.5677)	0.2471	0.5677
159	SET6_G20_CLUSTER_20272	gi|15239643|ref|NP_197410.1| heavy-metal-associated domain-containing protein [Arabidopsis thaliana] e_value:1.1949e-87	11686.m00437|LOC_Os12g05040|protein heavy metal-associated domain, putative e_value:0	0.304	(0.1677 0.5525)	0.1677	0.5525
160	SET1_O01_CLUSTER_30563	gi|23928446|gb|AAN40032.1| putative protein [Zea mays] e_value:6.75184e-56	11687.m03748|LOC_Os11g40200|protein expressed protein e_value:6.38178e-90	0.192	(0.1322 0.6899)	0.1322	0.6899
161	WT007_K08_CLUSTER_19988	gi|55775048|ref|XP_550450.1| unknown protein [Oryza sativa (japonica cultivar-group)] >gnl|BL_ORD_ID|1752791 gi|55295836|dbj|BAD67704.1| unknown protein [Oryza sativa (japonica cultivar-group)] e_value:0	11668.m00051|LOC_Os02g01490|protein hypothetical protein e_value:0	0.287	(0.1593 0.5545)	0.1593	0.5545
162	WT013_N13_CLUSTER_22241	gi|54290527|dbj|BAD61868.1| unknown protein [Oryza sativa (japonica cultivar-group)] e_value:3.62476e-23	11680.m02146|LOC_Os06g21910|protein abscisic acid-induced gene. e_value:6.96623e-25	0.506	(0.1834 0.3627)	0.1834	0.3627
163	SET3_E15_CLUSTER_3977	gi|21689719|gb|AAM67481.1| putative heat shock protein 18 [Arabidopsis thaliana] >gi|17979311|gb|AAL49881.1| putative heat shock protein 18 [Arabidopsis thaliana] >gi|16344|emb|CAA35183.1| unnamed protein product [Arabidopsis thaliana] >gi|975883	11667.m00355|LOC_Os01g04380|protein Hsp20/alpha crystallin family, putative e_value:2.52599e-38	1.16	(0.4472 0.3855)	0.4472	0.3855
164	WT011_C12_CLUSTER_10612	gi|52076919|dbj|BAD45931.1| putative geranyl diphosphat synthase [Oryza sativa (japonica cultivar-group)] >gnl|BL_ORD_ID|251662 gi|52076633|dbj|BAD45534.1| putative geranyl diphosphate synthase [Oryza sativa (japonica cultivar-group)] e_value:0	11680.m04583|LOC_Os06g46450|protein geranyl diphosphat synthase e_value:0	0.139	(0.0621 0.4480)	0.0621	0.448
165	WT009_O14_CLUSTER_32844	gi|53370662|gb|AAU89157.1| Zinc finger, C3HC4 type (RING finger) containing protein [Oryza sativa (japonica cultivar-group)] e_value:1.22984e-129	11686.m04046|LOC_Os12g40790|protein probable C3HC4 zinc finger protein [imported] - Arabidopsis thaliana	0.422	(0.2099 0.4977)	0.2099	0.4977
166	SET4_N23_CLUSTER_1093	gi|62701670|gb|AAX92743.1| expressed protein [Oryza sativa (japonica cultivar-group)] e_value:2.84064e-92	11687.m00433|LOC_Os11g05140|protein expressed protein e_value:6.36285e-94	0.472	(0.3799 0.8049)	0.3799	0.8049

PDF Complete
Your complimentary use period has ended. Thank you for using PDF Complete.
Click Here to upgrade to Unlimited Pages and Expanded Features

167	SET3_P11_CLUSTER_3731	gi|34902060|ref|NP_912376.1| hypothetical protein [Oryza sativa (japonica cultivar-group)] >gi|29893653|gb|AAP06907.1| hypothetical protein [Oryza sativa (japonica cultivar-group)] >gi|29893664|gb|AAP06918.1| unknown protein [Oryza sativa (japonica	11669.m01625|LOC_Os03g16230|protein oxidoreductase, short chain dehydrogenase/reductase family e_value:1.08555e-120	0.216	(0.1024 0.4744)	0.1024	0.4744
168	SET3_A02_CLUSTER_11807	gi|34898382|ref|NP_910537.1| EST AU055776(S20048) corresponds to a region of the predicted gene.~Similar to Arabidopsis thaliana AP2 domain containing protein RAP2.10 mRNA, partial cds.(AF003103) [Oryza sativa (japonica cultivar-group)] e_value:3.42693e-6	11680.m00650|LOC_Os06g07030|protein AP2 domain, putative e_value:2.12054e-61	0.564	(0.2108 0.3737)	0.2108	0.3737
169	WT013_B05_CLUSTER_16885	gi|53370662|gb|AAU89157.1| Zinc finger, C3HC4 type (RING finger) containing protein [Oryza sativa (japonica cultivar-group)] e_value:7.86823e-62	11669.m05920|LOC_Os03g58390|protein expressed protein e_value:8.73435e-139	0.393	(0.2165 0.5504)	0.2165	0.5504
170	WT013_C07_CLUSTER_22636	gi|50934513|ref|XP_476784.1| PHD finger transcription factor-like [Oryza sativa (japonica cultivar-group)] >gnl|BL_ORD_ID|1521453 gi|34394455|dbj|BAC83629.1| PHD finger transcription factor-like [Oryza sativa (japonica cultivar-group)] e_value:0	11673.m00698|LOC_Os07g07690|protein PHD-finger, putative e_value:0	0.466	(0.2614 0.5611)	0.2614	0.5611
171	WT012_P02_CLUSTER_22873	gi|55741422|ref|XP_550376.1| putative serine/threonine protein kinase [Oryza sativa (japonica cultivar-group)] >gnl|BL_ORD_ID|1743567 gi|55296058|dbj|BAD67620.1| putative serine/threonine protein kinase [Oryza sativa (japonica cultivar-group)] >gnl|	11680.m00670|LOC_Os06g07230|protein Protein kinase domain, putative e_value:0	0.101	(0.0708 0.7001)	0.0708	0.7001
172	WT012_F01_CLUSTER_18029	gi|54290764|dbj|BAD61385.1| putative nucleostemin [Oryza sativa (japonica cultivar-group)] >gnl|BL_ORD_ID|265440 gi|54290761|dbj|BAD61382.1| putative nucleostemin [Oryza sativa (japonica cultivar-group)] e_value:0	11667.m02667|LOC_Os01g27730|protein expressed protein e_value:0	0.168	(0.0999 0.5938)	0.0999	0.5938
173	WT011_L04_CLUSTER_15839	gi|51964868|ref|XP_507218.1| PREDICTED P0434E03.16-1 gene product [Oryza sativa (japonica cultivar-group)] >gnl|BL_ORD_ID|1606004 gi|50945259|ref|XP_482157.1| putative mitochondrial carrier protein [Oryza sativa (japonica cultivar-group)]	11674.m02922|LOC_Os08g29720|protein Mitochondrial carrier protein, putative e_value:0	0.209	(0.0835 0.3995)	0.0835	0.3995
174	WT011_F02_CLUSTER_13339	gi|14334566|gb|AAK59462.1| unknown protein [Arabidopsis thaliana] >gnl|BL_ORD_ID|1207287 gi|15237714|ref|NP_201255.1| membrane protein-related [Arabidopsis thaliana] >gnl|BL_ORD_ID|1207287 gi|25055018|gb|AAN71972.1| unknown protein [Arabidopsis thal	11670.m04303|LOC_Os04g44430|protein expressed protein e_value:0	0.276	(0.1352 0.4899)	0.1352	0.4899
175	WT009_J13_CLUSTER_25073	gi|34906250|ref|NP_914472.1| P0489A01.10 [Oryza sativa (japonica cultivar-group)] e_value:0	11667.m00831|LOC_Os01g08780|protein Endonuclease/Exonuclease/phosphatase family, putative	0.518	(0.1999 0.3856)	0.1999	0.3856
176	WT009_B17_CLUSTER_28983	gi|9755730|emb|CAC01842.1| putative protein [Arabidopsis thaliana] >gnl|BL_ORD_ID|1487640 gi|15237413|ref|NP_197180.1| SART-1 family protein [Arabidopsis thaliana] >gnl|BL_ORD_ID|1487640 gi|11357805|pir||T51510 hypothetical protein F5E19_120 - Arabi	11668.m02925|LOC_Os02g30730|protein SART-1 family e_value:0	0.268	(0.1335 0.4990)	0.1335	0.499
177	WT008_J18_CLUSTER_26842	gi|50916118|ref|XP_468523.1| C2 domain-containing protein-like [Oryza sativa (japonica cultivar-group)] >gnl|BL_ORD_ID|1583740 gi|48716325|dbj|BAD22937.1| C2 domain-containing protein-like [Oryza sativa (japonica cultivar-group)] e_value:0	11668.m05774|LOC_Os02g58230|protein At1g50260 e_value:0	0.19	(0.1055 0.5539)	0.1055	0.5539
178	WT008_C15_CLUSTER_11593	gi|38345952|emb|CAE04343.2| OSJNBb0038F03.7 [Oryza sativa (japonica cultivar-group)] >gnl|BL_ORD_ID|1523085 gi|50927315|ref|XP_473383.1| OSJNBb0038F03.7 [Oryza sativa (japonica cultivar-group)] e_value:0	11670.m04474|LOC_Os04g45990|protein Pre-SET motif, putative e_value:0	0.353	(0.2131 0.6032)	0.2131	0.6032
179	WT006_O03_CLUSTER_9748	gi|50400023|gb|AAT76411.1| expressed protein [Oryza sativa (japonica cultivar-group)] e_value:5.77706e-89	11669.m05889|LOC_Os03g58130|protein expressed protein e_value:1.14352e-90	0.188	(0.0863 0.4578)	0.0863	0.4578
180	WT005_O15_CLUSTER_19715	gi|50934245|ref|XP_476650.1| unknown protein [Oryza sativa (japonica cultivar-group)] >gnl|BL_ORD_ID|1521571 gi|34393399|dbj|BAC82910.1| unknown protein [Oryza sativa (japonica cultivar-group)] e_value:0	11673.m00476|LOC_Os07g05610|protein expressed protein e_value:0	0.21	(0.0884 0.4210)	0.0884	0.421
181	WT005_E09_CLUSTER_18522	gi|20146104|dbj|BAB88939.1| Rurm1 [Oryza sativa] >gnl|BL_ORD_ID|729678 gi|21624374|dbj|BAC01162.1| ubiquitin-related modifier-1 [Oryza sativa (indica cultivar-group)] e_value:1.02516e-46	11673.m02669|LOC_Os07g28280|protein expressed protein e_value:3.85337e-49	0.059	(0.0573 0.9691)	0.0573	0.9691
182	WT005_A17_CLUSTER_15470	gi|55296130|dbj|BAD67848.1| putative serine/threonine protein phosphatase PP1 [Oryza sativa (japonica cultivar-group)] e_value:0	11680.m00634|LOC_Os06g06880|protein serine/threonine protein phosphatase pp1 (ec 3.1.3.16). e_value:0	0.056	(0.0261 0.4661)	0.0261	0.4661
183	WT004_A22_CLUSTER_27260	gi|50948085|ref|XP_483570.1| putative biotin synthase [Oryza sativa (japonica cultivar-group)] >gnl|BL_ORD_ID|1610535 gi|50725683|dbj|BAD33149.1| putative biotin synthase [Oryza sativa (japonica cultivar-group)] e_value:0	11674.m04308|LOC_Os08g42730|protein biotin synthase, putative e_value:0	0.085	(0.0443 0.5226)	0.0443	0.5226
184	WT003_M06_CLUSTER_18322	gi|50910283|ref|XP_466630.1| zinc finger protein [Oryza sativa (japonica cultivar-group)] >gnl|BL_ORD_ID|1578029 gi|51964150|ref|XP_506858.1| PREDICTED OJ1058_F07.25 gene product [Oryza sativa (japonica cultivar-group)] >gnl|BL_ORD_ID|1578029 gi|474	11668.m03772|LOC_Os02g39360|protein B-box zinc finger, putative e_value:3.04216e-93	0.507	(0.1488 0.2936)	0.1488	0.2936
185	WT003_B22_CLUSTER_15965	gi|7269352|emb|CAB79411.1| hypothetical protein [Arabidopsis thaliana] >gnl|BL_ORD_ID|1459827 gi|4455245|emb|CAB36744.1| hypothetical protein [Arabidopsis thaliana] >gnl|BL_ORD_ID|1459827 gi|18416469|ref|NP_567715.1| KOW domain-containing protein /	11669.m01470|LOC_Os03g14860|protein G-patch domain, putative e_value:7.34679e-177	0.242	(0.1587 0.6560)	0.1587	0.656
186	WT002_O23_CLUSTER_11807	gi|34898382|ref|NP_910537.1| EST AU055776(S20048) corresponds to a region of the predicted gene.~Similar to Arabidopsis thaliana AP2 domain containing protein RAP2.10 mRNA, partial cds.(AF003103) [Oryza sativa (japonica cultivar-group)] e_value:1.61669e-6	11680.m00650|LOC_Os06g07030|protein AP2 domain, putative e_value:2.12054e-61	0.564	(0.2108 0.3737)	0.2108	0.3737
187	WT002_H16_CLUSTER_22076	gi|17979063|gb|AAL49799.1| putative tyrosine phosphatase [Arabidopsis thaliana] >gnl|BL_ORD_ID|1207519 gi|11994464|dbj|BAB02466.1| tyrosine phosphatase-like protein [Arabidopsis thaliana] >gnl|BL_ORD_ID|1207519 gi|15230379|ref|NP_188573.1| expressed	11686.m02087|LOC_Os12g21890|protein tyrosine phosphatase, putative e_value:0	0.149	(0.0846 0.5670)	0.0846	0.567
188	SET4_N02_CLUSTER_24316	gi|50908031|ref|XP_465504.1| hypothetical protein [Oryza sativa (japonica cultivar-group)] >gi|47496858|dbj|BAD19822.1| hypothetical protein [Oryza sativa (japonica cultivar-group)] e_value:1.91598e-14	11668.m02477|LOC_Os02g26470|protein hypothetical protein e_value:2.66655e-16	1.208	(0.3565 0.2951)	0.3565	0.2951
189	SET4_L21_CLUSTER_3567	gi|52076478|dbj|BAD45357.1| putative Noc3p [Oryza sativa (japonica cultivar-group)] e_value:0	11680.m02909|LOC_Os06g30320|protein At1g79140 e_value:0	0.237	(0.1406 0.5927)	0.1406	0.5927
190	SET4_F15_CLUSTER_28418	gi|53792620|dbj|BAD53634.1| putative arogenate dehydrogenase isoform 2 [Oryza sativa (japonica cultivar-group)] e_value:1.41463e-136	11680.m04920|LOC_Os06g49520|protein Prephenate dehydrogenase, putative e_value:6.99602e-138	0.415	(0.2105 0.5074)	0.2105	0.5074
191	SET4_B16_CLUSTER_7775	gi|34539782|gb|AAQ74769.1| 1-Cys-peroxiredoxine [Triticum aestivum] e_value:2.23152e-125	11673.m04369|LOC_Os07g44430|protein AhpC/TSA family, putative e_value:1.783e-109	0.199	(0.0812 0.4085)	0.0812	0.4085
192	SET4_K02_CLUSTER_5135	gi|54291017|dbj|BAD61695.1| putative Ntdin [Oryza sativa (japonica cultivar-group)] e_value:1.87778e-53	11680.m05079|LOC_Os06g50930|protein At5g66040/K2A18_11 e_value:3.58603e-55	0.235	(0.1757 0.7461)	0.1757	0.7461
193	WT005_N04_CLUSTER_33358	gi|48475129|gb|AAT44198.1| unknown protein [Oryza sativa (japonica cultivar-group)] e_value:0	11682.m04860|LOC_Os05g50480|protein hypothetical protein e_value:0	0.335	(0.2048 0.6108)	0.2048	0.6108
194	WT008_J05_CLUSTER_18806	gi|54287580|gb|AAV31324.1| putative gamma-tubulin complex component 4 [Oryza sativa (japonica cultivar-group)] e_value:0	11682.m00574|LOC_Os05g06260|protein Spc97 / Spc98 family e_value:0	0.123	(0.0616 0.4995)	0.0616	0.4995
195	WT002_N12_CLUSTER_7008	gi|38567721|emb|CAE76010.1| B1358B12.19 [Oryza sativa (japonica cultivar-group)] >gnl|BL_ORD_ID|1628196 gi|50924850|ref|XP_472770.1| B1358B12.19 [Oryza sativa (japonica cultivar-group)] e_value:3.82023e-100	11681.m02083|LOC_Os09g23300|protein Integral membrane protein e_value:9.63674e-118	0.156	(0.0676 0.4335)	0.0676	0.4335
196	SET3_K24_CLUSTER_5869	gi|21624267|dbj|BAC01119.1| transcription factor PCF8 [Oryza sativa (japonica cultivar-group)] e_value:4.49563e-88	11686.m04205|LOC_Os12g42190|protein transposon protein, putative, unclassified e_value:1.4772e-89	0.313	(0.1861 0.5952)	0.1861	0.5952
197	WT007_D24_CLUSTER_26321	gi|50428738|gb|AAT77089.1| putative serine esterase [Oryza sativa (japonica cultivar-group)] >gnl|BL_ORD_ID|929128 gi|41469267|gb|AAS07149.1| expressed protein [Oryza sativa (japonica cultivar-group)] e_value:0	11669.m05216|LOC_Os03g51670|protein hypothetical protein e_value:0	0.215	(0.1013 0.4704)	0.1013	0.4704
198	WT005_C04_CLUSTER_32211	gi|34914604|ref|NP_918649.1| putative TATA binding protein-associated factor [Oryza sativa (japonica cultivar-group)] >gnl|BL_ORD_ID|1542455 gi|20804496|dbj|BAB92191.1| putative TAF6 [Oryza sativa (japonica cultivar-group)] e_value:0	11667.m03089|LOC_Os01g32750|protein TATA box binding protein associated factor (TAF), putative e_value:0	0.115	(0.0598 0.5191)	0.0598	0.5191
199	WT010_I23_CLUSTER_4656	gi|52076969|dbj|BAD45979.1| thiF family protein-like [Oryza sativa (japonica cultivar-group)] >gnl|BL_ORD_ID|251927 gi|52076794|dbj|BAD45738.1| thiF family protein-like [Oryza sativa (japonica cultivar-group)] e_value:0	11680.m01387|LOC_Os06g14040|protein hypothetical protein e_value:0	0.103	(0.0549 0.5345)	0.0549	0.5345
200	WT005_E21_CLUSTER_34891	gi|50932485|ref|XP_475770.1| 'unknown protein, contains IQ calmodulin-binding motif' [Oryza sativa (japonica cultivar-group)] >gnl|BL_ORD_ID|1598813 gi|47900419|gb|AAT39213.1| 'unknown protein, contains IQ calmodulin-binding motif'	11682.m04407|LOC_Os05g45930|protein IQ calmodulin-binding motif, putative e_value:0	0.202	(0.1216 0.6008)	0.1216	0.6008
201	WT004_N10_CLUSTER_34160	gi|51964122|ref|XP_506844.1| PREDICTED OSJNBa0016G10.5 gene product [Oryza sativa (japonica cultivar-group)] >gnl|BL_ORD_ID|1577047 gi|50909995|ref|XP_466486.1| putative brix domain-containing protein [Oryza sativa (japonica cultivar-group)] >gnl|BL	11668.m03615|LOC_Os02g37870|protein Brix domain, putative e_value:9.88039e-167	0.105	(0.0556 0.5315)	0.0556	0.5315
202	WT002_I16_CLUSTER_6887	gi|50948177|ref|XP_483616.1| putative hydroxymethylglutaryl coenzyme A synthase [Oryza sativa (japonica cultivar-group)] >gnl|BL_ORD_ID|1610585 gi|42408555|dbj|BAD09733.1| putative hydroxymethylglutaryl coenzyme A synthase [Oryza sativa (japonica culti	11674.m04357|LOC_Os08g43170|protein hydroxymethylglutaryl-CoA synthase e_value:0	0.079	(0.0414 0.5275)	0.0414	0.5275
203	WT012_N22_CLUSTER_31346	gi|50725144|dbj|BAD33761.1| putative GTPase activating protein [Oryza sativa (japonica cultivar-group)] e_value:0	11681.m03188|LOC_Os09g35960|protein TBC domain, putative e_value:0	0.118	(0.0847 0.7178)	0.0847	0.7178
204	WT002_J06_CLUSTER_31929	gi|34896140|ref|NP_909414.1| putative glucose-6-phosphate/phosphate- tranlocator [Oryza sativa (japonica cultivar-group)] >gnl|BL_ORD_ID|1751349 gi|13486667|dbj|BAB39904.1| contains ESTs D48306(S14443),D24269(R1613),AU076096(E20048)~similar to Arabidop	11668.m03988|LOC_Os02g41780|protein At2g30460 e_value:6.97499e-124	0.102	(0.2502 2.4439)	0.2502	2.4439
205	WT013_G04_CLUSTER_32917	gi|50908931|ref|XP_465954.1| putative protein serine/threonine kinase [Oryza sativa (japonica cultivar-group)] >gnl|BL_ORD_ID|1576535 gi|48716573|dbj|BAD23244.1| putative protein serine/threonine kinase [Oryza sativa (japonica cultivar-group)] e_value:	11668.m02942|LOC_Os02g30900|protein serine/threonine-protein kinase pbs1 (ec 2.7.1.37) (avrpphbsusceptible protein 1)	0.103	(0.1049 1.0207)	0.1049	1.0207
206	SET2_E09_CLUSTER_10898	gi|50948789|ref|XP_493752.1| EST AU056133(S20320) corresponds to a region of the predicted gene.~Similar to Caenorhabditis elegans cosmid D1054; hypothetical protein (Z74030) [Oryza sativa (japonica cultivar-group)] e_value:0	11680.m00446|LOC_Os06g05150|protein hypothetical protein e_value:0	0.17	(0.0988 0.5817)	0.0988	0.5817
207	WT011_G12_CLUSTER_34976	gi|50912331|ref|XP_467573.1| unknown protein [Oryza sativa (japonica cultivar-group)] >gnl|BL_ORD_ID|1579229 gi|46390597|dbj|BAD16081.1| unknown protein [Oryza sativa (japonica cultivar-group)] e_value:2.95351e-140	11668.m04813|LOC_Os02g49450|protein BSD domain, putative e_value:7.81806e-142	0.201	(0.0623 0.3102)	0.0623	0.3102
208	WT012_P18_CLUSTER_34195	gi|50906357|ref|XP_464667.1| putative aminopeptidase M [Oryza sativa (japonica cultivar-group)] >gnl|BL_ORD_ID|1567089 gi|46805845|dbj|BAD17179.1| putative aminopeptidase M [Oryza sativa (japonica cultivar-group)] e_value:0	11668.m01161|LOC_Os02g12650|protein Peptidase family M1, putative e_value:0	0.126	(0.0687 0.5429)	0.0687	0.5429
209	SET5_H23_CLUSTER_34195	gi|50906357|ref|XP_464667.1| putative aminopeptidase M [Oryza sativa (japonica cultivar-group)] >gi|46805845|dbj|BAD17179.1| putative aminopeptidase M [Oryza sativa (japonica cultivar-group)] e_value:0	11668.m01161|LOC_Os02g12650|protein Peptidase family M1, putative e_value:0	0.125	(0.0681 0.5433)	0.0681	0.5433

PDF Complete
Your complimentary use period has ended. Thank you for using PDF Complete.
Click Here to upgrade to Unlimited Pages and Expanded Features

<table>
<tr><td>210</td><td>WT009_B14_CLUSTER_31971</td><td>gi|50251399|dbj|BAD28426.1| unknown protein [Oryza sativa (japonica cultivar-group)] e_value:0</td><td>11668.m03110|LOC_Os02g33010|protein hypothetical protein e_value:0</td><td>0.248</td><td>(0.1314 0.5292)</td><td>0.1314</td><td>0.5292</td></tr>
<tr><td>211</td><td>WT006_K12_CLUSTER_29089</td><td>gi|50948549|ref|XP_483802.1| putative ripening regulated protein DDTFR18 [Oryza sativa (japonica cultivar-group)] >gnl|BL_ORD_ID|1612304 gi|42408436|dbj|BAD09618.1| putative ripening regulated protein DDTFR18 [Oryza sativa (japonica cultivar-</td><td>11674.m04543|LOC_Os08g44870|protein MATE efflux family protein e_value:0</td><td>0.172</td><td>(0.1464 0.8530)</td><td>0.1464</td><td>0.853</td></tr>
<tr><td>212</td><td>SET2_P16_CLUSTER_33762</td><td>gi|63003513|dbj|BAD98161.1| ATP-dependent protease [Oryza sativa (japonica cultivar-group)] e_value:7.77692e-140</td><td>11669.m01981|LOC_Os03g19510|protein ATP-dependent clp proteinase (EC 3.4.21.-) chain P1 [imported] - Arabidopsis thaliana e_value:2.04044e-141</td><td>0.094</td><td>(0.0467 0.4981)</td><td>0.0467</td><td>0.4981</td></tr>
<tr><td>213</td><td>WT006_J02_CLUSTER_34113</td><td>gi|34912560|ref|NP_917627.1| P0410E03.18 [Oryza sativa (japonica cultivar-group)] >gnl|BL_ORD_ID|1542828 gi|12382018|dbj|BAB21287.1| putative integral membrane Yip1 family protein [Oryza sativa (japonica cultivar-group)] e_value:3.52146e-157</td><td>11667.m04097|LOC_Os01g42480|protein Yip1 domain, putative e_value:8.54575e-159</td><td>0.076</td><td>(0.0298 0.3918)</td><td>0.0298</td><td>0.3918</td></tr>
<tr><td>214</td><td>WT011_E02_CLUSTER_29813</td><td>gi|40736983|gb|AAG13565.2| hypothetical protein [Oryza sativa (japonica cultivar-group)] e_value:3.34609e-87</td><td>11676.m03318|LOC_Os10g36860|protein expressed protein e_value:2.88879e-88</td><td>0.524</td><td>(0.1780 0.3399)</td><td>0.178</td><td>0.3399</td></tr>
<tr><td>215</td><td>WT004_O06_CLUSTER_6628</td><td>gi|50905881|ref|XP_464429.1| putative thioredoxin peroxidase [Oryza sativa (japonica cultivar-group)] >gnl|BL_ORD_ID|1566510 gi|51963916|ref|XP_506741.1| PREDICTED P0453H10.33 gene product [Oryza sativa (japonica cultivar-group)] >gnl|BL_ORD_ID|1566</td><td>11668.m00918|LOC_Os02g09940|protein AhpC/TSA family, putative e_value:2.38927e-96</td><td>0.174</td><td>(0.0824 0.4737)</td><td>0.0824</td><td>0.4737</td></tr>
<tr><td>216</td><td>WT007_N06_CLUSTER_32456</td><td>gi|50510002|dbj|BAD30579.1| phosphoglycerate dehydrogenase-like protein [Oryza sativa (japonica cultivar-group)] >gnl|BL_ORD_ID|553583 gi|50509022|dbj|BAD31969.1| phosphoglycerate dehydrogenase-like protein [Oryza sativa (japonica cultivar-group)] e_va</td><td>11673.m01568|LOC_Os07g16040|protein D-isomer specific 2-hydroxyacid dehydrogenase, NAD binding domain, putative e_value:2.3529e-149</td><td>0.227</td><td>(0.1299 0.5729)</td><td>0.1299</td><td>0.5729</td></tr>
<tr><td>217</td><td>WT012_I09_CLUSTER_11386</td><td>gi|34894964|ref|NP_908807.1| B1088D01.3 [Oryza sativa (japonica cultivar-group)] >gnl|BL_ORD_ID|1560890 gi|15623925|dbj|BAB67981.1| STAM binding protein(associated molecule with the SH3 domain of STAM)-like [Oryza sativa (japonica cultivar-group)] e_va</td><td>11667.m02300|LOC_Os01g23640|protein Mov34/MPN/PAD-1 family, putative e_value:0</td><td>0.287</td><td>(0.1408 0.4906)</td><td>0.1408</td><td>0.4906</td></tr>
<tr><td>218</td><td>WT012__C24_CLUSTER_11386</td><td>gi|34894964|ref|NP_908807.1| B1088D01.3 [Oryza sativa (japonica cultivar-group)] >gnl|BL_ORD_ID|1560890 gi|15623925|dbj|BAB67981.1| STAM binding protein(associated molecule with the SH3 domain of STAM)-like [Oryza sativa (japonica cultivar-group)] e_va</td><td>11667.m02300|LOC_Os01g23640|protein Mov34/MPN/PAD-1 family, putative e_value:0</td><td>0.293</td><td>(0.1407 0.4807)</td><td>0.1407</td><td>0.4807</td></tr>
<tr><td>219</td><td>WT007__C11_CLUSTER_2389</td><td>gi|50917589|ref|XP_469191.1| expressed protein (with alternative splicing) [Oryza sativa (japonica cultivar-group)] >gnl|BL_ORD_ID|1586182 gi|40538909|gb|AAR87166.1| expressed protein (with alternative splicing) [Oryza sativa (japonica cultivar-</td><td>11669.m04643|LOC_Os03g46440|protein expressed protein (with alternative splicing) e_value:0</td><td>0.195</td><td>(0.1407 0.7207)</td><td>0.1407</td><td>0.7207</td></tr>
<tr><td>220</td><td>WT011_P17_CLUSTER_32438</td><td>gi|56201559|dbj|BAD73447.1| putative VHS2 protein [Oryza sativa (japonica cultivar-group)] e_value:0</td><td>11667.m06079|LOC_Os01g61030|protein VHS domain, putative e_value:0</td><td>0.225</td><td>(0.1337 0.5947)</td><td>0.1337</td><td>0.5947</td></tr>
<tr><td>221</td><td>WT004_K20_CLUSTER_28463</td><td>gi|51090393|dbj|BAD35315.1| putative aminopeptidase C [Oryza sativa (japonica cultivar-group)] e_value:0</td><td>11680.m01088|LOC_Os06g11180|protein Similar to acyl-peptide hydrolase e_value:0</td><td>0.134</td><td>(0.0721 0.5387)</td><td>0.0721</td><td>0.5387</td></tr>
<tr><td>222</td><td>WT012_M22_CLUSTER_12293</td><td>gi|50933243|ref|XP_476149.1| 'unknown protein, contains PPR repeat' [Oryza sativa (japonica cultivar-group)] >gnl|BL_ORD_ID|1600861 gi|48475165|gb|AAT44234.1| 'unknown protein, contains PPR repeat' [Oryza sativa (japonica</td><td>11682.m01149|LOC_Os05g12130|protein expressed protein e_value:0</td><td>0.231</td><td>(0.1584 0.6861)</td><td>0.1584</td><td>0.6861</td></tr>
<tr><td>223</td><td>WT008_F21_CLUSTER_20849</td><td>gi|50941259|ref|XP_480157.1| putative oxysterol binding protein [Oryza sativa (japonica cultivar-group)] >gnl|BL_ORD_ID|1601958 gi|37805974|dbj|BAC99388.1| putative oxysterol binding protein [Oryza sativa (japonica cultivar-group)] e_value:0</td><td>11674.m00510|LOC_Os08g05870|protein Oxysterol-binding protein, putative e_value:0</td><td>0.145</td><td>(0.0814 0.5622)</td><td>0.0814</td><td>0.5622</td></tr>
<tr><td>224</td><td>WT004_B15_CLUSTER_32966</td><td>gi|478404|pir||JQ2247 photosystem I chain D precursor - barley >gnl|BL_ORD_ID|2305837 gi|548603|sp|P36213|PSAD_HORVU Photosystem I reaction center subunit II, chloroplast precursor (Photosystem I 20 kDa subunit) (PSI-D) >gnl|BL_ORD_ID|2305837 gi|167</td><td>11674.m04519|LOC_Os08g44680|protein PsaD, putative e_value:5.79067e-96</td><td>0.232</td><td>(0.0852 0.3680)</td><td>0.0852</td><td>0.368</td></tr>
<tr><td>225</td><td>WT008_D08_CLUSTER_16791</td><td>gi|50918183|ref|XP_469488.1| putative snRNP protein [Oryza sativa] >gnl|BL_ORD_ID|1536976 gi|62733557|gb|AAX95674.1| DEAD/DEAH box helicase, putative [Oryza sativa (japonica cultivar-group)] e_value:0</td><td>11669.m05045|LOC_Os03g50090|protein transposon protein, putative, CACTA, En/Spm sub-class e_value:0</td><td>0.101</td><td>(0.0527 0.5224)</td><td>0.0527</td><td>0.5224</td></tr>
<tr><td>226</td><td>WT004_E17_CLUSTER_32288</td><td>gi|54291193|dbj|BAD61890.1| putative auxin-regulated protein [Oryza sativa (japonica cultivar-group)] e_value:1.01934e-105</td><td>11680.m02247|LOC_Os06g22870|protein AUX/IAA family e_value:1.99987e-107</td><td>0.188</td><td>(0.0818 0.4347)</td><td>0.0818</td><td>0.4347</td></tr>
<tr><td>227</td><td>WT005_M14_CLUSTER_28304</td><td>gi|42742277|gb|AAS45241.1| indole-3-glycerol phosphate lyase [Hordeum lechleri] e_value:1.412e-145</td><td>11669.m05912|LOC_Os03g58320|protein putative indole-3-glycerol phosphate lyase e_value:1.28214e-117</td><td>0.256</td><td>(0.1672 0.6535)</td><td>0.1672</td><td>0.6535</td></tr>
<tr><td>228</td><td>WT004_M12_CLUSTER_32505</td><td>gi|1915960|emb|CAA68913.1| peptidylprolyl isomerase [Triticum aestivum] >gnl|BL_ORD_ID|1714561 gi|7437329|pir||T06489 probable peptidylprolyl isomerase (EC 5.2.1.8) FKBP77 - wheat e_value:0</td><td>11670.m02685|LOC_Os04g28420|protein peptidyl-prolyl cis-trans isomerase, FKBP-type, putative e_value:0</td><td>0.107</td><td>(0.0956 0.8941)</td><td>0.0956</td><td>0.8941</td></tr>
<tr><td>229</td><td>WT012_J16_CLUSTER_21311</td><td>gi|54291302|dbj|BAD62070.1| putative golden2-like transcription factor [Oryza sativa (japonica cultivar-group)] e_value:7.98639e-180</td><td>11680.m02371|LOC_Os06g24070|protein myb-like DNA-binding domain, SHAQKYF class, putative e_value:0</td><td>0.201</td><td>(0.1648 0.8216)</td><td>0.1648</td><td>0.8216</td></tr>
<tr><td>230</td><td>WT008_L01_CLUSTER_14121</td><td>gi|34911088|ref|NP_916891.1| OJ1117_G01.11 [Oryza sativa (japonica cultivar-group)] >gnl|BL_ORD_ID|1545292 gi|21104740|dbj|BAB93328.1| Nt-iaa4.1 deduced protein-like [Oryza sativa (japonica cultivar-group)] e_value:2.20959e-110</td><td>11667.m04699|LOC_Os01g48450|protein AUX/IAA family e_value:5.10725e-112</td><td>0.201</td><td>(0.0889 0.4434)</td><td>0.0889</td><td>0.4434</td></tr>
<tr><td>231</td><td>WT008_E10_CLUSTER_5616</td><td>gi|30023732|gb|AAP13399.1| At3g05625 [Arabidopsis thaliana] >gnl|BL_ORD_ID|1221655 gi|27311659|gb|AAO00795.1| Unknown protein [Arabidopsis thaliana] >gnl|BL_ORD_ID|1221655 gi|30679555|ref|NP_683531.2| expressed protein [Arabidopsis thaliana] e_value</td><td>11667.m02347|LOC_Os01g24090|protein hypothetical protein e_value:4.63004e-105</td><td>0.192</td><td>(0.1177 0.6139)</td><td>0.1177</td><td>0.6139</td></tr>
<tr><td>232</td><td>WT005_G18_CLUSTER_21348</td><td>gi|50908821|ref|XP_465899.1| unknown protein [Oryza sativa (japonica cultivar-group)] >gnl|BL_ORD_ID|1576479 gi|51979246|ref|XP_507486.1| PREDICTED P0047E05.32-1 gene product [Oryza sativa (japonica cultivar-group)] >gnl|BL_ORD_ID|1576479 gi|5196405</td><td>11668.m02890|LOC_Os02g30410|protein expressed protein e_value:8.96456e-61</td><td>0.331</td><td>(0.2117 0.6402)</td><td>0.2117</td><td>0.6402</td></tr>
<tr><td>233</td><td>WT004__C09_CLUSTER_5105</td><td>gi|53791782|dbj|BAD53576.1| calcium-dependent protein kinase CPK1 adapter protein 2-like [Oryza sativa (japonica cultivar-group)] e_value:1.86505e-153</td><td>11680.m04994|LOC_Os06g50150|protein calcium-dependent protein kinase CPK1 adapter protein 2, putative</td><td>0.146</td><td>(0.0814 0.5574)</td><td>0.0814</td><td>0.5574</td></tr>
<tr><td>234</td><td>WT003__C12_CLUSTER_30742</td><td>gi|50919867|ref|XP_470294.1| ribulose-5-phosphate-3-epimerase [Oryza sativa (japonica cultivar-group)] >gnl|BL_ORD_ID|1539368 gi|19071636|gb|AAL84303.1| ribulose-5-phosphate-3-epimerase [Oryza sativa (japonica cultivar-group)] e_value:8.75703e-129</td><td>11669.m00693|LOC_Os03g07300|protein ribulose-5-phosphate-3-epimerase e_value:2.00942e-130</td><td>0.183</td><td>(0.0635 0.3465)</td><td>0.0635</td><td>0.3465</td></tr>
<tr><td>235</td><td>WT002_K16_CLUSTER_29394</td><td>gi|54291811|gb|AAV32180.1| unknown protein [Oryza sativa (japonica cultivar-group)] e_value:3.69028e-48</td><td>11682.m02526|LOC_Os05g27100|protein heavy metal-associated domain, putative e_value:5.85594e-50</td><td>0.357</td><td>(0.1483 0.4158)</td><td>0.1483</td><td>0.4158</td></tr>
<tr><td>236</td><td>WT002_B06_CLUSTER_14121</td><td>gi|34911088|ref|NP_916891.1| OJ1117_G01.11 [Oryza sativa (japonica cultivar-group)] >gnl|BL_ORD_ID|1545292 gi|21104740|dbj|BAB93328.1| Nt-iaa4.1 deduced protein-like [Oryza sativa (japonica cultivar-group)] e_value:2.23328e-110</td><td>11667.m04699|LOC_Os01g48450|protein AUX/IAA family e_value:5.10725e-112</td><td>0.201</td><td>(0.0889 0.4434)</td><td>0.0889</td><td>0.4434</td></tr>
<tr><td>237</td><td>SET5_O05_CLUSTER_16954</td><td>gi|4090293|emb|CAA10497.1| hypothetical protein [Secale cereale] e_value:9.64927e-99</td><td>11686.m01934|LOC_Os12g19410|protein Ribulose bisphosphate carboxylase, small subunit, putative e_value:4.37557e-88</td><td>0.187</td><td>(0.0945 0.5052)</td><td>0.0945</td><td>0.5052</td></tr>
<tr><td>238</td><td>SET2_P09_CLUSTER_16423</td><td>gi|56784302|dbj|BAD82228.1| unknown protein [Oryza sativa (japonica cultivar-group)] e_value:3.36944e-79</td><td>11667.m06646|LOC_Os01g66150|protein hypothetical protein e_value:5.55538e-75</td><td>0.427</td><td>(0.1876 0.4389)</td><td>0.1876</td><td>0.4389</td></tr>
<tr><td>239</td><td>WT013_K23_CLUSTER_21351</td><td>gi|34902024|ref|NP_912358.1| unknown protein [Oryza sativa (japonica cultivar-group)] >gnl|BL_ORD_ID|1588808 gi|29893628|gb|AAP06882.1| unknown protein [Oryza sativa (japonica cultivar-group)] >gnl|BL_ORD_ID|1588808 gi|1815660|gb|AAC78392.1| low mol</td><td>11669.m01603|LOC_Os03g16020|protein Hsp20/alpha crystallin family, putative e_value:4.27472e-75</td><td>0.459</td><td>(0.0538 0.1174)</td><td>0.0538</td><td>0.1174</td></tr>
<tr><td>240</td><td>WT012_E02_CLUSTER_29487</td><td>gi|38344541|emb|CAD40969.2| OSJNBa0027P08.9 [Oryza sativa (japonica cultivar-group)] >gnl|BL_ORD_ID|1534615 gi|50924568|ref|XP_472644.1| OSJNBa0027P08.9 [Oryza sativa (japonica cultivar-group)] e_value:5.42893e-78</td><td>11670.m03511|LOC_Os04g36750|protein Hsp20/alpha crystallin family, putative e_value:1.13684e-79</td><td>0.438</td><td>(0.2179 0.4972)</td><td>0.2179</td><td>0.4972</td></tr>
<tr><td>241</td><td>SET1_P11_CLUSTER_17613</td><td>gi|4028567|gb|AAC96314.1| heat shock protein HSP26 [Triticum aestivum] e_value:3.00034e-133</td><td>11669.m01398|LOC_Os03g14180|protein heat shock protein 26 e_value:5.46405e-94</td><td>0.289</td><td>(0.1199 0.4143)</td><td>0.1199</td><td>0.4143</td></tr>
<tr><td>242</td><td>SET5_M12_CLUSTER_675</td><td>gi|37718878|gb|AAR01749.1| ribosomal protein L17-like protein [Oryza sativa (japonica cultivar-group)] e_value:1.35793e-75</td><td>11669.m06108|LOC_Os03g60100|protein putative ribosomal protein e_value:3.16572e-77</td><td>0.385</td><td>(0.2149 0.5586)</td><td>0.2149</td><td>0.5586</td></tr>
<tr><td>243</td><td>WT004_F03_CLUSTER_5932</td><td>gi|42567719|ref|NP_196353.2| amidase family protein [Arabidopsis thaliana] e_value:0</td><td>11687.m03053|LOC_Os11g33090|protein amidase-like protein e_value:0</td><td>0.19</td><td>(0.1156 0.6080)</td><td>0.1156</td><td>0.608</td></tr>
<tr><td>244</td><td>WT003_O18_CLUSTER_18612</td><td>gi|50911775|ref|XP_467295.1| putative secretory carrier membrane protein [Oryza sativa (japonica cultivar-group)] >gnl|BL_ORD_ID|1580410 gi|41052954|dbj|BAD07864.1| putative secretory carrier membrane protein [Oryza sativa (japonica cultivar-</td><td>11668.m04553|LOC_Os02g47010|protein SCAMP family, putative e_value:4.92009e-129</td><td>0.107</td><td>(0.1106 1.0312)</td><td>0.1106</td><td>1.0312</td></tr>
<tr><td>245</td><td>WT003_F13_CLUSTER_960</td><td>gi|13434996|dbj|BAB39760.1| protoporphyrinogen oxidase I [Oryza sativa (japonica cultivar-group)] >gnl|BL_ORD_ID|3039 gi|13429992|dbj|BAB39744.1| protoporphyrinogen oxidase I [Oryza sativa (japonica cultivar-group)] e_value:0</td><td>11667.m01795|LOC_Os01g18320|protein protoporphyrinogen oxidase e_value:0</td><td>0.151</td><td>(0.0717 0.4739)</td><td>0.0717</td><td>0.4739</td></tr>
<tr><td>246</td><td>SET5_B04_CLUSTER_17082</td><td>gi|50911643|ref|XP_467229.1| unknown protein [Oryza sativa (japonica cultivar-group)] >gi|41052808|dbj|BAD07676.1| unknown protein [Oryza sativa (japonica cultivar-group)] e_value:1.10911e-75</td><td>11668.m04493|LOC_Os02g46440|protein expressed protein e_value:2.96344e-77</td><td>0.273</td><td>(0.0993 0.3644)</td><td>0.0993</td><td>0.3644</td></tr>
<tr><td>247</td><td>WT006__C16_CLUSTER_31303</td><td>gi|34914620|ref|NP_918657.1| P0520B06.24 [Oryza sativa (japonica cultivar-group)] e_value:0</td><td>11667.m03097|LOC_Os01g32830|protein LrgB-like family, putative e_value:0</td><td>0.182</td><td>(0.1410 0.7739)</td><td>0.141</td><td>0.7739</td></tr>
<tr><td>248</td><td>WT005_O03_CLUSTER_15504</td><td>gi|50940387|ref|XP_479721.1| putative adenylate kinase, chloroplast (ATP-AMP transphosphorylase) [Oryza sativa (japonica cultivar-group)] >gnl|BL_ORD_ID|1601172 gi|51964612|ref|XP_507090.1| PREDICTED P0007D08.12 gene product [Oryza sativa (japonica cul</td><td>11674.m00084|LOC_Os08g01770|protein adenylate kinases, putative e_value:1.82609e-121</td><td>0.212</td><td>(0.1101 0.5194)</td><td>0.1101</td><td>0.5194</td></tr>
<tr><td>249</td><td>SET1_G04_CLUSTER_2371</td><td>gi|50931405|ref|XP_475230.1| unknown protein [Oryza sativa (japonica cultivar-group)] >gi|49328158|gb|AAT58854.1| unknown protein [Oryza sativa (japonica cultivar-group)] e_value:1.47599e-49</td><td>11682.m03185|LOC_Os05g33880|protein Cys protease inhibitor. e_value:3.47819e-51</td><td>0.484</td><td>(0.2514 0.5192)</td><td>0.2514</td><td>0.5192</td></tr>
<tr><td>250</td><td>WT013_H12_CLUSTER_6529</td><td>gi|50940565|ref|XP_479810.1| putative adenylate kinase, chloroplast precursor (ATP-AMP transphosphorylase) [Oryza sativa (japonica cultivar-group)] >gnl|BL_ORD_ID|1601743 gi|42407906|dbj|BAD09046.1| putative adenylate kinase, chloroplast precursor (ATP</td><td>11674.m00163|LOC_Os08g02540|protein Adenylate kinase, putative e_value:7.57241e-137</td><td>0.175</td><td>(0.1012 0.5792)</td><td>0.1012</td><td>0.5792</td></tr>
<tr><td>251</td><td>SET5_B16_CLUSTER_17108</td><td>gi|4138871|gb|AAD03605.1| small heat shock protein Hsp23.6 [Triticum aestivum] e_value:1.59705e-119</td><td>11680.m01131|LOC_Os06g11610|protein small heat shock protein Hsp23.6 e_value:2.74801e-54</td><td>0.872</td><td>(0.2942 0.3373)</td><td>0.2942</td><td>0.3373</td></tr>
</table>

PDF Complete
Your complimentary use period has ended. Thank you for using PDF Complete.
Click Here to upgrade to Unlimited Pages and Expanded Features

<table>
<tr><td>252</td><td>WT009_F09_CLUSTER_32836</td><td>gi|50401177|sp|Q9MBC2|MMT1_HORVU Methionine S-methyltransferase (AdoMet:Met S-methyltransferase) (Hv-MMT1) >gnl|BL_ORD_ID|48432 gi|7634680|dbj|BAA94795.1| S-adenosyl-L-methionine: L-methionine S-methyltransferase [Hordeum vulgare subsp. vulgare] e_valu</td><td>11682.m00055|LOC_Os05g01470|protein expressed protein e_value:0</td><td>0.105</td><td>(0.0577 0.5510)</td><td>0.0577</td><td>0.551</td></tr>
<tr><td>253</td><td>SET5_B24_CLUSTER_32685</td><td>gi|6176440|gb|AAF05592.1| alanyl-tRNA synthetase [Dictyostelium discoideum] e_value:0</td><td>11676.m00907|LOC_Os10g10240|protein alanyl-tRNA synthetase e_value:1.79226e-168</td><td>0.143</td><td>(0.0731 0.5097)</td><td>0.0731</td><td>0.5097</td></tr>
<tr><td>254</td><td>WT008_H15_CLUSTER_7237</td><td>gi|31432660|gb|AAP54265.1| unknown protein [Oryza sativa (japonica cultivar-group)] >gnl|BL_ORD_ID|2012151 gi|37535352|ref|NP_921978.1| unknown protein [Oryza sativa (japonica cultivar-group)] >gnl|BL_ORD_ID|2012151 gi|13129505|gb|AAK13159.1| unknow</td><td>11676.m02963|LOC_Os10g33630|protein expressed protein e_value:5.49423e-139</td><td>0.16</td><td>(0.0736 0.4606)</td><td>0.0736</td><td>0.4606</td></tr>
<tr><td>255</td><td>WT013_I11_CLUSTER_25241</td><td>gi|50899522|ref|XP_450549.1| methylenetetrahydrofolate dehydrogenase-like [Oryza sativa (japonica cultivar-group)] >gnl|BL_ORD_ID|1563806 gi|48716904|dbj|BAD23599.1| methylenetetrahydrofolate dehydrogenase-like [Oryza sativa (japonica cultivar-group)]</td><td>11681.m01423|LOC_Os09g15810|protein methylenetetrahydrofolate dehydrogenase [imported] - Arabidopsis thaliana e_value:1.42471e-178</td><td>0.139</td><td>(0.0798 0.5727)</td><td>0.0798</td><td>0.5727</td></tr>
<tr><td>256</td><td>SET4_I15_CLUSTER_22725</td><td>gi|50920391|ref|XP_470556.1| Unknown protein [Oryza sativa] >gi|15217293|gb|AAK92637.1| Unknown protein [Oryza sativa] >gi|23396617|sp|Q94HF1|IF3C_ORYSA Eukaryotic translation initiation factor 3 subunit 12 (eIF-3 p25) (eIF3k) e_value:4.85595e-117</td><td>11669.m00820|LOC_Os03g08450|protein eukaryotic translation initiation factor 3 subunit 11 (eif-3 p25)(eif3k) e_value:9.96864e-</td><td>0.072</td><td>(0.0392 0.5447)</td><td>0.0392</td><td>0.5447</td></tr>
<tr><td>257</td><td>SET6_O08_CLUSTER_19190</td><td>gi|50910511|ref|XP_466744.1| leucine-rich repeat-like protein [Oryza sativa (japonica cultivar-group)] >gi|51979302|ref|XP_507509.1| PREDICTED OJ1792_D02.5-2 gene product [Oryza sativa (japonica cultivar-group)] >gi|51979300|ref|XP_507508.1| PREDICT</td><td>11668.m03877|LOC_Os02g40260|protein Leucine Rich Repeat, putative e_value:3.62805e-152</td><td>0.312</td><td>(0.2288 0.7329)</td><td>0.2288</td><td>0.7329</td></tr>
<tr><td>258</td><td>SET3_H23_CLUSTER_9059</td><td>gi|57900348|dbj|BAD87301.1| putative transcription activator RF2a [Oryza sativa (japonica cultivar-group)] e_value:1.45305e-108</td><td>11667.m05438|LOC_Os01g55150|protein bZIP transcription factor, putative e_value:2.92739e-110</td><td>0.302</td><td>(0.1134 0.3755)</td><td>0.1134</td><td>0.3755</td></tr>
<tr><td>259</td><td>WT010_O12_CLUSTER_31575</td><td>gi|29367599|gb|AAO72661.1| muscle derived-like protein [Oryza sativa (japonica cultivar-group)] e_value:8.83439e-155</td><td>11669.m01812|LOC_Os03g17930|protein hypothetical protein e_value:2.42792e-156</td><td>0.262</td><td>(0.1634 0.6226)</td><td>0.1634</td><td>0.6226</td></tr>
<tr><td>260</td><td>WT002_B22_CLUSTER_32465</td><td>gi|50929743|ref|XP_474399.1| OSJNBa0032F06.20 [Oryza sativa (japonica cultivar-group)] >gnl|BL_ORD_ID|1594581 gi|38345563|emb|CAE03437.2| OSJNBa0032F06.20 [Oryza sativa (japonica cultivar-group)] e_value:0</td><td>11670.m05807|LOC_Os04g58620|protein potassium efflux system protein, putative e_value:0</td><td>0.134</td><td>(0.0821 0.6132)</td><td>0.0821</td><td>0.6132</td></tr>
<tr><td>261</td><td>WT013_E04_CLUSTER_33595</td><td>gi|50939519|ref|XP_479287.1| putative protein arginine N-methyltransferase 3 [Oryza sativa (japonica cultivar-group)] e_value:0</td><td>11673.m04392|LOC_Os07g44640|protein Zinc finger, C2H2 type, putative e_value:0</td><td>0.272</td><td>(0.1287 0.4729)</td><td>0.1287</td><td>0.4729</td></tr>
<tr><td>262</td><td>SET1_P24_CLUSTER_19841</td><td>gi|52548248|gb|AAU82111.1| leucine-rich repeat protein [Triticum aestivum] e_value:5.72945e-117</td><td>11669.m03301|LOC_Os03g32580|protein putative leucine-rich repeat protein e_value:1.09096e-106</td><td>0.154</td><td>(0.0688 0.4465)</td><td>0.0688</td><td>0.4465</td></tr>
<tr><td>263</td><td>WT005_E15_CLUSTER_33443</td><td>gi|62733303|gb|AAX95420.1| conserved hypothetical protein protein [Oryza sativa (japonica cultivar-group)] e_value:3.25235e-46</td><td>11687.m04518|LOC_Os11g48030|protein expressed protein e_value:3.09082e-48</td><td>0.207</td><td>(0.1280 0.6192)</td><td>0.128</td><td>0.6192</td></tr>
<tr><td>264</td><td>SET2_H09_CLUSTER_32238</td><td>gi|50511367|gb|AAT77290.1| putative ABA-responsive element-binding protein 3 [Oryza sativa (japonica cultivar-group)] e_value:1.2475e-68</td><td>11682.m03925|LOC_Os05g41070|protein bZIP transcription factor, putative e_value:3.74546e-70</td><td>0.227</td><td>(0.4223 1.8618)</td><td>0.4223</td><td>1.8618</td></tr>
<tr><td>265</td><td>SET5_J23_CLUSTER_21372</td><td>gi|50906183|ref|XP_464580.1| ZIGA2 protein-like [Oryza sativa (japonica cultivar-group)] >gi|49387911|dbj|BAD25011.1| ZIGA2 protein-like [Oryza sativa (japonica cultivar-group)] >gi|49387899|dbj|BAD25002.1| ZIGA2 protein-like [Oryza sativa (japonica</td><td>11668.m01074|LOC_Os02g11820|protein hypothetical protein e_value:0</td><td>0.363</td><td>(0.2008 0.5525)</td><td>0.2008</td><td>0.5525</td></tr>
<tr><td>266</td><td>WT004_D15_CLUSTER_33137</td><td>gi|23505917|gb|AAN28818.1| At4g21800/F17L22_260 [Arabidopsis thaliana] >gnl|BL_ORD_ID|1268155 gi|42572979|ref|NP_974586.1| ATP-binding family protein [Arabidopsis thaliana] >gnl|BL_ORD_ID|1268155 gi|15234595|ref|NP_193911.1| ATP-binding family prote</td><td>11669.m02022|LOC_Os03g19870|protein At4g21800/F17L22_260 e_value:0</td><td>0.079</td><td>(0.0464 0.5864)</td><td>0.0464</td><td>0.5864</td></tr>
<tr><td>267</td><td>WT005_D24_CLUSTER_28817</td><td>gi|34914614|ref|NP_918654.1| putative 26S proteasome subunit [Oryza sativa (japonica cultivar-group)] >gnl|BL_ORD_ID|1541332 gi|14495192|dbj|BAB60911.1| putative 26S proteasome subunit RPN9b [Oryza sativa (japonica cultivar-group)] >gnl|BL_ORD_ID|15</td><td>11667.m03094|LOC_Os01g32800|protein PCI domain, putative e_value:6.78773e-176</td><td>0.082</td><td>(0.1353 1.6519)</td><td>0.1353</td><td>1.6519</td></tr>
<tr><td>268</td><td>WT003_I01_CLUSTER_34899</td><td>gi|50942045|ref|XP_480550.1| putative GTP-binding protein [Oryza sativa (japonica cultivar-group)] >gnl|BL_ORD_ID|1602983 gi|51979651|ref|XP_507572.1| PREDICTED OSJNBb0094P23.28 gene product [Oryza sativa (japonica cultivar-group)] >gnl|BL_ORD_ID|16</td><td>11674.m00941|LOC_Os08g09940|protein expressed protein e_value:0</td><td>0.11</td><td>(0.0604 0.5509)</td><td>0.0604</td><td>0.5509</td></tr>
<tr><td>269</td><td>SET2_A16_CLUSTER_33199</td><td>gi|50929549|ref|XP_474302.1| OSJNBa0043A12.39 [Oryza sativa (japonica cultivar-group)] >gi|32488409|emb|CAE02834.1| OSJNBa0043A12.39 [Oryza sativa (japonica cultivar-group)] e_value:0</td><td>11670.m05689|LOC_Os04g57550|protein amine oxidase, putative e_value:0</td><td>0.103</td><td>(0.0878 0.8514)</td><td>0.0878</td><td>0.8514</td></tr>
<tr><td>270</td><td>WT002_D02_CLUSTER_32425</td><td>gi|50904583|ref|XP_463780.1| putative HECT ubiquitin-protein ligase 3 [Oryza sativa (japonica cultivar-group)] >gnl|BL_ORD_ID|1565973 gi|41053228|dbj|BAD08189.1| putative HECT ubiquitin-protein ligase 3 [Oryza sativa (japonica cultivar-group)] >gnl|</td><td>11668.m00019|LOC_Os02g01170|protein HECT-domain (ubiquitin-transferase), putative e_value:0</td><td>0.154</td><td>(0.0816 0.5317)</td><td>0.0816</td><td>0.5317</td></tr>
<tr><td>271</td><td>WT011_B01_CLUSTER_34486</td><td>gi|50919725|ref|XP_470223.1| Putative anion transporter [Oryza sativa] >gnl|BL_ORD_ID|1539279 gi|15451603|gb|AAK98727.1| Putative anion transporter [Oryza sativa] e_value:0</td><td>11669.m00485|LOC_Os03g05390|protein expressed protein e_value:0</td><td>0.072</td><td>(0.0671 0.9255)</td><td>0.0671</td><td>0.9255</td></tr>
<tr><td>272</td><td>WT002_L10_CLUSTER_34017</td><td>gi|50934039|ref|XP_476547.1| putative succinate dehydrogenase flavoprotein alpha subunit [Oryza sativa (japonica cultivar-group)] >gnl|BL_ORD_ID|1520659 gi|51978944|ref|XP_507349.1| PREDICTED P0507H12.6 gene product [Oryza sativa (japonica cultivar-gro</td><td>11673.m00334|LOC_Os07g04240|protein succinate dehydrogenase, flavoprotein subunit e_value:0</td><td>0.061</td><td>(0.0439 0.7231)</td><td>0.0439</td><td>0.7231</td></tr>
<tr><td>273</td><td>WT013_A19_CLUSTER_3257</td><td>gi|25406923|pir||F86200 protein F12K11.17 [imported] - Arabidopsis thaliana >gnl|BL_ORD_ID|297404 gi|6692688|gb|AAF24822.1| F12K11.17 [Arabidopsis thaliana] e_value:0</td><td>11667.m03307|LOC_Os01g34880|protein expressed protein e_value:0</td><td>0.046</td><td>(0.0312 0.6844)</td><td>0.0312</td><td>0.6844</td></tr>
<tr><td>274</td><td>SET1_K20_CLUSTER_31900</td><td>gi|50918609|ref|XP_469701.1| putative cop-coated vesicle membrane protein [Oryza sativa (japonica cultivar-group)] >gi|30017578|gb|AAP13000.1| putative cop-coated vesicle membrane protein [Oryza sativa (japonica cultivar-group)] e_value:1.86076e-</td><td>11669.m05395|LOC_Os03g53310|protein putative cop-coated vesicle membrane protein e_value:3.11053e-111</td><td>0.114</td><td>(0.0418 0.3650)</td><td>0.0418</td><td>0.365</td></tr>
<tr><td>275</td><td>SET3_F05_CLUSTER_32613</td><td>gi|50923815|ref|XP_472268.1| OSJNBa0044M19.2 [Oryza sativa (japonica cultivar-group)] >gi|32489985|emb|CAE05015.1| OSJNBa0044M19.2 [Oryza sativa (japonica cultivar-group)] e_value:0</td><td>11670.m03013|LOC_Os04g31620|protein Similar to contains similarity to transcription or splicing factor~gene_id:MWD22.25</td><td>0.196</td><td>(0.1308 0.6683)</td><td>0.1308</td><td>0.6683</td></tr>
<tr><td>276</td><td>WT013_B16_CLUSTER_19317</td><td>gi|50946857|ref|XP_482956.1| putative patatin [Oryza sativa (japonica cultivar-group)] >gnl|BL_ORD_ID|1609184 gi|42407856|dbj|BAD08998.1| putative patatin [Oryza sativa (japonica cultivar-group)] e_value:7.04522e-144</td><td>11674.m03704|LOC_Os08g37250|protein Patatin-like phospholipase, putative e_value:7.88524e-119</td><td>0.262</td><td>(0.2125 0.8103)</td><td>0.2125</td><td>0.8103</td></tr>
<tr><td>277</td><td>WT008_G17_CLUSTER_17153</td><td>gi|31429829|gb|AAP51824.1| putative peroxidase [Oryza sativa (japonica cultivar-group)] >gnl|BL_ORD_ID|2007776 gi|37530470|ref|NP_919537.1| putative peroxidase [Oryza sativa (japonica cultivar-group)] >gnl|BL_ORD_ID|2007776 gi|19920087|gb|AAM08519.1</td><td>11676.m00110|LOC_Os10g02070|protein Peroxidase, putative e_value:2.74004e-143</td><td>0.245</td><td>(0.1513 0.6171)</td><td>0.1513</td><td>0.6171</td></tr>
<tr><td>278</td><td>SET2_M19_CLUSTER_13670</td><td>gi|31432574|gb|AAP54189.1| unknown protein [Oryza sativa (japonica cultivar-group)] >gi|37535200|ref|NP_921902.1| unknown protein [Oryza sativa (japonica cultivar-group)] >gi|13489176|gb|AAK27810.1| unknown protein [Oryza sativa (japonica cultivar-g</td><td>11676.m02884|LOC_Os10g32850|protein expressed protein e_value:1.16597e-150</td><td>0.464</td><td>(0.2961 0.6386)</td><td>0.2961</td><td>0.6386</td></tr>
<tr><td>279</td><td>WT013_L03_CLUSTER_31438</td><td>gi|50919431|ref|XP_470112.1| unknown protein [Oryza sativa (japonica cultivar-group)] >gnl|BL_ORD_ID|1588184 gi|28876024|gb|AAO60033.1| unknown protein [Oryza sativa (japonica cultivar-group)] e_value:1.4176e-163</td><td>11669.m06134|LOC_Os03g60340|protein expressed protein e_value:3.28637e-165</td><td>0.482</td><td>(0.1529 0.3173)</td><td>0.1529</td><td>0.3173</td></tr>
<tr><td>280</td><td>WT004_J19_CLUSTER_31438</td><td>gi|50919431|ref|XP_470112.1| unknown protein [Oryza sativa (japonica cultivar-group)] >gnl|BL_ORD_ID|1588184 gi|28876024|gb|AAO60033.1| unknown protein [Oryza sativa (japonica cultivar-group)] e_value:5.97597e-163</td><td>11669.m06134|LOC_Os03g60340|protein expressed protein e_value:1.24882e-164</td><td>0.474</td><td>(0.1501 0.3168)</td><td>0.1501</td><td>0.3168</td></tr>
<tr><td>281</td><td>SET4_H11_CLUSTER_32366</td><td>gi|50905317|ref|XP_464147.1| putative 26S proteasome regulatory subunit S2 [Oryza sativa (japonica cultivar-group)] >gi|51963868|ref|XP_506717.1| PREDICTED OJ1008_C03.11 gene product [Oryza sativa (japonica cultivar-group)] >gi|45736056|dbj|BAD13082</td><td>11668.m00472|LOC_Os02g05340|protein Proteasome/cyclosome repeat, putative e_value:0</td><td>0.059</td><td>(0.0370 0.6269)</td><td>0.037</td><td>0.6269</td></tr>
<tr><td>282</td><td>WT008_G09_CLUSTER_901</td><td>gi|31431872|gb|AAP53584.1| putative cucumisin-like serine protease [Oryza sativa (japonica cultivar-group)] >gnl|BL_ORD_ID|2011029 gi|37533990|ref|NP_921297.1| putative cucumisin-like serine protease [Oryza sativa (japonica cultivar-group)] >gnl|BL_</td><td>11676.m02161|LOC_Os10g25450|protein putative cucumisin-like serine protease e_value:0</td><td>0.158</td><td>(0.0988 0.6256)</td><td>0.0988</td><td>0.6256</td></tr>
<tr><td>283</td><td>WT004_P05_CLUSTER_1048</td><td>gi|50907711|ref|XP_465344.1| putative cytosolic chaperonin delta-subunit [Oryza sativa (japonica cultivar-group)] >gnl|BL_ORD_ID|1569410 gi|46390985|dbj|BAD16520.1| putative cytosolic chaperonin delta-subunit [Oryza sativa (japonica cultivar-</td><td>11668.m02206|LOC_Os02g22780|protein T-complex protein 1, delta subunit e_value:0</td><td>0.062</td><td>(0.0435 0.7029)</td><td>0.0435</td><td>0.7029</td></tr>
<tr><td>284</td><td>WT007_O05_CLUSTER_29059</td><td>gi|62733239|gb|AAX95356.1| F22G5.1-related [Oryza sativa (japonica cultivar-group)] e_value:0</td><td>11687.m04494|LOC_Os11g47800|protein Similar to F22G5.1 e_value:0</td><td>0.346</td><td>(0.1844 0.5332)</td><td>0.1844</td><td>0.5332</td></tr>
<tr><td>285</td><td>SET1_N09_CLUSTER_32525</td><td>gi|56713105|emb|CAH04983.1| type 1 non-specific lipid transfer protein precursor [Triticum aestivum] e_value:2.6732e-56</td><td>11687.m00142|LOC_Os11g02350|protein lipid transfer protein e_value:1.11712e-37</td><td>0.596</td><td>(0.2643 0.4435)</td><td>0.2643</td><td>0.4435</td></tr>
<tr><td>286</td><td>WT003_E20_CLUSTER_31595</td><td>gi|7433275|pir||T06238 omega-3 fatty acid desaturase (EC 1.14.99.-) FAD3 - wheat >gnl|BL_ORD_ID|157178 gi|3157460|dbj|BAA28358.1| omega-3 fatty acid desaturase [Triticum aestivum] e_value:0</td><td>11686.m00038|LOC_Os12g01370|protein probable omega-3 fatty acid desaturase (EC 1.14.99.-) - rice e_value:0</td><td>0.179</td><td>(0.1082 0.6044)</td><td>0.1082</td><td>0.6044</td></tr>
<tr><td>287</td><td>WT003_L04_CLUSTER_18490</td><td>gi|55296552|dbj|BAD68966.1| unknown protein [Oryza sativa (japonica cultivar-group)] e_value:4.49896e-32</td><td>11667.m02879|LOC_Os01g29740|protein hypothetical protein e_value:8.55903e-34</td><td>0.591</td><td>(0.2283 0.3860)</td><td>0.2283</td><td>0.386</td></tr>
<tr><td>288</td><td>SET4_I23_CLUSTER_23111</td><td>gi|34898438|ref|NP_910565.1| EST AU070273(S21075) corresponds to a region of the predicted gene.~Similar to Arabidopsis thaliana chromosome 1 BAC F28K20 sequence, F28K20.12. (AC004793) [Oryza sativa (japonica cultivar-group)] e_value:3.50767e-52</td><td>11680.m00723|LOC_Os06g07760|protein ParB-like nuclease domain, putative e_value:2.1614e-60</td><td>0.144</td><td>(0.1004 0.6989)</td><td>0.1004</td><td>0.6989</td></tr>
<tr><td>289</td><td>WT006_G10_CLUSTER_29326</td><td>gi|38345758|emb|CAE03486.2| OSJNBa0065O17.11 [Oryza sativa (japonica cultivar-group)] >gnl|BL_ORD_ID|1522645 gi|50927893|ref|XP_473474.1| OSJNBa0065O17.11 [Oryza sativa (japonica cultivar-group)] e_value:0</td><td>11670.m04597|LOC_Os04g47120|protein Acyl-CoA thioesterase, putative e_value:0</td><td>0.096</td><td>(0.0462 0.4798)</td><td>0.0462</td><td>0.4798</td></tr>
<tr><td>290</td><td>WT005_A11_CLUSTER_23076</td><td>gi|34905120|ref|NP_913907.1| unknown protein [Oryza sativa (japonica cultivar-group)] >gnl|BL_ORD_ID|1609651 gi|28201290|dbj|BAC56799.1| unknown protein [Oryza sativa (japonica cultivar-group)] >gnl|BL_ORD_ID|1609651 gi|28411836|dbj|BAC57311.1| unkn</td><td>11681.m02953|LOC_Os09g32330|protein expressed protein e_value:1.7292e-85</td><td>0.316</td><td>(0.1672 0.5290)</td><td>0.1672</td><td>0.529</td></tr>
<tr><td>291</td><td>WT007_P13_CLUSTER_4857</td><td>gi|56784683|dbj|BAD81774.1| unknown protein [Oryza sativa (japonica cultivar-group)] >gnl|BL_ORD_ID|775569 gi|56784491|dbj|BAD82642.1| unknown protein [Oryza sativa (japonica cultivar-group)] e_value:2.37667e-170</td><td>11667.m06311|LOC_Os01g63060|protein expressed protein e_value:4.68547e-172</td><td>0.121</td><td>(0.0620 0.5119)</td><td>0.062</td><td>0.5119</td></tr>
<tr><td>292</td><td>WT002_J20_CLUSTER_5711</td><td>gi|31432701|gb|AAP54299.1| putative homeodomain leucine zipper protein [Oryza sativa (japonica cultivar-group)] >gnl|BL_ORD_ID|2012185 gi|37535420|ref|NP_922012.1| putative homeodomain leucine zipper protein [Oryza sativa (japonica cultivar-</td><td>11676.m02999|LOC_Os10g33960|protein putative homeodomain-leucine zipper protein e_value:0</td><td>0.072</td><td>(0.0353 0.4915)</td><td>0.0353</td><td>0.4915</td></tr>
</table>

PDF Complete
Your complimentary use period has ended. Thank you for using PDF Complete.
Click Here to upgrade to Unlimited Pages and Expanded Features

<table>
<tr><td>293</td><td>WT011_L14_CLUSTER_31467</td><td>gi|34895162|ref|NP_908906.1| putative serine/threonine protein phosphatase [Oryza sativa (japonica cultivar-group)] >gnl|BL_ORD_ID|1561066 gi|21104823|dbj|BAB93408.1| putative protein phosphatase PP1 [Oryza sativa (japonica cultivar-group)]</td><td>11667.m02413|LOC_Os01g24750|protein Ser/Thr protein phosphatase family protein, putative e_value:3.45101e-175</td><td>0.087</td><td>(0.0551 0.6310)</td><td>0.0551</td><td>0.631</td></tr>
<tr><td>294</td><td>WT008_M07_CLUSTER_24633</td><td>gi|50904859|ref|XP_463918.1| putative linalool synthase [Oryza sativa (japonica cultivar-group)] >gnl|BL_ORD_ID|1565871 gi|41052749|dbj|BAD07605.1| putative linalool synthase [Oryza sativa (japonica cultivar-group)] e_value:1.24005e-103</td><td>11668.m00209|LOC_Os02g02930|protein Terpene synthase family, metal binding domain, putative e_value:5.07159e-103</td><td>0.21</td><td>(0.2342 1.1172)</td><td>0.2342</td><td>1.1172</td></tr>
<tr><td>295</td><td>SET3_M15_CLUSTER_7645</td><td>No hits found</td><td>11687.m04184|LOC_Os11g44380|protein expressed protein e_value:1.44588e-12</td><td>1.01</td><td>(0.9559 0.9465)</td><td>0.9559</td><td>0.9465</td></tr>
<tr><td>296</td><td>WT011_I22_CLUSTER_34034</td><td>gi|56201987|dbj|BAD73494.1| putative non-green plastid inner envelope membrane protein [Oryza sativa (japonica cultivar-group)] >gnl|BL_ORD_ID|542369 gi|56201709|dbj|BAD73440.1| putative non-green plastid inner envelope membrane protein [Oryza sativa (</td><td>11667.m06038|LOC_Os01g60650|protein hypothetical protein e_value:1.77671e-99</td><td>0.234</td><td>(0.1280 0.5465)</td><td>0.128</td><td>0.5465</td></tr>
<tr><td>297</td><td>WT009_A19_CLUSTER_33594</td><td>gi|61657664|emb|CAI64809.1| putative glucan endo-1,3-beta-D-glucosidase [Triticum aestivum] e_value:1.75402e-175</td><td>11667.m07158|LOC_Os01g71380|protein Glycosyl hydrolases family 17 e_value:4.73254e-134</td><td>0.554</td><td>(0.1837 0.3318)</td><td>0.1837</td><td>0.3318</td></tr>
<tr><td>298</td><td>SET2_C10_CLUSTER_13693</td><td>gi|50253027|dbj|BAD29277.1| putative fertility restorer homologue A [Oryza sativa (japonica cultivar-group)] e_value:0</td><td>11681.m02264|LOC_Os09g25050|protein expressed protein</td><td>0.3</td><td>(0.1399 0.4671)</td><td>0.1399</td><td>0.4671</td></tr>
<tr><td>299</td><td>WT011_L16_CLUSTER_5728</td><td>gi|54291862|gb|AAV32230.1| putative dihydrolipoamide dehydrogenase [Oryza sativa (japonica cultivar-group)] >gnl|BL_ORD_ID|1427071 gi|45267881|gb|AAS55780.1| putative lipoamide dehydrogenase [Oryza sativa (japonica cultivar-group)] e_value:0</td><td>11667.m02297|LOC_Os01g23610|protein Pyridine nucleotide-disulphide oxidoreductase, putative e_value:0</td><td>0.086</td><td>(0.0659 0.7634)</td><td>0.0659</td><td>0.7634</td></tr>
<tr><td>300</td><td>WT004_F09_CLUSTER_21362</td><td>gi|50937953|ref|XP_478504.1| putative isocitrate lyase [Oryza sativa (japonica cultivar-group)] >gnl|BL_ORD_ID|1528176 gi|51963456|ref|XP_506381.1| PREDICTED OSJNBa0007H12.40 gene product [Oryza sativa (japonica cultivar-group)] >gnl|BL_ORD_ID|15281</td><td>11673.m03318|LOC_Os07g34520|protein isocitrate lyase e_value:0</td><td>0.146</td><td>(0.0520 0.3563)</td><td>0.052</td><td>0.3563</td></tr>
<tr><td>301</td><td>WT004_E01_CLUSTER_13026</td><td>gi|50511474|gb|AAT77396.1| unknown protein [Oryza sativa (japonica cultivar-group)] e_value:1.80348e-94</td><td>11682.m04688|LOC_Os05g48900|protein Fasciclin domain, putative e_value:4.59125e-96</td><td>0.641</td><td>(0.2122 0.3311)</td><td>0.2122</td><td>0.3311</td></tr>
<tr><td>302</td><td>WT002_M12_CLUSTER_31411</td><td>gi|15220921|ref|NP_175113.1| FF domain-containing protein / WW domain-containing protein [Arabidopsis thaliana] e_value:0</td><td>11667.m03297|LOC_Os01g34780|protein FF domain, putative e_value:0</td><td>0.203</td><td>(0.1536 0.7553)</td><td>0.1536</td><td>0.7553</td></tr>
<tr><td>303</td><td>WT002_A04_CLUSTER_5737</td><td>gi|50945431|ref|XP_482243.1| putative ABC transporter [Oryza sativa (japonica cultivar-group)] >gnl|BL_ORD_ID|1606103 gi|37806016|dbj|BAC99428.1| putative ABC transporter [Oryza sativa (japonica cultivar-group)] e_value:0</td><td>11674.m03026|LOC_Os08g30740|protein ABC transporter protein, putative e_value:0</td><td>0.096</td><td>(0.0497 0.5190)</td><td>0.0497</td><td>0.519</td></tr>
<tr><td>304</td><td>SET5_N09_CLUSTER_2690</td><td>gi|51091279|dbj|BAD35986.1| unknown protein [Oryza sativa (japonica cultivar-group)] e_value:2.17168e-165</td><td>11680.m00988|LOC_Os06g10210|protein expressed protein e_value:4.85272e-167</td><td>0.392</td><td>(0.1739 0.4437)</td><td>0.1739</td><td>0.4437</td></tr>
<tr><td>305</td><td>SET5_E17_CLUSTER_15820</td><td>gi|20152986|gb|AAM13449.1| chalcone isomerase [Hordeum vulgare subsp. vulgare] e_value:3.88255e-119</td><td>11687.m00151|LOC_Os11g02440|protein contains similarity to chalcone-flavonone isomerase e_value:2.50449e-16</td><td>0.525</td><td>(0.8759 1.6694)</td><td>0.8759</td><td>1.6694</td></tr>
<tr><td>306</td><td>SET4_J11_CLUSTER_5007</td><td>gi|62734659|gb|AAX96768.1| expressed protein [Oryza sativa (japonica cultivar-group)] >gi|62734658|gb|AAX96767.1| expressed protein [Oryza sativa (japonica cultivar-group)] e_value:1.90129e-33</td><td>11687.m01327|LOC_Os11g14190|protein expressed protein e_value:8.11523e-36</td><td>0.424</td><td>(0.1634 0.3849)</td><td>0.1634</td><td>0.3849</td></tr>
<tr><td>307</td><td>SET4_F11_CLUSTER_34306</td><td>gi|57899797|dbj|BAD87542.1| putative pectinacetylesterase precursor [Oryza sativa (japonica cultivar-group)] e_value:1.5385e-147</td><td>11667.m06715|LOC_Os01g66830|protein pectinacetylesterase, putative e_value:1.64987e-149</td><td>0.303</td><td>(0.2545 0.8390)</td><td>0.2545</td><td>0.839</td></tr>
<tr><td>308</td><td>SET2_J23_CLUSTER_3825</td><td>gi|34910666|ref|NP_916680.1| sulfate transporter-like protein [Oryza sativa (japonica cultivar-group)] >gi|15624010|dbj|BAB68064.1| putative plasma membrane sulphate transporter [Oryza sativa (japonica cultivar-group)] e_value:0</td><td>11667.m05106|LOC_Os01g52130|protein sulfate permease e_value:0</td><td>0.296</td><td>(0.1089 0.3675)</td><td>0.1089</td><td>0.3675</td></tr>
<tr><td>309</td><td>SET2_I14_CLUSTER_2189</td><td>gi|10177579|dbj|BAB10810.1| unnamed protein product [Arabidopsis thaliana] >gi|42568351|ref|NP_568663.2| 16S rRNA processing protein RimM family [Arabidopsis thaliana] e_value:8.6033e-118</td><td>11682.m04713|LOC_Os05g49130|protein hypothetical protein e_value:0</td><td>0.237</td><td>(0.1453 0.6144)</td><td>0.1453</td><td>0.6144</td></tr>
<tr><td>310</td><td>WT013_P10_CLUSTER_12124</td><td>gi|38345797|emb|CAE03569.2| OSJNBa0085I10.14 [Oryza sativa (japonica cultivar-group)] >gnl|BL_ORD_ID|1522685 gi|50928649|ref|XP_473852.1| OSJNBa0085I10.14 [Oryza sativa (japonica cultivar-group)] e_value:9.81448e-169</td><td>11670.m05101|LOC_Os04g52130|protein coproporphyrinogen III oxidase, aerobic, putative e_value:1.35717e-169</td><td>0.121</td><td>(0.0961 0.7940)</td><td>0.0961</td><td>0.794</td></tr>
<tr><td>311</td><td>WT013_P07_CLUSTER_5230</td><td>gi|50904859|ref|XP_463918.1| putative linalool synthase [Oryza sativa (japonica cultivar-group)] >gnl|BL_ORD_ID|1565871 gi|41052749|dbj|BAD07605.1| putative linalool synthase [Oryza sativa (japonica cultivar-group)] e_value:0</td><td>11668.m00209|LOC_Os02g02930|protein Terpene synthase family, metal binding domain, putative e_value:0</td><td>0.275</td><td>(0.2623 0.9525)</td><td>0.2623</td><td>0.9525</td></tr>
<tr><td>312</td><td>WT013_M19_CLUSTER_14904</td><td>gi|453244|emb|CAA54616.1| flavonoid 7-O-methyltransferase [Hordeum vulgare subsp. vulgare] e_value:1.59645e-120</td><td>11674.m03503|LOC_Os08g35310|protein methyltransferase e_value:4.60476e-97</td><td>0.211</td><td>(0.3307 1.5694)</td><td>0.3307</td><td>1.5694</td></tr>
<tr><td>313</td><td>WT013_I15_CLUSTER_23063</td><td>gi|50930223|ref|XP_474639.1| OSJNBa0039G19.9 [Oryza sativa (japonica cultivar-group)] >gnl|BL_ORD_ID|1594855 gi|38347149|emb|CAE02433.2| OSJNBa0039G19.9 [Oryza sativa (japonica cultivar-group)] e_value:1.89673e-94</td><td>11670.m00822|LOC_Os04g09590|protein Transferase family e_value:6.32758e-96</td><td>0.619</td><td>(0.4457 0.7202)</td><td>0.4457</td><td>0.7202</td></tr>
<tr><td>314</td><td>WT013_A15_CLUSTER_29208</td><td>gi|34908868|ref|NP_915781.1| putative subtilase [Oryza sativa (japonica cultivar-group)] e_value:0</td><td>11667.m05783|LOC_Os01g58290|protein Subtilase family, putative e_value:0</td><td>0.264</td><td>(0.1435 0.5434)</td><td>0.1435</td><td>0.5434</td></tr>
<tr><td>315</td><td>WT013_C01_CLUSTER_21998</td><td>gi|62733814|gb|AAX95923.1| transposon protein, putative, unclassified [Oryza sativa (japonica cultivar-group)] e_value:4.81158e-97</td><td>11687.m02820|LOC_Os11g30810|protein Chain A, Crystal Structure Of An Arabidopsis Thaliana Putative Steroid Sulphotransferase. e_value:1.59541e-101</td><td>0.472</td><td>(0.3288 0.6968)</td><td>0.3288</td><td>0.6968</td></tr>
<tr><td>316</td><td>WT012_F04_CLUSTER_3747</td><td>gi|56784876|dbj|BAD82147.1| phosphoribulokinase/uridine kinase-like [Oryza sativa (japonica cultivar-group)] >gnl|BL_ORD_ID|803737 gi|56202305|dbj|BAD73764.1| phosphoribulokinase/uridine kinase-like [Oryza sativa (japonica cultivar-group)] e_value:0</td><td>11667.m04763|LOC_Os01g48990|protein expressed protein e_value:0</td><td>0.242</td><td>(0.0938 0.3872)</td><td>0.0938</td><td>0.3872</td></tr>
<tr><td>317</td><td>WT010_K14_CLUSTER_8394</td><td>gi|34905156|ref|NP_913925.1| unknown protein [Oryza sativa (japonica cultivar-group)] >gnl|BL_ORD_ID|1609665 gi|51965022|ref|XP_507295.1| PREDICTED P0700D12.120 gene product [Oryza sativa (japonica cultivar-group)] >gnl|BL_ORD_ID|1609665 gi|28411850</td><td>11674.m04077|LOC_Os08g40630|protein mTERF, putative e_value:4.961e-161</td><td>0.398</td><td>(0.3640 0.9136)</td><td>0.364</td><td>0.9136</td></tr>
<tr><td>318</td><td>WT010_K01_CLUSTER_5887</td><td>gi|46394406|tpg|DAA05141.1| TPA: WRKY transcription factor 76 [Oryza sativa (indica cultivar-group)] e_value:5.84302e-110</td><td>11681.m02265|LOC_Os09g25060|protein WRKY DNA -binding domain, putative e_value:2.11753e-111</td><td>0.466</td><td>(0.2543 0.5453)</td><td>0.2543</td><td>0.5453</td></tr>
<tr><td>319</td><td>WT010_E17_CLUSTER_4876</td><td>gi|53792122|dbj|BAD52755.1| putative SAC domain protein 9 [Oryza sativa (japonica cultivar-group)] >gnl|BL_ORD_ID|258870 gi|53791358|dbj|BAD52604.1| putative SAC domain protein 9 [Oryza sativa (japonica cultivar-group)] e_value:0</td><td>11667.m02471|LOC_Os01g25330|protein WW domain, putative e_value:0</td><td>0.194</td><td>(0.1109 0.5716)</td><td>0.1109</td><td>0.5716</td></tr>
<tr><td>320</td><td>WT008_O20_CLUSTER_3703</td><td>gi|50930609|ref|XP_474832.1| OSJNBa0052O21.7 [Oryza sativa (japonica cultivar-group)] >gnl|BL_ORD_ID|1595132 gi|38345004|emb|CAD40022.2| OSJNBa0052O21.7 [Oryza sativa (japonica cultivar-group)] e_value:0</td><td>11674.m04302|LOC_Os08g42670|protein resistance protein, putative e_value:3.15609e-162</td><td>0.395</td><td>(0.6463 1.6383)</td><td>0.6463</td><td>1.6383</td></tr>
<tr><td>321</td><td>WT008_I06_CLUSTER_25041</td><td>gi|50932879|ref|XP_475967.1| unknown protein [Oryza sativa (japonica cultivar-group)] >gnl|BL_ORD_ID|1599528 gi|48843801|gb|AAT47060.1| unknown protein [Oryza sativa (japonica cultivar-group)] e_value:0</td><td>11682.m04888|LOC_Os05g50750|protein ATPase, AAA family, putative e_value:0</td><td>0.141</td><td>(0.0943 0.6703)</td><td>0.0943</td><td>0.6703</td></tr>
<tr><td>322</td><td>WT008_E11_CLUSTER_8186</td><td>gi|50938537|ref|XP_478796.1| putative adenosine-5'-phosphosulfate kinase [Oryza sativa (japonica cultivar-group)] >gnl|BL_ORD_ID|1529561 gi|34393551|dbj|BAC83149.1| putative adenosine-5'-phosphosulfate kinase [Oryza sativa (japonica</td><td>11687.m03899|LOC_Os11g41650|protein adenylylsulfate kinase, putative e_value:3.10193e-97</td><td>0.187</td><td>(0.2863 1.5345)</td><td>0.2863</td><td>1.5345</td></tr>
<tr><td>323</td><td>WT008_D07_CLUSTER_5406</td><td>gi|50916160|ref|XP_468544.1| putative nuclear protein NAP [Oryza sativa (japonica cultivar-group)] >gnl|BL_ORD_ID|1583762 gi|48716394|dbj|BAD23003.1| putative nuclear protein NAP [Oryza sativa (japonica cultivar-group)] e_value:0</td><td>11668.m05798|LOC_Os02g58460|protein expressed protein e_value:0</td><td>0.098</td><td>(0.0607 0.6214)</td><td>0.0607</td><td>0.6214</td></tr>
<tr><td>324</td><td>WT008_C16_CLUSTER_10371</td><td>gi|57900115|dbj|BAD88177.1| putative potassium transporter [Oryza sativa (japonica cultivar-group)] >gnl|BL_ORD_ID|972451 gi|57899652|dbj|BAD87321.1| putative potassium transporter [Oryza sativa (japonica cultivar-group)] e_value:0</td><td>11667.m07065|LOC_Os01g70490|protein potassium uptake protein e_value:0</td><td>0.183</td><td>(0.0864 0.4716)</td><td>0.0864</td><td>0.4716</td></tr>
<tr><td>325</td><td>WT007_O09_CLUSTER_18651</td><td>gi|34906258|ref|NP_914476.1| putative phytochrome P450 [Oryza sativa (japonica cultivar-group)] >gnl|BL_ORD_ID|1751392 gi|9081784|dbj|BAA99523.1| putative cytochrome P450 [Oryza sativa (japonica cultivar-group)] e_value:6.4298e-118</td><td>11669.m00406|LOC_Os03g04660|protein Cytochrome P450 e_value:0</td><td>0.31</td><td>(0.1815 0.5856)</td><td>0.1815</td><td>0.5856</td></tr>
<tr><td>326</td><td>WT007_I11_CLUSTER_29071</td><td>gi|34912664|ref|NP_917679.1| P0686E09.1 [Oryza sativa (japonica cultivar-group)] e_value:0</td><td>11667.m04152|LOC_Os01g42950|protein Protein kinase domain, putative e_value:0</td><td>0.169</td><td>(0.0756 0.4474)</td><td>0.0756</td><td>0.4474</td></tr>
<tr><td>327</td><td>WT007_F03_CLUSTER_27757</td><td>gi|32816615|gb|AAP88588.1| putative zinc transporter [Oryza sativa (japonica cultivar-group)] e_value:1.54322e-142</td><td>11673.m01242|LOC_Os07g12890|protein ZIP zinc/iron transport family e_value:6.449e-142</td><td>0.2</td><td>(0.1124 0.5612)</td><td>0.1124</td><td>0.5612</td></tr>
<tr><td>328</td><td>WT004_H06_CLUSTER_737</td><td>gi|49532749|dbj|BAD26705.1| Radc1 [Oryza sativa (japonica cultivar-group)] e_value:0</td><td>11669.m00857|LOC_Os03g08790|protein Eukaryotic aspartyl protease e_value:0</td><td>0.277</td><td>(0.0813 0.2938)</td><td>0.0813</td><td>0.2938</td></tr>
<tr><td>329</td><td>WT006_E06_CLUSTER_9387</td><td>gi|56785364|dbj|BAD82322.1| copine III-like [Oryza sativa (japonica cultivar-group)] >gnl|BL_ORD_ID|972832 gi|56785120|dbj|BAD82758.1| copine III-like [Oryza sativa (japonica cultivar-group)] e_value:0</td><td>11667.m06858|LOC_Os01g68060|protein hypothetical protein e_value:0</td><td>0.181</td><td>(0.1157 0.6385)</td><td>0.1157</td><td>0.6385</td></tr>
<tr><td>330</td><td>SET6_C13_CLUSTER_9387</td><td>gi|56785364|dbj|BAD82322.1| copine III-like [Oryza sativa (japonica cultivar-group)] >gi|56785120|dbj|BAD82758.1| copine III-like [Oryza sativa (japonica cultivar-group)] e_value:0</td><td>11667.m06858|LOC_Os01g68060|protein hypothetical protein e_value:0</td><td>0.181</td><td>(0.1157 0.6385)</td><td>0.1157</td><td>0.6385</td></tr>
<tr><td>331</td><td>SET4_M10_CLUSTER_16991</td><td>gi|56785379|dbj|BAD82337.1| unknown protein [Oryza sativa (japonica cultivar-group)] >gi|56784386|dbj|BAD82425.1| unknown protein [Oryza sativa (japonica cultivar-group)] e_value:2.5784e-18</td><td>11667.m06883|LOC_Os01g68300|protein hypothetical protein e_value:3.86688e-20</td><td>2.103</td><td>(0.2583 0.1228)</td><td>0.2583</td><td>0.1228</td></tr>
<tr><td>332</td><td>WT005_O23_CLUSTER_34264</td><td>gi|50933623|ref|XP_476339.1| putative sulfolipid synthase [Oryza sativa (japonica cultivar-group)] >gnl|BL_ORD_ID|1517660 gi|50508912|dbj|BAD31817.1| putative sulfolipid synthase [Oryza sativa (japonica cultivar-group)] e_value:0</td><td>11673.m00004|LOC_Os07g01030|protein glycosyl transferase, group 1 family protein, putative e_value:0</td><td>0.13</td><td>(0.0990 0.7620)</td><td>0.099</td><td>0.762</td></tr>
<tr><td>333</td><td>SET6_I24_CLUSTER_21248</td><td>gi|21668465|dbj|BAA07785.3| plastid omega-3 fatty acid desaturase [Triticum aestivum] e_value:0</td><td>11673.m04907|LOC_Os07g49310|protein Fatty acid desaturase, putative e_value:0</td><td>0.144</td><td>(0.1232 0.8554)</td><td>0.1232</td><td>0.8554</td></tr>
<tr><td>334</td><td>WT009_I11_CLUSTER_34579</td><td>gi|51979667|ref|XP_507580.1| PREDICTED OSJNBb0011E04.125 gene product [Oryza sativa (japonica cultivar-group)] >gnl|BL_ORD_ID|1605824 gi|50945155|ref|XP_482105.1| putative oxidoreductase, zinc-binding [Oryza sativa (japonica cultivar-group)] >gnl|BL</td><td>11674.m02863|LOC_Os08g29170|protein oxidoreductase, zinc-binding dehydrogenase family, putative e_value:2.63717e-164</td><td>0.27</td><td>(0.1246 0.4610)</td><td>0.1246</td><td>0.461</td></tr>
<tr><td>335</td><td>WT010_C08_CLUSTER_16598</td><td>gi|56201917|dbj|BAD73367.1| unknown protein [Oryza sativa (japonica cultivar-group)] >gnl|BL_ORD_ID|542322 gi|56201864|dbj|BAD73314.1| unknown protein [Oryza sativa (japonica cultivar-group)] e_value:3.52879e-155</td><td>11667.m04683|LOC_Os01g48300|protein At2g31040/T16B12.15 e_value:8.39363e-157</td><td>0.263</td><td>(0.1289 0.4904)</td><td>0.1289</td><td>0.4904</td></tr>
</table>

PDF Complete
Your complimentary use period has ended. Thank you for using PDF Complete.
Click Here to upgrade to Unlimited Pages and Expanded Features

<table>
<tr><td>336</td><td>SET2_I18_CLUSTER_22751</td><td>gi|55583334|dbj|BAD69588.1| ARF-GAP [Arabidopsis thaliana] e_value:0</td><td>11681.m03040|LOC_Os09g33600|protein Putative GTP-ase activating protein for Arf e_value:0</td><td>0.1</td><td>(0.0634 0.6331)</td><td>0.0634</td><td>0.6331</td></tr>
<tr><td>337</td><td>WT004_I11_CLUSTER_27952</td><td>gi|53791962|dbj|BAD54224.1| putative 4-methyl-5(B-hydroxyethyl)-thiazol monophosphate biosynthesis enzyme [Oryza sativa (japonica cultivar-group)] e_value:0</td><td>11680.m03285|LOC_Os06g34040|protein DJ-1 family protein, putative e_value:0</td><td>0.11</td><td>(0.0560 0.5071)</td><td>0.056</td><td>0.5071</td></tr>
<tr><td>338</td><td>WT010_O01_CLUSTER_34974</td><td>gi|21281251|gb|AAM44965.1| putative ABC transporter ATPase [Arabidopsis thaliana] >gnl|BL_ORD_ID|1208477 gi|14334904|gb|AAK59630.1| putative ABC transporter ATPase [Arabidopsis thaliana] >gnl|BL_ORD_ID|1208477</td><td>11669.m02204|LOC_Os03g21490|protein FeS assembly ATPase SufC, putative e_value:1.00623e-128</td><td>0.275</td><td>(0.1433 0.5216)</td><td>0.1433</td><td>0.5216</td></tr>
<tr><td>339</td><td>WT013_L17_CLUSTER_15641</td><td>gi|50725145|dbj|BAD33762.1| putative 6-phosphogluconolactonase [Oryza sativa (japonica cultivar-group)] e_value:8.69819e-125</td><td>11681.m03189|LOC_Os09g35970|protein 6-phosphogluconolactonase e_value:2.24751e-126</td><td>0.189</td><td>(0.1040 0.5504)</td><td>0.104</td><td>0.5504</td></tr>
<tr><td>340</td><td>WT002_L01_CLUSTER_32440</td><td>gi|50878369|gb|AAT85144.1| unknown protein [Oryza sativa (japonica cultivar-group)] e_value:1.12939e-131</td><td>11682.m03118|LOC_Os05g33280|protein expressed protein e_value:3.59593e-133</td><td>0.564</td><td>(0.0825 0.1461)</td><td>0.0825</td><td>0.1461</td></tr>
<tr><td>341</td><td>WT013_I05_CLUSTER_21435</td><td>gi|50909971|ref|XP_466474.1| serine/threonine-protein kinase Nek4-like [Oryza sativa (japonica cultivar-group)] >gnl|BL_ORD_ID|1574654 gi|46805153|dbj|BAD17425.1| serine/threonine-protein kinase Nek4-like [Oryza sativa (japonica cultivar-group)]</td><td>11668.m03611|LOC_Os02g37830|protein Protein kinase domain, putative e_value:0</td><td>0.196</td><td>(0.1006 0.5122)</td><td>0.1006</td><td>0.5122</td></tr>
<tr><td>342</td><td>WT004_B09_CLUSTER_33574</td><td>gi|50929479|ref|XP_474267.1| OSJNBa0043A12.4 [Oryza sativa (japonica cultivar-group)] >gnl|BL_ORD_ID|1594397 gi|32488374|emb|CAE02799.1| OSJNBa0043A12.4 [Oryza sativa (japonica cultivar-group)] e_value:4.36703e-37</td><td>11676.m03531|LOC_Os10g38870|protein expressed protein e_value:8.68169e-39</td><td>0.496</td><td>(0.2858 0.5757)</td><td>0.2858</td><td>0.5757</td></tr>
<tr><td>343</td><td>WT005_H09_CLUSTER_6812</td><td>gi|57900109|dbj|BAD88171.1| unknown protein [Oryza sativa (japonica cultivar-group)] e_value:1.84145e-93</td><td>11667.m07055|LOC_Os01g70400|protein expressed protein e_value:4.30001e-95</td><td>0.303</td><td>(0.1573 0.5185)</td><td>0.1573</td><td>0.5185</td></tr>
<tr><td>344</td><td>WT002_G21_CLUSTER_31190</td><td>gi|34897384|ref|NP_910038.1| unknown protein [Oryza sativa (japonica cultivar-group)] >gnl|BL_ORD_ID|1588255 gi|27545037|gb|AAO18443.1| unknown protein [Oryza sativa (japonica cultivar-group)] e_value:4.51681e-138</td><td>11669.m06200|LOC_Os03g60920|protein expressed protein e_value:8.74051e-140</td><td>0.117</td><td>(0.0717 0.6128)</td><td>0.0717</td><td>0.6128</td></tr>
<tr><td>345</td><td>WT003_K10_CLUSTER_29124</td><td>gi|51090894|dbj|BAD35467.1| putative ids-4 protein [Oryza sativa (japonica cultivar-group)] e_value:1.35507e-130</td><td>11680.m03912|LOC_Os06g40120|protein SPX domain, putative e_value:3.1434e-132</td><td>0.139</td><td>(0.0883 0.6364)</td><td>0.0883</td><td>0.6364</td></tr>
<tr><td>346</td><td>SET2_J01_CLUSTER_8355</td><td>gi|51451356|gb|AAU03111.1| putative cytochrome P450 [Oryza sativa (japonica cultivar-group)] e_value:0</td><td>11682.m04180|LOC_Os05g43910|protein cytochrome P-450LXXIA1 [similarity] - avocado e_value:0</td><td>0.336</td><td>(0.2158 0.6424)</td><td>0.2158</td><td>0.6424</td></tr>
<tr><td>347</td><td>SET2_O15_CLUSTER_17787</td><td>gi|56783726|dbj|BAD81138.1| unc-84 homolog B -like [Oryza sativa (japonica cultivar-group)] e_value:3.45291e-177</td><td>11667.m01629|LOC_Os01g16220|protein expressed protein e_value:1.02212e-178</td><td>0.234</td><td>(0.1522 0.6516)</td><td>0.1522</td><td>0.6516</td></tr>
<tr><td>348</td><td>WT013__C06_CLUSTER_33781</td><td>gi|37718880|gb|AAR01751.1| putative AP2 domain transcription factor [Oryza sativa (japonica cultivar-group)] >gnl|BL_ORD_ID|2260650 gi|50919391|ref|XP_470092.1| putative AP2 domain transcription factor [Oryza sativa (japonica cultivar-group)] >gnl|B</td><td>11669.m06110|LOC_Os03g60120|protein putative AP2 domain transcription factor e_value:4.70019e-48</td><td>0.44</td><td>(0.3308 0.7510)</td><td>0.3308</td><td>0.751</td></tr>
<tr><td>349</td><td>WT002__C12_CLUSTER_17800</td><td>gi|50920095|ref|XP_470408.1| putative phytase [Oryza sativa (japonica cultivar-group)] >gnl|BL_ORD_ID|2402434 gi|29244681|gb|AAO73273.1| putative phytase [Oryza sativa (japonica cultivar-group)] >gnl|BL_ORD_ID|2402434 gi|28269395|gb|AAO37938.1| puta</td><td>11669.m06466|LOC_Os03g63080|protein putative phytase e_value:0</td><td>0.178</td><td>(0.0695 0.3899)</td><td>0.0695</td><td>0.3899</td></tr>
<tr><td>350</td><td>SET6_G12_CLUSTER_17800</td><td>gi|50920095|ref|XP_470408.1| putative phytase [Oryza sativa (japonica cultivar-group)] >gi|29244681|gb|AAO73273.1| putative phytase [Oryza sativa (japonica cultivar-group)] >gi|28269395|gb|AAO37938.1| putative phytase [Oryza sativa (japonica cultiva</td><td>11669.m06466|LOC_Os03g63080|protein putative phytase e_value:0</td><td>0.253</td><td>(0.1037 0.4095)</td><td>0.1037</td><td>0.4095</td></tr>
<tr><td>351</td><td>WT008_J14_CLUSTER_19078</td><td>gi|37538062|gb|AAQ92972.1| CTD-phosphatase-like protein [Hordeum vulgare subsp. vulgare] >gnl|BL_ORD_ID|2253343 gi|37538060|gb|AAQ92971.1| CTD-phosphatase-like protein [Hordeum vulgare subsp. vulgare] e_value:0</td><td>11667.m04599|LOC_Os01g47540|protein NLI interacting factor-like phosphatase, putative e_value:1.42956e-172</td><td>0.31</td><td>(0.1635 0.5268)</td><td>0.1635</td><td>0.5268</td></tr>
<tr><td>352</td><td>WT009_H06_CLUSTER_34156</td><td>gi|1167953|gb|AAA87041.1| putative 32.6 kDa jasmonate-induced protein [Hordeum vulgare] >gnl|BL_ORD_ID|699034 gi|2465426|gb|AAB72096.1| 32 kDa protein [Hordeum vulgare] >gnl|BL_ORD_ID|699034 gi|7489385|pir||T04374 jakalin homolog - barley e_value:6.</td><td>11686.m00934|LOC_Os12g09700|protein jacalin homolog - barley e_value:2.16934e-53</td><td>0.346</td><td>(0.5641 1.6295)</td><td>0.5641</td><td>1.6295</td></tr>
<tr><td>353</td><td>WT009_G05_CLUSTER_34156</td><td>gi|1167953|gb|AAA87041.1| putative 32.6 kDa jasmonate-induced protein [Hordeum vulgare] >gnl|BL_ORD_ID|699034 gi|2465426|gb|AAB72096.1| 32 kDa protein [Hordeum vulgare] >gnl|BL_ORD_ID|699034 gi|7489385|pir||T04374 jakalin homolog - barley e_value:6.</td><td>11686.m00934|LOC_Os12g09700|protein jacalin homolog - barley e_value:2.16934e-53</td><td>0.346</td><td>(0.5641 1.6295)</td><td>0.5641</td><td>1.6295</td></tr>
<tr><td>354</td><td>WT013_F17_CLUSTER_21504</td><td>gi|6706413|emb|CAB66099.1| putative protein [Arabidopsis thaliana] >gnl|BL_ORD_ID|1451110 gi|53828547|gb|AAU94383.1| At3g57420 [Arabidopsis thaliana] >gnl|BL_ORD_ID|1451110 gi|59958348|gb|AAX12884.1| At3g57420 [Arabidopsis thaliana] >gnl|BL_ORD_I</td><td>11682.m03036|LOC_Os05g32500|protein expressed protein e_value:0</td><td>0.107</td><td>(0.0644 0.5999)</td><td>0.0644</td><td>0.5999</td></tr>
<tr><td>355</td><td>WT003_G04_CLUSTER_6594</td><td>gi|55770537|ref|XP_550259.1| unknown protein [Oryza sativa (japonica cultivar-group)] >gnl|BL_ORD_ID|1753655 gi|55297508|dbj|BAD68310.1| unknown protein [Oryza sativa (japonica cultivar-group)] e_value:2.15562e-165</td><td>11682.m02329|LOC_Os05g25180|protein Zinc finger, C3HC4 type (RING finger), putative e_value:0</td><td>0.157</td><td>(0.0721 0.4603)</td><td>0.0721</td><td>0.4603</td></tr>
<tr><td>356</td><td>WT004_L11_CLUSTER_32991</td><td>gi|34907604|ref|NP_915149.1| putative calreticulin [Oryza sativa (japonica cultivar-group)] >gnl|BL_ORD_ID|1559298 gi|21952848|dbj|BAC06263.1| putative calreticulin [Oryza sativa (japonica cultivar-group)] e_value:0</td><td>11682.m04103|LOC_Os05g43170|protein calreticulin 3 e_value:0</td><td>0.107</td><td>(0.0944 0.8787)</td><td>0.0944</td><td>0.8787</td></tr>
<tr><td>357</td><td>SET4_H07_CLUSTER_34954</td><td>gi|1041425|emb|CAA63140.1| glutamyl-tRNA reductase [Hordeum vulgare subsp. vulgare] >gi|2495156|sp|Q42843|HEM11_HORVU Glutamyl-tRNA reductase 1, chloroplast precursor (GluTR) >gi|2967441|dbj|BAA25167.1| glutamyl-tRNA reductase [Hordeum vulgare</td><td>11676.m03205|LOC_Os10g35840|protein putative glutamyl-tRNA reductase e_value:0</td><td>0.052</td><td>(0.0223 0.4252)</td><td>0.0223</td><td>0.4252</td></tr>
<tr><td>358</td><td>WT010_E01_CLUSTER_34736</td><td>gi|45268533|gb|AAS55889.1| plasma membrane H+-ATPase [Triticum aestivum] >gnl|BL_ORD_ID|294543 gi|50400847|sp|P83970|PMA1_WHEAT Plasma membrane ATPase (Proton pump) e_value:0</td><td>11670.m05537|LOC_Os04g56160|protein plasma-membrane proton-efflux P-type ATPase e_value:0</td><td>0.072</td><td>(0.0398 0.5512)</td><td>0.0398</td><td>0.5512</td></tr>
<tr><td>359</td><td>SET1_M20_CLUSTER_12425</td><td>gi|51243724|gb|AAT99566.1| metallothionein [Triticum turgidum subsp. durum] >gi|51243722|gb|AAT99565.1| metallothionein [Triticum aestivum] >gi|51243720|gb|AAT99564.1| metallothionein [Triticum turgidum subsp. durum] >gi|51243718|gb|AAT99563.1| m</td><td>11669.m01806|LOC_Os03g17870|protein Metallothionein e_value:2.96328e-23</td><td>0.495</td><td>(0.1877 0.3797)</td><td>0.1877</td><td>0.3797</td></tr>
<tr><td>360</td><td>SET1_K06_CLUSTER_12425</td><td>gi|51243724|gb|AAT99566.1| metallothionein [Triticum turgidum subsp. durum] >gi|51243722|gb|AAT99565.1| metallothionein [Triticum aestivum] >gi|51243720|gb|AAT99564.1| metallothionein [Triticum turgidum subsp. durum] >gi|51243718|gb|AAT99563.1| m</td><td>11669.m01806|LOC_Os03g17870|protein Metallothionein e_value:2.96328e-23</td><td>0.509</td><td>(0.2002 0.3930)</td><td>0.2002</td><td>0.393</td></tr>
<tr><td>361</td><td>WT012_O20_CLUSTER_15102</td><td>gi|55775444|ref|XP_550648.1| cytokinin-regulated kinase-like [Oryza sativa (japonica cultivar-group)] >gnl|BL_ORD_ID|1752707 gi|55297279|dbj|BAD69064.1| cytokinin-regulated kinase-like [Oryza sativa (japonica cultivar-group)] >gnl|BL_ORD_ID|1752707</td><td>11686.m03069|LOC_Os12g31560|protein Protein kinase domain, putative e_value:3.31899e-139</td><td>0.329</td><td>(0.3442 1.0471)</td><td>0.3442</td><td>1.0471</td></tr>
<tr><td>362</td><td>WT010__C24_CLUSTER_26297</td><td>gi|50941685|ref|XP_480370.1| unknown protein [Oryza sativa (japonica cultivar-group)] >gnl|BL_ORD_ID|1602786 gi|46805054|dbj|BAD17035.1| unknown protein [Oryza sativa (japonica cultivar-group)] e_value:1.26598e-23</td><td>11669.m00983|LOC_Os03g10390|protein expressed protein e_value:3.70577e-85</td><td>0.378</td><td>(0.2690 0.7113)</td><td>0.269</td><td>0.7113</td></tr>
<tr><td>363</td><td>WT002_M09_CLUSTER_7312</td><td>gi|50933399|ref|XP_476227.1| putative tonoplast membrane integral protein [Oryza sativa (japonica cultivar-group)] >gnl|BL_ORD_ID|1600954 gi|46485863|gb|AAS98488.1| putative tonoplast membrane integral protein [Oryza sativa (japonica cultivar-</td><td>11682.m01314|LOC_Os05g14240|protein Similar to tonoplast membrane integral protein ZmTIP4-2 e_value:6.68624e-117</td><td>0.243</td><td>(0.1049 0.4317)</td><td>0.1049</td><td>0.4317</td></tr>
<tr><td>364</td><td>SET6_A11_CLUSTER_7312</td><td>gi|50933399|ref|XP_476227.1| putative tonoplast membrane integral protein [Oryza sativa (japonica cultivar-group)] >gi|46485863|gb|AAS98488.1| putative tonoplast membrane integral protein [Oryza sativa (japonica cultivar-group)] e_value:1.0111e-115</td><td>11682.m01314|LOC_Os05g14240|protein Similar to tonoplast membrane integral protein ZmTIP4-2 e_value:2.29803e-117</td><td>0.251</td><td>(0.1012 0.4028)</td><td>0.1012</td><td>0.4028</td></tr>
<tr><td>365</td><td>WT002_K19_CLUSTER_31451</td><td>gi|46095323|gb|AAS80159.1| thylakoid ascorbate peroxidase [Triticum aestivum] >gnl|BL_ORD_ID|1963970 gi|46095321|gb|AAS80158.1| thylakoid ascorbate peroxidase [Triticum aestivum] e_value:0</td><td>11668.m03298|LOC_Os02g34810|protein Peroxidase, putative e_value:0</td><td>0.16</td><td>(0.1069 0.6684)</td><td>0.1069</td><td>0.6684</td></tr>
<tr><td>366</td><td>SET5_L13_CLUSTER_16528</td><td>gi|50932201|ref|XP_475628.1| putative dihydrolipoamide dehydrogenase [Oryza sativa (japonica cultivar-group)] e_value:0</td><td>11667.m02185|LOC_Os01g22520|protein dihydrolipoamide dehydrogenase e_value:0</td><td>0.059</td><td>(0.0640 1.0832)</td><td>0.064</td><td>1.0832</td></tr>
<tr><td>367</td><td>WT006_A14_CLUSTER_34139</td><td>gi|51038249|gb|AAT94052.1| putative peroxidase [Oryza sativa (japonica cultivar-group)] e_value:3.87046e-136</td><td>11682.m00385|LOC_Os05g04500|protein peroxidase prx12 precursor e_value:1.06228e-137</td><td>0.317</td><td>(0.2087 0.6583)</td><td>0.2087</td><td>0.6583</td></tr>
<tr><td>368</td><td>WT005_M02_CLUSTER_28799</td><td>gi|15290589|gb|AAK94904.1| seven transmembrane-spanning protein [Triticum aestivum] e_value:0</td><td>11669.m00304|LOC_Os03g03700|protein Mlo family e_value:0</td><td>0.209</td><td>(0.1522 0.7279)</td><td>0.1522</td><td>0.7279</td></tr>
<tr><td>369</td><td>WT013_L09_CLUSTER_32048</td><td>gi|50934331|ref|XP_476693.1| unknown protein [Oryza sativa (japonica cultivar-group)] >gnl|BL_ORD_ID|1521647 gi|33146734|dbj|BAC79637.1| unknown protein [Oryza sativa (japonica cultivar-group)] >gnl|BL_ORD_ID|1521647 gi|50509615|dbj|BAD31445.1| unkn</td><td>11673.m00575|LOC_Os07g06550|protein hypothetical protein e_value:0</td><td>0.151</td><td>(0.0895 0.5917)</td><td>0.0895</td><td>0.5917</td></tr>
<tr><td>370</td><td>WT013_K09_CLUSTER_32048</td><td>gi|50934331|ref|XP_476693.1| unknown protein [Oryza sativa (japonica cultivar-group)] >gnl|BL_ORD_ID|1521647 gi|33146734|dbj|BAC79637.1| unknown protein [Oryza sativa (japonica cultivar-group)] >gnl|BL_ORD_ID|1521647 gi|50509615|dbj|BAD31445.1| unkn</td><td>11673.m00575|LOC_Os07g06550|protein hypothetical protein e_value:0</td><td>0.151</td><td>(0.0895 0.5917)</td><td>0.0895</td><td>0.5917</td></tr>
<tr><td>371</td><td>WT004_B16_CLUSTER_33842</td><td>gi|20799723|gb|AAM28624.1| adenosine monophosphate binding protein 7 AMPBP7 [Arabidopsis thaliana] >gnl|BL_ORD_ID|2057088 gi|19699128|gb|AAL90930.1| AT3g16910/K14A17_3 [Arabidopsis thaliana] >gnl|BL_ORD_ID|2057088 gi|18086379|gb|AAL57649.1|</td><td>11669.m01952|LOC_Os03g19240|protein adenosine monophosphate binding protein 7 AMPBP7 e_value:0</td><td>0.093</td><td>(0.0519 0.5556)</td><td>0.0519</td><td>0.5556</td></tr>
<tr><td>372</td><td>WT010_J21_CLUSTER_23120</td><td>gi|34912248|ref|NP_917471.1| cytosolic aldehyde dehydrogenase [Oryza sativa (japonica cultivar-group)] >gnl|BL_ORD_ID|1542331 gi|14164407|dbj|BAB55806.1| putative aldehyde dehydrogenase (NAD+) [Oryza sativa (japonica cultivar-group)] >gnl|BL_ORD_ID|</td><td>11667.m03934|LOC_Os01g40860|protein aldehyde dehydrogenase (NAD) family protein e_value:0</td><td>0.223</td><td>(0.1162 0.5200)</td><td>0.1162</td><td>0.52</td></tr>
<tr><td>373</td><td>SET4_A18_CLUSTER_4219</td><td>gi|57900406|dbj|BAD87642.1| putative dicarboxylate transporter [Oryza sativa (japonica cultivar-group)] >gi|57899246|dbj|BAD87491.1| putative dicarboxylate transporter [Oryza sativa (japonica cultivar-group)] e_value:0</td><td>11667.m03589|LOC_Os01g37590|protein POT family, putative e_value:0</td><td>0.258</td><td>(0.1004 0.3892)</td><td>0.1004</td><td>0.3892</td></tr>
<tr><td>374</td><td>WT005_F23_CLUSTER_34581</td><td>gi|38570261|gb|AAR24582.1| chloroplast Ptr ToxA-binding protein [Triticum aestivum] e_value:1.46715e-158</td><td>11673.m03609|LOC_Os07g37250|protein expressed protein e_value:1.95271e-139</td><td>0.099</td><td>(0.0639 0.6469)</td><td>0.0639</td><td>0.6469</td></tr>
<tr><td>375</td><td>WT008_F05_CLUSTER_28317</td><td>gi|9837152|gb|AAG00450.1| oxidase [Triticum aestivum] e_value:6.54306e-124</td><td>11670.m05665|LOC_Os04g57320|protein Alternative oxidase, putative e_value:1.47947e-112</td><td>0.333</td><td>(0.1628 0.4890)</td><td>0.1628</td><td>0.489</td></tr>
<tr><td>376</td><td>SET1_D12_CLUSTER_19845</td><td>gi|34894580|ref|NP_908615.1| zinc-induced protein-like [Oryza sativa (japonica cultivar-group)] e_value:1.38273e-23</td><td>11667.m02053|LOC_Os01g21250|protein Late embryogenesis abundant protein e_value:1.7875e-25</td><td>0.371</td><td>(0.1797 0.4840)</td><td>0.1797</td><td>0.484</td></tr>
<tr><td>377</td><td>WT009_B22_CLUSTER_34091</td><td>gi|23308263|gb|AAN18101.1| At1g03250/F15K9_13 [Arabidopsis thaliana] >gnl|BL_ORD_ID|1237096 gi|21593601|gb|AAM65568.1| unknown [Arabidopsis thaliana] >gnl|BL_ORD_ID|1237096 gi|22022560|gb|AAM83237.1| At1g03250/F15K9_13 [Arabidopsis thaliana] >gnl</td><td>11686.m00433|LOC_Os12g05000|protein At1g03250/F15K9_13 e_value:1.83468e-111</td><td>0.169</td><td>(0.0987 0.5838)</td><td>0.0987</td><td>0.5838</td></tr>
</table>

PDF Complete
Your complimentary use period has ended. Thank you for using PDF Complete.
Click Here to upgrade to Unlimited Pages and Expanded Features

<table>
<tr><th></th><th></th><th></th><th></th><th></th><th></th><th></th><th></th></tr>
<tr><td>378</td><td>WT006_M20_CLUSTER_34065</td><td>gi|55775318|ref|XP_550585.1| LMBR1 integral membrane family protein-like [Oryza sativa (japonica cultivar-group)] >gnl|BL_ORD_ID|1752842 gi|55296214|dbj|BAD67932.1| LMBR1 integral membrane family protein-like [Oryza sativa (japonica cultivar-group)] &g</td><td>11680.m00288|LOC_Os06g03760|protein LMBR1-like conserved region e_value:0</td><td>0.181</td><td>(0.0558 0.3080)</td><td>0.0558</td><td>0.308</td></tr>
<tr><td>379</td><td>WT004_J17_CLUSTER_34117</td><td>gi|1924921|emb|CAA69644.1| hydroxyacylglutathione hydrolase [Arabidopsis thaliana] >gnl|BL_ORD_ID|1714922 gi|6630545|gb|AAF19564.1| hydroxyacylglutathione hydrolase cytoplasmic (glyoxalase II) (GLX II) [Arabidopsis thaliana] >gnl|BL_ORD_ID|1714922 g</td><td>11669.m02200|LOC_Os03g21460|protein hydroxyacylglutathione hydrolase cytoplasmic (ec 3.1.2.6) (glyoxalaseii) (glx ii) e_value:1.32143e-136</td><td>0.128</td><td>(0.0584 0.4553)</td><td>0.0584</td><td>0.4553</td></tr>
<tr><td>380</td><td>WT002_G09_CLUSTER_32334</td><td>gi|7259224|emb|CAB77551.1| Toc34-2 protein [Zea mays] e_value:8.91244e-163</td><td>11669.m01344|LOC_Os03g13730|protein Toc34-1 protein e_value:3.35784e-170</td><td>0.107</td><td>(0.0509 0.4767)</td><td>0.0509</td><td>0.4767</td></tr>
<tr><td>381</td><td>SET6_E10_CLUSTER_26104</td><td>gi|50948587|ref|XP_483821.1| unknown protein [Oryza sativa (japonica cultivar-group)] >gi|45735910|dbj|BAD12942.1| unknown protein [Oryza sativa (japonica cultivar-group)] e_value:9.64375e-61</td><td>11674.m04562|LOC_Os08g45040|protein expressed protein e_value:1.56254e-60</td><td>0.191</td><td>(0.1031 0.5387)</td><td>0.1031</td><td>0.5387</td></tr>
<tr><td>382</td><td>WT002_H22_CLUSTER_33680</td><td>gi|31433719|gb|AAP55198.1| putative Clp protease [Oryza sativa (japonica cultivar-group)] >gnl|BL_ORD_ID|2013210 gi|37537218|ref|NP_922912.1| putative Clp protease [Oryza sativa (japonica cultivar-group)] >gnl|BL_ORD_ID|2013210 gi|12039365|gb|AAG461</td><td>11676.m04007|LOC_Os10g43050|protein putative Clp protease e_value:4.42984e-118</td><td>0.24</td><td>(0.1430 0.5945)</td><td>0.143</td><td>0.5945</td></tr>
<tr><td>383</td><td>SET5_M23_CLUSTER_9297</td><td>gi|51979387|ref|XP_507540.1| PREDICTED OSJNBa0054K20.23 gene product [Oryza sativa (japonica cultivar-group)] >gi|51964464|ref|XP_507017.1| PREDICTED OSJNBa0054K20.23 gene product [Oryza sativa (japonica cultivar-group)] e_value:1.92792e-</td><td>11667.m05418|LOC_Os01g54960|protein hypothetical protein e_value:6.05443e-101</td><td>0.217</td><td>(0.1480 0.6826)</td><td>0.148</td><td>0.6826</td></tr>
<tr><td>384</td><td>WT002_I19_CLUSTER_33232</td><td>gi|51535194|dbj|BAD38167.1| putative leucine zipper protein [Oryza sativa (japonica cultivar-group)] e_value:2.48643e-133</td><td>11680.m01243|LOC_Os06g12690|protein expressed protein e_value:4.03149e-135</td><td>0.097</td><td>(0.0447 0.4583)</td><td>0.0447</td><td>0.4583</td></tr>
<tr><td>385</td><td>WT002_B23_CLUSTER_7163</td><td>gi|15217558|ref|NP_174616.1| hypothetical protein [Arabidopsis thaliana] >gnl|BL_ORD_ID|804700 gi|25513752|pir||H86458 29.0K hypothetical protein F10C21.15 - Arabidopsis thaliana >gnl|BL_ORD_ID|804700 gi|12322391|gb|AAG51223.1| hypothetical protein;</td><td>11674.m02242|LOC_Os08g23140|protein hypothetical protein e_value:7.21887e-59</td><td>0.424</td><td>(0.2677 0.6320)</td><td>0.2677</td><td>0.632</td></tr>
<tr><td>386</td><td>WT010_B19_CLUSTER_21936</td><td>gi|34898580|ref|NP_910636.1| proteasome inhibitor-like protein [Oryza sativa (japonica cultivar-group)] >gnl|BL_ORD_ID|1528561 gi|28564795|dbj|BAC57725.1| proteasome inhibitor-like protein [Oryza sativa (japonica cultivar-group)] e_value:2.12407e-131</td><td>11673.m03522|LOC_Os07g36420|protein expressed protein e_value:6.05409e-130</td><td>0.149</td><td>(0.0979 0.6554)</td><td>0.0979</td><td>0.6554</td></tr>
<tr><td>387</td><td>WT009_N07_CLUSTER_31725</td><td>gi|34909474|ref|NP_916084.1| putative protein kinase [Oryza sativa (japonica cultivar-group)] >gnl|BL_ORD_ID|1549392 gi|14209526|dbj|BAB56022.1| putative protein kinase [Oryza sativa (japonica cultivar-group)] e_value:2.51571e-174</td><td>11682.m04224|LOC_Os05g44290|protein Protein kinase domain, putative e_value:0</td><td>0.215</td><td>(0.1724 0.8010)</td><td>0.1724</td><td>0.801</td></tr>
<tr><td>388</td><td>SET4_E12_CLUSTER_2982</td><td>gi|50942155|ref|XP_480605.1| putative copper chaperone [Oryza sativa (japonica cultivar-group)] >gi|42761303|dbj|BAD11546.1| putative copper chaperone [Oryza sativa (japonica cultivar-group)] >gi|40253404|dbj|BAD05334.1| putative copper chaperone [O</td><td>11674.m00998|LOC_Os08g10480|protein heavy metal-associated domain, putative e_value:2.16589e-34</td><td>0.065</td><td>(0.0528 0.8114)</td><td>0.0528</td><td>0.8114</td></tr>
<tr><td>389</td><td>WT009_G02_CLUSTER_3457</td><td>gi|50904629|ref|XP_463803.1| putative hepatocellular carcinoma-associated antigen 66 [Oryza sativa (japonica cultivar-group)] >gnl|BL_ORD_ID|1566046 gi|41052537|dbj|BAD08058.1| putative hepatocellular carcinoma-associated antigen 66 [Oryza sativa (japo</td><td>11668.m00037|LOC_Os02g01350|protein hypothetical protein e_value:0</td><td>0.208</td><td>(0.1001 0.4810)</td><td>0.1001</td><td>0.481</td></tr>
<tr><td>390</td><td>WT003_L09_CLUSTER_27411</td><td>gi|50944457|ref|XP_481756.1| putative GEKO1 [Oryza sativa (japonica cultivar-group)] >gnl|BL_ORD_ID|1605447 gi|38637389|dbj|BAD03648.1| putative GEKO1 [Oryza sativa (japonica cultivar-group)] >gnl|BL_ORD_ID|1605447 gi|38636945|dbj|BAD03207.1| putati</td><td>11674.m02447|LOC_Os08g25140|protein expressed protein e_value:1.51004e-151</td><td>0.082</td><td>(0.1292 1.5799)</td><td>0.1292</td><td>1.5799</td></tr>
<tr><td>391</td><td>WT002_K04_CLUSTER_9511</td><td>gi|55769721|ref|XP_549850.1| PEX14 -like [Oryza sativa (japonica cultivar-group)] >gnl|BL_ORD_ID|1753759 gi|52076231|dbj|BAD44885.1| PEX14 -like [Oryza sativa (japonica cultivar-group)] >gnl|BL_ORD_ID|1753759 gi|52076192|dbj|BAD44846.1| PEX14 -like</td><td>11667.m00075|LOC_Os01g01720|protein hypothetical protein e_value:1.58581e-156</td><td>0.326</td><td>(0.1983 0.6083)</td><td>0.1983</td><td>0.6083</td></tr>
<tr><td>392</td><td>WT012_O08_CLUSTER_10956</td><td>gi|34902102|ref|NP_912397.1| chloroplast import-associated channel protein homolog [Oryza sativa (japonica cultivar-group)] >gnl|BL_ORD_ID|1588835 gi|29893615|gb|AAP06869.1| chloroplast import-associated channel protein homolog [Oryza sativa (japonica</td><td>11669.m01648|LOC_Os03g16440|protein outer membrane protein, OMP85 family, putative e_value:0</td><td>0.083</td><td>(0.0418 0.5032)</td><td>0.0418</td><td>0.5032</td></tr>
<tr><td>393</td><td>WT010_C10_CLUSTER_10956</td><td>gi|34902102|ref|NP_912397.1| chloroplast import-associated channel protein homolog [Oryza sativa (japonica cultivar-group)] >gnl|BL_ORD_ID|1588835 gi|29893615|gb|AAP06869.1| chloroplast import-associated channel protein homolog [Oryza sativa (japonica</td><td>11669.m01648|LOC_Os03g16440|protein outer membrane protein, OMP85 family, putative e_value:0</td><td>0.081</td><td>(0.0397 0.4904)</td><td>0.0397</td><td>0.4904</td></tr>
<tr><td>394</td><td>SET5_K23_CLUSTER_33744</td><td>gi|50938129|ref|XP_478592.1| putative serine/threonine kinase protein [Oryza sativa (japonica cultivar-group)] >gi|50508317|dbj|BAD30125.1| putative serine/threonine kinase protein [Oryza sativa (japonica cultivar-group)] >gi|28812101|dbj|BAC65053.1</td><td>11673.m03441|LOC_Os07g35690|protein Protein kinase domain, putative e_value:0</td><td>0.266</td><td>(0.1502 0.5654)</td><td>0.1502</td><td>0.5654</td></tr>
<tr><td>395</td><td>SET5_C24_CLUSTER_32341</td><td>gi|24414272|gb|AAN59775.1| Unknown protein [Oryza sativa (japonica cultivar-group)] >gi|50920435|ref|XP_470578.1| Unknown protein [Oryza sativa (japonica cultivar-group)] e_value:9.57823e-75</td><td>11669.m00928|LOC_Os03g09870|protein Mitochondrial import inner membrane translocase subunit Tim17, putative</td><td>0.176</td><td>(0.1144 0.6516)</td><td>0.1144</td><td>0.6516</td></tr>
<tr><td>396</td><td>WT002_C07_CLUSTER_32927</td><td>gi|50934347|ref|XP_476701.1| unknown protein [Oryza sativa (japonica cultivar-group)] >gnl|BL_ORD_ID|1521661 gi|33146742|dbj|BAC79645.1| unknown protein [Oryza sativa (japonica cultivar-group)] e_value:0</td><td>11673.m00592|LOC_Os07g06710|protein TPR Domain, putative e_value:0</td><td>0.278</td><td>(0.1546 0.5563)</td><td>0.1546</td><td>0.5563</td></tr>
<tr><td>397</td><td>SET4_D08_CLUSTER_18783</td><td>gi|34908592|ref|NP_915643.1| P0505D12.24 [Oryza sativa (japonica cultivar-group)] e_value:9.24763e-35</td><td>11667.m06485|LOC_Os01g64680|protein Similar to At5g09830 e_value:3.77042e-36</td><td>0.267</td><td>(0.0842 0.3149)</td><td>0.0842</td><td>0.3149</td></tr>
<tr><td>398</td><td>SET3_C14_CLUSTER_33468</td><td>gi|23297471|gb|AAN12977.1| unknown protein [Arabidopsis thaliana] >gi|30692108|ref|NP_849520.1| bZIP protein [Arabidopsis thaliana] e_value:4.10397e-92</td><td>11674.m04349|LOC_Os08g43090|protein bZIP transcription factor, putative e_value:0</td><td>0.132</td><td>(0.0754 0.5725)</td><td>0.0754</td><td>0.5725</td></tr>
<tr><td>399</td><td>WT010_E19_CLUSTER_15769</td><td>gi|51535187|dbj|BAD38160.1| putative dehydroascorbate reductase [Oryza sativa (japonica cultivar-group)] e_value:1.37311e-120</td><td>11680.m01237|LOC_Os06g12630|protein glutathione dependent dehydroascorbate reductase precursor e_value:2.47483e-122</td><td>0.171</td><td>(0.0728 0.4265)</td><td>0.0728</td><td>0.4265</td></tr>
<tr><td>400</td><td>WT006_O15_CLUSTER_23286</td><td>gi|50919891|ref|XP_470306.1| putative protein tyrosine-serine-threonine kinase [Oryza sativa (japonica cultivar-group)] >gnl|BL_ORD_ID|1539441 gi|19071648|gb|AAL84315.1| putative protein tyrosine-serine-threonine kinase [Oryza sativa (japonica cultivar</td><td>11669.m00706|LOC_Os03g07430|protein Protein kinase domain, putative e_value:0</td><td>0.139</td><td>(0.0979 0.7057)</td><td>0.0979</td><td>0.7057</td></tr>
<tr><td>401</td><td>WT012_H19_CLUSTER_32662</td><td>gi|50937195|ref|XP_478125.1| auxin-regulated protein-like [Oryza sativa (japonica cultivar-group)] >gnl|BL_ORD_ID|1526880 gi|51963414|ref|XP_506344.1| PREDICTED P0683C09.107 gene product [Oryza sativa (japonica cultivar-group)] >gnl|BL_ORD_ID|152688</td><td>11673.m02881|LOC_Os07g30300|protein RhoGAP domain, putative e_value:0</td><td>0.091</td><td>(0.0493 0.5439)</td><td>0.0493</td><td>0.5439</td></tr>
<tr><td>402</td><td>SET2_P11_CLUSTER_33279</td><td>gi|33115140|gb|AAP95024.1| iron/ascorbate-dependent oxidoreductase [Hordeum vulgare] e_value:1.77444e-178</td><td>11668.m01703|LOC_Os02g17940|protein oxidoreductase, 2OG-Fe(II) oxygenase family, putative e_value:2.65501e-131</td><td>0.319</td><td>(0.2533 0.7934)</td><td>0.2533</td><td>0.7934</td></tr>
<tr><td>403</td><td>WT010_O19_CLUSTER_17770</td><td>gi|60677685|dbj|BAD90974.1| cytochrome P450 [Oryza sativa (japonica cultivar-group)] e_value:0</td><td>11686.m00374|LOC_Os12g04480|protein cytochrome p450 90a1 (ec 1.14.-.-). [mouse-ear cress] e_value:0</td><td>0.201</td><td>(0.0878 0.4365)</td><td>0.0878</td><td>0.4365</td></tr>
<tr><td>404</td><td>WT002_B02_CLUSTER_15014</td><td>gi|6522552|emb|CAB61996.1| putative protein [Arabidopsis thaliana] >gnl|BL_ORD_ID|1451073 gi|15229097|ref|NP_190483.1| pentatricopeptide (PPR) repeat-containing protein [Arabidopsis thaliana] >gnl|BL_ORD_ID|1451073 gi|11358281|pir||T46116 hypothetic</td><td>11687.m03108|LOC_Os11g34130|protein hypothetical protein e_value:3.85174e-176</td><td>0.162</td><td>(0.0936 0.5772)</td><td>0.0936</td><td>0.5772</td></tr>
<tr><td>405</td><td>WT009_N24_CLUSTER_28170</td><td>gi|12240033|gb|AAG49550.1| putative thiamine biosythesis protein ThiC [Poa secunda] e_value:0</td><td>11669.m04768|LOC_Os03g47610|protein putative thiamin biosynthesis protein e_value:0</td><td>0.072</td><td>(0.0395 0.5468)</td><td>0.0395</td><td>0.5468</td></tr>
<tr><td>406</td><td>WT009_B20_CLUSTER_14970</td><td>gi|34912136|ref|NP_917415.1| OSJNBb0024F06.20 [Oryza sativa (japonica cultivar-group)] e_value:3.12501e-70</td><td>11667.m03873|LOC_Os01g40310|protein hypothetical protein e_value:6.599e-72</td><td>0.325</td><td>(0.1469 0.4519)</td><td>0.1469</td><td>0.4519</td></tr>
<tr><td>407</td><td>WT004_G14_CLUSTER_17530</td><td>gi|56785263|dbj|BAD82172.1| unknown protein [Oryza sativa (japonica cultivar-group)] e_value:0</td><td>11667.m06756|LOC_Os01g67120|protein expressed protein</td><td>0.265</td><td>(0.1163 0.4392)</td><td>0.1163</td><td>0.4392</td></tr>
<tr><td>408</td><td>SET3_N07_CLUSTER_8367</td><td>gi|51854423|gb|AAU10802.1| 'putative endo-1,3;1,4-beta-D-glucanase' [Oryza sativa (japonica cultivar-group)] e_value:1.16359e-115</td><td>11682.m03098|LOC_Os05g33100|protein AT3g23600/MDB19_9 e_value:2.72488e-117</td><td>0.22</td><td>(0.0908 0.4123)</td><td>0.0908</td><td>0.4123</td></tr>
<tr><td>409</td><td>WT005_F15_CLUSTER_32135</td><td>gi|51963390|ref|XP_506295.1| PREDICTED P0443H10.4 gene product [Oryza sativa (japonica cultivar-group)] >gnl|BL_ORD_ID|1526005 gi|50936599|ref|XP_477827.1| putative CDPK substrate protein 1 [Oryza sativa (japonica cultivar-group)] >gnl|BL_ORD_ID|152</td><td>11669.m02176|LOC_Os03g21240|protein myb-like DNA-binding domain, SHAQKYF class, putative e_value:1.79114e-146</td><td>0.361</td><td>(0.2065 0.5713)</td><td>0.2065</td><td>0.5713</td></tr>
<tr><td>410</td><td>WT002_H13_CLUSTER_33900</td><td>gi|50901080|ref|XP_462973.1| putative MATE efflux family protein [Oryza sativa (japonica cultivar-group)] >gnl|BL_ORD_ID|1564608 gi|41393239|gb|AAS01962.1| putative MATE efflux family protein [Oryza sativa (japonica cultivar-group)] e_value:0</td><td>11669.m03698|LOC_Os03g37490|protein putative MATE efflux family protein e_value:0</td><td>0.286</td><td>(0.1098 0.3846)</td><td>0.1098</td><td>0.3846</td></tr>
<tr><td>411</td><td>WT005_I02_CLUSTER_14691</td><td>gi|47834387|gb|AAT38891.1| cycloartenol synthase [Avena strigosa] e_value:0</td><td>11668.m00409|LOC_Os02g04710|protein squalene/oxidosqualene cyclases e_value:0</td><td>0.132</td><td>(0.0696 0.5259)</td><td>0.0696</td><td>0.5259</td></tr>
<tr><td>412</td><td>WT011_D14_CLUSTER_4013</td><td>gi|34912214|ref|NP_917454.1| putative telomere repeat binding factor [Oryza sativa (japonica cultivar-group)] >gnl|BL_ORD_ID|1542314 gi|20146249|dbj|BAB89031.1| putative single myb histone 6 [Oryza sativa (japonica cultivar-group)] e_value:5.7696e-118</td><td>11667.m03915|LOC_Os01g40670|protein Myb-like DNA-binding domain, putative e_value:5.08312e-122</td><td>0.242</td><td>(0.1274 0.5275)</td><td>0.1274</td><td>0.5275</td></tr>
<tr><td>413</td><td>SET5_H09_CLUSTER_4013</td><td>gi|34912214|ref|NP_917454.1| putative telomere repeat binding factor [Oryza sativa (japonica cultivar-group)] >gi|20146249|dbj|BAB89031.1| putative single myb histone 6 [Oryza sativa (japonica cultivar-group)] e_value:6.35892e-118</td><td>11667.m03915|LOC_Os01g40670|protein Myb-like DNA-binding domain, putative e_value:5.08312e-122</td><td>0.237</td><td>(0.1274 0.5371)</td><td>0.1274</td><td>0.5371</td></tr>
<tr><td>414</td><td>WT005_E18_CLUSTER_11727</td><td>gi|56784260|dbj|BAD81942.1| unknown protein [Oryza sativa (japonica cultivar-group)] e_value:4.43474e-102</td><td>11667.m06349|LOC_Os01g63410|protein expressed protein e_value:1.13637e-103</td><td>0.231</td><td>(0.1277 0.5532)</td><td>0.1277</td><td>0.5532</td></tr>
<tr><td>415</td><td>WT003_M07_CLUSTER_22583</td><td>gi|34910924|ref|NP_916809.1| putative vacuolar protein sorting protein [Oryza sativa (japonica cultivar-group)] >gnl|BL_ORD_ID|1546975 gi|20161577|dbj|BAB90498.1| vacuolar protein sorting-associated protein VPS16-like [Oryza sativa (japonica cultivar-g</td><td>11667.m04611|LOC_Os01g47650|protein Vps16, C-terminal region, putative e_value:0</td><td>0.11</td><td>(0.0580 0.5284)</td><td>0.058</td><td>0.5284</td></tr>
<tr><td>416</td><td>WT003_E07_CLUSTER_12150</td><td>gi|50948459|ref|XP_483757.1| putative auxin-regulated protein [Oryza sativa (japonica cultivar-group)] >gnl|BL_ORD_ID|1612256 gi|42407953|dbj|BAD09092.1| putative auxin-regulated protein [Oryza sativa (japonica cultivar-group)] >gnl|BL_ORD_ID|161225</td><td>11674.m04486|LOC_Os08g44400|protein Glutathione S-transferase, N-terminal domain, putative e_value:4.53653e-146</td><td>0.289</td><td>(0.1547 0.5358)</td><td>0.1547</td><td>0.5358</td></tr>
<tr><td>417</td><td>SET5_B06_CLUSTER_859</td><td>gi|6179602|emb|CAB59894.1| glutathione peroxidase-like protein GPX15Hv [Hordeum vulgare subsp. vulgare] e_value:4.77793e-94</td><td>11670.m04581|LOC_Os04g46960|protein glutathione peroxidase 1 e_value:1.13493e-69</td><td>0.106</td><td>(0.1469 1.3823)</td><td>0.1469</td><td>1.3823</td></tr>
<tr><td>418</td><td>SET4_L22_CLUSTER_22457</td><td>gi|57899200|dbj|BAD87310.1| putative photosystem II protein reaction center W [Oryza sativa (japonica cultivar-group)] e_value:3.99292e-72</td><td>11667.m07138|LOC_Os01g71190|protein Photosystem II reaction centre W protein, PsbW, putative e_value:8.61143e-74</td><td>0.315</td><td>(0.1194 0.3795)</td><td>0.1194</td><td>0.3795</td></tr>
<tr><td>419</td><td>SET4_G11_CLUSTER_1032</td><td>gi|26450368|dbj|BAC42300.1| unknown protein [Arabidopsis thaliana] >gi|18398872|ref|NP_564425.1| expressed protein [Arabidopsis thaliana] >gi|12322387|gb|AAG51219.1| unknown protein; 76034-74699 [Arabidopsis thaliana] >gi|25403195|pir||G86458 unk</td><td>11682.m00061|LOC_Os05g01510|protein hypothetical protein e_value:1.09159e-61</td><td>0.326</td><td>(0.3217 0.9872)</td><td>0.3217</td><td>0.9872</td></tr>
</table>

PDF Complete
Your complimentary use period has ended. Thank you for using PDF Complete.
Click Here to upgrade to Unlimited Pages and Expanded Features

<table>
<tr><th></th><th></th><th></th><th></th><th></th><th></th><th></th><th></th></tr>
<tr><td>420</td><td>SET3_I24_CLUSTER_16296</td><td>gi|50918179|ref|XP_469486.1| unknown protein [Oryza sativa] >gi|13937298|gb|AAK50129.1| unknown protein [Oryza sativa] e_value:2.08154e-151</td><td>11669.m05042|LOC_Os03g50070|protein expressed protein e_value:4.13015e-153</td><td>0.108</td><td>(0.0778 0.7215)</td><td>0.0778</td><td>0.7215</td></tr>
<tr><td>421</td><td>WT005_L19_CLUSTER_34261</td><td>gi|53982664|gb|AAV25643.1| unknown protein [Oryza sativa (japonica cultivar-group)] e_value:1.34065e-158</td><td>11682.m03263|LOC_Os05g34630|protein expressed protein e_value:1.8353e-159</td><td>0.213</td><td>(0.1434 0.6731)</td><td>0.1434</td><td>0.6731</td></tr>
<tr><td>422</td><td>WT003_I07_CLUSTER_32728</td><td>gi|50943021|ref|XP_481038.1| putative Preprotein translocase secY subunit, chloroplast precursor [Oryza sativa (japonica cultivar-group)] >gnl|BL_ORD_ID|1605880 gi|37572923|dbj|BAC98517.1| putative Preprotein translocase secY subunit, chloroplast precu</td><td>11674.m01509|LOC_Os08g15460|protein preprotein translocase secy subunit, chloroplast precursor (cpsecy). [maize e_value:0</td><td>0.224</td><td>(0.0855 0.3812)</td><td>0.0855</td><td>0.3812</td></tr>
<tr><td>423</td><td>WT007_N21_CLUSTER_32589</td><td>gi|50949063|ref|XP_493889.1| putative protein kinase [Oryza sativa] >gnl|BL_ORD_ID|1742106 gi|52353638|gb|AAU44204.1| unknown protein [Oryza sativa (japonica cultivar-group)] >gnl|BL_ORD_ID|1742106 gi|14719339|gb|AAK73157.1| putative protein kinase</td><td>11669.m01683|LOC_Os03g16740|protein protein kinase, putative e_value:0</td><td>0.109</td><td>(0.0679 0.6242)</td><td>0.0679</td><td>0.6242</td></tr>
<tr><td>424</td><td>WT013_I24_CLUSTER_22216</td><td>gi|23308347|gb|AAN18143.1| At5g52030/MSG15_11 [Arabidopsis thaliana] >gnl|BL_ORD_ID|1237132 gi|19699285|gb|AAL91254.1| AT5g52030/MSG15_11 [Arabidopsis thaliana] e_value:4.05728e-108</td><td>11674.m04363|LOC_Os08g43230|protein TraB family, putative e_value:4.95868e-117</td><td>0.084</td><td>(0.0845 1.0067)</td><td>0.0845</td><td>1.0067</td></tr>
<tr><td>425</td><td>WT011_J20_CLUSTER_28619</td><td>gi|58531976|emb|CAE03618.3| OSJNBb0003B01.9 [Oryza sativa (japonica cultivar-group)] e_value:5.03729e-151</td><td>11670.m05372|LOC_Os04g54640|protein expressed protein e_value:2.92854e-134</td><td>0.051</td><td>(0.0521 1.0177)</td><td>0.0521</td><td>1.0177</td></tr>
<tr><td>426</td><td>SET1_M24_CLUSTER_4155</td><td>gi|34910520|ref|NP_916607.1| B1142C05.17 [Oryza sativa (japonica cultivar-group)] >gi|20146336|dbj|BAB89117.1| methyltransferase-like [Oryza sativa (japonica cultivar-group)] e_value:1.81464e-151</td><td>11667.m05035|LOC_Os01g51530|protein expressed protein e_value:5.85816e-153</td><td>0.147</td><td>(0.0660 0.4486)</td><td>0.066</td><td>0.4486</td></tr>
<tr><td>427</td><td>WT013_E17_CLUSTER_6340</td><td>gi|54290283|dbj|BAD61228.1| unknown protein [Oryza sativa (japonica cultivar-group)] e_value:0</td><td>11667.m04134|LOC_Os01g42800|protein expressed protein</td><td>0.185</td><td>(0.1014 0.5482)</td><td>0.1014</td><td>0.5482</td></tr>
<tr><td>428</td><td>WT012_F12_CLUSTER_28026</td><td>gi|10177307|dbj|BAB10568.1| unnamed protein product [Arabidopsis thaliana] >gnl|BL_ORD_ID|1093878 gi|15242694|ref|NP_201131.1| zinc finger (CCCH-type) family protein [Arabidopsis thaliana] >gnl|BL_ORD_ID|1093878 gi|62901484|sp|Q5RJC5|ZFNL5_ARATH Zin</td><td>11686.m01794|LOC_Os12g18120|protein Similar to contains similarity to zinc finger protein gene_id:MDC12.23 e_value:0</td><td>0.282</td><td>(0.2035 0.7204)</td><td>0.2035</td><td>0.7204</td></tr>
<tr><td>429</td><td>WT011_J17_CLUSTER_31289</td><td>gi|50919989|ref|XP_470355.1| unknown protein [Oryza sativa (japonica cultivar-group)] >gnl|BL_ORD_ID|1535399 gi|28376712|gb|AAO41142.1| unknown protein [Oryza sativa (japonica cultivar-group)] e_value:0</td><td>11669.m06347|LOC_Os03g62170|protein expressed protein e_value:0</td><td>0.199</td><td>(0.1118 0.5616)</td><td>0.1118</td><td>0.5616</td></tr>
<tr><td>430</td><td>WT010_K08_CLUSTER_16006</td><td>gi|51091709|dbj|BAD36510.1| putative VEF family protein [Oryza sativa (japonica cultivar-group)] e_value:0</td><td>11681.m01201|LOC_Os09g13630|protein VEF family protein</td><td>0.173</td><td>(0.0989 0.5707)</td><td>0.0989</td><td>0.5707</td></tr>
<tr><td>431</td><td>WT010_E08_CLUSTER_8489</td><td>gi|34911616|ref|NP_917155.1| P0039G05.10 [Oryza sativa (japonica cultivar-group)] >gnl|BL_ORD_ID|1553098 gi|20805120|dbj|BAB92791.1| integral membrane protein-like [Oryza sativa (japonica cultivar-group)] e_value:2.0261e-108</td><td>11667.m04362|LOC_Os01g45360|protein expressed protein e_value:1.69171e-123</td><td>0.371</td><td>(0.2150 0.5786)</td><td>0.215</td><td>0.5786</td></tr>
<tr><td>432</td><td>WT009_N13_CLUSTER_18015</td><td>gi|50932113|ref|XP_475584.1| unknown protein [Oryza sativa (japonica cultivar-group)] >gnl|BL_ORD_ID|1597079 gi|46391116|gb|AAS90643.1| unknown protein [Oryza sativa (japonica cultivar-group)] e_value:0</td><td>11682.m00437|LOC_Os05g04950|protein expressed protein e_value:0</td><td>0.198</td><td>(0.1229 0.6223)</td><td>0.1229</td><td>0.6223</td></tr>
<tr><td>433</td><td>WT008_E17_CLUSTER_9782</td><td>gi|50937183|ref|XP_478119.1| putative asparaginyl-tRNA synthetase, chloroplast/mitochondrial precursor [Oryza sativa (japonica cultivar-group)] >gnl|BL_ORD_ID|1526872 gi|50509311|dbj|BAD30675.1| putative asparaginyl-tRNA synthetase, chloroplast/mitocho</td><td>11673.m02870|LOC_Os07g30200|protein probable asparagine-tRNA ligase e_value:0</td><td>0.114</td><td>(0.0586 0.5144)</td><td>0.0586</td><td>0.5144</td></tr>
<tr><td>434</td><td>WT008_E16_CLUSTER_9782</td><td>gi|50937183|ref|XP_478119.1| putative asparaginyl-tRNA synthetase, chloroplast/mitochondrial precursor [Oryza sativa (japonica cultivar-group)] >gnl|BL_ORD_ID|1526872 gi|50509311|dbj|BAD30675.1| putative asparaginyl-tRNA synthetase, chloroplast/mitocho</td><td>11673.m02870|LOC_Os07g30200|protein probable asparagine-tRNA ligase e_value:0</td><td>0.114</td><td>(0.0586 0.5144)</td><td>0.0586</td><td>0.5144</td></tr>
<tr><td>435</td><td>WT008_A09_CLUSTER_6092</td><td>gi|4894963|gb|AAD32676.1| DNA-binding protein WRKY3 [Avena sativa] e_value:9.37931e-147</td><td>11670.m02026|LOC_Os04g21950|protein DNA-binding protein WRKY3 e_value:1.87951e-146</td><td>0.184</td><td>(0.0940 0.5121)</td><td>0.094</td><td>0.5121</td></tr>
<tr><td>436</td><td>WT007_J02_CLUSTER_26273</td><td>gi|34911448|ref|NP_917071.1| P0431H09.24 [Oryza sativa (japonica cultivar-group)] e_value:0</td><td>11667.m04878|LOC_Os01g50060|protein expressed protein e_value:4.77207e-180</td><td>0.195</td><td>(0.1218 0.6232)</td><td>0.1218</td><td>0.6232</td></tr>
<tr><td>437</td><td>WT007_H15_CLUSTER_14849</td><td>gi|25809276|emb|CAD45186.1| lipoxygenase 2 [Hordeum vulgare subsp. vulgare] >gnl|BL_ORD_ID|1403061 gi|34922539|sp|Q8GSM3|LOX22_HORVU Lipoxygenase 2.2, chloroplast precursor (LOX2:Hv:2) e_value:0</td><td>11686.m03661|LOC_Os12g37260|protein Lipoxygenase e_value:0</td><td>0.451</td><td>(0.3329 0.7378)</td><td>0.3329</td><td>0.7378</td></tr>
<tr><td>438</td><td>WT006_O18_CLUSTER_20200</td><td>gi|21536786|gb|AAM61118.1| unknown [Arabidopsis thaliana] >gnl|BL_ORD_ID|1093556 gi|21386971|gb|AAM47889.1| unknown protein [Arabidopsis thaliana] >gnl|BL_ORD_ID|1093556 gi|18379115|ref|NP_563687.1| photosystem II family protein [Arabidopsis thalian</td><td>11669.m02211|LOC_Os03g21560|protein expressed protein e_value:4.6926e-55</td><td>0.473</td><td>(0.1854 0.3923)</td><td>0.1854</td><td>0.3923</td></tr>
<tr><td>439</td><td>WT004_N01_CLUSTER_20564</td><td>gi|21655287|gb|AAM65355.1| At1g75100/F9E10_5 [Arabidopsis thaliana] >gnl|BL_ORD_ID|2371635 gi|18410769|ref|NP_565101.1| expressed protein [Arabidopsis thaliana] >gnl|BL_ORD_ID|2371635 gi|15982795|gb|AAL09745.1| At1g75100/F9E10_5 [Arabidopsis thalian</td><td>11669.m00961|LOC_Os03g10180|protein hypothetical protein e_value:0</td><td>0.306</td><td>(0.1770 0.5785)</td><td>0.177</td><td>0.5785</td></tr>
<tr><td>440</td><td>WT004_C17_CLUSTER_1706</td><td>gi|2105137|gb|AAC49729.1| ADP-glucose pyrophosphorylase large subunit [Hordeum vulgare] >gnl|BL_ORD_ID|405675 gi|7434885|pir||T06194 glucose-1-phosphate adenylyltransferase (EC 2.7.7.27) large chain - barley e_value:0</td><td>11669.m05300|LOC_Os03g52460|protein putative ADP-glucose pyrophosphorylase e_value:0</td><td>0.155</td><td>(0.0778 0.5022)</td><td>0.0778</td><td>0.5022</td></tr>
<tr><td>441</td><td>WT003_I03_CLUSTER_17761</td><td>gi|50912383|ref|XP_467599.1| extracellular calcium sensing receptor [Oryza sativa (japonica cultivar-group)] >gnl|BL_ORD_ID|1580204 gi|46390846|dbj|BAD16350.1| extracellular calcium sensing receptor [Oryza sativa (japonica cultivar-group)] e_value:7.02</td><td>11668.m04836|LOC_Os02g49680|protein extracellular calcium sensing receptor e_value:2.12133e-161</td><td>0.304</td><td>(0.1248 0.4111)</td><td>0.1248</td><td>0.4111</td></tr>
<tr><td>442</td><td>WT002_E21_CLUSTER_6776</td><td>gi|56202163|dbj|BAD73641.1| hypothetical protein [Oryza sativa (japonica cultivar-group)] e_value:9.78404e-67</td><td>11667.m04609|LOC_Os01g47630|protein expressed protein e_value:2.51453e-68</td><td>0.217</td><td>(0.1488 0.6865)</td><td>0.1488</td><td>0.6865</td></tr>
<tr><td>443</td><td>WT002_C16_CLUSTER_23772</td><td>gi|51964688|ref|XP_507128.1| PREDICTED OJ1368_G08.30 gene product [Oryza sativa (japonica cultivar-group)] >gnl|BL_ORD_ID|1601815 gi|50941005|ref|XP_480030.1| 27k vesicle-associated membrane protein-associated protein-like [Oryza sativa</td><td>11674.m00381|LOC_Os08g04620|protein MSP (Major sperm protein) domain, putative e_value:1.02335e-110</td><td>0.102</td><td>(0.0737 0.7191)</td><td>0.0737</td><td>0.7191</td></tr>
<tr><td>444</td><td>SET6_K02_CLUSTER_1183</td><td>gi|50926702|ref|XP_473298.1| OSJNBb0039L24.15 [Oryza sativa (japonica cultivar-group)] >gi|38346996|emb|CAE04576.2| OSJNBb0039L24.15 [Oryza sativa (japonica cultivar-group)] e_value:7.16417e-115</td><td>11670.m04377|LOC_Os04g45090|protein Cytochrome b561 e_value:1.33651e-116</td><td>0.175</td><td>(0.0614 0.3500)</td><td>0.0614</td><td>0.35</td></tr>
<tr><td>445</td><td>SET3_E11_CLUSTER_17883</td><td>gi|50926670|ref|XP_473282.1| OSJNBa0081C01.18 [Oryza sativa (japonica cultivar-group)] >gi|38346767|emb|CAE03868.2| OSJNBa0081C01.18 [Oryza sativa (japonica cultivar-group)] e_value:1.65074e-94</td><td>11668.m04099|LOC_Os02g42810|protein oxidoreductase, short chain dehydrogenase/reductase family e_value:1.47379e-95</td><td>0.319</td><td>(0.2940 0.9216)</td><td>0.294</td><td>0.9216</td></tr>
<tr><td>446</td><td>SET2_D14_CLUSTER_28081</td><td>gi|26006485|gb|AAN77294.1| Unknown protein [Oryza sativa (japonica cultivar-group)] e_value:7.10352e-34</td><td>11669.m01034|LOC_Os03g10860|protein hypothetical protein e_value:6.61562e-36</td><td>0.482</td><td>(0.1312 0.2725)</td><td>0.1312</td><td>0.2725</td></tr>
<tr><td>447</td><td>SET2_A23_CLUSTER_27544</td><td>gi|57899718|dbj|BAD87438.1| putative NADPH-dependent FMN and FAD containing oxidoreductase-like protein [Oryza sativa (japonica cultivar-group)] >gi|57899309|dbj|BAD87796.1| putative NADPH-dependent FMN and FAD containing oxidoreductase-like protein [O</td><td>11667.m05226|LOC_Os01g53250|protein flavodoxin, putative e_value:0</td><td>0.268</td><td>(0.1120 0.4173)</td><td>0.112</td><td>0.4173</td></tr>
<tr><td>448</td><td>SET1_C07_CLUSTER_20157</td><td>gi|52353526|gb|AAU44092.1| unknown protein [Oryza sativa (japonica cultivar-group)] e_value:0</td><td>11682.m04767|LOC_Os05g49610|protein expressed protein</td><td>0.166</td><td>(0.1014 0.6122)</td><td>0.1014</td><td>0.6122</td></tr>
<tr><td>449</td><td>WT013_M24_CLUSTER_27989</td><td>gi|34908940|ref|NP_915817.1| diphosphonucleotide phosphatase -like protein [Oryza sativa (japonica cultivar-group)] >gnl|BL_ORD_ID|1546602 gi|20804738|dbj|BAB92424.1| putative diphosphonucleotide phosphatase 1 [Oryza sativa (japonica cultivar-group)] &</td><td>11667.m05819|LOC_Os01g58640|protein Ser/Thr protein phosphatase family protein, putative e_value:0</td><td>0.259</td><td>(0.1525 0.5881)</td><td>0.1525</td><td>0.5881</td></tr>
<tr><td>450</td><td>SET1_D05_CLUSTER_32713</td><td>gi|50916931|ref|XP_468862.1| expressed protein [Oryza sativa (japonica cultivar-group)] >gi|40736997|gb|AAR89010.1| expressed protein [Oryza sativa (japonica cultivar-group)] e_value:2.12305e-127</td><td>11669.m03292|LOC_Os03g32490|protein expressed protein e_value:3.70141e-134</td><td>0.207</td><td>(0.1373 0.6642)</td><td>0.1373</td><td>0.6642</td></tr>
<tr><td>451</td><td>SET4_I17_CLUSTER_30910</td><td>gi|19025|emb|CAA33728.1| unnamed protein product [Hordeum vulgare subsp. vulgare] >gi|100628|pir||S07475 early light-induced protein, high molecular weight, precursor (clone HV58) - barley chloroplast >gi|119284|sp|P14895|ELI5_HORVU High molecular m</td><td>11667.m01427|LOC_Os01g14410|protein Chlorophyll A-B binding protein, putative e_value:7.55949e-63</td><td>0.396</td><td>(0.2414 0.6103)</td><td>0.2414</td><td>0.6103</td></tr>
<tr><td>452</td><td>SET1_D19_CLUSTER_30926</td><td>gi|55770541|ref|XP_550261.1| putative axi 1 [Oryza sativa (japonica cultivar-group)] >gi|55297510|dbj|BAD68312.1| putative axi 1 [Oryza sativa (japonica cultivar-group)] e_value:0</td><td>11667.m00677|LOC_Os01g07410|protein Plant protein family, putative e_value:0</td><td>0.128</td><td>(0.0718 0.5630)</td><td>0.0718</td><td>0.563</td></tr>
<tr><td>453</td><td>WT004_N15_CLUSTER_33597</td><td>gi|12083290|gb|AAG48804.1| unknown protein [Arabidopsis thaliana] >gnl|BL_ORD_ID|1241253 gi|21555588|gb|AAM63892.1| unknown [Arabidopsis thaliana] >gnl|BL_ORD_ID|1241253 gi|20453367|gb|AAM19922.1| At1g01820/T1N6_18 [Arabidopsis thaliana] >gnl|BL_</td><td>11669.m00184|LOC_Os03g02590|protein T1N6.24 protein - Arabidopsis thaliana e_value:3.86006e-116</td><td>0.066</td><td>(0.0652 0.9868)</td><td>0.0652</td><td>0.9868</td></tr>
<tr><td>454</td><td>SET2_C23_CLUSTER_9233</td><td>gi|50945279|ref|XP_482167.1| unknown protein [Oryza sativa (japonica cultivar-group)] >gi|40253488|dbj|BAD05438.1| unknown protein [Oryza sativa (japonica cultivar-group)] e_value:0</td><td>11674.m02928|LOC_Os08g29780|protein expressed protein e_value:0</td><td>0.186</td><td>(0.1161 0.6241)</td><td>0.1161</td><td>0.6241</td></tr>
<tr><td>455</td><td>WT002_O16_CLUSTER_32452</td><td>gi|50904581|ref|XP_463779.1| putative hydroxypyruvate reductase [Oryza sativa (japonica cultivar-group)] >gnl|BL_ORD_ID|1566107 gi|41053227|dbj|BAD08188.1| putative hydroxypyruvate reductase [Oryza sativa (japonica cultivar-group)] >gnl|BL_ORD_ID|15</td><td>11668.m00017|LOC_Os02g01150|protein D-isomer specific 2-hydroxyacid dehydrogenase, NAD binding domain, putative</td><td>0.058</td><td>(0.0408 0.7033)</td><td>0.0408</td><td>0.7033</td></tr>
<tr><td>456</td><td>WT009_F16_CLUSTER_13196</td><td>gi|50905841|ref|XP_464409.1| putative lycopene beta-cyclase [Oryza sativa (japonica cultivar-group)] >gnl|BL_ORD_ID|1566627 gi|46390965|dbj|BAD16478.1| putative lycopene beta-cyclase [Oryza sativa (japonica cultivar-group)] e_value:0</td><td>11668.m00898|LOC_Os02g09750|protein lycopene beta-cyclase e_value:0</td><td>0.186</td><td>(0.0780 0.4204)</td><td>0.078</td><td>0.4204</td></tr>
<tr><td>457</td><td>WT008_A19_CLUSTER_18883</td><td>gi|31433632|gb|AAP55124.1| putative carnitine/acylcarnitine translocase [Oryza sativa (japonica cultivar-group)] >gnl|BL_ORD_ID|2013147 gi|37537070|ref|NP_922837.1| putative carnitine/acylcarnitine translocase [Oryza sativa (japonica cultivar-</td><td>11676.m03910|LOC_Os10g42300|protein putative carnitine/acylcarnitine translocase e_value:6.32958e-158</td><td>0.133</td><td>(0.0680 0.5095)</td><td>0.068</td><td>0.5095</td></tr>
<tr><td>458</td><td>SET5_E20_CLUSTER_30422</td><td>gi|31433512|gb|AAP55017.1| putative Myb-related protein [Oryza sativa (japonica cultivar-group)] >gi|37536856|ref|NP_922730.1| putative Myb-related protein [Oryza sativa (japonica cultivar-group)] >gi|13569996|gb|AAK31280.1| putative Myb-related pro</td><td>11676.m03787|LOC_Os10g41200|protein Transposable element protein, putative e_value:3.55339e-153</td><td>0.204</td><td>(0.0723 0.3554)</td><td>0.0723</td><td>0.3554</td></tr>
<tr><td>459</td><td>WT005_G02_CLUSTER_14773</td><td>gi|37651975|emb|CAE51321.1| chopper chaperone [Hordeum vulgare subsp. vulgare] e_value:1.63325e-46</td><td>11668.m03089|LOC_Os02g32800|protein copper chaperone homolog CCH [imported] - rice e_value:5.27432e-35</td><td>0.139</td><td>(0.1291 0.9317)</td><td>0.1291</td><td>0.9317</td></tr>
<tr><td>460</td><td>WT002_P06_CLUSTER_1156</td><td>gi|50399957|gb|AAT76345.1| hypothetical protein [Oryza sativa (japonica cultivar-group)] e_value:2.4367e-27</td><td>11669.m03762|LOC_Os03g38120|protein hypothetical protein e_value:2.71259e-29</td><td>0.673</td><td>(0.2215 0.3291)</td><td>0.2215</td><td>0.3291</td></tr>
<tr><td>461</td><td>SET6_E24_CLUSTER_1156</td><td>gi|50399957|gb|AAT76345.1| hypothetical protein [Oryza sativa (japonica cultivar-group)] e_value:2.60729e-27</td><td>11669.m03762|LOC_Os03g38120|protein hypothetical protein e_value:2.71259e-29</td><td>0.732</td><td>(0.2214 0.3026)</td><td>0.2214</td><td>0.3026</td></tr>
<tr><td>462</td><td>SET4_N12_CLUSTER_9056</td><td>gi|50904335|ref|XP_463656.1| putative 50S ribosomal protein [Oryza sativa (japonica cultivar-group)] >gi|20161923|dbj|BAB90834.1| ribosomal protein L27 precursor [Oryza sativa (japonica cultivar-group)] >gi|20161735|dbj|BAB90651.1| ribosomal protein</td><td>11667.m07005|LOC_Os01g69950|protein ribosomal protein L27, putative e_value:8.90201e-82</td><td>0.308</td><td>(0.0967 0.3144)</td><td>0.0967</td><td>0.3144</td></tr>
<tr><td>463</td><td>WT002_P12_CLUSTER_17193</td><td>gi|34909042|ref|NP_915868.1| P0034E02.34 [Oryza sativa (japonica cultivar-group)] >gnl|BL_ORD_ID|1554744 gi|20804576|dbj|BAB92268.1| unknown protein [Oryza sativa (japonica cultivar-group)] e_value:5.47329e-64</td><td>11667.m05863|LOC_Os01g59080|protein expressed protein e_value:1.10651e-65</td><td>0.307</td><td>(0.2517 0.8191)</td><td>0.2517</td><td>0.8191</td></tr>
<tr><td>464</td><td>WT007_H13_CLUSTER_24642</td><td>gi|50909617|ref|XP_466297.1| unknown protein [Oryza sativa (japonica cultivar-group)] >gnl|BL_ORD_ID|1576837 gi|51964102|ref|XP_506834.1| PREDICTED P0020C11.43 gene product [Oryza sativa (japonica cultivar-group)] >gnl|BL_ORD_ID|1576837 gi|46806678|</td><td>11668.m03406|LOC_Os02g35840|protein expressed protein e_value:2.58877e-158</td><td>0.259</td><td>(0.1421 0.5490)</td><td>0.1421</td><td>0.549</td></tr>
</table>

PDF Complete
Your complimentary use period has ended. Thank you for using PDF Complete.
Click Here to upgrade to Unlimited Pages and Expanded Features

<table>
<tr><td>465</td><td>WT005_F22_CLUSTER_28062</td><td>gi|50902014|ref|XP_463440.1| P0512C01.12 [Oryza sativa (japonica cultivar-group)] >gnl|BL_ORD_ID|2302327 gi|20804672|dbj|BAB92360.1| unknown protein [Oryza sativa (japonica cultivar-group)] >gnl|BL_ORD_ID|2302327</td><td>11667.m05484|LOC_Os01g55570|protein hypothetical protein e_value:5.00601e-41</td><td>0.394</td><td>(0.2693 0.6837)</td><td>0.2693</td><td>0.6837</td></tr>
<tr><td>466</td><td>SET1_J21_CLUSTER_32399</td><td>gi|53793363|dbj|BAD52944.1| putative photosystem II reaction center W protein [Oryza sativa (japonica cultivar-group)] e_value:2.03106e-47</td><td>11667.m05608|LOC_Os01g56680|protein photosystem ii reaction center w protein, chloroplast precursor (psii6.1 kda protein) e_value:3.41723e-49</td><td>0.259</td><td>(0.0793 0.3067)</td><td>0.0793</td><td>0.3067</td></tr>
<tr><td>467</td><td>WT010_C12_CLUSTER_12863</td><td>gi|51536072|dbj|BAD38198.1| mitochondrial transcription termination factor-like [Oryza sativa (japonica cultivar-group)] >gnl|BL_ORD_ID|243011 gi|51535002|dbj|BAD37286.1| mitochondrial transcription termination factor-like [Oryza sativa (japonica culti</td><td>11680.m01182|LOC_Os06g12100|protein mTERF, putative e_value:1.74364e-107</td><td>0.585</td><td>(0.3308 0.5651)</td><td>0.3308</td><td>0.5651</td></tr>
<tr><td>468</td><td>WT009_O24_CLUSTER_32482</td><td>gi|52077177|dbj|BAD46222.1| putative beta-amylase [Oryza sativa (japonica cultivar-group)] e_value:0</td><td>11681.m03562|LOC_Os09g39570|protein beta-amylase (EC 3.2.1.2) - Arabidopsis thaliana e_value:0</td><td>0.14</td><td>(0.1158 0.8263)</td><td>0.1158</td><td>0.8263</td></tr>
<tr><td>469</td><td>SET4_A06_CLUSTER_9180</td><td>gi|50929309|ref|XP_474182.1| OSJNBa0071I13.18 [Oryza sativa (japonica cultivar-group)] >gi|39545740|emb|CAE75966.1| OSJNBa0071I13.18 [Oryza sativa (japonica cultivar-group)] e_value:0</td><td>11670.m05544|LOC_Os04g56230|protein farnesyl pyrophosphate synthase e_value:0</td><td>0.141</td><td>(0.0698 0.4932)</td><td>0.0698</td><td>0.4932</td></tr>
<tr><td>470</td><td>WT004_K14_CLUSTER_5611</td><td>gi|50936989|ref|XP_478022.1| unknown protein [Oryza sativa (japonica cultivar-group)] >gnl|BL_ORD_ID|1526687 gi|34393236|dbj|BAC83086.1| unknown protein [Oryza sativa (japonica cultivar-group)] e_value:1.8181e-49</td><td>11673.m02702|LOC_Os07g28610|protein expressed protein e_value:2.80086e-51</td><td>0.259</td><td>(0.1436 0.5540)</td><td>0.1436</td><td>0.554</td></tr>
<tr><td>471</td><td>SET1_D15_CLUSTER_32934</td><td>gi|31433494|gb|AAP54999.1| putative ethylene-forming enzyme [Oryza sativa (japonica cultivar-group)] >gi|37536820|ref|NP_922712.1| putative ethylene-forming enzyme [Oryza sativa (japonica cultivar-group)] >gi|18873856|gb|AAL79802.1| putative ethylen</td><td>11676.m03769|LOC_Os10g41020|protein putative ethylene-forming enzyme e_value:3.658e-149</td><td>0.247</td><td>(0.1701 0.6893)</td><td>0.1701</td><td>0.6893</td></tr>
<tr><td>472</td><td>SET1_D14_CLUSTER_32934</td><td>gi|31433494|gb|AAP54999.1| putative ethylene-forming enzyme [Oryza sativa (japonica cultivar-group)] >gi|37536820|ref|NP_922712.1| putative ethylene-forming enzyme [Oryza sativa (japonica cultivar-group)] >gi|18873856|gb|AAL79802.1| putative ethylen</td><td>11676.m03769|LOC_Os10g41020|protein putative ethylene-forming enzyme e_value:3.658e-149</td><td>0.247</td><td>(0.1701 0.6893)</td><td>0.1701</td><td>0.6893</td></tr>
<tr><td>473</td><td>WT003_N15_CLUSTER_34702</td><td>gi|51964188|ref|XP_506878.1| PREDICTED OJ1626_B09.5 gene product [Oryza sativa (japonica cultivar-group)] >gnl|BL_ORD_ID|1582720 gi|50910980|ref|XP_466923.1| putative nitrilase 1 [Oryza sativa (japonica cultivar-group)] >gnl|BL_ORD_ID|1582720 gi|493</td><td>11668.m04046|LOC_Os02g42330|protein nitrilase-like protein [imported] - rice e_value:8.24973e-175</td><td>0.14</td><td>(0.0653 0.4663)</td><td>0.0653</td><td>0.4663</td></tr>
<tr><td>474</td><td>WT006_J04_CLUSTER_31910</td><td>gi|21593463|gb|AAM65430.1| unknown [Arabidopsis thaliana] >gnl|BL_ORD_ID|1218421 gi|20259567|gb|AAM14126.1| unknown protein [Arabidopsis thaliana] >gnl|BL_ORD_ID|1218421 gi|15810565|gb|AAL07170.1| unknown protein [Arabidopsis thaliana] >gnl|BL_OR</td><td>11669.m01070|LOC_Os03g11200|protein expressed protein e_value:0</td><td>0.233</td><td>(0.1544 0.6625)</td><td>0.1544</td><td>0.6625</td></tr>
<tr><td>475</td><td>WT013_N21_CLUSTER_32059</td><td>gi|21436461|gb|AAM51431.1| unknown protein [Arabidopsis thaliana] >gnl|BL_ORD_ID|1208772 gi|17979159|gb|AAL49775.1| unknown protein [Arabidopsis thaliana] >gnl|BL_ORD_ID|1208772 gi|22331896|ref|NP_191667.2| fructosamine kinase family protein [Arabid</td><td>11669.m00189|LOC_Os03g02640|protein Fructosamine kinase e_value:4.33715e-176</td><td>0.089</td><td>(0.0615 0.6887)</td><td>0.0615</td><td>0.6887</td></tr>
<tr><td>476</td><td>WT013_C10_CLUSTER_24124</td><td>gi|20259017|gb|AAM14224.1| unknown protein [Arabidopsis thaliana] >gnl|BL_ORD_ID|1208057 gi|17380816|gb|AAL36095.1| unknown protein [Arabidopsis thaliana] >gnl|BL_ORD_ID|1208057 gi|9758109|dbj|BAB08581.1| unnamed protein product [Arabidopsis thalian</td><td>11686.m00841|LOC_Os12g08810|protein expressed protein e_value:0</td><td>0.129</td><td>(0.0743 0.5760)</td><td>0.0743</td><td>0.576</td></tr>
<tr><td>477</td><td>WT005_G13_CLUSTER_31867</td><td>gi|52854318|gb|AAU88198.1| somatic embryogenesis protein kinase 1 [Oryza sativa (japonica cultivar-group)] e_value:0</td><td>11670.m03646|LOC_Os04g38480|protein somatic embryogenesis receptor-like kinase 1 e_value:0</td><td>0.108</td><td>(0.0495 0.4575)</td><td>0.0495</td><td>0.4575</td></tr>
<tr><td>478</td><td>WT003_O07_CLUSTER_2885</td><td>gi|50938629|ref|XP_478842.1| putative succinyl-CoA ligase alpha subunit [Oryza sativa (japonica cultivar-group)] >gnl|BL_ORD_ID|1529726 gi|34393512|dbj|BAC83073.1| putative succinyl-CoA ligase alpha subunit [Oryza sativa (japonica cultivar-</td><td>11673.m03792|LOC_Os07g38970|protein succinyl-CoA ligase alpha subunit e_value:2.13649e-180</td><td>0.062</td><td>(0.0220 0.3578)</td><td>0.022</td><td>0.3578</td></tr>
<tr><td>479</td><td>SET4_O09_CLUSTER_24248</td><td>gi|34911416|ref|NP_917055.1| putative threonine synthase [Oryza sativa (japonica cultivar-group)] >gi|22535519|dbj|BAC10696.1| threonine synthase-like [Oryza sativa (japonica cultivar-group)] e_value:0</td><td>11667.m04860|LOC_Os01g49890|protein threonine synthase, putative e_value:0</td><td>0.103</td><td>(0.0492 0.4768)</td><td>0.0492</td><td>0.4768</td></tr>
<tr><td>480</td><td>WT007_L13_CLUSTER_4588</td><td>gi|12324600|gb|AAG52261.1| unknown protein; 19658-19089 [Arabidopsis thaliana] e_value:3.69745e-55</td><td>11667.m03952|LOC_Os01g41040|protein expressed protein e_value:7.26221e-77</td><td>0.165</td><td>(0.0459 0.2784)</td><td>0.0459</td><td>0.2784</td></tr>
<tr><td>481</td><td>WT008_B24_CLUSTER_8548</td><td>gi|56783880|dbj|BAD81292.1| putative VHS1 protein [Oryza sativa (japonica cultivar-group)] e_value:0</td><td>11667.m01294|LOC_Os01g13160|protein expressed protein</td><td>0.228</td><td>(0.0917 0.4023)</td><td>0.0917</td><td>0.4023</td></tr>
<tr><td>482</td><td>WT013_L16_CLUSTER_7966</td><td>gi|53980840|gb|AAV24761.1| unknown protein [Oryza sativa (japonica cultivar-group)] e_value:0</td><td>11682.m01406|LOC_Os05g15140|protein expressed protein</td><td>0.406</td><td>(0.2256 0.5557)</td><td>0.2256</td><td>0.5557</td></tr>
<tr><td>483</td><td>WT013_B01_CLUSTER_32127</td><td>gi|50252173|dbj|BAD28168.1| putative LepA protein [Oryza sativa (japonica cultivar-group)] >gnl|BL_ORD_ID|235098 gi|50251243|dbj|BAD28023.1| putative LepA protein [Oryza sativa (japonica cultivar-group)] e_value:0</td><td>11668.m00576|LOC_Os02g06300|protein GTP-binding protein LepA e_value:0</td><td>0.171</td><td>(0.0819 0.4784)</td><td>0.0819</td><td>0.4784</td></tr>
<tr><td>484</td><td>SET4_D11_CLUSTER_15006</td><td>gi|62733814|gb|AAX95923.1| transposon protein, putative, unclassified [Oryza sativa (japonica cultivar-group)] e_value:4.44202e-88</td><td>11687.m02830|LOC_Os11g30910|protein Sulfotransferase domain, putative e_value:2.93888e-93</td><td>0.626</td><td>(0.4102 0.6549)</td><td>0.4102</td><td>0.6549</td></tr>
<tr><td>485</td><td>SET1_P22_CLUSTER_10591</td><td>gi|40538953|gb|AAR87210.1| expressed protein [Oryza sativa (japonica cultivar-group)] >gi|50901390|ref|XP_463128.1| expressed protein [Oryza sativa (japonica cultivar-group)] e_value:1.84166e-35</td><td>11686.m03876|LOC_Os12g39220|protein expressed protein e_value:7.07018e-74</td><td>0.167</td><td>(0.1169 0.7007)</td><td>0.1169</td><td>0.7007</td></tr>
<tr><td>486</td><td>WT013_F06_CLUSTER_11561</td><td>gi|50932223|ref|XP_475639.1| unknown protein [Oryza sativa (japonica cultivar-group)] >gnl|BL_ORD_ID|1597160 gi|46981334|gb|AAT07652.1| unknown protein [Oryza sativa (japonica cultivar-group)] e_value:0</td><td>11682.m00651|LOC_Os05g06980|protein At5g13560 e_value:0</td><td>0.206</td><td>(0.0988 0.4795)</td><td>0.0988</td><td>0.4795</td></tr>
<tr><td>487</td><td>WT012_N10_CLUSTER_4139</td><td>gi|32489775|emb|CAE04677.1| OSJNBb0018A10.6 [Oryza sativa (japonica cultivar-group)] >gnl|BL_ORD_ID|1535350 gi|50922673|ref|XP_471697.1| OSJNBb0018A10.6 [Oryza sativa (japonica cultivar-group)] e_value:9.60414e-35</td><td>11670.m02188|LOC_Os04g23550|protein Helix-loop-helix DNA-binding domain, putative e_value:2.34377e-36</td><td>0.636</td><td>(0.5013 0.7886)</td><td>0.5013</td><td>0.7886</td></tr>
<tr><td>488</td><td>WT011_M06_CLUSTER_16273</td><td>gi|42565893|ref|NP_190907.2| transducin family protein / WD-40 repeat family protein [Arabidopsis thaliana] e_value:0</td><td>11686.m00696|LOC_Os12g07450|protein expressed protein</td><td>0.105</td><td>(0.0701 0.6698)</td><td>0.0701</td><td>0.6698</td></tr>
<tr><td>489</td><td>WT008_F24_CLUSTER_26214</td><td>gi|31433575|gb|AAP55073.1| putative chlorophyll synthase [Oryza sativa (japonica cultivar-group)] >gnl|BL_ORD_ID|2013096 gi|37536968|ref|NP_922786.1| putative chlorophyll synthase [Oryza sativa (japonica cultivar-group)] >gnl|BL_ORD_ID|2013096 gi|18</td><td>11676.m03853|LOC_Os10g41780|protein putative chlorophyll synthase e_value:0</td><td>0.087</td><td>(0.0730 0.8381)</td><td>0.073</td><td>0.8381</td></tr>
<tr><td>490</td><td>WT003_O11_CLUSTER_32863</td><td>gi|40538958|gb|AAR87215.1| expressed protein [Oryza sativa (japonica cultivar-group)] >gnl|BL_ORD_ID|2058568 gi|50901384|ref|XP_463125.1| expressed protein [Oryza sativa (japonica cultivar-group)] e_value:1.24942e-176</td><td>11669.m04068|LOC_Os03g41080|protein expressed protein e_value:3.31556e-178</td><td>0.194</td><td>(0.0941 0.4854)</td><td>0.0941</td><td>0.4854</td></tr>
<tr><td>491</td><td>WT009_G06_CLUSTER_4552</td><td>gi|38347184|emb|CAE02408.2| OSJNBa0024J22.12 [Oryza sativa (japonica cultivar-group)] >gnl|BL_ORD_ID|1536020 gi|50922769|ref|XP_471745.1| OSJNBa0024J22.12 [Oryza sativa (japonica cultivar-group)] e_value:0</td><td>11670.m02261|LOC_Os04g24220|protein Protein kinase domain, putative e_value:0</td><td>0.19</td><td>(0.2873 1.5139)</td><td>0.2873</td><td>1.5139</td></tr>
<tr><td>492</td><td>SET1_C10_CLUSTER_18371</td><td>gi|52076868|dbj|BAD45881.1| putative ionotropic glutamate receptor ortholog GLR6 [Oryza sativa (japonica cultivar-group)] >gi|52076586|dbj|BAD45488.1| putative ionotropic glutamate receptor ortholog GLR6 [Oryza sativa (japonica cultivar-group)] e_value</td><td>11680.m04605|LOC_Os06g46670|protein Receptor family ligand binding region, putative e_value:0</td><td>0.265</td><td>(0.1377 0.5200)</td><td>0.1377</td><td>0.52</td></tr>
<tr><td>493</td><td>WT008_O10_CLUSTER_32852</td><td>gi|50928883|ref|XP_473969.1| OSJNBb0060E08.11 [Oryza sativa (japonica cultivar-group)] >gnl|BL_ORD_ID|1592598 gi|39545840|emb|CAE04748.3| OSJNBb0060E08.11 [Oryza sativa (japonica cultivar-group)] e_value:1.98618e-131</td><td>11670.m05247|LOC_Os04g53490|protein expressed protein e_value:2.83804e-152</td><td>0.14</td><td>(0.1363 0.9762)</td><td>0.1363</td><td>0.9762</td></tr>
<tr><td>494</td><td>WT012_M14_CLUSTER_11370</td><td>gi|55168101|gb|AAV43969.1| 'putative glucan 1,3-beta-glucosidase' [Oryza sativa (japonica cultivar-group)] e_value:0</td><td>11682.m01443|LOC_Os05g15510|protein Cellulase (glycosyl hydrolase family 5), putative e_value:0</td><td>0.178</td><td>(0.1684 0.9466)</td><td>0.1684</td><td>0.9466</td></tr>
<tr><td>495</td><td>WT010_O08_CLUSTER_14863</td><td>gi|50726258|dbj|BAD33834.1| acyl-CoA thioester hydrolase-like [Oryza sativa (japonica cultivar-group)] e_value:0</td><td>11681.m03100|LOC_Os09g34190|protein expressed protein</td><td>0.239</td><td>(0.1436 0.6016)</td><td>0.1436</td><td>0.6016</td></tr>
<tr><td>496</td><td>SET6_A01_CLUSTER_33540</td><td>gi|50924053|ref|XP_472387.1| OJ000315_02.15 [Oryza sativa (japonica cultivar-group)] >gi|32487911|emb|CAE05370.1| OJ000315_02.15 [Oryza sativa (japonica cultivar-group)] e_value:1.12249e-114</td><td>11670.m03185|LOC_Os04g33220|protein expressed protein e_value:1.52874e-116</td><td>0.338</td><td>(0.1148 0.3393)</td><td>0.1148</td><td>0.3393</td></tr>
<tr><td>497</td><td>WT012_M19_CLUSTER_20576</td><td>gi|50929891|ref|XP_474473.1| OSJNBb0020J19.5 [Oryza sativa (japonica cultivar-group)] >gnl|BL_ORD_ID|1594822 gi|38567827|emb|CAE05776.2| OSJNBb0020J19.5 [Oryza sativa (japonica cultivar-group)] e_value:2.10741e-46</td><td>11670.m05899|LOC_Os04g59500|protein ribosomal protein S17, putative e_value:1.84114e-47</td><td>1.091</td><td>(0.2708 0.2483)</td><td>0.2708</td><td>0.2483</td></tr>
<tr><td>498</td><td>SET1_B20_CLUSTER_22737</td><td>gi|50725153|dbj|BAD33770.1| unknown protein [Oryza sativa (japonica cultivar-group)] e_value:3.27002e-109</td><td>11681.m03197|LOC_Os09g36040|protein AT4g27700/T29A15_190 e_value:7.83553e-111</td><td>0.133</td><td>(0.0677 0.5102)</td><td>0.0677</td><td>0.5102</td></tr>
<tr><td>499</td><td>WT009_N14_CLUSTER_6476</td><td>gi|62701920|gb|AAX92993.1| signal peptide peptidase, putative [Oryza sativa (japonica cultivar-group)] e_value:0</td><td>11687.m02186|LOC_Os11g24540|protein signal peptide peptidase, putative e_value:0</td><td>0.204</td><td>(0.1072 0.5257)</td><td>0.1072</td><td>0.5257</td></tr>
<tr><td>500</td><td>WT009_I24_CLUSTER_5378</td><td>gi|50725468|dbj|BAD32939.1| putative beta-ketoacyl-CoA synthase [Oryza sativa (japonica cultivar-group)] e_value:0</td><td>11687.m03503|LOC_Os11g37900|protein fatty acid elongase, putative e_value:0</td><td>0.263</td><td>(0.1299 0.4933)</td><td>0.1299</td><td>0.4933</td></tr>
<tr><td>501</td><td>WT009_I14_CLUSTER_29876</td><td>gi|50912237|ref|XP_467526.1| putative phosphatidylinositol transfer [Oryza sativa (japonica cultivar-group)] >gnl|BL_ORD_ID|1582672 gi|45735980|dbj|BAD13009.1| putative phosphatidylinositol transfer [Oryza sativa (japonica cultivar-group)] e_value:0</td><td>11668.m04761|LOC_Os02g48990|protein CRAL/TRIO, N-terminus, putative e_value:0</td><td>0.159</td><td>(0.0804 0.5044)</td><td>0.0804</td><td>0.5044</td></tr>
<tr><td>502</td><td>WT008_M20_CLUSTER_31219</td><td>gi|57900102|dbj|BAD88164.1| unknown protein [Oryza sativa (japonica cultivar-group)] e_value:6.20275e-121</td><td>11687.m02937|LOC_Os11g31960|protein expressed protein</td><td>0.348</td><td>(0.2098 0.6033)</td><td>0.2098</td><td>0.6033</td></tr>
<tr><td>503</td><td>WT008_E23_CLUSTER_24193</td><td>gi|50912463|ref|XP_467639.1| unknown protein [Oryza sativa (japonica cultivar-group)] >gnl|BL_ORD_ID|1582748 gi|51964342|ref|XP_506956.1| PREDICTED P0643A10.28 gene product [Oryza sativa (japonica cultivar-group)] >gnl|BL_ORD_ID|1582748 gi|46390662|</td><td>11668.m04869|LOC_Os02g50010|protein hypothetical protein e_value:0</td><td>0.166</td><td>(0.1051 0.6324)</td><td>0.1051</td><td>0.6324</td></tr>
<tr><td>504</td><td>WT006_H06_CLUSTER_27193</td><td>gi|53792565|dbj|BAD53554.1| putative amino acid transporter [Oryza sativa (japonica cultivar-group)] e_value:0</td><td>11680.m03503|LOC_Os06g36180|protein amino acid permease 6 e_value:0</td><td>0.203</td><td>(0.0994 0.4905)</td><td>0.0994</td><td>0.4905</td></tr>
<tr><td>505</td><td>WT005_O09_CLUSTER_7009</td><td>gi|50921083|ref|XP_470902.1| hypothetical protein [Oryza sativa (japonica cultivar-group)] >gnl|BL_ORD_ID|1588872 gi|29788823|gb|AAP03369.1| hypothetical protein [Oryza sativa (japonica cultivar-group)] e_value:0</td><td>11669.m02037|LOC_Os03g19990|protein hypothetical protein e_value:0</td><td>0.154</td><td>(0.1311 0.8524)</td><td>0.1311</td><td>0.8524</td></tr>
<tr><td>506</td><td>WT005_D23_CLUSTER_23684</td><td>gi|52077473|dbj|BAD45037.1| transmembrane domain, a coiled coil-4 domain, of eukaryotic origin (91.3 kD) -like protein [Oryza sativa (japonica cultivar-group)] e_value:0</td><td>11667.m01949|LOC_Os01g19760|protein At1g35220 e_value:0</td><td>0.232</td><td>(0.1217 0.5242)</td><td>0.1217</td><td>0.5242</td></tr>
<tr><td>507</td><td>WT005_C05_CLUSTER_27167</td><td>gi|50938457|ref|XP_478756.1| unknown protein [Oryza sativa (japonica cultivar-group)] >gnl|BL_ORD_ID|1529484 gi|33146768|dbj|BAC79686.1| unknown protein [Oryza sativa (japonica cultivar-group)] e_value:3.87859e-43</td><td>11673.m03705|LOC_Os07g38150|protein expressed protein e_value:6.91962e-45</td><td>0.361</td><td>(0.2558 0.7093)</td><td>0.2558</td><td>0.7093</td></tr>
<tr><td>508</td><td>WT004_C16_CLUSTER_28670</td><td>gi|38346871|emb|CAE02220.2| OSJNBb0002N06.11 [Oryza sativa (japonica cultivar-group)] >gnl|BL_ORD_ID|1535329 gi|50923355|ref|XP_472038.1| OSJNBb0002N06.11 [Oryza sativa (japonica cultivar-group)] e_value:4.84082e-157</td><td>11674.m01946|LOC_Os08g20200|protein AJ459250 fatty acyl coA reductase e_value:5.68223e-149</td><td>0.146</td><td>(0.3621 2.4815)</td><td>0.3621</td><td>2.4815</td></tr>
<tr><td>509</td><td>WT003_A03_CLUSTER_3526</td><td>gi|62733943|gb|AAX96052.1| Lipase, putative [Oryza sativa (japonica cultivar-group)] e_value:0</td><td>11687.m00843|LOC_Os11g09010|protein Lipase, putative</td><td>0.216</td><td>(0.1873 0.8657)</td><td>0.1873</td><td>0.8657</td></tr>
<tr><td>510</td><td>SET6_G22_CLUSTER_27193</td><td>gi|53792565|dbj|BAD53554.1| putative amino acid transporter [Oryza sativa (japonica cultivar-group)] e_value:0</td><td>11680.m03503|LOC_Os06g36180|protein amino acid permease 6 e_value:0</td><td>0.197</td><td>(0.0965 0.4910)</td><td>0.0965</td><td>0.491</td></tr>
</table>

PDF Complete
Your complimentary use period has ended. Thank you for using PDF Complete.
Click Here to upgrade to Unlimited Pages and Expanded Features

<table>
<tr><td>511</td><td>SET3_J09_CLUSTER_19122</td><td>gi|50923303|ref|XP_472012.1| OSJNBa0020I02.12 [Oryza sativa (japonica cultivar-group)] >gi|38346498|emb|CAD40347.2| OSJNBa0020I02.12 [Oryza sativa (japonica cultivar-group)] e_value:1.57377e-120</td><td>11670.m02649|LOC_Os04g28060|protein expressed protein e_value:6.31087e-122</td><td>0.175</td><td>(0.1019 0.5821)</td><td>0.1019</td><td>0.5821</td></tr>
<tr><td>512</td><td>SET3_I06_CLUSTER_21703</td><td>gi|50943119|ref|XP_481087.1| GCN5-related N-acetyltransferase-like [Oryza sativa (japonica cultivar-group)] >gi|37806163|dbj|BAC99667.1| GCN5-related N-acetyltransferase-like [Oryza sativa (japonica cultivar-group)]</td><td>11674.m01566|LOC_Os08g16010|protein acetyltransferase, GNAT family, putative e_value:6.46118e-116</td><td>0.221</td><td>(0.1279 0.5784)</td><td>0.1279</td><td>0.5784</td></tr>
<tr><td>513</td><td>SET3_G12_CLUSTER_30035</td><td>gi|34907894|ref|NP_915294.1| P0439E11.11 [Oryza sativa (japonica cultivar-group)] e_value:0</td><td>11667.m06080|LOC_Os01g61040|protein Transmembrane amino acid transporter protein e_value:0</td><td>0.374</td><td>(0.1040 0.2779)</td><td>0.104</td><td>0.2779</td></tr>
<tr><td>514</td><td>SET3_D04_CLUSTER_30850</td><td>gi|50905155|ref|XP_464066.1| unknown protein [Oryza sativa (japonica cultivar-group)] >gi|42409262|dbj|BAD10525.1| unknown protein [Oryza sativa (japonica cultivar-group)] e_value:1.84547e-47</td><td>11668.m00389|LOC_Os02g04520|protein expressed protein e_value:1.53688e-49</td><td>0.565</td><td>(0.2179 0.3858)</td><td>0.2179</td><td>0.3858</td></tr>
<tr><td>515</td><td>SET2_H15_CLUSTER_28430</td><td>gi|18449100|gb|AAL69988.1| cold acclimation WCOR413-like protein gamma form [Hordeum vulgare subsp. vulgare] e_value:3.1181e-106</td><td>11682.m04717|LOC_Os05g49170|protein Cold acclimation protein WCOR413 e_value:4.10038e-77</td><td>0.556</td><td>(0.1982 0.3565)</td><td>0.1982</td><td>0.3565</td></tr>
<tr><td>516</td><td>SET1_L20_CLUSTER_30689</td><td>gi|51535644|dbj|BAD37618.1| putative 50S ribosomal protein L35 [Oryza sativa (japonica cultivar-group)] >gi|51535422|dbj|BAD37321.1| putative 50S ribosomal protein L35 [Oryza sativa (japonica cultivar-group)] e_value:7.62642e-48</td><td>11680.m04314|LOC_Os06g43900|protein ribosomal protein L35, putative e_value:1.3832e-49</td><td>0.398</td><td>(0.1521 0.3820)</td><td>0.1521</td><td>0.382</td></tr>
<tr><td>517</td><td>SET1_H20_CLUSTER_19000</td><td>gi|55733820|gb|AAV59327.1| unknown protein [Oryza sativa (japonica cultivar-group)] >gi|50933345|ref|XP_476200.1| unknown protein [Oryza sativa (japonica cultivar-group)] e_value:2.22067e-68</td><td>11682.m01248|LOC_Os05g13580|protein EF hand, putative e_value:2.09667e-71</td><td>0.224</td><td>(0.0912 0.4077)</td><td>0.0912</td><td>0.4077</td></tr>
<tr><td>518</td><td>SET1__C01_CLUSTER_30784</td><td>gi|50918701|ref|XP_469747.1| putative helicase [Oryza sativa] >gi|18087663|gb|AAL58955.1| putative helicase [Oryza sativa] e_value:0</td><td>11669.m05445|LOC_Os03g53760|protein putative helicase</td><td>0.089</td><td>(0.0408 0.4589)</td><td>0.0408</td><td>0.4589</td></tr>
<tr><td>519</td><td>WT012_F10_CLUSTER_14014</td><td>gi|52353619|gb|AAU44185.1| unknown protein [Oryza sativa (japonica cultivar-group)] e_value:0</td><td>11682.m03781|LOC_Os05g39800|protein Nodulin, putative</td><td>0.491</td><td>(0.1501 0.3060)</td><td>0.1501</td><td>0.306</td></tr>
<tr><td>520</td><td>WT010_N07_CLUSTER_11781</td><td>gi|50251839|dbj|BAD27768.1| mitochondrial substrate carrier protein-like [Oryza sativa (japonica cultivar-group)] >gnl|BL_ORD_ID|228867 gi|50251364|dbj|BAD28391.1| mitochondrial substrate carrier protein-like [Oryza sativa (japonica cultivar-group)] e_</td><td>11668.m04295|LOC_Os02g44570|protein Mitochondrial carrier protein, putative e_value:0</td><td>0.386</td><td>(0.2361 0.6122)</td><td>0.2361</td><td>0.6122</td></tr>
<tr><td>521</td><td>WT002_H19_CLUSTER_12653</td><td>gi|55770587|ref|XP_550284.1| unknown protein [Oryza sativa (japonica cultivar-group)] >gnl|BL_ORD_ID|1753715 gi|55296345|dbj|BAD68261.1| unknown protein [Oryza sativa (japonica cultivar-group)] e_value:6.5165e-158</td><td>11667.m00707|LOC_Os01g07700|protein expressed protein e_value:1.59182e-159</td><td>0.225</td><td>(0.1344 0.5975)</td><td>0.1344</td><td>0.5975</td></tr>
<tr><td>522</td><td>SET1_O17_CLUSTER_5879</td><td>gi|21536896|gb|AAM61228.1| unknown [Arabidopsis thaliana] e_value:2.68842e-66</td><td>11669.m01819|LOC_Os03g17990|protein Yip1 domain, putative e_value:8.69209e-133</td><td>0.115</td><td>(0.0472 0.4091)</td><td>0.0472</td><td>0.4091</td></tr>
<tr><td>523</td><td>WT002_P14_CLUSTER_15211</td><td>gi|50912891|ref|XP_467853.1| putative ribosomal protein L29 [Oryza sativa (japonica cultivar-group)] >gnl|BL_ORD_ID|1582970 gi|51979368|ref|XP_507532.1| PREDICTED P0627E03.11 gene product [Oryza sativa (japonica cultivar-group)] >gnl|BL_ORD_ID|15829</td><td>11668.m05062|LOC_Os02g51790|protein ribosomal protein L29, putative e_value:4.47367e-65</td><td>0.372</td><td>(0.1075 0.2890)</td><td>0.1075</td><td>0.289</td></tr>
<tr><td>524</td><td>WT009_E01_CLUSTER_18428</td><td>gi|50726252|dbj|BAD33828.1| C2 domain-containing protein-like [Oryza sativa (japonica cultivar-group)] e_value:0</td><td>11681.m03094|LOC_Os09g34130|protein At3g59660 e_value:0</td><td>0.253</td><td>(0.1266 0.4993)</td><td>0.1266</td><td>0.4993</td></tr>
<tr><td>525</td><td>WT003_N20_CLUSTER_15312</td><td>gi|7267930|emb|CAB78272.1| copper amine oxidase-like protein [Arabidopsis thaliana] >gnl|BL_ORD_ID|1458406 gi|5281040|emb|CAB45976.1| copper amine oxidase-like protein [Arabidopsis thaliana] >gnl|BL_ORD_ID|1458406 gi|11279261|pir||T48139 copper amin</td><td>11673.m03737|LOC_Os07g38440|protein Copper amine oxidase, enzyme domain, putative e_value:2.35843e-173</td><td>0.671</td><td>(0.4758 0.7088)</td><td>0.4758</td><td>0.7088</td></tr>
<tr><td>526</td><td>WT004_O03_CLUSTER_113</td><td>gi|11066973|gb|AAG28786.1| asparaginase [Hordeum vulgare] e_value:0</td><td>11670.m05487|LOC_Os04g55710|protein transposon protein, putative, unclassified e_value:9.7413e-173</td><td>0.181</td><td>(0.0696 0.3846)</td><td>0.0696</td><td>0.3846</td></tr>
<tr><td>527</td><td>SET3_K17_CLUSTER_16038</td><td>gi|56202321|dbj|BAD73780.1| putative SF16 protein [Oryza sativa (japonica cultivar-group)] e_value:0</td><td>11667.m06156|LOC_Os01g61720|protein IQ calmodulin-binding motif, putative e_value:0</td><td>0.164</td><td>(0.0937 0.5699)</td><td>0.0937</td><td>0.5699</td></tr>
<tr><td>528</td><td>WT013_H19_CLUSTER_4871</td><td>gi|50940417|ref|XP_479736.1| putative shoot gravitropism 2 [Oryza sativa (japonica cultivar-group)] >gnl|BL_ORD_ID|1601236 gi|51964620|ref|XP_507094.1| PREDICTED P0007D08.37 gene product [Oryza sativa (japonica cultivar-group)] >gnl|BL_ORD_ID|160123</td><td>11674.m00099|LOC_Os08g01920|protein DDHD domain, putative e_value:0</td><td>0.2</td><td>(0.1142 0.5701)</td><td>0.1142</td><td>0.5701</td></tr>
<tr><td>529</td><td>WT008_K22_CLUSTER_14548</td><td>gi|50725434|dbj|BAD32906.1| putative receptor-like protein kinase [Oryza sativa (japonica cultivar-group)] e_value:0</td><td>11680.m03795|LOC_Os06g38970|protein Leucine Rich Repeat, putative e_value:0</td><td>0.293</td><td>(0.1540 0.5247)</td><td>0.154</td><td>0.5247</td></tr>
<tr><td>530</td><td>WT006_A24_CLUSTER_30030</td><td>gi|50940409|ref|XP_479732.1| unknown protein [Oryza sativa (japonica cultivar-group)] >gnl|BL_ORD_ID|1601232 gi|42408386|dbj|BAD09537.1| unknown protein [Oryza sativa (japonica cultivar-group)] e_value:0</td><td>11674.m00096|LOC_Os08g01890|protein expressed protein e_value:0</td><td>0.192</td><td>(0.0902 0.4689)</td><td>0.0902</td><td>0.4689</td></tr>
<tr><td>531</td><td>WT009_G15_CLUSTER_34200</td><td>gi|50917085|ref|XP_468939.1| putative ribosomal protein [Oryza sativa (japonica cultivar-group)] >gnl|BL_ORD_ID|1585030 gi|28209467|gb|AAO37485.1| putative ribosomal protein [Oryza sativa (japonica cultivar-group)] e_value:2.25953e-135</td><td>11669.m03448|LOC_Os03g34040|protein putative ribosomal protein S5 e_value:6.30149e-134</td><td>0.163</td><td>(0.1127 0.6897)</td><td>0.1127</td><td>0.6897</td></tr>
<tr><td>532</td><td>WT006_F22_CLUSTER_26539</td><td>gi|53792761|dbj|BAD53796.1| putative UOS1 [Oryza sativa (japonica cultivar-group)] e_value:0</td><td>11680.m04870|LOC_Os06g49120|protein expressed protein</td><td>0.183</td><td>(0.0952 0.5196)</td><td>0.0952</td><td>0.5196</td></tr>
<tr><td>533</td><td>SET6_K13_CLUSTER_26539</td><td>gi|53792761|dbj|BAD53796.1| putative UOS1 [Oryza sativa (japonica cultivar-group)] e_value:0</td><td>11680.m04870|LOC_Os06g49120|protein expressed protein</td><td>0.179</td><td>(0.0952 0.5325)</td><td>0.0952</td><td>0.5325</td></tr>
<tr><td>534</td><td>WT003_J21_CLUSTER_22181</td><td>gi|3758827|emb|CAA09867.1| amino acid selective channel protein [Hordeum vulgare subsp. vulgare] e_value:1.68197e-67</td><td>11682.m00124|LOC_Os05g02060|protein amino acid selective channel protein e_value:2.47686e-60</td><td>0.293</td><td>(0.1503 0.5123)</td><td>0.1503</td><td>0.5123</td></tr>
<tr><td>535</td><td>WT011__C02_CLUSTER_7720</td><td>gi|50909901|ref|XP_466439.1| putative glycine-rich protein [Oryza sativa (japonica cultivar-group)] >gnl|BL_ORD_ID|1577032 gi|46806283|dbj|BAD17491.1| putative glycine-rich protein [Oryza sativa (japonica cultivar-group)] >gnl|BL_ORD_ID|1577032 gi|4</td><td>11668.m03576|LOC_Os02g37490|protein Glycine rich protein family e_value:1.19039e-26</td><td>0.191</td><td>(0.2344 1.2282)</td><td>0.2344</td><td>1.2282</td></tr>
<tr><td>536</td><td>WT002_J12_CLUSTER_34799</td><td>gi|57900116|dbj|BAD88178.1| putative beta-glucosidase [Oryza sativa (japonica cultivar-group)] >gnl|BL_ORD_ID|972452 gi|57899653|dbj|BAD87322.1| putative beta-glucosidase [Oryza sativa (japonica cultivar-group)] e_value:0</td><td>11682.m02810|LOC_Os05g30350|protein Glycosyl hydrolase family 1 e_value:0</td><td>0.112</td><td>(0.2803 2.4929)</td><td>0.2803</td><td>2.4929</td></tr>
<tr><td>537</td><td>WT003_B21_CLUSTER_13736</td><td>gi|52076895|dbj|BAD45908.1| putative 50S ribosomal protein L24, chloroplast precursor [Oryza sativa (japonica cultivar-group)] >gnl|BL_ORD_ID|380363 gi|52076649|dbj|BAD45549.1| putative 50S ribosomal protein L24, chloroplast precursor [Oryza sativa (ja</td><td>11680.m04633|LOC_Os06g46930|protein ribosomal protein L24, putative e_value:9.69798e-87</td><td>0.155</td><td>(0.0769 0.4959)</td><td>0.0769</td><td>0.4959</td></tr>
<tr><td>538</td><td>SET3_N04_CLUSTER_10837</td><td>gi|21592331|gb|AAM64282.1| putative elongation factor P (EF-P) [Arabidopsis thaliana] >gi|21436359|gb|AAM51349.1| putative elongation factor P (EF-P) [Arabidopsis thaliana] >gi|17380806|gb|AAL36090.1| putative elongation factor P (EF-P) [Arabidopsis</td><td>11687.m00152|LOC_Os11g02450|protein Elongation factor P (EF-P), putative e_value:8.00764e-106</td><td>0.201</td><td>(0.0950 0.4720)</td><td>0.095</td><td>0.472</td></tr>
<tr><td>539</td><td>WT003_N06_CLUSTER_8608</td><td>gi|38345247|emb|CAD41091.2| OSJNBb0011N17.8 [Oryza sativa (japonica cultivar-group)] >gnl|BL_ORD_ID|1522074 gi|50925221|ref|XP_472912.1| OSJNBb0011N17.8 [Oryza sativa (japonica cultivar-group)] e_value:0</td><td>11670.m03874|LOC_Os04g40520|protein Raffinose synthase or seed imbibition protein Sip1 e_value:0</td><td>0.244</td><td>(0.1146 0.4698)</td><td>0.1146</td><td>0.4698</td></tr>
<tr><td>540</td><td>WT013_K10_CLUSTER_27094</td><td>gi|56784602|dbj|BAD81649.1| unknown protein [Oryza sativa (japonica cultivar-group)] >gnl|BL_ORD_ID|775516 gi|56784572|dbj|BAD81619.1| unknown protein [Oryza sativa (japonica cultivar-group)] e_value:0</td><td>11667.m01377|LOC_Os01g13940|protein expressed protein e_value:0</td><td>0.23</td><td>(0.1532 0.6668)</td><td>0.1532</td><td>0.6668</td></tr>
<tr><td>541</td><td>WT013_O21_CLUSTER_34396</td><td>gi|34899086|ref|NP_910889.1| unknown protein [Oryza sativa (japonica cultivar-group)] >gnl|BL_ORD_ID|1533405 gi|50510054|dbj|BAD30682.1| unknown protein [Oryza sativa (japonica cultivar-group)] >gnl|BL_ORD_ID|1533405 gi|22775630|dbj|BAC15484.1| unkn</td><td>11673.m04587|LOC_Os07g46520|protein REG-2-like, HAD superfamily (subfamily IA) hydrolase, putative e_value:2.15892e-135</td><td>0.195</td><td>(0.1016 0.5208)</td><td>0.1016</td><td>0.5208</td></tr>
<tr><td>542</td><td>WT012__C19_CLUSTER_33485</td><td>gi|498740|emb|CAA84022.1| beta-ketoacyl-ACP synthase [Hordeum vulgare subsp. vulgare] >gnl|BL_ORD_ID|2351379 gi|629772|pir||S47074 3-oxoacyl-[acyl-carrier-protein] synthase (EC 2.3.1.41) precursor [similarity] - barley e_value:0</td><td>11673.m04154|LOC_Os07g42420|protein Beta-ketoacyl synthase, N-terminal domain, putative e_value:0</td><td>0.107</td><td>(0.0536 0.4985)</td><td>0.0536</td><td>0.4985</td></tr>
<tr><td>543</td><td>WT002__C14_CLUSTER_10182</td><td>gi|31433320|gb|AAP54849.1| putative Mlo (pathogen resistance) protein [Oryza sativa (japonica cultivar-group)] >gnl|BL_ORD_ID|2012787 gi|37536520|ref|NP_922562.1| putative Mlo (pathogen resistance) protein [Oryza sativa (japonica cultivar-group)] >g</td><td>11676.m03603|LOC_Os10g39520|protein putative Mlo (pathogen resistance) protein e_value:0</td><td>0.153</td><td>(0.1065 0.6948)</td><td>0.1065</td><td>0.6948</td></tr>
<tr><td>544</td><td>WT004_P21_CLUSTER_23799</td><td>gi|510677|emb|CAA54042.1| protochlorophyilide reductase [Triticum aestivum] >gnl|BL_ORD_ID|2311860 gi|629829|pir||S39394 protochlorophyllide reductase (EC 1.3.1.33) precursor - wheat >gnl|BL_ORD_ID|2311860 gi|10720235|sp|Q41578|PORA_WHEAT</td><td>11670.m05760|LOC_Os04g58200|protein oxidoreductase, short chain dehydrogenase/reductase family, putative e_value:0</td><td>0.225</td><td>(0.1017 0.4525)</td><td>0.1017</td><td>0.4525</td></tr>
<tr><td>545</td><td>SET5_N04_CLUSTER_23799</td><td>gi|683476|emb|CAA59228.1| NADPH dehydrogenase [Hordeum vulgare] >gi|1076725|pir||S52285 NADPH2 dehydrogenase (EC 1.6.99.1) - barley >gi|10720236|sp|Q42850|PORB_HORVU Protochlorophyllide reductase B, chloroplast precursor (PCR B) (NADPH-protochloroph</td><td>11676.m03151|LOC_Os10g35370|protein putative dehydrogenase e_value:0</td><td>0.17</td><td>(0.0678 0.3991)</td><td>0.0678</td><td>0.3991</td></tr>
<tr><td>546</td><td>WT006_E17_CLUSTER_11952</td><td>gi|34908554|ref|NP_915624.1| putative kinase [Oryza sativa (japonica cultivar-group)] >gnl|BL_ORD_ID|1557565 gi|21644639|dbj|BAC01197.1| putative LSTK-1-like kinase [Oryza sativa (japonica cultivar-group)] >gnl|BL_ORD_ID|1557565 gi|15290125|dbj|BAB6</td><td>11667.m06461|LOC_Os01g64490|protein Protein kinase domain, putative e_value:0</td><td>0.318</td><td>(0.1381 0.4345)</td><td>0.1381</td><td>0.4345</td></tr>
<tr><td>547</td><td>SET4_K14_CLUSTER_22242</td><td>gi|57899183|dbj|BAD87235.1| thioredoxin M-like [Oryza sativa (japonica cultivar-group)] e_value:7.32736e-53</td><td>11670.m04347|LOC_Os04g44830|protein thioredoxin, putative e_value:4.92012e-19</td><td>0.595</td><td>(0.8569 1.4407)</td><td>0.8569</td><td>1.4407</td></tr>
<tr><td>548</td><td>SET6__C04_CLUSTER_10133</td><td>gi|1890575|emb|CAA63662.1| xyloglucan endotransglycosylase (XET) [Hordeum vulgare subsp. vulgare] >gi|7447412|pir||T06201 xyloglucan endo-1,4-beta-D-glucanase (EC 3.2.1.-) - barley e_value:7.57217e-164</td><td>11680.m04770|LOC_Os06g48200|protein xyloglucan endo-1,4-beta-D-glucanase (EC 3.2.1.-) - barley e_value:9.34327e-150</td><td>0.175</td><td>(0.0774 0.4419)</td><td>0.0774</td><td>0.4419</td></tr>
<tr><td>549</td><td>SET1_O18_CLUSTER_13536</td><td>gi|54287522|gb|AAV31266.1| unknown protein [Oryza sativa (japonica cultivar-group)] e_value:1.78827e-169</td><td>11682.m03050|LOC_Os05g32630|protein At3g05290/T12H1_26 e_value:4.11073e-171</td><td>0.091</td><td>(0.0419 0.4615)</td><td>0.0419</td><td>0.4615</td></tr>
<tr><td>550</td><td>SET4_F13_CLUSTER_22043</td><td>gi|56202288|dbj|BAD73747.1| unknown protein [Oryza sativa (japonica cultivar-group)] >gi|56202158|dbj|BAD73491.1| unknown protein [Oryza sativa (japonica cultivar-group)] e_value:1.39285e-44</td><td>11667.m04742|LOC_Os01g48820|protein hypothetical protein e_value:1.56788e-34</td><td>0.602</td><td>(0.8614 1.4311)</td><td>0.8614</td><td>1.4311</td></tr>
<tr><td>551</td><td>WT006_P22_CLUSTER_20674</td><td>gi|50934381|ref|XP_476718.1| putative prolyl-tRNA synthetase [Oryza sativa (japonica cultivar-group)] >gnl|BL_ORD_ID|1521328 gi|33146799|dbj|BAC79747.1| putative prolyl-tRNA synthetase [Oryza sativa (japonica cultivar-group)] e_value:0</td><td>11673.m00629|LOC_Os07g07060|protein prolyl-tRNA synthetase e_value:0</td><td>0.104</td><td>(0.0490 0.4690)</td><td>0.049</td><td>0.469</td></tr>
<tr><td>552</td><td>SET1_P12_CLUSTER_32384</td><td>gi|30102874|gb|AAP21355.1| At1g75690 [Arabidopsis thaliana] >gi|26450801|dbj|BAC42509.1| unknown protein [Arabidopsis thaliana] >gi|27311545|gb|AAO00738.1| unknown protein [Arabidopsis thaliana] >gi|15222330|ref|NP_177698.1| chaperone protein dna</td><td>11669.m01381|LOC_Os03g14040|protein expressed protein e_value:1.4672e-70</td><td>0.17</td><td>(0.1213 0.7145)</td><td>0.1213</td><td>0.7145</td></tr>
<tr><td>553</td><td>WT009_K05_CLUSTER_22224</td><td>gi|50904787|ref|XP_463882.1| unknown protein [Oryza sativa (japonica cultivar-group)] >gnl|BL_ORD_ID|1565831 gi|41052833|dbj|BAD07724.1| unknown protein [Oryza sativa (japonica cultivar-group)] e_value:2.38654e-156</td><td>11668.m00164|LOC_Os02g02520|protein expressed protein e_value:3.74686e-149</td><td>0.212</td><td>(0.1599 0.7542)</td><td>0.1599</td><td>0.7542</td></tr>
<tr><td>554</td><td>SET2_K15_CLUSTER_13668</td><td>gi|50931869|ref|XP_475462.1| unknown protein [Oryza sativa (japonica cultivar-group)] >gi|50080307|gb|AAT69641.1| unknown protein [Oryza sativa (japonica cultivar-group)] e_value:2.21007e-52</td><td>11682.m03939|LOC_Os05g41190|protein expressed protein e_value:2.95945e-54</td><td>0.49</td><td>(0.2654 0.5414)</td><td>0.2654</td><td>0.5414</td></tr>
<tr><td>555</td><td>SET1_F09_CLUSTER_25820</td><td>gi|50905187|ref|XP_464082.1| unknown protein [Oryza sativa (japonica cultivar-group)] >gi|42409278|dbj|BAD10541.1| unknown protein [Oryza sativa (japonica cultivar-group)] e_value:7.58901e-81</td><td>11668.m00402|LOC_Os02g04650|protein expressed protein e_value:1.30103e-82</td><td>0.207</td><td>(0.1321 0.6393)</td><td>0.1321</td><td>0.6393</td></tr>
</table>

PDF Complete
Your complimentary use period has ended. Thank you for using PDF Complete.
Click Here to upgrade to Unlimited Pages and Expanded Features

556	WT004_C20_CLUSTER_33718	gi|3328221|gb|AAC78473.1| thioredoxin peroxidase [Secale cereale] e_value:9.64894e-131	11668.m03158|LOC_Os02g33450|protein thioredoxin peroxidase e_value:3.92628e-120	0.149	(0.1032 0.6906)	0.1032	0.6906
557	WT005_F09_CLUSTER_15191	gi|54287502|gb|AAV31246.1| unknown protein [Oryza sativa (japonica cultivar-group)] >gnl|BL_ORD_ID|1420815 gi|53749294|gb|AAU90153.1| unknown protein [Oryza sativa (japonica cultivar-group)] e_value:8.11411e-45	11682.m00913|LOC_Os05g09400|protein plastid-specific ribosomal protein 4 precursor e_value:4.54185e-44	0.238	(0.1505 0.6326)	0.1505	0.6326
558	WT003_C17_CLUSTER_33364	gi|2695931|emb|CAA10984.1| hypothetical protein [Hordeum vulgare subsp. vulgare] >gnl|BL_ORD_ID|1083243 gi|6136559|sp|O48609|RRP3_HORVU Plastid-specific 30S ribosomal protein 3, chloroplast precursor (PSRP-3) >gnl|BL_ORD_ID|1083243 gi|7489373|pir||T	11670.m03676|LOC_Os04g38750|protein expressed protein e_value:1.645e-75	0.214	(0.1131 0.5288)	0.1131	0.5288
559	WT007_I12_CLUSTER_14610	gi|50911865|ref|XP_467340.1| putative protein kinase YakA [Oryza sativa (japonica cultivar-group)] >gnl|BL_ORD_ID|1580461 gi|41053118|dbj|BAD08061.1| putative protein kinase YakA [Oryza sativa (japonica cultivar-group)] >gnl|BL_ORD_ID|1580461 gi|410	11668.m04594|LOC_Os02g47410|protein Protein kinase domain, putative e_value:0	0.244	(0.1134 0.4644)	0.1134	0.4644
560	WT012_A06_CLUSTER_19481	gi|52353700|gb|AAU44266.1| unknown protein [Oryza sativa (japonica cultivar-group)] >gnl|BL_ORD_ID|1283068 gi|50080324|gb|AAT69658.1| unknown protein [Oryza sativa (japonica cultivar-group)] e_value:1.55908e-151	11682.m04232|LOC_Os05g44350|protein expressed protein e_value:3.45138e-153	0.116	(0.0495 0.4288)	0.0495	0.4288
561	SET4_M23_CLUSTER_12920	gi|52077208|dbj|BAD46252.1| unknown protein [Oryza sativa (japonica cultivar-group)] e_value:0	11681.m03544|LOC_Os09g39390|protein oxidoreductase, aldo/keto reductase family, putative e_value:0	0.094	(0.0842 0.8911)	0.0842	0.8911
562	WT006_A05_CLUSTER_4321	gi|31432321|gb|AAP53971.1| putative phosphoglycerate kinase [Oryza sativa (japonica cultivar-group)] >gnl|BL_ORD_ID|2011499 gi|37534764|ref|NP_921684.1| putative phosphoglycerate kinase [Oryza sativa (japonica cultivar-group)] e_value:0	11676.m02640|LOC_Os10g30550|protein phosphoglycerate kinase, putative e_value:0	0.286	(0.1246 0.4358)	0.1246	0.4358
563	WT011_P07_CLUSTER_8252	gi|51536170|dbj|BAD38343.1| unknown protein [Oryza sativa (japonica cultivar-group)] >gnl|BL_ORD_ID|243114 gi|51535145|dbj|BAD37835.1| unknown protein [Oryza sativa (japonica cultivar-group)] e_value:1.34983e-168	11680.m04410|LOC_Os06g44840|protein prenyltransferase, UbiA family, putative e_value:2.03111e-98	0.325	(0.2780 0.8545)	0.278	0.8545
564	WT008_O21_CLUSTER_33061	gi|50913067|ref|XP_467941.1| putative galactoside 2-alpha-L-fucosyltransferase [Oryza sativa (japonica cultivar-group)] >gnl|BL_ORD_ID|1583058 gi|46805722|dbj|BAD17109.1| putative galactoside 2-alpha-L-fucosyltransferase / xyloglucan alpha-(1,2)-	11668.m05154|LOC_Os02g52610|protein probable fucosyltransferase 8 (ec 2.4.1.-) (atfut8). e_value:0	0.263	(0.1588 0.6050)	0.1588	0.605
565	SET4_G05_CLUSTER_34128	gi|34914598|ref|NP_918646.1| P0520B06.12 [Oryza sativa (japonica cultivar-group)] >gi|20804493|dbj|BAB92188.1| tetratricopeptide repeat(TPR)-containing protein-like [Oryza sativa (japonica cultivar-group)] e_value:3.01239e-120	11667.m03087|LOC_Os01g32730|protein TPR Domain, putative e_value:9.07692e-122	0.216	(0.1638 0.7588)	0.1638	0.7588
566	WT006_N08_CLUSTER_13996	gi|34902200|ref|NP_912446.1| Putative hydroxymethylglutaryl coenzyme A synthase [Oryza sativa (japonica cultivar-group)] >gnl|BL_ORD_ID|1584315 gi|27452903|gb|AAO15287.1| Putative hydroxymethylglutaryl coenzyme A synthase [Oryza sativa (japonica	11669.m00198|LOC_Os03g02710|protein hydroxymethylglutaryl-CoA synthase e_value:0	0.105	(0.0593 0.5658)	0.0593	0.5658
567	WT004_B17_CLUSTER_31590	gi|50937463|ref|XP_478259.1| receptor-like protein kinase-like protein [Oryza sativa (japonica cultivar-group)] >gnl|BL_ORD_ID|1527333 gi|34393327|dbj|BAC83274.1| receptor-like protein kinase-like protein [Oryza sativa (japonica cultivar-group)] e_valu	11673.m03038|LOC_Os07g31840|protein F3M18.23 e_value:0	0.107	(0.0730 0.6815)	0.073	0.6815
568	WT005_F11_CLUSTER_24093	gi|34903866|ref|NP_913280.1| unnamed protein product [Oryza sativa (japonica cultivar-group)] >gnl|BL_ORD_ID|1752067 gi|8096617|dbj|BAA96189.1| unknown protein [Oryza sativa (japonica cultivar-group)] >gnl|BL_ORD_ID|1752067	11667.m01093|LOC_Os01g11240|protein hypothetical protein e_value:1.15888e-63	0.4	(0.1007 0.2521)	0.1007	0.2521
569	WT006_A13_CLUSTER_33463	gi|50251471|dbj|BAD28536.1| unknown protein [Oryza sativa (japonica cultivar-group)] e_value:0	11681.m02227|LOC_Os09g24700|protein expressed protein	0.386	(0.2357 0.6101)	0.2357	0.6101
570	WT009_J04_CLUSTER_11692	gi|50844561|gb|AAT84404.1| cell-wall invertase 4 [Oryza sativa (japonica cultivar-group)] >gnl|BL_ORD_ID|1344031 gi|57900173|dbj|BAD88258.1| cell wall invertase [Oryza sativa (japonica cultivar-group)] e_value:0	11667.m07390|LOC_Os01g73580|protein Glycosyl hydrolases family 32 e_value:0	0.171	(0.1357 0.7948)	0.1357	0.7948
571	WT012_K19_CLUSTER_34265	gi|51854363|gb|AAU10743.1| putative finger transcription factor [Oryza sativa (japonica cultivar-group)] e_value:0	11682.m00306|LOC_Os05g03760|protein zinc finger transcription factor-like protein e_value:0	0.219	(0.0908 0.4145)	0.0908	0.4145
572	WT004_G10_CLUSTER_34265	gi|50945195|ref|XP_482125.1| putative Peptidyl-prolyl cis-trans isomerase, chloroplast precursor (40 kDa thylakoid lumen rotamase) [Oryza sativa (japonica cultivar-group)] >gnl|BL_ORD_ID|1605849 gi|40253877|dbj|BAD05811.1| putative Peptidyl-prolyl cis-	11674.m02884|LOC_Os08g29370|protein expressed protein e_value:0	0.128	(0.0685 0.5342)	0.0685	0.5342
573	WT009_D24_CLUSTER_15238	gi|29367413|gb|AAO72579.1| H+-transporting ATP synthase chain 9-like protein [Oryza sativa (japonica cultivar-group)] e_value:1.71387e-87	11669.m01721|LOC_Os03g17070|protein ATP synthase B/B' CF(0), putative e_value:4.29265e-89	0.254	(0.0814 0.3205)	0.0814	0.3205
574	WT004_M16_CLUSTER_15238	gi|29367413|gb|AAO72579.1| H+-transporting ATP synthase chain 9-like protein [Oryza sativa (japonica cultivar-group)] e_value:4.66732e-85	11669.m01721|LOC_Os03g17070|protein ATP synthase B/B' CF(0), putative e_value:1.169e-86	0.333	(0.1187 0.3566)	0.1187	0.3566
575	WT005_N15_CLUSTER_24272	gi|50946945|ref|XP_483000.1| putative glutamate/malate translocator [Oryza sativa (japonica cultivar-group)] >gnl|BL_ORD_ID|1609235 gi|42409033|dbj|BAD10286.1| putative glutamate/malate translocator [Oryza sativa (japonica cultivar-group)] >gnl|BL_O	11674.m03743|LOC_Os08g37600|protein anion transporter, putative e_value:0	0.083	(0.0442 0.5348)	0.0442	0.5348
576	WT004_F17_CLUSTER_33188	gi|7269278|emb|CAB79338.1| hsp 70-like protein [Arabidopsis thaliana] >gnl|BL_ORD_ID|1459767 gi|5051770|emb|CAB45063.1| hsp 70-like protein [Arabidopsis thaliana] >gnl|BL_ORD_ID|1459767 gi|18175969|gb|AAL59960.1| putative hsp 70 protein [Arabidopsis	11682.m02183|LOC_Os05g23740|protein chaperone protein DnaK e_value:0	0.053	(0.0300 0.5686)	0.03	0.5686
577	SET1_N03_CLUSTER_33048	gi|50934599|ref|XP_476827.1| putative ribose-5-phosphate isomerase [Oryza sativa (japonica cultivar-group)] >gi|34393835|dbj|BAC83439.1| putative ribose-5-phosphate isomerase [Oryza sativa (japonica cultivar-group)] e_value:9.59874e-123	11673.m00734|LOC_Os07g08030|protein ribose 5-phosphate isomerase A e_value:2.7938e-124	0.138	(0.0882 0.6400)	0.0882	0.64
578	WT008_H24_CLUSTER_31297	gi|50937505|ref|XP_478280.1| putative flavonol 3-O-glucosyltransferase [Oryza sativa (japonica cultivar-group)] >gnl|BL_ORD_ID|1526828 gi|34394683|dbj|BAC83989.1| putative flavonol 3-O-glucosyltransferase [Oryza sativa (japonica cultivar-group)] e_valu	11673.m03056|LOC_Os07g32020|protein UDP-glucoronosyl and UDP-glucosyl transferase e_value:0	0.404	(0.1504 0.3723)	0.1504	0.3723
579	SET5_O23_CLUSTER_4420	gi|62320596|dbj|BAD95239.1| hypothetical protein [Arabidopsis thaliana] e_value:3.13065e-42	11668.m02804|LOC_Os02g29550|protein AP2 domain, putative e_value:4.2537e-103	0.134	(0.0858 0.6412)	0.0858	0.6412
580	WT002_J17_CLUSTER_17603	gi|50901048|ref|XP_462957.1| Putative hydrolase [Oryza sativa] >gnl|BL_ORD_ID|1564477 gi|14091862|gb|AAK53865.1| Putative hydrolase [Oryza sativa] e_value:2.67395e-121	11669.m03622|LOC_Os03g36750|protein HAD-superfamily hydrolase, subfamily IA, variant 3, putative e_value:6.85812e-144	0.204	(0.1001 0.4917)	0.1001	0.4917
581	WT007_H09_CLUSTER_11915	gi|31432965|gb|AAP54536.1| putative vesicle-associated membrane protein [Oryza sativa (japonica cultivar-group)] >gnl|BL_ORD_ID|2012567 gi|37535894|ref|NP_922249.1| putative vesicle-associated membrane protein [Oryza sativa (japonica cultivar-group)] &	11676.m03261|LOC_Os10g36350|protein putative vesicle-associated membrane protein e_value:1.23199e-111	0.422	(0.1570 0.3720)	0.157	0.372
582	WT004_J18_CLUSTER_34793	gi|481896|pir||S39979 chitinase (EC 3.2.1.14) - rice e_value:6.32879e-159	11682.m03101|LOC_Os05g33130|protein chitinase. e_value:5.59314e-160	0.518	(0.1404 0.2713)	0.1404	0.2713
583	WT003_M01_CLUSTER_31586	gi|50910077|ref|XP_466527.1| translational elongation factor Tu [Oryza sativa (japonica cultivar-group)] >gnl|BL_ORD_ID|1574590 gi|51979258|ref|XP_507491.1| PREDICTED OJ1126_D09.31-2 gene product [Oryza sativa (japonica cultivar-group)] >gnl|BL_ORD_	11668.m03655|LOC_Os02g38210|protein translation elongation factor Tu e_value:0	0.135	(0.0506 0.3757)	0.0506	0.3757
584	WT006_H11_CLUSTER_2850	gi|50908077|ref|XP_465527.1| putative sterol delta-7 reductase [Oryza sativa (japonica cultivar-group)] >gnl|BL_ORD_ID|1571646 gi|47496881|dbj|BAD19845.1| putative sterol delta-7 reductase [Oryza sativa (japonica cultivar-group)] e_value:0	11668.m02496|LOC_Os02g26650|protein Ergosterol biosynthesis ERG4/ERG24 family e_value:0	0.209	(0.0768 0.3670)	0.0768	0.367
585	WT010_F05_CLUSTER_33663	gi|30681070|ref|NP_172401.2| metallopeptidase M24 family protein [Arabidopsis thaliana] e_value:1.16484e-163	11686.m03705|LOC_Os12g37640|protein Aminopeptidase P, N-terminal domain, putative e_value:0	0.202	(0.0974 0.4813)	0.0974	0.4813
586	SET1_F14_CLUSTER_4874	gi|50878443|gb|AAT85217.1| unknown protein [Oryza sativa (japonica cultivar-group)] e_value:9.38217e-85	11682.m03142|LOC_Os05g33510|protein SelR domain, putative e_value:6.89865e-84	0.326	(0.1715 0.5268)	0.1715	0.5268
587	WT003_N23_CLUSTER_14199	gi|2460251|gb|AAB71887.1| ferrochelatase [Hordeum vulgare] >gnl|BL_ORD_ID|271711 gi|7437214|pir||T04373 probable ferrochelatase (EC 4.99.1.1) - barley e_value:0	11681.m01092|LOC_Os09g12560|protein Ferrochelatase, putative e_value:0	0.13	(0.0736 0.5657)	0.0736	0.5657
588	SET6_I04_CLUSTER_32208	gi|50899524|ref|XP_450550.1| apospory-associated protein C-like [Oryza sativa (japonica cultivar-group)] >gi|48716905|dbj|BAD23600.1| apospory-associated protein C-like [Oryza sativa (japonica cultivar-group)] e_value:4.34277e-165	11681.m01424|LOC_Os09g15820|protein apospory-associated protein C-like protein e_value:1.27241e-166	0.1	(0.0551 0.5519)	0.0551	0.5519
589	WT013_A06_CLUSTER_34864	gi|50897038|dbj|BAD34660.1| methionine synthase [Hordeum vulgare subsp. vulgare] e_value:0	11686.m04284|LOC_Os12g42890|protein 5-methyltetrahydropteroyltriglutamate--homocysteine S-methyltransferase e_value:0	0.083	(0.0362 0.4359)	0.0362	0.4359
590	WT002_K07_CLUSTER_32367	gi|31296711|gb|AAP46640.1| GAD1 [Hordeum vulgare] e_value:0	11674.m03607|LOC_Os08g36320|protein glutamate decarboxylase e_value:0	0.221	(0.1161 0.5246)	0.1161	0.5246
591	WT005_F03_CLUSTER_31815	gi|50912809|ref|XP_467812.1| putative H(+)-transporting ATP synthase [Oryza sativa (japonica cultivar-group)] >gnl|BL_ORD_ID|1582927 gi|46390205|dbj|BAD15636.1| putative H(+)-transporting ATP synthase [Oryza sativa (japonica cultivar-group)] e_value:5.	11668.m05026|LOC_Os02g51470|protein ATP synthase F1, delta subunit, putative e_value:1.40176e-95	0.523	(0.1281 0.2449)	0.1281	0.2449
592	WT005_M06_CLUSTER_17958	gi|51536208|dbj|BAD38379.1| unknown protein [Oryza sativa (japonica cultivar-group)] >gnl|BL_ORD_ID|243128 gi|51535252|dbj|BAD38515.1| unknown protein [Oryza sativa (japonica cultivar-group)] e_value:1.74443e-40	11681.m02641|LOC_Os09g28510|protein EF hand, putative e_value:1.58455e-42	0.334	(0.0782 0.2339)	0.0782	0.2339
593	WT003_E14_CLUSTER_5503	gi|50919107|ref|XP_469950.1| expressed protein [Oryza sativa (japonica cultivar-group)] >gnl|BL_ORD_ID|1587276 gi|51964580|ref|XP_507074.1| PREDICTED OSJNBb0060J21.23 gene product [Oryza sativa (japonica cultivar-group)] >gnl|BL_ORD_ID|1587276 gi|49	11669.m05852|LOC_Os03g57840|protein expressed protein e_value:0	0.098	(0.0451 0.4615)	0.0451	0.4615
594	WT003_C06_CLUSTER_31853	gi|50915872|ref|XP_468400.1| ABC1-like [Oryza sativa (japonica cultivar-group)] >gnl|BL_ORD_ID|1583977 gi|47848187|dbj|BAD22014.1| ABC1-like [Oryza sativa (japonica cultivar-group)] e_value:0	11668.m05649|LOC_Os02g57160|protein ABC1 family, putative e_value:0	0.12	(0.0744 0.6222)	0.0744	0.6222
595	SET1_N12_CLUSTER_2892	gi|31415934|gb|AAP50955.1| unknown protein [Oryza sativa (japonica cultivar-group)] >gi|50919007|ref|XP_469900.1| unknown protein [Oryza sativa (japonica cultivar-group)] e_value:2.76448e-43	11669.m05611|LOC_Os03g55720|protein expressed protein e_value:2.45579e-45	0.503	(0.1164 0.2313)	0.1164	0.2313
596	WT006_F13_CLUSTER_23147	gi|34912982|ref|NP_917838.1| putative chloroplast 50S ribosomal protein L31 [Oryza sativa (japonica cultivar-group)] >gnl|BL_ORD_ID|1545185 gi|20161500|dbj|BAB90423.1| chloroplast 50S ribosomal protein L31-like [Oryza sativa (japonica cultivar-	11667.m04291|LOC_Os01g44210|protein ribosomal protein L31, putative e_value:8.3522e-65	0.408	(0.0837 0.2051)	0.0837	0.2051
597	SET2_A20_CLUSTER_20301	gi|50932245|ref|XP_475650.1| 'putative 6-phosphofructo-2-kinase (EC 2.7.1.105) / fructose-2, 6-bisphosphate 2-phosphatase (EC 3.1.3.46)' [Oryza sativa (japonica cultivar-group)] >gi|50080286|gb|AAT69621.1| 'putative 6-phosphofructo-2-kin	11682.m00672|LOC_Os05g07130|protein 6-phosphofructo-2-kinase, putative e_value:0	0.142	(0.0700 0.4938)	0.07	0.4938
598	WT009_D15_CLUSTER_33401	gi|57899848|dbj|BAD87632.1| hydrolase-like [Oryza sativa (japonica cultivar-group)] e_value:0	11667.m06963|LOC_Os01g69060|protein AT4g12830/T20K18_180 e_value:0	0.177	(0.0867 0.4891)	0.0867	0.4891

PDF Complete
Your complimentary use period has ended. Thank you for using PDF Complete.
Click Here to upgrade to Unlimited Pages and Expanded Features

<table>
<tr><th></th><th></th><th></th><th></th><th></th><th></th><th></th><th></th></tr>
<tr><td>599</td><td>SET3_A22_CLUSTER_5707</td><td>gi|34903342|ref|NP_913018.1| unnamed protein product [Oryza sativa (japonica cultivar-group)] >gi|6498428|dbj|BAA87831.1| putative solute carrier family 17 (anion/sugar transporter), member 5 [Oryza sativa (japonica cultivar-group)] >gi|11138056|dbj</td><td>11667.m01736|LOC_Os01g17240|protein hypothetical protein e_value:0</td><td>0.227</td><td>(0.1239 0.5453)</td><td>0.1239</td><td>0.5453</td></tr>
<tr><td>600</td><td>WT005_B13_CLUSTER_12361</td><td>gi|51965122|ref|XP_507345.1| PREDICTED OSJNBa0044E16.27 gene product [Oryza sativa (japonica cultivar-group)] >gnl|BL_ORD_ID|1613321 gi|50948637|ref|XP_483846.1| unknown protein [Oryza sativa (japonica cultivar-group)] >gnl|BL_ORD_ID|1613321 gi|4240</td><td>11674.m04579|LOC_Os08g45190|protein expressed protein e_value:7.95077e-50</td><td>0.304</td><td>(0.1290 0.4238)</td><td>0.129</td><td>0.4238</td></tr>
<tr><td>601</td><td>SET4_O05_CLUSTER_34987</td><td>gi|167097|gb|AAA63164.1| ribulose 1,5-bisphosphate carboxylase activase isoform 2 [Hordeum vulgare subsp. vulgare] >gi|12643756|sp|Q40073|RCAA_HORVU Ribulose bisphosphate carboxylase/oxygenase activase A, chloroplast precursor (RuBisCO activase A) (RA</td><td>11687.m04512|LOC_Os11g47970|protein ATPase, AAA family, putative e_value:0</td><td>0.137</td><td>(0.0695 0.5074)</td><td>0.0695</td><td>0.5074</td></tr>
<tr><td>602</td><td>WT004__C08_CLUSTER_33528</td><td>gi|34908652|ref|NP_915673.1| putative photosystem II subunit (22KDa) precursor [Oryza sativa (japonica cultivar-group)] >gnl|BL_ORD_ID|1557664 gi|20160872|dbj|BAB89811.1| putative photosystem II subunit PsbS [Oryza sativa (japonica cultivar-group)] &gt</td><td>11667.m06522|LOC_Os01g64960|protein Chlorophyll A-B binding protein, putative e_value:4.44411e-114</td><td>0.198</td><td>(0.0858 0.4342)</td><td>0.0858</td><td>0.4342</td></tr>
<tr><td>603</td><td>SET2_D13_CLUSTER_33528</td><td>gi|34908652|ref|NP_915673.1| putative photosystem II subunit (22KDa) precursor [Oryza sativa (japonica cultivar-group)] >gi|20160872|dbj|BAB89811.1| putative photosystem II subunit PsbS [Oryza sativa (japonica cultivar-group)] >gi|15408693|dbj|BAB64</td><td>11667.m06522|LOC_Os01g64960|protein Chlorophyll A-B binding protein, putative e_value:9.06182e-115</td><td>0.196</td><td>(0.0896 0.4564)</td><td>0.0896</td><td>0.4564</td></tr>
<tr><td>604</td><td>WT009_E11_CLUSTER_31827</td><td>gi|100796|pir||S16585 phosphoribulokinase (EC 2.7.1.19) - wheat e_value:0</td><td>11668.m04554|LOC_Os02g47020|protein Phosphoribulokinase / Uridine kinase family, putative e_value:0</td><td>0.08</td><td>(0.0381 0.4766)</td><td>0.0381</td><td>0.4766</td></tr>
<tr><td>605</td><td>WT005_H02_CLUSTER_31827</td><td>gi|5924030|emb|CAB56544.1| phosphoribulokinase [Triticum aestivum] >gnl|BL_ORD_ID|2351653 gi|100839|pir||S15743 phosphoribulokinase (EC 2.7.1.19) - wheat >gnl|BL_ORD_ID|2351653 gi|125580|sp|P26302|KPPR_WHEAT Phosphoribulokinase, chloroplast</td><td>11668.m04554|LOC_Os02g47020|protein Phosphoribulokinase / Uridine kinase family, putative e_value:0</td><td>0.072</td><td>(0.0358 0.4981)</td><td>0.0358</td><td>0.4981</td></tr>
<tr><td>606</td><td>WT009_F02_CLUSTER_33077</td><td>gi|558499|gb|AAC41656.1| carbonic anhydrase >gnl|BL_ORD_ID|2353227 gi|7436817|pir||T04478 probable carbonate dehydratase (EC 4.2.1.1) - barley >gnl|BL_ORD_ID|2353227 gi|729003|sp|P40880|CAHC_HORVU Carbonic anhydrase, chloroplast precursor (Carbonate</td><td>11667.m04353|LOC_Os01g45280|protein Carbonic anhydrase, putative e_value:1.32757e-120</td><td>0.305</td><td>(0.1091 0.3579)</td><td>0.1091</td><td>0.3579</td></tr>
<tr><td>607</td><td>SET1_K16_CLUSTER_33077</td><td>gi|558499|gb|AAC41656.1| carbonic anhydrase >gi|729003|sp|P40880|CAHC_HORVU Carbonic anhydrase, chloroplast precursor (Carbonate dehydratase) >gi|7436817|pir||T04478 probable carbonate dehydratase (EC 4.2.1.1) - barley e_value:1.85891e-143</td><td>11667.m04353|LOC_Os01g45280|protein Carbonic anhydrase, putative e_value:1.32757e-120</td><td>0.305</td><td>(0.1091 0.3579)</td><td>0.1091</td><td>0.3579</td></tr>
<tr><td>608</td><td>WT004_I03_CLUSTER_19304</td><td>gi|50934469|ref|XP_476762.1| putative SHOOT1 protein [Oryza sativa (japonica cultivar-group)] >gnl|BL_ORD_ID|1521410 gi|50509325|dbj|BAD30783.1| putative SHOOT1 protein [Oryza sativa (japonica cultivar-group)] >gnl|BL_ORD_ID|1521410 gi|50509751|dbj|</td><td>11673.m00682|LOC_Os07g07540|protein hypothetical protein e_value:6.17645e-140</td><td>0.277</td><td>(0.1310 0.4734)</td><td>0.131</td><td>0.4734</td></tr>
<tr><td>609</td><td>SET5_N22_CLUSTER_16804</td><td>gi|23297147|gb|AAN13104.1| unknown protein [Arabidopsis thaliana] >gi|18407650|ref|NP_564801.1| elongation factor Tu family protein [Arabidopsis thaliana] >gi|6630460|gb|AAF19548.1| F23N19.11 [Arabidopsis thaliana] >gi|25299484|pir||E96652 protei</td><td>11670.m04421|LOC_Os04g45490|protein translation elongation factor G e_value:0</td><td>0.136</td><td>(0.0699 0.5147)</td><td>0.0699</td><td>0.5147</td></tr>
<tr><td>610</td><td>WT009_J19_CLUSTER_21873</td><td>gi|38568019|emb|CAE05204.3| OSJNBa0070C17.11 [Oryza sativa (japonica cultivar-group)] >gnl|BL_ORD_ID|1538875 gi|50928671|ref|XP_473863.1| OSJNBa0070C17.11 [Oryza sativa (japonica cultivar-group)] e_value:4.2175e-58</td><td>11670.m05115|LOC_Os04g52260|protein proline rich protein - apple tree (fragment) e_value:5.74496e-60</td><td>0.415</td><td>(0.1178 0.2841)</td><td>0.1178</td><td>0.2841</td></tr>
<tr><td>611</td><td>WT006_L15_CLUSTER_21873</td><td>gi|38568019|emb|CAE05204.3| OSJNBa0070C17.11 [Oryza sativa (japonica cultivar-group)] >gnl|BL_ORD_ID|1538875 gi|50928671|ref|XP_473863.1| OSJNBa0070C17.11 [Oryza sativa (japonica cultivar-group)] e_value:4.44318e-58</td><td>11670.m05115|LOC_Os04g52260|protein proline rich protein - apple tree (fragment) e_value:5.74496e-60</td><td>0.415</td><td>(0.1178 0.2841)</td><td>0.1178</td><td>0.2841</td></tr>
<tr><td>612</td><td>WT006_E03_CLUSTER_31745</td><td>gi|30023784|gb|AAP13425.1| At1g16880 [Arabidopsis thaliana] >gnl|BL_ORD_ID|1221671 gi|18394414|ref|NP_564010.1| uridylyltransferase-related [Arabidopsis thaliana] >gnl|BL_ORD_ID|1221671 gi|14423502|gb|AAK62433.1| Unknown protein [Arabidopsis</td><td>11674.m01407|LOC_Os08g14440|protein expressed protein e_value:1.09956e-123</td><td>0.184</td><td>(0.0892 0.4852)</td><td>0.0892</td><td>0.4852</td></tr>
<tr><td>613</td><td>SET5_L20_CLUSTER_21560</td><td>gi|34907850|ref|NP_915272.1| P0031D02.12 [Oryza sativa (japonica cultivar-group)] >gi|21104591|dbj|BAB93184.1| unknown protein [Oryza sativa (japonica cultivar-group)] e_value:2.91134e-67</td><td>11667.m06059|LOC_Os01g60830|protein expressed protein e_value:5.78693e-69</td><td>0.226</td><td>(0.1108 0.4900)</td><td>0.1108</td><td>0.49</td></tr>
<tr><td>614</td><td>SET2_M07_CLUSTER_29471</td><td>gi|6714482|gb|AAF26168.1| unknown protin [Arabidopsis thaliana] e_value:0</td><td>11682.m04154|LOC_Os05g43650|protein hypothetical protein e_value:6.99412e-180</td><td>0.157</td><td>(0.1065 0.6797)</td><td>0.1065</td><td>0.6797</td></tr>
<tr><td>615</td><td>WT003_D04_CLUSTER_23042</td><td>gi|56784400|dbj|BAD82439.1| unknown protein [Oryza sativa (japonica cultivar-group)] e_value:2.57665e-118</td><td>11667.m06901|LOC_Os01g68450|protein expressed protein e_value:8.11533e-120</td><td>0.224</td><td>(0.1353 0.6044)</td><td>0.1353</td><td>0.6044</td></tr>
<tr><td>616</td><td>WT004_B02_CLUSTER_12668</td><td>gi|34902248|ref|NP_912470.1| Putative serine/threonine kinase [Oryza sativa (japonica cultivar-group)] >gnl|BL_ORD_ID|1584050 gi|20330747|gb|AAM19110.1| Putative serine/threonine kinase [Oryza sativa (japonica cultivar-group)] e_value:0</td><td>11669.m00285|LOC_Os03g03510|protein Protein kinase domain, putative e_value:0</td><td>0.129</td><td>(0.0606 0.4680)</td><td>0.0606</td><td>0.468</td></tr>
<tr><td>617</td><td>SET5_G15_CLUSTER_32447</td><td>gi|62176934|emb|CAG25609.1| acid beta-fructofuranosidase precursor [Triticum aestivum] e_value:0</td><td>11668.m00061|LOC_Os02g01590|protein vacuolar acid invertase e_value:0</td><td>0.214</td><td>(0.1818 0.8485)</td><td>0.1818</td><td>0.8485</td></tr>
<tr><td>618</td><td>SET5_I13_CLUSTER_34515</td><td>gi|53792850|dbj|BAD53883.1| unknown protein [Oryza sativa (japonica cultivar-group)] >gi|53791829|dbj|BAD53895.1| unknown protein [Oryza sativa (japonica cultivar-group)] e_value:5.25357e-63</td><td>11680.m04744|LOC_Os06g47970|protein expressed protein e_value:9.88809e-65</td><td>0.228</td><td>(0.1476 0.6467)</td><td>0.1476</td><td>0.6467</td></tr>
<tr><td>619</td><td>WT005_D14_CLUSTER_34716</td><td>gi|20302473|emb|CAD30025.1| ferredoxin-NADP(H) oxidoreductase [Triticum aestivum] e_value:0</td><td>11668.m00036|LOC_Os02g01340|protein Oxidoreductase NAD-binding domain, putative e_value:6.42212e-173</td><td>0.222</td><td>(0.1297 0.5832)</td><td>0.1297</td><td>0.5832</td></tr>
<tr><td>620</td><td>WT003_A08_CLUSTER_34716</td><td>gi|33589718|gb|AAQ22625.1| At5g08050/F13G24_250 [Arabidopsis thaliana] >gnl|BL_ORD_ID|2105543 gi|17978981|gb|AAL47451.1| AT5g08050/F13G24_250 [Arabidopsis thaliana] e_value:1.02127e-36</td><td>11686.m03811|LOC_Os12g38640|protein At5g08050/F13G24_250 e_value:1.28945e-62</td><td>0.477</td><td>(0.1775 0.3722)</td><td>0.1775</td><td>0.3722</td></tr>
<tr><td>621</td><td>WT005_J18_CLUSTER_31955</td><td>gi|50947367|ref|XP_483211.1| putative aminotransferase [Oryza sativa (japonica cultivar-group)] >gnl|BL_ORD_ID|1609461 gi|51964998|ref|XP_507283.1| PREDICTED OJ1345_D02.38 gene product [Oryza sativa (japonica cultivar-group)]</td><td>11674.m03932|LOC_Os08g39300|protein aminotransferase, class V e_value:0</td><td>0.238</td><td>(0.0619 0.2597)</td><td>0.0619</td><td>0.2597</td></tr>
<tr><td>622</td><td>SET6_A05_CLUSTER_34777</td><td>gi|31126793|gb|AAP44712.1| putative glycine hydroxymethyltransferase [Oryza sativa (japonica cultivar-group)] >gi|50918513|ref|XP_469653.1| putative glycine hydroxymethyltransferase [Oryza sativa (japonica cultivar-group)] e_value:0</td><td>11669.m05343|LOC_Os03g52840|protein putative glycine hydroxymethyltransferase e_value:0</td><td>0.082</td><td>(0.0315 0.3862)</td><td>0.0315</td><td>0.3862</td></tr>
<tr><td>623</td><td>SET1_K19_CLUSTER_34507</td><td>gi|34906896|ref|NP_914795.1| putative thioredoxin [Oryza sativa (japonica cultivar-group)] >gi|20161376|dbj|BAB90300.1| putative thioredoxin F [Oryza sativa (japonica cultivar-group)] e_value:5.05972e-73</td><td>11667.m06904|LOC_Os01g68480|protein Thioredoxin, putative e_value:8.32842e-75</td><td>0.322</td><td>(0.1324 0.4109)</td><td>0.1324</td><td>0.4109</td></tr>
<tr><td>624</td><td>WT013_E02_CLUSTER_5294</td><td>gi|2565305|gb|AAB82711.1| glycine decarboxylase P subunit [x Tritordeum sp.] >gnl|BL_ORD_ID|2192753 gi|11360993|pir||T46636 glycine dehydrogenase (decarboxylating) (EC 1.4.4.2) [imported] - Hordeum sp. x Triticum sp e_value:0</td><td>11667.m05023|LOC_Os01g51410|protein glycine dehydrogenase e_value:0</td><td>0.095</td><td>(0.0382 0.4040)</td><td>0.0382</td><td>0.404</td></tr>
<tr><td>625</td><td>WT003_L17_CLUSTER_33255</td><td>gi|17887465|gb|AAL40895.1| phosphoethanolamine methyltransferase [Triticum aestivum] e_value:0</td><td>11682.m04535|LOC_Os05g47540|protein expressed protein</td><td>0.058</td><td>(0.0668 1.1450)</td><td>0.0668</td><td>1.145</td></tr>
<tr><td>626</td><td>WT005_E19_CLUSTER_18997</td><td>gi|50935071|ref|XP_477063.1| putative zeta-carotene desaturase precursor [Oryza sativa (japonica cultivar-group)] >gnl|BL_ORD_ID|2401807 gi|33146603|dbj|BAC79799.1| putative zeta-carotene desaturase precursor [Oryza sativa (japonica cultivar-group)] e_</td><td>11673.m00992|LOC_Os07g10490|protein amine oxidase, flavin-containing e_value:0</td><td>0.095</td><td>(0.0491 0.5140)</td><td>0.0491</td><td>0.514</td></tr>
<tr><td>627</td><td>SET3_E09_CLUSTER_32268</td><td>gi|13160411|emb|CAC32847.1| adenosine diphosphate glucose pyrophosphatase [Hordeum vulgare subsp. vulgare] e_value:3.59776e-114</td><td>11674.m03549|LOC_Os08g35760|protein Cupin, putative e_value:1.58326e-107</td><td>0.22</td><td>(0.0590 0.2680)</td><td>0.059</td><td>0.268</td></tr>
<tr><td>628</td><td>WT003_L06_CLUSTER_33030</td><td>gi|50918105|ref|XP_469449.1| putative 30S ribosomal protein S13 [Oryza sativa (japonica cultivar-group)] >gnl|BL_ORD_ID|1536809 gi|41469448|gb|AAS07249.1| putative 30S ribosomal protein S13 [Oryza sativa (japonica cultivar-group)] e_value:2.59394e-84</td><td>11669.m05001|LOC_Os03g49710|protein putative 30S ribosomal protein S13 e_value:5.07091e-86</td><td>0.086</td><td>(0.0506 0.5918)</td><td>0.0506</td><td>0.5918</td></tr>
<tr><td>629</td><td>WT007_L07_CLUSTER_24082</td><td>gi|21844|emb|CAA40670.1| 33kDa oxygen evolving protein of photosystem II [Triticum aestivum] >gnl|BL_ORD_ID|2329088 gi|100831|pir||S16260 photosystem II oxygen-evolving complex protein 1 - common wheat x Sanduri wheat >gnl|BL_ORD_ID|2329088 gi|13138</td><td>11667.m02976|LOC_Os01g31690|protein Manganese-stabilizing protein / photosystem II polypeptide e_value:3.38836e-170</td><td>0.126</td><td>(0.0477 0.3786)</td><td>0.0477</td><td>0.3786</td></tr>
<tr><td>630</td><td>WT007_F11_CLUSTER_24082</td><td>gi|21844|emb|CAA40670.1| 33kDa oxygen evolving protein of photosystem II [Triticum aestivum] >gnl|BL_ORD_ID|2329088 gi|100831|pir||S16260 photosystem II oxygen-evolving complex protein 1 - common wheat x Sanduri wheat >gnl|BL_ORD_ID|2329088 gi|13138</td><td>11667.m02976|LOC_Os01g31690|protein Manganese-stabilizing protein / photosystem II polypeptide e_value:2.19627e-169</td><td>0.143</td><td>(0.0492 0.3450)</td><td>0.0492</td><td>0.345</td></tr>
<tr><td>631</td><td>WT002_J22_CLUSTER_33946</td><td>gi|58617796|gb|AAW80518.1| aerobic Mg-protoporphyrin IX monomethyl ester cyclase [Hordeum vulgare] e_value:0</td><td>11667.m01728|LOC_Os01g17170|protein magnesium-protoporphyrin IX monomethyl ester aerobic oxidative cyclase</td><td>0.17</td><td>(0.0630 0.3715)</td><td>0.063</td><td>0.3715</td></tr>
<tr><td>632</td><td>WT004_N11_CLUSTER_33117</td><td>gi|19093|emb|CAA34218.1| 10.2 kDa photosystem I polypeptide [Hordeum vulgare] >gnl|BL_ORD_ID|2359352 gi|82412|pir||S05012 photosystem I protein psaH precursor - barley >gnl|BL_ORD_ID|2359352 gi|131196|sp|P20143|PSAH_HORVU Photosystem I reaction cent</td><td>11682.m04656|LOC_Os05g48630|protein Photosystem I reaction centre subunit VI e_value:2.50502e-65</td><td>0.165</td><td>(0.0806 0.4878)</td><td>0.0806</td><td>0.4878</td></tr>
<tr><td>633</td><td>WT004_L20_CLUSTER_34349</td><td>gi|1323748|gb|AAC49287.1| thiol protease >gnl|BL_ORD_ID|404073 gi|7435789|pir||T06276 benzothiadiazole-induced protein (clone WCI-4) - wheat e_value:4.36572e-87</td><td>11670.m00073|LOC_Os04g01710|protein Papain family cysteine protease, putative e_value:7.60627e-79</td><td>0.562</td><td>(0.5042 0.8966)</td><td>0.5042</td><td>0.8966</td></tr>
<tr><td>634</td><td>WT002_K20_CLUSTER_33509</td><td>gi|38347448|emb|CAE02489.2| OSJNBa0076N16.12 [Oryza sativa (japonica cultivar-group)] >gnl|BL_ORD_ID|1536874 gi|38345235|emb|CAD41136.2| OSJNBa0084K20.14 [Oryza sativa (japonica cultivar-group)] >gnl|BL_ORD_ID|1536874 gi|50925621|ref|XP_472987.1| OS</td><td>11670.m03968|LOC_Os04g41340|protein phosphoglycolate/pyridoxal phosphate phosphatase family, putative e_value:0</td><td>0.099</td><td>(0.0585 0.5894)</td><td>0.0585</td><td>0.5894</td></tr>
<tr><td>635</td><td>WT009_E17_CLUSTER_21688</td><td>gi|50943369|ref|XP_481212.1| unknown protein [Oryza sativa (japonica cultivar-group)] >gnl|BL_ORD_ID|1604213 gi|51979659|ref|XP_507576.1| PREDICTED OJ1118_F05.12 gene product [Oryza sativa (japonica cultivar-group)] >gnl|BL_ORD_ID|1604213 gi|5196480</td><td>11674.m01709|LOC_Os08g17390|protein T24D18.8 protein - Arabidopsis thaliana e_value:0</td><td>0.267</td><td>(0.1225 0.4595)</td><td>0.1225</td><td>0.4595</td></tr>
<tr><td>636</td><td>WT010_A01_CLUSTER_30187</td><td>gi|55770353|ref|XP_550167.1| putative PPF-1 protein [Oryza sativa (japonica cultivar-group)] >gnl|BL_ORD_ID|1753405 gi|54290223|dbj|BAD61111.1| putative PPF-1 protein [Oryza sativa (japonica cultivar-group)] e_value:0</td><td>11667.m00509|LOC_Os01g05800|protein Similar to ppf-1 protein - garden pea e_value:0</td><td>0.179</td><td>(0.0871 0.4856)</td><td>0.0871</td><td>0.4856</td></tr>
<tr><td>637</td><td>WT006_E21_CLUSTER_34122</td><td>gi|50932293|ref|XP_475674.1| putative NADH-ubiquinone oxidoreductase [Oryza sativa (japonica cultivar-group)] >gnl|BL_ORD_ID|1598101 gi|48475199|gb|AAT44268.1| putative NADH-ubiquinone oxidoreductase [Oryza sativa (japonica cultivar-group)] e_value:4.2</td><td>11682.m04124|LOC_Os05g43360|protein nadh-ubiquinone oxidoreductase 24 kda subunit, mitochondrial precursor(ec 1.6.5.3) (ec 1.6.99.3) e_value:8.90186e-142</td><td>0.101</td><td>(0.0554 0.5495)</td><td>0.0554</td><td>0.5495</td></tr>
<tr><td>638</td><td>WT011_O18_CLUSTER_33085</td><td>gi|34908888|ref|NP_915791.1| putative 3-hydroxybutyryl-CoA dehydrogenase [Oryza sativa (japonica cultivar-group)] >gnl|BL_ORD_ID|1545406 gi|20160959|dbj|BAB89894.1| putative S(+)-beta-hydroxybutyryl CoA dehydrogenase [Oryza sativa (japonica cultivar-gr</td><td>11667.m05793|LOC_Os01g58380|protein 3-hydroxyacyl-CoA dehydrogenase, NAD binding domain e_value:1.73765e-149</td><td>0.092</td><td>(0.0538 0.5822)</td><td>0.0538</td><td>0.5822</td></tr>
</table>

PDF Complete
Your complimentary use period has ended. Thank you for using PDF Complete.
Click Here to upgrade to Unlimited Pages and Expanded Features

639	WT004_H11_CLUSTER_21749	gi|19091|emb|CAA42727.1| photosystem I polypeptide PSI-G precursor [Hordeum vulgare] >gnl|BL_ORD_ID|2346955 gi|100606|pir||S20937 photosystem I chain V precursor - barley >gnl|BL_ORD_ID|2346955 gi|131192|sp|Q00327|PSAG_HORVU Photosystem I reaction c	11681.m02835|LOC_Os09g30340|protein photosystem i reaction center subunit v, chloroplast precursor (psi-g)(photosystem i 9 kda protein) e_value:9.55675e-59	0.279	(0.0862 0.3093)	0.0862	0.3093
640	WT006_P17_CLUSTER_34049	gi|50906793|ref|XP_464885.1| putative 50S ribosomal protein L21, chloroplast [Oryza sativa (japonica cultivar-group)] >gnl|BL_ORD_ID|1567460 gi|51963970|ref|XP_506768.1| PREDICTED OSJNBa0060K08.25 gene product [Oryza sativa (japonica cultivar-group)] &	11668.m01493|LOC_Os02g15900|protein ribosomal protein L21, putative e_value:1.20185e-85	0.197	(0.0805 0.4080)	0.0805	0.408
641	SET5_E08_CLUSTER_31813	gi|31753114|gb|AAH53854.1| Unknown (protein for IMAGE:5194336) [Homo sapiens] e_value:4.39564e-157	11667.m05117|LOC_Os01g52240|protein Chlorophyll A-B binding protein, putative e_value:1.33084e-139	0.153	(0.0480 0.3128)	0.048	0.3128
642	SET1_J22_CLUSTER_17939	gi|50924111|ref|XP_472416.1| OJ991214_12.3 [Oryza sativa (japonica cultivar-group)] >gi|32479727|emb|CAE01514.1| OJ991214_12.3 [Oryza sativa (japonica cultivar-group)] e_value:8.6238e-70	11670.m03254|LOC_Os04g33830|protein expressed protein e_value:1.39994e-71	0.478	(0.0832 0.1740)	0.0832	0.174
643	WT002_M13_CLUSTER_12889	gi|3550483|emb|CAA11893.1| cp31BHv [Hordeum vulgare subsp. vulgare] >gnl|BL_ORD_ID|1317115 gi|7446359|pir||T05727 nucleic acid-binding protein - barley e_value:7.84881e-151	11674.m04474|LOC_Os08g44290|protein RNA recognition motif. (a.k.a. RRM, RBD, or RNP domain), putative e_value:4.31836e-	0.318	(0.1295 0.4076)	0.1295	0.4076
644	WT004_J07_CLUSTER_31663	gi|50937699|ref|XP_478377.1| putative ATP synthase gamma chain 1, chloroplast (H(+)-transporting two-sector ATPase/F(1)-ATPase/ATPC1) [Oryza sativa (japonica cultivar-group)] >gnl|BL_ORD_ID|1527736 gi|50509502|dbj|BAD31183.1| putative ATP synthase gamm	11673.m03146|LOC_Os07g32880|protein ATP synthase F1, gamma subunit e_value:4.09766e-172	0.26	(0.0832 0.3193)	0.0832	0.3193
645	SET2_N14_CLUSTER_12853	gi|50905817|ref|XP_464397.1| ribosomal protein S21-like protein [Oryza sativa (japonica cultivar-group)] >gi|46390953|dbj|BAD16466.1| ribosomal protein S21-like protein [Oryza sativa (japonica cultivar-group)] >gi|46389907|dbj|BAD15528.1| ribosomal	11668.m00882|LOC_Os02g09590|protein ribosomal protein S21, putative e_value:1.06891e-52	0.439	(0.1699 0.3870)	0.1699	0.387
646	WT011_J06_CLUSTER_28442	gi|50948907|ref|XP_493811.1| EST C74302(E30840) corresponds to a region of the predicted gene.~similar to glyceraldehyde-3-phosphate dehydrogenase. (M64118) [Oryza sativa (japonica cultivar-group)] >gnl|BL_ORD_ID|1732292 gi|23306126|gb|AAN17393.1| Puta	11669.m00306|LOC_Os03g03720|protein glyceraldehyde-3-phosphate dehydrogenase, type I, putative e_value:0	0.15	(0.0550 0.3656)	0.055	0.3656
647	WT005_H18_CLUSTER_33127	gi|50905979|ref|XP_464478.1| putative chlorophyll a/b-binding protein type III precursor [Oryza sativa (japonica cultivar-group)] >gnl|BL_ORD_ID|1566734 gi|51979178|ref|XP_507457.1| PREDICTED OJ1524_D08.28-2 gene product [Oryza sativa (japonica cultiva	11668.m00966|LOC_Os02g10390|protein chlorophyll a/b binding protein. e_value:6.50073e-142	0.155	(0.0756 0.4876)	0.0756	0.4876
648	WT002_N19_CLUSTER_33127	gi|31433274|gb|AAP54812.1| hypothetical protein [Oryza sativa (japonica cultivar-group)] >gnl|BL_ORD_ID|2013732 gi|37536446|ref|NP_922525.1| hypothetical protein [Oryza sativa (japonica cultivar-group)] >gnl|BL_ORD_ID|2013732 gi|18057096|gb|AAL58119	11676.m03564|LOC_Os10g39150|protein hypothetical protein e_value:9.51181e-74	0.197	(0.1164 0.5919)	0.1164	0.5919
649	WT007_I13_CLUSTER_32083	gi|50928835|ref|XP_473945.1| OSJNBa0053K19.11 [Oryza sativa (japonica cultivar-group)] >gnl|BL_ORD_ID|1592042 gi|38344172|emb|CAE03503.2| OSJNBa0053K19.11 [Oryza sativa (japonica cultivar-group)] e_value:0	11670.m05219|LOC_Os04g53230|protein glycine cleavage system T protein e_value:0	0.063	(0.0326 0.5162)	0.0326	0.5162
650	WT002_F01_CLUSTER_27301	gi|1076728|pir||S51813 photosystem-I PSI-F chain precursor - barley >gnl|BL_ORD_ID|2312152 gi|469560|gb|AAA68147.1| photosystem-I PSI-F subunit precursor >gnl|BL_ORD_ID|2312152 gi|548604|sp|P13192|PSAF_HORVU Photosystem I reaction center subunit III	11669.m05718|LOC_Os03g56670|protein photosystem-1 F subunit precursor e_value:2.83074e-108	0.209	(0.0707 0.3377)	0.0707	0.3377
651	SET1_D16_CLUSTER_15970	gi|50918761|ref|XP_469777.1| putative ribosomal protein L6 [Oryza sativa (japonica cultivar-group)] >gi|28144880|gb|AAO32311.1| putative chloroplast 50S ribosomal protein L6 [Oryza sativa (japonica cultivar-group)] >gi|40538993|gb|AAR87250.1| putat	11669.m02429|LOC_Os03g24020|protein ribosomal protein L6, putative e_value:3.2068e-117	0.054	(0.0392 0.7222)	0.0392	0.7222
652	WT003_A20_CLUSTER_29947	gi|666054|emb|CAA59049.1| LHCI-680, photosystem I antenna protein [Hordeum vulgare subsp. vulgare] >gnl|BL_ORD_ID|995234 gi|1076724|pir||S52341 LHCI-680, photosystem I antenna protein - barley e_value:1.88501e-145	11673.m03791|LOC_Os07g38960|protein Chlorophyll A-B binding protein, putative e_value:4.22346e-135	0.292	(0.0582 0.1993)	0.0582	0.1993
653	WT002_N16_CLUSTER_26634	gi|21555497|gb|AAM63872.1| unknown [Arabidopsis thaliana] >gnl|BL_ORD_ID|1217149 gi|16323364|gb|AAL15176.1| unknown protein [Arabidopsis thaliana] >gnl|BL_ORD_ID|1217149 gi|14334924|gb|AAK59640.1| unknown protein [Arabidopsis thaliana] >gnl|BL_OR	11669.m01408|LOC_Os03g14280|protein Clp amino terminal domain, putative e_value:5.62836e-101	0.172	(0.0946 0.5500)	0.0946	0.55
654	WT004_P20_CLUSTER_33570	gi|32401369|gb|AAP80856.1| probable light-induced protein [Triticum aestivum] e_value:1.04294e-58	11673.m02504|LOC_Os07g26700|protein Uncharacterised protein family (UPF0041) e_value:4.56382e-55	0.07	(0.0449 0.6374)	0.0449	0.6374
655	WT003_O24_CLUSTER_31640	gi|34901804|ref|NP_912248.1| GTP-binding protein Rab6 [Oryza sativa (japonica cultivar-group)] >gnl|BL_ORD_ID|1527310 gi|24059912|dbj|BAC21376.1| GTP-binding protein Rab6 [Oryza sativa (japonica cultivar-group)] e_value:9.60967e-111	11673.m02990|LOC_Os07g31370|protein Ras family, putative e_value:1.57571e-112	0.004	(0.0021 0.4760)	0.0021	0.476
656	SET5_I21_CLUSTER_20143	gi|51090748|dbj|BAD35228.1| putative chaperonin 21 precursor [Oryza sativa (japonica cultivar-group)] e_value:3.41855e-111	11680.m00930|LOC_Os06g09670|protein chaperonin, 10 kDa, putative e_value:7.24741e-113	0.122	(0.0713 0.5861)	0.0713	0.5861
657	WT012_E06_CLUSTER_33438	gi|19879917|gb|AAM00197.1| acid phosphatase [Oryza sativa] >gnl|BL_ORD_ID|923281 gi|51535510|dbj|BAD37429.1| acid phosphatase [Oryza sativa (japonica cultivar-group)] >gnl|BL_ORD_ID|923281 gi|51535476|dbj|BAD37373.1| acid phosphatase [Oryza sativa (	11680.m04286|LOC_Os06g43640|protein acid phosphatase e_value:0	0.116	(0.0824 0.7091)	0.0824	0.7091
658	WT004_E14_CLUSTER_33459	gi|50946099|ref|XP_482577.1| zinc finger protein-like [Oryza sativa (japonica cultivar-group)] >gnl|BL_ORD_ID|1607588 gi|42408883|dbj|BAD10141.1| zinc finger protein-like [Oryza sativa (japonica cultivar-group)] e_value:1.13778e-119	11681.m02222|LOC_Os09g24650|protein Zinc finger, C3HC4 type (RING finger), putative e_value:3.28772e-144	0.105	(0.0697 0.6635)	0.0697	0.6635
659	WT003_G12_CLUSTER_34842	gi|18076790|emb|CAC85913.1| phosphoglucomutase [Triticum aestivum] e_value:0	11669.m05086|LOC_Os03g50480|protein phosphoglucomutase e_value:0	0.128	(0.0524 0.4094)	0.0524	0.4094
660	WT004_G04_CLUSTER_31633	gi|31430181|gb|AAP52133.1| putative proline-rich protein [Oryza sativa (japonica cultivar-group)] >gnl|BL_ORD_ID|2008506 gi|37531088|ref|NP_919846.1| putative proline-rich protein [Oryza sativa (japonica cultivar-group)] >gnl|BL_ORD_ID|2008506 gi|14	11676.m00511|LOC_Os10g05970|protein expressed protein e_value:8.6306e-66	0.441	(0.2779 0.6304)	0.2779	0.6304
661	WT003_L07_CLUSTER_23806	gi|50910968|ref|XP_466917.1| unknown protein [Oryza sativa (japonica cultivar-group)] >gnl|BL_ORD_ID|1579280 gi|49388184|dbj|BAD25310.1| unknown protein [Oryza sativa (japonica cultivar-group)] e_value:4.00026e-136	11668.m04042|LOC_Os02g42290|protein Clp protease, putative e_value:8.76521e-138	0.223	(0.0884 0.3972)	0.0884	0.3972
662	SET3_O13_CLUSTER_33094	gi|31433720|gb|AAP55199.1| unknown protein [Oryza sativa (japonica cultivar-group)] >gi|37537220|ref|NP_922913.1| unknown protein [Oryza sativa (japonica cultivar-group)] >gi|12039366|gb|AAG46152.1| unknown protein [Oryza sativa] e_value:6.79308e-15	11676.m04008|LOC_Os10g43060|protein expressed protein e_value:1.83324e-156	0.225	(0.1280 0.5696)	0.128	0.5696
663	SET2_F18_CLUSTER_11962	gi|50924582|ref|XP_472650.1| OSJNBa0027P08.15 [Oryza sativa (japonica cultivar-group)] >gi|38344547|emb|CAD40963.2| OSJNBa0027P08.15 [Oryza sativa (japonica cultivar-group)] e_value:6.89165e-62	11670.m03517|LOC_Os04g36810|protein Sm protein, putative e_value:3.21413e-66	0.075	(0.0210 0.2796)	0.021	0.2796
664	WT013_O14_CLUSTER_23034	gi|50428718|gb|AAT77069.1| putative DnaJ protein [Oryza sativa (japonica cultivar-group)] e_value:1.50927e-108	11669.m05233|LOC_Os03g51830|protein putative DnaJ protein e_value:2.4234e-110	0.238	(0.1129 0.4745)	0.1129	0.4745
665	WT003_L22_CLUSTER_23439	gi|38346957|emb|CAE03896.2| OSJNBb0026I12.4 [Oryza sativa (japonica cultivar-group)] >gnl|BL_ORD_ID|1535364 gi|50921893|ref|XP_471307.1| OSJNBb0026I12.4 [Oryza sativa (japonica cultivar-group)] e_value:0	11670.m01590|LOC_Os04g17650|protein Sucrose synthase, putative e_value:0	0.084	(0.0633 0.7571)	0.0633	0.7571
666	WT005_J20_CLUSTER_33804	gi|56785381|dbj|BAD82339.1| putative DEAD box RNA helicase [Oryza sativa (japonica cultivar-group)] >gnl|BL_ORD_ID|972849 gi|56784388|dbj|BAD82427.1| putative DEAD box RNA helicase [Oryza sativa (japonica cultivar-group)] e_value:0	11667.m06885|LOC_Os01g68320|protein DEAD/DEAH box helicase, putative e_value:0	0.167	(0.0970 0.5804)	0.097	0.5804
667	SET4_H17_CLUSTER_31525	gi|20197921|gb|AAD24640.2| putative pyruvate kinase [Arabidopsis thaliana] >gi|17978970|gb|AAL47446.1| At2g36580/F1O11.21 [Arabidopsis thaliana] >gi|18404169|ref|NP_565850.1| pyruvate kinase, putative [Arabidopsis thaliana] e_value:0	11687.m01046|LOC_Os11g10980|protein pyruvate kinase e_value:0	0.172	(0.0768 0.4450)	0.0768	0.445
668	WT003_H22_CLUSTER_33098	gi|48093487|gb|AAT40121.1| GAMYB-binding protein [Hordeum vulgare subsp. vulgare] e_value:0	11680.m02989|LOC_Os06g31100|protein GAMYB-binding protein e_value:0	0.09	(0.0501 0.5554)	0.0501	0.5554
669	WT002_L18_CLUSTER_277	gi|50931181|ref|XP_475118.1| putative mitochondrial carrier protein [Oryza sativa (japonica cultivar-group)] >gnl|BL_ORD_ID|1596354 gi|54287654|gb|AAV31398.1| putative mitochondrial carrier protein [Oryza sativa (japonica cultivar-group)] >gnl|BL_OR	11682.m02760|LOC_Os05g29860|protein Mitochondrial carrier protein, putative e_value:1.09781e-137	0.098	(0.0600 0.6110)	0.06	0.611
670	SET1_E05_CLUSTER_15464	gi|50905733|ref|XP_464355.1| aspartic acid-rich protein aspolin1-like protein [Oryza sativa (japonica cultivar-group)] >gi|49387532|dbj|BAD25065.1| aspartic acid-rich protein aspolin1-like protein [Oryza sativa (japonica cultivar-group)] e_value:2.6049	11681.m03439|LOC_Os09g38400|protein expressed protein e_value:8.83687e-48	0.2	(0.1726 0.8644)	0.1726	0.8644
671	SET6_O19_CLUSTER_1178	gi|50918743|ref|XP_469768.1| putative strictosidine synthase [Oryza sativa (japonica cultivar-group)] >gi|40538997|gb|AAR87254.1| putative strictosidine synthase [Oryza sativa (japonica cultivar-group)] e_value:0	11669.m05469|LOC_Os03g53950|protein putative strictosidine synthase e_value:0	0.217	(0.0835 0.3846)	0.0835	0.3846
672	WT008_O16_CLUSTER_11676	gi|50897226|gb|AAT85752.1| At5g55510 [Arabidopsis thaliana] >gnl|BL_ORD_ID|1001680 gi|46931218|gb|AAT06413.1| At5g55510 [Arabidopsis thaliana] e_value:1.33074e-44	11686.m03224|LOC_Os12g33020|protein AT4g26670/F10M23_10 e_value:1.0552e-96	0.22	(0.1237 0.5625)	0.1237	0.5625
673	WT005_P20_CLUSTER_32269	gi|34895462|ref|NP_909074.1| unknown protein [Oryza sativa (japonica cultivar-group)] >gnl|BL_ORD_ID|1751595 gi|14090380|dbj|BAB55538.1| unknown protein [Oryza sativa (japonica cultivar-group)] >gnl|BL_ORD_ID|1751595 gi|12328526|dbj|BAB21184.1| hypo	11667.m00261|LOC_Os01g03490|protein Heavy-metal-associated domain, putative e_value:1.4753e-163	0.176	(0.1315 0.7475)	0.1315	0.7475
674	WT002_H15_CLUSTER_30203	gi|50947313|ref|XP_483184.1| putative peroxisomal targeting signal 1 receptor [Oryza sativa (japonica cultivar-group)] >gnl|BL_ORD_ID|1609433 gi|42407351|dbj|BAD08812.1| putative peroxisomal targeting signal 1 receptor [Oryza sativa (japonica cultivar-	11674.m03908|LOC_Os08g39080|protein TPR Domain, putative e_value:0	0.109	(0.0668 0.6115)	0.0668	0.6115
675	SET5_O01_CLUSTER_3788	gi|50945265|ref|XP_482160.1| unknown protein [Oryza sativa (japonica cultivar-group)] >gi|40253481|dbj|BAD05431.1| unknown protein [Oryza sativa (japonica cultivar-group)] e_value:1.52658e-144	11674.m02923|LOC_Os08g29730|protein expressed protein e_value:0	0.206	(0.1308 0.6354)	0.1308	0.6354
676	SET5_E12_CLUSTER_32142	gi|15293043|gb|AAK93632.1| putative growth regulator protein [Arabidopsis thaliana] e_value:2.73712e-176	11686.m00705|LOC_Os12g07540|protein growth regulator, putative e_value:0	0.184	(0.1583 0.8613)	0.1583	0.8613
677	WT002_I02_CLUSTER_33076	gi|50921747|ref|XP_471234.1| OSJNBa0095E20.8 [Oryza sativa (japonica cultivar-group)] >gnl|BL_ORD_ID|1590169 gi|38347510|emb|CAE02420.2| OSJNBa0095E20.8 [Oryza sativa (japonica cultivar-group)] e_value:0	11670.m00415|LOC_Os04g05050|protein Pectate lyase, putative e_value:0	0.248	(0.1241 0.5013)	0.1241	0.5013
678	SET6_A21_CLUSTER_21416	gi|51535831|dbj|BAD37915.1| putative DegP2 protease [Oryza sativa (japonica cultivar-group)] >gi|51535111|dbj|BAD37774.1| putative DegP2 protease [Oryza sativa (japonica cultivar-group)] e_value:0	11680.m01254|LOC_Os06g12780|protein Trypsin, putative e_value:0	0.157	(0.0874 0.5582)	0.0874	0.5582
679	WT013_I21_CLUSTER_34421	gi|50898642|ref|XP_450109.1| nodulation receptor kinase-like protein [Oryza sativa (japonica cultivar-group)] >gnl|BL_ORD_ID|1563253 gi|47496991|dbj|BAD20101.1| nodulation receptor kinase-like protein [Oryza sativa (japonica cultivar-group)] e_value:0	11681.m00270|LOC_Os09g03620|protein Protein kinase domain, putative e_value:0	0.139	(0.0897 0.6463)	0.0897	0.6463
680	WT002_M06_CLUSTER_14913	gi|62510489|sp|Q6X4A2|CIPK1_ORYSA CIPK-like protein 1 (OsCK1) >gnl|BL_ORD_ID|563049 gi|32442211|gb|AAP82174.1| CIPK-like protein [Oryza sativa (japonica cultivar-group)] e_value:0	11669.m02079|LOC_Os03g20380|protein Protein kinase domain, putative e_value:0	0.071	(0.0376 0.5314)	0.0376	0.5314

PDF Complete
Your complimentary use period has ended. Thank you for using PDF Complete.
Click Here to upgrade to Unlimited Pages and Expanded Features

<table>
<tr><td>681</td><td>WT006_J16_CLUSTER_34861</td><td>gi|50906281|ref|XP_464629.1| putative pectate lyase precursor [Oryza sativa (japonica cultivar-group)] >gnl|BL_ORD_ID|1567029
gi|49387941|dbj|BAD25039.1| putative pectate lyase precursor [Oryza sativa (japonica cultivar-group)] e_value:1.9712e-165</td><td>11668.m01124|LOC_Os02g12300|protein pectate lyase precursor (ec 4.2.2.2) e_value:4.97836e-167</td><td>0.574</td><td>(0.2702 0.4708)</td><td>0.2702</td><td>0.4708</td></tr>
<tr><td>682</td><td>SET1_L02_CLUSTER_34861</td><td>gi|62734221|gb|AAX96330.1| GUN4, putative [Oryza sativa (japonica cultivar-group)] e_value:1.11649e-104</td><td>11687.m01523|LOC_Os11g16550|protein GUN4, putative e_value:2.53325e-106</td><td>0.262</td><td>(0.1639 0.6249)</td><td>0.1639</td><td>0.6249</td></tr>
<tr><td>683</td><td>WT006_B08_CLUSTER_21954</td><td>gi|51091406|dbj|BAD36149.1| unknown protein [Oryza sativa (japonica cultivar-group)] e_value:0</td><td>11681.m02460|LOC_Os09g26870|protein BRCA1 C Terminus (BRCT) domain, putative e_value:2.74994e-174</td><td>0.172</td><td>(0.1019 0.5919)</td><td>0.1019</td><td>0.5919</td></tr>
<tr><td>684</td><td>WT005_M23_CLUSTER_31961</td><td>gi|62732987|gb|AAX95106.1| Tub family, putative [Oryza sativa (japonica cultivar-group)] e_value:0</td><td>11687.m00574|LOC_Os11g06420|protein Tub family, putative e_value:0</td><td>0.146</td><td>(0.0970 0.6661)</td><td>0.097</td><td>0.6661</td></tr>
<tr><td>685</td><td>WT008_I02_CLUSTER_13039</td><td>gi|53792860|dbj|BAD53978.1| unknown protein [Oryza sativa (japonica cultivar-group)] e_value:4.61145e-79</td><td>11680.m01915|LOC_Os06g19690|protein expressed protein e_value:1.91481e-91</td><td>0.359</td><td>(0.1522 0.4242)</td><td>0.1522</td><td>0.4242</td></tr>
<tr><td>686</td><td>SET4_I22_CLUSTER_33434</td><td>gi|50905605|ref|XP_464291.1| putative glyceraldehyde-3-phosphate dehydrogenase [Oryza sativa (japonica cultivar-group)]
>gi|49388082|dbj|BAD25194.1| putative glyceraldehyde-3-phosphate dehydrogenase [Oryza sativa (japonica cultivar-group)] >gi|49387</td><td>11668.m00709|LOC_Os02g07490|protein glyceraldehyde-3-phosphate dehydrogenase, type I, putative e_value:0</td><td>0.131</td><td>(0.0539 0.4099)</td><td>0.0539</td><td>0.4099</td></tr>
<tr><td>687</td><td>WT009_K03_CLUSTER_15135</td><td>gi|51038058|gb|AAT93862.1| unknown protein [Oryza sativa (japonica cultivar-group)] e_value:1.51927e-150</td><td>11682.m04039|LOC_Os05g42040|protein expressed protein e_value:5.43419e-152</td><td>0.423</td><td>(0.3196 0.7552)</td><td>0.3196</td><td>0.7552</td></tr>
<tr><td>688</td><td>WT010_A11_CLUSTER_30848</td><td>gi|50918763|ref|XP_469778.1| expressed protein [Oryza sativa (japonica cultivar-group)] >gnl|BL_ORD_ID|1586196
gi|40538989|gb|AAR87246.1| expressed protein [Oryza sativa (japonica cultivar-group)] e_value:3.448e-96</td><td>11669.m05479|LOC_Os03g54050|protein hypothetical protein e_value:1.04044e-97</td><td>0.391</td><td>(0.1640 0.4196)</td><td>0.164</td><td>0.4196</td></tr>
<tr><td>689</td><td>WT007_K07_CLUSTER_30848</td><td>gi|31712047|gb|AAP68353.1| unknown protein [Oryza sativa (japonica cultivar-group)] >gnl|BL_ORD_ID|2050096
gi|21070928|gb|AAM34403.1| unknown protein [Oryza sativa (japonica cultivar-group)] e_value:9.19855e-97</td><td>11669.m05479|LOC_Os03g54050|protein hypothetical protein e_value:2.06488e-98</td><td>0.348</td><td>(0.1573 0.4515)</td><td>0.1573</td><td>0.4515</td></tr>
<tr><td>690</td><td>SET1_F20_CLUSTER_15454</td><td>gi|52075701|dbj|BAD44921.1| unknown protein [Oryza sativa (japonica cultivar-group)] >gi|52075691|dbj|BAD44911.1| unknown protein [Oryza sativa (japonica cultivar-group)] e_value:1.05218e-22</td><td>11669.m01950|LOC_Os03g19220|protein expressed protein e_value:1.15003e-32</td><td>0.19</td><td>(0.1506 0.7907)</td><td>0.1506</td><td>0.7907</td></tr>
<tr><td>691</td><td>WT012_G17_CLUSTER_19711</td><td>gi|32492425|emb|CAE05702.1| OSJNBa0083D01.24 [Oryza sativa (japonica cultivar-group)] >gnl|BL_ORD_ID|1536938
gi|32489059|emb|CAE03989.1| OSJNBb0089B03.3 [Oryza sativa (japonica cultivar-group)] >gnl|BL_ORD_ID|1536938
gi|50923725|ref|XP_472223.1| OSJ</td><td>11670.m02948|LOC_Os04g31030|protein Nitrate-induced NOI protein, putative e_value:1.89522e-78</td><td>0.325</td><td>(0.2450 0.7541)</td><td>0.245</td><td>0.7541</td></tr>
<tr><td>692</td><td>WT003_A04_CLUSTER_30468</td><td>gi|50921233|ref|XP_470977.1| OSJNBb0004G23.6 [Oryza sativa (japonica cultivar-group)] >gnl|BL_ORD_ID|1589071
gi|38346874|emb|CAE04608.2| OSJNBb0004G23.6 [Oryza sativa (japonica cultivar-group)] >gnl|BL_ORD_ID|1589071
gi|38345302|emb|CAE02754.2| OSJN</td><td>11670.m00056|LOC_Os04g01540|protein expressed protein e_value:3.74801e-109</td><td>0.17</td><td>(0.1019 0.6008)</td><td>0.1019</td><td>0.6008</td></tr>
<tr><td>693</td><td>SET4_E23_CLUSTER_14755</td><td>gi|20453277|gb|AAM19877.1| At1g50450/F11F12_20 [Arabidopsis thaliana] >gi|18403077|ref|NP_564570.1| expressed protein [Arabidopsis thaliana] >gi|14517416|gb|AAK62598.1| At1g50450/F11F12_20 [Arabidopsis thaliana] e_value:8.94848e-169</td><td>11686.m00351|LOC_Os12g04260|protein expressed protein e_value:0</td><td>0.183</td><td>(0.0924 0.5043)</td><td>0.0924</td><td>0.5043</td></tr>
<tr><td>694</td><td>WT004_O09_CLUSTER_32558</td><td>gi|403033|emb|CAA52980.1| ferredoxin [Triticum aestivum] >gnl|BL_ORD_ID|2378121 gi|462081|sp|P00228|FER_WHEAT Ferredoxin, chloroplast precursor e_value:2.22966e-74</td><td>11674.m00042|LOC_Os08g01380|protein ferredoxin i, chloroplast precursor (anti-disease protein 1) e_value:1.28953e-53</td><td>0.397</td><td>(0.1646 0.4147)</td><td>0.1646</td><td>0.4147</td></tr>
<tr><td>695</td><td>WT004_L08_CLUSTER_32920</td><td>gi|50938489|ref|XP_478772.1| putative Ribosome recycling factor, chloroplast precursor [Oryza sativa (japonica cultivar-group)]
>gnl|BL_ORD_ID|1529502 gi|34394208|dbj|BAC84660.1| putative Ribosome recycling factor, chloroplast precursor [Oryza sativa (</td><td>11673.m03721|LOC_Os07g38300|protein ribosome recycling factor, putative e_value:1.29502e-118</td><td>0.206</td><td>(0.1032 0.5016)</td><td>0.1032</td><td>0.5016</td></tr>
<tr><td>696</td><td>WT005_B24_CLUSTER_14806</td><td>gi|53793168|dbj|BAD54375.1| unknown protein [Oryza sativa (japonica cultivar-group)] e_value:5.53758e-64</td><td>11680.m01526|LOC_Os06g15400|protein expressed protein e_value:1.03746e-65</td><td>0.143</td><td>(0.0567 0.3967)</td><td>0.0567</td><td>0.3967</td></tr>
<tr><td>697</td><td>WT004_B04_CLUSTER_32166</td><td>gi|50933293|ref|XP_476174.1| putative transmembrane protein [Oryza sativa (japonica cultivar-group)] >gnl|BL_ORD_ID|1600909
gi|48843759|gb|AAT47018.1| putative transmembrane protein [Oryza sativa (japonica cultivar-group)] e_value:2.2041e-156</td><td>11682.m01186|LOC_Os05g12490|protein transmembrane protein, putative e_value:5.09785e-158</td><td>0.088</td><td>(0.0899 1.0243)</td><td>0.0899</td><td>1.0243</td></tr>
<tr><td>698</td><td>SET5_J17_CLUSTER_34644</td><td>gi|33323017|gb|AAQ07248.1| galactinol synthase 1 [Zea mays] e_value:2.82543e-174</td><td>11669.m02050|LOC_Os03g20120|protein galactinol synthase e_value:0</td><td>0.132</td><td>(0.0449 0.3406)</td><td>0.0449</td><td>0.3406</td></tr>
<tr><td>699</td><td>WT007_K09_CLUSTER_34033</td><td>gi|52077226|dbj|BAD46270.1| peudo-response regulator-like [Oryza sativa (japonica cultivar-group)] >gnl|BL_ORD_ID|381791
gi|52075943|dbj|BAD46023.1| peudo-response regulator-like [Oryza sativa (japonica cultivar-group)] >gnl|BL_ORD_ID|381791 gi|5157</td><td>11681.m03215|LOC_Os09g36220|protein pseudo-response regulator 3 e_value:0</td><td>0.272</td><td>(0.1843 0.6772)</td><td>0.1843</td><td>0.6772</td></tr>
<tr><td>700</td><td>SET2_D10_CLUSTER_9019</td><td>gi|50926566|ref|XP_473230.1| OSJNBa0038O10.2 [Oryza sativa (japonica cultivar-group)] >gi|32487646|emb|CAE05636.1| OSJNBa0038O10.2 [Oryza sativa (japonica cultivar-group)] e_value:2.14154e-97</td><td>11670.m04294|LOC_Os04g44340|protein expressed protein e_value:6.16313e-99</td><td>0.275</td><td>(0.1646 0.5976)</td><td>0.1646</td><td>0.5976</td></tr>
<tr><td>703</td><td>WT003_N13_CLUSTER_31749</td><td>gi|7269804|emb|CAB79664.1| putative protein [Arabidopsis thaliana] >gnl|BL_ORD_ID|1461312 gi|4972052|emb|CAB43920.1| putative protein [Arabidopsis thaliana] >gnl|BL_ORD_ID|1461312 gi|18417320|ref|NP_567820.1| elongation factor Ts family protein [Ara</td><td>11686.m03492|LOC_Os12g35630|protein Elongation factor TS, putative e_value:0</td><td>0.202</td><td>(0.1096 0.5421)</td><td>0.1096</td><td>0.5421</td></tr>
<tr><td>704</td><td>WT002_L20_CLUSTER_34784</td><td>gi|50252031|dbj|BAD27963.1| hydroxyproline-rich glycoprotein-like [Oryza sativa (japonica cultivar-group)] e_value:1.90136e-179</td><td>11668.m01478|LOC_Os02g15750|protein AT4g01050/F2N1_31 e_value:1.28011e-180</td><td>0.277</td><td>(0.1793 0.6482)</td><td>0.1793</td><td>0.6482</td></tr>
<tr><td>705</td><td>WT008_M23_CLUSTER_29116</td><td>gi|38347589|emb|CAE04880.2| OSJNBa0042I15.2 [Oryza sativa (japonica cultivar-group)] e_value:3.34185e-176</td><td>11670.m03191|LOC_Os04g33280|protein At5g47860/MCA23_20 e_value:0</td><td>0.165</td><td>(0.0907 0.5491)</td><td>0.0907</td><td>0.5491</td></tr>
<tr><td>706</td><td>SET3_A19_CLUSTER_32719</td><td>gi|50908337|ref|XP_465657.1| putative Poly(A)-binding protein binding protein [Oryza sativa (japonica cultivar-group)]
>gi|47848604|dbj|BAD22453.1| putative Poly(A)-binding protein binding protein [Oryza sativa (japonica cultivar-group)] >gi|4784815</td><td>11668.m02641|LOC_Os02g27950|protein Ataxin-2 N-terminal region, putative e_value:0</td><td>0.275</td><td>(0.1545 0.5627)</td><td>0.1545</td><td>0.5627</td></tr>
<tr><td>707</td><td>SET5_O20_CLUSTER_2017</td><td>gi|19033|emb|CAA33727.1| unnamed protein product [Hordeum vulgare subsp. vulgare] >gi|100630|pir||S07474 early light-induced protein, low molecular weight, precursor (clone HV90) - barley chloroplast >gi|119286|sp|P14897|ELI9_HORVU Low molecular mas</td><td>11673.m00747|LOC_Os07g08160|protein Chlorophyll A-B binding protein, putative e_value:5.04451e-48</td><td>0.403</td><td>(0.2488 0.6169)</td><td>0.2488</td><td>0.6169</td></tr>
<tr><td>708</td><td>SET3_C20_CLUSTER_30282</td><td>gi|50917431|ref|XP_469112.1| hypothetical protein [Oryza sativa (japonica cultivar-group)] >gi|27764685|gb|AAO23110.1| hypothetical protein [Oryza sativa (japonica cultivar-group)] e_value:4.09194e-53</td><td>11669.m04560|LOC_Os03g45720|protein expressed protein e_value:1.06643e-60</td><td>0.317</td><td>(0.0949 0.2994)</td><td>0.0949</td><td>0.2994</td></tr>
<tr><td>709</td><td>SET2_O19_CLUSTER_4484</td><td>gi|52077347|dbj|BAD46388.1| sunL protein-like [Oryza sativa (japonica cultivar-group)] e_value:0</td><td>11681.m02811|LOC_Os09g30100|protein sun protein fmu, putative e_value:0</td><td>0.188</td><td>(0.0896 0.4774)</td><td>0.0896</td><td>0.4774</td></tr>
<tr><td>710</td><td>SET1_O19_CLUSTER_1779</td><td>gi|50948731|ref|XP_493723.1| putative zinc finger transcription factor ZFP30 [Oryza sativa (japonica cultivar-group)]
>gi|5803246|dbj|BAA83556.1| putative zinc finger transcription factor ZFP30 [Oryza sativa (japonica cultivar-group)] e_value:3.85254e-</td><td>11680.m00417|LOC_Os06g04920|protein Zn-finger in Ran binding protein and others, putative e_value:4.19635e-67</td><td>0.17</td><td>(0.1129 0.6630)</td><td>0.1129</td><td>0.663</td></tr>
<tr><td>711</td><td>SET1_E09_CLUSTER_30800</td><td>gi|34911096|ref|NP_916895.1| OJ1117_G01.16 [Oryza sativa (japonica cultivar-group)] >gi|21104744|dbj|BAB93332.1| unknown protein [Oryza sativa (japonica cultivar-group)] e_value:9.9623e-104</td><td>11667.m04704|LOC_Os01g48500|protein expressed protein e_value:2.97477e-105</td><td>0.255</td><td>(0.0679 0.2662)</td><td>0.0679</td><td>0.2662</td></tr>
<tr><td>712</td><td>WT006_F14_CLUSTER_13890</td><td>gi|50920033|ref|XP_470377.1| putative plastid ribosomal protein S6 precursor [Oryza sativa (japonica cultivar-group)]
>gnl|BL_ORD_ID|1535243 gi|41469637|gb|AAS07360.1| putative plastid ribosomal protein S6 precursor [Oryza sativa (japonica cultivar-gro</td><td>11669.m06401|LOC_Os03g62630|protein putative plastid ribosomal protein S6 precursor e_value:2.39319e-84</td><td>0.18</td><td>(0.1226 0.6822)</td><td>0.1226</td><td>0.6822</td></tr>
<tr><td>713</td><td>WT003_P22_CLUSTER_19181</td><td>gi|50915754|ref|XP_468341.1| putative wall-associated kinase [Oryza sativa (japonica cultivar-group)] >gnl|BL_ORD_ID|1583877
gi|47848205|dbj|BAD22031.1| putative wall-associated kinase [Oryza sativa (japonica cultivar-group)] e_value:0</td><td>11668.m05570|LOC_Os02g56420|protein Protein kinase domain, putative e_value:0</td><td>0.238</td><td>(0.2531 1.0642)</td><td>0.2531</td><td>1.0642</td></tr>
<tr><td>714</td><td>WT003_I04_CLUSTER_25187</td><td>gi|51965068|ref|XP_507318.1| PREDICTED P0623F08.37 gene product [Oryza sativa (japonica cultivar-group)] >gnl|BL_ORD_ID|1610589
gi|50948183|ref|XP_483619.1| putative sorbitol dehydrogenase [Oryza sativa (japonica cultivar-group)] >gnl|BL_ORD_ID|1610</td><td>11674.m04359|LOC_Os08g43190|protein oxidoreductase, zinc-binding dehydrogenase family e_value:0</td><td>0.133</td><td>(0.0581 0.4374)</td><td>0.0581</td><td>0.4374</td></tr>
<tr><td>715</td><td>WT002_E20_CLUSTER_11479</td><td>gi|31433279|gb|AAP54817.1| unknown protein [Oryza sativa (japonica cultivar-group)] >gnl|BL_ORD_ID|2013737
gi|37536456|ref|NP_922530.1| unknown protein [Oryza sativa (japonica cultivar-group)] >gnl|BL_ORD_ID|2013737
gi|21717167|gb|AAM76360.1| unknow</td><td>11676.m03569|LOC_Os10g39200|protein expressed protein e_value:3.7306e-112</td><td>0.614</td><td>(0.6570 1.0698)</td><td>0.657</td><td>1.0698</td></tr>
<tr><td>716</td><td>WT002_C18_CLUSTER_10044</td><td>gi|50940891|ref|XP_479973.1| unknown protein [Oryza sativa (japonica cultivar-group)] >gnl|BL_ORD_ID|1601609
gi|38636820|dbj|BAD03060.1| unknown protein [Oryza sativa (japonica cultivar-group)] >gnl|BL_ORD_ID|1601609
gi|46390802|dbj|BAD16308.1| unkn</td><td>11674.m00330|LOC_Os08g04170|protein Zinc finger C-x8-C-x5-C-x3-H type (and similar), putative e_value:0</td><td>0.382</td><td>(0.2177 0.5697)</td><td>0.2177</td><td>0.5697</td></tr>
<tr><td>717</td><td>SET6_M16_CLUSTER_1563</td><td>gi|34895562|ref|NP_909127.1| unknown protein [Oryza sativa (japonica cultivar-group)] >gi|9049415|dbj|BAA99370.1| unknown protein [Oryza sativa (japonica cultivar-group)] e_value:1.09394e-44</td><td>11667.m00312|LOC_Os01g03980|protein expressed protein e_value:1.73902e-46</td><td>0.428</td><td>(0.2307 0.5390)</td><td>0.2307</td><td>0.539</td></tr>
<tr><td>718</td><td>SET5_N11_CLUSTER_14302</td><td>gi|50898790|ref|XP_450183.1| chloroplast RNA processing 1 -like protein [Oryza sativa (japonica cultivar-group)]
>gi|32526676|dbj|BAC79199.1| chloroplast RNA processing 1 -like protein [Oryza sativa (japonica cultivar-group)] e_value:0</td><td>11681.m03303|LOC_Os09g37070|protein hypothetical protein e_value:0</td><td>0.165</td><td>(0.1054 0.6383)</td><td>0.1054</td><td>0.6383</td></tr>
<tr><td>719</td><td>SET4_O10_CLUSTER_24083</td><td>gi|56785070|dbj|BAD82709.1| putative oligopeptide transporter [Oryza sativa (japonica cultivar-group)] >gi|56784227|dbj|BAD81722.1| putative oligopeptide transporter [Oryza sativa (japonica cultivar-group)] e_value:0</td><td>11667.m06540|LOC_Os01g65100|protein POT family, putative e_value:0</td><td>0.588</td><td>(0.2699 0.4593)</td><td>0.2699</td><td>0.4593</td></tr>
<tr><td>720</td><td>SET4_L08_CLUSTER_17872</td><td>gi|50939147|ref|XP_479101.1| unknown protein [Oryza sativa (japonica cultivar-group)] >gi|34395135|dbj|BAC84849.1| unknown protein [Oryza sativa (japonica cultivar-group)] e_value:8.26034e-46</td><td>11673.m04081|LOC_Os07g41700|protein expressed protein e_value:3.13955e-77</td><td>0.292</td><td>(0.1630 0.5576)</td><td>0.163</td><td>0.5576</td></tr>
<tr><td>721</td><td>SET4_G07_CLUSTER_14501</td><td>gi|34896628|ref|NP_909658.1| hypothetical protein [Oryza sativa] >gi|12583793|gb|AAG59645.1| hypothetical protein [Oryza sativa] e_value:1.15309e-56</td><td>11669.m03656|LOC_Os03g37090|protein expressed protein e_value:8.69405e-59</td><td>0.094</td><td>(0.0352 0.3744)</td><td>0.0352</td><td>0.3744</td></tr>
<tr><td>722</td><td>SET5_H17_CLUSTER_30950</td><td>gi|61657604|emb|CAI65403.1| dehydrin [Triticum turgidum subsp. durum] e_value:1.2815e-78</td><td>11667.m01231|LOC_Os01g12580|protein Late embryogenesis abundant protein e_value:5.04092e-70</td><td>0.334</td><td>(0.1115 0.3334)</td><td>0.1115</td><td>0.3334</td></tr>
<tr><td>723</td><td>WT002_H04_CLUSTER_5410</td><td>gi|50908187|ref|XP_465582.1| putative protein phosphatase 2C [Oryza sativa (japonica cultivar-group)] >gnl|BL_ORD_ID|1571638
gi|51964038|ref|XP_506802.1| PREDICTED P0403C01.30 gene product [Oryza sativa (japonica cultivar-group)] >gnl|BL_ORD_ID|1571</td><td>11668.m02561|LOC_Os02g27220|protein Protein phosphatase 2C, putative e_value:0</td><td>0.206</td><td>(0.1236 0.6008)</td><td>0.1236</td><td>0.6008</td></tr>
<tr><td>724</td><td>WT013_L01_CLUSTER_26888</td><td>gi|51964494|ref|XP_507032.1| PREDICTED OJ1111_C07.17-1 gene product [Oryza sativa (japonica cultivar-group)]
>gnl|BL_ORD_ID|1583460 gi|50915666|ref|XP_468297.1| putative epsin 2a [Oryza sativa (japonica cultivar-group)]
>gnl|BL_ORD_ID|1583460 gi|154</td><td>11668.m05554|LOC_Os02g56270|protein ENTH domain, putative e_value:0</td><td>0.243</td><td>(0.1479 0.6093)</td><td>0.1479</td><td>0.6093</td></tr>
</table>

PDF Complete
Your complimentary use period has ended. Thank you for using PDF Complete.
Click Here to upgrade to Unlimited Pages and Expanded Features

<table>
<tr><th></th><th></th><th></th><th></th><th></th><th></th><th></th><th></th></tr>
<tr><td>725</td><td>WT004_K21_CLUSTER_32002</td><td>gi|50252134|dbj|BAD28130.1| mechanosensitive ion channel domain-containing protein-like [Oryza sativa (japonica cultivar-group)] e_value:0</td><td>11668.m04318|LOC_Os02g44770|protein Mechanosensitive ion channel, putative e_value:0</td><td>0.23</td><td>(0.1239 0.5389)</td><td>0.1239</td><td>0.5389</td></tr>
<tr><td>726</td><td>WT005_K24_CLUSTER_503</td><td>gi|50934845|ref|XP_476950.1| unknown protein [Oryza sativa (japonica cultivar-group)] >gnl|BL_ORD_ID|1522326 gi|34394623|dbj|BAC83925.1| unknown protein [Oryza sativa (japonica cultivar-group)] >gnl|BL_ORD_ID|1522326 gi|50508955|dbj|BAD31859.1| unkn</td><td>11673.m00857|LOC_Os07g09150|protein expressed protein e_value:9.16383e-64</td><td>0.534</td><td>(0.3377 0.6324)</td><td>0.3377</td><td>0.6324</td></tr>
<tr><td>727</td><td>SET2_B17_CLUSTER_2349</td><td>gi|50920233|ref|XP_470477.1| putative ribosomal protein [Oryza sativa (japonica cultivar-group)] >gi|30102988|gb|AAP21401.1| putative ribosomal protein [Oryza sativa (japonica cultivar-group)] >gi|41393260|gb|AAS01983.1| putative ribosomal protein [</td><td>11669.m06566|LOC_Os03g63950|protein putative ribosomal protein e_value:1.16406e-97</td><td>0.617</td><td>(0.3155 0.5114)</td><td>0.3155</td><td>0.5114</td></tr>
<tr><td>728</td><td>SET1_E11_CLUSTER_20369</td><td>gi|50917147|ref|XP_468970.1| putative auxin-responsive protein [Oryza sativa (japonica cultivar-group)] >gi|41469496|gb|AAS07281.1| putative auxin-responsive protein [Oryza sativa (japonica cultivar-group)] >gi|53370658|gb|AAU89153.1| Auxin-responsi</td><td>11686.m04058|LOC_Os12g40890|protein aux/IAA protein e_value:6.40578e-104</td><td>0.265</td><td>(0.1155 0.4362)</td><td>0.1155</td><td>0.4362</td></tr>
<tr><td>729</td><td>WT008_H05_CLUSTER_30878</td><td>gi|50947563|ref|XP_483309.1| putative aminoacylase [Oryza sativa (japonica cultivar-group)] >gnl|BL_ORD_ID|1609608 gi|42408797|dbj|BAD10058.1| putative aminoacylase [Oryza sativa (japonica cultivar-group)] e_value:0</td><td>11674.m04021|LOC_Os08g40110|protein N-acyl-L-amino-acid amidohydrolase e_value:0</td><td>0.178</td><td>(0.0997 0.5598)</td><td>0.0997</td><td>0.5598</td></tr>
<tr><td>730</td><td>WT008_G20_CLUSTER_17870</td><td>gi|50919943|ref|XP_470332.1| putative transcription factor [Oryza sativa (japonica cultivar-group)] >gnl|BL_ORD_ID|1535145 gi|40714674|gb|AAR88580.1| putative transcription factor [Oryza sativa (japonica cultivar-group)] e_value:1.33539e-170</td><td>11686.m03482|LOC_Os12g35560|protein Similar to At3g05420 e_value:0</td><td>0.277</td><td>(0.1395 0.5034)</td><td>0.1395</td><td>0.5034</td></tr>
<tr><td>731</td><td>WT004_D01_CLUSTER_33940</td><td>gi|31431429|gb|AAP53210.1| putative nucleosid phosphatase [Oryza sativa (japonica cultivar-group)] >gnl|BL_ORD_ID|2010361 gi|37533242|ref|NP_920923.1| putative nucleosid phosphatase [Oryza sativa (japonica cultivar-group)] >gnl|BL_ORD_ID|2010361 gi|</td><td>11676.m01754|LOC_Os10g21000|protein GDA1/CD39 (nucleoside phosphatase) family, putative e_value:0</td><td>0.155</td><td>(0.0666 0.4309)</td><td>0.0666</td><td>0.4309</td></tr>
<tr><td>732</td><td>SET3_J02_CLUSTER_20406</td><td>gi|50905013|ref|XP_463995.1| putative amidotransferase [Oryza sativa (japonica cultivar-group)] >gi|41053060|dbj|BAD07990.1| putative amidotransferase [Oryza sativa (japonica cultivar-group)] >gi|41052553|dbj|BAD07735.1| putative amidotransferase [O</td><td>11668.m00305|LOC_Os02g03740|protein imidazoleglycerol-phosphate synthase subunit H, putative e_value:1.03592e-125</td><td>0.157</td><td>(0.0737 0.4693)</td><td>0.0737</td><td>0.4693</td></tr>
<tr><td>733</td><td>WT008_A05_CLUSTER_10774</td><td>gi|50936837|ref|XP_477946.1| putative salt tolerance protein [Oryza sativa (japonica cultivar-group)] >gnl|BL_ORD_ID|1526442 gi|51963402|ref|XP_506311.1| PREDICTED OSJNBa0027N13.110 gene product [Oryza sativa (japonica cultivar-group)] >gnl|BL_ORD_I</td><td>11673.m02568|LOC_Os07g27300|protein expressed protein e_value:5.68278e-123</td><td>0.119</td><td>(0.2143 1.7941)</td><td>0.2143</td><td>1.7941</td></tr>
<tr><td>734</td><td>SET5_B21_CLUSTER_34576</td><td>gi|31432870|gb|AAP54446.1| putative kinase [Oryza sativa (japonica cultivar-group)] >gi|37535714|ref|NP_922159.1| putative kinase [Oryza sativa (japonica cultivar-group)] >gi|18071420|gb|AAL58279.1| putative kinase [Oryza sativa (japonica cultivar-g</td><td>11676.m03160|LOC_Os10g35450|protein putative kinase e_value:0</td><td>0.15</td><td>(0.1449 0.9670)</td><td>0.1449</td><td>0.967</td></tr>
<tr><td>735</td><td>WT004_J01_CLUSTER_4542</td><td>gi|32488506|emb|CAE03258.1| OSJNBa0011J08.13 [Oryza sativa (japonica cultivar-group)] >gnl|BL_ORD_ID|1524070 gi|50928179|ref|XP_473617.1| OSJNBa0011J08.13 [Oryza sativa (japonica cultivar-group)] e_value:0</td><td>11670.m04811|LOC_Os04g48940|protein Mechanosensitive ion channel, putative e_value:0</td><td>0.337</td><td>(0.1723 0.5119)</td><td>0.1723</td><td>0.5119</td></tr>
<tr><td>736</td><td>WT004_L15_CLUSTER_12701</td><td>gi|31433634|gb|AAP55126.1| hypothetical protein [Oryza sativa (japonica cultivar-group)] >gnl|BL_ORD_ID|2013149 gi|37537074|ref|NP_922839.1| hypothetical protein [Oryza sativa (japonica cultivar-group)] >gnl|BL_ORD_ID|2013149 gi|12643043|gb|AAK00432</td><td>11676.m03912|LOC_Os10g42320|protein expressed protein e_value:0</td><td>0.182</td><td>(0.0819 0.4495)</td><td>0.0819</td><td>0.4495</td></tr>
<tr><td>737</td><td>SET6_E23_CLUSTER_18390</td><td>gi|55770019|ref|XP_549999.1| putative SufD [Oryza sativa (japonica cultivar-group)] >gi|53791281|dbj|BAD52546.1| putative SufD [Oryza sativa (japonica cultivar-group)] e_value:0</td><td>11667.m00277|LOC_Os01g03650|protein sufB/sufD domain, putative e_value:0</td><td>0.274</td><td>(0.1439 0.5245)</td><td>0.1439</td><td>0.5245</td></tr>
<tr><td>738</td><td>SET2_O16_CLUSTER_31920</td><td>gi|50926468|ref|XP_473181.1| OSJNBb0072M01.18 [Oryza sativa (japonica cultivar-group)] >gi|38344656|emb|CAD41482.2| OSJNBb0072M01.18 [Oryza sativa (japonica cultivar-group)] e_value:0</td><td>11670.m04222|LOC_Os04g43650|protein hypothetical protein e_value:3.40635e-177</td><td>0.129</td><td>(0.0663 0.5120)</td><td>0.0663</td><td>0.512</td></tr>
<tr><td>739</td><td>SET2_O22_CLUSTER_5784</td><td>gi|42528295|gb|AAS18416.1| benzothiadiazole-induced homeodomain protein 1; BTH-induced homeodomain protein 1 [Oryza sativa (indica cultivar-group)] e_value:0</td><td>11686.m04393|LOC_Os12g43950|protein expressed protein e_value:0</td><td>0.104</td><td>(0.0644 0.6194)</td><td>0.0644</td><td>0.6194</td></tr>
<tr><td>740</td><td>WT003_F11_CLUSTER_32947</td><td>gi|21537305|gb|AAM61646.1| unknown [Arabidopsis thaliana] e_value:0</td><td>11669.m01689|LOC_Os03g16800|protein Similar to AT4g32280/F10M6_80 e_value:0</td><td>0.075</td><td>(0.0449 0.5997)</td><td>0.0449</td><td>0.5997</td></tr>
<tr><td>741</td><td>WT009_K09_CLUSTER_34849</td><td>gi|51091721|dbj|BAD36521.1| putative splicing factor, arginine/serine-rich 7 [Oryza sativa (japonica cultivar-group)] >gnl|BL_ORD_ID|382892 gi|55773877|dbj|BAD72462.1| putative splicing factor, arginine/serine-rich 7 [Oryza sativa (japonica cultivar-gr</td><td>11668.m05392|LOC_Os02g54770|protein Transposable element protein, putative e_value:9.82991e-78</td><td>0.095</td><td>(0.0909 0.9574)</td><td>0.0909</td><td>0.9574</td></tr>
<tr><td>742</td><td>WT005_H06_CLUSTER_33296</td><td>gi|34894890|ref|NP_908770.1| B1140D12.10 [Oryza sativa (japonica cultivar-group)] e_value:3.51353e-66</td><td>11667.m02234|LOC_Os01g22990|protein hypothetical protein e_value:1.46175e-68</td><td>0.211</td><td>(0.1807 0.8552)</td><td>0.1807</td><td>0.8552</td></tr>
<tr><td>743</td><td>WT012__C02_CLUSTER_32282</td><td>gi|50924105|ref|XP_472413.1| OJ000126_13.12 [Oryza sativa (japonica cultivar-group)] >gnl|BL_ORD_ID|1591505 gi|38346330|emb|CAD40585.2| OJ000126_13.12 [Oryza sativa (japonica cultivar-group)] e_value:0</td><td>11670.m03249|LOC_Os04g33780|protein OTU-like cysteine protease, putative e_value:0</td><td>0.151</td><td>(0.0901 0.5959)</td><td>0.0901</td><td>0.5959</td></tr>
<tr><td>744</td><td>WT006_O09_CLUSTER_33909</td><td>gi|50947669|ref|XP_483362.1| putative mitochondrial energy transfer protein [Oryza sativa (japonica cultivar-group)] >gnl|BL_ORD_ID|1609688 gi|42409167|dbj|BAD10433.1| putative mitochondrial energy transfer protein [Oryza sativa (japonica cultivar-</td><td>11681.m02937|LOC_Os09g32200|protein mitochondrial carrier protein, putative e_value:3.54775e-177</td><td>0.187</td><td>(0.0972 0.5202)</td><td>0.0972</td><td>0.5202</td></tr>
<tr><td>745</td><td>WT006_H12_CLUSTER_1602</td><td>gi|50905143|ref|XP_464060.1| putative 50S ribosomal protein L3 [Oryza sativa (japonica cultivar-group)] >gnl|BL_ORD_ID|1565916 gi|42409256|dbj|BAD10519.1| putative 50S ribosomal protein L3 [Oryza sativa (japonica cultivar-group)] >gnl|BL_ORD_ID|1565</td><td>11668.m00382|LOC_Os02g04460|protein ribosomal protein L3, putative e_value:7.481e-127</td><td>0.174</td><td>(0.0658 0.3783)</td><td>0.0658</td><td>0.3783</td></tr>
<tr><td>746</td><td>SET6_O16_CLUSTER_1602</td><td>gi|50905143|ref|XP_464060.1| putative 50S ribosomal protein L3 [Oryza sativa (japonica cultivar-group)] >gi|42409256|dbj|BAD10519.1| putative 50S ribosomal protein L3 [Oryza sativa (japonica cultivar-group)] >gi|42409125|dbj|BAD10375.1| putative 50S</td><td>11668.m00382|LOC_Os02g04460|protein ribosomal protein L3, putative e_value:3.35807e-127</td><td>0.148</td><td>(0.0630 0.4251)</td><td>0.063</td><td>0.4251</td></tr>
<tr><td>747</td><td>WT002_N03_CLUSTER_31618</td><td>gi|50948223|ref|XP_483639.1| putative RNA-binding protein [Oryza sativa (japonica cultivar-group)] >gnl|BL_ORD_ID|1610610 gi|42408712|dbj|BAD09930.1| putative RNA-binding protein [Oryza sativa (japonica cultivar-group)] >gnl|BL_ORD_ID|1610610 gi|424</td><td>11674.m04377|LOC_Os08g43360|protein RNA recognition motif. (a.k.a. RRM, RBD, or RNP domain), putative e_value:4.15367e-</td><td>0.132</td><td>(0.0668 0.5079)</td><td>0.0668</td><td>0.5079</td></tr>
<tr><td>748</td><td>WT011_N17_CLUSTER_24720</td><td>gi|50251962|dbj|BAD27897.1| putative u1 small nuclear ribonucleoprotein C [Oryza sativa (japonica cultivar-group)] e_value:4.19645e-92</td><td>11668.m01567|LOC_Os02g16640|protein At4g03120 e_value:7.69713e-94</td><td>0.266</td><td>(0.1176 0.4427)</td><td>0.1176</td><td>0.4427</td></tr>
<tr><td>749</td><td>WT004_N12_CLUSTER_32961</td><td>gi|20334738|gb|AAM16230.1| At1g68530/T26J14_10 [Arabidopsis thaliana] >gnl|BL_ORD_ID|978641 gi|17979464|gb|AAL50069.1| At1g68530/T26J14_10 [Arabidopsis thaliana] e_value:0</td><td>11669.m01159|LOC_Os03g12030|protein very-long-chain fatty acid condensing enzyme CUT1 e_value:0</td><td>0.2</td><td>(0.0537 0.2688)</td><td>0.0537</td><td>0.2688</td></tr>
<tr><td>750</td><td>WT012_B07_CLUSTER_7714</td><td>gi|47827092|dbj|BAD21122.1| ATP-dependent RNA helicase [Hordeum vulgare subsp. vulgare] e_value:0</td><td>11669.m06233|LOC_Os03g61220|protein Transposable element protein, putative e_value:0</td><td>0.128</td><td>(0.0810 0.6308)</td><td>0.081</td><td>0.6308</td></tr>
<tr><td>751</td><td>SET3_B17_CLUSTER_31360</td><td>gi|51963354|ref|XP_506253.1| PREDICTED OSJNBa0004F07.8 gene product [Oryza sativa (japonica cultivar-group)] e_value:5.69482e-105</td><td>11673.m01577|LOC_Os07g16130|protein acetyltransferase, GNAT family, putative e_value:1.74603e-106</td><td>0.236</td><td>(0.1742 0.7374)</td><td>0.1742</td><td>0.7374</td></tr>
<tr><td>752</td><td>WT012_O06_CLUSTER_7850</td><td>gi|50915804|ref|XP_468366.1| putative LEUNIG [Oryza sativa (japonica cultivar-group)] >gnl|BL_ORD_ID|1583557 gi|47848544|dbj|BAD22396.1| putative LEUNIG [Oryza sativa (japonica cultivar-group)] >gnl|BL_ORD_ID|1583557 gi|47847864|dbj|BAD21657.1| puta</td><td>11670.m04165|LOC_Os04g43130|protein expressed protein e_value:0</td><td>0.122</td><td>(0.0740 0.6058)</td><td>0.074</td><td>0.6058</td></tr>
<tr><td>753</td><td>WT009_O01_CLUSTER_19008</td><td>gi|3928519|dbj|BAA34675.1| wpk4 protein kinase [Triticum aestivum] e_value:0</td><td>11682.m04173|LOC_Os05g43840|protein OsPK4 e_value:0</td><td>0.205</td><td>(0.1472 0.7196)</td><td>0.1472</td><td>0.7196</td></tr>
<tr><td>754</td><td>SET4_D19_CLUSTER_20182</td><td>gi|50931287|ref|XP_475171.1| unknown protein [Oryza sativa (japonica cultivar-group)] >gi|47777423|gb|AAT38057.1| unknown protein [Oryza sativa (japonica cultivar-group)] e_value:1.6009e-99</td><td>11682.m02946|LOC_Os05g31680|protein expressed protein e_value:0</td><td>0.306</td><td>(0.1711 0.5586)</td><td>0.1711</td><td>0.5586</td></tr>
<tr><td>755</td><td>WT002_N24_CLUSTER_28157</td><td>gi|38344933|emb|CAE03032.2| OSJNBa0084A10.7 [Oryza sativa (japonica cultivar-group)] >gnl|BL_ORD_ID|1521462 gi|50924372|ref|XP_472546.1| OSJNBa0084A10.7 [Oryza sativa (japonica cultivar-group)] e_value:2.91001e-166</td><td>11670.m03397|LOC_Os04g35190|protein F-box domain, putative e_value:1.04856e-171</td><td>0.162</td><td>(0.0747 0.4601)</td><td>0.0747</td><td>0.4601</td></tr>
<tr><td>756</td><td>WT008_B11_CLUSTER_34608</td><td>gi|50933665|ref|XP_476360.1| putative transmembrane protein(TOM3) [Oryza sativa (japonica cultivar-group)] >gnl|BL_ORD_ID|1517691 gi|50508933|dbj|BAD31838.1| putative transmembrane protein(TOM3) [Oryza sativa (japonica cultivar-group)] e_value:5.29028e</td><td>11673.m00028|LOC_Os07g01250|protein expressed protein e_value:1.60693e-144</td><td>0.119</td><td>(0.0679 0.5702)</td><td>0.0679</td><td>0.5702</td></tr>
<tr><td>757</td><td>WT006_G15_CLUSTER_33039</td><td>gi|50946053|ref|XP_482554.1| putative NAD-malate dehydrogenase [Oryza sativa (japonica cultivar-group)] >gnl|BL_ORD_ID|1606493 gi|42407501|dbj|BAD10618.1| putative NAD-malate dehydrogenase [Oryza sativa (japonica cultivar-group)] >gnl|BL_ORD_ID|1606</td><td>11667.m06116|LOC_Os01g61380|protein malate dehydrogenase, NAD-dependent, putative e_value:0</td><td>0.136</td><td>(0.0757 0.5548)</td><td>0.0757</td><td>0.5548</td></tr>
<tr><td>758</td><td>WT005_N07_CLUSTER_32928</td><td>gi|34903076|ref|NP_912885.1| unnamed protein product [Oryza sativa (japonica cultivar-group)] e_value:6.82448e-134</td><td>11667.m01602|LOC_Os01g16000|protein CRAL/TRIO, N-terminus, putative e_value:5.55937e-132</td><td>0.183</td><td>(0.1088 0.5961)</td><td>0.1088</td><td>0.5961</td></tr>
<tr><td>759</td><td>WT005_P05_CLUSTER_32877</td><td>gi|14586371|emb|CAC42902.1| putative protein [Arabidopsis thaliana] >gnl|BL_ORD_ID|1510334 gi|22022558|gb|AAM83236.1| At5g12410 [Arabidopsis thaliana] >gnl|BL_ORD_ID|1510334 gi|18416857|ref|NP_568274.1| THUMP domain-containing protein [Arabidopsis t</td><td>11687.m03312|LOC_Os11g36060|protein BT001143 At5g12410/At5g12410 e_value:3.14199e-121</td><td>0.256</td><td>(0.1539 0.6023)</td><td>0.1539</td><td>0.6023</td></tr>
<tr><td>760</td><td>WT005_I16_CLUSTER_36</td><td>gi|50932159|ref|XP_475607.1| unknown protein [Oryza sativa (japonica cultivar-group)] >gnl|BL_ORD_ID|1597105 gi|45267868|gb|AAS55767.1| unknown protein [Oryza sativa (japonica cultivar-group)] e_value:5.38475e-73</td><td>11682.m00601|LOC_Os05g06500|protein AT3g17020/K14A17_14 e_value:8.35727e-75</td><td>0.255</td><td>(0.1065 0.4178)</td><td>0.1065</td><td>0.4178</td></tr>
<tr><td>761</td><td>SET2_P04_CLUSTER_19539</td><td>gi|50919955|ref|XP_470338.1| putative RNA binding protein [Oryza sativa (japonica cultivar-group)] >gi|40714682|gb|AAR88588.1| putative RNA binding protein [Oryza sativa (japonica cultivar-group)] e_value:1.96166e-89</td><td>11669.m06325|LOC_Os03g61990|protein Transposable element protein, putative e_value:4.5863e-91</td><td>0.139</td><td>(0.0802 0.5774)</td><td>0.0802</td><td>0.5774</td></tr>
<tr><td>762</td><td>SET1_A24_CLUSTER_31455</td><td>gi|50925823|ref|XP_473006.1| OSJNBb0091E11.5 [Oryza sativa (japonica cultivar-group)] >gi|38345414|emb|CAD41536.2| OSJNBb0091E11.5 [Oryza sativa (japonica cultivar-group)] e_value:1.79707e-84</td><td>11670.m03995|LOC_Os04g41580|protein expressed protein e_value:3.74815e-85</td><td>0.36</td><td>(0.1999 0.5557)</td><td>0.1999</td><td>0.5557</td></tr>
<tr><td>763</td><td>WT009__C06_CLUSTER_6915</td><td>gi|42569688|ref|NP_181242.2| leucine-rich repeat family protein / protein kinase family protein [Arabidopsis thaliana] e_value:0</td><td>11687.m00020|LOC_Os11g01200|protein receptor protein kinase, putative e_value:0</td><td>0.147</td><td>(0.1067 0.7250)</td><td>0.1067</td><td>0.725</td></tr>
<tr><td>764</td><td>SET5_A12_CLUSTER_33717</td><td>gi|50932021|ref|XP_475538.1| hypothetical protein [Oryza sativa (japonica cultivar-group)] e_value:3.11515e-101</td><td>11682.m00259|LOC_Os05g03320|protein expressed protein e_value:2.81376e-109</td><td>0.402</td><td>(0.4837 1.2024)</td><td>0.4837</td><td>1.2024</td></tr>
<tr><td>765</td><td>SET2_J06_CLUSTER_34688</td><td>gi|50906349|ref|XP_464663.1| Serine/threonine protein phosphatase PP2A-3 catalytic subunit [Oryza sativa (japonica cultivar-group)] >gi|5231210|gb|AAD41126.1| serine/threonine protein phosphatase PP2A-3 catalytic subunit [Oryza sativa (indica cultivar-</td><td>11668.m01154|LOC_Os02g12580|protein serine/threonine protein phosphatase pp2a-3 catalytic subunit(ec 3.1.3.16)</td><td>0.026</td><td>(0.0113 0.4303)</td><td>0.0113</td><td>0.4303</td></tr>
<tr><td>766</td><td>WT012_G01_CLUSTER_34792</td><td>gi|38346760|emb|CAE03865.2| OSJNBa0081C01.11 [Oryza sativa (japonica cultivar-group)] >gnl|BL_ORD_ID|1535266 gi|38346629|emb|CAD41212.2| OSJNBa0074L08.23 [Oryza sativa (japonica cultivar-group)] >gnl|BL_ORD_ID|1535266 gi|50926656|ref|XP_473275.1| OS</td><td>11670.m04348|LOC_Os04g44840|protein beta-xylosidase, putative e_value:0</td><td>0.184</td><td>(0.1049 0.5689)</td><td>0.1049</td><td>0.5689</td></tr>
<tr><td>767</td><td>WT010_G11_CLUSTER_27459</td><td>gi|62733576|gb|AAX95693.1| CAP protein [Oryza sativa (japonica cultivar-group)] e_value:0</td><td>11669.m05169|LOC_Os03g51250|protein putative adenylyl cyclase associated protein e_value:0</td><td>0.215</td><td>(0.1125 0.5239)</td><td>0.1125</td><td>0.5239</td></tr>
</table>

PDF Complete
Your complimentary use period has ended. Thank you for using PDF Complete.
Click Here to upgrade to Unlimited Pages and Expanded Features

768	SET1_F04_CLUSTER_27155	gi|51090890|dbj|BAD35463.1| putative heme oxygenase 1 [Oryza sativa (japonica cultivar-group)] e_value:8.29986e-117	11680.m03908|LOC_Os06g40080|protein heme oxygenase 1 e_value:1.90518e-118	0.304	(0.1455 0.4778)	0.1455	0.4778
769	WT002_A13_CLUSTER_12109	gi|11281550|pir||T51606 probable 26S proteasome non-ATPase chain S5a [imported] - rice >gnl|BL_ORD_ID|247003 gi|17297981|dbj|BAB78488.1| 26S proteasome regulatory particle non-ATPase subunit10 [Oryza sativa (japonica cultivar-group)] >gnl|BL_ORD_ID|	11669.m01372|LOC_Os03g13970|protein probable 26S proteasome non-ATPase chain S5a [imported] - rice e_value:0	0.103	(0.0507 0.4932)	0.0507	0.4932
770	SET1_C19_CLUSTER_34771	gi|32966065|gb|AAP92129.1| GTP-binding protein GTP1 [Oryza sativa (japonica cultivar-group)] >gi|34909538|ref|NP_916116.1| putative GTP-binding protein [Oryza sativa (japonica cultivar-group)] >gi|14209558|dbj|BAB56054.1| GTP-binding protein GTP1 [O	11667.m05380|LOC_Os01g54590|protein Ras family, putative e_value:1.47935e-111	0.142	(0.0537 0.3780)	0.0537	0.378
771	WT010_J01_CLUSTER_8521	gi|62733310|gb|AAX95427.1| DNA Helicase-related [Oryza sativa (japonica cultivar-group)] e_value:0	11687.m04525|LOC_Os11g48090|protein DNA Helicase e_value:0	0.168	(0.0984 0.5858)	0.0984	0.5858
772	WT002_D16_CLUSTER_18789	gi|50921225|ref|XP_470973.1| OSJNBa0094O15.16 [Oryza sativa (japonica cultivar-group)] >gnl|BL_ORD_ID|1589067 gi|32489689|emb|CAE04604.1| OSJNBb0004G23.2 [Oryza sativa (japonica cultivar-group)] >gnl|BL_ORD_ID|1589067 gi|38346209|emb|CAD39347.2| OSJ	11670.m00052|LOC_Os04g01500|protein hypothetical protein e_value:1.18617e-38	0.745	(0.1949 0.2615)	0.1949	0.2615
773	WT003_C03_CLUSTER_4869	gi|9624495|gb|AAF90200.1| chlorophyll a/b-binding protein precursor [Hordeum vulgare] e_value:4.98361e-130	11674.m03347|LOC_Os08g33820|protein chlorophyll a/b-binding protein presursor e_value:1.81741e-116	0.264	(0.1157 0.4380)	0.1157	0.438
774	WT011_N23_CLUSTER_12095	gi|49328155|gb|AAT58851.1| putative 1-deoxy-D-xylulose-5-phosphate synthase [Oryza sativa (japonica cultivar-group)] e_value:0	11673.m00861|LOC_Os07g09190|protein 1-deoxy-D-xylulose-5-phosphate synthase e_value:0	0.352	(0.2355 0.6689)	0.2355	0.6689
775	WT005_N18_CLUSTER_34531	gi|31433330|gb|AAP54859.1| putative ubiquitin protein [Oryza sativa (japonica cultivar-group)] >gnl|BL_ORD_ID|2012797 gi|37536540|ref|NP_922572.1| putative ubiquitin protein [Oryza sativa (japonica cultivar-group)] >gnl|BL_ORD_ID|2012797 gi|10140758	11676.m03613|LOC_Os10g39620|protein putative ubiquitin protein e_value:0	0.205	(0.0985 0.4814)	0.0985	0.4814
776	WT007_P08_CLUSTER_21823	gi|50915654|ref|XP_468291.1| putative biquinone biosynthesis protein ubiB [Oryza sativa (japonica cultivar-group)] >gnl|BL_ORD_ID|1584199 gi|47497391|dbj|BAD19429.1| putative ubiquinone biosynthesis protein ubiB [Oryza sativa (japonica cultivar-	11668.m05546|LOC_Os02g56200|protein ABC1 family, putative e_value:0	0.23	(0.1405 0.6119)	0.1405	0.6119
777	SET5_P09_CLUSTER_11603	gi|50878311|gb|AAT85086.1| unknown protein [Oryza sativa (japonica cultivar-group)] e_value:2.6051e-116	11682.m03154|LOC_Os05g33620|protein expressed protein e_value:3.31632e-136	0.33	(0.1372 0.4156)	0.1372	0.4156
778	WT012_K16_CLUSTER_33751	gi|55168049|gb|AAV43917.1| unknown protein [Oryza sativa (japonica cultivar-group)] e_value:5.27305e-81	11682.m00621|LOC_Os05g06700|protein expressed protein e_value:4.926e-83	0.342	(0.1813 0.5299)	0.1813	0.5299
779	WT010_I18_CLUSTER_32564	gi|53792787|dbj|BAD53821.1| putative myo-inositol oxygenase [Oryza sativa (japonica cultivar-group)] >gnl|BL_ORD_ID|260327 gi|53792729|dbj|BAD53740.1| putative myo-inositol oxygenase [Oryza sativa (japonica cultivar-group)] e_value:1.0642e-162	11680.m03547|LOC_Os06g36560|protein expressed protein e_value:2.485e-164	0.126	(0.0542 0.4303)	0.0542	0.4303
780	WT013_C02_CLUSTER_33047	gi|26452440|dbj|BAC43305.1| unknown protein [Arabidopsis thaliana] >gnl|BL_ORD_ID|1092270 gi|26451774|dbj|BAC42982.1| unknown protein [Arabidopsis thaliana] >gnl|BL_ORD_ID|1092270 gi|51971491|dbj|BAD44410.1| unknown protein [Arabidopsis thaliana] &g	11682.m02211|LOC_Os05g24020|protein expressed protein e_value:0	0.154	(0.0748 0.4865)	0.0748	0.4865
781	WT002_F11_CLUSTER_14922	gi|50947629|ref|XP_483342.1| putative C2H2 zinc-finger protein [Oryza sativa (japonica cultivar-group)] >gnl|BL_ORD_ID|1609655 gi|42408759|dbj|BAD09995.1| putative C2H2 zinc-finger protein [Oryza sativa (japonica cultivar-group)] >gnl|BL_ORD_ID|1609	11674.m04068|LOC_Os08g40560|protein expressed protein e_value:0	0.202	(0.0868 0.4297)	0.0868	0.4297
782	WT004_N07_CLUSTER_30895	gi|32489879|emb|CAE04359.1| OSJNBa0060P14.10 [Oryza sativa (japonica cultivar-group)] >gnl|BL_ORD_ID|1535977 gi|50924870|ref|XP_472780.1| OSJNBa0060P14.10 [Oryza sativa (japonica cultivar-group)] e_value:2.9292e-132	11670.m03707|LOC_Os04g39040|protein UBX domain, putative e_value:1.42856e-138	0.154	(0.0902 0.5866)	0.0902	0.5866
783	SET2_O08_CLUSTER_4523	gi|55908872|gb|AAV67815.1| unknown protein [Oryza sativa (japonica cultivar-group)] >gi|50932557|ref|XP_475806.1| putative transcription factor (contains Myb-like DNA-binding domain), PF00249 [Oryza sativa (japonica cultivar-group)] e_value:0	11682.m04452|LOC_Os05g46330|protein Myb-like DNA-binding domain, putative e_value:0	0.163	(0.0734 0.4496)	0.0734	0.4496
784	WT006_G12_CLUSTER_32076	gi|50904927|ref|XP_463952.1| putative 3-isopropylmalate dehydratase large subunit [Oryza sativa (japonica cultivar-group)] >gnl|BL_ORD_ID|1566190 gi|41053038|dbj|BAD07969.1| putative 3-isopropylmalate dehydratase large subunit [Oryza sativa (japonica c	11668.m00254|LOC_Os02g03260|protein homoaconitate hydratase family protein e_value:0	0.103	(0.0491 0.4780)	0.0491	0.478
785	WT003_M13_CLUSTER_32275	gi|56783883|dbj|BAD81320.1| putative eukaryotic translation initiation factor 3 subunit 6 interacting protein [Oryza sativa (japonica cultivar-group)] >gnl|BL_ORD_ID|971217 gi|56784380|dbj|BAD82419.1| putative eukaryotic translation initiation factor 3	11667.m01262|LOC_Os01g12870|protein hypothetical protein e_value:0	0.1	(0.0562 0.5598)	0.0562	0.5598
786	WT003_B11_CLUSTER_13943	gi|50915400|ref|XP_468164.1| protein kinase-like [Oryza sativa (japonica cultivar-group)] >gnl|BL_ORD_ID|1583283 gi|47497159|dbj|BAD19207.1| protein kinase-like [Oryza sativa (japonica cultivar-group)] e_value:0	11668.m05365|LOC_Os02g54510|protein Protein kinase domain, putative e_value:0	0.177	(0.1135 0.6422)	0.1135	0.6422
787	WT013_J11_CLUSTER_4948	gi|50943729|ref|XP_481392.1| putative fatty acyl coA reductase [Oryza sativa (japonica cultivar-group)] e_value:0	11674.m01946|LOC_Os08g20200|protein AJ459250 fatty acyl coA reductase e_value:0	0.194	(0.1542 0.7952)	0.1542	0.7952
788	WT003_B01_CLUSTER_4948	gi|50508139|dbj|BAD30714.1| putative C2 domain-containing protein [Oryza sativa (japonica cultivar-group)] e_value:0	11670.m05432|LOC_Os04g55220|protein Ca2+-dependent lipid-binding protein, putative e_value:0	0.12	(0.2284 1.9069)	0.2284	1.9069
789	WT009_C03_CLUSTER_24982	gi|34996397|emb|CAE47438.1| putative 1-deoxy-D-xylulose 5-phosphate reductoisomerase [Hordeum vulgare subsp. vulgare] e_value:0	11667.m00074|LOC_Os01g01710|protein 1-deoxy-D-xylulose 5-phosphate reductoisomerase e_value:0	0.118	(0.0656 0.5577)	0.0656	0.5577
790	WT010_H21_CLUSTER_32889	gi|50949097|ref|XP_493906.1| similar to ABC transporter of Arabidopsis thaliana (AC004697) [Oryza sativa] >gnl|BL_ORD_ID|1742123 gi|8099136|dbj|BAA90508.1| similar to ABC transporter of Arabidopsis thaliana (AC004697) [Oryza sativa] e_value:0	11682.m00214|LOC_Os05g02890|protein ABC transporter, putative e_value:0	0.303	(0.1164 0.3837)	0.1164	0.3837
791	SET4_I09_CLUSTER_15735	gi|50399931|gb|AAT76319.1| putative myo-inositol monophosphatase [Oryza sativa (japonica cultivar-group)] e_value:1.79743e-126	11669.m03855|LOC_Os03g39000|protein putative myo-inositol monophosphatase e_value:4.77331e-128	0.1	(0.0406 0.4048)	0.0406	0.4048
792	WT003_G09_CLUSTER_5745	gi|7329665|emb|CAB82762.1| putative protein [Arabidopsis thaliana] >gnl|BL_ORD_ID|1455462 gi|29824313|gb|AAP04117.1| unknown protein [Arabidopsis thaliana] >gnl|BL_ORD_ID|1455462 gi|26450336|dbj|BAC42284.1| unknown protein [Arabidopsis thaliana] &gt	11682.m03822|LOC_Os05g40180|protein Protein kinase domain, putative e_value:0	0.446	(0.1774 0.3976)	0.1774	0.3976
793	WT008_M06_CLUSTER_30577	gi|34894492|ref|NP_908571.1| OJ1116_C07.9 [Oryza sativa (japonica cultivar-group)] >gnl|BL_ORD_ID|1560893 gi|57899467|dbj|BAD88403.1| lipase class 3-like protein [Oryza sativa (japonica cultivar-group)] >gnl|BL_ORD_ID|1560893 gi|20805147|dbj|BAB9281	11667.m02009|LOC_Os01g20840|protein Lipase 3 N-terminal region, putative e_value:0	0.135	(0.0715 0.5291)	0.0715	0.5291
794	WT007_I21_CLUSTER_33833	gi|50945881|ref|XP_482468.1| putative methylcrotonyl-CoA carboxylase beta chain, mitochondrial precursor [Oryza sativa (japonica cultivar-group)] >gnl|BL_ORD_ID|1606393 gi|37806289|dbj|BAC99804.1| putative methylcrotonyl-CoA carboxylase beta chain, mit	11674.m03244|LOC_Os08g32850|protein methylcrotonyl-coa carboxylase beta chain, mitochondrial precursor(ec 6.4.1.4) (3-methylcrotonyl-coa carboxylase 2) (mccase betasubunit) (3-methylcrotonyl-coa:carbon dioxide ligase beta subunit) e_value:0	0.128	(0.0499 0.3887)	0.0499	0.3887
795	WT009_E16_CLUSTER_342	gi|50911543|ref|XP_467179.1| putative Scl1 protein [Oryza sativa (japonica cultivar-group)] >gnl|BL_ORD_ID|1580281 gi|50251749|dbj|BAD27682.1| putative Scl1 protein [Oryza sativa (japonica cultivar-group)] >gnl|BL_ORD_ID|1580281	11668.m04274|LOC_Os02g44370|protein GRAS family transcription factor, putative e_value:0	0.183	(0.0965 0.5275)	0.0965	0.5275
796	SET5_F15_CLUSTER_17361	gi|34898712|ref|NP_910702.1| putative sphingosine kinase [Oryza sativa (japonica cultivar-group)] >gi|50508454|dbj|BAD30563.1| putative sphingosine kinase [Oryza sativa (japonica cultivar-group)] >gi|23617201|dbj|BAC20872.1| putative sphingosine kin	11673.m03644|LOC_Os07g37580|protein hypothetical protein e_value:0	0.321	(0.1537 0.4790)	0.1537	0.479
797	WT012_J15_CLUSTER_7384	gi|34902158|ref|NP_912425.1| Putative growth regulator protein [Oryza sativa (japonica cultivar-group)] >gnl|BL_ORD_ID|1588864 gi|24899401|gb|AAN65001.1| Putative growth regulator protein [Oryza sativa (japonica cultivar-group)] e_value:0	11669.m02160|LOC_Os03g21090|protein Plant protein family, putative e_value:0	0.141	(0.0744 0.5266)	0.0744	0.5266
798	WT011_L08_CLUSTER_2512	gi|9755821|emb|CAC01852.1| putative protein [Arabidopsis thaliana] >gnl|BL_ORD_ID|1488603 gi|30685676|ref|NP_568326.2| expressed protein [Arabidopsis thaliana] >gnl|BL_ORD_ID|1488603 gi|11346186|pir||T51481 hypothetical protein T21H19_30 - Arabidops	11686.m04052|LOC_Os12g40840|protein expressed protein e_value:2.50763e-52	0.315	(0.1420 0.4508)	0.142	0.4508
799	WT004_J09_CLUSTER_22153	gi|31432924|gb|AAP54495.1| putative folylpolyglutamate synthetase [Oryza sativa (japonica cultivar-group)] >gnl|BL_ORD_ID|2012543 gi|37535812|ref|NP_922208.1| putative folylpolyglutamate synthetase [Oryza sativa (japonica cultivar-group)] >gnl|BL_OR	11669.m00122|LOC_Os03g02030|protein dihydrofolate synthetase e_value:0	0.233	(0.1237 0.5318)	0.1237	0.5318
800	WT009_J20_CLUSTER_28544	gi|38346804|emb|CAE04154.2| OSJNBa0088A01.11 [Oryza sativa (japonica cultivar-group)] >gnl|BL_ORD_ID|1535297 gi|50928251|ref|XP_473653.1| OSJNBa0088A01.11 [Oryza sativa (japonica cultivar-group)] e_value:0	11670.m04852|LOC_Os04g49350|protein expressed protein e_value:0	0.205	(0.1102 0.5371)	0.1102	0.5371
801	WT013_D23_CLUSTER_15277	gi|50920477|ref|XP_470599.1| Unknown protein [Oryza sativa (japonica cultivar-group)] >gnl|BL_ORD_ID|1588743 gi|20532324|gb|AAM27470.1| Unknown protein [Oryza sativa (japonica cultivar-group)] e_value:1.5973e-85	11669.m01233|LOC_Os03g12700|protein expressed protein e_value:1.29659e-88	0.512	(0.2282 0.4455)	0.2282	0.4455
802	WT012_O17_CLUSTER_24206	gi|19699347|gb|AAL91283.1| At2g38860/T7F6.3 [Arabidopsis thaliana] >gnl|BL_ORD_ID|2330184 gi|15081759|gb|AAK82534.1| At2g38860/T7F6.3 [Arabidopsis thaliana] >gnl|BL_ORD_ID|2330184 gi|25408625|pir||C84810 hypothetical protein At2g38860 [imported] -	11687.m03505|LOC_Os11g37920|protein intracellular protease, PfpI family, putative e_value:7.6169e-175	0.357	(0.1278 0.3581)	0.1278	0.3581
803	WT012_J10_CLUSTER_23997	gi|50924141|ref|XP_472431.1| OJ991214_12.18 [Oryza sativa (japonica cultivar-group)] >gnl|BL_ORD_ID|1591541 gi|39545715|emb|CAD40929.3| OSJNBa0033G16.5 [Oryza sativa (japonica cultivar-group)] >gnl|BL_ORD_ID|1591541 gi|38344037|emb|CAE01529.2| OJ991	11670.m03271|LOC_Os04g34000|protein hypothetical protein e_value:0	0.203	(0.0908 0.4474)	0.0908	0.4474
804	WT011_H09_CLUSTER_28484	gi|50899972|ref|XP_450774.1| putative Avr9/Cf-9 rapidly elicited protein 276 [Oryza sativa (japonica cultivar-group)] >gnl|BL_ORD_ID|1564081 gi|49389067|dbj|BAD26307.1| putative Avr9/Cf-9 rapidly elicited protein 276 [Oryza sativa (japonica cultivar-gr	11681.m01911|LOC_Os09g21120|protein U-box domain, putative e_value:0	0.394	(0.1683 0.4270)	0.1683	0.427
805	WT010_H05_CLUSTER_24007	gi|50909767|ref|XP_466372.1| cryptochrome 1a [Oryza sativa (japonica cultivar-group)] >gnl|BL_ORD_ID|1576930 gi|46806340|dbj|BAD17529.1| cryptochrome 1a [Oryza sativa (japonica cultivar-group)] e_value:0	11668.m03462|LOC_Os02g36380|protein FAD binding domain of DNA photolyase, putative e_value:0	0.169	(0.0928 0.5481)	0.0928	0.5481
806	WT010_C05_CLUSTER_29349	gi|50900016|ref|XP_450796.1| hypothetical protein [Oryza sativa (japonica cultivar-group)] >gnl|BL_ORD_ID|1564103 gi|49388885|dbj|BAD26095.1| hypothetical protein [Oryza sativa (japonica cultivar-group)] e_value:4.06168e-65	11681.m01933|LOC_Os09g21330|protein hypothetical protein e_value:1.38051e-68	0.597	(0.4767 0.7980)	0.4767	0.798
807	WT008_K24_CLUSTER_12671	gi|34905060|ref|NP_913877.1| putative flavonol 4'-sulfotransferase [Oryza sativa (japonica cultivar-group)] >gnl|BL_ORD_ID|1609633 gi|28201268|dbj|BAC56777.1| putative flavonol 4'-sulfotransferase [Oryza sativa (japonica cultivar-	11674.m04050|LOC_Os08g40380|protein Sulfotransferase domain, putative e_value:1.00145e-90	0.43	(0.3256 0.7578)	0.3256	0.7578
808	WT008_K21_CLUSTER_23721	gi|34898170|ref|NP_910431.1| hypothetical protein [Oryza sativa (japonica cultivar-group)] >gnl|BL_ORD_ID|1534713 gi|22831181|dbj|BAC16040.1| hypothetical protein [Oryza sativa (japonica cultivar-group)] e_value:2.73503e-55	11686.m04290|LOC_Os12g42950|protein hypothetical protein e_value:3.13812e-60	0.469	(0.5534 1.1796)	0.5534	1.1796
809	WT008_J23_CLUSTER_20089	gi|34895392|ref|NP_909039.1| putative ATP-binding-cassette protein [Oryza sativa (japonica cultivar-group)] >gnl|BL_ORD_ID|1751586 gi|13486800|dbj|BAB40032.1| putative ATP-binding-cassette protein [Oryza sativa (japonica cultivar-group)] e_value:0	11667.m00227|LOC_Os01g03150|protein expressed protein e_value:0	0.089	(0.0543 0.6122)	0.0543	0.6122

PDF Complete
Your complimentary use period has ended. Thank you for using PDF Complete.
Click Here to upgrade to Unlimited Pages and Expanded Features

<table>
<tr><td>810</td><td>WT008_G05_CLUSTER_24233</td><td>gi|34894012|ref|NP_908331.1| putative DnaJ-like protein [Oryza sativa (japonica cultivar-group)] >gnl|BL_ORD_ID|1751522 gi|15408854|dbj|BAB64243.1| putative DnaJ-like protein [Oryza sativa (japonica cultivar-group)] >gnl|BL_ORD_ID|1751522 gi|2080443</td><td>11667.m00017|LOC_Os01g01160|protein DnaJ domain, putative e_value:4.13564e-59</td><td>0.532</td><td>(0.2516 0.4729)</td><td>0.2516</td><td>0.4729</td></tr>
<tr><td>811</td><td>WT006_H18_CLUSTER_9689</td><td>gi|51535286|dbj|BAD38549.1| putative PrMC3 [Oryza sativa (japonica cultivar-group)] e_value:3.99528e-157</td><td>11681.m02665|LOC_Os09g28750|protein expressed protein e_value:1.20838e-95</td><td>0.574</td><td>(0.3644 0.6348)</td><td>0.3644</td><td>0.6348</td></tr>
<tr><td>812</td><td>WT005_E20_CLUSTER_29591</td><td>gi|50933693|ref|XP_476374.1| PsbQ domain protein family, putative-like protein [Oryza sativa (japonica cultivar-group)] >gnl|BL_ORD_ID|2367682 gi|50510151|dbj|BAD31119.1| PsbQ domain protein family, putative-like protein [Oryza sativa (japonica cultiva</td><td>11673.m00051|LOC_Os07g01480|protein Oxygen evolving enhancer protein 3 (PsbQ) e_value:5.0042e-73</td><td>0.562</td><td>(0.2718 0.4837)</td><td>0.2718</td><td>0.4837</td></tr>
<tr><td>813</td><td>WT004_P19_CLUSTER_14636</td><td>gi|50908933|ref|XP_465955.1| putative nodulin 3 [Oryza sativa (japonica cultivar-group)] >gnl|BL_ORD_ID|1576536 gi|48716574|dbj|BAD23245.1| putative nodulin 3 [Oryza sativa (japonica cultivar-group)] e_value:1.11994e-122</td><td>11668.m02943|LOC_Os02g30910|protein N3 like protein e_value:2.57746e-124</td><td>0.351</td><td>(0.1617 0.4614)</td><td>0.1617</td><td>0.4614</td></tr>
<tr><td>814</td><td>WT004_O11_CLUSTER_9881</td><td>gi|22090446|emb|CAD36515.1| putative beta-glycosidase [Oryza sativa (japonica cultivar-group)] e_value:0</td><td>11687.m04325|LOC_Os11g45710|protein Glycosyl hydrolase family 1, putative e_value:0</td><td>0.189</td><td>(0.0841 0.4448)</td><td>0.0841</td><td>0.4448</td></tr>
<tr><td>815</td><td>WT004_N13_CLUSTER_22325</td><td>gi|50931439|ref|XP_475247.1| putative glycogenin [Oryza sativa (japonica cultivar-group)] >gnl|BL_ORD_ID|1595670 gi|46391126|gb|AAS90653.1| putative glycogenin [Oryza sativa (japonica cultivar-group)] e_value:0</td><td>11682.m03325|LOC_Os05g35200|protein Glycosyl transferase family 8, putative e_value:0</td><td>0.138</td><td>(0.0826 0.5995)</td><td>0.0826</td><td>0.5995</td></tr>
<tr><td>816</td><td>WT004_I13_CLUSTER_6377</td><td>gi|4894912|gb|AAD32651.1| nicotianamine synthase 2 [Hordeum vulgare] >gnl|BL_ORD_ID|719459 gi|27151636|sp|Q9XFB7|NAS9_HORVU Nicotianamine synthase 9 (S-adenosyl-L-methionine:S-adenosyl-L-methionine:S-adenosyl-methionine 3-amino-3-carboxypropyltransfera</td><td>11673.m04872|LOC_Os07g48980|protein probable nicotianamine synthase 3 (ec 2.5.1.43) (s-adenosyl-l-methionine:s-adenosyl-l-methionine:s-adenosyl-methion ine 3-amino-3-carboxypropyltransferase 3) (osnas3) e_value:2.5022e-131</td><td>0.491</td><td>(0.1598 0.3259)</td><td>0.1598</td><td>0.3259</td></tr>
<tr><td>817</td><td>WT003_H24_CLUSTER_10577</td><td>gi|50905973|ref|XP_464475.1| putative seven transmembrane protein Mlo7 [Oryza sativa (japonica cultivar-group)] >gnl|BL_ORD_ID|1566723 gi|49388153|dbj|BAD25281.1| putative seven transmembrane protein Mlo7 [Oryza sativa (japonica cultivar-</td><td>11668.m00961|LOC_Os02g10350|protein Mlo family e_value:0</td><td>0.28</td><td>(0.1376 0.4916)</td><td>0.1376</td><td>0.4916</td></tr>
<tr><td>818</td><td>WT002_K06_CLUSTER_23757</td><td>gi|6729509|emb|CAB67665.1| diaminopimelate epimerase-like protein [Arabidopsis thaliana] >gnl|BL_ORD_ID|1451060 gi|23505901|gb|AAN28810.1| At3g53580/F4P12_280 [Arabidopsis thaliana] >gnl|BL_ORD_ID|1451060 gi|22022530|gb|AAM83223.1| AT3g53580/F4P12_2</td><td>11686.m03739|LOC_Os12g37960|protein diaminopimelate epimerase e_value:3.99083e-180</td><td>0.127</td><td>(0.0651 0.5141)</td><td>0.0651</td><td>0.5141</td></tr>
<tr><td>819</td><td>WT002_E05_CLUSTER_671</td><td>gi|51979693|ref|XP_507593.1| PREDICTED B1168A08.31 gene product [Oryza sativa (japonica cultivar-group)] >gnl|BL_ORD_ID|1609570 gi|50947511|ref|XP_483283.1| putative beta-glucosidase isozyme 2 precursor [Oryza sativa (japonica cultivar-group)] >gnl|</td><td>11674.m03995|LOC_Os08g39860|protein Glycosyl hydrolase family 1 e_value:0</td><td>0.285</td><td>(0.1990 0.6976)</td><td>0.199</td><td>0.6976</td></tr>
<tr><td>820</td><td>SET5_I11_CLUSTER_19609</td><td>gi|21593865|gb|AAM65832.1| putative riboflavin synthase alpha chain [Arabidopsis thaliana] e_value:6.57988e-58</td><td>11686.m03486|LOC_Os12g35580|protein riboflavin synthase, alpha subunit, putative e_value:2.33622e-124</td><td>0.211</td><td>(0.1245 0.5891)</td><td>0.1245</td><td>0.5891</td></tr>
<tr><td>821</td><td>SET5_B14_CLUSTER_12699</td><td>gi|55773757|dbj|BAD72440.1| putative glutathione peroxidase [Oryza sativa (japonica cultivar-group)] e_value:3.63224e-92</td><td>11680.m00822|LOC_Os06g08670|protein phospholipid hydroperoxide glutathione peroxidase, chloroplastprecursor (ec 1.11.1.12) (phgpx) e_value:7.17595e-96</td><td>0.28</td><td>(0.1310 0.4679)</td><td>0.131</td><td>0.4679</td></tr>
<tr><td>822</td><td>SET4_D14_CLUSTER_27108</td><td>gi|21555410|gb|AAM63852.1| unknown [Arabidopsis thaliana] >gi|19310697|gb|AAL85079.1| unknown protein [Arabidopsis thaliana] >gi|15292669|gb|AAK92703.1| unknown protein [Arabidopsis thaliana] >gi|9758248|dbj|BAB08747.1| unnamed protein product [A</td><td>11687.m02991|LOC_Os11g32500|protein Rubredoxin, putative e_value:5.43267e-54</td><td>0.306</td><td>(0.1812 0.5929)</td><td>0.1812</td><td>0.5929</td></tr>
<tr><td>823</td><td>SET4_A04_CLUSTER_24617</td><td>gi|57899740|dbj|BAD87460.1| unknown protein [Oryza sativa (japonica cultivar-group)] >gi|57899103|dbj|BAD86922.1| unknown protein [Oryza sativa (japonica cultivar-group)] e_value:1.09871e-22</td><td>11667.m05174|LOC_Os01g52730|protein expressed protein e_value:8.12246e-25</td><td>0.797</td><td>(0.3005 0.3769)</td><td>0.3005</td><td>0.3769</td></tr>
<tr><td>824</td><td>SET1_B03_CLUSTER_4534</td><td>gi|52075930|dbj|BAD45876.1| putative peptidyl-prolyl cis-trans isomerase [Oryza sativa (japonica cultivar-group)] e_value:1.43711e-78</td><td>11680.m04463|LOC_Os06g45340|protein Similar to FK506 binding protein 1 e_value:6.36965e-79</td><td>0.395</td><td>(0.1650 0.4173)</td><td>0.165</td><td>0.4173</td></tr>
<tr><td>825</td><td>WT011__C04_CLUSTER_33145</td><td>gi|38345217|emb|CAD40200.2| OSJNBb0043H09.9 [Oryza sativa (japonica cultivar-group)] >gnl|BL_ORD_ID|1522065 gi|50921829|ref|XP_471275.1| OSJNBb0043H09.9 [Oryza sativa (japonica cultivar-group)] e_value:9.93876e-142</td><td>11670.m01528|LOC_Os04g17050|protein hypothetical protein e_value:2.5939e-143</td><td>0.172</td><td>(0.0890 0.5179)</td><td>0.089</td><td>0.5179</td></tr>
<tr><td>826</td><td>WT008_H07_CLUSTER_8390</td><td>gi|38569182|emb|CAD40835.2| OSJNBa0086B14.7 [Oryza sativa (japonica cultivar-group)] >gnl|BL_ORD_ID|1524037 gi|50924616|ref|XP_472665.1| OSJNBa0086B14.7 [Oryza sativa (japonica cultivar-group)] e_value:4.38797e-40</td><td>11670.m03568|LOC_Os04g37760|protein Expressed protein e_value:9.32872e-42</td><td>0.533</td><td>(0.3180 0.5962)</td><td>0.318</td><td>0.5962</td></tr>
<tr><td>827</td><td>WT007_K16_CLUSTER_5477</td><td>gi|50909443|ref|XP_466210.1| putative T-complex protein 11 [Oryza sativa (japonica cultivar-group)] >gnl|BL_ORD_ID|1576330 gi|46389863|dbj|BAD15464.1| putative T-complex protein 11 [Oryza sativa (japonica cultivar-group)] e_value:0</td><td>11668.m03325|LOC_Os02g35080|protein hypothetical protein e_value:0</td><td>0.325</td><td>(0.1611 0.4964)</td><td>0.1611</td><td>0.4964</td></tr>
<tr><td>828</td><td>WT004_M13_CLUSTER_34643</td><td>gi|50919283|ref|XP_470038.1| unknown protein [Oryza sativa (japonica cultivar-group)] >gnl|BL_ORD_ID|1588214 gi|30103018|gb|AAP21431.1| unknown protein [Oryza sativa (japonica cultivar-group)] e_value:3.00427e-78</td><td>11669.m06024|LOC_Os03g59320|protein expressed protein e_value:5.98703e-80</td><td>0.261</td><td>(0.1591 0.6098)</td><td>0.1591</td><td>0.6098</td></tr>
<tr><td>829</td><td>WT012_D10_CLUSTER_32605</td><td>gi|37700321|gb|AAR00611.1| putative aldo/keto reductase family protein [Oryza sativa (japonica cultivar-group)] >gnl|BL_ORD_ID|2260676 gi|50901464|ref|XP_463165.1| putative aldo/keto reductase family protein [Oryza sativa (japonica cultivar-</td><td>11669.m04113|LOC_Os03g41510|protein putative aldo/keto reductase family protein e_value:0</td><td>0.129</td><td>(0.0775 0.6029)</td><td>0.0775</td><td>0.6029</td></tr>
<tr><td>830</td><td>WT007_L15_CLUSTER_25202</td><td>gi|50932843|ref|XP_475949.1| putative polyprenyl diphosphate synthase [Oryza sativa (japonica cultivar-group)] >gnl|BL_ORD_ID|1599523 gi|48475134|gb|AAT44203.1| putative polyprenyl diphosphate synthase [Oryza sativa (japonica cultivar-group)] >gnl|B</td><td>11686.m01710|LOC_Os12g17320|protein Polyprenyl synthetase, putative e_value:2.70683e-167</td><td>0.103</td><td>(0.1289 1.2545)</td><td>0.1289</td><td>1.2545</td></tr>
<tr><td>831</td><td>WT012_A12_CLUSTER_21975</td><td>gi|50252065|dbj|BAD27995.1| putative ribosomal RNA apurinic site specific lyase [Oryza sativa (japonica cultivar-group)] >gnl|BL_ORD_ID|229093 gi|50252062|dbj|BAD27993.1| putative ribosomal RNA apurinic site specific lyase [Oryza sativa (japonica culti</td><td>11668.m00785|LOC_Os02g08160|protein F-box domain, putative e_value:1.71734e-151</td><td>0.637</td><td>(0.3022 0.4744)</td><td>0.3022</td><td>0.4744</td></tr>
<tr><td>832</td><td>WT003_G07_CLUSTER_13236</td><td>gi|50725202|dbj|BAD33953.1| putative carbonic anhydrase [Oryza sativa (japonica cultivar-group)] e_value:1.13108e-123</td><td>11681.m02684|LOC_Os09g28910|protein carbonate dehydratase, putative e_value:1.39154e-136</td><td>0.228</td><td>(0.1460 0.6396)</td><td>0.146</td><td>0.6396</td></tr>
<tr><td>833</td><td>WT004_M18_CLUSTER_33545</td><td>gi|50900234|ref|XP_450905.1| putative annexin [Oryza sativa (japonica cultivar-group)] >gnl|BL_ORD_ID|1564228 gi|51963772|ref|XP_506666.1| PREDICTED B1339H09.19 gene product [Oryza sativa (japonica cultivar-group)] >gnl|BL_ORD_ID|1564228 gi|49389211</td><td>11681.m02069|LOC_Os09g23160|protein annexin e_value:5.17961e-136</td><td>0.215</td><td>(0.1313 0.6120)</td><td>0.1313</td><td>0.612</td></tr>
<tr><td>834</td><td>WT007_P16_CLUSTER_21333</td><td>gi|52077176|dbj|BAD46221.1| putative genetic modifier [Oryza sativa (japonica cultivar-group)] e_value:1.00553e-160</td><td>11681.m03561|LOC_Os09g39560|protein HAD-superfamily hydrolase, subfamily IA, variant 3, putative e_value:2.46832e-162</td><td>0.117</td><td>(0.1509 1.2871)</td><td>0.1509</td><td>1.2871</td></tr>
<tr><td>835</td><td>SET4_H15_CLUSTER_2912</td><td>gi|50947037|ref|XP_483046.1| putative CfxY protein [Oryza sativa (japonica cultivar-group)] >gi|42408179|dbj|BAD09316.1| putative CfxY protein [Oryza sativa (japonica cultivar-group)] e_value:1.63397e-140</td><td>11674.m03779|LOC_Os08g37940|protein HAD-superfamily hydrolase, subfamily IA, variant 3, putative e_value:3.74559e-142</td><td>0.254</td><td>(0.1291 0.5088)</td><td>0.1291</td><td>0.5088</td></tr>
<tr><td>836</td><td>WT007_O13_CLUSTER_7520</td><td>gi|53370691|gb|AAU89186.1| expressed protein [Oryza sativa (japonica cultivar-group)] e_value:0</td><td>11669.m04451|LOC_Os03g44670|protein expressed protein</td><td>0.204</td><td>(0.1130 0.5530)</td><td>0.113</td><td>0.553</td></tr>
<tr><td>837</td><td>SET1_M02_CLUSTER_15491</td><td>gi|34903780|ref|NP_913237.1| unnamed protein product [Oryza sativa (japonica cultivar-group)] >gi|20804455|dbj|BAB92151.1| putative CBL-interacting protein kinase 2 [Oryza sativa (japonica cultivar-group)] >gi|7340882|dbj|BAA92972.1| putative CBL-in</td><td>11667.m01058|LOC_Os01g10890|protein Protein kinase domain, putative e_value:0</td><td>0.176</td><td>(0.0768 0.4366)</td><td>0.0768</td><td>0.4366</td></tr>
<tr><td>838</td><td>SET6_O13_CLUSTER_15350</td><td>gi|50926612|ref|XP_473253.1| OSJNBa0038O10.25 [Oryza sativa (japonica cultivar-group)] >gi|21742078|emb|CAD41189.1| OSJNBa0074L08.1 [Oryza sativa (japonica cultivar-group)] >gi|38344453|emb|CAE05659.2| OSJNBa0038O10.25 [Oryza sativa (japonica cultiv</td><td>11670.m04323|LOC_Os04g44600|protein expressed protein e_value:2.14375e-36</td><td>0.779</td><td>(0.3037 0.3897)</td><td>0.3037</td><td>0.3897</td></tr>
<tr><td>839</td><td>WT011__C24_CLUSTER_34584</td><td>gi|50919809|ref|XP_470265.1| Putative protein kinase [Oryza sativa (japonica cultivar-group)] >gnl|BL_ORD_ID|1539325 gi|22773239|gb|AAN06845.1| Putative protein kinase [Oryza sativa (japonica cultivar-group)] e_value:4.78236e-176</td><td>11669.m00585|LOC_Os03g06330|protein Protein kinase domain, putative e_value:0</td><td>0.066</td><td>(0.0649 0.9781)</td><td>0.0649</td><td>0.9781</td></tr>
<tr><td>840</td><td>WT005_M21_CLUSTER_14200</td><td>gi|50945735|ref|XP_482395.1| unknown protein [Oryza sativa (japonica cultivar-group)] >gnl|BL_ORD_ID|1606310 gi|37806205|dbj|BAC99708.1| unknown protein [Oryza sativa (japonica cultivar-group)] e_value:1.79445e-39</td><td>11681.m01892|LOC_Os09g20930|protein hypothetical protein e_value:1.89958e-43</td><td>0.48</td><td>(0.2439 0.5078)</td><td>0.2439</td><td>0.5078</td></tr>
<tr><td>841</td><td>WT003_K22_CLUSTER_10892</td><td>gi|20259017|gb|AAM14224.1| unknown protein [Arabidopsis thaliana] >gnl|BL_ORD_ID|1208057 gi|17380816|gb|AAL36095.1| unknown protein [Arabidopsis thaliana] >gnl|BL_ORD_ID|1208057 gi|9758109|dbj|BAB08581.1| unnamed protein product [Arabidopsis thalian</td><td>11686.m00841|LOC_Os12g08810|protein expressed protein e_value:0</td><td>0.102</td><td>(0.1067 1.0439)</td><td>0.1067</td><td>1.0439</td></tr>
<tr><td>842</td><td>SET1_J15_CLUSTER_34800</td><td>gi|21853|emb|CAA44342.1| rab protein [Triticum aestivum] >gi|100800|pir||S17367 rab15B protein - wheat e_value:2.1177e-139</td><td>11687.m02411|LOC_Os11g26790|protein probable dehydrin - rice e_value:5.43852e-43</td><td>0.356</td><td>(0.1086 0.3049)</td><td>0.1086</td><td>0.3049</td></tr>
<tr><td>843</td><td>WT006_K02_CLUSTER_20893</td><td>gi|50921413|ref|XP_471067.1| OSJNBa0020P07.12 [Oryza sativa (japonica cultivar-group)] >gnl|BL_ORD_ID|1589610 gi|38344869|emb|CAE01295.2| OSJNBa0020P07.12 [Oryza sativa (japonica cultivar-group)] e_value:0</td><td>11670.m00197|LOC_Os04g02910|protein expressed protein e_value:0</td><td>0.189</td><td>(0.1369 0.7233)</td><td>0.1369</td><td>0.7233</td></tr>
<tr><td>844</td><td>SET3_H02_CLUSTER_8686</td><td>gi|50937261|ref|XP_478158.1| 2Fe-2S iron-sulfur cluster protein-like [Oryza sativa (japonica cultivar-group)] >gi|33146986|dbj|BAC80058.1| 2Fe-2S iron-sulfur cluster protein-like [Oryza sativa (japonica cultivar-group)] e_value:1.63109e-69</td><td>11673.m02918|LOC_Os07g30670|protein 2Fe-2S iron-sulfur cluster binding domain, putative e_value:2.70485e-71</td><td>0.418</td><td>(0.2210 0.5289)</td><td>0.221</td><td>0.5289</td></tr>
<tr><td>845</td><td>WT011_J11_CLUSTER_19593</td><td>gi|31433511|gb|AAP55016.1| putative monosaccharide transporter [Oryza sativa (japonica cultivar-group)] >gnl|BL_ORD_ID|2013040 gi|37536854|ref|NP_922729.1| putative monosaccharide transporter [Oryza sativa (japonica cultivar-group)] >gnl|BL_ORD_ID|2</td><td>11669.m00018|LOC_Os03g01170|protein Sugar transporter e_value:0</td><td>0.163</td><td>(0.1304 0.8008)</td><td>0.1304</td><td>0.8008</td></tr>
<tr><td>846</td><td>WT003_D18_CLUSTER_7286</td><td>gi|50253330|dbj|BAD29598.1| cell division inhibitor-like [Oryza sativa (japonica cultivar-group)] e_value:4.75113e-144</td><td>11668.m05834|LOC_Os02g58790|protein SulA protein e_value:1.50835e-145</td><td>0.15</td><td>(0.1777 1.1821)</td><td>0.1777</td><td>1.1821</td></tr>
<tr><td>847</td><td>SET5_F11_CLUSTER_31499</td><td>gi|52548252|gb|AAU82113.1| histone deacetylase [Triticum aestivum] e_value:0</td><td>11680.m03745|LOC_Os06g38470|protein Histone deacetylase family, putative e_value:4.18456e-167</td><td>0.253</td><td>(0.3135 1.2375)</td><td>0.3135</td><td>1.2375</td></tr>
<tr><td>848</td><td>WT012_P12_CLUSTER_34016</td><td>gi|31431946|gb|AAP53652.1| putative Mic1 homolog [Oryza sativa (japonica cultivar-group)] >gnl|BL_ORD_ID|2011097 gi|37534126|ref|NP_921365.1| putative Mic1 homolog [Oryza sativa (japonica cultivar-group)] >gnl|BL_ORD_ID|2011097 gi|13940621|gb|AAK504</td><td>11676.m02234|LOC_Os10g26140|protein Colon cancer-associated protein Mic1, putative e_value:0</td><td>0.17</td><td>(0.0932 0.5478)</td><td>0.0932</td><td>0.5478</td></tr>
<tr><td>849</td><td>WT003_B05_CLUSTER_33585</td><td>gi|34902148|ref|NP_912420.1| Putative NAM (no apical meristem) protein [Oryza sativa (japonica cultivar-group)] >gnl|BL_ORD_ID|1588859 gi|24899396|gb|AAN64996.1| Putative NAM (no apical meristem) protein [Oryza sativa (japonica cultivar-group)]</td><td>11669.m02152|LOC_Os03g21030|protein No apical meristem (NAM) protein, putative e_value:1.47604e-139</td><td>0.382</td><td>(0.1317 0.3447)</td><td>0.1317</td><td>0.3447</td></tr>
<tr><td>850</td><td>WT004_O22_CLUSTER_9888</td><td>gi|53792621|dbj|BAD53635.1| F-box family protein-like [Oryza sativa (japonica cultivar-group)] e_value:2.5392e-164</td><td>11680.m04921|LOC_Os06g49530|protein F-box family protein, putative e_value:6.29964e-166</td><td>0.17</td><td>(0.1281 0.7536)</td><td>0.1281</td><td>0.7536</td></tr>
</table>

PDF Complete
Your complimentary use period has ended. Thank you for using PDF Complete.
Click Here to upgrade to Unlimited Pages and Expanded Features

851	WT013_B11_CLUSTER_4958	gi|53791784|dbj|BAD53578.1| putative brain and reproductive organ-expressed protein [Oryza sativa (japonica cultivar-group)] e_value:1.41165e-175	11680.m04998|LOC_Os06g50170|protein brain and reproductive organ-expressed protein isoform IV, putative e_value:3.58631e-	0.243	(0.1337 0.5512)	0.1337	0.5512
852	WT004__C22_CLUSTER_4019	gi|34904492|ref|NP_913593.1| putative receptor protein kinase [Oryza sativa (japonica cultivar-group)] >gnl|BL_ORD_ID|1751864 gi|13486864|dbj|BAB40094.1| putative leucine-rich receptor-like protein kinase [Oryza sativa (japonica cultivar-group)] e_valu	11667.m01363|LOC_Os01g13800|protein Leucine Rich Repeat, putative e_value:0	0.212	(0.1086 0.5123)	0.1086	0.5123
853	SET4_E15_CLUSTER_27142	gi|50251961|dbj|BAD27896.1| putative phosphoribosylanthranilate isomerase 1 (PAI1) [Oryza sativa (japonica cultivar-group)] e_value:3.42065e-95	11668.m01566|LOC_Os02g16630|protein phosphoribosylanthranilate isomerase e_value:1.47067e-96	0.215	(0.2296 1.0682)	0.2296	1.0682
854	WT012__C04_CLUSTER_32890	gi|55770293|ref|XP_550136.1| unknown protein [Oryza sativa (japonica cultivar-group)] >gnl|BL_ORD_ID|1753518 gi|54290395|dbj|BAD61265.1| unknown protein [Oryza sativa (japonica cultivar-group)] >gnl|BL_ORD_ID|1753518 gi|54290318|dbj|BAD61122.1| unkn	11667.m00469|LOC_Os01g05420|protein IWS1 C-terminus, putative e_value:0	0.17	(0.0856 0.5049)	0.0856	0.5049
855	SET5_D19_CLUSTER_34723	gi|7452979|emb|CAB86383.1| allene oxide synthase [Hordeum vulgare subsp. vulgare] e_value:0	11669.m01211|LOC_Os03g12500|protein expressed protein	0.454	(0.1266 0.2789)	0.1266	0.2789
856	WT009_K16_CLUSTER_3260	gi|55296125|dbj|BAD67843.1| putative prolyl aminopeptidase [Oryza sativa (japonica cultivar-group)] e_value:0	11680.m00628|LOC_Os06g06820|protein Similar to prolyl aminopeptidase-like protein e_value:0	0.155	(0.0753 0.4858)	0.0753	0.4858
857	WT004_H17_CLUSTER_4821	gi|57900228|dbj|BAD88333.1| peptidylprolyl cis-trans isomerase-like protein [Oryza sativa (japonica cultivar-group)] e_value:0	11667.m02587|LOC_Os01g26940|protein transposon protein, putative, CACTA, En/Spm sub-class e_value:0	0.286	(0.1712 0.5983)	0.1712	0.5983
858	WT011_I02_CLUSTER_25903	gi|50947659|ref|XP_483357.1| putative stress related-like protein interactor [Oryza sativa (japonica cultivar-group)] >gnl|BL_ORD_ID|1609683 gi|42408514|dbj|BAD09693.1| putative stress related-like protein interactor [Oryza sativa (japonica cultivar-gr	11674.m04093|LOC_Os08g40790|protein expressed protein e_value:0	0.275	(0.1375 0.4999)	0.1375	0.4999
859	WT004_D02_CLUSTER_1590	gi|11527004|gb|AAG36871.1| protein kinase CK2 regulatory subunit CK2B3 [Zea mays] e_value:1.19739e-148	11676.m03823|LOC_Os10g41520|protein putative casein kinase II beta subunit e_value:3.24128e-125	0.095	(0.1350 1.4270)	0.135	1.427
860	WT004_M23_CLUSTER_15169	gi|7188339|gb|AAF37734.1| 4-coumarate--CoA ligase 4CL3 [Lolium perenne] e_value:0	11668.m00775|LOC_Os02g08100|protein 4-coumarate coenzyme A ligase e_value:0	0.159	(0.0699 0.4410)	0.0699	0.441
861	WT006_N05_CLUSTER_14885	gi|32489525|emb|CAE04728.1| OSJNBa0043L24.16 [Oryza sativa (japonica cultivar-group)] >gnl|BL_ORD_ID|1523555 gi|50926340|ref|XP_473117.1| OSJNBa0043L24.16 [Oryza sativa (japonica cultivar-group)] e_value:0	11670.m04142|LOC_Os04g42920|protein isocitrate dehydrogenase, NADP-dependent e_value:0	0.11	(0.0455 0.4135)	0.0455	0.4135
862	SET3_O04_CLUSTER_16968	gi|50916082|ref|XP_468505.1| unknown protein [Oryza sativa (japonica cultivar-group)] >gi|48716450|dbj|BAD23057.1| unknown protein [Oryza sativa (japonica cultivar-group)] e_value:0	11668.m05761|LOC_Os02g58110|protein At1g17820 e_value:0	0.202	(0.1350 0.6695)	0.135	0.6695
863	WT010_G14_CLUSTER_21093	gi|55297698|dbj|BAD68288.1| unknown protein [Oryza sativa (japonica cultivar-group)] >gnl|BL_ORD_ID|270214 gi|55296321|dbj|BAD68139.1| unknown protein [Oryza sativa (japonica cultivar-group)] e_value:1.13705e-148	11667.m05825|LOC_Os01g58700|protein hypothetical protein e_value:3.62838e-149	0.19	(0.1447 0.7623)	0.1447	0.7623
864	WT007_K18_CLUSTER_30344	gi|50910661|ref|XP_466819.1| putative lysyl-tRNA synthetase [Oryza sativa (japonica cultivar-group)] >gnl|BL_ORD_ID|1580641 gi|48716234|dbj|BAD23770.1| putative lysyl-tRNA synthetase [Oryza sativa (japonica cultivar-group)] e_value:0	11668.m03952|LOC_Os02g41470|protein lysyl-tRNA synthetase e_value:0	0.125	(0.0492 0.3921)	0.0492	0.3921
865	WT012_I12_CLUSTER_33005	gi|57899212|dbj|BAD87361.1| lysine ketoglutarate reductase trans-splicing related 1-like [Oryza sativa (japonica cultivar-group)] e_value:0	11682.m00091|LOC_Os05g01760|protein expressed protein	0.076	(0.0501 0.6593)	0.0501	0.6593
866	SET2_O12_CLUSTER_34846	gi|31431324|gb|AAP53119.1| hypothetical protein [Oryza sativa (japonica cultivar-group)] >gi|37533060|ref|NP_920832.1| hypothetical protein [Oryza sativa (japonica cultivar-group)] >gi|15451592|gb|AAK98716.1| Hypothetical protein [Oryza sativa] e_va	11676.m01637|LOC_Os10g19880|protein expressed protein e_value:0	0.181	(0.1022 0.5634)	0.1022	0.5634
867	WT009_L02_CLUSTER_23940	gi|52353444|gb|AAU44012.1| unknown protein [Oryza sativa (japonica cultivar-group)] e_value:5.26068e-176	11682.m03618|LOC_Os05g38330|protein expressed protein e_value:1.07586e-162	0.24	(0.1293 0.5384)	0.1293	0.5384
868	WT008_H21_CLUSTER_27751	gi|62320618|dbj|BAD95271.1| hypothetical protein [Arabidopsis thaliana] >gnl|BL_ORD_ID|1160458 gi|45773954|gb|AAS76781.1| At1g08960 [Arabidopsis thaliana] >gnl|BL_ORD_ID|1160458 gi|25402504|pir||D86221 hypothetical protein [imported] - Arabidopsis t	11687.m00425|LOC_Os11g05070|protein Sodium/calcium exchanger protein, putative e_value:0	0.211	(0.1074 0.5083)	0.1074	0.5083
869	SET1_E19_CLUSTER_22866	gi|32129321|gb|AAP73848.1| putative cyclophilin [Oryza sativa (japonica cultivar-group)] >gi|50919309|ref|XP_470051.1| putative cyclophilin [Oryza sativa (japonica cultivar-group)] e_value:2.83499e-105	11669.m06065|LOC_Os03g59700|protein putative cyclophilin e_value:6.95685e-107	0.1	(0.0374 0.3732)	0.0374	0.3732
870	SET3_K02_CLUSTER_25934	gi|28393873|gb|AAO42344.1| unknown protein [Arabidopsis thaliana] >gi|27754314|gb|AAO22610.1| unknown protein [Arabidopsis thaliana] e_value:2.60962e-68	11669.m01434|LOC_Os03g14510|protein hypothetical protein e_value:1.81058e-99	0.128	(0.0886 0.6894)	0.0886	0.6894
871	WT011_O10_CLUSTER_30811	gi|56783742|dbj|BAD81154.1| unknown protein [Oryza sativa (japonica cultivar-group)] e_value:9.59035e-116	11667.m01717|LOC_Os01g17070|protein expressed protein e_value:2.77398e-117	0.313	(0.3791 1.2121)	0.3791	1.2121
872	WT006_P08_CLUSTER_28239	gi|50907635|ref|XP_465306.1| putative fruit protein [Oryza sativa (japonica cultivar-group)] >gnl|BL_ORD_ID|1569370 gi|51964028|ref|XP_506797.1| PREDICTED P0622F08.20 gene product [Oryza sativa (japonica cultivar-group)] >gnl|BL_ORD_ID|1569370 gi|46	11668.m02153|LOC_Os02g22260|protein probable oxidoreductase F9L1.8 - Arabidopsis thaliana e_value:2.79407e-101	0.262	(0.2076 0.7933)	0.2076	0.7933
873	WT012_B13_CLUSTER_11141	gi|7489667|pir||T06512 DNA-binding protein homolog HALF-1 - wheat >gnl|BL_ORD_ID|264592 gi|1304266|dbj|BAA10928.1| HALF-1 [Triticum aestivum] e_value:0	11673.m01036|LOC_Os07g10890|protein maize Em binding protein-1a e_value:2.75632e-166	0.182	(0.1052 0.5793)	0.1052	0.5793
874	WT006_M07_CLUSTER_34241	gi|50912703|ref|XP_467759.1| putative geranylgeranyl reductase [Oryza sativa (japonica cultivar-group)] >gnl|BL_ORD_ID|1582872 gi|51964368|ref|XP_506969.1| PREDICTED OJ1734_E02.38 gene product [Oryza sativa (japonica cultivar-group)] >gnl|BL_ORD_ID|	11668.m04986|LOC_Os02g51080|protein geranylgeranyl reductase e_value:0	0.216	(0.0535 0.2471)	0.0535	0.2471
875	WT005_B11_CLUSTER_4470	gi|28273408|gb|AAO38494.1| unknown protein [Oryza sativa (japonica cultivar-group)] e_value:9.12596e-53	11669.m04876|LOC_Os03g48620|protein hypothetical protein e_value:1.85548e-54	1.007	(0.3010 0.2989)	0.301	0.2989
876	WT011_P22_CLUSTER_32398	gi|7527353|dbj|BAA94257.1| endo-1,4-beta-glucanase Cel1 [Hordeum vulgare subsp. vulgare] e_value:0	11669.m05318|LOC_Os03g52630|protein putative endo-1,4-beta-glucanase e_value:0	0.102	(0.0530 0.5220)	0.053	0.522
877	SET1_K24_CLUSTER_20184	gi|34899264|ref|NP_910978.1| putative tubby related protein [Oryza sativa (japonica cultivar-group)] >gi|51963628|ref|XP_506548.1| PREDICTED P0450A04.117 gene product [Oryza sativa (japonica cultivar-group)] >gi|23495868|dbj|BAC20077.1| putative tub	11669.m02348|LOC_Os03g22800|protein Tub family, putative e_value:0	0.117	(0.0669 0.5700)	0.0669	0.57
878	WT005_B08_CLUSTER_4040	gi|50900462|ref|XP_462664.1| OSJNBa0064H22.16 [Oryza sativa (japonica cultivar-group)] >gnl|BL_ORD_ID|1564326 gi|38344835|emb|CAD40867.2| OSJNBa0064H22.16 [Oryza sativa (japonica cultivar-group)] e_value:0	11670.m03550|LOC_Os04g37620|protein Monooxygenase, putative e_value:0	0.244	(0.1360 0.5578)	0.136	0.5578
879	WT003_A09_CLUSTER_23950	gi|16209608|gb|AAL14249.1| glyoxalase II [Oryza sativa] >gnl|BL_ORD_ID|2281240 gi|50726249|dbj|BAD33825.1| glyoxalase II [Oryza sativa (japonica cultivar-group)] e_value:3.61164e-177	11681.m03091|LOC_Os09g34100|protein glyoxalase II e_value:9.1766e-179	0.119	(0.0512 0.4284)	0.0512	0.4284
880	WT007_B15_CLUSTER_2348	gi|50943697|ref|XP_481376.1| tetratricopeptide repeat protein-like [Oryza sativa (japonica cultivar-group)] e_value:1.5587e-159	11681.m03438|LOC_Os09g38390|protein PB1 domain, putative e_value:0	0.14	(0.0805 0.5756)	0.0805	0.5756
881	SET4_K12_CLUSTER_3748	gi|50908949|ref|XP_465963.1| unknown protein [Oryza sativa (japonica cultivar-group)] >gi|48716373|dbj|BAD22983.1| unknown protein [Oryza sativa (japonica cultivar-group)] e_value:1.21365e-41	11668.m03026|LOC_Os02g32190|protein hypothetical protein e_value:2.65785e-51	0.84	(0.7917 0.9426)	0.7917	0.9426
882	SET6_I20_CLUSTER_34224	gi|15236506|ref|NP_194077.1| beta-adaptin, putative [Arabidopsis thaliana] e_value:0	11669.m02421|LOC_Os03g23950|protein beta-adaptin homolog F8L21.170 - Arabidopsis thaliana e_value:0	0.056	(0.0294 0.5247)	0.0294	0.5247
883	WT007__C10_CLUSTER_11036	gi|50934323|ref|XP_476689.1| unknown protein [Oryza sativa (japonica cultivar-group)] >gnl|BL_ORD_ID|1521623 gi|34394888|dbj|BAC84337.1| unknown protein [Oryza sativa (japonica cultivar-group)] e_value:1.50377e-134	11673.m00569|LOC_Os07g06490|protein expressed protein e_value:3.70944e-136	0.382	(0.1862 0.4870)	0.1862	0.487
884	WT004_A05_CLUSTER_11377	gi|50921085|ref|XP_470903.1| hypothetical protein [Oryza sativa (japonica cultivar-group)] >gnl|BL_ORD_ID|1588873 gi|29788808|gb|AAP03354.1| hypothetical protein [Oryza sativa (japonica cultivar-group)] e_value:1.38069e-134	11686.m02340|LOC_Os12g24390|protein von Willebrand factor type A domain, putative e_value:2.62453e-168	0.54	(0.2845 0.5271)	0.2845	0.5271
885	WT010_F09_CLUSTER_23140	gi|50929689|ref|XP_474372.1| OSJNBb0017I01.13 [Oryza sativa (japonica cultivar-group)] >gnl|BL_ORD_ID|1594553 gi|32487399|emb|CAE05733.1| OSJNBb0017I01.13 [Oryza sativa (japonica cultivar-group)] e_value:3.50939e-87	11670.m05776|LOC_Os04g58330|protein expressed protein e_value:5.81024e-89	0.27	(0.1477 0.5474)	0.1477	0.5474
886	WT010_E10_CLUSTER_17106	gi|31431627|gb|AAP53378.1| putative disease resistance protein [Oryza sativa (japonica cultivar-group)] >gnl|BL_ORD_ID|2010508 gi|37533578|ref|NP_921091.1| putative disease resistance protein [Oryza sativa (japonica cultivar-group)] >gnl|BL_ORD_ID|2	11676.m01910|LOC_Os10g22510|protein NB-ARC domain, putative e_value:7.53228e-109	0.457	(0.6473 1.4153)	0.6473	1.4153
887	WT010__C11_CLUSTER_29865	gi|55770537|ref|XP_550259.1| unknown protein [Oryza sativa (japonica cultivar-group)] >gnl|BL_ORD_ID|1753655 gi|55297508|dbj|BAD68310.1| unknown protein [Oryza sativa (japonica cultivar-group)] e_value:0	11667.m00675|LOC_Os01g07390|protein Zinc finger, C3HC4 type (RING finger), putative e_value:0	0.16	(0.0934 0.5834)	0.0934	0.5834
888	WT009_L09_CLUSTER_15133	gi|18075958|emb|CAD12836.1| diphosphonucleotide phosphatase 1 [Lupinus luteus] e_value:0	11686.m03825|LOC_Os12g38750|protein diphosphonucleotide phosphatase 1 e_value:0	0.089	(0.0643 0.7207)	0.0643	0.7207
889	WT009_F11_CLUSTER_13501	gi|50947417|ref|XP_483236.1| unknown protein [Oryza sativa (japonica cultivar-group)] >gnl|BL_ORD_ID|1609487 gi|42408912|dbj|BAD10169.1| unknown protein [Oryza sativa (japonica cultivar-group)] >gnl|BL_ORD_ID|1609487 gi|42407683|dbj|BAD08832.1| unkn	11681.m02885|LOC_Os09g31230|protein hypothetical protein e_value:0	0.3	(0.1762 0.5883)	0.1762	0.5883
890	WT008_I01_CLUSTER_20990	gi|37999996|gb|AAR07083.1| unknown protein [Oryza sativa (japonica cultivar-group)] >gnl|BL_ORD_ID|2266078 gi|50918477|ref|XP_469635.1| unknown protein [Oryza sativa (japonica cultivar-group)] >gnl|BL_ORD_ID|2266078 gi|29788868|gb|AAP03414.1| unknow	11669.m05322|LOC_Os03g52660|protein expressed protein e_value:1.31791e-56	0.728	(0.2627 0.3610)	0.2627	0.361
891	WT008_B09_CLUSTER_408	gi|10178282|emb|CAC08340.1| putative protein [Arabidopsis thaliana] e_value:4.69358e-105	11668.m05762|LOC_Os02g58120|protein expressed protein e_value:7.05602e-129	0.179	(0.1083 0.6064)	0.1083	0.6064
892	WT007_I22_CLUSTER_8419	gi|50910197|ref|XP_466587.1| ankyrin repeat-like protein [Oryza sativa (japonica cultivar-group)] >gnl|BL_ORD_ID|1577936 gi|47848298|dbj|BAD22162.1| ankyrin repeat-like protein [Oryza sativa (japonica cultivar-group)] >gnl|BL_ORD_ID|1577936 gi|47497	11682.m02138|LOC_Os05g23320|protein expressed protein e_value:0	0.191	(0.1109 0.5803)	0.1109	0.5803
893	WT006_P02_CLUSTER_15632	gi|17978590|gb|AAL48316.1| adenylosuccinate-AMP lyase [Vigna unguiculata] e_value:0	11669.m02028|LOC_Os03g19930|protein adenylosuccinate lyase e_value:0	0.196	(0.1204 0.6144)	0.1204	0.6144

PDF Complete
Your complimentary use period has ended. Thank you for using PDF Complete.
Click Here to upgrade to Unlimited Pages and Expanded Features

<table>
<tr><td>894</td><td>WT006_L05_CLUSTER_28715</td><td>gi|50947001|ref|XP_483028.1| proline-rich protein family-like [Oryza sativa (japonica cultivar-group)] >gnl|BL_ORD_ID|1609263 gi|42409399|dbj|BAD10712.1| proline-rich protein family-like [Oryza sativa (japonica cultivar-group)] e_value:4.07027e-92</td><td>11674.m03765|LOC_Os08g37810|protein expressed protein e_value:5.24182e-93</td><td>0.481</td><td>(0.2835 0.5898)</td><td>0.2835</td><td>0.5898</td></tr>
<tr><td>895</td><td>WT006_J15_CLUSTER_29549</td><td>gi|57899942|dbj|BAD87854.1| putative ZR1 protein [Oryza sativa (japonica cultivar-group)] e_value:0</td><td>11682.m02975|LOC_Os05g31920|protein Regulator of chromosome condensation (RCC1), putative e_value:0</td><td>0.097</td><td>(0.0438 0.4505)</td><td>0.0438</td><td>0.4505</td></tr>
<tr><td>896</td><td>WT005_D02_CLUSTER_28342</td><td>gi|21655154|gb|AAL99264.1| CONSTANS-like protein CO5 [Hordeum vulgare subsp. vulgare] e_value:2.93655e-174</td><td>11668.m03810|LOC_Os02g39710|protein CCT motif, putative e_value:6.26087e-129</td><td>0.337</td><td>(0.1378 0.4085)</td><td>0.1378</td><td>0.4085</td></tr>
<tr><td>897</td><td>WT003_D16_CLUSTER_12937</td><td>gi|32488496|emb|CAE03248.1| OSJNBa0011J08.3 [Oryza sativa (japonica cultivar-group)] >gnl|BL_ORD_ID|1524060 gi|50928159|ref|XP_473607.1| OSJNBa0011J08.3 [Oryza sativa (japonica cultivar-group)] e_value:2.91547e-54</td><td>11670.m04798|LOC_Os04g48840|protein Similar to protein kinase, putative e_value:1.62742e-58</td><td>0.265</td><td>(0.1473 0.5551)</td><td>0.1473</td><td>0.5551</td></tr>
<tr><td>898</td><td>WT003_D03_CLUSTER_21775</td><td>gi|50924234|ref|XP_472477.1| OSJNBa0028I23.20 [Oryza sativa (japonica cultivar-group)] >gnl|BL_ORD_ID|1591454 gi|39546213|emb|CAE04638.3| OSJNBa0028I23.20 [Oryza sativa (japonica cultivar-group)] e_value:0</td><td>11670.m03319|LOC_Os04g34450|protein exocyst complex component sec5 e_value:0</td><td>0.135</td><td>(0.0635 0.4690)</td><td>0.0635</td><td>0.469</td></tr>
<tr><td>899</td><td>WT002_J09_CLUSTER_32227</td><td>gi|50933711|ref|XP_476383.1| disease resistance response protein-like protein [Oryza sativa (japonica cultivar-group)] >gnl|BL_ORD_ID|1518530 gi|34394264|dbj|BAC84745.1| disease resistance response protein-like protein [Oryza sativa (japonica cultivar-</td><td>11687.m03989|LOC_Os11g42500|protein Dirigent-like protein e_value:2.89375e-59</td><td>0.399</td><td>(0.1991 0.4984)</td><td>0.1991</td><td>0.4984</td></tr>
<tr><td>900</td><td>SET5_O04_CLUSTER_13987</td><td>gi|56202134|dbj|BAD73467.1| hypothetical protein [Oryza sativa (japonica cultivar-group)] e_value:0</td><td>11667.m04716|LOC_Os01g48620|protein expressed protein</td><td>0.237</td><td>(0.3689 1.5562)</td><td>0.3689</td><td>1.5562</td></tr>
<tr><td>901</td><td>SET5_N15_CLUSTER_6726</td><td>gi|51535631|dbj|BAD37605.1| methyltransferase-like [Oryza sativa (japonica cultivar-group)] >gi|51535533|dbj|BAD37452.1| methyltransferase-like [Oryza sativa (japonica cultivar-group)] e_value:1.38709e-157</td><td>11680.m04303|LOC_Os06g43800|protein expressed protein e_value:3.83117e-159</td><td>0.147</td><td>(0.1075 0.7295)</td><td>0.1075</td><td>0.7295</td></tr>
<tr><td>902</td><td>SET5_D13_CLUSTER_3102</td><td>gi|29294046|gb|AAO73883.1| DnaJ domain family [Arabidopsis thaliana] >gi|30688196|ref|NP_850862.1| DNAJ heat shock N-terminal domain-containing protein [Arabidopsis thaliana] e_value:2.4863e-43</td><td>11686.m03059|LOC_Os12g31460|protein Similar to DnaJ domain family e_value:9.22564e-96</td><td>0.163</td><td>(0.1153 0.7051)</td><td>0.1153</td><td>0.7051</td></tr>
<tr><td>903</td><td>SET5_C12_CLUSTER_19834</td><td>gi|50910123|ref|XP_466550.1| unknown protein [Oryza sativa (japonica cultivar-group)] >gi|47847635|dbj|BAD22121.1| unknown protein [Oryza sativa (japonica cultivar-group)] e_value:0</td><td>11668.m03678|LOC_Os02g38430|protein expressed protein e_value:0</td><td>0.155</td><td>(0.0888 0.5732)</td><td>0.0888</td><td>0.5732</td></tr>
<tr><td>904</td><td>SET4_B02_CLUSTER_10723</td><td>gi|50251643|dbj|BAD29646.1| putative ras-related GTP-binding protein [Oryza sativa (japonica cultivar-group)] e_value:5.97821e-110</td><td>11681.m00930|LOC_Os09g10940|protein GTP-binding protein. e_value:2.72734e-100</td><td>0.156</td><td>(0.1287 0.8259)</td><td>0.1287</td><td>0.8259</td></tr>
<tr><td>905</td><td>SET3_N15_CLUSTER_15317</td><td>gi|50253195|dbj|BAD29452.1| flavonoid 7-O-methyltransferase-like [Oryza sativa (japonica cultivar-group)] >gi|50252861|dbj|BAD29092.1| flavonoid 7-O-methyltransferase-like [Oryza sativa (japonica cultivar-group)] e_value:3.33899e-102</td><td>11681.m01599|LOC_Os09g17560|protein O-methyltransferase, putative e_value:1.06401e-103</td><td>0.183</td><td>(0.3592 1.9593)</td><td>0.3592</td><td>1.9593</td></tr>
<tr><td>906</td><td>SET3_M09_CLUSTER_12055</td><td>gi|55773842|dbj|BAD72380.1| PCD/DCoH-like protein 2-like [Oryza sativa (japonica cultivar-group)] e_value:9.9691e-51</td><td>11667.m04582|LOC_Os01g47420|protein PCD/DCoH-like protein 2 e_value:1.60135e-52</td><td>0.323</td><td>(0.2706 0.8389)</td><td>0.2706</td><td>0.8389</td></tr>
<tr><td>907</td><td>SET3_K03_CLUSTER_3355</td><td>gi|25809276|emb|CAD45186.1| lipoxygenase 2 [Hordeum vulgare subsp. vulgare] >gi|34922539|sp|Q8GSM3|LOX22_HORVU Lipoxygenase 2.2, chloroplast precursor (LOX2:Hv:2) e_value:0</td><td>11686.m03661|LOC_Os12g37260|protein Lipoxygenase e_value:0</td><td>0.484</td><td>(0.3388 0.7006)</td><td>0.3388</td><td>0.7006</td></tr>
<tr><td>908</td><td>SET3_G18_CLUSTER_446</td><td>gi|50915652|ref|XP_468290.1| unknown protein [Oryza sativa (japonica cultivar-group)] >gi|47497390|dbj|BAD19428.1| unknown protein [Oryza sativa (japonica cultivar-group)] >gi|47497340|dbj|BAD19380.1| unknown protein [Oryza sativa (japonica cultivar</td><td>11668.m05544|LOC_Os02g56190|protein expressed protein e_value:7.64256e-59</td><td>0.4</td><td>(0.1274 0.3184)</td><td>0.1274</td><td>0.3184</td></tr>
<tr><td>909</td><td>SET2_P20_CLUSTER_4897</td><td>gi|50904925|ref|XP_463951.1| unknown protein [Oryza sativa (japonica cultivar-group)] >gi|41053037|dbj|BAD07968.1| unknown protein [Oryza sativa (japonica cultivar-group)] e_value:3.11812e-52</td><td>11668.m00253|LOC_Os02g03250|protein hypothetical protein e_value:8.37753e-42</td><td>0.656</td><td>(0.3678 0.5605)</td><td>0.3678</td><td>0.5605</td></tr>
<tr><td>910</td><td>SET2_M24_CLUSTER_28314</td><td>gi|62733344|gb|AAX95461.1| bZIP transcriptional activator RSG-related [Oryza sativa (japonica cultivar-group)] e_value:2.53119e-114</td><td>11686.m00591|LOC_Os12g06520|protein susceptibility transcription factor RVS1 e_value:2.08415e-135</td><td>0.198</td><td>(0.1180 0.5964)</td><td>0.118</td><td>0.5964</td></tr>
<tr><td>911</td><td>SET1_O06_CLUSTER_3181</td><td>gi|50938941|ref|XP_478998.1| putative diaphanous homologue [Oryza sativa (japonica cultivar-group)] >gi|27817931|dbj|BAC55695.1| putative diaphanous homologue [Oryza sativa (japonica cultivar-group)] e_value:7.48973e-16</td><td>11669.m01815|LOC_Os03g17960|protein expressed protein e_value:0</td><td>0.399</td><td>(0.2378 0.5953)</td><td>0.2378</td><td>0.5953</td></tr>
<tr><td>912</td><td>SET1_J06_CLUSTER_25</td><td>gi|50251714|dbj|BAD27635.1| unknown protein [Oryza sativa (japonica cultivar-group)] e_value:3.04028e-21</td><td>11668.m03532|LOC_Os02g37060|protein hypothetical protein e_value:3.57463e-23</td><td>0.505</td><td>(0.2927 0.5800)</td><td>0.2927</td><td>0.58</td></tr>
<tr><td>913</td><td>SET1_F10_CLUSTER_6100</td><td>gi|50932497|ref|XP_475776.1| putative GTPase [Oryza sativa (japonica cultivar-group)] >gi|47900425|gb|AAT39219.1| putative GTPase [Oryza sativa (japonica cultivar-group)] e_value:3.02236e-93</td><td>11682.m04414|LOC_Os05g46000|protein RAB7A e_value:1.93065e-102</td><td>0.104</td><td>(0.0711 0.6864)</td><td>0.0711</td><td>0.6864</td></tr>
<tr><td>914</td><td>SET1_D24_CLUSTER_17008</td><td>gi|50253303|dbj|BAD29572.1| putative ORFX [Oryza sativa (japonica cultivar-group)] e_value:1.89716e-74</td><td>11668.m03519|LOC_Os02g36940|protein expressed protein e_value:3.35267e-76</td><td>0.195</td><td>(0.1003 0.5134)</td><td>0.1003</td><td>0.5134</td></tr>
<tr><td>915</td><td>WT013_F21_CLUSTER_3431</td><td>gi|50906783|ref|XP_464880.1| loricrin-like [Oryza sativa (japonica cultivar-group)] >gnl|BL_ORD_ID|1567456 gi|47497906|dbj|BAD20112.1| loricrin-like [Oryza sativa (japonica cultivar-group)] >gnl|BL_ORD_ID|1567456 gi|47497882|dbj|BAD20066.1| loricrin</td><td>11668.m01491|LOC_Os02g15880|protein protein F22C12.10 [imported] - Arabidopsis thaliana e_value:0</td><td>0.222</td><td>(0.1461 0.6579)</td><td>0.1461</td><td>0.6579</td></tr>
<tr><td>916</td><td>WT013_C17_CLUSTER_25150</td><td>gi|32394644|gb|AAM88439.1| putative Rieske Fe-S precursor protein [Triticum aestivum] e_value:9.63233e-129</td><td>11673.m03586|LOC_Os07g37030|protein Rieske Fe-S precursor protein e_value:2.30149e-112</td><td>0.248</td><td>(0.0756 0.3049)</td><td>0.0756</td><td>0.3049</td></tr>
<tr><td>917</td><td>WT012_P13_CLUSTER_30764</td><td>gi|50917169|ref|XP_468981.1| expressed protein (with alternative splicing) [Oryza sativa (japonica cultivar-group)] >gnl|BL_ORD_ID|1539642 gi|41469489|gb|AAS07274.1| expressed protein (with alternative splicing) [Oryza sativa (japonica cultivar-</td><td>11669.m04323|LOC_Os03g43510|protein hypothetical protein e_value:0</td><td>0.093</td><td>(0.0555 0.5939)</td><td>0.0555</td><td>0.5939</td></tr>
<tr><td>918</td><td>WT012_N24_CLUSTER_30237</td><td>gi|50907605|ref|XP_465291.1| putative pattern formation protein GNOM [Oryza sativa (japonica cultivar-group)] >gnl|BL_ORD_ID|1569354 gi|46390869|dbj|BAD16386.1| putative pattern formation protein GNOM [Oryza sativa (japonica cultivar-group)] >gnl|BL</td><td>11668.m02132|LOC_Os02g22090|protein Sec7 domain, putative e_value:0</td><td>0.121</td><td>(0.0654 0.5397)</td><td>0.0654</td><td>0.5397</td></tr>
<tr><td>919</td><td>WT012_E13_CLUSTER_29102</td><td>gi|34906596|ref|NP_914645.1| P0660F12.28 [Oryza sativa (japonica cultivar-group)] >gnl|BL_ORD_ID|1540791 gi|15290172|dbj|BAB63862.1| P0660F12.28 [Oryza sativa (japonica cultivar-group)] e_value:0</td><td>11667.m07197|LOC_Os01g71760|protein Amino acid permease e_value:0</td><td>0.248</td><td>(0.1207 0.4865)</td><td>0.1207</td><td>0.4865</td></tr>
<tr><td>920</td><td>WT011_P06_CLUSTER_3243</td><td>gi|31432605|gb|AAP54220.1| putative peptide transport protein [Oryza sativa (japonica cultivar-group)] >gnl|BL_ORD_ID|2012106 gi|37535262|ref|NP_921933.1| putative peptide transport protein [Oryza sativa (japonica cultivar-group)] >gnl|BL_ORD_ID|201</td><td>11676.m02916|LOC_Os10g33170|protein putative peptide transport protein e_value:0</td><td>0.337</td><td>(0.1266 0.3757)</td><td>0.1266</td><td>0.3757</td></tr>
<tr><td>921</td><td>WT011_N19_CLUSTER_17068</td><td>gi|34898788|ref|NP_910740.1| unknown protein [Oryza sativa (japonica cultivar-group)] >gnl|BL_ORD_ID|1528235 gi|50508288|dbj|BAD32137.1| unknown protein [Oryza sativa (japonica cultivar-group)] >gnl|BL_ORD_ID|1528235 gi|22831058|dbj|BAC15921.1| unkn</td><td>11673.m03375|LOC_Os07g35050|protein F-box domain, putative e_value:2.08295e-58</td><td>0.475</td><td>(0.5545 1.1673)</td><td>0.5545</td><td>1.1673</td></tr>
<tr><td>922</td><td>WT011_L20_CLUSTER_4471</td><td>gi|50726238|dbj|BAD33814.1| putative translation initiation factor IF-2 [Oryza sativa (japonica cultivar-group)] >gnl|BL_ORD_ID|235429 gi|50725362|dbj|BAD34434.1| putative translation initiation factor IF-2 [Oryza sativa (japonica cultivar-group)] e_va</td><td>11681.m03082|LOC_Os09g34010|protein translation initiation factor IF-2 e_value:0</td><td>0.126</td><td>(0.0616 0.4884)</td><td>0.0616</td><td>0.4884</td></tr>
<tr><td>923</td><td>WT011_K24_CLUSTER_6124</td><td>gi|55296928|dbj|BAD68379.1| unknown protein [Oryza sativa (japonica cultivar-group)] e_value:0</td><td>11680.m00207|LOC_Os06g02960|protein expressed protein</td><td>0.564</td><td>(0.4205 0.7457)</td><td>0.4205</td><td>0.7457</td></tr>
<tr><td>924</td><td>WT007_D17_CLUSTER_27787</td><td>gi|52353684|gb|AAU44250.1| unknown protein [Oryza sativa (japonica cultivar-group)] >gnl|BL_ORD_ID|1243916 gi|52353615|gb|AAU44181.1| unknown protein [Oryza sativa (japonica cultivar-group)] e_value:0</td><td>11682.m03776|LOC_Os05g39760|protein VHS domain, putative e_value:0</td><td>0.286</td><td>(0.1244 0.4351)</td><td>0.1244</td><td>0.4351</td></tr>
<tr><td>925</td><td>WT007_B23_CLUSTER_16627</td><td>gi|50919223|ref|XP_470008.1| putative helicase [Oryza sativa (japonica cultivar-group)] >gnl|BL_ORD_ID|1587484 gi|41469394|gb|AAS07217.1| putative helicase [Oryza sativa (japonica cultivar-group)] e_value:0</td><td>11687.m03582|LOC_Os11g38670|protein hypothetical protein e_value:0</td><td>0.134</td><td>(0.0739 0.5491)</td><td>0.0739</td><td>0.5491</td></tr>
<tr><td>926</td><td>WT010_H19_CLUSTER_6678</td><td>gi|50908549|ref|XP_465763.1| putative peptidylprolyl isomerase [Oryza sativa (japonica cultivar-group)] >gnl|BL_ORD_ID|1573663 gi|47848250|dbj|BAD22074.1| putative peptidylprolyl isomerase [Oryza sativa (japonica cultivar-group)] >gnl|BL_ORD_ID|1573</td><td>11668.m02744|LOC_Os02g28980|protein peptidylprolyl isomerase (EC 5.2.1.8) ROF1 - Arabidopsis thaliana e_value:0</td><td>0.256</td><td>(0.1312 0.5116)</td><td>0.1312</td><td>0.5116</td></tr>
<tr><td>927</td><td>WT013_M22_CLUSTER_18688</td><td>gi|50938537|ref|XP_478796.1| putative adenosine-5'-phosphosulfate kinase [Oryza sativa (japonica cultivar-group)] >gnl|BL_ORD_ID|1529561 gi|34393551|dbj|BAC83149.1| putative adenosine-5'-phosphosulfate kinase [Oryza sativa (japonica</td><td>11673.m03749|LOC_Os07g38560|protein adenylylsulfate kinase, putative e_value:3.91238e-123</td><td>0.292</td><td>(0.1857 0.6367)</td><td>0.1857</td><td>0.6367</td></tr>
<tr><td>928</td><td>WT006_D22_CLUSTER_24608</td><td>gi|50878307|gb|AAT85082.1| putative pyruvate orthophosphate dikinase [Oryza sativa (japonica cultivar-group)] e_value:0</td><td>11682.m03148|LOC_Os05g33570|protein pyruvate, phosphate dikinase e_value:0</td><td>0.104</td><td>(0.0524 0.5032)</td><td>0.0524</td><td>0.5032</td></tr>
<tr><td>929</td><td>WT006_J18_CLUSTER_19666</td><td>gi|51038254|gb|AAT94057.1| unknown protein [Oryza sativa (japonica cultivar-group)] >gnl|BL_ORD_ID|1020675 gi|46485791|gb|AAS98416.1| unknown protein [Oryza sativa (japonica cultivar-group)] e_value:0</td><td>11682.m00391|LOC_Os05g04550|protein F21D18.2 e_value:0</td><td>0.268</td><td>(0.1302 0.4852)</td><td>0.1302</td><td>0.4852</td></tr>
<tr><td>930</td><td>WT009_C23_CLUSTER_34207</td><td>gi|50909029|ref|XP_466003.1| putative copine III [Oryza sativa (japonica cultivar-group)] >gnl|BL_ORD_ID|1577509 gi|49388914|dbj|BAD26136.1| putative copine III [Oryza sativa (japonica cultivar-group)] e_value:0</td><td>11668.m03023|LOC_Os02g32160|protein At5g61900 e_value:0</td><td>0.117</td><td>(0.0619 0.5274)</td><td>0.0619</td><td>0.5274</td></tr>
<tr><td>931</td><td>SET2_C18_CLUSTER_25104</td><td>gi|51979050|ref|XP_507402.1| PREDICTED P0519E12.126 gene product [Oryza sativa (japonica cultivar-group)] >gi|50939429|ref|XP_479242.1| beta-1,3-glucanase-like protein [Oryza sativa (japonica cultivar-group)] >gi|51979048|ref|XP_507401.1| PREDICTED</td><td>11673.m04317|LOC_Os07g43940|protein expressed protein e_value:4.86534e-63</td><td>0.588</td><td>(0.2453 0.4173)</td><td>0.2453</td><td>0.4173</td></tr>
<tr><td>932</td><td>WT004_E04_CLUSTER_19171</td><td>gi|2213882|gb|AAB61598.1| 2-isopropylmalate synthase [Lycopersicon pennellii] >gnl|BL_ORD_ID|262520 gi|7387848|sp|O04973|LEU1A_LYCPN 2-isopropylmalate synthase A (Alpha-isopropylmalate synthase A) (Alpha-IPM synthetase A)</td><td>11687.m00383|LOC_Os11g04670|protein 2-isopropylmalate synthase e_value:0</td><td>0.113</td><td>(0.0572 0.5050)</td><td>0.0572</td><td>0.505</td></tr>
<tr><td>933</td><td>WT013_P12_CLUSTER_32858</td><td>gi|50934369|ref|XP_476712.1| putative histone deacetylase [Oryza sativa (japonica cultivar-group)] >gnl|BL_ORD_ID|2367662 gi|50510096|dbj|BAD30767.1| putative histone deacetylase [Oryza sativa (japonica cultivar-group)] >gnl|BL_ORD_ID|2367662 gi|331</td><td>11673.m00621|LOC_Os07g06980|protein histone deacetylase HDA110 isoform 1 e_value:0</td><td>0.244</td><td>(0.1100 0.4506)</td><td>0.11</td><td>0.4506</td></tr>
<tr><td>934</td><td>WT013_M06_CLUSTER_2785</td><td>gi|34904418|ref|NP_913556.1| unnamed protein product [Oryza sativa (japonica cultivar-group)] e_value:0</td><td>11667.m01328|LOC_Os01g13470|protein KH domain, putative e_value:0</td><td>0.306</td><td>(0.1430 0.4668)</td><td>0.143</td><td>0.4668</td></tr>
<tr><td>935</td><td>SET3_N24_CLUSTER_9954</td><td>gi|55297038|dbj|BAD68608.1| unknown protein [Oryza sativa (japonica cultivar-group)] e_value:7.58515e-111</td><td>11680.m03729|LOC_Os06g38320|protein expressed protein e_value:1.55849e-112</td><td>0.301</td><td>(0.1250 0.4155)</td><td>0.125</td><td>0.4155</td></tr>
<tr><td>936</td><td>WT013_J22_CLUSTER_32195</td><td>gi|55297446|dbj|BAD69297.1| SF16 protein-like [Oryza sativa (japonica cultivar-group)] >gnl|BL_ORD_ID|270066 gi|55296691|dbj|BAD69409.1| SF16 protein-like [Oryza sativa (japonica cultivar-group)] e_value:8.35797e-142</td><td>11680.m00559|LOC_Os06g06160|protein expressed protein e_value:1.5778e-143</td><td>0.177</td><td>(0.1183 0.6674)</td><td>0.1183</td><td>0.6674</td></tr>
<tr><td>937</td><td>WT003_G03_CLUSTER_28167</td><td>gi|50912043|ref|XP_467429.1| putative growth regulator [Oryza sativa (japonica cultivar-group)] >gnl|BL_ORD_ID|1580572 gi|41052863|dbj|BAD07777.1| putative growth regulator [Oryza sativa (japonica cultivar-group)] >gnl|BL_ORD_ID|1580572 gi|41052647|</td><td>11668.m04669|LOC_Os02g48110|protein dnaK protein, putative e_value:0</td><td>0.146</td><td>(0.0759 0.5210)</td><td>0.0759</td><td>0.521</td></tr>
</table>

PDF Complete
Your complimentary use period has ended. Thank you for using PDF Complete.
Click Here to upgrade to Unlimited Pages and Expanded Features

938	WT002_B09_CLUSTER_9624	gi|10086260|gb|AAG12459.1| calmodulin-binding protein MPCBP [Zea mays] e_value:0	11686.m03720|LOC_Os12g37780|protein calmodulin-binding protein MPCBP e_value:0	0.128	(0.0635 0.4951)	0.0635	0.4951
939	WT002_E01_CLUSTER_4117	gi|50913031|ref|XP_467923.1| putative aminopeptidase MAP1 precursor [Oryza sativa (japonica cultivar-group)] >gnl|BL_ORD_ID|1583040 gi|46805893|dbj|BAD17206.1| putative aminopeptidase MAP1 precursor [Oryza sativa (japonica cultivar-group)] e_value:4.94	11668.m05131|LOC_Os02g52420|protein methionine aminopeptidase, type I, putative e_value:8.76638e-172	0.178	(0.0900 0.5056)	0.09	0.5056
940	WT002_D09_CLUSTER_9389	gi|21387205|gb|AAM48006.1| unknown protein [Arabidopsis thaliana] >gnl|BL_ORD_ID|1033010 gi|15223402|ref|NP_174587.1| MATE efflux family protein [Arabidopsis thaliana] >gnl|BL_ORD_ID|1033010 gi|17065360|gb|AAL32834.1| Unknown protein [Arabidopsis th	11686.m00247|LOC_Os12g03260|protein MATE efflux family protein, putative e_value:0	0.118	(0.1426 1.2043)	0.1426	1.2043
941	WT012_F19_CLUSTER_5999	gi|50905793|ref|XP_464385.1| unknown protein [Oryza sativa (japonica cultivar-group)] >gnl|BL_ORD_ID|1566578 gi|46390049|dbj|BAD15425.1| unknown protein [Oryza sativa (japonica cultivar-group)] >gnl|BL_ORD_ID|1566578 gi|46389895|dbj|BAD15516.1| unkn	11680.m04232|LOC_Os06g43140|protein Uncharacterised protein family, putative e_value:2.62494e-85	0.226	(0.0940 0.4167)	0.094	0.4167
942	WT010_G06_CLUSTER_7683	gi|56784132|dbj|BAD81517.1| Graves disease mitochondrial solute carrier protein-like [Oryza sativa (japonica cultivar-group)] e_value:8.1494e-171	11667.m05005|LOC_Os01g51250|protein Mitochondrial carrier protein, putative e_value:1.69089e-172	0.106	(0.0624 0.5876)	0.0624	0.5876
943	WT013_K13_CLUSTER_17186	gi|50540744|gb|AAT77900.1| putative magnesium chelatase [Oryza sativa (japonica cultivar-group)] e_value:0	11669.m06059|LOC_Os03g59640|protein putative magnesium chelatase subunit chlD e_value:0	0.138	(0.0742 0.5360)	0.0742	0.536
944	WT012_J20_CLUSTER_6834	gi|50935225|ref|XP_477140.1| putative mRNA binding protein precursor [Oryza sativa (japonica cultivar-group)] >gnl|BL_ORD_ID|1522795 gi|51963328|ref|XP_506223.1| PREDICTED OJ1664_D08.105 gene product [Oryza sativa (japonica cultivar-group)] >gnl|BL_	11673.m01062|LOC_Os07g11110|protein expressed protein e_value:6.76638e-171	0.254	(0.1222 0.4805)	0.1222	0.4805
945	WT007_A09_CLUSTER_34877	gi|50912301|ref|XP_467558.1| sucrase-like protein [Oryza sativa (japonica cultivar-group)] >gnl|BL_ORD_ID|1580697 gi|51964326|ref|XP_506948.1| PREDICTED P0685G12.41 gene product [Oryza sativa (japonica cultivar-group)] >gnl|BL_ORD_ID|1580697 gi|4573	11668.m04799|LOC_Os02g49320|protein hypothetical protein e_value:3.49916e-167	0.462	(0.1607 0.3481)	0.1607	0.3481
946	WT005_P17_CLUSTER_31621	gi|18057165|gb|AAL58188.1| unknown protein [Oryza sativa (japonica cultivar-group)] >gnl|BL_ORD_ID|2066637 gi|31433679|gb|AAP55163.1| unknown protein [Oryza sativa (japonica cultivar-group)] >gnl|BL_ORD_ID|2066637	11676.m03954|LOC_Os10g42700|protein expressed protein e_value:4.40661e-172	0.137	(0.1144 0.8326)	0.1144	0.8326
947	WT006_O11_CLUSTER_33372	gi|50911657|ref|XP_467236.1| putative Clathrin coat assembly protein AP50 [Oryza sativa (japonica cultivar-group)] >gnl|BL_ORD_ID|1580342 gi|41052815|dbj|BAD07683.1| putative Clathrin coat assembly protein AP50 [Oryza sativa (japonica cultivar-	11668.m04500|LOC_Os02g46510|protein Adaptor complexes medium subunit family e_value:0	0.017	(0.0080 0.4680)	0.008	0.468
948	WT009_A11_CLUSTER_12359	gi|50928709|ref|XP_473882.1| OSJNBa0008M17.6 [Oryza sativa (japonica cultivar-group)] >gnl|BL_ORD_ID|1592029 gi|38344253|emb|CAD41791.2| OSJNBa0008M17.6 [Oryza sativa (japonica cultivar-group)] e_value:0	11674.m03100|LOC_Os08g31470|protein expressed protein e_value:0	0.09	(0.2139 2.3861)	0.2139	2.3861
949	WT010_O16_CLUSTER_32321	gi|51535981|dbj|BAD38062.1| putative coiled-coil protein [Oryza sativa (japonica cultivar-group)] >gnl|BL_ORD_ID|242954 gi|51535338|dbj|BAD38597.1| putative coiled-coil protein [Oryza sativa (japonica cultivar-group)] e_value:0	11668.m00528|LOC_Os02g05840|protein At4g30200 e_value:0	0.271	(0.1463 0.5405)	0.1463	0.5405
950	WT005_I15_CLUSTER_616	gi|51535836|dbj|BAD37920.1| leaf senescence protein-like [Oryza sativa (japonica cultivar-group)] >gnl|BL_ORD_ID|242731 gi|51535116|dbj|BAD37779.1| leaf senescence protein-like [Oryza sativa (japonica cultivar-group)] e_value:8.11094e-163	11680.m01258|LOC_Os06g12820|protein hypothetical protein e_value:2.14914e-174	0.544	(0.2408 0.4423)	0.2408	0.4423
951	WT004_A11_CLUSTER_1843	gi|38345584|emb|CAD41637.2| OSJNBb0012E24.2 [Oryza sativa (japonica cultivar-group)] >gnl|BL_ORD_ID|1522484 gi|50927849|ref|XP_473452.1| OSJNBb0012E24.2 [Oryza sativa (japonica cultivar-group)] e_value:0	11670.m04572|LOC_Os04g46880|protein major facilitator superfamily protein e_value:0	0.11	(0.0890 0.8121)	0.089	0.8121
952	SET2_O13_CLUSTER_26518	gi|31433539|gb|AAP55037.1| putative peptide methionine sulfoxide reductase [Oryza sativa (japonica cultivar-group)] >gi|37536896|ref|NP_922750.1| putative peptide methionine sulfoxide reductase [Oryza sativa (japonica cultivar-group)] >gi|12597894|g	11676.m03810|LOC_Os10g41400|protein putative peptide methionine sulfoxide reductase e_value:1.71785e-115	0.269	(0.1294 0.4818)	0.1294	0.4818
953	SET1_H17_CLUSTER_1338	gi|2150000|gb|AAB58718.1| aleurone ribonuclease [Hordeum vulgare] >gi|7435264|pir||T04419 aleurone ribonuclease (EC 3.1.-.-) - barley (fragment) e_value:3.28724e-135	11673.m04285|LOC_Os07g43670|protein Ribonuclease T2 family e_value:1.83625e-97	0.622	(0.2105 0.3383)	0.2105	0.3383
954	WT005_L01_CLUSTER_11410	gi|11994760|dbj|BAB03089.1| unnamed protein product [Arabidopsis thaliana] >gnl|BL_ORD_ID|1098003 gi|42565189|ref|NP_189197.2| hydroxyproline-rich glycoprotein family protein [Arabidopsis thaliana] >gnl|BL_ORD_ID|1098003 gi|28071265|dbj|BAC55960.1|	11687.m00045|LOC_Os11g01440|protein expressed protein e_value:0	0.148	(0.1123 0.7583)	0.1123	0.7583
955	WT007_F23_CLUSTER_22440	gi|1066163|emb|CAA63598.1| glyoxysomal beta-ketoacyl-thiolase [Brassica napus] >gnl|BL_ORD_ID|1712428 gi|7433644|pir||T07989 acetyl-CoA C-acyltransferase (EC 2.3.1.16) precursor, glyoxysomal - rape e_value:0	11676.m02790|LOC_Os10g31950|protein putative thiolase e_value:0	0.187	(0.1223 0.6527)	0.1223	0.6527
956	SET3_M10_CLUSTER_4173	gi|38016525|gb|AAR07598.1| fiber protein Fb19 [Gossypium barbadense] e_value:2.64839e-55	11686.m03594|LOC_Os12g36640|protein universal stress protein family e_value:1.95787e-77	0.149	(0.0717 0.4822)	0.0717	0.4822
957	SET4_I24_CLUSTER_33707	gi|53791808|dbj|BAD53753.1| putative SF21C1 protein [Oryza sativa (japonica cultivar-group)] e_value:0	11680.m03565|LOC_Os06g36740|protein SF21 protein e_value:3.81344e-175	0.082	(0.0514 0.6257)	0.0514	0.6257
958	WT004__C03_CLUSTER_5537	gi|50725801|dbj|BAD33331.1| putative cellulase [Oryza sativa (japonica cultivar-group)] e_value:0	11681.m03229|LOC_Os09g36350|protein protein F10A5.13 [imported] - Arabidopsis thaliana e_value:0	0.136	(0.0688 0.5062)	0.0688	0.5062
959	WT013_F08_CLUSTER_30360	gi|9758595|dbj|BAB09228.1| unnamed protein product [Arabidopsis thaliana] >gnl|BL_ORD_ID|1088517 gi|28393905|gb|AAO42360.1| unknown protein [Arabidopsis thaliana] >gnl|BL_ORD_ID|1088517 gi|27754360|gb|AAO22629.1| unknown protein [Arabidopsis thalian	11686.m03062|LOC_Os12g31490|protein expressed protein e_value:1.36591e-142	0.228	(0.1353 0.5943)	0.1353	0.5943
960	SET4_N08_CLUSTER_1464	gi|21593452|gb|AAM65419.1| unknown [Arabidopsis thaliana] e_value:4.4349e-74	11674.m01513|LOC_Os08g15500|protein expressed protein e_value:6.61904e-128	0.201	(0.1386 0.6906)	0.1386	0.6906
961	WT005_A24_CLUSTER_8551	gi|50916805|ref|XP_468799.1| expressed protein [Oryza sativa (japonica cultivar-group)] >gnl|BL_ORD_ID|1540243 gi|40538901|gb|AAR87158.1| expressed protein [Oryza sativa (japonica cultivar-group)] e_value:4.01757e-44	11669.m03207|LOC_Os03g31680|protein hypothetical protein e_value:4.08182e-46	0.164	(0.0816 0.4970)	0.0816	0.497
962	WT008_P16_CLUSTER_13485	gi|22530910|gb|AAM96959.1| putative protein [Arabidopsis thaliana] >gnl|BL_ORD_ID|1188115 gi|28059788|gb|AAO30093.1| putative protein [Arabidopsis thaliana] >gnl|BL_ORD_ID|1188115 gi|30690528|ref|NP_849503.1| expressed protein [Arabidopsis thaliana]	11669.m00561|LOC_Os03g06120|protein expressed protein e_value:0	0.184	(0.0839 0.4562)	0.0839	0.4562
963	WT008_F01_CLUSTER_26210	gi|38567882|emb|CAD40892.2| OSJNBa0036B21.10 [Oryza sativa (japonica cultivar-group)] >gnl|BL_ORD_ID|1524093 gi|50924756|ref|XP_472730.1| OSJNBa0036B21.10 [Oryza sativa (japonica cultivar-group)] e_value:0	11670.m03643|LOC_Os04g38450|protein gamma-glutamyltransferase e_value:0	0.137	(0.1081 0.7883)	0.1081	0.7883
964	WT012_O05_CLUSTER_33328	gi|34908048|ref|NP_915371.1| P0506B12.13 [Oryza sativa (japonica cultivar-group)] e_value:5.34144e-63	11667.m06177|LOC_Os01g61890|protein AT hook motif, putative e_value:5.4757e-64	0.595	(0.3768 0.6333)	0.3768	0.6333
965	WT005__C20_CLUSTER_32067	gi|30385246|gb|AAP23942.1| caffeic acid O-methyltransferase [Triticum aestivum] e_value:0	11674.m00533|LOC_Os08g06100|protein caffeic acid 3-o-methyltransferase (ec 2.1.1.68) (s-adenosyl-l-methionine:caffeic acid 3-o-methyltransferase) (comt) (caomt)	0.216	(0.1169 0.5405)	0.1169	0.5405
966	WT007_L06_CLUSTER_32598	gi|18483217|gb|AAL73968.1| laccase LAC5-6 [Lolium perenne] e_value:0	11667.m06248|LOC_Os01g62480|protein probable laccase (EC 1.10.3.2) - rice (fragment) e_value:0	0.374	(0.1101 0.2940)	0.1101	0.294
967	WT012_N11_CLUSTER_22847	gi|34911538|ref|NP_917116.1| P0421H07.18 [Oryza sativa (japonica cultivar-group)] >gnl|BL_ORD_ID|1547569 gi|20804599|dbj|BAB92290.1| RING zinc finger protein-like [Oryza sativa (japonica cultivar-group)] e_value:1.63008e-157	11667.m04953|LOC_Os01g50750|protein Zinc finger, C3HC4 type (RING finger), putative e_value:3.17528e-159	0.192	(0.0494 0.2572)	0.0494	0.2572
968	WT003_H05_CLUSTER_2109	gi|53792678|dbj|BAD53690.1| putative CPRD2 [Oryza sativa (japonica cultivar-group)] e_value:0	11680.m03448|LOC_Os06g35630|protein AT4g20860/T13K14_20 e_value:0	0.552	(0.2658 0.4814)	0.2658	0.4814
969	WT007__C22_CLUSTER_31465	gi|55276711|gb|AAV49983.1| ATPase 2 [Hordeum vulgare subsp. vulgare] e_value:0	11686.m04417|LOC_Os12g44190|protein contains similarity to AAA-type ATPase~gene_id:MYH19.21 e_value:0	0.4	(0.2306 0.5770)	0.2306	0.577
970	SET4_P17_CLUSTER_4381	gi|42733490|dbj|BAD11342.1| BRI1-KD interacting protein 114 [Oryza sativa (japonica cultivar-group)] e_value:1.1391e-77	11686.m03549|LOC_Os12g36200|protein Nucleoside diphosphate kinase e_value:1.41585e-76	0.051	(0.0376 0.7404)	0.0376	0.7404
971	WT002_O21_CLUSTER_33705	gi|55295966|dbj|BAD68006.1| Na(+) dependent transporter-like [Oryza sativa (japonica cultivar-group)] e_value:0	11667.m04402|LOC_Os01g45750|protein bile acid transporter e_value:0	0.142	(0.0685 0.4834)	0.0685	0.4834
972	WT008_E13_CLUSTER_34357	gi|51090498|dbj|BAD35700.1| putative aminoacylase [Oryza sativa (japonica cultivar-group)] e_value:0	11680.m01047|LOC_Os06g10770|protein N-acyl-L-amino-acid amidohydrolase e_value:0	0.203	(0.0936 0.4613)	0.0936	0.4613
973	WT003_E09_CLUSTER_33967	gi|50904565|ref|XP_463771.1| unknown protein [Oryza sativa (japonica cultivar-group)] >gnl|BL_ORD_ID|1566067 gi|41053219|dbj|BAD08180.1| unknown protein [Oryza sativa (japonica cultivar-group)] e_value:0	11668.m00008|LOC_Os02g01070|protein FG-GAP repeat, putative e_value:0	0.143	(0.0953 0.6671)	0.0953	0.6671
974	WT012_K23_CLUSTER_770	gi|62701764|gb|AAX92837.1| AT5g19150/T24G5_50 [Oryza sativa (japonica cultivar-group)] e_value:3.09065e-175	11687.m01633|LOC_Os11g17610|protein AT5g19150/T24G5_50 e_value:7.48048e-170	0.107	(0.0551 0.5125)	0.0551	0.5125
975	WT008_L04_CLUSTER_1308	gi|50916216|ref|XP_468567.1| Hypothetical protein [Oryza sativa (japonica cultivar-group)] >gnl|BL_ORD_ID|1584237 gi|24431602|gb|AAN61482.1| Hypothetical protein [Oryza sativa (japonica cultivar-group)] e_value:3.61743e-116	11669.m00107|LOC_Os03g01920|protein cyclin delta-3 e_value:1.4492e-161	0.148	(0.0945 0.6381)	0.0945	0.6381
976	SET2_D06_CLUSTER_1134	gi|21326121|gb|AAM47587.1| unknown protein [Sorghum bicolor] e_value:1.28415e-40	11669.m05527|LOC_Os03g54980|protein expressed protein e_value:1.08879e-48	0.309	(0.0920 0.2976)	0.092	0.2976
977	WT013_P19_CLUSTER_33344	gi|56783885|dbj|BAD81322.1| target of myb1 -like [Oryza sativa (japonica cultivar-group)] >gnl|BL_ORD_ID|971219 gi|56784382|dbj|BAD82421.1| target of myb1 -like [Oryza sativa (japonica cultivar-group)] e_value:0	11667.m01263|LOC_Os01g12880|protein hypothetical protein e_value:0	0.211	(0.1232 0.5831)	0.1232	0.5831
978	WT006_A11_CLUSTER_33553	gi|21537137|gb|AAM61478.1| myosin-like protein [Arabidopsis thaliana] >gnl|BL_ORD_ID|1214207 gi|29824283|gb|AAP04102.1| unknown protein [Arabidopsis thaliana] >gnl|BL_ORD_ID|1214207 gi|26452460|dbj|BAC43315.1| putative myosin [Arabidopsis thaliana]	11686.m00872|LOC_Os12g09110|protein expressed protein e_value:2.05152e-127	0.247	(0.1452 0.5872)	0.1452	0.5872
979	SET6_G16_CLUSTER_27764	gi|62733400|gb|AAX95517.1| Putative peptide chain release factor subunit 1 protein [Oryza sativa (japonica cultivar-group)] >gi|50918075|ref|XP_469434.1| eukaryotic peptide chain release factor subunit 1-3 (eRF1-3) [Oryza sativa (japonica cultivar-grou	11669.m04985|LOC_Os03g49580|protein eukaryotic peptide chain release factor subunit 1-3 (eRF1-3) e_value:0	0.057	(0.0312 0.5495)	0.0312	0.5495

PDF Complete
Your complimentary use period has ended. Thank you for using PDF Complete.
Click Here to upgrade to Unlimited Pages and Expanded Features

<table>
<tr><th></th><th></th><th></th><th></th><th></th><th></th><th></th><th></th></tr>
<tr><td>980</td><td>WT006_F11_CLUSTER_11494</td><td>gi|8118717|gb|AAF73075.1| molybdenum cofactor biosynthesis protein Cnx1 [Hordeum vulgare] e_value:0</td><td>11670.m05583|LOC_Os04g56620|protein Similar to molybdopterin biosynthesis cnx1 protein (molybdenum cofactor biosynthesis enzyme cnx1). [mouse-ear cress e_value:0</td><td>0.153</td><td>(0.0824 0.5384)</td><td>0.0824</td><td>0.5384</td></tr>
<tr><td>981</td><td>WT006_B22_CLUSTER_34078</td><td>gi|4091008|gb|AAC99620.1| methionyl-tRNA synthetase [Oryza sativa] >gnl|BL_ORD_ID|292398 gi|12230649|sp|Q9ZTS1|SYM_ORYSA Probable methionyl-tRNA synthetase (Methionine--tRNA ligase) (MetRS) e_value:0</td><td>11680.m03000|LOC_Os06g31210|protein probable methionyl-trna synthetase (ec 6.1.1.10) (methionine--trnaligase) (metrs)</td><td>0.057</td><td>(0.0363 0.6385)</td><td>0.0363</td><td>0.6385</td></tr>
<tr><td>982</td><td>WT012_F18_CLUSTER_23675</td><td>gi|50726384|dbj|BAD33995.1| peroxisomal biogenesis factor 3-like [Oryza sativa (japonica cultivar-group)] e_value:1.30869e-170</td><td>11681.m01290|LOC_Os09g14510|protein At1g48640/F11I4_17 e_value:3.38647e-172</td><td>0.151</td><td>(0.1035 0.6851)</td><td>0.1035</td><td>0.6851</td></tr>
<tr><td>983</td><td>WT013_P04_CLUSTER_7426</td><td>gi|62734079|gb|AAX96188.1| short-chain dehydrogenase Tic32 [Oryza sativa (japonica cultivar-group)] e_value:4.42419e-143</td><td>11687.m00732|LOC_Os11g07930|protein short-chain dehydrogenase Tic32 e_value:1.40124e-144</td><td>0.216</td><td>(0.1396 0.6451)</td><td>0.1396</td><td>0.6451</td></tr>
<tr><td>984</td><td>WT002_B16_CLUSTER_32045</td><td>gi|50920247|ref|XP_470484.1| unknown protein [Oryza sativa (japonica cultivar-group)] >gnl|BL_ORD_ID|1535816 gi|30102983|gb|AAP21396.1| unknown protein [Oryza sativa (japonica cultivar-group)] e_value:1.1155e-125</td><td>11669.m06573|LOC_Os03g64020|protein expressed protein e_value:2.66714e-127</td><td>0.199</td><td>(0.1449 0.7277)</td><td>0.1449</td><td>0.7277</td></tr>
<tr><td>985</td><td>WT004_M10_CLUSTER_32932</td><td>gi|62734077|gb|AAX96186.1| Endomembrane protein 70 [Oryza sativa (japonica cultivar-group)] e_value:0</td><td>11686.m00719|LOC_Os12g07670|protein endomembrane protein 70, putative e_value:0</td><td>0.071</td><td>(0.0449 0.6314)</td><td>0.0449</td><td>0.6314</td></tr>
<tr><td>986</td><td>WT002_G14_CLUSTER_32121</td><td>gi|50947591|ref|XP_483323.1| putative proton pump interactor [Oryza sativa (japonica cultivar-group)] >gnl|BL_ORD_ID|1609622 gi|42408811|dbj|BAD10072.1| putative proton pump interactor [Oryza sativa (japonica cultivar-group)] e_value:0</td><td>11674.m04037|LOC_Os08g40250|protein expressed protein e_value:0</td><td>0.555</td><td>(0.6667 1.2013)</td><td>0.6667</td><td>1.2013</td></tr>
<tr><td>987</td><td>WT003_O20_CLUSTER_33413</td><td>gi|52077312|dbj|BAD46353.1| putative membrane protein [Oryza sativa (japonica cultivar-group)] >gnl|BL_ORD_ID|252455 gi|52076044|dbj|BAD46497.1| putative membrane protein [Oryza sativa (japonica cultivar-group)] e_value:7.32623e-169</td><td>11681.m03162|LOC_Os09g35730|protein membrane protein e_value:1.91486e-170</td><td>0.137</td><td>(0.0685 0.4990)</td><td>0.0685</td><td>0.499</td></tr>
<tr><td>988</td><td>SET6_M08_CLUSTER_33413</td><td>gi|50948211|ref|XP_483633.1| putative membrane protein [Oryza sativa (japonica cultivar-group)] >gi|42408095|dbj|BAD09236.1| putative membrane protein [Oryza sativa (japonica cultivar-group)] e_value:8.27671e-160</td><td>11674.m04372|LOC_Os08g43320|protein Rhomboid family, putative e_value:1.68553e-161</td><td>0.121</td><td>(0.0758 0.6251)</td><td>0.0758</td><td>0.6251</td></tr>
<tr><td>989</td><td>WT006_J03_CLUSTER_20000</td><td>gi|38346912|emb|CAE03884.2| OSJNBb0015N08.12 [Oryza sativa (japonica cultivar-group)] >gnl|BL_ORD_ID|1535341 gi|38346541|emb|CAD41784.2| OSJNBa0035M09.2 [Oryza sativa (japonica cultivar-group)] >gnl|BL_ORD_ID|1535341 gi|50928545|ref|XP_473800.1| OSJ</td><td>11670.m05046|LOC_Os04g51610|protein calcium-translocating P-type ATPase, PMCA-type e_value:0</td><td>0.116</td><td>(0.0881 0.7573)</td><td>0.0881</td><td>0.7573</td></tr>
<tr><td>990</td><td>WT008_J16_CLUSTER_32943</td><td>gi|50929779|ref|XP_474417.1| OSJNBa0088H09.13 [Oryza sativa (japonica cultivar-group)] >gnl|BL_ORD_ID|1594601 gi|32488712|emb|CAE03455.1| OSJNBa0088H09.13 [Oryza sativa (japonica cultivar-group)] e_value:4.70556e-149</td><td>11670.m05828|LOC_Os04g58830|protein Ribosome biogenesis regulatory protein (RRS1), putative e_value:2.30392e-150</td><td>0.218</td><td>(0.1038 0.4763)</td><td>0.1038</td><td>0.4763</td></tr>
<tr><td>991</td><td>WT007_K17_CLUSTER_33089</td><td>gi|50898666|ref|XP_450121.1| ankyrin repeat protein E4_8-like [Oryza sativa (japonica cultivar-group)] >gnl|BL_ORD_ID|1563265 gi|47497788|dbj|BAD19887.1| ankyrin repeat protein E4_8-like [Oryza sativa (japonica cultivar-group)] e_value:3.41611e-118</td><td>11686.m04044|LOC_Os12g40770|protein ankyrin-like protein-like protein e_value:3.6267e-80</td><td>0.355</td><td>(0.5425 1.5273)</td><td>0.5425</td><td>1.5273</td></tr>
<tr><td>992</td><td>WT002_K14_CLUSTER_1800</td><td>gi|50948039|ref|XP_483547.1| putative glycyl-tRNA synthetase [Oryza sativa (japonica cultivar-group)] >gnl|BL_ORD_ID|1610512 gi|38175549|dbj|BAD01242.1| putative glycyl-tRNA synthetase [Oryza sativa (japonica cultivar-group)] e_value:0</td><td>11674.m04290|LOC_Os08g42560|protein glycyl-tRNA synthetase e_value:0</td><td>0.057</td><td>(0.0348 0.6087)</td><td>0.0348</td><td>0.6087</td></tr>
<tr><td>993</td><td>WT006__C24_CLUSTER_10349</td><td>gi|31432839|gb|AAP54426.1| putative signal tranduction protein [Oryza sativa (japonica cultivar-group)] >gnl|BL_ORD_ID|2012285 gi|37535674|ref|NP_922139.1| putative signal tranduction protein [Oryza sativa (japonica cultivar-group)] >gnl|BL_ORD_ID|2</td><td>11676.m03138|LOC_Os10g35250|protein putative signal tranduction protein e_value:0</td><td>0.148</td><td>(0.0759 0.5120)</td><td>0.0759</td><td>0.512</td></tr>
<tr><td>994</td><td>SET2_K08_CLUSTER_27709</td><td>gi|50905471|ref|XP_464224.1| putative receptor protein kinase PERK [Oryza sativa (japonica cultivar-group)] >gi|49388415|dbj|BAD25548.1| putative receptor protein kinase PERK [Oryza sativa (japonica cultivar-group)]</td><td>11668.m00645|LOC_Os02g06930|protein Protein kinase domain, putative e_value:1.80109e-179</td><td>0.253</td><td>(0.1021 0.4034)</td><td>0.1021</td><td>0.4034</td></tr>
<tr><td>995</td><td>WT004_K04_CLUSTER_33223</td><td>gi|38346118|emb|CAE04596.2| OSJNBb0006N15.13 [Oryza sativa (japonica cultivar-group)] >gnl|BL_ORD_ID|1523192 gi|50923675|ref|XP_472198.1| OSJNBb0006N15.13 [Oryza sativa (japonica cultivar-group)] e_value:0</td><td>11670.m02923|LOC_Os04g30780|protein Mov34/MPN/PAD-1 family, putative e_value:0</td><td>0.044</td><td>(0.0257 0.5859)</td><td>0.0257</td><td>0.5859</td></tr>
<tr><td>996</td><td>WT005_D07_CLUSTER_18512</td><td>gi|56567587|gb|AAV98703.1| BTH-induced ERF transcriptional factor 4 [Oryza sativa (indica cultivar-group)] >gnl|BL_ORD_ID|1920740 gi|50920401|ref|XP_470561.1| Putative EREBP-like protein [Oryza sativa] >gnl|BL_ORD_ID|1920740 gi|15217288|gb|AAK92632.</td><td>11669.m00826|LOC_Os03g08500|protein AP2 domain, putative e_value:1.26412e-98</td><td>0.754</td><td>(0.2494 0.3309)</td><td>0.2494</td><td>0.3309</td></tr>
<tr><td>997</td><td>WT010_J11_CLUSTER_12240</td><td>gi|55770101|ref|XP_550040.1| putative sterol-C5(6)-desaturase [Oryza sativa (japonica cultivar-group)] >gnl|BL_ORD_ID|1753741 gi|53792172|dbj|BAD52805.1| putative sterol-C5(6)-desaturase [Oryza sativa (japonica cultivar-group)] e_value:5.75077e-144</td><td>11667.m00343|LOC_Os01g04260|protein delta-7-sterol-c5(6)-desaturase (ec 1.3.3.-) (delta-7-c-5 steroldesaturase) (delta7-sterol-c5-desaturase) e_value:1.35014e-145</td><td>0.199</td><td>(0.0960 0.4822)</td><td>0.096</td><td>0.4822</td></tr>
<tr><td>998</td><td>WT010_I10_CLUSTER_12240</td><td>gi|55770101|ref|XP_550040.1| putative sterol-C5(6)-desaturase [Oryza sativa (japonica cultivar-group)] >gnl|BL_ORD_ID|1753741 gi|53792172|dbj|BAD52805.1| putative sterol-C5(6)-desaturase [Oryza sativa (japonica cultivar-group)] e_value:5.75077e-144</td><td>11667.m00343|LOC_Os01g04260|protein delta-7-sterol-c5(6)-desaturase (ec 1.3.3.-) (delta-7-c-5 steroldesaturase) (delta7-sterol-c5-desaturase) e_value:1.35014e-145</td><td>0.199</td><td>(0.0960 0.4822)</td><td>0.096</td><td>0.4822</td></tr>
<tr><td>999</td><td>WT005_D04_CLUSTER_33193</td><td>gi|5640157|emb|CAB51555.1| gibberellin response modulator [Triticum aestivum] e_value:0</td><td>11669.m05032|LOC_Os03g49990|protein gibberellin-insensitive protein OsGAI e_value:0</td><td>0.174</td><td>(0.0648 0.3729)</td><td>0.0648</td><td>0.3729</td></tr>
<tr><td>1000</td><td>WT006_M22_CLUSTER_33177</td><td>gi|56202166|dbj|BAD73644.1| 60S ribosomal protein L18A-like [Oryza sativa (japonica cultivar-group)] e_value:2.88913e-54</td><td>11682.m04702|LOC_Os05g49020|protein F20D23.22 protein - Arabidopsis thaliana e_value:5.35262e-74</td><td>0.308</td><td>(0.1593 0.5163)</td><td>0.1593</td><td>0.5163</td></tr>
<tr><td>1001</td><td>WT005_J12_CLUSTER_27018</td><td>gi|55168343|gb|AAV44208.1| putative kinesin [Oryza sativa (japonica cultivar-group)] e_value:0</td><td>11682.m00189|LOC_Os05g02670|protein kinesin-related protein-like - Arabidopsis thaliana e_value:0</td><td>0.111</td><td>(0.3055 2.7453)</td><td>0.3055</td><td>2.7453</td></tr>
<tr><td>1002</td><td>SET5_K20_CLUSTER_22669</td><td>gi|50905845|ref|XP_464411.1| putative Cgi67 serine protease [Oryza sativa (japonica cultivar-group)] >gi|46390967|dbj|BAD16480.1| putative Cgi67 serine protease [Oryza sativa (japonica cultivar-group)] e_value:0</td><td>11668.m00900|LOC_Os02g09770|protein expressed protein e_value:0</td><td>0.146</td><td>(0.0689 0.4710)</td><td>0.0689</td><td>0.471</td></tr>
<tr><td>1003</td><td>WT008_J12_CLUSTER_21316</td><td>gi|50939937|ref|XP_479496.1| putative acyl-CoA dehydrogenase [Oryza sativa (japonica cultivar-group)] >gnl|BL_ORD_ID|1534211 gi|50509815|dbj|BAD31978.1| putative acyl-CoA dehydrogenase [Oryza sativa (japonica cultivar-group)] >gnl|BL_ORD_ID|1534211</td><td>11673.m04738|LOC_Os07g47820|protein Phosphotransferase enzyme family, putative e_value:0</td><td>0.159</td><td>(0.0784 0.4932)</td><td>0.0784</td><td>0.4932</td></tr>
<tr><td>1004</td><td>WT010_O23_CLUSTER_18300</td><td>gi|2231149|gb|AAB61961.1| integral membrane protein [Oryza sativa] >gnl|BL_ORD_ID|263797 gi|7447786|pir||T03780 probable integral membrane protein - rice e_value:0</td><td>11669.m01049|LOC_Os03g11010|protein probable integral membrane protein - rice e_value:0</td><td>0.106</td><td>(0.0530 0.5000)</td><td>0.053</td><td>0.5</td></tr>
<tr><td>1005</td><td>WT007_M04_CLUSTER_16683</td><td>gi|47834699|gb|AAT39003.1| AML15 [Triticum aestivum] e_value:0</td><td>11668.m04740|LOC_Os02g48790|protein RNA recognition motif 2, putative e_value:0</td><td>0.153</td><td>(0.1094 0.7159)</td><td>0.1094</td><td>0.7159</td></tr>
<tr><td>1006</td><td>WT009_J03_CLUSTER_24791</td><td>gi|52076693|dbj|BAD45606.1| putative receptor-mediated endocytosis 1 isoform I [Oryza sativa (japonica cultivar-group)] >gnl|BL_ORD_ID|379067 gi|52077029|dbj|BAD46062.1| putative receptor-mediated endocytosis 1 isoform I [Oryza sativa (japonica cultiva</td><td>11680.m04677|LOC_Os06g47330|protein EF hand, putative e_value:0</td><td>0.095</td><td>(0.0488 0.5139)</td><td>0.0488</td><td>0.5139</td></tr>
<tr><td>1007</td><td>SET6_K04_CLUSTER_26767</td><td>gi|50912877|ref|XP_467846.1| putative annexin P35 [Oryza sativa (japonica cultivar-group)] >gi|46805936|dbj|BAD17230.1| putative annexin P35 [Oryza sativa (japonica cultivar-group)] >gi|46390136|dbj|BAD15571.1| putative annexin P35 [Oryza sativa (ja</td><td>11668.m05056|LOC_Os02g51750|protein Annexin, putative e_value:1.49659e-159</td><td>0.151</td><td>(0.0743 0.4923)</td><td>0.0743</td><td>0.4923</td></tr>
<tr><td>1008</td><td>WT007_P14_CLUSTER_31751</td><td>gi|34907434|ref|NP_915064.1| P0018C10.44 [Oryza sativa (japonica cultivar-group)] >gnl|BL_ORD_ID|1558774 gi|21952826|dbj|BAC06242.1| putative F-box protein Fbl2 [Oryza sativa (japonica cultivar-group)] >gnl|BL_ORD_ID|1558774</td><td>11667.m06623|LOC_Os01g65920|protein Leucine Rich Repeat, putative e_value:0</td><td>0.151</td><td>(0.0828 0.5498)</td><td>0.0828</td><td>0.5498</td></tr>
<tr><td>1009</td><td>WT009_K08_CLUSTER_33135</td><td>gi|50948755|ref|XP_493735.1| putative early nodulin [Oryza sativa (japonica cultivar-group)] >gnl|BL_ORD_ID|1752622 gi|5803258|dbj|BAA83568.1| putative early nodulin [Oryza sativa (japonica cultivar-group)] e_value:8.43263e-41</td><td>11680.m00428|LOC_Os06g05020|protein Early nodulin 93 ENOD93 protein, putative e_value:9.12057e-43</td><td>0.196</td><td>(0.1230 0.6285)</td><td>0.123</td><td>0.6285</td></tr>
<tr><td>1010</td><td>WT005_K16_CLUSTER_19486</td><td>gi|50917343|ref|XP_469068.1| hypothetical protein [Oryza sativa (japonica cultivar-group)] >gnl|BL_ORD_ID|1585108 gi|50582753|gb|AAT78823.1| hypothetical protein [Oryza sativa (japonica cultivar-group)] >gnl|BL_ORD_ID|1585108 gi|29244630|gb|AAO73223</td><td>11686.m04279|LOC_Os12g42860|protein expressed protein e_value:5.61334e-89</td><td>0.39</td><td>(0.3052 0.7817)</td><td>0.3052</td><td>0.7817</td></tr>
<tr><td>1011</td><td>SET3_H05_CLUSTER_32621</td><td>gi|31433080|gb|AAP54640.1| putative GTPase activating protein [Oryza sativa (japonica cultivar-group)] >gi|37536102|ref|NP_922353.1| putative GTPase activating protein [Oryza sativa (japonica cultivar-group)] >gi|13786461|gb|AAK39586.1| putative GTP</td><td>11676.m03376|LOC_Os10g37410|protein putative GTPase activating protein e_value:0</td><td>0.218</td><td>(0.1202 0.5518)</td><td>0.1202</td><td>0.5518</td></tr>
<tr><td>1012</td><td>WT007_H07_CLUSTER_5617</td><td>gi|53793465|dbj|BAD53225.1| myosin heavy chain-like [Oryza sativa (japonica cultivar-group)] e_value:0</td><td>11667.m03861|LOC_Os01g40200|protein DIL domain, putative e_value:0</td><td>0.211</td><td>(0.1482 0.7035)</td><td>0.1482</td><td>0.7035</td></tr>
<tr><td>1013</td><td>WT007__C14_CLUSTER_111</td><td>gi|22758281|gb|AAN05509.1| Unknown protein [Oryza sativa (japonica cultivar-group)] e_value:9.27794e-120</td><td>11669.m00348|LOC_Os03g04110|protein LysM domain, putative e_value:2.5308e-121</td><td>0.637</td><td>(0.2390 0.3754)</td><td>0.239</td><td>0.3754</td></tr>
<tr><td>1014</td><td>WT013_M03_CLUSTER_11037</td><td>gi|55733917|gb|AAV59424.1| putative deoxyribonucleoside kinase [Oryza sativa (japonica cultivar-group)] >gnl|BL_ORD_ID|1741334 gi|50931485|ref|XP_475270.1| putative deoxyribonucleoside kinase [Oryza sativa (japonica cultivar-group)] e_value:3.70867e-17</td><td>11667.m06566|LOC_Os01g65350|protein Deoxynucleoside kinase, putative e_value:0</td><td>0.239</td><td>(0.1309 0.5475)</td><td>0.1309</td><td>0.5475</td></tr>
<tr><td>1015</td><td>SET3_K04_CLUSTER_5265</td><td>gi|22655232|gb|AAM98206.1| unknown protein [Arabidopsis thaliana] >gi|28059472|gb|AAO30061.1| unknown protein [Arabidopsis thaliana] >gi|10177623|dbj|BAB10770.1| unnamed protein product [Arabidopsis thaliana] >gi|15240508|ref|NP_199768.1| transgl</td><td>11673.m03000|LOC_Os07g31460|protein Transglutaminase-like superfamily, putative e_value:0</td><td>0.256</td><td>(0.1166 0.4556)</td><td>0.1166</td><td>0.4556</td></tr>
<tr><td>1016</td><td>WT013_F22_CLUSTER_34749</td><td>gi|50944215|ref|XP_481635.1| putative glycosyltransferase [Oryza sativa (japonica cultivar-group)] >gnl|BL_ORD_ID|1605309 gi|38637194|dbj|BAD03445.1| putative glycosyltransferase [Oryza sativa (japonica cultivar-group)] >gnl|BL_ORD_ID|1605309 gi|384</td><td>11674.m02309|LOC_Os08g23780|protein Glycosyl transferase family 8, putative e_value:0</td><td>0.275</td><td>(0.1347 0.4902)</td><td>0.1347</td><td>0.4902</td></tr>
<tr><td>1017</td><td>WT011_E06_CLUSTER_10546</td><td>gi|56784642|dbj|BAD81689.1| putative protein kinase (AME2/AFC1) [Oryza sativa (japonica cultivar-group)] e_value:0</td><td>11667.m06200|LOC_Os01g62080|protein Protein kinase domain, putative e_value:0</td><td>0.074</td><td>(0.0376 0.5059)</td><td>0.0376</td><td>0.5059</td></tr>
<tr><td>1018</td><td>WT009_D04_CLUSTER_33498</td><td>gi|50918691|ref|XP_469742.1| putative growth inhibitory protein [Oryza sativa] >gnl|BL_ORD_ID|1539251 gi|18087670|gb|AAL58962.1| putative growth inhibitory protein [Oryza sativa] e_value:8.99502e-102</td><td>11669.m05439|LOC_Os03g53700|protein putative growth inhibitory protein e_value:2.11459e-126</td><td>0.188</td><td>(0.0845 0.4499)</td><td>0.0845</td><td>0.4499</td></tr>
<tr><td>1019</td><td>SET3_J12_CLUSTER_5974</td><td>gi|50428723|gb|AAT77074.1| expressed protein [Oryza sativa (japonica cultivar-group)] e_value:1.4418e-10</td><td>11669.m05202|LOC_Os03g51530|protein expressed protein e_value:2.22586e-12</td><td>0.748</td><td>(0.3904 0.5216)</td><td>0.3904</td><td>0.5216</td></tr>
<tr><td>1020</td><td>WT009_O08_CLUSTER_6774</td><td>gi|51091148|dbj|BAD35844.1| putative RNA polymerase transcriptional regulation mediator [Oryza sativa (japonica cultivar-group)] e_value:7.2978e-120</td><td>11680.m01107|LOC_Os06g11370|protein MED6 mediator sub complex component, putative e_value:2.0612e-123</td><td>0.161</td><td>(0.0636 0.3945)</td><td>0.0636</td><td>0.3945</td></tr>
<tr><td>1021</td><td>SET4_I16_CLUSTER_15874</td><td>gi|31430161|gb|AAP52113.1| putative AUX1-like permease [Oryza sativa (japonica cultivar-group)] >gi|37531048|ref|NP_919826.1| putative AUX1-like permease [Oryza sativa (japonica cultivar-group)] >gi|15209143|gb|AAK91876.1| Putative AUX1-like permeas</td><td>11676.m00482|LOC_Os10g05690|protein Transmembrane amino acid transporter protein, putative e_value:0</td><td>0.33</td><td>(0.0976 0.2958)</td><td>0.0976</td><td>0.2958</td></tr>
</table>

PDF Complete
Your complimentary use period has ended. Thank you for using PDF Complete.
Click Here to upgrade to Unlimited Pages and Expanded Features

<table>
<tr><th></th><th></th><th></th><th></th><th></th><th></th><th></th><th></th></tr>
<tr><td>1022</td><td>WT010_P05_CLUSTER_7598</td><td>gi|50933321|ref|XP_476188.1| unknown protein [Oryza sativa (japonica cultivar-group)] >gnl|BL_ORD_ID|1600930
gi|46981304|gb|AAT07622.1| unknown protein [Oryza sativa (japonica cultivar-group)] >gnl|BL_ORD_ID|1600930
gi|46981236|gb|AAT07554.1| unknow</td><td>11682.m01223|LOC_Os05g13330|protein F19K19.12 protein - Arabidopsis thaliana e_value:6.79539e-172</td><td>0.148</td><td>(0.0910 0.6160)</td><td>0.091</td><td>0.616</td></tr>
<tr><td>1023</td><td>WT009_F13_CLUSTER_10253</td><td>gi|50932051|ref|XP_475553.1| putative isovaleryl-CoA dehydrogenase [Oryza sativa (japonica cultivar-group)] >gnl|BL_ORD_ID|1596975
gi|47900455|gb|AAT39231.1| putative isovaleryl-CoA dehydrogenase [Oryza sativa (japonica cultivar-group)] >gnl|BL_ORD_</td><td>11682.m00278|LOC_Os05g03480|protein isovaleryl-coa dehydrogenase 2, mitochondrial precursor (ec 1.3.99.10)(ivd 2) (fragment) e_value:0</td><td>0.04</td><td>(0.0216 0.5350)</td><td>0.0216</td><td>0.535</td></tr>
<tr><td>1024</td><td>WT006_G16_CLUSTER_11432</td><td>gi|51964952|ref|XP_507260.1| PREDICTED B1111C03.19 gene product [Oryza sativa (japonica cultivar-group)] >gnl|BL_ORD_ID|1608952
gi|50946569|ref|XP_482812.1| major intrinsic protein-like [Oryza sativa (japonica cultivar-group)] >gnl|BL_ORD_ID|1608952</td><td>11674.m03576|LOC_Os08g36020|protein expressed protein e_value:1.86179e-168</td><td>0.19</td><td>(0.1383 0.7268)</td><td>0.1383</td><td>0.7268</td></tr>
<tr><td>1025</td><td>WT003_G21_CLUSTER_31960</td><td>gi|50252610|dbj|BAD28781.1| putative trehalose-6-phosphate synthase/phosphatase [Oryza sativa (japonica cultivar-group)] e_value:0</td><td>11681.m02088|LOC_Os09g23350|protein trehalose-phosphatase, putative e_value:0</td><td>0.216</td><td>(0.1307 0.6051)</td><td>0.1307</td><td>0.6051</td></tr>
<tr><td>1026</td><td>SET6_E12_CLUSTER_32718</td><td>gi|50913271|ref|XP_468043.1| putative dnaK-type molecular chaperone precursor [Oryza sativa (japonica cultivar-group)]
>gi|46805772|dbj|BAD17140.1| putative dnaK-type molecular chaperone precursor [Oryza sativa (japonica cultivar-group)] e_value:0</td><td>11668.m05243|LOC_Os02g53420|protein chaperone protein DnaK e_value:0</td><td>0.098</td><td>(0.1622 1.6505)</td><td>0.1622</td><td>1.6505</td></tr>
<tr><td>1027</td><td>WT006_H16_CLUSTER_25447</td><td>gi|55908869|gb|AAV67812.1| putative chaperonin [Oryza sativa (japonica cultivar-group)] e_value:0</td><td>11682.m04447|LOC_Os05g46290|protein chaperonin GroEL</td><td>0.129</td><td>(0.0657 0.5076)</td><td>0.0657</td><td>0.5076</td></tr>
<tr><td>1028</td><td>WT007_G04_CLUSTER_34189</td><td>gi|50920113|ref|XP_470417.1| translational elongation factor Tu [Oryza sativa (japonica cultivar-group)] >gnl|BL_ORD_ID|1535151
gi|21685576|gb|AAM74563.1| elongation factor Tu [Oryza sativa] >gnl|BL_ORD_ID|1535151 gi|27573344|gb|AAO20062.1| translat</td><td>11669.m06501|LOC_Os03g63410|protein translational elongation factor Tu e_value:0</td><td>0.136</td><td>(0.0750 0.5523)</td><td>0.075</td><td>0.5523</td></tr>
<tr><td>1029</td><td>WT007_I18_CLUSTER_1255</td><td>gi|50915986|ref|XP_468457.1| putative phosphatidylinositol-4-phosphate 5-kinase [Oryza sativa (japonica cultivar-group)]
>gnl|BL_ORD_ID|1583505 gi|51964552|ref|XP_507061.1| PREDICTED OJ1119_A01.20-1 gene product [Oryza sativa (japonica cultivar-group)]</td><td>11668.m05713|LOC_Os02g57660|protein phosphatidylinositol-4-phosphate 5-kinase, putative e_value:0</td><td>0.082</td><td>(0.0588 0.7130)</td><td>0.0588</td><td>0.713</td></tr>
<tr><td>1030</td><td>SET4_D05_CLUSTER_7651</td><td>gi|34897864|ref|NP_910278.1| selenium-binding protein-like [Oryza sativa (japonica cultivar-group)] >gi|7363276|dbj|BAA93020.1|
selenium-binding protein-like [Oryza sativa (japonica cultivar-group)] e_value:1.06174e-89</td><td>11680.m00118|LOC_Os06g02110|protein F-box domain, putative e_value:2.28785e-91</td><td>0.478</td><td>(0.3742 0.7823)</td><td>0.3742</td><td>0.7823</td></tr>
<tr><td>1031</td><td>WT011_L07_CLUSTER_570</td><td>gi|31433088|gb|AAP54648.1| putative Ste20-related protein kinase [Oryza sativa (japonica cultivar-group)] >gnl|BL_ORD_ID|2012679
gi|37536118|ref|NP_922361.1| putative Ste20-related protein kinase [Oryza sativa (japonica cultivar-group)] >gnl|BL_ORD_</td><td>11669.m00155|LOC_Os03g02320|protein Protein kinase domain, putative e_value:0</td><td>0.186</td><td>(0.1598 0.8578)</td><td>0.1598</td><td>0.8578</td></tr>
<tr><td>1032</td><td>WT005_P24_CLUSTER_33360</td><td>gi|50906477|ref|XP_464727.1| putative protein phosphatase-2C [Oryza sativa (japonica cultivar-group)] >gnl|BL_ORD_ID|1567291
gi|46805642|dbj|BAD17061.1| putative protein phosphatase-2C [Oryza sativa (japonica cultivar-group)] e_value:2.76754e-154</td><td>11668.m01206|LOC_Os02g13100|protein Protein phosphatase 2C, putative e_value:7.07777e-156</td><td>0.31</td><td>(0.1345 0.4339)</td><td>0.1345</td><td>0.4339</td></tr>
<tr><td>1033</td><td>WT013_E09_CLUSTER_19681</td><td>gi|53792985|dbj|BAD54158.1| hypothetical protein [Oryza sativa (japonica cultivar-group)] >gnl|BL_ORD_ID|260613
gi|52077159|dbj|BAD46205.1| hypothetical protein [Oryza sativa (japonica cultivar-group)] e_value:9.84587e-106</td><td>11680.m01464|LOC_Os06g14780|protein hypothetical protein e_value:5.12963e-108</td><td>0.359</td><td>(0.1405 0.3915)</td><td>0.1405</td><td>0.3915</td></tr>
<tr><td>1034</td><td>WT012_G07_CLUSTER_16783</td><td>gi|38346049|emb|CAE02004.2| OJ000223_09.3 [Oryza sativa (japonica cultivar-group)] >gnl|BL_ORD_ID|1523153
gi|38345340|emb|CAE03151.2| OSJNBa0081L15.13 [Oryza sativa (japonica cultivar-group)] >gnl|BL_ORD_ID|1523153
gi|50925273|ref|XP_472937.1| OSJNB</td><td>11670.m03910|LOC_Os04g40830|protein hypothetical protein e_value:0</td><td>0.18</td><td>(0.0961 0.5340)</td><td>0.0961</td><td>0.534</td></tr>
<tr><td>1035</td><td>WT011_N04_CLUSTER_24622</td><td>gi|56202292|dbj|BAD73751.1| unknown protein [Oryza sativa (japonica cultivar-group)] e_value:3.13564e-128</td><td>11667.m04744|LOC_Os01g48830|protein expressed protein e_value:8.30935e-122</td><td>0.355</td><td>(0.1569 0.4420)</td><td>0.1569</td><td>0.442</td></tr>
<tr><td>1036</td><td>WT011_H04_CLUSTER_440</td><td>gi|17104563|gb|AAL34170.1| putative N-hydroxycinnamoyl/benzoyltransferase [Arabidopsis thaliana] >gnl|BL_ORD_ID|1207358
gi|14334526|gb|AAK59460.1| putative N-hydroxycinnamoyl/benzoyltransferase [Arabidopsis thaliana] >gnl|BL_ORD_ID|1207358 gi|306937</td><td>11687.m02848|LOC_Os11g31090|protein N-hydroxycinnamoyl/benzoyltransferase-like protein e_value:0</td><td>0.317</td><td>(0.1211 0.3816)</td><td>0.1211</td><td>0.3816</td></tr>
<tr><td>1037</td><td>WT011_B16_CLUSTER_9868</td><td>gi|34902334|ref|NP_912513.1| Putative serine/threonine protein kinase [Oryza sativa (japonica cultivar-group)] >gnl|BL_ORD_ID|1588677
gi|24421689|gb|AAN60996.1| Putative serine/threonine protein kinase [Oryza sativa (japonica cultivar-group)] e_value:0</td><td>11669.m01230|LOC_Os03g12680|protein Protein kinase domain, putative e_value:0</td><td>0.286</td><td>(0.1365 0.4769)</td><td>0.1365</td><td>0.4769</td></tr>
<tr><td>1038</td><td>WT010_F11_CLUSTER_20514</td><td>gi|50939423|ref|XP_479239.1| putative ankyrin-kinase [Oryza sativa (japonica cultivar-group)] >gnl|BL_ORD_ID|1531465
gi|33146898|dbj|BAC79897.1| putative ankyrin-kinase [Oryza sativa (japonica cultivar-group)] e_value:0</td><td>11673.m04312|LOC_Os07g43900|protein Protein kinase domain, putative e_value:0</td><td>0.107</td><td>(0.0667 0.6235)</td><td>0.0667</td><td>0.6235</td></tr>
<tr><td>1039</td><td>WT009_O16_CLUSTER_8676</td><td>gi|7159851|emb|CAB76380.1| gamma tubulin [Hordeum vulgare subsp. vulgare] e_value:0</td><td>11682.m00596|LOC_Os05g06450|protein tubulin gamma-2 chain (gamma-2 tubulin). [rice e_value:0</td><td>0.041</td><td>(0.0173 0.4224)</td><td>0.0173</td><td>0.4224</td></tr>
<tr><td>1040</td><td>WT009_N10_CLUSTER_23074</td><td>gi|50929009|ref|XP_474032.1| OSJNBa0043L09.28 [Oryza sativa (japonica cultivar-group)] >gnl|BL_ORD_ID|1592695
gi|38344808|emb|CAE03009.2| OSJNBa0043L09.28 [Oryza sativa (japonica cultivar-group)] e_value:0</td><td>11670.m05341|LOC_Os04g54340|protein DNA repair protein (mre11), putative e_value:0</td><td>0.128</td><td>(0.0661 0.5179)</td><td>0.0661</td><td>0.5179</td></tr>
<tr><td>1041</td><td>WT009_K20_CLUSTER_16820</td><td>gi|50910675|ref|XP_466826.1| putative glycogenin 1 [Oryza sativa (japonica cultivar-group)] >gnl|BL_ORD_ID|1582678
gi|48716241|dbj|BAD23777.1| putative glycogenin 1 [Oryza sativa (japonica cultivar-group)] e_value:0</td><td>11668.m03957|LOC_Os02g41520|protein Glycosyl transferase family 8, putative e_value:0</td><td>0.153</td><td>(0.1328 0.8684)</td><td>0.1328</td><td>0.8684</td></tr>
<tr><td>1042</td><td>WT009_K19_CLUSTER_15509</td><td>gi|38605764|emb|CAE05865.3| OSJNBa0044K18.7 [Oryza sativa (japonica cultivar-group)] >gnl|BL_ORD_ID|1523983
gi|50925145|ref|XP_472878.1| OSJNBa0044K18.7 [Oryza sativa (japonica cultivar-group)] e_value:0</td><td>11670.m03833|LOC_Os04g40150|protein expressed protein e_value:0</td><td>0.098</td><td>(0.0591 0.6010)</td><td>0.0591</td><td>0.601</td></tr>
<tr><td>1043</td><td>WT009_B16_CLUSTER_19734</td><td>gi|15219791|ref|NP_171959.1| hypothetical protein [Arabidopsis thaliana] e_value:4.11106e-143</td><td>11669.m00448|LOC_Os03g05040|protein hypothetical protein e_value:0</td><td>0.386</td><td>(0.1840 0.4765)</td><td>0.184</td><td>0.4765</td></tr>
<tr><td>1044</td><td>WT008_L16_CLUSTER_6683</td><td>gi|50938901|ref|XP_478978.1| putative Nt-gh3 deduced protein [Oryza sativa (japonica cultivar-group)] >gnl|BL_ORD_ID|1529960
gi|33146510|dbj|BAC79627.1| putative Nt-gh3 deduced protein [Oryza sativa (japonica cultivar-group)] e_value:0</td><td>11673.m03932|LOC_Os07g40290|protein GH3 auxin-responsive promoter e_value:0</td><td>0.253</td><td>(0.0785 0.3103)</td><td>0.0785</td><td>0.3103</td></tr>
<tr><td>1045</td><td>WT007_E12_CLUSTER_7133</td><td>gi|50916727|ref|XP_468760.1| putative diacylglycerol kinase (with alternative splicing) [Oryza sativa (japonica cultivar-group)]
>gnl|BL_ORD_ID|1540228 gi|41469364|gb|AAS07206.1| putative diacylglycerol kinase (with alternative splicing) [Oryza sativa</td><td>11686.m01193|LOC_Os12g12260|protein diacylglycerol kinase 2 e_value:0</td><td>0.157</td><td>(0.0901 0.5722)</td><td>0.0901</td><td>0.5722</td></tr>
<tr><td>1046</td><td>WT007_D16_CLUSTER_13078</td><td>gi|57863814|gb|AAW56867.1| unkown protein [Oryza sativa (japonica cultivar-group)] e_value:0</td><td>11682.m03838|LOC_Os05g40270|protein Protein kinase domain, putative e_value:0</td><td>0.251</td><td>(0.2112 0.8403)</td><td>0.2112</td><td>0.8403</td></tr>
<tr><td>1047</td><td>WT007_A23_CLUSTER_21534</td><td>gi|18025342|gb|AAK38482.1| beta-D-xylosidase [Hordeum vulgare] e_value:0</td><td>11668.m05043|LOC_Os02g51620|protein beta-D-xylosidase e_value:0</td><td>0.146</td><td>(0.1061 0.7279)</td><td>0.1061</td><td>0.7279</td></tr>
<tr><td>1048</td><td>WT006_P20_CLUSTER_7556</td><td>gi|31430155|gb|AAP52107.1| putative thaumatin-like protein [Oryza sativa (japonica cultivar-group)] >gnl|BL_ORD_ID|2008480
gi|37531036|ref|NP_919820.1| putative thaumatin-like protein [Oryza sativa (japonica cultivar-group)] >gnl|BL_ORD_ID|2008480 g</td><td>11669.m01378|LOC_Os03g14030|protein pathogenesis-related protein F20M13.220 - Arabidopsis thaliana e_value:2.3629e-147</td><td>0.512</td><td>(0.1620 0.3161)</td><td>0.162</td><td>0.3161</td></tr>
<tr><td>1049</td><td>WT006_J19_CLUSTER_7468</td><td>gi|34911404|ref|NP_917049.1| DNA-binding protein RAV2-like [Oryza sativa (japonica cultivar-group)] >gnl|BL_ORD_ID|1547255
gi|18565433|dbj|BAB84620.1| DNA-binding protein RAV1-like [Oryza sativa (japonica cultivar-group)] e_value:6.07846e-169</td><td>11667.m04854|LOC_Os01g49830|protein B3 DNA binding domain, putative e_value:1.88554e-170</td><td>0.253</td><td>(0.0973 0.3838)</td><td>0.0973</td><td>0.3838</td></tr>
<tr><td>1050</td><td>WT005_G12_CLUSTER_761</td><td>gi|37718823|gb|AAR01694.1| ARE1-like protein [Oryza sativa (japonica cultivar-group)] >gnl|BL_ORD_ID|2260593
gi|31126698|gb|AAP44621.1| putative Vps52 / Sac2 family protein [Oryza sativa (japonica cultivar-group)] >gnl|BL_ORD_ID|2260593
gi|50916635|</td><td>11669.m03071|LOC_Os03g30460|protein ARE1-like protein e_value:0</td><td>0.086</td><td>(0.0495 0.5782)</td><td>0.0495</td><td>0.5782</td></tr>
<tr><td>1051</td><td>WT004_N22_CLUSTER_3117</td><td>gi|50905921|ref|XP_464449.1| tubulin-specific chaperone C-like protein [Oryza sativa (japonica cultivar-group)] >gnl|BL_ORD_ID|1566588
gi|51963922|ref|XP_506744.1| PREDICTED OJ1225_F07.5-1 gene product [Oryza sativa (japonica cultivar-group)] >gnl|B</td><td>11668.m00938|LOC_Os02g10130|protein expressed protein e_value:0</td><td>0.15</td><td>(0.0734 0.4890)</td><td>0.0734</td><td>0.489</td></tr>
<tr><td>1052</td><td>WT004_H12_CLUSTER_4327</td><td>gi|42561764|ref|NP_172167.2| carotenoid isomerase, putative [Arabidopsis thaliana] e_value:0</td><td>11687.m03353|LOC_Os11g36440|protein Similar to carotenoid isomerase e_value:0</td><td>0.265</td><td>(0.1478 0.5576)</td><td>0.1478</td><td>0.5576</td></tr>
<tr><td>1053</td><td>WT004_G07_CLUSTER_12656</td><td>gi|50947559|ref|XP_483307.1| putative PSR9 [Oryza sativa (japonica cultivar-group)] >gnl|BL_ORD_ID|1609606
gi|42408795|dbj|BAD10056.1| putative PSR9 [Oryza sativa (japonica cultivar-group)] e_value:0</td><td>11674.m04019|LOC_Os08g40090|protein Leucine Rich Repeat, putative e_value:0</td><td>0.35</td><td>(0.1453 0.4145)</td><td>0.1453</td><td>0.4145</td></tr>
<tr><td>1054</td><td>WT003_I10_CLUSTER_28631</td><td>gi|50939779|ref|XP_479417.1| unknown protein [Oryza sativa (japonica cultivar-group)] >gnl|BL_ORD_ID|1533421
gi|51979054|ref|XP_507404.1| PREDICTED P0496C02.113 gene product [Oryza sativa (japonica cultivar-group)] >gnl|BL_ORD_ID|1533421
gi|51979052</td><td>11673.m04603|LOC_Os07g46670|protein expressed protein e_value:7.09206e-46</td><td>0.619</td><td>(0.2275 0.3677)</td><td>0.2275</td><td>0.3677</td></tr>
<tr><td>1055</td><td>WT002_P17_CLUSTER_22972</td><td>gi|34897052|ref|NP_909872.1| hypothetical protein [Oryza sativa (japonica cultivar-group)] >gnl|BL_ORD_ID|1586602
gi|20303604|gb|AAM19031.1| hypothetical protein [Oryza sativa (japonica cultivar-group)] e_value:5.84748e-33</td><td>11686.m00126|LOC_Os12g02170|protein hypothetical protein e_value:2.58507e-38</td><td>0.417</td><td>(0.7111 1.7052)</td><td>0.7111</td><td>1.7052</td></tr>
<tr><td>1056</td><td>WT002_M02_CLUSTER_2175</td><td>gi|22137024|gb|AAM91357.1| At3g18370/MYF24_8 [Arabidopsis thaliana] >gnl|BL_ORD_ID|1123105 gi|15983787|gb|AAL10490.1|
AT3g18370/MYF24_8 [Arabidopsis thaliana] >gnl|BL_ORD_ID|1123105 gi|18401863|ref|NP_566607.1| C2 domain-containing protein</td><td>11667.m00296|LOC_Os01g03820|protein Similar to AT3g18370/MYF24_8 e_value:0</td><td>0.202</td><td>(0.1209 0.5994)</td><td>0.1209</td><td>0.5994</td></tr>
<tr><td>1057</td><td>WT002_C20_CLUSTER_15302</td><td>gi|51535992|dbj|BAD38072.1| putative Avr9/Cf-9 rapidly elicited protein 264 [Oryza sativa (japonica cultivar-group)] e_value:0</td><td>11681.m02477|LOC_Os09g27010|protein Protein kinase domain, putative e_value:0</td><td>0.123</td><td>(0.1002 0.8176)</td><td>0.1002</td><td>0.8176</td></tr>
<tr><td>1058</td><td>WT002_C04_CLUSTER_26423</td><td>gi|34897446|ref|NP_910069.1| putative conserved oligomeric Golgi complex component [Oryza sativa (japonica cultivar-group)]
>gnl|BL_ORD_ID|1535172 gi|28269403|gb|AAO37946.1| putative conserved oligomeric Golgi complex component [Oryza sativa (japonica</td><td>11669.m06484|LOC_Os03g63250|protein putative conserved oligomeric Golgi complex component e_value:0</td><td>0.17</td><td>(0.0707 0.4161)</td><td>0.0707</td><td>0.4161</td></tr>
<tr><td>1059</td><td>SET6_E18_CLUSTER_28256</td><td>gi|50908589|ref|XP_465783.1| putative ankyrin-like protein [Oryza sativa (japonica cultivar-group)] >gi|47848270|dbj|BAD22094.1|
putative ankyrin-like protein [Oryza sativa (japonica cultivar-group)] e_value:1.27528e-93</td><td>11686.m04045|LOC_Os12g40780|protein ankyrin-like protein-like protein e_value:1.77697e-145</td><td>0.38</td><td>(0.2241 0.5899)</td><td>0.2241</td><td>0.5899</td></tr>
<tr><td>1060</td><td>SET6_C20_CLUSTER_26021</td><td>gi|34908598|ref|NP_915646.1| putative tubby protein [Oryza sativa (japonica cultivar-group)] >gi|21644661|dbj|BAC01219.1| putative
tubby-like protein TULP10 [Oryza sativa (japonica cultivar-group)] e_value:0</td><td>11667.m06487|LOC_Os01g64700|protein Tub family, putative e_value:0</td><td>0.078</td><td>(0.0774 0.9870)</td><td>0.0774</td><td>0.987</td></tr>
<tr><td>1061</td><td>SET5_O24_CLUSTER_21609</td><td>gi|31431627|gb|AAP53378.1| putative disease resistance protein [Oryza sativa (japonica cultivar-group)] >gi|37533578|ref|NP_921091.1|
putative disease resistance protein [Oryza sativa (japonica cultivar-group)] >gi|20043014|gb|AAM08822.1| Putative d</td><td>11687.m02478|LOC_Os11g27430|protein NB-ARC domain, putative e_value:0</td><td>0.347</td><td>(0.4136 1.1909)</td><td>0.4136</td><td>1.1909</td></tr>
<tr><td>1062</td><td>SET5_L24_CLUSTER_7212</td><td>gi|50918197|ref|XP_469495.1| putative basic protein [Oryza sativa] >gi|62733550|gb|AAX95667.1| Plastocyanin-like domain, putative
[Oryza sativa (japonica cultivar-group)] e_value:1.29465e-49</td><td>11669.m05052|LOC_Os03g50160|protein putative basic protein e_value:1.01516e-51</td><td>0.688</td><td>(0.1214 0.1765)</td><td>0.1214</td><td>0.1765</td></tr>
<tr><td>1063</td><td>SET5_C23_CLUSTER_1531</td><td>gi|50901844|ref|XP_463355.1| B1097D05.16 [Oryza sativa (japonica cultivar-group)] e_value:4.55686e-63</td><td>11667.m03840|LOC_Os01g40000|protein Similar to BT005904 At3g53440 e_value:2.50524e-138</td><td>0.513</td><td>(0.3219 0.6270)</td><td>0.3219</td><td>0.627</td></tr>
</table>

PDF Complete
Your complimentary use period has ended. Thank you for using PDF Complete.
Click Here to upgrade to Unlimited Pages and Expanded Features

<table>
<tr><td>1064</td><td>SET4_H08_CLUSTER_17337</td><td>gi|50924119|ref|XP_472420.1| OJ991214_12.7 [Oryza sativa (japonica cultivar-group)] >gi|38344031|emb|CAE01518.2| OJ991214_12.7 [Oryza sativa (japonica cultivar-group)] e_value:1.92671e-76</td><td>11670.m03259|LOC_Os04g33880|protein F10A5.11, putative e_value:8.70273e-75</td><td>0.26</td><td>(0.1217 0.4682)</td><td>0.1217</td><td>0.4682</td></tr>
<tr><td>1065</td><td>SET4_C22_CLUSTER_18249</td><td>gi|34912726|ref|NP_917710.1| putative polygalacturonase [Oryza sativa (japonica cultivar-group)] e_value:0</td><td>11667.m04179|LOC_Os01g43160|protein Polygalacturonase (pectinase) e_value:0</td><td>0.123</td><td>(0.0838 0.6824)</td><td>0.0838</td><td>0.6824</td></tr>
<tr><td>1066</td><td>SET4_C20_CLUSTER_16730</td><td>gi|37575481|gb|AAQ93804.1| ribosomal protein S6 kinase [Zea mays] e_value:0</td><td>11673.m04788|LOC_Os07g48290|protein ribosomal protein S6 kinase homolog (clone Aspk11) - oat e_value:0</td><td>0.167</td><td>(0.0926 0.5539)</td><td>0.0926</td><td>0.5539</td></tr>
<tr><td>1067</td><td>SET3_A18_CLUSTER_21661</td><td>gi|50939471|ref|XP_479263.1| unknown protein [Oryza sativa (japonica cultivar-group)] >gi|33146722|dbj|BAC79527.1| unknown protein [Oryza sativa (japonica cultivar-group)] e_value:0</td><td>11673.m04356|LOC_Os07g44320|protein expressed protein e_value:0</td><td>0.455</td><td>(0.1701 0.3735)</td><td>0.1701</td><td>0.3735</td></tr>
<tr><td>1068</td><td>SET3_C24_CLUSTER_5124</td><td>gi|31433005|gb|AAP54569.1| hypothetical protein [Oryza sativa (japonica cultivar-group)] >gi|37535960|ref|NP_922282.1| hypothetical protein [Oryza sativa (japonica cultivar-group)] >gi|15144302|gb|AAK84443.1| hypothetical protein [Oryza sativa (japo</td><td>11676.m03298|LOC_Os10g36680|protein hypothetical protein e_value:9.68573e-180</td><td>0.163</td><td>(0.0964 0.5902)</td><td>0.0964</td><td>0.5902</td></tr>
<tr><td>1069</td><td>SET2_E15_CLUSTER_21616</td><td>gi|55297350|dbj|BAD69191.1| putative DNA topoisomerase I [Oryza sativa (japonica cultivar-group)] >gi|55297297|dbj|BAD69127.1| putative DNA topoisomerase I [Oryza sativa (japonica cultivar-group)] e_value:0</td><td>11680.m01688|LOC_Os06g16980|protein DNA topoisomerase I, putative e_value:0</td><td>0.247</td><td>(0.1364 0.5517)</td><td>0.1364</td><td>0.5517</td></tr>
<tr><td>1070</td><td>SET1_H14_CLUSTER_17245</td><td>gi|34906648|ref|NP_914671.1| putative NADPH:quinone oxidoreductase [Oryza sativa (japonica cultivar-group)] >gi|15528813|dbj|BAB64708.1| putative NADPH [Oryza sativa (japonica cultivar-group)] e_value:2.71783e-75</td><td>11667.m07267|LOC_Os01g72430|protein NADPH-dependent FMN reductase e_value:6.02026e-77</td><td>0.16</td><td>(0.1758 1.0999)</td><td>0.1758</td><td>1.0999</td></tr>
<tr><td>1071</td><td>SET1_F02_CLUSTER_5118</td><td>gi|50939885|ref|XP_479470.1| hypothetical protein [Oryza sativa (japonica cultivar-group)] >gi|22831136|dbj|BAC15997.1| hypothetical protein [Oryza sativa (japonica cultivar-group)] e_value:8.208e-49</td><td>11669.m02306|LOC_Os03g22410|protein hypothetical protein e_value:3.92586e-64</td><td>0.587</td><td>(0.3053 0.5201)</td><td>0.3053</td><td>0.5201</td></tr>
<tr><td>1072</td><td>SET1_A14_CLUSTER_14376</td><td>gi|51090453|dbj|BAD35423.1| unknown protein [Oryza sativa (japonica cultivar-group)] e_value:4.89525e-111</td><td>11668.m00934|LOC_Os02g10090|protein expressed protein e_value:4.24088e-148</td><td>0.261</td><td>(0.1286 0.4922)</td><td>0.1286</td><td>0.4922</td></tr>
<tr><td>1073</td><td>WT013_N02_CLUSTER_28800</td><td>gi|50944471|ref|XP_481763.1| PPR-repeat protein-like [Oryza sativa (japonica cultivar-group)] >gnl|BL_ORD_ID|1605454 gi|38423978|dbj|BAD01706.1| PPR-repeat protein-like [Oryza sativa (japonica cultivar-group)] >gnl|BL_ORD_ID|1605454 gi|38637396|dbj|</td><td>11674.m02461|LOC_Os08g25280|protein hypothetical protein e_value:0</td><td>0.177</td><td>(0.1346 0.7619)</td><td>0.1346</td><td>0.7619</td></tr>
<tr><td>1074</td><td>WT013_J18_CLUSTER_4329</td><td>gi|52353669|gb|AAU44235.1| hypothetical protein [Oryza sativa (japonica cultivar-group)] e_value:1.33208e-65</td><td>11669.m01886|LOC_Os03g18600|protein hypothetical protein e_value:4.27116e-84</td><td>0.368</td><td>(0.1744 0.4737)</td><td>0.1744</td><td>0.4737</td></tr>
<tr><td>1075</td><td>WT013_G11_CLUSTER_14551</td><td>gi|50901990|ref|XP_463428.1| OJ1116_H09.22 [Oryza sativa (japonica cultivar-group)] e_value:0</td><td>11667.m04906|LOC_Os01g50320|protein Surp module, putative e_value:0</td><td>0.265</td><td>(0.1646 0.6201)</td><td>0.1646</td><td>0.6201</td></tr>
<tr><td>1076</td><td>WT013_F01_CLUSTER_29079</td><td>gi|50510112|dbj|BAD30880.1| putative translation elongation factor EF-1 alpha [Oryza sativa (japonica cultivar-group)] e_value:0</td><td>11673.m00882|LOC_Os07g09400|protein CDS localized after complete sequencing of a cognate cDNA e_value:0</td><td>0.159</td><td>(0.0930 0.5840)</td><td>0.093</td><td>0.584</td></tr>
<tr><td>1077</td><td>WT005_A12_CLUSTER_25153</td><td>gi|9837115|gb|AAG00427.1| germin F [Hordeum vulgare] e_value:2.20639e-123</td><td>11674.m00838|LOC_Os08g08980|protein germin protein type 1 - rice e_value:1.10729e-101</td><td>0.127</td><td>(0.1070 0.8396)</td><td>0.107</td><td>0.8396</td></tr>
<tr><td>1078</td><td>SET1_I09_CLUSTER_33905</td><td>gi|34902528|ref|NP_912610.1| probable germin protein 4 [Oryza sativa (japonica cultivar-group)] >gi|15408833|dbj|BAB64225.1| putative nectarin I [Oryza sativa (japonica cultivar-group)] >gi|13486746|dbj|BAB39980.1| putative nectarin I [Oryza sativa</td><td>11667.m01780|LOC_Os01g18170|protein Cupin, putative e_value:8.84743e-84</td><td>0.184</td><td>(0.1099 0.5967)</td><td>0.1099</td><td>0.5967</td></tr>
<tr><td>1079</td><td>WT004_F23_CLUSTER_6841</td><td>gi|55168301|gb|AAV44167.1| unknown protein [Oryza sativa (japonica cultivar-group)] e_value:6.82363e-37</td><td>11682.m02594|LOC_Os05g27780|protein expressed protein e_value:1.02518e-38</td><td>0.72</td><td>(0.2786 0.3872)</td><td>0.2786</td><td>0.3872</td></tr>
<tr><td>1080</td><td>WT004_C11_CLUSTER_5460</td><td>gi|19851522|gb|AAL99744.1| pyruvate decarboxylase [Zea mays] e_value:0</td><td>11669.m01843|LOC_Os03g18220|protein pyruvate decarboxylase e_value:0</td><td>0.088</td><td>(0.0386 0.4381)</td><td>0.0386</td><td>0.4381</td></tr>
<tr><td>1081</td><td>WT008_D22_CLUSTER_14981</td><td>gi|50915536|ref|XP_468232.1| MutT/nudix protein-like [Oryza sativa (japonica cultivar-group)] >gnl|BL_ORD_ID|1583353 gi|47497142|dbj|BAD19191.1| MutT/nudix protein-like [Oryza sativa (japonica cultivar-group)] >gnl|BL_ORD_ID|1583353 gi|47497589|dbj|</td><td>11668.m05423|LOC_Os02g55030|protein hydrolase, NUDIX family, putative e_value:0</td><td>0.145</td><td>(0.0582 0.4001)</td><td>0.0582</td><td>0.4001</td></tr>
<tr><td>1082</td><td>SET3_B02_CLUSTER_20060</td><td>gi|7573472|emb|CAB87786.1| putative protein [Arabidopsis thaliana] >gi|11357550|pir||T48620 hypothetical protein F18O22.260 - Arabidopsis thaliana e_value:6.04405e-141</td><td>11687.m01055|LOC_Os11g11060|protein At3g01640/F4P13_18 e_value:5.39363e-151</td><td>0.101</td><td>(0.1625 1.6045)</td><td>0.1625</td><td>1.6045</td></tr>
<tr><td>1083</td><td>WT007_N05_CLUSTER_34769</td><td>gi|469148|emb|CAA81231.1| alanine aminotransferase [Hordeum vulgare subsp. vulgare] >gnl|BL_ORD_ID|2323723 gi|629770|pir||S42535 alanine transaminase (EC 2.6.1.2) - barley >gnl|BL_ORD_ID|2323723 gi|1703227|sp|P52894|ALA2_HORVU Alanine aminotransfera</td><td>11676.m02126|LOC_Os10g25130|protein alanine aminotransferase e_value:0</td><td>0.118</td><td>(0.0547 0.4630)</td><td>0.0547</td><td>0.463</td></tr>
<tr><td>1084</td><td>WT002_M17_CLUSTER_34769</td><td>gi|469148|emb|CAA81231.1| alanine aminotransferase [Hordeum vulgare subsp. vulgare] >gnl|BL_ORD_ID|2323723 gi|629770|pir||S42535 alanine transaminase (EC 2.6.1.2) - barley >gnl|BL_ORD_ID|2323723 gi|1703227|sp|P52894|ALA2_HORVU Alanine aminotransfera</td><td>11676.m02126|LOC_Os10g25130|protein alanine aminotransferase e_value:0</td><td>0.122</td><td>(0.0558 0.4567)</td><td>0.0558</td><td>0.4567</td></tr>
<tr><td>1085</td><td>SET3_E20_CLUSTER_16252</td><td>gi|34896124|ref|NP_909406.1| putative Ca(2+)-dependent nuclease [Oryza sativa (japonica cultivar-group)] >gi|15528760|dbj|BAB64802.1| putative Ca(2+)-dependent nuclease [Oryza sativa (japonica cultivar-group)] e_value:1.09935e-135</td><td>11667.m00656|LOC_Os01g07200|protein Staphylococcal nuclease homologue, putative e_value:3.14731e-137</td><td>0.137</td><td>(0.1598 1.1620)</td><td>0.1598</td><td>1.162</td></tr>
<tr><td>1086</td><td>WT004_O21_CLUSTER_22654</td><td>gi|34909872|ref|NP_916283.1| putative Regulatory protein NPR1 (Nonexpresser of PR genes 1) [Oryza sativa (japonica cultivar-group)] e_value:0</td><td>11667.m05554|LOC_Os01g56200|protein expressed protein e_value:0</td><td>0.168</td><td>(0.1100 0.6543)</td><td>0.11</td><td>0.6543</td></tr>
<tr><td>1087</td><td>WT008_B07_CLUSTER_4088</td><td>gi|57899511|dbj|BAD86973.1| putative trehalose-6-phosphate synthase/phosphatase [Oryza sativa (japonica cultivar-group)] e_value:0</td><td>11667.m05377|LOC_Os01g54560|protein trehalose-phosphatase, putative e_value:0</td><td>0.11</td><td>(0.0618 0.5643)</td><td>0.0618</td><td>0.5643</td></tr>
<tr><td>1088</td><td>SET2_O04_CLUSTER_9312</td><td>gi|50943587|ref|XP_481321.1| unknown protein [Oryza sativa (japonica cultivar-group)] >gi|38175670|dbj|BAD01375.1| unknown protein [Oryza sativa (japonica cultivar-group)] >gi|38175590|dbj|BAD01299.1| unknown protein [Oryza sativa (japonica cultivar</td><td>11674.m01854|LOC_Os08g19320|protein Similar to At5g11030 e_value:9.7042e-178</td><td>0.47</td><td>(0.2545 0.5414)</td><td>0.2545</td><td>0.5414</td></tr>
<tr><td>1089</td><td>WT011_B20_CLUSTER_4776</td><td>gi|49615735|gb|AAT67049.1| germin-like protein 4 [Triticum monococcum] e_value:1.42394e-123</td><td>11674.m00839|LOC_Os08g08990|protein Cupin, putative e_value:2.9775e-110</td><td>0.08</td><td>(0.0754 0.9430)</td><td>0.0754</td><td>0.943</td></tr>
<tr><td>1090</td><td>WT006_E05_CLUSTER_32022</td><td>gi|53793241|dbj|BAD54466.1| putative Exo70 exocyst complex subunit [Oryza sativa (japonica cultivar-group)] >gnl|BL_ORD_ID|260828 gi|53792078|dbj|BAD54663.1| putative Exo70 exocyst complex subunit [Oryza sativa (japonica cultivar-group)] e_value:0</td><td>11680.m04784|LOC_Os06g48330|protein Exo70 exocyst complex subunit e_value:0</td><td>0.199</td><td>(0.1065 0.5362)</td><td>0.1065</td><td>0.5362</td></tr>
<tr><td>1091</td><td>WT004_A24_CLUSTER_19903</td><td>gi|50878327|gb|AAT85102.1| unknown protein [Oryza sativa (japonica cultivar-group)] e_value:2.33715e-105</td><td>11682.m04652|LOC_Os05g48590|protein AUX/IAA family e_value:3.99446e-104</td><td>0.241</td><td>(0.1356 0.5617)</td><td>0.1356</td><td>0.5617</td></tr>
<tr><td>1092</td><td>WT012_A05_CLUSTER_20702</td><td>gi|40538974|gb|AAR87231.1| hypothetical protein [Oryza sativa (japonica cultivar-group)] >gnl|BL_ORD_ID|2058584 gi|50901354|ref|XP_463110.1| hypothetical protein [Oryza sativa (japonica cultivar-group)] e_value:0</td><td>11686.m03869|LOC_Os12g39160|protein expressed protein e_value:0</td><td>0.095</td><td>(0.0561 0.5896)</td><td>0.0561</td><td>0.5896</td></tr>
<tr><td>1093</td><td>SET4_H21_CLUSTER_3701</td><td>gi|50940385|ref|XP_479720.1| putative alcohol dehydrogenase (Zn containing)(adh-1) [Oryza sativa (japonica cultivar-group)] >gi|42408374|dbj|BAD09525.1| putative alcohol dehydrogenase (Zn containing)(adh-1) [Oryza sativa (japonica cultivar-group)] e_va</td><td>11674.m00083|LOC_Os08g01760|protein oxidoreductase, zinc-binding dehydrogenase family e_value:0</td><td>0.083</td><td>(0.0449 0.5422)</td><td>0.0449</td><td>0.5422</td></tr>
<tr><td>1094</td><td>WT003_M19_CLUSTER_14600</td><td>gi|38345468|emb|CAE01686.2| OSJNBa0010H02.6 [Oryza sativa (japonica cultivar-group)] >gnl|BL_ORD_ID|1522449 gi|50927809|ref|XP_473432.1| OSJNBa0010H02.6 [Oryza sativa (japonica cultivar-group)] e_value:0</td><td>11670.m04542|LOC_Os04g46620|protein t-complex polypeptide 1 e_value:0</td><td>0.062</td><td>(0.0384 0.6221)</td><td>0.0384</td><td>0.6221</td></tr>
<tr><td>1095</td><td>WT013_F02_CLUSTER_16126</td><td>gi|34904402|ref|NP_913548.1| unnamed protein product [Oryza sativa (japonica cultivar-group)] >gnl|BL_ORD_ID|1752211 gi|8468007|dbj|BAA96608.1| unknown protein [Oryza sativa (japonica cultivar-group)] >gnl|BL_ORD_ID|1752211</td><td>11667.m01322|LOC_Os01g13420|protein SOUL heme-binding protein e_value:5.78335e-105</td><td>0.14</td><td>(0.0816 0.5839)</td><td>0.0816</td><td>0.5839</td></tr>
<tr><td>1096</td><td>WT003_D10_CLUSTER_24679</td><td>gi|50253014|dbj|BAD29264.1| putative CCR4-NOT transcription complex subunit 7 [Oryza sativa (japonica cultivar-group)] >gnl|BL_ORD_ID|229606 gi|50251550|dbj|BAD28924.1| putative CCR4-NOT transcription complex subunit 7 [Oryza sativa (japonica</td><td>11681.m02258|LOC_Os09g24990|protein CCR4-associated factor, putative e_value:2.89003e-148</td><td>0.059</td><td>(0.0584 0.9885)</td><td>0.0584</td><td>0.9885</td></tr>
<tr><td>1097</td><td>WT008_L24_CLUSTER_15512</td><td>gi|50910377|ref|XP_466677.1| putative pentatricopeptide (PPR) repeat-containing protein [Oryza sativa (japonica cultivar-group)] >gnl|BL_ORD_ID|1578108 gi|47497609|dbj|BAD19678.1| putative pentatricopeptide (PPR) repeat-containing protein [Oryza sativa</td><td>11668.m03826|LOC_Os02g39820|protein expressed protein e_value:0</td><td>0.354</td><td>(0.1806 0.5100)</td><td>0.1806</td><td>0.51</td></tr>
<tr><td>1098</td><td>WT002_P24_CLUSTER_15103</td><td>gi|52076652|dbj|BAD45552.1| unknown protein [Oryza sativa (japonica cultivar-group)] e_value:4.43366e-28</td><td>11680.m04637|LOC_Os06g46970|protein expressed protein e_value:5.34208e-30</td><td>0.338</td><td>(0.1485 0.4392)</td><td>0.1485</td><td>0.4392</td></tr>
<tr><td>1099</td><td>WT004_K24_CLUSTER_33583</td><td>gi|34902812|ref|NP_912753.1| unnamed protein product [Oryza sativa (japonica cultivar-group)] >gnl|BL_ORD_ID|1752269 gi|7106529|dbj|BAA92214.1| putative protein kinase [Oryza sativa (japonica cultivar-group)] >gnl|BL_ORD_ID|1752269 gi|13620845|dbj|B</td><td>11667.m01479|LOC_Os01g14860|protein Protein kinase domain, putative e_value:0</td><td>0.056</td><td>(0.0281 0.4972)</td><td>0.0281</td><td>0.4972</td></tr>
<tr><td>1100</td><td>WT012_J23_CLUSTER_25838</td><td>gi|34895600|ref|NP_909148.1| OSJNBa0083M16.12 [Oryza sativa (japonica cultivar-group)] e_value:0</td><td>11667.m00331|LOC_Os01g04160|protein expressed protein</td><td>0.304</td><td>(0.2918 0.9608)</td><td>0.2918</td><td>0.9608</td></tr>
<tr><td>1101</td><td>WT006_E04_CLUSTER_9561</td><td>gi|57900192|dbj|BAD88277.1| splicing factor family protein-like [Oryza sativa (japonica cultivar-group)] >gnl|BL_ORD_ID|972542 gi|57900079|dbj|BAD88141.1| splicing factor family protein-like [Oryza sativa (japonica cultivar-group)] e_value:2.45971e-63</td><td>11669.m00389|LOC_Os03g04520|protein U2af2 protein e_value:0</td><td>0.385</td><td>(0.2265 0.5885)</td><td>0.2265</td><td>0.5885</td></tr>
<tr><td>1102</td><td>SET3_A09_CLUSTER_29018</td><td>gi|56785336|dbj|BAD82295.1| unknown protein [Oryza sativa (japonica cultivar-group)] >gi|15128395|dbj|BAB62581.1| unknown protein [Oryza sativa (japonica cultivar-group)] e_value:0</td><td>11667.m05988|LOC_Os01g60200|protein hypothetical protein e_value:0</td><td>0.116</td><td>(0.0657 0.5657)</td><td>0.0657</td><td>0.5657</td></tr>
<tr><td>1103</td><td>WT004_A18_CLUSTER_25864</td><td>gi|50945765|ref|XP_482410.1| putative RNA-binding region RNP-1 and Splicing factor PWI family member protein [Oryza sativa (japonica cultivar-group)] >gnl|BL_ORD_ID|1606325 gi|37573054|dbj|BAC98582.1| putative RNA-binding region RNP-1 and Splicing fact</td><td>11674.m03164|LOC_Os08g32100|protein transposon protein, putative, CACTA, En/Spm sub-class e_value:0</td><td>0.24</td><td>(0.1516 0.6306)</td><td>0.1516</td><td>0.6306</td></tr>
<tr><td>1104</td><td>WT011_P19_CLUSTER_1221</td><td>gi|52077280|dbj|BAD46322.1| putative Protein kinase G11A [Oryza sativa (japonica cultivar-group)] >gnl|BL_ORD_ID|381823 gi|52077355|dbj|BAD46396.1| putative Protein kinase G11A [Oryza sativa (japonica cultivar-group)] e_value:0</td><td>11681.m02816|LOC_Os09g30150|protein Protein kinase domain, putative e_value:0</td><td>0.101</td><td>(0.0853 0.8407)</td><td>0.0853</td><td>0.8407</td></tr>
<tr><td>1105</td><td>WT008_A03_CLUSTER_14055</td><td>gi|38345315|emb|CAE03385.2| OSJNBa0004N05.9 [Oryza sativa (japonica cultivar-group)] >gnl|BL_ORD_ID|1522112 gi|50926396|ref|XP_473145.1| OSJNBa0004N05.9 [Oryza sativa (japonica cultivar-group)] e_value:0</td><td>11670.m04175|LOC_Os04g43220|protein Zinc finger, C3HC4 type (RING finger), putative e_value:0</td><td>0.468</td><td>(0.2458 0.5249)</td><td>0.2458</td><td>0.5249</td></tr>
<tr><td>1106</td><td>WT007_C01_CLUSTER_325</td><td>gi|56784949|dbj|BAD82479.1| wall-associated kinase 4-like [Oryza sativa (japonica cultivar-group)] e_value:0</td><td>11667.m04818|LOC_Os01g49520|protein Protein kinase domain, putative e_value:0</td><td>0.473</td><td>(0.2088 0.4418)</td><td>0.2088</td><td>0.4418</td></tr>
<tr><td>1107</td><td>WT006_J22_CLUSTER_8123</td><td>gi|3777600|gb|AAC64970.1| 50S ribosomal protein L5 [Oryza sativa] >gnl|BL_ORD_ID|451824 gi|50400801|sp|Q9ZST0|RK5_ORYSA 50S ribosomal protein L5, chloroplast precursor e_value:5.52904e-126</td><td>11669.m00267|LOC_Os03g03360|protein ribosomal L5P family C-terminus, putative e_value:1.45709e-128</td><td>0.22</td><td>(0.0869 0.3950)</td><td>0.0869</td><td>0.395</td></tr>
<tr><td>1108</td><td>SET3_D02_CLUSTER_3025</td><td>gi|50945535|ref|XP_482295.1| putative 50S ribosomal protein L27 [Oryza sativa (japonica cultivar-group)] >gi|50945533|ref|XP_482294.1| putative 50S ribosomal protein L27 [Oryza sativa (japonica cultivar-group)] >gi|37806124|dbj|BAC99573.1| putative</td><td>11674.m03075|LOC_Os08g31230|protein ribosomal protein L27, putative e_value:4.59535e-70</td><td>0.181</td><td>(0.0792 0.4369)</td><td>0.0792</td><td>0.4369</td></tr>
</table>

PDF Complete
Your complimentary use period has ended. Thank you for using PDF Complete.
Click Here to upgrade to Unlimited Pages and Expanded Features

<table>
<tr><td></td><td></td><td></td><td></td><td></td><td></td><td></td><td></td></tr>
<tr><td>1109</td><td>WT006_E24_CLUSTER_33069</td><td>gi|52076246|dbj|BAD45014.1| egalitarian-like [Oryza sativa (japonica cultivar-group)] e_value:9.20348e-163</td><td>11667.m03695|LOC_Os01g38620|protein KH domain, putative e_value:2.82835e-164</td><td>0.116</td><td>(0.0466 0.4021)</td><td>0.0466</td><td>0.4021</td></tr>
<tr><td>1110</td><td>WT005_D05_CLUSTER_4977</td><td>gi|13021690|gb|AAK11516.1| DNA cytosine methyltransferase MET2a [Zea mays] e_value:0</td><td>11676.m00060|LOC_Os10g01570|protein Putative DNA cytosine methyltransferase MET2a e_value:0</td><td>0.161</td><td>(0.1711 1.0610)</td><td>0.1711</td><td>1.061</td></tr>
<tr><td>1111</td><td>WT005_I06_CLUSTER_9409</td><td>gi|51091378|dbj|BAD36111.1| putative BRI1-KD interacting protein 118 [Oryza sativa (japonica cultivar-group)] e_value:4.90169e-137</td><td>11680.m03945|LOC_Os06g40450|protein Targeting protein for Xklp2 (TPX2), putative e_value:1.18772e-140</td><td>0.217</td><td>(0.1376 0.6332)</td><td>0.1376</td><td>0.6332</td></tr>
<tr><td>1112</td><td>WT013_B06_CLUSTER_4450</td><td>gi|50918225|ref|XP_469509.1| putative CCAAT displacement protein [Oryza sativa] >gnl|BL_ORD_ID|1537018 gi|62733538|gb|AAX95655.1| CASP C terminal, putative [Oryza sativa (japonica cultivar-group)] e_value:0</td><td>11669.m05066|LOC_Os03g50300|protein putative CCAAT displacement protein e_value:0</td><td>0.076</td><td>(0.0353 0.4678)</td><td>0.0353</td><td>0.4678</td></tr>
<tr><td>1113</td><td>WT009_P09_CLUSTER_6623</td><td>gi|55167932|gb|AAV43801.1| unknown protein [Oryza sativa (japonica cultivar-group)] e_value:0</td><td>11682.m02603|LOC_Os05g27870|protein expressed protein</td><td>0.198</td><td>(0.0959 0.4851)</td><td>0.0959</td><td>0.4851</td></tr>
<tr><td>1114</td><td>SET5_I10_CLUSTER_32441</td><td>gi|14626935|gb|AAK70805.1| leucine-rich repeat resistance protein-like protein [Gossypium hirsutum] e_value:5.90969e-140</td><td>11669.m01084|LOC_Os03g11340|protein leucine-rich repeat resistance protein-like protein e_value:1.0316e-174</td><td>0.103</td><td>(0.0569 0.5541)</td><td>0.0569</td><td>0.5541</td></tr>
<tr><td>1115</td><td>WT006_B01_CLUSTER_34362</td><td>gi|50918191|ref|XP_469492.1| putative glutathione S-transferase [Oryza sativa] e_value:2.88593e-68</td><td>11669.m05049|LOC_Os03g50130|protein putative glutathione S-transferase e_value:4.76596e-70</td><td>0.372</td><td>(0.1003 0.2692)</td><td>0.1003</td><td>0.2692</td></tr>
<tr><td>1116</td><td>WT005_E01_CLUSTER_32035</td><td>gi|50947995|ref|XP_483525.1| putative DHHC-type zinc finger domain-containing protein [Oryza sativa (japonica cultivar-group)] >gnl|BL_ORD_ID|1609852 gi|45736080|dbj|BAD13105.1| putative DHHC-type zinc finger domain-containing protein [Oryza sativa (ja</td><td>11681.m03023|LOC_Os09g33450|protein DHHC zinc finger domain, putative e_value:0</td><td>0.086</td><td>(0.0515 0.5975)</td><td>0.0515</td><td>0.5975</td></tr>
<tr><td>1117</td><td>WT009_O15_CLUSTER_18461</td><td>gi|56784547|dbj|BAD82809.1| putative MTD2 [Oryza sativa (japonica cultivar-group)] e_value:9.73689e-134</td><td>11667.m06836|LOC_Os01g67850|protein Zinc finger, C3HC4 type (RING finger), putative e_value:1.82196e-135</td><td>0.075</td><td>(0.0424 0.5643)</td><td>0.0424</td><td>0.5643</td></tr>
<tr><td>1118</td><td>WT010_L23_CLUSTER_24710</td><td>gi|37700344|gb|AAR00634.1| unknown protein [Oryza sativa (japonica cultivar-group)] e_value:0</td><td>11669.m03681|LOC_Os03g37330|protein hypothetical protein e_value:0</td><td>0.21</td><td>(0.0880 0.4187)</td><td>0.088</td><td>0.4187</td></tr>
<tr><td>1119</td><td>SET2_B16_CLUSTER_13805</td><td>gi|50906387|ref|XP_464682.1| integral membrane family protein-like [Oryza sativa (japonica cultivar-group)] >gi|51979196|ref|XP_507465.1| PREDICTED P0027A02.30 gene product [Oryza sativa (japonica cultivar-group)]</td><td>11668.m01172|LOC_Os02g12760|protein plant integral membrane protein TIGR01569, putative e_value:1.62539e-70</td><td>0.483</td><td>(0.1872 0.3876)</td><td>0.1872</td><td>0.3876</td></tr>
<tr><td>1120</td><td>WT007_B08_CLUSTER_11224</td><td>gi|37991849|gb|AAR06295.1| hypothetical protein [Oryza sativa (japonica cultivar-group)] e_value:4.88416e-60</td><td>11669.m02972|LOC_Os03g29540|protein expressed protein e_value:9.61006e-62</td><td>0.32</td><td>(0.4047 1.2640)</td><td>0.4047</td><td>1.264</td></tr>
<tr><td>1121</td><td>WT005_B23_CLUSTER_33869</td><td>gi|6850853|emb|CAB71092.1| exonuclease RRP41 [Arabidopsis thaliana] >gnl|BL_ORD_ID|1451464 gi|26452705|dbj|BAC43435.1| putative exonuclease RRP41 [Arabidopsis thaliana] >gnl|BL_ORD_ID|1451464 gi|28973161|gb|AAO63905.1| putative exonuclease RRP41 [Ar</td><td>11681.m00604|LOC_Os09g07820|protein exonuclease RRP41 [imported] - Arabidopsis thaliana e_value:1.89631e-126</td><td>0.091</td><td>(0.0345 0.3771)</td><td>0.0345</td><td>0.3771</td></tr>
<tr><td>1122</td><td>WT013_L08_CLUSTER_32571</td><td>gi|21913313|gb|AAM81209.1| csAtPR5 [Aegilops tauschii] e_value:4.19864e-35</td><td>11687.m00028|LOC_Os11g01280|protein hypothetical protein e_value:5.4826e-36</td><td>0.47</td><td>(0.6789 1.4429)</td><td>0.6789</td><td>1.4429</td></tr>
<tr><td>1123</td><td>WT004_A02_CLUSTER_17179</td><td>gi|54290813|dbj|BAD61452.1| CUL1 [Oryza sativa (japonica cultivar-group)] e_value:0</td><td>11667.m02609|LOC_Os01g27150|protein Putative cullin</td><td>0.025</td><td>(0.0227 0.9107)</td><td>0.0227</td><td>0.9107</td></tr>
<tr><td>1124</td><td>WT012_J12_CLUSTER_34237</td><td>gi|50941339|ref|XP_480197.1| senescence-associated protein-like [Oryza sativa (japonica cultivar-group)] >gnl|BL_ORD_ID|1602358 gi|51964710|ref|XP_507139.1| PREDICTED P0498E12.115 gene product [Oryza sativa (japonica cultivar-group)] >gnl|BL_ORD_ID|</td><td>11674.m00553|LOC_Os08g06250|protein Tetraspanin family, putative e_value:3.13216e-128</td><td>0.197</td><td>(0.0887 0.4512)</td><td>0.0887</td><td>0.4512</td></tr>
<tr><td>1125</td><td>WT007_B14_CLUSTER_32548</td><td>gi|50907147|ref|XP_465062.1| putative WD repeat protein [Oryza sativa (japonica cultivar-group)] >gnl|BL_ORD_ID|1567655 gi|47848311|dbj|BAD22174.1| putative WD repeat protein [Oryza sativa (japonica cultivar-group)] e_value:0</td><td>11668.m01832|LOC_Os02g19210|protein expressed protein e_value:0</td><td>0.191</td><td>(0.0943 0.4930)</td><td>0.0943</td><td>0.493</td></tr>
<tr><td>1126</td><td>SET3_F23_CLUSTER_20167</td><td>gi|50918337|ref|XP_469565.1| putative splicing factor [Oryza sativa (japonica cultivar-group)] >gi|28301939|gb|AAO38832.1| putative splicing factor [Oryza sativa (japonica cultivar-group)] e_value:0</td><td>11669.m05126|LOC_Os03g50850|protein putative splicing factor e_value:0</td><td>0.118</td><td>(0.0646 0.5471)</td><td>0.0646</td><td>0.5471</td></tr>
<tr><td>1127</td><td>SET5_C13_CLUSTER_5619</td><td>gi|50923677|ref|XP_472199.1| OSJNBb0006N15.14 [Oryza sativa (japonica cultivar-group)] >gi|38346119|emb|CAE04597.2| OSJNBb0006N15.14 [Oryza sativa (japonica cultivar-group)] e_value:5.32454e-116</td><td>11681.m02127|LOC_Os09g23720|protein expressed protein e_value:5.76671e-111</td><td>0.332</td><td>(0.1825 0.5495)</td><td>0.1825</td><td>0.5495</td></tr>
<tr><td>1128</td><td>WT008_O23_CLUSTER_4360</td><td>gi|50428684|gb|AAT77035.1| putative oxidoreductase [Oryza sativa (japonica cultivar-group)] e_value:1.5996e-169</td><td>11669.m04180|LOC_Os03g42130|protein putative oxidoreductase e_value:4.36314e-171</td><td>0.307</td><td>(0.0899 0.2927)</td><td>0.0899</td><td>0.2927</td></tr>
<tr><td>1129</td><td>WT008_J02_CLUSTER_10203</td><td>gi|3882018|emb|CAA77151.1| cytokinin oxidase [Zea mays] >gnl|BL_ORD_ID|990746 gi|20138062|sp|Q9T0N8|CKX1_MAIZE Cytokinin dehydrogenase 1 precursor (Cytokinin oxidase 1) (CKO 1) e_value:0</td><td>11667.m00882|LOC_Os01g09260|protein probable cytokinin oxidase precursor (ec 1.4.3.-) (cko). [rice e_value:0</td><td>0.519</td><td>(0.1775 0.3422)</td><td>0.1775</td><td>0.3422</td></tr>
<tr><td>1130</td><td>WT007_A07_CLUSTER_14189</td><td>gi|34902348|ref|NP_912520.1| Hypothetical protein [Oryza sativa (japonica cultivar-group)] >gnl|BL_ORD_ID|1584104 gi|24418029|gb|AAN60479.1| Hypothetical protein [Oryza sativa (japonica cultivar-group)] e_value:3.41945e-32</td><td>11686.m00126|LOC_Os12g02170|protein hypothetical protein e_value:3.81972e-51</td><td>0.336</td><td>(0.5544 1.6489)</td><td>0.5544</td><td>1.6489</td></tr>
<tr><td>1131</td><td>WT012_F17_CLUSTER_20535</td><td>gi|34900336|ref|NP_911514.1| putative acyl carrier protein [Oryza sativa (japonica cultivar-group)] >gnl|BL_ORD_ID|1522929 gi|51978960|ref|XP_507357.1| PREDICTED P0592C06.117 gene product [Oryza sativa (japonica cultivar-group)] >gnl|BL_ORD_ID|15229</td><td>11673.m01166|LOC_Os07g12150|protein acyl carrier protein, putative e_value:8.09244e-59</td><td>0.226</td><td>(0.0608 0.2692)</td><td>0.0608</td><td>0.2692</td></tr>
<tr><td>1132</td><td>WT006_I19_CLUSTER_14560</td><td>gi|50920481|ref|XP_470601.1| Unknown protein [Oryza sativa (japonica cultivar-group)] >gnl|BL_ORD_ID|1588709 gi|20532322|gb|AAM27468.1| Unknown protein [Oryza sativa (japonica cultivar-group)] e_value:8.93237e-77</td><td>11669.m01235|LOC_Os03g12720|protein expressed protein e_value:2.98059e-83</td><td>0.392</td><td>(0.1689 0.4308)</td><td>0.1689</td><td>0.4308</td></tr>
<tr><td>1133</td><td>WT011_D23_CLUSTER_6027</td><td>gi|55701131|tpe|CAH69374.1| TPA: class III peroxidase 132 precursor [Oryza sativa (japonica cultivar-group)] >gnl|BL_ORD_ID|883249 gi|6979323|gb|AAF34416.1| putative peroxidase [Oryza sativa] e_value:3.47914e-146</td><td>11687.m00992|LOC_Os11g10460|protein peroxidase, putative e_value:2.62426e-146</td><td>0.405</td><td>(0.1415 0.3493)</td><td>0.1415</td><td>0.3493</td></tr>
<tr><td>1134</td><td>WT004_D18_CLUSTER_34602</td><td>gi|50917785|ref|XP_469289.1| unknown protein [Oryza sativa (japonica cultivar-group)] >gnl|BL_ORD_ID|1539731 gi|29150403|gb|AAO72412.1| unknown protein [Oryza sativa (japonica cultivar-group)] e_value:1.74103e-113</td><td>11669.m04755|LOC_Os03g47500|protein expressed protein e_value:3.26773e-115</td><td>0.097</td><td>(0.0773 0.8000)</td><td>0.0773</td><td>0.8</td></tr>
<tr><td>1135</td><td>WT007_E07_CLUSTER_34942</td><td>gi|51854365|gb|AAU10745.1| putative zinc transporter [Oryza sativa (japonica cultivar-group)] e_value:0</td><td>11682.m00308|LOC_Os05g03780|protein cation diffusion facilitator family transporter e_value:0</td><td>0.139</td><td>(0.0794 0.5716)</td><td>0.0794</td><td>0.5716</td></tr>
<tr><td>1136</td><td>SET1_K12_CLUSTER_6327</td><td>gi|50937773|ref|XP_478414.1| putative translational inhibitor protein [Oryza sativa (japonica cultivar-group)] >gi|23617012|dbj|BAC20708.1| putative translational inhibitor protein [Oryza sativa (japonica cultivar-group)]</td><td>11673.m03183|LOC_Os07g33240|protein Endoribonuclease L-PSP, putative e_value:2.44229e-62</td><td>0.17</td><td>(0.0818 0.4812)</td><td>0.0818</td><td>0.4812</td></tr>
<tr><td>1137</td><td>WT005_O19_CLUSTER_11449</td><td>gi|27542952|gb|AAO16560.1| mitogen-activated protein kinase [Triticum aestivum] e_value:0</td><td>11680.m00548|LOC_Os06g06090|protein Protein kinase domain, putative e_value:0</td><td>0.056</td><td>(0.0251 0.4458)</td><td>0.0251</td><td>0.4458</td></tr>
<tr><td>1138</td><td>WT011_L17_CLUSTER_34682</td><td>gi|50938011|ref|XP_478533.1| putative 1-acyl-sn-glycerol-3-phosphate acyltransferase [Oryza sativa (japonica cultivar-group)] >gnl|BL_ORD_ID|2128327 gi|27261043|dbj|BAC45159.1| putative 1-acyl-sn-glycerol-3-phosphate acyltransferase [Oryza sativa (japo</td><td>11673.m03342|LOC_Os07g34730|protein Acyltransferase, putative e_value:0</td><td>0.107</td><td>(0.0466 0.4361)</td><td>0.0466</td><td>0.4361</td></tr>
<tr><td>1139</td><td>WT013_L04_CLUSTER_34090</td><td>gi|32693279|gb|AAP81746.1| adenine phosphoribosyltransferase [Triticum aestivum] e_value:2.48317e-112</td><td>11668.m03847|LOC_Os02g40010|protein adenine phosphoribosyltransferase, putative e_value:5.40677e-101</td><td>0.201</td><td>(0.1031 0.5126)</td><td>0.1031</td><td>0.5126</td></tr>
<tr><td>1140</td><td>WT004_L22_CLUSTER_33712</td><td>gi|50934585|ref|XP_476820.1| putative CXC domain protein TSO1 [Oryza sativa (japonica cultivar-group)] >gnl|BL_ORD_ID|1521801 gi|34393829|dbj|BAC83433.1| putative CXC domain protein TSO1 [Oryza sativa (japonica cultivar-group)] e_value:4.35555e-128</td><td>11686.m04094|LOC_Os12g41230|protein DNA binding protein, putative e_value:0</td><td>0.362</td><td>(0.1738 0.4806)</td><td>0.1738</td><td>0.4806</td></tr>
<tr><td>1141</td><td>SET3_K08_CLUSTER_12529</td><td>gi|45510867|gb|AAS67031.1| N-myristoyl transferase [Triticum aestivum] e_value:0</td><td>11667.m04996|LOC_Os01g51170|protein Myristoyl-CoA:protein N-myristoyltransferase, N-terminal domain, putative e_value:0</td><td>0.109</td><td>(0.0406 0.3714)</td><td>0.0406</td><td>0.3714</td></tr>
<tr><td>1142</td><td>WT013_M21_CLUSTER_15659</td><td>gi|38346468|emb|CAD39573.2| OSJNBa0019G23.16 [Oryza sativa (japonica cultivar-group)] >gnl|BL_ORD_ID|1523477 gi|50930127|ref|XP_474591.1| OSJNBa0019G23.16 [Oryza sativa (japonica cultivar-group)] e_value:2.39468e-106</td><td>11668.m05722|LOC_Os02g57740|protein hypothetical protein e_value:2.08104e-84</td><td>0.132</td><td>(0.1382 1.0503)</td><td>0.1382</td><td>1.0503</td></tr>
<tr><td>1143</td><td>SET4_D24_CLUSTER_6708</td><td>gi|50935369|ref|XP_477212.1| hypothetical protein [Oryza sativa (japonica cultivar-group)] >gi|50510006|dbj|BAD30619.1| hypothetical protein [Oryza sativa (japonica cultivar-group)] >gi|33146684|dbj|BAC80079.1| hypothetical protein [Oryza sativa (ja</td><td>11686.m00068|LOC_Os12g01620|protein expressed protein e_value:3.75911e-64</td><td>0.383</td><td>(0.2810 0.7330)</td><td>0.281</td><td>0.733</td></tr>
<tr><td>1144</td><td>WT012_H18_CLUSTER_30730</td><td>gi|50931705|ref|XP_475380.1| putative lipin 2 [Oryza sativa (japonica cultivar-group)] >gnl|BL_ORD_ID|1596216 gi|47900357|gb|AAT39187.1| putative lipin 2 [Oryza sativa (japonica cultivar-group)] >gnl|BL_ORD_ID|1596216</td><td>11682.m03664|LOC_Os05g38710|protein lipin, N-terminal conserved region, putative e_value:0</td><td>0.267</td><td>(0.1764 0.6613)</td><td>0.1764</td><td>0.6613</td></tr>
<tr><td>1145</td><td>SET5_H21_CLUSTER_4416</td><td>gi|50926692|ref|XP_473293.1| OSJNBb0039L24.10 [Oryza sativa (japonica cultivar-group)] >gi|32489827|emb|CAE04571.1| OSJNBb0039L24.10 [Oryza sativa (japonica cultivar-group)] e_value:0</td><td>11670.m04371|LOC_Os04g45040|protein DEAD/DEAH box helicase, putative e_value:0</td><td>0.113</td><td>(0.0529 0.4676)</td><td>0.0529</td><td>0.4676</td></tr>
<tr><td>1146</td><td>WT007_K19_CLUSTER_33582</td><td>gi|14488151|emb|CAC42118.1| flavin containing polyamine oxidase [Hordeum vulgare subsp. vulgare] >gnl|BL_ORD_ID|1334996 gi|14485485|emb|CAC42080.1| polyamine oxidase [Hordeum vulgare subsp. vulgare] e_value:0</td><td>11681.m01823|LOC_Os09g20260|protein Similar to flavin containing polyamine oxidase e_value:0</td><td>0.257</td><td>(0.1405 0.5457)</td><td>0.1405</td><td>0.5457</td></tr>
<tr><td>1147</td><td>WT006_F21_CLUSTER_4306</td><td>gi|50882442|ref|NP_919090.2| blue copper-binding protein-like [Oryza sativa (japonica cultivar-group)] >gnl|BL_ORD_ID|1518631 gi|38175738|dbj|BAC22293.2| blue copper-binding protein-like [Oryza sativa (japonica cultivar-group)] >gnl|BL_ORD_ID|151863</td><td>11673.m00126|LOC_Os07g02200|protein Plastocyanin-like domain, putative e_value:6.89436e-67</td><td>0.938</td><td>(0.1867 0.1990)</td><td>0.1867</td><td>0.199</td></tr>
<tr><td>1148</td><td>WT006_L14_CLUSTER_29205</td><td>gi|55296019|dbj|BAD69163.1| putative Importin 9 [Oryza sativa (japonica cultivar-group)] >gnl|BL_ORD_ID|269626 gi|55295981|dbj|BAD68021.1| putative Importin 9 [Oryza sativa (japonica cultivar-group)] e_value:0</td><td>11680.m01616|LOC_Os06g16280|protein hypothetical protein e_value:0</td><td>0.221</td><td>(0.1017 0.4609)</td><td>0.1017</td><td>0.4609</td></tr>
<tr><td>1149</td><td>WT002_E04_CLUSTER_21974</td><td>gi|50934997|ref|XP_477026.1| putative coatomer protein gamma 2-subunit [Oryza sativa (japonica cultivar-group)] >gnl|BL_ORD_ID|1522560 gi|34394800|dbj|BAC84213.1| putative coatomer protein gamma 2-subunit [Oryza sativa (japonica cultivar-</td><td>11673.m00958|LOC_Os07g10150|protein Adaptin N terminal region, putative e_value:0</td><td>0.042</td><td>(0.0209 0.4944)</td><td>0.0209</td><td>0.4944</td></tr>
<tr><td>1150</td><td>WT012_I21_CLUSTER_10658</td><td>gi|50928719|ref|XP_473887.1| OSJNBa0008M17.11 [Oryza sativa (japonica cultivar-group)] >gnl|BL_ORD_ID|1592568 gi|38344258|emb|CAD41795.2| OSJNBa0008M17.11 [Oryza sativa (japonica cultivar-group)] e_value:0</td><td>11670.m05146|LOC_Os04g52540|protein Piwi domain, putative e_value:0</td><td>0.246</td><td>(0.1788 0.7272)</td><td>0.1788</td><td>0.7272</td></tr>
<tr><td>1151</td><td>WT010_C04_CLUSTER_34489</td><td>gi|50939775|ref|XP_479415.1| putative UDP-GlcNAc:dolichol phosphate N-acetylglucosamine-1-phosphate transferase [Oryza sativa (japonica cultivar-group)] >gnl|BL_ORD_ID|1533419 gi|34395184|dbj|BAC83573.1| putative UDP-GlcNAc:dolichol phosphate N-acetylg</td><td>11673.m04599|LOC_Os07g46640|protein Glycosyl transferase, putative e_value:0</td><td>0.208</td><td>(0.0777 0.3743)</td><td>0.0777</td><td>0.3743</td></tr>
<tr><td>1152</td><td>SET4_P11_CLUSTER_25469</td><td>gi|34897712|ref|NP_910202.1| putative Bplo [Oryza sativa (japonica cultivar-group)] >gi|6907083|dbj|BAA90610.1| putative Bplo [Oryza sativa (japonica cultivar-group)] e_value:0</td><td>11680.m00051|LOC_Os06g01490|protein Multicopper oxidase, putative e_value:0</td><td>0.081</td><td>(0.0879 1.0907)</td><td>0.0879</td><td>1.0907</td></tr>
<tr><td>1153</td><td>SET4_H12_CLUSTER_443</td><td>gi|21592450|gb|AAM64401.1| unknown [Arabidopsis thaliana] >gi|62319335|dbj|BAD94607.1| hypothetical protein [Arabidopsis thaliana] >gi|18411043|ref|NP_565127.1| expressed protein [Arabidopsis thaliana] e_value:2.03213e-11</td><td>11669.m01225|LOC_Os03g12630|protein hypothetical protein e_value:2.96076e-20</td><td>0.196</td><td>(0.3047 1.5557)</td><td>0.3047</td><td>1.5557</td></tr>
</table>

PDF Complete
Your complimentary use period has ended. Thank you for using PDF Complete.
Click Here to upgrade to Unlimited Pages and Expanded Features

<table>
<tr><td>1154</td><td>SET4_G23_CLUSTER_29410</td><td>gi|52353676|gb|AAU44242.1| unknown protein [Oryza sativa (japonica cultivar-group)] e_value:1.63879e-101</td><td>11682.m03767|LOC_Os05g39670|protein PRA1 family protein, putative e_value:3.27555e-103</td><td>0.226</td><td>(0.0651 0.2882)</td><td>0.0651</td><td>0.2882</td></tr>
<tr><td>1155</td><td>WT005_H22_CLUSTER_2959</td><td>gi|50916230|ref|XP_468571.1| Unknown protein [Oryza sativa (japonica cultivar-group)] >gnl|BL_ORD_ID|1584234 gi|24431598|gb|AAN61478.1| Unknown protein [Oryza sativa (japonica cultivar-group)] e_value:2.12957e-56</td><td>11669.m00112|LOC_Os03g01960|protein expressed protein e_value:1.67165e-59</td><td>0.849</td><td>(0.1516 0.1785)</td><td>0.1516</td><td>0.1785</td></tr>
<tr><td>1156</td><td>SET3_L08_CLUSTER_30023</td><td>gi|4678936|emb|CAB41327.1| putative protein [Arabidopsis thaliana] >gi|21537029|gb|AAM61370.1| unknown [Arabidopsis thaliana] >gi|13877705|gb|AAK43930.1| putative protein [Arabidopsis thaliana] >gi|15231123|ref|NP_190775.1| expressed protein [Ara</td><td>11669.m01133|LOC_Os03g11780|protein expressed protein e_value:1.2115e-36</td><td>0.52</td><td>(0.2047 0.3940)</td><td>0.2047</td><td>0.394</td></tr>
<tr><td>1157</td><td>WT013_B15_CLUSTER_3111</td><td>gi|50905339|ref|XP_464158.1| unknown protein [Oryza sativa (japonica cultivar-group)] >gnl|BL_ORD_ID|1565653 gi|45736067|dbj|BAD13093.1| unknown protein [Oryza sativa (japonica cultivar-group)] >gnl|BL_ORD_ID|1565653 gi|45736025|dbj|BAD13052.1| unkn</td><td>11668.m00482|LOC_Os02g05440|protein expressed protein e_value:2.74566e-105</td><td>0.302</td><td>(0.2114 0.7006)</td><td>0.2114</td><td>0.7006</td></tr>
<tr><td>1158</td><td>SET6_K10_CLUSTER_30940</td><td>gi|52077545|dbj|BAD46150.1| unknown protein [Oryza sativa (japonica cultivar-group)] >gi|52076177|dbj|BAD46717.1| unknown protein [Oryza sativa (japonica cultivar-group)] e_value:0</td><td>11667.m05565|LOC_Os01g56300|protein expressed protein e_value:0</td><td>0.198</td><td>(0.0909 0.4584)</td><td>0.0909</td><td>0.4584</td></tr>
<tr><td>1159</td><td>WT007_I05_CLUSTER_25461</td><td>gi|8347248|gb|AAF74568.1| hexose transporter [Zea mays] e_value:0</td><td>11667.m00335|LOC_Os01g04190|protein hexose transporter e_value:0</td><td>0.137</td><td>(0.0695 0.5084)</td><td>0.0695</td><td>0.5084</td></tr>
<tr><td>1160</td><td>SET6_C07_CLUSTER_31498</td><td>gi|1877219|emb|CAA70817.1| serine carboxypeptidase III, CP-MIII [Hordeum vulgare subsp. vulgare] >gi|2851577|sp|P21529|CBP3_HORVU Serine carboxypeptidase III precursor (CP-MIII) e_value:0</td><td>11668.m00138|LOC_Os02g02320|protein serine carboxypeptidase iii precursor (ec 3.4.16.5). e_value:0</td><td>0.136</td><td>(0.0837 0.6140)</td><td>0.0837</td><td>0.614</td></tr>
<tr><td>1161</td><td>WT005_D11_CLUSTER_32337</td><td>gi|31249759|gb|AAP46251.1| putative protein-transport protein [Oryza sativa (japonica cultivar-group)] >gnl|BL_ORD_ID|1999842 gi|34897242|ref|NP_909967.1| putative coat protein complex II (COPII) component [Oryza sativa (japonica cultivar-group)] >g</td><td>11669.m06279|LOC_Os03g61630|protein putative protein-transport protein e_value:3.13378e-149</td><td>0.171</td><td>(0.0906 0.5309)</td><td>0.0906</td><td>0.5309</td></tr>
<tr><td>1162</td><td>WT002_F16_CLUSTER_25595</td><td>gi|50932321|ref|XP_475688.1| 'unknown protein, contains BSD domain' [Oryza sativa (japonica cultivar-group)] >gnl|BL_ORD_ID|1598194 gi|48475068|gb|AAT44137.1| 'unknown protein, contains BSD domain' [Oryza sativa (japonica cultivar-g</td><td>11682.m04141|LOC_Os05g43520|protein BSD domain, putative e_value:1.6799e-170</td><td>0.274</td><td>(0.1569 0.5720)</td><td>0.1569</td><td>0.572</td></tr>
<tr><td>1163</td><td>WT005_N16_CLUSTER_32701</td><td>gi|53793170|dbj|BAD54377.1| asparagine synthetase [Oryza sativa (japonica cultivar-group)] >gnl|BL_ORD_ID|260766 gi|1432054|gb|AAB03991.1| asparagine synthetase >gnl|BL_ORD_ID|260766 gi|3182921|sp|Q43011|ASNS_ORYSA Asparagine synthetase [glutamine-h</td><td>11680.m01528|LOC_Os06g15420|protein asparagine synthase (glutamine-hydrolyzing), putative e_value:0</td><td>0.076</td><td>(0.0423 0.5537)</td><td>0.0423</td><td>0.5537</td></tr>
<tr><td>1164</td><td>SET2_E19_CLUSTER_6805</td><td>gi|47525233|gb|AAT35117.1| respiratory burst oxidase protein B [Oryza sativa (indica cultivar-group)] >gi|53793485|dbj|BAD53393.1| respiratory burst oxidase protein B [Oryza sativa (japonica cultivar-group)] e_value:0</td><td>11667.m02524|LOC_Os01g25820|protein respiratory burst oxidase protein B, putative e_value:0</td><td>0.107</td><td>(0.0776 0.7238)</td><td>0.0776</td><td>0.7238</td></tr>
<tr><td>1165</td><td>SET5_L15_CLUSTER_31661</td><td>gi|34894654|ref|NP_908652.1| P0028G04.28 [Oryza sativa (japonica cultivar-group)] >gi|21104862|dbj|BAB93446.1| putative pectinacetylesterase precursor [Oryza sativa (japonica cultivar-group)] >gi|15128424|dbj|BAB62609.1| putative pectinacetylesteras</td><td>11667.m02093|LOC_Os01g21630|protein Pectinacetylesterase e_value:7.59961e-141</td><td>0.439</td><td>(0.2649 0.6041)</td><td>0.2649</td><td>0.6041</td></tr>
<tr><td>1166</td><td>SET1_N16_CLUSTER_18561</td><td>gi|4007745|emb|CAA65316.1| purothionin [Secale cereale] e_value:1.13675e-13</td><td>11680.m03134|LOC_Os06g32550|protein Plant thionin, putative e_value:3.65731e-06</td><td>0.422</td><td>(0.8890 2.1077)</td><td>0.889</td><td>2.1077</td></tr>
<tr><td>1167</td><td>WT007_K15_CLUSTER_13722</td><td>gi|38345949|emb|CAE04337.2| OSJNBb0038F03.1 [Oryza sativa (japonica cultivar-group)] >gnl|BL_ORD_ID|1523079 gi|38345947|emb|CAE04271.2| OSJNBb0103I08.18 [Oryza sativa (japonica cultivar-group)] >gnl|BL_ORD_ID|1523079 gi|50927259|ref|XP_473377.1| OSJ</td><td>11670.m04468|LOC_Os04g45930|protein Similar to At2g44720/F16B22.21 e_value:0</td><td>0.117</td><td>(0.0575 0.4921)</td><td>0.0575</td><td>0.4921</td></tr>
<tr><td>1168</td><td>SET4_J05_CLUSTER_3200</td><td>gi|22329192|ref|NP_195292.2| glycoside hydrolase family 28 protein / polygalacturonase (pectinase) family protein [Arabidopsis thaliana] e_value:7.41466e-86</td><td>11673.m01021|LOC_Os07g10740|protein Polygalacturonase (pectinase) e_value:7.13337e-83</td><td>0.22</td><td>(0.5739 2.6109)</td><td>0.5739</td><td>2.6109</td></tr>
<tr><td>1169</td><td>SET3_P12_CLUSTER_600</td><td>gi|50252026|dbj|BAD27958.1| putative chemocyanin precursor [Oryza sativa (japonica cultivar-group)] >gi|50251681|dbj|BAD29705.1| putative chemocyanin precursor [Oryza sativa (japonica cultivar-group)] e_value:7.06152e-36</td><td>11668.m01474|LOC_Os02g15710|protein Plastocyanin-like domain, putative e_value:8.36653e-38</td><td>0.523</td><td>(0.3097 0.5925)</td><td>0.3097</td><td>0.5925</td></tr>
<tr><td>1170</td><td>WT013_C15_CLUSTER_15754</td><td>gi|72400|pir||KGZQHL histidine-rich glycoprotein precursor - Plasmodium lophurae >gnl|BL_ORD_ID|411821 gi|9999|emb|CAA25698.1| histidine-rich protein [Plasmodium lophurae] >gnl|BL_ORD_ID|411821 gi|123530|sp|P04929|HRPX_PLALO HISTIDINE-RICH GLYCOPROT</td><td>11670.m03984|LOC_Os04g41480|protein hypothetical protein e_value:5.00525e-39</td><td>0.272</td><td>(0.5014 1.8444)</td><td>0.5014</td><td>1.8444</td></tr>
<tr><td>1171</td><td>WT006_I09_CLUSTER_21569</td><td>gi|51963510|ref|XP_506432.1| PREDICTED OJ1060_D03.106 gene product [Oryza sativa (japonica cultivar-group)] >gnl|BL_ORD_ID|1529822 gi|50938777|ref|XP_478916.1| arm repeat containing protein homolog-like [Oryza sativa (japonica cultivar-</td><td>11673.m03860|LOC_Os07g39590|protein Armadillo/beta-catenin-like repeat, putative e_value:0</td><td>0.152</td><td>(0.0667 0.4386)</td><td>0.0667</td><td>0.4386</td></tr>
<tr><td>1172</td><td>WT006_B20_CLUSTER_21151</td><td>gi|46367508|emb|CAG25776.1| phosphoglucan, water dikinase [Arabidopsis thaliana] e_value:0</td><td>11686.m02011|LOC_Os12g20150|protein Starch binding domain, putative e_value:0</td><td>0.224</td><td>(0.1483 0.6626)</td><td>0.1483</td><td>0.6626</td></tr>
<tr><td>1173</td><td>WT005_H24_CLUSTER_23645</td><td>gi|52077070|dbj|BAD46102.1| DHHC-type zinc finger domain-containing protein -like [Oryza sativa (japonica cultivar-group)] e_value:0</td><td>11681.m03501|LOC_Os09g38970|protein DHHC zinc finger domain, putative e_value:0</td><td>0.124</td><td>(0.0706 0.5671)</td><td>0.0706</td><td>0.5671</td></tr>
<tr><td>1174</td><td>WT004_H03_CLUSTER_5131</td><td>gi|54306075|gb|AAV33309.1| putative SF16 protein [Oryza sativa (japonica cultivar-group)] >gnl|BL_ORD_ID|1431428 gi|57863803|gb|AAS72364.2| putative SF16 protein [Oryza sativa (japonica cultivar-group)] e_value:0</td><td>11682.m00246|LOC_Os05g03190|protein IQ calmodulin-binding motif, putative e_value:0</td><td>0.126</td><td>(0.0817 0.6504)</td><td>0.0817</td><td>0.6504</td></tr>
<tr><td>1175</td><td>WT003_P16_CLUSTER_29647</td><td>gi|34904202|ref|NP_913448.1| P0492F05.25 [Oryza sativa (japonica cultivar-group)] >gnl|BL_ORD_ID|2386195 gi|13027362|dbj|BAB32723.1| unknown protein [Oryza sativa (japonica cultivar-group)] >gnl|BL_ORD_ID|2386195</td><td>11667.m01238|LOC_Os01g12650|protein Reticulon, putative e_value:9.84587e-131</td><td>0.177</td><td>(0.1002 0.5657)</td><td>0.1002</td><td>0.5657</td></tr>
<tr><td>1176</td><td>SET4_K09_CLUSTER_2457</td><td>gi|51536072|dbj|BAD38198.1| mitochondrial transcription termination factor-like [Oryza sativa (japonica cultivar-group)] >gi|51535002|dbj|BAD37286.1| mitochondrial transcription termination factor-like [Oryza sativa (japonica cultivar-group)] e_value:2</td><td>11680.m01182|LOC_Os06g12100|protein mTERF, putative e_value:7.59064e-111</td><td>0.726</td><td>(0.3151 0.4341)</td><td>0.3151</td><td>0.4341</td></tr>
<tr><td>1177</td><td>SET3_I11_CLUSTER_21104</td><td>gi|50905819|ref|XP_464398.1| unknown protein [Oryza sativa (japonica cultivar-group)] >gi|46390954|dbj|BAD16467.1| unknown protein [Oryza sativa (japonica cultivar-group)] >gi|46389908|dbj|BAD15529.1| unknown protein [Oryza sativa (japonica cultivar</td><td>11668.m00883|LOC_Os02g09600|protein hypothetical protein e_value:1.00806e-46</td><td>0.262</td><td>(0.1482 0.5649)</td><td>0.1482</td><td>0.5649</td></tr>
<tr><td>1178</td><td>SET2_J21_CLUSTER_25336</td><td>gi|34906218|ref|NP_914456.1| unknown protein [Oryza sativa (japonica cultivar-group)] >gi|13122432|dbj|BAB32913.1| unknown protein [Oryza sativa (japonica cultivar-group)] e_value:1.45644e-40</td><td>11667.m00814|LOC_Os01g08650|protein expressed protein e_value:3.77449e-42</td><td>0.569</td><td>(0.7412 1.3027)</td><td>0.7412</td><td>1.3027</td></tr>
<tr><td>1179</td><td>WT013_G18_CLUSTER_2006</td><td>gi|51965060|ref|XP_507314.1| PREDICTED OJ1521_G02.31 gene product [Oryza sativa (japonica cultivar-group)] >gnl|BL_ORD_ID|1610570 gi|50948153|ref|XP_483604.1| putative hydroxyanthranilate hydroxycinnamoyltransferase 2 [Oryza sativa</td><td>11674.m04344|LOC_Os08g43040|protein Transferase family e_value:1.14084e-134</td><td>0.232</td><td>(0.3266 1.4085)</td><td>0.3266</td><td>1.4085</td></tr>
<tr><td>1180</td><td>WT013_C24_CLUSTER_32050</td><td>gi|50911613|ref|XP_467214.1| hypothetical protein [Oryza sativa (japonica cultivar-group)] >gnl|BL_ORD_ID|1580317 gi|41052793|dbj|BAD07661.1| hypothetical protein [Oryza sativa (japonica cultivar-group)] e_value:6.09704e-30</td><td>11668.m04476|LOC_Os02g46290|protein Plant invertase/pectin methylesterase inhibitor e_value:1.39087e-31</td><td>0.519</td><td>(0.4434 0.8546)</td><td>0.4434</td><td>0.8546</td></tr>
<tr><td>1181</td><td>WT011_K14_CLUSTER_20847</td><td>gi|55775180|ref|XP_550516.1| putative syntaxin of plants 41 [Oryza sativa (japonica cultivar-group)] >gnl|BL_ORD_ID|1752736 gi|55296198|dbj|BAD67916.1| putative syntaxin of plants 41 [Oryza sativa (japonica cultivar-group)] e_value:9.65772e-161</td><td>11680.m00167|LOC_Os06g02570|protein SNARE domain, putative e_value:1.99106e-162</td><td>0.11</td><td>(0.0586 0.5308)</td><td>0.0586</td><td>0.5308</td></tr>
<tr><td>1182</td><td>WT010_A24_CLUSTER_21689</td><td>gi|50920351|ref|XP_470536.1| Putative potassium-dependent sodium-calcium exchanger-like protein [Oryza sativa (japonica cultivar-group)] >gnl|BL_ORD_ID|1534988 gi|27436756|gb|AAO13475.1| Putative potassium-dependent sodium-calcium exchanger-like protei</td><td>11669.m00793|LOC_Os03g08230|protein Sodium/calcium exchanger protein, putative e_value:0</td><td>0.159</td><td>(0.0841 0.5281)</td><td>0.0841</td><td>0.5281</td></tr>
<tr><td>1183</td><td>WT009_P02_CLUSTER_17058</td><td>gi|50838910|gb|AAT81671.1| cytochrome P450 [Oryza sativa (japonica cultivar-group)] >gnl|BL_ORD_ID|1025934 gi|27127269|dbj|BAC45000.1| cytochrome P450 [Oryza sativa (japonica cultivar-group)] >gnl|BL_ORD_ID|1025934 gi|62510495|sp|Q8GSQ1|C85A1_ORYSA</td><td>11669.m04014|LOC_Os03g40540|protein cytochrome P450 e_value:0</td><td>0.144</td><td>(0.0850 0.5906)</td><td>0.085</td><td>0.5906</td></tr>
<tr><td>1184</td><td>WT008_H14_CLUSTER_2100</td><td>gi|52076014|dbj|BAD46467.1| bzip-related transcription factor -like [Oryza sativa (japonica cultivar-group)] e_value:0</td><td>11681.m03350|LOC_Os09g37520|protein expressed protein</td><td>0.215</td><td>(0.1378 0.6394)</td><td>0.1378</td><td>0.6394</td></tr>
<tr><td>1185</td><td>WT008_B23_CLUSTER_21528</td><td>gi|40794967|gb|AAR91037.1| RNA-directed RNA polymerase 2 [Hordeum vulgare subsp. vulgare] e_value:0</td><td>11668.m04902|LOC_Os02g50330|protein Similar to RNA-directed RNA polymerase e_value:0</td><td>0.236</td><td>(0.1382 0.5862)</td><td>0.1382</td><td>0.5862</td></tr>
<tr><td>1186</td><td>WT007_N02_CLUSTER_5089</td><td>gi|51090451|dbj|BAD35421.1| putative phosphoribosyl pyrophosphate synthase [Oryza sativa (japonica cultivar-group)] e_value:1.18618e-150</td><td>11680.m04046|LOC_Os06g41360|protein ribose-phosphate diphosphokinase (EC 2.7.6.1) isoform II [validated] - Arabidopsis thaliana (fragment) e_value:0</td><td>0.141</td><td>(0.0772 0.5478)</td><td>0.0772</td><td>0.5478</td></tr>
<tr><td>1187</td><td>WT009_N05_CLUSTER_28352</td><td>gi|50931893|ref|XP_475474.1| hypothetical protein [Oryza sativa (japonica cultivar-group)] >gnl|BL_ORD_ID|1596319 gi|50080319|gb|AAT69653.1| hypothetical protein [Oryza sativa (japonica cultivar-group)] e_value:1.90524e-146</td><td>11682.m03960|LOC_Os05g41390|protein Cyclin, N-terminal domain, putative e_value:5.34e-148</td><td>0.293</td><td>(0.2270 0.7742)</td><td>0.227</td><td>0.7742</td></tr>
<tr><td>1188</td><td>SET2_K13_CLUSTER_31317</td><td>gi|50933811|ref|XP_476433.1| tetratricopeptide repeat-containing protein-like [Oryza sativa (japonica cultivar-group)] >gi|38175649|dbj|BAC83787.2| tetratricopeptide repeat-containing protein-like [Oryza sativa (japonica cultivar-group)] e_value:7.3094</td><td>11673.m00215|LOC_Os07g03070|protein TPR Domain, putative e_value:2.16983e-179</td><td>0.273</td><td>(0.1523 0.5573)</td><td>0.1523</td><td>0.5573</td></tr>
<tr><td>1189</td><td>SET5_B09_CLUSTER_26705</td><td>gi|55297584|dbj|BAD68930.1| hydrolase-like [Oryza sativa (japonica cultivar-group)] e_value:1.85898e-153</td><td>11667.m04320|LOC_Os01g44960|protein hydrolase, alpha/beta fold family, putative e_value:5.32277e-155</td><td>0.31</td><td>(0.1012 0.3263)</td><td>0.1012</td><td>0.3263</td></tr>
<tr><td>1190</td><td>SET5_K06_CLUSTER_2378</td><td>gi|50947237|ref|XP_483146.1| putative cytochrome c oxidase subunit 15(COX15) homolog isoform 1 precursor [Oryza sativa (japonica cultivar-group)] >gi|42408865|dbj|BAD10124.1| putative cytochrome c oxidase subunit 15(COX15) homolog isoform 1 precursor [</td><td>11674.m03864|LOC_Os08g38720|protein Cytochrome oxidase assembly protein, putative e_value:0</td><td>0.157</td><td>(0.0770 0.4909)</td><td>0.077</td><td>0.4909</td></tr>
<tr><td>1191</td><td>SET4_I04_CLUSTER_14051</td><td>gi|21618032|gb|AAM67082.1| unknown [Arabidopsis thaliana] >gi|28973401|gb|AAO64025.1| unknown protein [Arabidopsis thaliana] >gi|26451706|dbj|BAC42948.1| unknown protein [Arabidopsis thaliana] >gi|4220484|gb|AAD12707.1| expressed protein [Arabido</td><td>11686.m01382|LOC_Os12g14110|protein hypothetical protein e_value:2.22542e-56</td><td>0.23</td><td>(0.1559 0.6772)</td><td>0.1559</td><td>0.6772</td></tr>
<tr><td>1192</td><td>SET1_A08_CLUSTER_3901</td><td>gi|50251225|dbj|BAD27669.1| putative actin related protein 2/3 complex, 21 kDa subunit [Oryza sativa (japonica cultivar-group)] e_value:1.84147e-90</td><td>11668.m01301|LOC_Os02g14000|protein P21-ARC (ARP2/3 complex 21 kDa subunit) e_value:2.2129e-92</td><td>0.068</td><td>(0.0422 0.6250)</td><td>0.0422</td><td>0.625</td></tr>
<tr><td>1193</td><td>WT004_H08_CLUSTER_10384</td><td>gi|34894598|ref|NP_908624.1| putative sarcosine oxidase [Oryza sativa (japonica cultivar-group)] >gnl|BL_ORD_ID|1560730 gi|20161421|dbj|BAB90345.1| putative L-pipecolic acid oxidase [Oryza sativa (japonica cultivar-group)] e_value:1.95661e-147</td><td>11681.m02947|LOC_Os09g32290|protein hypothetical protein e_value:2.9707e-169</td><td>0.475</td><td>(0.1674 0.3524)</td><td>0.1674</td><td>0.3524</td></tr>
<tr><td>1194</td><td>SET1_J17_CLUSTER_33484</td><td>gi|50925983|ref|XP_473039.1| OSJNBa0067K08.16 [Oryza sativa (japonica cultivar-group)] >gi|38345387|emb|CAD41249.2| OSJNBa0067K08.16 [Oryza sativa (japonica cultivar-group)] e_value:5.68738e-82</td><td>11670.m04039|LOC_Os04g41990|protein Autophagocytosis associated protein, C-terminal domain, putative</td><td>0.355</td><td>(0.1604 0.4513)</td><td>0.1604</td><td>0.4513</td></tr>
<tr><td>1195</td><td>SET5_K01_CLUSTER_13556</td><td>gi|533692|gb|AAC97524.1| protease inhibitor [Glycine max] >gi|7451427|pir||T06381 proteinase inhibitor - soybean e_value:1.07997e-15</td><td>11669.m00315|LOC_Os03g03810|protein proteinase inhibitor - rice e_value:4.29947e-14</td><td>0.273</td><td>(0.3949 1.4471)</td><td>0.3949</td><td>1.4471</td></tr>
</table>

PDF Complete
Your complimentary use period has ended. Thank you for using PDF Complete.
Click Here to upgrade to Unlimited Pages and Expanded Features

<table>
<tr><th></th><th></th><th></th><th></th><th></th><th></th><th></th><th></th></tr>
<tr><td>1196</td><td>WT007_O10_CLUSTER_23020</td><td>gi|50905113|ref|XP_464045.1| unknown protein [Oryza sativa (japonica cultivar-group)] >gnl|BL_ORD_ID|1566306 gi|42409110|dbj|BAD10360.1| unknown protein [Oryza sativa (japonica cultivar-group)] >gnl|BL_ORD_ID|1566306 gi|42408840|dbj|BAD10100.1| unkn</td><td>11668.m00358|LOC_Os02g04260|protein expressed protein e_value:3.28264e-133</td><td>0.23</td><td>(0.1381 0.6014)</td><td>0.1381</td><td>0.6014</td></tr>
<tr><td>1197</td><td>SET3_O07_CLUSTER_20118</td><td>gi|50947311|ref|XP_483183.1| putative COP9 complex subunit 6 [Oryza sativa (japonica cultivar-group)] >gi|42407349|dbj|BAD08810.1| putative COP9 complex subunit 6 [Oryza sativa (japonica cultivar-group)] e_value:8.94759e-156</td><td>11674.m03907|LOC_Os08g39070|protein Mov34/MPN/PAD-1 family, putative e_value:2.00668e-157</td><td>0.136</td><td>(0.0574 0.4222)</td><td>0.0574</td><td>0.4222</td></tr>
<tr><td>1198</td><td>WT010_F01_CLUSTER_1771</td><td>gi|55296600|dbj|BAD69198.1| unknown protein [Oryza sativa (japonica cultivar-group)] e_value:1.45904e-106</td><td>11680.m00584|LOC_Os06g06410|protein expressed protein e_value:2.18467e-108</td><td>0.059</td><td>(0.0286 0.4859)</td><td>0.0286</td><td>0.4859</td></tr>
<tr><td>1199</td><td>WT005_E10_CLUSTER_9837</td><td>gi|34900328|ref|NP_911510.1| putative eIF3e (subunits of eukaryotic translation initiation factor 3) [Oryza sativa (japonica cultivar-group)] >gnl|BL_ORD_ID|1522906 gi|27261072|dbj|BAC45186.1| putative eIF3e (subunits of eukaryotic translation initiati</td><td>11673.m01162|LOC_Os07g12110|protein PCI domain, putative e_value:0</td><td>0.14</td><td>(0.1100 0.7873)</td><td>0.11</td><td>0.7873</td></tr>
<tr><td>1200</td><td>SET2_M18_CLUSTER_31589</td><td>gi|50725344|dbj|BAD34416.1| putative TGB12K interacting protein 3 [Oryza sativa (japonica cultivar-group)] e_value:2.41374e-142</td><td>11681.m03062|LOC_Os09g33810|protein expressed protein e_value:5.71729e-144</td><td>0.221</td><td>(0.1538 0.6972)</td><td>0.1538</td><td>0.6972</td></tr>
<tr><td>1201</td><td>WT005_F13_CLUSTER_33634</td><td>gi|51091461|dbj|BAD36202.1| hydroxyproline-rich glycoprotein-like [Oryza sativa (japonica cultivar-group)] >gnl|BL_ORD_ID|238984 gi|51091151|dbj|BAD35846.1| hydroxyproline-rich glycoprotein-like [Oryza sativa (japonica cultivar-group)] e_value:0</td><td>11680.m01011|LOC_Os06g10430|protein expressed protein e_value:0</td><td>0.325</td><td>(0.1763 0.5415)</td><td>0.1763</td><td>0.5415</td></tr>
<tr><td>1202</td><td>WT011_H15_CLUSTER_28545</td><td>gi|34895394|ref|NP_909040.1| putative xylosyltransferase I [Oryza sativa (japonica cultivar-group)] >gnl|BL_ORD_ID|1751587 gi|13486801|dbj|BAB40033.1| putative xylosyltransferase I [Oryza sativa (japonica cultivar-group)] e_value:0</td><td>11667.m00228|LOC_Os01g03160|protein Core-2/I-Branching enzyme, putative e_value:0</td><td>0.119</td><td>(0.0686 0.5780)</td><td>0.0686</td><td>0.578</td></tr>
<tr><td>1203</td><td>WT005_F20_CLUSTER_7946</td><td>gi|21636171|gb|AAM69851.1| unknown [Aegilops tauschii] e_value:1.19191e-27</td><td>11669.m01087|LOC_Os03g11370|protein auxin response factor 23, putative e_value:2.08356e-68</td><td>0.708</td><td>(0.3567 0.5035)</td><td>0.3567</td><td>0.5035</td></tr>
<tr><td>1204</td><td>WT006_M18_CLUSTER_19793</td><td>gi|55770507|ref|XP_550244.1| putative Kelch-like protein 5 [Oryza sativa (japonica cultivar-group)] >gnl|BL_ORD_ID|1753639 gi|55296854|dbj|BAD68291.1| putative Kelch-like protein 5 [Oryza sativa (japonica cultivar-group)] e_value:0</td><td>11667.m00649|LOC_Os01g07140|protein Kelch motif, putative e_value:0</td><td>0.509</td><td>(0.2773 0.5449)</td><td>0.2773</td><td>0.5449</td></tr>
<tr><td>1205</td><td>SET2_D11_CLUSTER_34053</td><td>gi|51965060|ref|XP_507314.1| PREDICTED OJ1521_G02.31 gene product [Oryza sativa (japonica cultivar-group)] >gi|50948153|ref|XP_483604.1| putative hydroxyanthranilate hydroxycinnamoyltransferase 2 [Oryza sativa (japonica cultivar-group)] >gi|42407846</td><td>11674.m04344|LOC_Os08g43040|protein Transferase family e_value:1.07255e-127</td><td>0.298</td><td>(0.3641 1.2227)</td><td>0.3641</td><td>1.2227</td></tr>
<tr><td>1206</td><td>WT012_D05_CLUSTER_33559</td><td>gi|50872434|gb|AAT85034.1| putative SPFH domain / Band 7 family [Oryza sativa (japonica cultivar-group)] e_value:4.68798e-178</td><td>11669.m03105|LOC_Os03g30790|protein putative SPFH domain / Band 7 family e_value:1.22251e-179</td><td>0.125</td><td>(0.0723 0.5780)</td><td>0.0723</td><td>0.578</td></tr>
<tr><td>1207</td><td>WT003_L15_CLUSTER_24897</td><td>gi|50940567|ref|XP_479811.1| putative COP9 complex subunit 3, FUS11 [Oryza sativa (japonica cultivar-group)] >gnl|BL_ORD_ID|1601744 gi|42407907|dbj|BAD09047.1| putative COP9 complex subunit 3, FUS11 [Oryza sativa (japonica cultivar-group)] e_value:0</td><td>11674.m00164|LOC_Os08g02550|protein PCI domain, putative e_value:0</td><td>0.186</td><td>(0.0796 0.4287)</td><td>0.0796</td><td>0.4287</td></tr>
<tr><td>1208</td><td>SET5_F21_CLUSTER_22490</td><td>gi|26006491|gb|AAN77300.1| Unknown protein [Oryza sativa (japonica cultivar-group)] e_value:1.18427e-58</td><td>11669.m01041|LOC_Os03g10930|protein Mitochondrial ribosomal protein L51 / S25 / CI-B8 domain, putative e_value:7.11218e-</td><td>0.124</td><td>(0.0511 0.4129)</td><td>0.0511</td><td>0.4129</td></tr>
<tr><td>1209</td><td>SET5_D02_CLUSTER_10817</td><td>gi|20465813|gb|AAM20011.1| unknown protein [Arabidopsis thaliana] >gi|17381208|gb|AAL36416.1| unknown protein [Arabidopsis thaliana] >gi|15238890|ref|NP_197366.1| zinc finger (C3HC4-type RING finger) family protein [Arabidopsis thaliana] e_value:1.5</td><td>11669.m02334|LOC_Os03g22680|protein CHY zinc finger, putative e_value:1.05509e-141</td><td>0.092</td><td>(0.0709 0.7667)</td><td>0.0709</td><td>0.7667</td></tr>
<tr><td>1210</td><td>WT005_I05_CLUSTER_14504</td><td>gi|57899563|dbj|BAD87142.1| putative chorismate mutase precursor [Oryza sativa (japonica cultivar-group)] e_value:5.98513e-129</td><td>11667.m05518|LOC_Os01g55870|protein chorismate mutase, putative e_value:1.46247e-130</td><td>0.22</td><td>(0.1184 0.5378)</td><td>0.1184</td><td>0.5378</td></tr>
<tr><td>1211</td><td>WT006_N01_CLUSTER_32206</td><td>gi|50947997|ref|XP_483526.1| putative small zinc finger-related protein [Oryza sativa (japonica cultivar-group)] >gnl|BL_ORD_ID|1609853 gi|45736081|dbj|BAD13106.1| putative small zinc finger-related protein [Oryza sativa (japonica cultivar-group)] ></td><td>11674.m04269|LOC_Os08g42380|protein mitochondrial import inner membrane translocase subunit tim8 e_value:5.60303e-30</td><td>0.143</td><td>(0.1247 0.8707)</td><td>0.1247</td><td>0.8707</td></tr>
<tr><td>1212</td><td>WT008_K19_CLUSTER_31797</td><td>gi|34913780|ref|NP_918237.1| OSJNBa0026J14.18 [Oryza sativa (japonica cultivar-group)] >gnl|BL_ORD_ID|1541772 gi|20146451|dbj|BAB89231.1| fertility restorer -like protein [Oryza sativa (japonica cultivar-group)] e_value:9.74517e-147</td><td>11667.m03486|LOC_Os01g36600|protein expressed protein e_value:2.81635e-148</td><td>0.173</td><td>(0.1021 0.5894)</td><td>0.1021</td><td>0.5894</td></tr>
<tr><td>1213</td><td>SET5_J15_CLUSTER_10261</td><td>gi|55770759|ref|XP_550421.1| putative choline kinase [Oryza sativa (japonica cultivar-group)] >gi|55295919|dbj|BAD67787.1| putative choline kinase [Oryza sativa (japonica cultivar-group)] e_value:3.85252e-169</td><td>11667.m00829|LOC_Os01g08760|protein Choline/ethanolamine kinase, putative e_value:1.23005e-170</td><td>0.237</td><td>(0.0992 0.4189)</td><td>0.0992</td><td>0.4189</td></tr>
<tr><td>1214</td><td>WT013_I04_CLUSTER_33531</td><td>gi|18855044|gb|AAL79736.1| putative transcription factor [Oryza sativa] e_value:1.51061e-95</td><td>11680.m05013|LOC_Os06g50310|protein hypothetical protein e_value:2.55462e-97</td><td>0.183</td><td>(0.1585 0.8669)</td><td>0.1585</td><td>0.8669</td></tr>
<tr><td>1215</td><td>WT012_N08_CLUSTER_33382</td><td>gi|50947759|ref|XP_483407.1| putative signal recognition particle receptor beta subunit (SR-beta) [Oryza sativa (japonica cultivar-group)] >gnl|BL_ORD_ID|1609732 gi|42407742|dbj|BAD08889.1| putative signal recognition particle receptor beta subunit (SR</td><td>11674.m04148|LOC_Os08g41250|protein expressed protein e_value:5.308e-130</td><td>0.089</td><td>(0.0547 0.6139)</td><td>0.0547</td><td>0.6139</td></tr>
<tr><td>1216</td><td>WT008_D17_CLUSTER_9516</td><td>gi|50909333|ref|XP_466155.1| putative polymyositis/scleroderma autoantigen 1 [Oryza sativa (japonica cultivar-group)] >gnl|BL_ORD_ID|1574617 gi|50725756|dbj|BAD33267.1| putative polymyositis/scleroderma autoantigen 1 [Oryza sativa (japonica cultivar-</td><td>11668.m03274|LOC_Os02g34570|protein 3' exoribonuclease family, domain 2, putative e_value:8.4113e-164</td><td>0.137</td><td>(0.0946 0.6895)</td><td>0.0946</td><td>0.6895</td></tr>
<tr><td>1217</td><td>WT004_K23_CLUSTER_11316</td><td>gi|50906841|ref|XP_464909.1| putative uracil phosphoribosyltransferase [Oryza sativa (japonica cultivar-group)] >gnl|BL_ORD_ID|1567486 gi|50252285|dbj|BAD28290.1| putative uracil phosphoribosyltransferase [Oryza sativa (japonica cultivar-group)] >gn</td><td>11668.m01636|LOC_Os02g17320|protein uridine kinase, putative e_value:0</td><td>0.099</td><td>(0.0503 0.5099)</td><td>0.0503</td><td>0.5099</td></tr>
<tr><td>1218</td><td>SET2_B03_CLUSTER_2444</td><td>gi|50511485|gb|AAT77407.1| unknown protein [Oryza sativa (japonica cultivar-group)] e_value:3.89406e-132</td><td>11682.m04706|LOC_Os05g49060|protein expressed protein e_value:9.97727e-134</td><td>0.128</td><td>(0.0907 0.7089)</td><td>0.0907</td><td>0.7089</td></tr>
<tr><td>1219</td><td>WT007_J06_CLUSTER_32637</td><td>gi|7208213|gb|AAF40112.1| constitutive photomorphogenic 11 [Oryza sativa subsp. indica] e_value:0</td><td>11669.m00179|LOC_Os03g02540|protein PCI domain, putative e_value:0</td><td>0.157</td><td>(0.0762 0.4841)</td><td>0.0762</td><td>0.4841</td></tr>
<tr><td>1220</td><td>WT007_E15_CLUSTER_27030</td><td>gi|50901676|ref|XP_463271.1| putative oxalyl-CoA decarboxylase [Oryza sativa (japonica cultivar-group)] e_value:0</td><td>11667.m03019|LOC_Os01g32080|protein Thiamine pyrophosphate enzyme, central domain, putative e_value:0</td><td>0.095</td><td>(0.0547 0.5752)</td><td>0.0547</td><td>0.5752</td></tr>
<tr><td>1221</td><td>WT005_K17_CLUSTER_31916</td><td>gi|50934433|ref|XP_476744.1| putative iron deficiency protein Ids3 [Oryza sativa (japonica cultivar-group)] >gnl|BL_ORD_ID|1521385 gi|50509732|dbj|BAD31784.1| putative iron deficiency protein Ids3 [Oryza sativa (japonica cultivar-group)] e_value:4.3945</td><td>11673.m00668|LOC_Os07g07410|protein oxidoreductase, 2OG-Fe(II) oxygenase family, putative e_value:1.20439e-146</td><td>0.322</td><td>(0.1407 0.4370)</td><td>0.1407</td><td>0.437</td></tr>
<tr><td>1222</td><td>WT008_E21_CLUSTER_32757</td><td>gi|9759192|dbj|BAB09729.1| vacuolar sorting protein-like; embryogenesis protein H beta 58-like protein [Arabidopsis thaliana] >gnl|BL_ORD_ID|1088915 gi|15238773|ref|NP_200165.1| vacuolar protein sorting-associated protein 26, putative / VPS26, putative</td><td>11687.m03845|LOC_Os11g41130|protein vacuolar sorting protein-like; embryogenesis protein H beta 58-like protein e_value:1.51521e-161</td><td>0.099</td><td>(0.0559 0.5652)</td><td>0.0559</td><td>0.5652</td></tr>
<tr><td>1223</td><td>SET1_D04_CLUSTER_30876</td><td>gi|51535940|dbj|BAD38022.1| unknown protein [Oryza sativa (japonica cultivar-group)] e_value:4.15936e-73</td><td>11681.m02583|LOC_Os09g27960|protein Uncharacterised protein family (UPF0220) e_value:4.36487e-75</td><td>0.111</td><td>(0.0468 0.4230)</td><td>0.0468</td><td>0.423</td></tr>
<tr><td>1224</td><td>WT006_K23_CLUSTER_11636</td><td>gi|19849248|gb|AAL99536.1| cinnamyl alcohol dehydrogenase [Lolium perenne] e_value:0</td><td>11676.m02527|LOC_Os10g29470|protein oxidoreductase, zinc-binding dehydrogenase family e_value:1.19325e-174</td><td>0.304</td><td>(0.1311 0.4312)</td><td>0.1311</td><td>0.4312</td></tr>
<tr><td>1225</td><td>SET1_P23_CLUSTER_6440</td><td>gi|24796800|gb|AAN64476.1| hypothetical protein [Oryza sativa (japonica cultivar-group)] >gi|50948969|ref|XP_493842.1| hypothetical protein [Oryza sativa (japonica cultivar-group)] e_value:5.58981e-74</td><td>11669.m01762|LOC_Os03g17450|protein expressed protein e_value:1.05204e-75</td><td>0.388</td><td>(0.2262 0.5834)</td><td>0.2262</td><td>0.5834</td></tr>
<tr><td>1226</td><td>SET2_G06_CLUSTER_2754</td><td>gi|50904871|ref|XP_463924.1| putative eukaryotic translation initiation factor 3, subunit 1 alpha, 35kDa [Oryza sativa (japonica cultivar-group)] >gi|51963824|ref|XP_506695.1| PREDICTED P0575F10.1 gene product [Oryza sativa (japonica cultivar-group)] &</td><td>11668.m00220|LOC_Os02g02990|protein expressed protein e_value:4.27555e-101</td><td>0.185</td><td>(0.1071 0.5795)</td><td>0.1071</td><td>0.5795</td></tr>
<tr><td>1227</td><td>WT002_G16_CLUSTER_32202</td><td>gi|32968028|gb|AAP92528.1| HDA1 [Zea mays] e_value:0</td><td>11673.m04017|LOC_Os07g41090|protein HDA1 e_value:0</td><td>0.239</td><td>(0.1096 0.4591)</td><td>0.1096</td><td>0.4591</td></tr>
<tr><td>1228</td><td>WT006_K04_CLUSTER_3832</td><td>gi|51535947|dbj|BAD38029.1| putative cysteine conjugate beta-lyase [Oryza sativa (japonica cultivar-group)] e_value:0</td><td>11681.m02595|LOC_Os09g28050|protein aminotransferase, putative e_value:0</td><td>0.109</td><td>(0.1016 0.9359)</td><td>0.1016</td><td>0.9359</td></tr>
<tr><td>1229</td><td>SET4_H04_CLUSTER_34229</td><td>gi|50912493|ref|XP_467654.1| MutT/nudix-like [Oryza sativa (japonica cultivar-group)] >gi|51964346|ref|XP_506958.1| PREDICTED P0643A10.49 gene product [Oryza sativa (japonica cultivar-group)] >gi|46390677|dbj|BAD16159.1| MutT/nudix-like [Oryza sativ</td><td>11668.m04881|LOC_Os02g50130|protein hydrolase, NUDIX family e_value:8.67251e-72</td><td>0.435</td><td>(0.0992 0.2279)</td><td>0.0992</td><td>0.2279</td></tr>
<tr><td>1230</td><td>WT010_G09_CLUSTER_14399</td><td>gi|50939767|ref|XP_479411.1| putative developmental protein sina [Oryza sativa (japonica cultivar-group)] >gnl|BL_ORD_ID|1533408 gi|50510057|dbj|BAD30685.1| putative developmental protein sina [Oryza sativa (japonica cultivar-group)] >gnl|BL_ORD_ID|</td><td>11669.m02431|LOC_Os03g24040|protein Seven in absentia protein family, putative e_value:2.80796e-168</td><td>0.049</td><td>(0.0585 1.1963)</td><td>0.0585</td><td>1.1963</td></tr>
<tr><td>1231</td><td>WT007_H18_CLUSTER_8375</td><td>gi|57900106|dbj|BAD88168.1| putative serine palmitoyltransferase [Oryza sativa (japonica cultivar-group)] e_value:0</td><td>11667.m07052|LOC_Os01g70380|protein aminotransferase, classes I and II, putative e_value:0</td><td>0.064</td><td>(0.0322 0.4999)</td><td>0.0322</td><td>0.4999</td></tr>
<tr><td>1232</td><td>WT013_H13_CLUSTER_33835</td><td>gi|9294695|dbj|BAB03095.1| unnamed protein product [Arabidopsis thaliana] >gnl|BL_ORD_ID|1098028 gi|15294240|gb|AAK95297.1| AT3g18860/MCB22_3 [Arabidopsis thaliana] >gnl|BL_ORD_ID|1098028 gi|18402031|ref|NP_566620.1| transducin family protein / WD-4</td><td>11673.m00224|LOC_Os07g03160|protein expressed protein e_value:0</td><td>0.115</td><td>(0.0697 0.6058)</td><td>0.0697</td><td>0.6058</td></tr>
<tr><td>1233</td><td>WT003_H09_CLUSTER_33167</td><td>gi|50929105|ref|XP_474080.1| OSJNBa0063C18.17 [Oryza sativa (japonica cultivar-group)] >gnl|BL_ORD_ID|1594480 gi|38347326|emb|CAE05976.2| OSJNBa0063C18.17 [Oryza sativa (japonica cultivar-group)] >gnl|BL_ORD_ID|1594480 gi|38344879|emb|CAD41902.2| OS</td><td>11670.m05413|LOC_Os04g55040|protein V-type ATPase, D subunit, putative e_value:6.55531e-134</td><td>0.121</td><td>(0.0446 0.3678)</td><td>0.0446</td><td>0.3678</td></tr>
<tr><td>1234</td><td>WT002_L21_CLUSTER_34254</td><td>gi|52353421|gb|AAU43989.1| unknown protein [Oryza sativa (japonica cultivar-group)] e_value:9.53775e-170</td><td>11682.m04476|LOC_Os05g46490|protein hypothetical protein e_value:8.10365e-109</td><td>0.376</td><td>(0.2085 0.5549)</td><td>0.2085</td><td>0.5549</td></tr>
<tr><td>1235</td><td>WT013_N01_CLUSTER_4304</td><td>gi|8809587|dbj|BAA97138.1| unnamed protein product [Arabidopsis thaliana] >gnl|BL_ORD_ID|1095510 gi|15237323|ref|NP_200110.1| expressed protein [Arabidopsis thaliana] >gnl|BL_ORD_ID|1095510 gi|48310362|gb|AAT41806.1| At5g52980 [Arabidopsis thaliana]</td><td>11668.m01216|LOC_Os02g13200|protein At5g52980 e_value:1.79593e-81</td><td>0.301</td><td>(0.1209 0.4015)</td><td>0.1209</td><td>0.4015</td></tr>
<tr><td>1236</td><td>SET5_K11_CLUSTER_28232</td><td>gi|31433611|gb|AAP55103.1| putative nuclear protein [Oryza sativa (japonica cultivar-group)] >gi|37537028|ref|NP_922816.1| putative nuclear protein [Oryza sativa (japonica cultivar-group)] >gi|19225002|gb|AAL86478.1| putative nuclear protein [Oryza</td><td>11676.m03886|LOC_Os10g42090|protein putative nuclear protein e_value:3.06139e-78</td><td>0.185</td><td>(0.0678 0.3666)</td><td>0.0678</td><td>0.3666</td></tr>
<tr><td>1237</td><td>WT004_I18_CLUSTER_14092</td><td>gi|1109672|gb|AAC49121.1| 3'(2'),5-diphosphonucleoside 3'(2') phosphohydrolase >gnl|BL_ORD_ID|403735 gi|7489522|pir||T03305 probable 3'(2'),5'-bisphosphate nucleotidase (EC 3.1.3.7) - rice >gnl|BL_ORD_ID|403735 gi|</td><td>11686.m00786|LOC_Os12g08280|protein 3'(2'),5'-bisphosphate nucleotidase</td><td>0.17</td><td>(0.1093 0.6442)</td><td>0.1093</td><td>0.6442</td></tr>
<tr><td>1238</td><td>SET4_G17_CLUSTER_317</td><td>gi|50929673|ref|XP_474364.1| OSJNBb0017I01.5 [Oryza sativa (japonica cultivar-group)] >gi|32487391|emb|CAE05725.1| OSJNBb0017I01.5 [Oryza sativa (japonica cultivar-group)] e_value:3.75804e-127</td><td>11670.m05765|LOC_Os04g58240|protein expressed protein e_value:1.02117e-128</td><td>0.278</td><td>(0.1252 0.4504)</td><td>0.1252</td><td>0.4504</td></tr>
</table>

PDF Complete
Your complimentary use period has ended. Thank you for using PDF Complete.
Click Here to upgrade to Unlimited Pages and Expanded Features

1239	WT004_G21_CLUSTER_14044	gi|34895518|ref|NP_909102.1| unknown protein [Oryza sativa (japonica cultivar-group)] >gnl|BL_ORD_ID|1751976 gi|9558458|dbj|BAB03379.1| unknown protein [Oryza sativa (japonica cultivar-group)] e_value:0	11667.m00290|LOC_Os01g03760|protein expressed protein e_value:0	0.31	(0.1901 0.6123)	0.1901	0.6123
1240	WT003_N03_CLUSTER_31421	gi|56785168|dbj|BAD81844.1| putative ring finger protein 5 [Oryza sativa (japonica cultivar-group)] >gnl|BL_ORD_ID|776240 gi|56202103|dbj|BAD73632.1| putative ring finger protein 5 [Oryza sativa (japonica cultivar-group)] e_value:0	11667.m06120|LOC_Os01g61420|protein Zinc finger, C3HC4 type (RING finger), putative e_value:0	0.193	(0.1212 0.6270)	0.1212	0.627
1241	SET3_F15_CLUSTER_26746	gi|56201842|dbj|BAD73292.1| putative ATP-dependent Clp protease, proteolytic subunit [Oryza sativa (japonica cultivar-group)] e_value:1.25647e-142	11667.m03048|LOC_Os01g32350|protein ATP-dependent Clp protease, proteolytic subunit ClpP, putative e_value:2.91413e-	0.179	(0.0899 0.5034)	0.0899	0.5034
1242	WT004_L03_CLUSTER_32336	gi|21928093|gb|AAM78075.1| AT3g61860/F21F14_30 [Arabidopsis thaliana] >gnl|BL_ORD_ID|1084397 gi|16612268|gb|AAL27502.1| AT3g61860/F21F14_30 [Arabidopsis thaliana] >gnl|BL_ORD_ID|1084397 gi|26454661|sp|P92964|RSP31_ARATH Arginine/serine-rich	11668.m00227|LOC_Os02g03040|protein RNA recognition motif. (a.k.a. RRM, RBD, or RNP domain), putative e_value:2.01071e-66		(0.4677 -1.0000)	0.4677	-1
1243	SET3_H20_CLUSTER_19639	gi|34902138|ref|NP_912415.1| hypothetical protein [Oryza sativa (japonica cultivar-group)] >gi|29893604|gb|AAP06858.1| hypothetical protein [Oryza sativa (japonica cultivar-group)] e_value:1.91957e-168	11669.m01566|LOC_Os03g15700|protein At2g32600/T26B15.16 e_value:9.81165e-175	0.123	(0.0651 0.5306)	0.0651	0.5306
1244	WT006_G08_CLUSTER_31599	gi|50902382|ref|XP_463624.1| putative stearoyl-acyl-carrier protein desaturase [Oryza sativa (japonica cultivar-group)] >gnl|BL_ORD_ID|1560089 gi|19386730|dbj|BAB86112.1| putative stearoyl-Acyl-carrier protein desaturase [Oryza sativa (japonica cultiva	11667.m06965|LOC_Os01g69080|protein Fatty acid desaturase e_value:0	0.103	(0.0567 0.5508)	0.0567	0.5508
1245	WT010_K05_CLUSTER_2452	gi|62734051|gb|AAX96160.1| Zinc finger, C3HC4 type (RING finger), putative [Oryza sativa (japonica cultivar-group)] e_value:1.22789e-86	11676.m00434|LOC_Os10g05230|protein Zinc finger, C3HC4 type (RING finger), putative e_value:6.44454e-65	0.165	(0.1356 0.8219)	0.1356	0.8219
1246	WT004_A23_CLUSTER_30024	gi|34901636|ref|NP_912164.1| putative thioredoxin-like protein CDSP32 [Oryza sativa (japonica cultivar-group)] >gnl|BL_ORD_ID|1526770 gi|29837201|dbj|BAC75581.1| putative thioredoxin-like protein CDSP32 [Oryza sativa (japonica cultivar-group)] e_value:	11673.m02786|LOC_Os07g29410|protein Thioredoxin, putative e_value:1.3725e-122	0.662	(0.1811 0.2735)	0.1811	0.2735
1247	WT013_I23_CLUSTER_31230	gi|34895468|ref|NP_909077.1| P0037C04.30 [Oryza sativa (japonica cultivar-group)] >gnl|BL_ORD_ID|1510635 gi|12328529|dbj|BAB21187.1| contains ESTs D22419(C10991),AU100646(C10991)~similar to Arabidopsis thaliana chromosome 2,	11667.m00264|LOC_Os01g03520|protein Ubiquitin-conjugating enzyme, putative e_value:0	0.214	(0.1152 0.5392)	0.1152	0.5392
1248	WT002_P23_CLUSTER_25787	gi|50904529|ref|XP_463753.1| B1139B11.7 [Oryza sativa (japonica cultivar-group)] >gnl|BL_ORD_ID|1540898 gi|20161866|dbj|BAB90779.1| putative phosphatidylglycerol specific phospholipase C [Oryza sativa (japonica cultivar-group)] e_value:0	11667.m07276|LOC_Os01g72520|protein Phosphoesterase family, putative e_value:0	0.185	(0.0957 0.5174)	0.0957	0.5174
1249	WT006_B15_CLUSTER_33784	gi|34909286|ref|NP_915990.1| putative receptor-like protein kinase [Oryza sativa (japonica cultivar-group)] >gnl|BL_ORD_ID|1554713 gi|21104781|dbj|BAB93368.1| putative receptor-like protein kinase [Oryza sativa (japonica cultivar-group)] >gnl|BL_ORD	11667.m06004|LOC_Os01g60330|protein Protein kinase domain, putative e_value:0	0.115	(0.0703 0.6099)	0.0703	0.6099
1250	WT006_B10_CLUSTER_7754	gi|50946099|ref|XP_482577.1| zinc finger protein-like [Oryza sativa (japonica cultivar-group)] >gnl|BL_ORD_ID|1607588 gi|42408883|dbj|BAD10141.1| zinc finger protein-like [Oryza sativa (japonica cultivar-group)] e_value:1.21854e-90	11687.m03596|LOC_Os11g38800|protein Zinc finger, C3HC4 type (RING finger), putative e_value:2.68367e-127	0.127	(0.0661 0.5223)	0.0661	0.5223
1251	WT002_J08_CLUSTER_31592	gi|34908198|ref|NP_915446.1| putative AAA-metalloprotease [Oryza sativa (japonica cultivar-group)] >gnl|BL_ORD_ID|1556451 gi|19571026|dbj|BAB86453.1| putative AAA-metalloprotease FtsH [Oryza sativa (japonica cultivar-group)] e_value:0	11682.m03628|LOC_Os05g38400|protein ATP-dependent metalloprotease FtsH, putative e_value:0	0.15	(0.0764 0.5080)	0.0764	0.508
1252	WT004__C15_CLUSTER_2074	gi|56412213|gb|AAV88601.1| low temperature and salt responsive protein [Pennisetum glaucum] e_value:1.15302e-23	11673.m04342|LOC_Os07g44180|protein Uncharacterized protein family e_value:3.30637e-25	0.456	(0.0386 0.0847)	0.0386	0.0847
1253	SET1_O12_CLUSTER_10635	gi|21592963|gb|AAM64912.1| unknown [Arabidopsis thaliana] e_value:2.28609e-89	11686.m00499|LOC_Os12g05650|protein ACT domain, putative e_value:1.91875e-122	0.183	(0.0764 0.4184)	0.0764	0.4184
1254	SET3_D21_CLUSTER_31488	gi|50901788|ref|XP_463327.1| putative carbamoyl phosphate synthetase [Oryza sativa (japonica cultivar-group)] e_value:0	11667.m03730|LOC_Os01g38970|protein carbamoyl-phosphate synthase, large subunit e_value:0	0.088	(0.0479 0.5447)	0.0479	0.5447
1255	SET1_H22_CLUSTER_33636	gi|50932689|ref|XP_475872.1| putative anti-silencing protein [Oryza sativa (japonica cultivar-group)] >gi|49328026|gb|AAT58727.1| putative anti-silencing protein [Oryza sativa (japonica cultivar-group)] e_value:1.35159e-86	11682.m04586|LOC_Os05g48030|protein Similar to Anti-silencing protein, ASF1 e_value:3.31286e-88	0.135	(0.0833 0.6155)	0.0833	0.6155
1256	WT006_G03_CLUSTER_34770	gi|6478922|gb|AAF14027.1| putative oxysterol-binding protein [Arabidopsis thaliana] >gnl|BL_ORD_ID|1558960 gi|20260050|gb|AAM13372.1| putative oxysterol-binding protein [Arabidopsis thaliana] >gnl|BL_ORD_ID|1558960	11669.m01676|LOC_Os03g16690|protein oxysterol-binding protein e_value:0	0.119	(0.0698 0.5867)	0.0698	0.5867
1257	WT005_D21_CLUSTER_8277	gi|52077043|dbj|BAD46075.1| unknown protein [Oryza sativa (japonica cultivar-group)] >gnl|BL_ORD_ID|251961 gi|52076949|dbj|BAD45960.1| unknown protein [Oryza sativa (japonica cultivar-group)] e_value:1.4599e-44	11681.m03475|LOC_Os09g38740|protein expressed protein e_value:1.86984e-46	0.082	(0.0265 0.3238)	0.0265	0.3238
1258	WT002_B21_CLUSTER_33410	gi|100561|pir||S17928 acyl carrier protein 3 precursor, chloroplast - barley >gnl|BL_ORD_ID|2317630 gi|113172|sp|P15543|ACP3_HORVU Acyl carrier protein III, chloroplast precursor (ACP III) >gnl|BL_ORD_ID|2317630 gi|166971|gb|AAA32922.1| acyl carrier	11674.m04399|LOC_Os08g43580|protein acyl carrier protein, putative e_value:3.75908e-56	0.171	(0.0818 0.4775)	0.0818	0.4775
1259	SET6_M06_CLUSTER_33410	gi|100561|pir||S17928 acyl carrier protein 3 precursor, chloroplast - barley >gi|113172|sp|P15543|ACP3_HORVU Acyl carrier protein III, chloroplast precursor (ACP III) >gi|166971|gb|AAA32922.1| acyl carrier protein III e_value:5.16994e-66	11674.m04399|LOC_Os08g43580|protein acyl carrier protein, putative e_value:3.75908e-56	0.171	(0.0818 0.4775)	0.0818	0.4775
1260	WT005_J07_CLUSTER_8296	gi|50909039|ref|XP_466008.1| unknown protein [Oryza sativa (japonica cultivar-group)] >gnl|BL_ORD_ID|1577409 gi|49388919|dbj|BAD26141.1| unknown protein [Oryza sativa (japonica cultivar-group)] e_value:1.0756e-48	11668.m03027|LOC_Os02g32200|protein expressed protein e_value:2.44695e-50	0.567	(0.3265 0.5755)	0.3265	0.5755
1261	WT002_O13_CLUSTER_34839	gi|50940457|ref|XP_479756.1| putative fructokinase [Oryza sativa (japonica cultivar-group)] >gnl|BL_ORD_ID|1601257 gi|51964626|ref|XP_507097.1| PREDICTED P0498H04.29 gene product [Oryza sativa (japonica cultivar-group)] >gnl|BL_ORD_ID|1601257 gi|424	11674.m00119|LOC_Os08g02120|protein kinase, pfkB family e_value:4.29992e-176	0.145	(0.0620 0.4266)	0.062	0.4266
1262	WT002_D03_CLUSTER_33144	gi|34903262|ref|NP_912978.1| unnamed protein product [Oryza sativa (japonica cultivar-group)] >gnl|BL_ORD_ID|1752470 gi|6539562|dbj|BAA88179.1| putative isocitrate dehydrogenase [Oryza sativa (japonica cultivar-group)] e_value:0	11667.m01698|LOC_Os01g16900|protein isocitrate dehydrogenase, NAD-dependent e_value:0	0.045	(0.0272 0.6024)	0.0272	0.6024
1263	SET4_P22_CLUSTER_28958	gi|53792521|dbj|BAD53485.1| unknown protein [Oryza sativa (japonica cultivar-group)] e_value:5.36745e-105	11680.m01907|LOC_Os06g19620|protein expressed protein e_value:2.93741e-102	0.2	(0.0788 0.3933)	0.0788	0.3933
1264	SET4_D09_CLUSTER_34694	gi|56784353|dbj|BAD82374.1| putative signal recognition particle 72 [Oryza sativa (japonica cultivar-group)] e_value:0	11667.m06687|LOC_Os01g66560|protein expressed protein	0.15	(0.0890 0.5929)	0.089	0.5929
1265	WT011_O06_CLUSTER_34220	gi|34902116|ref|NP_912404.1| unknown protein [Oryza sativa (japonica cultivar-group)] >gnl|BL_ORD_ID|1588755 gi|29893593|gb|AAP06847.1| unknown protein [Oryza sativa (japonica cultivar-group)] e_value:0	11669.m01551|LOC_Os03g15570|protein Protein kinase domain, putative e_value:0	0.258	(0.1530 0.5933)	0.153	0.5933
1266	WT010__C03_CLUSTER_10414	gi|33324486|gb|AAQ07984.1| COP8-like protein [Lilium longiflorum] e_value:0	11669.m00955|LOC_Os03g10120|protein COP8 (constitutive photomorphogenic) homolog e_value:0	0.076	(0.0316 0.4163)	0.0316	0.4163
1267	SET5__C17_CLUSTER_28682	gi|50900042|ref|XP_450809.1| unknown protein [Oryza sativa (japonica cultivar-group)] >gi|49387644|dbj|BAD25838.1| unknown protein [Oryza sativa (japonica cultivar-group)] e_value:6.00672e-27	11681.m01947|LOC_Os09g21470|protein expressed protein e_value:2.68533e-29	0.473	(0.1353 0.2858)	0.1353	0.2858
1268	SET1_F23_CLUSTER_26034	No hits found	11682.m00933|LOC_Os05g09580|protein expressed protein e_value:3.43219e-19	1.146	(0.2264 0.1976)	0.2264	0.1976
1269	WT005_D18_CLUSTER_29061	gi|50932097|ref|XP_475576.1| aspartic proteinase [Oryza sativa (japonica cultivar-group)] >gnl|BL_ORD_ID|1597066 gi|46485798|gb|AAS98423.1| aspartic proteinase [Oryza sativa (japonica cultivar-group)] e_value:0	11682.m00404|LOC_Os05g04630|protein aspartic proteinase precursor (ec 3.4.23.-) e_value:0	0.103	(0.0775 0.7546)	0.0775	0.7546
1270	WT003_J10_CLUSTER_34311	gi|51979316|ref|XP_507513.1| PREDICTED OJ1626_B09.4 gene product [Oryza sativa (japonica cultivar-group)] >gnl|BL_ORD_ID|1582721 gi|50910978|ref|XP_466922.1| alpha 2 subunit of 20S proteasome [Oryza sativa (japonica cultivar-group)] >gnl|BL_ORD_ID|1	11668.m04045|LOC_Os02g42320|protein proteasome subunit alpha type 2 (ec 3.4.25.1) (20s proteasome alphasubunit b) (20s proteasome subunit alpha-2) e_value:4.33091e-128	0.014	(0.0056 0.4174)	0.0056	0.4174
1271	WT003_D09_CLUSTER_34124	gi|47848282|dbj|BAD22146.1| putative 26S proteasome non-ATPase regulatory subunit 6 (26S proteasome regulatory particle non-ATPase subunit 7) (OsRPN7) [Oryza sativa (japonica cultivar-group)] e_value:0	11668.m03716|LOC_Os02g38810|protein 26s proteasome non-atpase regulatory subunit 6 (26s proteasomeregulatory particle non-atpase subunit 7) (osrpn7). e_value:0	0.099	(0.0411 0.4146)	0.0411	0.4146
1272	SET1_B14_CLUSTER_34352	gi|18092339|gb|AAL59231.1| ribosomal protein L35A [Zea mays] e_value:8.74869e-53	11682.m04610|LOC_Os05g48220|protein Ribosomal protein L35Ae e_value:9.82105e-50	0.441	(0.1217 0.2761)	0.1217	0.2761
1273	SET5_J09_CLUSTER_28030	gi|34897116|ref|NP_909904.1| putative clathrin assembly protein [Oryza sativa] >gi|17149112|gb|AAL35902.1| clathrin assembly protein AP19-like protein [Oryza sativa] >gi|14718316|gb|AAK72894.1| putative clathrin assembly protein [Oryza sativa] e_val	11669.m05761|LOC_Os03g57040|protein putative clathrin assembly protein e_value:2.00116e-86	0.036	(0.0162 0.4427)	0.0162	0.4427
1274	SET5_I03_CLUSTER_15653	gi|50942933|ref|XP_480994.1| putative mec-8 [Oryza sativa (japonica cultivar-group)] >gi|40253912|dbj|BAD05845.1| putative mec-8 [Oryza sativa (japonica cultivar-group)] >gi|40253748|dbj|BAD05688.1| putative mec-8 [Oryza sativa (japonica cultivar-gr	11674.m01471|LOC_Os08g15080|protein RNA recognition motif. (a.k.a. RRM, RBD, or RNP domain), putative e_value:3.74559e-	0.159	(0.0777 0.4873)	0.0777	0.4873
1275	SET3_D09_CLUSTER_34548	gi|56783956|dbj|BAD81393.1| putative floral homeotic protein HUA1 [Oryza sativa (japonica cultivar-group)] >gi|62901483|sp|Q5NAV3|ZFNL3_ORYSA Zinc finger CCCH type domain containing protein ZFN-like 3 e_value:0	11667.m01532|LOC_Os01g15350|protein Zinc finger C-x8-C-x5-C-x3-H type (and similar), putative e_value:0	0.321	(0.1495 0.4658)	0.1495	0.4658
1276	WT002__C24_CLUSTER_33968	gi|50919439|ref|XP_470116.1| putative cinnamoyl-CoA reductase [Oryza sativa (japonica cultivar-group)] >gnl|BL_ORD_ID|1588193 gi|29124112|gb|AAO65853.1| putative cinnamoyl-CoA reductase [Oryza sativa (japonica cultivar-group)] >gnl|BL_ORD_ID|1588193	11669.m06139|LOC_Os03g60380|protein putative cinnamoyl-CoA reductase e_value:1.14078e-157	0.304	(0.0963 0.3162)	0.0963	0.3162
1277	SET6__C09_CLUSTER_11800	gi|50912995|ref|XP_467905.1| unknown protein [Oryza sativa (japonica cultivar-group)] >gi|47497361|dbj|BAD19400.1| unknown protein [Oryza sativa (japonica cultivar-group)] e_value:2.50322e-53	11668.m05112|LOC_Os02g52260|protein expressed protein e_value:5.73848e-55	0.605	(0.2538 0.4193)	0.2538	0.4193
1278	SET3_P17_CLUSTER_985	gi|51534988|dbj|BAD38112.1| magnesium transporter CorA-like [Oryza sativa (japonica cultivar-group)] e_value:0	11680.m04339|LOC_Os06g44150|protein CorA-like Mg2+ transporter protein e_value:0	0.115	(0.0549 0.4776)	0.0549	0.4776
1279	WT009_D20_CLUSTER_22184	gi|23505957|gb|AAN28838.1| At5g42240/K5J14_4 [Arabidopsis thaliana] >gnl|BL_ORD_ID|1268172 gi|10177009|dbj|BAB10197.1| serine carboxypeptidase II-like [Arabidopsis thaliana] >gnl|BL_ORD_ID|1268172 gi|13605557|gb|AAK32772.1| AT5g42240/K5J14_4 [Arabid	11687.m01023|LOC_Os11g10750|protein At5g42240/K5J14_4 e_value:0	0.117	(0.0652 0.5567)	0.0652	0.5567
1280	SET5_A17_CLUSTER_30682	gi|50915874|ref|XP_468401.1| unknown protein [Oryza sativa (japonica cultivar-group)] >gi|47848188|dbj|BAD22015.1| unknown protein [Oryza sativa (japonica cultivar-group)] >gi|47847736|dbj|BAD21514.1| unknown protein [Oryza sativa (japonica cultivar	11668.m05650|LOC_Os02g57170|protein expressed protein e_value:5.05916e-58	0.227	(0.1398 0.6160)	0.1398	0.616
1281	WT002_G19_CLUSTER_7324	gi|50948087|ref|XP_483571.1| putative ariadne [Oryza sativa (japonica cultivar-group)] >gnl|BL_ORD_ID|1610536 gi|38636670|dbj|BAD03091.1| putative ariadne [Oryza sativa (japonica cultivar-group)] >gnl|BL_ORD_ID|1610536 gi|50725684|dbj|BAD33150.1| pu	11674.m04310|LOC_Os08g42740|protein Transposable element protein, putative e_value:0	0.152	(0.0927 0.6108)	0.0927	0.6108

PDF Complete
Your complimentary use period has ended. Thank you for using PDF Complete.
Click Here to upgrade to Unlimited Pages and Expanded Features

1282	SET6_I15_CLUSTER_34529	gi|50947995|ref|XP_483525.1| putative DHHC-type zinc finger domain-containing protein [Oryza sativa (japonica cultivar-group)] >gi|45736080|dbj|BAD13105.1| putative DHHC-type zinc finger domain-containing protein [Oryza sativa (japonica cultivar-group)	11674.m04268|LOC_Os08g42370|protein DHHC zinc finger domain, putative e_value:0	0.2	(0.0949 0.4754)	0.0949	0.4754
1283	WT011_H12_CLUSTER_1692	gi|29824217|gb|AAP04069.1| unknown protein [Arabidopsis thaliana] >gnl|BL_ORD_ID|1213755 gi|9280220|dbj|BAB01710.1| unnamed protein product [Arabidopsis thaliana] >gnl|BL_ORD_ID|1213755 gi|28973699|gb|AAO64166.1| unknown protein [Arabidopsis thalian	11681.m02952|LOC_Os09g32320|protein Plant protein family, putative e_value:7.92865e-178	0.286	(0.1406 0.4911)	0.1406	0.4911
1284	SET6_M10_CLUSTER_28366	gi|50907537|ref|XP_465257.1| putative nonclathrin coat protein zeta2-COP [Oryza sativa (japonica cultivar-group)] >gi|50251725|dbj|BAD27645.1| putative nonclathrin coat protein zeta2-COP [Oryza sativa (japonica cultivar-group)] >gi|46389933|dbj|BAD1	11668.m02046|LOC_Os02g21250|protein nonclathrin coat protein zeta2-COP e_value:6.34208e-93	0.053	(0.0296 0.5578)	0.0296	0.5578
1285	WT011_F19_CLUSTER_25502	gi|50934021|ref|XP_476538.1| putative amino acid permease [Oryza sativa (japonica cultivar-group)] >gnl|BL_ORD_ID|1520651 gi|50509305|dbj|BAD30612.1| putative amino acid permease [Oryza sativa (japonica cultivar-group)] >gnl|BL_ORD_ID|1520651 gi|343	11673.m00328|LOC_Os07g04180|protein Transmembrane amino acid transporter protein e_value:0	0.108	(0.0645 0.5982)	0.0645	0.5982
1286	SET1_G09_CLUSTER_11547	gi|50909577|ref|XP_466277.1| unknown protein [Oryza sativa (japonica cultivar-group)] >gi|46390350|dbj|BAD15815.1| unknown protein [Oryza sativa (japonica cultivar-group)] >gi|46390154|dbj|BAD15588.1| unknown protein [Oryza sativa (japonica cultivar	11668.m03383|LOC_Os02g35610|protein expressed protein e_value:5.14226e-31	0.103	(0.0278 0.2696)	0.0278	0.2696
1287	WT008_A18_CLUSTER_30612	gi|57900420|dbj|BAD87656.1| RWD domain containing 1-like protein [Oryza sativa (japonica cultivar-group)] >gnl|BL_ORD_ID|972864 gi|57899330|dbj|BAD87941.1| RWD domain containing 1-like protein [Oryza sativa (japonica cultivar-group)] e_value:8.62748e-1	11667.m03607|LOC_Os01g37770|protein RWD domain, putative e_value:2.02353e-115	0.233	(0.1032 0.4432)	0.1032	0.4432
1288	WT003_M11_CLUSTER_9630	gi|26986190|emb|CAD58960.1| apoplastic invertase 1 [Hordeum vulgare subsp. vulgare] e_value:0	11670.m03245|LOC_Os04g33740|protein Glycosyl hydrolases family 32 e_value:0	0.267	(0.1302 0.4882)	0.1302	0.4882
1289	WT008_K12_CLUSTER_29165	gi|38346759|emb|CAE03864.2| OSJNBa0081C01.10 [Oryza sativa (japonica cultivar-group)] >gnl|BL_ORD_ID|1535265 gi|38346628|emb|CAD41211.2| OSJNBa0074L08.22 [Oryza sativa (japonica cultivar-group)] >gnl|BL_ORD_ID|1535265 gi|50926654|ref|XP_473274.1| OS	11670.m04347|LOC_Os04g44830|protein thioredoxin, putative e_value:4.89604e-72	0.276	(0.1180 0.4276)	0.118	0.4276
1290	WT004_I12_CLUSTER_22199	gi|38567912|emb|CAD41576.3| OSJNBa0088I22.8 [Oryza sativa (japonica cultivar-group)] >gnl|BL_ORD_ID|1524386 gi|50928063|ref|XP_473559.1| OSJNBa0088I22.8 [Oryza sativa (japonica cultivar-group)] e_value:6.84639e-110	11670.m04731|LOC_Os04g48260|protein Zinc finger, C3HC4 type (RING finger), putative e_value:1.57414e-110	0.274	(0.1300 0.4752)	0.13	0.4752
1291	WT006_O17_CLUSTER_18239	gi|34915464|ref|NP_919189.1| glutamate receptor, ionotropic kainate 5 precursor-like protein [Oryza sativa (japonica cultivar-group)] >gnl|BL_ORD_ID|1517668 gi|22324479|dbj|BAC10393.1| glutamate receptor, ionotropic kainate 5 precursor-like protein [Or	11673.m00034|LOC_Os07g01310|protein Receptor family ligand binding region, putative e_value:0	0.41	(0.1801 0.4394)	0.1801	0.4394
1292	WT010_M08_CLUSTER_34854	gi|50902216|ref|XP_463541.1| ankyrin-like protein [Oryza sativa (japonica cultivar-group)] e_value:0	11667.m06642|LOC_Os01g66110|protein methyltransferase, putative e_value:0	0.15	(0.0912 0.6068)	0.0912	0.6068
1293	SET5__C04_CLUSTER_11936	gi|50916134|ref|XP_468531.1| putative BRICK1 [Oryza sativa (japonica cultivar-group)] >gi|48716333|dbj|BAD22945.1| putative BRICK1 [Oryza sativa (japonica cultivar-group)] >gi|29371416|gb|AAO72702.1| unknown [Oryza sativa (japonica cultivar-group)]	11668.m05784|LOC_Os02g58320|protein expressed protein e_value:3.1567e-38	0.056	(0.0261 0.4673)	0.0261	0.4673
1294	SET1_I01_CLUSTER_5917	gi|50902308|ref|XP_463587.1| P0497A05.16 [Oryza sativa (japonica cultivar-group)] >gi|20804926|dbj|BAB92605.1| unknown protein [Oryza sativa (japonica cultivar-group)] >gi|56785117|dbj|BAD82755.1| unknown protein [Oryza sativa (japonica cultivar-gro	11667.m06855|LOC_Os01g68030|protein hypothetical protein e_value:1.92009e-25	0.319	(0.0818 0.2561)	0.0818	0.2561
1295	WT004_B10_CLUSTER_34443	gi|31433006|gb|AAP54570.1| unknown protein [Oryza sativa (japonica cultivar-group)] >gnl|BL_ORD_ID|2012601 gi|37535962|ref|NP_922283.1| unknown protein [Oryza sativa (japonica cultivar-group)] >gnl|BL_ORD_ID|2012601 gi|15144305|gb|AAK84446.1| unknow	11676.m03300|LOC_Os10g36690|protein expressed protein e_value:0	0.146	(0.0958 0.6573)	0.0958	0.6573
1296	SET3_I23_CLUSTER_22397	gi|50926660|ref|XP_473277.1| OSJNBa0081C01.13 [Oryza sativa (japonica cultivar-group)] >gi|38346762|emb|CAE03867.2| OSJNBa0081C01.13 [Oryza sativa (japonica cultivar-group)] >gi|3603473|gb|AAC35866.1| elicitor-responsive gene-3 [Oryza sativa]	11670.m04351|LOC_Os04g44870|protein elicitor-responsive gene 3 [imported] - rice e_value:5.1526e-66	0.223	(0.1136 0.5095)	0.1136	0.5095
1297	WT006_G23_CLUSTER_34754	gi|52353475|gb|AAU44041.1| unknown protein [Oryza sativa (japonica cultivar-group)] e_value:0	11682.m03971|LOC_Os05g41480|protein expressed protein	0.088	(0.0337 0.3810)	0.0337	0.381
1298	WT012_H06_CLUSTER_23163	gi|7267959|emb|CAB78300.1| polyubiquitin-like protein [Arabidopsis thaliana] >gnl|BL_ORD_ID|1458661 gi|5823568|emb|CAB41727.2| polyubiquitin-like protein [Arabidopsis thaliana] >gnl|BL_ORD_ID|1458661 gi|25407523|pir||H85134 polyubiquitin-like protei	11681.m02866|LOC_Os09g31090|protein Similar to polyubiquitin-like protein [imported] - Arabidopsis thaliana e_value:0	0.425	(0.2170 0.5109)	0.217	0.5109
1299	WT006_O07_CLUSTER_21581	gi|50251234|dbj|BAD27820.1| putative MAP kinase [Oryza sativa (japonica cultivar-group)] e_value:0	11668.m04356|LOC_Os02g45130|protein Ser/Thr kinase	0.246	(0.1441 0.5852)	0.1441	0.5852
1300	WT005_O10_CLUSTER_32167	gi|40643250|emb|CAC83720.1| cathepsin B [Hordeum vulgare subsp. vulgare] e_value:0	11682.m02264|LOC_Os05g24550|protein cathepsin B e_value:4.5759e-165	0.209	(0.1397 0.6689)	0.1397	0.6689
1301	WT003_I18_CLUSTER_3777	gi|50912633|ref|XP_467724.1| unknown protein [Oryza sativa (japonica cultivar-group)] >gnl|BL_ORD_ID|1582837 gi|46390323|dbj|BAD15772.1| unknown protein [Oryza sativa (japonica cultivar-group)] >gnl|BL_ORD_ID|1582837 gi|46390279|dbj|BAD15729.1| unkn	11668.m04954|LOC_Os02g50830|protein expressed protein e_value:3.55786e-88	0.101	(0.0325 0.3205)	0.0325	0.3205
1302	SET1_F07_CLUSTER_9568	gi|50922265|ref|XP_471493.1| OSJNBb0067G11.14 [Oryza sativa (japonica cultivar-group)] >gi|38347039|emb|CAD39891.2| OSJNBb0067G11.14 [Oryza sativa (japonica cultivar-group)] >gi|38344078|emb|CAE01738.2| OSJNBb0056F09.1 [Oryza sativa (japonica cultiv	11670.m01858|LOC_Os04g20280|protein Protein tyrosine phosphatase-like protein, PTPLA, putative e_value:4.98179e-104	0.1	(0.1174 1.1689)	0.1174	1.1689
1303	WT004_J20_CLUSTER_28725	gi|34910490|ref|NP_916592.1| putative mitochondrial processing peptidase beta subunit [Oryza sativa (japonica cultivar-group)] e_value:0	11667.m05021|LOC_Os01g51390|protein Insulinase (Peptidase family M16), putative e_value:0	0.242	(0.0829 0.3421)	0.0829	0.3421
1304	WT005_P01_CLUSTER_31494	gi|18855039|gb|AAL79731.1| putative protein phosphatase [Oryza sativa] >gnl|BL_ORD_ID|867115 gi|54291045|dbj|BAD61722.1| putative protein phosphatase 2C homolog [Oryza sativa (japonica cultivar-group)] e_value:0	11680.m05020|LOC_Os06g50380|protein Protein phosphatase 2C, putative e_value:0	0.098	(0.0437 0.4480)	0.0437	0.448
1305	SET3_P15_CLUSTER_4065	gi|50918667|ref|XP_469730.1| unknown protein [Oryza sativa (japonica cultivar-group)] >gi|14626292|gb|AAK71560.1| unknown protein [Oryza sativa (japonica cultivar-group)] e_value:1.23196e-124	11669.m05426|LOC_Os03g53590|protein expressed protein e_value:0	0.33	(0.2373 0.7201)	0.2373	0.7201
1306	WT010_E09_CLUSTER_2796	gi|34902044|ref|NP_912368.1| name [Oryza sativa (japonica cultivar-group)] >gnl|BL_ORD_ID|1588791 gi|29893654|gb|AAP06908.1| name [Oryza sativa (japonica cultivar-group)] >gnl|BL_ORD_ID|1588791 gi|29893655|gb|AAP06909.1| hypothetical protein [Oryza	11669.m01616|LOC_Os03g16140|protein expressed protein e_value:0	0.071	(0.0461 0.6465)	0.0461	0.6465
1307	WT007_I15_CLUSTER_2524	gi|50936739|ref|XP_477897.1| auxin-regulated protein-like protein [Oryza sativa (japonica cultivar-group)] >gnl|BL_ORD_ID|1526185 gi|33146928|dbj|BAC79949.1| auxin-regulated protein-like protein [Oryza sativa (japonica cultivar-group)] e_value:0	11673.m02483|LOC_Os07g26490|protein expressed protein e_value:0	0.179	(0.0983 0.5487)	0.0983	0.5487
1308	SET5_F14_CLUSTER_33573	gi|40363753|dbj|BAD06321.1| putative caffeoyl CoA O-methyltransferase [Triticum aestivum] e_value:9.2507e-144	11674.m03889|LOC_Os08g38910|protein O-methyltransferase e_value:3.80715e-98	0.733	(0.1792 0.2444)	0.1792	0.2444
1309	WT009_H01_CLUSTER_7070	gi|21740741|emb|CAD40550.1| OSJNBa0072K14.7 [Oryza sativa (japonica cultivar-group)] >gnl|BL_ORD_ID|1522093 gi|50923907|ref|XP_472314.1| OSJNBa0072K14.7 [Oryza sativa (japonica cultivar-group)] e_value:3.55456e-106	11670.m03091|LOC_Os04g32350|protein RNA polymerase III RPC4, putative e_value:9.646e-108	0.26	(0.1625 0.6253)	0.1625	0.6253
1310	WT011_B11_CLUSTER_26572	gi|50909997|ref|XP_466487.1| cyclin G-associated kinase-like protein [Oryza sativa (japonica cultivar-group)] >gnl|BL_ORD_ID|1577396 gi|50726471|dbj|BAD34080.1| cyclin G-associated kinase-like protein [Oryza sativa (japonica cultivar-group)] e_value:0	11668.m03616|LOC_Os02g37880|protein Protein kinase domain, putative e_value:0	0.152	(0.0891 0.5865)	0.0891	0.5865
1311	WT003_E23_CLUSTER_9137	gi|55733937|gb|AAV59444.1| putative esterase [Oryza sativa (japonica cultivar-group)] >gnl|BL_ORD_ID|1741354 gi|50931395|ref|XP_475225.1| putative esterase [Oryza sativa (japonica cultivar-group)] >gnl|BL_ORD_ID|1741354 gi|49328153|gb|AAT58849.1| un	11682.m03179|LOC_Os05g33820|protein expressed protein e_value:0	0.112	(0.0464 0.4130)	0.0464	0.413
1312	WT010_D13_CLUSTER_33760	gi|50940317|ref|XP_479686.1| F-box protein family-like [Oryza sativa (japonica cultivar-group)] >gnl|BL_ORD_ID|1601471 gi|51964602|ref|XP_507085.1| PREDICTED OJ1300_E01.9 gene product [Oryza sativa (japonica cultivar-group)] >gnl|BL_ORD_ID|1601471	11674.m00048|LOC_Os08g01430|protein F-box domain, putative e_value:0	0.316	(0.1391 0.4397)	0.1391	0.4397
1313	WT010_P03_CLUSTER_30153	gi|50941331|ref|XP_480193.1| putative Band 7 protein [Oryza sativa (japonica cultivar-group)] >gnl|BL_ORD_ID|1602353 gi|51964706|ref|XP_507137.1| PREDICTED P0498E12.110 gene product [Oryza sativa (japonica cultivar-group)] >gnl|BL_ORD_ID|1602353	11674.m00548|LOC_Os08g06200|protein SPFH domain / Band 7 family, putative e_value:5.88317e-121	0.239	(0.2701 1.1291)	0.2701	1.1291
1314	WT005_J05_CLUSTER_32043	gi|50899020|ref|XP_450298.1| putative acetyl-CoA C-acyltransferase [Oryza sativa (japonica cultivar-group)] >gnl|BL_ORD_ID|1563548 gi|47848479|dbj|BAD22334.1| putative acetyl-CoA C-acyltransferase [Oryza sativa (japonica cultivar-group)] e_value:0	11681.m00606|LOC_Os09g07830|protein acetyl-CoA acyltransferases e_value:0	0.083	(0.0415 0.4992)	0.0415	0.4992
1315	SET5_E10_CLUSTER_6733	gi|51979370|ref|XP_507533.1| PREDICTED OJ1175_B01.8-1 gene product [Oryza sativa (japonica cultivar-group)] >gi|51964402|ref|XP_506986.1| PREDICTED OJ1175_B01.8-1 gene product [Oryza sativa (japonica cultivar-group)] >gi|50912987|ref|XP_467901.1| pu	11668.m05108|LOC_Os02g52230|protein F-box domain, putative e_value:0	0.181	(0.0833 0.4608)	0.0833	0.4608
1316	WT003_G14_CLUSTER_25759	gi|22074759|gb|AAM19067.1| RNase L inhibitor-like protein [Oryza sativa (japonica cultivar-group)] e_value:0	11687.m03136|LOC_Os11g34350|protein ABC transporter, putative e_value:0	0.054	(0.0413 0.7638)	0.0413	0.7638
1317	WT010_P15_CLUSTER_27389	gi|50905193|ref|XP_464085.1| putative beta-1,3-glucanase [Oryza sativa (japonica cultivar-group)] >gnl|BL_ORD_ID|1565941 gi|51963864|ref|XP_506715.1| PREDICTED OSJNBa0026E05.37 gene product [Oryza sativa (japonica cultivar-group)]	11668.m00404|LOC_Os02g04670|protein Glycosyl hydrolases family 17, putative e_value:0	0.271	(0.1446 0.5328)	0.1446	0.5328
1318	WT009_P20_CLUSTER_22519	gi|50933211|ref|XP_476133.1| putative alpha-mannosidase [Oryza sativa (japonica cultivar-group)] >gnl|BL_ORD_ID|1600924 gi|46576022|gb|AAT01383.1| putative alpha-mannosidase [Oryza sativa (japonica cultivar-group)] e_value:0	11682.m01120|LOC_Os05g11850|protein endoplasmic reticulum alpha-mannosidase, putative e_value:0	0.17	(0.0652 0.3832)	0.0652	0.3832
1319	WT004_F06_CLUSTER_33284	gi|51963444|ref|XP_506365.1| PREDICTED P0048D08.105 gene product [Oryza sativa (japonica cultivar-group)] >gnl|BL_ORD_ID|1527767 gi|50937751|ref|XP_478403.1| CDP2_ORYSA Calcium-dependent protein kinase [Oryza sativa (japonica cultivar-group)] >gnl|B	11673.m03170|LOC_Os07g33110|protein Protein kinase domain, putative e_value:0	0.049	(0.0330 0.6757)	0.033	0.6757
1320	WT009_I10_CLUSTER_32201	gi|18481703|gb|AAL73525.1| hypothetical protein S250_18C08.11 [Sorghum bicolor] e_value:9.24317e-117	11680.m04967|LOC_Os06g49930|protein F-box domain, putative e_value:1.10373e-107	0.433	(0.3769 0.8709)	0.3769	0.8709
1321	WT005_K18_CLUSTER_31167	gi|9082278|gb|AAF82787.1| alpha 1,4-glucan phosphorylase; cytosolic starch phosphorylase [Triticum aestivum] >gnl|BL_ORD_ID|1820480 gi|14916632|sp|Q9LKJ3|PHSH_WHEAT Alpha-glucan phosphorylase, H isozyme (Starch phosphorylase H) e_value:0	11667.m06333|LOC_Os01g63270|protein Carbohydrate phosphorylase e_value:0	0.063	(0.0340 0.5435)	0.034	0.5435
1322	WT012_H16_CLUSTER_19295	gi|50726532|dbj|BAD34139.1| GDSL-motif lipase/hydrolase-like [Oryza sativa (japonica cultivar-group)] >gnl|BL_ORD_ID|382878 gi|47848444|dbj|BAD22300.1| GDSL-motif lipase/hydrolase-like [Oryza sativa (japonica cultivar-group)] e_value:1.99108e-162	11681.m00386|LOC_Os09g04710|protein GDSL-like Lipase/Acylhydrolase e_value:5.32156e-164	0.359	(0.1195 0.3331)	0.1195	0.3331
1323	WT005_L24_CLUSTER_31226	gi|2655098|gb|AAC32034.1| peptide transporter [Hordeum vulgare] >gnl|BL_ORD_ID|361171 gi|7489404|pir||T04378 peptide transport protein - barley e_value:0	11667.m00422|LOC_Os01g04950|protein POT family, putative e_value:0	0.261	(0.1178 0.4517)	0.1178	0.4517

PDF Complete
Your complimentary use period has ended. Thank you for using PDF Complete.
Click Here to upgrade to Unlimited Pages and Expanded Features

<table>
<tr><td>1324</td><td>SET1_J16_CLUSTER_33523</td><td>gi|50931505|ref|XP_475280.1| unknown protein [Oryza sativa (japonica cultivar-group)] >gi|49328048|gb|AAT58749.1| unknown protein [Oryza sativa (japonica cultivar-group)] >gi|48843787|gb|AAT47046.1| putative gibberellin regulated protein [Oryza sati</td><td>11682.m03376|LOC_Os05g35690|protein Similar to GAST-like gene product e_value:4.35997e-40</td><td>0.668</td><td>(0.1990 0.2978)</td><td>0.199</td><td>0.2978</td></tr>
<tr><td>1325</td><td>SET3_A15_CLUSTER_32223</td><td>gi|50918197|ref|XP_469495.1| putative basic protein [Oryza sativa] >gi|62733550|gb|AAX95667.1| Plastocyanin-like domain, putative [Oryza sativa (japonica cultivar-group)] e_value:9.39503e-43</td><td>11669.m05052|LOC_Os03g50160|protein putative basic protein e_value:7.08603e-45</td><td>0.468</td><td>(0.1772 0.3785)</td><td>0.1772</td><td>0.3785</td></tr>
<tr><td>1326</td><td>SET2_F08_CLUSTER_33157</td><td>gi|34912300|ref|NP_917497.1| P0451D05.13 [Oryza sativa (japonica cultivar-group)] >gi|20521301|dbj|BAB91815.1| unknown protein [Oryza sativa (japonica cultivar-group)] >gi|20804621|dbj|BAB92311.1| unknown protein [Oryza sativa (japonica cultivar-gro</td><td>11667.m03962|LOC_Os01g41140|protein hypothetical protein e_value:7.43465e-37</td><td>0.706</td><td>(0.4183 0.5925)</td><td>0.4183</td><td>0.5925</td></tr>
<tr><td>1327</td><td>WT004_A07_CLUSTER_14029</td><td>gi|50931213|ref|XP_475134.1| putative serine protease [Oryza sativa (japonica cultivar-group)] >gnl|BL_ORD_ID|1596342 gi|47777389|gb|AAT38023.1| putative serine protease [Oryza sativa (japonica cultivar-group)] e_value:0</td><td>11682.m02833|LOC_Os05g30580|protein Subtilisin N-terminal Region, putative e_value:0</td><td>0.507</td><td>(0.2049 0.4040)</td><td>0.2049</td><td>0.404</td></tr>
<tr><td>1328</td><td>SET6_G02_CLUSTER_33044</td><td>gi|50947989|ref|XP_483522.1| putative MtN3 [Oryza sativa (japonica cultivar-group)] >gi|51965048|ref|XP_507308.1| PREDICTED P0702C09.33 gene product [Oryza sativa (japonica cultivar-group)] >gi|45736077|dbj|BAD13102.1| putative MtN3 [Oryza sativa (j</td><td>11674.m04265|LOC_Os08g42350|protein MtN3/saliva family, putative e_value:5.02105e-95</td><td>0.833</td><td>(0.2610 0.3134)</td><td>0.261</td><td>0.3134</td></tr>
<tr><td>1329</td><td>WT010_G04_CLUSTER_20346</td><td>gi|40363459|dbj|BAD06219.1| plastidic 2-oxoglutarate/malate transporter [Zea mays] e_value:0</td><td>11686.m03230|LOC_Os12g33080|protein 2-oxoglutarate/malate translocator precursor-like protein e_value:0</td><td>0.102</td><td>(0.0732 0.7212)</td><td>0.0732</td><td>0.7212</td></tr>
<tr><td>1330</td><td>SET2_G14_CLUSTER_11461</td><td>gi|55832256|gb|AAV65331.1| pg4 [Hordeum vulgare] e_value:6.04344e-115</td><td>11669.m01289|LOC_Os03g13220|protein PG4 e_value:8.73295e-</td><td>0.089</td><td>(0.0443 0.4979)</td><td>0.0443</td><td>0.4979</td></tr>
<tr><td>1331</td><td>SET4_M15_CLUSTER_24462</td><td>gi|62734083|gb|AAX96192.1| F-box domain, putative [Oryza sativa (japonica cultivar-group)] e_value:0</td><td>11687.m00736|LOC_Os11g07970|protein F-box domain, putative e_value:0</td><td>0.235</td><td>(0.1163 0.4957)</td><td>0.1163</td><td>0.4957</td></tr>
<tr><td>1332</td><td>WT009__C09_CLUSTER_13455</td><td>gi|50915498|ref|XP_468213.1| putative nucleotide sugar epimerase [Oryza sativa (japonica cultivar-group)] >gnl|BL_ORD_ID|1583334 gi|47497123|dbj|BAD19172.1| putative nucleotide sugar epimerase [Oryza sativa (japonica cultivar-group)] >gnl|BL_ORD_ID|</td><td>11668.m05407|LOC_Os02g54890|protein nucleotide sugar epimerase-like protein [imported] - Arabidopsis thaliana</td><td>0.109</td><td>(0.0310 0.2856)</td><td>0.031</td><td>0.2856</td></tr>
<tr><td>1333</td><td>SET2_I23_CLUSTER_33793</td><td>gi|34912454|ref|NP_917574.1| P0681B11.31 [Oryza sativa (japonica cultivar-group)] >gi|20804777|dbj|BAB92461.1| MtN3-like [Oryza sativa (japonica cultivar-group)] >gi|17385713|dbj|BAB78664.1| MtN3-like [Oryza sativa (japonica cultivar-group)] e_value</td><td>11667.m04060|LOC_Os01g42110|protein MtN3/saliva family, putative e_value:5.71583e-102</td><td>0.353</td><td>(0.1807 0.5125)</td><td>0.1807</td><td>0.5125</td></tr>
<tr><td>1334</td><td>WT013_L23_CLUSTER_23531</td><td>gi|31193910|gb|AAP44745.1| unknown protein [Oryza sativa (japonica cultivar-group)] >gnl|BL_ORD_ID|1917589 gi|50920311|ref|XP_470516.1| unknown protein [Oryza sativa (japonica cultivar-group)] e_value:4.94832e-143</td><td>11669.m06611|LOC_Os03g64350|protein expressed protein e_value:6.49273e-154</td><td>0.243</td><td>(0.2283 0.9411)</td><td>0.2283</td><td>0.9411</td></tr>
<tr><td>1335</td><td>WT013_J13_CLUSTER_20898</td><td>gi|10177227|dbj|BAB10601.1| alanyl-tRNA synthetase [Arabidopsis thaliana] e_value:0</td><td>11680.m01345|LOC_Os06g13660|protein alanyl-tRNA synthetase, putative e_value:0</td><td>0.144</td><td>(0.0908 0.6312)</td><td>0.0908</td><td>0.6312</td></tr>
<tr><td>1336</td><td>WT012_N18_CLUSTER_33616</td><td>gi|50943155|ref|XP_481105.1| putative cytochrome P450 [Oryza sativa (japonica cultivar-group)] >gnl|BL_ORD_ID|1605948 gi|51964796|ref|XP_507182.1| PREDICTED OSJNBb0005C03.18 gene product [Oryza sativa (japonica cultivar-group)] >gnl|BL_ORD_ID|160594</td><td>11674.m01592|LOC_Os08g16260|protein probable cytochrome P450 [imported] - Arabidopsis thaliana e_value:1.87587e-163</td><td>0.322</td><td>(0.3485 1.0827)</td><td>0.3485</td><td>1.0827</td></tr>
<tr><td>1337</td><td>WT012_L08_CLUSTER_12835</td><td>gi|33302327|gb|AAQ01784.1| resistance protein LR10 [Triticum aestivum] e_value:0</td><td>11668.m03340|LOC_Os02g35210|protein NB-ARC domain, putative e_value:0</td><td>0.243</td><td>(0.5431 2.2345)</td><td>0.5431</td><td>2.2345</td></tr>
<tr><td>1338</td><td>WT012_J07_CLUSTER_6170</td><td>gi|50253214|dbj|BAD29470.1| GL2-type homeobox genes [Oryza sativa (japonica cultivar-group)] e_value:0</td><td>11668.m04369|LOC_Os02g45250|protein START domain, putative e_value:0</td><td>0.126</td><td>(0.0632 0.5024)</td><td>0.0632</td><td>0.5024</td></tr>
<tr><td>1339</td><td>WT011_P24_CLUSTER_24014</td><td>gi|31429980|gb|AAP51957.1| putative transposase [Oryza sativa (japonica cultivar-group)] >gnl|BL_ORD_ID|2007908 gi|37530736|ref|NP_919670.1| putative transposase [Oryza sativa (japonica cultivar-group)] >gnl|BL_ORD_ID|2007908 gi|20042909|gb|AAM08737</td><td>11668.m01273|LOC_Os02g13770|protein transposon protein, putative, CACTA, En/Spm sub-class e_value:8.07408e-153</td><td>0.285</td><td>(0.1912 0.6720)</td><td>0.1912</td><td>0.672</td></tr>
<tr><td>1340</td><td>WT010_I07_CLUSTER_29350</td><td>gi|29126360|gb|AAO66552.1| putative cleavage and polyadenylation specifity factor [Oryza sativa (japonica cultivar-group)] >gnl|BL_ORD_ID|2058538 gi|50920149|ref|XP_470435.1| putative cleavage and polyadenylation specifity factor protein [Oryza sativa</td><td>11669.m06521|LOC_Os03g63590|protein putative cleavage and polyadenylation specifity factor e_value:0</td><td>0.064</td><td>(0.0448 0.7035)</td><td>0.0448</td><td>0.7035</td></tr>
<tr><td>1341</td><td>WT009_P24_CLUSTER_22808</td><td>gi|31432758|gb|AAP54351.1| putative cytochrome P450 protein [Oryza sativa (japonica cultivar-group)] >gnl|BL_ORD_ID|2012237 gi|37535524|ref|NP_922064.1| putative cytochrome P450 protein [Oryza sativa (japonica cultivar-group)] >gnl|BL_ORD_ID|2012237</td><td>11676.m03056|LOC_Os10g34480|protein putative cytochrome P450 protein e_value:0</td><td>0.282</td><td>(0.0939 0.3335)</td><td>0.0939</td><td>0.3335</td></tr>
<tr><td>1342</td><td>WT007_F12_CLUSTER_6161</td><td>gi|56784406|dbj|BAD82445.1| putative nitrate transporter NRT1-5 [Oryza sativa (japonica cultivar-group)] e_value:0</td><td>11667.m06907|LOC_Os01g68510|protein POT family, putative e_value:0</td><td>0.23</td><td>(0.1253 0.5445)</td><td>0.1253</td><td>0.5445</td></tr>
<tr><td>1343</td><td>WT007_E11_CLUSTER_7158</td><td>gi|51091382|dbj|BAD36115.1| phosphatidic acid phosphatase-like [Oryza sativa (japonica cultivar-group)] e_value:3.95535e-110</td><td>11680.m03952|LOC_Os06g40500|protein At3g15820/MSJ11_22 e_value:1.06236e-111</td><td>0.507</td><td>(0.1952 0.3848)</td><td>0.1952</td><td>0.3848</td></tr>
<tr><td>1344</td><td>WT005_H11_CLUSTER_3618</td><td>gi|50252184|dbj|BAD28179.1| unknown protein [Oryza sativa (japonica cultivar-group)] >gnl|BL_ORD_ID|235109 gi|50251254|dbj|BAD28034.1| unknown protein [Oryza sativa (japonica cultivar-group)] e_value:2.36874e-71</td><td>11668.m00586|LOC_Os02g06390|protein expressed protein e_value:2.32442e-73</td><td>0.277</td><td>(0.1136 0.4097)</td><td>0.1136</td><td>0.4097</td></tr>
<tr><td>1345</td><td>WT003_H23_CLUSTER_7079</td><td>gi|21740742|emb|CAD40551.1| OSJNBa0072K14.6 [Oryza sativa (japonica cultivar-group)] >gnl|BL_ORD_ID|1522092 gi|50923905|ref|XP_472313.1| OSJNBa0072K14.6 [Oryza sativa (japonica cultivar-group)] e_value:0</td><td>11670.m03090|LOC_Os04g32340|protein expressed protein e_value:0</td><td>0.305</td><td>(0.1391 0.4567)</td><td>0.1391</td><td>0.4567</td></tr>
<tr><td>1346</td><td>SET5_A08_CLUSTER_6593</td><td>gi|50939727|ref|XP_479391.1| unknown protein [Oryza sativa (japonica cultivar-group)] >gi|51449865|gb|AAU01905.1| hypothetical protein [Oryza sativa (indica cultivar-group)] >gi|23617114|dbj|BAC20796.1| unknown protein [Oryza sativa (japonica cultiv</td><td>11673.m04554|LOC_Os07g46210|protein hypothetical protein e_value:1.10395e-93</td><td>0.404</td><td>(0.2379 0.5891)</td><td>0.2379</td><td>0.5891</td></tr>
<tr><td>1347</td><td>SET4_G02_CLUSTER_32667</td><td>gi|34907342|ref|NP_915018.1| putative elicitor response protein [Oryza sativa (japonica cultivar-group)] >gi|22202663|dbj|BAC07321.1| putative Avr9 elicitor response protein [Oryza sativa (japonica cultivar-group)] e_value:0</td><td>11667.m06590|LOC_Os01g65590|protein Galactosyltransferase, putative e_value:0</td><td>0.324</td><td>(0.1073 0.3316)</td><td>0.1073</td><td>0.3316</td></tr>
<tr><td>1348</td><td>SET4_D03_CLUSTER_31335</td><td>gi|50945893|ref|XP_482474.1| unknown protein [Oryza sativa (japonica cultivar-group)] >gi|37572928|dbj|BAC98558.1| unknown protein [Oryza sativa (japonica cultivar-group)] >gi|37806295|dbj|BAC99810.1| unknown protein [Oryza sativa (japonica cultivar</td><td>11674.m03250|LOC_Os08g32910|protein hypothetical protein e_value:0</td><td>0.179</td><td>(0.1034 0.5775)</td><td>0.1034</td><td>0.5775</td></tr>
<tr><td>1349</td><td>SET4_A12_CLUSTER_23533</td><td>gi|50927438|ref|XP_473395.1| OSJNBa0079A21.6 [Oryza sativa (japonica cultivar-group)] >gi|38346159|emb|CAD41462.2| OSJNBa0079A21.6 [Oryza sativa (japonica cultivar-group)] e_value:2.49042e-101</td><td>11670.m04491|LOC_Os04g46140|protein expressed protein e_value:7.08828e-103</td><td>1.009</td><td>(0.2797 0.2771)</td><td>0.2797</td><td>0.2771</td></tr>
<tr><td>1350</td><td>SET4__C01_CLUSTER_6615</td><td>gi|34898790|ref|NP_910741.1| unknown protein [Oryza sativa (japonica cultivar-group)] >gi|51963462|ref|XP_506389.1| PREDICTED OJ1131_E05.126 gene product [Oryza sativa (japonica cultivar-group)] >gi|50508290|dbj|BAD32139.1| unknown protein [Oryza sa</td><td>11673.m03376|LOC_Os07g35060|protein F-box domain, putative e_value:4.69619e-61</td><td>0.432</td><td>(0.5485 1.2685)</td><td>0.5485</td><td>1.2685</td></tr>
<tr><td>1351</td><td>SET3_E24_CLUSTER_9759</td><td>gi|50940057|ref|XP_479556.1| putative ATP-dependent DNA helicase recQ [Oryza sativa (japonica cultivar-group)] >gi|33146670|dbj|BAC80016.1| putative ATP-dependent DNA helicase recQ [Oryza sativa (japonica cultivar-group)] e_value:0</td><td>11673.m04797|LOC_Os07g48360|protein ATP-dependent DNA helicase, RecQ family, putative e_value:0</td><td>0.248</td><td>(0.1345 0.5428)</td><td>0.1345</td><td>0.5428</td></tr>
<tr><td>1352</td><td>SET1_J11_CLUSTER_28730</td><td>gi|50924127|ref|XP_472424.1| OJ991214_12.11 [Oryza sativa (japonica cultivar-group)] >gi|32479735|emb|CAE01522.1| OJ991214_12.11 [Oryza sativa (japonica cultivar-group)] e_value:2.29218e-36</td><td>11670.m03263|LOC_Os04g33920|protein Protease inhibitor/seed storage/LTP family, putative e_value:2.85947e-38</td><td>0.456</td><td>(0.3087 0.6769)</td><td>0.3087</td><td>0.6769</td></tr>
<tr><td>1353</td><td>SET1_F13_CLUSTER_28536</td><td>gi|4090293|emb|CAA10497.1| hypothetical protein [Secale cereale] e_value:9.77065e-99</td><td>11686.m01934|LOC_Os12g19410|protein Ribulose bisphosphate carboxylase, small subunit, putative e_value:4.37557e-88</td><td>0.194</td><td>(0.0903 0.4656)</td><td>0.0903</td><td>0.4656</td></tr>
<tr><td>1354</td><td>SET1_D21_CLUSTER_3445</td><td>gi|50909475|ref|XP_466226.1| unknown protein [Oryza sativa (japonica cultivar-group)] >gi|46390911|dbj|BAD16426.1| unknown protein [Oryza sativa (japonica cultivar-group)] e_value:4.2084e-35</td><td>11668.m03339|LOC_Os02g35200|protein molybdopterin converting factor, subunit 1, putative e_value:7.43885e-37</td><td>0.465</td><td>(0.1706 0.3671)</td><td>0.1706</td><td>0.3671</td></tr>
<tr><td>1355</td><td>SET1__C03_CLUSTER_33616</td><td>gi|21553376|gb|AAM62469.1| acyl carrier protein, putative [Arabidopsis thaliana] e_value:5.07846e-33</td><td>11669.m02364|LOC_Os03g22950|protein acyl carrier protein, putative e_value:1.40736e-53</td><td>0.241</td><td>(0.1080 0.4482)</td><td>0.108</td><td>0.4482</td></tr>
<tr><td>1356</td><td>WT013_N06_CLUSTER_18458</td><td>gi|57900688|gb|AAW57813.1| putative cytochrome P450 [Oryza sativa (japonica cultivar-group)] e_value:0</td><td>11682.m00857|LOC_Os05g08850|protein Cytochrome P450</td><td>0.544</td><td>(0.1740 0.3201)</td><td>0.174</td><td>0.3201</td></tr>
<tr><td>1357</td><td>WT013_I09_CLUSTER_33379</td><td>gi|50910621|ref|XP_466799.1| putative CER1 protein [Oryza sativa (japonica cultivar-group)] >gnl|BL_ORD_ID|1579181 gi|47847803|dbj|BAD21579.1| putative CER1 protein [Oryza sativa (japonica cultivar-group)] >gnl|BL_ORD_ID|1579181</td><td>11668.m03930|LOC_Os02g40780|protein expressed protein e_value:0</td><td>0.122</td><td>(0.0859 0.7013)</td><td>0.0859</td><td>0.7013</td></tr>
<tr><td>1358</td><td>WT005_L20_CLUSTER_21278</td><td>gi|38567631|emb|CAE02615.1| RAFTIN1b protein [Triticum aestivum] >gnl|BL_ORD_ID|1107401 gi|38567629|emb|CAE02614.1| RAFTIN1b protein [Triticum aestivum] e_value:0</td><td>11674.m03873|LOC_Os08g38810|protein BURP domain, putative e_value:1.07169e-119</td><td>0.684</td><td>(0.2563 0.3748)</td><td>0.2563</td><td>0.3748</td></tr>
<tr><td>1359</td><td>WT002_M07_CLUSTER_955</td><td>gi|51535041|dbj|BAD37412.1| putative Gl1 [Oryza sativa (japonica cultivar-group)] e_value:0</td><td>11680.m04356|LOC_Os06g44300|protein glossy1 homolog - rice (fragment) e_value:0</td><td>0.232</td><td>(0.1180 0.5089)</td><td>0.118</td><td>0.5089</td></tr>
<tr><td>1360</td><td>WT010_P17_CLUSTER_3178</td><td>gi|55741322|ref|XP_550326.1| proline-rich cell wall protein-like [Oryza sativa (japonica cultivar-group)] >gnl|BL_ORD_ID|1743614 gi|55296060|dbj|BAD67622.1| proline-rich cell wall protein-like [Oryza sativa (japonica cultivar-group)] e_value:2.97466e-9</td><td>11680.m00597|LOC_Os06g06530|protein expressed protein e_value:6.66387e-100</td><td>0.248</td><td>(0.1132 0.4562)</td><td>0.1132</td><td>0.4562</td></tr>
<tr><td>1361</td><td>WT009_M21_CLUSTER_29104</td><td>gi|54287472|gb|AAV31216.1| putative transcription factor IIIA [Oryza sativa (japonica cultivar-group)] e_value:1.12358e-129</td><td>11668.m00152|LOC_Os02g02420|protein Zinc finger, C2H2 type, putative e_value:2.30492e-168</td><td>0.2</td><td>(0.1588 0.7954)</td><td>0.1588</td><td>0.7954</td></tr>
<tr><td>1362</td><td>SET1_J24_CLUSTER_18732</td><td>gi|34894220|ref|NP_908435.1| putative nodulin-26 precursor [Oryza sativa (japonica cultivar-group)] >gi|14587262|dbj|BAB61180.1| putative nodulin-26 precursor [Oryza sativa (japonica cultivar-group)] >gi|13486658|dbj|BAB39896.1| putative nodulin-26</td><td>11667.m00129|LOC_Os01g02190|protein Major intrinsic protein, putative e_value:2.12113e-93</td><td>0.47</td><td>(0.2347 0.4990)</td><td>0.2347</td><td>0.499</td></tr>
<tr><td>1363</td><td>WT006_M23_CLUSTER_23887</td><td>gi|52076908|dbj|BAD45920.1| putative Rab geranylgeranyl transferase, a subunit [Oryza sativa (japonica cultivar-group)] >gnl|BL_ORD_ID|251654 gi|52076622|dbj|BAD45523.1| putative Rab geranylgeranyl transferase, a subunit [Oryza sativa (japonica cultiva</td><td>11680.m04576|LOC_Os06g46380|protein Protein prenyltransferase alpha subunit repeat, putative e_value:0</td><td>0.181</td><td>(0.1171 0.6467)</td><td>0.1171</td><td>0.6467</td></tr>
<tr><td>1364</td><td>WT011_N21_CLUSTER_34341</td><td>gi|50911927|ref|XP_467371.1| putative tubby-like protein [Oryza sativa (japonica cultivar-group)] >gnl|BL_ORD_ID|1580502 gi|41053094|dbj|BAD08037.1| putative tubby-like protein [Oryza sativa (japonica cultivar-group)] e_value:0</td><td>11668.m04621|LOC_Os02g47640|protein F-box domain, putative e_value:0</td><td>0.123</td><td>(0.0572 0.4661)</td><td>0.0572</td><td>0.4661</td></tr>
<tr><td>1365</td><td>SET5_F20_CLUSTER_33815</td><td>gi|50929259|ref|XP_474157.1| OSJNBa0060D06.16 [Oryza sativa (japonica cultivar-group)] >gi|32488878|emb|CAE03550.1| OSJNBa0060D06.16 [Oryza sativa (japonica cultivar-group)] e_value:3.40344e-64</td><td>11670.m05510|LOC_Os04g55920|protein expressed protein e_value:6.14041e-66</td><td>0.291</td><td>(0.1649 0.5673)</td><td>0.1649</td><td>0.5673</td></tr>
<tr><td>1366</td><td>WT004_B13_CLUSTER_10494</td><td>gi|21592319|gb|AAM64270.1| unknown [Arabidopsis thaliana] e_value:2.15716e-32</td><td>11686.m03730|LOC_Os12g37880|protein expressed protein e_value:6.25155e-52</td><td>0.129</td><td>(0.0455 0.3522)</td><td>0.0455</td><td>0.3522</td></tr>
<tr><td>1367</td><td>SET4_F12_CLUSTER_450</td><td>gi|50918917|ref|XP_469855.1| putative protein of gibberellin-stimulated transcript [Oryza sativa (japonica cultivar-group)] >gi|14488366|gb|AAK63933.1| putative protein of gibberellin-stimulated transcript [Oryza sativa (japonica cultivar-group)] >g</td><td>11669.m05563|LOC_Os03g55290|protein putative protein of gibberellin-stimulated transcript e_value:8.35834e-44</td><td>0.222</td><td>(0.0858 0.3874)</td><td>0.0858</td><td>0.3874</td></tr>
</table>

PDF Complete
Your complimentary use period has ended. Thank you for using PDF Complete.
Click Here to upgrade to Unlimited Pages and Expanded Features

1368	WT004_I20_CLUSTER_4601	gi|3309066|gb|AAC26003.1| bifunctional dihydrofolate reductase-thymidylate synthase [Zea mays] >gnl|BL_ORD_ID|741146 gi|6685381|sp|O81395|DRTS_MAIZE Bifunctional dihydrofolate reductase-thymidylate synthase (DHFR-TS) [Includes: Dihydrofolate	11686.m02510|LOC_Os12g26060|protein thymidylate synthase, putative e_value:0	0.137	(0.0895 0.6548)	0.0895	0.6548
1369	WT003_E16_CLUSTER_15878	gi|53793314|dbj|BAD54535.1| putative ATPase, aminophospholipid transporter (APLT), class I, type 8A, member 1 [Oryza sativa (japonica cultivar-group)] >gnl|BL_ORD_ID|261111 gi|53793271|dbj|BAD54494.1| putative ATPase, aminophospholipid transporter (APL	11680.m03591|LOC_Os06g36990|protein Similar to potential phospholipid-transporting atpase 4 (ec 3.6.3.1)(aminophospholipid flippase 4) e_value:0	0.145	(0.0576 0.3968)	0.0576	0.3968
1370	WT007__C09_CLUSTER_8733	gi|56784589|dbj|BAD81636.1| putative beta-1,3-glucanase [Oryza sativa (japonica cultivar-group)] >gnl|BL_ORD_ID|775503 gi|56784550|dbj|BAD81597.1| putative beta-1,3-glucanase [Oryza sativa (japonica cultivar-group)] e_value:2.25042e-53	11667.m01399|LOC_Os01g14140|protein hypothetical protein e_value:2.52145e-50	0.203	(0.1350 0.6662)	0.135	0.6662
1371	WT009_N11_CLUSTER_15301	gi|34897088|ref|NP_909890.1| hypothetical protein [Oryza sativa (japonica cultivar-group)] >gnl|BL_ORD_ID|1587223 gi|12957704|gb|AAK09222.1| hypothetical protein [Oryza sativa (japonica cultivar-group)] e_value:7.10589e-97	11673.m00734|LOC_Os07g08030|protein ribose 5-phosphate isomerase A e_value:1.9861e-25	0.551	(0.7921 1.4373)	0.7921	1.4373
1372	SET1_O03_CLUSTER_13017	gi|50920527|ref|XP_470624.1| Hypothetical protein [Oryza sativa (japonica cultivar-group)] >gi|20330770|gb|AAM19133.1| Hypothetical protein [Oryza sativa (japonica cultivar-group)] e_value:9.31626e-172	11669.m01269|LOC_Os03g13040|protein Haemolysin-III related, putative e_value:0	0.148	(0.0776 0.5241)	0.0776	0.5241
1373	WT013_D13_CLUSTER_33864	gi|31433051|gb|AAP54611.1| putative cytochrome P450 [Oryza sativa (japonica cultivar-group)] >gnl|BL_ORD_ID|2012642 gi|37536044|ref|NP_922324.1| putative cytochrome P450 [Oryza sativa (japonica cultivar-group)] >gnl|BL_ORD_ID|2012642	11676.m03344|LOC_Os10g37110|protein transposon protein, putative, unclassified e_value:0	0.356	(0.1430 0.4022)	0.143	0.4022
1374	WT009_E21_CLUSTER_23734	gi|51091315|dbj|BAD36050.1| putative endomembrane protein emp70 precursor [Oryza sativa (japonica cultivar-group)] e_value:0	11668.m05465|LOC_Os02g55440|protein Similar to endosomal protein e_value:0	0.102	(0.0680 0.6648)	0.068	0.6648
1375	WT009_D23_CLUSTER_33006	gi|38345944|emb|CAE04269.2| OSJNBb0103I08.15 [Oryza sativa (japonica cultivar-group)] >gnl|BL_ORD_ID|1522749 gi|50927230|ref|XP_473374.1| OSJNBb0103I08.15 [Oryza sativa (japonica cultivar-group)] >gnl|BL_ORD_ID|1522749 gi|44953685|gb|AAS49494.1| iro	11670.m04463|LOC_Os04g45900|protein transposon protein, putative, unclassified e_value:0	0.203	(0.0688 0.3396)	0.0688	0.3396
1376	WT008_N12_CLUSTER_17075	gi|31431995|gb|AAP53694.1| unknown protein [Oryza sativa (japonica cultivar-group)] >gnl|BL_ORD_ID|2011139 gi|37534210|ref|NP_921407.1| unknown protein [Oryza sativa (japonica cultivar-group)] >gnl|BL_ORD_ID|2011139 gi|15451557|gb|AAK98681.1| Unknow	11676.m02287|LOC_Os10g26660|protein expressed protein e_value:3.09524e-161	0.254	(0.0890 0.3495)	0.089	0.3495
1377	WT004_H09_CLUSTER_9034	gi|50919433|ref|XP_470113.1| unknown protein [Oryza sativa (japonica cultivar-group)] >gnl|BL_ORD_ID|1588187 gi|28876013|gb|AAO60022.1| unknown protein [Oryza sativa (japonica cultivar-group)] e_value:0	11669.m06135|LOC_Os03g60350|protein expressed protein e_value:0	0.152	(0.0875 0.5746)	0.0875	0.5746
1378	SET3_G20_CLUSTER_4739	gi|54291347|dbj|BAD62113.1| unknown protein [Oryza sativa (japonica cultivar-group)] e_value:2.20582e-22	11680.m05126|LOC_Os06g51320|protein gibberellin-regulated protein, putative e_value:1.44741e-24	0.48	(0.4359 0.9077)	0.4359	0.9077
1379	SET5_E05_CLUSTER_11081	gi|50901996|ref|XP_463431.1| putative dynein light chain [Oryza sativa (japonica cultivar-group)] >gi|14587295|dbj|BAB61206.1| putative dynein light chain [Oryza sativa (japonica cultivar-group)] e_value:5.47019e-41	11667.m05478|LOC_Os01g55510|protein Dynein light chain type 1 e_value:5.40357e-43	0.232	(0.0763 0.3285)	0.0763	0.3285
1380	SET2_I03_CLUSTER_17399	gi|50917575|ref|XP_469184.1| putative small GTP-binding protein [Oryza sativa (japonica cultivar-group)] >gi|40538929|gb|AAR87186.1| putative small GTP-binding protein [Oryza sativa (japonica cultivar-group)] e_value:6.50151e-102	11669.m04636|LOC_Os03g46390|protein putative small GTP-binding protein e_value:1.29953e-103	0.046	(0.0263 0.5768)	0.0263	0.5768
1381	WT004_M15_CLUSTER_33398	gi|37730876|gb|AAO63625.1| legumin-like protein [Zea mays] e_value:9.48082e-175	11667.m07487|LOC_Os01g74480|protein Cupin, putative e_value:8.26349e-176	0.294	(0.1255 0.4271)	0.1255	0.4271
1382	SET3_N08_CLUSTER_25649	gi|50940147|ref|XP_479601.1| unknown protein [Oryza sativa (japonica cultivar-group)] >gi|51963660|ref|XP_506581.1| PREDICTED OJ1200_C08.123 gene product [Oryza sativa (japonica cultivar-group)] >gi|50509152|dbj|BAD30292.1| unknown protein [Oryza sa	11673.m04844|LOC_Os07g48770|protein expressed protein e_value:3.58852e-120	0.172	(0.1106 0.6445)	0.1106	0.6445
1383	SET4_L14_CLUSTER_12315	gi|55770331|ref|XP_550156.1| putative tyrosine phosphatase [Oryza sativa (japonica cultivar-group)] >gi|54290212|dbj|BAD61100.1| putative tyrosine phosphatase [Oryza sativa (japonica cultivar-group)] e_value:3.7428e-88	11670.m01858|LOC_Os04g20280|protein Protein tyrosine phosphatase-like protein, PTPLA, putative e_value:2.11362e-89	0.337	(0.1963 0.5817)	0.1963	0.5817
1384	SET4_O24_CLUSTER_33657	gi|51091689|dbj|BAD36472.1| putative phosphatidic acid phosphatase beta [Oryza sativa (japonica cultivar-group)] >gi|51091484|dbj|BAD36223.1| putative phosphatidic acid phosphatase beta [Oryza sativa (japonica cultivar-group)] e_value:2.09625e-	11674.m02646|LOC_Os08g27040|protein PAP2 superfamily, putative e_value:1.79972e-142	0.12	(0.1628 1.3509)	0.1628	1.3509
1385	SET3_B15_CLUSTER_14131	gi|50911236|ref|XP_467036.1| putative glutaredoxin I [Oryza sativa (japonica cultivar-group)] >gi|49388384|dbj|BAD25520.1| putative glutaredoxin I [Oryza sativa (japonica cultivar-group)] >gi|49387625|dbj|BAD25821.1| putative glutaredoxin I [Oryza s	11668.m04136|LOC_Os02g43180|protein Glutaredoxin, putative e_value:2.48642e-50	0.264	(0.1158 0.4385)	0.1158	0.4385
1386	WT009_J05_CLUSTER_4096	gi|57899508|dbj|BAD86970.1| putative ankyrin-kinase [Oryza sativa (japonica cultivar-group)] e_value:0	11667.m05366|LOC_Os01g54480|protein At4g18950/F13C5_120 e_value:0	0.18	(0.0855 0.4746)	0.0855	0.4746
1387	WT009_D22_CLUSTER_32478	gi|2894378|emb|CAA74910.1| putative ribophorin I homologue [Hordeum vulgare subsp. vulgare] >gnl|BL_ORD_ID|1717241 gi|7489438|pir||T04482 ribophorin I homolog - barley (fragment) e_value:0	11682.m02168|LOC_Os05g23600|protein ribophorin I homolog - barley (fragment) e_value:0	0.151	(0.0786 0.5218)	0.0786	0.5218
1388	WT006_B19_CLUSTER_31803	gi|38344237|emb|CAD41330.2| OJ991113_30.14 [Oryza sativa (japonica cultivar-group)] >gnl|BL_ORD_ID|1521110 gi|50925396|ref|XP_472963.1| OJ991113_30.14 [Oryza sativa (japonica cultivar-group)] e_value:0	11670.m03943|LOC_Os04g41100|protein Protein kinase domain, putative e_value:0	0.144	(0.0956 0.6630)	0.0956	0.663
1389	WT003_P14_CLUSTER_12033	gi|17149110|gb|AAL35901.1| clathrin assembly protein AP17-like protein [Oryza sativa] e_value:7.48483e-72	11686.m01020|LOC_Os12g10560|protein clathrin assembly protein AP17-like protein e_value:8.58969e-74	0.073	(0.0386 0.5299)	0.0386	0.5299
1390	WT007_O04_CLUSTER_31884	gi|56783934|dbj|BAD81371.1| ubiquitin-conjugating enzyme -like [Oryza sativa (japonica cultivar-group)] e_value:8.04104e-174	11667.m01295|LOC_Os01g13170|protein Ubiquitin-conjugating enzyme, putative e_value:2.41948e-175	0.275	(0.1998 0.7275)	0.1998	0.7275
1391	WT006_K10_CLUSTER_32956	gi|31711970|gb|AAP68341.1| At2g26510 [Arabidopsis thaliana] >gnl|BL_ORD_ID|2049921 gi|2739376|gb|AAC14499.1| putative membrane transporter [Arabidopsis thaliana] >gnl|BL_ORD_ID|2049921 gi|13877635|gb|AAK43895.1| putative membrane transporter [Arabid	11686.m03902|LOC_Os12g39420|protein probable membrane transporter At2g26510 [imported] - Arabidopsis thaliana	0.175	(0.0859 0.4917)	0.0859	0.4917
1392	SET5_A16_CLUSTER_15709	gi|36939187|gb|AAQ86958.1| ETTIN-like auxin response factor [Triticum aestivum] e_value:0	11682.m04685|LOC_Os05g48870|protein Arabidopsis ETTIN-like protein 1 e_value:0	0.228	(0.1265 0.5540)	0.1265	0.554
1393	SET4_F21_CLUSTER_15709	gi|36939187|gb|AAQ86958.1| ETTIN-like auxin response factor [Triticum aestivum] e_value:0	11682.m04685|LOC_Os05g48870|protein Arabidopsis ETTIN-like protein 1 e_value:0	0.227	(0.1275 0.5608)	0.1275	0.5608
1394	WT006_I23_CLUSTER_16204	gi|34900034|ref|NP_911363.1| putative zinc finger POZ domain protein [Oryza sativa (japonica cultivar-group)] >gnl|BL_ORD_ID|1521348 gi|22202734|dbj|BAC07391.1| putative zinc finger POZ domain protein [Oryza sativa (japonica cultivar-group)] e_value:0	11669.m05854|LOC_Os03g57860|protein expressed protein e_value:7.68295e-172	0.205	(0.1061 0.5190)	0.1061	0.519
1395	WT010_E18_CLUSTER_11071	gi|54291575|dbj|BAD62499.1| putative lysine ketoglutarate reductase trans-splicing related 1 [Oryza sativa (japonica cultivar-group)] e_value:1.52256e-175	11680.m05150|LOC_Os06g51520|protein expressed protein e_value:3.5606e-177	0.126	(0.0935 0.7426)	0.0935	0.7426
1396	WT012_G23_CLUSTER_16763	gi|38346458|emb|CAE02114.2| OSJNBa0019G23.6 [Oryza sativa (japonica cultivar-group)] >gnl|BL_ORD_ID|1523468 gi|50930107|ref|XP_474581.1| OSJNBa0019G23.6 [Oryza sativa (japonica cultivar-group)] e_value:6.30268e-140	11670.m00742|LOC_Os04g08320|protein Hrf1 family e_value:1.44821e-141	0.068	(0.0488 0.7123)	0.0488	0.7123
1397	WT006_D14_CLUSTER_34728	No hits found	11687.m03198|LOC_Os11g34950|protein AT5g43680/MQO24_4 e_value:1.06107e-66	0.4	(0.2422 0.6060)	0.2422	0.606
1398	WT003_A22_CLUSTER_21271	gi|34911544|ref|NP_917119.1| putative clathrin-associated protein [Oryza sativa (japonica cultivar-group)] e_value:0	11667.m04956|LOC_Os01g50770|protein Adaptor complexes medium subunit family e_value:0	0.039	(0.0289 0.7461)	0.0289	0.7461
1399	SET6_K16_CLUSTER_32232	gi|28611030|emb|CAD62368.1| chimeric SDH2-RPS14 protein [Triticum aestivum] e_value:0	11674.m00173|LOC_Os08g02640|protein ribosomal protein S14p/S29e, putative e_value:0	0.18	(0.0492 0.2732)	0.0492	0.2732
1400	WT008_B14_CLUSTER_33654	gi|57899854|dbj|BAD87638.1| putative dynamin like protein 2a [Oryza sativa (japonica cultivar-group)] >gnl|BL_ORD_ID|972197 gi|57899424|dbj|BAD88362.1| putative dynamin like protein 2a [Oryza sativa (japonica cultivar-group)] e_value:0	11667.m06970|LOC_Os01g69130|protein dynamin like protein 2a e_value:0	0.132	(0.0765 0.5798)	0.0765	0.5798
1401	SET1_E02_CLUSTER_14186	gi|51979520|ref|XP_507554.1| PREDICTED OJ1124_D06.11-2 gene product [Oryza sativa (japonica cultivar-group)] >gi|50916122|ref|XP_468525.1| metallo-beta-lactamase-like [Oryza sativa (japonica cultivar-group)] >gi|51979516|ref|XP_507553.1| PREDICTED O	11668.m05777|LOC_Os02g58260|protein expressed protein e_value:5.70009e-149	0.151	(0.1414 0.9357)	0.1414	0.9357
1402	WT004_I07_CLUSTER_31715	gi|50905349|ref|XP_464163.1| MAP kinase MAPK2 [Oryza sativa (japonica cultivar-group)] >gnl|BL_ORD_ID|1566010 gi|11869997|gb|AAG40581.1| MAP kinase 3 [Oryza sativa] >gnl|BL_ORD_ID|1566010 gi|45736030|dbj|BAD13057.1| MAP kinase MAPK2 [Oryza sativa (j	11668.m00487|LOC_Os02g05480|protein Protein kinase domain, putative e_value:0	0.035	(0.0199 0.5733)	0.0199	0.5733
1403	WT004_I19_CLUSTER_13401	gi|21592984|gb|AAM64933.1| photolyase/blue-light receptor PHR2 [Arabidopsis thaliana] e_value:1.75524e-118	11669.m02293|LOC_Os03g22330|protein photolyase/blue-light receptor PHR2 e_value:0	0.139	(0.0605 0.4360)	0.0605	0.436
1404	WT007_E18_CLUSTER_33714	gi|51490665|emb|CAG26903.1| ALY protein [Nicotiana benthamiana] e_value:5.78244e-64	11669.m01715|LOC_Os03g17010|protein transcriptional coactivator-like protein e_value:5.6827e-111	0.238	(0.1100 0.4615)	0.11	0.4615
1405	WT005_K11_CLUSTER_33387	gi|51039064|gb|AAT94297.1| endotransglucosylase/hydrolase XTH5 [Triticum aestivum] e_value:7.12806e-166	11680.m04765|LOC_Os06g48160|protein xyloglucan endo-1,4-beta-D-glucanase (EC 3.2.1.-) - barley e_value:4.51732e-152	0.262	(0.0773 0.2947)	0.0773	0.2947
1406	WT004_J06_CLUSTER_31155	gi|20257409|gb|AAM15877.1| thaumatin-like protein [Triticum aestivum] e_value:6.13499e-131	11686.m04345|LOC_Os12g43490|protein thaumatin-like protein TLP7 e_value:6.6635e-99	0.527	(0.1954 0.3707)	0.1954	0.3707
1407	SET4_L12_CLUSTER_5106	gi|20259633|gb|AAM14173.1| putative GAR1 protein [Arabidopsis thaliana] >gi|17380836|gb|AAL36230.1| putative GAR1 protein [Arabidopsis thaliana] >gi|18396707|ref|NP_566217.1| Gar1 RNA-binding region family protein [Arabidopsis thaliana] e_value:6.85	11687.m03417|LOC_Os11g37080|protein Similar to gene_id:MRG7.14 e_value:1.42993e-90	0.123	(0.0773 0.6285)	0.0773	0.6285
1408	WT010_A10_CLUSTER_34255	gi|38347199|emb|CAD40524.2| OSJNBa0023J03.11 [Oryza sativa (japonica cultivar-group)] >gnl|BL_ORD_ID|1536041 gi|32483270|emb|CAE02397.1| OSJNBa0024J22.1 [Oryza sativa (japonica cultivar-group)] >gnl|BL_ORD_ID|1536041 gi|50922747|ref|XP_471734.1| OSJ	11670.m02257|LOC_Os04g24180|protein Sec63 domain, putative e_value:0	0.083	(0.0439 0.5287)	0.0439	0.5287

PDF Complete
Your complimentary use period has ended. Thank you for using PDF Complete.
Click Here to upgrade to Unlimited Pages and Expanded Features

1409	WT004_I14_CLUSTER_7771	gi|15218866|ref|NP_176175.1| nucleoporin family protein [Arabidopsis thaliana] e_value:0	11686.m00632|LOC_Os12g06870|protein protein F14N23.29 [imported] – Arabidopsis thaliana e_value:0	0.331	(0.2143 0.6480)	0.2143	0.648
1410	SET1_H01_CLUSTER_5486	gi|50931507|ref|XP_475281.1| unknown protein [Oryza sativa (japonica cultivar-group)] >gi|49328049|gb|AAT58750.1| unknown protein [Oryza sativa (japonica cultivar-group)] >gi|48843788|gb|AAT47047.1| unknown protein [Oryza sativa (japonica cultivar-g	11682.m03380|LOC_Os05g35710|protein hypothetical protein e_value:0	0.241	(0.0903 0.3745)	0.0903	0.3745
1411	SET1_C14_CLUSTER_33875	gi|50509216|dbj|BAD30468.1| putative COP9 complex subunit, FUS5 [Oryza sativa (japonica cultivar-group)] e_value:2.01219e-111	11673.m02936|LOC_Os07g30840|protein COP9 complex chain FUS5 [imported] – Arabidopsis thaliana e_value:3.11446e-101	0.274	(0.1507 0.5492)	0.1507	0.5492
1412	SET3_F07_CLUSTER_32974	gi|34914298|ref|NP_918496.1| putative FtsH protease [Oryza sativa (japonica cultivar-group)] >gi|20521392|dbj|BAB91903.1| cell division protein ftsH (ftsH)-like [Oryza sativa (japonica cultivar-group)] e_value:0	11667.m03763|LOC_Os01g39260|protein ATP-dependent metalloprotease FtsH, putative e_value:0	0.089	(0.1102 1.2406)	0.1102	1.2406
1413	WT004_F13_CLUSTER_4197	gi|61162194|dbj|BAD91079.1| beta-D-galactosidase [Pyrus pyrifolia] e_value:0	11669.m01491|LOC_Os03g15020|protein beta-galactosidase e_value:0		(0.4377 -1.0000)	0.4377	-1
1414	WT007_G05_CLUSTER_12039	gi|50901626|ref|XP_463246.1| putative LIM domain containing protein [Oryza sativa (japonica cultivar-group)] e_value:0	11669.m04252|LOC_Os03g42820|protein putative LIM domain containing protein e_value:0	0.273	(0.1514 0.5545)	0.1514	0.5545
1415	WT006_M04_CLUSTER_7415	gi|2909424|emb|CAA12028.1| Glyoxalase I [Cicer arietinum] >gnl|BL_ORD_ID|1317147 gi|3334245|sp|O49818|LGUL_CICAR Lactoylglutathione lyase (Methylglyoxalase) (Aldoketomutase) (Glyoxalase I) (Glx I) (Ketone-aldehyde mutase) (S-D-lactoylglutathione	11682.m02103|LOC_Os05g22970|protein lactoylglutathione lyase e_value:6.94227e-98	0.091	(0.0499 0.5495)	0.0499	0.5495
1416	WT002_M08_CLUSTER_1562	gi|31415899|gb|AAP50920.1| putative 3-beta hydroxysteroid dehydrogenase/isomerase [Oryza sativa (japonica cultivar-group)] >gnl|BL_ORD_ID|2005404 gi|50921121|ref|XP_470921.1| putative 3-beta hydroxysteroid dehydrogenase/isomerase [Oryza sativa (japonic	11669.m02932|LOC_Os03g29170|protein putative 3-beta hydroxysteroid dehydrogenase/isomerase e_value:0	0.145	(0.0775 0.5346)	0.0775	0.5346
1417	WT007_P03_CLUSTER_16551	gi|42570185|ref|NP_849578.2| glycosyl hydrolase family 1 protein [Arabidopsis thaliana] e_value:5.22487e-147	11681.m03052|LOC_Os09g33710|protein F22D16.15 protein – Arabidopsis thaliana e_value:0	0.299	(0.1935 0.6478)	0.1935	0.6478
1418	SET5_J16_CLUSTER_14093	gi|50929405|ref|XP_474230.1| OSJNBa0084K01.14 [Oryza sativa (japonica cultivar-group)] >gi|38346074|emb|CAE04842.2| OSJNBa0084K01.14 [Oryza sativa (japonica cultivar-group)] e_value:0	11670.m05598|LOC_Os04g56740|protein IQ calmodulin-binding motif, putative e_value:0	0.329	(0.1718 0.5217)	0.1718	0.5217
1419	WT011_C18_CLUSTER_33138	gi|6979343|gb|AAF34436.1| similar to mitogen-activated protein kinases [Oryza sativa] e_value:0	11687.m00955|LOC_Os11g10100|protein similar to mitogen-activated protein kinases e_value:0	0.256	(0.1268 0.4950)	0.1268	0.495
1420	WT013_B20_CLUSTER_30655	gi|34910296|ref|NP_916495.1| putative UDP-glucose: flavonoid 7-O-glucosyltransferase [Oryza sativa (japonica cultivar-group)] >gnl|BL_ORD_ID|1751373 gi|11034537|dbj|BAB17061.1| putative UDP-glucose: flavonoid 7-O-glucosyltransferase [Oryza sativa (japo	11667.m00757|LOC_Os01g08110|protein UDP-glucoronosyl and UDP-glucosyl transferase e_value:0	0.231	(0.1455 0.6303)	0.1455	0.6303
1421	WT005_J10_CLUSTER_5798	gi|6685070|gb|AAF23771.1| FtsZ protein [Gentiana lutea] >gnl|BL_ORD_ID|743500 gi|11277908|pir||T51088 plastid division protein ftsZ [imported] – Gentiana lutea e_value:0	11669.m04422|LOC_Os03g44420|protein cell division protein FtsZ, putative e_value:0	0.151	(0.0886 0.5868)	0.0886	0.5868
1422	WT009_P01_CLUSTER_28318	gi|54290844|dbj|BAD61505.1| lysophospholipase 2-like [Oryza sativa (japonica cultivar-group)] >gnl|BL_ORD_ID|265465 gi|54290270|dbj|BAD61215.1| lysophospholipase 2-like [Oryza sativa (japonica cultivar-group)] e_value:5.47968e-109	11667.m04123|LOC_Os01g42690|protein Phospholipase/Carboxylesterase, putative e_value:4.59187e-123	0.117	(0.1055 0.8999)	0.1055	0.8999
1423	WT008_D16_CLUSTER_2737	gi|50919225|ref|XP_470009.1| serine/threonine protein phosphatase PP2A-2 catalytic subunit [Oryza sativa (japonica cultivar-group)] >gnl|BL_ORD_ID|1587485 gi|4530611|gb|AAD22116.1| serine/threonine protein phosphatase PP2A-2 catalytic subunit [Oryza sa	11669.m05994|LOC_Os03g59060|protein serine/threonine protein phosphatase PP2A-2 catalytic subunit	0.029	(0.0367 1.2549)	0.0367	1.2549
1424	SET1_N22_CLUSTER_4929	gi|34900358|ref|NP_911525.1| hypothetical protein [Oryza sativa (japonica cultivar-group)] >gi|22093623|dbj|BAC06919.1| hypothetical protein [Oryza sativa (japonica cultivar-group)] e_value:8.98088e-104	11673.m01174|LOC_Os07g12220|protein hypothetical protein e_value:3.6048e-107	0.158	(0.0890 0.5637)	0.089	0.5637
1425	SET6_C19_CLUSTER_31204	gi|48374963|gb|AAT42161.1| putative cis-zeatin O-glucosyltransferase [Sorghum bicolor] e_value:7.65522e-177	11670.m04666|LOC_Os04g47720|protein UDP-glucoronosyl and UDP-glucosyl transferase e_value:1.76297e-176	0.552	(0.2236 0.4052)	0.2236	0.4052
1426	WT009_O19_CLUSTER_32653	gi|50900448|ref|XP_462657.1| OSJNBa0064H22.9 [Oryza sativa (japonica cultivar-group)] >gnl|BL_ORD_ID|1564342 gi|38344828|emb|CAE01568.2| OSJNBa0064H22.9 [Oryza sativa (japonica cultivar-group)] e_value:0	11670.m03541|LOC_Os04g37540|protein expressed protein e_value:0	0.412	(0.2119 0.5139)	0.2119	0.5139
1427	SET3_B21_CLUSTER_33943	gi|50253039|dbj|BAD29288.1| putative beta-alanine synthase [Oryza sativa (japonica cultivar-group)] >gi|50251406|dbj|BAD28433.1| putative beta-alanine synthase [Oryza sativa (japonica cultivar-group)] >gi|14289301|dbj|BAB59126.1| putative hydrolase	11668.m03118|LOC_Os02g33080|protein hydrolase, carbon-nitrogen family, putative e_value:1.54436e-158	0.08	(0.0513 0.6443)	0.0513	0.6443
1428	WT002_I06_CLUSTER_21343	gi|50936809|ref|XP_477932.1| water-stress protein-like protein [Oryza sativa (japonica cultivar-group)] >gnl|BL_ORD_ID|1526399 gi|34395354|dbj|BAC84424.1| water-stress protein-like protein [Oryza sativa (japonica cultivar-group)] e_value:3.79426e-156	11673.m02546|LOC_Os07g27110|protein RNA recognition motif. (a.k.a. RRM, RBD, or RNP domain), putative e_value:1.04405e-	0.4	(0.2588 0.6470)	0.2588	0.647
1429	WT012_P24_CLUSTER_19240	gi|32488444|emb|CAE03377.1| OSJNBa0004N05.1 [Oryza sativa (japonica cultivar-group)] >gnl|BL_ORD_ID|1522105 gi|32488304|emb|CAE03370.1| OSJNBb0065L13.13 [Oryza sativa (japonica cultivar-group)] >gnl|BL_ORD_ID|1522105 gi|50926380|ref|XP_473137.1| OSJ	11670.m04167|LOC_Os04g43150|protein Nuclear transport factor 2 (NTF2) domain, putative e_value:1.11534e-148	0.372	(0.2082 0.5598)	0.2082	0.5598
1430	WT005_A01_CLUSTER_14793	gi|51518075|ref|YP_068003.1| LF3 [Cercopithecine herpesvirus 15] >gnl|BL_ORD_ID|1259572 gi|18025526|gb|AAK95470.1| LF3 [cercopithicine herpesvirus 15] e_value:6.34931e-28	11676.m03710|LOC_Os10g40500|protein transposon protein, putative, unclassified e_value:1.33282e-06	0.589	(1.0391 1.7631)	1.0391	1.7631
1431	WT009_A03_CLUSTER_32124	gi|38567900|emb|CAE03655.2| OSJNBa0060N03.20 [Oryza sativa (japonica cultivar-group)] >gnl|BL_ORD_ID|1524375 gi|50928619|ref|XP_473837.1| OSJNBa0060N03.20 [Oryza sativa (japonica cultivar-group)] e_value:0	11670.m05083|LOC_Os04g51970|protein At3g13050/MGH6_16 e_value:0	0.151	(0.1010 0.6698)	0.101	0.6698
1432	WT005_N17_CLUSTER_3513	gi|31432079|gb|AAP53764.1| putative beta-ketoacyl-CoA synthase [Oryza sativa (japonica cultivar-group)] >gnl|BL_ORD_ID|2011209 gi|37534350|ref|NP_921477.1| putative beta-ketoacyl-CoA synthase [Oryza sativa (japonica cultivar-group)] e_value:0	11676.m02380|LOC_Os10g28060|protein Chalcone and stilbene synthases, C-terminal domain, putative e_value:0	0.206	(0.0780 0.3794)	0.078	0.3794
1433	WT005_C19_CLUSTER_32768	gi|50932229|ref|XP_475642.1| putative Arginyl-tRNA synthetase [Oryza sativa (japonica cultivar-group)] >gnl|BL_ORD_ID|1597163 gi|46981337|gb|AAT07655.1| putative Arginyl-tRNA synthetase [Oryza sativa (japonica cultivar-group)] e_value:0	11682.m00657|LOC_Os05g07030|protein arginyl-tRNA synthetase e_value:0	0.108	(0.0626 0.5805)	0.0626	0.5805
1434	WT009_G14_CLUSTER_16243	gi|18033228|gb|AAL57037.1| UDP-glucosyltransferase BX8 [Zea mays] e_value:1.3421e-172	11687.m02305|LOC_Os11g25730|protein UDP-glucosyltransferase BX8 e_value:1.4433e-135	0.313	(0.3620 1.1568)	0.362	1.1568
1435	WT005_O12_CLUSTER_14823	gi|4589961|gb|AAD26479.1| unknown protein [Arabidopsis thaliana] >gnl|BL_ORD_ID|940950 gi|25408152|pir||C84720 hypothetical protein At2g31400 [imported] – Arabidopsis thaliana >gnl|BL_ORD_ID|940950 gi|15224671|ref|NP_180698.1| pentatricopeptide (PPR	11686.m03693|LOC_Os12g37550|protein expressed protein e_value:0	0.153	(0.0996 0.6526)	0.0996	0.6526
1436	SET5_I06_CLUSTER_15814	gi|19387264|gb|AAL87175.1| unknown [Oryza sativa (japonica cultivar-group)] >gi|50929323|ref|XP_474189.1| OSJNBa0011F23.5 [Oryza sativa (japonica cultivar-group)] >gi|38346399|emb|CAE04232.2| OSJNBa0011F23.5 [Oryza sativa (japonica cultivar-group)]	11670.m05551|LOC_Os04g56300|protein hypothetical protein e_value:8.65253e-167	0.271	(0.1755 0.6477)	0.1755	0.6477
1437	WT012_N03_CLUSTER_6667	gi|62148976|dbj|BAD93371.1| P450 [Triticum aestivum] e_value:0	11674.m00050|LOC_Os08g01450|protein Cytochrome P450 e_value:1.60321e-133	0.204	(0.3967 1.9419)	0.3967	1.9419
1438	WT002_O09_CLUSTER_20056	gi|62733407|gb|AAX95524.1| Expressed protein [Oryza sativa (japonica cultivar-group)] e_value:1.21422e-103	11669.m04974|LOC_Os03g49490|protein hypothetical protein e_value:2.73168e-105	0.33	(0.1525 0.4620)	0.1525	0.462
1439	SET4_B10_CLUSTER_29582	gi|15667361|emb|CAB71340.2| putative proteinase inhibitor [Hordeum vulgare subsp. vulgare] e_value:2.44539e-30	11686.m03550|LOC_Os12g36210|protein subtilisin/chymotrypsin inhibitor – maize e_value:3.28436e-22	0.343	(0.2415 0.7049)	0.2415	0.7049
1440	SET4_I20_CLUSTER_34167	gi|50511360|gb|AAT77283.1| hypothetical protein [Oryza sativa (japonica cultivar-group)] e_value:1.18769e-143	11682.m03916|LOC_Os05g40980|protein Zinc finger, C3HC4 type (RING finger), putative e_value:2.94049e-145	0.24	(0.1472 0.6126)	0.1472	0.6126
1441	WT009_H04_CLUSTER_14052	gi|50919833|ref|XP_470277.1| putative integral membrane protein [Oryza sativa (japonica cultivar-group)] >gnl|BL_ORD_ID|1539351 gi|19071633|gb|AAL84300.1| putative integral membrane protein [Oryza sativa (japonica cultivar-group)] e_value:0	11669.m00675|LOC_Os03g07130|protein putative integral membrane protein e_value:0	0.133	(0.1060 0.7984)	0.106	0.7984
1442	WT009_G03_CLUSTER_14052	gi|50919833|ref|XP_470277.1| putative integral membrane protein [Oryza sativa (japonica cultivar-group)] >gnl|BL_ORD_ID|1539351 gi|19071633|gb|AAL84300.1| putative integral membrane protein [Oryza sativa (japonica cultivar-group)] e_value:0	11669.m00675|LOC_Os03g07130|protein putative integral membrane protein e_value:0	0.133	(0.1060 0.7984)	0.106	0.7984
1443	WT009_P18_CLUSTER_4246	gi|50940231|ref|XP_479643.1| putative KH domain protein [Oryza sativa (japonica cultivar-group)] >gnl|BL_ORD_ID|1560160 gi|38637286|dbj|BAD03549.1| putative KH domain protein [Oryza sativa (japonica cultivar-group)] e_value:0	11674.m00007|LOC_Os08g01070|protein expressed protein e_value:0	0.443	(0.2876 0.6495)	0.2876	0.6495
1444	WT012_K20_CLUSTER_26514	gi|38344450|emb|CAE05656.2| OSJNBa0038O10.22 [Oryza sativa (japonica cultivar-group)] >gnl|BL_ORD_ID|1534551 gi|50926606|ref|XP_473250.1| OSJNBa0038O10.22 [Oryza sativa (japonica cultivar-group)] e_value:7.51971e-145	11670.m04318|LOC_Os04g44560|protein expressed protein e_value:1.91922e-146	0.2	(0.0805 0.4015)	0.0805	0.4015
1445	WT008_P06_CLUSTER_3182	gi|7267401|emb|CAB80871.1| putative tetrahydrofolate synthase [Arabidopsis thaliana] >gnl|BL_ORD_ID|1456032 gi|23197716|gb|AAN15385.1| Unknown protein [Arabidopsis thaliana] >gnl|BL_ORD_ID|1456032 gi|16649149|gb|AAL24426.1| Unknown protein [Arabidop	11668.m00201|LOC_Os02g02850|protein probable methylenetetrahydrofolate dehydrogenase (NADP) (EC 1.5.1.5) F6N23.26 – Arabidopsis thaliana e_value:1.92976e-180	0.185	(0.0775 0.4188)	0.0775	0.4188
1446	SET6_M07_CLUSTER_13525	gi|22136914|gb|AAM91801.1| unknown protein [Arabidopsis thaliana] >gi|13877883|gb|AAK44019.1| unknown protein [Arabidopsis thaliana] >gi|15912313|gb|AAL08290.1| AT5g24690/MXC17_8 [Arabidopsis thaliana] >gi|22327037|ref|NP_197857.2| expressed prot	11686.m01847|LOC_Os12g18630|protein hypothetical protein e_value:3.21247e-168	0.322	(0.2410 0.7487)	0.241	0.7487
1447	WT011_J05_CLUSTER_22451	gi|51979370|ref|XP_507533.1| PREDICTED OJ1175_B01.8-1 gene product [Oryza sativa (japonica cultivar-group)] >gnl|BL_ORD_ID|1583017 gi|51964402|ref|XP_506986.1| PREDICTED OJ1175_B01.8-1 gene product [Oryza sativa (japonica cultivar-	11668.m05108|LOC_Os02g52230|protein F-box domain, putative e_value:0	0.17	(0.1349 0.7916)	0.1349	0.7916
1448	WT011_B06_CLUSTER_9729	gi|7267604|emb|CAB80916.1| putative flavonol glucosyltransferase [Arabidopsis thaliana] >gnl|BL_ORD_ID|1456075 gi|14532902|gb|AAK64133.1| putative flavonol glucosyltransferase [Arabidopsis thaliana] >gnl|BL_ORD_ID|1456075 gi|13430700|gb|AAK25972.1|	11687.m03580|LOC_Os11g38650|protein UDP-glucoronosyl and UDP-glucosyl transferase e_value:0	0.299	(0.1497 0.5005)	0.1497	0.5005
1449	WT010_M10_CLUSTER_4349	gi|50929247|ref|XP_474151.1| OSJNBa0060D06.10 [Oryza sativa (japonica cultivar-group)] >gnl|BL_ORD_ID|1593257 gi|38345900|emb|CAE03544.2| OSJNBa0060D06.10 [Oryza sativa (japonica cultivar-group)] e_value:2.19731e-116	11670.m05502|LOC_Os04g55850|protein S1/P1 Nuclease e_value:5.69468e-118	0.273	(0.1841 0.6753)	0.1841	0.6753
1450	WT009_N06_CLUSTER_22493	gi|50934563|ref|XP_476809.1| protease inhibitor-like protein [Oryza sativa (japonica cultivar-group)] >gnl|BL_ORD_ID|1521755 gi|51963294|ref|XP_506191.1| PREDICTED OJ1656_E11.137 gene product [Oryza sativa (japonica cultivar-group)] >gnl|BL_ORD_ID|1	11673.m00723|LOC_Os07g07930|protein Protease inhibitor/seed storage/LTP family, putative e_value:2.12088e-52	0.63	(0.2452 0.3889)	0.2452	0.3889
1451	WT008_E08_CLUSTER_6729	gi|55741398|ref|XP_550364.1| putative RING finger 1 [Oryza sativa (japonica cultivar-group)] >gnl|BL_ORD_ID|1743553 gi|55296046|dbj|BAD67608.1| putative RING finger 1 [Oryza sativa (japonica cultivar-group)] >gnl|BL_ORD_ID|1743553 gi|55296217|dbj|BA	11680.m00657|LOC_Os06g07100|protein Zinc finger, C3HC4 type (RING finger), putative e_value:9.99715e-121	0.509	(0.2090 0.4108)	0.209	0.4108

PDF Complete Your complimentary use period has ended. Thank you for using PDF Complete.
Click Here to upgrade to Unlimited Pages and Expanded Features

<table>
<tr><th></th><th></th><th></th><th></th><th></th><th></th><th></th><th></th></tr>
<tr><td>1452</td><td>WT008_E04_CLUSTER_28685</td><td>gi|32488074|emb|CAE03027.1| OSJNBa0084A10.2 [Oryza sativa (japonica cultivar-group)] >gnl|BL_ORD_ID|1521457 gi|50924362|ref|XP_472541.1| OSJNBa0084A10.2 [Oryza sativa (japonica cultivar-group)] e_value:0</td><td>11670.m03392|LOC_Os04g35140|protein Subtilisin N-terminal Region, putative e_value:0</td><td>0.368</td><td>(0.1540 0.4184)</td><td>0.154</td><td>0.4184</td></tr>
<tr><td>1453</td><td>WT007__C23_CLUSTER_27144</td><td>gi|31433112|gb|AAP54665.1| putative trehalase [Oryza sativa (japonica cultivar-group)] >gnl|BL_ORD_ID|2012696 gi|37536152|ref|NP_922378.1| putative trehalase [Oryza sativa (japonica cultivar-group)] >gnl|BL_ORD_ID|2012696 gi|10122053|gb|AAG13442.1|</td><td>11676.m03403|LOC_Os10g37660|protein putative trehalase e_value:0</td><td>0.248</td><td>(0.1112 0.4484)</td><td>0.1112</td><td>0.4484</td></tr>
<tr><td>1454</td><td>WT004_P10_CLUSTER_19536</td><td>gi|50932641|ref|XP_475848.1| putative peptidyl-tRNA hydrolase (PTH) [Oryza sativa (japonica cultivar-group)] >gnl|BL_ORD_ID|1598899 gi|47900494|gb|AAT39251.1| putative peptidyl-tRNA hydrolase (PTH) [Oryza sativa (japonica cultivar-group)] e_value:9.123</td><td>11682.m04545|LOC_Os05g47630|protein peptidyl-tRNA hydrolase, putative e_value:2.17814e-102</td><td>0.205</td><td>(0.1281 0.6264)</td><td>0.1281</td><td>0.6264</td></tr>
<tr><td>1455</td><td>WT004_G08_CLUSTER_2518</td><td>gi|31430514|gb|AAP52416.1| putative calcium-binding protein [Oryza sativa (japonica cultivar-group)] >gnl|BL_ORD_ID|2013638 gi|37531654|ref|NP_920129.1| putative calcium-binding protein [Oryza sativa (japonica cultivar-group)] >gnl|BL_ORD_ID|2013638</td><td>11676.m00861|LOC_Os10g09850|protein probable calcium-binding protein (clone Y8) - potato e_value:1.72405e-160</td><td>0.188</td><td>(0.1295 0.6900)</td><td>0.1295</td><td>0.69</td></tr>
<tr><td>1456</td><td>WT002_N02_CLUSTER_20469</td><td>gi|54291347|dbj|BAD62113.1| unknown protein [Oryza sativa (japonica cultivar-group)] e_value:1.1309e-33</td><td>11680.m05126|LOC_Os06g51320|protein gibberellin-regulated protein, putative e_value:1.0256e-35</td><td>0.436</td><td>(0.3544 0.8123)</td><td>0.3544</td><td>0.8123</td></tr>
<tr><td>1457</td><td>SET4_D10_CLUSTER_13319</td><td>gi|34905952|ref|NP_914323.1| OJ1656_A11.17 [Oryza sativa (japonica cultivar-group)] >gi|18844859|dbj|BAB85328.1| unknown protein [Oryza sativa (japonica cultivar-group)] e_value:1.06806e-30</td><td>11667.m07412|LOC_Os01g73780|protein expressed protein e_value:8.23948e-33</td><td>0.361</td><td>(0.2224 0.6165)</td><td>0.2224</td><td>0.6165</td></tr>
<tr><td>1458</td><td>SET3_I08_CLUSTER_20821</td><td>gi|50926602|ref|XP_473248.1| OSJNBa0038O10.20 [Oryza sativa (japonica cultivar-group)] >gi|38344448|emb|CAE05654.2| OSJNBa0038O10.20 [Oryza sativa (japonica cultivar-group)] e_value:3.9618e-158</td><td>11670.m04316|LOC_Os04g44540|protein expressed protein e_value:1.43398e-133</td><td>0.217</td><td>(0.0857 0.3942)</td><td>0.0857</td><td>0.3942</td></tr>
<tr><td>1459</td><td>SET3_A23_CLUSTER_13754</td><td>gi|50911775|ref|XP_467295.1| putative secretory carrier membrane protein [Oryza sativa (japonica cultivar-group)] >gi|41052954|dbj|BAD07864.1| putative secretory carrier membrane protein [Oryza sativa (japonica cultivar-group)] e_value:4.21958e-</td><td>11668.m04553|LOC_Os02g47010|protein SCAMP family, putative e_value:1.01262e-137</td><td>0.132</td><td>(0.0749 0.5694)</td><td>0.0749</td><td>0.5694</td></tr>
<tr><td>1460</td><td>SET2_L17_CLUSTER_29904</td><td>gi|66856533|ref|ZP_00400598.1| hypothetical protein AdehDRAFT_1984 [Anaeromyxobacter dehalogenans 2CP-C] >gi|66777777|gb|EAL78869.1| hypothetical protein AdehDRAFT_1984 [Anaeromyxobacter dehalogenans 2CP-C] e_value:2.48702e-15</td><td>11669.m00969|LOC_Os03g10260|protein hypothetical protein e_value:2.7311e-49</td><td>0.93</td><td>(0.3028 0.3258)</td><td>0.3028</td><td>0.3258</td></tr>
<tr><td>1461</td><td>WT013_D17_CLUSTER_6275</td><td>gi|34899824|ref|NP_911258.1| unknown protein [Oryza sativa (japonica cultivar-group)] >gnl|BL_ORD_ID|1529463 gi|51963482|ref|XP_506414.1| PREDICTED OJ1092_A07.123 gene product [Oryza sativa (japonica cultivar-group)] >gnl|BL_ORD_ID|1529463 gi|278179</td><td>11673.m03694|LOC_Os07g38060|protein expressed protein e_value:0</td><td>0.192</td><td>(0.0966 0.5039)</td><td>0.0966</td><td>0.5039</td></tr>
<tr><td>1462</td><td>WT013_A03_CLUSTER_20549</td><td>gi|50907031|ref|XP_465004.1| putative Neurofilament triplet M protein [Oryza sativa (japonica cultivar-group)] >gnl|BL_ORD_ID|1567593 gi|47847930|dbj|BAD21720.1| putative Neurofilament triplet M protein [Oryza sativa (japonica cultivar-group)] e_value:</td><td>11668.m01776|LOC_Os02g18660|protein neurofilament triplet m protein (160 kda neurofilament protein)(neurofilament medium polypeptide) (nf-m) (fragment) e_value:2.13438e-106</td><td>0.488</td><td>(0.4533 0.9291)</td><td>0.4533</td><td>0.9291</td></tr>
<tr><td>1463</td><td>WT012_M17_CLUSTER_22648</td><td>gi|54291559|dbj|BAD62483.1| putative oligopeptidase B [Oryza sativa (japonica cultivar-group)] >gnl|BL_ORD_ID|266134 gi|54291358|dbj|BAD62124.1| putative oligopeptidase B [Oryza sativa (japonica cultivar-group)] e_value:0</td><td>11680.m05136|LOC_Os06g51410|protein prolyl oligopeptidase family, putative e_value:0</td><td>0.115</td><td>(0.0753 0.6547)</td><td>0.0753</td><td>0.6547</td></tr>
<tr><td>1464</td><td>WT012_I11_CLUSTER_7940</td><td>gi|2618696|gb|AAB84343.1| hypothetical protein [Arabidopsis thaliana] >gnl|BL_ORD_ID|938502 gi|7487596|pir||T00817 hypothetical protein At2g41610 [imported] - Arabidopsis thaliana e_value:9.10142e-57</td><td>11676.m01164|LOC_Os10g12750|protein expressed protein e_value:1.38084e-150</td><td>0.186</td><td>(0.0771 0.4140)</td><td>0.0771</td><td>0.414</td></tr>
<tr><td>1465</td><td>WT011_J21_CLUSTER_29441</td><td>gi|4096103|gb|AAD10483.1| p34cdc2 [Triticum aestivum] e_value:1.40832e-167</td><td>11669.m00194|LOC_Os03g02680|protein cell division control protein 2 homolog 1 (ec 2.7.1.-). e_value:5.46845e-161</td><td>0.1</td><td>(0.0395 0.3941)</td><td>0.0395</td><td>0.3941</td></tr>
<tr><td>1466</td><td>WT008_A12_CLUSTER_22094</td><td>gi|55275991|gb|AAV49759.1| non-specific lipid transfer protein 6 [Hordeum vulgare subsp. vulgare] e_value:3.79518e-55</td><td>11686.m00138|LOC_Os12g02290|protein nonspecific lipid-transfer protein 5 precursor (ltp 5). e_value:7.54607e-39</td><td>0.485</td><td>(0.1939 0.3996)</td><td>0.1939</td><td>0.3996</td></tr>
<tr><td>1467</td><td>WT002_E16_CLUSTER_33079</td><td>gi|33340129|gb|AAQ14552.1| sucrose-phosphate synthase [Triticum aestivum] e_value:0</td><td>11687.m01231|LOC_Os11g12810|protein glycosyl transferase, group 1 family protein, putative e_value:0</td><td>0.35</td><td>(0.1218 0.3476)</td><td>0.1218</td><td>0.3476</td></tr>
<tr><td>1468</td><td>WT006_P13_CLUSTER_31535</td><td>gi|30681617|ref|NP_187724.2| clathrin heavy chain, putative [Arabidopsis thaliana] e_value:0</td><td>11687.m00038|LOC_Os11g01380|protein clathrin heavy chain, putative e_value:0</td><td>0.031</td><td>(0.0163 0.5216)</td><td>0.0163</td><td>0.5216</td></tr>
<tr><td>1469</td><td>WT013_N07_CLUSTER_22992</td><td>gi|3132476|gb|AAC16265.1| unknown protein [Arabidopsis thaliana] >gnl|BL_ORD_ID|938612 gi|45752746|gb|AAS76271.1| At2g34690 [Arabidopsis thaliana] >gnl|BL_ORD_ID|938612 gi|15226804|ref|NP_181016.1| expressed protein [Arabidopsis thaliana] >gnl|BL</td><td>11669.m05774|LOC_Os03g57140|protein expressed protein e_value:1.99791e-99</td><td>0.176</td><td>(0.0769 0.4381)</td><td>0.0769</td><td>0.4381</td></tr>
<tr><td>1470</td><td>WT008_A06_CLUSTER_2887</td><td>gi|50947155|ref|XP_483105.1| putative ribonucleoprotein [Oryza sativa (japonica cultivar-group)] >gnl|BL_ORD_ID|1609349 gi|51964986|ref|XP_507277.1| PREDICTED P0686H11.12 gene product [Oryza sativa (japonica cultivar-group)] >gnl|BL_ORD_ID|1609349 g</td><td>11674.m03830|LOC_Os08g38410|protein RNA recognition motif. (a.k.a. RRM, RBD, or RNP domain), putative e_value:2.75548e-</td><td>0.313</td><td>(0.1297 0.4142)</td><td>0.1297</td><td>0.4142</td></tr>
<tr><td>1471</td><td>SET4_N13_CLUSTER_11155</td><td>gi|50940053|ref|XP_479554.1| unknown protein [Oryza sativa (japonica cultivar-group)] >gi|33146668|dbj|BAC80014.1| unknown protein [Oryza sativa (japonica cultivar-group)] e_value:0</td><td>11673.m04795|LOC_Os07g48350|protein expressed protein e_value:0</td><td>0.179</td><td>(0.0989 0.5531)</td><td>0.0989</td><td>0.5531</td></tr>
<tr><td>1472</td><td>SET2_K21_CLUSTER_19855</td><td>gi|42572335|ref|NP_974263.1| mRNA capping enzyme family protein [Arabidopsis thaliana] e_value:0</td><td>11687.m01057|LOC_Os11g11070|protein mRNA capping enzyme - like protein e_value:0</td><td>0.13</td><td>(0.0685 0.5293)</td><td>0.0685</td><td>0.5293</td></tr>
<tr><td>1473</td><td>WT004__C21_CLUSTER_23162</td><td>gi|57900284|dbj|BAD87117.1| putative Ser/Arg-related nuclear matrix protein [Oryza sativa (japonica cultivar-group)] e_value:0</td><td>11667.m07315|LOC_Os01g72890|protein transposon protein, putative, CACTA, En/Spm sub-class e_value:0</td><td>0.183</td><td>(0.1169 0.6381)</td><td>0.1169</td><td>0.6381</td></tr>
<tr><td>1474</td><td>WT006_P18_CLUSTER_10103</td><td>gi|31126767|gb|AAP44687.1| unknown protein [Oryza sativa (japonica cultivar-group)] >gnl|BL_ORD_ID|1858634 gi|34897230|ref|NP_909961.1| unknown protein [Oryza sativa (japonica cultivar-group)] e_value:1.1519e-160</td><td>11669.m05902|LOC_Os03g58230|protein hypothetical protein e_value:2.45043e-162</td><td>0.307</td><td>(0.1210 0.3942)</td><td>0.121</td><td>0.3942</td></tr>
<tr><td>1475</td><td>WT007_J10_CLUSTER_31750</td><td>gi|38346580|emb|CAE04227.2| OSJNBa0064D20.11 [Oryza sativa (japonica cultivar-group)] e_value:0</td><td>11668.m03331|LOC_Os02g35140|protein Auxin response factor, putative e_value:0</td><td>0.2</td><td>(0.1836 0.9178)</td><td>0.1836</td><td>0.9178</td></tr>
<tr><td>1476</td><td>SET2_K09_CLUSTER_2195</td><td>gi|34894172|ref|NP_908411.1| putative acetoacetyl-coenzyme A thiolase [Oryza sativa (japonica cultivar-group)] >gi|13486634|dbj|BAB39872.1| putative acetoacetyl-coenzyme A thiolase [Oryza sativa (japonica cultivar-group)] e_value:0</td><td>11667.m00108|LOC_Os01g02020|protein acetyl-CoA acyltransferases e_value:0</td><td>0.16</td><td>(0.0824 0.5165)</td><td>0.0824</td><td>0.5165</td></tr>
<tr><td>1477</td><td>SET6_M04_CLUSTER_13058</td><td>gi|51090358|dbj|BAD35619.1| SNF7 protein-like [Oryza sativa (japonica cultivar-group)] e_value:3.48401e-106</td><td>11680.m03965|LOC_Os06g40620|protein Similar to copia-like retroelement pol polyprotein [imported] - Arabidopsis thaliana e_value:5.86626e-108</td><td>0.078</td><td>(0.0410 0.5290)</td><td>0.041</td><td>0.529</td></tr>
<tr><td>1478</td><td>SET4_N20_CLUSTER_21287</td><td>gi|50905585|ref|XP_464281.1| putative glycine decarboxylase complex H-protein [Oryza sativa (japonica cultivar-group)] >gi|51963898|ref|XP_506732.1| PREDICTED OJ1116_A06.24 gene product [Oryza sativa (japonica cultivar-group)] >gi|49388072|dbj|BAD25</td><td>11668.m00701|LOC_Os02g07410|protein glycine cleavage system H protein, putative e_value:1.51479e-75</td><td>0.238</td><td>(0.0755 0.3175)</td><td>0.0755</td><td>0.3175</td></tr>
<tr><td>1479</td><td>WT002_L19_CLUSTER_345</td><td>gi|50929237|ref|XP_474146.1| OSJNBb0059K02.23 [Oryza sativa (japonica cultivar-group)] >gnl|BL_ORD_ID|1593252 gi|38345913|emb|CAE04513.2| OSJNBb0059K02.23 [Oryza sativa (japonica cultivar-group)] >gnl|BL_ORD_ID|1593252 gi|38345895|emb|CAE03539.2| OS</td><td>11680.m00447|LOC_Os06g05160|protein probable sulfate transporter 3.4 e_value:0</td><td>0.251</td><td>(0.3575 1.4234)</td><td>0.3575</td><td>1.4234</td></tr>
<tr><td>1480</td><td>WT011_D11_CLUSTER_31799</td><td>gi|55773942|dbj|BAD72330.1| putative acyl-CoA synthetase [Oryza sativa (japonica cultivar-group)] e_value:0</td><td>11667.m04511|LOC_Os01g46750|protein probable long-chain-fatty-acid-CoA ligase (EC 6.2.1.3) isoform 2 - rape e_value:0</td><td>0.115</td><td>(0.0500 0.4336)</td><td>0.05</td><td>0.4336</td></tr>
<tr><td>1481</td><td>WT006_B12_CLUSTER_151</td><td>gi|50905547|ref|XP_464262.1| putative porphobilinogen deaminase [Oryza sativa (japonica cultivar-group)] >gnl|BL_ORD_ID|1566165 gi|49388602|dbj|BAD25717.1| putative porphobilinogen deaminase [Oryza sativa (japonica cultivar-group)] e_value:4.04756e-171</td><td>11668.m00679|LOC_Os02g07230|protein porphobilinogen deaminase e_value:1.15439e-171</td><td>0.205</td><td>(0.0699 0.3411)</td><td>0.0699</td><td>0.3411</td></tr>
<tr><td>1482</td><td>SET2_G23_CLUSTER_33281</td><td>gi|50906591|ref|XP_464784.1| putative protodermal factor [Oryza sativa (japonica cultivar-group)] >gi|49388954|dbj|BAD26174.1| putative protodermal factor [Oryza sativa (japonica cultivar-group)] e_value:1.30614e-94</td><td>11668.m01262|LOC_Os02g13660|protein Similar to En/Spm-like transposon protein [imported] - Arabidopsis thaliana e_value:3.27147e-96</td><td>0.21</td><td>(0.0999 0.4750)</td><td>0.0999</td><td>0.475</td></tr>
<tr><td>1483</td><td>WT011_F06_CLUSTER_1530</td><td>gi|50944513|ref|XP_481784.1| unknown protein [Oryza sativa (japonica cultivar-group)] >gnl|BL_ORD_ID|1605478 gi|38637014|dbj|BAD03272.1| unknown protein [Oryza sativa (japonica cultivar-group)] e_value:0</td><td>11674.m02480|LOC_Os08g25460|protein TFIIE alpha subunit, putative e_value:0</td><td>0.138</td><td>(0.0739 0.5347)</td><td>0.0739</td><td>0.5347</td></tr>
<tr><td>1484</td><td>WT013_K21_CLUSTER_15801</td><td>gi|54287455|gb|AAV31199.1| putative sister-chromatid cohesion protein [Oryza sativa (japonica cultivar-group)] e_value:0</td><td>11682.m00937|LOC_Os05g09620|protein sister-chromatide cohesion protein, putative e_value:0</td><td>0.17</td><td>(0.0892 0.5263)</td><td>0.0892</td><td>0.5263</td></tr>
<tr><td>1485</td><td>WT013_H03_CLUSTER_15801</td><td>gi|54287455|gb|AAV31199.1| putative sister-chromatid cohesion protein [Oryza sativa (japonica cultivar-group)] e_value:0</td><td>11682.m00937|LOC_Os05g09620|protein sister-chromatide cohesion protein, putative e_value:0</td><td>0.168</td><td>(0.0878 0.5218)</td><td>0.0878</td><td>0.5218</td></tr>
<tr><td>1486</td><td>WT008_L18_CLUSTER_780</td><td>gi|22135459|gb|AAM93210.1| chromdomain-containing protein CRD101 [Zea mays] e_value:7.91317e-107</td><td>11676.m01475|LOC_Os10g17770|protein 'chromo' (CHRromatin Organization MOdifier) domain, putative e_value:1.6331e-100</td><td>0.287</td><td>(0.3133 1.0899)</td><td>0.3133</td><td>1.0899</td></tr>
<tr><td>1487</td><td>WT010_L03_CLUSTER_9223</td><td>gi|50910619|ref|XP_466798.1| putative SET domain protein SUVR2 [Oryza sativa (japonica cultivar-group)] >gnl|BL_ORD_ID|1579175 gi|47847802|dbj|BAD21578.1| putative SET domain protein SUVR2 [Oryza sativa (japonica cultivar-group)] >gnl|BL_ORD_ID|1579</td><td>11668.m03929|LOC_Os02g40770|protein expressed protein e_value:1.64583e-142</td><td></td><td>(0.5735 -1.0000)</td><td>0.5735</td><td>-1</td></tr>
<tr><td>1488</td><td>WT006_K14_CLUSTER_13273</td><td>gi|18103933|emb|CAC83301.1| cytochrome P450 reductase [Triticum aestivum] e_value:0</td><td>11674.m01420|LOC_Os08g14570|protein FAD binding domain, putative e_value:0</td><td>0.128</td><td>(0.1738 1.3558)</td><td>0.1738</td><td>1.3558</td></tr>
<tr><td>1489</td><td>WT012_F23_CLUSTER_6703</td><td>gi|50252182|dbj|BAD28177.1| putative Chain C, Structure Of The Plant Transcriptional Regulator Pbf-2 [Oryza sativa (japonica cultivar-group)] >gnl|BL_ORD_ID|235107 gi|50251252|dbj|BAD28032.1| putative Chain C, Structure Of The Plant Transcriptional Reg</td><td>11668.m00583|LOC_Os02g06370|protein expressed protein e_value:1.89875e-104</td><td>0.17</td><td>(0.0933 0.5477)</td><td>0.0933</td><td>0.5477</td></tr>
<tr><td>1490</td><td>WT005_N12_CLUSTER_33697</td><td>gi|1218004|gb|AAC49183.1| SDL5A >gnl|BL_ORD_ID|404036 gi|2129826|pir||S63668 phragmoplastin 5 - soybean e_value:0</td><td>11682.m04612|LOC_Os05g48240|protein phragmoplastin 5 - soybean e_value:0</td><td>0.055</td><td>(0.0269 0.4881)</td><td>0.0269</td><td>0.4881</td></tr>
<tr><td>1491</td><td>WT006_L10_CLUSTER_32414</td><td>gi|55775374|ref|XP_550613.1| putative 5-enolpyruvylshikimate-3-phosphate synthase [Oryza sativa (japonica cultivar-group)] >gnl|BL_ORD_ID|1752866 gi|55297191|dbj|BAD68865.1| putative 5-enolpyruvylshikimate-3-phosphate synthase [Oryza sativa (japonica c</td><td>11680.m00348|LOC_Os06g04280|protein 3-phosphoshikimate 1-carboxyvinyltransferase e_value:0</td><td>0.141</td><td>(0.0834 0.5919)</td><td>0.0834</td><td>0.5919</td></tr>
</table>

PDF Complete
Your complimentary use period has ended. Thank you for using PDF Complete.
Click Here to upgrade to Unlimited Pages and Expanded Features

<table>
<tr><td>1492</td><td>WT003_N17_CLUSTER_29814</td><td>gi|18060|emb|CAA46927.1| ribosomal protein S1 [Spinacia oleracea] >gnl|BL_ORD_ID|2318463 gi|322404|pir||A44121 ribosomal protein S1 precursor, chloroplast - spinach >gnl|BL_ORD_ID|2318463 gi|133872|sp|P29344|RR1_SPIOL 30S ribosomal protein S1, chlor</td><td>11669.m02048|LOC_Os03g20100|protein Similar to 30s ribosomal protein s1, chloroplast precursor (cs1). [spinach e_value:0</td><td>0.125</td><td>(0.0452 0.3626)</td><td>0.0452</td><td>0.3626</td></tr>
<tr><td>1493</td><td>WT005_J09_CLUSTER_34727</td><td>gi|45183575|gb|AAS55470.1| little protein 1 [Oryza sativa (japonica cultivar-group)] e_value:1.2288e-32</td><td>11667.m04392|LOC_Os01g45660|protein hypothetical protein e_value:2.19283e-34</td><td>0.266</td><td>(0.0868 0.3257)</td><td>0.0868</td><td>0.3257</td></tr>
<tr><td>1494</td><td>WT002_E17_CLUSTER_33622</td><td>gi|34902012|ref|NP_912352.1| putative LIM-domain protein [Oryza sativa (japonica cultivar-group)] >gnl|BL_ORD_ID|1588816 gi|29893622|gb|AAP06876.1| putative LIM-domain protein [Oryza sativa (japonica cultivar-group)] e_value:3.3031e-97</td><td>11669.m01595|LOC_Os03g15940|protein LIM domain, putative e_value:5.59572e-99</td><td>0.099</td><td>(0.0478 0.4822)</td><td>0.0478</td><td>0.4822</td></tr>
<tr><td>1495</td><td>SET5_K10_CLUSTER_32944</td><td>gi|31433253|gb|AAP54791.1| putative serine/threonine protein kinase [Oryza sativa (japonica cultivar-group)] >gi|37536404|ref|NP_922504.1| putative serine/threonine protein kinase [Oryza sativa (japonica cultivar-group)] >gi|22002138|gb|AAM88622.1|</td><td>11676.m03539|LOC_Os10g38950|protein putative serine/threonine protein kinase e_value:0</td><td>0.073</td><td>(0.0320 0.4374)</td><td>0.032</td><td>0.4374</td></tr>
<tr><td>1496</td><td>WT009_O21_CLUSTER_25781</td><td>gi|50909649|ref|XP_466313.1| putative nucleic acid binding protein [Oryza sativa (japonica cultivar-group)] >gnl|BL_ORD_ID|1576860 gi|46806694|dbj|BAD17764.1| putative nucleic acid binding protein [Oryza sativa (japonica cultivar-group)] e_value:1.6735</td><td>11668.m03417|LOC_Os02g35950|protein RNA recognition motif. (a.k.a. RRM, RBD, or RNP domain), putative e_value:0</td><td>0.17</td><td>(0.0789 0.4649)</td><td>0.0789</td><td>0.4649</td></tr>
<tr><td>1497</td><td>WT002_A03_CLUSTER_23022</td><td>gi|50910073|ref|XP_466525.1| putative NAD-dependent isocitrate dehydrogenase [Oryza sativa (japonica cultivar-group)] >gnl|BL_ORD_ID|1577075 gi|46805298|dbj|BAD16830.1| putative NAD-dependent isocitrate dehydrogenase [Oryza sativa (japonica cultivar-gr</td><td>11668.m03654|LOC_Os02g38200|protein isocitrate dehydrogenase, NAD-dependent e_value:0</td><td>0.112</td><td>(0.0581 0.5202)</td><td>0.0581</td><td>0.5202</td></tr>
<tr><td>1498</td><td>WT008_M05_CLUSTER_32985</td><td>gi|50931743|ref|XP_475399.1| unknown protein [Oryza sativa (japonica cultivar-group)] >gnl|BL_ORD_ID|1596235 gi|49328091|gb|AAT58790.1| unknown protein [Oryza sativa (japonica cultivar-group)] >gnl|BL_ORD_ID|1596235 gi|49328068|gb|AAT58768.1| unknow</td><td>11682.m03707|LOC_Os05g39110|protein selT/selW/selH selenoprotein domain, putative e_value:1.67462e-125</td><td>0.133</td><td>(0.0686 0.5154)</td><td>0.0686</td><td>0.5154</td></tr>
<tr><td>1499</td><td>SET6_O10_CLUSTER_31874</td><td>gi|50900434|ref|XP_462650.1| OSJNBa0064H22.2 [Oryza sativa (japonica cultivar-group)] >gi|38344821|emb|CAD40881.2| OSJNBa0064H22.2 [Oryza sativa (japonica cultivar-group)] e_value:0</td><td>11670.m03533|LOC_Os04g37460|protein glutamate decarboxylase e_value:0</td><td>0.162</td><td>(0.0619 0.3829)</td><td>0.0619</td><td>0.3829</td></tr>
<tr><td>1500</td><td>WT008_I14_CLUSTER_24410</td><td>gi|34913328|ref|NP_918011.1| ripening-related protein-like [Oryza sativa (japonica cultivar-group)] >gnl|BL_ORD_ID|1531482 gi|22093833|dbj|BAC07120.1| ripening-related protein-like [Oryza sativa (japonica cultivar-group)] e_value:8.76176e-115</td><td>11673.m04329|LOC_Os07g44060|protein HAD-superfamily hydrolase, subfamily IA, variant 3, putative e_value:1.86861e-116</td><td>0.093</td><td>(0.0733 0.7868)</td><td>0.0733</td><td>0.7868</td></tr>
<tr><td>1501</td><td>WT007_D10_CLUSTER_17721</td><td>gi|50904515|ref|XP_463746.1| UMP synthase 1 [Oryza sativa (japonica cultivar-group)] >gnl|BL_ORD_ID|1540865 gi|19386829|dbj|BAB86207.1| UMP synthase [Oryza sativa (japonica cultivar-group)] >gnl|BL_ORD_ID|1540865 gi|7381462|gb|AAF61491.1| UMP syntha</td><td>11667.m07248|LOC_Os01g72240|protein Orotidine 5'-phosphate decarboxylase / HUMPS family, putative e_value:0</td><td>0.127</td><td>(0.0554 0.4379)</td><td>0.0554</td><td>0.4379</td></tr>
<tr><td>1502</td><td>WT012_M24_CLUSTER_14033</td><td>gi|32401360|gb|AAP80852.1| EREBP transcription factor [Triticum aestivum] e_value:0</td><td>11680.m00900|LOC_Os06g09390|protein AP2 domain, putative e_value:2.08317e-120</td><td>0.448</td><td>(0.2203 0.4919)</td><td>0.2203</td><td>0.4919</td></tr>
<tr><td>1503</td><td>WT002_O12_CLUSTER_14033</td><td>gi|32401360|gb|AAP80852.1| EREBP transcription factor [Triticum aestivum] e_value:0</td><td>11680.m00900|LOC_Os06g09390|protein AP2 domain, putative e_value:7.21798e-121</td><td>0.474</td><td>(0.2373 0.5006)</td><td>0.2373</td><td>0.5006</td></tr>
<tr><td>1504</td><td>SET4_A16_CLUSTER_33935</td><td>gi|50942917|ref|XP_480986.1| aspartate carbamoyltransferase [Oryza sativa (japonica cultivar-group)] >gi|40253904|dbj|BAD05837.1| aspartate carbamoyl transferase [Oryza sativa (japonica cultivar-group)] >gi|40253740|dbj|BAD05680.1| aspartate carbamo</td><td>11674.m01466|LOC_Os08g15030|protein aspartate carbamoyltransferase e_value:3.12501e-172</td><td>0.14</td><td>(0.0724 0.5162)</td><td>0.0724</td><td>0.5162</td></tr>
<tr><td>1505</td><td>WT013_J16_CLUSTER_9655</td><td>gi|47550675|gb|AAT35236.1| At1g77550 [Arabidopsis thaliana] e_value:0</td><td>11669.m00784|LOC_Os03g08140|protein Tubulin-tyrosine ligase family, putative e_value:0</td><td>0.158</td><td>(0.0897 0.5680)</td><td>0.0897</td><td>0.568</td></tr>
<tr><td>1506</td><td>WT004_J05_CLUSTER_1891</td><td>gi|29468122|gb|AAO85403.1| leucine-rich repeat protein [Oryza sativa] >gnl|BL_ORD_ID|1110710 gi|29468120|gb|AAO85402.1| leucine-rich repeat protein [Oryza sativa] >gnl|BL_ORD_ID|1110710 gi|55297657|dbj|BAD68228.1| leucine-rich repeat protein [Oryza</td><td>11667.m05903|LOC_Os01g59440|protein leucine-rich repeat protein e_value:3.46067e-98</td><td>0.097</td><td>(0.0480 0.4951)</td><td>0.048</td><td>0.4951</td></tr>
<tr><td>1507</td><td>WT012_H02_CLUSTER_34668</td><td>gi|15223534|ref|NP_176037.1| expressed protein [Arabidopsis thaliana] >gnl|BL_ORD_ID|2370286 gi|12323035|gb|AAG51512.1| unknown protein [Arabidopsis thaliana] >gnl|BL_ORD_ID|2370286 gi|25373132|pir||A96606 unknown protein F13N6.17 [imported] - Arabi</td><td>11686.m03674|LOC_Os12g37390|protein expressed protein e_value:4.37872e-76</td><td>0.317</td><td>(0.1414 0.4457)</td><td>0.1414</td><td>0.4457</td></tr>
<tr><td>1508</td><td>WT012_G19_CLUSTER_13369</td><td>gi|54291775|gb|AAV32144.1| unknown protein [Oryza sativa (japonica cultivar-group)] e_value:2.5584e-172</td><td>11682.m02297|LOC_Os05g24880|protein quinone reductase-like protein - Arabidopsis thaliana e_value:7.1324e-174</td><td>0.146</td><td>(0.0756 0.5182)</td><td>0.0756</td><td>0.5182</td></tr>
<tr><td>1509</td><td>WT008_I17_CLUSTER_34461</td><td>gi|11561808|gb|AAC83689.2| 101 kDa heat shock protein; HSP101 [Triticum aestivum] e_value:0</td><td>11682.m04231|LOC_Os05g44340|protein Clp amino terminal domain, putative e_value:0</td><td>0.122</td><td>(0.0365 0.2990)</td><td>0.0365</td><td>0.299</td></tr>
<tr><td>1510</td><td>WT004_B14_CLUSTER_8636</td><td>gi|36939192|gb|AAQ86960.1| ETTIN-like auxin response factor [Triticum aestivum] e_value:0</td><td>11667.m05421|LOC_Os01g54990|protein B3 DNA binding domain, putative e_value:0</td><td>0.286</td><td>(0.1733 0.6063)</td><td>0.1733</td><td>0.6063</td></tr>
<tr><td>1511</td><td>WT004__C23_CLUSTER_32570</td><td>gi|8886324|gb|AAF80449.1| Sec61p [Triticum aestivum] e_value:0</td><td>11681.m01629|LOC_Os09g17840|protein preprotein translocase, SecY subunit e_value:0</td><td>0.019</td><td>(0.0085 0.4370)</td><td>0.0085</td><td>0.437</td></tr>
<tr><td>1512</td><td>WT003_O17_CLUSTER_34790</td><td>gi|40317278|gb|AAR84297.1| UDP-glucose dehydrogenase [Cinnamomum osmophloeum] e_value:0</td><td>11669.m05536|LOC_Os03g55070|protein putative UDP-glucose dehydrogenase e_value:0</td><td>0.03</td><td>(0.0591 1.9704)</td><td>0.0591</td><td>1.9704</td></tr>
<tr><td>1513</td><td>WT013_E06_CLUSTER_7544</td><td>gi|51963834|ref|XP_506700.1| PREDICTED P0482F12.6 gene product [Oryza sativa (japonica cultivar-group)] e_value:4.03563e-97</td><td>11668.m00261|LOC_Os02g03330|protein hypothetical protein e_value:1.19352e-97</td><td>0.396</td><td>(0.1314 0.3320)</td><td>0.1314</td><td>0.332</td></tr>
<tr><td>1514</td><td>SET2_M03_CLUSTER_18308</td><td>gi|53791877|dbj|BAD53999.1| putative CIL [Oryza sativa (japonica cultivar-group)] e_value:1.70336e-144</td><td>11680.m04814|LOC_Os06g48610|protein CCT motif, putative e_value:4.07192e-146</td><td>0.397</td><td>(0.2201 0.5538)</td><td>0.2201</td><td>0.5538</td></tr>
<tr><td>1515</td><td>SET5__C22_CLUSTER_12607</td><td>gi|52077279|dbj|BAD46321.1| putative myb factor [Oryza sativa (japonica cultivar-group)] >gi|52077140|dbj|BAD46186.1| putative myb factor [Oryza sativa (japonica cultivar-group)] e_value:1.14204e-110</td><td>11680.m01452|LOC_Os06g14670|protein Myb-like DNA-binding domain, putative e_value:1.80006e-112</td><td>0.603</td><td>(0.1648 0.2734)</td><td>0.1648</td><td>0.2734</td></tr>
<tr><td>1516</td><td>SET1_L21_CLUSTER_18564</td><td>gi|31433360|gb|AAP54879.1| putative C-4 sterol methyl oxidase [Oryza sativa (japonica cultivar-group)] >gi|37536580|ref|NP_922592.1| putative C-4 sterol methyl oxidase [Oryza sativa (japonica cultivar-group)] >gi|13357250|gb|AAK20047.1| putative C-4</td><td>11676.m03636|LOC_Os10g39810|protein putative C-4 sterol methyl oxidase e_value:9.36929e-156</td><td>0.208</td><td>(0.0882 0.4241)</td><td>0.0882</td><td>0.4241</td></tr>
<tr><td>1517</td><td>WT004_H13_CLUSTER_13962</td><td>gi|34910752|ref|NP_916723.1| P0042A10.5 [Oryza sativa (japonica cultivar-group)] >gnl|BL_ORD_ID|1548667 gi|20161135|dbj|BAB90063.1| BNR repeat domain protein-like [Oryza sativa (japonica cultivar-group)] e_value:0</td><td>11667.m05161|LOC_Os01g52630|protein Regulator of chromosome condensation (RCC1), putative e_value:0</td><td>0.139</td><td>(0.0770 0.5549)</td><td>0.077</td><td>0.5549</td></tr>
<tr><td>1518</td><td>WT011_P03_CLUSTER_21466</td><td>gi|7268763|emb|CAB78969.1| putative protein [Arabidopsis thaliana] >gnl|BL_ORD_ID|1459047 gi|3250676|emb|CAA19684.1| putative protein [Arabidopsis thaliana] >gnl|BL_ORD_ID|1459047 gi|15235140|ref|NP_193702.1| zinc finger (C3HC4-type RING finger) fam</td><td>11686.m04352|LOC_Os12g43560|protein Transposable element protein, putative e_value:0</td><td>0.122</td><td>(0.0867 0.7096)</td><td>0.0867</td><td>0.7096</td></tr>
<tr><td>1519</td><td>WT010_I13_CLUSTER_4628</td><td>gi|18855022|gb|AAL79714.1| putative senescence-associated protein [Oryza sativa] >gnl|BL_ORD_ID|867098 gi|54291040|dbj|BAD61717.1| putative ERD7 protein [Oryza sativa (japonica cultivar-group)] >gnl|BL_ORD_ID|867098</td><td>11680.m05015|LOC_Os06g50330|protein Senescence-associated protein, putative e_value:1.78063e-171</td><td>0.222</td><td>(0.1275 0.5733)</td><td>0.1275</td><td>0.5733</td></tr>
<tr><td>1520</td><td>WT002_K13_CLUSTER_28022</td><td>gi|50932753|ref|XP_475904.1| putative ribosomal protein L21 [Oryza sativa (japonica cultivar-group)] >gnl|BL_ORD_ID|1599478 gi|50878314|gb|AAT85089.1| putative 50S ribosomal protein L21 [Oryza sativa (japonica cultivar-group)] >gnl|BL_ORD_ID|1599478</td><td>11682.m04631|LOC_Os05g48410|protein ribosomal protein L21, putative e_value:9.86833e-108</td><td>0.264</td><td>(0.0998 0.3785)</td><td>0.0998</td><td>0.3785</td></tr>
<tr><td>1521</td><td>WT012_P03_CLUSTER_5280</td><td>gi|28268680|dbj|BAC56865.1| respiratory burst oxidase homolog [Nicotiana benthamiana] e_value:0</td><td>11687.m03056|LOC_Os11g33120|protein respiratory burst oxidase homolog e_value:0</td><td>0.219</td><td>(0.0867 0.3962)</td><td>0.0867</td><td>0.3962</td></tr>
<tr><td>1522</td><td>SET4_I11_CLUSTER_15912</td><td>gi|50931173|ref|XP_475114.1| unknown protein [Oryza sativa (japonica cultivar-group)] >gi|54287650|gb|AAV31394.1| unknown protein [Oryza sativa (japonica cultivar-group)] >gi|47777465|gb|AAT38098.1| unknown protein [Oryza sativa (japonica cultivar-g</td><td>11682.m02755|LOC_Os05g29810|protein Similar to AP2/ERF-domain protein e_value:7.85299e-54</td><td>0.417</td><td>(0.2826 0.6776)</td><td>0.2826</td><td>0.6776</td></tr>
<tr><td>1523</td><td>WT009_G04_CLUSTER_18773</td><td>gi|21617972|gb|AAM67022.1| unknown [Arabidopsis thaliana] >gnl|BL_ORD_ID|1219478 gi|8843853|dbj|BAA97379.1| unnamed protein product [Arabidopsis thaliana] >gnl|BL_ORD_ID|1219478 gi|28973371|gb|AAO64010.1| unknown protein [Arabidopsis thaliana] >g</td><td>11668.m04167|LOC_Os02g43480|protein expressed protein e_value:1.44097e-131</td><td>0.16</td><td>(0.0937 0.5853)</td><td>0.0937</td><td>0.5853</td></tr>
<tr><td>1524</td><td>WT004_F08_CLUSTER_25067</td><td>gi|62733529|gb|AAX95646.1| HAD-superfamily hydrolase, subfamily IA, variant 3, putative [Oryza sativa (japonica cultivar-group)] >gnl|BL_ORD_ID|2113782 gi|50918045|ref|XP_469419.1| putative sugar-starvation induced protein [Oryza sativa (japonica culti</td><td>11669.m04969|LOC_Os03g49440|protein putative sugar-starvation induced protein e_value:3.3945e-109</td><td>0.475</td><td>(0.1556 0.3274)</td><td>0.1556</td><td>0.3274</td></tr>
<tr><td>1525</td><td>WT004_L19_CLUSTER_32627</td><td>gi|50904389|ref|XP_463683.1| OSJNBa0093F16.13 [Oryza sativa (japonica cultivar-group)] >gnl|BL_ORD_ID|2301926 gi|20805226|dbj|BAB92893.1| putative pectin-glucuronyltransferase [Oryza sativa (japonica cultivar-group)] >gnl|BL_ORD_ID|2301926 gi|201607</td><td>11667.m07030|LOC_Os01g70200|protein Exostosin family, putative e_value:0</td><td>0.089</td><td>(0.0521 0.5851)</td><td>0.0521</td><td>0.5851</td></tr>
<tr><td>1526</td><td>WT005_E23_CLUSTER_31475</td><td>gi|50948387|ref|XP_483721.1| putative PHG1A protein [Oryza sativa (japonica cultivar-group)] >gnl|BL_ORD_ID|1611348 gi|50725550|dbj|BAD33019.1| putative PHG1A protein [Oryza sativa (japonica cultivar-group)] >gnl|BL_ORD_ID|1611348</td><td>11681.m03453|LOC_Os09g38530|protein Endomembrane protein 70 e_value:0</td><td>0.089</td><td>(0.1227 1.3720)</td><td>0.1227</td><td>1.372</td></tr>
<tr><td>1527</td><td>WT004_K03_CLUSTER_33033</td><td>gi|54291838|gb|AAV32206.1| unknown protein [Oryza sativa (japonica cultivar-group)] >gnl|BL_ORD_ID|1426753 gi|52353577|gb|AAU44143.1| unknow protein [Oryza sativa (japonica cultivar-group)] >gnl|BL_ORD_ID|1426753 gi|56757690|sp|Q9FRA7|Y1A7_ORYSA Hyp</td><td>11682.m00542|LOC_Os05g05940|protein Rubber elongation factor protein (REF) e_value:2.66718e-110</td><td>0.182</td><td>(0.1003 0.5527)</td><td>0.1003</td><td>0.5527</td></tr>
<tr><td>1528</td><td>WT008_M04_CLUSTER_34718</td><td>gi|34907808|ref|NP_915251.1| similar to methionyl-tRNA synthetase [Oryza sativa (japonica cultivar-group)] >gnl|BL_ORD_ID|1546615 gi|19571060|dbj|BAB86486.1| methionyl-tRNA synthetase-like protein [Oryza sativa (japonica cultivar-group)] >gnl|BL_ORD</td><td>11667.m06039|LOC_Os01g60660|protein tRNA binding domain, putative e_value:0</td><td>0.195</td><td>(0.1055 0.5420)</td><td>0.1055</td><td>0.542</td></tr>
<tr><td>1529</td><td>SET1_O10_CLUSTER_17469</td><td>gi|50940357|ref|XP_479706.1| putative Shwachman-Bodian-Diamond syndrome protein [Oryza sativa (japonica cultivar-group)] >gi|51964604|ref|XP_507086.1| PREDICTED P0450B04.21 gene product [Oryza sativa (japonica cultivar-group)]</td><td>11674.m00068|LOC_Os08g01620|protein Uncharacterized protein family UPF0023, putative e_value:3.09429e-159</td><td>0.236</td><td>(0.1332 0.5653)</td><td>0.1332</td><td>0.5653</td></tr>
<tr><td>1530</td><td>SET2_K01_CLUSTER_6375</td><td>gi|51039851|tpg|DAA04955.1| TPA: growth-regulating factor 11 [Oryza sativa (japonica cultivar-group)] >gi|50509463|dbj|BAD31080.1| growth-regulating factor 1-like protein [Oryza sativa (japonica cultivar-group)] e_value:2.73116e-107</td><td>11673.m02684|LOC_Os07g28430|protein expressed protein e_value:6.99059e-109</td><td>0.256</td><td>(0.1528 0.5969)</td><td>0.1528</td><td>0.5969</td></tr>
<tr><td>1531</td><td>WT010_M06_CLUSTER_21364</td><td>gi|5734758|gb|AAD50023.1| Similar to tuftelin-interacting protein [Arabidopsis thaliana] >gnl|BL_ORD_ID|1557763 gi|15220011|ref|NP_173150.1| D111/G-patch domain-containing protein [Arabidopsis thaliana] >gnl|BL_ORD_ID|1557763 gi|25354195|pir||E86306</td><td>11686.m04200|LOC_Os12g42140|protein G-patch domain, putative e_value:0</td><td>0.151</td><td>(0.1013 0.6719)</td><td>0.1013</td><td>0.6719</td></tr>
<tr><td>1532</td><td>WT008_P19_CLUSTER_17494</td><td>gi|50918143|ref|XP_469468.1| unknown protein [Oryza sativa] >gnl|BL_ORD_ID|1536819 gi|13937305|gb|AAK50136.1| unknown protein [Oryza sativa] e_value:0</td><td>11669.m05020|LOC_Os03g49900|protein expressed protein e_value:0</td><td>0.107</td><td>(0.0475 0.4419)</td><td>0.0475</td><td>0.4419</td></tr>
</table>

Your complimentary use period has ended. Thank you for using PDF Complete.
PDF Complete
Click Here to upgrade to Unlimited Pages and Expanded Features

<table>
<tr><td>1533</td><td>WT002_G04_CLUSTER_4493</td><td>gi|22001285|gb|AAM88383.1| peroxidase 1 [Triticum aestivum] >gnl|BL_ORD_ID|1095013 gi|28916432|gb|AAO59389.1| peroxidase precursor [Aegilops tauschii subsp. strangulata] e_value:0</td><td>11667.m07353|LOC_Os01g73220|protein Peroxidase, putative e_value:2.07704e−139</td><td>0.438 (0.2407 0.5499)</td><td>0.2407</td><td>0.5499</td></tr>
<tr><td>1534</td><td>WT003_O15_CLUSTER_20328</td><td>gi|31432916|gb|AAP54492.1| putative Rhizobium−induced nodule development associated protein [Oryza sativa (japonica cultivar−group)] >gnl|BL_ORD_ID|2012540 gi|37535806|ref|NP_922205.1| putative Rhizobium−induced nodule development associated protein [O</td><td>11676.m03213|LOC_Os10g35920|protein putative Rhizobium−induced nodule development associated protein</td><td>0.174 (0.0965 0.5538)</td><td>0.0965</td><td>0.5538</td></tr>
<tr><td>1535</td><td>SET4_M24_CLUSTER_32181</td><td>gi|50931245|ref|XP_475150.1| hypothetical protein [Oryza sativa (japonica cultivar−group)] >gi|49328139|gb|AAT58837.1| hypothetical protein [Oryza sativa (japonica cultivar−group)] e_value:0</td><td>11682.m02872|LOC_Os05g30970|protein Similar to At5g61900 e_value:0</td><td>0.183 (0.1125 0.6152)</td><td>0.1125</td><td>0.6152</td></tr>
<tr><td>1536</td><td>WT006_K16_CLUSTER_12675</td><td>gi|53791362|dbj|BAD52714.1| unknown protein [Oryza sativa (japonica cultivar−group)] >gnl|BL_ORD_ID|774219 gi|53793568|dbj|BAD53338.1| unknown protein [Oryza sativa (japonica cultivar−group)] e_value:0</td><td>11667.m05565|LOC_Os01g56300|protein expressed protein e_value:0</td><td>0.18 (0.1635 0.9091)</td><td>0.1635</td><td>0.9091</td></tr>
<tr><td>1537</td><td>WT004_A03_CLUSTER_34365</td><td>gi|53793441|dbj|BAD53164.1| RNA−binding protein−like [Oryza sativa (japonica cultivar−group)] e_value:2.6662e−128</td><td>11667.m03852|LOC_Os01g40110|protein Zinc finger, C2H2 type, putative e_value:7.03734e−130</td><td>0.29 (0.1502 0.5179)</td><td>0.1502</td><td>0.5179</td></tr>
<tr><td>1538</td><td>WT013_N05_CLUSTER_4149</td><td>gi|50900222|ref|XP_450899.1| putative endo−beta−1,4−glucanase precursor [Oryza sativa (japonica cultivar−group)] >gnl|BL_ORD_ID|1564205 gi|49389205|dbj|BAD26493.1| putative endo−beta−1,4−glucanase precursor [Oryza sativa (japonica cultivar−group)] ></td><td>11668.m04918|LOC_Os02g50490|protein Glycosyl hydrolase family 9 e_value:0</td><td>0.526 (0.2529 0.4811)</td><td>0.2529</td><td>0.4811</td></tr>
<tr><td>1539</td><td>SET4_J02_CLUSTER_17212</td><td>gi|56783782|dbj|BAD81194.1| unknown protein [Oryza sativa (japonica cultivar−group)] >gi|56783767|dbj|BAD81179.1| unknown protein [Oryza sativa (japonica cultivar−group)] e_value:7.34809e−95</td><td>11667.m01659|LOC_Os01g16510|protein hypothetical protein e_value:6.71767e−99</td><td>0.223 (0.1086 0.4879)</td><td>0.1086</td><td>0.4879</td></tr>
<tr><td>1540</td><td>WT009_E15_CLUSTER_32898</td><td>gi|51978970|ref|XP_507362.1| PREDICTED OSJNBa0077F02.127 gene product [Oryza sativa (japonica cultivar−group)] >gnl|BL_ORD_ID|1525935 gi|50936293|ref|XP_477674.1| unknown protein [Oryza sativa (japonica cultivar−group)] >gnl|BL_ORD_ID|1525935 gi|519</td><td>11673.m02148|LOC_Os07g23240|protein ribosomal protein L23, putative e_value:1.26984e−89</td><td>0.221 (0.0942 0.4267)</td><td>0.0942</td><td>0.4267</td></tr>
<tr><td>1541</td><td>WT008_G11_CLUSTER_9951</td><td>gi|51091318|dbj|BAD36053.1| putative beta−1,2−N− acetylglucosaminyltransferase II [Oryza sativa (japonica cultivar−group)] e_value:0</td><td>11668.m05468|LOC_Os02g55470|protein probable N−acetylglucosaminyltransferase [imported] − Arabidopsis thaliana e_value:0</td><td>0.308 (0.1061 0.3444)</td><td>0.1061</td><td>0.3444</td></tr>
<tr><td>1542</td><td>SET1_D23_CLUSTER_14821</td><td>gi|57900478|dbj|BAD87890.1| unknown protein [Oryza sativa (japonica cultivar−group)] e_value:9.90125e−65</td><td>11667.m07252|LOC_Os01g72280|protein expressed protein e_value:1.84381e−66</td><td>0.167 (0.1458 0.8736)</td><td>0.1458</td><td>0.8736</td></tr>
<tr><td>1543</td><td>SET5_M20_CLUSTER_3394</td><td>gi|50929025|ref|XP_474040.1| OSJNBb0034I13.7 [Oryza sativa (japonica cultivar−group)] >gi|32492177|emb|CAE04164.1| OSJNBb0034I13.7 [Oryza sativa (japonica cultivar−group)] e_value:1.6301e−82</td><td>11687.m00267|LOC_Os11g03570|protein hypothetical protein e_value:0</td><td>0.331 (0.1583 0.4788)</td><td>0.1583</td><td>0.4788</td></tr>
<tr><td>1544</td><td>SET5_D16_CLUSTER_16298</td><td>gi|66356278|gb|AAY45744.1| WSCI proteinase inhibitor precursor [Triticum aestivum] e_value:9.74413e−39</td><td>11686.m03550|LOC_Os12g36210|protein subtilisin/chymotrypsin inhibitor − maize e_value:1.88252e−14</td><td>0.666 (0.4321 0.6487)</td><td>0.4321</td><td>0.6487</td></tr>
<tr><td>1545</td><td>SET4_P23_CLUSTER_4549</td><td>gi|51090893|dbj|BAD35466.1| unknown protein [Oryza sativa (japonica cultivar−group)] e_value:5.59191e−87</td><td>11680.m03911|LOC_Os06g40110|protein At5g23710 e_value:1.38788e−88</td><td>0.646 (0.2926 0.4532)</td><td>0.2926</td><td>0.4532</td></tr>
<tr><td>1546</td><td>SET2_O07_CLUSTER_24732</td><td>gi|50947659|ref|XP_483357.1| putative stress related−like protein interactor [Oryza sativa (japonica cultivar−group)] >gi|42408514|dbj|BAD09693.1| putative stress related−like protein interactor [Oryza sativa (japonica cultivar−group)] e_value:5.54389e</td><td>11681.m02943|LOC_Os09g32250|protein expressed protein e_value:5.43692e−175</td><td>0.413 (0.1686 0.4085)</td><td>0.1686</td><td>0.4085</td></tr>
<tr><td>1547</td><td>SET1_O08_CLUSTER_24760</td><td>gi|55296476|dbj|BAD68672.1| unknown protein [Oryza sativa (japonica cultivar−group)] e_value:5.89365e−123</td><td>11680.m00400|LOC_Os06g04780|protein expressed protein e_value:1.5185e−124</td><td>0.292 (0.1767 0.6053)</td><td>0.1767</td><td>0.6053</td></tr>
<tr><td>1548</td><td>SET1_G13_CLUSTER_7264</td><td>gi|50921739|ref|XP_471230.1| OSJNBa0095E20.4 [Oryza sativa (japonica cultivar−group)] >gi|38347506|emb|CAE02417.2| OSJNBa0095E20.4 [Oryza sativa (japonica cultivar−group)] e_value:3.30731e−77</td><td>11670.m00411|LOC_Os04g05010|protein CBS domain, putative e_value:5.67393e−79</td><td>0.398 (0.1659 0.4166)</td><td>0.1659</td><td>0.4166</td></tr>
<tr><td>1549</td><td>WT013_N09_CLUSTER_17531</td><td>gi|52077307|dbj|BAD46348.1| unknown protein [Oryza sativa (japonica cultivar−group)] >gnl|BL_ORD_ID|252451 gi|52076039|dbj|BAD46492.1| unknown protein [Oryza sativa (japonica cultivar−group)] e_value:8.77044e−82</td><td>11681.m03158|LOC_Os09g35690|protein Similar to At3g63530 e_value:5.61956e−77</td><td>0.139 (0.0846 0.6072)</td><td>0.0846</td><td>0.6072</td></tr>
<tr><td>1550</td><td>WT013_I10_CLUSTER_452</td><td>gi|55741372|ref|XP_550351.1| unknown protein [Oryza sativa (japonica cultivar−group)] >gnl|BL_ORD_ID|1744089 gi|55296085|dbj|BAD67647.1| unknown protein [Oryza sativa (japonica cultivar−group)] e_value:5.41465e−137</td><td>11680.m00624|LOC_Os06g06780|protein expressed protein e_value:1.53514e−138</td><td>0.505 (0.1267 0.2507)</td><td>0.1267</td><td>0.2507</td></tr>
<tr><td>1551</td><td>WT013_H23_CLUSTER_19537</td><td>gi|39546276|emb|CAD40692.3| OSJNBa0083D01.9 [Oryza sativa (japonica cultivar−group)] >gnl|BL_ORD_ID|1536923 gi|50923695|ref|XP_472208.1| OSJNBa0083D01.9 [Oryza sativa (japonica cultivar−group)] e_value:2.54812e−177</td><td>11687.m02635|LOC_Os11g28990|protein TBC domain, putative e_value:6.46602e−157</td><td>0.126 (0.2338 1.8490)</td><td>0.2338</td><td>1.849</td></tr>
<tr><td>1552</td><td>WT013_G12_CLUSTER_22618</td><td>gi|34897432|ref|NP_910062.1| hypothetical protein [Oryza sativa (japonica cultivar−group)] >gnl|BL_ORD_ID|1535164 gi|28269410|gb|AAO37953.1| hypothetical protein [Oryza sativa (japonica cultivar−group)] >gnl|BL_ORD_ID|1535164 gi|27573342|gb|AAO20060</td><td>11669.m06491|LOC_Os03g63320|protein hypothetical protein e_value:2.22146e−96</td><td>0.223 (0.1318 0.5921)</td><td>0.1318</td><td>0.5921</td></tr>
<tr><td>1553</td><td>WT013_A24_CLUSTER_12182</td><td>gi|13605609|gb|AAK32798.1| At1g66140/F15E12_19 [Arabidopsis thaliana] >gnl|BL_ORD_ID|2356214 gi|15218939|ref|NP_176788.1| zinc finger (C2H2 type) family protein [Arabidopsis thaliana] >gnl|BL_ORD_ID|2356214 gi|16604378|gb|AAL24195.1| At1g66140/F15E1</td><td>11669.m01331|LOC_Os03g13600|protein expressed protein e_value:2.70874e−72</td><td>0.737 (0.2067 0.2806)</td><td>0.2067</td><td>0.2806</td></tr>
<tr><td>1554</td><td>WT012_P05_CLUSTER_27097</td><td>gi|50905767|ref|XP_464372.1| putative cytochrome P450 [Oryza sativa (japonica cultivar−group)] >gnl|BL_ORD_ID|1566671 gi|46390067|dbj|BAD15442.1| putative cytochrome P450 [Oryza sativa (japonica cultivar−group)] >gnl|BL_ORD_ID|1566671 gi|46390036|db</td><td>11668.m00851|LOC_Os02g09290|protein Cytochrome P450 e_value:0</td><td>0.421 (0.2179 0.5171)</td><td>0.2179</td><td>0.5171</td></tr>
<tr><td>1555</td><td>WT012_M07_CLUSTER_22540</td><td>gi|34915034|ref|NP_918864.1| putative acetone−cyanohydrin lyase [Oryza sativa (japonica cultivar−group)] e_value:1.05092e−106</td><td>11667.m03593|LOC_Os01g37630|protein hydrolase, alpha/beta fold family e_value:3.44757e−108</td><td>0.433 (0.1747 0.4032)</td><td>0.1747</td><td>0.4032</td></tr>
<tr><td>1556</td><td>WT012_E19_CLUSTER_15021</td><td>gi|50251931|dbj|BAD27867.1| tobamovirus multiplication protein 3−like [Oryza sativa (japonica cultivar−group)] >gnl|BL_ORD_ID|229009 gi|50251908|dbj|BAD27846.1| tobamovirus multiplication protein 3−like [Oryza sativa (japonica cultivar−group)] e_value:</td><td>11668.m04433|LOC_Os02g45870|protein hypothetical protein e_value:1.17291e−139</td><td>0.153 (0.0819 0.5341)</td><td>0.0819</td><td>0.5341</td></tr>
<tr><td>1557</td><td>WT012_E18_CLUSTER_15021</td><td>gi|50251931|dbj|BAD27867.1| tobamovirus multiplication protein 3−like [Oryza sativa (japonica cultivar−group)] >gnl|BL_ORD_ID|229009 gi|50251908|dbj|BAD27846.1| tobamovirus multiplication protein 3−like [Oryza sativa (japonica cultivar−group)] e_value:</td><td>11668.m04433|LOC_Os02g45870|protein hypothetical protein e_value:1.17291e−139</td><td>0.153 (0.0819 0.5341)</td><td>0.0819</td><td>0.5341</td></tr>
<tr><td>1558</td><td>WT011_L11_CLUSTER_8198</td><td>gi|50938775|ref|XP_478915.1| putative heterogeneous nuclear ribonucleoprotein A1 [Oryza sativa (japonica cultivar−group)] >gnl|BL_ORD_ID|1529818 gi|38175737|dbj|BAC55617.2| putative heterogeneous nuclear ribonucleoprotein A1 [Oryza sativa (japonica cul</td><td>11687.m03923|LOC_Os11g41890|protein RNA recognition motif. (a.k.a. RRM, RBD, or RNP domain), putative e_value:0</td><td>0.08 (0.0394 0.4904)</td><td>0.0394</td><td>0.4904</td></tr>
<tr><td>1559</td><td>WT011_I20_CLUSTER_11339</td><td>gi|39579226|gb|AAR28765.1| bZIP transcription factor RF2b [Oryza sativa (japonica cultivar−group)] e_value:2.30304e−143</td><td>11673.m04776|LOC_Os07g48180|protein bZIP transcription factor, putative e_value:4.53928e−123</td><td>0.304 (0.1999 0.6574)</td><td>0.1999</td><td>0.6574</td></tr>
<tr><td>1560</td><td>WT010_F03_CLUSTER_18656</td><td>gi|50897260|gb|AAT85769.1| At2g05810 [Arabidopsis thaliana] >gnl|BL_ORD_ID|1001690 gi|20466608|gb|AAM20621.1| unknown protein [Arabidopsis thaliana] >gnl|BL_ORD_ID|1001690 gi|20197848|gb|AAM15279.1| hypothetical protein [Arabidopsis thaliana] >gn</td><td>11681.m00421|LOC_Os09g05030|protein Armadillo/beta−catenin−like repeat, putative e_value:0</td><td>0.156 (0.0954 0.6114)</td><td>0.0954</td><td>0.6114</td></tr>
<tr><td>1561</td><td>WT009_M02_CLUSTER_18762</td><td>gi|52075626|dbj|BAD44797.1| unknown protein [Oryza sativa (japonica cultivar−group)] e_value:6.8807e−81</td><td>11680.m00308|LOC_Os06g03920|protein expressed protein e_value:1.97947e−82</td><td>0.279 (0.1892 0.6790)</td><td>0.1892</td><td>0.679</td></tr>
<tr><td>1562</td><td>WT008_P01_CLUSTER_21967</td><td>gi|51535811|dbj|BAD37896.1| ARG1−like protein [Oryza sativa (japonica cultivar−group)] >gnl|BL_ORD_ID|242708 gi|51535147|dbj|BAD37859.1| ARG1−like protein [Oryza sativa (japonica cultivar−group)] e_value:0</td><td>11680.m01282|LOC_Os06g13060|protein DnaJ domain, putative e_value:8.74099e−175</td><td>0.196 (0.1144 0.5840)</td><td>0.1144</td><td>0.584</td></tr>
<tr><td>1563</td><td>WT008_F02_CLUSTER_24008</td><td>gi|50915328|ref|XP_468128.1| putative pectinesterase [Oryza sativa (japonica cultivar−group)] >gnl|BL_ORD_ID|1583247 gi|47497485|dbj|BAD19539.1| putative pectinesterase [Oryza sativa (japonica cultivar−group)] e_value:0</td><td>11668.m05330|LOC_Os02g54190|protein Pectinesterase, putative e_value:0</td><td>0.44 (0.2179 0.4955)</td><td>0.2179</td><td>0.4955</td></tr>
<tr><td>1564</td><td>WT007_L02_CLUSTER_11320</td><td>gi|42569790|ref|NP_181548.2| glycosyl transferase family 1 protein [Arabidopsis thaliana] e_value:0</td><td>11686.m03888|LOC_Os12g39340|protein glycosyl transferase, group 1 family protein, putative e_value:0</td><td>0.171 (0.0777 0.4544)</td><td>0.0777</td><td>0.4544</td></tr>
<tr><td>1565</td><td>WT006_N20_CLUSTER_9637</td><td>gi|31430030|gb|AAP52001.1| putative disease resistance protein [Oryza sativa (japonica cultivar−group)] >gnl|BL_ORD_ID|2007952 gi|37530824|ref|NP_919714.1| putative disease resistance protein [Oryza sativa (japonica cultivar−group)] >gnl|BL_ORD_ID|2</td><td>11687.m04378|LOC_Os11g46210|protein NB−ARC domain, putative e_value:0</td><td>0.293 (0.3662 1.2498)</td><td>0.3662</td><td>1.2498</td></tr>
<tr><td>1566</td><td>WT006_J06_CLUSTER_7167</td><td>gi|50929909|ref|XP_474482.1| OSJNBb0020J19.14 [Oryza sativa (japonica cultivar−group)] >gnl|BL_ORD_ID|1594832 gi|38567836|emb|CAE05785.2| OSJNBb0020J19.14 [Oryza sativa (japonica cultivar−group)] e_value:0</td><td>11670.m05911|LOC_Os04g59590|protein Putative methyltransferase e_value:0</td><td>0.221 (0.1806 0.8183)</td><td>0.1806</td><td>0.8183</td></tr>
<tr><td>1567</td><td>WT005_D15_CLUSTER_17566</td><td>gi|58843559|gb|AAW82908.1| pyrroline−5−carboxylate reductase [Triticum aestivum] e_value:3.94251e−156</td><td>11667.m07221|LOC_Os01g71990|protein pyrroline−5−carboxylate reductase e_value:6.06705e−133</td><td>0.146 (0.0804 0.5518)</td><td>0.0804</td><td>0.5518</td></tr>
<tr><td>1568</td><td>WT003_A24_CLUSTER_21633</td><td>gi|8118432|gb|AAF72988.1| beta−expansin [Oryza sativa] e_value:3.17895e−133</td><td>11669.m00029|LOC_Os03g01270|protein beta−expansin 3 e_value:8.06183e−135</td><td>0.28 (0.2090 0.7463)</td><td>0.209</td><td>0.7463</td></tr>
<tr><td>1569</td><td>SET5_N05_CLUSTER_24208</td><td>gi|479388|pir||S33631 tritin − wheat >gi|391929|dbj|BAA02948.1| tritin [Triticum aestivum] e_value:2.5504e−155</td><td>11667.m00666|LOC_Os01g07300|protein Ribosome inactivating protein e_value:3.36074e−88</td><td>0.712 (0.2543 0.3570)</td><td>0.2543</td><td>0.357</td></tr>
<tr><td>1570</td><td>WT006_H14_CLUSTER_8151</td><td>gi|51964976|ref|XP_507272.1| PREDICTED P0419H09.18 gene product [Oryza sativa (japonica cultivar−group)] >gnl|BL_ORD_ID|1609261 gi|50946997|ref|XP_483026.1| serine/threonine protein kinase(OSK4) [Oryza sativa (japonica cultivar−group)] >gnl|BL_ORD_I</td><td>11674.m03764|LOC_Os08g37800|protein Protein kinase domain, putative e_value:0</td><td>0.086 (0.0490 0.5696)</td><td>0.049</td><td>0.5696</td></tr>
<tr><td>1571</td><td>WT008_K14_CLUSTER_2123</td><td>gi|21885|emb|CAA43844.1| wheat type V thionin [Triticum aestivum] >gnl|BL_ORD_ID|2349291 gi|21887|emb|CAA43845.1| wheat type V thionin [Triticum aestivum] >gnl|BL_ORD_ID|2349291 gi|100807|pir||S23511 thionin type V precursor − wheat >gnl|BL_ORD_I</td><td>11680.m03134|LOC_Os06g32550|protein Plant thionin, putative e_value:7.19177e−15</td><td>0.418 (0.6002 1.4344)</td><td>0.6002</td><td>1.4344</td></tr>
<tr><td>1572</td><td>WT006_N16_CLUSTER_3430</td><td>gi|47777361|gb|AAT37995.1| putative receptor protein kinase [Oryza sativa (japonica cultivar−group)] e_value:0</td><td>11682.m04923|LOC_Os05g51070|protein Leucine Rich Repeat, putative e_value:0</td><td>0.436 (0.2246 0.5156)</td><td>0.2246</td><td>0.5156</td></tr>
<tr><td>1573</td><td>WT002_O18_CLUSTER_27813</td><td>gi|15428276|gb|AAK97808.1| cinnamyl alcohol dehydrogenase 1a [Festuca arundinacea] e_value:0</td><td>11668.m00872|LOC_Os02g09490|protein oxidoreductase, zinc−binding dehydrogenase family e_value:0</td><td>0.209 (0.0703 0.3354)</td><td>0.0703</td><td>0.3354</td></tr>
<tr><td>1574</td><td>WT002_E15_CLUSTER_30611</td><td>gi|50929079|ref|XP_474067.1| OSJNBb0079B02.9 [Oryza sativa (japonica cultivar−group)] >gnl|BL_ORD_ID|1594453 gi|38347315|emb|CAE05963.2| OSJNBa0063C18.4 [Oryza sativa (japonica cultivar−group)] >gnl|BL_ORD_ID|1594453 gi|38344906|emb|CAD41850.2| OSJN</td><td>11670.m05396|LOC_Os04g54870|protein RNA recognition motif. (a.k.a. RRM, RBD, or RNP domain), putative e_value:1.16972e−115</td><td>0.156 (0.0821 0.5282)</td><td>0.0821</td><td>0.5282</td></tr>
</table>

PDF Complete
Your complimentary use period has ended. Thank you for using PDF Complete.
Click Here to upgrade to Unlimited Pages and Expanded Features

<table>
<tr><td>1575</td><td>SET6_A20_CLUSTER_30611</td><td>gi|50929079|ref|XP_474067.1| OSJNBb0079B02.9 [Oryza sativa (japonica cultivar-group)] >gi|38347315|emb|CAE05963.2| OSJNBa0063C18.4 [Oryza sativa (japonica cultivar-group)] >gi|38344906|emb|CAD41850.2| OSJNBb0079B02.9 [Oryza sativa (japonica cultivar</td><td>11670.m05396|LOC_Os04g54870|protein RNA recognition motif. (a.k.a. RRM, RBD, or RNP domain), putative e_value:3.40335e-115</td><td>0.143</td><td>(0.0842 0.5870)</td><td>0.0842</td><td>0.587</td></tr>
<tr><td>1576</td><td>WT005_E22_CLUSTER_33981</td><td>gi|31429892|gb|AAP51881.1| putative purple acid phosphatase [Oryza sativa (japonica cultivar-group)] >gnl|BL_ORD_ID|2007832 gi|37530584|ref|NP_919594.1| putative purple acid phosphatase [Oryza sativa (japonica cultivar-group)] >gnl|BL_ORD_ID|2007832</td><td>11669.m01324|LOC_Os03g13540|protein Ser/Thr protein phosphatase family protein, putative e_value:2.42613e-163</td><td>0.232</td><td>(0.1141 0.4928)</td><td>0.1141</td><td>0.4928</td></tr>
<tr><td>1577</td><td>SET4__C09_CLUSTER_9915</td><td>gi|29028804|gb|AAO64781.1| At2g03350 [Arabidopsis thaliana] >gi|4335763|gb|AAD17440.1| expressed protein [Arabidopsis thaliana] >gi|25364434|pir||D84447 hypothetical protein At2g03350 [imported] - Arabidopsis thaliana >gi|18395537|ref|NP_565300.1</td><td>11669.m01071|LOC_Os03g11210|protein hypothetical protein e_value:1.04975e-86</td><td>0.09</td><td>(0.0527 0.5842)</td><td>0.0527</td><td>0.5842</td></tr>
<tr><td>1578</td><td>WT005_B06_CLUSTER_607</td><td>gi|38345411|emb|CAE03102.2| OSJNBa0017B10.17 [Oryza sativa (japonica cultivar-group)] >gnl|BL_ORD_ID|1522200 gi|50927997|ref|XP_473526.1| OSJNBa0017B10.17 [Oryza sativa (japonica cultivar-group)] e_value:7.06964e-46</td><td>11670.m04676|LOC_Os04g47820|protein hypothetical protein e_value:9.06765e-48</td><td>0.437</td><td>(0.1174 0.2686)</td><td>0.1174</td><td>0.2686</td></tr>
<tr><td>1579</td><td>WT003__C05_CLUSTER_21384</td><td>gi|50901994|ref|XP_463430.1| putative permease 1 [Oryza sativa (japonica cultivar-group)] >gnl|BL_ORD_ID|2302317 gi|20804662|dbj|BAB92350.1| putative permease 1 [Oryza sativa (japonica cultivar-group)] >gnl|BL_ORD_ID|2302317 gi|14587294|dbj|BAB61205</td><td>11681.m01357|LOC_Os09g15170|protein xanthine/uracil permease family protein, putative e_value:0</td><td>0.049</td><td>(0.1291 2.6622)</td><td>0.1291</td><td>2.6622</td></tr>
<tr><td>1580</td><td>SET3__C09_CLUSTER_31759</td><td>gi|48209969|gb|AAT40532.1| hypothetical protein [Solanum demissum] e_value:1.92102e-164</td><td>11669.m01809|LOC_Os03g17900|protein hydrolase, alpha/beta fold family, putative e_value:0</td><td>0.204</td><td>(0.0871 0.4275)</td><td>0.0871</td><td>0.4275</td></tr>
<tr><td>1581</td><td>WT003_F07_CLUSTER_4435</td><td>gi|51536369|dbj|BAD37500.1| putative cytochrome P450 [Oryza sativa (japonica cultivar-group)] e_value:0</td><td>11680.m04257|LOC_Os06g43380|protein hypothetical protein e_value:6.79295e-85</td><td>0.488</td><td>(0.2458 0.5037)</td><td>0.2458</td><td>0.5037</td></tr>
<tr><td>1582</td><td>WT012_H12_CLUSTER_17918</td><td>gi|34897492|ref|NP_910092.1| putative protein kinase [Oryza sativa (japonica cultivar-group)] >gnl|BL_ORD_ID|1535201 gi|29244698|gb|AAO73290.1| putative protein kinase [Oryza sativa (japonica cultivar-group)] e_value:0</td><td>11669.m06455|LOC_Os03g63020|protein putative protein kinase e_value:0</td><td>0.138</td><td>(0.0876 0.6338)</td><td>0.0876</td><td>0.6338</td></tr>
<tr><td>1583</td><td>SET4_K06_CLUSTER_5778</td><td>gi|50911060|ref|XP_466963.1| double-stranded RNA-binding domain (DsRBD)-containing protein-like [Oryza sativa (japonica cultivar-group)] >gi|51964196|ref|XP_506882.1| PREDICTED OSJNBa0014E22.37 gene product [Oryza sativa (japonica cultivar-group)] ></td><td>11668.m04077|LOC_Os02g42600|protein Double-stranded RNA binding motif, putative e_value:0</td><td>0.163</td><td>(0.0727 0.4467)</td><td>0.0727</td><td>0.4467</td></tr>
<tr><td>1584</td><td>SET3_M03_CLUSTER_15382</td><td>gi|31431642|gb|AAP53386.1| putative Pollen specific protein C13 precursor [Oryza sativa (japonica cultivar-group)] >gi|23396189|gb|AAN31783.1| Putataive pollen specific protein C13 precursor [Oryza sativa (japonica cultivar-group)]</td><td>11676.m01918|LOC_Os10g22590|protein Pollen proteins Ole e I family, putative e_value:2.14928e-73</td><td>0.184</td><td>(0.1113 0.6053)</td><td>0.1113</td><td>0.6053</td></tr>
<tr><td>1585</td><td>SET1_J07_CLUSTER_34142</td><td>gi|33327292|gb|AAQ09002.1| hypothetical protein [Phaseolus vulgaris] e_value:1.31786e-25</td><td>11686.m03217|LOC_Os12g32950|protein hypothetical protein e_value:2.72007e-32</td><td>0.012</td><td>(0.0065 0.5352)</td><td>0.0065</td><td>0.5352</td></tr>
<tr><td>1586</td><td>WT003_N08_CLUSTER_32533</td><td>gi|50582733|gb|AAT78803.1| putative endoplasmic reticulum oxidoreductin [Oryza sativa (japonica cultivar-group)] e_value:0</td><td>11669.m05288|LOC_Os03g52340|protein putative endoplasmic reticulum oxidoreductin e_value:0</td><td>0.134</td><td>(0.0570 0.4264)</td><td>0.057</td><td>0.4264</td></tr>
<tr><td>1587</td><td>WT012_N15_CLUSTER_18553</td><td>gi|38346229|emb|CAE02051.2| OJ990528_30.9 [Oryza sativa (japonica cultivar-group)] >gnl|BL_ORD_ID|1523417 gi|21740790|emb|CAD41535.1| OSJNBb0091E11.4 [Oryza sativa (japonica cultivar-group)] >gnl|BL_ORD_ID|1523417 gi|50925813|ref|XP_473005.1| OJ9905</td><td>11670.m03994|LOC_Os04g41570|protein hypothetical protein e_value:2.9807e-72</td><td>0.141</td><td>(0.0767 0.5437)</td><td>0.0767</td><td>0.5437</td></tr>
<tr><td>1588</td><td>WT013_E10_CLUSTER_274</td><td>gi|50941187|ref|XP_480121.1| putative transcription factor Myb1 [Oryza sativa (japonica cultivar-group)] >gnl|BL_ORD_ID|1601920 gi|28812130|dbj|BAC64998.1| putative transcription factor Myb1 [Oryza sativa (japonica cultivar-group)] e_value:6.83776e-109</td><td>11674.m00470|LOC_Os08g05510|protein myb-like DNA-binding domain, SHAQKYF class, putative e_value:1.94043e-131</td><td>0.389</td><td>(0.1598 0.4113)</td><td>0.1598</td><td>0.4113</td></tr>
<tr><td>1589</td><td>WT006_A19_CLUSTER_15808</td><td>gi|57834050|emb|CAE05423.2| OSJNBa0035I04.11 [Oryza sativa (japonica cultivar-group)] e_value:2.3063e-75</td><td>11670.m03347|LOC_Os04g34720|protein Similar to At2g19260 e_value:1.38501e-37</td><td>0.522</td><td>(0.6228 1.1924)</td><td>0.6228</td><td>1.1924</td></tr>
<tr><td>1590</td><td>WT004_B24_CLUSTER_33169</td><td>gi|57900353|dbj|BAD87343.1| putative aspartate aminotransferase [Oryza sativa (japonica cultivar-group)] e_value:0</td><td>11667.m05481|LOC_Os01g55540|protein aspartate aminotransferase, cytoplasmic (ec 2.6.1.1) (transaminase a).</td><td>0.084</td><td>(0.0314 0.3738)</td><td>0.0314</td><td>0.3738</td></tr>
<tr><td>1591</td><td>WT005_O11_CLUSTER_31888</td><td>gi|1621627|gb|AAB68035.1| manganese superoxide dismutase [Triticum aestivum] >gnl|BL_ORD_ID|269058 gi|7433342|pir||T06258 superoxide dismutase (EC 1.15.1.1) (Mn) precursor - wheat e_value:3.69537e-126</td><td>11682.m02396|LOC_Os05g25850|protein superoxide dismutase e_value:4.04261e-96</td><td>0.416</td><td>(0.1376 0.3310)</td><td>0.1376</td><td>0.331</td></tr>
<tr><td>1592</td><td>WT003_H08_CLUSTER_34168</td><td>gi|34898866|ref|NP_910779.1| putative NADH dehydrogenase [Oryza sativa (japonica cultivar-group)] >gnl|BL_ORD_ID|1532289 gi|51963608|ref|XP_506513.1| PREDICTED P0503D09.102 gene product [Oryza sativa (japonica cultivar-group)] >gnl|BL_ORD_ID|1532289</td><td>11673.m04439|LOC_Os07g45090|protein NADH-quinone oxidoreductase, F subunit e_value:0</td><td>0.058</td><td>(0.0275 0.4768)</td><td>0.0275</td><td>0.4768</td></tr>
<tr><td>1593</td><td>WT002__C03_CLUSTER_31984</td><td>gi|451193|gb|AAC37416.1| wali7 >gnl|BL_ORD_ID|375283 gi|7489671|pir||T06984 hypothetical protein wali7 - wheat (fragment) >gnl|BL_ORD_ID|375283 gi|1090845|prf||2019486B wali7 gene e_value:2.47909e-121</td><td>11687.m00423|LOC_Os11g05050|protein aluminium-induced protein, putative e_value:1.00828e-116</td><td>0.083</td><td>(0.0584 0.7078)</td><td>0.0584</td><td>0.7078</td></tr>
<tr><td>1594</td><td>WT013_N24_CLUSTER_34266</td><td>gi|51091326|dbj|BAD36061.1| putative protein phosphatase 2C [Oryza sativa (japonica cultivar-group)] e_value:3.56241e-172</td><td>11668.m05478|LOC_Os02g55560|protein Protein phosphatase 2C, putative e_value:7.26025e-174</td><td>0.073</td><td>(0.0647 0.8902)</td><td>0.0647</td><td>0.8902</td></tr>
<tr><td>1595</td><td>WT007_P01_CLUSTER_2515</td><td>gi|51090737|dbj|BAD35217.1| putative Sac domain-containing inositol phosphatase 3 [Oryza sativa (japonica cultivar-group)] e_value:0</td><td>11680.m00916|LOC_Os06g09540|protein Similar to SAC domain protein 3 e_value:0</td><td>0.273</td><td>(0.1302 0.4764)</td><td>0.1302</td><td>0.4764</td></tr>
<tr><td>1596</td><td>WT013__C22_CLUSTER_7405</td><td>gi|31433117|gb|AAP54670.1| putative alpha/beta hydrolase [Oryza sativa (japonica cultivar-group)] >gnl|BL_ORD_ID|2012701 gi|37536162|ref|NP_922383.1| putative alpha/beta hydrolase [Oryza sativa (japonica cultivar-group)] >gnl|BL_ORD_ID|2012701 gi|10</td><td>11669.m00177|LOC_Os03g02520|protein hydrolase, alpha/beta fold family, putative e_value:8.10518e-176</td><td>0.176</td><td>(0.1158 0.6575)</td><td>0.1158</td><td>0.6575</td></tr>
<tr><td>1597</td><td>WT006_F07_CLUSTER_2249</td><td>gi|40641240|emb|CAE47490.1| cytochrome P450 [Triticum aestivum] e_value:0</td><td>11682.m03965|LOC_Os05g41440|protein cytochrome p450 98a1 (ec 1.14.-.-) e_value:0</td><td>0.215</td><td>(0.0666 0.3100)</td><td>0.0666</td><td>0.31</td></tr>
<tr><td>1598</td><td>WT008_M12_CLUSTER_32726</td><td>gi|50928747|ref|XP_473901.1| OSJNBa0093O08.9 [Oryza sativa (japonica cultivar-group)] >gnl|BL_ORD_ID|1592117 gi|38345607|emb|CAD41890.2| OSJNBa0093O08.9 [Oryza sativa (japonica cultivar-group)] e_value:0</td><td>11670.m05161|LOC_Os04g52690|protein ATPase, AAA family, putative e_value:0</td><td>0.215</td><td>(0.1003 0.4658)</td><td>0.1003</td><td>0.4658</td></tr>
<tr><td>1599</td><td>WT005__C23_CLUSTER_19433</td><td>gi|50941185|ref|XP_480120.1| putative tyrosyl-tRNA synthetase [Oryza sativa (japonica cultivar-group)] >gnl|BL_ORD_ID|1601919 gi|37573038|dbj|BAC98550.1| putative tyrosyl-tRNA synthetase [Oryza sativa (japonica cultivar-group)] e_value:0</td><td>11674.m00468|LOC_Os08g05490|protein tyrosyl-tRNA synthetase e_value:0</td><td>0.148</td><td>(0.1018 0.6868)</td><td>0.1018</td><td>0.6868</td></tr>
<tr><td>1600</td><td>SET2_A18_CLUSTER_33170</td><td>gi|50939335|ref|XP_479195.1| putative splicing factor, arginine/serine-rich 2 (Splicing factor SC35) [Oryza sativa (japonica cultivar-group)] >gi|33146621|dbj|BAC79909.1| putative splicing factor, arginine/serine-rich 2 (Splicing factor SC35) [Oryza sa</td><td>11673.m04222|LOC_Os07g43050|protein RNA recognition motif. (a.k.a. RRM, RBD, or RNP domain), putative e_value:3.73124e-</td><td>0.235</td><td>(0.1598 0.6816)</td><td>0.1598</td><td>0.6816</td></tr>
<tr><td>1601</td><td>WT013_L22_CLUSTER_4199</td><td>gi|51535036|dbj|BAD37407.1| putative GDP-4-keto-6-deoxy-D-mannose-3,5- epimerase-4-reductase [Oryza sativa (japonica cultivar-group)] e_value:2.14595e-171</td><td>11680.m04353|LOC_Os06g44270|protein expressed protein e_value:5.5851e-173</td><td>0.155</td><td>(0.0460 0.2972)</td><td>0.046</td><td>0.2972</td></tr>
<tr><td>1602</td><td>WT004_L09_CLUSTER_19891</td><td>gi|51535964|dbj|BAD38045.1| putative DEAD-box protein abstrakt [Oryza sativa (japonica cultivar-group)] e_value:0</td><td>11668.m00509|LOC_Os02g05660|protein Transposable element protein, putative e_value:0</td><td>0.106</td><td>(0.0634 0.5976)</td><td>0.0634</td><td>0.5976</td></tr>
<tr><td>1603</td><td>WT009_N18_CLUSTER_33392</td><td>gi|51963650|ref|XP_506569.1| PREDICTED OJ1409_C08.26 gene product [Oryza sativa (japonica cultivar-group)] >gnl|BL_ORD_ID|1534256 gi|50940013|ref|XP_479534.1| putative alpha-galactosidase [Oryza sativa (japonica cultivar-group)]</td><td>11673.m04774|LOC_Os07g48160|protein Melibiase, putative e_value:0</td><td>0.182</td><td>(0.0716 0.3934)</td><td>0.0716</td><td>0.3934</td></tr>
<tr><td>1604</td><td>SET3_O12_CLUSTER_10002</td><td>gi|50878390|gb|AAT85165.1| unknown protein [Oryza sativa (japonica cultivar-group)] e_value:0</td><td>11676.m00291|LOC_Os10g03830|protein expressed protein</td><td>0.186</td><td>(0.0874 0.4709)</td><td>0.0874</td><td>0.4709</td></tr>
<tr><td>1605</td><td>WT003_O03_CLUSTER_24754</td><td>gi|50948535|ref|XP_483795.1| putative NAC2 protein [Oryza sativa (japonica cultivar-group)] >gnl|BL_ORD_ID|1612297 gi|51965118|ref|XP_507343.1| PREDICTED P0604E01.48-1 gene product [Oryza sativa (japonica cultivar-group)] >gnl|BL_ORD_ID|1612297 gi|4</td><td>11674.m04534|LOC_Os08g44820|protein No apical meristem (NAM) protein, putative e_value:0</td><td>0.235</td><td>(0.1289 0.5487)</td><td>0.1289</td><td>0.5487</td></tr>
<tr><td>1606</td><td>WT009_O11_CLUSTER_34300</td><td>gi|55733912|gb|AAV59419.1| putative cellular retinaldehyde-binding/triple function [Oryza sativa (japonica cultivar-group)] >gnl|BL_ORD_ID|1741329 gi|50931475|ref|XP_475265.1| putative cellular retinaldehyde-binding/triple function [Oryza sativa (japon</td><td>11682.m03352|LOC_Os05g35460|protein CRAL/TRIO, N-terminus, putative e_value:0</td><td>0.277</td><td>(0.1159 0.4182)</td><td>0.1159</td><td>0.4182</td></tr>
<tr><td>1607</td><td>WT006_G20_CLUSTER_34300</td><td>gi|55733912|gb|AAV59419.1| putative cellular retinaldehyde-binding/triple function [Oryza sativa (japonica cultivar-group)] >gnl|BL_ORD_ID|1741329 gi|50931475|ref|XP_475265.1| putative cellular retinaldehyde-binding/triple function [Oryza sativa (japon</td><td>11682.m03352|LOC_Os05g35460|protein CRAL/TRIO, N-terminus, putative e_value:0</td><td>0.248</td><td>(0.1121 0.4514)</td><td>0.1121</td><td>0.4514</td></tr>
<tr><td>1608</td><td>WT003_O12_CLUSTER_1969</td><td>gi|51536102|dbj|BAD38226.1| putative formate--tetrahydrofolate ligase [Oryza sativa (japonica cultivar-group)] e_value:0</td><td>11681.m02520|LOC_Os09g27420|protein formate--tetrahydrofolate ligase e_value:0</td><td>0.131</td><td>(0.0585 0.4460)</td><td>0.0585</td><td>0.446</td></tr>
<tr><td>1609</td><td>WT002_O04_CLUSTER_23045</td><td>gi|5731259|gb|AAD48837.1| alanine:glyoxylate aminotransferase 2 homolog [Arabidopsis thaliana] e_value:0</td><td>11669.m00720|LOC_Os03g07570|protein alanine:glyoxylate aminotransferase 2 homolog e_value:0</td><td>0.111</td><td>(0.0754 0.6797)</td><td>0.0754</td><td>0.6797</td></tr>
<tr><td>1610</td><td>WT005_D20_CLUSTER_33988</td><td>gi|50901228|ref|XP_463047.1| expressed protein [Oryza sativa (japonica cultivar-group)] >gnl|BL_ORD_ID|1564788 gi|41469312|gb|AAS07168.1| expressed protein [Oryza sativa (japonica cultivar-group)] e_value:2.99684e-135</td><td>11669.m03804|LOC_Os03g38540|protein expressed protein e_value:8.12006e-137</td><td>0.196</td><td>(0.1402 0.7142)</td><td>0.1402</td><td>0.7142</td></tr>
<tr><td>1611</td><td>SET3_F20_CLUSTER_13690</td><td>gi|23499291|emb|CAA78262.2| eukaryotic initiation factor 4E p26 subunit [Triticum aestivum] >gi|30923212|sp|P29557|IF4E1_WHEAT Eukaryotic translation initiation factor 4E-1 (eIF4E-1) (eIF-4E-1) (mRNA cap-binding protein) (eIF-4F 25 kDa subunit) (eIF-4F</td><td>11667.m07422|LOC_Os01g73880|protein translation initiation factor eIF-4F chain p26 - wheat e_value:2.34801e-106</td><td>0.19</td><td>(0.1044 0.5507)</td><td>0.1044</td><td>0.5507</td></tr>
<tr><td>1612</td><td>WT009_B06_CLUSTER_32352</td><td>gi|50901098|ref|XP_462982.1| hypothetical protein [Oryza sativa (japonica cultivar-group)] >gnl|BL_ORD_ID|1564617 gi|41393229|gb|AAS01952.1| hypothetical protein [Oryza sativa (japonica cultivar-group)] e_value:2.67261e-107</td><td>11669.m00855|LOC_Os03g08770|protein expressed protein e_value:7.47756e-112</td><td>0.141</td><td>(0.0755 0.5361)</td><td>0.0755</td><td>0.5361</td></tr>
<tr><td>1613</td><td>WT008_I15_CLUSTER_20292</td><td>gi|50942161|ref|XP_480608.1| putative gamma-aminobutyrate transaminase subunit precursor isozyme 3 [Oryza sativa (japonica cultivar-group)] >gnl|BL_ORD_ID|1603046 gi|42761306|dbj|BAD11549.1| putative gamma-aminobutyrate transaminase subunit precursor i</td><td>11674.m01001|LOC_Os08g10510|protein aminotransferase, class III e_value:0</td><td>0.13</td><td>(0.0677 0.5225)</td><td>0.0677</td><td>0.5225</td></tr>
<tr><td>1614</td><td>SET5_D15_CLUSTER_32075</td><td>gi|16323258|gb|AAL15363.1| At1g26660/T24P13_4 [Arabidopsis thaliana] >gi|18396004|ref|NP_564254.1| expressed protein [Arabidopsis thaliana] >gi|13926327|gb|AAK49628.1| At1g26660/T24P13_4 [Arabidopsis thaliana] e_value:3.21276e-61</td><td>11681.m03184|LOC_Os09g35920|protein At1g26660/T24P13_4 e_value:2.13859e-81</td><td>0.195</td><td>(0.1167 0.5971)</td><td>0.1167</td><td>0.5971</td></tr>
<tr><td>1615</td><td>WT012_O12_CLUSTER_31756</td><td>gi|728594|emb|CAA88558.1| glycine rich protein, RNA binding protein [Hordeum vulgare subsp. vulgare] >gnl|BL_ORD_ID|1721251 gi|1076731|pir||S53050 RNA binding protein - barley e_value:1.48259e-90</td><td>11669.m04679|LOC_Os03g46770|protein retrotransposon protein, putative, Ty1-copia sub-class e_value:6.26524e-71</td><td>0.338</td><td>(0.1282 0.3797)</td><td>0.1282</td><td>0.3797</td></tr>
<tr><td>1616</td><td>WT009_N03_CLUSTER_20806</td><td>gi|55168010|gb|AAV43878.1| importin beta 1 [Oryza sativa (japonica cultivar-group)] >gnl|BL_ORD_ID|1465378 gi|3983663|dbj|BAA34861.1| importin-beta1 [Oryza sativa (japonica cultivar-group)] e_value:7.74867e-134</td><td>11682.m02670|LOC_Os05g28510|protein Importin-beta N-terminal domain, putative e_value:2.57269e-135</td><td>0.716</td><td>(0.5378 0.7511)</td><td>0.5378</td><td>0.7511</td></tr>
<tr><td>1617</td><td>WT008_J20_CLUSTER_14549</td><td>gi|5763821|emb|CAB53258.1| E2F protein [Triticum sp.] e_value:0</td><td>11668.m03156|LOC_Os02g33430|protein E2F homolog e_value:0</td><td>0.214</td><td>(0.1069 0.5008)</td><td>0.1069</td><td>0.5008</td></tr>
</table>

<table>
<tr><th></th><th></th><th></th><th></th><th></th><th></th><th></th><th></th></tr>
<tr><td>1618</td><td>WT013_P24_CLUSTER_4078</td><td>gi|50905035|ref|XP_464006.1| putative Anthranilate phosphoribosyltransferase [Oryza sativa (japonica cultivar-group)] >gnl|BL_ORD_ID|1566250 gi|41052564|dbj|BAD07746.1| putative Anthranilate phosphoribosyltransferase [Oryza sativa (japonica cultivar-</td><td>11668.m00316|LOC_Os02g03850|protein Glycosyl transferase family, a/b domain, putative e_value:0</td><td>0.186</td><td>(0.0761 0.4087)</td><td>0.0761</td><td>0.4087</td></tr>
<tr><td>1619</td><td>SET6_G03_CLUSTER_3144</td><td>gi|34912830|ref|NP_917762.1| P0501G01.24 [Oryza sativa (japonica cultivar-group)] e_value:0</td><td>11667.m04220|LOC_Os01g43520|protein Neutral/alkaline nonlysosomal ceramidase e_value:0</td><td>0.137</td><td>(0.0823 0.5998)</td><td>0.0823</td><td>0.5998</td></tr>
<tr><td>1620</td><td>WT007__C16_CLUSTER_29306</td><td>gi|50941687|ref|XP_480371.1| putative mRNA cap methyltransferase [Oryza sativa (japonica cultivar-group)] >gnl|BL_ORD_ID|1602787 gi|51964728|ref|XP_507148.1| PREDICTED P0455A11.11 gene product [Oryza sativa (japonica cultivar-group)] >gnl|BL_ORD_ID|</td><td>11674.m00759|LOC_Os08g08200|protein mRNA capping enzyme, large subunit, putative e_value:0</td><td>0.077</td><td>(0.0448 0.5788)</td><td>0.0448</td><td>0.5788</td></tr>
<tr><td>1621</td><td>SET2_M16_CLUSTER_32421</td><td>gi|34895876|ref|NP_909282.1| putative pod-specific dehydrogenase SAC25 [Oryza sativa (japonica cultivar-group)] >gi|13872934|dbj|BAB44039.1| putative pod-specific dehydrogenase SAC25 [Oryza sativa (japonica cultivar-group)] >gi|9663977|dbj|BAB03618.</td><td>11667.m00514|LOC_Os01g05840|protein oxidoreductase, short chain dehydrogenase/reductase family e_value:1.20497e-156</td><td>0.123</td><td>(0.0847 0.6889)</td><td>0.0847</td><td>0.6889</td></tr>
<tr><td>1622</td><td>WT002_F03_CLUSTER_383</td><td>gi|3617837|gb|AAC36055.1| gibberellin action negative regulator SPY [Hordeum vulgare] e_value:0</td><td>11674.m04500|LOC_Os08g44510|protein TPR Domain, putative e_value:0</td><td>0.197</td><td>(0.0887 0.4513)</td><td>0.0887</td><td>0.4513</td></tr>
<tr><td>1623</td><td>SET5_N18_CLUSTER_32780</td><td>gi|27764549|gb|AAO23079.1| unknown [Glycine max] e_value:7.27288e-35</td><td>11669.m01155|LOC_Os03g11990|protein BolA-like protein, putative e_value:1.72062e-49</td><td>0.043</td><td>(0.0218 0.5111)</td><td>0.0218</td><td>0.5111</td></tr>
<tr><td>1624</td><td>WT011_D13_CLUSTER_21494</td><td>gi|21280855|gb|AAM45085.1| putative protein phosphatase-2C [Arabidopsis thaliana] >gnl|BL_ORD_ID|1208569 gi|19310556|gb|AAL85011.1| putative protein phosphatase-2C [Arabidopsis thaliana] >gnl|BL_ORD_ID|1208569</td><td>11687.m00081|LOC_Os11g01790|protein Similar to F21B7.20 e_value:0</td><td>0.134</td><td>(0.1205 0.8985)</td><td>0.1205</td><td>0.8985</td></tr>
<tr><td>1625</td><td>WT008_N10_CLUSTER_27487</td><td>gi|51091884|dbj|BAD36696.1| CAX-interacting protein 4 (CAXIP4)-like [Oryza sativa (japonica cultivar-group)] >gnl|BL_ORD_ID|382992 gi|50725214|dbj|BAD34148.1| CAX-interacting protein 4 (CAXIP4)-like [Oryza sativa (japonica cultivar-group)] e_value:8.77</td><td>11681.m00067|LOC_Os09g01640|protein Transposable element protein, putative e_value:2.43601e-143</td><td>0.253</td><td>(0.1571 0.6203)</td><td>0.1571</td><td>0.6203</td></tr>
<tr><td>1626</td><td>WT004_D03_CLUSTER_17136</td><td>gi|13516481|dbj|BAB40450.1| long-chain acyl-CoA synthetase [Arabidopsis thaliana] e_value:0</td><td>11686.m00431|LOC_Os12g04990|protein long-chain acyl-CoA synthetase e_value:0</td><td>0.154</td><td>(0.0653 0.4231)</td><td>0.0653</td><td>0.4231</td></tr>
<tr><td>1627</td><td>WT013_M08_CLUSTER_15681</td><td>gi|38347596|emb|CAE04887.2| OSJNBa0042I15.9 [Oryza sativa (japonica cultivar-group)] e_value:0</td><td>11670.m03202|LOC_Os04g33370|protein cytochrom P450-like protein e_value:0</td><td>0.338</td><td>(0.1255 0.3708)</td><td>0.1255</td><td>0.3708</td></tr>
<tr><td>1628</td><td>WT005_J01_CLUSTER_33367</td><td>gi|50945595|ref|XP_482325.1| unknown protein [Oryza sativa (japonica cultivar-group)] >gnl|BL_ORD_ID|1606188 gi|51964886|ref|XP_507227.1| PREDICTED P0453D01.16-2 gene product [Oryza sativa (japonica cultivar-group)] >gnl|BL_ORD_ID|1606188 gi|3757295</td><td>11674.m03100|LOC_Os08g31470|protein expressed protein e_value:0</td><td>0.078</td><td>(0.0689 0.8805)</td><td>0.0689</td><td>0.8805</td></tr>
<tr><td>1629</td><td>WT005_G16_CLUSTER_9048</td><td>gi|50918241|ref|XP_469517.1| putative RNA polymerase I subunit [Oryza sativa] >gnl|BL_ORD_ID|1537061 gi|13324793|gb|AAK18841.1| putative RNA polymerase I subunit [Oryza sativa] e_value:8.03565e-48</td><td>11669.m05075|LOC_Os03g50380|protein putative RNA polymerase I subunit e_value:5.55478e-50</td><td>0.385</td><td>(0.1946 0.5054)</td><td>0.1946</td><td>0.5054</td></tr>
<tr><td>1630</td><td>WT005_M20_CLUSTER_34478</td><td>gi|34914002|ref|NP_918348.1| dnaJ-like protein [Oryza sativa (japonica cultivar-group)] >gnl|BL_ORD_ID|1541898 gi|20146299|dbj|BAB89081.1| dnaJ-like protein [Oryza sativa (japonica cultivar-group)] e_value:3.99947e-178</td><td>11667.m03586|LOC_Os01g37560|protein DnaJ domain, putative e_value:1.23801e-151</td><td>0.167</td><td>(0.1115 0.6688)</td><td>0.1115</td><td>0.6688</td></tr>
<tr><td>1631</td><td>WT007_F18_CLUSTER_17192</td><td>gi|50915312|ref|XP_468120.1| putative CCR4-NOT transcription complex, subunit 2; NOT2 [Oryza sativa (japonica cultivar-group)] >gnl|BL_ORD_ID|1583239 gi|47497477|dbj|BAD19531.1| putative CCR4-NOT transcription complex, subunit 2; NOT2 [Oryza sativa (ja</td><td>11668.m05322|LOC_Os02g54120|protein NOT2 / NOT3 / NOT5 family, putative e_value:0</td><td>0.188</td><td>(0.2016 1.0729)</td><td>0.2016</td><td>1.0729</td></tr>
<tr><td>1632</td><td>SET5_J18_CLUSTER_10973</td><td>gi|50252067|dbj|BAD27997.1| transport protein SEC61 [Oryza sativa (japonica cultivar-group)] >gi|585963|sp|P38385|SC61G_ORYSA Protein transport protein SEC61 gamma subunit e_value:1.60725e-31</td><td>11668.m00787|LOC_Os02g08180|protein protein translocase SEC61 complex gamma subunit, archaeal and eukaryotic e_value:2.45425e-33</td><td>0.04</td><td>(0.0064 0.1574)</td><td>0.0064</td><td>0.1574</td></tr>
<tr><td>1633</td><td>WT004_A15_CLUSTER_34183</td><td>gi|1705612|sp|P55307|CATA1_HORVU Catalase isozyme 1 >gnl|BL_ORD_ID|2354834 gi|684946|gb|AAA96947.1| catalase e_value:0</td><td>11680.m05102|LOC_Os06g51150|protein catalase, putative</td><td>0.084</td><td>(0.0423 0.5044)</td><td>0.0423</td><td>0.5044</td></tr>
<tr><td>1634</td><td>SET1_B04_CLUSTER_34096</td><td>gi|34914212|ref|NP_918453.1| P0697C12.13 [Oryza sativa (japonica cultivar-group)] e_value:5.2328e-32</td><td>11667.m03683|LOC_Os01g38510|protein Sec61beta family, putative e_value:1.06422e-33</td><td>0.082</td><td>(0.0594 0.7250)</td><td>0.0594</td><td>0.725</td></tr>
<tr><td>1635</td><td>WT009_J22_CLUSTER_24875</td><td>gi|30692538|gb|AAP33475.1| polygalacturonase-like protein [Fragaria x ananassa] e_value:3.18517e-166</td><td>11669.m00266|LOC_Os03g03350|protein polygalacturonase-like protein e_value:0</td><td>0.179</td><td>(0.0748 0.4172)</td><td>0.0748</td><td>0.4172</td></tr>
<tr><td>1636</td><td>SET1_F11_CLUSTER_33093</td><td>gi|53793323|dbj|BAD54544.1| unknown protein [Oryza sativa (japonica cultivar-group)] e_value:2.13193e-31</td><td>11680.m03600|LOC_Os06g37070|protein Uncharacterised protein family (UPF0197) e_value:3.15088e-33</td><td>0.074</td><td>(0.0368 0.4944)</td><td>0.0368</td><td>0.4944</td></tr>
<tr><td>1637</td><td>SET2_O06_CLUSTER_12099</td><td>gi|21693585|gb|AAM75355.1| ABA response element binding factor [Triticum aestivum] e_value:0</td><td>11667.m06412|LOC_Os01g64000|protein bZIP transcription factor, putative e_value:2.21413e-126</td><td>0.221</td><td>(0.2293 1.0386)</td><td>0.2293</td><td>1.0386</td></tr>
<tr><td>1638</td><td>SET2_J08_CLUSTER_34840</td><td>gi|29367421|gb|AAO72583.1| putative PHD-finger protein [Oryza sativa (japonica cultivar-group)] >gi|32352174|dbj|BAC78580.1| hypothetical protein [Oryza sativa (japonica cultivar-group)] >gi|29367607|gb|AAO72665.1| putative PHD-finger protein [Oryza</td><td>11669.m00442|LOC_Os03g04980|protein PHD-finger, putative e_value:1.72712e-103</td><td>0.191</td><td>(0.1081 0.5649)</td><td>0.1081</td><td>0.5649</td></tr>
<tr><td>1639</td><td>SET1_A19_CLUSTER_18407</td><td>gi|51964684|ref|XP_507126.1| PREDICTED OJ1613_G04.25 gene product [Oryza sativa (japonica cultivar-group)] e_value:2.09042e-55</td><td>11674.m00347|LOC_Os08g04310|protein Plastocyanin-like domain, putative e_value:3.63129e-57</td><td>0.486</td><td>(0.2861 0.5882)</td><td>0.2861</td><td>0.5882</td></tr>
<tr><td>1640</td><td>WT011_L01_CLUSTER_379</td><td>gi|1731990|emb|CAA70815.1| serine carboxypeptidase II, CP-MII [Hordeum vulgare subsp. vulgare] e_value:0</td><td>11667.m00581|LOC_Os01g06490|protein Serine carboxypeptidase e_value:0</td><td>0.153</td><td>(0.2639 1.7248)</td><td>0.2639</td><td>1.7248</td></tr>
<tr><td>1641</td><td>SET2_G05_CLUSTER_23746</td><td>gi|31087733|gb|AAO20339.1| puroindoline a [Aegilops tauschii] >gi|30039050|gb|AAP07051.1| puroindoline-a [Aegilops tauschii] >gi|30039048|gb|AAP07050.1| puroindoline-a [Aegilops tauschii] >gi|30039046|gb|AAP07049.1| puroindoline-a [Aegilops tausc</td><td>11680.m02986|LOC_Os06g31070|protein prolamin e_value:1.27769e-06</td><td></td><td>(0.7869 -1.0000)</td><td>0.7869</td><td>-1</td></tr>
<tr><td>1642</td><td>SET2_M11_CLUSTER_13068</td><td>gi|4007850|emb|CAA65313.1| alpha purothionin [Triticum aestivum] e_value:2.12628e-77</td><td>11680.m03134|LOC_Os06g32550|protein Plant thionin, putative e_value:3.2873e-24</td><td>0.321</td><td>(0.5259 1.6387)</td><td>0.5259</td><td>1.6387</td></tr>
<tr><td>1643</td><td>SET1_A16_CLUSTER_13068</td><td>gi|4007850|emb|CAA65313.1| alpha purothionin [Triticum aestivum] e_value:2.12628e-77</td><td>11680.m03134|LOC_Os06g32550|protein Plant thionin, putative e_value:3.2873e-24</td><td>0.321</td><td>(0.5259 1.6387)</td><td>0.5259</td><td>1.6387</td></tr>
<tr><td>1644</td><td>WT004_D11_CLUSTER_24775</td><td>gi|34898484|ref|NP_910588.1| Similar to Prunus armeniaca ethylene-forming-enzyme-like dioxygenase. (U97530) [Oryza sativa (japonica cultivar-group)] >gnl|BL_ORD_ID|1743610 gi|34898464|ref|NP_910578.1| Similar to Prunus armeniaca ethylene-forming-enzyme</td><td>11680.m00752|LOC_Os06g08020|protein oxidoreductase, 2OG-Fe(II) oxygenase family, putative e_value:2.35155e-103</td><td>0.383</td><td>(0.2673 0.6972)</td><td>0.2673</td><td>0.6972</td></tr>
<tr><td>1645</td><td>SET1_K15_CLUSTER_1515</td><td>gi|533692|gb|AAC97524.1| protease inhibitor [Glycine max] >gi|7451427|pir||T06381 proteinase inhibitor - soybean e_value:2.66581e-15</td><td>11669.m00315|LOC_Os03g03810|protein proteinase inhibitor - rice e_value:1.20587e-16</td><td>0.632</td><td>(0.3715 0.5874)</td><td>0.3715</td><td>0.5874</td></tr>
<tr><td>1646</td><td>WT005_P11_CLUSTER_33989</td><td>gi|30793446|dbj|BAC76688.1| 27K protein [Triticum aestivum] e_value:3.11559e-70</td><td>11669.m01870|LOC_Os03g18450|protein 27K protein e_value:3.3004e-87</td><td>0.441</td><td>(0.2791 0.6330)</td><td>0.2791</td><td>0.633</td></tr>
<tr><td>1647</td><td>SET5_G11_CLUSTER_21657</td><td>gi|65993807|gb|AAY42617.1| dimeric alpha-amylase inhibitor [Triticum aestivum] e_value:3.93873e-80</td><td>11673.m01084|LOC_Os07g11330|protein Protease inhibitor/seed storage/LTP family, putative e_value:5.95975e-21</td><td>0.546</td><td>(0.5520 1.0109)</td><td>0.552</td><td>1.0109</td></tr>
<tr><td>1648</td><td>SET4_P12_CLUSTER_15892</td><td>gi|39578552|gb|AAR28678.1| alpha amylase inhibitor CM3 [Triticum turgidum subsp. durum] >gi|38098487|gb|AAR10959.1| alpha amylase inhibitor protein [Triticum aestivum] >gi|21713|emb|CAA35597.1| unnamed protein product [Triticum aestivum] >gi|2192</td><td>11673.m01159|LOC_Os07g12080|protein Protease inhibitor/seed storage/LTP family, putative e_value:1.70252e-22</td><td>0.402</td><td>(0.4410 1.0957)</td><td>0.441</td><td>1.0957</td></tr>
<tr><td>1649</td><td>WT011_N12_CLUSTER_16769</td><td>gi|50915540|ref|XP_468234.1| hypothetical protein [Oryza sativa (japonica cultivar-group)] >gnl|BL_ORD_ID|1583355 gi|47497144|dbj|BAD19193.1| hypothetical protein [Oryza sativa (japonica cultivar-group)] >gnl|BL_ORD_ID|1583355</td><td>11668.m05425|LOC_Os02g55050|protein F-box domain, putative e_value:1.24423e-119</td><td>0.426</td><td>(0.3824 0.8979)</td><td>0.3824</td><td>0.8979</td></tr>
<tr><td>1650</td><td>SET4_J09_CLUSTER_18018</td><td>gi|34910446|ref|NP_916570.1| P0510F09.19 [Oryza sativa (japonica cultivar-group)] e_value:9.12559e-150</td><td>11667.m04998|LOC_Os01g51190|protein expressed protein e_value:9.49718e-179</td><td>0.147</td><td>(0.0520 0.3552)</td><td>0.052</td><td>0.3552</td></tr>
<tr><td>1651</td><td>WT005_G24_CLUSTER_11600</td><td>gi|53792659|dbj|BAD53672.1| protodermal factor-like [Oryza sativa (japonica cultivar-group)] e_value:5.10484e-67</td><td>11680.m03482|LOC_Os06g35970|protein expressed protein e_value:1.03928e-68</td><td>0.375</td><td>(0.1751 0.4672)</td><td>0.1751</td><td>0.4672</td></tr>
<tr><td>1652</td><td>SET6_A19_CLUSTER_33886</td><td>gi|4154279|gb|AAD04882.1| C13 endopeptidase NP1 precursor [Hordeum vulgare] e_value:0</td><td>11670.m04418|LOC_Os04g45470|protein Peptidase C13 family, putative e_value:0</td><td>0.279</td><td>(0.1681 0.6031)</td><td>0.1681</td><td>0.6031</td></tr>
<tr><td>1653</td><td>SET1_L15_CLUSTER_21013</td><td>gi|10803445|emb|CAC13149.1| lipid transfer protein [Triticum turgidum subsp. durum] e_value:4.82996e-50</td><td>11676.m03242|LOC_Os10g36170|protein putative lipid transfer protein e_value:3.54641e-26</td><td>1.376</td><td>(0.4020 0.2922)</td><td>0.402</td><td>0.2922</td></tr>
<tr><td>1654</td><td>SET3_O03_CLUSTER_31269</td><td>gi|31431131|gb|AAP52953.1| putative beta-glucosidase [Oryza sativa (japonica cultivar-group)] >gi|37532728|ref|NP_920666.1| putative beta-glucosidase [Oryza sativa (japonica cultivar-group)] >gi|15217237|gb|AAK92581.1| Putative beta-glucosidase [Ory</td><td>11676.m01463|LOC_Os10g17650|protein Similar to beta-glucosidase-like protein e_value:0</td><td>0.137</td><td>(0.1034 0.7548)</td><td>0.1034</td><td>0.7548</td></tr>
<tr><td>1655</td><td>SET2_K16_CLUSTER_32639</td><td>gi|66840998|emb|CAI64398.1| 5a2 protein [Triticum aestivum] e_value:4.976e-51</td><td>11669.m02569|LOC_Os03g25350|protein expressed protein e_value:7.90545e-39</td><td>0.213</td><td>(0.2228 1.0476)</td><td>0.2228</td><td>1.0476</td></tr>
<tr><td>1656</td><td>SET3_G07_CLUSTER_15252</td><td>gi|2894148|emb|CAA11410.1| monomeric alpha-amylase inhibitor [Triticum aestivum] >gi|7438308|pir||T06517 alpha-amylase inhibitor Ima1 precursor, monomeric - wheat e_value:1.10489e-87</td><td>11673.m01087|LOC_Os07g11360|protein Protease inhibitor/seed storage/LTP family, putative e_value:4.04074e-27</td><td>0.527</td><td>(0.5200 0.9873)</td><td>0.52</td><td>0.9873</td></tr>
<tr><td>1657</td><td>WT009_G01_CLUSTER_24345</td><td>gi|34902516|ref|NP_912604.1| P0581F09.16 [Oryza sativa (japonica cultivar-group)] >gnl|BL_ORD_ID|1510980 gi|15408827|dbj|BAB64219.1| unknown protein [Oryza sativa (japonica cultivar-group)] >gnl|BL_ORD_ID|1510980</td><td>11667.m01773|LOC_Os01g18100|protein Rhomboid family, putative e_value:1.13279e-131</td><td>0.261</td><td>(0.1870 0.7171)</td><td>0.187</td><td>0.7171</td></tr>
<tr><td>1658</td><td>WT008_H02_CLUSTER_29474</td><td>gi|21742575|emb|CAD40790.1| OSJNBb0076A22.1 [Oryza sativa (japonica cultivar-group)] >gnl|BL_ORD_ID|1539762 gi|50923579|ref|XP_472150.1| OSJNBb0076A22.1 [Oryza sativa (japonica cultivar-group)] e_value:1.08595e-132</td><td>11686.m03244|LOC_Os12g33220|protein hypothetical protein e_value:6.02145e-140</td><td>0.509</td><td>(0.3488 0.6854)</td><td>0.3488</td><td>0.6854</td></tr>
<tr><td>1659</td><td>WT008_F12_CLUSTER_388</td><td>gi|47156872|gb|AAT12275.1| plastidial ADP-glucose transporter [Hordeum vulgare subsp. vulgare] e_value:0</td><td>11668.m01014|LOC_Os02g10800|protein Similar to brittle-1 protein, chloroplast precursor e_value:9.52933e-158</td><td>0.366</td><td>(0.1729 0.4722)</td><td>0.1729</td><td>0.4722</td></tr>
<tr><td>1660</td><td>WT007_N15_CLUSTER_24370</td><td>gi|50911913|ref|XP_467364.1| putative ornithine carbamoyltransferase [Oryza sativa (japonica cultivar-group)] >gnl|BL_ORD_ID|1580493 gi|41053142|dbj|BAD08085.1| putative ornithine carbamoyltransferase [Oryza sativa (japonica cultivar-group)] e_value:0</td><td>11668.m04614|LOC_Os02g47590|protein ornithine carbamoyltransferase e_value:0</td><td>0.105</td><td>(0.0567 0.5384)</td><td>0.0567</td><td>0.5384</td></tr>
</table>

PDF Complete
Your complimentary use period has ended. Thank you for using PDF Complete.
Click Here to upgrade to Unlimited Pages and Expanded Features

<table>
<tr><td>1661</td><td>WT007_B20_CLUSTER_16882</td><td>gi|50948827|ref|XP_493771.1| putative pyruvate decarboxylase [Oryza sativa (japonica cultivar-group)] >gnl|BL_ORD_ID|2298928 gi|8570064|dbj|BAA96769.1| putative pyruvate decarboxylase [Oryza sativa (japonica cultivar-group)] >gnl|BL_ORD_ID|2298928 g</td><td>11667.m00598|LOC_Os01g06660|protein Thiamine pyrophosphate enzyme, central domain, putative e_value:0</td><td>0.122 (0.0700 0.5741)</td><td>0.07</td><td>0.5741</td></tr>
<tr><td>1662</td><td>WT005_M16_CLUSTER_28828</td><td>gi|50725389|dbj|BAD32863.1| putative ethylene-responsive transcriptional coactivator [Oryza sativa (japonica cultivar-group)] e_value:2.69192e-68</td><td>11680.m03822|LOC_Os06g39240|protein ethylene-responsive transciptional coactivator-like protein e_value:5.39255e-70</td><td>0.179 (0.0521 0.2907)</td><td>0.0521</td><td>0.2907</td></tr>
<tr><td>1663</td><td>WT004_F01_CLUSTER_16244</td><td>gi|32479664|emb|CAE01493.1| P0041A24.5 [Oryza sativa (japonica cultivar-group)] >gnl|BL_ORD_ID|1588354 gi|50924542|ref|XP_472631.1| P0041A24.5 [Oryza sativa (japonica cultivar-group)] e_value:0</td><td>11670.m03496|LOC_Os04g36610|protein Glycosyl hydrolase family 9 e_value:0</td><td>0.419 (0.1436 0.3428)</td><td>0.1436</td><td>0.3428</td></tr>
<tr><td>1664</td><td>WT003_O09_CLUSTER_4414</td><td>gi|32400820|gb|AAP80642.1| 19 kDa globulin [Triticum aestivum] e_value:6.66332e-133</td><td>11682.m04031|LOC_Os05g41970|protein alpha-globulin precursor - rice e_value:1.23721e-31</td><td>0.715 (0.3830 0.5360)</td><td>0.383</td><td>0.536</td></tr>
<tr><td>1665</td><td>SET6_G13_CLUSTER_10263</td><td>gi|33591098|gb|AAQ23056.1| heat shock factor RHSF2 [Oryza sativa (japonica cultivar-group)] e_value:5.07755e-138</td><td>11674.m04374|LOC_Os08g43340|protein HSF-type DNA-binding domain, putative e_value:1.31939e-136</td><td>0.365 (0.2008 0.5506)</td><td>0.2008</td><td>0.5506</td></tr>
<tr><td>1666</td><td>SET5_M18_CLUSTER_20438</td><td>gi|50927903|ref|XP_473479.1| OSJNBa0065O17.16 [Oryza sativa (japonica cultivar-group)] >gi|38345837|emb|CAE01841.2| OSJNBa0084K11.1 [Oryza sativa (japonica cultivar-group)] >gi|38345763|emb|CAE03491.2| OSJNBa0065O17.16 [Oryza sativa (japonica cultiv</td><td>11670.m04606|LOC_Os04g47190|protein branched-chain amino acid aminotransferase, putative e_value:0</td><td>0.191 (0.1207 0.6334)</td><td>0.1207</td><td>0.6334</td></tr>
<tr><td>1667</td><td>SET5_D12_CLUSTER_16391</td><td>gi|33242917|gb|AAQ01162.1| MADS box protein [Oryza sativa (japonica cultivar-group)] >gi|5295990|dbj|BAA81886.1| MADS box-like protein [Oryza sativa (japonica cultivar-group)] e_value:2.96552e-73</td><td>11676.m03561|LOC_Os10g39130|protein putative transcription factor e_value:8.86822e-64</td><td>0.397 (0.3013 0.7597)</td><td>0.3013</td><td>0.7597</td></tr>
<tr><td>1668</td><td>SET4_O19_CLUSTER_5806</td><td>gi|51535274|dbj|BAD38537.1| putative PrMC3 [Oryza sativa (japonica cultivar-group)] e_value:1.94407e-133</td><td>11681.m02656|LOC_Os09g28660|protein expressed protein e_value:2.55837e-135</td><td>0.376 (0.1335 0.3552)</td><td>0.1335</td><td>0.3552</td></tr>
<tr><td>1669</td><td>SET3_G17_CLUSTER_23160</td><td>gi|18029187|gb|AAL56401.1| cystatin [Triticum aestivum] e_value:8.6005e-48</td><td>11669.m03189|LOC_Os03g31510|protein putative cysteine proteinase inhibitor e_value:6.79578e-16</td><td>0.599 (0.6901 1.1527)</td><td>0.6901</td><td>1.1527</td></tr>
<tr><td>1670</td><td>SET2_N10_CLUSTER_2620</td><td>gi|62701872|gb|AAX92945.1| TB2/DP1, HVA22 family, putative [Oryza sativa (japonica cultivar-group)] e_value:5.6527e-76</td><td>11687.m00500|LOC_Os11g05800|protein TB2/DP1, HVA22 family, putative e_value:9.28793e-78</td><td>0.221 (0.1769 0.7992)</td><td>0.1769</td><td>0.7992</td></tr>
<tr><td>1671</td><td>SET2_G04_CLUSTER_28338</td><td>gi|34914972|ref|NP_918833.1| Ser/Thr protein kinase-like protein [Oryza sativa (japonica cultivar-group)] >gi|21952864|dbj|BAC06279.1| receptor protein kinase PERK1-like protein [Oryza sativa (japonica cultivar-group)] e_value:0</td><td>11667.m02777|LOC_Os01g28730|protein Protein kinase domain, putative e_value:0</td><td>0.164 (0.0968 0.5888)</td><td>0.0968</td><td>0.5888</td></tr>
<tr><td>1672</td><td>SET2_B02_CLUSTER_27847</td><td>gi|50941951|ref|XP_480503.1| putative N7 protein [Oryza sativa (japonica cultivar-group)] >gi|40253673|dbj|BAD05616.1| putative N7 protein [Oryza sativa (japonica cultivar-group)] e_value:5.94458e-130</td><td>11674.m00891|LOC_Os08g09450|protein F-box domain, putative e_value:1.40821e-129</td><td>0.313 (0.1853 0.5923)</td><td>0.1853</td><td>0.5923</td></tr>
<tr><td>1673</td><td>WT013_E24_CLUSTER_15972</td><td>gi|34904098|ref|NP_913396.1| unnamed protein product [Oryza sativa (japonica cultivar-group)] e_value:1.13465e-90</td><td>11667.m01192|LOC_Os01g12200|protein hypothetical protein e_value:3.96492e-113</td><td>0.335 (0.1712 0.5116)</td><td>0.1712</td><td>0.5116</td></tr>
<tr><td>1674</td><td>WT013_D12_CLUSTER_26627</td><td>gi|50936627|ref|XP_477841.1| putative glycin-rich protein [Oryza sativa (japonica cultivar-group)] >gnl|BL_ORD_ID|1526016 gi|34394861|dbj|BAC84308.1| putative glycin-rich protein [Oryza sativa (japonica cultivar-group)] >gnl|BL_ORD_ID|1526016 gi|505</td><td>11673.m02414|LOC_Os07g25810|protein transposon protein, putative, CACTA, En/Spm sub-class e_value:5.56952e-170</td><td>0.1 (0.1290 1.2881)</td><td>0.129</td><td>1.2881</td></tr>
<tr><td>1675</td><td>WT012_F14_CLUSTER_2897</td><td>gi|22655378|gb|AAM98281.1| At1g31800/68069_m00159 [Arabidopsis thaliana] >gnl|BL_ORD_ID|1197059 gi|18398119|ref|NP_564384.1| cytochrome P450 family protein [Arabidopsis thaliana] >gnl|BL_ORD_ID|1197059 gi|16648793|gb|AAL25587.1| At1g31800/68069_m001</td><td>11668.m05669|LOC_Os02g57290|protein probable cytochrome P450 [imported] - Arabidopsis thaliana e_value:0</td><td>0.185 (0.1059 0.5738)</td><td>0.1059</td><td>0.5738</td></tr>
<tr><td>1676</td><td>WT009_L03_CLUSTER_3612</td><td>gi|34908342|ref|NP_915518.1| ataxin 3-like protein [Oryza sativa (japonica cultivar-group)] >gnl|BL_ORD_ID|1557108 gi|20805182|dbj|BAB92851.1| putative Machado-Joseph disease gene product ataxin-3 [Oryza sativa (japonica cultivar-group)]</td><td>11667.m06331|LOC_Os01g63250|protein Josephin, putative e_value:1.35497e-145</td><td>0.237 (0.1211 0.5107)</td><td>0.1211</td><td>0.5107</td></tr>
<tr><td>1677</td><td>SET1_O02_CLUSTER_28231</td><td>gi|50928167|ref|XP_473611.1| OSJNBa0011J08.7 [Oryza sativa (japonica cultivar-group)] >gi|32488500|emb|CAE03252.1| OSJNBa0011J08.7 [Oryza sativa (japonica cultivar-group)] e_value:7.57392e-143</td><td>11670.m04803|LOC_Os04g48880|protein beta-carotene hydroxylase, putative e_value:1.67459e-144</td><td>0.3 (0.0955 0.3186)</td><td>0.0955</td><td>0.3186</td></tr>
<tr><td>1678</td><td>WT013_K08_CLUSTER_5909</td><td>gi|50929569|ref|XP_474312.1| OSJNBb0004A17.10 [Oryza sativa (japonica cultivar-group)] >gnl|BL_ORD_ID|1593193 gi|38345629|emb|CAE03608.2| OSJNBb0004A17.10 [Oryza sativa (japonica cultivar-group)] e_value:2.22662e-107</td><td>11670.m05702|LOC_Os04g57680|protein expressed protein e_value:3.92894e-109</td><td>0.209 (0.0950 0.4543)</td><td>0.095</td><td>0.4543</td></tr>
<tr><td>1679</td><td>WT006_B18_CLUSTER_17143</td><td>gi|50399951|gb|AAT76339.1| putative DNA helicase [Oryza sativa (japonica cultivar-group)] e_value:0</td><td>11669.m03854|LOC_Os03g38990|protein putative DNA helicase e_value:0</td><td>0.095 (0.0434 0.4568)</td><td>0.0434</td><td>0.4568</td></tr>
<tr><td>1680</td><td>WT009_H10_CLUSTER_34438</td><td>gi|50941249|ref|XP_480152.1| putative transaldolase ToTAL2 [Oryza sativa (japonica cultivar-group)] >gnl|BL_ORD_ID|1601953 gi|27817922|dbj|BAC55687.1| putative transaldolase ToTAL2 [Oryza sativa (japonica cultivar-group)] e_value:0</td><td>11674.m00506|LOC_Os08g05830|protein expressed protein e_value:0</td><td>0.095 (0.0662 0.6947)</td><td>0.0662</td><td>0.6947</td></tr>
<tr><td>1681</td><td>WT009_N19_CLUSTER_19936</td><td>gi|32487857|emb|CAE05625.1| OSJNBb0061C13.7 [Oryza sativa (japonica cultivar-group)] e_value:6.33428e-30</td><td>11673.m02489|LOC_Os07g26550|protein hypothetical protein e_value:2.11246e-08</td><td>0.263 (0.4908 1.8640)</td><td>0.4908</td><td>1.864</td></tr>
<tr><td>1682</td><td>WT009_A21_CLUSTER_34225</td><td>gi|34910018|ref|NP_916356.1| P0413G02.10 [Oryza sativa (japonica cultivar-group)] e_value:6.45056e-63</td><td>11673.m04645|LOC_Os07g47030|protein expressed protein e_value:8.24354e-169</td><td>0.522 (0.3746 0.7175)</td><td>0.3746</td><td>0.7175</td></tr>
<tr><td>1683</td><td>WT012_D12_CLUSTER_514</td><td>gi|21397274|gb|AAM51838.1| Unknown protein [Oryza sativa (japonica cultivar-group)] e_value:1.16101e-175</td><td>11669.m00556|LOC_Os03g06070|protein La domain, putative e_value:2.95113e-177</td><td>0.311 (0.1863 0.5995)</td><td>0.1863</td><td>0.5995</td></tr>
<tr><td>1684</td><td>WT006_L16_CLUSTER_31848</td><td>gi|11869988|gb|AAG40578.1| MAP kinase kinase 1 [Oryza sativa] >gnl|BL_ORD_ID|1133371 gi|56201753|dbj|BAD73135.1| putative protein kinase ZmMEK1 [Oryza sativa (japonica cultivar-group)] >gnl|BL_ORD_ID|1133371 gi|56201729|dbj|BAD73553.1| putative prot</td><td>11667.m03079|LOC_Os01g32660|protein MAP kinase kinase 1 e_value:0</td><td>0.064 (0.0292 0.4580)</td><td>0.0292</td><td>0.458</td></tr>
<tr><td>1685</td><td>SET6_M18_CLUSTER_13886</td><td>gi|55770525|ref|XP_550253.1| unknown protein [Oryza sativa (japonica cultivar-group)] >gi|55296863|dbj|BAD68300.1| unknown protein [Oryza sativa (japonica cultivar-group)] e_value:0</td><td>11667.m00662|LOC_Os01g07260|protein hypothetical protein e_value:0</td><td>0.151 (0.0729 0.4820)</td><td>0.0729</td><td>0.482</td></tr>
<tr><td>1686</td><td>WT006_N22_CLUSTER_16463</td><td>gi|50725359|dbj|BAD34431.1| bromodomain protein 103-like [Oryza sativa (japonica cultivar-group)] e_value:0</td><td>11681.m03079|LOC_Os09g33980|protein bromodomain protein 103 e_value:0</td><td>0.152 (0.0717 0.4716)</td><td>0.0717</td><td>0.4716</td></tr>
<tr><td>1687</td><td>SET2_L19_CLUSTER_15966</td><td>gi|19310613|gb|AAL85037.1| putative signal recognition particle protein [Arabidopsis thaliana] >gi|15293137|gb|AAK93679.1| putative signal recognition particle protein [Arabidopsis thaliana] >gi|9758397|dbj|BAB08884.1| signal recognition particle 68</td><td>11674.m02264|LOC_Os08g23360|protein hypothetical protein e_value:0</td><td>0.205 (0.1438 0.7020)</td><td>0.1438</td><td>0.702</td></tr>
<tr><td>1688</td><td>WT009_I05_CLUSTER_7808</td><td>gi|38346640|emb|CAD40738.2| OSJNBa0072D21.10 [Oryza sativa (japonica cultivar-group)] >gnl|BL_ORD_ID|2018366 gi|50923773|ref|XP_472247.1| OSJNBa0072D21.10 [Oryza sativa (japonica cultivar-group)] e_value:0</td><td>11670.m02985|LOC_Os04g31340|protein AKIN gamma e_value:0</td><td>0.125 (0.0519 0.4163)</td><td>0.0519</td><td>0.4163</td></tr>
<tr><td>1689</td><td>WT009_M03_CLUSTER_22377</td><td>gi|42733522|dbj|BAD11358.1| BRI1-KD interacting protein 130 [Oryza sativa (japonica cultivar-group)] e_value:1.2542e-76</td><td>11682.m00147|LOC_Os05g02260|protein expressed protein e_value:2.76431e-78</td><td>0.334 (0.2506 0.7512)</td><td>0.2506</td><td>0.7512</td></tr>
<tr><td>1690</td><td>SET5_B15_CLUSTER_5319</td><td>gi|50929563|ref|XP_474309.1| OSJNBb0004A17.7 [Oryza sativa (japonica cultivar-group)] >gi|38345627|emb|CAE03605.2| OSJNBb0004A17.7 [Oryza sativa (japonica cultivar-group)] e_value:0</td><td>11670.m05698|LOC_Os04g57640|protein expressed protein e_value:0</td><td>0.427 (0.2480 0.5808)</td><td>0.248</td><td>0.5808</td></tr>
<tr><td>1691</td><td>WT007_F09_CLUSTER_30858</td><td>gi|34901686|ref|NP_912189.1| carboxypeptidase C cbp31 [Oryza sativa (japonica cultivar-group)] >gnl|BL_ORD_ID|1526787 gi|50509558|dbj|BAD31260.1| carboxypeptidase C cbp31 [Oryza sativa (japonica cultivar-group)] >gnl|BL_ORD_ID|1526787 gi|27260997|db</td><td>11673.m02808|LOC_Os07g29620|protein Serine carboxypeptidase, putative e_value:0</td><td>0.227 (0.1095 0.4833)</td><td>0.1095</td><td>0.4833</td></tr>
<tr><td>1692</td><td>WT003_C10_CLUSTER_30858</td><td>gi|34901686|ref|NP_912189.1| carboxypeptidase C cbp31 [Oryza sativa (japonica cultivar-group)] >gnl|BL_ORD_ID|1526787 gi|50509558|dbj|BAD31260.1| carboxypeptidase C cbp31 [Oryza sativa (japonica cultivar-group)] >gnl|BL_ORD_ID|1526787 gi|27260997|db</td><td>11673.m02808|LOC_Os07g29620|protein Serine carboxypeptidase, putative e_value:0</td><td>0.227 (0.1156 0.5095)</td><td>0.1156</td><td>0.5095</td></tr>
<tr><td>1693</td><td>SET2_O17_CLUSTER_14444</td><td>gi|51535200|dbj|BAD38249.1| hydroxyproline-rich glycoprotein family protein-like [Oryza sativa (japonica cultivar-group)] >gi|50253286|dbj|BAD29556.1| hydroxyproline-rich glycoprotein family protein-like [Oryza sativa (japonica cultivar-group)] e_value</td><td>11681.m00693|LOC_Os09g08680|protein expressed protein e_value:0</td><td>0.396 (0.2306 0.5830)</td><td>0.2306</td><td>0.583</td></tr>
<tr><td>1694</td><td>WT007_B22_CLUSTER_23610</td><td>gi|50251419|dbj|BAD28457.1| unknown protein [Oryza sativa (japonica cultivar-group)] e_value:5.3673e-27</td><td>11668.m05821|LOC_Os02g58670|protein bZIP transcription factor, putative e_value:7.03341e-29</td><td>0.472 (0.2937 0.6218)</td><td>0.2937</td><td>0.6218</td></tr>
<tr><td>1695</td><td>SET5_M02_CLUSTER_17312</td><td>gi|42541823|gb|AAS19619.1| LON1 protease [Triticum aestivum] e_value:0</td><td>11681.m03224|LOC_Os09g36300|protein ATP-dependent protease La e_value:0</td><td>0.064 (0.0294 0.4567)</td><td>0.0294</td><td>0.4567</td></tr>
<tr><td>1696</td><td>WT002_L13_CLUSTER_32620</td><td>gi|31432309|gb|AAP53959.1| putative splicing factor-like protein [Oryza sativa (japonica cultivar-group)] >gnl|BL_ORD_ID|2011487 gi|37534740|ref|NP_921672.1| putative splicing factor-like protein [Oryza sativa (japonica cultivar-group)] e_value:0</td><td>11669.m00531|LOC_Os03g05830|protein transposon protein, putative, CACTA, En/Spm sub-class e_value:0</td><td>0.218 (0.2328 1.0690)</td><td>0.2328</td><td>1.069</td></tr>
<tr><td>1697</td><td>WT005_E17_CLUSTER_33753</td><td>gi|34907582|ref|NP_915138.1| putative fructokinase I [Oryza sativa (japonica cultivar-group)] >gnl|BL_ORD_ID|1559287 gi|20161284|dbj|BAB90210.1| putative fructokinase [Oryza sativa (japonica cultivar-group)] >gnl|BL_ORD_ID|1559287 gi|16566707|gb|AAL</td><td>11667.m06733|LOC_Os01g66940|protein kinase, pfkB family e_value:1.14098e-162</td><td>0.157 (0.0572 0.3643)</td><td>0.0572</td><td>0.3643</td></tr>
<tr><td>1698</td><td>WT007_A11_CLUSTER_28087</td><td>gi|50912573|ref|XP_467694.1| putative Avr9/Cf-9 rapidly elicited protein 231 [Oryza sativa (japonica cultivar-group)] >gnl|BL_ORD_ID|1582804 gi|46390559|dbj|BAD16045.1| putative Avr9/Cf-9 rapidly elicited protein 231 [Oryza sativa (japonica cultivar-gr</td><td>11668.m04929|LOC_Os02g50600|protein Glycosyl transferase family 8, putative e_value:0</td><td>0.182 (0.0760 0.4172)</td><td>0.076</td><td>0.4172</td></tr>
<tr><td>1699</td><td>SET4_H05_CLUSTER_17797</td><td>gi|34913144|ref|NP_917919.1| putative receptor-like protein kinase [Oryza sativa (japonica cultivar-group)] >gi|22093779|dbj|BAC07070.1| putative receptor-like protein kinase [Oryza sativa (japonica cultivar-group)] e_value:0</td><td>11673.m04253|LOC_Os07g43350|protein Leucine Rich Repeat, putative e_value:0</td><td>0.148 (0.0735 0.4964)</td><td>0.0735</td><td>0.4964</td></tr>
<tr><td>1700</td><td>WT002_H23_CLUSTER_29211</td><td>gi|6175164|gb|AAF04890.1| putative tryptophanyl-tRNA synthetase [Arabidopsis thaliana] >gnl|BL_ORD_ID|1558098 gi|21436361|gb|AAM51350.1| putative tryptophanyl-tRNA synthetase [Arabidopsis thaliana] >gnl|BL_ORD_ID|1558098 gi|19310593|gb|AAL85027.1| p</td><td>11686.m03483|LOC_Os12g35570|protein tryptophanyl-tRNA synthetase, putative e_value:0</td><td>0.103 (0.0486 0.4700)</td><td>0.0486</td><td>0.47</td></tr>
<tr><td>1701</td><td>WT002_I08_CLUSTER_26505</td><td>gi|50944099|ref|XP_481577.1| putative heterogeneous nuclear ribonucleoprotein A3 homolog 1 (hnRNP A3(A)) [Oryza sativa (japonica cultivar-group)] >gnl|BL_ORD_ID|1605229 gi|42409159|dbj|BAD10426.1| RNA recognition motif (RRM)-containing protein-like [Or</td><td>11674.m02239|LOC_Os08g23120|protein RNA recognition motif. (a.k.a. RRM, RBD, or RNP domain), putative e_value:3.56303e-</td><td>0.29 (0.1163 0.4005)</td><td>0.1163</td><td>0.4005</td></tr>
<tr><td>1702</td><td>SET5_A06_CLUSTER_15097</td><td>gi|57899979|dbj|BAD87915.1| putative kinesin heavy chain [Oryza sativa (japonica cultivar-group)] >gi|57899771|dbj|BAD87516.1| putative kinesin heavy chain [Oryza sativa (japonica cultivar-group)] e_value:0</td><td>11667.m05321|LOC_Os01g54080|protein Kinesin motor domain, putative e_value:0</td><td>0.22 (0.1208 0.5483)</td><td>0.1208</td><td>0.5483</td></tr>
<tr><td>1703</td><td>SET3_B12_CLUSTER_32289</td><td>gi|22652528|gb|AAN03740.1| NBS-LRR-like protein [Oryza sativa (japonica cultivar-group)] e_value:0</td><td>11687.m01146|LOC_Os11g11960|protein NBS-LRR disease resistance protein homologue e_value:0</td><td>0.224 (0.1820 0.8132)</td><td>0.182</td><td>0.8132</td></tr>
</table>

PDF Complete
Your complimentary use period has ended. Thank you for using PDF Complete.
Click Here to upgrade to Unlimited Pages and Expanded Features

1704	WT012_D11_CLUSTER_21514	gi|50919269|ref|XP_470031.1| unknown protein [Oryza sativa (japonica cultivar-group)] >gnl|BL_ORD_ID|1588233 gi|30103017|gb|AAP21430.1| unknown protein [Oryza sativa (japonica cultivar-group)] e_value:3.93777e-99	11669.m06016|LOC_Os03g59250|protein expressed protein e_value:4.75536e-101	0.068	(0.0700 1.0247)	0.07	1.0247
1705	WT006_H13_CLUSTER_33264	gi|50920231|ref|XP_470476.1| putative protein kinase AKINbetagamma-2 [Oryza sativa (japonica cultivar-group)] >gnl|BL_ORD_ID|1535470 gi|30102976|gb|AAP21389.1| putative protein kinase AKINbetagamma [Oryza sativa (japonica cultivar-group)]	11669.m06565|LOC_Os03g63940|protein putative protein kinase AKINbetagamma-2 e_value:0	0.095	(0.0698 0.7379)	0.0698	0.7379
1706	WT008_N05_CLUSTER_13087	gi|49343276|gb|AAT64946.1| U1 snRNP [Zea mays] >gnl|BL_ORD_ID|834653 gi|49343272|gb|AAT64945.1| U1 snRNP [Zea mays] e_value:0	11676.m00170|LOC_Os10g02630|protein transposon protein, putative, CACTA, En/Spm sub-class e_value:0	0.259	(0.1700 0.6554)	0.17	0.6554
1707	WT008_H10_CLUSTER_2736	gi|53792086|dbj|BAD54671.1| putative C2H2 zinc-finger protein [Oryza sativa (japonica cultivar-group)] e_value:0	11680.m04805|LOC_Os06g48530|protein expressed protein	0.165	(0.0931 0.5640)	0.0931	0.564
1708	WT010_K15_CLUSTER_31472	gi|10177688|dbj|BAB11014.1| unnamed protein product [Arabidopsis thaliana] >gnl|BL_ORD_ID|1093936 gi|18421863|ref|NP_568564.1| expressed protein [Arabidopsis thaliana] >gnl|BL_ORD_ID|1093936 gi|4099092|gb|AAD09232.1| unknown [Arabidopsis thaliana] e	11673.m03968|LOC_Os07g40620|protein expressed protein e_value:0	0.242	(0.1081 0.4468)	0.1081	0.4468
1709	WT009_O02_CLUSTER_34223	gi|50909807|ref|XP_466392.1| putative non-cell-autonomous protein pathway2; plasmodesmal receptor [Oryza sativa (japonica cultivar-group)] >gnl|BL_ORD_ID|1576953 gi|50725828|dbj|BAD33358.1| putative non-cell-autonomous protein pathway2; plasmodesmal re	11668.m03484|LOC_Os02g36600|protein plasmodesmal receptor e_value:7.59016e-137	0.351	(0.2302 0.6563)	0.2302	0.6563
1710	WT011_D02_CLUSTER_18158	gi|50934253|ref|XP_476654.1| putative MAGE [Oryza sativa (japonica cultivar-group)] >gnl|BL_ORD_ID|1521575 gi|33146706|dbj|BAC79510.1| putative MAGE [Oryza sativa (japonica cultivar-group)] >gnl|BL_ORD_ID|1521575 gi|50509782|dbj|BAD31908.1| putative	11673.m00482|LOC_Os07g05650|protein expressed protein e_value:2.61984e-109	0.15	(0.0788 0.5256)	0.0788	0.5256
1711	WT007_N19_CLUSTER_30593	gi|50929781|ref|XP_474418.1| OSJNBa0088H09.14 [Oryza sativa (japonica cultivar-group)] >gnl|BL_ORD_ID|1594602 gi|32488713|emb|CAE03456.1| OSJNBa0088H09.14 [Oryza sativa (japonica cultivar-group)] e_value:0	11670.m05829|LOC_Os04g58840|protein Eukaryotic aspartyl protease e_value:0	0.53	(0.2221 0.4190)	0.2221	0.419
1712	WT007_J13_CLUSTER_1792	gi|50915728|ref|XP_468328.1| peroxisomal membrane protein-like [Oryza sativa (japonica cultivar-group)] >gnl|BL_ORD_ID|1583955 gi|47497094|dbj|BAD19145.1| peroxisomal membrane protein-like [Oryza sativa (japonica cultivar-group)] e_value:0	11668.m05580|LOC_Os02g56520|protein expressed protein e_value:0	0.232	(0.1472 0.6335)	0.1472	0.6335
1713	WT007_F05_CLUSTER_21167	gi|50872452|gb|AAT85052.1| expressed protein [Oryza sativa (japonica cultivar-group)] e_value:2.73693e-176	11669.m05649|LOC_Os03g56070|protein expressed protein e_value:3.79204e-142	0.42	(0.2550 0.6076)	0.255	0.6076
1714	WT006_A16_CLUSTER_8097	gi|27476088|gb|AAO17019.1| Hypothetical protein [Oryza sativa (japonica cultivar-group)] e_value:0	11669.m00151|LOC_Os03g02290|protein Kinesin motor domain, putative e_value:0	0.206	(0.1811 0.8779)	0.1811	0.8779
1715	WT004_E07_CLUSTER_17207	gi|50252211|dbj|BAD28219.1| putative aminopropyl transferase [Oryza sativa (japonica cultivar-group)] >gnl|BL_ORD_ID|229159 gi|50251663|dbj|BAD29687.1| putative aminopropyl transferase [Oryza sativa (japonica cultivar-group)] e_value:0	11668.m01458|LOC_Os02g15550|protein spermidine synthase, putative e_value:0	0.129	(0.0712 0.5539)	0.0712	0.5539
1716	WT003_K07_CLUSTER_14512	gi|50915982|ref|XP_468455.1| KH domain-containing protein NOVA-like [Oryza sativa (japonica cultivar-group)] >gnl|BL_ORD_ID|1583507 gi|48716278|dbj|BAD22893.1| KH domain-containing protein NOVA-like [Oryza sativa (japonica cultivar-group)] >gnl|BL_O	11668.m05711|LOC_Os02g57640|protein KH domain, putative e_value:1.00697e-49	0.166	(0.5000 3.0150)	0.5	3.015
1717	WT003_H20_CLUSTER_18296	gi|50931663|ref|XP_475359.1| unknown protein [Oryza sativa (japonica cultivar-group)] >gnl|BL_ORD_ID|1595756 gi|48843782|gb|AAT47041.1| unknown protein [Oryza sativa (japonica cultivar-group)] e_value:0	11682.m03579|LOC_Os05g38000|protein expressed protein e_value:0	0.414	(0.1348 0.3253)	0.1348	0.3253
1718	WT003_E03_CLUSTER_3494	gi|50918745|ref|XP_469769.1| putative transcriptional adaptor [Oryza sativa (japonica cultivar-group)] >gnl|BL_ORD_ID|1586215 gi|40538991|gb|AAR87248.1| putative transcriptional adaptor [Oryza sativa (japonica cultivar-group)] e_value:0	11669.m05470|LOC_Os03g53960|protein putative transcriptional adaptor e_value:0	0.122	(0.0555 0.4535)	0.0555	0.4535
1719	WT002_P07_CLUSTER_9414	gi|9755775|emb|CAC01895.1| glutamine-rich protein [Arabidopsis thaliana] >gnl|BL_ORD_ID|1487654 gi|20259119|gb|AAM14275.1| putative glutamine-rich protein [Arabidopsis thaliana] >gnl|BL_ORD_ID|1487654 gi|17979157|gb|AAL49774.1| putative glutamine-ri	11686.m02708|LOC_Os12g27990|protein Similar to glutamine-rich protein - Arabidopsis thaliana e_value:5.37764e-145	0.156	(0.1155 0.7381)	0.1155	0.7381
1720	WT002_D17_CLUSTER_23881	gi|34911048|ref|NP_916871.1| P0007F06.19 [Oryza sativa (japonica cultivar-group)] >gnl|BL_ORD_ID|1545357 gi|21644618|dbj|BAC01177.1| hypothetical protein [Oryza sativa (japonica cultivar-group)] >gnl|BL_ORD_ID|1545357	11667.m04678|LOC_Os01g48250|protein F-box domain, putative e_value:4.47305e-114	0.433	(0.3009 0.6944)	0.3009	0.6944
1721	WT002__C02_CLUSTER_27825	gi|50948157|ref|XP_483606.1| unknown protein [Oryza sativa (japonica cultivar-group)] >gnl|BL_ORD_ID|1610572 gi|42407848|dbj|BAD08991.1| unknown protein [Oryza sativa (japonica cultivar-group)] >gnl|BL_ORD_ID|1610572 gi|42408545|dbj|BAD09723.1| unkn	11674.m04346|LOC_Os08g43060|protein expressed protein e_value:0	0.277	(0.1438 0.5189)	0.1438	0.5189
1722	SET6_I18_CLUSTER_187	gi|30696986|ref|NP_200608.2| phosphatase-related [Arabidopsis thaliana] e_value:3.01493e-81	11674.m01757|LOC_Os08g17870|protein Reticulon, putative e_value:0	0.312	(0.1891 0.6066)	0.1891	0.6066
1723	SET5_P05_CLUSTER_26136	gi|50907049|ref|XP_465013.1| hypothetical protein [Oryza sativa (japonica cultivar-group)] >gi|47847939|dbj|BAD21729.1| hypothetical protein [Oryza sativa (japonica cultivar-group)] e_value:2.71286e-66	11668.m01782|LOC_Os02g18720|protein hypothetical protein e_value:6.8747e-68	0.518	(0.5692 1.0992)	0.5692	1.0992
1724	SET5_D10_CLUSTER_25248	gi|34897624|ref|NP_910158.1| hypothetical protein [Oryza sativa] >gi|54291848|gb|AAV32216.1| unknown protein [Oryza sativa (japonica cultivar-group)] e_value:2.20228e-60	11682.m00578|LOC_Os05g06290|protein expressed protein e_value:2.75431e-62	0.201	(0.0836 0.4158)	0.0836	0.4158
1725	SET4_J03_CLUSTER_21471	gi|57900683|gb|AAW57808.1| putative MAR binding protein [Oryza sativa (japonica cultivar-group)] e_value:0	11682.m00851|LOC_Os05g08790|protein hypothetical protein e_value:0	0.499	(0.2479 0.4969)	0.2479	0.4969
1726	SET3_N03_CLUSTER_15230	gi|24414275|gb|AAN59778.1| Unknown protein [Oryza sativa (japonica cultivar-group)] >gi|50920429|ref|XP_470575.1| Unknown protein [Oryza sativa (japonica cultivar-group)] e_value:1.40731e-144	11669.m00924|LOC_Os03g09840|protein C2 domain, putative e_value:3.44007e-146	0.246	(0.1849 0.7516)	0.1849	0.7516
1727	SET3_M17_CLUSTER_19808	gi|54290291|dbj|BAD61236.1| ubiquitin-activating enzyme E1-like [Oryza sativa (japonica cultivar-group)] e_value:0	11667.m04141|LOC_Os01g42850|protein E1-like protein-activating enzyme Gsa7p/Apg7p, putative e_value:0	0.36	(0.1611 0.4471)	0.1611	0.4471
1728	SET3_K21_CLUSTER_29298	gi|50927639|ref|XP_473415.1| OSJNBb0034G17.6 [Oryza sativa (japonica cultivar-group)] >gi|38605848|emb|CAD41604.3| OSJNBb0034G17.6 [Oryza sativa (japonica cultivar-group)] >gi|32455220|gb|AAP83324.1| transcription factor [Oryza sativa (japonica cult	11670.m04522|LOC_Os04g46440|protein Similar to apetala2 domain TINY like protein [imported] - Arabidopsis thaliana e_value:2.95787e-85	0.373	(0.1339 0.3590)	0.1339	0.359
1729	SET3_I20_CLUSTER_24212	gi|40538974|gb|AAR87231.1| hypothetical protein [Oryza sativa (japonica cultivar-group)] >gi|50901354|ref|XP_463110.1| hypothetical protein [Oryza sativa (japonica cultivar-group)] e_value:0	11669.m04053|LOC_Os03g40930|protein hypothetical protein e_value:0	0.112	(0.0583 0.5183)	0.0583	0.5183
1730	SET3_G16_CLUSTER_15092	gi|50926290|ref|XP_473092.1| OSJNBb0116K07.16 [Oryza sativa (japonica cultivar-group)] >gi|21743072|emb|CAD41177.1| OSJNBb0002J11.1 [Oryza sativa (japonica cultivar-group)] >gi|32490274|emb|CAE05563.1| OSJNBb0116K07.16 [Oryza sativa (japonica cultiv	11670.m04117|LOC_Os04g42670|protein F-box domain, putative e_value:0	0.141	(0.0940 0.6664)	0.094	0.6664
1731	SET3_B13_CLUSTER_2170	gi|50921221|ref|XP_470971.1| OSJNBa0094O15.14 [Oryza sativa (japonica cultivar-group)] >gi|38346207|emb|CAD39345.2| OSJNBa0094O15.14 [Oryza sativa (japonica cultivar-group)] e_value:0	11670.m00048|LOC_Os04g01480|protein Zinc finger C-x8-C-x5-C-x3-H type (and similar), putative e_value:0	0.349	(0.1931 0.5528)	0.1931	0.5528
1732	SET2__C12_CLUSTER_26836	gi|15240310|ref|NP_198005.1| plastocyanin-like domain-containing protein / mavicyanin, putative [Arabidopsis thaliana] >gi|3319353|gb|AAC26242.1| contains similarity to copper-binding proteins [Arabidopsis thaliana] >gi|45752728|gb|AAS76262.1| At5g2	11673.m03720|LOC_Os07g38290|protein Plastocyanin-like domain, putative e_value:1.52912e-60	0.502	(0.2170 0.4325)	0.217	0.4325
1733	SET1_I24_CLUSTER_7091	gi|31126737|gb|AAP44659.1| putative beta 1,3-glucanase [Oryza sativa (japonica cultivar-group)] >gi|50917635|ref|XP_469214.1| putative beta 1,3-glucanase [Oryza sativa (japonica cultivar-group)] e_value:0	11669.m04666|LOC_Os03g46660|protein putative beta 1,3-glucanase e_value:0	0.337	(0.1228 0.3639)	0.1228	0.3639
1734	SET1_D08_CLUSTER_10348	gi|52077319|dbj|BAD46360.1| unknown protein [Oryza sativa (japonica cultivar-group)] e_value:1.145e-132	11681.m03171|LOC_Os09g35810|protein NC domain, putative e_value:2.9071e-134	0.143	(0.0783 0.5490)	0.0783	0.549
1735	WT013_N16_CLUSTER_23127	gi|57899483|dbj|BAD86944.1| putative PTEN-like phosphatase [Oryza sativa (japonica cultivar-group)] e_value:3.05296e-71	11680.m00522|LOC_Os06g05870|protein hypothetical protein e_value:7.66136e-98	0.308	(0.1368 0.4449)	0.1368	0.4449
1736	WT012_O11_CLUSTER_29779	gi|56381951|gb|AAV85694.1| At3g02490 [Arabidopsis thaliana] >gnl|BL_ORD_ID|1828409 gi|6957712|gb|AAF32456.1| unknown protein [Arabidopsis thaliana] >gnl|BL_ORD_ID|1828409 gi|51536500|gb|AAU05488.1| At3g02490 [Arabidopsis thaliana] >gnl|BL_ORD_ID|	11667.m07433|LOC_Os01g73950|protein hypothetical protein e_value:5.39118e-77	0.549	(0.7485 1.3635)	0.7485	1.3635
1737	WT012_N01_CLUSTER_18598	gi|53792530|dbj|BAD53494.1| putative Synoviolin 1 [Oryza sativa (japonica cultivar-group)] >gnl|BL_ORD_ID|542431 gi|53792858|dbj|BAD53976.1| putative Synoviolin 1 [Oryza sativa (japonica cultivar-group)] e_value:0	11680.m01914|LOC_Os06g19680|protein zinc finger protein, putative e_value:0	0.176	(0.0643 0.3655)	0.0643	0.3655
1738	WT012_G02_CLUSTER_1382	gi|34896276|ref|NP_909482.1| hypothetical protein [Oryza sativa] >gnl|BL_ORD_ID|1511052 gi|12039344|gb|AAG46131.1| hypothetical protein [Oryza sativa] e_value:2.74548e-123	11669.m02577|LOC_Os03g25430|protein hypothetical protein e_value:5.75441e-123	0.377	(0.1724 0.4578)	0.1724	0.4578
1739	WT012_F21_CLUSTER_23024	gi|57900136|dbj|BAD88198.1| systemin receptor-like [Oryza sativa (japonica cultivar-group)] e_value:1.8912e-169	11667.m07037|LOC_Os01g70260|protein Protein kinase domain, putative e_value:4.39233e-171	0.211	(0.1555 0.7383)	0.1555	0.7383
1740	WT011_D06_CLUSTER_13868	gi|50947495|ref|XP_483275.1| putative ethylene-insensitive protein (EIL) [Oryza sativa (japonica cultivar-group)] >gnl|BL_ORD_ID|1609562 gi|42409349|dbj|BAD10664.1| putative ethylene-insensitive protein (EIL) [Oryza sativa (japonica cultivar-	11681.m02904|LOC_Os09g31400|protein Ethylene insensitive 3, putative e_value:0	0.245	(0.1250 0.5091)	0.125	0.5091
1741	WT011__C08_CLUSTER_27942	gi|50936273|ref|XP_477664.1| rac GTPase activating protein 3 -like protein [Oryza sativa (japonica cultivar-group)] >gnl|BL_ORD_ID|1527543 gi|33354208|dbj|BAC81174.1| rac GTPase activating protein 3 -like protein [Oryza sativa (japonica cultivar-	11673.m02080|LOC_Os07g22580|protein RhoGAP domain, putative e_value:1.48537e-170	0.314	(0.2127 0.6776)	0.2127	0.6776
1742	WT009_L10_CLUSTER_12798	gi|22165052|gb|AAM93669.1| putative leaf protein [Oryza sativa (japonica cultivar-group)] >gnl|BL_ORD_ID|2269807 gi|31432900|gb|AAP54476.1| putative leaf protein [Oryza sativa (japonica cultivar-group)] >gnl|BL_ORD_ID|2269807	11676.m03196|LOC_Os10g35750|protein putative leaf protein e_value:3.63868e-172	0.449	(0.2520 0.5612)	0.252	0.5612
1743	WT009_K07_CLUSTER_3253	gi|50929799|ref|XP_474427.1| OSJNBa0070M12.5 [Oryza sativa (japonica cultivar-group)] >gnl|BL_ORD_ID|1594614 gi|38345822|emb|CAD41927.2| OSJNBa0070M12.5 [Oryza sativa (japonica cultivar-group)] e_value:0	11670.m05841|LOC_Os04g58960|protein Regulator of chromosome condensation (RCC1), putative e_value:0	0.146	(0.0819 0.5604)	0.0819	0.5604
1744	WT009_I13_CLUSTER_27458	gi|50931861|ref|XP_475458.1| 'unknown protein, contains f-box domain' [Oryza sativa (japonica cultivar-group)] >gnl|BL_ORD_ID|1596938 gi|50080303|gb|AAT69637.1| 'unknown protein, contains f-box domain' [Oryza sativa (japonica cultiv	11682.m03933|LOC_Os05g41130|protein F-box domain, putative e_value:0	0.078	(0.0543 0.6920)	0.0543	0.692
1745	WT009_D13_CLUSTER_16053	No hits found	11674.m03187|LOC_Os08g32330|protein expressed protein e_value:7.43484e-10	0.68	(0.7952 1.1701)	0.7952	1.1701
1746	WT008_O19_CLUSTER_13868	gi|50947495|ref|XP_483275.1| putative ethylene-insensitive protein (EIL) [Oryza sativa (japonica cultivar-group)] >gnl|BL_ORD_ID|1609562 gi|42409349|dbj|BAD10664.1| putative ethylene-insensitive protein (EIL) [Oryza sativa (japonica cultivar-	11681.m02904|LOC_Os09g31400|protein Ethylene insensitive 3, putative e_value:0	0.245	(0.1250 0.5091)	0.125	0.5091

PDF Complete
Your complimentary use period has ended. Thank you for using PDF Complete.
Click Here to upgrade to Unlimited Pages and Expanded Features

1747	WT002_L03_CLUSTER_24140	gi|40644782|emb|CAE53899.1| putative transcription factor MADS32 [Triticum aestivum] e_value:1.12669e-95	11667.m05169|LOC_Os01g52680|protein SRF-type transcription factor (DNA-binding and dimerisation domain), putative e_value:1.42429e-96	0.174	(0.0628 0.3609)	0.0628	0.3609
1748	WT013_H08_CLUSTER_31577	gi|4886754|gb|AAD32030.1| RAD51 homolog RAD51B [Zea mays] >gnl|BL_ORD_ID|2037541 gi|55976630|sp|Q9XED7|R512_MAIZE DNA repair protein RAD51 homolog B (Rad51-like protein B) (RAD51B) (ZmRAD51b) e_value:8.23302e-177	11687.m03743|LOC_Os11g40150|protein DNA repair protein RAD51 e_value:2.04927e-173	0.072	(0.0485 0.6784)	0.0485	0.6784
1749	WT006_K19_CLUSTER_23784	gi|56381953|gb|AAV85695.1| At4g28600 [Arabidopsis thaliana] >gnl|BL_ORD_ID|1828410 gi|51536508|gb|AAU05492.1| At4g28600 [Arabidopsis thaliana] >gnl|BL_ORD_ID|1828410 gi|22329002|ref|NP_194589.2| calmodulin-binding protein [Arabidopsis thaliana] e_va	11676.m02929|LOC_Os10g33290|protein New cDNA-based Gene e_value:0	0.142	(0.0754 0.5295)	0.0754	0.5295
1750	WT004_E05_CLUSTER_16665	gi|31339097|dbj|BAC77155.1| GL2-type homeodomain protein [Oryza sativa (japonica cultivar-group)] e_value:0	11674.m00822|LOC_Os08g08820|protein Roc1 e_value:0	0.224	(0.1325 0.5926)	0.1325	0.5926
1751	WT011_J13_CLUSTER_7995	gi|50921079|ref|XP_470900.1| putative RNA polymerase III [Oryza sativa (japonica cultivar-group)] >gnl|BL_ORD_ID|1588877 gi|29788820|gb|AAP03366.1| putative RNA polymerase III [Oryza sativa (japonica cultivar-group)] e_value:0	11669.m02910|LOC_Os03g28960|protein putative RNA polymerase III e_value:0	0.137	(0.0708 0.5179)	0.0708	0.5179
1752	WT012_K08_CLUSTER_4208	gi|50725904|dbj|BAD33432.1| S-adenosylmethionine decarboxylase [Oryza sativa (japonica cultivar-group)] >gnl|BL_ORD_ID|235311 gi|49532747|dbj|BAD26704.1| S-adenosylmethionine decarboxylase [Oryza sativa (japonica cultivar-group)] e_value:0	11681.m02327|LOC_Os09g25620|protein Adenosylmethionine decarboxylase e_value:0	0.119	(0.1062 0.8936)	0.1062	0.8936
1753	WT009_L17_CLUSTER_3933	gi|50905465|ref|XP_464221.1| putative auxin response transcription factor(ARF6) [Oryza sativa (japonica cultivar-group)] >gnl|BL_ORD_ID|1566469 gi|51963884|ref|XP_506725.1| PREDICTED OJ1661_C12.26 gene product [Oryza sativa (japonica cultivar-group)] &	11668.m00642|LOC_Os02g06910|protein auxin response factor 3 e_value:0	0.14	(0.0773 0.5518)	0.0773	0.5518
1754	WT006_L22_CLUSTER_25217	gi|4581155|gb|AAD24639.1| putative receptor-like protein kinase [Arabidopsis thaliana] >gnl|BL_ORD_ID|940574 gi|25408487|pir||B84782 probable receptor-like protein kinase [imported] - Arabidopsis thaliana >gnl|BL_ORD_ID|940574 gi|15227998|ref|NP_181	11686.m00446|LOC_Os12g05120|protein Similar to probable receptor-like protein kinase [imported] - Arabidopsis thaliana e_value:0	0.273	(0.1177 0.4316)	0.1177	0.4316
1755	WT003_M08_CLUSTER_8791	gi|50934399|ref|XP_476727.1| putative snRNP(small nuclear ribonucleoprotein associated protein) [Oryza sativa (japonica cultivar-group)] e_value:3.15404e-123	11673.m00645|LOC_Os07g07220|protein Sm protein, putative e_value:7.04577e-125	0.093	(0.0691 0.7446)	0.0691	0.7446
1756	WT010_O13_CLUSTER_34814	gi|57900122|dbj|BAD88184.1| putative zisp [Oryza sativa (japonica cultivar-group)] >gnl|BL_ORD_ID|972476 gi|57899394|dbj|BAD88041.1| putative zisp [Oryza sativa (japonica cultivar-group)] e_value:0	11667.m07020|LOC_Os01g70100|protein DHHC zinc finger domain, putative e_value:0	0.081	(0.0444 0.5467)	0.0444	0.5467
1757	WT006_M13_CLUSTER_537	gi|53793554|dbj|BAD53324.1| putative prolylcarboxypeptidase, isoform 1 [Oryza sativa (japonica cultivar-group)] >gnl|BL_ORD_ID|261274 gi|53792444|dbj|BAD53352.1| putative prolylcarboxypeptidase, isoform 1 [Oryza sativa (japonica cultivar-group)] e_valu	11667.m05549|LOC_Os01g56150|protein lysosomal Pro-X carboxypeptidase e_value:0	0.196	(0.1066 0.5432)	0.1066	0.5432
1758	WT003_O23_CLUSTER_30033	gi|50947269|ref|XP_483162.1| putative phosphatidylinositol- phosphatidylcholine transfer protein [Oryza sativa (japonica cultivar-group)] >gnl|BL_ORD_ID|1609409 gi|42407309|dbj|BAD08712.1| putative phosphatidylinositol- phosphatidylcholine transfer pro	11674.m03877|LOC_Os08g38850|protein CRAL/TRIO, N-terminus, putative e_value:0	0.189	(0.1064 0.5636)	0.1064	0.5636
1759	WT003_L23_CLUSTER_34274	gi|51090844|dbj|BAD35372.1| DNA-binding protein-like [Oryza sativa (japonica cultivar-group)] e_value:0	11680.m04087|LOC_Os06g41730|protein ARID/BRIGHT DNA binding domain, putative e_value:0	0.259	(0.1706 0.6589)	0.1706	0.6589
1760	WT003_I16_CLUSTER_9534	gi|57900503|dbj|BAD88098.1| putative replication licensing factor MCM4 [Oryza sativa (japonica cultivar-group)] e_value:0	11667.m03465|LOC_Os01g36390|protein MCM2/3/5 family, putative e_value:0	0.065	(0.0399 0.6161)	0.0399	0.6161
1761	WT002_E02_CLUSTER_34751	gi|50921193|ref|XP_470957.1| OSJNBa0068L06.10 [Oryza sativa (japonica cultivar-group)] >gnl|BL_ORD_ID|1589050 gi|38344767|emb|CAE01584.2| OSJNBa0068L06.10 [Oryza sativa (japonica cultivar-group)] e_value:0	11670.m00025|LOC_Os04g01250|protein Similar to gene_id:T12B11.3 e_value:0	0.231	(0.1107 0.4782)	0.1107	0.4782
1762	SET3_H15_CLUSTER_18560	gi|34912652|ref|NP_917673.1| putative acetyl transferase [Oryza sativa (japonica cultivar-group)] >gi|11034585|dbj|BAB17109.1| 10-deacetylbaccatin III-10-O-acetyl transferase-like [Oryza sativa (japonica cultivar-group)] e_value:0	11667.m04143|LOC_Os01g42870|protein Transferase family e_value:0	0.235	(0.1091 0.4636)	0.1091	0.4636
1763	WT004_L23_CLUSTER_34640	gi|34902046|ref|NP_912369.1| COG1208, GCD1, Nucleoside-diphosphate-sugar pyrophosphorylase involved in lipopolysaccharide biosynthesis/translation initiation factor 2B, gamma/epsilon subunits (eIF-2Bgamma/eIF-2Bepsilon) [Cell envelope biogenesis, outer me	11669.m01617|LOC_Os03g16150|protein Nucleotidyl transferase, putative e_value:0	0.1	(0.0303 0.3019)	0.0303	0.3019
1764	SET5_H15_CLUSTER_1851	gi|62732989|gb|AAX95108.1| CAA303718.1 protein [Oryza sativa (japonica cultivar-group)] e_value:0	11687.m00577|LOC_Os11g06440|protein CAA303718.1 protein e_value:0	0.232	(0.0924 0.3982)	0.0924	0.3982
1765	WT010_M22_CLUSTER_3299	gi|50929631|ref|XP_474343.1| OSJNBa0064G10.8 [Oryza sativa (japonica cultivar-group)] >gnl|BL_ORD_ID|1594512 gi|32487513|emb|CAE05757.1| OSJNBa0064G10.8 [Oryza sativa (japonica cultivar-group)] e_value:6.18993e-133	11670.m05739|LOC_Os04g58020|protein myb-like DNA-binding domain, SHAQKYF class, putative e_value:1.20288e-134	0.093	(0.0862 0.9235)	0.0862	0.9235
1766	WT002_I07_CLUSTER_11345	gi|51965014|ref|XP_507291.1| PREDICTED OSJNBa0016N23.125 gene product [Oryza sativa (japonica cultivar-group)] >gnl|BL_ORD_ID|1609582 gi|50947535|ref|XP_483295.1| unknown protein [Oryza sativa (japonica cultivar-group)] >gnl|BL_ORD_ID|1609582 gi|284	11674.m04009|LOC_Os08g39990|protein hypothetical protein e_value:5.52155e-132	0.346	(0.1512 0.4374)	0.1512	0.4374
1767	SET4_G24_CLUSTER_7891	gi|21742313|emb|CAD40770.1| OSJNBb0039F02.1 [Oryza sativa (japonica cultivar-group)] >gi|50923931|ref|XP_472326.1| OSJNBb0039F02.1 [Oryza sativa (japonica cultivar-group)] e_value:2.78375e-48	11670.m03104|LOC_Os04g32470|protein expressed protein e_value:1.20862e-48	0.297	(0.2136 0.7195)	0.2136	0.7195
1768	WT009_H24_CLUSTER_32744	gi|34908236|ref|NP_915465.1| putative pumilio/Mpt5 family RNA-binding protein [Oryza sativa (japonica cultivar-group)] >gnl|BL_ORD_ID|1556499 gi|20160612|dbj|BAB89558.1| putative pumilio domain-containing protein PPD1 [Oryza sativa (japonica cultivar-	11667.m06267|LOC_Os01g62650|protein Pumilio-family RNA binding repeat, putative e_value:0	0.222	(0.1021 0.4597)	0.1021	0.4597
1769	WT010_L19_CLUSTER_32236	gi|34907348|ref|NP_915021.1| putative male sterility 1 protein [Oryza sativa (japonica cultivar-group)] >gnl|BL_ORD_ID|1558216 gi|22202666|dbj|BAC07324.1| putative male sterility 1 protein [Oryza sativa (japonica cultivar-group)] e_value:0	11667.m06591|LOC_Os01g65600|protein PHD-finger, putative e_value:0	0.169	(0.0821 0.4847)	0.0821	0.4847
1770	WT002_D23_CLUSTER_13884	gi|50929277|ref|XP_474166.1| OSJNBb0022F16.7 [Oryza sativa (japonica cultivar-group)] >gnl|BL_ORD_ID|1593835 gi|38343968|emb|CAE01552.2| OSJNBb0022F16.7 [Oryza sativa (japonica cultivar-group)] >gnl|BL_ORD_ID|1593835 gi|24636586|dbj|BAC22747.1| JUN-	11670.m05528|LOC_Os04g56070|protein JUN-activation-domain-binding protein homolog - rice e_value:0	0.135	(0.0335 0.2485)	0.0335	0.2485
1771	SET4__C07_CLUSTER_4023	gi|56783783|dbj|BAD81195.1| putative ATP-dependent Clp protease proteolytic subunit [Oryza sativa (japonica cultivar-group)] e_value:2.55548e-116	11667.m01661|LOC_Os01g16530|protein Similar to ATP-dependent Clp protease proteolytic subunit ClpR4, putative e_value:5.49822e-118	0.245	(0.1225 0.4992)	0.1225	0.4992
1772	WT004_K02_CLUSTER_4132	gi|57900172|dbj|BAD88257.1| vacuolar protein sorting 55 family-like [Oryza sativa (japonica cultivar-group)] e_value:5.15316e-54	11667.m07389|LOC_Os01g73570|protein AT3g11530/F24K9_21 e_value:3.0324e-56	0.07	(0.0699 0.9978)	0.0699	0.9978
1773	WT013_N17_CLUSTER_34929	gi|7439979|pir||S71453 glycine-rich RNA-binding protein, low-temperature-responsive - barley >gnl|BL_ORD_ID|193306 gi|1229138|gb|AAB07749.1| low temperature-responsive RNA-binding protein e_value:2.61874e-87	11669.m04679|LOC_Os03g46770|protein retrotransposon protein, putative, Ty1-copia sub-class e_value:1.36961e-66	0.477	(0.1565 0.3284)	0.1565	0.3284
1774	WT011_B22_CLUSTER_34094	gi|34908614|ref|NP_915654.1| P0677H08.7 [Oryza sativa (japonica cultivar-group)] >gnl|BL_ORD_ID|1557625 gi|20160853|dbj|BAB89792.1| membrane protein COV-like [Oryza sativa (japonica cultivar-group)] e_value:1.87437e-140	11667.m06501|LOC_Os01g64780|protein expressed protein e_value:3.61342e-142	0.073	(0.0304 0.4161)	0.0304	0.4161
1775	WT013_J12_CLUSTER_32965	gi|38606518|emb|CAE05993.3| OSJNBa0016O02.3 [Oryza sativa (japonica cultivar-group)] >gnl|BL_ORD_ID|1523954 gi|50924910|ref|XP_472798.1| OSJNBa0016O02.3 [Oryza sativa (japonica cultivar-group)] e_value:3.86379e-171	11670.m03727|LOC_Os04g39210|protein Porphyromonas-type peptidyl-arginine deiminase e_value:0	0.183	(0.0942 0.5132)	0.0942	0.5132
1776	SET1_L09_CLUSTER_32788	gi|50931081|ref|XP_475068.1| putative 26S proteasome regulatory subunit [Oryza sativa (japonica cultivar-group)] >gi|52353598|gb|AAU44164.1| putative 26S proteasome regulatory subunit [Oryza sativa (japonica cultivar-group)] >gi|46359906|gb|AAS88838	11682.m00051|LOC_Os05g01450|protein Mov34/MPN/PAD-1 family, putative e_value:2.36333e-140	0.118	(0.0571 0.4852)	0.0571	0.4852
1777	WT003_M02_CLUSTER_32408	gi|50912419|ref|XP_467617.1| putative nucellin-like aspartic protease [Oryza sativa (japonica cultivar-group)] >gnl|BL_ORD_ID|1580594 gi|51964340|ref|XP_506955.1| PREDICTED OSJNBa0072H09.41 gene product [Oryza sativa (japonica cultivar-group)] >gnl|	11668.m04848|LOC_Os02g49800|protein Eukaryotic aspartyl protease, putative e_value:0	0.151	(0.0835 0.5524)	0.0835	0.5524
1778	WT004_P24_CLUSTER_32837	gi|1296809|emb|CAA64545.1| xylose isomerase [Hordeum vulgare subsp. vulgare] >gnl|BL_ORD_ID|2350469 gi|6175480|sp|Q40082|XYLA_HORVU Xylose isomerase e_value:0	11673.m04677|LOC_Os07g47290|protein xylose isomerase e_value:0	0.081	(0.0515 0.6353)	0.0515	0.6353
1779	WT004_M09_CLUSTER_17141	gi|50909083|ref|XP_466030.1| putative WD40 repeat protein [Oryza sativa (japonica cultivar-group)] >gnl|BL_ORD_ID|1575668 gi|49388269|dbj|BAD25387.1| putative WD40 repeat protein [Oryza sativa (japonica cultivar-group)] e_value:0	11668.m03051|LOC_Os02g32430|protein WD40 repeat protein e_value:0	0.103	(0.0621 0.6047)	0.0621	0.6047
1780	WT007_K14_CLUSTER_33870	gi|50913229|ref|XP_468022.1| putative KOW domain-containing transcription factor [Oryza sativa (japonica cultivar-group)] >gnl|BL_ORD_ID|1583139 gi|46805362|dbj|BAD16863.1| putative KOW domain-containing transcription factor [Oryza sativa (japonica cul	11680.m01030|LOC_Os06g10620|protein KOW motif, putative e_value:0	0.199	(0.1489 0.7493)	0.1489	0.7493
1781	WT005_B03_CLUSTER_32028	gi|38344553|emb|CAD40958.2| OSJNBa0027P08.21 [Oryza sativa (japonica cultivar-group)] >gnl|BL_ORD_ID|1534627 gi|50924594|ref|XP_472656.1| OSJNBa0027P08.21 [Oryza sativa (japonica cultivar-group)] e_value:8.38284e-172	11670.m03525|LOC_Os04g36890|protein peptidyl-prolyl cis-trans isomerase, FKBP-type, putative e_value:2.4675e-173	0.27	(0.1754 0.6504)	0.1754	0.6504
1782	WT002_F15_CLUSTER_30782	gi|50945983|ref|XP_482519.1| putative UVB-resistance protein UVR8 [Oryza sativa (japonica cultivar-group)] >gnl|BL_ORD_ID|1606457 gi|51964906|ref|XP_507237.1| PREDICTED OJ1124_B05.9 gene product [Oryza sativa (japonica cultivar-group)] >gnl|BL_ORD_I	11674.m03303|LOC_Os08g33390|protein UVB-resistance protein-like - Arabidopsis thaliana e_value:0	0.116	(0.0710 0.6135)	0.071	0.6135
1783	WT002_B04_CLUSTER_19034	gi|53793245|dbj|BAD54469.1| putative mlo2 protein [Oryza sativa (japonica cultivar-group)] e_value:0	11680.m03261|LOC_Os06g33810|protein 5730410I19Rik protein e_value:1.03116e-169	0.241	(0.1240 0.5154)	0.124	0.5154
1784	WT002_H14_CLUSTER_26999	gi|34902022|ref|NP_912357.1| unknown protein [Oryza sativa (japonica cultivar-group)] >gnl|BL_ORD_ID|1588784 gi|29893627|gb|AAP06881.1| unknown protein [Oryza sativa (japonica cultivar-group)] e_value:8.83102e-175	11669.m01601|LOC_Os03g16000|protein SH3 domain, putative e_value:4.52618e-180	0.211	(0.1006 0.4779)	0.1006	0.4779
1785	WT013_N10_CLUSTER_4658	gi|55770087|ref|XP_550033.1| unknown protein [Oryza sativa (japonica cultivar-group)] >gnl|BL_ORD_ID|1753669 gi|53792165|dbj|BAD52798.1| unknown protein [Oryza sativa (japonica cultivar-group)] e_value:0	11667.m00337|LOC_Os01g04200|protein At1g25370/F4F7_22 e_value:0	0.294	(0.1516 0.5162)	0.1516	0.5162
1786	WT011_N22_CLUSTER_15168	gi|34912330|ref|NP_917512.1| putative ribosomal RNA apurinic site specific lyase [Oryza sativa (japonica cultivar-group)] >gnl|BL_ORD_ID|1542372 gi|20521316|dbj|BAB91830.1| putative ribosomal RNA apurinic site specific lyase [Oryza sativa (japonica cul	11667.m03974|LOC_Os01g41260|protein F-box domain, putative e_value:0	0.486	(0.2088 0.4295)	0.2088	0.4295
1787	WT011_F24_CLUSTER_26522	gi|50932919|ref|XP_475987.1| unknown protein [Oryza sativa (japonica cultivar-group)] >gnl|BL_ORD_ID|1600111 gi|48475092|gb|AAT44161.1| unknown protein [Oryza sativa (japonica cultivar-group)] e_value:3.41549e-147	11682.m04916|LOC_Os05g51000|protein expressed protein e_value:7.67616e-149	0.154	(0.1123 0.7299)	0.1123	0.7299

PDF Complete
Your complimentary use period has ended. Thank you for using PDF Complete.
Click Here to upgrade to Unlimited Pages and Expanded Features

<table>
<tr><td>1788</td><td>WT009_A15_CLUSTER_4185</td><td>gi|51536072|dbj|BAD38198.1| mitochondrial transcription termination factor-like [Oryza sativa (japonica cultivar-group)] >gnl|BL_ORD_ID|243011 gi|51535002|dbj|BAD37286.1| mitochondrial transcription termination factor-like [Oryza sativa (japonica culti</td><td>11680.m01182|LOC_Os06g12100|protein mTERF, putative e_value:1.23596e-116</td><td>0.533</td><td>(0.3349 0.6277)</td><td>0.3349</td><td>0.6277</td></tr>
<tr><td>1789</td><td>WT005_J11_CLUSTER_32563</td><td>gi|50399950|gb|AAT76338.1| putative Lysyl-tRNA synthetase [Oryza sativa (japonica cultivar-group)] >gnl|BL_ORD_ID|919020 gi|55583785|sp|Q6F2U9|SYK_ORYSA Lysyl-tRNA synthetase (Lysine--tRNA ligase) (LysRS) e_value:0</td><td>11669.m03852|LOC_Os03g38980|protein putative Lysyl-tRNA synthetase e_value:0</td><td>0.174</td><td>(0.0961 0.5510)</td><td>0.0961</td><td>0.551</td></tr>
<tr><td>1790</td><td>WT005_C15_CLUSTER_32729</td><td>gi|55741370|ref|XP_550350.1| unknown protein [Oryza sativa (japonica cultivar-group)] >gnl|BL_ORD_ID|1743542 gi|55296084|dbj|BAD67646.1| unknown protein [Oryza sativa (japonica cultivar-group)] e_value:1.32227e-170</td><td>11680.m00623|LOC_Os06g06770|protein At5g25770 e_value:4.03261e-172</td><td>0.179</td><td>(0.0994 0.5540)</td><td>0.0994</td><td>0.554</td></tr>
<tr><td>1791</td><td>WT003_P21_CLUSTER_25966</td><td>gi|22136272|gb|AAM91214.1| putative WD-40 repeat protein [Arabidopsis thaliana] >gnl|BL_ORD_ID|1122593 gi|4191784|gb|AAD10153.1| putative WD-40 repeat protein [Arabidopsis thaliana] >gnl|BL_ORD_ID|1122593 gi|13877611|gb|AAK43883.1| putative WD-40 re</td><td>11687.m00310|LOC_Os11g03990|protein probable WD-40 repeat protein [imported] - Arabidopsis thaliana e_value:0</td><td>0.15</td><td>(0.0896 0.5988)</td><td>0.0896</td><td>0.5988</td></tr>
<tr><td>1792</td><td>WT002_O06_CLUSTER_34309</td><td>gi|22136914|gb|AAM91801.1| unknown protein [Arabidopsis thaliana] >gnl|BL_ORD_ID|1208994 gi|13877883|gb|AAK44019.1| unknown protein [Arabidopsis thaliana] >gnl|BL_ORD_ID|1208994 gi|22327037|ref|NP_197857.2| expressed protein [Arabidopsis thaliana] &</td><td>11686.m01847|LOC_Os12g18630|protein hypothetical protein e_value:0</td><td>0.157</td><td>(0.0952 0.6056)</td><td>0.0952</td><td>0.6056</td></tr>
<tr><td>1793</td><td>WT003_L20_CLUSTER_31000</td><td>gi|34903626|ref|NP_913160.1| lysophospholipase - like [Oryza sativa (japonica cultivar-group)] >gnl|BL_ORD_ID|1751486 gi|20160469|dbj|BAB89422.1| monoglyceride lipase isoform 2 -like [Oryza sativa (japonica cultivar-group)] e_value:0</td><td>11667.m00987|LOC_Os01g10250|protein hydrolase, alpha/beta fold family, putative e_value:0</td><td>0.152</td><td>(0.0912 0.5987)</td><td>0.0912</td><td>0.5987</td></tr>
<tr><td>1794</td><td>WT013_A14_CLUSTER_34578</td><td>gi|31433151|gb|AAP54704.1| putative phospholipase [Oryza sativa (japonica cultivar-group)] >gnl|BL_ORD_ID|2012735 gi|37536230|ref|NP_922417.1| putative phospholipase [Oryza sativa (japonica cultivar-group)] >gnl|BL_ORD_ID|2012735</td><td>11676.m03449|LOC_Os10g38060|protein putative phospholipase e_value:0</td><td>0.219</td><td>(0.1275 0.5823)</td><td>0.1275</td><td>0.5823</td></tr>
<tr><td>1795</td><td>SET2_G07_CLUSTER_33277</td><td>gi|50911691|ref|XP_467253.1| DnaJ protein-like [Oryza sativa (japonica cultivar-group)] >gi|51964234|ref|XP_506901.1| PREDICTED OJ1148_D05.25 gene product [Oryza sativa (japonica cultivar-group)] >gi|41053274|dbj|BAD07700.1| DnaJ protein-like [Oryza</td><td>11668.m04513|LOC_Os02g46640|protein Transposable element protein, putative e_value:2.97159e-59</td><td>0.182</td><td>(0.0793 0.4360)</td><td>0.0793</td><td>0.436</td></tr>
<tr><td>1796</td><td>WT003_A16_CLUSTER_11290</td><td>gi|50932297|ref|XP_475676.1| unknown protein [Oryza sativa (japonica cultivar-group)] >gnl|BL_ORD_ID|1598094 gi|48475201|gb|AAT44270.1| unknown protein [Oryza sativa (japonica cultivar-group)] e_value:6.78514e-169</td><td>11682.m04126|LOC_Os05g43380|protein Tesmin/TSO1-like CXC domain, putative e_value:1.8104e-170</td><td>0.204</td><td>(0.1221 0.5985)</td><td>0.1221</td><td>0.5985</td></tr>
<tr><td>1797</td><td>WT008_E19_CLUSTER_20125</td><td>gi|51091146|dbj|BAD35842.1| putative transcription factor MADS55 [Oryza sativa (japonica cultivar-group)] e_value:7.92947e-97</td><td>11668.m05121|LOC_Os02g52340|protein SRF-type transcription factor (DNA-binding and dimerisation domain), putative e_value:1.45288e-85</td><td>0.178</td><td>(0.1562 0.8755)</td><td>0.1562</td><td>0.8755</td></tr>
<tr><td>1798</td><td>WT006_N18_CLUSTER_11261</td><td>gi|6911847|emb|CAB72147.1| putative protein [Arabidopsis thaliana] >gnl|BL_ORD_ID|1454755 gi|11290092|pir||T47449 hypothetical protein T14D3.30 - Arabidopsis thaliana e_value:0</td><td>11668.m05680|LOC_Os02g57400|protein hypothetical protein e_value:0</td><td>0.14</td><td>(0.0898 0.6419)</td><td>0.0898</td><td>0.6419</td></tr>
<tr><td>1799</td><td>SET4_E21_CLUSTER_30044</td><td>gi|50726036|dbj|BAD33561.1| putative ABI3-interacting protein 2, AIP2 [Oryza sativa (japonica cultivar-group)] e_value:2.27245e-151</td><td>11681.m02407|LOC_Os09g26400|protein Similar to ABI3-interacting protein 2 e_value:6.27529e-153</td><td>0.193</td><td>(0.0919 0.4757)</td><td>0.0919</td><td>0.4757</td></tr>
<tr><td>1800</td><td>SET3_E02_CLUSTER_32001</td><td>gi|62733969|gb|AAX96078.1| At3g03330/T21P5_25 [Oryza sativa (japonica cultivar-group)] e_value:7.26305e-159</td><td>11687.m01509|LOC_Os11g16410|protein At3g03330/T21P5_25 e_value:1.85518e-160</td><td>0.182</td><td>(0.0730 0.4016)</td><td>0.073</td><td>0.4016</td></tr>
<tr><td>1801</td><td>WT004_A14_CLUSTER_34614</td><td>gi|32493110|gb|AAP85546.1| putative RING-H2 zinc finger protein [Oryza sativa (japonica cultivar-group)] e_value:0</td><td>11687.m03351|LOC_Os11g36430|protein Zinc finger, C3HC4 type (RING finger), putative e_value:0</td><td>0.198</td><td>(0.0940 0.4758)</td><td>0.094</td><td>0.4758</td></tr>
<tr><td>1802</td><td>SET1_C13_CLUSTER_17750</td><td>gi|50943559|ref|XP_481307.1| unknown protein [Oryza sativa (japonica cultivar-group)] >gi|38175640|dbj|BAD01347.1| unknown protein [Oryza sativa (japonica cultivar-group)] >gi|38175656|dbj|BAD01361.1| unknown protein [Oryza sativa (japonica cultivar</td><td>11674.m01838|LOC_Os08g19170|protein Similar to At3g09850/F8A24_10 e_value:0</td><td>0.234</td><td>(0.1415 0.6042)</td><td>0.1415</td><td>0.6042</td></tr>
<tr><td>1803</td><td>SET3_P22_CLUSTER_9058</td><td>gi|53982665|gb|AAV25644.1| putative nucleic acid binding protein [Oryza sativa (japonica cultivar-group)] e_value:9.43041e-137</td><td>11682.m03264|LOC_Os05g34640|protein nucleic acid binding protein - rice e_value:1.88614e-138</td><td>0.091</td><td>(0.0609 0.6732)</td><td>0.0609</td><td>0.6732</td></tr>
<tr><td>1804</td><td>SET1_A07_CLUSTER_26201</td><td>gi|50946241|ref|XP_482648.1| splicing factor 3B subunit 5-like protein [Oryza sativa (japonica cultivar-group)] >gi|42407437|dbj|BAD10044.1| splicing factor 3B subunit 5-like protein [Oryza sativa (japonica cultivar-group)] e_value:6.56621e-44</td><td>11674.m03415|LOC_Os08g34480|protein expressed protein e_value:4.12369e-46</td><td>0.017</td><td>(0.0147 0.8498)</td><td>0.0147</td><td>0.8498</td></tr>
<tr><td>1805</td><td>WT010_C20_CLUSTER_5888</td><td>gi|51536089|dbj|BAD38214.1| putative protein disulfide isomerase-related protein [Oryza sativa (japonica cultivar-group)] >gnl|BL_ORD_ID|243027 gi|51535926|dbj|BAD38008.1| putative protein disulfide isomerase-related protein [Oryza sativa (japonica cul</td><td>11681.m02570|LOC_Os09g27830|protein protein disulfide-isomerase domain, putative e_value:0</td><td>0.123</td><td>(0.0589 0.4794)</td><td>0.0589</td><td>0.4794</td></tr>
<tr><td>1806</td><td>WT010_C19_CLUSTER_33603</td><td>gi|21591637|gb|AAM64166.1| cleavage stimulation factor 77 [Arabidopsis thaliana] e_value:0</td><td>11686.m03781|LOC_Os12g38380|protein Suppressor of forked protein (Suf) e_value:0</td><td>0.171</td><td>(0.0866 0.5054)</td><td>0.0866</td><td>0.5054</td></tr>
<tr><td>1807</td><td>WT002_C10_CLUSTER_33603</td><td>gi|21591637|gb|AAM64166.1| cleavage stimulation factor 77 [Arabidopsis thaliana] e_value:0</td><td>11686.m03781|LOC_Os12g38380|protein Suppressor of forked protein (Suf) e_value:0</td><td>0.239</td><td>(0.1288 0.5399)</td><td>0.1288</td><td>0.5399</td></tr>
<tr><td>1808</td><td>WT013_J04_CLUSTER_25053</td><td>gi|34900946|ref|NP_911819.1| putative PRLI-interacting factor G [Oryza sativa (japonica cultivar-group)] >gnl|BL_ORD_ID|1524126 gi|50509921|dbj|BAD30221.1| putative PRLI-interacting factor G [Oryza sativa (japonica cultivar-group)] >gnl|BL_ORD_ID|15</td><td>11670.m01923|LOC_Os04g20920|protein expressed protein e_value:0</td><td>0.224</td><td>(0.1744 0.7803)</td><td>0.1744</td><td>0.7803</td></tr>
<tr><td>1809</td><td>WT006_P01_CLUSTER_19426</td><td>gi|50944199|ref|XP_481627.1| Nuclear inhibitor of PP1-like [Oryza sativa (japonica cultivar-group)] >gnl|BL_ORD_ID|1605302 gi|38637002|dbj|BAD03261.1| Nuclear inhibitor of PP1-like [Oryza sativa (japonica cultivar-group)] >gnl|BL_ORD_ID|1605302 gi|2</td><td>11674.m02299|LOC_Os08g23680|protein FHA domain, putative e_value:1.00061e-166</td><td>0.198</td><td>(0.1226 0.6208)</td><td>0.1226</td><td>0.6208</td></tr>
<tr><td>1810</td><td>WT011_N11_CLUSTER_20839</td><td>gi|34907918|ref|NP_915306.1| putative leucine zipper protein [Oryza sativa (japonica cultivar-group)] >gnl|BL_ORD_ID|1555560 gi|15624046|dbj|BAB68099.1| leucine zipper protein-like [Oryza sativa (japonica cultivar-group)] e_value:0</td><td>11667.m06094|LOC_Os01g61180|protein Exo70 exocyst complex subunit e_value:0</td><td>0.096</td><td>(0.0571 0.5914)</td><td>0.0571</td><td>0.5914</td></tr>
<tr><td>1811</td><td>WT011_L21_CLUSTER_25013</td><td>gi|50921241|ref|XP_470981.1| OSJNBb0004G23.10 [Oryza sativa (japonica cultivar-group)] >gnl|BL_ORD_ID|1589074 gi|38346876|emb|CAE04612.2| OSJNBb0004G23.10 [Oryza sativa (japonica cultivar-group)] >gnl|BL_ORD_ID|1589074 gi|32488415|emb|CAE02758.1| OS</td><td>11670.m00061|LOC_Os04g01590|protein arginase (ec 3.5.3.1). [mouse-ear cress e_value:4.65374e-178</td><td>0.114</td><td>(0.0528 0.4634)</td><td>0.0528</td><td>0.4634</td></tr>
<tr><td>1812</td><td>WT013_J03_CLUSTER_10009</td><td>gi|38345151|emb|CAE02933.2| OSJNBa0014K14.5 [Oryza sativa (japonica cultivar-group)] >gnl|BL_ORD_ID|1521544 gi|50926227|ref|XP_473074.1| OSJNBa0014K14.5 [Oryza sativa (japonica cultivar-group)] e_value:0</td><td>11670.m04086|LOC_Os04g42430|protein EF hand, putative e_value:0</td><td>0.212</td><td>(0.1310 0.6189)</td><td>0.131</td><td>0.6189</td></tr>
<tr><td>1813</td><td>WT013_O19_CLUSTER_7024</td><td>gi|50928925|ref|XP_473990.1| OSJNBa0089N06.12 [Oryza sativa (japonica cultivar-group)] >gnl|BL_ORD_ID|1592620 gi|39546242|emb|CAE04251.3| OSJNBa0089N06.12 [Oryza sativa (japonica cultivar-group)] e_value:0</td><td>11670.m05272|LOC_Os04g53700|protein expressed protein e_value:0</td><td>0.227</td><td>(0.1041 0.4582)</td><td>0.1041</td><td>0.4582</td></tr>
<tr><td>1814</td><td>WT006_I10_CLUSTER_6017</td><td>gi|52076063|dbj|BAD46576.1| unknown protein [Oryza sativa (japonica cultivar-group)] e_value:2.00878e-170</td><td>11681.m03285|LOC_Os09g36890|protein expressed protein e_value:6.5011e-172</td><td>0.304</td><td>(0.1425 0.4684)</td><td>0.1425</td><td>0.4684</td></tr>
<tr><td>1815</td><td>WT010_M02_CLUSTER_32174</td><td>gi|55733917|gb|AAV59424.1| putative deoxyribonucleoside kinase [Oryza sativa (japonica cultivar-group)] >gnl|BL_ORD_ID|1741334 gi|50931485|ref|XP_475270.1| putative deoxyribonucleoside kinase [Oryza sativa (japonica cultivar-group)] e_value:0</td><td>11667.m06566|LOC_Os01g65350|protein Deoxynucleoside kinase, putative e_value:0</td><td>0.145</td><td>(0.1527 1.0520)</td><td>0.1527</td><td>1.052</td></tr>
<tr><td>1816</td><td>WT008_B12_CLUSTER_28974</td><td>gi|34908970|ref|NP_915832.1| putative 4-diphosphocytidyl-2-C-methyl-D- erythritol kinase [Oryza sativa (japonica cultivar-group)] >gnl|BL_ORD_ID|1553070 gi|19571001|dbj|BAB86428.1| putative isopentenyl monophosphate kinase [Oryza sativa (japonica culti</td><td>11667.m05834|LOC_Os01g58790|protein 4-diphosphocytidyl-2C-methyl-D-erythritol kinase, putative e_value:0</td><td>0.206</td><td>(0.1125 0.5455)</td><td>0.1125</td><td>0.5455</td></tr>
<tr><td>1817</td><td>WT009_O06_CLUSTER_12722</td><td>gi|50935107|ref|XP_477081.1| putative LRR receptor-like kinase 2 [Oryza sativa (japonica cultivar-group)] >gnl|BL_ORD_ID|1522612 gi|34393312|dbj|BAC83241.1| putative LRR receptor-like kinase 2 [Oryza sativa (japonica cultivar-group)] e_value:0</td><td>11673.m01007|LOC_Os07g10630|protein Leucine Rich Repeat, putative e_value:0</td><td>0.167</td><td>(0.1034 0.6204)</td><td>0.1034</td><td>0.6204</td></tr>
<tr><td>1818</td><td>WT008_L20_CLUSTER_33782</td><td>gi|50929181|ref|XP_474118.1| OSJNBa0010D21.9 [Oryza sativa (japonica cultivar-group)] >gnl|BL_ORD_ID|1594474 gi|38345496|emb|CAD41707.2| OSJNBa0010D21.9 [Oryza sativa (japonica cultivar-group)] e_value:0</td><td>11670.m05466|LOC_Os04g55510|protein Zinc finger, C3HC4 type (RING finger), putative e_value:0</td><td>0.185</td><td>(0.1603 0.8646)</td><td>0.1603</td><td>0.8646</td></tr>
<tr><td>1819</td><td>WT005_D01_CLUSTER_3917</td><td>gi|50916226|ref|XP_468569.1| Putative polyprotein from transposon TNT [Oryza sativa (japonica cultivar-group)] >gnl|BL_ORD_ID|1584231 gi|24431600|gb|AAN61480.1| Putative polyprotein from transposon TNT [Oryza sativa (japonica cultivar-group)] e_value:5</td><td>11673.m04645|LOC_Os07g47030|protein expressed protein e_value:5.71974e-155</td><td>0.467</td><td>(0.4046 0.8669)</td><td>0.4046</td><td>0.8669</td></tr>
<tr><td>1820</td><td>WT004_N17_CLUSTER_17577</td><td>gi|20562907|gb|AAM22747.1| pyruvate kinase-like [Deschampsia antarctica] e_value:0</td><td>11687.m00429|LOC_Os11g05110|protein pyruvate kinase</td><td>0.049</td><td>(0.0275 0.5569)</td><td>0.0275</td><td>0.5569</td></tr>
<tr><td>1821</td><td>WT003_P15_CLUSTER_10428</td><td>gi|32490267|emb|CAE05556.1| OSJNBb0116K07.9 [Oryza sativa (japonica cultivar-group)] >gnl|BL_ORD_ID|1536958 gi|38345162|emb|CAE02944.2| OSJNBa0014K14.16 [Oryza sativa (japonica cultivar-group)] >gnl|BL_ORD_ID|1536958 gi|50926276|ref|XP_473085.1| OSJ</td><td>11670.m04108|LOC_Os04g42580|protein carboxyphosphonoenolpyruvate mutase, putative e_value:0</td><td>0.167</td><td>(0.0794 0.4743)</td><td>0.0794</td><td>0.4743</td></tr>
<tr><td>1822</td><td>WT003_I06_CLUSTER_21665</td><td>gi|34905260|ref|NP_913977.1| putative TPA: Cgi67 serine protease precursor [Oryza sativa (japonica cultivar-group)] >gnl|BL_ORD_ID|1602371 gi|29170589|dbj|BAC57808.2| putative TPA: Cgi67 serine protease precursor [Oryza sativa (japonica cultivar-group)</td><td>11674.m00573|LOC_Os08g06420|protein hypothetical protein e_value:9.00161e-177</td><td>0.142</td><td>(0.0720 0.5050)</td><td>0.072</td><td>0.505</td></tr>
<tr><td>1823</td><td>WT003_A11_CLUSTER_30342</td><td>gi|50948579|ref|XP_483817.1| putative iron inhibited ABC transporter 2 [Oryza sativa (japonica cultivar-group)] >gnl|BL_ORD_ID|1612319 gi|28071305|dbj|BAC55994.1| putative iron inhibited ABC transporter 2 [Oryza sativa (japonica cultivar-group)] >gn</td><td>11674.m04559|LOC_Os08g45010|protein ABC transporter, putative e_value:0</td><td>0.062</td><td>(0.0410 0.6621)</td><td>0.041</td><td>0.6621</td></tr>
<tr><td>1824</td><td>WT002_N21_CLUSTER_5113</td><td>gi|31126733|gb|AAP44655.1| putative RNA helicase [Oryza sativa (japonica cultivar-group)] >gnl|BL_ORD_ID|1858603 gi|50917625|ref|XP_469209.1| putative RNA helicase [Oryza sativa (japonica cultivar-group)] >gnl|BL_ORD_ID|1858603 gi|53370642|gb|AAU891</td><td>11669.m04660|LOC_Os03g46610|protein putative RNA helicase e_value:0</td><td>0.231</td><td>(0.1206 0.5214)</td><td>0.1206</td><td>0.5214</td></tr>
<tr><td>1825</td><td>SET5_P23_CLUSTER_12414</td><td>gi|50938949|ref|XP_479002.1| putative cyclin-dependent kinase CDC2C [Oryza sativa (japonica cultivar-group)] >gi|33146814|dbj|BAC79804.1| putative cyclin-dependent kinase CDC2C [Oryza sativa (japonica cultivar-group)] e_value:0</td><td>11673.m03961|LOC_Os07g40550|protein Protein kinase domain, putative e_value:0</td><td>0.147</td><td>(0.0787 0.5339)</td><td>0.0787</td><td>0.5339</td></tr>
<tr><td>1826</td><td>SET5_L18_CLUSTER_26391</td><td>gi|50251345|dbj|BAD28321.1| unknown protein [Oryza sativa (japonica cultivar-group)] >gi|50252159|dbj|BAD28155.1| unknown protein [Oryza sativa (japonica cultivar-group)] e_value:5.63193e-35</td><td>11668.m04336|LOC_Os02g44940|protein Rapid ALkalinization Factor (RALF), putative e_value:1.24863e-36</td><td>0.437</td><td>(0.2006 0.4588)</td><td>0.2006</td><td>0.4588</td></tr>
<tr><td>1827</td><td>SET5_D11_CLUSTER_26826</td><td>gi|55733899|gb|AAV59406.1| unknown protein [Oryza sativa (japonica cultivar-group)] >gi|50932531|ref|XP_475793.1| unknown protein [Oryza sativa (japonica cultivar-group)] e_value:3.92248e-109</td><td>11682.m04436|LOC_Os05g46210|protein expressed protein e_value:1.23412e-100</td><td>0.226</td><td>(0.1287 0.5703)</td><td>0.1287</td><td>0.5703</td></tr>
<tr><td>1828</td><td>SET4_B09_CLUSTER_30342</td><td>gi|50948579|ref|XP_483817.1| putative iron inhibited ABC transporter 2 [Oryza sativa (japonica cultivar-group)] >gi|28071305|dbj|BAC55994.1| putative iron inhibited ABC transporter 2 [Oryza sativa (japonica cultivar-group)] >gi|42408451|dbj|BAD09633</td><td>11674.m04559|LOC_Os08g45010|protein ABC transporter, putative e_value:0</td><td>0.056</td><td>(0.0387 0.6901)</td><td>0.0387</td><td>0.6901</td></tr>
<tr><td>1829</td><td>SET2_A05_CLUSTER_23466</td><td>gi|52075921|dbj|BAD45867.1| putative protein kinase [Oryza sativa (japonica cultivar-group)] e_value:0</td><td>11680.m04457|LOC_Os06g45280|protein protein kinase, putative e_value:0</td><td>0.064</td><td>(0.0433 0.6817)</td><td>0.0433</td><td>0.6817</td></tr>
</table>

PDF Complete
Your complimentary use period has ended. Thank you for using PDF Complete.
Click Here to upgrade to Unlimited Pages and Expanded Features

<table>
<tr><th></th><th></th><th></th><th></th><th></th><th></th><th></th><th></th></tr>
<tr><td>1830</td><td>SET1_M23_CLUSTER_7260</td><td>gi|53793125|dbj|BAD54334.1| putative susceptibility homeodomain transcription factor [Oryza sativa (japonica cultivar-group)] >gi|53793040|dbj|BAD54251.1| putative susceptibility homeodomain transcription factor [Oryza sativa (japonica cultivar-group)]</td><td>11680.m03362|LOC_Os06g34790|protein susceptibility homeodomain transciption factor e_value:1.3978e-65</td><td>0.435</td><td>(0.1340 0.3083)</td><td>0.134</td><td>0.3083</td></tr>
<tr><td>1831</td><td>WT012_K22_CLUSTER_5167</td><td>gi|31430030|gb|AAP52001.1| putative disease resistance protein [Oryza sativa (japonica cultivar-group)] >gnl|BL_ORD_ID|2007952 gi|37530824|ref|NP_919714.1| putative disease resistance protein [Oryza sativa (japonica cultivar-group)] >gnl|BL_ORD_ID|2</td><td>11687.m04378|LOC_Os11g46210|protein NB-ARC domain, putative e_value:0</td><td>0.307</td><td>(0.2282 0.7428)</td><td>0.2282</td><td>0.7428</td></tr>
<tr><td>1832</td><td>WT011_L23_CLUSTER_24129</td><td>gi|50919171|ref|XP_469982.1| putative AAA-type ATPase [Oryza sativa (japonica cultivar-group)] >gnl|BL_ORD_ID|1587457 gi|29150372|gb|AAO72381.1| putative AAA-type ATPase [Oryza sativa (japonica cultivar-group)] e_value:0</td><td>11669.m05964|LOC_Os03g58790|protein putative AAA-type ATPase e_value:0</td><td>0.564</td><td>(0.2241 0.3973)</td><td>0.2241</td><td>0.3973</td></tr>
<tr><td>1833</td><td>WT011_F04_CLUSTER_1810</td><td>gi|30686992|ref|NP_194307.2| expressed protein [Arabidopsis thaliana] e_value:6.14802e-124</td><td>11668.m05372|LOC_Os02g54580|protein Putative serine esterase (DUF676) e_value:2.01689e-115</td><td>0.309</td><td>(0.3948 1.2762)</td><td>0.3948</td><td>1.2762</td></tr>
<tr><td>1834</td><td>WT010_P21_CLUSTER_4361</td><td>gi|50934667|ref|XP_476861.1| unknown protein [Oryza sativa (japonica cultivar-group)] >gnl|BL_ORD_ID|1521852 gi|34393483|dbj|BAC83043.1| unknown protein [Oryza sativa (japonica cultivar-group)] e_value:2.11082e-123</td><td>11673.m00761|LOC_Os07g08290|protein expressed protein e_value:6.14547e-125</td><td>0.471</td><td>(0.5514 1.1707)</td><td>0.5514</td><td>1.1707</td></tr>
<tr><td>1835</td><td>WT010_M21_CLUSTER_12220</td><td>gi|34915288|ref|NP_919101.1| pentatricopeptide (PPR) repeat-containing protein-like [Oryza sativa (japonica cultivar-group)] >gnl|BL_ORD_ID|1518642 gi|24414055|dbj|BAC22304.1| pentatricopeptide (PPR) repeat-containing protein-like [Oryza sativa (japoni</td><td>11673.m00134|LOC_Os07g02280|protein hypothetical protein e_value:0</td><td>0.132</td><td>(0.0940 0.7144)</td><td>0.094</td><td>0.7144</td></tr>
<tr><td>1836</td><td>WT010_M03_CLUSTER_16717</td><td>gi|55775274|ref|XP_550563.1| putative sexual differentiation process protein isp4 [Oryza sativa (japonica cultivar-group)] >gnl|BL_ORD_ID|1752575 gi|55295864|dbj|BAD67732.1| putative sexual differentiation process protein isp4 [Oryza sativa (japonica c</td><td>11680.m00265|LOC_Os06g03540|protein small oligopeptide transporter, OPT family e_value:0</td><td>0.079</td><td>(0.0436 0.5544)</td><td>0.0436</td><td>0.5544</td></tr>
<tr><td>1837</td><td>WT009_O20_CLUSTER_7669</td><td>gi|50902002|ref|XP_463434.1| P0512C01.6 [Oryza sativa (japonica cultivar-group)] >gnl|BL_ORD_ID|2302321 gi|20804666|dbj|BAB92354.1| unknown protein [Oryza sativa (japonica cultivar-group)] >gnl|BL_ORD_ID|2302321 gi|14587298|dbj|BAB61209.1| hypotheti</td><td>11667.m05480|LOC_Os01g55530|protein hypothetical protein e_value:0</td><td>0.17</td><td>(0.0757 0.4458)</td><td>0.0757</td><td>0.4458</td></tr>
<tr><td>1838</td><td>WT009_L05_CLUSTER_8635</td><td>gi|51963548|ref|XP_506469.1| PREDICTED P0616D06.133-1 gene product [Oryza sativa (japonica cultivar-group)] e_value:0</td><td>11673.m04152|LOC_Os07g42400|protein transposon protein, putative, unclassified e_value:0</td><td>0.155</td><td>(0.0821 0.5287)</td><td>0.0821</td><td>0.5287</td></tr>
<tr><td>1839</td><td>WT008_P18_CLUSTER_24844</td><td>gi|32129315|gb|AAP73842.1| putative short chain alcohol dehydrogenase [Oryza sativa (japonica cultivar-group)] >gnl|BL_ORD_ID|2061489 gi|50540752|gb|AAT77908.1| putative alcohol dehydrogenase [Oryza sativa (japonica cultivar-group)]</td><td>11669.m06056|LOC_Os03g59610|protein putative short chain alcohol dehydrogenase e_value:1.25471e-130</td><td>0.083</td><td>(0.0953 1.1527)</td><td>0.0953</td><td>1.1527</td></tr>
<tr><td>1840</td><td>WT008_M21_CLUSTER_18579</td><td>gi|52353384|gb|AAU43952.1| putative flavonol glucosyltransferase [Oryza sativa (japonica cultivar-group)] >gnl|BL_ORD_ID|1282445 gi|52353499|gb|AAU44065.1| putative flavonol glucosyltransferase [Oryza sativa (japonica cultivar-group)] e_value:0</td><td>11682.m04311|LOC_Os05g45080|protein UDP-glucoronosyl and UDP-glucosyl transferase e_value:0</td><td>0.264</td><td>(0.1116 0.4230)</td><td>0.1116</td><td>0.423</td></tr>
<tr><td>1841</td><td>WT008_I13_CLUSTER_21584</td><td>gi|50916172|ref|XP_468550.1| putative homoserine kinase [Oryza sativa (japonica cultivar-group)] >gnl|BL_ORD_ID|1583768 gi|48716400|dbj|BAD23009.1| putative homoserine kinase [Oryza sativa (japonica cultivar-group)] e_value:6.18917e-161</td><td>11668.m05803|LOC_Os02g58510|protein homoserine kinase e_value:4.10353e-162</td><td>0.177</td><td>(0.1134 0.6402)</td><td>0.1134</td><td>0.6402</td></tr>
<tr><td>1842</td><td>WT008_E07_CLUSTER_22057</td><td>gi|50929211|ref|XP_474133.1| OSJNBb0059K02.10 [Oryza sativa (japonica cultivar-group)] >gnl|BL_ORD_ID|1593242 gi|32488919|emb|CAE04500.1| OSJNBb0059K02.10 [Oryza sativa (japonica cultivar-group)] e_value:2.79019e-176</td><td>11670.m05482|LOC_Os04g55670|protein Glycosyltransferase family 43, putative e_value:8.29022e-178</td><td>0.44</td><td>(0.1451 0.3298)</td><td>0.1451</td><td>0.3298</td></tr>
<tr><td>1843</td><td>WT007_O20_CLUSTER_11209</td><td>gi|38346766|emb|CAD41145.2| OSJNBa0081C01.17 [Oryza sativa (japonica cultivar-group)] >gnl|BL_ORD_ID|1535272 gi|50926668|ref|XP_473281.1| OSJNBa0081C01.17 [Oryza sativa (japonica cultivar-group)] e_value:0</td><td>11670.m04357|LOC_Os04g44910|protein Protein kinase domain, putative e_value:0</td><td>0.24</td><td>(0.0832 0.3461)</td><td>0.0832</td><td>0.3461</td></tr>
<tr><td>1844</td><td>WT007_D04_CLUSTER_23208</td><td>gi|50945711|ref|XP_482383.1| unknown protein [Oryza sativa (japonica cultivar-group)] >gnl|BL_ORD_ID|1606298 gi|37806193|dbj|BAC99696.1| unknown protein [Oryza sativa (japonica cultivar-group)] e_value:4.55856e-156</td><td>11674.m03139|LOC_Os08g31850|protein expressed protein e_value:1.36097e-157</td><td>0.406</td><td>(0.1268 0.3121)</td><td>0.1268</td><td>0.3121</td></tr>
<tr><td>1845</td><td>WT007_B19_CLUSTER_14339</td><td>gi|34909578|ref|NP_916136.1| P0046E05.21 [Oryza sativa (japonica cultivar-group)] >gnl|BL_ORD_ID|1550258 gi|20160583|dbj|BAB89530.1| lipase (class 3)-like protein [Oryza sativa (japonica cultivar-group)] >gnl|BL_ORD_ID|1550258</td><td>11667.m05402|LOC_Os01g54810|protein hypothetical protein e_value:0</td><td>0.132</td><td>(0.0846 0.6399)</td><td>0.0846</td><td>0.6399</td></tr>
<tr><td>1846</td><td>WT006_J12_CLUSTER_9514</td><td>gi|31430159|gb|AAP52111.1| putative salt-inducible protein [Oryza sativa (japonica cultivar-group)] >gnl|BL_ORD_ID|2008484 gi|37531044|ref|NP_919824.1| putative salt-inducible protein [Oryza sativa (japonica cultivar-group)] >gnl|BL_ORD_ID|2008484 g</td><td>11676.m00480|LOC_Os10g05670|protein Putative salt-inducible protein e_value:0</td><td>0.307</td><td>(0.1859 0.6048)</td><td>0.1859</td><td>0.6048</td></tr>
<tr><td>1847</td><td>WT006_D02_CLUSTER_3434</td><td>gi|53793294|dbj|BAD54516.1| putative brassinosteroid insensitive 1 gene [Oryza sativa (japonica cultivar-group)] e_value:0</td><td>11680.m04709|LOC_Os06g47650|protein Leucine Rich Repeat, putative e_value:0</td><td>0.289</td><td>(0.1607 0.5550)</td><td>0.1607</td><td>0.555</td></tr>
<tr><td>1848</td><td>WT005_L06_CLUSTER_5167</td><td>gi|31430030|gb|AAP52001.1| putative disease resistance protein [Oryza sativa (japonica cultivar-group)] >gnl|BL_ORD_ID|2007952 gi|37530824|ref|NP_919714.1| putative disease resistance protein [Oryza sativa (japonica cultivar-group)] >gnl|BL_ORD_ID|2</td><td>11687.m04378|LOC_Os11g46210|protein NB-ARC domain, putative e_value:0</td><td>0.351</td><td>(0.2206 0.6282)</td><td>0.2206</td><td>0.6282</td></tr>
<tr><td>1849</td><td>WT013_F09_CLUSTER_1916</td><td>gi|50932259|ref|XP_475657.1| hypothetical protein [Oryza sativa (japonica cultivar-group)] >gnl|BL_ORD_ID|1597184 gi|50080294|gb|AAT69629.1| hypothetical protein [Oryza sativa (japonica cultivar-group)] e_value:4.88204e-57</td><td>11682.m00684|LOC_Os05g07240|protein hypothetical protein e_value:8.89002e-59</td><td>0.284</td><td>(0.1166 0.4110)</td><td>0.1166</td><td>0.411</td></tr>
<tr><td>1850</td><td>WT009_H12_CLUSTER_31261</td><td>gi|62732908|gb|AAX95027.1| hypothetical protein LOC_Os11g08080 [Oryza sativa (japonica cultivar-group)] e_value:0</td><td>11687.m00747|LOC_Os11g08080|protein F8K7.13 protein - Arabidopsis thaliana e_value:0</td><td>0.195</td><td>(0.1805 0.9244)</td><td>0.1805</td><td>0.9244</td></tr>
<tr><td>1851</td><td>SET3_J05_CLUSTER_16109</td><td>gi|50936265|ref|XP_477660.1| putative DNA primase large subunit [Oryza sativa (japonica cultivar-group)] >gi|50510256|dbj|BAD31461.1| putative DNA primase large subunit [Oryza sativa (japonica cultivar-group)] >gi|50510201|dbj|BAD31369.1| putative D</td><td>11673.m02061|LOC_Os07g22400|protein Eukaryotic-type DNA primase, large subunit, putative e_value:1.77034e-155</td><td>0.2</td><td>(0.1222 0.6101)</td><td>0.1222</td><td>0.6101</td></tr>
<tr><td>1852</td><td>SET2_L09_CLUSTER_7099</td><td>gi|50912851|ref|XP_467833.1| unknown protein [Oryza sativa (japonica cultivar-group)] >gi|46390226|dbj|BAD15657.1| unknown protein [Oryza sativa (japonica cultivar-group)] >gi|46390123|dbj|BAD15558.1| unknown protein [Oryza sativa (japonica cultivar</td><td>11668.m05045|LOC_Os02g51640|protein hypothetical protein e_value:2.68508e-81</td><td>0.343</td><td>(0.2479 0.7233)</td><td>0.2479</td><td>0.7233</td></tr>
<tr><td>1853</td><td>SET4_O22_CLUSTER_31578</td><td>gi|50948625|ref|XP_483840.1| putative transcriptional regulator [Oryza sativa (japonica cultivar-group)] >gi|45735918|dbj|BAD12950.1| putative transcriptional regulator [Oryza sativa (japonica cultivar-group)] >gi|42409084|dbj|BAD10335.1| putative t</td><td>11674.m04575|LOC_Os08g45160|protein expressed protein e_value:0</td><td>0.202</td><td>(0.0975 0.4836)</td><td>0.0975</td><td>0.4836</td></tr>
<tr><td>1854</td><td>WT007_I16_CLUSTER_2424</td><td>gi|34901432|ref|NP_912062.1| tetratricopeptide repeat (TPR)-containing protein-like protein [Oryza sativa (japonica cultivar-group)] >gnl|BL_ORD_ID|1526408 gi|28971999|dbj|BAC65407.1| tetratricopeptide repeat (TPR)-containing protein-like protein [Oryz</td><td>11673.m02555|LOC_Os07g27180|protein TPR Domain, putative e_value:0</td><td>0.293</td><td>(0.1593 0.5443)</td><td>0.1593</td><td>0.5443</td></tr>
<tr><td>1855</td><td>WT004_B20_CLUSTER_28218</td><td>gi|50911961|ref|XP_467388.1| putative cytosolic monodehydroascorbate reductase [Oryza sativa (japonica cultivar-group)] >gnl|BL_ORD_ID|1580523 gi|41053156|dbj|BAD08098.1| putative cytosolic monodehydroascorbate reductase [Oryza sativa (japonica</td><td>11668.m04638|LOC_Os02g47800|protein Pyridine nucleotide-disulphide oxidoreductase, putative e_value:0</td><td>0.143</td><td>(0.0773 0.5419)</td><td>0.0773</td><td>0.5419</td></tr>
<tr><td>1856</td><td>WT002_P09_CLUSTER_34346</td><td>gi|37496995|dbj|BAC98428.1| flap endonuclease-1b [Oryza sativa (japonica cultivar-group)] >gnl|BL_ORD_ID|1021283 gi|29467052|dbj|BAC66965.1| flap endonuclease-1b [Oryza sativa (japonica cultivar-group)] e_value:1.45744e-180</td><td>11669.m06301|LOC_Os03g61820|protein flap endonuclease-1b e_value:0</td><td>0.265</td><td>(0.1162 0.4392)</td><td>0.1162</td><td>0.4392</td></tr>
<tr><td>1857</td><td>WT010_I05_CLUSTER_30345</td><td>gi|21326124|gb|AAM47590.1| putative glucosyl transferase [Sorghum bicolor] e_value:0</td><td>11669.m05532|LOC_Os03g55030|protein putative glucosyltransferase e_value:3.41147e-161</td><td>0.468</td><td>(0.2704 0.5774)</td><td>0.2704</td><td>0.5774</td></tr>
<tr><td>1858</td><td>SET3_N21_CLUSTER_10745</td><td>gi|28416709|gb|AAO42885.1| At4g38650 [Arabidopsis thaliana] >gi|42567513|ref|NP_195577.2| glycosyl hydrolase family 10 protein [Arabidopsis thaliana] e_value:5.15674e-173</td><td>11669.m01376|LOC_Os03g14010|protein At4g38650 e_value:0</td><td>0.227</td><td>(0.0931 0.4093)</td><td>0.0931</td><td>0.4093</td></tr>
<tr><td>1859</td><td>SET4_L11_CLUSTER_6760</td><td>gi|50925004|ref|XP_472839.1| OSJNBb0022F23.7 [Oryza sativa (japonica cultivar-group)] >gi|38344814|emb|CAE02870.2| OSJNBb0022F23.7 [Oryza sativa (japonica cultivar-group)] e_value:0</td><td>11670.m03782|LOC_Os04g39660|protein F-box domain, putative e_value:0</td><td>0.223</td><td>(0.1600 0.7178)</td><td>0.16</td><td>0.7178</td></tr>
<tr><td>1860</td><td>WT006_J01_CLUSTER_33140</td><td>gi|54290767|dbj|BAD61388.1| putative dehydroquinate dehydratase [Oryza sativa (japonica cultivar-group)] e_value:0</td><td>11667.m02669|LOC_Os01g27750|protein Shikimate / quinate 5-dehydrogenase, putative e_value:0</td><td>0.141</td><td>(0.0626 0.4435)</td><td>0.0626</td><td>0.4435</td></tr>
<tr><td>1861</td><td>WT007_N01_CLUSTER_9745</td><td>gi|15217747|ref|NP_174106.1| expressed protein [Arabidopsis thaliana] >gnl|BL_ORD_ID|804004 gi|12320884|gb|AAG50575.1| transposon protein, putative [Arabidopsis thaliana] >gnl|BL_ORD_ID|804004 gi|25403038|pir||F86403 probable transposon protein [imp</td><td>11686.m01337|LOC_Os12g13670|protein Similar to probable transposon protein [imported] - Arabidopsis thaliana e_value:0</td><td>0.313</td><td>(0.1756 0.5611)</td><td>0.1756</td><td>0.5611</td></tr>
<tr><td>1862</td><td>SET2_J05_CLUSTER_20640</td><td>gi|20143573|gb|AAM12330.1| putative glutathione S-transferase [Oryza sativa (japonica cultivar-group)] >gi|31433200|gb|AAP54742.1| putative glutathione S-transferase [Oryza sativa (japonica cultivar-group)] >gi|37536306|ref|NP_922455.1| putative glu</td><td>11676.m03491|LOC_Os10g38470|protein putative glutathione S-transferase e_value:1.95874e-85</td><td>0.511</td><td>(0.2200 0.4301)</td><td>0.22</td><td>0.4301</td></tr>
<tr><td>1863</td><td>WT008_E03_CLUSTER_22348</td><td>gi|50941595|ref|XP_480325.1| putative somatic embryogenesis receptor kinase 1 [Oryza sativa (japonica cultivar-group)] >gnl|BL_ORD_ID|1602735 gi|57753893|dbj|BAD86793.1| SERK-family receptor-like protein kinase [Oryza sativa (japonica cultivar-group)]</td><td>11674.m00713|LOC_Os08g07760|protein Protein kinase domain, putative e_value:0</td><td>0.07</td><td>(0.0342 0.4872)</td><td>0.0342</td><td>0.4872</td></tr>
<tr><td>1864</td><td>SET2_J02_CLUSTER_33695</td><td>gi|34906294|ref|NP_914494.1| putative phosducin-like 3 [Oryza sativa (japonica cultivar-group)] >gi|9558421|dbj|BAB03357.1| putative phosducin-like 3 [Oryza sativa (japonica cultivar-group)] e_value:1.63467e-120</td><td>11667.m00850|LOC_Os01g08960|protein Phosducin e_value:2.36406e-118</td><td>0.148</td><td>(0.0712 0.4825)</td><td>0.0712</td><td>0.4825</td></tr>
<tr><td>1865</td><td>WT013_L11_CLUSTER_24194</td><td>gi|50907649|ref|XP_465313.1| unknown protein [Oryza sativa (japonica cultivar-group)] >gnl|BL_ORD_ID|1569377 gi|46389946|dbj|BAD15798.1| unknown protein [Oryza sativa (japonica cultivar-group)] e_value:0</td><td>11668.m02165|LOC_Os02g22370|protein expressed protein e_value:0</td><td>0.147</td><td>(0.0775 0.5280)</td><td>0.0775</td><td>0.528</td></tr>
<tr><td>1866</td><td>WT007_H19_CLUSTER_3861</td><td>gi|34905154|ref|NP_913924.1| putative microtubule-associated protein [Oryza sativa (japonica cultivar-group)] >gnl|BL_ORD_ID|1609664 gi|28411849|dbj|BAC57324.1| putative microtubule-associated protein [Oryza sativa (japonica cultivar-group)] e_value:0</td><td>11674.m04076|LOC_Os08g40620|protein hypothetical protein e_value:0</td><td>0.231</td><td>(0.1193 0.5161)</td><td>0.1193</td><td>0.5161</td></tr>
<tr><td>1867</td><td>WT003_C23_CLUSTER_23004</td><td>gi|50898952|ref|XP_450264.1| putative ABI3-interacting protein 2 [Oryza sativa (japonica cultivar-group)] >gnl|BL_ORD_ID|1563447 gi|49388810|dbj|BAD26002.1| putative ABI3-interacting protein 2 [Oryza sativa (japonica cultivar-group)] >gnl|BL_ORD_ID|</td><td>11681.m00558|LOC_Os09g07380|protein expressed protein e_value:0</td><td>0.15</td><td>(0.1210 0.8088)</td><td>0.121</td><td>0.8088</td></tr>
<tr><td>1868</td><td>WT005_B16_CLUSTER_31544</td><td>gi|50931935|ref|XP_475495.1| putative B2 protein [Oryza sativa (japonica cultivar-group)] >gnl|BL_ORD_ID|1596911 gi|51038049|gb|AAT93853.1| unknown protein [Oryza sativa (japonica cultivar-group)] >gnl|BL_ORD_ID|1596911 gi|48475219|gb|AAT44288.1| pu</td><td>11682.m04027|LOC_Os05g41930|protein expressed protein e_value:2.66248e-161</td><td>0.112</td><td>(0.0679 0.6050)</td><td>0.0679</td><td>0.605</td></tr>
<tr><td>1869</td><td>WT003_L02_CLUSTER_32109</td><td>gi|50912505|ref|XP_467660.1| Erwinia induced protein 2 [Oryza sativa (japonica cultivar-group)] >gnl|BL_ORD_ID|1582770 gi|46390427|dbj|BAD15889.1| Erwinia induced protein 2 [Oryza sativa (japonica cultivar-group)] e_value:2.23155e-66</td><td>11668.m04889|LOC_Os02g50210|protein expressed protein e_value:2.50978e-68</td><td>0.154</td><td>(0.0240 0.1558)</td><td>0.024</td><td>0.1558</td></tr>
<tr><td>1870</td><td>WT007_K21_CLUSTER_34001</td><td>gi|21742575|emb|CAD40790.1| OSJNBb0076A22.1 [Oryza sativa (japonica cultivar-group)] >gnl|BL_ORD_ID|1539762 gi|50923579|ref|XP_472150.1| OSJNBb0076A22.1 [Oryza sativa (japonica cultivar-group)] e_value:3.14384e-145</td><td>11670.m02866|LOC_Os04g30210|protein hypothetical protein e_value:8.79053e-147</td><td>0.39</td><td>(0.3061 0.7855)</td><td>0.3061</td><td>0.7855</td></tr>
<tr><td>1871</td><td>WT003_A17_CLUSTER_32086</td><td>gi|50898976|ref|XP_450276.1| phosphatase 2A regulatory A subunit [Oryza sativa (japonica cultivar-group)] >gnl|BL_ORD_ID|1563510 gi|5679686|emb|CAB51804.1| protein phosphatase 2A A subunit [Oryza sativa] >gnl|BL_ORD_ID|1563510 gi|5679684|emb|CAB5180</td><td>11681.m00572|LOC_Os09g07510|protein HEAT repeat, putative e_value:0</td><td>0.04</td><td>(0.0220 0.5502)</td><td>0.022</td><td>0.5502</td></tr>
<tr><td>1872</td><td>SET5_K09_CLUSTER_9192</td><td>gi|50939271|ref|XP_479163.1| putative RNA Polymerase II subunit 14.5 kD [Oryza sativa (japonica cultivar-group)] >gi|23237936|dbj|BAC16509.1| putative RNA Polymerase II subunit 14.5 kD [Oryza sativa (japonica cultivar-group)] >gi|24060023|dbj|BAC214</td><td>11686.m03672|LOC_Os12g37370|protein RNA Polymerase II subunit 14.5 kD, putative e_value:2.25708e-38</td><td></td><td>(0.3413 -1.0000)</td><td>0.3413</td><td>-1</td></tr>
</table>

PDF Complete
Your complimentary use period has ended. Thank you for using PDF Complete.
Click Here to upgrade to Unlimited Pages and Expanded Features

1873	WT012_E15_CLUSTER_14680	gi|53793395|dbj|BAD53054.1| putative LEUNIG [Oryza sativa (japonica cultivar-group)] >gnl|BL_ORD_ID|261147 gi|53792190|dbj|BAD52823.1| putative LEUNIG [Oryza sativa (japonica cultivar-group)] e_value:0	11667.m04075|LOC_Os01g42260|protein Single-stranded DNA binding protein, SSDP, putative e_value:0	0.138	(0.0783 0.5654)	0.0783	0.5654
1874	WT011_I24_CLUSTER_34794	gi|50881435|gb|AAT85280.1| homeobox leucine-zipper protein, putative [Oryza sativa (japonica cultivar-group)] e_value:0	11686.m04166|LOC_Os12g41860|protein homeodomain transcription factor (ATHB-14) [imported] - Arabidopsis thaliana	0.144	(0.0672 0.4665)	0.0672	0.4665
1875	WT006_E20_CLUSTER_32360	gi|50939075|ref|XP_479065.1| putative receptor-like protein kinase [Oryza sativa (japonica cultivar-group)] >gnl|BL_ORD_ID|1530167 gi|34394917|dbj|BAC84469.1| putative receptor-like protein kinase [Oryza sativa (japonica cultivar-group)] >gnl|BL_ORD	11673.m04022|LOC_Os07g41140|protein Leucine Rich Repeat, putative e_value:0	0.152	(0.0772 0.5076)	0.0772	0.5076
1876	WT006_E07_CLUSTER_32078	gi|56784276|dbj|BAD81958.1| hypothetical protein [Oryza sativa (japonica cultivar-group)] >gnl|BL_ORD_ID|971491 gi|53792434|dbj|BAD53272.1| hypothetical protein [Oryza sativa (japonica cultivar-group)] e_value:2.36239e-169	11667.m05743|LOC_Os01g57920|protein F-box domain, putative e_value:1.98983e-151	0.38	(0.2693 0.7087)	0.2693	0.7087
1877	WT005_P12_CLUSTER_32409	gi|51090950|dbj|BAD35553.1| putative multiple stress-responsive zinc-finger protein [Oryza sativa (japonica cultivar-group)] >gnl|BL_ORD_ID|238369 gi|51090916|dbj|BAD35521.1| putative multiple stress-responsive zinc-finger protein [Oryza sativa (japoni	11680.m04009|LOC_Os06g41010|protein AN1-like Zinc finger, putative e_value:7.62662e-79	0.107	(0.0935 0.8762)	0.0935	0.8762
1878	SET3_O10_CLUSTER_32409	gi|51090950|dbj|BAD35553.1| putative multiple stress-responsive zinc-finger protein [Oryza sativa (japonica cultivar-group)] >gi|51090916|dbj|BAD35521.1| putative multiple stress-responsive zinc-finger protein [Oryza sativa (japonica cultivar-group)] e	11680.m04009|LOC_Os06g41010|protein AN1-like Zinc finger, putative e_value:2.21901e-78	0.111	(0.0965 0.8711)	0.0965	0.8711
1879	WT010_L11_CLUSTER_31886	gi|21593993|gb|AAM65913.1| unknown [Arabidopsis thaliana] >gnl|BL_ORD_ID|1219087 gi|19310645|gb|AAL85053.1| unknown protein [Arabidopsis thaliana] >gnl|BL_ORD_ID|1219087 gi|15028333|gb|AAK76643.1| unknown protein [Arabidopsis thaliana] >gnl|BL_OR	11687.m00042|LOC_Os11g01420|protein ribosomal protein L10, putative e_value:2.28978e-113	0.122	(0.0617 0.5044)	0.0617	0.5044
1880	WT003_J05_CLUSTER_31443	gi|53793115|dbj|BAD54324.1| putative CCT chaperonin gamma subunit [Oryza sativa (japonica cultivar-group)] e_value:0	11680.m03352|LOC_Os06g34690|protein chaperonin gamma chain, putative e_value:0	0.08	(0.0417 0.5223)	0.0417	0.5223
1881	SET5_L17_CLUSTER_23685	gi|50725436|dbj|BAD32908.1| putative receptor-like protein kinase 2 [Oryza sativa (japonica cultivar-group)] e_value:0	11680.m03797|LOC_Os06g38990|protein probable Protein kinase [imported] - Arabidopsis thaliana e_value:0	0.173	(0.0619 0.3575)	0.0619	0.3575
1882	SET3_G13_CLUSTER_29444	gi|50251773|dbj|BAD27705.1| putative heat stress protein [Oryza sativa (japonica cultivar-group)] e_value:8.37961e-77	11668.m01276|LOC_Os02g13800|protein HSF-type DNA-binding domain, putative e_value:2.34508e-78	0.584	(0.2974 0.5095)	0.2974	0.5095
1883	SET2_L04_CLUSTER_26080	gi|57900332|dbj|BAD87285.1| DNA-binding protein-related-like [Oryza sativa (japonica cultivar-group)] >gi|57899617|dbj|BAD87196.1| DNA-binding protein-related-like [Oryza sativa (japonica cultivar-group)] e_value:3.69243e-62	11667.m05425|LOC_Os01g55030|protein F20B24.1 e_value:6.47119e-60	0.229	(0.0754 0.3293)	0.0754	0.3293
1884	SET2_K05_CLUSTER_26080	gi|57900332|dbj|BAD87285.1| DNA-binding protein-related-like [Oryza sativa (japonica cultivar-group)] >gi|57899617|dbj|BAD87196.1| DNA-binding protein-related-like [Oryza sativa (japonica cultivar-group)] e_value:3.69243e-62	11667.m05425|LOC_Os01g55030|protein F20B24.1 e_value:6.47119e-60	0.229	(0.0754 0.3293)	0.0754	0.3293
1885	SET3_O22_CLUSTER_34705	gi|50915842|ref|XP_468385.1| alcohol dehydrogenase class III [Oryza sativa (japonica cultivar-group)] >gi|47848172|dbj|BAD21999.1| alcohol dehydrogenase class III [Oryza sativa (japonica cultivar-group)] >gi|47847883|dbj|BAD21676.1| alcohol dehydrog	11668.m05634|LOC_Os02g57040|protein oxidoreductase, zinc-binding dehydrogenase family e_value:0	0.072	(0.0384 0.5321)	0.0384	0.5321
1886	SET3_D13_CLUSTER_19868	gi|34898652|ref|NP_910672.1| contains EST AU031225(E61165)˜nhp2-like protein [Oryza sativa (japonica cultivar-group)] >gi|23589940|dbj|BAC20622.1| putative nucleolar protein family A member 2 [Oryza sativa (japonica cultivar-group)] >gi|11862950|dbj	11668.m04829|LOC_Os02g49610|protein Ribosomal protein L7Ae, putative e_value:1.30244e-70	0.21	(0.0732 0.3488)	0.0732	0.3488
1887	WT007_A12_CLUSTER_32141	gi|9759450|dbj|BAB10366.1| unnamed protein product [Arabidopsis thaliana] >gnl|BL_ORD_ID|1093732 gi|21928059|gb|AAM78058.1| AT5g61030/maf19_30 [Arabidopsis thaliana] >gnl|BL_ORD_ID|1093732 gi|15239505|ref|NP_200911.1| RNA-binding protein, putative [	11686.m03093|LOC_Os12g31800|protein RNA-binding protein precursor e_value:1.80523e-69	0.26	(0.3441 1.3252)	0.3441	1.3252
1888	WT004_B21_CLUSTER_31082	gi|50929795|ref|XP_474425.1| OSJNBa0088H09.21 [Oryza sativa (japonica cultivar-group)] >gnl|BL_ORD_ID|1594612 gi|21741125|emb|CAD41925.1| OSJNBa0070M12.3 [Oryza sativa (japonica cultivar-group)] >gnl|BL_ORD_ID|1594612 gi|32488720|emb|CAE03463.1| OSJ	11670.m05837|LOC_Os04g58910|protein Protein kinase domain, putative e_value:0	0.264	(0.1697 0.6430)	0.1697	0.643
1889	WT002_O05_CLUSTER_34596	gi|50934451|ref|XP_476753.1| high-glucose-regulated protein 8-like [Oryza sativa (japonica cultivar-group)] >gnl|BL_ORD_ID|1521400 gi|50509741|dbj|BAD31793.1| high-glucose-regulated protein 8-like [Oryza sativa (japonica cultivar-group)] e_value:0	11673.m00676|LOC_Os07g07490|protein YT521-B-like family, putative e_value:0	0.245	(0.2914 1.1871)	0.2914	1.1871
1890	WT003_A19_CLUSTER_31722	gi|34903768|ref|NP_913231.1| unnamed protein product [Oryza sativa (japonica cultivar-group)] >gnl|BL_ORD_ID|1752044 gi|7340876|dbj|BAA92966.1| putative shaggy-like kinase dzeta [Oryza sativa (japonica cultivar-group)] e_value:0	11682.m01108|LOC_Os05g11730|protein Protein kinase domain, putative e_value:0	0.106	(0.0702 0.6616)	0.0702	0.6616
1891	WT012_B10_CLUSTER_6449	gi|19172018|gb|AAL85701.1| Mei2-like protein [Hordeum vulgare subsp. vulgare] e_value:0	11668.m02983|LOC_Os02g31290|protein Mei2-like protein	0.209	(0.2170 1.0393)	0.217	1.0393
1892	WT006_D12_CLUSTER_11714	gi|50899666|ref|XP_450621.1| unknown protein [Oryza sativa (japonica cultivar-group)] >gnl|BL_ORD_ID|1563922 gi|50726194|dbj|BAD33713.1| unknown protein [Oryza sativa (japonica cultivar-group)] >gnl|BL_ORD_ID|1563922 gi|48716731|dbj|BAD23412.1| unkn	11681.m01786|LOC_Os09g19890|protein hypothetical protein e_value:0	0.184	(0.1495 0.8120)	0.1495	0.812
1893	WT003_M05_CLUSTER_31538	gi|50947677|ref|XP_483366.1| putative oligouridylate binding protein [Oryza sativa (japonica cultivar-group)] >gnl|BL_ORD_ID|1609692 gi|42409171|dbj|BAD10437.1| putative oligouridylate binding protein [Oryza sativa (japonica cultivar-group)] >gnl|BL	11674.m04104|LOC_Os08g40880|protein RNA recognition motif. (a.k.a. RRM, RBD, or RNP domain), putative e_value:0	0.061	(0.0290 0.4770)	0.029	0.477
1894	WT002__C17_CLUSTER_33542	gi|62899852|sp|Q6EUN0|ASCL1_ORYSA ASC1-like protein 1 (Alternaria stem canker resistance-like protein 1) >gnl|BL_ORD_ID|592363 gi|50251718|dbj|BAD27639.1| putative ASC1 [Oryza sativa (japonica cultivar-group)] e_value:4.78875e-129	11681.m01861|LOC_Os09g20630|protein resistance protein, putative e_value:3.94753e-152	0.139	(0.0780 0.5593)	0.078	0.5593
1895	WT006_A02_CLUSTER_34937	gi|62734287|gb|AAX96396.1| At1g05520/T25N20_16 [Oryza sativa (japonica cultivar-group)] e_value:0	11687.m02188|LOC_Os11g24560|protein At1g05520/T25N20_16 e_value:0	0.057	(0.0297 0.5186)	0.0297	0.5186
1896	WT013_I14_CLUSTER_29576	gi|38346757|emb|CAE03862.2| OSJNBa0081C01.8 [Oryza sativa (japonica cultivar-group)] >gnl|BL_ORD_ID|1535263 gi|38346626|emb|CAD41209.2| OSJNBa0074L08.20 [Oryza sativa (japonica cultivar-group)] >gnl|BL_ORD_ID|1535263 gi|50926650|ref|XP_473272.1| OSJ	11686.m00517|LOC_Os12g05810|protein syntaxin 81 (atsyp81). [mouse-ear cress e_value:1.36883e-128	0.151	(0.0707 0.4674)	0.0707	0.4674
1897	SET4_O21_CLUSTER_34821	gi|50511477|gb|AAT77399.1| 'U2 snRNP auxiliary factor, small subunit' [Oryza sativa (japonica cultivar-group)] e_value:1.25807e-136	11682.m04696|LOC_Os05g48960|protein U2 snRNP auxiliary factor, small subunit e_value:9.31848e-139	0.089	(0.0870 0.9748)	0.087	0.9748
1898	WT012_L21_CLUSTER_13753	gi|34098799|gb|AAQ56782.1| At2g27460 [Arabidopsis thaliana] >gnl|BL_ORD_ID|2185877 gi|4314388|gb|AAD15598.1| expressed protein [Arabidopsis thaliana] >gnl|BL_ORD_ID|2185877 gi|15450830|gb|AAK96686.1| Unknown protein [Arabidopsis thaliana] >gnl|BL	11682.m03479|LOC_Os05g37120|protein Sec23/Sec24 trunk domain, putative e_value:0	0.238	(0.0893 0.3758)	0.0893	0.3758
1899	WT002_K02_CLUSTER_23254	gi|56236078|gb|AAV84495.1| At4g28370 [Arabidopsis thaliana] e_value:0	11673.m03039|LOC_Os07g31850|protein Zinc finger, C3HC4 type (RING finger), putative e_value:0	0.081	(0.0442 0.5465)	0.0442	0.5465
1900	WT007_M20_CLUSTER_10342	gi|50941893|ref|XP_480474.1| putative glycinamide ribonucleotide synthetase [Oryza sativa (japonica cultivar-group)] >gnl|BL_ORD_ID|1602894 gi|51964736|ref|XP_507152.1| PREDICTED P0610E02.33 gene product [Oryza sativa (japonica cultivar-group)] >gnl	11674.m00861|LOC_Os08g09210|protein phosphoribosylamine--glycine ligase e_value:0	0.132	(0.1007 0.7654)	0.1007	0.7654
1901	WT011_G24_CLUSTER_611	gi|51038201|gb|AAT94004.1| unknown protein [Oryza sativa (japonica cultivar-group)] >gnl|BL_ORD_ID|1020631 gi|51038161|gb|AAT93964.1| unknown protein [Oryza sativa (japonica cultivar-group)] e_value:1.47243e-76	11682.m00476|LOC_Os05g05330|protein expressed protein e_value:1.55934e-80	0.565	(0.6820 1.2067)	0.682	1.2067
1902	SET5_J21_CLUSTER_14102	gi|50904313|ref|XP_463645.1| P0592G05.9 [Oryza sativa (japonica cultivar-group)] e_value:0	11667.m06998|LOC_Os01g69900|protein CBS domain, putative e_value:0	0.061	(0.0391 0.6364)	0.0391	0.6364
1903	WT002_L07_CLUSTER_25698	gi|13374863|emb|CAC34497.1| putative protein [Arabidopsis thaliana] >gnl|BL_ORD_ID|1488112 gi|54261725|gb|AAV31166.1| At5g22040 [Arabidopsis thaliana] >gnl|BL_ORD_ID|1488112 gi|22326954|ref|NP_680191.1| expressed protein [Arabidopsis thaliana] >g	11669.m01682|LOC_Os03g16730|protein hypothetical protein e_value:6.96122e-127	0.289	(0.1473 0.5094)	0.1473	0.5094
1904	SET2_O05_CLUSTER_33396	gi|50929151|ref|XP_474103.1| OSJNBa0070O11.6 [Oryza sativa (japonica cultivar-group)] >gi|34908438|ref|NP_915566.1| putative G10 protein [Oryza sativa (japonica cultivar-group)] >gi|38344007|emb|CAE03175.2| OSJNBa0070O11.6 [Oryza sativa (japonica cu	11670.m05441|LOC_Os04g55290|protein G10 protein e_value:9.53754e-84	0.023	(0.0208 0.9218)	0.0208	0.9218
1905	WT012_L17_CLUSTER_33381	gi|31296709|gb|AAP46638.1| PG1 [Hordeum vulgare] e_value:0	11687.m03790|LOC_Os11g40590|protein expressed protein	0.28	(0.2328 0.8325)	0.2328	0.8325
1906	SET4_E09_CLUSTER_7199	gi|52076158|dbj|BAD46671.1| unknown protein [Oryza sativa (japonica cultivar-group)] >gi|52075585|dbj|BAD46695.1| unknown protein [Oryza sativa (japonica cultivar-group)] e_value:9.00413e-56	11681.m03122|LOC_Os09g34850|protein expressed protein e_value:1.17177e-57	0.191	(0.1373 0.7176)	0.1373	0.7176
1907	SET3_D22_CLUSTER_9296	gi|51860752|gb|AAU11500.1| rhodanese-like protein [Holcus lanatus] e_value:8.75022e-66	11669.m00090|LOC_Os03g01770|protein expressed protein e_value:8.11474e-59	0.227	(0.0956 0.4207)	0.0956	0.4207
1908	WT009_L08_CLUSTER_26994	gi|27413497|gb|AAO11666.1| hypothetical protein [Arabidopsis thaliana] >gnl|BL_ORD_ID|2281528 gi|61742576|gb|AAX55109.1| hypothetical protein At2g06025 [Arabidopsis thaliana] >gnl|BL_ORD_ID|2281528 gi|62318765|dbj|BAD93799.1| hypothetical protein [A	11669.m00765|LOC_Os03g07990|protein acetyltransferase, GNAT family, putative e_value:2.85051e-139	0.243	(0.1216 0.5011)	0.1216	0.5011
1909	SET2_P19_CLUSTER_26994	gi|27413497|gb|AAO11666.1| hypothetical protein [Arabidopsis thaliana] >gi|61742576|gb|AAX55109.1| hypothetical protein At2g06025 [Arabidopsis thaliana] >gi|62318765|dbj|BAD93799.1| hypothetical protein [Arabidopsis thaliana] >gi|30678254|ref|NP_	11669.m00765|LOC_Os03g07990|protein acetyltransferase, GNAT family, putative e_value:8.86106e-141	0.217	(0.1054 0.4863)	0.1054	0.4863
1910	WT003_F10_CLUSTER_25349	gi|50933293|ref|XP_476174.1| putative transmembrane protein [Oryza sativa (japonica cultivar-group)] >gnl|BL_ORD_ID|1600909 gi|48843759|gb|AAT47018.1| putative transmembrane protein [Oryza sativa (japonica cultivar-group)] e_value:1.09131e-140	11686.m00514|LOC_Os12g05780|protein hypothetical protein e_value:1.89276e-168	0.121	(0.0649 0.5379)	0.0649	0.5379
1911	WT006_J14_CLUSTER_34014	gi|53792834|dbj|BAD53867.1| zinc finger protein-like [Oryza sativa (japonica cultivar-group)] e_value:0	11680.m04729|LOC_Os06g47840|protein zinc finger protein, putative e_value:0	0.121	(0.0703 0.5816)	0.0703	0.5816
1912	WT003_N22_CLUSTER_33934	gi|31249753|gb|AAP46245.1| putative 5'-phosphoribosyl-5-aminoimidazole synthetase [Oryza sativa (japonica cultivar-group)] >gnl|BL_ORD_ID|1999836 gi|34897250|ref|NP_909971.1| putative 5'-phosphoribosyl-5-aminoimidazole synthetase [Oryza sativ	11669.m06276|LOC_Os03g61600|protein putative 5'-phosphoribosyl-5-aminoimidazole synthetase e_value:1.63605e-	0.153	(0.0961 0.6263)	0.0961	0.6263
1913	WT013_B04_CLUSTER_34474	gi|50921405|ref|XP_471063.1| OSJNBa0020P07.8 [Oryza sativa (japonica cultivar-group)] >gnl|BL_ORD_ID|1589606 gi|38344865|emb|CAE01291.2| OSJNBa0020P07.8 [Oryza sativa (japonica cultivar-group)] e_value:6.97591e-118	11670.m00192|LOC_Os04g02870|protein RNA recognition motif. (a.k.a. RRM, RBD, or RNP domain), putative e_value:1.85517e-	0.156	(0.0732 0.4704)	0.0732	0.4704
1914	SET3_L18_CLUSTER_27197	gi|46155797|ref|ZP_00204600.1| hypothetical protein Haso02002276 [Haemophilus somnus 2336] e_value:2.08415e-57	11687.m02671|LOC_Os11g29340|protein At5g63460 e_value:1.26746e-66	0.209	(0.1564 0.7484)	0.1564	0.7484
1915	WT005_F12_CLUSTER_33148	gi|34904174|ref|NP_913434.1| putative PRLI-interacting factor N [Oryza sativa (japonica cultivar-group)] e_value:0	11667.m01226|LOC_Os01g12530|protein Similar to PRLI-interacting factor N e_value:0	0.247	(0.1770 0.7172)	0.177	0.7172

PDF Complete
Your complimentary use period has ended. Thank you for using PDF Complete.
Click Here to upgrade to Unlimited Pages and Expanded Features

1916	SET4_C06_CLUSTER_7307	gi|31432836|gb|AAP54423.1| putative RNA-binding protein [Oryza sativa (japonica cultivar-group)] >gi|37535668|ref|NP_922136.1| putative RNA-binding protein [Oryza sativa (japonica cultivar-group)] >gi|22128716|gb|AAM92828.1| putative RNA-binding pro	11676.m03135|LOC_Os10g35220|protein putative RNA-binding protein e_value:0	0.305	(0.1383 0.4540)	0.1383	0.454
1917	WT008_L09_CLUSTER_7827	gi|50929499|ref|XP_474277.1| OSJNBa0043A12.14 [Oryza sativa (japonica cultivar-group)] >gnl|BL_ORD_ID|1593154 gi|32488384|emb|CAE02809.1| OSJNBa0043A12.14 [Oryza sativa (japonica cultivar-group)] e_value:0	11670.m05663|LOC_Os04g57300|protein Phosphatidylinositol 3- and 4-kinase, putative e_value:0	0.161	(0.0948 0.5869)	0.0948	0.5869
1918	WT013_O24_CLUSTER_24036	gi|50905191|ref|XP_464084.1| putative kinase-binding protein 1 [Oryza sativa (japonica cultivar-group)] >gnl|BL_ORD_ID|1565940 gi|51963862|ref|XP_506714.1| PREDICTED OSJNBa0026E05.36 gene product [Oryza sativa (japonica cultivar-group)] >gnl|BL_ORD_	11668.m00403|LOC_Os02g04660|protein Skb1 methyltransferase, putative e_value:0	0.1	(0.0527 0.5261)	0.0527	0.5261
1919	WT002__C05_CLUSTER_5390	gi|62701930|gb|AAX93003.1| Amidase, putative [Oryza sativa (japonica cultivar-group)] e_value:0	11686.m00663|LOC_Os12g07150|protein N-acylethanolamine amidohydrolase e_value:0	0.119	(0.0806 0.6788)	0.0806	0.6788
1920	WT006_H01_CLUSTER_34573	gi|31433361|gb|AAP54880.1| putative zinc finger protein [Oryza sativa (japonica cultivar-group)] >gnl|BL_ORD_ID|2012907 gi|37536582|ref|NP_922593.1| putative zinc finger protein [Oryza sativa (japonica cultivar-group)] >gnl|BL_ORD_ID|2012907 gi|1335	11676.m03637|LOC_Os10g39820|protein Transposable element protein, putative e_value:7.37954e-139	0.091	(0.0531 0.5861)	0.0531	0.5861
1921	WT004_P03_CLUSTER_1320	gi|56784224|dbj|BAD81719.1| putative aspartate aminotransferase [Oryza sativa (japonica cultivar-group)] e_value:0	11667.m06538|LOC_Os01g65090|protein probable aspartate aminotransferase [imported] - Arabidopsis thaliana e_value:0	0.153	(0.0876 0.5726)	0.0876	0.5726
1922	WT006_G05_CLUSTER_34327	gi|50428682|gb|AAT77033.1| putative TCP-1/cpn60 chaperonin family protein [Oryza sativa (japonica cultivar-group)] e_value:0	11669.m04189|LOC_Os03g42220|protein putative TCP-1/cpn60 chaperonin family protein e_value:0	0.053	(0.0296 0.5542)	0.0296	0.5542
1923	SET5_A10_CLUSTER_22816	gi|51963588|ref|XP_506497.1| PREDICTED OJ1136_D11.106 gene product [Oryza sativa (japonica cultivar-group)] >gi|50508331|dbj|BAD30182.1| SWI/SNF-related matrix-associated actin-dependent regulator of chromatin a-like [Oryza sativa (japonica cultivar-gr	11673.m04345|LOC_Os07g44210|protein Helicase conserved C-terminal domain, putative e_value:0	0.186	(0.0793 0.4261)	0.0793	0.4261
1924	WT013_K18_CLUSTER_6016	gi|50915574|ref|XP_468251.1| unknown protein [Oryza sativa (japonica cultivar-group)] >gnl|BL_ORD_ID|1583372 gi|51964482|ref|XP_507026.1| PREDICTED OJ1695_H09.11 gene product [Oryza sativa (japonica cultivar-group)] >gnl|BL_ORD_ID|1583372 gi|4749722	11668.m05441|LOC_Os02g55200|protein expressed protein e_value:9.36809e-167	0.134	(0.0997 0.7418)	0.0997	0.7418
1925	WT012_N09_CLUSTER_2263	gi|17017400|gb|AAL33654.1| histone acetyl transferase [Zea mays] >gnl|BL_ORD_ID|1839550 gi|18447805|emb|CAD22097.1| histone acetyltransferase [Zea mays] >gnl|BL_ORD_ID|1839550 gi|18369787|emb|CAD21650.1| GCN5 protein [Zea mays] e_value:0	11676.m02378|LOC_Os10g28040|protein histone acetyltransferase e_value:0	0.1	(0.0548 0.5462)	0.0548	0.5462
1926	WT007_A20_CLUSTER_10198	gi|14532596|gb|AAK64026.1| unknown protein [Arabidopsis thaliana] e_value:2.28708e-121	11687.m00033|LOC_Os11g01330|protein expressed protein	0.276	(0.1635 0.5925)	0.1635	0.5925
1927	WT003_N01_CLUSTER_27141	gi|55769781|ref|XP_549880.1| unknown protein [Oryza sativa (japonica cultivar-group)] >gnl|BL_ORD_ID|1753731 gi|52075723|dbj|BAD44943.1| unknown protein [Oryza sativa (japonica cultivar-group)] e_value:0	11667.m00118|LOC_Os01g02090|protein expressed protein e_value:0	0.203	(0.1190 0.5864)	0.119	0.5864
1928	WT002_O15_CLUSTER_34038	gi|50939903|ref|XP_479479.1| putative pre-mRNA splicing factor SF2 [Oryza sativa (japonica cultivar-group)] >gnl|BL_ORD_ID|1534194 gi|33146854|dbj|BAC79849.1| putative pre-mRNA splicing factor SF2 [Oryza sativa (japonica cultivar-group)] e_value:1.3291	11673.m04713|LOC_Os07g47630|protein RNA recognition motif. (a.k.a. RRM, RBD, or RNP domain), putative e_value:9.91864e-	0.144	(0.0911 0.6344)	0.0911	0.6344
1929	WT012_B08_CLUSTER_31158	gi|50929365|ref|XP_474210.1| OSJNBa0015K02.14 [Oryza sativa (japonica cultivar-group)] >gnl|BL_ORD_ID|1594248 gi|38345202|emb|CAE02897.2| OSJNBa0015K02.14 [Oryza sativa (japonica cultivar-group)] e_value:0	11670.m05574|LOC_Os04g56530|protein Protein kinase domain, putative e_value:0	0.124	(0.0624 0.5027)	0.0624	0.5027
1930	WT013_A08_CLUSTER_33237	gi|47606403|gb|AAT36215.1| DNA repair and transcription factor XPB1 [Arabidopsis thaliana] e_value:0	11667.m04836|LOC_Os01g49680|protein DNA repair and transcription factor XPB1 e_value:0	0.077	(0.0462 0.6043)	0.0462	0.6043
1931	WT006_E09_CLUSTER_33390	gi|50878370|gb|AAT85145.1| unknown protein [Oryza sativa (japonica cultivar-group)] e_value:9.60863e-94	11682.m03120|LOC_Os05g33300|protein Tat (twin-arginine translocation) pathway signal sequence, putative e_value:3.29275e-95	0.297	(0.1876 0.6309)	0.1876	0.6309
1932	SET3_F04_CLUSTER_31587	gi|50929007|ref|XP_474031.1| OSJNBa0043L09.27 [Oryza sativa (japonica cultivar-group)] >gi|38344807|emb|CAE03008.2| OSJNBa0043L09.27 [Oryza sativa (japonica cultivar-group)] e_value:1.80057e-101	11670.m05340|LOC_Os04g54330|protein acetyltransferase, GNAT family, putative e_value:3.50002e-103	0.134	(0.0475 0.3552)	0.0475	0.3552
1933	WT002_M11_CLUSTER_10168	gi|50937279|ref|XP_478167.1| putative calmodulin-binding protein [Oryza sativa (japonica cultivar-group)] >gnl|BL_ORD_ID|1526936 gi|33146995|dbj|BAC80067.1| putative calmodulin-binding protein [Oryza sativa (japonica cultivar-group)] e_value:0	11673.m02929|LOC_Os07g30770|protein IQ calmodulin-binding motif, putative e_value:0	0.329	(0.1439 0.4377)	0.1439	0.4377
1934	WT004_A13_CLUSTER_5757	gi|34910022|ref|NP_916358.1| P0413G02.12 [Oryza sativa (japonica cultivar-group)] >gnl|BL_ORD_ID|1552023 gi|22202697|dbj|BAC07355.1| unknown protein [Oryza sativa (japonica cultivar-group)] e_value:2.43368e-61	11667.m05631|LOC_Os01g56890|protein expressed protein e_value:1.05111e-58	0.247	(0.1350 0.5463)	0.135	0.5463
1935	WT010_G02_CLUSTER_33445	gi|22328551|ref|NP_192745.2| KH domain-containing protein [Arabidopsis thaliana] e_value:2.10951e-98	11674.m00100|LOC_Os08g01930|protein KH domain, putative e_value:0	0.226	(0.1278 0.5646)	0.1278	0.5646
1936	WT009__C02_CLUSTER_31773	gi|50915968|ref|XP_468448.1| putative fibrillarin [Oryza sativa (japonica cultivar-group)] >gnl|BL_ORD_ID|1583528 gi|48716271|dbj|BAD22886.1| putative fibrillarin [Oryza sativa (japonica cultivar-group)] >gnl|BL_ORD_ID|1583528 gi|48716513|dbj|BAD231	11682.m00801|LOC_Os05g08360|protein fibrillarin - Arabidopsis thaliana e_value:1.3474e-157	0.094	(0.0519 0.5547)	0.0519	0.5547
1937	WT004_N03_CLUSTER_33757	gi|50919281|ref|XP_470037.1| putative 40S ribosomal protein S2 [Oryza sativa (japonica cultivar-group)] >gnl|BL_ORD_ID|1588215 gi|30103021|gb|AAP21434.1| putative 40S ribosomal protein S2 [Oryza sativa (japonica cultivar-group)] e_value:5.50819e-148	11669.m06023|LOC_Os03g59310|protein putative 40S ribosomal protein S2 e_value:1.4974e-149	0.104	(0.0394 0.3802)	0.0394	0.3802
1938	SET5_L01_CLUSTER_3707	gi|15222176|ref|NP_172157.1| expressed protein [Arabidopsis thaliana] e_value:0	11669.m02208|LOC_Os03g21530|protein expressed protein	0.162	(0.0918 0.5657)	0.0918	0.5657
1939	WT012_F20_CLUSTER_21084	gi|31433670|gb|AAP55154.1| unknown protein [Oryza sativa (japonica cultivar-group)] >gnl|BL_ORD_ID|2013177 gi|37537130|ref|NP_922867.1| unknown protein [Oryza sativa (japonica cultivar-group)] >gnl|BL_ORD_ID|2013177 gi|18266654|gb|AAL67600.1| unknow	11676.m03943|LOC_Os10g42610|protein expressed protein e_value:1.70092e-57	0.903	(0.3444 0.3813)	0.3444	0.3813
1940	WT007_N23_CLUSTER_26921	gi|52076013|dbj|BAD46466.1| unknown protein [Oryza sativa (japonica cultivar-group)] e_value:2.30259e-160	11681.m03349|LOC_Os09g37510|protein expressed protein e_value:5.63019e-178	0.248	(0.1663 0.6707)	0.1663	0.6707
1941	SET5__C19_CLUSTER_348	gi|57900433|dbj|BAD87726.1| putative poly-glutamine tract-binding protein [Oryza sativa (japonica cultivar-group)] >gi|56784944|dbj|BAD82402.1| putative poly-glutamine tract-binding protein [Oryza sativa (japonica cultivar-group)] e_value:0	11667.m06932|LOC_Os01g68760|protein hypothetical protein e_value:0	0.225	(0.1188 0.5287)	0.1188	0.5287
1942	SET3_M08_CLUSTER_3008	gi|50917943|ref|XP_469368.1| putative CLC-d chloride channel protein [Oryza sativa (japonica cultivar-group)] >gi|27552547|gb|AAO19370.1| putative CLC-d chloride channel protein [Oryza sativa (japonica cultivar-group)] e_value:0	11669.m04912|LOC_Os03g48940|protein putative CLC-d chloride channel protein e_value:0	0.128	(0.0611 0.4763)	0.0611	0.4763
1943	WT007_A15_CLUSTER_33631	gi|55908888|gb|AAV67831.1| unknown protein [Oryza sativa (japonica cultivar-group)] >gnl|BL_ORD_ID|1794554 gi|50933435|ref|XP_476245.1| unknown protein [Oryza sativa (japonica cultivar-group)] >gnl|BL_ORD_ID|1794554 gi|55168099|gb|AAV43967.1| unknow	11682.m01773|LOC_Os05g18770|protein expressed protein e_value:0	0.215	(0.1302 0.6069)	0.1302	0.6069
1944	WT011_B04_CLUSTER_18255	gi|34908792|ref|NP_915743.1| P0415A04.33 [Oryza sativa (japonica cultivar-group)] >gnl|BL_ORD_ID|1553023 gi|20160828|dbj|BAB89768.1| putative partner of Nob1 [Oryza sativa (japonica cultivar-group)] >gnl|BL_ORD_ID|1553023	11669.m00167|LOC_Os03g02420|protein expressed protein e_value:1.4776e-114	0.113	(0.0424 0.3760)	0.0424	0.376
1945	WT008_A07_CLUSTER_33018	gi|50913151|ref|XP_467983.1| putative inorganic pyrophosphatase [Oryza sativa (japonica cultivar-group)] >gnl|BL_ORD_ID|2363349 gi|51979373|ref|XP_507534.1| PREDICTED OJ1767_D02.15-2 gene product [Oryza sativa (japonica cultivar-group)] >gnl|BL_ORD_	11668.m05189|LOC_Os02g52940|protein inorganic pyrophosphatase, putative e_value:7.88714e-149	0.116	(0.0479 0.4124)	0.0479	0.4124
1946	WT003__C15_CLUSTER_2337	gi|62734700|gb|AAX96809.1| expressed protein [Oryza sativa (japonica cultivar-group)] e_value:0	11687.m01800|LOC_Os11g19250|protein expressed protein	0.154	(0.0839 0.5446)	0.0839	0.5446
1947	WT010_I16_CLUSTER_34500	gi|50947885|ref|XP_483470.1| putative uridine kinase/uracil phosphoribosyltransferase [Oryza sativa (japonica cultivar-group)] >gnl|BL_ORD_ID|1609796 gi|42407979|dbj|BAD09117.1| putative uridine kinase/uracil phosphoribosyltransferase [Oryza sativa (ja	11681.m03007|LOC_Os09g32820|protein uridine kinase, putative e_value:0	0.093	(0.0454 0.4898)	0.0454	0.4898
1948	WT013_I02_CLUSTER_20921	gi|50919157|ref|XP_469975.1| putative RNA binding ribonucleoprotein [Oryza sativa (japonica cultivar-group)] >gnl|BL_ORD_ID|1587450 gi|29150367|gb|AAO72376.1| putative RNA binding ribonucleoprotein [Oryza sativa (japonica cultivar-group)] e_value:0	11669.m05956|LOC_Os03g58720|protein putative RNA binding ribonucleoprotein e_value:0	0.256	(0.1391 0.5438)	0.1391	0.5438
1949	WT009_G19_CLUSTER_34055	gi|52353467|gb|AAU44034.1| putative tonneau 2 [Oryza sativa (japonica cultivar-group)] e_value:0	11682.m00518|LOC_Os05g05710|protein EF hand, putative	0.025	(0.0137 0.5442)	0.0137	0.5442
1950	SET2__C17_CLUSTER_32567	gi|58257479|emb|CAF22032.1| DRP1 protein [Triticum turgidum subsp. durum] e_value:0	11674.m00877|LOC_Os08g09320|protein YL1 nuclear protein e_value:1.50835e-145	0.157	(0.1031 0.6575)	0.1031	0.6575
1951	WT013_O15_CLUSTER_12069	gi|55775186|ref|XP_550519.1| putative GFA2 [Oryza sativa (japonica cultivar-group)] >gnl|BL_ORD_ID|1752739 gi|55296201|dbj|BAD67919.1| putative GFA2 [Oryza sativa (japonica cultivar-group)] e_value:8.49807e-125	11686.m00652|LOC_Os12g07060|protein DnaJ protein, putative e_value:0	0.234	(0.0993 0.4239)	0.0993	0.4239
1952	WT013_K19_CLUSTER_21353	gi|34897078|ref|NP_909885.1| putative fatty acid hydroxylase [Oryza sativa (japonica cultivar-group)] >gnl|BL_ORD_ID|1587218 gi|12957715|gb|AAK09233.1| putative fatty acid hydroxylase [Oryza sativa (japonica cultivar-group)] e_value:1.71301e-116	11669.m05735|LOC_Os03g56820|protein putative fatty acid hydroxylase e_value:3.15773e-118	0.027	(0.1326 4.9254)	0.1326	4.9254
1953	WT002_O02_CLUSTER_12434	gi|50941197|ref|XP_480126.1| putative monodehydroascorbate reductase [Oryza sativa (japonica cultivar-group)] >gnl|BL_ORD_ID|1601925 gi|37573040|dbj|BAC98552.1| putative monodehydroascorbate reductase [Oryza sativa (japonica cultivar-	11674.m00477|LOC_Os08g05570|protein Pyridine nucleotide-disulphide oxidoreductase, putative e_value:0	0.148	(0.0778 0.5257)	0.0778	0.5257
1954	WT011_J08_CLUSTER_310	gi|50899460|ref|XP_450518.1| putative oleosin [Oryza sativa (japonica cultivar-group)] >gnl|BL_ORD_ID|1563842 gi|51963736|ref|XP_506648.1| PREDICTED OSJNBb0024J13.29 gene product [Oryza sativa (japonica cultivar-group)] >gnl|BL_ORD_ID|1563842 gi|487	11681.m01394|LOC_Os09g15520|protein oleosin zm-i (oleosin 16 kda) (lipid body-associated major protein)(lipid body-associated protein l3) e_value:1.72063e-51	0.544	(0.2078 0.3818)	0.2078	0.3818
1955	WT002__C09_CLUSTER_310	gi|50899460|ref|XP_450518.1| putative oleosin [Oryza sativa (japonica cultivar-group)] >gnl|BL_ORD_ID|1563842 gi|51963736|ref|XP_506648.1| PREDICTED OSJNBb0024J13.29 gene product [Oryza sativa (japonica cultivar-group)] >gnl|BL_ORD_ID|1563842 gi|487	11681.m01394|LOC_Os09g15520|protein oleosin zm-i (oleosin 16 kda) (lipid body-associated major protein)(lipid body-associated protein l3) e_value:3.83316e-51	0.593	(0.2119 0.3575)	0.2119	0.3575
1956	SET4__C23_CLUSTER_24551	gi|30089732|gb|AAP20836.1| hypothetical protein Os03g31010 [Oryza sativa (japonica cultivar-group)] >gi|50916695|ref|XP_468744.1| unknown protein [Oryza sativa (japonica cultivar-group)] e_value:0	11669.m03127|LOC_Os03g31010|protein hypothetical protein e_value:0	0.204	(0.1051 0.5152)	0.1051	0.5152
1957	WT013_P03_CLUSTER_34606	gi|7269693|emb|CAB79641.1| ADP, ATP carrier-like protein [Arabidopsis thaliana] >gnl|BL_ORD_ID|1461248 gi|2842480|emb|CAA16877.1| ADP, ATP carrier-like protein [Arabidopsis thaliana] >gnl|BL_ORD_ID|1461248 gi|26451091|dbj|BAC42650.1| putative ADP,AT	11682.m02180|LOC_Os05g23720|protein adenine nucleotide translocator e_value:1.97804e-172	0.123	(0.1300 1.0575)	0.13	1.0575
1958	WT010_M20_CLUSTER_14408	gi|50872458|gb|AAT85058.1| putative c-type cytochrome synthesis protein [Oryza sativa (japonica cultivar-group)] >gnl|BL_ORD_ID|995627 gi|40539072|gb|AAR87329.1| putative c-type cytochrome biogenesis protein [Oryza sativa (japonica cultivar-	11669.m05639|LOC_Os03g55970|protein putative c-type cytochrome synthesis protein e_value:0	0.153	(0.1013 0.6620)	0.1013	0.662

PDF Complete
Your complimentary use period has ended. Thank you for using PDF Complete.
Click Here to upgrade to Unlimited Pages and Expanded Features

<table>
<tr><th></th><th></th><th></th><th></th><th></th><th></th><th></th><th></th></tr>
<tr><td>1959</td><td>WT009_L21_CLUSTER_2513</td><td>gi|21436363|gb|AAM51351.1| putative lysine and histidine specific transporter protein [Arabidopsis thaliana] >gnl|BL_ORD_ID|1208707 gi|17529318|gb|AAL38886.1| putative lysine and histidine specific transporter protein [Arabidopsis thaliana] >gnl|BL_</td><td>11686.m02916|LOC_Os12g30040|protein Transmembrane amino acid transporter protein e_value:0</td><td>0.162</td><td>(0.0786 0.4839)</td><td>0.0786</td><td>0.4839</td></tr>
<tr><td>1960</td><td>WT009_K15_CLUSTER_17007</td><td>gi|34903692|ref|NP_913193.1| P0489A05.10 [Oryza sativa (japonica cultivar-group)] e_value:2.47418e-142</td><td>11667.m01021|LOC_Os01g10540|protein expressed protein</td><td>0.474</td><td>(0.3673 0.7751)</td><td>0.3673</td><td>0.7751</td></tr>
<tr><td>1961</td><td>WT008_C04_CLUSTER_23354</td><td>gi|56409866|emb|CAI30081.1| glycosyltransferase [Triticum aestivum] e_value:0</td><td>11668.m02193|LOC_Os02g22650|protein expressed protein</td><td>0.303</td><td>(0.1459 0.4813)</td><td>0.1459</td><td>0.4813</td></tr>
<tr><td>1962</td><td>WT007_O11_CLUSTER_16855</td><td>gi|50913221|ref|XP_468018.1| putative beta-1,3-glucanase precursor [Oryza sativa (japonica cultivar-group)] >gnl|BL_ORD_ID|1583135 gi|51964434|ref|XP_507002.1| PREDICTED OJ1353_F08.18 gene product [Oryza sativa (japonica cultivar-group)] >gnl|BL_ORD</td><td>11668.m05220|LOC_Os02g53200|protein Glycosyl hydrolases family 17, putative e_value:0</td><td>0.246</td><td>(0.0818 0.3323)</td><td>0.0818</td><td>0.3323</td></tr>
<tr><td>1963</td><td>WT007_O06_CLUSTER_2532</td><td>gi|3415134|gb|AAC31614.1| Phyb1 [Pimpinella brachycarpa] e_value:2.00886e-88</td><td>11687.m03935|LOC_Os11g42000|protein Similar to Phyb1 e_value:1.50751e-121</td><td>0.117</td><td>(0.0726 0.6201)</td><td>0.0726</td><td>0.6201</td></tr>
<tr><td>1964</td><td>WT006_O02_CLUSTER_15996</td><td>gi|31433442|gb|AAP54955.1| putative flavin-containing monooxygenase [Oryza sativa (japonica cultivar-group)] >gnl|BL_ORD_ID|2012979 gi|37536732|ref|NP_922668.1| putative flavin-containing monooxygenase [Oryza sativa (japonica cultivar-group)] >gnl|B</td><td>11676.m03718|LOC_Os10g40570|protein putative flavin-containing monooxygenase e_value:1.3577e-165</td><td>0.227</td><td>(0.2353 1.0371)</td><td>0.2353</td><td>1.0371</td></tr>
<tr><td>1965</td><td>WT006_J11_CLUSTER_12024</td><td>gi|23506069|gb|AAN28894.1| At2g28370/T1B3.11 [Arabidopsis thaliana] >gnl|BL_ORD_ID|1268215 gi|21553379|gb|AAM62472.1| unknown [Arabidopsis thaliana] >gnl|BL_ORD_ID|1268215 gi|4432840|gb|AAD20689.1| expressed protein [Arabidopsis thaliana] >gnl|BL</td><td>11676.m01676|LOC_Os10g20250|protein expressed protein e_value:1.10778e-70</td><td>0.21</td><td>(0.1474 0.7035)</td><td>0.1474</td><td>0.7035</td></tr>
<tr><td>1966</td><td>WT005_O24_CLUSTER_11158</td><td>gi|50915540|ref|XP_468234.1| hypothetical protein [Oryza sativa (japonica cultivar-group)] >gnl|BL_ORD_ID|1583355 gi|47497144|dbj|BAD19193.1| hypothetical protein [Oryza sativa (japonica cultivar-group)] >gnl|BL_ORD_ID|1583355</td><td>11668.m05425|LOC_Os02g55050|protein F-box domain, putative e_value:8.94438e-109</td><td>0.324</td><td>(0.4404 1.3609)</td><td>0.4404</td><td>1.3609</td></tr>
<tr><td>1967</td><td>WT005_F10_CLUSTER_20371</td><td>gi|15239643|ref|NP_197410.1| heavy-metal-associated domain-containing protein [Arabidopsis thaliana] e_value:9.4879e-104</td><td>11686.m00437|LOC_Os12g05040|protein heavy metal-associated domain, putative e_value:0</td><td>0.192</td><td>(0.1033 0.5379)</td><td>0.1033</td><td>0.5379</td></tr>
<tr><td>1968</td><td>WT003_B04_CLUSTER_11041</td><td>gi|50898520|ref|XP_450048.1| CCAAT-box-binding transcription factor-like protein [Oryza sativa (japonica cultivar-group)] >gnl|BL_ORD_ID|1563346 gi|46806646|dbj|BAD17726.1| CCAAT-box-binding transcription factor-like protein [Oryza sativa (japonica cul</td><td>11681.m00188|LOC_Os09g02810|protein CBF/Mak21 family, putative e_value:0</td><td>0.241</td><td>(0.1476 0.6117)</td><td>0.1476</td><td>0.6117</td></tr>
<tr><td>1969</td><td>WT002_H20_CLUSTER_31552</td><td>gi|55775098|ref|XP_550475.1| putative cell survival CED-4-interacting protein MAC-1 [Oryza sativa (japonica cultivar-group)] >gnl|BL_ORD_ID|1752815 gi|55296176|dbj|BAD67894.1| putative cell survival CED-4-interacting protein MAC-1 [Oryza sativa (japoni</td><td>11680.m00105|LOC_Os06g01980|protein ATPase, AAA family, putative e_value:0</td><td>0.29</td><td>(0.1516 0.5230)</td><td>0.1516</td><td>0.523</td></tr>
<tr><td>1970</td><td>SET4_P21_CLUSTER_26313</td><td>gi|50924906|ref|XP_472796.1| OSJNBa0016O02.1 [Oryza sativa (japonica cultivar-group)] >gi|38606517|emb|CAE05991.3| OSJNBa0016O02.1 [Oryza sativa (japonica cultivar-group)] e_value:0</td><td>11670.m03724|LOC_Os04g39190|protein expressed protein e_value:0</td><td>0.299</td><td>(0.1391 0.4656)</td><td>0.1391</td><td>0.4656</td></tr>
<tr><td>1971</td><td>SET4_K17_CLUSTER_28216</td><td>gi|34895404|ref|NP_909045.1| unknown protein [Oryza sativa (japonica cultivar-group)] >gi|13486806|dbj|BAB40038.1| unknown protein [Oryza sativa (japonica cultivar-group)] e_value:2.73038e-32</td><td>11667.m00231|LOC_Os01g03190|protein expressed protein e_value:6.52665e-34</td><td>0.948</td><td>(0.7030 0.7417)</td><td>0.703</td><td>0.7417</td></tr>
<tr><td>1972</td><td>SET3_L04_CLUSTER_28987</td><td>gi|50911735|ref|XP_467275.1| unknown protein [Oryza sativa (japonica cultivar-group)] >gi|41053194|dbj|BAD08157.1| unknown protein [Oryza sativa (japonica cultivar-group)] e_value:3.91981e-40</td><td>11668.m04535|LOC_Os02g46840|protein hypothetical protein e_value:4.69416e-42</td><td>0.265</td><td>(0.0482 0.1818)</td><td>0.0482</td><td>0.1818</td></tr>
<tr><td>1973</td><td>SET1_E17_CLUSTER_31165</td><td>gi|50908635|ref|XP_465806.1| unknown protein [Oryza sativa (japonica cultivar-group)] >gi|48716420|dbj|BAD23028.1| unknown protein [Oryza sativa (japonica cultivar-group)] e_value:2.32247e-29</td><td>11668.m02788|LOC_Os02g29400|protein expressed protein e_value:2.84718e-31</td><td>0.354</td><td>(0.1846 0.5221)</td><td>0.1846</td><td>0.5221</td></tr>
<tr><td>1974</td><td>WT012_G04_CLUSTER_22236</td><td>gi|57899922|dbj|BAD87834.1| ABC-type transport system-like [Oryza sativa (japonica cultivar-group)] >gnl|BL_ORD_ID|972273 gi|57899704|dbj|BAD87424.1| ABC-type transport system-like [Oryza sativa (japonica cultivar-group)] e_value:8.81554e-164</td><td>11667.m07466|LOC_Os01g74280|protein mce related protein, putative e_value:1.99263e-165</td><td>0.186</td><td>(0.1088 0.5848)</td><td>0.1088</td><td>0.5848</td></tr>
<tr><td>1975</td><td>WT011_L10_CLUSTER_27961</td><td>gi|31433704|gb|AAP55183.1| putative peptide transporter [Oryza sativa (japonica cultivar-group)] >gnl|BL_ORD_ID|2013196 gi|37537188|ref|NP_922897.1| putative peptide transporter [Oryza sativa (japonica cultivar-group)] >gnl|BL_ORD_ID|2013196 gi|1203</td><td>11676.m03986|LOC_Os10g42900|protein putative peptide transporter e_value:0</td><td>0.448</td><td>(0.2105 0.4701)</td><td>0.2105</td><td>0.4701</td></tr>
<tr><td>1976</td><td>WT011_F08_CLUSTER_14966</td><td>gi|51535339|dbj|BAD38598.1| unknown protein [Oryza sativa (japonica cultivar-group)] e_value:6.97313e-125</td><td>11668.m00532|LOC_Os02g05870|protein expressed protein e_value:1.45152e-126</td><td>0.232</td><td>(0.1363 0.5865)</td><td>0.1363</td><td>0.5865</td></tr>
<tr><td>1977</td><td>WT011_B07_CLUSTER_17196</td><td>gi|50725143|dbj|BAD33760.1| putative cytochrome P450 [Oryza sativa (japonica cultivar-group)] e_value:0</td><td>11681.m03186|LOC_Os09g35940|protein Cytochrome P450</td><td>0.193</td><td>(0.1195 0.6184)</td><td>0.1195</td><td>0.6184</td></tr>
<tr><td>1978</td><td>WT008_C20_CLUSTER_34157</td><td>gi|50949055|ref|XP_493885.1| putative ATP-dependent RNA helicase [Oryza sativa] >gnl|BL_ORD_ID|1742077 gi|14719335|gb|AAK73153.1| putative ATP-dependent RNA helicase [Oryza sativa] e_value:0</td><td>11682.m00116|LOC_Os05g01990|protein ATP-dependent RNA helicase, putative e_value:0</td><td>0.129</td><td>(0.0713 0.5548)</td><td>0.0713</td><td>0.5548</td></tr>
<tr><td>1979</td><td>SET5_E18_CLUSTER_21050</td><td>gi|37700299|gb|AAR00589.1| putative NPH3 family protein [Oryza sativa (japonica cultivar-group)] >gi|51964272|ref|XP_506921.1| PREDICTED OSJNBa0034J04.14 gene product [Oryza sativa (japonica cultivar-group)] >gi|50901436|ref|XP_463151.1| putative NP</td><td>11669.m01028|LOC_Os03g10800|protein transposon protein, putative, mutator sub-class e_value:0</td><td>0.173</td><td>(0.1021 0.5914)</td><td>0.1021</td><td>0.5914</td></tr>
<tr><td>1980</td><td>WT009_M17_CLUSTER_26043</td><td>gi|50947041|ref|XP_483048.1| putative potential copper-transporting ATPase [Oryza sativa (japonica cultivar-group)] >gnl|BL_ORD_ID|1609287 gi|42408181|dbj|BAD09318.1| putative potential copper-transporting ATPase [Oryza sativa (japonica cultivar-group)</td><td>11674.m03780|LOC_Os08g37950|protein copper-translocating P-type ATPase, putative e_value:0</td><td>0.173</td><td>(0.0830 0.4803)</td><td>0.083</td><td>0.4803</td></tr>
<tr><td>1981</td><td>WT005_E14_CLUSTER_26364</td><td>gi|48596907|dbj|BAD22766.1| LEA protein [Bromus inermis] e_value:9.71903e-83</td><td>11682.m04475|LOC_Os05g46480|protein LEA protein - rice e_value:1.9224e-54</td><td>0.707</td><td>(0.2451 0.3468)</td><td>0.2451</td><td>0.3468</td></tr>
<tr><td>1982</td><td>SET1_E10_CLUSTER_8018</td><td>gi|34897148|ref|NP_909920.1| unknown protein [Oryza sativa (japonica cultivar-group)] >gi|28209503|gb|AAO37521.1| unknown protein [Oryza sativa (japonica cultivar-group)] e_value:0</td><td>11669.m05948|LOC_Os03g58640|protein expressed protein e_value:0</td><td>0.351</td><td>(0.1920 0.5470)</td><td>0.192</td><td>0.547</td></tr>
<tr><td>1983</td><td>WT009_O13_CLUSTER_19986</td><td>gi|50920225|ref|XP_470473.1| putative H-box binding protein, KAP2, with alternative splicing isoforms [Oryza sativa (japonica cultivar-group)] >gnl|BL_ORD_ID|1535464 gi|41393254|gb|AAS01977.1| putative H-box binding protein, KAP2, with alternative spli</td><td>11669.m06563|LOC_Os03g63920|protein putative H-box binding protein, KAP2, with alternative splicing isoforms e_value:0</td><td>0.249</td><td>(0.1134 0.4548)</td><td>0.1134</td><td>0.4548</td></tr>
<tr><td>1984</td><td>WT005_D08_CLUSTER_27511</td><td>gi|15231945|ref|NP_188106.1| DNA-binding protein-related [Arabidopsis thaliana] e_value:2.54143e-32</td><td>11682.m04658|LOC_Os05g48650|protein Similar to tumor-related protein e_value:2.30883e-99</td><td>0.393</td><td>(0.1129 0.2874)</td><td>0.1129</td><td>0.2874</td></tr>
<tr><td>1985</td><td>SET6_O15_CLUSTER_21855</td><td>gi|50906727|ref|XP_464852.1| putative pre-mRNA splicing factor [Oryza sativa (japonica cultivar-group)] >gi|51963962|ref|XP_506764.1| PREDICTED OJ1113_G05.32 gene product [Oryza sativa (japonica cultivar-group)] >gi|47497696|dbj|BAD19762.1| putative</td><td>11686.m03786|LOC_Os12g38430|protein RNA recognition motif. (a.k.a. RRM, RBD, or RNP domain), putative e_value:5.37369e-</td><td>0.208</td><td>(0.1048 0.5033)</td><td>0.1048</td><td>0.5033</td></tr>
<tr><td>1986</td><td>SET1_G23_CLUSTER_29189</td><td>gi|50940297|ref|XP_479676.1| unknown protein [Oryza sativa (japonica cultivar-group)] >gi|50725712|dbj|BAD33178.1| unknown protein [Oryza sativa (japonica cultivar-group)] e_value:2.64472e-15</td><td>11674.m00041|LOC_Os08g01370|protein expressed protein e_value:3.00919e-17</td><td>0.569</td><td>(0.4589 0.8071)</td><td>0.4589</td><td>0.8071</td></tr>
<tr><td>1987</td><td>SET1_B08_CLUSTER_12285</td><td>gi|21724|emb|CAA48350.1| Ec protein, Metallothionein(class II) [Triticum aestivum] >gi|21735|emb|CAA48349.1| Ec protein, Metallothionein(class II) [Triticum aestivum] >gi|232025|sp|P30569|EC1_WHEAT EC protein I/II (Zinc-metallothionein class II) &gt</td><td>11676.m03612|LOC_Os10g39610|protein putative zinc-metallothionein e_value:1.59456e-37</td><td>0.371</td><td>(0.1313 0.3537)</td><td>0.1313</td><td>0.3537</td></tr>
<tr><td>1988</td><td>SET2_A22_CLUSTER_17868</td><td>gi|34895374|ref|NP_909030.1| putative histidine-rich Ca2+-binding protein [Oryza sativa (japonica cultivar-group)] >gi|11034640|dbj|BAB17164.1| putative histidine-rich Ca2+-binding protein [Oryza sativa (japonica cultivar-group)]</td><td>11667.m00216|LOC_Os01g03060|protein hypothetical protein e_value:3.11446e-101</td><td>0.174</td><td>(0.1568 0.9012)</td><td>0.1568</td><td>0.9012</td></tr>
<tr><td>1989</td><td>SET2_E04_CLUSTER_13453</td><td>gi|34908378|ref|NP_915536.1| P0529E05.15 [Oryza sativa (japonica cultivar-group)] e_value:0</td><td>11667.m06350|LOC_Os01g63420|protein Leucine Rich Repeat, putative e_value:0</td><td>0.129</td><td>(0.0708 0.5474)</td><td>0.0708</td><td>0.5474</td></tr>
<tr><td>1990</td><td>WT013_K12_CLUSTER_27580</td><td>gi|38345038|emb|CAD41494.2| OSJNBa0029H02.22 [Oryza sativa (japonica cultivar-group)] >gnl|BL_ORD_ID|1521521 gi|50926183|ref|XP_473066.1| OSJNBa0029H02.22 [Oryza sativa (japonica cultivar-group)] e_value:2.98594e-84</td><td>11670.m04076|LOC_Os04g42340|protein expressed protein e_value:4.76017e-86</td><td>0.22</td><td>(0.1096 0.4972)</td><td>0.1096</td><td>0.4972</td></tr>
<tr><td>1991</td><td>WT012_E21_CLUSTER_11075</td><td>gi|34897676|ref|NP_910184.1| putative ribosomal RNA apurinic site specific lyase [Oryza sativa (japonica cultivar-group)] >gnl|BL_ORD_ID|1524938 gi|24414168|dbj|BAC22411.1| putative ribosomal RNA apurinic site specific lyase [Oryza sativa (japonica cul</td><td>11673.m01816|LOC_Os07g18510|protein F-box domain, putative e_value:1.99955e-87</td><td>0.59</td><td>(0.4699 0.7963)</td><td>0.4699</td><td>0.7963</td></tr>
<tr><td>1992</td><td>WT012_I06_CLUSTER_19559</td><td>gi|50918895|ref|XP_469844.1| hypothetical protein [Oryza sativa (japonica cultivar-group)] >gnl|BL_ORD_ID|1586223 gi|14488359|gb|AAK63926.1| hypothetical protein [Oryza sativa (japonica cultivar-group)] e_value:7.53705e-144</td><td>11686.m03837|LOC_Os12g38850|protein hypothetical protein e_value:0</td><td>0.138</td><td>(0.0814 0.5902)</td><td>0.0814</td><td>0.5902</td></tr>
<tr><td>1993</td><td>SET2_F10_CLUSTER_17731</td><td>gi|50908341|ref|XP_465659.1| unknown protein [Oryza sativa (japonica cultivar-group)] >gi|47848606|dbj|BAD22455.1| unknown protein [Oryza sativa (japonica cultivar-group)] >gi|47848158|dbj|BAD21939.1| unknown protein [Oryza sativa (japonica cultivar</td><td>11668.m02642|LOC_Os02g27960|protein expressed protein e_value:2.18139e-50</td><td>0.266</td><td>(0.1345 0.5051)</td><td>0.1345</td><td>0.5051</td></tr>
<tr><td>1994</td><td>WT007_P20_CLUSTER_32747</td><td>gi|56961773|gb|AAW31855.1| tousled-like protein kinase [Triticum aestivum] e_value:0</td><td>11669.m05461|LOC_Os03g53880|protein putative protein kinase e_value:0</td><td>0.115</td><td>(0.0570 0.4969)</td><td>0.057</td><td>0.4969</td></tr>
<tr><td>1995</td><td>WT004_D20_CLUSTER_3453</td><td>gi|50902220|ref|XP_463543.1| OsNAC6 protein [Oryza sativa (japonica cultivar-group)] >gnl|BL_ORD_ID|1558798 gi|20161457|dbj|BAB90381.1| OsNAC6 protein [Oryza sativa (japonica cultivar-group)] >gnl|BL_ORD_ID|1558798 gi|13272281|gb|AAK17067.1| NAC6 [O</td><td>11667.m06643|LOC_Os01g66120|protein No apical meristem (NAM) protein, putative e_value:5.62318e-137</td><td>0.309</td><td>(0.0923 0.2986)</td><td>0.0923</td><td>0.2986</td></tr>
<tr><td>1996</td><td>WT013_P11_CLUSTER_13454</td><td>gi|3608154|gb|AAC36187.1| unknown protein [Arabidopsis thaliana] >gnl|BL_ORD_ID|939453 gi|25408371|pir||C84767 hypothetical protein At2g35330 [imported] - Arabidopsis thaliana e_value:2.53445e-99</td><td>11681.m01853|LOC_Os09g20550|protein apoptosis inhibitor homolog T4I9.12 - Arabidopsis thaliana e_value:0</td><td>0.24</td><td>(0.1571 0.6558)</td><td>0.1571</td><td>0.6558</td></tr>
<tr><td>1997</td><td>WT009_F04_CLUSTER_26306</td><td>gi|50907513|ref|XP_465245.1| Kelch repeat containing F-box protein family-like [Oryza sativa (japonica cultivar-group)] >gnl|BL_ORD_ID|1569301 gi|51964012|ref|XP_506789.1| PREDICTED P0572D06.16 gene product [Oryza sativa (japonica cultivar-group)] ></td><td>11668.m02032|LOC_Os02g21110|protein hypothetical protein e_value:0</td><td>0.209</td><td>(0.1033 0.4950)</td><td>0.1033</td><td>0.495</td></tr>
<tr><td>1998</td><td>WT009_C12_CLUSTER_11159</td><td>gi|31193911|gb|AAP44746.1| putative extensin [Oryza sativa (japonica cultivar-group)] >gnl|BL_ORD_ID|1917590 gi|50920281|ref|XP_470501.1| putative extensin [Oryza sativa (japonica cultivar-group)] e_value:5.48835e-101</td><td>11669.m06590|LOC_Os03g64190|protein putative extensin e_value:1.32889e-102</td><td>0.365</td><td>(0.3100 0.8486)</td><td>0.31</td><td>0.8486</td></tr>
<tr><td>1999</td><td>SET1_G02_CLUSTER_34466</td><td>gi|34912090|ref|NP_917392.1| putative mannan endo-1,4-beta-mannosidase [Oryza sativa (japonica cultivar-group)] >gi|20521230|dbj|BAB91747.1| putative (1-4)-beta-mannan endohydrolase [Oryza sativa (japonica cultivar-group)] e_value:0</td><td>11667.m04579|LOC_Os01g47400|protein expressed protein e_value:2.38756e-169</td><td>0.409</td><td>(0.1445 0.3532)</td><td>0.1445</td><td>0.3532</td></tr>
<tr><td>2000</td><td>SET3_A20_CLUSTER_14926</td><td>gi|51091152|dbj|BAD35847.1| gb protein-like [Oryza sativa (japonica cultivar-group)] e_value:9.5511e-75</td><td>11680.m01012|LOC_Os06g10440|protein expressed protein e_value:1.84804e-76</td><td>0.267</td><td>(0.0888 0.3321)</td><td>0.0888</td><td>0.3321</td></tr>
<tr><td>2001</td><td>WT010_F23_CLUSTER_20985</td><td>gi|27476082|gb|AAO17013.1| Putative thymidine kinase [Oryza sativa (japonica cultivar-group)] e_value:1.46248e-104</td><td>11669.m00142|LOC_Os03g02200|protein thymidine kinase, putative e_value:2.83793e-106</td><td>0.218</td><td>(0.1375 0.6319)</td><td>0.1375</td><td>0.6319</td></tr>
<tr><td>2002</td><td>SET5_I22_CLUSTER_19600</td><td>gi|50907533|ref|XP_465255.1| putative N7 protein [Oryza sativa (japonica cultivar-group)] >gi|46389931|dbj|BAD15715.1| putative N7 protein [Oryza sativa (japonica cultivar-group)] e_value:3.76427e-124</td><td>11668.m02045|LOC_Os02g21240|protein N7-like protein e_value:8.73731e-126</td><td>0.212</td><td>(0.1835 0.8671)</td><td>0.1835</td><td>0.8671</td></tr>
</table>

PDF Complete
Your complimentary use period has ended. Thank you for using PDF Complete.
Click Here to upgrade to Unlimited Pages and Expanded Features

2003	WT006_F24_CLUSTER_23218	gi|50933409|ref|XP_476232.1| putative ubiquitin-onjugating enzyme E2 [Oryza sativa (japonica cultivar-group)] >gnl|BL_ORD_ID|1600959 gi|50300518|gb|AAT73660.1| putative ubiquitin-onjugating enzyme E2 [Oryza sativa (japonica cultivar-group)] >gnl|BL_	11682.m01320|LOC_Os05g14300|protein Ubiquitin-conjugating enzyme, putative e_value:0	0.239	(0.1670 0.6972)	0.167	0.6972
2004	SET2_B10_CLUSTER_22846	gi|9758251|dbj|BAB08750.1| unnamed protein product [Arabidopsis thaliana] >gi|15241359|ref|NP_199917.1| expressed protein [Arabidopsis thaliana] e_value:1.83833e-48	11687.m02989|LOC_Os11g32480|protein expressed protein e_value:9.55957e-75	0.247	(0.1025 0.4148)	0.1025	0.4148
2005	WT002_D06_CLUSTER_25516	gi|30725438|gb|AAP37741.1| At3g44600 [Arabidopsis thaliana] >gnl|BL_ORD_ID|1853602 gi|17065154|gb|AAL32731.1| putative protein [Arabidopsis thaliana] >gnl|BL_ORD_ID|1853602 gi|22331588|ref|NP_190046.2| peptidyl-prolyl cis-trans isomerase cyclophilin	11674.m04478|LOC_Os08g44330|protein At3g44600 e_value:0	0.081	(0.0421 0.5210)	0.0421	0.521
2006	SET4_C05_CLUSTER_23270	gi|50921317|ref|XP_471019.1| OSJNBb0060M15.9 [Oryza sativa (japonica cultivar-group)] >gi|38345283|emb|CAE03197.2| OSJNBb0060M15.9 [Oryza sativa (japonica cultivar-group)] >gi|38345219|emb|CAE01640.2| OSJNBb0021I10.2 [Oryza sativa (japonica cultivar	11670.m00119|LOC_Os04g02150|protein hypothetical protein e_value:0	0.188	(0.1250 0.6646)	0.125	0.6646
2007	SET6_K07_CLUSTER_15380	gi|50920111|ref|XP_470416.1| putative transcription factor [Oryza sativa (japonica cultivar-group)] >gi|27573340|gb|AAO20058.1| putative transcription factor [Oryza sativa (japonica cultivar-group)] e_value:5.84559e-77	11669.m06499|LOC_Os03g63400|protein putative transcription factor e_value:8.44092e-79	0.059	(0.0281 0.4743)	0.0281	0.4743
2008	WT010_D07_CLUSTER_7022	gi|53792525|dbj|BAD53489.1| putative decoy [Oryza sativa (japonica cultivar-group)] e_value:2.9883e-115	11680.m01909|LOC_Os06g19640|protein At1g14620/T5E21_15 e_value:6.24635e-117	0.199	(0.0834 0.4198)	0.0834	0.4198
2009	WT005_D22_CLUSTER_1475	gi|50920709|ref|XP_470715.1| putative nucleic acid binding protein [Oryza sativa] >gnl|BL_ORD_ID|1511086 gi|18921324|gb|AAL82529.1| putative nucleic acid binding protein [Oryza sativa] e_value:0	11669.m02636|LOC_Os03g25970|protein putative nucleic acid binding protein e_value:0	0.167	(0.0809 0.4851)	0.0809	0.4851
2010	WT006_J21_CLUSTER_12791	gi|50931097|ref|XP_475076.1| 'unknown protein, contains ACT domain, PF01842' [Oryza sativa (japonica cultivar-group)] >gnl|BL_ORD_ID|1517618 gi|45680438|gb|AAS75239.1| unknown protein [Oryza sativa (japonica cultivar-group)] e_value:0	11682.m00141|LOC_Os05g02220|protein ACT domain, putative e_value:0	0.299	(0.1894 0.6343)	0.1894	0.6343
2011	WT002_J03_CLUSTER_1654	gi|51535961|dbj|BAD38042.1| putative protein phosphatase 2C [Oryza sativa (japonica cultivar-group)] e_value:2.23082e-142	11668.m00505|LOC_Os02g05630|protein protein phosphatase 2C e_value:1.32288e-143	0.209	(0.1505 0.7203)	0.1505	0.7203
2012	WT004_I04_CLUSTER_21337	gi|50906041|ref|XP_464509.1| putative SEC14 cytosolic factor (SEC14) [Oryza sativa (japonica cultivar-group)] >gnl|BL_ORD_ID|1566796 gi|46390380|dbj|BAD15844.1| putative SEC14 cytosolic factor (SEC14) [Oryza sativa (japonica cultivar-group)] e_value:3.	11668.m00999|LOC_Os02g10650|protein CRAL/TRIO, N-terminus, putative e_value:8.02853e-172	0.097	(0.0592 0.6082)	0.0592	0.6082
2013	SET4_I13_CLUSTER_16071	gi|34911930|ref|NP_917312.1| OSJNBb0006H05.28 [Oryza sativa (japonica cultivar-group)] >gi|20161527|dbj|BAB90450.1| hypothetical protein [Oryza sativa (japonica cultivar-group)] e_value:5.55549e-28	11667.m04492|LOC_Os01g46600|protein expressed protein e_value:9.34648e-30	0.63	(0.1378 0.2187)	0.1378	0.2187
2014	WT013_G24_CLUSTER_17102	gi|53792527|dbj|BAD53491.1| WD-40 repeat protein-like [Oryza sativa (japonica cultivar-group)] e_value:0	11680.m01911|LOC_Os06g19660|protein expressed protein	0.243	(0.1177 0.4842)	0.1177	0.4842
2015	WT010_K02_CLUSTER_19033	gi|34901974|ref|NP_912333.1| unknown protein [Oryza sativa (japonica cultivar-group)] >gnl|BL_ORD_ID|1588788 gi|29893571|gb|AAP06825.1| unknown protein [Oryza sativa (japonica cultivar-group)] >gnl|BL_ORD_ID|1588788 gi|62899872|sp|Q84QC0|ASCL3_ORYSA	11669.m01572|LOC_Os03g15750|protein Longevity-assurance protein (LAG1), putative e_value:2.28408e-139	0.15	(0.0572 0.3822)	0.0572	0.3822
2016	SET4_L23_CLUSTER_20526	gi|50940155|ref|XP_479605.1| bZIP transcription factor [Oryza sativa (japonica cultivar-group)] >gi|33146487|dbj|BAC79596.1| bZIP transcription factor [Oryza sativa (japonica cultivar-group)] >gi|50509157|dbj|BAD30297.1| bZIP transcription factor [O	11673.m04851|LOC_Os07g48820|protein expressed protein e_value:3.17508e-171	0.052	(0.0388 0.7518)	0.0388	0.7518
2017	WT012_G06_CLUSTER_30774	gi|50939565|ref|XP_479310.1| unknown protein [Oryza sativa (japonica cultivar-group)] >gnl|BL_ORD_ID|2367841 gi|23237912|dbj|BAC16486.1| unknown protein [Oryza sativa (japonica cultivar-group)] >gnl|BL_ORD_ID|2367841 gi|50509934|dbj|BAD30255.1| unkn	11673.m04410|LOC_Os07g44820|protein expressed protein e_value:2.39902e-178	0.278	(0.1503 0.5400)	0.1503	0.54
2018	WT009__C16_CLUSTER_32748	gi|34903150|ref|NP_912922.1| unnamed protein product [Oryza sativa (japonica cultivar-group)] >gnl|BL_ORD_ID|1752409 gi|6721539|dbj|BAA89569.1| unknown protein [Oryza sativa (japonica cultivar-group)] >gnl|BL_ORD_ID|1752409	11667.m01641|LOC_Os01g16330|protein UBA/TS-N domain, putative e_value:3.8021e-130	0.2	(0.0906 0.4521)	0.0906	0.4521
2019	WT005_E05_CLUSTER_3165	gi|56201873|dbj|BAD73323.1| unknown protein [Oryza sativa (japonica cultivar-group)] >gnl|BL_ORD_ID|542293 gi|56201766|dbj|BAD73216.1| unknown protein [Oryza sativa (japonica cultivar-group)] e_value:8.98073e-36	11667.m00969|LOC_Os01g10080|protein hypothetical protein e_value:2.0202e-37	0.426	(0.5344 1.2549)	0.5344	1.2549
2020	SET5_B11_CLUSTER_10506	gi|34897074|ref|NP_909883.1| putative inosine monophosphate dehydrogenase [Oryza sativa (japonica cultivar-group)] >gi|12957707|gb|AAK09225.1| putative inosine monophosphate dehydrogenase [Oryza sativa (japonica cultivar-group)] e_value:0	11669.m05732|LOC_Os03g56800|protein putative inosine monophosphate dehydrogenase e_value:0	0.113	(0.0574 0.5071)	0.0574	0.5071
2021	WT013_P13_CLUSTER_20805	gi|51090793|dbj|BAD35271.1| putative ubiquitin conjugating enzyme [Oryza sativa (japonica cultivar-group)] e_value:4.58767e-122	11680.m00892|LOC_Os06g09330|protein ubiquitin conjugating enzyme 2 e_value:8.74533e-124	0.132	(0.0656 0.4955)	0.0656	0.4955
2022	SET5_H12_CLUSTER_3029	gi|50725343|dbj|BAD34415.1| unknown protein [Oryza sativa (japonica cultivar-group)] e_value:2.75025e-109	11681.m03061|LOC_Os09g33800|protein expressed protein e_value:4.87941e-111	0.048	(0.0316 0.6550)	0.0316	0.655
2023	WT007_I08_CLUSTER_32474	gi|50510178|dbj|BAD31272.1| ARF GAP-like zinc finger-containing protein-like [Oryza sativa (japonica cultivar-group)] e_value:0	11673.m03651|LOC_Os07g37650|protein Putative GTP-ase activating protein for Arf e_value:0	0.331	(0.1345 0.4063)	0.1345	0.4063
2024	WT013_A07_CLUSTER_32706	gi|50899610|ref|XP_450593.1| putative holocarboxylase synthetase [Oryza sativa (japonica cultivar-group)] >gnl|BL_ORD_ID|1563876 gi|48716651|dbj|BAD23319.1| putative holocarboxylase synthetase [Oryza sativa (japonica cultivar-group)] e_value:4.26169e-9	11681.m01760|LOC_Os09g19640|protein expressed protein e_value:7.23873e-93	0.19	(0.0747 0.3934)	0.0747	0.3934
2025	WT003_D23_CLUSTER_31872	gi|20465603|gb|AAM20284.1| putative WD-repeat membrane protein [Arabidopsis thaliana] >gnl|BL_ORD_ID|1208354 gi|16604679|gb|AAL24132.1| putative WD-repeat membrane protein [Arabidopsis thaliana] >gnl|BL_ORD_ID|1208354 gi|18412751|ref|NP_567275.1| tr	11680.m04291|LOC_Os06g43690|protein WD-repeat membrane protein, putative e_value:0	0.123	(0.0674 0.5463)	0.0674	0.5463
2026	WT004_L14_CLUSTER_34490	gi|34901604|ref|NP_912148.1| putative EBNA1-binding protein homolog; Ebp2p [Oryza sativa (japonica cultivar-group)] >gnl|BL_ORD_ID|1526755 gi|29027795|dbj|BAC65931.1| putative EBNA1-binding protein homolog; Ebp2p [Oryza sativa (japonica cultivar-group)	11673.m02774|LOC_Os07g29320|protein Eukaryotic rRNA processing protein EBP2 e_value:3.19303e-132	0.153	(0.0983 0.6440)	0.0983	0.644
2027	WT002_D24_CLUSTER_34809	gi|50252084|dbj|BAD28014.1| putative U2-associated SR140 protein [Oryza sativa (japonica cultivar-group)] e_value:0	11668.m00805|LOC_Os02g08360|protein Surp module, putative e_value:0	0.174	(0.0764 0.4398)	0.0764	0.4398
2028	WT005__C10_CLUSTER_20708	gi|52075900|dbj|BAD45846.1| unknown protein [Oryza sativa (japonica cultivar-group)] >gnl|BL_ORD_ID|253398 gi|52077382|dbj|BAD46422.1| unknown protein [Oryza sativa (japonica cultivar-group)] e_value:1.78483e-38	11680.m04433|LOC_Os06g45070|protein At2g15910 e_value:2.6152e-40	0.296	(0.0246 0.0831)	0.0246	0.0831
2029	WT002_A08_CLUSTER_21535	gi|21281026|gb|AAM44953.1| unknown protein [Arabidopsis thaliana] >gnl|BL_ORD_ID|1208469 gi|14334432|gb|AAK59414.1| unknown protein [Arabidopsis thaliana] >gnl|BL_ORD_ID|1208469 gi|20196948|gb|AAB84345.2| expressed protein [Arabidopsis thaliana] &gt	11669.m02336|LOC_Os03g22690|protein Similar to protein at2g41620. [mouse-ear cress e_value:0	0.114	(0.0466 0.4100)	0.0466	0.41
2030	WT004_J11_CLUSTER_15861	gi|50929813|ref|XP_474434.1| OSJNBa0070M12.12 [Oryza sativa (japonica cultivar-group)] >gnl|BL_ORD_ID|1594623 gi|38345829|emb|CAD41934.2| OSJNBa0070M12.12 [Oryza sativa (japonica cultivar-group)] e_value:1.86788e-110	11670.m05850|LOC_Os04g59040|protein inorganic pyrophosphatase, putative e_value:4.58463e-112	0.034	(0.0211 0.6142)	0.0211	0.6142
2031	SET6_M09_CLUSTER_34131	gi|16755057|gb|AAL26702.1| 68 kDa protein HP68 [Triticum aestivum] e_value:0	11687.m03136|LOC_Os11g34350|protein ABC transporter, putative e_value:0	0.043	(0.0230 0.5300)	0.023	0.53
2032	WT012_J19_CLUSTER_32845	gi|50938507|ref|XP_478781.1| putative potassium channel regulatory factor [Oryza sativa (japonica cultivar-group)] >gnl|BL_ORD_ID|1529514 gi|34394217|dbj|BAC84669.1| putative potassium channel regulatory factor [Oryza sativa (japonica cultivar-	11673.m03735|LOC_Os07g38420|protein Prp18 domain, putative e_value:0	0.179	(0.0836 0.4675)	0.0836	0.4675
2033	WT013_A11_CLUSTER_31982	gi|50938165|ref|XP_478610.1| putative bHLH protein [Oryza sativa (japonica cultivar-group)] >gnl|BL_ORD_ID|1528503 gi|32352190|dbj|BAC78588.1| hypothetical protein [Oryza sativa (japonica cultivar-group)] >gnl|BL_ORD_ID|1528503	11673.m03465|LOC_Os07g35870|protein Helix-loop-helix DNA-binding domain, putative e_value:1.11306e-108	0.189	(0.1151 0.6076)	0.1151	0.6076
2034	WT003_G22_CLUSTER_19881	gi|50897258|gb|AAT85768.1| At3g10300 [Arabidopsis thaliana] >gnl|BL_ORD_ID|1001689 gi|17064844|gb|AAL32576.1| Unknown protein [Arabidopsis thaliana] >gnl|BL_ORD_ID|1001689 gi|22330957|ref|NP_187641.2| calcium-binding EF hand family protein [Arabidop	11687.m00363|LOC_Os11g04480|protein expressed protein e_value:3.96499e-150	0.16	(0.0656 0.4100)	0.0656	0.41
2035	SET1_L07_CLUSTER_11959	gi|55769719|ref|XP_549849.1| putative ring-box protein [Oryza sativa (japonica cultivar-group)] >gi|52076230|dbj|BAD44884.1| putative ring-box protein [Oryza sativa (japonica cultivar-group)] >gi|52076191|dbj|BAD44845.1| putative ring-box protein [O	11667.m00073|LOC_Os01g01700|protein ZYP protein e_value:4.68064e-65	0.063	(0.0336 0.5320)	0.0336	0.532
2036	SET1_J08_CLUSTER_31852	gi|50511435|gb|AAT77358.1| unknown protein [Oryza sativa (japonica cultivar-group)] e_value:5.8107e-81	11682.m00824|LOC_Os05g08570|protein AUX/IAA family e_value:9.14937e-83	0.312	(0.0958 0.3070)	0.0958	0.307
2037	WT012_M02_CLUSTER_28393	gi|53982672|gb|AAV25651.1| unknown protein [Oryza sativa (japonica cultivar-group)] >gnl|BL_ORD_ID|1317331 gi|48475174|gb|AAT44243.1| unknown protein [Oryza sativa (japonica cultivar-group)] e_value:0	11682.m03277|LOC_Os05g34760|protein hypothetical protein e_value:0	0.405	(0.2211 0.5455)	0.2211	0.5455
2038	WT006_P06_CLUSTER_24540	gi|53791916|dbj|BAD54038.1| squamosa promoter binding protein 2-like [Oryza sativa (japonica cultivar-group)] e_value:1.16666e-135	11668.m00405|LOC_Os02g04680|protein SBP domain, putative e_value:2.64166e-172	0.329	(0.1901 0.5786)	0.1901	0.5786
2039	WT012_F22_CLUSTER_20505	gi|15451579|gb|AAK98703.1| Putative PAP-specific phosphatase [Oryza sativa] e_value:0	11668.m05542|LOC_Os02g56170|protein 3'(2'),5'-bisphosphate nucleotidase e_value:0	0.179	(0.0989 0.5516)	0.0989	0.5516
2040	WT013_N18_CLUSTER_29193	gi|48927668|gb|AAT47546.1| SET domain protein [Triticum aestivum] e_value:0	11667.m05923|LOC_Os01g59620|protein YDG/SRA domain, putative e_value:0	0.364	(0.1567 0.4302)	0.1567	0.4302
2041	WT005_B04_CLUSTER_32052	gi|50943775|ref|XP_481415.1| putative chloride channel [Oryza sativa (japonica cultivar-group)] >gnl|BL_ORD_ID|1604689 gi|35215062|dbj|BAC92420.1| putative chloride channel protein [Oryza sativa (japonica cultivar-group)] >gnl|BL_ORD_ID|1604689 gi|3	11674.m01983|LOC_Os08g20570|protein putative chloride channel e_value:0	0.232	(0.0689 0.2967)	0.0689	0.2967
2042	SET3_B24_CLUSTER_31268	gi|21594566|gb|AAM66022.1| unknown [Arabidopsis thaliana] >gi|7270188|emb|CAB77803.1| predicted protein of unknown function [Arabidopsis thaliana] >gi|15236259|ref|NP_192227.1| expressed protein [Arabidopsis thaliana] >gi|4262146|gb|AAD14446.1| p	11686.m03452|LOC_Os12g35270|protein expressed protein e_value:3.28735e-80	0.263	(0.1231 0.4680)	0.1231	0.468
2043	SET2_F04_CLUSTER_23988	gi|34904114|ref|NP_913404.1| unnamed protein product [Oryza sativa (japonica cultivar-group)] >gi|7573622|dbj|BAA94531.1| unknown protein [Oryza sativa (japonica cultivar-group)] e_value:1.59802e-17	11667.m01199|LOC_Os01g12270|protein hypothetical protein e_value:1.49565e-19	0.584	(0.1793 0.3069)	0.1793	0.3069
2044	WT011_I18_CLUSTER_32162	gi|52076930|dbj|BAD45941.1| putative cyclic nucleotide gated channel homolog [Oryza sativa (japonica cultivar-group)] e_value:0	11681.m03459|LOC_Os09g38580|protein cation channel family protein, putative e_value:0	0.124	(0.0983 0.7950)	0.0983	0.795

PDF Complete
Your complimentary use period has ended. Thank you for using PDF Complete.
Click Here to upgrade to Unlimited Pages and Expanded Features

<table>
<tr><th></th><th></th><th></th><th></th><th></th><th></th><th></th><th></th></tr>
<tr><td>2045</td><td>WT012_K11_CLUSTER_33966</td><td>gi|34906386|ref|NP_914540.1| P0710E05.5 [Oryza sativa (japonica cultivar-group)] e_value:7.85732e-15</td><td>11686.m00743|LOC_Os12g07890|protein hypothetical protein e_value:2.78091e-33</td><td>0.508</td><td>(0.7886 1.5538)</td><td>0.7886</td><td>1.5538</td></tr>
<tr><td>2046</td><td>WT012_B15_CLUSTER_33824</td><td>gi|50907169|ref|XP_465073.1| unknown protein [Oryza sativa (japonica cultivar-group)] >gnl|BL_ORD_ID|1567670 gi|47848322|dbj|BAD22185.1| unknown protein [Oryza sativa (japonica cultivar-group)] e_value:0</td><td>11668.m01844|LOC_Os02g19330|protein expressed protein e_value:0</td><td>0.24</td><td>(0.1138 0.4738)</td><td>0.1138</td><td>0.4738</td></tr>
<tr><td>2047</td><td>SET6_K19_CLUSTER_28</td><td>gi|51535668|dbj|BAD37640.1| dihydrodipicolinate reductase-like [Oryza sativa (japonica cultivar-group)] e_value:5.30665e-160</td><td>11668.m02231|LOC_Os02g24020|protein dihydrodipicolinate reductase, putative e_value:1.36614e-161</td><td>0.172</td><td>(0.0842 0.4896)</td><td>0.0842</td><td>0.4896</td></tr>
<tr><td>2048</td><td>WT013_O23_CLUSTER_25033</td><td>gi|34899120|ref|NP_910906.1| putative adenosine deaminase [Oryza sativa (japonica cultivar-group)] >gnl|BL_ORD_ID|1533418 gi|22831206|dbj|BAC16064.1| putative adenosine deaminase [Oryza sativa (japonica cultivar-group)] e_value:0</td><td>11673.m04598|LOC_Os07g46630|protein Adenosine/AMP deaminase e_value:0</td><td>0.122</td><td>(0.0733 0.6008)</td><td>0.0733</td><td>0.6008</td></tr>
<tr><td>2049</td><td>SET5_C14_CLUSTER_19192</td><td>gi|50938509|ref|XP_478782.1| putative WD repeat domain 5 protein [Oryza sativa (japonica cultivar-group)] >gi|34394218|dbj|BAC84670.1| putative WD repeat domain 5 protein [Oryza sativa (japonica cultivar-group)] e_value:1.83051e-168</td><td>11673.m03736|LOC_Os07g38430|protein expressed protein e_value:4.50507e-170</td><td>0.175</td><td>(0.0733 0.4182)</td><td>0.0733</td><td>0.4182</td></tr>
<tr><td>2050</td><td>WT012_I19_CLUSTER_30775</td><td>gi|38346638|emb|CAD40740.2| OSJNBa0072D21.8 [Oryza sativa (japonica cultivar-group)] >gnl|BL_ORD_ID|2018364 gi|50923769|ref|XP_472245.1| OSJNBa0072D21.8 [Oryza sativa (japonica cultivar-group)] e_value:0</td><td>11670.m02983|LOC_Os04g31320|protein SWIB/MDM2 domain, putative e_value:0</td><td>0.179</td><td>(0.1512 0.8442)</td><td>0.1512</td><td>0.8442</td></tr>
<tr><td>2051</td><td>WT013_I07_CLUSTER_9903</td><td>gi|51090649|dbj|BAD36430.1| serine protease-like protein [Oryza sativa (japonica cultivar-group)] e_value:9.45015e-148</td><td>11681.m02428|LOC_Os09g26560|protein expressed protein e_value:2.68246e-149</td><td>0.494</td><td>(0.3086 0.6246)</td><td>0.3086</td><td>0.6246</td></tr>
<tr><td>2052</td><td>WT012_L15_CLUSTER_3661</td><td>gi|50918653|ref|XP_469723.1| unknown protein [Oryza sativa (japonica cultivar-group)] >gnl|BL_ORD_ID|1539232 gi|14626291|gb|AAK71559.1| unknown protein [Oryza sativa (japonica cultivar-group)] e_value:1.47513e-62</td><td>11669.m05419|LOC_Os03g53520|protein expressed protein e_value:2.67624e-64</td><td>0.47</td><td>(0.2370 0.5045)</td><td>0.237</td><td>0.5045</td></tr>
<tr><td>2053</td><td>WT007_E16_CLUSTER_11797</td><td>gi|50939755|ref|XP_479405.1| putative 30S ribosomal protein S9 [Oryza sativa (japonica cultivar-group)] >gnl|BL_ORD_ID|1533399 gi|50510137|dbj|BAD31102.1| putative 30S ribosomal protein S9 [Oryza sativa (japonica cultivar-group)] >gnl|BL_ORD_ID|1533</td><td>11673.m04578|LOC_Os07g46440|protein ribosomal protein S9, putative e_value:1.67749e-165</td><td>0.261</td><td>(0.1434 0.5499)</td><td>0.1434</td><td>0.5499</td></tr>
<tr><td>2054</td><td>WT012_K10_CLUSTER_3437</td><td>gi|50906049|ref|XP_464513.1| putative BRI1-KD interacting protein [Oryza sativa (japonica cultivar-group)] >gnl|BL_ORD_ID|1566800 gi|46390384|dbj|BAD15848.1| putative BRI1-KD interacting protein [Oryza sativa (japonica cultivar-group)] e_value:6.60944e</td><td>11668.m01003|LOC_Os02g10690|protein Targeting protein for Xklp2 (TPX2), putative e_value:1.52497e-130</td><td>0.299</td><td>(0.2205 0.7364)</td><td>0.2205</td><td>0.7364</td></tr>
<tr><td>2055</td><td>WT002_A07_CLUSTER_27158</td><td>gi|7442275|pir||T10175 acyl carrier protein II - barley >gnl|BL_ORD_ID|199466 gi|113169|sp|P08817|ACP2_HORVU Acyl carrier protein II, chloroplast precursor (ACP II) >gnl|BL_ORD_ID|199466 gi|166969|gb|AAA32921.1| acyl carrier protein II >gnl|BL_OR</td><td>11681.m03281|LOC_Os09g36860|protein acyl carrier protein, putative e_value:6.31813e-45</td><td>0.2</td><td>(0.1634 0.8154)</td><td>0.1634</td><td>0.8154</td></tr>
<tr><td>2056</td><td>WT002_H08_CLUSTER_29451</td><td>gi|34896936|ref|NP_909812.1| hypothetical protein [Oryza sativa (japonica cultivar-group)] >gnl|BL_ORD_ID|2340742 gi|20303602|gb|AAM19029.1| hypothetical protein [Oryza sativa (japonica cultivar-group)] >gnl|BL_ORD_ID|2340742 gi|24899455|gb|AAN65025</td><td>11669.m05712|LOC_Os03g56620|protein hypothetical protein e_value:3.64557e-147</td><td>0.405</td><td>(0.1867 0.4608)</td><td>0.1867</td><td>0.4608</td></tr>
<tr><td>2057</td><td>WT010_P11_CLUSTER_22500</td><td>gi|50910215|ref|XP_466596.1| 2 coiled coil domains of eukaryotic origin (31.3 kD)-like protein [Oryza sativa (japonica cultivar-group)] >gnl|BL_ORD_ID|1577956 gi|47848307|dbj|BAD22171.1| 2 coiled coil domains of eukaryotic origin (31.3 kD)-like protein</td><td>11668.m03741|LOC_Os02g39050|protein AT3g18790/MVE11_17 e_value:6.80369e-165</td><td>0.087</td><td>(0.0414 0.4728)</td><td>0.0414</td><td>0.4728</td></tr>
<tr><td>2058</td><td>WT010_M19_CLUSTER_14957</td><td>gi|37999994|gb|AAR07081.1| unknown protein [Oryza sativa (japonica cultivar-group)] >gnl|BL_ORD_ID|2266076 gi|50918481|ref|XP_469637.1| unknown protein [Oryza sativa (japonica cultivar-group)] >gnl|BL_ORD_ID|2266076 gi|29788874|gb|AAP03420.1| unknow</td><td>11669.m05324|LOC_Os03g52680|protein expressed protein e_value:2.6781e-85</td><td>0.421</td><td>(0.1417 0.3363)</td><td>0.1417</td><td>0.3363</td></tr>
<tr><td>2059</td><td>WT009_N16_CLUSTER_12562</td><td>gi|57900279|dbj|BAD87112.1| RNA recognition motif (RRM)-containing protein-like [Oryza sativa (japonica cultivar-group)] >gnl|BL_ORD_ID|972608 gi|57900099|dbj|BAD88161.1| RNA recognition motif (RRM)-containing protein-like [Oryza sativa (japonica culti</td><td>11667.m06586|LOC_Os01g65550|protein RNA recognition motif. (a.k.a. RRM, RBD, or RNP domain), putative e_value:1.98105e-63</td><td>0.224</td><td>(0.4551 2.0279)</td><td>0.4551</td><td>2.0279</td></tr>
<tr><td>2060</td><td>WT009_E03_CLUSTER_12632</td><td>gi|50937979|ref|XP_478517.1| putative tyrosyl-DNA phosphodiesterase [Oryza sativa (japonica cultivar-group)] >gnl|BL_ORD_ID|1528186 gi|33146648|dbj|BAC79984.1| putative tyrosyl-DNA phosphodiesterase [Oryza sativa (japonica cultivar-group)] e_value:0</td><td>11673.m03328|LOC_Os07g34600|protein hypothetical protein e_value:0</td><td>0.207</td><td>(0.1094 0.5271)</td><td>0.1094</td><td>0.5271</td></tr>
<tr><td>2061</td><td>WT009_D06_CLUSTER_12322</td><td>gi|2292907|emb|CAA71179.1| P-glycoprotein homologue [Hordeum vulgare subsp. vulgare] >gnl|BL_ORD_ID|1715477 gi|7442649|pir||T06165 multidrug resistance protein 1 homolog - barley e_value:0</td><td>11667.m05153|LOC_Os01g52550|protein ABC transporter, putative e_value:0</td><td>0.081</td><td>(0.0433 0.5316)</td><td>0.0433</td><td>0.5316</td></tr>
<tr><td>2062</td><td>WT007_L24_CLUSTER_20366</td><td>gi|50938651|ref|XP_478853.1| putative 5-formyltetrahydrofolate cycloligase [Oryza sativa (japonica cultivar-group)] >gnl|BL_ORD_ID|1529737 gi|34393337|dbj|BAC83306.1| putative 5-formyltetrahydrofolate cycloligase [Oryza sativa (japonica cultivar-</td><td>11673.m03803|LOC_Os07g39070|protein 5-formyltetrahydrofolate cyclo-ligase, putative e_value:1.96321e-</td><td>0.347</td><td>(0.0994 0.2866)</td><td>0.0994</td><td>0.2866</td></tr>
<tr><td>2063</td><td>WT007_E19_CLUSTER_16688</td><td>gi|51038216|gb|AAT94019.1| unknown protein [Oryza sativa (japonica cultivar-group)] >gnl|BL_ORD_ID|1020646 gi|51038156|gb|AAT93959.1| unknown protein [Oryza sativa (japonica cultivar-group)] e_value:0</td><td>11682.m03752|LOC_Os05g39520|protein Putative methyltransferase e_value:0</td><td>0.311</td><td>(0.1527 0.4913)</td><td>0.1527</td><td>0.4913</td></tr>
<tr><td>2064</td><td>WT007_B12_CLUSTER_14403</td><td>gi|21553398|gb|AAM62491.1| putative SCO1 protein [Arabidopsis thaliana] >gnl|BL_ORD_ID|1215155 gi|20465763|gb|AAM20370.1| putative SCO1 protein [Arabidopsis thaliana] >gnl|BL_ORD_ID|1215155 gi|17979327|gb|AAL49889.1| putative SCO1 protein [Arabidops</td><td>11668.m00595|LOC_Os02g06480|protein SCO1 protein, putative e_value:2.17689e-121</td><td>0.239</td><td>(0.1995 0.8353)</td><td>0.1995</td><td>0.8353</td></tr>
<tr><td>2065</td><td>WT005_I01_CLUSTER_16049</td><td>gi|18496653|gb|AAL74185.1| putative serine/threonine kinase [Triticum monococcum] e_value:0</td><td>11669.m05504|LOC_Os03g54780|protein putative protein kinase e_value:0</td><td>0.165</td><td>(0.0712 0.4326)</td><td>0.0712</td><td>0.4326</td></tr>
<tr><td>2066</td><td>WT004_P13_CLUSTER_18225</td><td>gi|50929611|ref|XP_474333.1| OSJNBa0018M05.20 [Oryza sativa (japonica cultivar-group)] >gnl|BL_ORD_ID|1594488 gi|38344929|emb|CAE03245.2| OSJNBa0018M05.20 [Oryza sativa (japonica cultivar-group)] e_value:0</td><td>11670.m05726|LOC_Os04g57890|protein Oligosaccharyl transferase STT3 subunit e_value:0</td><td>0.061</td><td>(0.0247 0.4070)</td><td>0.0247</td><td>0.407</td></tr>
<tr><td>2067</td><td>WT002_P05_CLUSTER_10828</td><td>gi|21281139|gb|AAM44996.1| unknown protein [Arabidopsis thaliana] >gnl|BL_ORD_ID|1208490 gi|15028089|gb|AAK76575.1| unknown protein [Arabidopsis thaliana] >gnl|BL_ORD_ID|1208490 gi|18412757|ref|NP_567276.1| amidohydrolase family protein [Arabidopsis</td><td>11670.m05782|LOC_Os04g58390|protein Amidohydrolase family, putative e_value:0</td><td>0.135</td><td>(0.1609 1.1892)</td><td>0.1609</td><td>1.1892</td></tr>
<tr><td>2068</td><td>SET6_O24_CLUSTER_22678</td><td>gi|54287526|gb|AAV31270.1| putative pale cress protein [Oryza sativa (japonica cultivar-group)] e_value:7.74099e-139</td><td>11682.m03055|LOC_Os05g32680|protein PAC e_value:2.11317e-</td><td>0.224</td><td>(0.1319 0.5881)</td><td>0.1319</td><td>0.5881</td></tr>
<tr><td>2069</td><td>SET6_G10_CLUSTER_24808</td><td>gi|50905679|ref|XP_464328.1| putative cinnamoyl-CoA reductase [Oryza sativa (japonica cultivar-group)] >gi|49388016|dbj|BAD25132.1| putative cinnamoyl-CoA reductase [Oryza sativa (japonica cultivar-group)] e_value:4.00035e-168</td><td>11668.m00811|LOC_Os02g08420|protein cinnamoyl-CoA reductase e_value:1.05692e-169</td><td>0.247</td><td>(0.0954 0.3857)</td><td>0.0954</td><td>0.3857</td></tr>
<tr><td>2070</td><td>SET4_O16_CLUSTER_20726</td><td>gi|50948681|ref|XP_493698.1| putative CYP10L [Oryza sativa (japonica cultivar-group)] >gi|6006361|dbj|BAA84791.1| putative CYP10L [Oryza sativa (japonica cultivar-group)] e_value:8.88221e-83</td><td>11680.m00316|LOC_Os06g04000|protein peptidyl-prolyl cis-trans isomerase, cyclophilin-type e_value:1.41172e-84</td><td>0.251</td><td>(0.0746 0.2974)</td><td>0.0746</td><td>0.2974</td></tr>
<tr><td>2071</td><td>SET4_M02_CLUSTER_30891</td><td>gi|52353464|gb|AAU44031.1| putative 1-aminocyclopropane-1-carboxylate oxidase [Oryza sativa (japonica cultivar-group)] e_value:4.26004e-155</td><td>11682.m00512|LOC_Os05g05680|protein 1-aminocyclopropane-1-carboxylate oxidase (EC 1.4.3.-) ACO1 [similarity] - sorghum e_value:1.14629e-156</td><td>0.288</td><td>(0.0743 0.2575)</td><td>0.0743</td><td>0.2575</td></tr>
<tr><td>2072</td><td>SET3_J15_CLUSTER_23811</td><td>gi|27476085|gb|AAO17016.1| Hypothetical protein [Oryza sativa (japonica cultivar-group)] e_value:0</td><td>11669.m00146|LOC_Os03g02240|protein hypothetical protein e_value:0</td><td>0.552</td><td>(0.2666 0.4830)</td><td>0.2666</td><td>0.483</td></tr>
<tr><td>2073</td><td>SET2_L16_CLUSTER_10584</td><td>gi|50947771|ref|XP_483413.1| putative auxin-induced protein [Oryza sativa (japonica cultivar-group)] >gi|29647484|dbj|BAC75413.1| putative auxin-induced protein [Oryza sativa (japonica cultivar-group)] e_value:8.68934e-98</td><td>11674.m04152|LOC_Os08g41290|protein expressed protein e_value:2.23984e-99</td><td>0.444</td><td>(0.1938 0.4362)</td><td>0.1938</td><td>0.4362</td></tr>
<tr><td>2074</td><td>SET2_F13_CLUSTER_431</td><td>gi|34910736|ref|NP_916715.1| putative phosphoribosyl pyrophosphate synthase [Oryza sativa (japonica cultivar-group)] >gi|20160549|dbj|BAB89498.1| putative phosphoribosyl pyrophosphate synthase [Oryza sativa (japonica cultivar-group)] e_value:0</td><td>11667.m05151|LOC_Os01g52530|protein ribose-phosphate pyrophosphokinase, putative e_value:0</td><td>0.238</td><td>(0.1100 0.4618)</td><td>0.11</td><td>0.4618</td></tr>
<tr><td>2075</td><td>WT013_D18_CLUSTER_4504</td><td>gi|17017402|gb|AAL33655.1| histone deacetylase [Zea mays] e_value:0</td><td>11680.m03745|LOC_Os06g38470|protein Histone deacetylase family, putative e_value:3.39984e-155</td><td></td><td>(0.3684 -1.0000)</td><td>0.3684</td><td>-1</td></tr>
<tr><td>2076</td><td>WT012_G08_CLUSTER_29149</td><td>gi|52075621|dbj|BAD44792.1| SPX (SYG1/Pho81/XPR1) domain-containing protein-like [Oryza sativa (japonica cultivar-group)] e_value:0</td><td>11680.m00303|LOC_Os06g03860|protein SPX domain, putative e_value:0</td><td>0.077</td><td>(0.0421 0.5445)</td><td>0.0421</td><td>0.5445</td></tr>
<tr><td>2077</td><td>WT012_D17_CLUSTER_25570</td><td>gi|22450578|gb|AAM97150.1| hypothetical protein [Oryza sativa (japonica cultivar-group)] >gnl|BL_ORD_ID|2040113 gi|50918129|ref|XP_469461.1| hypothetical protein [Oryza sativa (japonica cultivar-group)] e_value:2.46197e-52</td><td>11669.m01547|LOC_Os03g15530|protein expressed protein e_value:2.46493e-124</td><td>0.52</td><td>(0.2101 0.4043)</td><td>0.2101</td><td>0.4043</td></tr>
<tr><td>2078</td><td>WT002_I03_CLUSTER_34847</td><td>gi|51091565|dbj|BAD36302.1| putative U4/U6.U5 tri-snRNP-associated 65 kDa protein [Oryza sativa (japonica cultivar-group)] >gnl|BL_ORD_ID|239047 gi|51091509|dbj|BAD36247.1| putative U4/U6.U5 tri-snRNP-associated 65 kDa protein [Oryza sativa (japonica c</td><td>11681.m02181|LOC_Os09g24250|protein USP39 protein e_value:0</td><td>0.162</td><td>(0.0894 0.5533)</td><td>0.0894</td><td>0.5533</td></tr>
<tr><td>2079</td><td>SET1_J14_CLUSTER_32922</td><td>gi|50919665|ref|XP_470193.1| Putative glutathione S-transferase [Oryza sativa (japonica cultivar-group)] >gi|22758267|gb|AAN05495.1| Putative glutathione S-transferase [Oryza sativa (japonica cultivar-group)] e_value:7.7832e-111</td><td>11669.m00363|LOC_Os03g04260|protein Glutathione S-transferase, N-terminal domain, putative e_value:1.82093e-112</td><td>0.122</td><td>(0.0683 0.5606)</td><td>0.0683</td><td>0.5606</td></tr>
<tr><td>2080</td><td>WT004_P22_CLUSTER_21146</td><td>gi|26451476|dbj|BAC42837.1| unknown protein [Arabidopsis thaliana] >gnl|BL_ORD_ID|1091927 gi|30694112|ref|NP_850703.1| expressed protein [Arabidopsis thaliana] >gnl|BL_ORD_ID|1091927 gi|30694110|ref|NP_191035.2| expressed protein [Arabidopsis thalia</td><td>11686.m04258|LOC_Os12g42700|protein expressed protein e_value:0</td><td>0.2</td><td>(0.1306 0.6541)</td><td>0.1306</td><td>0.6541</td></tr>
<tr><td>2081</td><td>SET1_B19_CLUSTER_21439</td><td>gi|50941271|ref|XP_480163.1| putative nitrate transporter [Oryza sativa (japonica cultivar-group)] >gi|37805980|dbj|BAC99394.1| putative nitrate transporter [Oryza sativa (japonica cultivar-group)] e_value:0</td><td>11674.m00514|LOC_Os08g05910|protein nitrate transporter e_value:0</td><td>0.185</td><td>(0.0726 0.3930)</td><td>0.0726</td><td>0.393</td></tr>
<tr><td>2082</td><td>WT013_E20_CLUSTER_25186</td><td>gi|20284|emb|CAA37979.1| proliferating cell nuclear antigen [Oryza sativa (japonica cultivar-group)] >gnl|BL_ORD_ID|2322050 gi|50915640|ref|XP_468284.1| SPATULA-like [Oryza sativa (japonica cultivar-group)] >gnl|BL_ORD_ID|2322050 gi|15451583|gb|AAK9</td><td>11668.m05538|LOC_Os02g56130|protein proliferating cell nuclear antigen (pcna) e_value:3.6725e-142</td><td>0.042</td><td>(0.0191 0.4559)</td><td>0.0191</td><td>0.4559</td></tr>
<tr><td>2083</td><td>WT004_G23_CLUSTER_33771</td><td>gi|55168246|gb|AAV44112.1| unknown protein [Oryza sativa (japonica cultivar-group)] >gnl|BL_ORD_ID|1465770 gi|55168198|gb|AAV44064.1| unknown protein [Oryza sativa (japonica cultivar-group)] e_value:2.12456e-177</td><td>11682.m02638|LOC_Os05g28190|protein expressed protein e_value:5.65173e-179</td><td>0.179</td><td>(0.1097 0.6114)</td><td>0.1097</td><td>0.6114</td></tr>
<tr><td>2084</td><td>WT006_C18_CLUSTER_19679</td><td>gi|37201994|gb|AAQ89612.1| At5g26850 [Arabidopsis thaliana] >gnl|BL_ORD_ID|2239844 gi|26449532|dbj|BAC41892.1| unknown protein [Arabidopsis thaliana] e_value:0</td><td>11669.m01983|LOC_Os03g19520|protein expressed protein e_value:0</td><td>0.155</td><td>(0.0794 0.5112)</td><td>0.0794</td><td>0.5112</td></tr>
<tr><td>2085</td><td>WT012_G09_CLUSTER_13386</td><td>gi|50948483|ref|XP_483769.1| putative dihydroxy-acid dehydratase [Oryza sativa (japonica cultivar-group)] >gnl|BL_ORD_ID|1612270 gi|51979723|ref|XP_507608.1| PREDICTED P0562A06.23 gene product [Oryza sativa (japonica cultivar-group)] >gnl|BL_ORD_ID|</td><td>11674.m04502|LOC_Os08g44530|protein dihydroxy-acid dehydratase e_value:0</td><td>0.074</td><td>(0.0371 0.5016)</td><td>0.0371</td><td>0.5016</td></tr>
<tr><td>2086</td><td>WT004_H02_CLUSTER_31948</td><td>gi|50910233|ref|XP_466605.1| putative GCPE protein [Oryza sativa (japonica cultivar-group)] >gnl|BL_ORD_ID|1577986 gi|51964146|ref|XP_506856.1| PREDICTED OJ1669_F01.30 gene product [Oryza sativa (japonica cultivar-group)] >gnl|BL_ORD_ID|1577986 gi|4</td><td>11668.m03752|LOC_Os02g39160|protein 4-hydroxy-3-methylbut-2-en-1-yl diphosphate synthase e_value:0</td><td>0.082</td><td>(0.0367 0.4470)</td><td>0.0367</td><td>0.447</td></tr>
<tr><td>2087</td><td>SET5_L10_CLUSTER_8566</td><td>gi|50938763|ref|XP_478909.1| Yippee-like protein [Oryza sativa (japonica cultivar-group)] >gi|34393694|dbj|BAC82961.1| Yippee-like protein [Oryza sativa (japonica cultivar-group)] e_value:1.39966e-52</td><td>11673.m03851|LOC_Os07g39510|protein Yippee putative zinc-binding protein e_value:7.7847e-55</td><td>0.129</td><td>(0.0564 0.4377)</td><td>0.0564</td><td>0.4377</td></tr>
</table>

PDF Complete
Your complimentary use period has ended. Thank you for using PDF Complete.
Click Here to upgrade to Unlimited Pages and Expanded Features

<table>
<tr><th></th><th></th><th></th><th></th><th></th><th></th><th></th></tr>
<tr><td>2088</td><td>WT005_E07_CLUSTER_33341</td><td>gi|31432429|gb|AAP54059.1| unknown protein [Oryza sativa (japonica cultivar-group)] >gnl|BL_ORD_ID|2011587 gi|37534940|ref|NP_921772.1| unknown protein [Oryza sativa (japonica cultivar-group)] e_value:0</td><td>11676.m02743|LOC_Os10g31520|protein ribosomal rna assembly protein mis3 e_value:0</td><td>0.117 (0.0771 0.6584)</td><td>0.0771</td><td>0.6584</td></tr>
<tr><td>2089</td><td>WT005__C02_CLUSTER_33197</td><td>gi|24030242|gb|AAN41297.1| unknown protein [Arabidopsis thaliana] >gnl|BL_ORD_ID|1512334 gi|30688336|ref|NP_030605.2| appr-1-p processing enzyme family protein [Arabidopsis thaliana] e_value:4.65219e-65</td><td>11669.m02240|LOC_Os03g21830|protein expressed protein e_value:4.4671e-93</td><td>0.186 (0.1007 0.5408)</td><td>0.1007</td><td>0.5408</td></tr>
<tr><td>2090</td><td>WT004_P11_CLUSTER_2285</td><td>gi|50904957|ref|XP_463967.1| putative small nuclear ribonucleoprotein polypeptide E [Oryza sativa (japonica cultivar-group)] >gnl|BL_ORD_ID|1566209 gi|41053075|dbj|BAD08019.1| putative small nuclear ribonucleoprotein polypeptide E [Oryza sativa (japoni</td><td>11674.m00473|LOC_Os08g05530|protein Sm protein, putative e_value:5.55919e-43</td><td>0.055 (0.0220 0.3995)</td><td>0.022</td><td>0.3995</td></tr>
<tr><td>2091</td><td>WT003_K23_CLUSTER_20319</td><td>gi|56409864|emb|CAI30080.1| glycosyltransferase [Zea mays] e_value:0</td><td>11676.m03113|LOC_Os10g35020|protein expressed protein</td><td>0.243 (0.1278 0.5254)</td><td>0.1278</td><td>0.5254</td></tr>
<tr><td>2092</td><td>WT002_M20_CLUSTER_5003</td><td>gi|50916114|ref|XP_468521.1| putative DNA-binding protein PD3, chloroplast [Oryza sativa (japonica cultivar-group)] >gnl|BL_ORD_ID|1583738 gi|48716466|dbj|BAD23073.1| putative DNA-binding protein PD3, chloroplast [Oryza sativa (japonica cultivar-group)</td><td>11673.m03597|LOC_Os07g37140|protein expressed protein e_value:1.53976e-140</td><td>0.247 (0.2582 1.0442)</td><td>0.2582</td><td>1.0442</td></tr>
<tr><td>2093</td><td>SET1_P06_CLUSTER_30538</td><td>gi|50938533|ref|XP_478794.1| putative 40S RIBOSOMAL PROTEIN S13 [Oryza sativa (japonica cultivar-group)] >gi|34393549|dbj|BAC83147.1| putative 40S RIBOSOMAL PROTEIN S13 [Oryza sativa (japonica cultivar-group)] e_value:3.89144e-63</td><td>11674.m00149|LOC_Os08g02400|protein ribosomal protein S15, putative e_value:2.86241e-65</td><td>0.253 (0.1539 0.6081)</td><td>0.1539</td><td>0.6081</td></tr>
<tr><td>2094</td><td>WT013_M04_CLUSTER_34450</td><td>gi|55770689|ref|XP_550386.1| putative BRI1-KD interacting protein 128 [Oryza sativa (japonica cultivar-group)] >gnl|BL_ORD_ID|1753781 gi|55296255|dbj|BAD67996.1| putative BRI1-KD interacting protein 128 [Oryza sativa (japonica cultivar-group)] >gnl|</td><td>11667.m00790|LOC_Os01g08410|protein hypothetical protein e_value:9.2686e-125</td><td>0.222 (0.1174 0.5301)</td><td>0.1174</td><td>0.5301</td></tr>
<tr><td>2095</td><td>WT013_J08_CLUSTER_5039</td><td>gi|50916369|ref|XP_468587.1| Unknown protein [Oryza sativa (japonica cultivar-group)] >gnl|BL_ORD_ID|1584032 gi|25446691|gb|AAN74838.1| Unknown protein [Oryza sativa (japonica cultivar-group)] e_value:2.19906e-103</td><td>11669.m00245|LOC_Os03g03140|protein RNA recognition motif. (a.k.a. RRM, RBD, or RNP domain), putative e_value:5.67791e-</td><td>0.216 (0.1474 0.6832)</td><td>0.1474</td><td>0.6832</td></tr>
<tr><td>2096</td><td>WT005_P10_CLUSTER_8637</td><td>gi|32879543|emb|CAE11882.1| beta3-glucuronyltransferase [Zea mays] e_value:6.49088e-91</td><td>11669.m01804|LOC_Os03g17850|protein Glycosyltransferase family 43, putative e_value:5.29441e-140</td><td>0.387 (0.1764 0.4556)</td><td>0.1764</td><td>0.4556</td></tr>
<tr><td>2097</td><td>WT004_L04_CLUSTER_14844</td><td>gi|50905911|ref|XP_464444.1| KH domain-containing protein-like [Oryza sativa (japonica cultivar-group)] >gnl|BL_ORD_ID|1566517 gi|46389843|dbj|BAD15406.1| KH domain-containing protein-like [Oryza sativa (japonica cultivar-group)] e_value:2.75897e-139</td><td>11668.m00932|LOC_Os02g10080|protein Zinc finger C-x8-C-x5-C-x3-H type (and similar), putative e_value:6.4521e-141</td><td>0.161 (0.1159 0.7206)</td><td>0.1159</td><td>0.7206</td></tr>
<tr><td>2098</td><td>SET1_F05_CLUSTER_1095</td><td>gi|50939517|ref|XP_479286.1| putative thymidylate kinase [Oryza sativa (japonica cultivar-group)] >gi|27261100|dbj|BAC45213.1| putative thymidylate kinase [Oryza sativa (japonica cultivar-group)] e_value:8.09011e-105</td><td>11673.m04391|LOC_Os07g44630|protein Thymidylate kinase, putative e_value:1.35238e-106</td><td>0.141 (0.0839 0.5971)</td><td>0.0839</td><td>0.5971</td></tr>
<tr><td>2099</td><td>WT013_H18_CLUSTER_21270</td><td>gi|57900102|dbj|BAD88164.1| unknown protein [Oryza sativa (japonica cultivar-group)] e_value:0</td><td>11667.m07045|LOC_Os01g70320|protein expressed protein</td><td>0.209 (0.0936 0.4475)</td><td>0.0936</td><td>0.4475</td></tr>
<tr><td>2100</td><td>WT003_O08_CLUSTER_21938</td><td>gi|50252332|dbj|BAD28365.1| putative peptidyl-prolyl cis-trans isomerase NIMA-interacting 4 [Oryza sativa (japonica cultivar-group)] e_value:8.01684e-71</td><td>11681.m02211|LOC_Os09g24540|protein PPIC-type PPIASE domain, putative e_value:1.026e-72</td><td>0.193 (0.0677 0.3509)</td><td>0.0677</td><td>0.3509</td></tr>
<tr><td>2101</td><td>SET2_A14_CLUSTER_27256</td><td>gi|56785053|dbj|BAD82692.1| unknown protein [Oryza sativa (japonica cultivar-group)] e_value:5.13074e-101</td><td>11667.m05955|LOC_Os01g59900|protein expressed protein e_value:8.82866e-103</td><td>0.392 (0.2390 0.6101)</td><td>0.239</td><td>0.6101</td></tr>
<tr><td>2102</td><td>WT009_F17_CLUSTER_33592</td><td>gi|60393199|gb|AAX19515.1| serine/threonine protein kinase [Triticum aestivum] e_value:2.56123e-95</td><td>11687.m01128|LOC_Os11g11780|protein Similar to stem rust resistance protein e_value:2.18705e-89</td><td>0.297 (0.3410 1.1472)</td><td>0.341</td><td>1.1472</td></tr>
<tr><td>2103</td><td>WT013_H20_CLUSTER_23497</td><td>gi|50912253|ref|XP_467534.1| putative zinc finger and C2 domain protein [Oryza sativa (japonica cultivar-group)] >gnl|BL_ORD_ID|1580636 gi|45735988|dbj|BAD13017.1| putative zinc finger and C2 domain protein [Oryza sativa (japonica cultivar-</td><td>11668.m04769|LOC_Os02g49070|protein Putative GTP-ase activating protein for Arf e_value:4.67285e-159</td><td>0.225 (0.1057 0.4692)</td><td>0.1057</td><td>0.4692</td></tr>
<tr><td>2104</td><td>SET1_L24_CLUSTER_9972</td><td>gi|50509945|dbj|BAD30267.1| NADH-ubiquinone oxidoreductase-related-like protein [Oryza sativa (japonica cultivar-group)] e_value:5.65554e-79</td><td>11673.m03872|LOC_Os07g39710|protein ETC complex I subunit conserved region, putative e_value:9.16865e-81</td><td>0.106 (0.0409 0.3854)</td><td>0.0409</td><td>0.3854</td></tr>
<tr><td>2105</td><td>WT012_B05_CLUSTER_9214</td><td>gi|57610636|gb|AAW52556.1| At3g17000 [Arabidopsis thaliana] >gnl|BL_ORD_ID|2077561 gi|7670024|dbj|BAA94978.1| unnamed protein product [Arabidopsis thaliana] >gnl|BL_ORD_ID|2077561 gi|14423396|gb|AAK62380.1| Unknown protein [Arabidopsis thaliana] &gt</td><td>11669.m01980|LOC_Os03g19500|protein expressed protein e_value:2.98579e-141</td><td>0.177 (0.0949 0.5370)</td><td>0.0949</td><td>0.537</td></tr>
<tr><td>2106</td><td>WT005_F18_CLUSTER_34955</td><td>gi|60729577|pir||JC7906 sucrose 1F-fructosyltransferase (EC 2.4.1.99) - wheat >gnl|BL_ORD_ID|1323721 gi|55847605|dbj|BAD72792.1| sucrose:sucrose 1-fructosyltransferase [Triticum aestivum] >gnl|BL_ORD_ID|1323721 gi|18146823|dbj|BAB82470.1| sucrose:su</td><td>11668.m00061|LOC_Os02g01590|protein vacuolar acid invertase e_value:0</td><td>0.529 (0.3133 0.5921)</td><td>0.3133</td><td>0.5921</td></tr>
<tr><td>2107</td><td>WT011_N03_CLUSTER_2600</td><td>gi|50945247|ref|XP_482151.1| cytochrome b5-like [Oryza sativa (japonica cultivar-group)] >gnl|BL_ORD_ID|1605997 gi|51964866|ref|XP_507217.1| PREDICTED P0434E03.7 gene product [Oryza sativa (japonica cultivar-group)] >gnl|BL_ORD_ID|1605997 gi|4025347</td><td>11674.m02914|LOC_Os08g29670|protein Cytochrome b5-like Heme/Steroid binding domain, putative e_value:3.32056e-50</td><td>0.194 (0.1439 0.7426)</td><td>0.1439</td><td>0.7426</td></tr>
<tr><td>2108</td><td>WT008_L05_CLUSTER_34019</td><td>gi|22531207|gb|AAM97107.1| ATP-dependent Clp protease proteolytic subunit (ClpR3), putative [Arabidopsis thaliana] >gnl|BL_ORD_ID|1188523 gi|18390982|ref|NP_563836.1| ATP-dependent Clp protease proteolytic subunit, putative [Arabidopsis thaliana] >g</td><td>11669.m02308|LOC_Os03g22430|protein Clp protease, putative e_value:1.21459e-162</td><td>0.135 (0.0758 0.5598)</td><td>0.0758</td><td>0.5598</td></tr>
<tr><td>2109</td><td>WT007_J01_CLUSTER_33233</td><td>gi|50920805|ref|XP_470763.1| putative capping protein beta subunit [Oryza sativa (japonica cultivar-group)] >gnl|BL_ORD_ID|1511190 gi|40882704|gb|AAR96245.1| putative capping protein beta subunit [Oryza sativa (japonica cultivar-group)] e_value:1.74153</td><td>11669.m02686|LOC_Os03g26450|protein putative capping protein beta subunit e_value:3.57151e-139</td><td>0.052 (0.0255 0.4910)</td><td>0.0255</td><td>0.491</td></tr>
<tr><td>2110</td><td>SET5_B22_CLUSTER_34009</td><td>gi|50925313|ref|XP_472952.1| OJ991113_30.3 [Oryza sativa (japonica cultivar-group)] >gi|38344227|emb|CAE02055.2| OJ991113_30.3 [Oryza sativa (japonica cultivar-group)] e_value:2.2153e-69</td><td>11670.m03926|LOC_Os04g40980|protein Uncharacterized BCR, COG1963 e_value:2.72152e-71</td><td>0.174 (0.0714 0.4104)</td><td>0.0714</td><td>0.4104</td></tr>
<tr><td>2111</td><td>SET6_G14_CLUSTER_27461</td><td>gi|50942139|ref|XP_480597.1| unknown protein [Oryza sativa (japonica cultivar-group)] >gi|40253396|dbj|BAD05326.1| unknown protein [Oryza sativa (japonica cultivar-group)] e_value:3.99498e-134</td><td>11674.m00991|LOC_Os08g10410|protein expressed protein e_value:1.14428e-135</td><td>0.068 (0.0392 0.5747)</td><td>0.0392</td><td>0.5747</td></tr>
<tr><td>2112</td><td>WT003_L16_CLUSTER_22320</td><td>gi|50726579|dbj|BAD34213.1| unknown protein [Oryza sativa (japonica cultivar-group)] e_value:4.96618e-137</td><td>11681.m03240|LOC_Os09g36460|protein Zinc finger, C3HC4 type (RING finger), putative e_value:1.22307e-138</td><td>0.215 (0.1298 0.6034)</td><td>0.1298</td><td>0.6034</td></tr>
<tr><td>2113</td><td>WT012_O16_CLUSTER_15336</td><td>gi|38345839|emb|CAD41079.2| OSJNBa0084K11.3 [Oryza sativa (japonica cultivar-group)] >gnl|BL_ORD_ID|1522694 gi|50927907|ref|XP_473481.1| OSJNBa0084K11.3 [Oryza sativa (japonica cultivar-group)] e_value:0</td><td>11670.m04611|LOC_Os04g47240|protein Protein kinase domain, putative e_value:0</td><td>0.18 (0.0883 0.4896)</td><td>0.0883</td><td>0.4896</td></tr>
<tr><td>2114</td><td>WT007_P19_CLUSTER_15136</td><td>gi|34909150|ref|NP_915922.1| P0468B07.18 [Oryza sativa (japonica cultivar-group)] >gnl|BL_ORD_ID|1554034 gi|20160696|dbj|BAB89639.1| unknown protein [Oryza sativa (japonica cultivar-group)] e_value:0</td><td>11667.m05912|LOC_Os01g59510|protein expressed protein e_value:0</td><td>0.069 (0.0358 0.5202)</td><td>0.0358</td><td>0.5202</td></tr>
<tr><td>2115</td><td>WT011_P13_CLUSTER_33246</td><td>gi|34910142|ref|NP_916418.1| putative auxin-responsive GH3 [Oryza sativa (japonica cultivar-group)] >gnl|BL_ORD_ID|1552910 gi|20804910|dbj|BAB92590.1| putative auxin-regulated protein GH3 [Oryza sativa (japonica cultivar-group)] e_value:0</td><td>11667.m05712|LOC_Os01g57610|protein GH3 auxin-responsive promoter e_value:0</td><td>0.215 (0.0669 0.3106)</td><td>0.0669</td><td>0.3106</td></tr>
<tr><td>2116</td><td>WT007_O24_CLUSTER_2029</td><td>gi|34907164|ref|NP_914929.1| P0423A12.19 [Oryza sativa (japonica cultivar-group)] >gnl|BL_ORD_ID|1540655 gi|21104657|dbj|BAB93248.1| putative WD repeat domain 45 [Oryza sativa (japonica cultivar-group)] e_value:0</td><td>11667.m07094|LOC_Os01g70780|protein expressed protein e_value:0</td><td>0.118 (0.0836 0.7059)</td><td>0.0836</td><td>0.7059</td></tr>
<tr><td>2117</td><td>WT013_I22_CLUSTER_31677</td><td>gi|50659024|gb|AAT80325.1| UDP-D-glucuronate decarboxylase [Hordeum vulgare] e_value:0</td><td>11673.m04724|LOC_Os07g47700|protein NAD dependent epimerase/dehydratase family, putative e_value:7.04023e-163</td><td>0.103 (0.2392 2.3156)</td><td>0.2392</td><td>2.3156</td></tr>
<tr><td>2118</td><td>SET3_K18_CLUSTER_13801</td><td>gi|53793155|dbj|BAD54363.1| zinc finger protein-like [Oryza sativa (japonica cultivar-group)] e_value:2.87528e-148</td><td>11680.m01519|LOC_Os06g15330|protein CCT motif, putative e_value:5.42919e-153</td><td>0.508 (0.1905 0.3748)</td><td>0.1905</td><td>0.3748</td></tr>
<tr><td>2119</td><td>WT013_H04_CLUSTER_33985</td><td>gi|50251178|dbj|BAD29666.1| putative Na+/H+ antiporter, isoform 2 [Oryza sativa (japonica cultivar-group)] e_value:0</td><td>11681.m00981|LOC_Os09g11450|protein hypothetical protein e_value:0</td><td>0.208 (0.1103 0.5304)</td><td>0.1103</td><td>0.5304</td></tr>
<tr><td>2120</td><td>WT010_O04_CLUSTER_8782</td><td>gi|52353366|gb|AAU43934.1| unknown protein [Oryza sativa (japonica cultivar-group)] e_value:3.64271e-141</td><td>11682.m04090|LOC_Os05g43040|protein TPR Domain, putative e_value:9.09597e-143</td><td>0.12 (0.0909 0.7552)</td><td>0.0909</td><td>0.7552</td></tr>
<tr><td>2121</td><td>SET3__C22_CLUSTER_21324</td><td>gi|31580630|gb|AAP49821.1| phosphate transporter 2-1 [Triticum aestivum] e_value:0</td><td>11668.m03631|LOC_Os02g38020|protein Phosphate transporter family, putative e_value:0</td><td>0.144 (0.0571 0.3958)</td><td>0.0571</td><td>0.3958</td></tr>
<tr><td>2122</td><td>WT012_D24_CLUSTER_2958</td><td>gi|27469354|gb|AAO06116.1| bZIP transcription factor ZIP1 [Hordeum vulgare subsp. vulgare] e_value:1.05069e-144</td><td>11686.m01287|LOC_Os12g13170|protein G-box binding factor 1A - rice e_value:2.89622e-141</td><td>0.316 (0.1016 0.3211)</td><td>0.1016</td><td>0.3211</td></tr>
<tr><td>2123</td><td>SET2_N09_CLUSTER_17849</td><td>gi|53793325|dbj|BAD54546.1| putative ascorbate oxidase AO4 [Oryza sativa (japonica cultivar-group)] e_value:0</td><td>11680.m03601|LOC_Os06g37080|protein l-ascorbate oxidase precursor (ec 1.10.3.3) (ascorbase) (aso) e_value:0</td><td>0.338 (0.0984 0.2911)</td><td>0.0984</td><td>0.2911</td></tr>
<tr><td>2124</td><td>WT005_I20_CLUSTER_24914</td><td>gi|6911879|emb|CAB72179.1| hypothetical protein [Arabidopsis thaliana] >gnl|BL_ORD_ID|1454977 gi|21593138|gb|AAM65087.1| unknown [Arabidopsis thaliana] >gnl|BL_ORD_ID|1454977 gi|20453349|gb|AAM19913.1| AT3g57090/F24I3_170 [Arabidopsis thaliana] ></td><td>11669.m02433|LOC_Os03g24060|protein expressed protein e_value:2.3708e-80</td><td>0.133 (0.0634 0.4771)</td><td>0.0634</td><td>0.4771</td></tr>
<tr><td>2125</td><td>WT002_H17_CLUSTER_34569</td><td>gi|54290419|dbj|BAD61289.1| acetyltransferase 1-like [Oryza sativa (japonica cultivar-group)] e_value:0</td><td>11667.m04164|LOC_Os01g43030|protein TPR Domain, putative e_value:0</td><td>0.082 (0.0511 0.6235)</td><td>0.0511</td><td>0.6235</td></tr>
<tr><td>2126</td><td>WT003_D06_CLUSTER_31943</td><td>gi|52548240|gb|AAU82107.1| 20S proteasome beta 5 subunit [Triticum aestivum] e_value:2.32486e-157</td><td>11680.m00541|LOC_Os06g06030|protein Proteasome A-type and B-type, putative e_value:9.34012e-131</td><td>0.208 (0.0946 0.4546)</td><td>0.0946</td><td>0.4546</td></tr>
<tr><td>2127</td><td>SET3_P04_CLUSTER_34361</td><td>gi|50920807|ref|XP_470764.1| putative ripening regulated protein [Oryza sativa (japonica cultivar-group)] >gi|40882701|gb|AAR96242.1| putative ripening regulated protein [Oryza sativa (japonica cultivar-group)] e_value:2.97894e-72</td><td>11669.m02687|LOC_Os03g26460|protein putative ripening regulated protein e_value:5.10932e-74</td><td>0.26 (0.1213 0.4670)</td><td>0.1213</td><td>0.467</td></tr>
<tr><td>2128</td><td>SET1_P01_CLUSTER_19965</td><td>gi|38346641|emb|CAD40737.2| OSJNBa0072D21.11 [Oryza sativa (japonica cultivar-group)] >gi|50923775|ref|XP_472248.1| OSJNBa0072D21.11 [Oryza sativa (japonica cultivar-group)] e_value:9.96641e-70</td><td>11670.m02986|LOC_Os04g31350|protein expressed protein e_value:1.50314e-71</td><td>0.323 (0.1428 0.4420)</td><td>0.1428</td><td>0.442</td></tr>
<tr><td>2129</td><td>SET4_B11_CLUSTER_21379</td><td>gi|55297457|dbj|BAD69308.1| putative lipase [Oryza sativa (japonica cultivar-group)] >gi|55296702|dbj|BAD69420.1| putative lipase [Oryza sativa (japonica cultivar-group)] e_value:0</td><td>11680.m00568|LOC_Os06g06250|protein GDSL-like Lipase/Acylhydrolase e_value:0</td><td>0.265 (0.0933 0.3519)</td><td>0.0933</td><td>0.3519</td></tr>
<tr><td>2130</td><td>SET3_I14_CLUSTER_14648</td><td>gi|56784110|dbj|BAD81481.1| branched-chain amino acid aminotransferase -like [Oryza sativa (japonica cultivar-group)] e_value:5.88194e-166</td><td>11667.m01351|LOC_Os01g13690|protein aminotransferase, class IV e_value:1.2271e-151</td><td>0.439 (0.1463 0.3331)</td><td>0.1463</td><td>0.3331</td></tr>
<tr><td>2131</td><td>SET1_N10_CLUSTER_34774</td><td>gi|62701864|gb|AAX92937.1| Harpin-induced protein 1 (Hin1), putative [Oryza sativa (japonica cultivar-group)] e_value:8.66039e-101</td><td>11687.m00508|LOC_Os11g05860|protein Harpin-induced protein 1 (Hin1), putative e_value:1.54733e-102</td><td>0.4 (0.0894 0.2233)</td><td>0.0894</td><td>0.2233</td></tr>
</table>

PDF Complete
Your complimentary use period has ended. Thank you for using PDF Complete.
Click Here to upgrade to Unlimited Pages and Expanded Features

2132	WT003_N09_CLUSTER_33660	gi|26518370|gb|AAN80141.1| extracellular invertase; beta-fructofuranosidase [Triticum monococcum] e_value:0	11681.m00631|LOC_Os09g08080|protein Glycosyl hydrolases family 32 e_value:0	0.411	(0.2152 0.5229)	0.2152	0.5229
2133	WT002_J18_CLUSTER_17643	gi|51979697|ref|XP_507595.1| PREDICTED OJ1081_B12.117 gene product [Oryza sativa (japonica cultivar-group)] >gnl|BL_ORD_ID|1609713 gi|50947723|ref|XP_483389.1| putative chitinase precursor [Oryza sativa (japonica cultivar-group)]	11681.m02925|LOC_Os09g32080|protein Chitinase class I, putative e_value:1.26959e-164	0.709	(0.1376 0.1940)	0.1376	0.194
2134	WT004_E18_CLUSTER_32705	gi|62898547|dbj|BAD97369.1| EREBP/AP2 type transcription factor [Triticum aestivum] e_value:0	11682.m02612|LOC_Os05g27930|protein AP2 transcriptional activator DRF1.3 e_value:8.13306e-60	0.327	(0.3613 1.1062)	0.3613	1.1062
2135	WT003_F03_CLUSTER_22062	gi|57863909|gb|AAS88823.2| unknown protein [Oryza sativa (japonica cultivar-group)] >gnl|BL_ORD_ID|6461 gi|57863902|gb|AAG03104.2| unknown protein [Oryza sativa (japonica cultivar-group)] e_value:1.28233e-118	11682.m00029|LOC_Os05g01250|protein hypothetical protein e_value:6.56128e-113	0.088	(0.0483 0.5469)	0.0483	0.5469
2136	WT007_H14_CLUSTER_32439	gi|50918703|ref|XP_469748.1| putative arginine-rich protein [Oryza sativa] >gnl|BL_ORD_ID|1539257 gi|18087665|gb|AAL58957.1| putative arginine-rich protein [Oryza sativa] e_value:6.52086e-126	11669.m05446|LOC_Os03g53770|protein putative arginine-rich protein e_value:1.21186e-127	0.209	(0.0632 0.3016)	0.0632	0.3016
2137	SET4_P24_CLUSTER_23041	gi|62701832|gb|AAX92905.1| expressed protein [Oryza sativa (japonica cultivar-group)] e_value:3.72232e-71	11686.m01257|LOC_Os12g12880|protein expressed protein e_value:2.8977e-75	0.29	(0.2050 0.7074)	0.205	0.7074
2138	SET3_J17_CLUSTER_25141	gi|55771374|dbj|BAD72541.1| putative response regulator 9 [Oryza sativa (japonica cultivar-group)] e_value:0	11680.m00798|LOC_Os06g08440|protein myb-like DNA-binding domain, SHAQKYF class, putative e_value:0	0.197	(0.1601 0.8142)	0.1601	0.8142
2139	SET6_K17_CLUSTER_19465	gi|31430164|gb|AAP52116.1| unknown protein [Oryza sativa (japonica cultivar-group)] >gi|37531054|ref|NP_919829.1| unknown protein [Oryza sativa (japonica cultivar-group)] >gi|15209146|gb|AAK91879.1| Unknown protein [Oryza sativa] e_value:4.78924e-30	11676.m00485|LOC_Os10g05720|protein Protease inhibitor/seed storage/LTP family, putative e_value:1.65564e-33	0.464	(0.2448 0.5281)	0.2448	0.5281
2140	SET3_F08_CLUSTER_12192	gi|34894768|ref|NP_908709.1| B1135C02.14 [Oryza sativa (japonica cultivar-group)] >gi|13366005|dbj|BAB39282.1| unknown protein [Oryza sativa (japonica cultivar-group)] >gi|52077582|dbj|BAD45707.1| unknown protein [Oryza sativa (japonica cultivar-gro	11667.m02170|LOC_Os01g22380|protein hypothetical protein e_value:3.87129e-33	0.198	(0.1955 0.9876)	0.1955	0.9876
2141	WT004_G05_CLUSTER_32647	gi|50930837|ref|XP_474946.1| OSJNBb0072N21.3 [Oryza sativa (japonica cultivar-group)] >gnl|BL_ORD_ID|1595358 gi|38344124|emb|CAD39837.2| OSJNBb0072N21.3 [Oryza sativa (japonica cultivar-group)] e_value:0	11681.m03363|LOC_Os09g37650|protein expressed protein e_value:0	0.283	(0.2014 0.7104)	0.2014	0.7104
2142	WT003_P23_CLUSTER_25752	gi|50725735|dbj|BAD33246.1| putative Pyrophosphate--fructose 6-phosphate 1-phosphotransferase alpha subunit [Oryza sativa (japonica cultivar-group)] e_value:0	11680.m02162|LOC_Os06g22060|protein Phosphofructokinase, putative e_value:0	0.104	(0.0533 0.5125)	0.0533	0.5125
2143	WT003_P19_CLUSTER_30749	gi|21740425|emb|CAD41610.1| OSJNBa0091D06.25 [Oryza sativa (japonica cultivar-group)] >gnl|BL_ORD_ID|1525100 gi|32488097|emb|CAE02779.1| OSJNBa0011L07.3 [Oryza sativa (japonica cultivar-group)] >gnl|BL_ORD_ID|1525100 gi|50926979|ref|XP_473347.1| OSJ	11670.m04432|LOC_Os04g45600|protein hypothetical protein e_value:3.13225e-87	0.396	(0.1370 0.3460)	0.137	0.346
2144	WT003_O01_CLUSTER_27908	gi|15217311|gb|AAK92655.1| Putative vacuolar sorting receptor protein homolog [Oryza sativa] e_value:0	11676.m01717|LOC_Os10g20630|protein PA domain, putative e_value:0	0.1	(0.0565 0.5647)	0.0565	0.5647
2145	WT003_N04_CLUSTER_29946	gi|50913187|ref|XP_468001.1| putative HYS1 [Oryza sativa (japonica cultivar-group)] >gnl|BL_ORD_ID|1583116 gi|46805415|dbj|BAD16917.1| putative HYS1 [Oryza sativa (japonica cultivar-group)] e_value:0	11668.m05204|LOC_Os02g53070|protein expressed protein e_value:0	0.254	(0.1401 0.5526)	0.1401	0.5526
2146	WT003_G19_CLUSTER_23747	gi|55769783|ref|XP_549881.1| unknown protein [Oryza sativa (japonica cultivar-group)] >gnl|BL_ORD_ID|1753292 gi|52076271|dbj|BAD45056.1| unknown protein [Oryza sativa (japonica cultivar-group)] >gnl|BL_ORD_ID|1753292 gi|52075724|dbj|BAD44944.1| unkn	11667.m00125|LOC_Os01g02150|protein hypothetical protein e_value:8.09752e-56	0.327	(0.1844 0.5641)	0.1844	0.5641
2147	WT002_P13_CLUSTER_7600	gi|15238813|ref|NP_197343.1| expressed protein [Arabidopsis thaliana] e_value:1.33243e-118	11686.m00925|LOC_Os12g09610|protein expressed protein	0.153	(0.0797 0.5193)	0.0797	0.5193
2148	WT002_N15_CLUSTER_14795	gi|50900836|ref|XP_462851.1| B1146F03.16 [Oryza sativa (japonica cultivar-group)] >gnl|BL_ORD_ID|2368922 gi|11967921|dbj|BAB19776.1| contains EST AU078264(S21150)~unknown protein [Oryza sativa (japonica cultivar-group)]	11667.m01947|LOC_Os01g19740|protein CP12 domain, putative e_value:1.32148e-43	0.362	(0.1895 0.5235)	0.1895	0.5235
2149	WT002_L02_CLUSTER_14275	gi|3108209|gb|AAC17220.1| novel cap-binding protein nCBP [Arabidopsis thaliana] >gnl|BL_ORD_ID|693404 gi|25453555|pir||T52138 eukaryotic cap-binding protein [imported] - Arabidopsis thaliana e_value:5.95475e-92	11669.m01553|LOC_Os03g15590|protein eukaryotic cap-binding protein [imported] - Arabidopsis thaliana e_value:3.71709e-116	0.135	(0.0690 0.5113)	0.069	0.5113
2150	WT002_I21_CLUSTER_14524	gi|50948717|ref|XP_493716.1| putative lung seven transmembrane receptor 1 [Oryza sativa (japonica cultivar-group)] >gnl|BL_ORD_ID|1752609 gi|28190667|gb|AAO33145.1| unknown [Oryza sativa (japonica cultivar-group)] >gnl|BL_ORD_ID|1752609 gi|6006379|d	11680.m00333|LOC_Os06g04130|protein hypothetical protein e_value:0	0.227	(0.0749 0.3301)	0.0749	0.3301
2151	WT002_G12_CLUSTER_3158	gi|53792305|dbj|BAD53012.1| subtilisin-like serine proteinase [Oryza sativa (japonica cultivar-group)] e_value:0	11667.m05778|LOC_Os01g58240|protein Subtilase family, putative e_value:0	0.203	(0.2389 1.1776)	0.2389	1.1776
2152	SET6_E13_CLUSTER_7600	gi|15238813|ref|NP_197343.1| expressed protein [Arabidopsis thaliana] e_value:1.53097e-118	11686.m00925|LOC_Os12g09610|protein expressed protein	0.153	(0.0797 0.5193)	0.0797	0.5193
2153	SET3_O18_CLUSTER_5821	gi|50937475|ref|XP_478265.1| putative MATE efflux protein family protein [Oryza sativa (japonica cultivar-group)] >gi|34394668|dbj|BAC83974.1| putative MATE efflux protein family protein [Oryza sativa (japonica cultivar-group)] e_value:0	11673.m03042|LOC_Os07g31880|protein MATE efflux family protein e_value:0	0.196	(0.1200 0.6114)	0.12	0.6114
2154	SET3_O01_CLUSTER_17853	gi|56784791|dbj|BAD82012.1| unknown protein [Oryza sativa (japonica cultivar-group)] >gi|56784212|dbj|BAD81707.1| unknown protein [Oryza sativa (japonica cultivar-group)] e_value:8.96811e-135	11667.m06519|LOC_Os01g64930|protein hypothetical protein e_value:2.47966e-136	0.156	(0.0696 0.4472)	0.0696	0.4472
2155	SET3_K19_CLUSTER_25236	gi|28950767|gb|AAO63307.1| At5g59770 [Arabidopsis thaliana] >gi|26453242|dbj|BAC43694.1| unknown protein [Arabidopsis thaliana] e_value:1.71385e-60	11682.m03652|LOC_Os05g38590|protein Protein tyrosine phosphatase-like protein, PTPLA, putative e_value:9.78366e-108	0.21	(0.0862 0.4110)	0.0862	0.411
2156	SET3_I17_CLUSTER_14693	gi|56784955|dbj|BAD82485.1| receptor serine/threonine kinase PR5K-like [Oryza sativa (japonica cultivar-group)] e_value:0	11667.m04828|LOC_Os01g49620|protein Protein kinase domain, putative e_value:4.52737e-167	0.413	(0.3510 0.8490)	0.351	0.849
2157	SET2_P06_CLUSTER_3786	gi|56785235|dbj|BAD82123.1| hypothetical protein [Oryza sativa (japonica cultivar-group)] >gi|56784313|dbj|BAD82239.1| hypothetical protein [Oryza sativa (japonica cultivar-group)] e_value:1.31998e-66	11667.m06663|LOC_Os01g66320|protein hypothetical protein e_value:1.24518e-68	0.167	(0.0492 0.2953)	0.0492	0.2953
2158	SET2_O01_CLUSTER_15859	gi|15293221|gb|AAK93721.1| unknown protein [Arabidopsis thaliana] >gi|13430752|gb|AAK25998.1| unknown protein [Arabidopsis thaliana] >gi|10178112|dbj|BAB11405.1| unnamed protein product [Arabidopsis thaliana] >gi|15240088|ref|NP_196274.1| express	11687.m03383|LOC_Os11g36740|protein expressed protein e_value:1.38538e-118	0.417	(0.2287 0.5488)	0.2287	0.5488
2159	SET2_A09_CLUSTER_9694	gi|51963664|ref|XP_506584.1| PREDICTED OJ1165_F02.106 gene product [Oryza sativa (japonica cultivar-group)] >gi|34898102|ref|NP_910397.1| unknown protein [Oryza sativa (japonica cultivar-group)] >gi|24059882|dbj|BAC21348.1| unknown protein [Oryza sa	11673.m04854|LOC_Os07g48840|protein Ser/Thr protein phosphatase, putative e_value:2.65441e-175	0.069	(0.0556 0.8057)	0.0556	0.8057
2160	SET1_M10_CLUSTER_29472	gi|50924127|ref|XP_472424.1| OJ991214_12.11 [Oryza sativa (japonica cultivar-group)] >gi|32479735|emb|CAE01522.1| OJ991214_12.11 [Oryza sativa (japonica cultivar-group)] e_value:3.23992e-45	11670.m03263|LOC_Os04g33920|protein Protease inhibitor/seed storage/LTP family, putative e_value:2.62379e-47	0.559	(0.1702 0.3046)	0.1702	0.3046
2161	SET1_J19_CLUSTER_15773	gi|50935781|ref|XP_477418.1| putative B12D protein [Oryza sativa (japonica cultivar-group)] >gi|34395047|dbj|BAC84630.1| putative B12D protein [Oryza sativa (japonica cultivar-group)] >gi|50508847|dbj|BAD31622.1| putative B12D protein [Oryza sativa	11673.m01696|LOC_Os07g17310|protein B12D protein e_value:1.28486e-39	0.076	(0.0747 0.9797)	0.0747	0.9797
2162	SET1_B16_CLUSTER_10505	gi|34907986|ref|NP_915340.1| P0446G04.25 [Oryza sativa (japonica cultivar-group)] >gi|20160656|dbj|BAB89601.1| unknown protein [Oryza sativa (japonica cultivar-group)] >gi|20805244|dbj|BAB92910.1| unknown protein [Oryza sativa (japonica cultivar-gro	11667.m06136|LOC_Os01g61580|protein hypothetical protein e_value:1.2477e-104	0.327	(0.1452 0.4437)	0.1452	0.4437
2163	WT013_C23_CLUSTER_12279	gi|56784869|dbj|BAD82109.1| putative recA protein [Oryza sativa (japonica cultivar-group)] >gnl|BL_ORD_ID|803730 gi|56784510|dbj|BAD82767.1| putative recA protein [Oryza sativa (japonica cultivar-group)] e_value:0	11667.m06797|LOC_Os01g67510|protein recA protein, putative e_value:1.47541e-172	0.182	(0.0894 0.4907)	0.0894	0.4907
2164	WT012_O15_CLUSTER_30200	gi|34451568|gb|AAQ72348.1| Rho GTPase activating protein 2 [Oryza sativa (japonica cultivar-group)] e_value:0	11668.m04406|LOC_Os02g45600|protein RhoGAP domain, putative e_value:0	0.246	(0.1243 0.5053)	0.1243	0.5053
2165	WT012_H10_CLUSTER_4947	gi|55296294|dbj|BAD68074.1| putative calcium-dependent protein kinase [Oryza sativa (japonica cultivar-group)] >gnl|BL_ORD_ID|971395 gi|55297649|dbj|BAD68220.1| putative calcium-dependent protein kinase [Oryza sativa (japonica cultivar-group)] e_value:	11667.m05895|LOC_Os01g59360|protein Protein kinase domain, putative e_value:0	0.089	(0.0410 0.4614)	0.041	0.4614
2166	WT012_G16_CLUSTER_8849	gi|32479666|emb|CAE01495.1| P0041A24.7 [Oryza sativa (japonica cultivar-group)] >gnl|BL_ORD_ID|1588356 gi|50924546|ref|XP_472633.1| P0041A24.7 [Oryza sativa (japonica cultivar-group)] e_value:1.07662e-136	11670.m03499|LOC_Os04g36630|protein expressed protein e_value:3.64125e-134	0.207	(0.1419 0.6870)	0.1419	0.687
2167	WT011_J09_CLUSTER_273	gi|62701876|gb|AAX92949.1| At2g24280/F27D4.19 [Oryza sativa (japonica cultivar-group)] e_value:0	11687.m00496|LOC_Os11g05760|protein At2g24280/F27D4.19 e_value:0	0.474	(0.2386 0.5030)	0.2386	0.503
2168	WT011_H10_CLUSTER_22220	gi|34898234|ref|NP_910463.1| leaf senescence related protein-like [Oryza sativa (japonica cultivar-group)] >gnl|BL_ORD_ID|1534741 gi|29837187|dbj|BAC75569.1| leaf senescence related protein-like [Oryza sativa (japonica cultivar-group)] e_value:1.56751e	11669.m01835|LOC_Os03g18140|protein expressed protein e_value:0	0.416	(0.1018 0.2446)	0.1018	0.2446
2169	WT011_B05_CLUSTER_23452	gi|50945165|ref|XP_482110.1| putative polygalacturonase isoenzyme 1 beta subunit homolog [Oryza sativa (japonica cultivar-group)] >gnl|BL_ORD_ID|1605832 gi|51964862|ref|XP_507215.1| PREDICTED P0709D11.28 gene product [Oryza sativa (japonica cultivar-gr	11674.m02866|LOC_Os08g29200|protein BURP domain, putative e_value:0	0.31	(0.1689 0.5450)	0.1689	0.545
2170	WT009_O17_CLUSTER_23706	gi|50910285|ref|XP_466631.1| putative met-10+ protein [Oryza sativa (japonica cultivar-group)] >gnl|BL_ORD_ID|1578030 gi|47497926|dbj|BAD20131.1| putative met-10+ protein [Oryza sativa (japonica cultivar-group)] e_value:2.19084e-178	11668.m03773|LOC_Os02g39370|protein Met-10+ like-protein, putative e_value:0	0.307	(0.1525 0.4971)	0.1525	0.4971
2171	WT008_N24_CLUSTER_25767	gi|54290950|dbj|BAD61631.1| putative HEN4 [Oryza sativa (japonica cultivar-group)] e_value:0	11680.m02310|LOC_Os06g23460|protein At5g53060 e_value:0	0.237	(0.0976 0.4128)	0.0976	0.4128
2172	WT008_A16_CLUSTER_17410	gi|50932389|ref|XP_475722.1| unknown protein [Oryza sativa (japonica cultivar-group)] >gnl|BL_ORD_ID|1598091 gi|46575963|gb|AAT01324.1| unknown protein [Oryza sativa (japonica cultivar-group)] e_value:0	11682.m04211|LOC_Os05g44190|protein expressed protein e_value:0	0.7	(0.3691 0.5272)	0.3691	0.5272
2173	WT008_C09_CLUSTER_28263	gi|38345951|emb|CAE04340.2| OSJNBb0038F03.4 [Oryza sativa (japonica cultivar-group)] >gnl|BL_ORD_ID|1523082 gi|50927285|ref|XP_473380.1| OSJNBb0038F03.4 [Oryza sativa (japonica cultivar-group)] e_value:0	11670.m04471|LOC_Os04g45960|protein serine proteinase e_value:0	0.287	(0.1181 0.4119)	0.1181	0.4119
2174	WT007_A01_CLUSTER_11100	gi|50912669|ref|XP_467742.1| putative MAP kinase kinase kinase [Oryza sativa (japonica cultivar-group)] >gnl|BL_ORD_ID|1582855 gi|46390625|dbj|BAD16108.1| putative MAP kinase kinase kinase [Oryza sativa (japonica cultivar-group)] e_value:0	11668.m04970|LOC_Os02g50970|protein Protein kinase domain, putative e_value:0	0.203	(0.1042 0.5131)	0.1042	0.5131
2175	WT006_P11_CLUSTER_8622	gi|50905027|ref|XP_464002.1| unknown protein [Oryza sativa (japonica cultivar-group)] >gnl|BL_ORD_ID|1566246 gi|41052560|dbj|BAD07742.1| unknown protein [Oryza sativa (japonica cultivar-group)] e_value:1.21317e-157	11668.m00310|LOC_Os02g03790|protein expressed protein e_value:1.42576e-158	0.194	(0.1637 0.8426)	0.1637	0.8426

PDF Complete
Your complimentary use period has ended. Thank you for using PDF Complete.
Click Here to upgrade to Unlimited Pages and Expanded Features

<table>
<tr><td>2176</td><td>WT013_F14_CLUSTER_13855</td><td>gi|57899842|dbj|BAD87626.1| sucrose phosphate synthase [Oryza sativa (japonica cultivar-group)] e_value:0</td><td>11667.m06959|LOC_Os01g69030|protein glycosyl transferase, group 1 family protein, putative e_value:0</td><td>0.171</td><td>(0.0928 0.5440)</td><td>0.0928</td><td>0.544</td></tr>
<tr><td>2177</td><td>WT005_D13_CLUSTER_17204</td><td>gi|34909242|ref|NP_915968.1| putative L-ascorbate oxidase homolog [Oryza sativa (japonica cultivar-group)] >gnl|BL_ORD_ID|1552935 gi|15528783|dbj|BAB64824.1| putative L-ascorbate oxidase [Oryza sativa (japonica cultivar-group)] e_value:0</td><td>11667.m05976|LOC_Os01g60080|protein Multicopper oxidase, putative e_value:0</td><td>0.614</td><td>(0.1456 0.2374)</td><td>0.1456</td><td>0.2374</td></tr>
<tr><td>2178</td><td>WT010_A20_CLUSTER_31124</td><td>gi|31745238|gb|AAP68898.1| hypothetical protein [Oryza sativa (japonica cultivar-group)] >gnl|BL_ORD_ID|2050093 gi|34896880|ref|NP_909784.1| hypothetical protein [Oryza sativa (japonica cultivar-group)] e_value:0</td><td>11669.m01351|LOC_Os03g13790|protein Myb-like DNA-binding domain, putative e_value:0</td><td>0.295</td><td>(0.1526 0.5179)</td><td>0.1526</td><td>0.5179</td></tr>
<tr><td>2179</td><td>WT009_H16_CLUSTER_7395</td><td>gi|31431820|gb|AAP53539.1| Centrin [Oryza sativa (japonica cultivar-group)] >gnl|BL_ORD_ID|2010984 gi|37533900|ref|NP_921252.1| Centrin [Oryza sativa (japonica cultivar-group)] >gnl|BL_ORD_ID|2010984 gi|13129449|gb|AAK13107.1| Centrin [Oryza sativa]</td><td>11676.m02114|LOC_Os10g25010|protein EF hand, putative e_value:9.08967e-76</td><td>0.218</td><td>(0.1282 0.5887)</td><td>0.1282</td><td>0.5887</td></tr>
<tr><td>2180</td><td>SET2_G18_CLUSTER_34355</td><td>gi|50910033|ref|XP_466505.1| putative protein kinase [Oryza sativa (japonica cultivar-group)] >gi|46805026|dbj|BAD16891.1| putative protein kinase [Oryza sativa (japonica cultivar-group)] e_value:0</td><td>11668.m03642|LOC_Os02g38080|protein Protein kinase domain, putative e_value:0</td><td>0.061</td><td>(0.0312 0.5079)</td><td>0.0312</td><td>0.5079</td></tr>
<tr><td>2181</td><td>SET3_B11_CLUSTER_28927</td><td>gi|21536847|gb|AAM61179.1| unknown [Arabidopsis thaliana] >gi|20197958|gb|AAD21730.2| expressed protein [Arabidopsis thaliana] >gi|20148709|gb|AAM10245.1| unknown protein [Arabidopsis thaliana] >gi|16226328|gb|AAL16136.1| At2g42780/F7D19.22 [Arab</td><td>11674.m00662|LOC_Os08g07280|protein expressed protein e_value:9.02287e-81</td><td>0.353</td><td>(0.2482 0.7026)</td><td>0.2482</td><td>0.7026</td></tr>
<tr><td>2182</td><td>WT012_A11_CLUSTER_1310</td><td>gi|50933517|ref|XP_476286.1| hypothetical protein~similar to Oryza sativa chromosome 10, OSJNBa0005K07.2 [Oryza sativa (japonica cultivar-group)] e_value:0</td><td>11680.m00046|LOC_Os06g01450|protein Putative methyltransferase e_value:0</td><td>0.109</td><td>(0.0929 0.8565)</td><td>0.0929</td><td>0.8565</td></tr>
<tr><td>2183</td><td>WT004_I15_CLUSTER_5227</td><td>gi|50938719|ref|XP_478887.1| homeodomain protein-like [Oryza sativa (japonica cultivar-group)] >gnl|BL_ORD_ID|1529787 gi|50509227|dbj|BAD30497.1| homeodomain protein-like [Oryza sativa (japonica cultivar-group)] >gnl|BL_ORD_ID|1529787 gi|33146844|db</td><td>11673.m03830|LOC_Os07g39320|protein Homeobox domain, putative e_value:4.18367e-94</td><td>0.213</td><td>(0.1449 0.6791)</td><td>0.1449</td><td>0.6791</td></tr>
<tr><td>2184</td><td>WT004_B23_CLUSTER_15547</td><td>gi|52353701|gb|AAU44267.1| putative oligosaccharyl transferase STT3 [Oryza sativa (japonica cultivar-group)] >gnl|BL_ORD_ID|1283069 gi|50080325|gb|AAT69659.1| putative oligosaccharyl transferase STT3 subunit [Oryza sativa (japonica cultivar-group)] e_v</td><td>11682.m04234|LOC_Os05g44360|protein oligosaccharyl transferase STT3, putative e_value:0</td><td>0.107</td><td>(0.0429 0.4027)</td><td>0.0429</td><td>0.4027</td></tr>
<tr><td>2185</td><td>SET5_F24_CLUSTER_33417</td><td>gi|51964362|ref|XP_506966.1| PREDICTED OJ1734_E02.21-2 gene product [Oryza sativa (japonica cultivar-group)] >gi|50912677|ref|XP_467746.1| putative NEP1-interacting protein [Oryza sativa (japonica cultivar-group)] >gi|46390629|dbj|BAD16112.1| putati</td><td>11668.m04975|LOC_Os02g50990|protein Zinc finger, C3HC4 type (RING finger), putative e_value:1.35647e-86</td><td>0.231</td><td>(0.1835 0.7931)</td><td>0.1835</td><td>0.7931</td></tr>
<tr><td>2186</td><td>WT013_F13_CLUSTER_11166</td><td>gi|62733540|gb|AAX95657.1| hypothetical protein [Oryza sativa (japonica cultivar-group)] e_value:2.88597e-104</td><td>11669.m05064|LOC_Os03g50280|protein expressed protein e_value:5.76519e-106</td><td>0.175</td><td>(0.0823 0.4701)</td><td>0.0823</td><td>0.4701</td></tr>
<tr><td>2187</td><td>WT004_E03_CLUSTER_26614</td><td>gi|45357048|gb|AAS58477.1| unknown [Hordeum vulgare subsp. vulgare] e_value:5.47861e-106</td><td>11669.m00022|LOC_Os03g01210|protein hypothetical protein e_value:1.6183e-104</td><td>0.154</td><td>(0.1553 1.0116)</td><td>0.1553</td><td>1.0116</td></tr>
<tr><td>2188</td><td>WT003_P17_CLUSTER_23792</td><td>gi|50934997|ref|XP_477026.1| putative coatomer protein gamma 2-subunit [Oryza sativa (japonica cultivar-group)] >gnl|BL_ORD_ID|1522560 gi|34394800|dbj|BAC84213.1| putative coatomer protein gamma 2-subunit [Oryza sativa (japonica cultivar-</td><td>11669.m01221|LOC_Os03g12590|protein coatomer gamma-2 subunit (gamma-2 coat protein) (gamma-2 cop) e_value:0</td><td>0.08</td><td>(0.0334 0.4155)</td><td>0.0334</td><td>0.4155</td></tr>
<tr><td>2189</td><td>WT003_I11_CLUSTER_14333</td><td>gi|44894802|gb|AAS48882.1| expansin EXPB4 [Triticum aestivum] e_value:1.27547e-150</td><td>11669.m00069|LOC_Os03g01650|protein Pollen allergen, putative e_value:3.57849e-108</td><td>0.55</td><td>(0.2335 0.4244)</td><td>0.2335</td><td>0.4244</td></tr>
<tr><td>2190</td><td>SET4_N01_CLUSTER_31218</td><td>gi|56783742|dbj|BAD81154.1| unknown protein [Oryza sativa (japonica cultivar-group)] e_value:1.26703e-95</td><td>11667.m01717|LOC_Os01g17070|protein expressed protein e_value:3.07667e-94</td><td>0.393</td><td>(0.4244 1.0805)</td><td>0.4244</td><td>1.0805</td></tr>
<tr><td>2191</td><td>SET4_L07_CLUSTER_27925</td><td>gi|50946803|ref|XP_482929.1| putative PrMC3 [Oryza sativa (japonica cultivar-group)] >gi|51964970|ref|XP_507269.1| PREDICTED P0451G12.24 gene product [Oryza sativa (japonica cultivar-group)] >gi|42408051|dbj|BAD09193.1| putative PrMC3 [Oryza sativa</td><td>11674.m03685|LOC_Os08g37060|protein expressed protein e_value:6.53556e-153</td><td>0.458</td><td>(0.1067 0.2331)</td><td>0.1067</td><td>0.2331</td></tr>
<tr><td>2192</td><td>SET3_E16_CLUSTER_23874</td><td>gi|7211975|gb|AAF40446.1| Similar to the auxin-independent growth promoter (axi 1) gene product from Nicotiana tabacum gb|X80301. ESTs gb|T88041, gb|AA394631 and gb|AA720157 come from this gene. [Arabidopsis thaliana] >gi|25336203|pir||E86182 hypothet</td><td>11687.m02649|LOC_Os11g29120|protein Plant protein family, putative e_value:0</td><td>0.154</td><td>(0.0829 0.5390)</td><td>0.0829</td><td>0.539</td></tr>
<tr><td>2193</td><td>WT008_H04_CLUSTER_15058</td><td>gi|34915188|ref|NP_919051.1| unknown protein [Oryza sativa (japonica cultivar-group)] >gnl|BL_ORD_ID|1564919 gi|30017493|gb|AAP12915.1| hypothetical protein Os03g40370 [Oryza sativa (japonica cultivar-group)] e_value:5.40377e-75</td><td>11670.m04647|LOC_Os04g47550|protein F-box domain, putative e_value:6.39642e-79</td><td>0.22</td><td>(0.5060 2.2997)</td><td>0.506</td><td>2.2997</td></tr>
<tr><td>2194</td><td>WT007_D21_CLUSTER_20855</td><td>gi|50938307|ref|XP_478681.1| hypothetical protein [Oryza sativa (japonica cultivar-group)] >gnl|BL_ORD_ID|1528603 gi|34393355|dbj|BAC83333.1| hypothetical protein [Oryza sativa (japonica cultivar-group)] e_value:4.2369e-37</td><td>11673.m03571|LOC_Os07g36890|protein F-box domain, putative e_value:1.33596e-38</td><td></td><td>(0.9116 -1.0000)</td><td>0.9116</td><td>-1</td></tr>
<tr><td>2195</td><td>WT005_J16_CLUSTER_23874</td><td>gi|31711926|gb|AAP68319.1| At1g04910 [Arabidopsis thaliana] >gnl|BL_ORD_ID|2049862 gi|22329335|ref|NP_171983.2| expressed protein [Arabidopsis thaliana] >gnl|BL_ORD_ID|2049862 gi|17065372|gb|AAL32840.1| Similar to auxin-independent growth promoter (</td><td>11687.m02649|LOC_Os11g29120|protein Plant protein family, putative e_value:0</td><td>0.152</td><td>(0.0820 0.5383)</td><td>0.082</td><td>0.5383</td></tr>
<tr><td>2196</td><td>WT003_E01_CLUSTER_22896</td><td>gi|2501850|gb|AAB80717.1| GDP dissociation inhibitor [Nicotiana tabacum] >gnl|BL_ORD_ID|296381 gi|7489133|pir||T01782 GDP dissociation inhibitor - common tobacco e_value:0</td><td>11669.m01699|LOC_Os03g16900|protein GDP dissociation inhibitor - Arabidopsis thaliana e_value:0</td><td>0.058</td><td>(0.0306 0.5287)</td><td>0.0306</td><td>0.5287</td></tr>
<tr><td>2197</td><td>WT008_D04_CLUSTER_29384</td><td>gi|50946295|ref|XP_482675.1| putative phosphoglycerate dehydrogenase [Oryza sativa (japonica cultivar-group)] >gnl|BL_ORD_ID|1608485 gi|42409460|dbj|BAD09817.1| putative phosphoglycerate dehydrogenase [Oryza sativa (japonica cultivar-group)] >gnl|BL</td><td>11674.m03440|LOC_Os08g34720|protein D-3-phosphoglycerate dehydrogenase e_value:0</td><td>0.185</td><td>(0.0851 0.4592)</td><td>0.0851</td><td>0.4592</td></tr>
<tr><td>2198</td><td>SET3_K16_CLUSTER_29384</td><td>gi|50946295|ref|XP_482675.1| putative phosphoglycerate dehydrogenase [Oryza sativa (japonica cultivar-group)] >gi|42409460|dbj|BAD09817.1| putative phosphoglycerate dehydrogenase [Oryza sativa (japonica cultivar-group)] >gi|42408279|dbj|BAD09434.1|</td><td>11674.m03440|LOC_Os08g34720|protein D-3-phosphoglycerate dehydrogenase e_value:0</td><td>0.158</td><td>(0.0795 0.5039)</td><td>0.0795</td><td>0.5039</td></tr>
<tr><td>2199</td><td>WT009_O09_CLUSTER_7879</td><td>gi|50904985|ref|XP_463981.1| putative heat shock protein 40 [Oryza sativa (japonica cultivar-group)] >gnl|BL_ORD_ID|1566223 gi|41053046|dbj|BAD07976.1| putative heat shock protein 40 [Oryza sativa (japonica cultivar-group)] e_value:2.11936e-163</td><td>11668.m00290|LOC_Os02g03600|protein DnaJ domain, putative e_value:2.07659e-154</td><td>0.172</td><td>(0.0975 0.5653)</td><td>0.0975</td><td>0.5653</td></tr>
<tr><td>2200</td><td>SET5_K22_CLUSTER_33633</td><td>gi|34902246|ref|NP_912469.1| Unknown protein [Oryza sativa (japonica cultivar-group)] >gi|20330746|gb|AAM19109.1| Unknown protein [Oryza sativa (japonica cultivar-group)] e_value:1.60038e-69</td><td>11686.m02228|LOC_Os12g23280|protein Heavy-metal-associated domain, putative e_value:1.53293e-84</td><td>0.343</td><td>(0.1217 0.3545)</td><td>0.1217</td><td>0.3545</td></tr>
<tr><td>2201</td><td>WT007_I17_CLUSTER_28936</td><td>gi|52077302|dbj|BAD46344.1| hypothetical protein [Oryza sativa (japonica cultivar-group)] >gnl|BL_ORD_ID|381845 gi|50725868|dbj|BAD33397.1| hypothetical protein [Oryza sativa (japonica cultivar-group)] e_value:0</td><td>11681.m02836|LOC_Os09g30350|protein hypothetical protein e_value:0</td><td>0.315</td><td>(0.1940 0.6159)</td><td>0.194</td><td>0.6159</td></tr>
<tr><td>2202</td><td>WT003_L03_CLUSTER_31968</td><td>gi|34903256|ref|NP_912975.1| unnamed protein product [Oryza sativa (japonica cultivar-group)] >gnl|BL_ORD_ID|1752467 gi|6539559|dbj|BAA88176.1| putative zwille protein [Oryza sativa (japonica cultivar-group)] e_value:0</td><td>11670.m00587|LOC_Os04g06770|protein Piwi domain, putative e_value:0</td><td>0.1</td><td>(0.1095 1.0933)</td><td>0.1095</td><td>1.0933</td></tr>
<tr><td>2203</td><td>WT007_A14_CLUSTER_28583</td><td>gi|12321945|gb|AAG51002.1| ankyrin-like protein; 93648-91299 [Arabidopsis thaliana] >gnl|BL_ORD_ID|303224 gi|15230470|ref|NP_187842.1| ankyrin repeat family protein [Arabidopsis thaliana] e_value:0</td><td>11669.m01741|LOC_Os03g17240|protein ankyrin-like protein e_value:0</td><td>0.109</td><td>(0.0537 0.4920)</td><td>0.0537</td><td>0.492</td></tr>
<tr><td>2204</td><td>WT006_F03_CLUSTER_19876</td><td>gi|22088|emb|CAA41250.1| chalcone synthase [Hordeum vulgare] >gnl|BL_ORD_ID|2324476 gi|100601|pir||S16275 naringenin-chalcone synthase (EC 2.3.1.74) - barley >gnl|BL_ORD_ID|2324476 gi|116393|sp|P26018|CHS1_HORVU Chalcone synthase 1 (Naringenin-chalc</td><td>11687.m03006|LOC_Os11g32650|protein Chalcone and stilbene synthases, N-terminal domain, putative e_value:0</td><td>0.122</td><td>(0.0509 0.4185)</td><td>0.0509</td><td>0.4185</td></tr>
<tr><td>2205</td><td>WT003_K24_CLUSTER_34621</td><td>gi|38502276|emb|CAD57742.1| RAC-ROP-like G-protein [Hordeum vulgare subsp. vulgare] e_value:6.35768e-119</td><td>11668.m04958|LOC_Os02g50860|protein Ras family, putative e_value:6.05651e-115</td><td>0.065</td><td>(0.0287 0.4412)</td><td>0.0287</td><td>0.4412</td></tr>
<tr><td>2206</td><td>WT006__C22_CLUSTER_14752</td><td>gi|53792246|dbj|BAD52879.1| ATP synthase beta subunit/transcription termination factor rho-like [Oryza sativa (japonica cultivar-group)] e_value:0</td><td>11667.m03906|LOC_Os01g40620|protein expressed protein e_value:0</td><td>0.14</td><td>(0.0878 0.6260)</td><td>0.0878</td><td>0.626</td></tr>
<tr><td>2207</td><td>SET4_K08_CLUSTER_33211</td><td>gi|50905795|ref|XP_464386.1| unknown protein [Oryza sativa (japonica cultivar-group)] >gi|46390050|dbj|BAD15426.1| unknown protein [Oryza sativa (japonica cultivar-group)] >gi|46389896|dbj|BAD15517.1| unknown protein [Oryza sativa (japonica cultivar</td><td>11668.m00870|LOC_Os02g09470|protein expressed protein e_value:1.94376e-109</td><td>0.268</td><td>(0.1187 0.4432)</td><td>0.1187</td><td>0.4432</td></tr>
<tr><td>2208</td><td>SET2_N03_CLUSTER_32188</td><td>gi|50904385|ref|XP_463681.1| OSJNBa0093F16.11 [Oryza sativa (japonica cultivar-group)] >gi|20805224|dbj|BAB92891.1| putative pectin-glucuronyltransferase [Oryza sativa (japonica cultivar-group)] >gi|20160726|dbj|BAB89668.1| hypothetical protein~simi</td><td>11667.m07028|LOC_Os01g70180|protein Exostosin family, putative e_value:0</td><td>0.223</td><td>(0.1191 0.5337)</td><td>0.1191</td><td>0.5337</td></tr>
<tr><td>2209</td><td>WT007_E24_CLUSTER_27927</td><td>gi|50928983|ref|XP_474019.1| OSJNBa0043L09.15 [Oryza sativa (japonica cultivar-group)] >gnl|BL_ORD_ID|1592680 gi|38344795|emb|CAE02996.2| OSJNBa0043L09.15 [Oryza sativa (japonica cultivar-group)] e_value:0</td><td>11670.m05325|LOC_Os04g54200|protein Diacylglycerol kinase catalytic domain (presumed), putative e_value:0</td><td>0.149</td><td>(0.0938 0.6293)</td><td>0.0938</td><td>0.6293</td></tr>
<tr><td>2210</td><td>WT004_P04_CLUSTER_32496</td><td>gi|50917231|ref|XP_469012.1| putative yabby protein [Oryza sativa (japonica cultivar-group)] >gnl|BL_ORD_ID|1585064 gi|3859570|gb|AAC72848.1| unknown [Oryza sativa] >gnl|BL_ORD_ID|1585064 gi|11280864|pir||T51588 hypothetical protein 2 [imported] -</td><td>11669.m04455|LOC_Os03g44710|protein putative yabby protein e_value:1.0983e-87</td><td>0.116</td><td>(0.0713 0.6157)</td><td>0.0713</td><td>0.6157</td></tr>
<tr><td>2211</td><td>SET4_B23_CLUSTER_31878</td><td>gi|51090829|dbj|BAD35357.1| putative yip1 interacting factor [Oryza sativa (japonica cultivar-group)] >gi|51090475|dbj|BAD35445.1| putative yip1 interacting factor [Oryza sativa (japonica cultivar-group)] e_value:2.66095e-140</td><td>11680.m04073|LOC_Os06g41590|protein Hrf1 family e_value:1.52853e-143</td><td>0.048</td><td>(0.0547 1.1355)</td><td>0.0547</td><td>1.1355</td></tr>
<tr><td>2212</td><td>WT006_F17_CLUSTER_32374</td><td>gi|12802327|gb|AAK07827.1| mitochondrial processing peptidase beta subunit [Cucumis melo] e_value:0</td><td>11669.m01092|LOC_Os03g11410|protein mitochondrial processing peptidase beta subunit e_value:0</td><td>0.146</td><td>(0.0680 0.4653)</td><td>0.068</td><td>0.4653</td></tr>
<tr><td>2213</td><td>WT008_N07_CLUSTER_2527</td><td>gi|50912907|ref|XP_467861.1| putative early-responsive to dehydration stress protein [Oryza sativa (japonica cultivar-group)] >gnl|BL_ORD_ID|1582978 gi|46805951|dbj|BAD17245.1| putative early-responsive to dehydration stress protein [Oryza sativa (japo</td><td>11668.m05070|LOC_Os02g51860|protein Putative methyltransferase e_value:0</td><td>0.196</td><td>(0.0687 0.3515)</td><td>0.0687</td><td>0.3515</td></tr>
<tr><td>2214</td><td>WT009_M15_CLUSTER_33003</td><td>gi|50917579|ref|XP_469186.1| expressed protein [Oryza sativa (japonica cultivar-group)] >gnl|BL_ORD_ID|1586176 gi|40538924|gb|AAR87181.1| expressed protein [Oryza sativa (japonica cultivar-group)] e_value:4.22072e-134</td><td>11669.m04638|LOC_Os03g46410|protein expressed protein e_value:6.83499e-136</td><td>0.146</td><td>(0.0691 0.4735)</td><td>0.0691</td><td>0.4735</td></tr>
<tr><td>2215</td><td>WT008_I05_CLUSTER_32624</td><td>gi|50251688|dbj|BAD27593.1| putative aspartate transaminase [Oryza sativa (japonica cultivar-group)] e_value:0</td><td>11668.m01312|LOC_Os02g14110|protein aspartate transaminase (EC 2.6.1.1) precursor, mitochondrial - rice e_value:0</td><td>0.078</td><td>(0.0408 0.5260)</td><td>0.0408</td><td>0.526</td></tr>
<tr><td>2216</td><td>WT007_A02_CLUSTER_34277</td><td>gi|11992279|gb|AAG42497.1| small GTP-binding protein RAB5B [Oryza sativa] e_value:1.13835e-98</td><td>11669.m00522|LOC_Os03g05740|protein small GTP-binding protein RAB5B e_value:2.18974e-100</td><td>0.123</td><td>(0.0512 0.4162)</td><td>0.0512</td><td>0.4162</td></tr>
<tr><td>2217</td><td>SET3_O11_CLUSTER_7132</td><td>gi|4741844|gb|AAD28731.1| small GTP-binding protein [Triticum aestivum] e_value:1.25988e-111</td><td>11686.m04351|LOC_Os12g43550|protein Ras family, putative e_value:1.26311e-101</td><td>0.045</td><td>(0.0380 0.8403)</td><td>0.038</td><td>0.8403</td></tr>
<tr><td>2218</td><td>SET2_K06_CLUSTER_34388</td><td>gi|50912961|ref|XP_467888.1| putative poly(A) binding protein [Oryza sativa (japonica cultivar-group)] >gi|46805689|dbj|BAD17090.1| putative poly(A) binding protein [Oryza sativa (japonica cultivar-group)] e_value:1.84293e-112</td><td>11668.m05098|LOC_Os02g52140|protein RNA recognition motif. (a.k.a. RRM, RBD, or RNP domain), putative e_value:2.09641e-</td><td>0.047</td><td>(0.0283 0.5995)</td><td>0.0283</td><td>0.5995</td></tr>
</table>

PDF Complete
Your complimentary use period has ended. Thank you for using PDF Complete.
Click Here to upgrade to Unlimited Pages and Expanded Features

2219	WT006_I08_CLUSTER_33702	gi|56784423|dbj|BAD82462.1| phospholipid/glycerol acyltransferase-like protein [Oryza sativa (japonica cultivar-group)] e_value:0	11667.m06366|LOC_Os01g63580|protein A_IG005I10.4 gene product e_value:0	0.222	(0.0565 0.2550)	0.0565	0.255
2220	SET6_I08_CLUSTER_34875	gi|2454184|gb|AAB86804.1| pyruvate dehydrogenase E1 beta subunit [Arabidopsis thaliana] >gi|21593321|gb|AAM65270.1| pyruvate dehydrogenase E1 beta subunit, putative [Arabidopsis thaliana] e_value:7.34447e-160	11686.m04210|LOC_Os12g42230|protein pyruvate dehydrogenase E1 beta subunit e_value:0	0.156	(0.0966 0.6175)	0.0966	0.6175
2221	WT004_O14_CLUSTER_32517	gi|38489178|gb|AAR21278.1| fatty aldehyde dehydrogenase 1 [Zea mays] e_value:0	11670.m04445|LOC_Os04g45720|protein aldehyde dehydrogenase (NAD) family protein e_value:0	0.241	(0.2430 1.0067)	0.243	1.0067
2222	WT005_B21_CLUSTER_22977	gi|50726120|dbj|BAD33641.1| putative hydroxycinnamoyl transferase [Oryza sativa (japonica cultivar-group)] e_value:0	11681.m02310|LOC_Os09g25460|protein Transferase family e_value:0	0.237	(0.0709 0.2989)	0.0709	0.2989
2223	WT004_P01_CLUSTER_22374	gi|15320410|dbj|BAB63914.1| ERD3 protein [Arabidopsis thaliana] >gnl|BL_ORD_ID|390598 gi|30684664|ref|NP_849408.1| early-responsive to dehydration stress protein (ERD3) [Arabidopsis thaliana] >gnl|BL_ORD_ID|390598 gi|18415244|ref|NP_567575.1| early-	11668.m04375|LOC_Os02g45310|protein Putative methyltransferase e_value:3.04998e-155	0.854	(0.5375 0.6296)	0.5375	0.6296
2224	WT006_P14_CLUSTER_26890	gi|50900440|ref|XP_462653.1| OSJNBa0064H22.5 [Oryza sativa (japonica cultivar-group)] >gnl|BL_ORD_ID|1564337 gi|38344824|emb|CAD40878.2| OSJNBa0064H22.5 [Oryza sativa (japonica cultivar-group)] e_value:5.87404e-139	11670.m03536|LOC_Os04g37490|protein oxidoreductase, aldo/keto reductase family, putative e_value:1.62538e-140	0.393	(0.1497 0.3813)	0.1497	0.3813
2225	SET3_H14_CLUSTER_33466	gi|51964266|ref|XP_506918.1| PREDICTED OSJNBa0083F15.4 gene product [Oryza sativa (japonica cultivar-group)] e_value:6.991e-40	11669.m03189|LOC_Os03g31510|protein putative cysteine proteinase inhibitor e_value:5.59896e-42	0.431	(0.1769 0.4101)	0.1769	0.4101
2226	SET5__C02_CLUSTER_34074	gi|50251955|dbj|BAD27890.1| putative vacuolar protein sorting; Vps29p [Oryza sativa (japonica cultivar-group)] e_value:6.60282e-104	11668.m01558|LOC_Os02g16550|protein Similar to VPS29-like phosphoesterase-related protein T23J7.140 [similarity] - Arabidopsis thaliana e_value:3.72294e-99	0.034	(0.0153 0.4547)	0.0153	0.4547
2227	WT004_E06_CLUSTER_33097	gi|50919526|ref|XP_470151.1| putative phospholipase [Oryza sativa] >gnl|BL_ORD_ID|2383606 gi|18855062|gb|AAL79754.1| putative phospholipase [Oryza sativa] >gnl|BL_ORD_ID|2383606 gi|15042826|gb|AAK82449.1| putative phospholipase [Oryza sativa] e_valu	11669.m06224|LOC_Os03g61130|protein putative phospholipase e_value:0	0.153	(0.0676 0.4422)	0.0676	0.4422
2228	SET2_F09_CLUSTER_30173	gi|445116|prf||1908421A light-harvesting complex IIa protein; e_value:1.53284e-162	11673.m03608|LOC_Os07g37240|protein probable chlorophyll a/b-binding protein - rice e_value:1.76206e-148	0.286	(0.0659 0.2302)	0.0659	0.2302
2229	WT007_G14_CLUSTER_31487	gi|57899699|dbj|BAD87419.1| radical SAM domain-containing protein-like [Oryza sativa (japonica cultivar-group)] >gnl|BL_ORD_ID|971991 gi|57899226|dbj|BAD87375.1| radical SAM domain-containing protein-like [Oryza sativa (japonica cultivar-group)] e_valu	11667.m07457|LOC_Os01g74190|protein radical SAM domain protein, putative e_value:0	0.102	(0.0659 0.6468)	0.0659	0.6468
2230	WT006__C09_CLUSTER_14924	gi|50931789|ref|XP_475422.1| unknown protein [Oryza sativa (japonica cultivar-group)] >gnl|BL_ORD_ID|1596259 gi|46576005|gb|AAT01366.1| unknown protein [Oryza sativa (japonica cultivar-group)] e_value:2.07492e-101	11682.m03808|LOC_Os05g40040|protein expressed protein e_value:9.34853e-104	0.476	(0.2944 0.6185)	0.2944	0.6185
2231	WT003_B10_CLUSTER_33673	gi|55773689|dbj|BAD72272.1| putative GAMYB-binding protein [Oryza sativa (japonica cultivar-group)] >gnl|BL_ORD_ID|377741 gi|55773651|dbj|BAD72190.1| putative GAMYB-binding protein [Oryza sativa (japonica cultivar-group)] e_value:0	11680.m00701|LOC_Os06g07540|protein GAMYB-binding protein e_value:0	0.185	(0.0897 0.4840)	0.0897	0.484
2232	WT013_D16_CLUSTER_34807	gi|7024413|emb|CAB75881.1| sucrose transporter 2 [Hordeum vulgare subsp. vulgare] e_value:0	11686.m04438|LOC_Os12g44380|protein sucrose/H+ symporter e_value:0	0.103	(0.0978 0.9533)	0.0978	0.9533
2233	WT007_B21_CLUSTER_32514	gi|34907918|ref|NP_915306.1| putative leucine zipper protein [Oryza sativa (japonica cultivar-group)] >gnl|BL_ORD_ID|1555560 gi|15624046|dbj|BAB68099.1| leucine zipper protein-like [Oryza sativa (japonica cultivar-group)] e_value:0	11682.m03761|LOC_Os05g39610|protein Exo70 exocyst complex subunit e_value:0	0.101	(0.0564 0.5606)	0.0564	0.5606
2234	WT005_H15_CLUSTER_29682	gi|50899994|ref|XP_450785.1| putative beta-glucan binding protein [Oryza sativa (japonica cultivar-group)] >gnl|BL_ORD_ID|1564092 gi|51963760|ref|XP_506660.1| PREDICTED P0027G10.24 gene product [Oryza sativa (japonica cultivar-group)] >gnl|BL_ORD_ID	11681.m01920|LOC_Os09g21210|protein beta-glucan elicitor receptor, putative e_value:0	0.15	(0.0788 0.5254)	0.0788	0.5254
2235	WT010_D09_CLUSTER_6152	gi|50929605|ref|XP_474330.1| OSJNBa0018M05.17 [Oryza sativa (japonica cultivar-group)] >gnl|BL_ORD_ID|1594461 gi|38344926|emb|CAE03242.2| OSJNBa0018M05.17 [Oryza sativa (japonica cultivar-group)] e_value:5.53746e-151	11674.m00006|LOC_Os08g01060|protein hypothetical protein e_value:0	0.496	(0.2890 0.5827)	0.289	0.5827
2236	SET4_N17_CLUSTER_34109	gi|50919823|ref|XP_470272.1| Hypothetical protein [Oryza sativa (japonica cultivar-group)] >gi|22773248|gb|AAN06854.1| Hypothetical protein [Oryza sativa (japonica cultivar-group)] >gi|20219051|gb|AAM15795.1| Hypothetical protein [Oryza sativa (japo	11669.m00635|LOC_Os03g06750|protein hypothetical protein e_value:2.19612e-53	0.557	(0.5868 1.0532)	0.5868	1.0532
2237	WT006_I07_CLUSTER_14122	gi|55233175|gb|AAV48542.1| beta-cyanoalanine synthase [Oryza sativa (indica cultivar-group)] >gnl|BL_ORD_ID|1494239 gi|10241630|emb|CAC09469.1| cysteine synthase [Oryza sativa (indica cultivar-group)] e_value:2.38609e-171	11670.m00746|LOC_Os04g08350|protein cysteine synthase A, putative e_value:1.15963e-172	0.133	(0.1275 0.9606)	0.1275	0.9606
2238	SET2_O20_CLUSTER_553	gi|29367419|gb|AAO72582.1| unknown [Oryza sativa (japonica cultivar-group)] e_value:5.39993e-71	11669.m02491|LOC_Os03g24600|protein methionine-R-sulfoxide reductase e_value:1.76797e-72	0.035	(0.0269 0.7678)	0.0269	0.7678
2239	WT002_O17_CLUSTER_31646	gi|31432762|gb|AAP54355.1| putative thioredoxin-like U5 small ribonucleoprotein particle protein [Oryza sativa (japonica cultivar-group)] >gnl|BL_ORD_ID|2012241 gi|37535532|ref|NP_922068.1| putative thioredoxin-like U5 small ribonucleoprotein particle	11676.m03061|LOC_Os10g34520|protein putative thioredoxin-like U5 small ribonucleoprotein particle protein	0.034	(0.0183 0.5341)	0.0183	0.5341
2240	SET1_H05_CLUSTER_63	gi|34904326|ref|NP_913510.1| unnamed protein product [Oryza sativa (japonica cultivar-group)] e_value:4.72961e-45	11680.m04833|LOC_Os06g48780|protein acidic ribosomal protein P3a - maize e_value:4.46314e-47	0.412	(0.1145 0.2780)	0.1145	0.278
2241	WT006__C23_CLUSTER_32307	gi|57899277|dbj|BAD87678.1| putative PRLI-interacting factor K [Oryza sativa (japonica cultivar-group)] e_value:0	11667.m03324|LOC_Os01g35040|protein Ubiquitin fusion degradation protein UFD1, putative e_value:0	0.107	(0.0610 0.5723)	0.061	0.5723
2242	SET3_N18_CLUSTER_34011	gi|21618056|gb|AAM67106.1| unknown [Arabidopsis thaliana] >gi|20259081|gb|AAM14256.1| unknown protein [Arabidopsis thaliana] >gi|17529030|gb|AAL38725.1| unknown protein [Arabidopsis thaliana] >gi|2924772|gb|AAC04901.1| expressed protein [Arabidop	11669.m00904|LOC_Os03g09210|protein GRIM-19 protein e_value:2.42069e-68	0.158	(0.0758 0.4800)	0.0758	0.48
2243	WT012_L10_CLUSTER_9454	gi|50540680|gb|AAT77837.1| putative peptide transporter 1 [Oryza sativa (japonica cultivar-group)] >gnl|BL_ORD_ID|930760 gi|15076663|dbj|BAB62327.1| peptide transporter [Oryza sativa (japonica cultivar-group)] >gnl|BL_ORD_ID|930760 gi|15076661|dbj|B	11669.m05148|LOC_Os03g51050|protein putative peptide transporter 1 e_value:0	0.082	(0.0586 0.7170)	0.0586	0.717
2244	WT009_D10_CLUSTER_34045	gi|50939537|ref|XP_479296.1| putative calcium/calmodulin-dependent protein kinase CaMK [Oryza sativa (japonica cultivar-group)] >gnl|BL_ORD_ID|1532262 gi|23237898|dbj|BAC16472.1| putative calcium/calmodulin-dependent protein kinase CaMK [Oryza sativa (	11669.m02541|LOC_Os03g25070|protein CaMK1 e_value:0	0.102	(0.0769 0.7510)	0.0769	0.751
2245	WT008_H08_CLUSTER_6249	gi|3152572|gb|AAC17053.1| Contains homology to DNAJ heatshock protein gb|U32803 from Haemophilus influenzae. [Arabidopsis thaliana] >gnl|BL_ORD_ID|342040 gi|7487909|pir||T01052 hypothetical protein YUP8H12R.35 - Arabidopsis thaliana e_value:4.60664e-17	11686.m04162|LOC_Os12g41820|protein S-locus protein 5 e_value:0	0.149	(0.0896 0.5996)	0.0896	0.5996
2246	SET1_N02_CLUSTER_34701	gi|55773745|dbj|BAD72428.1| putative density regulated protein drp1 [Oryza sativa (japonica cultivar-group)] >gi|55773670|dbj|BAD72209.1| putative density regulated protein drp1 [Oryza sativa (japonica cultivar-group)] e_value:2.32835e-96	11667.m00949|LOC_Os01g09890|protein density-regulated protein DRP1 e_value:4.61201e-98	0.138	(0.0663 0.4810)	0.0663	0.481
2247	SET2_E16_CLUSTER_34772	gi|53791965|dbj|BAD54227.1| putative lipase [Oryza sativa (japonica cultivar-group)] e_value:0	11680.m03288|LOC_Os06g34070|protein GDSL-like Lipase/Acylhydrolase e_value:0	0.149	(0.0759 0.5086)	0.0759	0.5086
2248	WT002_N11_CLUSTER_7189	gi|50929519|ref|XP_474287.1| OSJNBa0043A12.24 [Oryza sativa (japonica cultivar-group)] >gnl|BL_ORD_ID|1593220 gi|32488394|emb|CAE02819.1| OSJNBa0043A12.24 [Oryza sativa (japonica cultivar-group)] e_value:0	11670.m05673|LOC_Os04g57400|protein 5-methylthioribose kinase e_value:0	0.144	(0.0669 0.4652)	0.0669	0.4652
2249	SET2_A07_CLUSTER_9024	gi|50932823|ref|XP_475939.1| unknown protein [Oryza sativa (japonica cultivar-group)] >gi|47900287|gb|AAT39155.1| unknown protein [Oryza sativa (japonica cultivar-group)] e_value:1.23715e-69	11682.m04834|LOC_Os05g50220|protein Peroxisomal membrane anchor protein (Pex14p) conserved region, putative e_value:5.07198e-71	0.427	(0.2636 0.6176)	0.2636	0.6176
2250	WT011_A22_CLUSTER_34546	gi|51860687|gb|AAU11463.1| mitochondrial uncoupling protein 2 [Saccharum officinarum] e_value:8.26836e-155	11687.m04519|LOC_Os11g48040|protein Mitochondrial carrier protein, putative e_value:1.62262e-155	0.122	(0.0586 0.4798)	0.0586	0.4798
2251	SET3_L05_CLUSTER_30163	gi|31430657|gb|AAP52538.1| putative serine palmitoyltransferase [Oryza sativa (japonica cultivar-group)] >gi|37531898|ref|NP_920251.1| putative serine palmitoyltransferase [Oryza sativa (japonica cultivar-group)] e_value:0	11669.m01464|LOC_Os03g14800|protein aminotransferase, classes I and II, putative e_value:0	0.079	(0.0484 0.6126)	0.0484	0.6126
2252	WT009_E20_CLUSTER_16104	gi|50912099|ref|XP_467457.1| putative phosphoribosyl pyrophosphate synthetase [Oryza sativa (japonica cultivar-group)] >gnl|BL_ORD_ID|1579221 gi|51979348|ref|XP_507525.1| PREDICTED OJ1479_B12.26 gene product [Oryza sativa (japonica cultivar-group)] &gt	11668.m04698|LOC_Os02g48390|protein ribose-phosphate pyrophosphokinase e_value:5.48271e-168	0.134	(0.0543 0.4041)	0.0543	0.4041
2253	WT002_G01_CLUSTER_33915	gi|38567884|emb|CAD40901.2| OSJNBa0036B21.19 [Oryza sativa (japonica cultivar-group)] >gnl|BL_ORD_ID|1524102 gi|50924776|ref|XP_472739.1| OSJNBa0036B21.19 [Oryza sativa (japonica cultivar-group)] e_value:2.84947e-170	11670.m03653|LOC_Os04g38540|protein non-cell-autonomous protein pathway2 e_value:7.01728e-172	0.312	(0.1161 0.3724)	0.1161	0.3724
2254	WT002_B03_CLUSTER_33162	gi|32488960|emb|CAE04341.1| OSJNBb0038F03.5 [Oryza sativa (japonica cultivar-group)] >gnl|BL_ORD_ID|1523083 gi|50927294|ref|XP_473381.1| OSJNBb0038F03.5 [Oryza sativa (japonica cultivar-group)] e_value:0	11670.m04472|LOC_Os04g45970|protein glutamate dehydrogenase 2 (ec 1.4.1.3) (gdh 2). [mouse-ear cress e_value:0	0.076	(0.0405 0.5329)	0.0405	0.5329
2255	WT012_G21_CLUSTER_25355	gi|31433122|gb|AAP54675.1| putative membrane protein [Oryza sativa (japonica cultivar-group)] >gnl|BL_ORD_ID|2012706 gi|37536172|ref|NP_922388.1| putative membrane protein [Oryza sativa (japonica cultivar-group)] >gnl|BL_ORD_ID|2012706 gi|22122915|g	11669.m00178|LOC_Os03g02530|protein Rhomboid family, putative e_value:2.66514e-154	0.268	(0.1381 0.5162)	0.1381	0.5162
2256	WT004_G13_CLUSTER_33619	gi|21263612|sp|Q9ZRI8|FDH_HORVU Formate dehydrogenase, mitochondrial precursor (NAD-dependent formate dehydrogenase) (FDH) >gnl|BL_ORD_ID|178917 gi|4062934|dbj|BAA36181.1| formate dehydrogenase [Hordeum vulgare subsp. vulgare] e_value:0	11680.m02793|LOC_Os06g29180|protein formate dehydrogenase, mitochondrial precursor (ec 1.2.1.2) (nad-dependent formate dehydrogenase) (fdh) e_value:0	0.057	(0.0299 0.5240)	0.0299	0.524
2257	WT006_A12_CLUSTER_33907	gi|51090495|dbj|BAD35697.1| putative MtN21 [Oryza sativa (japonica cultivar-group)] e_value:1.19054e-145	11680.m01045|LOC_Os06g10750|protein Integral membrane protein DUF6, putative e_value:2.96325e-147	0.549	(0.2103 0.3834)	0.2103	0.3834
2258	SET2_H19_CLUSTER_23284	gi|22136956|gb|AAM91707.1| unknown protein [Arabidopsis thaliana] >gi|19423876|gb|AAL87316.1| unknown protein [Arabidopsis thaliana] >gi|7340708|emb|CAB82951.1| putative protein [Arabidopsis thaliana] >gi|15228739|ref|NP_191796.1| expressed prote	11669.m01989|LOC_Os03g19580|protein expressed protein e_value:0	0.11	(0.0714 0.6474)	0.0714	0.6474
2259	SET1_P04_CLUSTER_11706	gi|50929733|ref|XP_474394.1| OSJNBa0032F06.15 [Oryza sativa (japonica cultivar-group)] >gi|38345558|emb|CAE03432.2| OSJNBa0032F06.15 [Oryza sativa (japonica cultivar-group)] e_value:1.57963e-53	11670.m05801|LOC_Os04g58570|protein C2 domain, putative e_value:1.7619e-55	0.219	(0.1248 0.5693)	0.1248	0.5693
2260	WT011_D07_CLUSTER_12550	gi|50929783|ref|XP_474419.1| OSJNBa0088H09.15 [Oryza sativa (japonica cultivar-group)] >gnl|BL_ORD_ID|1594604 gi|32488714|emb|CAE03457.1| OSJNBa0088H09.15 [Oryza sativa (japonica cultivar-group)] e_value:1.17067e-81	11670.m05830|LOC_Os04g58850|protein expressed protein e_value:1.55122e-83	0.658	(0.1873 0.2846)	0.1873	0.2846

PDF Complete
Your complimentary use period has ended. Thank you for using PDF Complete.
Click Here to upgrade to Unlimited Pages and Expanded Features

<table>
<tr><td>2261</td><td>SET5_F01_CLUSTER_34820</td><td>gi|50917149|ref|XP_468971.1| putative auxin-induced protein [Oryza sativa (japonica cultivar-group)] >gi|41469494|gb|AAS07279.1| putative auxin-induced protein [Oryza sativa (japonica cultivar-group)] e_value:2.57801e-69</td><td>11669.m04313|LOC_Os03g43410|protein putative auxin-induced protein e_value:4.18325e-71</td><td>0.315</td><td>(0.1542 0.4894)</td><td>0.1542</td><td>0.4894</td></tr>
<tr><td>2262</td><td>WT006_I11_CLUSTER_1978</td><td>gi|14522834|dbj|BAB61039.1| iron-deficiency induced gene [Hordeum vulgare] e_value:1.42649e-116</td><td>11669.m00617|LOC_Os03g06620|protein probable submergence induced, nickel-binding protein 2A - rice e_value:1.44723e-112</td><td>0.087</td><td>(0.0368 0.4220)</td><td>0.0368</td><td>0.422</td></tr>
<tr><td>2263</td><td>WT004_A19_CLUSTER_32883</td><td>gi|51978966|ref|XP_507360.1| PREDICTED P0492E07.108 gene product [Oryza sativa (japonica cultivar-group)] >gnl|BL_ORD_ID|1527537 gi|51963376|ref|XP_506281.1| PREDICTED P0492E07.108 gene product [Oryza sativa (japonica cultivar-group)] >gnl|BL_ORD_ID</td><td>11673.m02082|LOC_Os07g22600|protein spermidine synthase e_value:8.17646e-177</td><td>0.016</td><td>(0.0318 1.9749)</td><td>0.0318</td><td>1.9749</td></tr>
<tr><td>2264</td><td>SET2_I24_CLUSTER_7030</td><td>gi|19569591|gb|AAL92109.1| ferredoxin precursor [Triticum aestivum] e_value:1.54881e-78</td><td>11682.m03484|LOC_Os05g37140|protein ferredoxin [2Fe-2S], root - rice e_value:1.37277e-67</td><td>0.149</td><td>(0.0817 0.5469)</td><td>0.0817</td><td>0.5469</td></tr>
<tr><td>2265</td><td>WT005_K04_CLUSTER_3915</td><td>gi|34900796|ref|NP_911744.1| unknown protein [Oryza sativa (japonica cultivar-group)] >gnl|BL_ORD_ID|1523809 gi|51963348|ref|XP_506244.1| PREDICTED P0021G06.122-2 gene product [Oryza sativa (japonica cultivar-group)] >gnl|BL_ORD_ID|1523809 gi|234959</td><td>11669.m06331|LOC_Os03g62030|protein Transposable element protein, putative e_value:7.33992e-37</td><td>0.095</td><td>(0.1432 1.5045)</td><td>0.1432</td><td>1.5045</td></tr>
<tr><td>2266</td><td>WT012_A07_CLUSTER_26397</td><td>gi|31432783|gb|AAP54376.1| putative DNA ligase [Oryza sativa (japonica cultivar-group)] >gnl|BL_ORD_ID|2012262 gi|37535574|ref|NP_922089.1| putative DNA ligase [Oryza sativa (japonica cultivar-group)] >gnl|BL_ORD_ID|2012262 gi|16905197|gb|AAL31067.1</td><td>11676.m03086|LOC_Os10g34750|protein putative DNA ligase e_value:0</td><td>0.19</td><td>(0.1115 0.5861)</td><td>0.1115</td><td>0.5861</td></tr>
<tr><td>2267</td><td>WT006_M01_CLUSTER_914</td><td>gi|52076880|dbj|BAD45893.1| putative peroxidase [Oryza sativa (japonica cultivar-group)] e_value:5.57715e-83</td><td>11680.m04618|LOC_Os06g46790|protein Similar to peroxidase e_value:1.70144e-84</td><td>0.739</td><td>(0.1790 0.2422)</td><td>0.179</td><td>0.2422</td></tr>
<tr><td>2268</td><td>WT008_N04_CLUSTER_30889</td><td>gi|50941351|ref|XP_480203.1| putative RNA helicase [Oryza sativa (japonica cultivar-group)] >gnl|BL_ORD_ID|2403479 gi|37806159|dbj|BAC99664.1| putative RNA helicase [Oryza sativa (japonica cultivar-group)] >gnl|BL_ORD_ID|2403479</td><td>11674.m00565|LOC_Os08g06350|protein transposon protein, putative, CACTA, En/Spm sub-class e_value:0</td><td>0.158</td><td>(0.0990 0.6279)</td><td>0.099</td><td>0.6279</td></tr>
<tr><td>2269</td><td>WT012_D23_CLUSTER_14886</td><td>gi|50920555|ref|XP_470638.1| Putative NADPH-dependent oxidoreductase [Oryza sativa (japonica cultivar-group)] >gnl|BL_ORD_ID|1588727 gi|27357979|gb|AAO06971.1| Putative NADPH-dependent oxidoreductase [Oryza sativa (japonica cultivar-</td><td>11669.m01306|LOC_Os03g13390|protein oxidoreductase, aldo/keto reductase family, putative e_value:8.73203e-160</td><td>0.284</td><td>(0.0911 0.3211)</td><td>0.0911</td><td>0.3211</td></tr>
<tr><td>2270</td><td>SET1_E03_CLUSTER_32018</td><td>gi|50924139|ref|XP_472430.1| OJ991214_12.17 [Oryza sativa (japonica cultivar-group)] >gi|39545714|emb|CAD40930.3| OSJNBa0033G16.4 [Oryza sativa (japonica cultivar-group)] >gi|38344036|emb|CAE01528.2| OJ991214_12.17 [Oryza sativa (japonica</td><td>11670.m03270|LOC_Os04g33990|protein hypothetical protein e_value:6.45033e-104</td><td>0.501</td><td>(0.0997 0.1990)</td><td>0.0997</td><td>0.199</td></tr>
<tr><td>2271</td><td>WT013_G15_CLUSTER_6010</td><td>gi|50948335|ref|XP_483695.1| hypothetical protein [Oryza sativa (japonica cultivar-group)] >gnl|BL_ORD_ID|1611227 gi|42408954|dbj|BAD10210.1| hypothetical protein [Oryza sativa (japonica cultivar-group)] e_value:6.40458e-98</td><td>11674.m04423|LOC_Os08g43820|protein hypothetical protein e_value:3.82055e-100</td><td>0.302</td><td>(0.1228 0.4069)</td><td>0.1228</td><td>0.4069</td></tr>
<tr><td>2272</td><td>SET4_L01_CLUSTER_18499</td><td>gi|50948571|ref|XP_483813.1| zinc finger (C3HC4-type RING finger) protein family-like [Oryza sativa (japonica cultivar-group)] >gi|28071301|dbj|BAC55990.1| zinc finger (C3HC4-type RING finger) protein family-like [Oryza sativa (japonica cultivar-group)</td><td>11674.m04553|LOC_Os08g44950|protein Zinc finger, C3HC4 type (RING finger), putative e_value:1.19034e-46</td><td>0.803</td><td>(0.4382 0.5456)</td><td>0.4382</td><td>0.5456</td></tr>
<tr><td>2273</td><td>WT010_G12_CLUSTER_2391</td><td>gi|38344446|emb|CAE05652.2| OSJNBa0038O10.18 [Oryza sativa (japonica cultivar-group)] >gnl|BL_ORD_ID|1534547 gi|50926598|ref|XP_473246.1| OSJNBa0038O10.18 [Oryza sativa (japonica cultivar-group)] e_value:4.1481e-85</td><td>11670.m04311|LOC_Os04g44510|protein GRAM domain, putative e_value:6.18405e-87</td><td>0.388</td><td>(0.1290 0.3328)</td><td>0.129</td><td>0.3328</td></tr>
<tr><td>2274</td><td>SET3_L07_CLUSTER_33293</td><td>gi|21829|emb|CAA39486.1| peroxidase [Triticum aestivum] >gi|82613|pir||S13375 peroxidase (EC 1.11.1.7) precursor, pathogen-induced - wheat e_value:3.3495e-174</td><td>11673.m04763|LOC_Os07g48050|protein peroxidase POC1 e_value:6.44365e-139</td><td>0.238</td><td>(0.1379 0.5804)</td><td>0.1379</td><td>0.5804</td></tr>
<tr><td>2275</td><td>WT010_J13_CLUSTER_19608</td><td>gi|52076054|dbj|BAD46567.1| putative dehydroquinate synthase [Oryza sativa (japonica cultivar-group)] e_value:0</td><td>11681.m03275|LOC_Os09g36800|protein 3-dehydroquinate synthase, putative e_value:0</td><td>0.113</td><td>(0.0613 0.5415)</td><td>0.0613</td><td>0.5415</td></tr>
<tr><td>2276</td><td>WT005_O20_CLUSTER_33749</td><td>gi|62734422|gb|AAX96531.1| O-methyltransferase [Oryza sativa (japonica cultivar-group)] e_value:1.05515e-128</td><td>11687.m01884|LOC_Os11g20090|protein O-methyltransferase e_value:3.04156e-130</td><td>0.341</td><td>(0.2472 0.7258)</td><td>0.2472</td><td>0.7258</td></tr>
<tr><td>2277</td><td>WT009_K02_CLUSTER_27643</td><td>gi|732976|emb|CAA59487.1| peroxidase [Triticum aestivum] >gnl|BL_ORD_ID|2352995 gi|2117619|pir||S61408 peroxidase (EC 1.11.1.7) 4 precursor - wheat e_value:6.17287e-178</td><td>11673.m04755|LOC_Os07g47990|protein Peroxidase e_value:5.77861e-143</td><td>0.29</td><td>(0.1368 0.4726)</td><td>0.1368</td><td>0.4726</td></tr>
<tr><td>2278</td><td>WT009_A10_CLUSTER_5933</td><td>gi|50937877|ref|XP_478466.1| putative glucose-6-phosphate/phosphate-transloca tor precursor [Oryza sativa (japonica cultivar-group)] >gnl|BL_ORD_ID|1528135 gi|50937869|ref|XP_478462.1| putative glucose-6-phosphate/phosphate-transloca tor precursor [Ory</td><td>11673.m03261|LOC_Os07g33960|protein Tpt phosphate/phosphoenolpyruvate translocator, putative e_value:0</td><td>0.171</td><td>(0.0734 0.4281)</td><td>0.0734</td><td>0.4281</td></tr>
<tr><td>2279</td><td>SET5_J22_CLUSTER_8773</td><td>gi|50508264|dbj|BAD32075.1| putative AtRer1A [Oryza sativa (japonica cultivar-group)] >gi|50508077|dbj|BAD32077.1| putative AtRer1A [Oryza sativa (japonica cultivar-group)] e_value:1.86076e-98</td><td>11680.m04910|LOC_Os06g49460|protein rer1a protein (atrer1a) e_value:1.34571e-69</td><td>0.279</td><td>(0.2734 0.9812)</td><td>0.2734</td><td>0.9812</td></tr>
<tr><td>2280</td><td>SET5_K19_CLUSTER_13051</td><td>gi|34909798|ref|NP_916246.1| glutathione S-transferase II [Oryza sativa (japonica cultivar-group)] >gi|15289890|dbj|BAB63585.1| putative glutathione transferase I [Oryza sativa (japonica cultivar-group)] >gi|3746581|gb|AAC64007.1| glutathione S-tran</td><td>11667.m05514|LOC_Os01g55830|protein Glutathione S-transferase, N-terminal domain, putative e_value:7.86901e-73</td><td>0.362</td><td>(0.2882 0.7962)</td><td>0.2882</td><td>0.7962</td></tr>
<tr><td>2281</td><td>SET4_F02_CLUSTER_28673</td><td>gi|50911371|ref|XP_467093.1| putative uclacyanin 3 [Oryza sativa (japonica cultivar-group)] >gi|49387518|dbj|BAD24983.1| putative uclacyanin 3 [Oryza sativa (japonica cultivar-group)] e_value:1.53723e-71</td><td>11668.m04187|LOC_Os02g43660|protein uclacyanin 3-like protein e_value:3.09191e-73</td><td>0.5</td><td>(0.1798 0.3594)</td><td>0.1798</td><td>0.3594</td></tr>
<tr><td>2282</td><td>SET4_D20_CLUSTER_11120</td><td>gi|57900693|gb|AAW57818.1| hypothetical protein [Oryza sativa (japonica cultivar-group)] e_value:2.12121e-45</td><td>11682.m00864|LOC_Os05g08920|protein hypothetical protein e_value:2.06678e-47</td><td>0.325</td><td>(0.1333 0.4099)</td><td>0.1333</td><td>0.4099</td></tr>
<tr><td>2283</td><td>SET4_A01_CLUSTER_28964</td><td>gi|50251950|dbj|BAD27885.1| unknown protein [Oryza sativa (japonica cultivar-group)] e_value:3.56755e-19</td><td>11668.m01553|LOC_Os02g16500|protein expressed protein e_value:1.81547e-20</td><td>0.139</td><td>(0.1670 1.2054)</td><td>0.167</td><td>1.2054</td></tr>
<tr><td>2284</td><td>SET3_F16_CLUSTER_12332</td><td>gi|50917867|ref|XP_469330.1| hypothetical protein [Oryza sativa] >gi|13174247|gb|AAK14421.1| hypothetical protein [Oryza sativa] e_value:1.57185e-86</td><td>11669.m04810|LOC_Os03g48030|protein expressed protein e_value:5.60891e-94</td><td>0.223</td><td>(0.1582 0.7094)</td><td>0.1582</td><td>0.7094</td></tr>
<tr><td>2285</td><td>SET2_M13_CLUSTER_18617</td><td>gi|28557153|dbj|BAC57497.1| origin recognition complex2 [Oryza sativa (japonica cultivar-group)] >gi|66153722|gb|AAD29700.2| origin recognition complex subunit 2 [Oryza sativa (japonica cultivar-group)] e_value:0</td><td>11669.m00841|LOC_Os03g08640|protein Origin recognition complex subunit 2 e_value:0</td><td>0.105</td><td>(0.0586 0.5571)</td><td>0.0586</td><td>0.5571</td></tr>
<tr><td>2286</td><td>SET2_D07_CLUSTER_21671</td><td>gi|50931071|ref|XP_475063.1| unknown protein [Oryza sativa (japonica cultivar-group)] e_value:6.20203e-87</td><td>11682.m00042|LOC_Os05g01370|protein polygalacturonase-inhibiting protein e_value:6.57615e-92</td><td>0.583</td><td>(0.3912 0.6712)</td><td>0.3912</td><td>0.6712</td></tr>
<tr><td>2287</td><td>SET2_A12_CLUSTER_26676</td><td>gi|50725157|dbj|BAD33774.1| putative Cytochrome P450 [Oryza sativa (japonica cultivar-group)] e_value:0</td><td>11681.m03201|LOC_Os09g36080|protein cytochrome P-450LXXIA1 [similarity] - avocado e_value:0</td><td>0.469</td><td>(0.2141 0.4560)</td><td>0.2141</td><td>0.456</td></tr>
<tr><td>2288</td><td>SET1_I06_CLUSTER_23132</td><td>gi|50927931|ref|XP_473493.1| OSJNBa0084K11.15 [Oryza sativa (japonica cultivar-group)] >gi|38345851|emb|CAD41063.2| OSJNBa0084K11.15 [Oryza sativa (japonica cultivar-group)] e_value:3.60868e-108</td><td>11670.m04626|LOC_Os04g47370|protein expressed protein e_value:6.18841e-111</td><td>0.344</td><td>(0.0662 0.1926)</td><td>0.0662</td><td>0.1926</td></tr>
<tr><td>2289</td><td>SET1_H04_CLUSTER_25250</td><td>gi|21553945|gb|AAM63026.1| unknown [Arabidopsis thaliana] >gi|9662993|emb|CAC00737.1| putative protein [Arabidopsis thaliana] >gi|28950711|gb|AAO63279.1| At3g56740 [Arabidopsis thaliana] >gi|15228999|ref|NP_191233.1| ubiquitin-associated (UBA)/TS</td><td>11667.m05873|LOC_Os01g59160|protein UBA/TS-N domain, putative e_value:2.49604e-126</td><td>0.253</td><td>(0.1106 0.4370)</td><td>0.1106</td><td>0.437</td></tr>
<tr><td>2290</td><td>WT013_K17_CLUSTER_12948</td><td>gi|18146791|dbj|BAB82454.1| D-protein [Hordeum vulgare] e_value:0</td><td>11681.m00567|LOC_Os09g07460|protein Kelch motif, putative e_value:4.13291e-160</td><td>0.217</td><td>(0.0969 0.4462)</td><td>0.0969</td><td>0.4462</td></tr>
<tr><td>2291</td><td>WT013_E07_CLUSTER_30210</td><td>gi|50511358|gb|AAT77281.1| unknown protein [Oryza sativa (japonica cultivar-group)] e_value:1.59167e-60</td><td>11686.m04354|LOC_Os12g43580|protein hypothetical protein e_value:8.06256e-102</td><td>1.156</td><td>(0.4061 0.3512)</td><td>0.4061</td><td>0.3512</td></tr>
<tr><td>2292</td><td>WT012_P17_CLUSTER_4464</td><td>gi|31429842|gb|AAP51837.1| putative LeOPT1 - oligopeptide transporter [Oryza sativa (japonica cultivar-group)] >gnl|BL_ORD_ID|2007789 gi|37530496|ref|NP_919550.1| putative LeOPT1 - oligopeptide transporter [Oryza sativa (japonica cultivar-</td><td>11676.m00124|LOC_Os10g02210|protein Putative LeOPT1 - oligopeptide transporter e_value:0</td><td>0.25</td><td>(0.2161 0.8636)</td><td>0.2161</td><td>0.8636</td></tr>
<tr><td>2293</td><td>WT012_A15_CLUSTER_27299</td><td>gi|50909297|ref|XP_466137.1| putative immediate-early fungal elicitor protein CMPG1 [Oryza sativa (japonica cultivar-group)] >gnl|BL_ORD_ID|1574653 gi|46390762|dbj|BAD16270.1| putative immediate-early fungal elicitor protein CMPG1 [Oryza sativa (japoni</td><td>11668.m03256|LOC_Os02g34410|protein U-box domain, putative e_value:0</td><td>0.121</td><td>(0.0860 0.7083)</td><td>0.086</td><td>0.7083</td></tr>
<tr><td>2294</td><td>WT011_F15_CLUSTER_1392</td><td>gi|58532019|emb|CAD41558.3| OSJNBa0006A01.12 [Oryza sativa (japonica cultivar-group)] >gnl|BL_ORD_ID|1538780 gi|32489375|emb|CAE04147.1| OSJNBa0009P12.32 [Oryza sativa (japonica cultivar-group)] e_value:2.39734e-137</td><td>11670.m04951|LOC_Os04g50770|protein P-type R2R3 Myb protein e_value:6.06695e-139</td><td>0.384</td><td>(0.1600 0.4166)</td><td>0.16</td><td>0.4166</td></tr>
<tr><td>2295</td><td>WT011_A12_CLUSTER_2792</td><td>gi|39579184|gb|AAR28761.1| putative ribokinase [Triticum turgidum subsp. durum] e_value:0</td><td>11667.m00065|LOC_Os01g01620|protein kinase, pfkB family, putative e_value:3.65311e-179</td><td>0.208</td><td>(0.0903 0.4346)</td><td>0.0903</td><td>0.4346</td></tr>
<tr><td>2296</td><td>WT010_K21_CLUSTER_10778</td><td>gi|50920395|ref|XP_470558.1| Putative AP2 domain containing protein [Oryza sativa] >gnl|BL_ORD_ID|1534980 gi|15217291|gb|AAK92635.1| Putative AP2 domain containing protein [Oryza sativa] e_value:2.30452e-72</td><td>11669.m00823|LOC_Os03g08470|protein AP2 domain, putative e_value:5.63757e-74</td><td>0.449</td><td>(0.2954 0.6582)</td><td>0.2954</td><td>0.6582</td></tr>
<tr><td>2297</td><td>WT010_E20_CLUSTER_6192</td><td>gi|50912757|ref|XP_467786.1| ANT-like protein [Oryza sativa (japonica cultivar-group)] >gnl|BL_ORD_ID|1582899 gi|46390831|dbj|BAD16336.1| ANT-like protein [Oryza sativa (japonica cultivar-group)] >gnl|BL_ORD_ID|1582899 gi|46390932|dbj|BAD16446.1| AN</td><td>11668.m05008|LOC_Os02g51300|protein AP2 domain, putative e_value:1.2857e-123</td><td>0.368</td><td>(0.2288 0.6218)</td><td>0.2288</td><td>0.6218</td></tr>
<tr><td>2298</td><td>WT009_N02_CLUSTER_13295</td><td>gi|50911787|ref|XP_467301.1| putative WRKY DNA-binding protein [Oryza sativa (japonica cultivar-group)] >gnl|BL_ORD_ID|1580418 gi|41052960|dbj|BAD07870.1| putative WRKY DNA-binding protein [Oryza sativa (japonica cultivar-group)] e_value:3.67444e-141</td><td>11668.m04558|LOC_Os02g47060|protein WRKY DNA -binding domain, putative e_value:1.0006e-142</td><td>0.512</td><td>(0.2907 0.5675)</td><td>0.2907</td><td>0.5675</td></tr>
<tr><td>2299</td><td>WT009_B18_CLUSTER_21175</td><td>No hits found</td><td>11686.m00392|LOC_Os12g04650|protein Zinc finger, C3HC4 type (RING finger), putative e_value:3.59082e-32</td><td>0.457</td><td>(0.3983 0.8707)</td><td>0.3983</td><td>0.8707</td></tr>
<tr><td>2300</td><td>WT008_P14_CLUSTER_31428</td><td>gi|50252365|dbj|BAD28472.1| unknown protein [Oryza sativa (japonica cultivar-group)] e_value:0</td><td>11681.m01621|LOC_Os09g17760|protein expressed protein</td><td>0.214</td><td>(0.1887 0.8832)</td><td>0.1887</td><td>0.8832</td></tr>
<tr><td>2301</td><td>WT008_H19_CLUSTER_28581</td><td>gi|55701037|tpe|CAH69327.1| TPA: class III peroxidase 85 precursor [Oryza sativa (japonica cultivar-group)] >gnl|BL_ORD_ID|882772 gi|54290986|dbj|BAD61665.1| putative bacterial-induced peroxidase precursor [Oryza sativa (japonica cultivar-group)] e_val</td><td>11680.m03175|LOC_Os06g32960|protein bacterial-induced peroxidase precursor e_value:1.52153e-106</td><td>0.519</td><td>(0.2945 0.5674)</td><td>0.2945</td><td>0.5674</td></tr>
<tr><td>2302</td><td>WT008_B16_CLUSTER_28369</td><td>gi|62733160|gb|AAX95277.1| SCARECROW gene regulator, putative [Oryza sativa (japonica cultivar-group)] e_value:0</td><td>11687.m04504|LOC_Os11g47900|protein SCARECROW gene regulator, putative e_value:0</td><td>0.269</td><td>(0.2400 0.8922)</td><td>0.24</td><td>0.8922</td></tr>
<tr><td>2303</td><td>WT006_G01_CLUSTER_25637</td><td>gi|34895816|ref|NP_909252.1| unknown protein [Oryza sativa (japonica cultivar-group)] >gnl|BL_ORD_ID|1751642 gi|13872890|dbj|BAB43997.1| unknown protein [Oryza sativa (japonica cultivar-group)] e_value:2.8973e-85</td><td>11667.m00483|LOC_Os01g05540|protein expressed protein e_value:7.81372e-87</td><td>0.547</td><td>(0.4325 0.7905)</td><td>0.4325</td><td>0.7905</td></tr>
</table>

PDF Complete
Your complimentary use period has ended. Thank you for using PDF Complete.
Click Here to upgrade to Unlimited Pages and Expanded Features

<table>
<tr><th></th><th></th><th></th><th></th><th></th><th></th><th></th><th></th></tr>
<tr><td>2304</td><td>WT005_B02_CLUSTER_15905</td><td>gi|31415925|gb|AAP50946.1| putative NPH1 photoreceptor-interacting protein [Oryza sativa (japonica cultivar-group)] >gnl|BL_ORD_ID|2005430 gi|50919031|ref|XP_469912.1| putative NPH1 photoreceptor-interacting protein [Oryza sativa (japonica cultivar-gro</td><td>11669.m05624|LOC_Os03g55830|protein transposon protein, putative, mutator sub-class e_value:0</td><td>0.158</td><td>(0.0725 0.4591)</td><td>0.0725</td><td>0.4591</td></tr>
<tr><td>2305</td><td>WT003_K14_CLUSTER_13437</td><td>gi|50913285|ref|XP_468050.1| putative replication factor C 36kDa subunit [Oryza sativa (japonica cultivar-group)] >gnl|BL_ORD_ID|1583167 gi|46806135|dbj|BAD17365.1| putative replication factor C 36kDa subunit [Oryza sativa (japonica cultivar-</td><td>11668.m05252|LOC_Os02g53500|protein ATPase, AAA family, putative e_value:1.31512e-178</td><td>0.14</td><td>(0.0612 0.4358)</td><td>0.0612</td><td>0.4358</td></tr>
<tr><td>2306</td><td>WT002_E24_CLUSTER_2142</td><td>gi|50911795|ref|XP_467305.1| putative nitrate transporter NRT1 [Oryza sativa (japonica cultivar-group)] >gnl|BL_ORD_ID|1580422 gi|41052964|dbj|BAD07874.1| putative nitrate transporter NRT1 [Oryza sativa (japonica cultivar-group)] e_value:0</td><td>11668.m04561|LOC_Os02g47090|protein POT family, putative e_value:0</td><td>0.457</td><td>(0.1757 0.3844)</td><td>0.1757</td><td>0.3844</td></tr>
<tr><td>2307</td><td>WT002_B07_CLUSTER_27755</td><td>gi|50947279|ref|XP_483167.1| putative caffeoyl-CoA O-methyltransferase 1 [Oryza sativa (japonica cultivar-group)] >gnl|BL_ORD_ID|1609415 gi|51979689|ref|XP_507591.1| PREDICTED P0026F07.24 gene product [Oryza sativa (japonica cultivar-group)] >gnl|BL</td><td>11674.m03887|LOC_Os08g38900|protein O-methyltransferase e_value:5.54914e-113</td><td>0.334</td><td>(0.1228 0.3679)</td><td>0.1228</td><td>0.3679</td></tr>
<tr><td>2308</td><td>SET6_A22_CLUSTER_15914</td><td>gi|15594016|emb|CAC69856.1| inorganic phosphate transporter [Triticum aestivum/Thinopyrum intermedium alien addition line] e_value:0</td><td>11669.m00512|LOC_Os03g05640|protein phosphate:H+ symporter e_value:0</td><td>0.22</td><td>(0.1319 0.5988)</td><td>0.1319</td><td>0.5988</td></tr>
<tr><td>2309</td><td>WT010_O15_CLUSTER_2739</td><td>gi|37718872|gb|AAR01743.1| putative kinase interacting protein [Oryza sativa (japonica cultivar-group)] >gnl|BL_ORD_ID|2260642 gi|50917205|ref|XP_468999.1| putative kinase interacting protein [Oryza sativa (japonica cultivar-group)] e_value:0</td><td>11669.m04342|LOC_Os03g43680|protein putative kinase interacting protein e_value:0</td><td>0.219</td><td>(0.1615 0.7381)</td><td>0.1615</td><td>0.7381</td></tr>
<tr><td>2310</td><td>WT007_J17_CLUSTER_32388</td><td>gi|479687|pir||S35156 beta-glucanase - barley e_value:2.9472e-176</td><td>11667.m07154|LOC_Os01g71350|protein Glycosyl hydrolases family 17 e_value:9.47694e-124</td><td>0.535</td><td>(0.1987 0.3711)</td><td>0.1987</td><td>0.3711</td></tr>
<tr><td>2311</td><td>SET2_I08_CLUSTER_14013</td><td>gi|54610287|gb|AAV35210.1| NAR2.1 [Triticum aestivum] e_value:2.868e-107</td><td>11670.m03862|LOC_Os04g40410|protein expressed protein e_value:2.11908e-63</td><td>0.51</td><td>(0.2387 0.4682)</td><td>0.2387</td><td>0.4682</td></tr>
<tr><td>2312</td><td>WT002_M23_CLUSTER_8421</td><td>gi|52353425|gb|AAU43993.1| unknown protein [Oryza sativa (japonica cultivar-group)] e_value:1.80392e-161</td><td>11682.m04479|LOC_Os05g46520|protein polygalacturonase e_value:5.82299e-163</td><td>0.428</td><td>(0.2376 0.5545)</td><td>0.2376</td><td>0.5545</td></tr>
<tr><td>2313</td><td>SET4_P15_CLUSTER_30444</td><td>gi|50905999|ref|XP_464488.1| putative zinc finger and C2 domain protein [Oryza sativa (japonica cultivar-group)] >gi|49388351|dbj|BAD25461.1| putative zinc finger and C2 domain protein [Oryza sativa (japonica cultivar-group)] e_value:7.41141e-147</td><td>11668.m00980|LOC_Os02g10480|protein At4g21160 e_value:1.59951e-148</td><td>0.138</td><td>(0.1079 0.7793)</td><td>0.1079</td><td>0.7793</td></tr>
<tr><td>2314</td><td>WT012_N17_CLUSTER_12867</td><td>gi|8778846|gb|AAF79845.1| T6D22.6 [Arabidopsis thaliana] >gnl|BL_ORD_ID|928616 gi|15223059|ref|NP_172275.1| expressed protein [Arabidopsis thaliana] >gnl|BL_ORD_ID|928616 gi|25407016|pir||H86214 protein T6D22.6 [imported] - Arabidopsis thaliana e_va</td><td>11669.m01814|LOC_Os03g17950|protein protein T6D22.6 [imported] - Arabidopsis thaliana e_value:0</td><td>0.159</td><td>(0.0736 0.4619)</td><td>0.0736</td><td>0.4619</td></tr>
<tr><td>2315</td><td>WT011_P01_CLUSTER_13578</td><td>gi|50910645|ref|XP_466811.1| putative protein kinase (ADK1) [Oryza sativa (japonica cultivar-group)] >gnl|BL_ORD_ID|1579213 gi|47847774|dbj|BAD21551.1| putative protein kinase (ADK1) [Oryza sativa (japonica cultivar-group)] e_value:0</td><td>11668.m03938|LOC_Os02g40860|protein Protein kinase domain, putative e_value:0</td><td>0.128</td><td>(0.0532 0.4151)</td><td>0.0532</td><td>0.4151</td></tr>
<tr><td>2316</td><td>WT004_F19_CLUSTER_20606</td><td>gi|10177247|dbj|BAB10715.1| unnamed protein product [Arabidopsis thaliana] >gnl|BL_ORD_ID|1093799 gi|50253540|gb|AAT71972.1| At5g53850 [Arabidopsis thaliana] >gnl|BL_ORD_ID|1093799 gi|42573670|ref|NP_974931.1| haloacid dehalogenase-like hydrolase fa</td><td>11687.m02674|LOC_Os11g29370|protein 2,3-diketo-5-methylthio-1-phosphopentane phosphatase, putative e_value:0</td><td>0.165</td><td>(0.0811 0.4927)</td><td>0.0811</td><td>0.4927</td></tr>
<tr><td>2317</td><td>WT004_A17_CLUSTER_34995</td><td>gi|13447801|gb|AAK26758.1| plasma membrane integral protein ZmPIP2-1 [Zea mays] e_value:8.37206e-151</td><td>11673.m02503|LOC_Os07g26690|protein MIP family channel proteins, putative e_value:3.84492e-151</td><td>0.189</td><td>(0.0507 0.2678)</td><td>0.0507</td><td>0.2678</td></tr>
<tr><td>2318</td><td>WT003_B24_CLUSTER_14639</td><td>No hits found</td><td>11687.m01148|LOC_Os11g11980|protein expressed protein e_value:6.71241e-12</td><td>0.6</td><td>(0.3721 0.6206)</td><td>0.3721</td><td>0.6206</td></tr>
<tr><td>2319</td><td>SET5__C01_CLUSTER_32215</td><td>gi|34897132|ref|NP_909912.1| ferredoxin-NADP+ reductase [Oryza sativa] >gi|14718314|gb|AAK72892.1| ferredoxin-NADP+ reductase [Oryza sativa] >gi|729480|sp|P41345|FENR2_ORYSA Ferredoxin--NADP reductase, root isozyme, chloroplast precursor (FNR) >g</td><td>11669.m05772|LOC_Os03g57120|protein ferredoxin-NADP+ reductase e_value:0</td><td>0.096</td><td>(0.0698 0.7289)</td><td>0.0698</td><td>0.7289</td></tr>
<tr><td>2320</td><td>WT008_O14_CLUSTER_16851</td><td>gi|50920005|ref|XP_470363.1| unknown protein [Oryza sativa (japonica cultivar-group)] >gnl|BL_ORD_ID|1535384 gi|28376707|gb|AAO41137.1| unknown protein [Oryza sativa (japonica cultivar-group)] e_value:2.09295e-99</td><td>11669.m06355|LOC_Os03g62250|protein expressed protein e_value:4.27848e-101</td><td>0.16</td><td>(0.0627 0.3915)</td><td>0.0627</td><td>0.3915</td></tr>
<tr><td>2321</td><td>SET2_H11_CLUSTER_8475</td><td>gi|31415935|gb|AAP50956.1| putative allene oxide synthase [Oryza sativa (japonica cultivar-group)] >gi|50919025|ref|XP_469909.1| putative allene oxide synthase [Oryza sativa (japonica cultivar-group)] >gi|41351507|dbj|BAD08330.1| allene oxide syntha</td><td>11669.m05621|LOC_Os03g55800|protein putative allene oxide synthase e_value:0</td><td>0.309</td><td>(0.0887 0.2870)</td><td>0.0887</td><td>0.287</td></tr>
<tr><td>2322</td><td>SET2_G01_CLUSTER_19137</td><td>gi|62530909|gb|AAX85684.1| NAC protein [Oryza sativa (japonica cultivar-group)] e_value:6.25947e-123</td><td>11667.m05970|LOC_Os01g60020|protein No apical meristem (NAM) protein, putative e_value:1.55571e-124</td><td>0.326</td><td>(0.1374 0.4219)</td><td>0.1374</td><td>0.4219</td></tr>
<tr><td>2323</td><td>WT012_O13_CLUSTER_18147</td><td>gi|38347196|emb|CAD40521.2| OSJNBa0023J03.8 [Oryza sativa (japonica cultivar-group)] >gnl|BL_ORD_ID|1536038 gi|50922741|ref|XP_471731.1| OSJNBa0023J03.8 [Oryza sativa (japonica cultivar-group)] e_value:1.73393e-110</td><td>11670.m02249|LOC_Os04g24140|protein expressed protein e_value:1.22938e-110</td><td>0.219</td><td>(0.1108 0.5053)</td><td>0.1108</td><td>0.5053</td></tr>
<tr><td>2324</td><td>WT010_O24_CLUSTER_1317</td><td>gi|46394256|tpg|DAA05066.1| TPA: WRKY transcription factor 1 [Oryza sativa (japonica cultivar-group)] e_value:0</td><td>11667.m01430|LOC_Os01g14440|protein WRKY DNA -binding domain, putative e_value:0</td><td>0.219</td><td>(0.0949 0.4336)</td><td>0.0949</td><td>0.4336</td></tr>
<tr><td>2325</td><td>WT002_P11_CLUSTER_21972</td><td>gi|55770431|ref|XP_550206.1| unknown protein [Oryza sativa (japonica cultivar-group)] >gnl|BL_ORD_ID|1753763 gi|54290799|dbj|BAD61438.1| unknown protein [Oryza sativa (japonica cultivar-group)] >gnl|BL_ORD_ID|1753763 gi|9757666|dbj|BAB08185.1| ESTs</td><td>11667.m00578|LOC_Os01g06460|protein hypothetical protein e_value:1.13399e-106</td><td>0.306</td><td>(0.1084 0.3546)</td><td>0.1084</td><td>0.3546</td></tr>
<tr><td>2326</td><td>WT006_P07_CLUSTER_4223</td><td>gi|50725356|dbj|BAD34428.1| unknown protein [Oryza sativa (japonica cultivar-group)] e_value:2.69602e-70</td><td>11681.m03076|LOC_Os09g33950|protein expressed protein e_value:3.60667e-72</td><td>0.339</td><td>(0.0797 0.2351)</td><td>0.0797</td><td>0.2351</td></tr>
<tr><td>2327</td><td>WT006_L21_CLUSTER_22689</td><td>gi|50251897|dbj|BAD27835.1| putative mitochondrial import inner membrane translocase subunit Tim13 [Oryza sativa (japonica cultivar-group)] e_value:9.41048e-34</td><td>11668.m04428|LOC_Os02g45820|protein mitochondrial import inner membrane translocase subunit tim13 e_value:1.1256e-35</td><td>0.145</td><td>(0.0577 0.3981)</td><td>0.0577</td><td>0.3981</td></tr>
<tr><td>2328</td><td>WT006_I04_CLUSTER_7473</td><td>gi|34897150|ref|NP_909921.1| putative thioredoxin [Oryza sativa (japonica cultivar-group)] >gnl|BL_ORD_ID|1587440 gi|28209505|gb|AAO37523.1| putative thioredoxin [Oryza sativa (japonica cultivar-group)] e_value:2.84964e-49</td><td>11669.m05947|LOC_Os03g58630|protein putative thioredoxin e_value:2.79547e-51</td><td>0.362</td><td>(0.1812 0.5007)</td><td>0.1812</td><td>0.5007</td></tr>
<tr><td>2329</td><td>WT004_E22_CLUSTER_7445</td><td>gi|34904816|ref|NP_913755.1| unknown protein [Oryza sativa (japonica cultivar-group)] >gnl|BL_ORD_ID|1532286 gi|22093725|dbj|BAC07018.1| unknown protein [Oryza sativa (japonica cultivar-group)] e_value:2.0757e-87</td><td>11673.m04436|LOC_Os07g45060|protein hypothetical protein e_value:3.75683e-89</td><td>0.147</td><td>(0.1158 0.7902)</td><td>0.1158</td><td>0.7902</td></tr>
<tr><td>2330</td><td>SET6_K03_CLUSTER_17576</td><td>gi|50940451|ref|XP_479753.1| unknown protein [Oryza sativa (japonica cultivar-group)] >gi|42408360|dbj|BAD09512.1| unknown protein [Oryza sativa (japonica cultivar-group)] e_value:1.70031e-68</td><td>11674.m00115|LOC_Os08g02080|protein expressed protein e_value:2.47852e-70</td><td>0.327</td><td>(0.0916 0.2804)</td><td>0.0916</td><td>0.2804</td></tr>
<tr><td>2331</td><td>SET1_H11_CLUSTER_22036</td><td>gi|50921369|ref|XP_471045.1| OSJNBb0050O03.8 [Oryza sativa (japonica cultivar-group)] >gi|38344111|emb|CAE01718.2| OSJNBb0050O03.8 [Oryza sativa (japonica cultivar-group)] e_value:1.24418e-14</td><td>11670.m00171|LOC_Os04g02670|protein hypothetical protein e_value:2.85183e-16</td><td>0.629</td><td>(0.1417 0.2252)</td><td>0.1417</td><td>0.2252</td></tr>
<tr><td>2332</td><td>WT012_M20_CLUSTER_30855</td><td>gi|56784661|dbj|BAD81752.1| putative 50S ribosomal protein L17 [Oryza sativa (japonica cultivar-group)] >gnl|BL_ORD_ID|775547 gi|56784654|dbj|BAD81701.1| putative 50S ribosomal protein L17 [Oryza sativa (japonica cultivar-group)] e_value:1.25415e-80</td><td>11667.m06214|LOC_Os01g62210|protein ribosomal protein L17, putative e_value:1.7531e-82</td><td>0.078</td><td>(0.0458 0.5881)</td><td>0.0458</td><td>0.5881</td></tr>
<tr><td>2333</td><td>SET1_G17_CLUSTER_30855</td><td>gi|56784661|dbj|BAD81752.1| putative 50S ribosomal protein L17 [Oryza sativa (japonica cultivar-group)] >gi|56784654|dbj|BAD81701.1| putative 50S ribosomal protein L17 [Oryza sativa (japonica cultivar-group)] e_value:1.30428e-80</td><td>11667.m06214|LOC_Os01g62210|protein ribosomal protein L17, putative e_value:1.7531e-82</td><td>0.078</td><td>(0.0458 0.5881)</td><td>0.0458</td><td>0.5881</td></tr>
<tr><td>2334</td><td>SET3_F14_CLUSTER_31932</td><td>gi|50253311|dbj|BAD29580.1| putative prohibitin [Oryza sativa (japonica cultivar-group)] >gi|50251706|dbj|BAD27627.1| putative prohibitin [Oryza sativa (japonica cultivar-group)] e_value:4.75412e-132</td><td>11668.m03526|LOC_Os02g37000|protein prohibitin e_value:1.199e-133</td><td>0.17</td><td>(0.0829 0.4887)</td><td>0.0829</td><td>0.4887</td></tr>
<tr><td>2335</td><td>SET5_G20_CLUSTER_31518</td><td>gi|50252724|dbj|BAD28950.1| putative glutathione transferase [Oryza sativa (japonica cultivar-group)] e_value:1.61718e-170</td><td>11668.m02067|LOC_Os02g21460|protein expressed protein e_value:1.11015e-173</td><td>0.07</td><td>(0.0717 1.0197)</td><td>0.0717</td><td>1.0197</td></tr>
<tr><td>2336</td><td>WT003_M20_CLUSTER_33819</td><td>gi|520936|emb|CAA56553.1| gamma-TIP-like protein [Hordeum vulgare subsp. vulgare] >gnl|BL_ORD_ID|1709435 gi|629784|pir||S47037 tonoplast intrinsic protein gamma - barley e_value:1.44557e-138</td><td>11669.m00474|LOC_Os03g05290|protein tonoplast intrinsic protein, gamma (gamma tip) (aquaporin-tip) e_value:1.81386e-</td><td>0.305</td><td>(0.0686 0.2249)</td><td>0.0686</td><td>0.2249</td></tr>
<tr><td>2337</td><td>SET2_B13_CLUSTER_20287</td><td>gi|50899778|ref|XP_450677.1| DRE binding factor 2 [Oryza sativa (japonica cultivar-group)] >gi|49388786|dbj|BAD25981.1| DRE binding factor 2 [Oryza sativa (japonica cultivar-group)] >gi|49387678|dbj|BAD25924.1| DRE binding factor 2 [Oryza sativa (ja</td><td>11681.m01832|LOC_Os09g20350|protein AP2 domain, putative e_value:3.19812e-126</td><td>0.171</td><td>(0.0787 0.4598)</td><td>0.0787</td><td>0.4598</td></tr>
<tr><td>2338</td><td>SET4_M21_CLUSTER_20579</td><td>gi|53792452|dbj|BAD53360.1| unknown protein [Oryza sativa (japonica cultivar-group)] e_value:2.30203e-130</td><td>11667.m05705|LOC_Os01g57550|protein expressed protein e_value:4.36342e-158</td><td>0.231</td><td>(0.1418 0.6152)</td><td>0.1418</td><td>0.6152</td></tr>
<tr><td>2339</td><td>SET1_K05_CLUSTER_32300</td><td>gi|50931571|ref|XP_475313.1| putative soluble inorganic pyrophosphatase [Oryza sativa (japonica cultivar-group)] >gi|46981295|gb|AAT07613.1| putative soluble inorganic pyrophosphatase [Oryza sativa (japonica cultivar-group)] e_value:3.77404e-100</td><td>11682.m03440|LOC_Os05g36260|protein inorganic pyrophosphatase, putative e_value:7.88499e-102</td><td>0.113</td><td>(0.0897 0.7902)</td><td>0.0897</td><td>0.7902</td></tr>
<tr><td>2340</td><td>WT006_D11_CLUSTER_15224</td><td>gi|50936911|ref|XP_477983.1| gamma-glutamylcysteine synthetase [Oryza sativa (japonica cultivar-group)] >gnl|BL_ORD_ID|1526494 gi|34394064|dbj|BAC84166.1| gamma-glutamylcysteine synthetase [Oryza sativa (japonica cultivar-group)] e_value:0</td><td>11673.m02619|LOC_Os07g27790|protein glutamate--cysteine ligase, putative e_value:0</td><td>0.095</td><td>(0.0549 0.5783)</td><td>0.0549</td><td>0.5783</td></tr>
<tr><td>2341</td><td>WT007_O14_CLUSTER_31501</td><td>gi|50251990|dbj|BAD27924.1| bZIP protein-like [Oryza sativa (japonica cultivar-group)] >gnl|BL_ORD_ID|549177 gi|50252666|dbj|BAD28835.1| bZIP protein-like [Oryza sativa (japonica cultivar-group)] e_value:2.07306e-131</td><td>11668.m03169|LOC_Os02g33560|protein At5g04840 e_value:4.61855e-133</td><td>0.194</td><td>(0.1384 0.7134)</td><td>0.1384</td><td>0.7134</td></tr>
<tr><td>2342</td><td>WT005_M19_CLUSTER_28741</td><td>gi|62734192|gb|AAX96301.1| Protein-tyrosine phosphatase [Oryza sativa (japonica cultivar-group)] e_value:8.1567e-87</td><td>11686.m00711|LOC_Os12g07590|protein protein tyrosine phosphatase e_value:2.99211e-90</td><td>0.345</td><td>(0.3312 0.9588)</td><td>0.3312</td><td>0.9588</td></tr>
<tr><td>2343</td><td>WT008_G08_CLUSTER_18635</td><td>gi|50928791|ref|XP_473923.1| OSJNBa0058K23.21 [Oryza sativa (japonica cultivar-group)] >gnl|BL_ORD_ID|1592020 gi|38344339|emb|CAD41755.2| OSJNBa0058K23.21 [Oryza sativa (japonica cultivar-group)] e_value:9.62374e-166</td><td>11670.m05190|LOC_Os04g52960|protein RNA recognition motif. (a.k.a. RRM, RBD, or RNP domain), putative e_value:1.47827e-</td><td>0.381</td><td>(0.3214 0.8435)</td><td>0.3214</td><td>0.8435</td></tr>
<tr><td>2344</td><td>SET1_H07_CLUSTER_32959</td><td>gi|66271071|gb|AAY43812.1| beta1 proteasome-1D [Aegilops tauschii] e_value:2.64411e-134</td><td>11680.m00402|LOC_Os06g04800|protein Proteasome A-type and B-type, putative e_value:1.62842e-125</td><td>0.118</td><td>(0.0498 0.4208)</td><td>0.0498</td><td>0.4208</td></tr>
<tr><td>2345</td><td>WT006_D19_CLUSTER_33476</td><td>gi|51965088|ref|XP_507328.1| PREDICTED OJ1125_C01.28 gene product [Oryza sativa (japonica cultivar-group)] e_value:0</td><td>11681.m03453|LOC_Os09g38530|protein Endomembrane protein 70 e_value:0</td><td>0.086</td><td>(0.0586 0.6844)</td><td>0.0586</td><td>0.6844</td></tr>
<tr><td>2346</td><td>SET6_E14_CLUSTER_33577</td><td>gi|34912076|ref|NP_917385.1| putative nifU-like protein [Oryza sativa (japonica cultivar-group)] >gi|20521223|dbj|BAB91740.1| putative iron-sulfur cofactor synthesis protein iscU [Oryza sativa (japonica cultivar-group)] e_value:1.44723e-77</td><td>11667.m04572|LOC_Os01g47340|protein NifU-like N terminal domain, putative e_value:2.87164e-79</td><td>0.192</td><td>(0.0725 0.3781)</td><td>0.0725</td><td>0.3781</td></tr>
</table>

PDF Complete
Your complimentary use period has ended. Thank you for using PDF Complete.
Click Here to upgrade to Unlimited Pages and Expanded Features

<table>
<tr><td>2347</td><td>WT003_K12_CLUSTER_31625</td><td>gi|51039056|gb|AAT94293.1| endotransglucosylase/hydrolase XTH1 [Triticum aestivum] e_value:3.62728e-170</td><td>11674.m01350|LOC_Os08g13920|protein Glycosyl hydrolases family 16, putative e_value:6.19247e-130</td><td>0.427</td><td>(0.1503 0.3523)</td><td>0.1503</td><td>0.3523</td></tr>
<tr><td>2348</td><td>WT008_E06_CLUSTER_13927</td><td>gi|50915730|ref|XP_468329.1| ankyrin repeat protein-like [Oryza sativa (japonica cultivar-group)] >gnl|BL_ORD_ID|1583931 gi|47497095|dbj|BAD19146.1| ankyrin repeat protein-like [Oryza sativa (japonica cultivar-group)] e_value:0</td><td>11668.m05581|LOC_Os02g56530|protein expressed protein e_value:0</td><td>0.088</td><td>(0.0549 0.6209)</td><td>0.0549</td><td>0.6209</td></tr>
<tr><td>2349</td><td>SET1_K04_CLUSTER_26853</td><td>gi|50936921|ref|XP_477988.1| unknown protein [Oryza sativa (japonica cultivar-group)] >gi|34394069|dbj|BAC84171.1| unknown protein [Oryza sativa (japonica cultivar-group)] e_value:9.43199e-87</td><td>11673.m02621|LOC_Os07g27810|protein expressed protein e_value:1.71391e-88</td><td>0.199</td><td>(0.0876 0.4414)</td><td>0.0876</td><td>0.4414</td></tr>
<tr><td>2350</td><td>WT003_O04_CLUSTER_17632</td><td>gi|50946359|ref|XP_482707.1| fiber protein-like [Oryza sativa (japonica cultivar-group)] >gnl|BL_ORD_ID|1608547 gi|42407626|dbj|BAD08741.1| fiber protein-like [Oryza sativa (japonica cultivar-group)] e_value:0</td><td>11681.m02406|LOC_Os09g26390|protein ARID/BRIGHT DNA binding domain, putative e_value:0</td><td>0.106</td><td>(0.0600 0.5654)</td><td>0.06</td><td>0.5654</td></tr>
<tr><td>2351</td><td>WT003_E18_CLUSTER_34938</td><td>gi|50948007|ref|XP_483531.1| putative pyruvate dehydrogenase E1 beta subunit isoform 1 protein [Oryza sativa (japonica cultivar-group)] >gnl|BL_ORD_ID|1609858 gi|51965052|ref|XP_507310.1| PREDICTED OSJNBa0033D24.29 gene product [Oryza sativa (japonica</td><td>11681.m03028|LOC_Os09g33500|protein pyruvate dehydrogenase E1 beta subunit isoform 3 e_value:0</td><td>0.074</td><td>(0.0510 0.6862)</td><td>0.051</td><td>0.6862</td></tr>
<tr><td>2352</td><td>SET2_I01_CLUSTER_32562</td><td>gi|56784942|dbj|BAD82400.1| putative immunophilin [Oryza sativa (japonica cultivar-group)] e_value:1.95939e-68</td><td>11667.m06927|LOC_Os01g68710|protein peptidyl-prolyl cis-trans isomerase, FKBP-type, putative e_value:4.26889e-70</td><td>0.13</td><td>(0.0795 0.6118)</td><td>0.0795</td><td>0.6118</td></tr>
<tr><td>2353</td><td>WT012_O10_CLUSTER_3314</td><td>gi|56783662|dbj|BAD81074.1| putative epoxide hydrolase [Oryza sativa (japonica cultivar-group)] e_value:9.22426e-150</td><td>11667.m01510|LOC_Os01g15130|protein hydrolase, alpha/beta fold family e_value:2.62434e-151</td><td>0.284</td><td>(0.1399 0.4929)</td><td>0.1399</td><td>0.4929</td></tr>
<tr><td>2354</td><td>WT007_N10_CLUSTER_29418</td><td>gi|61614851|gb|AAQ21371.2| beta-galactosidase [Sandersonia aurantiaca] e_value:0</td><td>11669.m01491|LOC_Os03g15020|protein beta-galactosidase e_value:0</td><td>0.187</td><td>(0.0915 0.4897)</td><td>0.0915</td><td>0.4897</td></tr>
<tr><td>2355</td><td>SET2_M17_CLUSTER_34572</td><td>gi|50937609|ref|XP_478332.1| putative Dof zinc finger protein [Oryza sativa (japonica cultivar-group)] >gi|22093688|dbj|BAC06982.1| putative Dof zinc finger protein [Oryza sativa (japonica cultivar-group)] e_value:4.40233e-124</td><td>11673.m03107|LOC_Os07g32510|protein Dof domain, zinc finger, putative e_value:7.48218e-126</td><td>0.304</td><td>(0.2028 0.6667)</td><td>0.2028</td><td>0.6667</td></tr>
<tr><td>2356</td><td>WT004_M05_CLUSTER_32694</td><td>gi|50947895|ref|XP_483475.1| Moco containing protein(OsMCP) [Oryza sativa (japonica cultivar-group)] >gnl|BL_ORD_ID|1609801 gi|42407984|dbj|BAD09122.1| Moco containing protein(OsMCP) [Oryza sativa (japonica cultivar-group)] >gnl|BL_ORD_ID|1609801 gi</td><td>11674.m04211|LOC_Os08g41830|protein Oxidoreductase molybdopterin binding domain, putative e_value:0</td><td>0.089</td><td>(0.0419 0.4711)</td><td>0.0419</td><td>0.4711</td></tr>
<tr><td>2357</td><td>WT004_B08_CLUSTER_32444</td><td>gi|52076506|dbj|BAD45384.1| Dreg-2 like protein [Oryza sativa (japonica cultivar-group)] e_value:2.07474e-132</td><td>11680.m04476|LOC_Os06g45440|protein Dreg-2 like protein e_value:3.77363e-134</td><td>0.094</td><td>(0.0816 0.8696)</td><td>0.0816</td><td>0.8696</td></tr>
<tr><td>2358</td><td>WT003_P18_CLUSTER_471</td><td>gi|34898490|ref|NP_910591.1| putative isopentenyl pyrophosphate:dimethyllallyl pyrophosphate isomerase [Oryza sativa (japonica cultivar-group)] >gnl|BL_ORD_ID|2285279 gi|51963472|ref|XP_506401.1| PREDICTED OJ1612_A04.101 gene product [Oryza sativa (jap</td><td>11673.m03498|LOC_Os07g36190|protein isopentenyl-diphosphate delta-isomerase, type 1, putative e_value:3.10374e-</td><td>0.086</td><td>(0.0426 0.4949)</td><td>0.0426</td><td>0.4949</td></tr>
<tr><td>2359</td><td>SET4_H20_CLUSTER_18238</td><td>gi|21555545|gb|AAM63882.1| unknown [Arabidopsis thaliana] >gi|18424670|ref|NP_568965.1| expressed protein [Arabidopsis thaliana] e_value:1.35514e-19</td><td>11668.m03633|LOC_Os02g38030|protein hypothetical protein e_value:1.2526e-47</td><td>0.133</td><td>(0.0324 0.2430)</td><td>0.0324</td><td>0.243</td></tr>
<tr><td>2360</td><td>WT003_B03_CLUSTER_31908</td><td>gi|56797970|emb|CAI39214.1| BCL-2 binding anthanogene-1 [Hordeum vulgare subsp. vulgare] e_value:4.63992e-135</td><td>11667.m06128|LOC_Os01g61500|protein BAG domain, putative e_value:1.43085e-114</td><td>0.246</td><td>(0.1092 0.4434)</td><td>0.1092</td><td>0.4434</td></tr>
<tr><td>2361</td><td>WT002_H06_CLUSTER_14031</td><td>gi|50928931|ref|XP_473993.1| OSJNBa0089N06.15 [Oryza sativa (japonica cultivar-group)] >gnl|BL_ORD_ID|1592623 gi|39546245|emb|CAE04254.3| OSJNBa0089N06.15 [Oryza sativa (japonica cultivar-group)] e_value:5.68935e-47</td><td>11670.m05276|LOC_Os04g53740|protein thioredoxin h e_value:1.76637e-49</td><td>0.12</td><td>(0.1418 1.1810)</td><td>0.1418</td><td>1.181</td></tr>
<tr><td>2362</td><td>WT004_O17_CLUSTER_3553</td><td>gi|34904314|ref|NP_913504.1| unnamed protein product [Oryza sativa (japonica cultivar-group)] e_value:1.03057e-102</td><td>11667.m01279|LOC_Os01g13030|protein AUX/IAA family e_value:7.67115e-99</td><td>0.318</td><td>(0.1375 0.4319)</td><td>0.1375</td><td>0.4319</td></tr>
<tr><td>2363</td><td>WT002_E12_CLUSTER_19182</td><td>gi|53793163|dbj|BAD54370.1| putative RAD23 protein [Oryza sativa (japonica cultivar-group)] >gnl|BL_ORD_ID|260759 gi|53793157|dbj|BAD54365.1| putative RAD23 protein [Oryza sativa (japonica cultivar-group)] e_value:0</td><td>11680.m01522|LOC_Os06g15360|protein UV excision repair protein Rad23 e_value:0</td><td>0.189</td><td>(0.0748 0.3948)</td><td>0.0748</td><td>0.3948</td></tr>
<tr><td>2364</td><td>WT005_I13_CLUSTER_18790</td><td>gi|50932855|ref|XP_475955.1| unknown protein, contains hAT family dimerisation domain, PF05699 [Oryza sativa (japonica cultivar-group)] e_value:0</td><td>11669.m05285|LOC_Os03g52310|protein putative transposases e_value:0</td><td>0.124</td><td>(0.0873 0.7062)</td><td>0.0873</td><td>0.7062</td></tr>
<tr><td>2365</td><td>SET2__C15_CLUSTER_2009</td><td>gi|32352152|dbj|BAC78569.1| katanin [Oryza sativa (japonica cultivar-group)] >gi|57899262|dbj|BAD87507.1| katanin [Oryza sativa (japonica cultivar-group)] e_value:0</td><td>11667.m05449|LOC_Os01g55260|protein ATPase, AAA family, putative e_value:0</td><td>0.091</td><td>(0.0422 0.4621)</td><td>0.0422</td><td>0.4621</td></tr>
<tr><td>2366</td><td>WT005_K02_CLUSTER_33031</td><td>gi|50948919|ref|XP_493817.1| ESTs AU078175(C51476),AU068986(C51476) correspond to a region of the predicted gene.~similar to NADH dehydrogenase. (AC006532) [Oryza sativa (japonica cultivar-group)] >gnl|BL_ORD_ID|1732298 gi|6063548|dbj|BAA85408.1| ESTs</td><td>11669.m00311|LOC_Os03g03770|protein NADH-ubiquinone oxidoreductase B18 subunit (NDUFB7) e_value:1.79467e-51</td><td>0.446</td><td>(0.0652 0.1464)</td><td>0.0652</td><td>0.1464</td></tr>
<tr><td>2367</td><td>WT005_F06_CLUSTER_23135</td><td>gi|50920553|ref|XP_470637.1| Putative endosomal protein [Oryza sativa (japonica cultivar-group)] >gnl|BL_ORD_ID|1588750 gi|27357978|gb|AAO06970.1| Putative endosomal protein [Oryza sativa (japonica cultivar-group)] e_value:0</td><td>11676.m00140|LOC_Os10g02350|protein Endomembrane protein 70, putative e_value:0</td><td>0.079</td><td>(0.0735 0.9250)</td><td>0.0735</td><td>0.925</td></tr>
<tr><td>2368</td><td>WT006_F12_CLUSTER_18891</td><td>gi|52353624|gb|AAU44190.1| replication origin activator [Oryza sativa (japonica cultivar-group)] e_value:0</td><td>11682.m03786|LOC_Os05g39850|protein replication origin activator 3 e_value:0</td><td>0.088</td><td>(0.0361 0.4105)</td><td>0.0361</td><td>0.4105</td></tr>
<tr><td>2369</td><td>SET1_N24_CLUSTER_34484</td><td>gi|50912267|ref|XP_467541.1| hypothetical protein [Oryza sativa (japonica cultivar-group)] >gi|45735999|dbj|BAD13027.1| hypothetical protein [Oryza sativa (japonica cultivar-group)] e_value:1.30987e-70</td><td>11668.m04778|LOC_Os02g49150|protein expressed protein e_value:2.04755e-74</td><td>0.352</td><td>(0.2442 0.6946)</td><td>0.2442</td><td>0.6946</td></tr>
<tr><td>2370</td><td>WT003_O06_CLUSTER_33457</td><td>gi|14091665|gb|AAK53813.1| Ran binding protein-1 [Lycopersicon esculentum] e_value:1.13848e-69</td><td>11682.m02648|LOC_Os05g28290|protein Ran binding protein 1 homolog e_value:3.68293e-102</td><td>0.138</td><td>(0.0822 0.5934)</td><td>0.0822</td><td>0.5934</td></tr>
<tr><td>2371</td><td>WT002_J15_CLUSTER_31643</td><td>gi|6272285|emb|CAA66900.2| annexin p33 [Zea mays] e_value:7.08963e-156</td><td>11680.m01150|LOC_Os06g11800|protein annexin p33 e_value:2.62914e-156</td><td>0.229</td><td>(0.0854 0.3734)</td><td>0.0854</td><td>0.3734</td></tr>
<tr><td>2372</td><td>WT003__C07_CLUSTER_28270</td><td>gi|50935149|ref|XP_477102.1| putative dolichyl-di-phosphooligosaccharide- protein [Oryza sativa (japonica cultivar-group)] >gnl|BL_ORD_ID|1522754 gi|51963326|ref|XP_506221.1| PREDICTED OJ1136_A05.4 gene product [Oryza sativa (japonica cultivar-group)]</td><td>11673.m01030|LOC_Os07g10830|protein Dolichyl-diphosphooligosaccharide-protein glycosyltransferase 48kD subunit e_value:0</td><td>0.111</td><td>(0.0663 0.5989)</td><td>0.0663</td><td>0.5989</td></tr>
<tr><td>2373</td><td>WT012_B12_CLUSTER_34042</td><td>gi|57899379|dbj|BAD88026.1| RNA-binding like protein [Oryza sativa (japonica cultivar-group)] e_value:2.10929e-163</td><td>11667.m07198|LOC_Os01g71770|protein RNA recognition motif. (a.k.a. RRM, RBD, or RNP domain), putative e_value:5.13218e-</td><td>0.192</td><td>(0.1623 0.8456)</td><td>0.1623</td><td>0.8456</td></tr>
<tr><td>2374</td><td>WT006_H05_CLUSTER_4625</td><td>gi|50508559|dbj|BAD30858.1| thioredoxin family-like protein [Oryza sativa (japonica cultivar-group)] e_value:0</td><td>11673.m03268|LOC_Os07g34030|protein Thioredoxin, putative e_value:0</td><td>0.071</td><td>(0.0321 0.4520)</td><td>0.0321</td><td>0.452</td></tr>
<tr><td>2375</td><td>WT013_N08_CLUSTER_5854</td><td>gi|9188572|dbj|BAA99574.1| OsPol delta small subunit [Oryza sativa (japonica cultivar-group)] >gnl|BL_ORD_ID|675968 gi|13431489|sp|Q9LRE5|DPOD2_ORYSA DNA polymerase delta small subunit e_value:0</td><td>11669.m00299|LOC_Os03g03650|protein hypothetical protein e_value:0</td><td>0.17</td><td>(0.1033 0.6074)</td><td>0.1033</td><td>0.6074</td></tr>
<tr><td>2376</td><td>WT003_J04_CLUSTER_32406</td><td>gi|19104|emb|CAA44599.1| ribosomal protein L17-2 [Hordeum vulgare subsp. vulgare] >gnl|BL_ORD_ID|2347701 gi|464640|sp|P35267|RL172_HORVU 60S ribosomal protein L17-2 e_value:2.01397e-95</td><td>11681.m00668|LOC_Os09g08430|protein ribosomal protein L22 e_value:3.11856e-83</td><td>0.11</td><td>(0.1103 1.0032)</td><td>0.1103</td><td>1.0032</td></tr>
<tr><td>2377</td><td>WT008_N03_CLUSTER_33612</td><td>gi|50252360|dbj|BAD28467.1| BRI1-KD interacting protein 103 [Oryza sativa (japonica cultivar-group)] >gnl|BL_ORD_ID|381723 gi|50252882|dbj|BAD29113.1| BRI1-KD interacting protein 103 [Oryza sativa (japonica cultivar-group)] >gnl|BL_ORD_ID|381723 gi|</td><td>11681.m01618|LOC_Os09g17730|protein hypothetical protein e_value:0</td><td>0.311</td><td>(0.1564 0.5024)</td><td>0.1564</td><td>0.5024</td></tr>
<tr><td>2378</td><td>WT004_N20_CLUSTER_18176</td><td>gi|50909573|ref|XP_466275.1| putative glutathione s-transferase [Oryza sativa (japonica cultivar-group)] >gnl|BL_ORD_ID|1576393 gi|46390348|dbj|BAD15813.1| putative glutathione s-transferase [Oryza sativa (japonica cultivar-group)] >gnl|BL_ORD_ID|15</td><td>11687.m01310|LOC_Os11g14040|protein maleylacetoacetate isomerase e_value:5.27401e-73</td><td>0.159</td><td>(0.2368 1.4935)</td><td>0.2368</td><td>1.4935</td></tr>
<tr><td>2379</td><td>SET2_M23_CLUSTER_31941</td><td>gi|50941899|ref|XP_480477.1| putative microtubial binding protein [Oryza sativa (japonica cultivar-group)] >gi|51964738|ref|XP_507153.1| PREDICTED OSJNBa0056O06.5 gene product [Oryza sativa (japonica cultivar-group)] >gi|40253647|dbj|BAD05590.1| put</td><td>11674.m00865|LOC_Os08g09240|protein Microtubule associated protein 1A/1B, light chain 3 e_value:1.3413e-59</td><td>0.033</td><td>(0.0221 0.6744)</td><td>0.0221</td><td>0.6744</td></tr>
<tr><td>2380</td><td>WT005_J04_CLUSTER_31856</td><td>gi|50948547|ref|XP_483801.1| putative aminopeptidase N [Oryza sativa (japonica cultivar-group)] >gnl|BL_ORD_ID|1612303 gi|42408435|dbj|BAD09617.1| putative aminopeptidase N [Oryza sativa (japonica cultivar-group)] e_value:0</td><td>11674.m04542|LOC_Os08g44860|protein Peptidase family M1, putative e_value:0</td><td>0.112</td><td>(0.0649 0.5815)</td><td>0.0649</td><td>0.5815</td></tr>
<tr><td>2381</td><td>WT008_F11_CLUSTER_9880</td><td>gi|50912285|ref|XP_467550.1| zinc-finger protein [Oryza sativa (japonica cultivar-group)] >gnl|BL_ORD_ID|1582681 gi|45736008|dbj|BAD13036.1| zinc-finger protein [Oryza sativa (japonica cultivar-group)] >gnl|BL_ORD_ID|1582681 gi|7489605|pir||JE0113</td><td>11668.m04790|LOC_Os02g49230|protein zinc-finger protein S3574 [imported] - rice e_value:5.69457e-128</td><td>0.212</td><td>(0.1929 0.9111)</td><td>0.1929</td><td>0.9111</td></tr>
<tr><td>2382</td><td>SET2_P13_CLUSTER_14771</td><td>gi|51090972|dbj|BAD35574.1| smr domain-containing protein -like [Oryza sativa (japonica cultivar-group)] >gi|51091830|dbj|BAD36644.1| smr domain-containing protein -like [Oryza sativa (japonica cultivar-group)] e_value:0</td><td>11680.m00953|LOC_Os06g09890|protein Smr domain, putative e_value:0</td><td>0.152</td><td>(0.0960 0.6303)</td><td>0.096</td><td>0.6303</td></tr>
<tr><td>2383</td><td>WT006_G07_CLUSTER_31139</td><td>gi|50940601|ref|XP_479828.1| putative MA3 domain-containing protein [Oryza sativa (japonica cultivar-group)] >gnl|BL_ORD_ID|1601365 gi|42407587|dbj|BAD10818.1| putative MA3 domain-containing protein [Oryza sativa (japonica cultivar-group)] e_value:0</td><td>11674.m00178|LOC_Os08g02690|protein Transposable element protein, putative e_value:0</td><td>0.158</td><td>(0.0675 0.4280)</td><td>0.0675</td><td>0.428</td></tr>
<tr><td>2384</td><td>WT004_L01_CLUSTER_31503</td><td>gi|34902832|ref|NP_912763.1| unnamed protein product [Oryza sativa (japonica cultivar-group)] >gnl|BL_ORD_ID|1752279 gi|6498466|dbj|BAA87855.1| putative importin alpha 2 [Oryza sativa (japonica cultivar-group)] >gnl|BL_ORD_ID|1752279 gi|62900360|sp|</td><td>11667.m01489|LOC_Os01g14950|protein Armadillo/beta-catenin-like repeat, putative e_value:0</td><td>0.117</td><td>(0.0535 0.4565)</td><td>0.0535</td><td>0.4565</td></tr>
<tr><td>2385</td><td>WT002_G15_CLUSTER_33628</td><td>gi|52076692|dbj|BAD45605.1| putative t-complex protein 1 theta chain [Oryza sativa (japonica cultivar-group)] >gnl|BL_ORD_ID|379066 gi|52077028|dbj|BAD46061.1| putative t-complex protein 1 theta chain [Oryza sativa (japonica cultivar-group)] e_value:0</td><td>11680.m04676|LOC_Os06g47320|protein AT3g11830/F26K24_12 e_value:0</td><td>0.054</td><td>(0.0389 0.7245)</td><td>0.0389</td><td>0.7245</td></tr>
<tr><td>2386</td><td>WT006_J08_CLUSTER_33130</td><td>gi|58220767|gb|AAW68015.1| putative polynucleotide adenylyltransferase [Oryza sativa (indica cultivar-group)] e_value:0</td><td>11680.m03525|LOC_Os06g36360|protein At4g32850/T16I18_60 e_value:0</td><td>0.144</td><td>(0.1005 0.6987)</td><td>0.1005</td><td>0.6987</td></tr>
<tr><td>2387</td><td>WT004_K08_CLUSTER_34272</td><td>gi|38344577|emb|CAE05535.2| OSJNBa0053B21.9 [Oryza sativa (japonica cultivar-group)] >gnl|BL_ORD_ID|1534753 gi|50923857|ref|XP_472289.1| OSJNBa0053B21.9 [Oryza sativa (japonica cultivar-group)] e_value:0</td><td>11668.m02912|LOC_Os02g30620|protein DnaJ domain, putative e_value:0</td><td>0.128</td><td>(0.1331 1.0401)</td><td>0.1331</td><td>1.0401</td></tr>
<tr><td>2388</td><td>WT004_J03_CLUSTER_31484</td><td>gi|34898202|ref|NP_910447.1| 26S proteasome regulatory subunit 4 homolog [Oryza sativa (japonica cultivar-group)] >gnl|BL_ORD_ID|1534727 gi|51979076|ref|XP_507415.1| PREDICTED P0034A04.112 gene product [Oryza sativa (japonica cultivar-group)] >gnl|B</td><td>11673.m04890|LOC_Os07g49150|protein 26S proteasome subunit P45 family e_value:0</td><td>0.031</td><td>(0.0139 0.4510)</td><td>0.0139</td><td>0.451</td></tr>
<tr><td>2389</td><td>WT003_B13_CLUSTER_34905</td><td>gi|30090032|gb|AAO86520.1| phytochelatin synthetase [Triticum monococcum] e_value:0</td><td>11682.m02994|LOC_Os05g32110|protein phytochelatin synthetase-like protein 2 e_value:0</td><td>0.146</td><td>(0.0807 0.5520)</td><td>0.0807</td><td>0.552</td></tr>
</table>

PDF Complete
Your complimentary use period has ended. Thank you for using PDF Complete.
Click Here to upgrade to Unlimited Pages and Expanded Features

<table>
<tr><td></td><td></td><td></td><td></td><td></td><td></td><td></td><td></td></tr>
<tr><td>2390</td><td>WT005_F14_CLUSTER_33059</td><td>gi|11527563|dbj|BAB18682.1| vacuolar proton-ATPase [Hordeum vulgare subsp. vulgare] e_value:0</td><td>11680.m04440|LOC_Os06g45120|protein V-type ATPase, A subunit e_value:0</td><td>0.066</td><td>(0.0361 0.5471)</td><td>0.0361</td><td>0.5471</td></tr>
<tr><td>2391</td><td>SET3_H13_CLUSTER_27574</td><td>gi|6996144|emb|CAB75506.1| VIP2 protein [Avena fatua] e_value:0</td><td>11682.m00576|LOC_Os05g06270|protein VIP2 protein, putative e_value:0</td><td>0.108</td><td>(0.0623 0.5745)</td><td>0.0623</td><td>0.5745</td></tr>
<tr><td>2392</td><td>WT004_A21_CLUSTER_31600</td><td>gi|29367429|gb|AAO72587.1| porin-like protein [Oryza sativa (japonica cultivar-group)] e_value:4.61059e-129</td><td>11669.m00998|LOC_Os03g10510|protein Eukaryotic porin e_value:9.23337e-131</td><td>0.243</td><td>(0.0998 0.4111)</td><td>0.0998</td><td>0.4111</td></tr>
<tr><td>2393</td><td>WT003_D20_CLUSTER_21604</td><td>gi|50919263|ref|XP_470028.1| putative ubiquinol-cytochrome c reductase [Oryza sativa (japonica cultivar-group)] >gnl|BL_ORD_ID|1588216 gi|30103022|gb|AAP21435.1| putative ubiquinol-cytochrome c reductase [Oryza sativa (japonica cultivar-group)] e_value</td><td>11673.m00993|LOC_Os07g10500|protein ubiquinol-cytochrome c reductase complex 14 kda protein (ec 1.10.2.2)(cr14) e_value:3.70686e-54</td><td>0.241</td><td>(0.1403 0.5834)</td><td>0.1403</td><td>0.5834</td></tr>
<tr><td>2394</td><td>WT008_I12_CLUSTER_33265</td><td>gi|34911052|ref|NP_916873.1| ubiquitin-conjugating enzyme E2 [Oryza sativa (japonica cultivar-group)] >gnl|BL_ORD_ID|1545339 gi|21644620|dbj|BAC01179.1| putative ubiquitin-conjugating enzyme E2 [Oryza sativa (japonica cultivar-group)] >gnl|BL_ORD_ID</td><td>11667.m04681|LOC_Os01g48280|protein Ubiquitin-conjugating enzyme e_value:1.98981e-61</td><td>0.029</td><td>(0.0371 1.2986)</td><td>0.0371</td><td>1.2986</td></tr>
<tr><td>2395</td><td>WT003_G10_CLUSTER_33168</td><td>gi|50937547|ref|XP_478301.1| unknown protein [Oryza sativa (japonica cultivar-group)] >gnl|BL_ORD_ID|1527567 gi|51978976|ref|XP_507365.1| PREDICTED OSJNBb0062D12.107 gene product [Oryza sativa (japonica cultivar-group)] >gnl|BL_ORD_ID|1527567 gi|519</td><td>11673.m03078|LOC_Os07g32230|protein expressed protein e_value:4.59182e-163</td><td>0.054</td><td>(0.0355 0.6511)</td><td>0.0355</td><td>0.6511</td></tr>
<tr><td>2396</td><td>SET6_I13_CLUSTER_33168</td><td>gi|50937547|ref|XP_478301.1| unknown protein [Oryza sativa (japonica cultivar-group)] >gi|51978976|ref|XP_507365.1| PREDICTED OSJNBb0062D12.107 gene product [Oryza sativa (japonica cultivar-group)] >gi|51963434|ref|XP_506358.1| PREDICTED OSJNBb0062D</td><td>11673.m03078|LOC_Os07g32230|protein expressed protein e_value:1.81684e-162</td><td>0.066</td><td>(0.0421 0.6364)</td><td>0.0421</td><td>0.6364</td></tr>
<tr><td>2397</td><td>SET2_I09_CLUSTER_32054</td><td>gi|50901224|ref|XP_463045.1| expressed protein [Oryza sativa (japonica cultivar-group)] >gi|41469325|gb|AAS07181.1| expressed protein [Oryza sativa (japonica cultivar-group)] e_value:2.68732e-27</td><td>11669.m03802|LOC_Os03g38520|protein hypothetical protein e_value:1.99867e-29</td><td>0.217</td><td>(0.1790 0.8253)</td><td>0.179</td><td>0.8253</td></tr>
<tr><td>2398</td><td>SET5_J02_CLUSTER_33027</td><td>gi|6437555|gb|AAF08582.1| unknown protein [Arabidopsis thaliana] >gi|21592632|gb|AAM64581.1| NADH:ubiquinone oxidoreductase-like protein [Arabidopsis thaliana] >gi|22136294|gb|AAM91225.1| unknown protein [Arabidopsis thaliana] >gi|20260662|gb|AAM</td><td>11669.m01865|LOC_Os03g18420|protein hypothetical protein e_value:3.38588e-58</td><td>0.086</td><td>(0.0357 0.4148)</td><td>0.0357</td><td>0.4148</td></tr>
<tr><td>2399</td><td>WT002_O11_CLUSTER_12335</td><td>gi|51468996|emb|CAH17998.1| RAB1-like [Poa pratensis] e_value:3.3561e-109</td><td>11667.m00794|LOC_Os01g08450|protein GTP-binding protein, putative e_value:3.03362e-105</td><td>0.031</td><td>(0.0523 1.6818)</td><td>0.0523</td><td>1.6818</td></tr>
<tr><td>2400</td><td>WT006_D16_CLUSTER_33929</td><td>gi|50932987|ref|XP_476021.1| putative translation initiation factor IF-2 [Oryza sativa (japonica cultivar-group)] >gnl|BL_ORD_ID|1600187 gi|48475233|gb|AAT44302.1| putative translation initiation factor IF-2 [Oryza sativa (japonica cultivar-group)] e_v</td><td>11682.m04971|LOC_Os05g51500|protein translation initiation factor e_value:0</td><td>0.172</td><td>(0.1065 0.6176)</td><td>0.1065</td><td>0.6176</td></tr>
<tr><td>2401</td><td>WT005_D17_CLUSTER_28397</td><td>gi|50906535|ref|XP_464756.1| early nodulin [Oryza sativa (japonica cultivar-group)] >gnl|BL_ORD_ID|1566960 gi|51963958|ref|XP_506762.1| PREDICTED P0495C02.34 gene product [Oryza sativa (japonica cultivar-group)] >gnl|BL_ORD_ID|1566960</td><td>11668.m01234|LOC_Os02g13380|protein Early nodulin 93 ENOD93 protein e_value:1.06009e-38</td><td>0.243</td><td>(0.0925 0.3802)</td><td>0.0925</td><td>0.3802</td></tr>
<tr><td>2402</td><td>WT006_N21_CLUSTER_33851</td><td>gi|53791783|dbj|BAD53577.1| putative SSR alpha subunit [Oryza sativa (japonica cultivar-group)] e_value:9.22263e-124</td><td>11680.m04995|LOC_Os06g50160|protein expressed protein e_value:2.2927e-125</td><td>0.163</td><td>(0.0693 0.4249)</td><td>0.0693</td><td>0.4249</td></tr>
<tr><td>2403</td><td>WT009_K12_CLUSTER_32866</td><td>gi|53791886|dbj|BAD54008.1| unknown protein [Oryza sativa (japonica cultivar-group)] e_value:0</td><td>11680.m04824|LOC_Os06g48700|protein expressed protein</td><td>0.128</td><td>(0.0662 0.5169)</td><td>0.0662</td><td>0.5169</td></tr>
<tr><td>2404</td><td>WT002_K12_CLUSTER_33067</td><td>gi|55297254|dbj|BAD69039.1| putative nucleosome assembly protein 1 [Oryza sativa (japonica cultivar-group)] >gnl|BL_ORD_ID|269782 gi|55297061|dbj|BAD68630.1| putative nucleosome assembly protein 1 [Oryza sativa (japonica cultivar-group)] e_value:0</td><td>11680.m00499|LOC_Os06g05660|protein nucleosome assembly protein 1-like protein 1 e_value:0</td><td>0.132</td><td>(0.0718 0.5433)</td><td>0.0718</td><td>0.5433</td></tr>
<tr><td>2405</td><td>WT007_B17_CLUSTER_25006</td><td>gi|50900110|ref|XP_450843.1| putative zinc-finger motif [Oryza sativa (japonica cultivar-group)] >gnl|BL_ORD_ID|1564153 gi|51963770|ref|XP_506665.1| PREDICTED OSJNBa0048A13.16 gene product [Oryza sativa (japonica cultivar-group)]</td><td>11681.m01978|LOC_Os09g21770|protein BAH domain, putative e_value:2.88202e-120</td><td>0.125</td><td>(0.0482 0.3850)</td><td>0.0482</td><td>0.385</td></tr>
<tr><td>2406</td><td>WT005_G03_CLUSTER_34062</td><td>gi|3122234|sp|O24473|IF2B_WHEAT Eukaryotic translation initiation factor 2 beta subunit (eIF-2-beta) (P38) >gnl|BL_ORD_ID|642367 gi|2306768|gb|AAB65774.1| eIF-2 beta subunit [Triticum aestivum] e_value:4.22961e-151</td><td>11669.m02210|LOC_Os03g21550|protein eukaryotic translation initiation factor 2 beta subunit (eif-2-beta)(p38) e_value:2.26135e-142</td><td>0.098</td><td>(0.0413 0.4193)</td><td>0.0413</td><td>0.4193</td></tr>
<tr><td>2407</td><td>SET4_L06_CLUSTER_10442</td><td>gi|50948469|ref|XP_483762.1| putative F1F0-ATPase inhibitor protein [Oryza sativa (japonica cultivar-group)] >gi|51965100|ref|XP_507334.1| PREDICTED P0562A06.11 gene product [Oryza sativa (japonica cultivar-group)]</td><td>11674.m04495|LOC_Os08g44460|protein expressed protein e_value:1.25055e-26</td><td>0.278</td><td>(0.1675 0.6035)</td><td>0.1675</td><td>0.6035</td></tr>
<tr><td>2408</td><td>WT006_E19_CLUSTER_34867</td><td>gi|54290851|dbj|BAD61512.1| putative auxin-induced protein [Oryza sativa (japonica cultivar-group)] e_value:6.19279e-169</td><td>11667.m04172|LOC_Os01g43090|protein expressed protein e_value:1.64429e-170</td><td>0.26</td><td>(0.1101 0.4239)</td><td>0.1101</td><td>0.4239</td></tr>
<tr><td>2409</td><td>WT005__C03_CLUSTER_34789</td><td>gi|38344582|emb|CAE05540.2| OSJNBa0053B21.14 [Oryza sativa (japonica cultivar-group)] >gnl|BL_ORD_ID|1534758 gi|50923867|ref|XP_472294.1| OSJNBa0053B21.14 [Oryza sativa (japonica cultivar-group)] e_value:1.6139e-154</td><td>11670.m03055|LOC_Os04g32030|protein heavy metal-associated domain, putative e_value:4.03503e-156</td><td>0.133</td><td>(0.0808 0.6072)</td><td>0.0808</td><td>0.6072</td></tr>
<tr><td>2410</td><td>WT005_E04_CLUSTER_31527</td><td>gi|34905462|ref|NP_914078.1| putative ATP citrate lyase [Oryza sativa (japonica cultivar-group)] >gnl|BL_ORD_ID|1511030 gi|15623805|dbj|BAB67865.1| putative ATP citrate lyase a-subunit [Oryza sativa (japonica cultivar-group)] >gnl|BL_ORD_ID|1511030</td><td>11667.m01918|LOC_Os01g19450|protein CoA-ligase, putative e_value:0</td><td>0.137</td><td>(0.0543 0.3975)</td><td>0.0543</td><td>0.3975</td></tr>
<tr><td>2411</td><td>WT003_E08_CLUSTER_33866</td><td>gi|31431903|gb|AAP53615.1| putative Cell elongation protein DIMINUTO (Cell elongation protein Dwarf1) [Oryza sativa (japonica cultivar-group)] >gnl|BL_ORD_ID|2011060 gi|37534052|ref|NP_921328.1| putative Cell elongation protein DIMINUTO (Cell elongatio</td><td>11676.m02195|LOC_Os10g25780|protein dwarf protein, OSDIM - rice e_value:0</td><td>0.028</td><td>(0.0525 1.8977)</td><td>0.0525</td><td>1.8977</td></tr>
<tr><td>2412</td><td>WT005_P09_CLUSTER_33789</td><td>gi|50252368|dbj|BAD28475.1| unknown protein [Oryza sativa (japonica cultivar-group)] e_value:2.67404e-101</td><td>11681.m01622|LOC_Os09g17770|protein protein F9L1.16 [imported] - Arabidopsis thaliana e_value:6.02034e-103</td><td>0.416</td><td>(0.1829 0.4397)</td><td>0.1829</td><td>0.4397</td></tr>
<tr><td>2413</td><td>WT006_M10_CLUSTER_32626</td><td>gi|58531987|emb|CAE03629.3| OSJNBb0003B01.20 [Oryza sativa (japonica cultivar-group)] e_value:7.11458e-66</td><td>11670.m05384|LOC_Os04g54750|protein hypothetical protein e_value:2.1458e-67</td><td>0.675</td><td>(0.5153 0.7636)</td><td>0.5153</td><td>0.7636</td></tr>
<tr><td>2414</td><td>WT004_M01_CLUSTER_32689</td><td>gi|45558475|gb|AAS68103.1| minichromosomal maintenance factor [Triticum aestivum] e_value:0</td><td>11687.m02675|LOC_Os11g29380|protein minichromosomal maintenance factor e_value:0</td><td>0.049</td><td>(0.0246 0.5067)</td><td>0.0246</td><td>0.5067</td></tr>
<tr><td>2415</td><td>WT003__C01_CLUSTER_19179</td><td>gi|50932983|ref|XP_476019.1| putative DNA damage binding protein 1 [Oryza sativa (japonica cultivar-group)] >gnl|BL_ORD_ID|1600172 gi|48475231|gb|AAT44300.1| putative DNA damage binding protein 1 [Oryza sativa (japonica cultivar-group)] e_value:0</td><td>11682.m04969|LOC_Os05g51480|protein CPSF A subunit region, putative e_value:0</td><td>0.052</td><td>(0.0314 0.6004)</td><td>0.0314</td><td>0.6004</td></tr>
<tr><td>2416</td><td>WT005_D09_CLUSTER_34968</td><td>gi|50940087|ref|XP_479571.1| r40g2 protein [Oryza sativa (japonica cultivar-group)] >gnl|BL_ORD_ID|1534651 gi|34394517|dbj|BAC83804.1| r40g2 protein [Oryza sativa (japonica cultivar-group)] e_value:1.78143e-177</td><td>11673.m04813|LOC_Os07g48490|protein QXW lectin repeat, putative e_value:4.33504e-179</td><td>0.461</td><td>(0.1038 0.2250)</td><td>0.1038</td><td>0.225</td></tr>
<tr><td>2417</td><td>WT010_K17_CLUSTER_33672</td><td>gi|50907245|ref|XP_465111.1| putative NEC1 [Oryza sativa (japonica cultivar-group)] >gnl|BL_ORD_ID|1568233 gi|51979203|ref|XP_507468.1| PREDICTED OSJNBa0010K08.10 gene product [Oryza sativa (japonica cultivar-group)] >gnl|BL_ORD_ID|1568233 gi|519792</td><td>11668.m01896|LOC_Os02g19820|protein MtN3/saliva family, putative e_value:1.43939e-119</td><td>0.402</td><td>(0.0940 0.2339)</td><td>0.094</td><td>0.2339</td></tr>
<tr><td>2418</td><td>SET5_O03_CLUSTER_9203</td><td>gi|50919949|ref|XP_470335.1| putative ferredoxin [Oryza sativa (japonica cultivar-group)] >gi|40714664|gb|AAR88570.1| putative ferredoxin [Oryza sativa (japonica cultivar-group)] e_value:1.05619e-62</td><td>11669.m06319|LOC_Os03g61960|protein putative ferredoxin e_value:1.44643e-64</td><td>0.082</td><td>(0.1083 1.3189)</td><td>0.1083</td><td>1.3189</td></tr>
<tr><td>2419</td><td>SET5_N03_CLUSTER_9203</td><td>gi|50919949|ref|XP_470335.1| putative ferredoxin [Oryza sativa (japonica cultivar-group)] >gi|40714664|gb|AAR88570.1| putative ferredoxin [Oryza sativa (japonica cultivar-group)] e_value:1.05619e-62</td><td>11669.m06319|LOC_Os03g61960|protein putative ferredoxin e_value:1.44643e-64</td><td>0.082</td><td>(0.1083 1.3189)</td><td>0.1083</td><td>1.3189</td></tr>
<tr><td>2420</td><td>WT003_M15_CLUSTER_33309</td><td>gi|4160402|emb|CAA10616.1| eukaryotic translation initiation factor 5 [Zea mays] e_value:0</td><td>11680.m04786|LOC_Os06g48350|protein Domain found in IF2B/IF5, putative e_value:0</td><td>0.161</td><td>(0.1478 0.9200)</td><td>0.1478</td><td>0.92</td></tr>
<tr><td>2421</td><td>SET5_N10_CLUSTER_33441</td><td>gi|14585847|gb|AAK67625.1| hypothetical protein CX157 [Homo sapiens] e_value:2.60478e-62</td><td>11680.m04976|LOC_Os06g50000|protein Complex 1 protein (LYR family), putative e_value:2.83541e-57</td><td>0.193</td><td>(0.0559 0.2898)</td><td>0.0559</td><td>0.2898</td></tr>
<tr><td>2422</td><td>WT009_H15_CLUSTER_34897</td><td>gi|50915890|ref|XP_468409.1| putative potassium channel beta subunit [Oryza sativa (japonica cultivar-group)] >gnl|BL_ORD_ID|1583890 gi|51964526|ref|XP_507048.1| PREDICTED P0643F09.35 gene product [Oryza sativa (japonica cultivar-group)] >gnl|BL_ORD</td><td>11668.m05657|LOC_Os02g57240|protein oxidoreductase, aldo/keto reductase family e_value:1.98184e-178</td><td>0.081</td><td>(0.0480 0.5939)</td><td>0.048</td><td>0.5939</td></tr>
<tr><td>2423</td><td>WT004_K01_CLUSTER_34319</td><td>gi|3915029|sp|Q40731|STAD_ORYSA Acyl-[acyl-carrier-protein] desaturase, chloroplast precursor (Stearoyl-ACP desaturase) >gnl|BL_ORD_ID|2332685 gi|976257|dbj|BAA07631.1| stearyl-ACP desaturase [Oryza sativa (japonica cultivar-group)] e_value:0</td><td>11670.m02952|LOC_Os04g31070|protein acyl-[acyl-carrier protein] desaturase, chloroplast precursor(ec 1.14.19.2) (stearoyl-acp desaturase) e_value:0</td><td>0.072</td><td>(0.0424 0.5922)</td><td>0.0424</td><td>0.5922</td></tr>
<tr><td>2424</td><td>SET3_B19_CLUSTER_32485</td><td>gi|34913256|ref|NP_917975.1| unknown protein [Oryza sativa (japonica cultivar-group)] >gi|22296365|dbj|BAC10134.1| unknown protein [Oryza sativa (japonica cultivar-group)] >gi|25453308|sp|Q8LHP0|U222_ORYSA Hypothetical UPF0222 protein P0519E12.3 e_v</td><td>11668.m00347|LOC_Os02g04160|protein expressed protein e_value:2.75198e-42</td><td></td><td>(0.0811 -1.0000)</td><td>0.0811</td><td>-1</td></tr>
<tr><td>2425</td><td>SET2_G21_CLUSTER_4287</td><td>gi|62733950|gb|AAX96059.1| dna-directed rna polymerase ii 8.2 kda polypeptide (ec 2.7.7.6)(rpb10) (rp10) (abc10) [Oryza sativa (japonica cultivar-group)] e_value:1.87852e-34</td><td>11687.m00836|LOC_Os11g08940|protein dna-directed rna polymerase ii 8.2 kda polypeptide (ec 2.7.7.6)(rpb10) (rp10) (abc10) e_value:3.0824e-36</td><td>0.029</td><td>(0.0185 0.6456)</td><td>0.0185</td><td>0.6456</td></tr>
<tr><td>2426</td><td>WT008_L12_CLUSTER_32072</td><td>gi|54290862|dbj|BAD61523.1| polypyrimidine tract-binding protein 1-like [Oryza sativa (japonica cultivar-group)] e_value:0</td><td>11667.m04182|LOC_Os01g43170|protein RNA recognition motif. (a.k.a. RRM, RBD, or RNP domain), putative e_value:0</td><td>0.222</td><td>(0.1127 0.5066)</td><td>0.1127</td><td>0.5066</td></tr>
<tr><td>2427</td><td>SET1_G07_CLUSTER_16821</td><td>gi|38347601|emb|CAE04892.2| OSJNBa0042I15.14 [Oryza sativa (japonica cultivar-group)] >gi|52000821|sp|Q7XLX6|S1FA2_ORYSA Putative DNA binding protein S1FA2 e_value:1.07633e-34</td><td>11670.m03211|LOC_Os04g33440|protein dna binding protein s1fa e_value:1.35325e-36</td><td>0.124</td><td>(0.0635 0.5124)</td><td>0.0635</td><td>0.5124</td></tr>
<tr><td>2428</td><td>WT005_K15_CLUSTER_15963</td><td>gi|50907587|ref|XP_465282.1| putative 26S proteasome regulatory particle triple-A ATPase subunit3 [Oryza sativa (japonica cultivar-group)] >gnl|BL_ORD_ID|1569342 gi|46390257|dbj|BAD15686.1| putative 26S proteasome regulatory particle triple-A ATPase su</td><td>11668.m02120|LOC_Os02g21970|protein 26S proteasome subunit P45 family e_value:0</td><td>0.041</td><td>(0.0207 0.5034)</td><td>0.0207</td><td>0.5034</td></tr>
<tr><td>2429</td><td>WT003_O13_CLUSTER_15963</td><td>gi|50907587|ref|XP_465282.1| putative 26S proteasome regulatory particle triple-A ATPase subunit3 [Oryza sativa (japonica cultivar-group)] >gnl|BL_ORD_ID|1569342 gi|46390257|dbj|BAD15686.1| putative 26S proteasome regulatory particle triple-A ATPase su</td><td>11668.m02120|LOC_Os02g21970|protein 26S proteasome subunit P45 family e_value:0</td><td>0.04</td><td>(0.0183 0.4596)</td><td>0.0183</td><td>0.4596</td></tr>
<tr><td>2430</td><td>SET4_D01_CLUSTER_13348</td><td>gi|34906436|ref|NP_914565.1| putative zinc finger transcription factor [Oryza sativa (japonica cultivar-group)] >gi|9988428|dbj|BAB12694.1| CCCH-type zinc finger protein -like [Oryza sativa (japonica cultivar-group)] e_value:4.22793e-180</td><td>11667.m00922|LOC_Os01g09620|protein Zinc finger C-x8-C-x5-C-x3-H type (and similar), putative e_value:0</td><td>0.203</td><td>(0.0948 0.4669)</td><td>0.0948</td><td>0.4669</td></tr>
<tr><td>2431</td><td>WT003_J03_CLUSTER_31939</td><td>gi|50916366|ref|XP_468586.1| Putative ubiquitin-conjugating enzyme [Oryza sativa (japonica cultivar-group)] >gnl|BL_ORD_ID|1584026 gi|25446690|gb|AAN74837.1| Putative ubiquitin-conjugating enzyme [Oryza sativa (japonica cultivar-group)] e_value:1.43582</td><td>11669.m00244|LOC_Os03g03130|protein Ubiquitin-conjugating enzyme e_value:2.06607e-91</td><td>0.065</td><td>(0.0334 0.5172)</td><td>0.0334</td><td>0.5172</td></tr>
</table>

PDF Complete
Your complimentary use period has ended. Thank you for using PDF Complete.
Click Here to upgrade to Unlimited Pages and Expanded Features

<table>
<tr><td>2432</td><td>WT013_I06_CLUSTER_31698</td><td>gi|50929717|ref|XP_474386.1| OSJNBb0017I01.27 [Oryza sativa (japonica cultivar-group)] >gnl|BL_ORD_ID|1594567 gi|32488629|emb|CAE03422.1| OSJNBa0032F06.5 [Oryza sativa (japonica cultivar-group)] >gnl|BL_ORD_ID|1594567 gi|32487413|emb|CAE05747.1| OSJ</td><td>11670.m05790|LOC_Os04g58470|protein hypothetical protein e_value:0</td><td>0.091</td><td>(0.0907 1.0025)</td><td>0.0907</td><td>1.0025</td></tr>
<tr><td>2433</td><td>WT003_F21_CLUSTER_34915</td><td>gi|50906727|ref|XP_464852.1| putative pre-mRNA splicing factor [Oryza sativa (japonica cultivar-group)] >gnl|BL_ORD_ID|1567424 gi|51963962|ref|XP_506764.1| PREDICTED OJ1113_G05.32 gene product [Oryza sativa (japonica cultivar-group)] >gnl|BL_ORD_ID|</td><td>11686.m03786|LOC_Os12g38430|protein RNA recognition motif. (a.k.a. RRM, RBD, or RNP domain), putative e_value:4.57354e-</td><td>0.184</td><td>(0.0743 0.4046)</td><td>0.0743</td><td>0.4046</td></tr>
<tr><td>2434</td><td>WT002_E14_CLUSTER_7596</td><td>gi|34902280|ref|NP_912486.1| Putative trehalose-6-phosphate synthase [Oryza sativa (japonica cultivar-group)] >gnl|BL_ORD_ID|1588591 gi|24308617|gb|AAN52740.1| Putative trehalose-6-phosphate synthase [Oryza sativa (japonica cultivar-group)]</td><td>11681.m02088|LOC_Os09g23350|protein trehalose-phosphatase, putative e_value:0</td><td>0.165</td><td>(0.2153 1.3082)</td><td>0.2153</td><td>1.3082</td></tr>
<tr><td>2435</td><td>SET1_M13_CLUSTER_32550</td><td>gi|54290412|dbj|BAD61282.1| 50S ribosomal protein L22-like [Oryza sativa (japonica cultivar-group)] e_value:2.64296e-101</td><td>11667.m04157|LOC_Os01g42980|protein ribosomal protein L22, putative e_value:6.12849e-103</td><td>0.23</td><td>(0.1950 0.8477)</td><td>0.195</td><td>0.8477</td></tr>
<tr><td>2436</td><td>WT002__C19_CLUSTER_33670</td><td>gi|55168305|gb|AAV44171.1| hypothetical protein [Oryza sativa (japonica cultivar-group)] >gnl|BL_ORD_ID|1465822 gi|55167930|gb|AAV43799.1| hypothetical protein [Oryza sativa (japonica cultivar-group)] e_value:0</td><td>11682.m02598|LOC_Os05g27820|protein CRAL/TRIO, N-terminus, putative e_value:0</td><td>0.187</td><td>(0.0475 0.2537)</td><td>0.0475</td><td>0.2537</td></tr>
<tr><td>2437</td><td>SET3_A13_CLUSTER_31576</td><td>gi|34898478|ref|NP_910585.1| ESTs AU058081(E3082),AU075427(E30384) correspond to a region of the predicted gene.~Similar to Spinacia oleracea proteasome 27 kD subunit (P52427) [Oryza sativa (japonica cultivar-group)] >gi|34898458|ref|NP_910575.1| ESTs</td><td>11680.m00748|LOC_Os06g07980|protein proteasome subunit alpha type 4 (ec 3.4.25.1) (20s proteasome alphasubunit c) (20s proteasome subunit alpha-3) e_value:1.06092e-135</td><td>0.064</td><td>(0.0206 0.3234)</td><td>0.0206</td><td>0.3234</td></tr>
<tr><td>2438</td><td>SET2_D03_CLUSTER_14623</td><td>gi|3445292|emb|CAA70936.1| B12Dg1 [Hordeum vulgare subsp. vulgare] >gi|471319|emb|CAA54065.1| HvB12D [Hordeum vulgare subsp. vulgare] >gi|2130028|pir||S60284 B12D protein - barley e_value:4.81689e-44</td><td>11680.m01347|LOC_Os06g13680|protein G-box binding protein, putative e_value:1.28486e-39</td><td>0.27</td><td>(0.1137 0.4219)</td><td>0.1137</td><td>0.4219</td></tr>
<tr><td>2439</td><td>WT012_L13_CLUSTER_20635</td><td>gi|51091558|dbj|BAD36295.1| putative RAD23 protein [Oryza sativa (japonica cultivar-group)] >gnl|BL_ORD_ID|239040 gi|51091502|dbj|BAD36240.1| putative RAD23 protein [Oryza sativa (japonica cultivar-group)] >gnl|BL_ORD_ID|239040 gi|55976306|sp|Q40742</td><td>11681.m02175|LOC_Os09g24200|protein UV excision repair protein Rad23 e_value:1.07031e-176</td><td>0.184</td><td>(0.1051 0.5708)</td><td>0.1051</td><td>0.5708</td></tr>
<tr><td>2440</td><td>WT003_O10_CLUSTER_33126</td><td>gi|50915896|ref|XP_468412.1| putative 3-ketoacyl-CoA thiolase; acetyl-CoA acyltransferase [Oryza sativa (japonica cultivar-group)] >gnl|BL_ORD_ID|1583881 gi|51964530|ref|XP_507050.1| PREDICTED OJ1136_C12.17 gene product [Oryza sativa (japonica cultivar</td><td>11668.m05659|LOC_Os02g57260|protein acetyl-CoA acyltransferases e_value:0</td><td>0.108</td><td>(0.0673 0.6244)</td><td>0.0673</td><td>0.6244</td></tr>
<tr><td>2441</td><td>WT008_G12_CLUSTER_32287</td><td>gi|14140154|emb|CAC39071.1| DnaJ-like protein [Oryza sativa] e_value:0</td><td>11668.m04224|LOC_Os02g43930|protein DnaJ-like protein</td><td>0.091</td><td>(0.0664 0.7328)</td><td>0.0664</td><td>0.7328</td></tr>
<tr><td>2442</td><td>SET4_G18_CLUSTER_18032</td><td>gi|6996146|emb|CAB75507.1| VIP3 protein [Avena fatua] >gi|12230463|sp|Q9M4C4|PFD4_AVEFA Probable prefoldin subunit 4 (VIP3 protein) e_value:2.48008e-61</td><td>11669.m04274|LOC_Os03g43020|protein VIP3 protein, putative e_value:1.07603e-61</td><td>0.083</td><td>(0.0484 0.5802)</td><td>0.0484</td><td>0.5802</td></tr>
<tr><td>2443</td><td>SET1_J03_CLUSTER_31603</td><td>gi|14018082|gb|AAK52145.1| putative transport protein particle component [Oryza sativa (japonica cultivar-group)] >gi|50939555|ref|XP_479305.1| transport protein particle component Bet3-like protein [Oryza sativa (japonica cultivar-group)]</td><td>11673.m04407|LOC_Os07g44790|protein Transport protein particle (TRAPP) component, Bet3 e_value:7.92458e-102</td><td>0.055</td><td>(0.0216 0.3908)</td><td>0.0216</td><td>0.3908</td></tr>
<tr><td>2444</td><td>SET5_N08_CLUSTER_33308</td><td>gi|50929027|ref|XP_474041.1| OSJNBb0034I13.8 [Oryza sativa (japonica cultivar-group)] >gi|39545744|emb|CAE04165.3| OSJNBb0034I13.8 [Oryza sativa (japonica cultivar-group)] e_value:1.85317e-55</td><td>11670.m05350|LOC_Os04g54430|protein expressed protein e_value:2.97898e-57</td><td>0.15</td><td>(0.1710 1.1373)</td><td>0.171</td><td>1.1373</td></tr>
<tr><td>2445</td><td>SET1_B12_CLUSTER_33460</td><td>gi|53792608|dbj|BAD53623.1| zinc finger protein-like [Oryza sativa (japonica cultivar-group)] >gi|53792616|dbj|BAD53630.1| zinc finger protein-like [Oryza sativa (japonica cultivar-group)] e_value:7.80443e-52</td><td>11680.m04915|LOC_Os06g49490|protein AT3g02790/F13E7_27 e_value:3.77996e-54</td><td>0.292</td><td>(0.0789 0.2703)</td><td>0.0789</td><td>0.2703</td></tr>
<tr><td>2446</td><td>WT005_F04_CLUSTER_32247</td><td>gi|32400796|gb|AAP80630.1| acidic ribosomal protein [Triticum aestivum] e_value:2.11372e-65</td><td>11682.m03504|LOC_Os05g37330|protein 60s acidic ribosomal protein p2b e_value:5.21966e-43</td><td>0.184</td><td>(0.1303 0.7073)</td><td>0.1303</td><td>0.7073</td></tr>
<tr><td>2447</td><td>WT004_P18_CLUSTER_34284</td><td>gi|34897938|ref|NP_910315.1| unknown protein [Oryza sativa (japonica cultivar-group)] >gnl|BL_ORD_ID|1752570 gi|7248408|dbj|BAA92731.1| unknown protein [Oryza sativa (japonica cultivar-group)] >gnl|BL_ORD_ID|1752570 gi|55296186|dbj|BAD67904.1| unkno</td><td>11680.m00153|LOC_Os06g02440|protein hypothetical protein e_value:4.26988e-62</td><td>0.194</td><td>(0.1334 0.6874)</td><td>0.1334</td><td>0.6874</td></tr>
<tr><td>2448</td><td>SET1_H15_CLUSTER_34990</td><td>gi|50934403|ref|XP_476729.1| OsRad6 [Oryza sativa (japonica cultivar-group)] >gi|50509962|dbj|BAD30372.1| OsRad6 [Oryza sativa (japonica cultivar-group)] >gi|33146810|dbj|BAC79758.1| OsRad6 [Oryza sativa (japonica cultivar-group)] e_value:3.50904e-8</td><td>11669.m05846|LOC_Os03g57790|protein ubiquitin carrier protein e_value:1.75588e-86</td><td>0.024</td><td>(0.0143 0.5851)</td><td>0.0143</td><td>0.5851</td></tr>
<tr><td>2449</td><td>WT005_J06_CLUSTER_34691</td><td>gi|6056371|gb|AAF02835.1| nucleolar protein [Arabidopsis thaliana] >gnl|BL_ORD_ID|1559572 gi|21592692|gb|AAM64641.1| SAR DNA binding protein, putative [Arabidopsis thaliana] >gnl|BL_ORD_ID|1559572 gi|20857405|gb|AAM26718.1| At1g56110/T6H22_9 [Arabid</td><td>11669.m02357|LOC_Os03g22880|protein Putative snoRNA binding domain e_value:0</td><td>0.155</td><td>(0.1020 0.6577)</td><td>0.102</td><td>0.6577</td></tr>
<tr><td>2450</td><td>SET6_O20_CLUSTER_34691</td><td>gi|6056371|gb|AAF02835.1| nucleolar protein [Arabidopsis thaliana] >gi|21592692|gb|AAM64641.1| SAR DNA binding protein, putative [Arabidopsis thaliana] >gi|20857405|gb|AAM26718.1| At1g56110/T6H22_9 [Arabidopsis thaliana] >gi|15223458|ref|NP_17600</td><td>11669.m02357|LOC_Os03g22880|protein Putative snoRNA binding domain e_value:0</td><td>0.111</td><td>(0.0926 0.8340)</td><td>0.0926</td><td>0.834</td></tr>
<tr><td>2451</td><td>WT005_H14_CLUSTER_31691</td><td>gi|50939569|ref|XP_479312.1| putative transcription factor APFI [Oryza sativa (japonica cultivar-group)] >gnl|BL_ORD_ID|2367842 gi|23237914|dbj|BAC16488.1| putative transcription factor APFI [Oryza sativa (japonica cultivar-group)] >gnl|BL_ORD_ID|23</td><td>11686.m00670|LOC_Os12g07220|protein expressed protein e_value:3.92657e-142</td><td>0.074</td><td>(0.0379 0.5145)</td><td>0.0379</td><td>0.5145</td></tr>
<tr><td>2452</td><td>WT002_F13_CLUSTER_33291</td><td>gi|11967891|emb|CAC19494.1| maize 20S proteasome alpha subunit [Zea mays] e_value:4.67939e-130</td><td>11669.m00798|LOC_Os03g08280|protein proteasome subunit alpha type 6 (ec 3.4.25.1) (20s proteasome alpha subunit a) (20s proteasome subunit alpha-1). [rice e_value:2.01417e-131</td><td>0.071</td><td>(0.0320 0.4502)</td><td>0.032</td><td>0.4502</td></tr>
<tr><td>2453</td><td>SET1_F16_CLUSTER_34067</td><td>gi|51090706|dbj|BAD36714.1| proteasome maturation factor-like [Oryza sativa (japonica cultivar-group)] e_value:3.72151e-66</td><td>11668.m05486|LOC_Os02g55640|protein At1g67250 e_value:7.04313e-68</td><td>0.246</td><td>(0.0909 0.3697)</td><td>0.0909</td><td>0.3697</td></tr>
<tr><td>2454</td><td>WT005_M01_CLUSTER_33690</td><td>gi|21803|emb|CAA77641.1| high mobility group protein [Triticum aestivum] >gnl|BL_ORD_ID|1718599 gi|729738|sp|P40621|HMGL_WHEAT HMG1/2-like protein e_value:1.98727e-84</td><td>11680.m05112|LOC_Os06g51220|protein HMG (high mobility group) box, putative e_value:2.71092e-75</td><td>0.112</td><td>(0.0572 0.5111)</td><td>0.0572</td><td>0.5111</td></tr>
<tr><td>2455</td><td>SET4_H09_CLUSTER_33690</td><td>gi|21803|emb|CAA77641.1| high mobility group protein [Triticum aestivum] >gi|729738|sp|P40621|HMGL_WHEAT HMG1/2-like protein e_value:1.11415e-85</td><td>11680.m05112|LOC_Os06g51220|protein HMG (high mobility group) box, putative e_value:9.31731e-76</td><td>0.12</td><td>(0.0543 0.4529)</td><td>0.0543</td><td>0.4529</td></tr>
<tr><td>2456</td><td>WT012_B16_CLUSTER_33690</td><td>gi|21803|emb|CAA77641.1| high mobility group protein [Triticum aestivum] >gnl|BL_ORD_ID|1718599 gi|729738|sp|P40621|HMGL_WHEAT HMG1/2-like protein e_value:3.16481e-85</td><td>11680.m05112|LOC_Os06g51220|protein HMG (high mobility group) box, putative e_value:2.71092e-75</td><td>0.096</td><td>(0.0572 0.5956)</td><td>0.0572</td><td>0.5956</td></tr>
<tr><td>2457</td><td>WT012__C17_CLUSTER_33690</td><td>gi|21803|emb|CAA77641.1| high mobility group protein [Triticum aestivum] >gnl|BL_ORD_ID|1718599 gi|729738|sp|P40621|HMGL_WHEAT HMG1/2-like protein e_value:3.16481e-85</td><td>11680.m05112|LOC_Os06g51220|protein HMG (high mobility group) box, putative e_value:2.71092e-75</td><td>0.096</td><td>(0.0572 0.5956)</td><td>0.0572</td><td>0.5956</td></tr>
<tr><td>2458</td><td>WT003_K03_CLUSTER_14437</td><td>gi|50947933|ref|XP_483494.1| nuclear transport factor 2 (NTF-2) [Oryza sativa (japonica cultivar-group)] >gnl|BL_ORD_ID|1609822 gi|42761381|dbj|BAD11649.1| nuclear transport factor 2 (NTF-2) [Oryza sativa (japonica cultivar-group)] >gnl|BL_ORD_ID|16</td><td>11674.m04229|LOC_Os08g42000|protein nuclear transport factor 2 (ntf-2) e_value:3.3905e-55</td><td>0.412</td><td>(0.1145 0.2779)</td><td>0.1145</td><td>0.2779</td></tr>
<tr><td>2459</td><td>SET1_E04_CLUSTER_8303</td><td>gi|54291778|gb|AAV32147.1| putative small nuclear ribonucleoprotein D2 [Oryza sativa (japonica cultivar-group)] e_value:2.20496e-50</td><td>11682.m02306|LOC_Os05g24970|protein small nuclear ribonucleoprotein-like protein e_value:9.61904e-53</td><td>0.067</td><td>(0.0307 0.4603)</td><td>0.0307</td><td>0.4603</td></tr>
<tr><td>2460</td><td>WT005_D03_CLUSTER_31925</td><td>gi|50936535|ref|XP_477795.1| 26S proteasome regulatory particle non-ATPase subunit12 [Oryza sativa (japonica cultivar-group)] >gnl|BL_ORD_ID|1525480 gi|34394724|dbj|BAC84087.1| 26S proteasome regulatory particle non-ATPase subunit12 [Oryza sativa (japo</td><td>11673.m02371|LOC_Os07g25420|protein 26S proteasome non-ATPase regulatory subunit Nin1/mts3, putative</td><td>0.112</td><td>(0.1036 0.9215)</td><td>0.1036</td><td>0.9215</td></tr>
<tr><td>2461</td><td>SET1_B15_CLUSTER_25261</td><td>gi|66840992|emb|CAI64395.1| putative high mobility group protein [Triticum aestivum] e_value:2.79934e-56</td><td>11670.m04662|LOC_Os04g47690|protein HMG (high mobility group) box, putative e_value:2.0025e-39</td><td>0.273</td><td>(0.1650 0.6049)</td><td>0.165</td><td>0.6049</td></tr>
<tr><td>2462</td><td>SET1_M01_CLUSTER_32648</td><td>gi|37991869|gb|AAR06315.1| putative small nuclear ribonucleoprotein polypeptide G [Oryza sativa (japonica cultivar-group)] >gi|50916481|ref|XP_468637.1| putative small nuclear ribonucleoprotein polypeptide G [Oryza sativa (japonica cultivar-group)] e_v</td><td>11669.m02993|LOC_Os03g29740|protein putative small nuclear ribonucleoprotein polypeptide G e_value:4.65106e-37</td><td>0.059</td><td>(0.0530 0.9047)</td><td>0.053</td><td>0.9047</td></tr>
<tr><td>2463</td><td>WT004_P23_CLUSTER_33241</td><td>gi|1352427|sp|P47815|IF1A_WHEAT Eukaryotic translation initiation factor 1A (EIF-1A) (EIF-4C) e_value:9.96448e-80</td><td>11680.m02308|LOC_Os06g23440|protein translation initiation factor eIF-1A, putative e_value:1.54423e-78</td><td>0.146</td><td>(0.0255 0.1745)</td><td>0.0255</td><td>0.1745</td></tr>
<tr><td>2464</td><td>SET1_I18_CLUSTER_1481</td><td>gi|50931949|ref|XP_475502.1| putative 60S ribosomal protein L38 [Oryza sativa (japonica cultivar-group)] >gi|46981281|gb|AAT07599.1| putative 60S ribosomal protein L38 [Oryza sativa (japonica cultivar-group)] e_value:8.92581e-32</td><td>11682.m04102|LOC_Os05g43160|protein 60s ribosomal protein l38. [mouse-ear cress e_value:1.87915e-33</td><td>0.016</td><td>(0.0127 0.8058)</td><td>0.0127</td><td>0.8058</td></tr>
<tr><td>2465</td><td>SET1_A03_CLUSTER_12680</td><td>gi|5107214|gb|AAD40019.1| small zinc finger-like protein [Oryza sativa] >gi|11358962|pir||T51188 small zinc finger-like protein [imported] - rice >gi|12230181|sp|Q9XGX7|TIM9_ORYSA Mitochondrial import inner membrane translocase subunit Tim9 e_value:</td><td>11686.m03774|LOC_Os12g38310|protein mitochondrial import inner membrane translocase subunit tim9 e_value:2.0154e-45</td><td>0.056</td><td>(0.0493 0.8805)</td><td>0.0493</td><td>0.8805</td></tr>
<tr><td>2466</td><td>SET4_M19_CLUSTER_31128</td><td>gi|34895672|ref|NP_909185.1| putative ribosomal protein L26 [Oryza sativa (japonica cultivar-group)] >gi|12328551|dbj|BAB21209.1| putative ribosomal protein L26 [Oryza sativa (japonica cultivar-group)] e_value:2.02796e-80</td><td>11667.m00395|LOC_Os01g04730|protein ribosomal protein L24, putative e_value:3.6614e-82</td><td>0.057</td><td>(0.0232 0.4050)</td><td>0.0232</td><td>0.405</td></tr>
<tr><td>2467</td><td>SET1_B24_CLUSTER_34494</td><td>gi|56202147|dbj|BAD73480.1| putative ribosomal protein L37a [Oryza sativa (japonica cultivar-group)] e_value:1.43896e-46</td><td>11682.m04620|LOC_Os05g48320|protein Ribosomal L37ae protein family e_value:1.95257e-48</td><td>0.024</td><td>(0.0094 0.3896)</td><td>0.0094</td><td>0.3896</td></tr>
<tr><td>2468</td><td>SET1_L16_CLUSTER_2357</td><td>gi|34901296|ref|NP_911994.1| putative 60S ribosomal protein L44 [Oryza sativa (japonica cultivar-group)] >gi|22831013|dbj|BAC15877.1| putative 60S ribosomal protein L44 [Oryza sativa (japonica cultivar-group)] e_value:6.05797e-56</td><td>11673.m03265|LOC_Os07g34000|protein Ribosomal protein L44, putative e_value:7.64463e-58</td><td>0.017</td><td>(0.0122 0.7209)</td><td>0.0122</td><td>0.7209</td></tr>
<tr><td>2469</td><td>SET1_K09_CLUSTER_7865</td><td>gi|1668773|emb|CAA67922.1| ubiquitin-like protein [Oryza sativa] >gi|57900450|dbj|BAD87743.1| putative SUMO protein [Oryza sativa (japonica cultivar-group)] >gi|19386713|dbj|BAB86095.1| putative SUMO protein [Oryza sativa (japonica cultivar-group)]</td><td>11667.m06951|LOC_Os01g68950|protein ubiquitin-like protein smt3. [rice e_value:1.04662e-46</td><td>0.074</td><td>(0.0470 0.6335)</td><td>0.047</td><td>0.6335</td></tr>
<tr><td>2470</td><td>SET4_K20_CLUSTER_17709</td><td>gi|31432043|gb|AAP53735.1| contains similarity to 40S ribosomal protein S17 [Oryza sativa (japonica cultivar-group)] >gi|37534292|ref|NP_921448.1| contains similarity to 40S ribosomal protein S17 [Oryza sativa (japonica cultivar-group)] e_value:1.73389</td><td>11676.m02341|LOC_Os10g27190|protein Ribosomal S17 e_value:2.59229e-62</td><td>0.204</td><td>(0.0810 0.3970)</td><td>0.081</td><td>0.397</td></tr>
<tr><td>2471</td><td>SET1_O20_CLUSTER_17709</td><td>gi|31432043|gb|AAP53735.1| contains similarity to 40S ribosomal protein S17 [Oryza sativa (japonica cultivar-group)] >gi|37534292|ref|NP_921448.1| contains similarity to 40S ribosomal protein S17 [Oryza sativa (japonica cultivar-group)] e_value:2.93229</td><td>11676.m02341|LOC_Os10g27190|protein Ribosomal S17 e_value:4.42178e-62</td><td>0.188</td><td>(0.0810 0.4300)</td><td>0.081</td><td>0.43</td></tr>
<tr><td>2472</td><td>WT004_D13_CLUSTER_33868</td><td>gi|55770401|ref|XP_550191.1| putative splicing factor [Oryza sativa (japonica cultivar-group)] >gnl|BL_ORD_ID|1753770 gi|54290784|dbj|BAD61423.1| putative splicing factor [Oryza sativa (japonica cultivar-group)] e_value:2.26438e-151</td><td>11667.m00561|LOC_Os01g06290|protein Transposable element protein, putative e_value:6.27529e-153</td><td>0.116</td><td>(0.0648 0.5605)</td><td>0.0648</td><td>0.5605</td></tr>
<tr><td>2473</td><td>WT006_G17_CLUSTER_31667</td><td>gi|51854269|gb|AAU10650.1| unknown protein [Oryza sativa (japonica cultivar-group)] e_value:1.46457e-96</td><td>11682.m04674|LOC_Os05g48800|protein expressed protein e_value:2.5733e-98</td><td>0.222</td><td>(0.0986 0.4439)</td><td>0.0986</td><td>0.4439</td></tr>
</table>

PDF Complete
Your complimentary use period has ended. Thank you for using PDF Complete.
Click Here to upgrade to Unlimited Pages and Expanded Features

<table>
<tr><td>2474</td><td>WT003_H01_CLUSTER_33040</td><td>gi|50941929|ref|XP_480492.1| putative nucleolin [Oryza sativa (japonica cultivar-group)] >gnl|BL_ORD_ID|1602913 gi|51449869|gb|AAU01907.1| putative nucleolin [Oryza sativa (indica cultivar-group)] >gnl|BL_ORD_ID|1602913 gi|40253662|dbj|BAD05605.1| p</td><td>11674.m00881|LOC_Os08g09350|protein RNA recognition motif. (a.k.a. RRM, RBD, or RNP domain), putative e_value:0</td><td>0.301</td><td>(0.2548 0.8452)</td><td>0.2548</td><td>0.8452</td></tr>
<tr><td>2475</td><td>SET1_E23_CLUSTER_32684</td><td>gi|52077016|dbj|BAD46049.1| unknown protein [Oryza sativa (japonica cultivar-group)] e_value:6.69681e-24</td><td>11680.m04664|LOC_Os06g47230|protein expressed protein e_value:1.1203e-25</td><td>0.15</td><td>(0.0679 0.4526)</td><td>0.0679</td><td>0.4526</td></tr>
<tr><td>2476</td><td>WT003_O14_CLUSTER_34993</td><td>gi|50355737|gb|AAT75262.1| putative DnaJ like protein [Oryza sativa (japonica cultivar-group)] e_value:0</td><td>11669.m05796|LOC_Os03g57340|protein putative DnaJ like protein e_value:0</td><td>0.069</td><td>(0.0499 0.7262)</td><td>0.0499</td><td>0.7262</td></tr>
<tr><td>2477</td><td>SET5_J20_CLUSTER_32235</td><td>gi|21070379|gb|AAM34280.1| translationally controlled tumor protein [Triticum aestivum] e_value:2.38844e-93</td><td>11687.m04135|LOC_Os11g43900|protein translationally controlled tumor protein homolog (tctp). e_value:1.32419e-83</td><td>0.211</td><td>(0.0907 0.4291)</td><td>0.0907</td><td>0.4291</td></tr>
<tr><td>2478</td><td>SET5_F12_CLUSTER_32082</td><td>gi|575355|emb|CAA57339.1| putative tumor suppresser [Oryza sativa (indica cultivar-group)] >gi|1172811|sp|P45635|RL101_ORYSA 60S ribosomal protein L10-1 (Putative tumor suppressor SC34) e_value:3.91627e-110</td><td>11687.m01089|LOC_Os11g11390|protein Ribosomal L10, putative e_value:3.44201e-113</td><td>0.128</td><td>(0.0614 0.4799)</td><td>0.0614</td><td>0.4799</td></tr>
<tr><td>2479</td><td>WT003_B09_CLUSTER_31651</td><td>gi|34910390|ref|NP_916542.1| ribosomal protein L28-like [Oryza sativa (japonica cultivar-group)] e_value:2.05082e-66</td><td>11667.m04981|LOC_Os01g51020|protein Ribosomal L28e protein family e_value:3.16152e-68</td><td>0.18</td><td>(0.0689 0.3829)</td><td>0.0689</td><td>0.3829</td></tr>
<tr><td>2480</td><td>SET5_G22_CLUSTER_33501</td><td>gi|50940807|ref|XP_479931.1| 60S acidic ribosomal protein P0 [Oryza sativa (japonica cultivar-group)] >gi|29467534|dbj|BAC66723.1| 60S acidic ribosomal protein P0 [Oryza sativa (japonica cultivar-group)] >gi|455401|dbj|BAA04668.1| acidic ribosomal p</td><td>11686.m00313|LOC_Os12g03880|protein ribosomal protein L10, putative e_value:2.77147e-161</td><td>0.124</td><td>(0.0531 0.4298)</td><td>0.0531</td><td>0.4298</td></tr>
<tr><td>2481</td><td>SET1_L22_CLUSTER_9843</td><td>gi|42733478|dbj|BAD11336.1| BRI1-KD interacting protein 108 [Oryza sativa (japonica cultivar-group)] e_value:1.85753e-62</td><td>11669.m02294|LOC_Os03g22340|protein Ribosomal L22e protein family e_value:2.87947e-64</td><td>0.223</td><td>(0.0472 0.2112)</td><td>0.0472</td><td>0.2112</td></tr>
<tr><td>2482</td><td>WT003_B18_CLUSTER_32017</td><td>gi|19919694|gb|AAM08320.1| small Ran-related GTP-binding protein [Triticum aestivum] >gnl|BL_ORD_ID|929517 gi|16903082|gb|AAL30396.1| small Ras-related GTP-binding protein [Triticum aestivum] e_value:2.33321e-129</td><td>11682.m04797|LOC_Os05g49890|protein Ras family, putative e_value:1.01974e-128</td><td>0.025</td><td>(0.0121 0.4801)</td><td>0.0121</td><td>0.4801</td></tr>
<tr><td>2483</td><td>WT003_M03_CLUSTER_34370</td><td>gi|50911805|ref|XP_467310.1| putative 60S ribosomal protein L12 [Oryza sativa (japonica cultivar-group)] >gnl|BL_ORD_ID|1580427 gi|41052969|dbj|BAD07879.1| putative 60S ribosomal protein L12 [Oryza sativa (japonica cultivar-group)] e_value:9.37802e-82</td><td>11670.m04974|LOC_Os04g50990|protein 60s ribosomal protein l12 e_value:1.68471e-83</td><td>0.122</td><td>(0.0455 0.3727)</td><td>0.0455</td><td>0.3727</td></tr>
<tr><td>2484</td><td>WT004_K18_CLUSTER_34227</td><td>gi|57471716|gb|AAW50989.1| ribosomal protein L7 [Triticum aestivum] e_value:3.5691e-127</td><td>11670.m05048|LOC_Os04g51630|protein 60S ribosomal protein L7 e_value:9.8793e-123</td><td>0.134</td><td>(0.0631 0.4721)</td><td>0.0631</td><td>0.4721</td></tr>
<tr><td>2485</td><td>SET1_M21_CLUSTER_13714</td><td>gi|34905656|ref|NP_914175.1| P0475H04.11 [Oryza sativa (japonica cultivar-group)] >gi|55168072|gb|AAV43940.1| putative 60S ribosomal protein L29 [Oryza sativa (japonica cultivar-group)] >gi|12060516|dbj|BAB20645.1| putative ribosomal protein L29 [Or</td><td>11682.m02695|LOC_Os05g28750|protein Ribosomal L29e protein family, putative e_value:4.2877e-25</td><td>0.49</td><td>(0.0944 0.1928)</td><td>0.0944</td><td>0.1928</td></tr>
<tr><td>2486</td><td>WT004_D08_CLUSTER_26555</td><td>gi|7711024|emb|CAB90214.1| putative elongation factor 1 beta [Hordeum vulgare subsp. vulgare] e_value:7.6639e-122</td><td>11673.m04142|LOC_Os07g42300|protein translation elongation factor eEF-1 beta - rice e_value:1.35357e-99</td><td>0.123</td><td>(0.1102 0.8952)</td><td>0.1102</td><td>0.8952</td></tr>
<tr><td>2487</td><td>WT002_O10_CLUSTER_21037</td><td>gi|57471712|gb|AAW50987.1| ribosomal protein l34 [Triticum aestivum] e_value:2.27099e-61</td><td>11674.m03359|LOC_Os08g33920|protein Ribosomal protein L34e e_value:1.21315e-60</td><td>0.035</td><td>(0.0306 0.8698)</td><td>0.0306</td><td>0.8698</td></tr>
<tr><td>2488</td><td>SET1_P18_CLUSTER_31995</td><td>gi|9931636|gb|AAG02240.1| ribosomal protein s6 RPS6-2 [Zea mays] e_value:1.12366e-122</td><td>11673.m04211|LOC_Os07g42950|protein Ribosomal protein S6e, putative e_value:9.31568e-124</td><td>0.163</td><td>(0.0674 0.4140)</td><td>0.0674</td><td>0.414</td></tr>
<tr><td>2489</td><td>SET6_I23_CLUSTER_34252</td><td>gi|7440266|pir||T04083 probable ribosomal protein S16 - rice >gi|1172818|sp|P46294|RS16_ORYSA 40S ribosomal protein S16 >gi|538428|gb|AAA33916.1| ribosomal protein S16 >gi|1096552|prf||2111468A ribosomal protein S16 e_value:7.95252e-74</td><td>11687.m00250|LOC_Os11g03400|protein ribosomal protein S9 e_value:3.14132e-77</td><td>0.084</td><td>(0.0449 0.5332)</td><td>0.0449</td><td>0.5332</td></tr>
<tr><td>2490</td><td>SET3_B14_CLUSTER_33575</td><td>gi|50944205|ref|XP_481630.1| 60S ribosomal protein L7A [Oryza sativa (japonica cultivar-group)] >gi|51979663|ref|XP_507578.1| PREDICTED P0703C03.43 gene product [Oryza sativa (japonica cultivar-group)] >gi|51964820|ref|XP_507194.1| PREDICTED P0703C0</td><td>11674.m02302|LOC_Os08g23710|protein 60s ribosomal protein l7a e_value:1.28518e-139</td><td>0.052</td><td>(0.0206 0.3994)</td><td>0.0206</td><td>0.3994</td></tr>
<tr><td>2491</td><td>SET1_N05_CLUSTER_34151</td><td>gi|55775376|ref|XP_550614.1| 40S subunit ribosomal protein [Oryza sativa (japonica cultivar-group)] >gi|55297192|dbj|BAD68866.1| 40S subunit ribosomal protein [Oryza sativa (japonica cultivar-group)] >gi|55296170|dbj|BAD67888.1| 40S subunit ribosoma</td><td>11680.m00349|LOC_Os06g04290|protein Ribosomal protein S10p/S20e, putative e_value:6.29053e-62</td><td>0.166</td><td>(0.0719 0.4340)</td><td>0.0719</td><td>0.434</td></tr>
<tr><td>2492</td><td>WT004__C05_CLUSTER_33742</td><td>gi|50916422|ref|XP_468609.1| putative ribosomal protein L27a [Oryza sativa (japonica cultivar-group)] >gnl|BL_ORD_ID|1539929 gi|30017566|gb|AAP12988.1| putative ribosomal protein L27a [Oryza sativa (japonica cultivar-group)] e_value:1.86637e-72</td><td>11669.m02963|LOC_Os03g29460|protein putative ribosomal protein L27a e_value:3.07525e-74</td><td>0.186</td><td>(0.0623 0.3354)</td><td>0.0623</td><td>0.3354</td></tr>
<tr><td>2493</td><td>SET1_N11_CLUSTER_16310</td><td>gi|32400871|gb|AAP80667.1| ribosomal Pr 117 [Triticum aestivum] e_value:1.05869e-75</td><td>11676.m02891|LOC_Os10g32920|protein 60S ribosomal protein L17 e_value:5.95827e-77</td><td>0.01</td><td>(0.0032 0.3034)</td><td>0.0032</td><td>0.3034</td></tr>
<tr><td>2494</td><td>SET5_N16_CLUSTER_34533</td><td>gi|50938417|ref|XP_478736.1| putative 40S ribosomal protein S11 [Oryza sativa (japonica cultivar-group)] >gi|33146750|dbj|BAC79661.1| putative 40S ribosomal protein S11 [Oryza sativa (japonica cultivar-group)] >gi|50508298|dbj|BAD30107.1| putative 4</td><td>11673.m03665|LOC_Os07g37780|protein ribosomal protein S17, putative e_value:5.81576e-81</td><td>0.155</td><td>(0.0835 0.5401)</td><td>0.0835</td><td>0.5401</td></tr>
<tr><td>2495</td><td>SET1_J10_CLUSTER_32310</td><td>gi|57471698|gb|AAW50980.1| ribosomal protein L36 [Triticum aestivum] e_value:9.53549e-54</td><td>11682.m03642|LOC_Os05g38520|protein Ribosomal protein L36e e_value:2.11998e-52</td><td>0.143</td><td>(0.0471 0.3281)</td><td>0.0471</td><td>0.3281</td></tr>
<tr><td>2496</td><td>WT003_J11_CLUSTER_3118</td><td>gi|38345032|emb|CAE01633.2| OSJNBa0029H02.16 [Oryza sativa (japonica cultivar-group)] >gnl|BL_ORD_ID|1521515 gi|50926169|ref|XP_473060.1| OSJNBa0029H02.16 [Oryza sativa (japonica cultivar-group)] >gnl|BL_ORD_ID|1521515 gi|10241664|emb|CAC09501.1| pu</td><td>11670.m04068|LOC_Os04g42270|protein ribosomal protein L23, putative e_value:2.80981e-76</td><td>0.071</td><td>(0.0436 0.6103)</td><td>0.0436</td><td>0.6103</td></tr>
<tr><td>2497</td><td>WT004_D14_CLUSTER_34873</td><td>gi|33087067|gb|AAP92747.1| ribosomal L9-like protein [Oryza sativa (japonica cultivar-group)] e_value:1.51364e-90</td><td>11681.m02877|LOC_Os09g31180|protein ribosomal protein L6, putative e_value:3.36565e-92</td><td>0.177</td><td>(0.0639 0.3608)</td><td>0.0639</td><td>0.3608</td></tr>
<tr><td>2498</td><td>SET4_K23_CLUSTER_34873</td><td>gi|50907049|ref|XP_465013.1| hypothetical protein [Oryza sativa (japonica cultivar-group)] >gi|47847939|dbj|BAD21729.1| hypothetical protein [Oryza sativa (japonica cultivar-group)] e_value:4.80038e-62</td><td>11668.m01782|LOC_Os02g18720|protein hypothetical protein e_value:1.09589e-63</td><td>0.466</td><td>(0.5439 1.1661)</td><td>0.5439</td><td>1.1661</td></tr>
<tr><td>2499</td><td>WT004_D23_CLUSTER_32612</td><td>gi|5106775|gb|AAD39838.1| ribosomal protein S12 [Hordeum vulgare] >gnl|BL_ORD_ID|645914 gi|12229949|sp|Q9XHS0|RS12_HORVU 40S ribosomal protein S12 e_value:2.08929e-73</td><td>11673.m01218|LOC_Os07g12650|protein Ribosomal protein L7Ae, putative e_value:2.18942e-69</td><td>0.052</td><td>(0.0275 0.5249)</td><td>0.0275</td><td>0.5249</td></tr>
<tr><td>2500</td><td>WT011_D16_CLUSTER_32739</td><td>gi|50924099|ref|XP_472410.1| OJ000126_13.9 [Oryza sativa (japonica cultivar-group)] >gnl|BL_ORD_ID|1591448 gi|38346327|emb|CAE02065.2| OJ000126_13.9 [Oryza sativa (japonica cultivar-group)] >gnl|BL_ORD_ID|1591448 gi|50253050|dbj|BAD29299.1| 40S ribo</td><td>11670.m03246|LOC_Os04g33750|protein ribosomal protein S11, putative e_value:9.16543e-80</td><td>0.06</td><td>(0.0151 0.2501)</td><td>0.0151</td><td>0.2501</td></tr>
<tr><td>2501</td><td>WT003_L11_CLUSTER_34222</td><td>gi|50511482|gb|AAT77404.1| putative 60S ribosomal protein L18a [Oryza sativa (japonica cultivar-group)] e_value:3.18021e-95</td><td>11667.m04612|LOC_Os01g47660|protein Ribosomal L18ae protein family e_value:3.41976e-96</td><td>0.138</td><td>(0.0676 0.4918)</td><td>0.0676</td><td>0.4918</td></tr>
<tr><td>2502</td><td>WT002_O01_CLUSTER_32477</td><td>gi|48596901|dbj|BAD22763.1| ribosomal protein [Bromus inermis] e_value:1.2835e-149</td><td>11668.m00058|LOC_Os02g01560|protein 40s ribosomal protein s4 e_value:9.45661e-146</td><td>0.076</td><td>(0.0363 0.4779)</td><td>0.0363</td><td>0.4779</td></tr>
<tr><td>2503</td><td>SET6__C02_CLUSTER_32477</td><td>gi|48596901|dbj|BAD22763.1| ribosomal protein [Bromus inermis] e_value:1.41401e-149</td><td>11668.m00058|LOC_Os02g01560|protein 40s ribosomal protein s4 e_value:9.45661e-146</td><td>0.085</td><td>(0.0369 0.4356)</td><td>0.0369</td><td>0.4356</td></tr>
<tr><td>2504</td><td>WT012_G22_CLUSTER_33054</td><td>gi|32489919|emb|CAE05511.1| OSJNBa0038P21.4 [Oryza sativa (japonica cultivar-group)] e_value:3.39422e-111</td><td>11670.m02661|LOC_Os04g28180|protein Ribosomal protein S8e e_value:8.10788e-113</td><td>0.078</td><td>(0.0411 0.5251)</td><td>0.0411</td><td>0.5251</td></tr>
<tr><td>2505</td><td>WT011_H16_CLUSTER_33499</td><td>gi|949878|emb|CAA90651.1| elongation factor 1-alpha [Hordeum vulgare subsp. vulgare] >gnl|BL_ORD_ID|2318694 gi|322865|pir||JC1454 translation elongation factor eEF-1 alpha chain - wheat >gnl|BL_ORD_ID|2318694 gi|399414|sp|Q03033|EF1A_WHEAT ELONGATIO</td><td>11669.m00772|LOC_Os03g08050|protein translation elongation factor EF-1, subunit alpha e_value:0</td><td>0.069</td><td>(0.0233 0.3386)</td><td>0.0233</td><td>0.3386</td></tr>
<tr><td>2506</td><td>SET1_J09_CLUSTER_5005</td><td>gi|50906961|ref|XP_464969.1| putative 60S ribosomal protein L27 [Oryza sativa (japonica cultivar-group)] >gi|47848339|dbj|BAD22201.1| putative 60S ribosomal protein L27 [Oryza sativa (japonica cultivar-group)] >gi|47847708|dbj|BAD21487.1| putative 6</td><td>11668.m01748|LOC_Os02g18380|protein Ribosomal L27e protein family, putative e_value:2.84002e-68</td><td>0.333</td><td>(0.0675 0.2029)</td><td>0.0675</td><td>0.2029</td></tr>
<tr><td>2507</td><td>WT013_P17_CLUSTER_11579</td><td>gi|34893994|ref|NP_908322.1| putative 40S ribosomal protein S5 [Oryza sativa (japonica cultivar-group)] >gnl|BL_ORD_ID|1751520 gi|15408845|dbj|BAB64234.1| putative 40S ribosomal protein S5 [Oryza sativa (japonica cultivar-group)] >gnl|BL_ORD_ID|1751</td><td>11667.m00007|LOC_Os01g01060|protein ribosomal protein S7 e_value:1.93769e-96</td><td>0.202</td><td>(0.0710 0.3518)</td><td>0.071</td><td>0.3518</td></tr>
<tr><td>2508</td><td>WT004_B11_CLUSTER_11579</td><td>gi|34893994|ref|NP_908322.1| putative 40S ribosomal protein S5 [Oryza sativa (japonica cultivar-group)] >gnl|BL_ORD_ID|1751520 gi|15408845|dbj|BAB64234.1| putative 40S ribosomal protein S5 [Oryza sativa (japonica cultivar-group)] >gnl|BL_ORD_ID|1751</td><td>11667.m00007|LOC_Os01g01060|protein ribosomal protein S7 e_value:8.69788e-97</td><td>0.195</td><td>(0.0686 0.3522)</td><td>0.0686</td><td>0.3522</td></tr>
<tr><td>2509</td><td>SET1_H19_CLUSTER_33178</td><td>gi|14495010|emb|CAC42163.1| putative ribosomal protein S27 [Hordeum vulgare subsp. vulgare] >gi|14495008|emb|CAC42162.1| putative ribosomal protein S27 [Hordeum vulgare subsp. vulgare] >gi|14495006|emb|CAC42134.1| putative ribosomal protein S27 [Hor</td><td>11670.m02628|LOC_Os04g27860|protein zinc finger protein, putative e_value:7.05181e-46</td><td>0.032</td><td>(0.0153 0.4730)</td><td>0.0153</td><td>0.473</td></tr>
<tr><td>2510</td><td>WT004_O01_CLUSTER_2435</td><td>gi|57471704|gb|AAW50983.1| ribosomal protein L11 [Triticum aestivum] e_value:3.04455e-96</td><td>11667.m01051|LOC_Os01g10820|protein ribosomal L5P family C-terminus, putative e_value:1.16724e-91</td><td>0.074</td><td>(0.0461 0.6219)</td><td>0.0461</td><td>0.6219</td></tr>
<tr><td>2511</td><td>SET1_L10_CLUSTER_34725</td><td>gi|57471710|gb|AAW50986.1| ribosomal protein L30 [Triticum aestivum] e_value:2.16497e-57</td><td>11682.m03930|LOC_Os05g41110|protein 60s ribosomal protein l30 e_value:1.08333e-56</td><td>0.195</td><td>(0.0438 0.2248)</td><td>0.0438</td><td>0.2248</td></tr>
<tr><td>2512</td><td>SET1_B18_CLUSTER_32372</td><td>gi|50919443|ref|XP_470118.1| 40S ribosomal protein S23 [Oryza sativa (japonica cultivar-group)] >gi|34908032|ref|NP_915363.1| 40S ribosomal protein S23 [Oryza sativa (japonica cultivar-group)] >gi|34908030|ref|NP_915362.1| 40S ribosomal protein S23</td><td>11669.m06141|LOC_Os03g60400|protein 40S ribosomal protein S23 e_value:8.68359e-79</td><td>0.012</td><td>(0.0092 0.7478)</td><td>0.0092</td><td>0.7478</td></tr>
<tr><td>2513</td><td>WT002__C23_CLUSTER_33970</td><td>gi|57471700|gb|AAW50981.1| ribosomal protein L6 [Triticum aestivum] e_value:4.3338e-121</td><td>11670.m03786|LOC_Os04g39700|protein protein 60S ribosomal protein L6 F2P9.8 [imported] - Arabidopsis thaliana e_value:4.89708e-107</td><td>0.13</td><td>(0.0810 0.6249)</td><td>0.081</td><td>0.6249</td></tr>
<tr><td>2514</td><td>SET6_M02_CLUSTER_33970</td><td>gi|57471700|gb|AAW50981.1| ribosomal protein L6 [Triticum aestivum] e_value:4.2383e-113</td><td>11670.m03786|LOC_Os04g39700|protein protein 60S ribosomal protein L6 F2P9.8 [imported] - Arabidopsis thaliana e_value:4.00606e-109</td><td>0.131</td><td>(0.0654 0.4995)</td><td>0.0654</td><td>0.4995</td></tr>
<tr><td>2515</td><td>WT004_I16_CLUSTER_33858</td><td>gi|56785265|dbj|BAD82174.1| putative ribosomal protein L5 [Oryza sativa (japonica cultivar-group)] e_value:9.32655e-154</td><td>11667.m06758|LOC_Os01g67140|protein Ribosomal L18p/L5e family, putative e_value:2.73996e-155</td><td>0.126</td><td>(0.0801 0.6355)</td><td>0.0801</td><td>0.6355</td></tr>
</table>

PDF Complete
Your complimentary use period has ended. Thank you for using PDF Complete.
Click Here to upgrade to Unlimited Pages and Expanded Features

<table>
<tr><td>2516</td><td>SET1_A15_CLUSTER_34230</td><td>gi|54290930|dbj|BAD61612.1| putative 60S ribosomal protein L31 [Oryza sativa (japonica cultivar-group)] e_value:3.87413e-58</td><td>11680.m02100|LOC_Os06g21480|protein Ribosomal protein L31e, putative e_value:5.95429e-60</td><td>0.102</td><td>(0.0603 0.5918)</td><td>0.0603</td><td>0.5918</td></tr>
<tr><td>2517</td><td>WT003_E04_CLUSTER_6006</td><td>gi|48596905|dbj|BAD22765.1| glycoprotein [Bromus inermis] e_value:2.22745e-68</td><td>11668.m03941|LOC_Os02g40880|protein Ribosomal protein L14, putative e_value:4.50636e-68</td><td>0.064</td><td>(0.0312 0.4868)</td><td>0.0312</td><td>0.4868</td></tr>
<tr><td>2518</td><td>SET1_F24_CLUSTER_22504</td><td>gi|50915832|ref|XP_468380.1| putative ribosomal protein L37 [Oryza sativa (japonica cultivar-group)] >gi|47847878|dbj|BAD21671.1| putative ribosomal protein L37 [Oryza sativa (japonica cultivar-group)] e_value:4.33167e-47</td><td>11668.m05628|LOC_Os02g56990|protein Ribosomal protein L37e, putative e_value:3.89383e-49</td><td>0.163</td><td>(0.0579 0.3553)</td><td>0.0579</td><td>0.3553</td></tr>
<tr><td>2519</td><td>WT004_N19_CLUSTER_31899</td><td>gi|50912175|ref|XP_467495.1| ATP/ADP translocator [Oryza sativa (japonica cultivar-group)] >gnl|BL_ORD_ID|2374943 gi|51979354|ref|XP_507526.1| PREDICTED OJ2056_H01.33 gene product [Oryza sativa (japonica cultivar-group)] >gnl|BL_ORD_ID|2374943 gi|51</td><td>11668.m04732|LOC_Os02g48720|protein adp,atp carrier protein, mitochondrial precursor (adp/atp translocase)(adenine nucleotide translocator) (ant) e_value:0</td><td>0.085</td><td>(0.0362 0.4239)</td><td>0.0362</td><td>0.4239</td></tr>
<tr><td>2520</td><td>SET5_J08_CLUSTER_32009</td><td>gi|2286153|gb|AAB64290.1| cytoplasmic malate dehydrogenase [Zea mays] >gi|7431171|pir||T02935 malate dehydrogenase (EC 1.1.1.-), cytosolic - maize >gi|18202485|sp|Q08062|MDHC_MAIZE Malate dehydrogenase, cytoplasmic e_value:8.86418e-176</td><td>11676.m02982|LOC_Os10g33800|protein cytoplasmic malate dehydrogenase e_value:1.22942e-175</td><td>0.09</td><td>(0.0505 0.5582)</td><td>0.0505</td><td>0.5582</td></tr>
<tr><td>2521</td><td>SET1_G19_CLUSTER_11920</td><td>gi|1070356|emb|CAA92107.1| cytochrome c oxidase, Vc subunit [Hordeum vulgare subsp. vulgare] >gi|7489419|pir||T05365 probable cytochrome-c oxidase (EC 1.9.3.1) chain Vc - barley >gi|2493852|sp|Q42841|COX5C_HORVU Cytochrome c oxidase polypeptide Vc (</td><td>11686.m03677|LOC_Os12g37420|protein Cytochrome c oxidase subunit Vc (COX5C) e_value:3.36532e-30</td><td>0.075</td><td>(0.0208 0.2776)</td><td>0.0208</td><td>0.2776</td></tr>
<tr><td>2522</td><td>WT005__C14_CLUSTER_34280</td><td>gi|61675574|dbj|BAD91202.1| mitochondrial F1-ATPase gamma subunit [Ipomoea nil] e_value:1.80529e-138</td><td>11676.m01426|LOC_Os10g17280|protein ATP synthase F1, gamma subunit e_value:1.92447e-133</td><td>0.268</td><td>(0.1468 0.5474)</td><td>0.1468</td><td>0.5474</td></tr>
<tr><td>2523</td><td>WT003_O21_CLUSTER_6305</td><td>gi|34905998|ref|NP_914346.1| putative cytochrome B5 [Oryza sativa (japonica cultivar-group)] >gnl|BL_ORD_ID|1541116 gi|15289978|dbj|BAB63673.1| putative cytochrome b5 [Oryza sativa (japonica cultivar-group)] e_value:1.34883e-66</td><td>11667.m07437|LOC_Os01g73990|protein Cytochrome b5-like Heme/Steroid binding domain, putative e_value:1.66498e-68</td><td>0.085</td><td>(0.0613 0.7166)</td><td>0.0613</td><td>0.7166</td></tr>
<tr><td>2524</td><td>SET6_A03_CLUSTER_372</td><td>gi|34897072|ref|NP_909882.1| putative actin-depolymerizing factor [Oryza sativa (japonica cultivar-group)] >gi|12957717|gb|AAK09235.1| putative actin-depolymerizing factor [Oryza sativa (japonica cultivar-group)] e_value:4.47778e-72</td><td>11669.m05730|LOC_Os03g56790|protein putative actin-depolymerizing factor e_value:5.24557e-74</td><td>0.062</td><td>(0.0371 0.5951)</td><td>0.0371</td><td>0.5951</td></tr>
<tr><td>2525</td><td>WT002_F08_CLUSTER_18055</td><td>gi|50251207|dbj|BAD27614.1| putative fiber protein Fb15 [Oryza sativa (japonica cultivar-group)] e_value:5.24588e-31</td><td>11668.m00755|LOC_Os02g07910|protein fiber protein Fb15 e_value:6.82876e-33</td><td>0.606</td><td>(0.1670 0.2758)</td><td>0.167</td><td>0.2758</td></tr>
<tr><td>2526</td><td>WT006_N15_CLUSTER_27824</td><td>gi|50917879|ref|XP_469336.1| unknown protein [Oryza sativa] >gnl|BL_ORD_ID|1535884 gi|32352140|dbj|BAC78563.1| hypothetical protein [Oryza sativa (japonica cultivar-group)] >gnl|BL_ORD_ID|1535884 gi|13174237|gb|AAK14411.1| unknown protein [Oryza sat</td><td>11669.m04816|LOC_Os03g48080|protein expressed protein e_value:4.13866e-60</td><td>0.199</td><td>(0.1052 0.5282)</td><td>0.1052</td><td>0.5282</td></tr>
<tr><td>2527</td><td>SET5_P12_CLUSTER_27824</td><td>gi|50917879|ref|XP_469336.1| unknown protein [Oryza sativa] >gi|32352140|dbj|BAC78563.1| hypothetical protein [Oryza sativa (japonica cultivar-group)] >gi|13174237|gb|AAK14411.1| unknown protein [Oryza sativa] e_value:1.29615e-56</td><td>11669.m04816|LOC_Os03g48080|protein expressed protein e_value:1.59391e-58</td><td>0.213</td><td>(0.1166 0.5463)</td><td>0.1166</td><td>0.5463</td></tr>
<tr><td>2528</td><td>SET2_F19_CLUSTER_34845</td><td>gi|4098321|gb|AAD10488.1| beta-tubulin 2 [Triticum aestivum] >gi|8928430|sp|Q9ZRB1|TBB2_WHEAT Tubulin beta-2 chain (Beta-2 tubulin) e_value:0</td><td>11667.m05872|LOC_Os01g59150|protein beta-tubulin R1623 - rice e_value:0</td><td>0.02</td><td>(0.0097 0.4831)</td><td>0.0097</td><td>0.4831</td></tr>
<tr><td>2529</td><td>WT007_N24_CLUSTER_34798</td><td>gi|4165488|emb|CAA10663.1| alpha-tubulin 3 [Hordeum vulgare subsp. vulgare] >gnl|BL_ORD_ID|1315966 gi|8928432|sp|Q9ZRR5|TBA3_HORVU Tubulin alpha-3 chain e_value:0</td><td>11687.m01331|LOC_Os11g14220|protein Tubulin/FtsZ family, GTPase domain, putative e_value:0</td><td>0.022</td><td>(0.0097 0.4428)</td><td>0.0097</td><td>0.4428</td></tr>
<tr><td>2530</td><td>WT007_L03_CLUSTER_34798</td><td>gi|1619297|emb|CAA69724.1| alpha-tubulin 2 [Hordeum vulgare subsp. vulgare] >gnl|BL_ORD_ID|1714925 gi|8928422|sp|Q96460|TBA2_HORVU Tubulin alpha-2 chain e_value:0</td><td>11687.m01331|LOC_Os11g14220|protein Tubulin/FtsZ family, GTPase domain, putative e_value:0</td><td>0.023</td><td>(0.0117 0.5186)</td><td>0.0117</td><td>0.5186</td></tr>
<tr><td>2531</td><td>WT005_A05_CLUSTER_34984</td><td>gi|15808779|gb|AAL08496.1| ascorbate peroxidase [Hordeum vulgare] e_value:7.18596e-146</td><td>11673.m04917|LOC_Os07g49400|protein Peroxidase e_value:1.65004e-131</td><td>0.12</td><td>(0.0556 0.4627)</td><td>0.0556</td><td>0.4627</td></tr>
<tr><td>2532</td><td>WT004_P16_CLUSTER_31647</td><td>gi|50919483|ref|XP_470138.1| putative actin depolymerizing factor [Oryza sativa (japonica cultivar-group)] >gnl|BL_ORD_ID|1587506 gi|29124123|gb|AAO65864.1| putative actin depolymerizing factor [Oryza sativa (japonica cultivar-group)] e_value:4.4975e-5</td><td>11669.m06163|LOC_Os03g60590|protein putative actin depolymerizing factor e_value:5.32072e-54</td><td>0.214</td><td>(0.1844 0.8634)</td><td>0.1844</td><td>0.8634</td></tr>
<tr><td>2533</td><td>WT005_N01_CLUSTER_32122</td><td>gi|38346061|emb|CAE02009.2| OJ000223_09.15 [Oryza sativa (japonica cultivar-group)] >gnl|BL_ORD_ID|1523165 gi|50925303|ref|XP_472949.1| OJ000223_09.15 [Oryza sativa (japonica cultivar-group)] e_value:1.0967e-180</td><td>11670.m03922|LOC_Os04g40950|protein glyceraldehyde-3-phosphate dehydrogenase, type I e_value:0</td><td>0.065</td><td>(0.0363 0.5605)</td><td>0.0363</td><td>0.5605</td></tr>
<tr><td>2534</td><td>SET1_L01_CLUSTER_31959</td><td>gi|33113259|gb|AAP94211.1| enolase [Oryza sativa (japonica cultivar-group)] e_value:0</td><td>11676.m00727|LOC_Os10g08550|protein enolase e_value:0</td><td>0.056</td><td>(0.0244 0.4352)</td><td>0.0244</td><td>0.4352</td></tr>
<tr><td>2535</td><td>WT005_H08_CLUSTER_1528</td><td>gi|34909292|ref|NP_915993.1| ubiquitin conjugating enzyme [Oryza sativa (japonica cultivar-group)] >gnl|BL_ORD_ID|1554096 gi|34909298|ref|NP_915996.1| ubiquitin conjugating enzyme [Oryza sativa (japonica cultivar-group)] >gnl|BL_ORD_ID|1554096 gi|21</td><td>11667.m06014|LOC_Os01g60410|protein Ubiquitin-conjugating enzyme e_value:1.80268e-85</td><td>0.015</td><td>(0.0059 0.3941)</td><td>0.0059</td><td>0.3941</td></tr>
<tr><td>2536</td><td>SET5_E23_CLUSTER_31957</td><td>gi|478306|pir||JN0839 translation initiation factor eIF-4A - wheat >gi|1170509|sp|P41378|IF4A_WHEAT Eukaryotic initiation factor 4A (eIF4A) (eIF-4A) e_value:0</td><td>11680.m04829|LOC_Os06g48750|protein eukaryotic initiation factor 4a (eif4a) (eif-4a) e_value:0</td><td>0.016</td><td>(0.0095 0.5820)</td><td>0.0095</td><td>0.582</td></tr>
<tr><td>2537</td><td>SET1_B07_CLUSTER_31834</td><td>gi|50923665|ref|XP_472193.1| OSJNBb0006N15.8 [Oryza sativa (japonica cultivar-group)] >gi|38346113|emb|CAE04591.2| OSJNBb0006N15.8 [Oryza sativa (japonica cultivar-group)] e_value:1.24119e-55</td><td>11670.m02918|LOC_Os04g30730|protein ribosomal protein L29, putative e_value:1.62604e-57</td><td>0.176</td><td>(0.0298 0.1697)</td><td>0.0298</td><td>0.1697</td></tr>
<tr><td>2538</td><td>WT007_L21_CLUSTER_31763</td><td>gi|31126753|gb|AAP44673.1| putative 60S ribosomal protein L1 [Oryza sativa (japonica cultivar-group)] >gnl|BL_ORD_ID|1858620 gi|34897236|ref|NP_909964.1| putative 60S ribosomal protein L1 [Oryza sativa (japonica cultivar-group)] >gnl|BL_ORD_ID|18586</td><td>11669.m05899|LOC_Os03g58200|protein putative 60S ribosomal protein L1 e_value:0</td><td>0.179</td><td>(0.0884 0.4945)</td><td>0.0884</td><td>0.4945</td></tr>
<tr><td>2539</td><td>WT004_N23_CLUSTER_31763</td><td>gi|51978958|ref|XP_507356.1| PREDICTED OJ1014_E09.28 gene product [Oryza sativa (japonica cultivar-group)] >gnl|BL_ORD_ID|1521856 gi|50934675|ref|XP_476865.1| putative 60S ribosomal protein L4/L1 [Oryza sativa (japonica cultivar-group)]</td><td>11673.m00768|LOC_Os07g08330|protein ribosomal protein L4/L1 family, putative e_value:0</td><td>0.261</td><td>(0.0937 0.3587)</td><td>0.0937</td><td>0.3587</td></tr>
<tr><td>2540</td><td>WT003_D01_CLUSTER_32116</td><td>gi|31432723|gb|AAP54321.1| putative transcription factor [Oryza sativa (japonica cultivar-group)] >gnl|BL_ORD_ID|2012207 gi|37535464|ref|NP_922034.1| putative transcription factor [Oryza sativa (japonica cultivar-group)] >gnl|BL_ORD_ID|2012207 gi|22</td><td>11676.m03023|LOC_Os10g34180|protein putative transcription factor e_value:7.16227e-82</td><td>0.064</td><td>(0.0286 0.4452)</td><td>0.0286</td><td>0.4452</td></tr>
<tr><td>2541</td><td>SET1_F22_CLUSTER_32149</td><td>gi|31745220|gb|AAP68880.1| putative ribosomal protein S29 [Oryza sativa (japonica cultivar-group)] >gi|34915198|ref|NP_919056.1| putative ribosomal protein S29 [Oryza sativa (japonica cultivar-group)] e_value:9.44612e-29</td><td>11687.m03895|LOC_Os11g41610|protein ribosomal protein S14p/S29e e_value:1.53593e-30</td><td>0.029</td><td>(0.0232 0.7908)</td><td>0.0232</td><td>0.7908</td></tr>
<tr><td>2542</td><td>WT002_I09_CLUSTER_32503</td><td>gi|31431694|gb|AAP53433.1| putative Ras-related protein Rab [Oryza sativa (japonica cultivar-group)] >gnl|BL_ORD_ID|2010563 gi|37533688|ref|NP_921146.1| putative Ras-related protein Rab [Oryza sativa (japonica cultivar-group)] >gnl|BL_ORD_ID|2010563</td><td>11676.m01972|LOC_Os10g23100|protein Ras family, putative e_value:6.21874e-115</td><td>0.037</td><td>(0.0225 0.6023)</td><td>0.0225</td><td>0.6023</td></tr>
<tr><td>2543</td><td>WT003_N19_CLUSTER_35003</td><td>gi|31433074|gb|AAP54634.1| putative gamma-lyase [Oryza sativa (japonica cultivar-group)] >gnl|BL_ORD_ID|2012665 gi|37536090|ref|NP_922347.1| putative gamma-lyase [Oryza sativa (japonica cultivar-group)] >gnl|BL_ORD_ID|2012665 gi|13786446|gb|AAK39571</td><td>11676.m03369|LOC_Os10g37340|protein putative gamma-lyase e_value:0</td><td>0.319</td><td>(0.0831 0.2608)</td><td>0.0831</td><td>0.2608</td></tr>
<tr><td>2544</td><td>WT003_D24_CLUSTER_35003</td><td>gi|50428662|gb|AAT77013.1| putative F8K7.10 protein [Oryza sativa (japonica cultivar-group)] e_value:0</td><td>11669.m06367|LOC_Os03g62370|protein putative F8K7.10 protein e_value:0</td><td>0.345</td><td>(0.1316 0.3815)</td><td>0.1316</td><td>0.3815</td></tr>
<tr><td>2545</td><td>WT002_N23_CLUSTER_35003</td><td>gi|34910808|ref|NP_916751.1| GDSL-motif lipase/hydrolase-like protein [Oryza sativa (japonica cultivar-group)] e_value:0</td><td>11667.m05178|LOC_Os01g52770|protein GDSL-like Lipase/Acylhydrolase e_value:0</td><td>0.234</td><td>(0.0668 0.2856)</td><td>0.0668</td><td>0.2856</td></tr>
<tr><td>2546</td><td>WT002_A22_CLUSTER_35003</td><td>gi|11385533|gb|AAG34849.1| glutathione S-transferase GST 41 [Zea mays] e_value:7.7992e-117</td><td>11682.m03212|LOC_Os05g34150|protein glutathione S-transferase GST 41 e_value:6.82383e-118</td><td>0.406</td><td>(0.1177 0.2896)</td><td>0.1177</td><td>0.2896</td></tr>
<tr><td>2547</td><td>WT010_G15_CLUSTER_35003</td><td>gi|21105742|gb|AAM34770.1| nam-like protein 7 [Petunia x hybrida] e_value:3.90195e-59</td><td>11681.m02921|LOC_Os09g32040|protein Similar to nam-like protein 7 e_value:0</td><td>0.471</td><td>(0.2247 0.4765)</td><td>0.2247</td><td>0.4765</td></tr>
<tr><td>2548</td><td>WT010_D17_CLUSTER_35003</td><td>gi|50932583|ref|XP_475819.1| unknown protein [Oryza sativa (japonica cultivar-group)] e_value:3.74166e-177</td><td>11682.m04469|LOC_Os05g46460|protein hydrolase, alpha/beta fold family, putative e_value:8.19946e-179</td><td>0.19</td><td>(0.0774 0.4073)</td><td>0.0774</td><td>0.4073</td></tr>
<tr><td>2549</td><td>WT009_G22_CLUSTER_35003</td><td>gi|50940191|ref|XP_479623.1| putative glycosyltransferase family [Oryza sativa (japonica cultivar-group)] >gnl|BL_ORD_ID|1534748 gi|34394028|dbj|BAC84059.1| putative glycosyltransferase family [Oryza sativa (japonica cultivar-group)] e_value:3.47945e-8</td><td>11669.m01804|LOC_Os03g17850|protein Glycosyltransferase family 43, putative e_value:6.65382e-87</td><td>0.492</td><td>(0.3622 0.7364)</td><td>0.3622</td><td>0.7364</td></tr>
<tr><td>2550</td><td>WT009_A01_CLUSTER_35003</td><td>gi|50899146|ref|XP_450361.1| hypothetical protein [Oryza sativa (japonica cultivar-group)] >gnl|BL_ORD_ID|1563626 gi|48717074|dbj|BAD23762.1| hypothetical protein [Oryza sativa (japonica cultivar-group)] e_value:1.07463e-133</td><td>11681.m00680|LOC_Os09g08550|protein F-box domain, putative e_value:3.00155e-135</td><td>0.906</td><td>(0.3208 0.3543)</td><td>0.3208</td><td>0.3543</td></tr>
<tr><td>2551</td><td>SET5_B02_CLUSTER_33951</td><td>gi|21592866|gb|AAM64816.1| Ribosomal protein L7Ae-like [Arabidopsis thaliana] e_value:5.01386e-59</td><td>11669.m01352|LOC_Os03g13800|protein AT5g20160/F5O24_50 e_value:2.3034e-64</td><td>0.048</td><td>(0.0287 0.6006)</td><td>0.0287</td><td>0.6006</td></tr>
<tr><td>2552</td><td>WT004_I10_CLUSTER_33412</td><td>gi|34911282|ref|NP_916988.1| guanine nucleotide-binding protein beta subujit-like protein (GPB-LR) (RWD) [Oryza sativa (japonica cultivar-group)] >gnl|BL_ORD_ID|2352740 gi|540535|dbj|BAA07404.1| q group of receptor for activated C-kinase [Oryza sativa</td><td>11667.m04794|LOC_Os01g49290|protein guanine nucleotide-binding protein beta subunit-like protein (gpb-lr) (rwd). [rice e_value:1.08652e-171</td><td>0.229</td><td>(0.0949 0.4154)</td><td>0.0949</td><td>0.4154</td></tr>
<tr><td>2553</td><td>SET1_E08_CLUSTER_12989</td><td>gi|30102524|gb|AAP21180.1| At3g08610 [Arabidopsis thaliana] e_value:3.04193e-23</td><td>11682.m03842|LOC_Os05g40300|protein hypothetical protein e_value:9.41586e-25</td><td>0.56</td><td>(0.1393 0.2487)</td><td>0.1393</td><td>0.2487</td></tr>
<tr><td>2554</td><td>WT008__C11_CLUSTER_31526</td><td>gi|50905713|ref|XP_464345.1| Proteasome subunit beta type 3 [Oryza sativa (japonica cultivar-group)] >gnl|BL_ORD_ID|1566122 gi|49388033|dbj|BAD25149.1| Proteasome subunit beta type 3 [Oryza sativa (japonica cultivar-group)] e_value:1.58982e-107</td><td>11668.m00821|LOC_Os02g08520|protein proteasome subunit beta type 3 (ec 3.4.25.1) (20s proteasome alphasubunit c) (20s proteasome subunit beta-3) e_value:3.49682e-109</td><td>0.082</td><td>(0.0306 0.3749)</td><td>0.0306</td><td>0.3749</td></tr>
<tr><td>2555</td><td>WT002_E23_CLUSTER_31526</td><td>gi|50905713|ref|XP_464345.1| Proteasome subunit beta type 3 [Oryza sativa (japonica cultivar-group)] >gnl|BL_ORD_ID|1566122 gi|49388033|dbj|BAD25149.1| Proteasome subunit beta type 3 [Oryza sativa (japonica cultivar-group)] e_value:7.84561e-107</td><td>11668.m00821|LOC_Os02g08520|protein proteasome subunit beta type 3 (ec 3.4.25.1) (20s proteasome alphasubunit c) (20s proteasome subunit beta-3) e_value:1.73545e-108</td><td>0.075</td><td>(0.0453 0.6042)</td><td>0.0453</td><td>0.6042</td></tr>
<tr><td>2556</td><td>SET6_I21_CLUSTER_31526</td><td>gi|50905713|ref|XP_464345.1| Proteasome subunit beta type 3 [Oryza sativa (japonica cultivar-group)] >gi|49388033|dbj|BAD25149.1| Proteasome subunit beta type 3 [Oryza sativa (japonica cultivar-group)] e_value:7.7883e-107</td><td>11668.m00821|LOC_Os02g08520|protein proteasome subunit beta type 3 (ec 3.4.25.1) (20s proteasome alphasubunit c) (20s proteasome subunit beta-3) e_value:1.73545e-108</td><td>0.065</td><td>(0.0431 0.6660)</td><td>0.0431</td><td>0.666</td></tr>
<tr><td>2557</td><td>WT005_I03_CLUSTER_2965</td><td>gi|34909656|ref|NP_916175.1| OJ1414_E05.5 [Oryza sativa (japonica cultivar-group)] >gnl|BL_ORD_ID|1550302 gi|20161213|dbj|BAB90140.1| unknown protein [Oryza sativa (japonica cultivar-group)] e_value:0</td><td>11667.m05442|LOC_Os01g55190|protein expressed protein e_value:0</td><td>0.068</td><td>(0.0561 0.8225)</td><td>0.0561</td><td>0.8225</td></tr>
</table>

PDF Complete
Your complimentary use period has ended. Thank you for using PDF Complete.
Click Here to upgrade to Unlimited Pages and Expanded Features

<table>
<tr><td>2558</td><td>WT008_B17_CLUSTER_31533</td><td>gi|62733732|gb|AAX95841.1| probable zinc finger protein – alfalfa (fragment) [Oryza sativa (japonica cultivar–group)] >gnl|BL_ORD_ID|154592 gi|62701860|gb|AAX92933.1| probable zinc finger protein – alfalfa (fragment) [Oryza sativa (japonica cultivar–gr</td><td>11687.m01307|LOC_Os11g14010|protein probable zinc finger protein – alfalfa (fragment) e_value:8.65495e–125</td><td>0.159</td><td>(0.1071 0.6742)</td><td>0.1071</td><td>0.6742</td></tr>
<tr><td>2559</td><td>WT002_A02_CLUSTER_7314</td><td>gi|50911579|ref|XP_467197.1| putative GAMM1 protein [Oryza sativa (japonica cultivar–group)] >gnl|BL_ORD_ID|1580300 gi|41052722|dbj|BAD07579.1| putative GAMM1 protein [Oryza sativa (japonica cultivar–group)] e_value:0</td><td>11668.m04461|LOC_Os02g46150|protein Uncharacterised protein family (UPF0160) e_value:0</td><td>0.104</td><td>(0.0490 0.4709)</td><td>0.049</td><td>0.4709</td></tr>
<tr><td>2560</td><td>WT010_C18_CLUSTER_34778</td><td>gi|4325045|gb|AAD17232.1| S–adenosylmethionine decarboxylase precursor [Triticum aestivum] e_value:0</td><td>11670.m04049|LOC_Os04g42090|protein Adenosylmethionine decarboxylase, putative e_value:0</td><td>0.095</td><td>(0.0766 0.8035)</td><td>0.0766</td><td>0.8035</td></tr>
<tr><td>2561</td><td>SET3_F17_CLUSTER_34778</td><td>gi|61820747|ref|XP_594900.1| PREDICTED: similar to germinal histone H4 gene [Bos taurus] e_value:3.82288e–43</td><td>11682.m03701|LOC_Os05g39050|protein histone H4 – Arabidopsis thaliana e_value:3.83027e–44</td><td>0</td><td>(0.0000 0.1185)</td><td>0</td><td>0.1185</td></tr>
<tr><td>2562</td><td>WT013_D07_CLUSTER_34977</td><td>gi|2258469|gb|AAB71836.1| replication protein A1 [Oryza sativa] >gnl|BL_ORD_ID|2036362 gi|7440032|pir||T03582 probable replication protein A1 – rice e_value:0</td><td>11669.m01105|LOC_Os03g11540|protein replication protein A1 e_value:0</td><td>0.22</td><td>(0.1202 0.5455)</td><td>0.1202</td><td>0.5455</td></tr>
<tr><td>2563</td><td>SET1_C09_CLUSTER_34181</td><td>gi|2641619|gb|AAC12662.1| ubiquitin–conjugating enzyme protein E2 [Zea mays] >gi|7437989|pir||T01329 ubiquitin–conjugating enzyme E2 – maize e_value:1.10497e–88</td><td>11682.m03646|LOC_Os05g38550|protein Ubiquitin–conjugating enzyme e_value:1.93495e–90</td><td>0.08</td><td>(0.0353 0.4405)</td><td>0.0353</td><td>0.4405</td></tr>
<tr><td>2564</td><td>WT005_B07_CLUSTER_33554</td><td>gi|50944921|ref|XP_481988.1| putative ethylene–responsive transcriptional coactivator [Oryza sativa (japonica cultivar–group)] >gnl|BL_ORD_ID|1605706 gi|38637102|dbj|BAD03357.1| putative ethylene–responsive transcriptional coactivator [Oryza sativa (ja</td><td>11674.m02728|LOC_Os08g27850|protein Helix–turn–helix, putative e_value:4.77591e–69</td><td>0.086</td><td>(0.0424 0.4923)</td><td>0.0424</td><td>0.4923</td></tr>
<tr><td>2565</td><td>SET1_H09_CLUSTER_233</td><td>gi|19912029|dbj|BAB88326.1| BLE1 protein [Oryza sativa] >gi|57863802|gb|AAS72370.2| BLE1 protein [Oryza sativa (japonica cultivar–group)] e_value:4.40209e–40</td><td>11680.m05083|LOC_Os06g50970|protein BLE1 protein e_value:4.30983e–35</td><td>0.13</td><td>(0.0628 0.4825)</td><td>0.0628</td><td>0.4825</td></tr>
<tr><td>2566</td><td>SET1_C16_CLUSTER_33938</td><td>gi|50932369|ref|XP_475712.1| putative GTP–binding protein Rab7a [Oryza sativa (japonica cultivar–group)] >gi|46575953|gb|AAT01314.1| putative GTP–binding protein Rab7a [Oryza sativa (japonica cultivar–group)] e_value:4.12616e–114</td><td>11682.m04194|LOC_Os05g44050|protein ras–related protein rab7 (possible apospory–associated protein) e_value:6.70719e–</td><td>0.018</td><td>(0.0084 0.4690)</td><td>0.0084</td><td>0.469</td></tr>
<tr><td>2567</td><td>WT003_G18_CLUSTER_33591</td><td>gi|8671498|dbj|BAA96832.1| alpha 5 subunit of 20S proteasome [Oryza sativa (japonica cultivar–group)] >gnl|BL_ORD_ID|52236 gi|12229920|sp|Q9LSU1|PSA5_ORYSA Proteasome subunit alpha type 5 (20S proteasome alpha subunit E) (20S proteasome subunit alpha–5</td><td>11687.m03741|LOC_Os11g40140|protein proteasome subunit alpha type 5 (ec 3.4.25.1) (20s proteasome alphasubunit e) (20s proteasome subunit alpha–5) e_value:4.39603e–128</td><td>0.038</td><td>(0.0197 0.5214)</td><td>0.0197</td><td>0.5214</td></tr>
<tr><td>2568</td><td>SET2_E20_CLUSTER_33591</td><td>gi|8671498|dbj|BAA96832.1| alpha 5 subunit of 20S proteasome [Oryza sativa (japonica cultivar–group)] >gi|12229920|sp|Q9LSU1|PSA5_ORYSA Proteasome subunit alpha type 5 (20S proteasome alpha subunit E) (20S proteasome subunit alpha–5) e_value:1.88685e–1</td><td>11687.m03741|LOC_Os11g40140|protein proteasome subunit alpha type 5 (ec 3.4.25.1) (20s proteasome alphasubunit e) (20s proteasome subunit alpha–5) e_value:4.39603e–128</td><td>0.038</td><td>(0.0197 0.5214)</td><td>0.0197</td><td>0.5214</td></tr>
<tr><td>2569</td><td>SET1_N04_CLUSTER_20996</td><td>gi|34897872|ref|NP_910282.1| putative phosphogluconate dehydrogenase [Oryza sativa (japonica cultivar–group)] >gi|7363280|dbj|BAA93024.1| putative phosphogluconate dehydrogenase [Oryza sativa (japonica cultivar–group)] >gi|19569141|gb|AAL92029.1| cy</td><td>11680.m00122|LOC_Os06g02150|protein 6–phosphogluconate dehydrogenase, decarboxylating e_value:0</td><td>0.077</td><td>(0.0411 0.5326)</td><td>0.0411</td><td>0.5326</td></tr>
<tr><td>2570</td><td>SET1_P10_CLUSTER_2487</td><td>gi|12060388|dbj|BAB20581.1| response regulator 6 [Zea mays] e_value:3.92806e–70</td><td>11687.m00388|LOC_Os11g04720|protein response regulator 6 e_value:1.51498e–77</td><td>0.236</td><td>(0.1251 0.5307)</td><td>0.1251</td><td>0.5307</td></tr>
<tr><td>2571</td><td>WT005_N23_CLUSTER_23954</td><td>gi|52076355|dbj|BAD45176.1| unknown protein [Oryza sativa (japonica cultivar–group)] >gnl|BL_ORD_ID|250517 gi|52076293|dbj|BAD45078.1| unknown protein [Oryza sativa (japonica cultivar–group)] e_value:3.57475e–75</td><td>11667.m03680|LOC_Os01g38480|protein hypothetical protein e_value:5.91097e–77</td><td>0.661</td><td>(0.1713 0.2591)</td><td>0.1713</td><td>0.2591</td></tr>
<tr><td>2572</td><td>SET1_J20_CLUSTER_29855</td><td>gi|50934495|ref|XP_476775.1| putative DNA–directed RNA polymerase II 13.6K chain [Oryza sativa (japonica cultivar–group)] >gi|34394446|dbj|BAC83620.1| putative DNA–directed RNA polymerase II 13.6K chain [Oryza sativa (japonica cultivar–group)] e_value:</td><td>11673.m00690|LOC_Os07g07610|protein RNA polymerase Rpb3/Rpb11 dimerisation domain, putative e_value:2.69516e–60</td><td>0.084</td><td>(0.0510 0.6096)</td><td>0.051</td><td>0.6096</td></tr>
<tr><td>2573</td><td>WT008_K05_CLUSTER_33879</td><td>gi|51965042|ref|XP_507305.1| PREDICTED P0702E04.27 gene product [Oryza sativa (japonica cultivar–group)] >gnl|BL_ORD_ID|1609829 gi|50947949|ref|XP_483502.1| coated vesicle membrane protein–like [Oryza sativa (japonica cultivar–group)] >gnl|BL_ORD_ID</td><td>11674.m04235|LOC_Os08g42050|protein emp24/gp25L/p24 family e_value:3.11775e–103</td><td>0.07</td><td>(0.0869 1.2450)</td><td>0.0869</td><td>1.245</td></tr>
<tr><td>2574</td><td>SET1_P07_CLUSTER_11458</td><td>gi|32401365|gb|AAP80854.1| autophagy [Triticum aestivum] e_value:1.72105e–61</td><td>11673.m03136|LOC_Os07g32800|protein Microtubule associated protein 1A/1B, light chain 3 e_value:1.027e–59</td><td>0.053</td><td>(0.0296 0.5566)</td><td>0.0296</td><td>0.5566</td></tr>
<tr><td>2575</td><td>WT013_L15_CLUSTER_33871</td><td>gi|30270581|gb|AAP15161.1| superal1 [Zea mays] e_value:3.66513e–104</td><td>11680.m04281|LOC_Os06g43590|protein SNF7 family e_value:9.8545e–104</td><td>0.097</td><td>(0.0248 0.2554)</td><td>0.0248</td><td>0.2554</td></tr>
<tr><td>2576</td><td>WT005_J02_CLUSTER_30557</td><td>gi|4099148|gb|AAD10335.1| YLP [Hordeum vulgare] e_value:2.40331e–121</td><td>11667.m04535|LOC_Os01g46980|protein ATP synthase (E/31 kDa) subunit e_value:1.11363e–109</td><td>0.188</td><td>(0.0768 0.4089)</td><td>0.0768</td><td>0.4089</td></tr>
<tr><td>2577</td><td>SET6_A17_CLUSTER_34838</td><td>gi|6911856|emb|CAB72156.1| putative protein [Arabidopsis thaliana] >gi|15230632|ref|NP_190104.1| ubiquitin family protein [Arabidopsis thaliana] >gi|11358004|pir||T47458 hypothetical protein T14D3.120 – Arabidopsis thaliana e_value:2.65641e–33</td><td>11687.m00405|LOC_Os11g04880|protein Ubiquitin family e_value:8.07202e–37</td><td>0.128</td><td>(0.0218 0.1705)</td><td>0.0218</td><td>0.1705</td></tr>
<tr><td>2578</td><td>SET6_A16_CLUSTER_34838</td><td>gi|6911856|emb|CAB72156.1| putative protein [Arabidopsis thaliana] >gi|15230632|ref|NP_190104.1| ubiquitin family protein [Arabidopsis thaliana] >gi|11358004|pir||T47458 hypothetical protein T14D3.120 – Arabidopsis thaliana e_value:2.65641e–33</td><td>11687.m00405|LOC_Os11g04880|protein Ubiquitin family e_value:8.07202e–37</td><td>0.128</td><td>(0.0218 0.1705)</td><td>0.0218</td><td>0.1705</td></tr>
<tr><td>2579</td><td>WT004_E20_CLUSTER_9804</td><td>gi|50909323|ref|XP_466150.1| putative Vacuolar ATP synthase 16 kDa proteolipid subunit [Oryza sativa (japonica cultivar–group)] >gnl|BL_ORD_ID|1574629 gi|50725751|dbj|BAD33262.1| putative Vacuolar ATP synthase 16 kDa proteolipid subunit [Oryza sativa (</td><td>11682.m00068|LOC_Os05g01560|protein V–type ATPase, C subunit, putative e_value:8.07966e–86</td><td>0.025</td><td>(0.0125 0.5063)</td><td>0.0125</td><td>0.5063</td></tr>
<tr><td>2580</td><td>WT012_P10_CLUSTER_34703</td><td>gi|18483235|gb|AAL73979.1| methionine synthase protein [Sorghum bicolor] e_value:0</td><td>11686.m04284|LOC_Os12g42890|protein 5–methyltetrahydropteroyltriglutamate––homocysteine S–methyltransferase e_value:0</td><td>0.068</td><td>(0.0408 0.6036)</td><td>0.0408</td><td>0.6036</td></tr>
<tr><td>2581</td><td>WT009_H23_CLUSTER_31456</td><td>gi|50948653|ref|XP_483854.1| type 1 membrane protein –like [Oryza sativa (japonica cultivar–group)] >gnl|BL_ORD_ID|1613379 gi|42409098|dbj|BAD10349.1| type 1 membrane protein –like [Oryza sativa (japonica cultivar–group)] e_value:8.88856e–134</td><td>11674.m04582|LOC_Os08g45220|protein expressed protein e_value:2.42303e–135</td><td>0.391</td><td>(0.1923 0.4924)</td><td>0.1923</td><td>0.4924</td></tr>
<tr><td>2582</td><td>WT013_L14_CLUSTER_34104</td><td>gi|22652|emb|CAA48143.1| GRP94 homologue [Hordeum vulgare] >gnl|BL_ORD_ID|2380647 gi|544242|sp|P36183|ENPL_HORVU Endoplasmin homolog precursor (GRP94 homolog) e_value:0</td><td>11680.m05012|LOC_Os06g50300|protein Hsp90 protein, putative e_value:0</td><td>0.101</td><td>(0.0429 0.4238)</td><td>0.0429</td><td>0.4238</td></tr>
<tr><td>2583</td><td>WT004_L17_CLUSTER_31665</td><td>gi|34913460|ref|NP_918077.1| putative carboxymethylenebutenolidase [Oryza sativa (japonica cultivar–group)] >gnl|BL_ORD_ID|1541486 gi|20521349|dbj|BAB91862.1| carboxymethylenebutenolidase–like protein [Oryza sativa (japonica cultivar–group)] e_value:1.</td><td>11667.m03289|LOC_Os01g34700|protein carboxymethylenebutenolidase family, putative</td><td>0.1</td><td>(0.0537 0.5360)</td><td>0.0537</td><td>0.536</td></tr>
<tr><td>2584</td><td>SET1_C05_CLUSTER_34339</td><td>gi|1572627|gb|AAB67991.1| Cu/Zn superoxide dismutase [Triticum aestivum] >gi|7433318|pir||T06800 superoxide dismutase (EC 1.15.1.1) (Cu–Zn) 2, chloroplast – wheat e_value:2.71087e–108</td><td>11674.m04529|LOC_Os08g44770|protein Copper/zinc superoxide dismutase, putative e_value:5.50248e–99</td><td>0.146</td><td>(0.0629 0.4298)</td><td>0.0629</td><td>0.4298</td></tr>
<tr><td>2585</td><td>WT003_N14_CLUSTER_12309</td><td>gi|8980491|emb|CAB96931.1| thioredoxin h [Triticum aestivum] >gnl|BL_ORD_ID|1344426 gi|9502274|gb|AAF88067.1| thioredoxin H [Triticum aestivum] e_value:1.06333e–60</td><td>11682.m04112|LOC_Os05g43250|protein Thioredoxin e_value:1.1161e–50</td><td>0.276</td><td>(0.1224 0.4438)</td><td>0.1224</td><td>0.4438</td></tr>
<tr><td>2586</td><td>SET1_L11_CLUSTER_34520</td><td>gi|50944921|ref|XP_481988.1| putative ethylene–responsive transcriptional coactivator [Oryza sativa (japonica cultivar–group)] >gi|38637102|dbj|BAD03357.1| putative ethylene–responsive transcriptional coactivator [Oryza sativa (japonica cultivar–group)</td><td>11674.m02728|LOC_Os08g27850|protein Helix–turn–helix, putative e_value:2.99838e–63</td><td>0.143</td><td>(0.1033 0.7215)</td><td>0.1033</td><td>0.7215</td></tr>
<tr><td>2587</td><td>WT005_G05_CLUSTER_17954</td><td>gi|21553376|gb|AAM62469.1| acyl carrier protein, putative [Arabidopsis thaliana] e_value:1.00954e–36</td><td>11682.m02907|LOC_Os05g31290|protein acyl carrier protein, putative e_value:9.04013e–55</td><td>0.128</td><td>(0.0698 0.5466)</td><td>0.0698</td><td>0.5466</td></tr>
<tr><td>2588</td><td>WT003_E19_CLUSTER_33530</td><td>gi|34897646|ref|NP_910169.1| putative disulfide–isomerase precursor [Oryza sativa] >gnl|BL_ORD_ID|1742342 gi|54291859|gb|AAV32227.1| putative disulfide–isomerase [Oryza sativa (japonica cultivar–group)] >gnl|BL_ORD_ID|1742342</td><td>11667.m02311|LOC_Os01g23740|protein protein disulfide–isomerase domain, putative e_value:6.10484e–171</td><td>0.181</td><td>(0.1205 0.6645)</td><td>0.1205</td><td>0.6645</td></tr>
<tr><td>2589</td><td>SET1_O14_CLUSTER_34593</td><td>gi|34915302|ref|NP_919108.1| unknown protein [Oryza sativa (japonica cultivar–group)] >gi|22831317|dbj|BAC16171.1| unknown protein [Oryza sativa (japonica cultivar–group)] e_value:2.57526e–34</td><td>11673.m00140|LOC_Os07g02340|protein hypothetical protein e_value:3.03729e–36</td><td>0.288</td><td>(0.0743 0.2584)</td><td>0.0743</td><td>0.2584</td></tr>
<tr><td>2590</td><td>WT004_C14_CLUSTER_32332</td><td>gi|7489796|pir||T01169 phosphate transport protein, mitochondrial – maize >gnl|BL_ORD_ID|68678 gi|3318613|dbj|BAA31583.1| mitochondrial phosphate transporter [Zea mays] e_value:4.76239e–178</td><td>11668.m05180|LOC_Os02g52860|protein Mitochondrial carrier protein, putative e_value:3.72177e–179</td><td>0.144</td><td>(0.0849 0.5912)</td><td>0.0849</td><td>0.5912</td></tr>
<tr><td>2591</td><td>WT004_H24_CLUSTER_31747</td><td>gi|50941905|ref|XP_480480.1| glyoxalase I [Oryza sativa (japonica cultivar–group)] >gnl|BL_ORD_ID|1602901 gi|51979645|ref|XP_507569.1| PREDICTED OSJNBa0056O06.9–2 gene product [Oryza sativa (japonica cultivar–group)] >gnl|BL_ORD_ID|1602901 gi|519647</td><td>11674.m00866|LOC_Os08g09250|protein lactoylglutathione lyase, putative e_value:1.91409e–158</td><td>0.062</td><td>(0.0430 0.6919)</td><td>0.043</td><td>0.6919</td></tr>
<tr><td>2592</td><td>SET1_P02_CLUSTER_30415</td><td>gi|52548238|gb|AAU82106.1| 20S proteasome beta 4 subunit [Triticum aestivum] e_value:1.84582e–115</td><td>11669.m04911|LOC_Os03g48930|protein 20S proteasome beta 4 subunit e_value:2.63934e–102</td><td>0.105</td><td>(0.0965 0.9144)</td><td>0.0965</td><td>0.9144</td></tr>
<tr><td>2593</td><td>WT013_I18_CLUSTER_31953</td><td>gi|17221690|gb|AAL36459.1| cytochrome b5 reductase isoform II [Zea mays] e_value:2.19864e–147</td><td>11682.m03917|LOC_Os05g40990|protein cytochrome b5 reductase isoform II e_value:5.45035e–147</td><td>0.102</td><td>(0.0480 0.4714)</td><td>0.048</td><td>0.4714</td></tr>
<tr><td>2594</td><td>WT005_O17_CLUSTER_34036</td><td>gi|34898218|ref|NP_910455.1| putative eukaryotic translation initiation factor 2 alpha subunit eIF2 [Oryza sativa (japonica cultivar–group)] >gnl|BL_ORD_ID|1534733 gi|29837180|dbj|BAC75562.1| putative eukaryotic translation initiation factor 2 alpha su</td><td>11669.m01877|LOC_Os03g18510|protein S1 RNA binding domain, putative e_value:1.44048e–179</td><td>0.114</td><td>(0.0402 0.3516)</td><td>0.0402</td><td>0.3516</td></tr>
<tr><td>2595</td><td>SET5_D06_CLUSTER_9901</td><td>gi|31433598|gb|AAP55090.1| putative eukaryotic initiation factor subunit [Oryza sativa (japonica cultivar–group)] >gi|37537002|ref|NP_922803.1| putative eukaryotic initiation factor subunit [Oryza sativa (japonica cultivar–group)] >gi|19224988|gb|AA</td><td>11676.m03873|LOC_Os10g41960|protein putative eukaryotic initiation factor subunit e_value:0</td><td>0.101</td><td>(0.0739 0.7310)</td><td>0.0739</td><td>0.731</td></tr>
<tr><td>2596</td><td>WT003_K02_CLUSTER_34059</td><td>gi|10177098|dbj|BAB10432.1| unnamed protein product [Arabidopsis thaliana] >gnl|BL_ORD_ID|1094296 gi|18958052|gb|AAL79599.1| AT5g52840/MXC20_6 [Arabidopsis thaliana] >gnl|BL_ORD_ID|1094296 gi|18423437|ref|NP_568778.1| NADH–ubiquinone oxidoreductase–</td><td>11669.m02024|LOC_Os03g19890|protein NADH–ubiquinone oxidoreductase e_value:4.55347e–90</td><td>0.161</td><td>(0.0474 0.2944)</td><td>0.0474</td><td>0.2944</td></tr>
<tr><td>2597</td><td>WT005_G22_CLUSTER_22455</td><td>gi|50931251|ref|XP_475153.1| putative nascent polypeptide associated complex alpha chain [Oryza sativa (japonica cultivar–group)] >gnl|BL_ORD_ID|1596358 gi|32352154|dbj|BAC78570.1| nascent polypeptide associated complex alpha chain [Oryza sativa (japon</td><td>11682.m02876|LOC_Os05g31000|protein NAC domain, putative e_value:1.70909e–79</td><td>0.182</td><td>(0.2007 1.1037)</td><td>0.2007</td><td>1.1037</td></tr>
<tr><td>2598</td><td>WT005_P03_CLUSTER_32827</td><td>gi|62733402|gb|AAX95519.1| Putative Glycosyl hydrolase family 1 protein [Oryza sativa (japonica cultivar–group)] >gnl|BL_ORD_ID|2110002 gi|50918079|ref|XP_469436.1| beta–glucosidase (with alternative splicing) [Oryza sativa (japonica cultivar–</td><td>11669.m04987|LOC_Os03g49600|protein beta–glucosidase (with alternative splicing) e_value:0</td><td>0.174</td><td>(0.1053 0.6053)</td><td>0.1053</td><td>0.6053</td></tr>
<tr><td>2599</td><td>WT003_I21_CLUSTER_13405</td><td>gi|34911078|ref|NP_916886.1| peroxiredoxin [Oryza sativa (japonica cultivar–group)] >gnl|BL_ORD_ID|1545298 gi|21104735|dbj|BAB93323.1| putative thioredoxin peroxidase [Oryza sativa (japonica cultivar–group)] >gnl|BL_ORD_ID|1545298</td><td>11667.m04696|LOC_Os01g48420|protein AhpC/TSA family e_value:7.00157e–79</td><td>0.22</td><td>(0.0829 0.3765)</td><td>0.0829</td><td>0.3765</td></tr>
</table>

PDF Complete
Your complimentary use period has ended. Thank you for using PDF Complete.
Click Here to upgrade to Unlimited Pages and Expanded Features

2600	WT004_M19_CLUSTER_11683	gi|50901186|ref|XP_463026.1| putative Mob1-like protein [Oryza sativa (japonica cultivar-group)] >gnl|BL_ORD_ID|1564694 gi|38093736|gb|AAR10852.1| putative Mob1-like protein [Oryza sativa (japonica cultivar-group)] e_value:5.98976e-118	11669.m03752|LOC_Os03g38020|protein putative Mob1-like protein e_value:1.08564e-119	0.037	(0.0163 0.4434)	0.0163	0.4434
2601	WT006_M21_CLUSTER_30148	gi|100841|pir||A38373 ubiquitin-protein ligase (EC 6.3.2.19) E1 - wheat >gnl|BL_ORD_ID|2315794 gi|170780|gb|AAA34308.1| ubiquitin-activating enzyme E1 >gnl|BL_ORD_ID|2315794 gi|136632|sp|P20973|UBA1_WHEAT Ubiquitin-activating enzyme E1 1 e_value:0	11687.m00053|LOC_Os11g01510|protein ubiquitin-activating enzyme e1 2 e_value:0	0.115	(0.0599 0.5224)	0.0599	0.5224
2602	SET2_L13_CLUSTER_16577	gi|50932895|ref|XP_475975.1| putative peroxisomal Ca-dependent solute carrier [Oryza sativa (japonica cultivar-group)] >gi|48843809|gb|AAT47068.1| putative peroxisomal Ca-dependent solute carrier [Oryza sativa (japonica cultivar-group)] e_value:0	11682.m04898|LOC_Os05g50840|protein mitochondrial solute carrier protein homolog - Arabidopsis thaliana e_value:0	0.079	(0.0447 0.5639)	0.0447	0.5639
2603	WT011_J04_CLUSTER_32722	gi|51964144|ref|XP_506855.1| PREDICTED OJ1669_F01.17 gene product [Oryza sativa (japonica cultivar-group)] >gnl|BL_ORD_ID|1577958 gi|50910219|ref|XP_466598.1| NFkB activating protein-like [Oryza sativa (japonica cultivar-group)] >gnl|BL_ORD_ID|15779	11668.m03743|LOC_Os02g39070|protein Got1-like family, putative e_value:3.12075e-69	0.056	(0.0302 0.5424)	0.0302	0.5424
2604	SET3_F12_CLUSTER_23110	gi|34898576|ref|NP_910634.1| hypothetical protein [Oryza sativa (japonica cultivar-group)] >gi|28564794|dbj|BAC57724.1| unknown protein [Oryza sativa (japonica cultivar-group)] e_value:1.86909e-69	11673.m03521|LOC_Os07g36410|protein expressed protein e_value:4.0745e-75	0.384	(0.1910 0.4976)	0.191	0.4976
2605	WT003_D11_CLUSTER_7014	gi|31432670|gb|AAP54275.1| hypothetical protein [Oryza sativa (japonica cultivar-group)] >gnl|BL_ORD_ID|2012161 gi|37535372|ref|NP_921988.1| hypothetical protein [Oryza sativa (japonica cultivar-group)] >gnl|BL_ORD_ID|2012161 gi|13129503|gb|AAK13157	11676.m02973|LOC_Os10g33720|protein expressed protein e_value:0	0.08	(0.0413 0.5154)	0.0413	0.5154
2606	WT003_J17_CLUSTER_31784	gi|50939441|ref|XP_479248.1| putative centromere/microtubule binding protein [Oryza sativa (japonica cultivar-group)] >gnl|BL_ORD_ID|1531500 gi|23237811|dbj|BAC16386.1| putative centromere/microtubule binding protein [Oryza sativa (japonica cultivar-	11673.m04343|LOC_Os07g44190|protein TruB family pseudouridylate synthase (N terminal domain), putative e_value:0	0.106	(0.0616 0.5795)	0.0616	0.5795
2607	WT007__C06_CLUSTER_32973	gi|24030391|gb|AAN41356.1| putative 3-hydroxyisobutyryl-coenzyme A hydrolase [Arabidopsis thaliana] >gnl|BL_ORD_ID|1512379 gi|9759578|dbj|BAB11141.1| 3-hydroxyisobutyryl-coenzyme A hydrolase [Arabidopsis thaliana] >gnl|BL_ORD_ID|1512379 gi|15239206|	11686.m01612|LOC_Os12g16350|protein 3-hydroxyisobutyryl-coenzyme A hydrolase, putative e_value:6.41866e-179	0.205	(0.0943 0.4610)	0.0943	0.461
2608	WT002_I15_CLUSTER_22624	gi|50932771|ref|XP_475913.1| putative malate dehydrogenase [Oryza sativa (japonica cultivar-group)] >gnl|BL_ORD_ID|1599498 gi|52353548|gb|AAU44114.1| putative malate dehydrogenase [Oryza sativa (japonica cultivar-group)] >gnl|BL_ORD_ID|1599498 gi|50	11682.m04796|LOC_Os05g49880|protein malate dehydrogenase, NAD-dependent e_value:1.36297e-169	0.128	(0.0568 0.4449)	0.0568	0.4449
2609	WT004_D21_CLUSTER_34765	gi|34894960|ref|NP_908805.1| putative GTP-binding protein [Oryza sativa (japonica cultivar-group)] >gnl|BL_ORD_ID|1560714 gi|15623923|dbj|BAB67979.1| putative small GTP-binding protein Bsar1a [Oryza sativa (japonica cultivar-group)] >gnl|BL_ORD_ID|1	11686.m03671|LOC_Os12g37360|protein ADP-ribosylation factor family e_value:3.87041e-107	0.024	(0.0113 0.4643)	0.0113	0.4643
2610	WT003_B08_CLUSTER_31991	gi|3288883|dbj|BAA31260.1| SAR DNA binding protein [Oryza sativa] e_value:0	11669.m02341|LOC_Os03g22730|protein SAR DNA binding protein e_value:0	0.086	(0.0750 0.8701)	0.075	0.8701
2611	WT002_N10_CLUSTER_34384	gi|19352190|dbj|BAB85987.1| Acyl-CoA-binding protein [Panax ginseng] e_value:1.65774e-35	11674.m00587|LOC_Os08g06550|protein Acyl CoA binding protein e_value:1.89561e-35	0.141	(0.1328 0.9437)	0.1328	0.9437
2612	SET1_B21_CLUSTER_34590	gi|625337|pir||AYBH acyl carrier protein I precursor - barley >gi|113165|sp|P02902|ACP1_HORVU Acyl carrier protein I, chloroplast precursor (ACP I) >gi|166973|gb|AAA32923.1| acyl carrier protein I precursor >gi|166967|gb|AAA32920.1| acyl carrier	11686.m03413|LOC_Os12g34890|protein acyl carrier protein, putative e_value:5.7648e-55	0.173	(0.0912 0.5257)	0.0912	0.5257
2613	WT004_F04_CLUSTER_7094	gi|22204116|gb|AAM92706.1| putative cytochrome c oxidase subunit [Triticum aestivum] e_value:1.06931e-87	11669.m02777|LOC_Os03g27290|protein cytochrome c oxidase subunit 6b-1 e_value:7.66998e-64	0.143	(0.1235 0.8650)	0.1235	0.865
2614	WT012_I08_CLUSTER_33404	gi|54287494|gb|AAV31238.1| putative 26S proteasome non-ATPase regulatory subunit 14 [Oryza sativa (japonica cultivar-group)] e_value:6.55003e-171	11682.m02855|LOC_Os05g30800|protein Mov34/MPN/PAD-1 family, putative e_value:1.93664e-172	0.023	(0.0114 0.5020)	0.0114	0.502
2615	WT007_G09_CLUSTER_33404	gi|54287494|gb|AAV31238.1| putative 26S proteasome non-ATPase regulatory subunit 14 [Oryza sativa (japonica cultivar-group)] e_value:6.80293e-171	11682.m02855|LOC_Os05g30800|protein Mov34/MPN/PAD-1 family, putative e_value:1.93664e-172	0.026	(0.0114 0.4396)	0.0114	0.4396
2616	WT005_B19_CLUSTER_32577	gi|50939261|ref|XP_479158.1| unknown protein [Oryza sativa (japonica cultivar-group)] >gnl|BL_ORD_ID|1530490 gi|51963544|ref|XP_506466.1| PREDICTED P0616D06.125 gene product [Oryza sativa (japonica cultivar-group)] >gnl|BL_ORD_ID|1530490 gi|23237931	11673.m04149|LOC_Os07g42370|protein hypothetical protein e_value:9.16976e-83	0.298	(0.1335 0.4474)	0.1335	0.4474
2617	WT009_L20_CLUSTER_30917	gi|12407304|dbj|BAB21393.1| IDI2 [Hordeum vulgare subsp. vulgare] e_value:3.18253e-179	11687.m01054|LOC_Os11g11050|protein IDI2 e_value:2.13691e-	0.103	(0.0726 0.7021)	0.0726	0.7021
2618	WT006_A03_CLUSTER_32429	gi|50909553|ref|XP_466265.1| putative gamma hydroxybutyrate dehydrogenase [Oryza sativa (japonica cultivar-group)] >gnl|BL_ORD_ID|1576379 gi|51979248|ref|XP_507487.1| PREDICTED OJ1712_E04.5 gene product [Oryza sativa (japonica cultivar-group)] >gnl|	11668.m03371|LOC_Os02g35500|protein NAD binding domain of 6-phosphogluconate dehydrogenase, putative e_value:9.83045e-155	0.06	(0.0262 0.4341)	0.0262	0.4341
2619	WT002_O08_CLUSTER_31410	gi|50948465|ref|XP_483760.1| unknown protein [Oryza sativa (japonica cultivar-group)] >gnl|BL_ORD_ID|1612261 gi|45736099|dbj|BAD13130.1| unknown protein [Oryza sativa (japonica cultivar-group)] e_value:1.20458e-101	11674.m04490|LOC_Os08g44440|protein expressed protein e_value:2.0505e-103	0.128	(0.0870 0.6815)	0.087	0.6815
2620	WT002_N09_CLUSTER_31983	gi|27447203|gb|AAL82576.1| putative sterol 4-alpha-methyl-oxidase [Zea mays] e_value:7.18149e-147	11687.m04517|LOC_Os11g48020|protein sterol 4-alpha-methyl-oxidase, putative e_value:7.04037e-149	0.08	(0.0428 0.5324)	0.0428	0.5324
2621	WT007_M21_CLUSTER_31588	gi|51535367|dbj|BAD37238.1| putative poly(A) binding protein II [Oryza sativa (japonica cultivar-group)] e_value:4.88104e-100	11680.m01132|LOC_Os06g11620|protein RNA recognition motif. (a.k.a. RRM, RBD, or RNP domain), putative e_value:9.67128e-	0.103	(0.0665 0.6475)	0.0665	0.6475
2622	WT007_F15_CLUSTER_31588	gi|51535367|dbj|BAD37238.1| putative poly(A) binding protein II [Oryza sativa (japonica cultivar-group)] e_value:4.6924e-100	11680.m01132|LOC_Os06g11620|protein RNA recognition motif. (a.k.a. RRM, RBD, or RNP domain), putative e_value:9.67128e-	0.105	(0.0665 0.6306)	0.0665	0.6306
2623	SET5_O12_CLUSTER_34666	gi|34894688|ref|NP_908669.1| P0426D06.10 [Oryza sativa (japonica cultivar-group)] e_value:0	11667.m02115|LOC_Os01g21850|protein Sec23/Sec24 trunk domain, putative e_value:0	0.18	(0.0964 0.5340)	0.0964	0.534
2624	WT003_D15_CLUSTER_34289	gi|62701922|gb|AAX92995.1| hypothetical protein LOC_Os11g06760 [Oryza sativa (japonica cultivar-group)] e_value:3.91236e-117	11687.m00610|LOC_Os11g06760|protein hypothetical protein e_value:4.26804e-117	0.256	(0.1596 0.6235)	0.1596	0.6235
2625	WT004_E10_CLUSTER_31969	gi|22135471|gb|AAM93216.1| histone H1-like protein HON101 [Zea mays] e_value:1.92665e-70	11670.m01636|LOC_Os04g18090|protein linker histone H1 and H5 family, putative e_value:6.77108e-69	0.278	(0.1169 0.4213)	0.1169	0.4213
2626	SET5_I09_CLUSTER_29372	gi|23296760|gb|AAN13164.1| putative shaggy kinase alpha [Arabidopsis thaliana] >gi|15028149|gb|AAK76698.1| putative shaggy kinase alpha [Arabidopsis thaliana] >gi|16226768|gb|AAL16257.1| AT5g26750/F2P16_10 [Arabidopsis thaliana] >gi|18421039|ref|	11667.m01479|LOC_Os01g14860|protein Protein kinase domain, putative e_value:0	0.045	(0.0898 1.9760)	0.0898	1.976
2627	WT005_M24_CLUSTER_34811	gi|50725468|dbj|BAD32939.1| putative beta-ketoacyl-CoA synthase [Oryza sativa (japonica cultivar-group)] e_value:0	11680.m03875|LOC_Os06g39750|protein fatty acid elongase, putative e_value:0	0.167	(0.1023 0.6112)	0.1023	0.6112
2628	SET2_N24_CLUSTER_34570	gi|50945999|ref|XP_482527.1| unknown protein [Oryza sativa (japonica cultivar-group)] >gi|38175483|dbj|BAD01180.1| unknown protein [Oryza sativa (japonica cultivar-group)] >gi|37805925|dbj|BAC99342.1| unknown protein [Oryza sativa (japonica cultivar	11674.m03310|LOC_Os08g33460|protein hypothetical protein e_value:2.17155e-52	0.145	(0.0224 0.1540)	0.0224	0.154
2629	WT002_A09_CLUSTER_18675	gi|32401384|gb|AAP80861.1| cyclophilin [Triticum aestivum] >gnl|BL_ORD_ID|2070334 gi|32351008|gb|AAP76508.1| cyclophilin [Triticum aestivum] e_value:3.72396e-125	11680.m04912|LOC_Os06g49480|protein peptidylprolyl isomerase (EC 5.2.1.8) ROC7 [similarity] - Arabidopsis thaliana	0.15	(0.0763 0.5104)	0.0763	0.5104
2630	SET5_J19_CLUSTER_13759	gi|12621070|gb|AAG54079.1| nuclear cap-binding protein CBP80 [Oryza sativa subsp. japonica] e_value:0	11669.m02323|LOC_Os03g22570|protein MIF4G domain, putative e_value:0	0.133	(0.0619 0.4639)	0.0619	0.4639
2631	WT012_F09_CLUSTER_28805	gi|558652|emb|CAA57646.1| Voltage dependent anion channel (VDAC) [Triticum aestivum] >gnl|BL_ORD_ID|2360875 gi|2130109|pir||S59547 porin VDAC3 - wheat e_value:5.41534e-152	11667.m03901|LOC_Os01g40570|protein Eukaryotic porin e_value:3.02416e-142	0.064	(0.0400 0.6273)	0.04	0.6273
2632	SET5_O09_CLUSTER_18442	gi|31432698|gb|AAP54296.1| unknown protein [Oryza sativa (japonica cultivar-group)] >gi|37535414|ref|NP_922009.1| unknown protein [Oryza sativa (japonica cultivar-group)] >gi|13384376|gb|AAK21344.1| unknown protein [Oryza sativa (japonica cultivar-g	11676.m02995|LOC_Os10g33930|protein expressed protein e_value:0	0.13	(0.0573 0.4403)	0.0573	0.4403
2633	SET3_O17_CLUSTER_30378	No hits found	11669.m01665|LOC_Os03g16580|protein hypothetical protein e_value:3.93947e-77	0.306	(0.2411 0.7868)	0.2411	0.7868
2634	SET5_L22_CLUSTER_9288	gi|15420575|gb|AAK97388.1| succinate dehydrogenase subunit 4 [Oryza sativa] e_value:1.45464e-86	11667.m07116|LOC_Os01g70980|protein expressed protein e_value:1.37082e-83	0.293	(0.1520 0.5182)	0.152	0.5182
2635	SET1_J02_CLUSTER_27037	gi|28394053|gb|AAO42434.1| unknown protein [Arabidopsis thaliana] >gi|27754660|gb|AAO22774.1| unknown protein [Arabidopsis thaliana] >gi|30687213|ref|NP_194371.2| expressed protein [Arabidopsis thaliana] e_value:1.23084e-37	11669.m02130|LOC_Os03g20860|protein expressed protein e_value:3.54763e-94	0.204	(0.0973 0.4776)	0.0973	0.4776
2636	WT002_B05_CLUSTER_23133	gi|18481707|gb|AAL73529.1| putative beta-1,3-glucanase [Sorghum bicolor] e_value:2.59747e-38	11674.m00327|LOC_Os08g04140|protein expressed protein e_value:8.83899e-40	0.383	(0.2658 0.6941)	0.2658	0.6941
2637	SET4_E11_CLUSTER_22388	gi|34899754|ref|NP_911223.1| putative secretory carrier membrane protein [Oryza sativa (japonica cultivar-group)] >gi|51963480|ref|XP_506412.1| PREDICTED OJ1112_E08.118 gene product [Oryza sativa (japonica cultivar-group)] >gi|22830947|dbj|BAC15812.	11673.m03660|LOC_Os07g37740|protein SCAMP family, putative e_value:3.01655e-157	0.062	(0.0436 0.7028)	0.0436	0.7028
2638	WT007_N08_CLUSTER_34763	gi|32489187|emb|CAE04372.1| OSJNBa0027G07.8 [Oryza sativa (japonica cultivar-group)] >gnl|BL_ORD_ID|1524421 gi|50924674|ref|XP_472692.1| OSJNBa0027G07.8 [Oryza sativa (japonica cultivar-group)] e_value:0	11670.m03600|LOC_Os04g38020|protein Sugar transporter e_value:0	0.285	(0.0689 0.2417)	0.0689	0.2417
2639	WT002_G08_CLUSTER_1130	gi|50937053|ref|XP_478054.1| plectin-like protein [Oryza sativa (japonica cultivar-group)] >gnl|BL_ORD_ID|1526727 gi|34395179|dbj|BAC83565.1| plectin-like protein [Oryza sativa (japonica cultivar-group)] e_value:0	11673.m02736|LOC_Os07g28940|protein hypothetical protein e_value:0	0.272	(0.1782 0.6542)	0.1782	0.6542
2640	WT011_K18_CLUSTER_14389	gi|50942921|ref|XP_480988.1| putative ADP-ribosylation factor 3 [Oryza sativa (japonica cultivar-group)] >gnl|BL_ORD_ID|1603386 gi|40253906|dbj|BAD05839.1| putative ADP-ribosylation factor 3 [Oryza sativa (japonica cultivar-group)] >gnl|BL_ORD_ID|16	11674.m01467|LOC_Os08g15040|protein ADP-ribosylation factor family e_value:1.90955e-89	0.094	(0.0292 0.3116)	0.0292	0.3116
2641	SET2_P24_CLUSTER_20320	gi|34913982|ref|NP_918338.1| peptide deformylase-like protein [Oryza sativa (japonica cultivar-group)] e_value:8.49769e-96	11667.m03580|LOC_Os01g37510|protein polypeptide deformylase, putative e_value:2.62928e-113	0.15	(0.1057 0.7050)	0.1057	0.705
2642	WT006_N14_CLUSTER_5956	gi|55773698|dbj|BAD72281.1| putative seed imbibition protein [Oryza sativa (japonica cultivar-group)] e_value:0	11680.m00707|LOC_Os06g07600|protein imbibition protein homolog, putative e_value:0	0.287	(0.1405 0.4890)	0.1405	0.489

PDF Complete
Your complimentary use period has ended. Thank you for using PDF Complete.
Click Here to upgrade to Unlimited Pages and Expanded Features

2643	WT006_M03_CLUSTER_23350	gi|30089750|gb|AAP20854.1| putative stearoyl-acyl-carrier protein desaturase [Oryza sativa (japonica cultivar-group)] >gnl|BL_ORD_ID|2040852 gi|50916683|ref|XP_468738.1| putative stearoyl-acyl-carrier protein desaturase [Oryza sativa (japonica cultivar	11669.m03121|LOC_Os03g30950|protein putative stearoyl-acyl-carrier protein desaturase e_value:3.96223e-139	0.432	(0.2522 0.5840)	0.2522	0.584
2644	WT005_N03_CLUSTER_16271	gi|50933547|ref|XP_476301.1| putative phi-1 [Oryza sativa (japonica cultivar-group)] >gnl|BL_ORD_ID|1752530 gi|28190675|gb|AAO33153.1| unknown [Oryza sativa (japonica cultivar-group)] >gnl|BL_ORD_ID|1752530 gi|11875173|dbj|BAB19386.1|	11680.m00345|LOC_Os06g04250|protein Phosphate-induced protein 1 conserved region e_value:3.52503e-173	0.294	(0.1087 0.3694)	0.1087	0.3694
2645	WT005_L16_CLUSTER_15792	gi|4775576|emb|CAB42554.1| MUS1 protein [Zea mays] >gnl|BL_ORD_ID|1321360 gi|11386957|sp|Q9XGC9|MSH2_MAIZE DNA mismatch repair protein MSH2 (MUS1) e_value:0	11682.m01824|LOC_Os05g19270|protein Similar to dna mismatch repair protein msh2 (mus1) e_value:0	0.157	(0.0641 0.4072)	0.0641	0.4072
2646	WT004_N18_CLUSTER_18333	gi|50910459|ref|XP_466718.1| unknown protein [Oryza sativa (japonica cultivar-group)] >gnl|BL_ORD_ID|1578181 gi|47497655|dbj|BAD19723.1| unknown protein [Oryza sativa (japonica cultivar-group)] e_value:1.90324e-64	11668.m03850|LOC_Os02g40040|protein expressed protein e_value:2.8358e-66	0.173	(0.0729 0.4206)	0.0729	0.4206
2647	WT004_J04_CLUSTER_18104	gi|50872473|gb|AAT85073.1| hypothetical protein [Oryza sativa (japonica cultivar-group)] e_value:1.62497e-51	11669.m04830|LOC_Os03g48220|protein expressed protein e_value:4.66636e-53	0.934	(0.4484 0.4803)	0.4484	0.4803
2648	WT004_A12_CLUSTER_22614	gi|50906483|ref|XP_464730.1| high molecular weight glutenin subunit x-like protein [Oryza sativa (japonica cultivar-group)] >gnl|BL_ORD_ID|1566926 gi|46805645|dbj|BAD17064.1| high molecular weight glutenin subunit x-like protein [Oryza sativa (japonica	11668.m01209|LOC_Os02g13130|protein KH domain, putative e_value:0	0.207	(0.1101 0.5313)	0.1101	0.5313
2649	WT003_H21_CLUSTER_1948	gi|50910207|ref|XP_466592.1| putative PITSLRE alpha 2-1 [Oryza sativa (japonica cultivar-group)] >gnl|BL_ORD_ID|1577952 gi|47848303|dbj|BAD22167.1| putative PITSLRE alpha 2-1 [Oryza sativa (japonica cultivar-group)] >gnl|BL_ORD_ID|1577952 gi|4749729	11668.m03737|LOC_Os02g39010|protein Protein kinase domain, putative e_value:0	0.286	(0.1685 0.5897)	0.1685	0.5897
2650	WT003_F23_CLUSTER_2400	gi|55775304|ref|XP_550578.1| putative RNA-binding protein [Oryza sativa (japonica cultivar-group)] >gnl|BL_ORD_ID|1752835 gi|25553563|dbj|BAC24834.1| putative RNA-binding protein [Oryza sativa (japonica cultivar-group)] >gnl|BL_ORD_ID|1752835 gi|552	11680.m00281|LOC_Os06g03690|protein hypothetical protein e_value:2.73802e-97	0.303	(0.1557 0.5131)	0.1557	0.5131
2651	WT003_B16_CLUSTER_1241	gi|34895664|ref|NP_909181.1| putative aspartic proteinase nepenthesin I [Oryza sativa (japonica cultivar-group)] >gnl|BL_ORD_ID|1751615 gi|12328547|dbj|BAB21205.1| putative aspartic proteinase nepenthesin I [Oryza sativa (japonica cultivar-group)]	11667.m00390|LOC_Os01g04710|protein Eukaryotic aspartyl protease e_value:0	0.302	(0.1286 0.4262)	0.1286	0.4262
2652	WT003_A06_CLUSTER_18906	gi|34914848|ref|NP_918771.1| putative cyclin homolog [Oryza sativa (japonica cultivar-group)] >gnl|BL_ORD_ID|1560419 gi|13365979|dbj|BAB39257.1| putative ania-6a type cyclin [Oryza sativa (japonica cultivar-group)] e_value:0	11667.m02688|LOC_Os01g27940|protein Cyclin, N-terminal domain, putative e_value:0	0.158	(0.0914 0.5789)	0.0914	0.5789
2653	WT002_I20_CLUSTER_23211	gi|38345246|emb|CAD41090.2| OSJNBb0011N17.7 [Oryza sativa (japonica cultivar-group)] >gnl|BL_ORD_ID|1522073 gi|50925219|ref|XP_472911.1| OSJNBb0011N17.7 [Oryza sativa (japonica cultivar-group)] e_value:0	11670.m03873|LOC_Os04g40510|protein Cellulase (glycosyl hydrolase family 5), putative e_value:0	0.529	(0.1384 0.2618)	0.1384	0.2618
2654	WT002_A23_CLUSTER_22443	gi|50939567|ref|XP_479311.1| unknown protein [Oryza sativa (japonica cultivar-group)] >gnl|BL_ORD_ID|1532267 gi|33146645|dbj|BAC79975.1| unknown protein [Oryza sativa (japonica cultivar-group)] >gnl|BL_ORD_ID|1532267 gi|50509935|dbj|BAD30256.1| unkn	11669.m02528|LOC_Os03g24940|protein hypothetical protein e_value:0	0.157	(0.0774 0.4936)	0.0774	0.4936
2655	SET6_A04_CLUSTER_23350	gi|30089750|gb|AAP20854.1| putative stearoyl-acyl-carrier protein desaturase [Oryza sativa (japonica cultivar-group)] >gi|50916683|ref|XP_468738.1| putative stearoyl-acyl-carrier protein desaturase [Oryza sativa (japonica cultivar-group)] e_value:5.779	11669.m03121|LOC_Os03g30950|protein putative stearoyl-acyl-carrier protein desaturase e_value:1.24722e-135	0.593	(0.3009 0.5076)	0.3009	0.5076
2656	SET5_A24_CLUSTER_19062	gi|50899796|ref|XP_450686.1| putative SenC [Oryza sativa (japonica cultivar-group)] >gi|49389106|dbj|BAD26385.1| putative SenC [Oryza sativa (japonica cultivar-group)] e_value:4.50371e-124	11681.m01841|LOC_Os09g20430|protein SCO1/SenC, putative e_value:9.25342e-126	0.294	(0.1137 0.3875)	0.1137	0.3875
2657	SET4_O23_CLUSTER_14959	gi|50937987|ref|XP_478521.1| putative ATP/GTP-binding protein [Oryza sativa (japonica cultivar-group)] >gi|27261033|dbj|BAC45149.1| putative ATP/GTP-binding protein [Oryza sativa (japonica cultivar-group)] e_value:6.85146e-171	11673.m03332|LOC_Os07g34640|protein hypothetical protein e_value:0	0.328	(0.1541 0.4696)	0.1541	0.4696
2658	SET4_G16_CLUSTER_27693	gi|34903234|ref|NP_912964.1| unnamed protein product [Oryza sativa (japonica cultivar-group)] >gi|6815106|dbj|BAA90392.1| putative cyclin-selective ubiquitin carrier protein E2-C [Oryza sativa (japonica cultivar-group)] e_value:2.19193e-85	11667.m01673|LOC_Os01g16650|protein Ubiquitin-conjugating enzyme, putative e_value:4.17354e-87	0.215	(0.0813 0.3781)	0.0813	0.3781
2659	SET3_M04_CLUSTER_25607	gi|50902262|ref|XP_463564.1| putative ATP-dependent Clp protease regulatory subunit CLPX [Oryza sativa (japonica cultivar-group)] e_value:0	11667.m06664|LOC_Os01g66330|protein ATP-dependent Clp protease, ATP-binding subunit ClpX, putative e_value:0	0.194	(0.1052 0.5409)	0.1052	0.5409
2660	SET3_L11_CLUSTER_9380	gi|50921391|ref|XP_471056.1| OSJNBa0020P07.1 [Oryza sativa (japonica cultivar-group)] >gi|32451277|emb|CAE01284.1| OSJNBa0020P07.1 [Oryza sativa (japonica cultivar-group)] e_value:0	11670.m00182|LOC_Os04g02780|protein Amidase e_value:0	0.15	(0.1414 0.9406)	0.1414	0.9406
2661	SET1_K21_CLUSTER_1440	gi|1617206|emb|CAA96570.1| CP12 [Pisum sativum] >gi|7488793|pir||T06562 CP12 protein precursor, chloroplast - garden pea e_value:1.07353e-27	11669.m01967|LOC_Os03g19380|protein nonenzymatic protein CP12 - Chlamydomonas reinhardtii (fragment) e_value:3.11426e-	0.547	(0.1560 0.2854)	0.156	0.2854
2662	WT013_A13_CLUSTER_6458	gi|53749343|gb|AAU90202.1| putative transcriptional regulator [Oryza sativa (japonica cultivar-group)] e_value:0	11682.m00465|LOC_Os05g05230|protein transcriptional regulator, putative e_value:0	0.149	(0.0930 0.6258)	0.093	0.6258
2663	WT012_C10_CLUSTER_21056	gi|34902296|ref|NP_912494.1| Unknown protein [Oryza sativa (japonica cultivar-group)] >gnl|BL_ORD_ID|1588599 gi|24308625|gb|AAN52748.1| Unknown protein [Oryza sativa (japonica cultivar-group)] e_value:0	11669.m01206|LOC_Os03g12450|protein Nup133 nucleoporin, putative e_value:0	0.197	(0.0965 0.4911)	0.0965	0.4911
2664	WT011_N13_CLUSTER_17726	No hits found	11687.m03013|LOC_Os11g32720|protein Transposable element protein, putative e_value:2.11164e-79	0.764	(0.3990 0.5220)	0.399	0.522
2665	WT011_L02_CLUSTER_23924	gi|40363457|dbj|BAD06218.1| cation/proton exchanger 1a [Oryza sativa (japonica cultivar-group)] >gnl|BL_ORD_ID|1044846 gi|57900413|dbj|BAD87649.1| cation/proton exchanger 1a [Oryza sativa (japonica cultivar-group)] >gnl|BL_ORD_ID|1044846 gi|57899253	11667.m03599|LOC_Os01g37690|protein calcium/proton exchanger, putative e_value:0	0.154	(0.0954 0.6205)	0.0954	0.6205
2666	WT010_E02_CLUSTER_12089	gi|12322909|gb|AAG51447.1| hypothetical protein; 89863-88075 [Arabidopsis thaliana] >gnl|BL_ORD_ID|302998 gi|15229797|ref|NP_187764.1| expressed protein [Arabidopsis thaliana] e_value:2.57249e-111	11687.m00426|LOC_Os11g05080|protein expressed protein e_value:3.28066e-91		(0.5846 -1.0000)	0.5846	-1
2667	WT010_C23_CLUSTER_21617	gi|50872418|gb|AAT85018.1| expressed protein [Oryza sativa (japonica cultivar-group)] e_value:1.68331e-18	11669.m01100|LOC_Os03g11490|protein expressed protein e_value:1.83243e-159	0.569	(0.4686 0.8230)	0.4686	0.823
2668	WT009_F18_CLUSTER_10458	gi|9294582|dbj|BAB02863.1| transcription factor-like protein [Arabidopsis thaliana] >gnl|BL_ORD_ID|1096533 gi|15233006|ref|NP_189488.1| myb family transcription factor (MYB35) [Arabidopsis thaliana] >gnl|BL_ORD_ID|1096533	11669.m01873|LOC_Os03g18480|protein typical P-type R2R3 Myb protein e_value:1.90706e-127	0.249	(0.1062 0.4258)	0.1062	0.4258
2669	WT009_E23_CLUSTER_19867	gi|55296762|dbj|BAD67954.1| putative glutamine cyclotransferase precursor [Oryza sativa (japonica cultivar-group)] e_value:2.86375e-141	11680.m00042|LOC_Os06g01410|protein Glutamine cyclotransferase e_value:8.07859e-143	0.257	(0.1527 0.5943)	0.1527	0.5943
2670	WT009_E05_CLUSTER_225	gi|7270954|emb|CAB80633.1| putative protein [Arabidopsis thaliana] >gnl|BL_ORD_ID|1461473 gi|3080439|emb|CAA18756.1| putative protein [Arabidopsis thaliana] >gnl|BL_ORD_ID|1461473 gi|15235984|ref|NP_195680.1| heavy-metal-associated domain-containing	11669.m02708|LOC_Os03g26650|protein Heavy-metal-associated domain, putative e_value:2.12283e-51	0.73	(0.2349 0.3220)	0.2349	0.322
2671	WT008_H20_CLUSTER_31040	gi|34906224|ref|NP_914459.1| P0666G04.18 [Oryza sativa (japonica cultivar-group)] e_value:3.98362e-108	11667.m00817|LOC_Os01g08680|protein expressed protein e_value:4.00014e-106	0.385	(0.2848 0.7398)	0.2848	0.7398
2672	WT008_C06_CLUSTER_12303	gi|53793291|dbj|BAD54513.1| putative IAA-amino acid hydrolase [Oryza sativa (japonica cultivar-group)] e_value:0	11680.m04706|LOC_Os06g47620|protein IAA-amino acid conjugate hydrolase-like protein e_value:0	0.415	(0.1428 0.3440)	0.1428	0.344
2673	WT007_K03_CLUSTER_18857	gi|50920057|ref|XP_470389.1| putative GDSL-like lipase/acylhydrolase [Oryza sativa (japonica cultivar-group)] >gnl|BL_ORD_ID|1535230 gi|41469650|gb|AAS07373.1| putative GDSL-like lipase/acylhydrolase [Oryza sativa (japonica cultivar-group)] e_value:5	11669.m06420|LOC_Os03g62740|protein putative GDSL-like lipase/acylhydrolase e_value:1.63003e-161	0.527	(0.1580 0.2996)	0.158	0.2996
2674	WT007_G08_CLUSTER_18840	gi|51091387|dbj|BAD36120.1| putative ATP-binding cassette transporter1 [Oryza sativa (japonica cultivar-group)] >gnl|BL_ORD_ID|239323 gi|51090351|dbj|BAD35612.1| putative ATP-binding cassette transporter1 [Oryza sativa (japonica cultivar-	11680.m03957|LOC_Os06g40550|protein Similar to ABC transporter-like protein e_value:0	0.158	(0.0901 0.5702)	0.0901	0.5702
2675	WT007_A22_CLUSTER_5071	gi|55769741|ref|XP_549860.1| putative prolyl endopeptidase [Oryza sativa (japonica cultivar-group)] >gnl|BL_ORD_ID|1753564 gi|52076202|dbj|BAD44856.1| putative prolyl endopeptidase [Oryza sativa (japonica cultivar-group)] e_value:0	11667.m00087|LOC_Os01g01830|protein prolyl oligopeptidase family, putative e_value:0	0.136	(0.0755 0.5566)	0.0755	0.5566
2676	WT005_I17_CLUSTER_3391	gi|34902230|ref|NP_912461.1| Putative peroxidase [Oryza sativa (japonica cultivar-group)] >gnl|BL_ORD_ID|1584269 gi|21426120|gb|AAM52317.1| Putative peroxidase [Oryza sativa (japonica cultivar-group)] >gnl|BL_ORD_ID|1584269 gi|55700933|tpe|CAH69275.	11669.m00222|LOC_Os03g02920|protein Peroxidase, putative e_value:5.16749e-142	0.489	(0.1628 0.3330)	0.1628	0.333
2677	WT002_B19_CLUSTER_20743	gi|62898531|dbj|BAD97365.1| bZIP transcription factor [Triticum aestivum] e_value:4.36893e-78	11682.m00319|LOC_Os05g03860|protein oxidase lip19 - rice e_value:4.96076e-62	0.323	(0.1066 0.3307)	0.1066	0.3307
2678	WT002_B24_CLUSTER_20447	gi|50929129|ref|XP_474092.1| OSJNBa0033G05.15 [Oryza sativa (japonica cultivar-group)] >gnl|BL_ORD_ID|1594450 gi|38344891|emb|CAE01544.2| OSJNBa0033G05.15 [Oryza sativa (japonica cultivar-group)] e_value:2.93203e-81	11670.m05425|LOC_Os04g55160|protein Similar to extensin-like protein e_value:5.08801e-87	0.38	(0.2583 0.6796)	0.2583	0.6796
2679	SET6_I09_CLUSTER_20447	gi|50929129|ref|XP_474092.1| OSJNBa0033G05.15 [Oryza sativa (japonica cultivar-group)] >gi|38344891|emb|CAE01544.2| OSJNBa0033G05.15 [Oryza sativa (japonica cultivar-group)] e_value:2.95865e-81	11670.m05425|LOC_Os04g55160|protein Similar to extensin-like protein e_value:5.08801e-87	0.38	(0.2583 0.6796)	0.2583	0.6796
2680	WT012_H01_CLUSTER_13772	gi|34898454|ref|NP_910573.1| EST AU082557(R0845) corresponds to a region of the predicted gene.~Similar to Arabidopsis thaliana cystathionine beta-lyase precursor (P53780) [Oryza sativa (japonica cultivar-group)] >gnl|BL_ORD_ID|1743602 gi|8096317|dbj|B	11680.m00734|LOC_Os06g07860|protein cystathionine beta-lyase, putative e_value:0	0.199	(0.0935 0.4700)	0.0935	0.47
2681	WT008_C07_CLUSTER_31841	gi|50935223|ref|XP_477139.1| unknown protein [Oryza sativa (japonica cultivar-group)] >gnl|BL_ORD_ID|1522793 gi|50508901|dbj|BAD31697.1| unknown protein [Oryza sativa (japonica cultivar-group)] >gnl|BL_ORD_ID|1522793 gi|34393295|dbj|BAC83224.1| unkn	11673.m01057|LOC_Os07g11060|protein expressed protein e_value:1.09027e-160	0.164	(0.1160 0.7082)	0.116	0.7082
2682	WT008_K11_CLUSTER_16010	gi|50931407|ref|XP_475231.1| putative microtubule-associated protein [Oryza sativa (japonica cultivar-group)] >gnl|BL_ORD_ID|1595652 gi|49328159|gb|AAT58855.1| putative microtubule-associated protein [Oryza sativa (japonica cultivar-group)] e_value:0	11682.m03186|LOC_Os05g33890|protein microtubule-associated protein MAP65-1a e_value:0	0.222	(0.1156 0.5208)	0.1156	0.5208
2683	SET1_M14_CLUSTER_32342	gi|50899674|ref|XP_450625.1| putative adhesion of calyx edges protein ACE [Oryza sativa (japonica cultivar-group)] >gi|50726198|dbj|BAD33717.1| putative adhesion of calyx edges protein ACE [Oryza sativa (japonica cultivar-group)]	11681.m01790|LOC_Os09g19930|protein GMC oxidoreductase, putative e_value:0	0.191	(0.0866 0.4545)	0.0866	0.4545
2684	SET6_M23_CLUSTER_7003	gi|34906526|ref|NP_914610.1| P0432C03.20 [Oryza sativa (japonica cultivar-group)] >gi|18844963|dbj|BAB85431.1| purple acid phosphatase-like [Oryza sativa (japonica cultivar-group)] e_value:4.58963e-170	11667.m07162|LOC_Os01g71420|protein Ser/Thr protein phosphatase family protein, putative e_value:1.30105e-171	0.151	(0.1341 0.8903)	0.1341	0.8903
2685	SET3_B01_CLUSTER_34233	gi|37999990|gb|AAR07077.1| unknown protein [Oryza sativa (japonica cultivar-group)] >gi|50918491|ref|XP_469642.1| unknown protein [Oryza sativa (japonica cultivar-group)] >gi|29788863|gb|AAP03409.1| unknown protein [Oryza sativa (japonica cultivar-g	11669.m05330|LOC_Os03g52730|protein expressed protein e_value:4.99234e-84	0.127	(0.0707 0.5573)	0.0707	0.5573

PDF Complete
Your complimentary use period has ended. Thank you for using PDF Complete.
Click Here to upgrade to Unlimited Pages and Expanded Features

<table>
<tr><th></th><th></th><th></th><th></th><th></th><th></th><th></th><th></th></tr>
<tr><td>2686</td><td>WT004_I05_CLUSTER_13939</td><td>gi|50941329|ref|XP_480192.1| NAC2 protein-like [Oryza sativa (japonica cultivar-group)] >gnl|BL_ORD_ID|1601994 gi|37806148|dbj|BAC99653.1| NAC2 protein-like [Oryza sativa (japonica cultivar-group)] e_value:0</td><td>11674.m00542|LOC_Os08g06140|protein No apical meristem (NAM) protein, putative e_value:0</td><td>0.387</td><td>(0.2486 0.6417)</td><td>0.2486</td><td>0.6417</td></tr>
<tr><td>2687</td><td>WT003_I19_CLUSTER_4158</td><td>gi|50920117|ref|XP_470419.1| putative proteasome regulatory non-ATPase subunit [Oryza sativa (japonica cultivar-group)] >gnl|BL_ORD_ID|1535147 gi|27573351|gb|AAO20069.1| putative proteasome regulatory non-ATPase subunit [Oryza sativa (japonica</td><td>11669.m06503|LOC_Os03g63430|protein putative proteasome regulatory non-ATPase subunit e_value:0</td><td>0.08</td><td>(0.0466 0.5816)</td><td>0.0466</td><td>0.5816</td></tr>
<tr><td>2688</td><td>WT004_O18_CLUSTER_32542</td><td>gi|40644754|emb|CAE53885.1| putative SKP1 protein [Triticum aestivum] e_value:4.59221e-94</td><td>11687.m02425|LOC_Os11g26910|protein Skp1 family, dimerisation domain, putative e_value:2.53776e-80</td><td>0.169</td><td>(0.0599 0.3545)</td><td>0.0599</td><td>0.3545</td></tr>
<tr><td>2689</td><td>WT008_A10_CLUSTER_3581</td><td>gi|520943|emb|CAA56552.1| structural protein [Hordeum vulgare subsp. vulgare] >gnl|BL_ORD_ID|991822 gi|629788|pir||S47036 structural protein - barley e_value:3.71354e-54</td><td>11667.m01476|LOC_Os01g14850|protein expressed protein e_value:4.79062e-33</td><td>0.403</td><td>(0.1671 0.4151)</td><td>0.1671</td><td>0.4151</td></tr>
<tr><td>2690</td><td>WT002_J19_CLUSTER_10925</td><td>gi|37991911|gb|AAR06357.1| putative transthyretin, having alternative splicing products [Oryza sativa (japonica cultivar-group)] >gnl|BL_ORD_ID|2263185 gi|50920893|ref|XP_470807.1| putative transthyretin, having alternative splicing products [Oryza sat</td><td>11669.m02782|LOC_Os03g27320|protein putative transthyretin, having alternative splicing products e_value:1.00596e-161</td><td>0.172</td><td>(0.0791 0.4594)</td><td>0.0791</td><td>0.4594</td></tr>
<tr><td>2691</td><td>WT004_J16_CLUSTER_32869</td><td>gi|31193919|gb|AAP44754.1| putative rubisco subunit binding-protein alpha subunit precursor (60 kDa chaperonin alpha subunit) [Oryza sativa (japonica cultivar-group)] >gnl|BL_ORD_ID|1917598 gi|50920285|ref|XP_470503.1| putative rubisco subunit binding-</td><td>11669.m06594|LOC_Os03g64210|protein putative rubisco subunit binding-protein alpha subunit precursor (60 kDa chaperonin alpha subunit) e_value:0</td><td>0.11</td><td>(0.0640 0.5798)</td><td>0.064</td><td>0.5798</td></tr>
<tr><td>2692</td><td>SET4_P10_CLUSTER_5742</td><td>gi|50933333|ref|XP_476194.1| unknown protein [Oryza sativa (japonica cultivar-group)] >gi|46981310|gb|AAT07628.1| unknown protein [Oryza sativa (japonica cultivar-group)] >gi|46981242|gb|AAT07560.1| unknown protein [Oryza sativa (japonica cultivar-g</td><td>11682.m01234|LOC_Os05g13440|protein expressed protein e_value:9.84204e-113</td><td>0.109</td><td>(0.0448 0.4113)</td><td>0.0448</td><td>0.4113</td></tr>
<tr><td>2693</td><td>WT013_E12_CLUSTER_33825</td><td>gi|50946339|ref|XP_482697.1| putative pectinesterase [Oryza sativa (japonica cultivar-group)] >gnl|BL_ORD_ID|1608522 gi|42407616|dbj|BAD08731.1| putative pectinesterase [Oryza sativa (japonica cultivar-group)] e_value:0</td><td>11674.m03458|LOC_Os08g34900|protein Pectinesterase, putative e_value:0</td><td>0.396</td><td>(0.1576 0.3979)</td><td>0.1576</td><td>0.3979</td></tr>
<tr><td>2694</td><td>WT012_D21_CLUSTER_27534</td><td>gi|50939433|ref|XP_479244.1| hypothetical protein [Oryza sativa (japonica cultivar-group)] >gnl|BL_ORD_ID|1531471 gi|33146903|dbj|BAC79902.1| hypothetical protein [Oryza sativa (japonica cultivar-group)] e_value:1.17945e-50</td><td>11673.m04319|LOC_Os07g43960|protein expressed protein e_value:3.13963e-52</td><td>0.659</td><td>(0.7284 1.1055)</td><td>0.7284</td><td>1.1055</td></tr>
<tr><td>2695</td><td>WT010_A03_CLUSTER_27658</td><td>gi|50725125|dbj|BAD33742.1| unknown protein [Oryza sativa (japonica cultivar-group)] e_value:1.10253e-88</td><td>11681.m03368|LOC_Os09g37700|protein expressed protein e_value:2.58602e-90</td><td>0.518</td><td>(0.3494 0.6745)</td><td>0.3494</td><td>0.6745</td></tr>
<tr><td>2696</td><td>WT009_M11_CLUSTER_34395</td><td>gi|50911653|ref|XP_467234.1| putative protein phosphatase 2C [Oryza sativa (japonica cultivar-group)] >gnl|BL_ORD_ID|1580340 gi|41052813|dbj|BAD07681.1| putative protein phosphatase 2C [Oryza sativa (japonica cultivar-group)] e_value:0</td><td>11668.m04498|LOC_Os02g46490|protein Protein phosphatase 2C, putative e_value:0</td><td>0.107</td><td>(0.0616 0.5742)</td><td>0.0616</td><td>0.5742</td></tr>
<tr><td>2697</td><td>WT007_F20_CLUSTER_32671</td><td>gi|13374867|emb|CAC34501.1| putative protein [Arabidopsis thaliana] >gnl|BL_ORD_ID|1488116 gi|26453260|dbj|BAC43703.1| unknown protein [Arabidopsis thaliana] >gnl|BL_ORD_ID|1488116 gi|22326960|ref|NP_680194.1| DNAJ heat shock N-terminal domain-conta</td><td>11669.m01542|LOC_Os03g15480|protein expressed protein e_value:1.068e-132</td><td>0.06</td><td>(0.0280 0.4656)</td><td>0.028</td><td>0.4656</td></tr>
<tr><td>2698</td><td>SET4_N14_CLUSTER_26287</td><td>gi|50925985|ref|XP_473040.1| OSJNBa0067K08.17 [Oryza sativa (japonica cultivar-group)] >gi|38345388|emb|CAD41248.2| OSJNBa0067K08.17 [Oryza sativa (japonica cultivar-group)] e_value:1.66423e-92</td><td>11670.m04040|LOC_Os04g42000|protein 6,7-dimethyl-8-ribityllumazine synthase, putative e_value:3.62995e-94</td><td>0.217</td><td>(0.1051 0.4833)</td><td>0.1051</td><td>0.4833</td></tr>
<tr><td>2699</td><td>SET3_F02_CLUSTER_12809</td><td>gi|30090033|gb|AAO86521.1| cytochrome B5 [Triticum monococcum] e_value:5.96147e-72</td><td>11682.m00098|LOC_Os05g01820|protein cytochrome B5 e_value:2.83495e-25</td><td>0.651</td><td>(0.7004 1.0758)</td><td>0.7004</td><td>1.0758</td></tr>
<tr><td>2700</td><td>WT008__C05_CLUSTER_4693</td><td>gi|50920097|ref|XP_470409.1| putative aspartate kinase [Oryza sativa (japonica cultivar-group)] >gnl|BL_ORD_ID|1535163 gi|27573345|gb|AAO20063.1| putative aspartate kinase [Oryza sativa (japonica cultivar-group)] e_value:0</td><td>11669.m06492|LOC_Os03g63330|protein putative aspartate kinase e_value:0</td><td>0.101</td><td>(0.0610 0.6061)</td><td>0.061</td><td>0.6061</td></tr>
<tr><td>2701</td><td>WT009_B23_CLUSTER_32831</td><td>gi|51469000|emb|CAH18000.1| Ankyrin protein kinase-like [Poa pratensis] e_value:0</td><td>11681.m02249|LOC_Os09g24900|protein At1g26850/T2P11_4 e_value:0</td><td>0.077</td><td>(0.0448 0.5812)</td><td>0.0448</td><td>0.5812</td></tr>
<tr><td>2702</td><td>WT003__C24_CLUSTER_31547</td><td>gi|34903030|ref|NP_912862.1| unnamed protein product [Oryza sativa (japonica cultivar-group)] e_value:5.98164e-91</td><td>11667.m01577|LOC_Os01g15770|protein expressed protein e_value:1.30626e-92</td><td>0.509</td><td>(0.1551 0.3044)</td><td>0.1551</td><td>0.3044</td></tr>
<tr><td>2703</td><td>SET6_A24_CLUSTER_32240</td><td>gi|50921191|ref|XP_470956.1| OSJNBa0068L06.9 [Oryza sativa (japonica cultivar-group)] >gi|38344766|emb|CAE01583.2| OSJNBa0068L06.9 [Oryza sativa (japonica cultivar-group)] e_value:1.56858e-97</td><td>11670.m00024|LOC_Os04g01240|protein hypothetical protein e_value:2.56058e-99</td><td>0.137</td><td>(0.0869 0.6327)</td><td>0.0869</td><td>0.6327</td></tr>
<tr><td>2704</td><td>WT003_K11_CLUSTER_32428</td><td>gi|19880313|gb|AAM00285.1| putative EREBP-type transcription factor [Oryza sativa] e_value:2.42519e-140</td><td>11681.m02410|LOC_Os09g26420|protein EREBP-type transcription factor, putative e_value:6.18332e-142</td><td>0.306</td><td>(0.1746 0.5704)</td><td>0.1746</td><td>0.5704</td></tr>
<tr><td>2705</td><td>WT002_N17_CLUSTER_27616</td><td>gi|50901642|ref|XP_463254.1| putative RNA binding protein [Oryza sativa] >gnl|BL_ORD_ID|1565629 gi|16924113|gb|AAL31692.1| putative RNA binding protein [Oryza sativa] e_value:1.65808e-178</td><td>11686.m04022|LOC_Os12g40560|protein KH domain, putative e_value:0</td><td>0.224</td><td>(0.1298 0.5791)</td><td>0.1298</td><td>0.5791</td></tr>
<tr><td>2706</td><td>WT002_L16_CLUSTER_24966</td><td>gi|50912455|ref|XP_467635.1| putative ADP-ribosylation factor [Oryza sativa (japonica cultivar-group)] >gnl|BL_ORD_ID|1579248 gi|46390658|dbj|BAD16140.1| putative ADP-ribosylation factor [Oryza sativa (japonica cultivar-group)] e_value:7.95711e-97</td><td>11668.m04866|LOC_Os02g49980|protein ADP-ribosylation factor family e_value:1.76463e-98</td><td>0.063</td><td>(0.0280 0.4433)</td><td>0.028</td><td>0.4433</td></tr>
<tr><td>2707</td><td>WT003_M22_CLUSTER_25383</td><td>gi|54291574|dbj|BAD62498.1| unknown protein [Oryza sativa (japonica cultivar-group)] e_value:1.51982e-114</td><td>11680.m05147|LOC_Os06g51510|protein expressed protein e_value:3.40733e-116</td><td>0.196</td><td>(0.1273 0.6497)</td><td>0.1273</td><td>0.6497</td></tr>
<tr><td>2708</td><td>WT003_G16_CLUSTER_34970</td><td>gi|53792840|dbj|BAD53873.1| putative ZEITLUPE [Oryza sativa (japonica cultivar-group)] e_value:0</td><td>11680.m04735|LOC_Os06g47890|protein At5g57360/MSF19_2 e_value:0</td><td>0.081</td><td>(0.0477 0.5867)</td><td>0.0477</td><td>0.5867</td></tr>
<tr><td>2709</td><td>SET2_E22_CLUSTER_7273</td><td>gi|21536972|gb|AAM61313.1| putative RNA-binding protein [Arabidopsis thaliana] >gi|20197470|gb|AAM15089.1| putative RNA-binding protein [Arabidopsis thaliana] >gi|25408558|pir||F84793 probable RNA-binding protein [imported] - Arabidopsis thaliana &g</td><td>11669.m01720|LOC_Os03g17060|protein Similar to probable RNA-binding protein [imported] - Arabidopsis thaliana</td><td>0.164</td><td>(0.0915 0.5561)</td><td>0.0915</td><td>0.5561</td></tr>
<tr><td>2710</td><td>SET1_G14_CLUSTER_8847</td><td>gi|31249767|gb|AAP46259.1| unknown protein [Oryza sativa (japonica cultivar-group)] >gi|50919603|ref|XP_470162.1| unknown protein [Oryza sativa (japonica cultivar-group)] e_value:9.34408e-54</td><td>11669.m06284|LOC_Os03g61680|protein expressed protein e_value:8.77323e-56</td><td>0.077</td><td>(0.0451 0.5818)</td><td>0.0451</td><td>0.5818</td></tr>
<tr><td>2711</td><td>SET1_F01_CLUSTER_18230</td><td>gi|10140699|gb|AAG13533.1| unknown protein [Oryza sativa (japonica cultivar-group)] >gi|31432814|gb|AAP54401.1| unknown protein [Oryza sativa (japonica cultivar-group)] >gi|37535624|ref|NP_922114.1| unknown protein [Oryza sativa (japonica cultivar-g</td><td>11676.m03111|LOC_Os10g35000|protein hypothetical protein e_value:3.70165e-49</td><td>0.159</td><td>(0.0870 0.5488)</td><td>0.087</td><td>0.5488</td></tr>
<tr><td>2712</td><td>WT011_P10_CLUSTER_33949</td><td>gi|50911973|ref|XP_467394.1| ethylene-responsive protein-like [Oryza sativa (japonica cultivar-group)] >gnl|BL_ORD_ID|1580530 gi|51964298|ref|XP_506934.1| PREDICTED OSJNBb0060O16.24 gene product [Oryza sativa (japonica cultivar-group)] >gnl|BL_ORD_I</td><td>11668.m04642|LOC_Os02g47840|protein universal stress protein family e_value:2.56653e-91</td><td>0.13</td><td>(0.0580 0.4476)</td><td>0.058</td><td>0.4476</td></tr>
<tr><td>2713</td><td>WT009_F14_CLUSTER_422</td><td>gi|34903200|ref|NP_912947.1| unnamed protein product [Oryza sativa (japonica cultivar-group)] >gnl|BL_ORD_ID|1752433 gi|6815089|dbj|BAA90375.1| putative multifunctional amino acid-tRNA ligase [Oryza sativa (japonica cultivar-group)] e_value:0</td><td>11667.m01660|LOC_Os01g16520|protein glutamyl-tRNA synthetase e_value:0</td><td>0.115</td><td>(0.0669 0.5841)</td><td>0.0669</td><td>0.5841</td></tr>
<tr><td>2714</td><td>WT008_O05_CLUSTER_34491</td><td>gi|34907628|ref|NP_915161.1| putative cyclin-dependent kinase B1-1 [Oryza sativa (japonica cultivar-group)] >gnl|BL_ORD_ID|1559311 gi|21952860|dbj|BAC06275.1| putative cyclin-dependent kinase B1-1 [Oryza sativa (japonica cultivar-group)] e_value:4.4836</td><td>11667.m06760|LOC_Os01g67160|protein Protein kinase domain e_value:1.86515e-135</td><td>0.192</td><td>(0.0818 0.4257)</td><td>0.0818</td><td>0.4257</td></tr>
<tr><td>2715</td><td>SET4_L13_CLUSTER_31990</td><td>gi|55773674|dbj|BAD72232.1| putative protein kinase p46XlEg22 [Oryza sativa (japonica cultivar-group)] >gi|55771320|dbj|BAD72229.1| putative protein kinase p46XlEg22 [Oryza sativa (japonica cultivar-group)] e_value:1.1448e-158</td><td>11667.m00918|LOC_Os01g09580|protein serine/threonine protein kinase, putative e_value:2.67089e-160</td><td>0.034</td><td>(0.0307 0.9092)</td><td>0.0307</td><td>0.9092</td></tr>
<tr><td>2716</td><td>SET6_M19_CLUSTER_28121</td><td>gi|50948261|ref|XP_483658.1| putative RING-H2 finger protein RHG1a [Oryza sativa (japonica cultivar-group)] >gi|42408731|dbj|BAD09949.1| putative RING-H2 finger protein RHG1a [Oryza sativa (japonica cultivar-group)] >gi|42407550|dbj|BAD10755.1| puta</td><td>11674.m04389|LOC_Os08g43480|protein Zinc finger, C3HC4 type (RING finger), putative e_value:4.55572e-104</td><td>0.385</td><td>(0.1776 0.4615)</td><td>0.1776</td><td>0.4615</td></tr>
<tr><td>2717</td><td>SET1_N17_CLUSTER_16054</td><td>gi|38605539|sp|Q41542|XTH_WHEAT Probable xyloglucan endotransglucosylase/hydrolase precursor >gi|469511|dbj|BAA03924.1| endo-xyloglucan transferase [Triticum aestivum] e_value:8.43137e-174</td><td>11687.m03071|LOC_Os11g33270|protein probable xyloglucan endotransglucosylase/hydrolase precursor(ec 2.4.1.207) e_value:9.65273e-158</td><td>0.327</td><td>(0.0709 0.2171)</td><td>0.0709</td><td>0.2171</td></tr>
<tr><td>2718</td><td>WT002_K21_CLUSTER_33641</td><td>gi|51038046|gb|AAT93850.1| unknown protein [Oryza sativa (japonica cultivar-group)] >gnl|BL_ORD_ID|1020535 gi|46485855|gb|AAS98480.1| unknown protein [Oryza sativa (japonica cultivar-group)] e_value:0</td><td>11682.m01307|LOC_Os05g14170|protein expressed protein e_value:0</td><td>0.177</td><td>(0.0830 0.4694)</td><td>0.083</td><td>0.4694</td></tr>
<tr><td>2719</td><td>SET6_K22_CLUSTER_33641</td><td>gi|51038046|gb|AAT93850.1| unknown protein [Oryza sativa (japonica cultivar-group)] >gi|46485855|gb|AAS98480.1| unknown protein [Oryza sativa (japonica cultivar-group)] e_value:0</td><td>11682.m01307|LOC_Os05g14170|protein expressed protein e_value:0</td><td>0.185</td><td>(0.0878 0.4760)</td><td>0.0878</td><td>0.476</td></tr>
<tr><td>2720</td><td>SET5_P03_CLUSTER_32286</td><td>gi|21618051|gb|AAM67101.1| unknown [Arabidopsis thaliana] >gi|20260058|gb|AAM13376.1| unknown protein [Arabidopsis thaliana] >gi|16649041|gb|AAL24372.1| Unknown protein [Arabidopsis thaliana] >gi|18401463|ref|NP_566572.1| expressed protein [Arabi</td><td>11686.m03727|LOC_Os12g37850|protein expressed protein e_value:1.61336e-42</td><td>0.072</td><td>(0.0592 0.8181)</td><td>0.0592</td><td>0.8181</td></tr>
<tr><td>2721</td><td>SET2_B06_CLUSTER_33065</td><td>gi|50726385|dbj|BAD33996.1| integral membrane protein-like [Oryza sativa (japonica cultivar-group)] e_value:1.23513e-159</td><td>11681.m01292|LOC_Os09g14520|protein expressed protein e_value:2.99164e-161</td><td>0.14</td><td>(0.0983 0.7028)</td><td>0.0983</td><td>0.7028</td></tr>
<tr><td>2722</td><td>SET1_L19_CLUSTER_34144</td><td>gi|53792725|dbj|BAD53736.1| EREBP-4 like protein [Oryza sativa (japonica cultivar-group)] e_value:3.4003e-101</td><td>11680.m03539|LOC_Os06g36490|protein expressed protein e_value:4.9857e-103</td><td>0.17</td><td>(0.0749 0.4399)</td><td>0.0749</td><td>0.4399</td></tr>
<tr><td>2723</td><td>WT006_F09_CLUSTER_5313</td><td>gi|30682530|ref|NP_849649.1| alanine racemase family protein [Arabidopsis thaliana] >gnl|BL_ORD_ID|801708 gi|3157934|gb|AAC17617.1| Similar to hypothetical protein F09E5.8 gb|U37429 from C. elegans. ESTs gb|T42019 and gb|N97000 come from this gene. [A</td><td>11682.m00521|LOC_Os05g05740|protein proline synthetase associated protein, putative e_value:5.56084e-67</td><td>0.32</td><td>(0.1926 0.6010)</td><td>0.1926</td><td>0.601</td></tr>
<tr><td>2724</td><td>SET5_B17_CLUSTER_33262</td><td>gi|50928379|ref|XP_473717.1| OSJNBa0086O06.17 [Oryza sativa (japonica cultivar-group)] >gi|38346719|emb|CAE04869.2| OSJNBa0086O06.17 [Oryza sativa (japonica cultivar-group)] e_value:9.94134e-61</td><td>11673.m04463|LOC_Os07g45330|protein hypothetical protein e_value:3.88121e-147</td><td>0.191</td><td>(0.2230 1.1691)</td><td>0.223</td><td>1.1691</td></tr>
<tr><td>2725</td><td>SET2_M08_CLUSTER_33602</td><td>gi|50940947|ref|XP_480001.1| helix-loop-helix-like protein [Oryza sativa (japonica cultivar-group)] >gi|38636768|dbj|BAD03011.1| helix-loop-helix-like protein [Oryza sativa (japonica cultivar-group)] e_value:1.94902e-111</td><td>11674.m00356|LOC_Os08g04390|protein Helix-loop-helix DNA-binding domain, putative e_value:8.14494e-114</td><td>0.125</td><td>(0.0848 0.6801)</td><td>0.0848</td><td>0.6801</td></tr>
<tr><td>2726</td><td>WT011_H07_CLUSTER_33437</td><td>gi|50251349|dbj|BAD28325.1| putative amino acid transport protein [Oryza sativa (japonica cultivar-group)] e_value:0</td><td>11668.m04340|LOC_Os02g44980|protein Transmembrane amino acid transporter protein e_value:0</td><td>0.252</td><td>(0.0734 0.2907)</td><td>0.0734</td><td>0.2907</td></tr>
<tr><td>2727</td><td>SET1_N15_CLUSTER_8477</td><td>gi|560610|gb|AAB31685.1| trypsin inhibitor, WTI [Triticum aestivum=wheat, variety San Pastore, endosperm, Peptide, 71 aa] >gi|14285484|sp|P81713|IBB3_WHEAT Bowman-Birk type trypsin inhibitor (WTI) e_value:1.48959e-23</td><td>11669.m06192|LOC_Os03g60840|protein putative Bowman-Birk serine protease inhibitor e_value:3.09067e-22</td><td>0.788</td><td>(0.3806 0.4827)</td><td>0.3806</td><td>0.4827</td></tr>
<tr><td>2728</td><td>SET5_D17_CLUSTER_2501</td><td>gi|50948769|ref|XP_493742.1| protein arginine N-methyltransferase protein -like [Oryza sativa (japonica cultivar-group)] >gi|5803265|dbj|BAA83575.1| protein arginine N-methyltransferase protein -like [Oryza sativa (japonica cultivar-group)] e_value:0</td><td>11680.m00435|LOC_Os06g05090|protein expressed protein e_value:0</td><td>0.139</td><td>(0.0700 0.5021)</td><td>0.07</td><td>0.5021</td></tr>
</table>

PDF Complete
Your complimentary use period has ended. Thank you for using PDF Complete.
Click Here to upgrade to Unlimited Pages and Expanded Features

<table>
<tr><th></th><th></th><th></th><th></th><th></th><th></th><th></th><th></th></tr>
<tr><td>2729</td><td>SET5_C08_CLUSTER_26566</td><td>gi|31432296|gb|AAP53946.1| putative kinetochore protein Skp1 [Oryza sativa (japonica cultivar-group)] >gi|37534714|ref|NP_921659.1| putative kinetochore protein Skp1 [Oryza sativa (japonica cultivar-group)] e_value:3.71038e-77</td><td>11676.m02604|LOC_Os10g30200|protein UIP2 e_value:1.10609e-78</td><td>0.42</td><td>(0.1836 0.4376)</td><td>0.1836</td><td>0.4376</td></tr>
<tr><td>2730</td><td>SET3_A10_CLUSTER_30209</td><td>gi|37991868|gb|AAR06314.1| expressed protein [Oryza sativa (japonica cultivar-group)] >gi|50916479|ref|XP_468636.1| expressed protein [Oryza sativa (japonica cultivar-group)] e_value:7.4983e-125</td><td>11669.m02991|LOC_Os03g29730|protein expressed protein e_value:1.43141e-127</td><td>0.307</td><td>(0.1485 0.4842)</td><td>0.1485</td><td>0.4842</td></tr>
<tr><td>2731</td><td>SET2_H23_CLUSTER_10553</td><td>gi|50906189|ref|XP_464583.1| putative coatomer protein complex, subunit beta 2 (beta prime) [Oryza sativa (japonica cultivar-group)] >gi|49387914|dbj|BAD25014.1| putative coatomer protein complex, subunit beta 2 (beta prime) [Oryza sativa (japonica cul</td><td>11670.m04388|LOC_Os04g45190|protein 27k vesicle-associated membrane protein-associated protein - curled-leaved tobacco e_value:5.22796e-16</td><td>0.513</td><td>(0.6794 1.3236)</td><td>0.6794</td><td>1.3236</td></tr>
<tr><td>2732</td><td>SET2_A04_CLUSTER_10278</td><td>gi|50938435|ref|XP_478745.1| putative protein phosphatase 2C [Oryza sativa (japonica cultivar-group)] >gi|33146759|dbj|BAC79670.1| putative protein phosphatase 2C [Oryza sativa (japonica cultivar-group)] e_value:0</td><td>11673.m03677|LOC_Os07g37890|protein Protein phosphatase 2C, putative e_value:0</td><td>0.091</td><td>(0.0564 0.6182)</td><td>0.0564</td><td>0.6182</td></tr>
<tr><td>2733</td><td>SET1_C12_CLUSTER_22941</td><td>gi|50931063|ref|XP_475059.1| putative ankyrin protein [Oryza sativa (japonica cultivar-group)] >gi|46359897|gb|AAS88829.1| putative ankyrin protein [Oryza sativa (japonica cultivar-group)] e_value:0</td><td>11682.m00035|LOC_Os05g01310|protein TPR Domain, putative e_value:0</td><td>0.241</td><td>(0.1577 0.6550)</td><td>0.1577</td><td>0.655</td></tr>
<tr><td>2734</td><td>WT013_M14_CLUSTER_5384</td><td>gi|50920523|ref|XP_470622.1| Unknown protein [Oryza sativa (japonica cultivar-group)] >gnl|BL_ORD_ID|1588714 gi|20330772|gb|AAM19135.1| Unknown protein [Oryza sativa (japonica cultivar-group)] e_value:0</td><td>11669.m01265|LOC_Os03g13010|protein U-box domain, putative e_value:0</td><td>0.073</td><td>(0.0473 0.6506)</td><td>0.0473</td><td>0.6506</td></tr>
<tr><td>2735</td><td>WT013_J01_CLUSTER_13618</td><td>gi|50911070|ref|XP_466968.1| putative sphingolipid delta 4 desaturase [Oryza sativa (japonica cultivar-group)] >gnl|BL_ORD_ID|1608594 gi|49388725|dbj|BAD25906.1| putative sphingolipid delta 4 desaturase [Oryza sativa (japonica cultivar-group)] >gnl|</td><td>11668.m04083|LOC_Os02g42660|protein sphingolipid delta 4 desaturase DES-1, putative e_value:2.93912e-177</td><td>0.026</td><td>(0.0618 2.3741)</td><td>0.0618</td><td>2.3741</td></tr>
<tr><td>2736</td><td>WT012_I02_CLUSTER_7636</td><td>gi|11990497|gb|AAG42167.1| stripe rust resistance protein Yr10 [Triticum aestivum] e_value:0</td><td>11687.m03200|LOC_Os11g34970|protein LZ-NBS-LRR class RGA e_value:0</td><td>0.382</td><td>(0.2999 0.7845)</td><td>0.2999</td><td>0.7845</td></tr>
<tr><td>2737</td><td>WT012_H21_CLUSTER_1434</td><td>gi|6606266|gb|AAF19148.1| Vrga1 [Aegilops ventricosa] e_value:0</td><td>11670.m00115|LOC_Os04g02110|protein NB-ARC domain, putative e_value:0</td><td>0.431</td><td>(0.3703 0.8587)</td><td>0.3703</td><td>0.8587</td></tr>
<tr><td>2738</td><td>WT012_C09_CLUSTER_19946</td><td>gi|21554193|gb|AAM63272.1| unknown [Arabidopsis thaliana] >gnl|BL_ORD_ID|1216242 gi|17979075|gb|AAL49805.1| unknown protein [Arabidopsis thaliana] >gnl|BL_ORD_ID|1216242 gi|18421489|ref|NP_568530.1| EXS family protein / ERD1/XPR1/SYG1 family protein</td><td>11686.m03998|LOC_Os12g40340|protein hypothetical protein e_value:0</td><td>0.092</td><td>(0.0530 0.5740)</td><td>0.053</td><td>0.574</td></tr>
<tr><td>2739</td><td>WT011_F12_CLUSTER_19259</td><td>gi|50915940|ref|XP_468434.1| kinesin light chain-like [Oryza sativa (japonica cultivar-group)] >gnl|BL_ORD_ID|1583551 gi|48716499|dbj|BAD23104.1| kinesin light chain-like [Oryza sativa (japonica cultivar-group)] >gnl|BL_ORD_ID|1583551 gi|48716364|db</td><td>11668.m05692|LOC_Os02g57470|protein probable kinesin light chain [imported] - Arabidopsis thaliana e_value:0</td><td>0.21</td><td>(0.1147 0.5466)</td><td>0.1147</td><td>0.5466</td></tr>
<tr><td>2740</td><td>WT010_O03_CLUSTER_3469</td><td>gi|52077256|dbj|BAD46298.1| putative DNA repair protein [Oryza sativa (japonica cultivar-group)] >gnl|BL_ORD_ID|252427 gi|52076415|dbj|BAD45245.1| putative DNA repair protein [Oryza sativa (japonica cultivar-group)] e_value:0</td><td>11673.m02368|LOC_Os07g25390|protein Helicase conserved C-terminal domain, putative e_value:0</td><td>0.292</td><td>(0.1944 0.6650)</td><td>0.1944</td><td>0.665</td></tr>
<tr><td>2741</td><td>WT009_M05_CLUSTER_12665</td><td>gi|50940109|ref|XP_479582.1| transfactor-like protein [Oryza sativa (japonica cultivar-group)] >gnl|BL_ORD_ID|1534663 gi|34394528|dbj|BAC83815.1| transfactor-like protein [Oryza sativa (japonica cultivar-group)] e_value:2.23392e-87</td><td>11669.m02135|LOC_Os03g20900|protein Similar to transfactor e_value:1.21701e-160</td><td>0.185</td><td>(0.0981 0.5310)</td><td>0.0981</td><td>0.531</td></tr>
<tr><td>2742</td><td>WT009_B01_CLUSTER_14659</td><td>gi|50911144|ref|XP_467005.1| putative YABBY transcription factor CDM51 [Oryza sativa (japonica cultivar-group)] >gnl|BL_ORD_ID|1579947 gi|49388646|dbj|BAD25781.1| putative YABBY transcription factor CDM51 [Oryza sativa (japonica cultivar-</td><td>11668.m04113|LOC_Os02g42950|protein yabby15 protein e_value:1.17844e-118</td><td>0.174</td><td>(0.0737 0.4246)</td><td>0.0737</td><td>0.4246</td></tr>
<tr><td>2743</td><td>WT007_J24_CLUSTER_28969</td><td>gi|50540694|gb|AAT77851.1| putative protein kinase [Oryza sativa (japonica cultivar-group)] e_value:0</td><td>11669.m05145|LOC_Os03g51020|protein putative protein kinase e_value:0</td><td>0.089</td><td>(0.0614 0.6890)</td><td>0.0614</td><td>0.689</td></tr>
<tr><td>2744</td><td>WT007_J18_CLUSTER_12199</td><td>gi|22136510|gb|AAM91333.1| unknown protein [Arabidopsis thaliana] >gnl|BL_ORD_ID|1123080 gi|20260258|gb|AAM13027.1| unknown protein [Arabidopsis thaliana] >gnl|BL_ORD_ID|1123080 gi|22329527|ref|NP_172762.2| sphere organelles protein-related [Arabido</td><td>11686.m01895|LOC_Os12g19040|protein Similar to protein F3F19.5 [imported] - Arabidopsis thaliana e_value:3.19685e-97</td><td>0.313</td><td>(0.2383 0.7613)</td><td>0.2383</td><td>0.7613</td></tr>
<tr><td>2745</td><td>WT007_G18_CLUSTER_5225</td><td>gi|31431512|gb|AAP53285.1| hypothetical protein [Oryza sativa (japonica cultivar-group)] >gnl|BL_ORD_ID|2010431 gi|37533392|ref|NP_920998.1| hypothetical protein [Oryza sativa (japonica cultivar-group)] >gnl|BL_ORD_ID|2010431 gi|18057116|gb|AAL58139</td><td>11669.m00967|LOC_Os03g10240|protein hypothetical protein e_value:0</td><td>0.16</td><td>(0.0571 0.3557)</td><td>0.0571</td><td>0.3557</td></tr>
<tr><td>2746</td><td>WT006_C14_CLUSTER_21598</td><td>gi|55770685|ref|XP_550384.1| ATP synthetase alpha chain -like [Oryza sativa (japonica cultivar-group)] >gnl|BL_ORD_ID|1753692 gi|55296253|dbj|BAD67994.1| ATP synthetase alpha chain -like [Oryza sativa (japonica cultivar-group)] >gnl|BL_ORD_ID|175369</td><td>11667.m00783|LOC_Os01g08340|protein RING zinc finger protein; 53384-54880, putative e_value:2.12401e-175</td><td>0.269</td><td>(0.1598 0.5951)</td><td>0.1598</td><td>0.5951</td></tr>
<tr><td>2747</td><td>WT005_O18_CLUSTER_30676</td><td>gi|50916076|ref|XP_468502.1| putative prolyl 4-hydroxylase [Oryza sativa (japonica cultivar-group)] >gnl|BL_ORD_ID|1583616 gi|48716447|dbj|BAD23054.1| putative prolyl 4-hydroxylase [Oryza sativa (japonica cultivar-group)] e_value:2.65772e-154</td><td>11668.m05757|LOC_Os02g58070|protein F14O10.12 protein - Arabidopsis thaliana e_value:5.95225e-156</td><td>0.129</td><td>(0.0722 0.5588)</td><td>0.0722</td><td>0.5588</td></tr>
<tr><td>2748</td><td>WT005_N19_CLUSTER_315</td><td>gi|38346715|emb|CAE04865.2| OSJNBa0086O06.13 [Oryza sativa (japonica cultivar-group)] >gnl|BL_ORD_ID|1535119 gi|50928371|ref|XP_473713.1| OSJNBa0086O06.13 [Oryza sativa (japonica cultivar-group)] e_value:1.07821e-167</td><td>11670.m04921|LOC_Os04g49990|protein AT hook motif, putative e_value:2.99037e-169</td><td>0.142</td><td>(0.0876 0.6176)</td><td>0.0876</td><td>0.6176</td></tr>
<tr><td>2749</td><td>WT004_M22_CLUSTER_5483</td><td>gi|50939761|ref|XP_479408.1| nucleoid DNA-binding-like protein [Oryza sativa (japonica cultivar-group)] >gnl|BL_ORD_ID|2378799 gi|50510141|dbj|BAD31106.1| nucleoid DNA-binding-like protein [Oryza sativa (japonica cultivar-group)] >gnl|BL_ORD_ID|2378</td><td>11673.m04582|LOC_Os07g46480|protein Eukaryotic aspartyl protease e_value:0</td><td>0.343</td><td>(0.0880 0.2562)</td><td>0.088</td><td>0.2562</td></tr>
<tr><td>2750</td><td>WT003_L12_CLUSTER_16834</td><td>gi|50929895|ref|XP_474475.1| OSJNBb0020J19.7 [Oryza sativa (japonica cultivar-group)] >gnl|BL_ORD_ID|1594824 gi|38567829|emb|CAE05778.3| OSJNBb0020J19.7 [Oryza sativa (japonica cultivar-group)] e_value:0</td><td>11670.m05901|LOC_Os04g59520|protein C2 domain, putative e_value:0</td><td>0.339</td><td>(0.1483 0.4380)</td><td>0.1483</td><td>0.438</td></tr>
<tr><td>2751</td><td>WT002_K03_CLUSTER_20677</td><td>gi|34901986|ref|NP_912339.1| unknown protein [Oryza sativa (japonica cultivar-group)] >gnl|BL_ORD_ID|1588792 gi|29893577|gb|AAP06831.1| unknown protein [Oryza sativa (japonica cultivar-group)] e_value:0</td><td>11669.m01580|LOC_Os03g15810|protein ATPase, AAA family, putative e_value:0</td><td>0.248</td><td>(0.1272 0.5131)</td><td>0.1272</td><td>0.5131</td></tr>
<tr><td>2752</td><td>SET5_P06_CLUSTER_14145</td><td>gi|50918921|ref|XP_469857.1| hypothetical protein [Oryza sativa (japonica cultivar-group)] >gi|14488372|gb|AAK63939.1| hypothetical protein [Oryza sativa (japonica cultivar-group)] e_value:2.29451e-44</td><td>11669.m05565|LOC_Os03g55310|protein expressed protein e_value:3.18879e-46</td><td>0.326</td><td>(0.1698 0.5206)</td><td>0.1698</td><td>0.5206</td></tr>
<tr><td>2753</td><td>SET5_O02_CLUSTER_5336</td><td>gi|25140432|gb|AAN71760.1| cinnamoyl CoA reductase [Hordeum vulgare] e_value:0</td><td>11681.m02276|LOC_Os09g25150|protein cinnamoyl CoA reductase e_value:1.07219e-177</td><td>0.136</td><td>(0.0384 0.2822)</td><td>0.0384</td><td>0.2822</td></tr>
<tr><td>2754</td><td>SET5_J11_CLUSTER_14910</td><td>gi|30679129|ref|NP_567232.2| U3 ribonucleoprotein (Utp) family protein [Arabidopsis thaliana] e_value:0</td><td>11669.m02292|LOC_Os03g22320|protein hypothetical protein e_value:0</td><td>0.304</td><td>(0.1617 0.5312)</td><td>0.1617</td><td>0.5312</td></tr>
<tr><td>2755</td><td>WT002_D15_CLUSTER_16171</td><td>gi|50919279|ref|XP_470036.1| unknown protein [Oryza sativa (japonica cultivar-group)] >gnl|BL_ORD_ID|1588213 gi|30102997|gb|AAP21410.1| unknown protein [Oryza sativa (japonica cultivar-group)] e_value:0</td><td>11669.m06021|LOC_Os03g59300|protein expressed protein e_value:0</td><td>0.146</td><td>(0.0579 0.3951)</td><td>0.0579</td><td>0.3951</td></tr>
<tr><td>2756</td><td>WT002_M05_CLUSTER_20071</td><td>gi|34897392|ref|NP_910042.1| putative permease [Oryza sativa (japonica cultivar-group)] >gnl|BL_ORD_ID|1588251 gi|27545049|gb|AAO18455.1| putative permease [Oryza sativa (japonica cultivar-group)] e_value:0</td><td>11669.m06196|LOC_Os03g60880|protein putative permease e_value:0</td><td>0.081</td><td>(0.0352 0.4342)</td><td>0.0352</td><td>0.4342</td></tr>
<tr><td>2757</td><td>SET5_L11_CLUSTER_14740</td><td>gi|55297001|dbj|BAD68476.1| DNA-binding bromodomain-containing protein-like [Oryza sativa (japonica cultivar-group)] >gi|55296460|dbj|BAD68656.1| DNA-binding bromodomain-containing protein-like [Oryza sativa (japonica cultivar-group)]</td><td>11680.m00385|LOC_Os06g04640|protein Similar to IMB1 e_value:7.58477e-134</td><td>0.188</td><td>(0.1577 0.8394)</td><td>0.1577</td><td>0.8394</td></tr>
<tr><td>2758</td><td>WT009_L07_CLUSTER_489</td><td>gi|50944103|ref|XP_481579.1| putative indole-3-glycerol phosphate synthase [Oryza sativa (japonica cultivar-group)] >gnl|BL_ORD_ID|1605231 gi|38424034|dbj|BAD01725.1| putative indole-3-glycerol phosphate synthase [Oryza sativa (japonica cultivar-group)</td><td>11674.m02243|LOC_Os08g23150|protein indole-3-glycerol phosphate synthase, putative e_value:5.38671e-170</td><td>0.243</td><td>(0.1088 0.4479)</td><td>0.1088</td><td>0.4479</td></tr>
<tr><td>2759</td><td>WT003_I22_CLUSTER_31700</td><td>gi|50929179|ref|XP_474117.1| OSJNBa0010D21.8 [Oryza sativa (japonica cultivar-group)] >gnl|BL_ORD_ID|1593216 gi|38345495|emb|CAE01666.2| OSJNBa0010D21.8 [Oryza sativa (japonica cultivar-group)] e_value:1.17508e-126</td><td>11670.m05465|LOC_Os04g55500|protein expressed protein e_value:1.48167e-132</td><td>0.551</td><td>(0.4335 0.7870)</td><td>0.4335</td><td>0.787</td></tr>
<tr><td>2760</td><td>WT013_D15_CLUSTER_20995</td><td>gi|50904645|ref|XP_463811.1| unknown protein [Oryza sativa (japonica cultivar-group)] >gnl|BL_ORD_ID|1565855 gi|41052547|dbj|BAD07539.1| unknown protein [Oryza sativa (japonica cultivar-group)] >gnl|BL_ORD_ID|1565855 gi|40363769|dbj|BAD06279.1| unkn</td><td>11668.m00047|LOC_Os02g01450|protein Similar to At2g45060/T14P1.13 e_value:3.25438e-123</td><td>0.197</td><td>(0.0997 0.5062)</td><td>0.0997</td><td>0.5062</td></tr>
<tr><td>2761</td><td>SET5_N23_CLUSTER_30820</td><td>gi|50924852|ref|XP_472771.1| B1358B12.20 [Oryza sativa (japonica cultivar-group)] >gi|38567722|emb|CAE76011.1| B1358B12.20 [Oryza sativa (japonica cultivar-group)] >gi|21741992|emb|CAD41042.1| OSJNBa0060P14.1 [Oryza sativa (japonica cultivar-group)]</td><td>11670.m03698|LOC_Os04g38950|protein glutamine amidotransferase of anthranilate synthase or para-aminobenzoate synthase, putative e_value:1.14379e-123</td><td>0.192</td><td>(0.1291 0.6713)</td><td>0.1291</td><td>0.6713</td></tr>
<tr><td>2762</td><td>SET2_L01_CLUSTER_11364</td><td>gi|50933855|ref|XP_476455.1| putative CSLC9(cellulose synthase-like) [Oryza sativa (japonica cultivar-group)] >gi|28201308|dbj|BAC56816.1| putative CSLC9(cellulose synthase-like) [Oryza sativa (japonica cultivar-group)] e_value:0</td><td>11673.m00235|LOC_Os07g03260|protein glycosyl transferase, group 2 family protein, putative e_value:0</td><td>0.138</td><td>(0.1043 0.7550)</td><td>0.1043</td><td>0.755</td></tr>
<tr><td>2763</td><td>WT011_N18_CLUSTER_3370</td><td>gi|21740732|emb|CAD40853.1| OSJNBa0086B14.26 [Oryza sativa (japonica cultivar-group)] >gnl|BL_ORD_ID|1524056 gi|50924658|ref|XP_472684.1| OSJNBa0086B14.26 [Oryza sativa (japonica cultivar-group)] e_value:0</td><td>11670.m03593|LOC_Os04g37950|protein probable ubiquitin carboxyl terminal hydrolase [imported] - Arabidopsis thaliana</td><td>0.024</td><td>(0.0121 0.5060)</td><td>0.0121</td><td>0.506</td></tr>
<tr><td>2764</td><td>WT005_H23_CLUSTER_34683</td><td>gi|6102957|emb|CAB59202.1| serine carboxylase II-2 [Hordeum vulgare subsp. vulgare] >gnl|BL_ORD_ID|1709244 gi|2493494|sp|P55748|CBP22_HORVU Serine carboxypeptidase II-2 precursor (CP-MII.2) >gnl|BL_ORD_ID|1709244 gi|619351|gb|AAB31590.1| CP-MII.2=se</td><td>11680.m00828|LOC_Os06g08720|protein serine carboxypeptidase ii-2 precursor (ec 3.4.16.6) (cp-mii.2)(fragment) e_value:0</td><td>0.182</td><td>(0.0943 0.5175)</td><td>0.0943</td><td>0.5175</td></tr>
<tr><td>2765</td><td>WT011_J03_CLUSTER_25233</td><td>gi|9758171|dbj|BAB08556.1| unnamed protein product [Arabidopsis thaliana] >gnl|BL_ORD_ID|1088946 gi|15240501|ref|NP_200350.1| hydroxyproline-rich glycoprotein family protein [Arabidopsis thaliana] e_value:1.9431e-64</td><td>11686.m01197|LOC_Os12g12300|protein expressed protein e_value:1.03892e-84</td><td>0.569</td><td>(0.4228 0.7425)</td><td>0.4228</td><td>0.7425</td></tr>
<tr><td>2766</td><td>WT005_I21_CLUSTER_8272</td><td>gi|34912860|ref|NP_917777.1| beta-adaptin-like protein A [Oryza sativa (japonica cultivar-group)] e_value:0</td><td>11667.m04232|LOC_Os01g43630|protein Adaptin N terminal region, putative e_value:0</td><td>0.091</td><td>(0.0455 0.5010)</td><td>0.0455</td><td>0.501</td></tr>
<tr><td>2767</td><td>WT007_D20_CLUSTER_22997</td><td>gi|50919225|ref|XP_470009.1| serine/threonine protein phosphatase PP2A-2 catalytic subunit [Oryza sativa (japonica cultivar-group)] >gnl|BL_ORD_ID|1587485 gi|4530611|gb|AAD22116.1| serine/threonine protein phosphatase PP2A-2 catalytic subunit [Oryza sa</td><td>11669.m05994|LOC_Os03g59060|protein serine/threonine protein phosphatase PP2A-2 catalytic subunit e_value:0</td><td>0.019</td><td>(0.0085 0.4380)</td><td>0.0085</td><td>0.438</td></tr>
<tr><td>2768</td><td>SET5_M22_CLUSTER_4137</td><td>gi|57231736|gb|AAW47581.1| diacylglycerol acyltransferase [Oryza sativa (japonica cultivar-group)] e_value:0</td><td>11682.m01012|LOC_Os05g10810|protein MBOAT family, putative e_value:0</td><td>0.174</td><td>(0.0830 0.4778)</td><td>0.083</td><td>0.4778</td></tr>
<tr><td>2769</td><td>WT013_I20_CLUSTER_12878</td><td>gi|20197202|gb|AAM14969.1| putative small nuclear ribonucleoprotein Prp4p [Arabidopsis thaliana] >gnl|BL_ORD_ID|938951 gi|2618685|gb|AAB84332.1| putative small nuclear ribonucleoprotein Prp4p [Arabidopsis thaliana] >gnl|BL_ORD_ID|938951 gi|58652074|</td><td>11669.m02419|LOC_Os03g23930|protein hypothetical protein e_value:0</td><td>0.163</td><td>(0.0770 0.4734)</td><td>0.077</td><td>0.4734</td></tr>
</table>

PDF Complete
Your complimentary use period has ended. Thank you for using PDF Complete.
Click Here to upgrade to Unlimited Pages and Expanded Features

2770	SET6_G18_CLUSTER_3418	gi|50932231|ref|XP_475643.1| putative nucleic acid binding (PHD-finger) protein [Oryza sativa (japonica cultivar-group)] >gi|46981338|gb|AAT07656.1| putative nucleic acid binding (PHD-finger) protein [Oryza sativa (japonica cultivar-group)] e_value:9.3	11682.m00658|LOC_Os05g07040|protein PHD-finger, putative e_value:1.96187e-132	0.139	(0.0578 0.4170)	0.0578	0.417
2771	WT002_A12_CLUSTER_6584	gi|15218889|ref|NP_174226.1| transducin family protein / WD-40 repeat family protein [Arabidopsis thaliana] e_value:4.41588e-117	11673.m03091|LOC_Os07g32350|protein hypothetical protein e_value:4.87671e-180	0.252	(0.1216 0.4823)	0.1216	0.4823
2772	WT009_J11_CLUSTER_31865	gi|37788310|gb|AAP44536.1| cyclophilin-like protein [Triticum aestivum] e_value:0	11673.m00750|LOC_Os07g08190|protein Similar to cyclophylin-like protein - Arabidopsis thaliana e_value:0	0.317	(0.1727 0.5452)	0.1727	0.5452
2773	WT005_O05_CLUSTER_28295	gi|50948139|ref|XP_483597.1| putative RNA recognition motif (RRM)-containing protein [Oryza sativa (japonica cultivar-group)] >gnl|BL_ORD_ID|1610563 gi|51965058|ref|XP_507313.1| PREDICTED OJ1521_G02.20 gene product [Oryza sativa (japonica cultivar-grou	11674.m04337|LOC_Os08g42980|protein La domain, putative e_value:6.27452e-153	0.279	(0.1622 0.5821)	0.1622	0.5821
2774	WT009_J06_CLUSTER_19119	gi|50909451|ref|XP_466214.1| putative DDT domain-containing protein [Oryza sativa (japonica cultivar-group)] >gnl|BL_ORD_ID|1576334 gi|46389867|dbj|BAD15468.1| putative DDT domain-containing protein [Oryza sativa (japonica cultivar-group)] e_value:0	11668.m03327|LOC_Os02g35100|protein expressed protein e_value:0	0.219	(0.2080 0.9501)	0.208	0.9501
2775	SET4_C17_CLUSTER_19995	gi|21281189|gb|AAM45052.1| putative DNA binding protein ACBF [Arabidopsis thaliana] >gi|18377731|gb|AAL67015.1| putative DNA binding protein ACBF [Arabidopsis thaliana] >gi|15239715|ref|NP_197436.1| RNA-binding protein 45 (RBP45), putative [Arabidop	11669.m00744|LOC_Os03g07800|protein DNA binding protein ACBF, putative e_value:0	0.153	(0.0704 0.4586)	0.0704	0.4586
2776	WT011_G06_CLUSTER_23089	gi|31415971|gb|AAP50991.1| putative 3-isopropylmalate dehydrogenase [Oryza sativa (japonica cultivar-group)] >gnl|BL_ORD_ID|2005475 gi|50917349|ref|XP_469071.1| putative 3-isopropylmalate dehydrogenase [Oryza sativa (japonica cultivar-group)] e_value:0	11669.m04520|LOC_Os03g45320|protein putative 3-isopropylmalate dehydrogenase e_value:0	0.172	(0.0799 0.4633)	0.0799	0.4633
2777	WT005_L09_CLUSTER_20392	gi|31432929|gb|AAP54500.1| putative RNA helicase [Oryza sativa (japonica cultivar-group)] >gnl|BL_ORD_ID|2012548 gi|37535822|ref|NP_922213.1| putative RNA helicase [Oryza sativa (japonica cultivar-group)] >gnl|BL_ORD_ID|2012548 gi|10140782|gb|AAG136	11676.m03223|LOC_Os10g35990|protein putative RNA helicase e_value:0	0.107	(0.0528 0.4948)	0.0528	0.4948
2778	WT009_D03_CLUSTER_3295	gi|31432468|gb|AAP54098.1| hypothetical protein [Oryza sativa (japonica cultivar-group)] >gnl|BL_ORD_ID|2011626 gi|37535018|ref|NP_921811.1| hypothetical protein [Oryza sativa (japonica cultivar-group)] >gnl|BL_ORD_ID|2011626 gi|14140297|gb|AAK54303	11676.m02788|LOC_Os10g31930|protein hypothetical protein e_value:3.38983e-74	0.287	(0.2525 0.8788)	0.2525	0.8788
2779	SET3_P18_CLUSTER_32538	gi|50946711|ref|XP_482883.1| putative dioscorin class A precursor [Oryza sativa (japonica cultivar-group)] >gi|42408633|dbj|BAD09854.1| putative dioscorin class A precursor [Oryza sativa (japonica cultivar-group)] e_value:3.47583e-95	11674.m03640|LOC_Os08g36630|protein Eukaryotic-type carbonic anhydrase e_value:9.12609e-97	0.423	(0.2681 0.6333)	0.2681	0.6333
2780	WT013_I08_CLUSTER_17351	gi|50920565|ref|XP_470643.1| Unknown protein [Oryza sativa (japonica cultivar-group)] >gnl|BL_ORD_ID|1588732 gi|27357984|gb|AAO06976.1| Unknown protein [Oryza sativa (japonica cultivar-group)] e_value:0	11669.m01315|LOC_Os03g13460|protein Microtubule associated protein (MAP65/ASE1 family) e_value:0	0.128	(0.0772 0.6036)	0.0772	0.6036
2781	WT008_J09_CLUSTER_22287	gi|50912019|ref|XP_467417.1| putative nuclear matrix constituent protein 1 [Oryza sativa (japonica cultivar-group)] >gnl|BL_ORD_ID|1580560 gi|32352206|dbj|BAC78596.1| hypothetical protein [Oryza sativa (japonica cultivar-group)] >gnl|BL_ORD_ID|15805	11668.m04659|LOC_Os02g48010|protein expressed protein e_value:0	0.213	(0.1454 0.6833)	0.1454	0.6833
2782	WT009_K11_CLUSTER_19974	gi|54290874|dbj|BAD61535.1| SEC13 protein homolog YGL100w-like [Oryza sativa (japonica cultivar-group)] >gnl|BL_ORD_ID|972033 gi|54290371|dbj|BAD61210.1| SEC13 protein homolog YGL100w-like [Oryza sativa (japonica cultivar-group)] e_value:1.24111e-166	11667.m04190|LOC_Os01g43250|protein hypothetical protein e_value:7.95669e-140	0.143	(0.0679 0.4743)	0.0679	0.4743
2783	SET3_H24_CLUSTER_5991	gi|31433627|gb|AAP55119.1| unknown protein [Oryza sativa (japonica cultivar-group)] >gi|37537060|ref|NP_922832.1| unknown protein [Oryza sativa (japonica cultivar-group)] >gi|12643052|gb|AAK00441.1| unknown protein [Oryza sativa] e_value:1.28059e-11	11676.m03904|LOC_Os10g42250|protein expressed protein e_value:2.48087e-116	0.069	(0.0345 0.4996)	0.0345	0.4996
2784	WT012_B01_CLUSTER_5211	gi|50932059|ref|XP_475557.1| unknown protein [Oryza sativa (japonica cultivar-group)] >gnl|BL_ORD_ID|1597003 gi|47900459|gb|AAT39235.1| unknown protein [Oryza sativa (japonica cultivar-group)] >gnl|BL_ORD_ID|1597003 gi|46391150|gb|AAS90677.1| unknow	11682.m00284|LOC_Os05g03540|protein hypothetical protein e_value:4.93744e-75	0.295	(0.1259 0.4262)	0.1259	0.4262
2785	WT006_M11_CLUSTER_17454	gi|46200513|gb|AAL73527.2| putative AP endonuclease [Sorghum bicolor] e_value:1.33718e-153	11686.m01802|LOC_Os12g18200|protein AP endonuclease, putative e_value:0	0.15	(0.0763 0.5080)	0.0763	0.508
2786	SET5_F03_CLUSTER_19528	gi|50911837|ref|XP_467326.1| putative growth-regulating factor 1 [Oryza sativa (japonica cultivar-group)] >gi|51039837|tpg|DAA05208.1| TPA: growth-regulating factor 4 [Oryza sativa (japonica cultivar-group)] >gi|41052677|dbj|BAD07524.1| putative gro	11668.m04580|LOC_Os02g47280|protein expressed protein e_value:7.30241e-152	0.192	(0.1429 0.7460)	0.1429	0.746
2787	WT012_K04_CLUSTER_29310	gi|22003088|emb|CAD30695.1| fatty acyl coA reductase [Triticum aestivum] >gnl|BL_ORD_ID|984204 gi|22003086|emb|CAD30694.1| fatty acyl coA reductase [Triticum aestivum] e_value:0	11681.m03546|LOC_Os09g39410|protein Male sterility protein, putative e_value:0	0.286	(0.2070 0.7247)	0.207	0.7247
2788	SET2_D24_CLUSTER_33388	gi|5091606|gb|AAD39595.1| 10A19I.10 [Oryza sativa (japonica cultivar-group)] e_value:2.97275e-103	11682.m04848|LOC_Os05g50360|protein Anaphase-promoting complex, subunit 10 (APC10) e_value:5.73723e-104	0.119	(0.0506 0.4254)	0.0506	0.4254
2789	SET3_H11_CLUSTER_1652	gi|50932439|ref|XP_475747.1| unknown protein [Oryza sativa (japonica cultivar-group)] >gi|48843819|gb|AAT47078.1| unknown protein [Oryza sativa (japonica cultivar-group)] e_value:0	11682.m04369|LOC_Os05g45590|protein hexokinase e_value:0	0.153	(0.0720 0.4701)	0.072	0.4701
2790	WT011_G04_CLUSTER_13708	gi|55770715|ref|XP_550399.1| hypothetical protein [Oryza sativa (japonica cultivar-group)] >gnl|BL_ORD_ID|1753480 gi|55296306|dbj|BAD68086.1| hypothetical protein [Oryza sativa (japonica cultivar-group)] >gnl|BL_ORD_ID|1753480	11667.m00807|LOC_Os01g08580|protein hypothetical protein e_value:0	0.278	(0.1850 0.6660)	0.185	0.666
2791	WT008__C19_CLUSTER_1154	gi|50509095|dbj|BAD30155.1| unknown protein [Oryza sativa (japonica cultivar-group)] e_value:0	11673.m00900|LOC_Os07g09580|protein expressed protein	0.24	(0.1298 0.5402)	0.1298	0.5402
2792	WT011_L13_CLUSTER_32120	gi|34910440|ref|NP_916567.1| histone H1-like protein [Oryza sativa (japonica cultivar-group)] e_value:3.3648e-92	11667.m04995|LOC_Os01g51160|protein linker histone H1 and H5 family, putative e_value:6.30818e-62	0.421	(0.2185 0.5190)	0.2185	0.519
2793	SET2_B19_CLUSTER_32309	gi|50945065|ref|XP_482060.1| putative RUB1 conjugating enzyme [Oryza sativa (japonica cultivar-group)] >gi|51979665|ref|XP_507579.1| PREDICTED P0690C12.28 gene product [Oryza sativa (japonica cultivar-group)] >gi|51964848|ref|XP_507208.1| PREDICTED	11674.m02812|LOC_Os08g28680|protein Ubiquitin-conjugating enzyme, putative e_value:1.52771e-94	0.07	(0.0715 1.0222)	0.0715	1.0222
2794	WT005_F02_CLUSTER_32725	gi|55733815|gb|AAV59322.1| hexokinase [Oryza sativa (japonica cultivar-group)] >gnl|BL_ORD_ID|1794520 gi|55168165|gb|AAV44032.1| hexokinase 1 [Oryza sativa (japonica cultivar-group)] >gnl|BL_ORD_ID|1794520 gi|13991925|gb|AAK51559.1| hexokinase I [Or	11682.m04277|LOC_Os05g44760|protein hexokinase I e_value:0	0.144	(0.0751 0.5206)	0.0751	0.5206
2795	WT011_D03_CLUSTER_27561	gi|53830029|gb|AAU94922.1| floral homeotic protein [Triticum aestivum] >gnl|BL_ORD_ID|1329321 gi|53830027|gb|AAU94921.1| floral homeotic protein [Triticum turgidum] >gnl|BL_ORD_ID|1329321 gi|53830019|gb|AAU94917.1| floral homeotic protein [Triticum	11669.m06144|LOC_Os03g60430|protein APETALA2-like protein e_value:1.64536e-168	0.281	(0.1456 0.5179)	0.1456	0.5179
2796	SET2_L20_CLUSTER_11592	gi|50916180|ref|XP_468554.1| putative chloroplast channel forming outer membrane protein [Oryza sativa (japonica cultivar-group)] >gi|48716404|dbj|BAD23013.1| putative chloroplast channel forming outer membrane protein [Oryza sativa (japonica cultivar-	11668.m05808|LOC_Os02g58550|protein At1g20810/F2D10_27 e_value:1.3372e-57	0.387	(0.2640 0.6830)	0.264	0.683
2797	WT005_L07_CLUSTER_32305	gi|50929747|ref|XP_474401.1| OSJNBa0032F06.22 [Oryza sativa (japonica cultivar-group)] >gnl|BL_ORD_ID|1594583 gi|32488646|emb|CAE03439.1| OSJNBa0032F06.22 [Oryza sativa (japonica cultivar-group)] e_value:3.82938e-111	11670.m05809|LOC_Os04g58640|protein expressed protein e_value:6.74255e-113	0.049	(0.0152 0.3116)	0.0152	0.3116
2798	SET3_A17_CLUSTER_33829	gi|50945221|ref|XP_482138.1| putative cytochrome b-561 [Oryza sativa (japonica cultivar-group)] >gi|40253890|dbj|BAD05824.1| putative cytochrome b-561 [Oryza sativa (japonica cultivar-group)] e_value:2.04079e-114	11674.m02899|LOC_Os08g29520|protein Cytochrome b561 e_value:4.92936e-116	0.13	(0.0699 0.5367)	0.0699	0.5367
2799	WT003_I13_CLUSTER_34998	gi|50251454|dbj|BAD28519.1| unknown protein [Oryza sativa (japonica cultivar-group)] e_value:6.65203e-142	11681.m02121|LOC_Os09g23660|protein ZIM motif, putative e_value:1.68494e-143	0.247	(0.1665 0.6751)	0.1665	0.6751
2800	WT012_D03_CLUSTER_5672	gi|50882144|ref|NP_910923.2| unknown protein [Oryza sativa (japonica cultivar-group)] >gnl|BL_ORD_ID|1533424 gi|38175743|dbj|BAC22424.2| unknown protein [Oryza sativa (japonica cultivar-group)] e_value:1.25471e-29	11673.m04611|LOC_Os07g46710|protein expressed protein e_value:1.07639e-30	0.436	(0.1608 0.3685)	0.1608	0.3685
2801	WT006_F01_CLUSTER_13560	gi|51964056|ref|XP_506811.1| PREDICTED P0047E05.40 gene product [Oryza sativa (japonica cultivar-group)] >gnl|BL_ORD_ID|1576486 gi|50908835|ref|XP_465906.1| unknown protein [Oryza sativa (japonica cultivar-group)] >gnl|BL_ORD_ID|1576486 gi|48716957|	11668.m02896|LOC_Os02g30470|protein hypothetical protein e_value:6.68355e-31	0.88	(0.0481 0.0547)	0.0481	0.0547
2802	WT003_D13_CLUSTER_17825	gi|34905280|ref|NP_913987.1| putative syntaxin SYP111 [Oryza sativa (japonica cultivar-group)] >gnl|BL_ORD_ID|1602377 gi|28564634|dbj|BAC57816.1| putative syntaxin SYP111 [Oryza sativa (japonica cultivar-group)] e_value:0	11676.m00140|LOC_Os10g02350|protein Endomembrane protein 70, putative e_value:0	0.076	(0.1531 2.0100)	0.1531	2.01
2803	WT004_A10_CLUSTER_3810	gi|50252077|dbj|BAD28007.1| putative RAD23 protein [Oryza sativa (japonica cultivar-group)] e_value:5.68324e-164	11668.m00799|LOC_Os02g08300|protein UV excision repair protein Rad23 e_value:1.31728e-165	0.194	(0.1025 0.5295)	0.1025	0.5295
2804	SET4_J22_CLUSTER_33121	gi|55296987|dbj|BAD68462.1| putative ATP-dependent Clp protease proteolytic subunit [Oryza sativa (japonica cultivar-group)] e_value:6.45502e-153	11680.m00374|LOC_Os06g04530|protein ATP-dependent Clp protease, proteolytic subunit ClpP, putative e_value:1.73993e-	0.119	(0.0506 0.4248)	0.0506	0.4248
2805	WT002_J05_CLUSTER_33716	gi|50943671|ref|XP_481363.1| putative threonyl-tRNA synthetase [Oryza sativa (japonica cultivar-group)] >gnl|BL_ORD_ID|1604466 gi|38175524|dbj|BAD01219.1| putative threonyl-tRNA synthetase [Oryza sativa (japonica cultivar-group)] >gnl|BL_ORD_ID|1604	11674.m01910|LOC_Os08g19850|protein threonyl-tRNA synthetase, putative e_value:0	0.141	(0.0952 0.6732)	0.0952	0.6732
2806	WT013_A21_CLUSTER_32507	gi|50725969|dbj|BAD33496.1| 26S proteasome regulatory subunit-like protein [Oryza sativa (japonica cultivar-group)] e_value:3.35591e-95	11681.m02294|LOC_Os09g25310|protein At5g57950 e_value:7.438e-97	0.257	(0.0896 0.3485)	0.0896	0.3485
2807	WT009_D07_CLUSTER_34612	gi|20302435|emb|CAD29296.1| plasma membrane H+ ATPase [Oryza sativa (japonica cultivar-group)] e_value:0	11686.m04413|LOC_Os12g44150|protein plasma-membrane proton-efflux P-type ATPase e_value:0	0.048	(0.0288 0.6025)	0.0288	0.6025
2808	WT003_P13_CLUSTER_32586	gi|440869|dbj|BAA04257.1| major intrinsic protein [Oryza sativa] >gnl|BL_ORD_ID|2367386 gi|50251783|dbj|BAD27715.1| major intrinsic protein [Oryza sativa (japonica cultivar-group)] >gnl|BL_ORD_ID|2367386 gi|1076748|pir||S52003 major intrinsic protei	11668.m01284|LOC_Os02g13870|protein major intrinsic protein - rice e_value:3.29535e-136	0.184	(0.0711 0.3862)	0.0711	0.3862
2809	SET1_I13_CLUSTER_34020	gi|13194796|gb|AAK15560.1| unknown protein [Arabidopsis thaliana] >gi|19310813|gb|AAL85137.1| unknown protein [Arabidopsis thaliana] >gi|15028183|gb|AAK76588.1| unknown protein [Arabidopsis thaliana] >gi|21536763|gb|AAM61095.1| unknown [Arabidops	11686.m03706|LOC_Os12g37650|protein expressed protein e_value:4.14656e-71	0.752	(0.2776 0.3692)	0.2776	0.3692
2810	WT011_N24_CLUSTER_11028	gi|28393871|gb|AAO42343.1| unknown protein [Arabidopsis thaliana] >gnl|BL_ORD_ID|982370 gi|27754564|gb|AAO22729.1| unknown protein [Arabidopsis thaliana] >gnl|BL_ORD_ID|982370 gi|18416732|ref|NP_568256.1| conserved oligomeric Golgi complex component	11686.m03454|LOC_Os12g35290|protein Dor1-like family, putative e_value:0	0.158	(0.0836 0.5274)	0.0836	0.5274
2811	WT012__C23_CLUSTER_32394	gi|50919181|ref|XP_469987.1| synaptobrevin-like protein [Oryza sativa (japonica cultivar-group)] >gnl|BL_ORD_ID|1587462 gi|29150380|gb|AAO72389.1| synaptobrevin-like protein [Oryza sativa (japonica cultivar-group)] e_value:1.52673e-113	11669.m05969|LOC_Os03g58840|protein synaptobrevin-like protein e_value:2.90465e-115	0.086	(0.0441 0.5151)	0.0441	0.5151
2812	WT005_I10_CLUSTER_32791	gi|51963726|ref|XP_506643.1| PREDICTED B1008E06.13 gene product [Oryza sativa (japonica cultivar-group)] e_value:3.74179e-106	11681.m00776|LOC_Os09g09480|protein SNF7 family e_value:7.53389e-108	0.085	(0.0537 0.6350)	0.0537	0.635

PDF Complete
Your complimentary use period has ended. Thank you for using PDF Complete.
Click Here to upgrade to Unlimited Pages and Expanded Features

2813	WT008_K02_CLUSTER_13626	gi|50899124|ref|XP_450350.1| putative protein kinase G11A [Oryza sativa (japonica cultivar-group)] >gnl|BL_ORD_ID|1563611 gi|48717063|dbj|BAD23751.1| putative protein kinase G11A [Oryza sativa (japonica cultivar-group)] >gnl|BL_ORD_ID|1563611 gi|487	11681.m00667|LOC_Os09g08420|protein probable protein kinase - maize (fragment) e_value:0	0.12	(0.0967 0.8066)	0.0967	0.8066
2814	SET4_O06_CLUSTER_33513	gi|54291490|dbj|BAD62311.1| putative microtubule-associated protein [Oryza sativa (japonica cultivar-group)] >gi|54290620|dbj|BAD62191.1| putative microtubule-associated protein [Oryza sativa (japonica cultivar-group)] e_value:0	11680.m01985|LOC_Os06g20370|protein Microtubule associated protein (MAP65/ASE1 family) e_value:0	0.085	(0.0492 0.5803)	0.0492	0.5803
2815	SET4_K21_CLUSTER_20736	gi|54291178|dbj|BAD61850.1| putative coclaurine N-methyltransferase [Oryza sativa (japonica cultivar-group)] e_value:3.57193e-171	11680.m03657|LOC_Os06g37610|protein coclaurine N-methyltransferase e_value:1.78464e-132	0.061	(0.0405 0.6616)	0.0405	0.6616
2816	WT003_E21_CLUSTER_24037	gi|32401386|gb|AAP80862.1| Emr1 [Triticum aestivum] e_value:5.94273e-172	11686.m00922|LOC_Os12g09580|protein Emr1 e_value:0	0.232	(0.1528 0.6577)	0.1528	0.6577
2817	WT003_K16_CLUSTER_31743	gi|50944209|ref|XP_481632.1| putative GMP synthetase [Oryza sativa (japonica cultivar-group)] >gnl|BL_ORD_ID|1605306 gi|24414065|dbj|BAC22314.1| putative GMP synthetase [Oryza sativa (japonica cultivar-group)] e_value:0	11674.m02304|LOC_Os08g23730|protein GMP synthase, C-terminal domain, putative e_value:0	0.134	(0.0497 0.3707)	0.0497	0.3707
2818	WT009_D14_CLUSTER_33247	gi|56202373|dbj|BAD73735.1| unknown protein [Oryza sativa (japonica cultivar-group)] >gnl|BL_ORD_ID|552192 gi|56202213|dbj|BAD73812.1| unknown protein [Oryza sativa (japonica cultivar-group)] e_value:1.37448e-163	11667.m03248|LOC_Os01g34330|protein BAH domain, putative e_value:4.66156e-159	0.428	(0.2371 0.5538)	0.2371	0.5538
2819	SET5_A18_CLUSTER_33247	gi|56202373|dbj|BAD73735.1| unknown protein [Oryza sativa (japonica cultivar-group)] >gi|56202213|dbj|BAD73812.1| unknown protein [Oryza sativa (japonica cultivar-group)] e_value:0	11667.m03248|LOC_Os01g34330|protein BAH domain, putative e_value:0	0.341	(0.2121 0.6212)	0.2121	0.6212
2820	WT013_B13_CLUSTER_19690	gi|50932855|ref|XP_475955.1| unknown protein, contains hAT family dimerisation domain, PF05699 [Oryza sativa (japonica cultivar-group)] e_value:0	11669.m05285|LOC_Os03g52310|protein putative transposases e_value:0	0.122	(0.1037 0.8526)	0.1037	0.8526
2821	WT012_G14_CLUSTER_32065	gi|50920599|ref|XP_470660.1| Unknown protein [Oryza sativa (japonica cultivar-group)] >gnl|BL_ORD_ID|1588851 gi|27476067|gb|AAO16998.1| Unknown protein [Oryza sativa (japonica cultivar-group)] e_value:1.57258e-110	11669.m01790|LOC_Os03g17710|protein Transposable element protein, putative e_value:6.37297e-140	0.13	(0.0985 0.7575)	0.0985	0.7575
2822	WT005_K19_CLUSTER_31804	gi|31745233|gb|AAP68893.1| putative NADH dehydrogenase [Oryza sativa (japonica cultivar-group)] >gnl|BL_ORD_ID|2050089 gi|34915206|ref|NP_919060.1| putative NADH dehydrogenase [Oryza sativa (japonica cultivar-group)] e_value:1.41544e-117	11669.m05676|LOC_Os03g56300|protein putative NADH dehydrogenase e_value:3.34328e-119	0.072	(0.0439 0.6110)	0.0439	0.611
2823	SET3_D07_CLUSTER_33355	gi|50252087|dbj|BAD28017.1| putative protein phosphatase type-2C [Oryza sativa (japonica cultivar-group)] e_value:0	11680.m04346|LOC_Os06g44210|protein protein phosphatase type-2C e_value:9.86643e-180	0.107	(0.0870 0.8152)	0.087	0.8152
2824	WT004_F12_CLUSTER_17172	gi|54291833|gb|AAV32201.1| histidyl-tRNA synthetase [Oryza sativa (japonica cultivar-group)] e_value:0	11682.m00532|LOC_Os05g05840|protein histidyl-tRNA synthetase, putative e_value:0	0.172	(0.1033 0.6000)	0.1033	0.6
2825	SET2_B11_CLUSTER_1303	gi|50939579|ref|XP_479317.1| unknown protein [Oryza sativa (japonica cultivar-group)] >gi|50509939|dbj|BAD30260.1| unknown protein [Oryza sativa (japonica cultivar-group)] >gi|33146729|dbj|BAC79620.1| unknown protein [Oryza sativa (japonica cultivar	11673.m04425|LOC_Os07g44960|protein expressed protein e_value:2.71754e-162	0.105	(0.0979 0.9300)	0.0979	0.93
2826	WT004_E08_CLUSTER_34680	gi|50915876|ref|XP_468402.1| putative NADH dehydrogenase [Oryza sativa (japonica cultivar-group)] >gnl|BL_ORD_ID|1583938 gi|47848189|dbj|BAD22016.1| putative NADH dehydrogenase [Oryza sativa (japonica cultivar-group)] >gnl|BL_ORD_ID|1583938 gi|47847	11668.m05651|LOC_Os02g57180|protein expressed protein e_value:0	0.215	(0.0852 0.3961)	0.0852	0.3961
2827	WT002_H02_CLUSTER_32071	gi|51038703|gb|AAT94161.1| cell death-related protein SPL11 [Oryza sativa (japonica cultivar-group)] >gnl|BL_ORD_ID|1282505 gi|51038701|gb|AAT94160.1| cell death-related protein SPL11 [Oryza sativa (japonica cultivar-group)] >gnl|BL_ORD_ID|1282505 g	11686.m03764|LOC_Os12g38210|protein arm repeat containing protein homolog - Arabidopsis thaliana e_value:0	0.102	(0.0639 0.6273)	0.0639	0.6273
2828	WT005_M18_CLUSTER_34855	gi|50943911|ref|XP_481483.1| putative TGF-beta receptor-interacting protein [Oryza sativa (japonica cultivar-group)] >gnl|BL_ORD_ID|1604786 gi|35210527|dbj|BAC92643.1| putative TGF-beta receptor-interacting protein [Oryza sativa (japonica cultivar-	11674.m02093|LOC_Os08g21660|protein putative TGF-beta receptor-interacting protein 1 e_value:3.24479e-173	0.124	(0.0611 0.4924)	0.0611	0.4924
2829	SET5_M05_CLUSTER_14819	gi|51090849|dbj|BAD35377.1| bZIP transcription factor-like [Oryza sativa (japonica cultivar-group)] e_value:3.00836e-96	11680.m04091|LOC_Os06g41770|protein Similar to probable bZIP transcription factor [imported] - Arabidopsis thaliana e_value:8.9584e-98	0.485	(0.1769 0.3646)	0.1769	0.3646
2830	SET1_B02_CLUSTER_24057	gi|50911146|ref|XP_467006.1| thylakoid lumenal protein-like [Oryza sativa (japonica cultivar-group)] >gi|49388647|dbj|BAD25782.1| thylakoid lumenal protein-like [Oryza sativa (japonica cultivar-group)] e_value:5.45897e-118	11668.m04114|LOC_Os02g42960|protein expressed protein e_value:1.17852e-119	0.146	(0.1004 0.6874)	0.1004	0.6874
2831	WT007_I09_CLUSTER_22170	gi|32966063|gb|AAP92128.1| putative ATPase ATP1 [Oryza sativa (japonica cultivar-group)] >gnl|BL_ORD_ID|2088204 gi|34911210|ref|NP_916952.1| putative CAD ATPase [Oryza sativa (japonica cultivar-group)] >gnl|BL_ORD_ID|2088204 gi|19386661|dbj|BAB86043	11667.m04764|LOC_Os01g49000|protein ATPase, AAA family, putative e_value:0	0.061	(0.0337 0.5484)	0.0337	0.5484
2832	WT008_F10_CLUSTER_11867	gi|50930921|ref|XP_474988.1| OSJNBa0065B15.8 [Oryza sativa (japonica cultivar-group)] >gnl|BL_ORD_ID|1595414 gi|38344562|emb|CAD39904.2| OSJNBa0065B15.8 [Oryza sativa (japonica cultivar-group)] e_value:0	11670.m01414|LOC_Os04g15920|protein oxidoreductase, zinc-binding dehydrogenase family e_value:0	0.166	(0.0812 0.4879)	0.0812	0.4879
2833	WT006_N17_CLUSTER_973	gi|34894702|ref|NP_908676.1| P0426D06.17 [Oryza sativa (japonica cultivar-group)] >gnl|BL_ORD_ID|1560777 gi|12328578|dbj|BAB21237.1| putative WD-40 repeat protein [Oryza sativa (japonica cultivar-group)] e_value:0	11667.m02124|LOC_Os01g21940|protein expressed protein e_value:0	0.056	(0.0279 0.4947)	0.0279	0.4947
2834	WT007_M23_CLUSTER_13969	gi|50911909|ref|XP_467362.1| unknown protein [Oryza sativa (japonica cultivar-group)] >gnl|BL_ORD_ID|1580490 gi|41053140|dbj|BAD08083.1| unknown protein [Oryza sativa (japonica cultivar-group)] e_value:0	11668.m04613|LOC_Os02g47580|protein expressed protein e_value:0	0.365	(0.1673 0.4585)	0.1673	0.4585
2835	SET3_L13_CLUSTER_8012	gi|56784626|dbj|BAD81673.1| glycogenin-like protein [Oryza sativa (japonica cultivar-group)] e_value:0	11667.m06609|LOC_Os01g65780|protein Glycosyl transferase family 8, putative e_value:0	0.089	(0.0708 0.7967)	0.0708	0.7967
2836	WT006_G24_CLUSTER_14770	gi|50929729|ref|XP_474392.1| OSJNBa0032F06.13 [Oryza sativa (japonica cultivar-group)] >gnl|BL_ORD_ID|1594574 gi|32488637|emb|CAE03430.1| OSJNBa0032F06.13 [Oryza sativa (japonica cultivar-group)] e_value:0	11669.m02788|LOC_Os03g27350|protein putative autophagy protein (with alternative splicing) e_value:0	0.203	(0.1371 0.6741)	0.1371	0.6741
2837	SET5_D07_CLUSTER_25222	gi|31432749|gb|AAP54342.1| hypothetical protein [Oryza sativa (japonica cultivar-group)] >gi|37535506|ref|NP_922055.1| hypothetical protein [Oryza sativa (japonica cultivar-group)] >gi|18087872|gb|AAL59026.1| hypothetical protein [Oryza sativa] e_va	11676.m03045|LOC_Os10g34390|protein expressed protein e_value:0	0.354	(0.1821 0.5147)	0.1821	0.5147
2838	WT003_P04_CLUSTER_13370	gi|7547109|gb|AAF63781.1| unknown protein [Arabidopsis thaliana] >gnl|BL_ORD_ID|1558557 gi|26451670|dbj|BAC42931.1| unknown protein [Arabidopsis thaliana] >gnl|BL_ORD_ID|1558557 gi|28973333|gb|AAO63991.1| putative RRM-containing protein [Arabidopsis	11668.m03944|LOC_Os02g40900|protein RRM-containing protein, putative e_value:2.40545e-110	0.118	(0.0700 0.5955)	0.07	0.5955
2839	SET4_O04_CLUSTER_9531	gi|7269979|emb|CAB79796.1| putative protein [Arabidopsis thaliana] >gi|28973227|gb|AAO63938.1| unknown protein [Arabidopsis thaliana] >gi|28393759|gb|AAO42289.1| unknown protein [Arabidopsis thaliana] >gi|5725442|emb|CAB52451.1| putative protein	11681.m00327|LOC_Os09g04160|protein expressed protein e_value:0	0.202	(0.1194 0.5908)	0.1194	0.5908
2840	WT003_H19_CLUSTER_14276	gi|31433347|gb|AAP54876.1| putative protein phosphatase 2C [Oryza sativa (japonica cultivar-group)] >gnl|BL_ORD_ID|2012814 gi|37536574|ref|NP_922589.1| putative protein phosphatase 2C [Oryza sativa (japonica cultivar-group)] e_value:0	11676.m03633|LOC_Os10g39780|protein putative protein phosphatase 2C e_value:0	0.147	(0.0677 0.4596)	0.0677	0.4596
2841	SET2_B01_CLUSTER_32902	gi|8778999|gb|AAF79914.1| Contains a weak similarity to Rap8 (Rac-3-1) gene from Rhynchosciara americana gb|U69899. [Arabidopsis thaliana] >gi|25518429|pir||C86333 hypothetical protein T20H2.23 - Arabidopsis thaliana e_value:1.21956e-35	11669.m01441|LOC_Os03g14580|protein expressed protein e_value:1.31283e-88	0.402	(0.2433 0.6055)	0.2433	0.6055
2842	WT006_P04_CLUSTER_7235	gi|31430853|gb|AAP52712.1| putative hydroxyproline-rich glycoprotein [Oryza sativa (japonica cultivar-group)] >gnl|BL_ORD_ID|2009371 gi|37532246|ref|NP_920425.1| putative hydroxyproline-rich glycoprotein [Oryza sativa (japonica cultivar-group)] >gnl	11669.m01486|LOC_Os03g15000|protein Zinc finger, C3HC4 type (RING finger), putative e_value:0	0.146	(0.0913 0.6232)	0.0913	0.6232
2843	WT010_N21_CLUSTER_16068	gi|37699750|gb|AAR00586.1| acyl-CoA oxidase [Phalaenopsis cv. 'True Lady'] e_value:0	11687.m03645|LOC_Os11g39220|protein acyl-CoA oxidase homolog e_value:0	0.122	(0.0723 0.5915)	0.0723	0.5915
2844	SET4_I18_CLUSTER_18984	gi|50898970|ref|XP_450273.1| integral membrane protein-like [Oryza sativa (japonica cultivar-group)] >gi|47497809|dbj|BAD19907.1| integral membrane protein-like [Oryza sativa (japonica cultivar-group)] e_value:8.05032e-40	11681.m00569|LOC_Os09g07480|protein expressed protein e_value:6.23021e-42	0.434	(0.3269 0.7539)	0.3269	0.7539
2845	WT005_G17_CLUSTER_10093	gi|13398414|gb|AAK21880.1| arabinoxylan arabinofuranohydrolase isoenzyme AXAH-II [Hordeum vulgare] e_value:0	11687.m00289|LOC_Os11g03780|protein arabinoxylan arabinofuranohydrolase isoenzyme AXAH-II e_value:0	0.193	(0.1329 0.6892)	0.1329	0.6892
2846	WT004_P06_CLUSTER_12138	gi|50939217|ref|XP_479136.1| unknown protein [Oryza sativa (japonica cultivar-group)] >gnl|BL_ORD_ID|1530452 gi|33146698|dbj|BAC79498.1| unknown protein [Oryza sativa (japonica cultivar-group)] e_value:3.46267e-88	11673.m04120|LOC_Os07g42080|protein expressed protein e_value:5.34733e-90	0.181	(0.1219 0.6749)	0.1219	0.6749
2847	WT006_L20_CLUSTER_24749	gi|51964548|ref|XP_507059.1| PREDICTED P0474F11.28-1 gene product [Oryza sativa (japonica cultivar-group)] >gnl|BL_ORD_ID|1583524 gi|50915974|ref|XP_468451.1| hydroxyproline-rich glycoprotein-like [Oryza sativa (japonica cultivar-group)]	11670.m05149|LOC_Os04g52570|protein expressed protein e_value:3.5786e-143	0.171	(0.0761 0.4440)	0.0761	0.444
2848	WT004_B19_CLUSTER_23608	gi|50907131|ref|XP_465054.1| putative X1 [Oryza sativa (japonica cultivar-group)] >gnl|BL_ORD_ID|1567647 gi|47847697|dbj|BAD21477.1| putative X1 [Oryza sativa (japonica cultivar-group)] e_value:0	11668.m01823|LOC_Os02g19130|protein XS domain, putative e_value:0	0.234	(0.1649 0.7048)	0.1649	0.7048
2849	WT008_M09_CLUSTER_33322	gi|2290204|gb|AAB96883.1| nucellin [Hordeum vulgare] >gnl|BL_ORD_ID|306616 gi|2290202|gb|AAB96882.1| nucellin [Hordeum vulgare] >gnl|BL_ORD_ID|306616 gi|45357050|gb|AAS58479.1| nucellin [Hordeum vulgare subsp. vulgare] >gnl|BL_ORD_ID|306616 gi|74	11687.m00750|LOC_Os11g08100|protein nucellin-like aspartic protease e_value:7.23665e-91	0.382	(0.4167 1.0909)	0.4167	1.0909
2850	WT004_L24_CLUSTER_16093	gi|4996640|dbj|BAA78572.1| Dof zinc finger protein [Oryza sativa] e_value:3.48952e-144	11670.m04698|LOC_Os04g47990|protein Dof zinc finger protein e_value:7.0321e-146	0.402	(0.1615 0.4020)	0.1615	0.402
2851	WT004_A04_CLUSTER_11465	gi|52076720|dbj|BAD45633.1| putative thaumatin-protein [Oryza sativa (japonica cultivar-group)] >gnl|BL_ORD_ID|379094 gi|53793288|dbj|BAD54510.1| putative thaumatin-protein [Oryza sativa (japonica cultivar-group)] e_value:4.01389e-120	11680.m04704|LOC_Os06g47600|protein thaumatin-like protein e_value:8.68001e-114	0.37	(0.1574 0.4254)	0.1574	0.4254
2852	WT002_L06_CLUSTER_24482	gi|50941331|ref|XP_480193.1| putative Band 7 protein [Oryza sativa (japonica cultivar-group)] >gnl|BL_ORD_ID|1602353 gi|51964706|ref|XP_507137.1| PREDICTED P0498E12.110 gene product [Oryza sativa (japonica cultivar-group)] >gnl|BL_ORD_ID|1602353	11674.m00548|LOC_Os08g06200|protein SPFH domain / Band 7 family, putative e_value:4.5205e-158	0.216	(0.1201 0.5552)	0.1201	0.5552
2853	WT002_H03_CLUSTER_10579	gi|50929029|ref|XP_474042.1| OSJNBb0034I13.9 [Oryza sativa (japonica cultivar-group)] >gnl|BL_ORD_ID|1592705 gi|21741248|emb|CAD41732.1| OSJNBb0034I13.9 [Oryza sativa (japonica cultivar-group)] e_value:0	11670.m05351|LOC_Os04g54440|protein RNA recognition motif. (a.k.a. RRM, RBD, or RNP domain), putative e_value:0	0.211	(0.1147 0.5426)	0.1147	0.5426
2854	SET6_K14_CLUSTER_24482	gi|50941331|ref|XP_480193.1| putative Band 7 protein [Oryza sativa (japonica cultivar-group)] >gi|51964706|ref|XP_507137.1| PREDICTED P0498E12.110 gene product [Oryza sativa (japonica cultivar-group)] >gi|37806149|dbj|BAC99654.1| putative Band 7 pro	11674.m00548|LOC_Os08g06200|protein SPFH domain / Band 7 family, putative e_value:1.00706e-157	0.224	(0.1221 0.5442)	0.1221	0.5442
2855	SET5_M07_CLUSTER_7485	gi|13377508|gb|AAK20745.1| unknown [Triticum aestivum] e_value:1.86247e-160	11673.m00761|LOC_Os07g08290|protein expressed protein e_value:1.68162e-161	0.375	(0.3906 1.0412)	0.3906	1.0412
2856	SET5_I17_CLUSTER_29303	gi|53792589|dbj|BAD53604.1| putative HGA1 [Oryza sativa (japonica cultivar-group)] >gi|53792782|dbj|BAD53817.1| putative HGA1 [Oryza sativa (japonica cultivar-group)] e_value:0	11680.m04894|LOC_Os06g49320|protein expressed protein e_value:0	0.21	(0.1025 0.4886)	0.1025	0.4886

Your complimentary use period has ended. Thank you for using PDF Complete.
PDF Complete
Click Here to upgrade to Unlimited Pages and Expanded Features

<table>
<tr><td>2857</td><td>SET5_B20_CLUSTER_11024</td><td>gi|31431844|gb|AAP53563.1| hypothetical protein [Oryza sativa (japonica cultivar-group)] >gi|37533948|ref|NP_921276.1| hypothetical protein [Oryza sativa (japonica cultivar-group)] >gi|15451625|gb|AAK98749.1| Hypothetical protein [Oryza sativa] e_va</td><td>11676.m02136|LOC_Os10g25230|protein expressed protein e_value:7.79461e-33</td><td>0.457</td><td>(0.2870 0.6276)</td><td>0.287</td><td>0.6276</td></tr>
<tr><td>2858</td><td>SET4_O02_CLUSTER_7439</td><td>gi|38346045|emb|CAE01903.2| OSJNBa0059D20.17 [Oryza sativa (japonica cultivar-group)] >gi|50930449|ref|XP_474752.1| OSJNBa0059D20.17 [Oryza sativa (japonica cultivar-group)] e_value:2.63734e-34</td><td>11670.m01012|LOC_Os04g11450|protein F-box domain, putative e_value:6.63038e-36</td><td>0.541</td><td>(0.8032 1.4856)</td><td>0.8032</td><td>1.4856</td></tr>
<tr><td>2859</td><td>SET4_B12_CLUSTER_22086</td><td>gi|50838978|gb|AAT81739.1| subtilase family protein [Oryza sativa (japonica cultivar-group)] e_value:0</td><td>11669.m03202|LOC_Os03g31630|protein subtilase family protein e_value:0</td><td>0.215</td><td>(0.3233 1.5048)</td><td>0.3233</td><td>1.5048</td></tr>
<tr><td>2860</td><td>SET3_D14_CLUSTER_2226</td><td>gi|50926452|ref|XP_473173.1| OSJNBb0070J16.14 [Oryza sativa (japonica cultivar-group)] >gi|32482945|emb|CAE02349.1| OSJNBb0072M01.10 [Oryza sativa (japonica cultivar-group)] >gi|38345698|emb|CAE01918.2| OSJNBb0070J16.14 [Oryza sativa (japonica culti</td><td>11670.m04212|LOC_Os04g43550|protein hypothetical protein e_value:4.12151e-53</td><td>0.441</td><td>(0.1162 0.2632)</td><td>0.1162</td><td>0.2632</td></tr>
<tr><td>2861</td><td>SET3_A12_CLUSTER_5608</td><td>gi|34899018|ref|NP_910855.1| hypothetical protein [Oryza sativa (japonica cultivar-group)] >gi|22831270|dbj|BAC16125.1| hypothetical protein [Oryza sativa (japonica cultivar-group)] >gi|50510128|dbj|BAD31094.1| hypothetical protein [Oryza sativa (ja</td><td>11673.m04564|LOC_Os07g46300|protein expressed protein e_value:0</td><td>0.206</td><td>(0.0981 0.4767)</td><td>0.0981</td><td>0.4767</td></tr>
<tr><td>2862</td><td>SET2_K18_CLUSTER_357</td><td>gi|50929547|ref|XP_474301.1| OSJNBa0043A12.38 [Oryza sativa (japonica cultivar-group)] >gi|32488408|emb|CAE02833.1| OSJNBa0043A12.38 [Oryza sativa (japonica cultivar-group)] e_value:6.98431e-102</td><td>11670.m05688|LOC_Os04g57540|protein probable adenylate kinase 1, chloroplast precursor (ec 2.7.4.3) (atp- amp transphosphorylase). [mouse-ear cress] e_value:8.1431e-104</td><td>0.289</td><td>(0.1280 0.4426)</td><td>0.128</td><td>0.4426</td></tr>
<tr><td>2863</td><td>SET2_F24_CLUSTER_7628</td><td>No hits found</td><td>11682.m01022|LOC_Os05g10910|protein hypothetical protein e_value:4.41192e-08</td><td>0.591</td><td>(0.5563 0.9416)</td><td>0.5563</td><td>0.9416</td></tr>
<tr><td>2864</td><td>WT013_M20_CLUSTER_30285</td><td>gi|50940917|ref|XP_479986.1| putative Polycomb group protein FIE2 [Oryza sativa (japonica cultivar-group)] >gnl|BL_ORD_ID|1601768 gi|38636833|dbj|BAD03073.1| putative Polycomb group protein FIE2 [Oryza sativa (japonica cultivar-group)] e_value:0</td><td>11674.m00343|LOC_Os08g04270|protein expressed protein e_value:0</td><td>0.167</td><td>(0.0757 0.4534)</td><td>0.0757</td><td>0.4534</td></tr>
<tr><td>2865</td><td>WT013_K02_CLUSTER_22480</td><td>gi|52353371|gb|AAU43939.1| hypothetical protein [Oryza sativa (japonica cultivar-group)] e_value:4.89969e-139</td><td>11682.m04096|LOC_Os05g43100|protein hypothetical protein e_value:1.57941e-140</td><td>0.205</td><td>(0.2509 1.2259)</td><td>0.2509</td><td>1.2259</td></tr>
<tr><td>2866</td><td>WT013_J24_CLUSTER_26727</td><td>gi|27368821|emb|CAD59568.1| PDR-like ABC transporter [Oryza sativa (japonica cultivar-group)] >gnl|BL_ORD_ID|998275 gi|28144343|tpg|DAA00886.1| TPA: PDR3 ABC transporter [Oryza sativa (japonica cultivar-group)] e_value:0</td><td>11667.m04087|LOC_Os01g42380|protein ABC transporter, putative e_value:0</td><td>0.101</td><td>(0.0589 0.5838)</td><td>0.0589</td><td>0.5838</td></tr>
<tr><td>2867</td><td>WT012_P04_CLUSTER_24333</td><td>gi|34904686|ref|NP_913690.1| putative pentatricopeptide (PPR) repeat-containing protein [Oryza sativa (japonica cultivar-group)] >gnl|BL_ORD_ID|1525122 gi|50509708|dbj|BAD31746.1| putative pentatricopeptide (PPR) repeat-containing protein [Oryza sativa</td><td>11673.m01905|LOC_Os07g19400|protein expressed protein e_value:0</td><td>0.205</td><td>(0.1165 0.5692)</td><td>0.1165</td><td>0.5692</td></tr>
<tr><td>2868</td><td>WT012_O22_CLUSTER_30296</td><td>gi|56783755|dbj|BAD81167.1| unknown protein [Oryza sativa (japonica cultivar-group)] >gnl|BL_ORD_ID|773560 gi|56783734|dbj|BAD81146.1| unknown protein [Oryza sativa (japonica cultivar-group)] e_value:3.59208e-120</td><td>11667.m01642|LOC_Os01g16340|protein hypothetical protein e_value:2.07656e-110</td><td>0.257</td><td>(0.1464 0.5687)</td><td>0.1464</td><td>0.5687</td></tr>
<tr><td>2869</td><td>WT012_F05_CLUSTER_28033</td><td>gi|55167986|gb|AAV43854.1| unknown protein [Oryza sativa (japonica cultivar-group)] >gnl|BL_ORD_ID|1465360 gi|47777404|gb|AAT38038.1| unknown protein [Oryza sativa (japonica cultivar-group)] e_value:7.33385e-128</td><td>11682.m02725|LOC_Os05g29020|protein expressed protein e_value:2.01744e-129</td><td>0.301</td><td>(0.1183 0.3928)</td><td>0.1183</td><td>0.3928</td></tr>
<tr><td>2870</td><td>WT011_J12_CLUSTER_4589</td><td>gi|50905731|ref|XP_464354.1| putative ankyrin repeat-containing protein [Oryza sativa (japonica cultivar-group)] >gnl|BL_ORD_ID|1566681 gi|49387531|dbj|BAD25064.1| putative ankyrin repeat-containing protein [Oryza sativa (japonica cultivar-group)]</td><td>11668.m00834|LOC_Os02g09130|protein CG6618-PB, putative e_value:0</td><td>0.157</td><td>(0.0627 0.3985)</td><td>0.0627</td><td>0.3985</td></tr>
<tr><td>2871</td><td>WT010_K16_CLUSTER_29864</td><td>gi|21314337|gb|AAM46895.1| early drought induced protein [Oryza sativa (indica cultivar-group)] e_value:0</td><td>11667.m00851|LOC_Os01g08970|protein Structure-specific recognition protein, putative e_value:0</td><td>0.217</td><td>(0.0859 0.3958)</td><td>0.0859</td><td>0.3958</td></tr>
<tr><td>2872</td><td>WT010_G23_CLUSTER_13727</td><td>gi|56201964|dbj|BAD73414.1| putative Hv1LRR2 [Oryza sativa (japonica cultivar-group)] e_value:0</td><td>11687.m03262|LOC_Os11g35580|protein NB-ARC domain, putative e_value:0</td><td>0.281</td><td>(0.3202 1.1413)</td><td>0.3202</td><td>1.1413</td></tr>
<tr><td>2873</td><td>WT009_L22_CLUSTER_33536</td><td>gi|6016700|gb|AAF01527.1| unknown protein [Arabidopsis thaliana] >gnl|BL_ORD_ID|1558171 gi|18399058|ref|NP_566383.1| protein phosphatase-related [Arabidopsis thaliana] e_value:3.30465e-95</td><td>11687.m00122|LOC_Os11g02180|protein Dual specificity phosphatase, catalytic domain, putative e_value:2.27173e-116</td><td>0.195</td><td>(0.1402 0.7200)</td><td>0.1402</td><td>0.72</td></tr>
<tr><td>2874</td><td>WT009_A04_CLUSTER_3524</td><td>gi|62732927|gb|AAX95046.1| expressed protein [Oryza sativa (japonica cultivar-group)] e_value:5.60909e-79</td><td>11687.m00927|LOC_Os11g09820|protein hypothetical protein e_value:3.59252e-66</td><td>0.795</td><td>(0.8448 1.0631)</td><td>0.8448</td><td>1.0631</td></tr>
<tr><td>2875</td><td>WT008_C18_CLUSTER_30244</td><td>gi|50938639|ref|XP_478847.1| putative subtilisin-like serine protease [Oryza sativa (japonica cultivar-group)] >gnl|BL_ORD_ID|1529731 gi|50508423|dbj|BAD30472.1| putative subtilisin-like serine protease [Oryza sativa (japonica cultivar-group)] >gnl|</td><td>11673.m03798|LOC_Os07g39020|protein Subtilisin N-terminal Region, putative e_value:0</td><td>0.72</td><td>(0.2827 0.3926)</td><td>0.2827</td><td>0.3926</td></tr>
<tr><td>2876</td><td>WT007_P10_CLUSTER_3857</td><td>gi|50929149|ref|XP_474102.1| OSJNBa0070O11.5 [Oryza sativa (japonica cultivar-group)] >gnl|BL_ORD_ID|1593208 gi|38344006|emb|CAE03174.2| OSJNBa0070O11.5 [Oryza sativa (japonica cultivar-group)] e_value:0</td><td>11670.m05436|LOC_Os04g55260|protein expressed protein e_value:0</td><td>0.128</td><td>(0.0601 0.4700)</td><td>0.0601</td><td>0.47</td></tr>
<tr><td>2877</td><td>WT007_L17_CLUSTER_11384</td><td>gi|50906083|ref|XP_464530.1| unknown protein [Oryza sativa (japonica cultivar-group)] >gnl|BL_ORD_ID|1566817 gi|51979180|ref|XP_507458.1| PREDICTED OJ1135_F06.7 gene product [Oryza sativa (japonica cultivar-group)] >gnl|BL_ORD_ID|1566817 gi|51963938</td><td>11668.m01016|LOC_Os02g10820|protein expressed protein e_value:4.01759e-145</td><td>0.094</td><td>(0.0589 0.6238)</td><td>0.0589</td><td>0.6238</td></tr>
<tr><td>2878</td><td>WT007_I19_CLUSTER_27396</td><td>gi|38344540|emb|CAD40970.2| OSJNBa0027P08.8 [Oryza sativa (japonica cultivar-group)] >gnl|BL_ORD_ID|1534614 gi|50924566|ref|XP_472643.1| OSJNBa0027P08.8 [Oryza sativa (japonica cultivar-group)] e_value:0</td><td>11670.m03510|LOC_Os04g36740|protein stelar K+ outward rectifying channel e_value:0</td><td>0.211</td><td>(0.1144 0.5428)</td><td>0.1144</td><td>0.5428</td></tr>
<tr><td>2879</td><td>WT007_D06_CLUSTER_17745</td><td>gi|51970620|dbj|BAD44002.1| unknown protein [Arabidopsis thaliana] e_value:0</td><td>11682.m00488|LOC_Os05g05450|protein hypothetical protein e_value:0</td><td>0.189</td><td>(0.1226 0.6479)</td><td>0.1226</td><td>0.6479</td></tr>
<tr><td>2880</td><td>WT006_I20_CLUSTER_26161</td><td>gi|19070613|gb|AAL83927.1| D-type cyclin [Zea mays] e_value:4.23145e-104</td><td>11669.m01013|LOC_Os03g10650|protein Cyclin, N-terminal domain, putative e_value:8.467e-90</td><td>0.439</td><td>(0.2349 0.5352)</td><td>0.2349</td><td>0.5352</td></tr>
<tr><td>2881</td><td>WT010_C02_CLUSTER_29033</td><td>gi|57900571|dbj|BAD87023.1| putative farnesyltransferase beta subunit [Oryza sativa (japonica cultivar-group)] e_value:0</td><td>11667.m05264|LOC_Os01g53600|protein beta subunit of protein farnesyl transferase ERA1 e_value:0</td><td>0.231</td><td>(0.1127 0.4873)</td><td>0.1127</td><td>0.4873</td></tr>
<tr><td>2882</td><td>WT006_P15_CLUSTER_33543</td><td>gi|31432870|gb|AAP54446.1| putative kinase [Oryza sativa (japonica cultivar-group)] >gnl|BL_ORD_ID|2012520 gi|37535714|ref|NP_922159.1| putative kinase [Oryza sativa (japonica cultivar-group)] >gnl|BL_ORD_ID|2012520</td><td>11667.m04281|LOC_Os01g44110|protein Protein kinase domain, putative e_value:0</td><td>0.081</td><td>(0.0430 0.5282)</td><td>0.043</td><td>0.5282</td></tr>
<tr><td>2883</td><td>SET5_A09_CLUSTER_23753</td><td>gi|57899680|dbj|BAD87386.1| putative choline kinase CK2 [Oryza sativa (japonica cultivar-group)] e_value:4.92539e-174</td><td>11667.m05078|LOC_Os01g51920|protein Choline kinase N terminus, putative e_value:1.05443e-175</td><td>0.169</td><td>(0.0856 0.5066)</td><td>0.0856</td><td>0.5066</td></tr>
<tr><td>2884</td><td>SET4_E08_CLUSTER_21081</td><td>gi|498742|emb|CAA84023.1| beta-ketoacyl-ACP synthase [Hordeum vulgare subsp. vulgare] >gi|629774|pir||S47076 3-oxoacyl-[acyl-carrier-protein] synthase (EC 2.3.1.41) precursor [similarity] - barley e_value:0</td><td>11673.m04154|LOC_Os07g42420|protein Beta-ketoacyl synthase, N-terminal domain, putative e_value:0</td><td>0.139</td><td>(0.1003 0.7214)</td><td>0.1003</td><td>0.7214</td></tr>
<tr><td>2885</td><td>WT007_G16_CLUSTER_7155</td><td>gi|54290849|dbj|BAD61510.1| lanatoside 15'-O-acetylesterase-like [Oryza sativa (japonica cultivar-group)] >gnl|BL_ORD_ID|265470 gi|54290275|dbj|BAD61220.1| lanatoside 15'-O-acetylesterase-like [Oryza sativa (japonica cultivar-group)] e_value:</td><td>11667.m04127|LOC_Os01g42730|protein GDSL-like Lipase/Acylhydrolase, putative e_value:0</td><td>0.154</td><td>(0.1010 0.6546)</td><td>0.101</td><td>0.6546</td></tr>
<tr><td>2886</td><td>WT006_C04_CLUSTER_28537</td><td>gi|22748323|gb|AAN05325.1| Putative beta-1,3-glucanase [Oryza sativa (japonica cultivar-group)] e_value:0</td><td>11669.m01171|LOC_Os03g12140|protein Glycosyl hydrolases family 17, putative e_value:0</td><td>0.198</td><td>(0.0592 0.2984)</td><td>0.0592</td><td>0.2984</td></tr>
<tr><td>2887</td><td>WT003_E24_CLUSTER_34458</td><td>gi|53828635|gb|AAU94427.1| At1g58110 [Arabidopsis thaliana] >gnl|BL_ORD_ID|1283918 gi|28393180|gb|AAO42021.1| putative bZIP family transcription factor [Arabidopsis thaliana] e_value:2.5332e-61</td><td>11687.m01060|LOC_Os11g11100|protein bZIP family transcription factor, putative e_value:2.63735e-152</td><td>0.147</td><td>(0.0923 0.6299)</td><td>0.0923</td><td>0.6299</td></tr>
<tr><td>2888</td><td>WT005_K09_CLUSTER_13092</td><td>gi|50918315|ref|XP_469554.1| TGF beta receptor associated protein-like protein [Oryza sativa (japonica cultivar-group)] >gnl|BL_ORD_ID|1537110 gi|18071340|gb|AAL58199.1| TGF beta receptor associated protein-like protein [Oryza sativa (japonica cultivar</td><td>11669.m05114|LOC_Os03g50740|protein TGF beta receptor associated protein-like protein e_value:0</td><td>0.13</td><td>(0.0607 0.4673)</td><td>0.0607</td><td>0.4673</td></tr>
<tr><td>2889</td><td>WT004_H07_CLUSTER_19530</td><td>gi|21314227|gb|AAM44081.1| type IIB calcium ATPase MCA5 [Medicago truncatula] e_value:0</td><td>11669.m01012|LOC_Os03g10640|protein calcium-translocating P-type ATPase, PMCA-type e_value:0</td><td>0.07</td><td>(0.0581 0.8256)</td><td>0.0581</td><td>0.8256</td></tr>
<tr><td>2890</td><td>WT006_D01_CLUSTER_33548</td><td>gi|50934647|ref|XP_476851.1| bifunctional phosphopantetheine adenylyl transferase dephospho CoA kinase-like protein [Oryza sativa (japonica cultivar-group)] >gnl|BL_ORD_ID|1521842 gi|34393473|dbj|BAC83033.1| bifunctional phosphopantetheine adenylyl tra</td><td>11673.m00753|LOC_Os07g08210|protein cytidylyltransferase-related domain, putative e_value:7.11313e-82</td><td>0.28</td><td>(0.0995 0.3558)</td><td>0.0995</td><td>0.3558</td></tr>
<tr><td>2891</td><td>WT003_J19_CLUSTER_31734</td><td>gi|50838909|gb|AAT81670.1| putative glycine rich protein [Oryza sativa (japonica cultivar-group)] >gnl|BL_ORD_ID|1025933 gi|50582683|gb|AAT78753.1| hypothetical protein [Oryza sativa (japonica cultivar-group)] e_value:1.95305e-111</td><td>11686.m03808|LOC_Os12g38620|protein Bromodomain associated, putative e_value:5.42799e-164</td><td>0.178</td><td>(0.1176 0.6620)</td><td>0.1176</td><td>0.662</td></tr>
<tr><td>2892</td><td>WT009_C11_CLUSTER_25834</td><td>gi|50913029|ref|XP_467922.1| unknown protein [Oryza sativa (japonica cultivar-group)] >gnl|BL_ORD_ID|1583039 gi|46805892|dbj|BAD17205.1| unknown protein [Oryza sativa (japonica cultivar-group)] e_value:7.59596e-157</td><td>11680.m01077|LOC_Os06g11070|protein expressed protein e_value:2.29896e-142</td><td>0.198</td><td>(0.1465 0.7388)</td><td>0.1465</td><td>0.7388</td></tr>
<tr><td>2893</td><td>SET6_I11_CLUSTER_15666</td><td>gi|19387263|gb|AAL87174.1| putative apospory-associated protein C [Oryza sativa (japonica cultivar-group)] >gi|50929321|ref|XP_474188.1| OSJNBa0011F23.4 [Oryza sativa (japonica cultivar-group)] >gi|38346398|emb|CAE04231.2|</td><td>11670.m05550|LOC_Os04g56290|protein Aldose 1-epimerase e_value:3.36279e-173</td><td>0.191</td><td>(0.0609 0.3183)</td><td>0.0609</td><td>0.3183</td></tr>
<tr><td>2894</td><td>WT011_J15_CLUSTER_34072</td><td>gi|50910599|ref|XP_466788.1| kinetochore protein-like [Oryza sativa (japonica cultivar-group)] >gnl|BL_ORD_ID|1578659 gi|51964170|ref|XP_506869.1| PREDICTED OJ1372_D06.21 gene product [Oryza sativa (japonica cultivar-group)]</td><td>11668.m03920|LOC_Os02g40680|protein Mis12 protein, putative e_value:2.83139e-64</td><td>0.532</td><td>(0.2548 0.4793)</td><td>0.2548</td><td>0.4793</td></tr>
<tr><td>2895</td><td>WT013_J05_CLUSTER_32013</td><td>gi|11037534|gb|AAG27623.1| starch branching enzyme 2 [Triticum aestivum] e_value:0</td><td>11668.m03075|LOC_Os02g32660|protein starch branching enzyme isoform RBE3 - rice e_value:0</td><td>0.145</td><td>(0.1351 0.9287)</td><td>0.1351</td><td>0.9287</td></tr>
<tr><td>2896</td><td>SET3_O23_CLUSTER_9499</td><td>gi|34914608|ref|NP_918651.1| P0520B06.17 [Oryza sativa (japonica cultivar-group)] e_value:2.12064e-80</td><td>11667.m03091|LOC_Os01g32770|protein expressed protein e_value:5.30311e-82</td><td>0.653</td><td>(0.3373 0.5163)</td><td>0.3373</td><td>0.5163</td></tr>
<tr><td>2897</td><td>SET5_K03_CLUSTER_34504</td><td>gi|50911573|ref|XP_467194.1| putative aspartate-tRNA ligase [Oryza sativa (japonica cultivar-group)] >gi|51979332|ref|XP_507519.1| PREDICTED OJ1717_A09.34 gene product [Oryza sativa (japonica cultivar-group)] >gi|51964226|ref|XP_506897.1| PREDICTED</td><td>11668.m04459|LOC_Os02g46130|protein aspartyl-tRNA synthetase e_value:0</td><td>0.091</td><td>(0.0515 0.5626)</td><td>0.0515</td><td>0.5626</td></tr>
<tr><td>2898</td><td>WT009_G10_CLUSTER_20998</td><td>gi|50915564|ref|XP_468246.1| putative leucine aminopeptidase [Oryza sativa (japonica cultivar-group)] >gnl|BL_ORD_ID|1583367 gi|47497603|dbj|BAD19673.1| putative leucine aminopeptidase [Oryza sativa (japonica cultivar-group)] >gnl|BL_ORD_ID|1583367</td><td>11668.m05434|LOC_Os02g55140|protein cytosol aminopeptidase (ec 3.4.11.1) (leucine aminopeptidase) (lap)(leucyl aminopeptidase) (proline aminopeptidase) (ec 3.4.11.5)</td><td>0.155</td><td>(0.1124 0.7249)</td><td>0.1124</td><td>0.7249</td></tr>
</table>

PDF Complete
Your complimentary use period has ended. Thank you for using PDF Complete.
Click Here to upgrade to Unlimited Pages and Expanded Features

<table>
<tr><th></th><th></th><th></th><th></th><th></th><th></th><th></th><th></th></tr>
<tr><td>2899</td><td>WT007_H03_CLUSTER_12091</td><td>gi|34912398|ref|NP_917546.1| putative Mla1 [Oryza sativa (japonica cultivar-group)] e_value:0</td><td>11667.m04037|LOC_Os01g41890|protein NB-ARC domain, putative e_value:0</td><td>0.322</td><td>(0.4292 1.3330)</td><td>0.4292</td><td>1.333</td></tr>
<tr><td>2900</td><td>SET2_I04_CLUSTER_8244</td><td>gi|50931613|ref|XP_475334.1| putative plastid division protein ftsZ [Oryza sativa (japonica cultivar-group)] >gi|50080277|gb|AAT69612.1| putative cell division protein FtsZ [Oryza sativa (japonica cultivar-group)] e_value:0</td><td>11682.m03486|LOC_Os05g37160|protein cell division protein FtsZ, putative e_value:0</td><td>0.173</td><td>(0.1040 0.6030)</td><td>0.104</td><td>0.603</td></tr>
<tr><td>2901</td><td>WT009_B19_CLUSTER_18951</td><td>gi|38346756|emb|CAE03861.2| OSJNBa0081C01.7 [Oryza sativa (japonica cultivar-group)] >gnl|BL_ORD_ID|1535262 gi|38346625|emb|CAD41208.2| OSJNBa0074L08.19 [Oryza sativa (japonica cultivar-group)] >gnl|BL_ORD_ID|1535262 gi|50926648|ref|XP_473271.1| OSJ</td><td>11670.m04344|LOC_Os04g44800|protein hypothetical protein e_value:4.55722e-136</td><td>0.168</td><td>(0.1080 0.6411)</td><td>0.108</td><td>0.6411</td></tr>
<tr><td>2902</td><td>WT002_P21_CLUSTER_9336</td><td>gi|55416165|gb|AAV50044.1| stem-specific protein [Saccharum hybrid cultivar] e_value:2.12504e-23</td><td>11681.m02865|LOC_Os09g31080|protein expressed protein e_value:3.52998e-20</td><td>0.64</td><td>(0.4437 0.6929)</td><td>0.4437</td><td>0.6929</td></tr>
<tr><td>2903</td><td>WT008_D05_CLUSTER_10729</td><td>gi|50939295|ref|XP_479175.1| unknown protein [Oryza sativa (japonica cultivar-group)] >gnl|BL_ORD_ID|1530528 gi|33146876|dbj|BAC79874.1| unknown protein [Oryza sativa (japonica cultivar-group)] >gnl|BL_ORD_ID|1530528 gi|33146872|dbj|BAC79870.1| unkn</td><td>11673.m04178|LOC_Os07g42640|protein FYVE zinc finger, putative e_value:2.40829e-148</td><td>0.128</td><td>(0.0707 0.5535)</td><td>0.0707</td><td>0.5535</td></tr>
<tr><td>2904</td><td>SET2_F14_CLUSTER_21313</td><td>gi|34903948|ref|NP_913321.1| OSJNBa0038J17.19 [Oryza sativa (japonica cultivar-group)] >gi|14164476|dbj|BAB55727.1| unknown protein [Oryza sativa (japonica cultivar-group)] >gi|7523515|dbj|BAA94243.1| unknown protein [Oryza sativa (japonica cultivar</td><td>11667.m01126|LOC_Os01g11570|protein GDSL-like Lipase/Acylhydrolase e_value:1.37917e-113</td><td>0.223</td><td>(0.1117 0.4998)</td><td>0.1117</td><td>0.4998</td></tr>
<tr><td>2905</td><td>SET3_P06_CLUSTER_1036</td><td>gi|50917797|ref|XP_469295.1| unknown protein [Oryza sativa (japonica cultivar-group)] >gi|29150393|gb|AAO72402.1| unknown protein [Oryza sativa (japonica cultivar-group)] >gi|50881425|gb|AAT85270.1| expressed protein [Oryza sativa (japonica cultivar</td><td>11669.m04760|LOC_Os03g47550|protein expressed protein e_value:9.43239e-119</td><td>0.07</td><td>(0.0365 0.5230)</td><td>0.0365</td><td>0.523</td></tr>
<tr><td>2906</td><td>WT012_H07_CLUSTER_27742</td><td>gi|50920603|ref|XP_470662.1| Putative phosphate/phosphoenolpyruvate translocator protein [Oryza sativa (japonica cultivar-group)] >gnl|BL_ORD_ID|1588853 gi|27476065|gb|AAO16996.1| Putative phosphate/phosphoenolpyruvate translocator protein [Oryza sativ</td><td>11669.m01793|LOC_Os03g17740|protein expressed protein e_value:9.26925e-173</td><td>0.078</td><td>(0.0284 0.3657)</td><td>0.0284</td><td>0.3657</td></tr>
<tr><td>2907</td><td>WT013_E11_CLUSTER_146</td><td>gi|56785168|dbj|BAD81844.1| putative ring finger protein 5 [Oryza sativa (japonica cultivar-group)] >gnl|BL_ORD_ID|776240 gi|56202103|dbj|BAD73632.1| putative ring finger protein 5 [Oryza sativa (japonica cultivar-group)] e_value:0</td><td>11682.m03735|LOC_Os05g39380|protein At3g58030, putative e_value:0</td><td>0.216</td><td>(0.1189 0.5501)</td><td>0.1189</td><td>0.5501</td></tr>
<tr><td>2908</td><td>WT012_L23_CLUSTER_33428</td><td>gi|50912565|ref|XP_467690.1| putative dynamin homolog [Oryza sativa (japonica cultivar-group)] >gnl|BL_ORD_ID|1582800 gi|46390555|dbj|BAD16041.1| putative dynamin homolog [Oryza sativa (japonica cultivar-group)] e_value:0</td><td>11668.m04924|LOC_Os02g50550|protein Dynamin central region, putative e_value:0</td><td>0.137</td><td>(0.0619 0.4534)</td><td>0.0619</td><td>0.4534</td></tr>
<tr><td>2909</td><td>WT005_M03_CLUSTER_34416</td><td>gi|50915634|ref|XP_468281.1| putative ribonucleotide reductase [Oryza sativa (japonica cultivar-group)] >gnl|BL_ORD_ID|1584166 gi|47497381|dbj|BAD19419.1| putative ribonucleotide reductase [Oryza sativa (japonica cultivar-group)] e_value:0</td><td>11668.m05535|LOC_Os02g56100|protein ribonucleotide reductase large subunit A e_value:0</td><td>0.099</td><td>(0.0461 0.4640)</td><td>0.0461</td><td>0.464</td></tr>
<tr><td>2910</td><td>SET4_J06_CLUSTER_19727</td><td>gi|50926362|ref|XP_473128.1| OSJNBb0065L13.4 [Oryza sativa (japonica cultivar-group)] >gi|32488295|emb|CAE03361.1| OSJNBb0065L13.4 [Oryza sativa (japonica cultivar-group)] e_value:5.32939e-78</td><td>11670.m04155|LOC_Os04g43040|protein expressed protein e_value:1.2009e-79</td><td>0.303</td><td>(0.1765 0.5826)</td><td>0.1765</td><td>0.5826</td></tr>
<tr><td>2911</td><td>SET4_J17_CLUSTER_17845</td><td>gi|24308633|gb|AAN52756.1| Unknown protein [Oryza sativa (japonica cultivar-group)] >gi|34902312|ref|NP_912502.1| Unknown protein [Oryza sativa (japonica cultivar-group)] e_value:0</td><td>11669.m01214|LOC_Os03g12530|protein cation diffusion facilitator 8 e_value:0</td><td>0.076</td><td>(0.0360 0.4704)</td><td>0.036</td><td>0.4704</td></tr>
<tr><td>2912</td><td>WT013_L21_CLUSTER_15789</td><td>gi|55773805|dbj|BAD72343.1| unknown protein [Oryza sativa (japonica cultivar-group)] e_value:0</td><td>11667.m04586|LOC_Os01g47460|protein expressed protein</td><td>0.187</td><td>(0.1071 0.5732)</td><td>0.1071</td><td>0.5732</td></tr>
<tr><td>2913</td><td>WT007_F19_CLUSTER_22262</td><td>gi|22002966|emb|CAD43075.1| putative CENP-E like kinetochore protein [Hordeum vulgare subsp. vulgare] e_value:0</td><td>11682.m00730|LOC_Os05g07680|protein hypothetical protein e_value:0</td><td>0.313</td><td>(0.1716 0.5480)</td><td>0.1716</td><td>0.548</td></tr>
<tr><td>2914</td><td>SET4__C19_CLUSTER_31842</td><td>gi|52077496|dbj|BAD45140.1| putative calmodulin-binding protein [Oryza sativa (japonica cultivar-group)] e_value:0</td><td>11667.m03732|LOC_Os01g38980|protein IQ calmodulin-binding motif, putative e_value:0</td><td>0.15</td><td>(0.1098 0.7341)</td><td>0.1098</td><td>0.7341</td></tr>
<tr><td>2915</td><td>WT004_M08_CLUSTER_24894</td><td>gi|9294583|dbj|BAB02864.1| prolyl 4-hydroxylase alpha subunit-like protein [Arabidopsis thaliana] e_value:8.95701e-116</td><td>11669.m05974|LOC_Os03g58890|protein putative oxidoreductase e_value:1.47467e-113</td><td>0.14</td><td>(0.2568 1.8337)</td><td>0.2568</td><td>1.8337</td></tr>
<tr><td>2916</td><td>SET2_K20_CLUSTER_15465</td><td>gi|50915458|ref|XP_468193.1| putative Splicing factor, arginine/serine-rich 7 [Oryza sativa (japonica cultivar-group)] >gi|51964466|ref|XP_507018.1| PREDICTED OSJNBa0054K20.35 gene product [Oryza sativa (japonica cultivar-group)] >gi|47497773|dbj|BA</td><td>11668.m05392|LOC_Os02g54770|protein Transposable element protein, putative e_value:2.38991e-80</td><td>0.179</td><td>(0.0715 0.3994)</td><td>0.0715</td><td>0.3994</td></tr>
<tr><td>2917</td><td>WT010_I22_CLUSTER_550</td><td>gi|21592409|gb|AAM64360.1| unknown [Arabidopsis thaliana] e_value:8.11066e-84</td><td>11680.m04783|LOC_Os06g48320|protein expressed protein e_value:6.68917e-147</td><td>0.275</td><td>(0.1170 0.4255)</td><td>0.117</td><td>0.4255</td></tr>
<tr><td>2918</td><td>SET6_E09_CLUSTER_515</td><td>gi|31433120|gb|AAP54673.1| putative shaggy-like kinase [Oryza sativa (japonica cultivar-group)] >gi|37536168|ref|NP_922386.1| putative shaggy-like kinase [Oryza sativa (japonica cultivar-group)] >gi|22122918|gb|AAM92301.1| putative shaggy-like kinas</td><td>11676.m03412|LOC_Os10g37740|protein putative shaggy-like kinase e_value:0</td><td>0.096</td><td>(0.0374 0.3903)</td><td>0.0374</td><td>0.3903</td></tr>
<tr><td>2919</td><td>WT002_A17_CLUSTER_33889</td><td>gi|38345258|emb|CAD41102.2| OSJNBb0011N17.19 [Oryza sativa (japonica cultivar-group)] >gnl|BL_ORD_ID|1522085 gi|50925243|ref|XP_472923.1| OSJNBb0011N17.19 [Oryza sativa (japonica cultivar-group)] e_value:4.38833e-66</td><td>11686.m01900|LOC_Os12g19090|protein expressed protein e_value:3.65237e-74</td><td>0.431</td><td>(0.2473 0.5742)</td><td>0.2473</td><td>0.5742</td></tr>
<tr><td>2920</td><td>WT004_E16_CLUSTER_34874</td><td>gi|29409364|gb|AAM29178.1| biostress-resistance-related protein [Triticum aestivum] e_value:2.14123e-180</td><td>11667.m00741|LOC_Os01g07960|protein Phospholipase/Carboxylesterase e_value:3.79516e-136</td><td>0.102</td><td>(0.0558 0.5446)</td><td>0.0558</td><td>0.5446</td></tr>
<tr><td>2921</td><td>WT003_B17_CLUSTER_34595</td><td>gi|435175|emb|CAA53397.1| t complex polypeptide 1 [Avena sativa] >gnl|BL_ORD_ID|2378813 gi|1729864|sp|P54411|TCPE2_AVESA T-complex protein 1, epsilon subunit (TCP-1-epsilon) (CCT-epsilon) (TCP-K36) e_value:0</td><td>11680.m03561|LOC_Os06g36700|protein T-complex protein 1, epsilon subunit e_value:0</td><td>0.079</td><td>(0.0414 0.5263)</td><td>0.0414</td><td>0.5263</td></tr>
<tr><td>2922</td><td>WT003_J09_CLUSTER_33304</td><td>gi|55168203|gb|AAV44069.1| putative DNA-binding protein GBP16 [Oryza sativa (japonica cultivar-group)] e_value:0</td><td>11682.m02647|LOC_Os05g28280|protein probable DNA-binding protein GBP16 - rice e_value:0</td><td>0.111</td><td>(0.0705 0.6355)</td><td>0.0705</td><td>0.6355</td></tr>
<tr><td>2923</td><td>SET3_G02_CLUSTER_34135</td><td>gi|56201958|dbj|BAD73408.1| putative Ruvbl1 protein [Oryza sativa (japonica cultivar-group)] e_value:0</td><td>11673.m00748|LOC_Os07g08170|protein TIP49 C-terminus, putative e_value:0</td><td>0.065</td><td>(0.0341 0.5209)</td><td>0.0341</td><td>0.5209</td></tr>
<tr><td>2924</td><td>WT006_H09_CLUSTER_34880</td><td>gi|30984550|gb|AAP42738.1| At5g02270 [Arabidopsis thaliana] >gnl|BL_ORD_ID|1857002 gi|7406423|emb|CAB85532.1| ABC transporter-like protein [Arabidopsis thaliana] >gnl|BL_ORD_ID|1857002 gi|21554262|gb|AAM63337.1| ABC transporter-like protein [Arabido</td><td>11669.m02055|LOC_Os03g20170|protein ABC transporter-like protein - Arabidopsis thaliana e_value:2.3384e-165</td><td>0.078</td><td>(0.0415 0.5328)</td><td>0.0415</td><td>0.5328</td></tr>
<tr><td>2925</td><td>WT007_H04_CLUSTER_25284</td><td>gi|50904785|ref|XP_463881.1| putative 15.9 kDa subunit of RNA polymerase II [Oryza sativa (japonica cultivar-group)] >gnl|BL_ORD_ID|1565830 gi|41052832|dbj|BAD07723.1| putative 15.9 kDa subunit of RNA polymerase II [Oryza sativa (japonica cultivar-</td><td>11668.m00162|LOC_Os02g02510|protein RNA polymerase Rpb4 e_value:1.3039e-60</td><td>0.309</td><td>(0.1076 0.3485)</td><td>0.1076</td><td>0.3485</td></tr>
<tr><td>2926</td><td>WT007_B05_CLUSTER_18321</td><td>gi|9758225|dbj|BAB08724.1| receptor-protein kinase-like protein [Arabidopsis thaliana] >gnl|BL_ORD_ID|1090432 gi|15237872|ref|NP_197789.1| protein kinase family protein [Arabidopsis thaliana] e_value:1.09971e-69</td><td>11669.m00260|LOC_Os03g03290|protein expressed protein e_value:0</td><td>0.313</td><td>(0.1353 0.4319)</td><td>0.1353</td><td>0.4319</td></tr>
<tr><td>2927</td><td>WT005_A22_CLUSTER_31693</td><td>gi|50937781|ref|XP_478418.1| putative RNA Binding Protein [Oryza sativa (japonica cultivar-group)] e_value:0</td><td>11673.m03192|LOC_Os07g33330|protein RNA recognition motif. (a.k.a. RRM, RBD, or RNP domain), putative e_value:0</td><td>0.339</td><td>(0.1395 0.4122)</td><td>0.1395</td><td>0.4122</td></tr>
<tr><td>2928</td><td>WT005_J08_CLUSTER_28527</td><td>gi|50909329|ref|XP_466153.1| unknown protein [Oryza sativa (japonica cultivar-group)] >gnl|BL_ORD_ID|1574621 gi|50725754|dbj|BAD33265.1| unknown protein [Oryza sativa (japonica cultivar-group)] >gnl|BL_ORD_ID|1574621 gi|46390702|dbj|BAD16203.1| unkn</td><td>11668.m03269|LOC_Os02g34540|protein TPR Domain, putative e_value:4.98376e-142</td><td>0.19</td><td>(0.1291 0.6785)</td><td>0.1291</td><td>0.6785</td></tr>
<tr><td>2929</td><td>SET5_G03_CLUSTER_20582</td><td>gi|30683535|ref|NP_196685.2| transducin family protein / WD-40 repeat family protein [Arabidopsis thaliana] e_value:3.88842e-142</td><td>11669.m01911|LOC_Os03g18840|protein expressed protein</td><td>0.174</td><td>(0.1063 0.6111)</td><td>0.1063</td><td>0.6111</td></tr>
<tr><td>2930</td><td>SET6_M05_CLUSTER_31913</td><td>gi|50934609|ref|XP_476832.1| putative z-protein [Oryza sativa (japonica cultivar-group)] >gi|50509166|dbj|BAD30317.1| putative z-protein [Oryza sativa (japonica cultivar-group)] >gi|34393841|dbj|BAC83445.1| putative z-protein [Oryza sativa (japonica</td><td>11673.m00738|LOC_Os07g08070|protein Uncharacterized protein family UPF0005 e_value:5.06483e-100</td><td>0.306</td><td>(0.1604 0.5243)</td><td>0.1604</td><td>0.5243</td></tr>
<tr><td>2931</td><td>SET1_M07_CLUSTER_33294</td><td>gi|55167983|gb|AAV43851.1| unknown protein [Oryza sativa (japonica cultivar-group)] e_value:2.92115e-104</td><td>11682.m02721|LOC_Os05g28980|protein expressed protein e_value:2.75227e-63</td><td>0.668</td><td>(0.3622 0.5425)</td><td>0.3622</td><td>0.5425</td></tr>
<tr><td>2932</td><td>WT007_D23_CLUSTER_33776</td><td>gi|55775032|ref|XP_550442.1| calmodulin-binding protein-like [Oryza sativa (japonica cultivar-group)] >gnl|BL_ORD_ID|1752783 gi|55295828|dbj|BAD67696.1| calmodulin-binding protein-like [Oryza sativa (japonica cultivar-group)] e_value:3.1969e-149</td><td>11680.m00052|LOC_Os06g01500|protein IQ calmodulin-binding motif, putative e_value:8.01994e-151</td><td>0.307</td><td>(0.1848 0.6024)</td><td>0.1848</td><td>0.6024</td></tr>
<tr><td>2933</td><td>SET5__C21_CLUSTER_3207</td><td>gi|27461140|gb|AAL67139.1| thioredoxin H [Triticum aestivum] e_value:3.77422e-62</td><td>11673.m00823|LOC_Os07g08840|protein thioredoxin, putative e_value:8.17881e-49</td><td>0.172</td><td>(0.1192 0.6925)</td><td>0.1192</td><td>0.6925</td></tr>
<tr><td>2934</td><td>WT009_B09_CLUSTER_18394</td><td>gi|50913253|ref|XP_468034.1| putative 1-aminocyclopropane-1-carboxylate deaminase [Oryza sativa (japonica cultivar-group)] >gnl|BL_ORD_ID|1583151 gi|46805374|dbj|BAD16875.1| putative 1-aminocyclopropane-1-carboxylate deaminase [Oryza sativa (japonica</td><td>11668.m05234|LOC_Os02g53330|protein pyridoxal phosphate-dependent enzymes, D-cysteine desulfhydrase family e_value:0</td><td>0.099</td><td>(0.0509 0.5148)</td><td>0.0509</td><td>0.5148</td></tr>
<tr><td>2935</td><td>WT004_K16_CLUSTER_31787</td><td>gi|21536525|gb|AAM60857.1| dihydrolipoamide S-acetyltransferase, putative [Arabidopsis thaliana] e_value:4.39942e-173</td><td>11686.m00772|LOC_Os12g08170|protein Similar to dihydrolipoamide S-acetyltransferase, putative e_value:0</td><td>0.132</td><td>(0.0860 0.6536)</td><td>0.086</td><td>0.6536</td></tr>
<tr><td>2936</td><td>SET2__C02_CLUSTER_20309</td><td>gi|23306125|gb|AAN17392.1| Putative mitotic control protein dis3 [Oryza sativa (japonica cultivar-group)] e_value:0</td><td>11669.m00305|LOC_Os03g03710|protein RNB-like protein, putative e_value:0</td><td>0.091</td><td>(0.0451 0.4939)</td><td>0.0451</td><td>0.4939</td></tr>
<tr><td>2937</td><td>WT008_K09_CLUSTER_26582</td><td>gi|50941635|ref|XP_480345.1| putative RAE1 (RNA export 1, S.pombe) homolog [Oryza sativa (japonica cultivar-group)] >gnl|BL_ORD_ID|1602761 gi|38636974|dbj|BAD03234.1| putative RAE1 (RNA export 1, S.pombe) homolog [Oryza sativa (japonica cultivar-group)</td><td>11674.m00733|LOC_Os08g07960|protein hypothetical protein e_value:0</td><td>0.05</td><td>(0.0368 0.7290)</td><td>0.0368</td><td>0.729</td></tr>
<tr><td>2938</td><td>WT003_O02_CLUSTER_33088</td><td>gi|50947853|ref|XP_483454.1| putative transmembrane protein(TPA regulated locus protein) [Oryza sativa (japonica cultivar-group)] >gnl|BL_ORD_ID|1609779 gi|42407963|dbj|BAD09101.1| putative transmembrane protein(TPA regulated locus protein) [Oryza sati</td><td>11674.m04195|LOC_Os08g41670|protein Uncharacterized protein family UPF0016, putative e_value:3.5651e-125</td><td>0.154</td><td>(0.0936 0.6074)</td><td>0.0936</td><td>0.6074</td></tr>
<tr><td>2939</td><td>SET2_L07_CLUSTER_32909</td><td>gi|56783940|dbj|BAD81377.1| ubiquitin-conjugating enzyme -like [Oryza sativa (japonica cultivar-group)] e_value:7.90383e-152</td><td>11667.m01307|LOC_Os01g13280|protein Ubiquitin-conjugating enzyme, putative e_value:2.05646e-153</td><td>0.338</td><td>(0.1692 0.5006)</td><td>0.1692</td><td>0.5006</td></tr>
<tr><td>2940</td><td>WT006_F04_CLUSTER_7530</td><td>gi|34913150|ref|NP_917922.1| putative histone acetyltransferase [Oryza sativa (japonica cultivar-group)] >gnl|BL_ORD_ID|1530901 gi|22093781|dbj|BAC07072.1| putative histone acetyltransferase [Oryza sativa (japonica cultivar-group)] e_value:0</td><td>11673.m04254|LOC_Os07g43360|protein MOZ/SAS family, putative e_value:0</td><td>0.045</td><td>(0.0202 0.4482)</td><td>0.0202</td><td>0.4482</td></tr>
<tr><td>2941</td><td>WT006_L07_CLUSTER_32524</td><td>gi|34902994|ref|NP_912844.1| unnamed protein product [Oryza sativa (japonica cultivar-group)] >gnl|BL_ORD_ID|1752333 gi|9558529|dbj|BAB03447.1| putative NAM [Oryza sativa (japonica cultivar-group)] >gnl|BL_ORD_ID|1752333 gi|7228440|dbj|BAA92400.1|</td><td>11667.m01564|LOC_Os01g15640|protein No apical meristem (NAM) protein, putative e_value:1.10373e-149</td><td>0.425</td><td>(0.2639 0.6214)</td><td>0.2639</td><td>0.6214</td></tr>
</table>

PDF Complete
Your complimentary use period has ended. Thank you for using PDF Complete.
Click Here to upgrade to Unlimited Pages and Expanded Features

<table>
<tr><td>2942</td><td>WT008_P20_CLUSTER_15511</td><td>gi|52075842|dbj|BAD45450.1| OTU-like cysteine protease-like [Oryza sativa (japonica cultivar-group)] e_value:2.23664e-118</td><td>11680.m04520|LOC_Os06g45850|protein OTU-like cysteine protease, putative e_value:4.07985e-120</td><td>0.11</td><td>(0.0545 0.4966)</td><td>0.0545</td><td>0.4966</td></tr>
<tr><td>2943</td><td>WT008_P15_CLUSTER_32901</td><td>gi|27368877|emb|CAD59596.1| MRP-like ABC transporter [Oryza sativa (japonica cultivar-group)] >gnl|BL_ORD_ID|1415321 gi|53792125|dbj|BAD52758.1| putative AtMRP4 [Oryza sativa (japonica cultivar-group)] e_value:0</td><td>11670.m01191|LOC_Os04g13220|protein ABC transporter, putative e_value:0</td><td>0.183</td><td>(0.3484 1.9016)</td><td>0.3484</td><td>1.9016</td></tr>
<tr><td>2944</td><td>WT007_D18_CLUSTER_15337</td><td>gi|50917247|ref|XP_469020.1| putative translation elongation factor [Oryza sativa (japonica cultivar-group)] >gnl|BL_ORD_ID|1585078 gi|62733415|gb|AAX95532.1| Putative Translation Elongation factor protein [Oryza sativa (japonica cultivar-group)] e_val</td><td>11669.m04464|LOC_Os03g44780|protein putative translation elongation factor e_value:0</td><td>0.181</td><td>(0.0914 0.5038)</td><td>0.0914</td><td>0.5038</td></tr>
<tr><td>2945</td><td>WT005_J13_CLUSTER_34876</td><td>gi|50940045|ref|XP_479550.1| putative receptor-like protein kinase [Oryza sativa (japonica cultivar-group)] >gnl|BL_ORD_ID|1534273 gi|51979072|ref|XP_507413.1| PREDICTED OSJNBa0008J01.18 gene product [Oryza sativa (japonica cultivar-group)] >gnl|BL_</td><td>11669.m02206|LOC_Os03g21510|protein Protein kinase domain, putative e_value:0</td><td>0.144</td><td>(0.0874 0.6086)</td><td>0.0874</td><td>0.6086</td></tr>
<tr><td>2946</td><td>WT010_C14_CLUSTER_24562</td><td>gi|50934247|ref|XP_476651.1| putative CBL-interacting protein kinase 23 [Oryza sativa (japonica cultivar-group)] >gnl|BL_ORD_ID|1521572 gi|34393400|dbj|BAC82911.1| putative CBL-interacting protein kinase 23 [Oryza sativa (japonica cultivar-</td><td>11673.m00479|LOC_Os07g05620|protein Protein kinase domain, putative e_value:0</td><td>0.043</td><td>(0.0268 0.6176)</td><td>0.0268</td><td>0.6176</td></tr>
<tr><td>2947</td><td>WT008_N08_CLUSTER_22840</td><td>gi|1076781|pir||A54415 transcription factor HBP-1a(c14) - wheat >gnl|BL_ORD_ID|2334143 gi|497895|dbj|BAA02304.1| transcription factor HBP-1a(c14) [Triticum aestivum] e_value:0</td><td>11669.m01332|LOC_Os03g13610|protein transcription factor HBP-1a(c14) - wheat e_value:0</td><td>0.232</td><td>(0.0859 0.3699)</td><td>0.0859</td><td>0.3699</td></tr>
<tr><td>2948</td><td>WT005_K03_CLUSTER_21694</td><td>gi|9759403|dbj|BAB09858.1| nuclear protein-like [Arabidopsis thaliana] >gnl|BL_ORD_ID|1098480 gi|15237657|ref|NP_201232.1| splicing factor, putative [Arabidopsis thaliana] e_value:0</td><td>11668.m00469|LOC_Os02g05310|protein Similar to nuclear protein e_value:0</td><td>0.066</td><td>(0.0300 0.4566)</td><td>0.03</td><td>0.4566</td></tr>
<tr><td>2949</td><td>WT004_G06_CLUSTER_32326</td><td>gi|31432537|gb|AAP54159.1| mitochondrial chaperonin-60 [Oryza sativa (japonica cultivar-group)] >gnl|BL_ORD_ID|2012046 gi|37535140|ref|NP_921872.1| mitochondrial chaperonin-60 [Oryza sativa (japonica cultivar-group)] >gnl|BL_ORD_ID|2012046 gi|227583</td><td>11676.m02851|LOC_Os10g32550|protein mitochondrial chaperonin-60 e_value:0</td><td>0.058</td><td>(0.0285 0.4865)</td><td>0.0285</td><td>0.4865</td></tr>
<tr><td>2950</td><td>SET6_C22_CLUSTER_15340</td><td>gi|53791335|dbj|BAD54714.1| putative early nodule-specific protein ENOD8 [Oryza sativa (japonica cultivar-group)] e_value:1.36579e-131</td><td>11667.m02198|LOC_Os01g22640|protein GDSL-like Lipase/Acylhydrolase, putative e_value:3.73792e-133</td><td>0.717</td><td>(0.3888 0.5422)</td><td>0.3888</td><td>0.5422</td></tr>
<tr><td>2951</td><td>WT002_A14_CLUSTER_32969</td><td>gi|50938251|ref|XP_478653.1| CHP-rich zinc finger protein-like [Oryza sativa (japonica cultivar-group)] >gnl|BL_ORD_ID|1528575 gi|33146953|dbj|BAC80026.1| CHP-rich zinc finger protein-like [Oryza sativa (japonica cultivar-group)] e_value:1.59065e-116</td><td>11673.m03540|LOC_Os07g36600|protein universal stress protein family, putative e_value:3.592e-118</td><td>0.207</td><td>(0.0903 0.4370)</td><td>0.0903</td><td>0.437</td></tr>
<tr><td>2952</td><td>WT011_D04_CLUSTER_34617</td><td>gi|34911800|ref|NP_917247.1| lipase-like protein [Oryza sativa (japonica cultivar-group)] >gnl|BL_ORD_ID|1553044 gi|20146410|dbj|BAB89190.1| lipase-like [Oryza sativa (japonica cultivar-group)] e_value:5.45981e-161</td><td>11667.m04436|LOC_Os01g46080|protein GDSL-like Lipase/Acylhydrolase e_value:2.79363e-169</td><td>0.346</td><td>(0.1704 0.4932)</td><td>0.1704</td><td>0.4932</td></tr>
<tr><td>2953</td><td>WT005_P21_CLUSTER_30515</td><td>gi|21689693|gb|AAM67468.1| unknown protein [Arabidopsis thaliana] >gnl|BL_ORD_ID|1208787 gi|20259547|gb|AAM13893.1| unknown protein [Arabidopsis thaliana] >gnl|BL_ORD_ID|1208787 gi|30682392|ref|NP_851033.1| expressed protein [Arabidopsis thaliana] &</td><td>11673.m02790|LOC_Os07g29450|protein expressed protein e_value:0</td><td>0.152</td><td>(0.0820 0.5402)</td><td>0.082</td><td>0.5402</td></tr>
<tr><td>2954</td><td>WT004_O15_CLUSTER_15679</td><td>gi|31433109|gb|AAP54662.1| unknown protein [Oryza sativa (japonica cultivar-group)] >gnl|BL_ORD_ID|2012693 gi|37536146|ref|NP_922375.1| unknown protein [Oryza sativa (japonica cultivar-group)] >gnl|BL_ORD_ID|2012693 gi|10122044|gb|AAG13433.1| unknow</td><td>11676.m03400|LOC_Os10g37630|protein expressed protein e_value:0</td><td>0.122</td><td>(0.0646 0.5277)</td><td>0.0646</td><td>0.5277</td></tr>
<tr><td>2955</td><td>WT005_F17_CLUSTER_32960</td><td>gi|50917137|ref|XP_468965.1| putative membrane protein [Oryza sativa (japonica cultivar-group)] >gnl|BL_ORD_ID|1585059 gi|29244652|gb|AAO73245.1| putative membrane protein [Oryza sativa (japonica cultivar-group)] e_value:0</td><td>11669.m03474|LOC_Os03g34300|protein putative membrane protein e_value:0</td><td>0.125</td><td>(0.0642 0.5146)</td><td>0.0642</td><td>0.5146</td></tr>
<tr><td>2956</td><td>WT011_A10_CLUSTER_33664</td><td>gi|20260148|gb|AAM12972.1| strong similarity to initiation factor eIF-2, gb|U37354 from S [Arabidopsis thaliana] >gnl|BL_ORD_ID|975285 gi|15219669|ref|NP_171913.1| eukaryotic translation initiation factor 2 subunit 3, putative / eIF2S3, putative / eIF-</td><td>11686.m04115|LOC_Os12g41400|protein translation initiation factor eIF-2 gamma chain F20D22.6 - Arabidopsis thaliana</td><td>0.026</td><td>(0.0139 0.5328)</td><td>0.0139</td><td>0.5328</td></tr>
<tr><td>2957</td><td>WT005_P23_CLUSTER_33664</td><td>gi|20260148|gb|AAM12972.1| strong similarity to initiation factor eIF-2, gb|U37354 from S [Arabidopsis thaliana] >gnl|BL_ORD_ID|975285 gi|15219669|ref|NP_171913.1| eukaryotic translation initiation factor 2 subunit 3, putative / eIF2S3, putative / eIF-</td><td>11686.m04115|LOC_Os12g41400|protein translation initiation factor eIF-2 gamma chain F20D22.6 - Arabidopsis thaliana</td><td>0.024</td><td>(0.0148 0.6089)</td><td>0.0148</td><td>0.6089</td></tr>
<tr><td>2958</td><td>WT009_G23_CLUSTER_34673</td><td>gi|50917709|ref|XP_469251.1| expressed protein [Oryza sativa (japonica cultivar-group)] >gnl|BL_ORD_ID|1539705 gi|40538933|gb|AAR87190.1| expressed protein [Oryza sativa (japonica cultivar-group)] e_value:0</td><td>11669.m00274|LOC_Os03g03430|protein At1g47330/T3F24_2 e_value:9.01283e-137</td><td>0.147</td><td>(0.3638 2.4795)</td><td>0.3638</td><td>2.4795</td></tr>
<tr><td>2959</td><td>WT006_J10_CLUSTER_4389</td><td>gi|22122905|gb|AAM92288.1| putative cytochrome oxidase assembly protein [Oryza sativa (japonica cultivar-group)] e_value:2.9082e-169</td><td>11676.m03407|LOC_Os10g37690|protein putative cytochrome oxidase assembly protein e_value:7.30488e-171</td><td>0.325</td><td>(0.1483 0.4562)</td><td>0.1483</td><td>0.4562</td></tr>
<tr><td>2960</td><td>WT013_C05_CLUSTER_32467</td><td>gi|34894710|ref|NP_908680.1| Putative Pto kinase interactor 1 [Oryza sativa (japonica cultivar-group)] >gnl|BL_ORD_ID|1560782 gi|29027802|dbj|BAC65877.1| putative Pto kinase interactor 1 [Oryza sativa (japonica cultivar-group)] >gnl|BL_ORD_ID|156078</td><td>11667.m02127|LOC_Os01g21970|protein Protein kinase domain e_value:0</td><td>0.128</td><td>(0.0881 0.6874)</td><td>0.0881</td><td>0.6874</td></tr>
<tr><td>2961</td><td>SET2_J18_CLUSTER_28425</td><td>gi|50355738|gb|AAT75263.1| putative ML domain protein [Oryza sativa (japonica cultivar-group)] e_value:1.17742e-56</td><td>11669.m05804|LOC_Os03g57420|protein putative ML domain protein e_value:2.09526e-58</td><td>0.329</td><td>(0.1791 0.5449)</td><td>0.1791</td><td>0.5449</td></tr>
<tr><td>2962</td><td>WT009_H14_CLUSTER_28842</td><td>gi|38346052|emb|CAD41393.2| OJ000223_09.6 [Oryza sativa (japonica cultivar-group)] >gnl|BL_ORD_ID|1523156 gi|38345343|emb|CAE03154.2| OSJNBa0081L15.16 [Oryza sativa (japonica cultivar-group)] >gnl|BL_ORD_ID|1523156 gi|50925285|ref|XP_472940.1| OSJNB</td><td>11670.m03913|LOC_Os04g40860|protein transposon protein, putative, unclassified e_value:0</td><td>0.087</td><td>(0.0525 0.6064)</td><td>0.0525</td><td>0.6064</td></tr>
<tr><td>2963</td><td>WT004_P08_CLUSTER_6404</td><td>gi|31432776|gb|AAP54369.1| putative c-myc binding protein [Oryza sativa (japonica cultivar-group)] >gnl|BL_ORD_ID|2012255 gi|37535560|ref|NP_922082.1| putative c-myc binding protein [Oryza sativa (japonica cultivar-group)] >gnl|BL_ORD_ID|2012255 gi|</td><td>11676.m03077|LOC_Os10g34670|protein putative c-myc binding protein e_value:8.44217e-70</td><td>0.156</td><td>(0.0594 0.3811)</td><td>0.0594</td><td>0.3811</td></tr>
<tr><td>2964</td><td>WT004_O05_CLUSTER_32217</td><td>gi|53791771|dbj|BAD53565.1| putative spastin protein [Oryza sativa (japonica cultivar-group)] e_value:0</td><td>11680.m04981|LOC_Os06g50050|protein ATPase, AAA family, putative e_value:0</td><td>0.151</td><td>(0.0555 0.3671)</td><td>0.0555</td><td>0.3671</td></tr>
<tr><td>2965</td><td>WT011_J14_CLUSTER_32216</td><td>gi|55733904|gb|AAV59411.1| putative glycosyltransferase [Oryza sativa (japonica cultivar-group)] >gnl|BL_ORD_ID|1741323 gi|50932541|ref|XP_475798.1| putative glycosyltransferase [Oryza sativa (japonica cultivar-group)] e_value:0</td><td>11682.m04444|LOC_Os05g46260|protein expressed protein e_value:0</td><td>0.086</td><td>(0.0725 0.8427)</td><td>0.0725</td><td>0.8427</td></tr>
<tr><td>2966</td><td>WT004_F10_CLUSTER_34442</td><td>gi|19912727|dbj|BAB88646.1| alternative oxidase [Triticum aestivum] e_value:0</td><td>11668.m04572|LOC_Os02g47200|protein transposon protein, putative, unclassified e_value:1.50034e-155</td><td>0.347</td><td>(0.1117 0.3216)</td><td>0.1117</td><td>0.3216</td></tr>
<tr><td>2967</td><td>SET3_J23_CLUSTER_12348</td><td>gi|31430873|gb|AAP52725.1| unknown protein [Oryza sativa (japonica cultivar-group)] >gi|37532272|ref|NP_920438.1| unknown protein [Oryza sativa (japonica cultivar-group)] >gi|20279484|gb|AAM18764.1| unknown protein [Oryza sativa (japonica cultivar-g</td><td>11676.m01223|LOC_Os10g13800|protein expressed protein e_value:5.1224e-106</td><td>0.037</td><td>(0.0122 0.3327)</td><td>0.0122</td><td>0.3327</td></tr>
<tr><td>2968</td><td>SET1_G22_CLUSTER_1107</td><td>gi|31431902|gb|AAP53614.1| putative Transcription initiation factor IIE, beta subunit [Oryza sativa (japonica cultivar-group)] >gi|37534050|ref|NP_921327.1| putative Transcription initiation factor IIE, beta subunit [Oryza sativa (japonica cultivar-gro</td><td>11676.m02194|LOC_Os10g25770|protein expressed protein e_value:2.20758e-119</td><td>0.146</td><td>(0.1394 0.9562)</td><td>0.1394</td><td>0.9562</td></tr>
<tr><td>2969</td><td>SET4_D18_CLUSTER_34143</td><td>gi|50912865|ref|XP_467840.1| unknown protein [Oryza sativa (japonica cultivar-group)] >gi|46390130|dbj|BAD15565.1| unknown protein [Oryza sativa (japonica cultivar-group)] e_value:2.31302e-96</td><td>11668.m05051|LOC_Os02g51700|protein expressed protein e_value:5.69151e-98</td><td>0.18</td><td>(0.1004 0.5566)</td><td>0.1004</td><td>0.5566</td></tr>
<tr><td>2970</td><td>SET5_O08_CLUSTER_10362</td><td>gi|34909866|ref|NP_916280.1| OSJNBb0053G03.14 [Oryza sativa (japonica cultivar-group)] >gi|53793556|dbj|BAD53326.1| unknown protein [Oryza sativa (japonica cultivar-group)] >gi|22535590|dbj|BAC10765.1| unknown protein [Oryza sativa (japonica cultiva</td><td>11667.m05552|LOC_Os01g56180|protein expressed protein e_value:0</td><td>0.111</td><td>(0.0576 0.5197)</td><td>0.0576</td><td>0.5197</td></tr>
<tr><td>2971</td><td>WT006_O22_CLUSTER_13145</td><td>gi|52353639|gb|AAU44205.1| unknown protein [Oryza sativa (japonica cultivar-group)] e_value:0</td><td>11682.m00122|LOC_Os05g02040|protein Transposable element protein, putative e_value:0</td><td>0.5</td><td>(0.3822 0.7641)</td><td>0.3822</td><td>0.7641</td></tr>
<tr><td>2972</td><td>SET4_G14_CLUSTER_33228</td><td>gi|55168341|gb|AAV44206.1| unknow protein [Oryza sativa (japonica cultivar-group)] e_value:2.27506e-135</td><td>11682.m00186|LOC_Os05g02640|protein hydrolase, NUDIX family, putative e_value:5.62318e-137</td><td>0.206</td><td>(0.1335 0.6476)</td><td>0.1335</td><td>0.6476</td></tr>
<tr><td>2973</td><td>WT010_H11_CLUSTER_34123</td><td>gi|6692816|dbj|BAA89423.1| allyl alcohol dehydrogenase [Nicotiana tabacum] e_value:4.23692e-127</td><td>11686.m01228|LOC_Os12g12590|protein oxidoreductase, zinc-binding dehydrogenase family e_value:9.12337e-145</td><td>0.208</td><td>(0.1768 0.8500)</td><td>0.1768</td><td>0.85</td></tr>
<tr><td>2974</td><td>WT004_O23_CLUSTER_34123</td><td>gi|38345384|emb|CAD41251.2| OSJNBa0067K08.13 [Oryza sativa (japonica cultivar-group)] >gnl|BL_ORD_ID|1522174 gi|50925977|ref|XP_473036.1| OSJNBa0067K08.13 [Oryza sativa (japonica cultivar-group)] e_value:5.09974e-130</td><td>11686.m01228|LOC_Os12g12590|protein oxidoreductase, zinc-binding dehydrogenase family e_value:9.5857e-147</td><td>0.186</td><td>(0.1725 0.9274)</td><td>0.1725</td><td>0.9274</td></tr>
<tr><td>2975</td><td>WT008_H13_CLUSTER_34662</td><td>gi|50918673|ref|XP_469733.1| putative embryonic cell protein [Oryza sativa (japonica cultivar-group)] >gnl|BL_ORD_ID|1539242 gi|14626282|gb|AAK71550.1| putative embryonic cell protein [Oryza sativa (japonica cultivar-group)] e_value:7.79349e-83</td><td>11669.m00589|LOC_Os03g06360|protein Seed maturation protein, putative e_value:2.10137e-60</td><td>0.722</td><td>(0.3855 0.5340)</td><td>0.3855</td><td>0.534</td></tr>
<tr><td>2976</td><td>WT010_E06_CLUSTER_5717</td><td>gi|21425571|emb|CAD32696.1| triacylglycerol lipase [Triticum aestivum] e_value:0</td><td>11667.m07119|LOC_Os01g71010|protein triacylglycerol lipase e_value:1.00481e-175</td><td>0.111</td><td>(0.0750 0.6749)</td><td>0.075</td><td>0.6749</td></tr>
<tr><td>2977</td><td>WT004_L21_CLUSTER_7666</td><td>gi|21877|emb|CAA79268.1| DNA binding protein [Triticum aestivum] >gnl|BL_ORD_ID|2318695 gi|422014|pir||S30216 transcription initiation factor IID - wheat >gnl|BL_ORD_ID|2318695 gi|417882|sp|Q02879|TF22_WHEAT Transcription initiation factor TFIID-2 (</td><td>11669.m04529|LOC_Os03g45410|protein TATA-binding protein TBP2 e_value:1.59727e-106</td><td>0.022</td><td>(0.0200 0.9082)</td><td>0.02</td><td>0.9082</td></tr>
<tr><td>2978</td><td>WT003_C13_CLUSTER_22886</td><td>gi|50919077|ref|XP_469935.1| putative aldehyde oxidase [Oryza sativa (japonica cultivar-group)] >gnl|BL_ORD_ID|1587261 gi|27819516|gb|AAO24920.1| putative aldehyde oxidase [Oryza sativa (japonica cultivar-group)] e_value:0</td><td>11669.m05835|LOC_Os03g57690|protein putative aldehyde oxidase e_value:0</td><td>0.204</td><td>(0.1250 0.6127)</td><td>0.125</td><td>0.6127</td></tr>
<tr><td>2979</td><td>WT009_C13_CLUSTER_19865</td><td>gi|55296827|dbj|BAD68171.1| putative UDP-glucose:salicylic acid glucosyltransferase [Oryza sativa (japonica cultivar-group)] e_value:0</td><td>11667.m05866|LOC_Os01g59110|protein UDP-glucoronosyl and UDP-glucosyl transferase e_value:0</td><td>0.309</td><td>(0.1438 0.4651)</td><td>0.1438</td><td>0.4651</td></tr>
<tr><td>2980</td><td>SET3_E12_CLUSTER_33113</td><td>gi|31433347|gb|AAP54876.1| putative protein phosphatase 2C [Oryza sativa (japonica cultivar-group)] >gi|37536574|ref|NP_922589.1| putative protein phosphatase 2C [Oryza sativa (japonica cultivar-group)] e_value:0</td><td>11669.m00380|LOC_Os03g04430|protein Protein phosphatase 2C, putative e_value:0</td><td>0.104</td><td>(0.0694 0.6694)</td><td>0.0694</td><td>0.6694</td></tr>
<tr><td>2981</td><td>WT003_K13_CLUSTER_28761</td><td>gi|50915314|ref|XP_468121.1| unknown protein [Oryza sativa (japonica cultivar-group)] >gnl|BL_ORD_ID|1583240 gi|47497478|dbj|BAD19532.1| unknown protein [Oryza sativa (japonica cultivar-group)] e_value:4.86916e-61</td><td>11668.m05323|LOC_Os02g54130|protein DnaJ domain, putative e_value:1.81124e-112</td><td>0.26</td><td>(0.1391 0.5347)</td><td>0.1391</td><td>0.5347</td></tr>
<tr><td>2982</td><td>WT006_B14_CLUSTER_33541</td><td>gi|50919127|ref|XP_469960.1| putative serine/threonine protein kinase [Oryza sativa (japonica cultivar-group)] >gnl|BL_ORD_ID|1587287 gi|28269422|gb|AAO37965.1| putative serine/threonine protein kinase [Oryza sativa (japonica cultivar-group)] e_value:0</td><td>11669.m05864|LOC_Os03g57940|protein putative serine/threonine protein kinase e_value:0</td><td>0.129</td><td>(0.0672 0.5223)</td><td>0.0672</td><td>0.5223</td></tr>
<tr><td>2983</td><td>WT008_M11_CLUSTER_16812</td><td>gi|50540691|gb|AAT77848.1| putative lipase [Oryza sativa (japonica cultivar-group)] e_value:6.63833e-176</td><td>11669.m05144|LOC_Os03g51010|protein putative lipase e_value:1.59828e-177</td><td>0.161</td><td>(0.1240 0.7685)</td><td>0.124</td><td>0.7685</td></tr>
<tr><td>2984</td><td>SET5_L07_CLUSTER_3292</td><td>gi|34910678|ref|NP_916686.1| putative NADH dehydrogenase (ubiquinone) chain PSST precursor [Oryza sativa (japonica cultivar-group)] >gi|18461218|dbj|BAB84415.1| putative NADH2 dehydrogenase (ubiquinone) chain PSST precursor [Oryza sativa (japonica cult</td><td>11682.m04384|LOC_Os05g45730|protein NADH dehydrogenase subunit 10 e_value:8.00565e-106</td><td>0.107</td><td>(0.0504 0.4704)</td><td>0.0504</td><td>0.4704</td></tr>
</table>

PDF Complete
Your complimentary use period has ended. Thank you for using PDF Complete.
Click Here to upgrade to Unlimited Pages and Expanded Features

2985	WT012_K01_CLUSTER_2663	gi|57222454|gb|AAP12933.2| putative cell division control protein [Oryza sativa (japonica cultivar-group)] e_value:0	11669.m02853|LOC_Os03g27950|protein putative cell division control protein e_value:0	0.17	(0.0945 0.5564)	0.0945	0.5564
2986	WT011_N15_CLUSTER_18334	gi|21740710|emb|CAD40831.1| OSJNBa0086B14.3 [Oryza sativa (japonica cultivar-group)] >gnl|BL_ORD_ID|1524033 gi|50924606|ref|XP_472661.1| OSJNBa0086B14.3 [Oryza sativa (japonica cultivar-group)] e_value:0	11670.m03564|LOC_Os04g37720|protein expressed protein e_value:0	0.189	(0.1205 0.6362)	0.1205	0.6362
2987	SET3_I12_CLUSTER_18582	gi|34896098|ref|NP_909393.1| putative RNA helicase [Oryza sativa (japonica cultivar-group)] >gi|15528747|dbj|BAB64789.1| putative RNA helicase [Oryza sativa (japonica cultivar-group)] >gi|21327991|dbj|BAC00580.1| putative RNA helicase [Oryza sativa	11667.m00642|LOC_Os01g07080|protein DEAD/DEAH box helicase, putative e_value:0	0.2	(0.1041 0.5209)	0.1041	0.5209
2988	WT013_O22_CLUSTER_21639	gi|50919791|ref|XP_470256.1| Hypothetical protein [Oryza sativa (japonica cultivar-group)] >gnl|BL_ORD_ID|1539280 gi|22773230|gb|AAN06836.1| Hypothetical protein [Oryza sativa (japonica cultivar-group)] e_value:2.37534e-151	11669.m00572|LOC_Os03g06230|protein expressed protein e_value:9.00299e-159	0.288	(0.1062 0.3682)	0.1062	0.3682
2989	WT013_O01_CLUSTER_28331	gi|38346620|emb|CAE02139.2| OSJNBa0074L08.11 [Oryza sativa (japonica cultivar-group)] >gnl|BL_ORD_ID|1535071 gi|50926632|ref|XP_473263.1| OSJNBa0074L08.11 [Oryza sativa (japonica cultivar-group)] e_value:0	11668.m04076|LOC_Os02g42590|protein hypothetical protein e_value:0	0.203	(0.2144 1.0544)	0.2144	1.0544
2990	WT012_M09_CLUSTER_20558	gi|50940093|ref|XP_479574.1| putative thioredoxin [Oryza sativa (japonica cultivar-group)] >gnl|BL_ORD_ID|1534655 gi|51979074|ref|XP_507414.1| PREDICTED OSJNBa0060O17.14 gene product [Oryza sativa (japonica cultivar-group)] >gnl|BL_ORD_ID|1534655 gi	11673.m04815|LOC_Os07g48510|protein Thioredoxin, putative e_value:1.0019e-113	0.284	(0.1403 0.4946)	0.1403	0.4946
2991	WT011_J24_CLUSTER_27441	gi|3059131|emb|CAA04117.1| cytochrome P450 [Helianthus tuberosus] e_value:8.01124e-120	11686.m03884|LOC_Os12g39300|protein cytochrome P450, putative e_value:0	0.409	(0.2012 0.4915)	0.2012	0.4915
2992	WT011_G14_CLUSTER_12418	gi|21741306|emb|CAD41269.1| OSJNBb0103I08.8 [Oryza sativa (japonica cultivar-group)] >gnl|BL_ORD_ID|1522742 gi|50927168|ref|XP_473367.1| OSJNBb0103I08.8 [Oryza sativa (japonica cultivar-group)] e_value:3.05437e-66	11670.m04456|LOC_Os04g45830|protein expressed protein e_value:5.02317e-68	0.518	(0.1615 0.3116)	0.1615	0.3116
2993	WT011__C10_CLUSTER_6230	gi|50919667|ref|XP_470194.1| Putative F-box protein [Oryza sativa (japonica cultivar-group)] >gnl|BL_ORD_ID|1539624 gi|22758266|gb|AAN05494.1| Putative F-box protein [Oryza sativa (japonica cultivar-group)] e_value:0	11669.m00364|LOC_Os03g04270|protein F-box domain, putative e_value:0	0.276	(0.1556 0.5648)	0.1556	0.5648
2994	WT009_O18_CLUSTER_24922	gi|57899962|dbj|BAD87898.1| putative LRK1 protein [Oryza sativa (japonica cultivar-group)] e_value:0	11682.m04278|LOC_Os05g44770|protein Leucine Rich Repeat, putative e_value:0	0.417	(0.1782 0.4276)	0.1782	0.4276
2995	WT009_B24_CLUSTER_12453	gi|51090726|dbj|BAD36734.1| putative beta-glucuronidase precursor [Oryza sativa (japonica cultivar-group)] >gnl|BL_ORD_ID|238022 gi|51090574|dbj|BAD36026.1| putative beta-glucuronidase precursor [Oryza sativa (japonica cultivar-group)] e_value:0	11668.m05509|LOC_Os02g55870|protein Glycosyl hydrolase family 79, N-terminal domain, putative e_value:0	0.215	(0.1452 0.6745)	0.1452	0.6745
2996	WT008_K17_CLUSTER_4924	gi|50938125|ref|XP_478590.1| putative serine/threonine kinase protein [Oryza sativa (japonica cultivar-group)] >gnl|BL_ORD_ID|1528464 gi|50508315|dbj|BAD30123.1| putative serine/threonine kinase protein [Oryza sativa (japonica cultivar-group)] >gnl|	11673.m03440|LOC_Os07g35680|protein Protein kinase domain, putative e_value:0	0.257	(0.1491 0.5808)	0.1491	0.5808
2997	WT008_D15_CLUSTER_2716	gi|7406456|emb|CAB85558.1| 3-oxoacyl-[acyl-carrier-protein] synthase-like protein [Arabidopsis thaliana] >gnl|BL_ORD_ID|1466892 gi|15238192|ref|NP_196072.1| expressed protein [Arabidopsis thaliana] >gnl|BL_ORD_ID|1466892 gi|11280850|pir||T48448 3-ox	11669.m02514|LOC_Os03g24830|protein 3-oxoacyl-[acyl-carrier-protein] synthase-like protein - Arabidopsis thaliana e_value:3.00232e-165	0.286	(0.1314 0.4593)	0.1314	0.4593
2998	WT008_B22_CLUSTER_7580	gi|50905341|ref|XP_464159.1| putative pathogenesis related homeodomain protein PRHA [Oryza sativa (japonica cultivar-group)] >gnl|BL_ORD_ID|1566106 gi|51979134|ref|XP_507438.1| PREDICTED P0479D12.6 gene product [Oryza sativa (japonica cultivar-group)]	11668.m00483|LOC_Os02g05450|protein PHD-finger, putative e_value:0	0.341	(0.2230 0.6545)	0.223	0.6545
2999	WT008__C24_CLUSTER_5019	gi|50902310|ref|XP_463588.1| P0497A05.17 [Oryza sativa (japonica cultivar-group)] >gnl|BL_ORD_ID|1559697 gi|20804927|dbj|BAB92606.1| putative MRS2-7 [Oryza sativa (japonica cultivar-group)] >gnl|BL_ORD_ID|1559697	11667.m06856|LOC_Os01g68040|protein CorA-like Mg2+ transporter protein e_value:0	0.161	(0.0808 0.5025)	0.0808	0.5025
3000	WT006_J09_CLUSTER_29594	gi|51963446|ref|XP_506367.1| PREDICTED P0048D08.116 gene product [Oryza sativa (japonica cultivar-group)] e_value:0	11673.m03179|LOC_Os07g33200|protein hypothetical protein e_value:2.07933e-157	0.727	(0.2436 0.3349)	0.2436	0.3349
3001	WT005_G14_CLUSTER_20095	gi|50912867|ref|XP_467841.1| putative elicitor-inducible protein EIG-J7 [Oryza sativa (japonica cultivar-group)] >gnl|BL_ORD_ID|1582958 gi|51964382|ref|XP_506976.1| PREDICTED OJ1288_G09.19 gene product [Oryza sativa (japonica cultivar-group)] >gnl|B	11668.m05052|LOC_Os02g51710|protein PLAT/LH2 domain, putative e_value:6.5251e-77	0.447	(0.1522 0.3403)	0.1522	0.3403
3002	WT004_N14_CLUSTER_6790	gi|31432643|gb|AAP54251.1| putative serine protease [Oryza sativa (japonica cultivar-group)] >gnl|BL_ORD_ID|2012137 gi|37535324|ref|NP_921964.1| putative serine protease [Oryza sativa (japonica cultivar-group)] >gnl|BL_ORD_ID|2012137 gi|16905164|gb|	11676.m02949|LOC_Os10g33490|protein putative serine protease e_value:2.70261e-143	0.837	(0.4084 0.4878)	0.4084	0.4878
3003	WT004_L05_CLUSTER_3262	gi|50907997|ref|XP_465487.1| unknown protein [Oryza sativa (japonica cultivar-group)] >gnl|BL_ORD_ID|1570639 gi|47497565|dbj|BAD19636.1| unknown protein [Oryza sativa (japonica cultivar-group)] e_value:2.41912e-117	11668.m02460|LOC_Os02g26300|protein expressed protein e_value:7.83073e-119	0.657	(0.4123 0.6279)	0.4123	0.6279
3004	WT004_H10_CLUSTER_21639	gi|50919791|ref|XP_470256.1| Hypothetical protein [Oryza sativa (japonica cultivar-group)] >gnl|BL_ORD_ID|1539280 gi|22773230|gb|AAN06836.1| Hypothetical protein [Oryza sativa (japonica cultivar-group)] e_value:2.26438e-151	11669.m00572|LOC_Os03g06230|protein expressed protein e_value:9.00299e-159	0.288	(0.1062 0.3682)	0.1062	0.3682
3005	WT002_G03_CLUSTER_8795	gi|28273408|gb|AAO38494.1| unknown protein [Oryza sativa (japonica cultivar-group)] e_value:3.58219e-64	11669.m04876|LOC_Os03g48620|protein hypothetical protein e_value:5.74899e-66	0.603	(0.1634 0.2710)	0.1634	0.271
3006	WT002_F23_CLUSTER_18880	gi|50932807|ref|XP_475931.1| unknown protein [Oryza sativa (japonica cultivar-group)] >gnl|BL_ORD_ID|1599514 gi|47900279|gb|AAT39147.1| unknown protein [Oryza sativa (japonica cultivar-group)] e_value:3.66741e-58	11682.m04821|LOC_Os05g50110|protein Oleosin, putative e_value:7.70565e-60	0.339	(0.1929 0.5698)	0.1929	0.5698
3007	SET6_K08_CLUSTER_15805	gi|50948461|ref|XP_483758.1| hypothetical protein [Oryza sativa (japonica cultivar-group)] >gi|45736097|dbj|BAD13128.1| hypothetical protein [Oryza sativa (japonica cultivar-group)] e_value:3.23557e-82	11674.m04487|LOC_Os08g44410|protein Harpin-induced protein 1 (Hin1), putative e_value:8.61302e-84	0.472	(0.1181 0.2504)	0.1181	0.2504
3008	SET6__C01_CLUSTER_8795	gi|28273408|gb|AAO38494.1| unknown protein [Oryza sativa (japonica cultivar-group)] e_value:4.40063e-65	11669.m04876|LOC_Os03g48620|protein hypothetical protein e_value:6.60244e-67	0.589	(0.1339 0.2272)	0.1339	0.2272
3009	SET5_K14_CLUSTER_25792	gi|51535804|dbj|BAD37889.1| ankyrin repeat protein chloroplast precursor-like [Oryza sativa (japonica cultivar-group)] e_value:3.03212e-113	11680.m01276|LOC_Os06g13000|protein ankyrin repeat protein EMB506, putative e_value:1.43155e-114	0.242	(0.1522 0.6298)	0.1522	0.6298
3010	SET4_K10_CLUSTER_22149	gi|50428690|gb|AAT77041.1| putative Cyclin [Oryza sativa (japonica cultivar-group)] e_value:1.98509e-96	11686.m03945|LOC_Os12g39830|protein Cyclin, N-terminal domain, putative e_value:1.4705e-142	0.387	(0.1274 0.3293)	0.1274	0.3293
3011	SET1_A13_CLUSTER_10121	gi|53792568|dbj|BAD53557.1| putative amino acid carrier [Oryza sativa (japonica cultivar-group)] e_value:0	11680.m03506|LOC_Os06g36210|protein probable amino acid carrier [imported] - Arabidopsis thaliana e_value:0	0.401	(0.1034 0.2580)	0.1034	0.258
3012	SET1_D20_CLUSTER_31466	gi|4741842|gb|AAD28730.1| chitinase II precursor [Triticum aestivum] e_value:8.19144e-134	11670.m03999|LOC_Os04g41620|protein probable chitinase (EC 3.2.1.14) IIb - rice e_value:1.77572e-78	0.425	(0.2557 0.6016)	0.2557	0.6016
3013	WT010_A15_CLUSTER_6414	gi|56201907|dbj|BAD73357.1| polymerase (DNA directed) sigma-like [Oryza sativa (japonica cultivar-group)] >gnl|BL_ORD_ID|542312 gi|56201854|dbj|BAD73304.1| polymerase (DNA directed) sigma-like [Oryza sativa (japonica cultivar-group)] e_value:0	11667.m04674|LOC_Os01g48210|protein PAP/25A associated domain, putative e_value:0	0.192	(0.1243 0.6486)	0.1243	0.6486
3014	WT009_M01_CLUSTER_14642	gi|34894174|ref|NP_908412.1| putative LRR receptor-like protein kinase [Oryza sativa (japonica cultivar-group)] >gnl|BL_ORD_ID|1751542 gi|13486635|dbj|BAB39873.1| putative LRR receptor-like protein kinase [Oryza sativa (japonica cultivar-group)] e_valu	11667.m00109|LOC_Os01g02040|protein transposon protein, putative, CACTA, En/Spm sub-class e_value:0	0.182	(0.1524 0.8393)	0.1524	0.8393
3015	WT006_I15_CLUSTER_33662	gi|13517761|gb|AAK28975.1| SET1 [Oryza sativa] e_value:0	11687.m03606|LOC_Os11g38900|protein SET1 e_value:0	0.196	(0.0992 0.5066)	0.0992	0.5066
3016	WT006_A17_CLUSTER_34447	gi|38345037|emb|CAE01636.2| OSJNBa0029H02.21 [Oryza sativa (japonica cultivar-group)] >gnl|BL_ORD_ID|1521520 gi|50926181|ref|XP_473065.1| OSJNBa0029H02.21 [Oryza sativa (japonica cultivar-group)] e_value:0	11670.m04074|LOC_Os04g42330|protein Spc97 / Spc98 family, putative e_value:0	0.08	(0.0481 0.6035)	0.0481	0.6035
3017	SET1_I14_CLUSTER_7283	gi|50947725|ref|XP_483390.1| DnaJ protein family-like [Oryza sativa (japonica cultivar-group)] >gi|42407725|dbj|BAD08872.1| DnaJ protein family-like [Oryza sativa (japonica cultivar-group)] >gi|42407330|dbj|BAD08769.1| DnaJ protein family-like [Oryz	11674.m04133|LOC_Os08g41110|protein DnaJ domain, putative e_value:0	0.155	(0.0788 0.5088)	0.0788	0.5088
3018	WT012_K24_CLUSTER_3811	gi|51090413|dbj|BAD35335.1| loricrin-like [Oryza sativa (japonica cultivar-group)] e_value:3.8975e-94	11680.m02654|LOC_Os06g27830|protein expressed protein e_value:4.78296e-94	0.364	(0.4321 1.1869)	0.4321	1.1869
3019	SET3_D18_CLUSTER_3819	gi|451195|gb|AAC37417.1| wali6 >gi|7489670|pir||T06986 hypothetical protein wali6 - wheat >gi|1090844|prf||2019486A wali6 gene e_value:3.51397e-28	11667.m00319|LOC_Os01g04040|protein Bowman-Birk serine protease inhibitor family, putative e_value:4.37913e-16	0.671	(0.4325 0.6449)	0.4325	0.6449
3020	WT002_A24_CLUSTER_32249	gi|50252106|dbj|BAD28092.1| unknown protein [Oryza sativa (japonica cultivar-group)] e_value:4.86093e-172	11668.m00075|LOC_Os02g01720|protein expressed protein e_value:4.31084e-153	0.209	(0.0831 0.3982)	0.0831	0.3982
3021	SET6_K21_CLUSTER_20515	gi|50924734|ref|XP_472720.1| OSJNBa0006M15.16 [Oryza sativa (japonica cultivar-group)] >gi|38347101|emb|CAE02573.2| OSJNBa0006M15.16 [Oryza sativa (japonica cultivar-group)] e_value:0	11670.m03631|LOC_Os04g38330|protein lipoic acid synthetase e_value:0	0.117	(0.0619 0.5288)	0.0619	0.5288
3022	SET4_P18_CLUSTER_7813	gi|34894200|ref|NP_908425.1| putative SP2G [Oryza sativa (japonica cultivar-group)] >gi|13486648|dbj|BAB39886.1| putative SP2G [Oryza sativa (japonica cultivar-group)] e_value:6.80449e-71	11667.m00122|LOC_Os01g02120|protein Phosphatidylethanolamine-binding protein e_value:1.15682e-72	0.48	(0.1665 0.3473)	0.1665	0.3473
3023	WT008_H18_CLUSTER_11176	gi|50919781|ref|XP_470251.1| Hypothetical protein [Oryza sativa (japonica cultivar-group)] >gnl|BL_ORD_ID|1539295 gi|22773225|gb|AAN06831.1| Hypothetical protein [Oryza sativa (japonica cultivar-group)] >gnl|BL_ORD_ID|1539295 gi|21397265|gb|AAM51829	11669.m00567|LOC_Os03g06180|protein hypothetical protein e_value:0	0.175	(0.1244 0.7100)	0.1244	0.71
3024	WT005_G10_CLUSTER_33921	gi|34915410|ref|NP_919162.1| hypothetical protein [Oryza sativa (japonica cultivar-group)] >gnl|BL_ORD_ID|1518852 gi|22535638|dbj|BAC10812.1| hypothetical protein [Oryza sativa (japonica cultivar-group)] e_value:9.63993e-47	11673.m00193|LOC_Os07g02850|protein expressed protein e_value:1.49945e-48	0.191	(0.2056 1.0741)	0.2056	1.0741
3025	WT010_J05_CLUSTER_25438	gi|54291562|dbj|BAD62486.1| unknown protein [Oryza sativa (japonica cultivar-group)] >gnl|BL_ORD_ID|266137 gi|54291361|dbj|BAD62127.1| unknown protein [Oryza sativa (japonica cultivar-group)] e_value:9.00871e-71	11680.m05137|LOC_Os06g51420|protein At3g05070 e_value:1.7756e-72	0.23	(0.1629 0.7090)	0.1629	0.709
3026	WT013_H16_CLUSTER_31623	gi|50908085|ref|XP_465531.1| putative OsCTTP [Oryza sativa (japonica cultivar-group)] >gnl|BL_ORD_ID|1571653 gi|51979234|ref|XP_507481.1| PREDICTED OJ1342_D02.8 gene product [Oryza sativa (japonica cultivar-group)] >gnl|BL_ORD_ID|1571653 gi|51979232	11668.m02503|LOC_Os02g26700|protein ChaC-like protein e_value:2.84661e-107	0.097	(0.0630 0.6519)	0.063	0.6519
3027	WT005_M08_CLUSTER_32476	gi|50929555|ref|XP_474305.1| OSJNBb0004A17.3 [Oryza sativa (japonica cultivar-group)] >gnl|BL_ORD_ID|1594456 gi|32488724|emb|CAE03601.1| OSJNBb0004A17.3 [Oryza sativa (japonica cultivar-group)] e_value:0	11670.m05693|LOC_Os04g57590|protein intracellular protease, PfpI family, putative e_value:0	0.07	(0.0598 0.8588)	0.0598	0.8588

PDF Complete
Your complimentary use period has ended. Thank you for using PDF Complete.
Click Here to upgrade to Unlimited Pages and Expanded Features

<table>
<tr><td>3028</td><td>SET4_K15_CLUSTER_34773</td><td>gi|34897474|ref|NP_910083.1| unknown protein [Oryza sativa (japonica cultivar-group)] >gi|28269417|gb|AAO37960.1| unknown protein [Oryza sativa (japonica cultivar-group)] e_value:6.94464e-100</td><td>11669.m06469|LOC_Os03g63110|protein expressed protein e_value:7.95356e-102</td><td>0.111</td><td>(0.0689 0.6216)</td><td>0.0689</td><td>0.6216</td></tr>
<tr><td>3029</td><td>WT008_P12_CLUSTER_3807</td><td>gi|32488518|emb|CAE03270.1| OSJNBa0011J08.25 [Oryza sativa (japonica cultivar-group)] >gnl|BL_ORD_ID|1524082 gi|38345861|emb|CAD41158.2| OSJNBa0064M23.3 [Oryza sativa (japonica cultivar-group)] >gnl|BL_ORD_ID|1524082 gi|50928203|ref|XP_473629.1| OSJ</td><td>11670.m04823|LOC_Os04g49060|protein hypothetical protein e_value:0</td><td>0.102</td><td>(0.0481 0.4709)</td><td>0.0481</td><td>0.4709</td></tr>
<tr><td>3030</td><td>WT008_O11_CLUSTER_23080</td><td>gi|50726035|dbj|BAD33560.1| putative alanine aminotransferase [Oryza sativa (japonica cultivar-group)] e_value:0</td><td>11681.m02405|LOC_Os09g26380|protein alanine aminotransferase e_value:0</td><td>0.179</td><td>(0.1301 0.7254)</td><td>0.1301</td><td>0.7254</td></tr>
<tr><td>3031</td><td>WT009_M07_CLUSTER_11814</td><td>gi|31432073|gb|AAP53758.1| unknown protein [Oryza sativa (japonica cultivar-group)] >gnl|BL_ORD_ID|2011203 gi|37534338|ref|NP_921471.1| unknown protein [Oryza sativa (japonica cultivar-group)] e_value:0</td><td>11676.m02373|LOC_Os10g27990|protein Exocyst complex subunit Sec15, putative e_value:0</td><td>0.189</td><td>(0.1255 0.6637)</td><td>0.1255</td><td>0.6637</td></tr>
<tr><td>3032</td><td>WT008_M16_CLUSTER_785</td><td>gi|34895730|ref|NP_909214.1| unknown protein [Oryza sativa (japonica cultivar-group)] >gnl|BL_ORD_ID|1751627 gi|13486890|dbj|BAB40119.1| unknown protein [Oryza sativa (japonica cultivar-group)] >gnl|BL_ORD_ID|1751627 gi|10798833|dbj|BAB16464.1| unkn</td><td>11667.m00428|LOC_Os01g05010|protein Mitochondrial glycoprotein, putative e_value:6.66201e-104</td><td>0.227</td><td>(0.1474 0.6482)</td><td>0.1474</td><td>0.6482</td></tr>
<tr><td>3033</td><td>SET3_L21_CLUSTER_6499</td><td>gi|34900032|ref|NP_911362.1| putative NAD synthetase [Oryza sativa (japonica cultivar-group)] >gi|22202733|dbj|BAC07390.1| putative NAD synthetase [Oryza sativa (japonica cultivar-group)] e_value:0</td><td>11673.m00651|LOC_Os07g07260|protein hydrolase, carbon-nitrogen family, putative e_value:0</td><td>0.107</td><td>(0.0553 0.5185)</td><td>0.0553</td><td>0.5185</td></tr>
<tr><td>3034</td><td>WT004_D07_CLUSTER_15986</td><td>gi|50919393|ref|XP_470093.1| putative transcription elongation factor [Oryza sativa (japonica cultivar-group)] >gnl|BL_ORD_ID|1588162 gi|28876018|gb|AAO60027.1| putative transcription elongation factor [Oryza sativa (japonica cultivar-group)] e_value:3</td><td>11669.m06111|LOC_Os03g60130|protein putative transcription elongation factor e_value:8.47237e-155</td><td>0.132</td><td>(0.1272 0.9667)</td><td>0.1272</td><td>0.9667</td></tr>
<tr><td>3035</td><td>WT002_K01_CLUSTER_9883</td><td>gi|54287488|gb|AAV31232.1| putative anthranilate phosphoribosyltransferase [Oryza sativa (japonica cultivar-group)] e_value:0</td><td>11682.m02850|LOC_Os05g30750|protein anthranilate phosphoribosyltransferase-like protein – Arabidopsis thaliana e_value:0</td><td>0.032</td><td>(0.0189 0.5840)</td><td>0.0189</td><td>0.584</td></tr>
<tr><td>3036</td><td>SET2_G02_CLUSTER_20141</td><td>gi|34897178|ref|NP_909935.1| unknown protein [Oryza sativa (japonica cultivar-group)] >gi|28209523|gb|AAO37541.1| unknown protein [Oryza sativa (japonica cultivar-group)] e_value:1.82101e-89</td><td>11669.m05932|LOC_Os03g58490|protein expressed protein e_value:5.90063e-112</td><td>0.226</td><td>(0.4882 2.1628)</td><td>0.4882</td><td>2.1628</td></tr>
<tr><td>3037</td><td>WT009_L01_CLUSTER_32046</td><td>gi|55771322|dbj|BAD72247.1| putative serine/threonine kinase 38 [Oryza sativa (japonica cultivar-group)] e_value:0</td><td>11667.m00876|LOC_Os01g09200|protein Protein kinase domain, putative e_value:0</td><td>0.164</td><td>(0.1085 0.6608)</td><td>0.1085</td><td>0.6608</td></tr>
<tr><td>3038</td><td>WT008_M18_CLUSTER_32046</td><td>gi|55771322|dbj|BAD72247.1| putative serine/threonine kinase 38 [Oryza sativa (japonica cultivar-group)] e_value:0</td><td>11667.m00876|LOC_Os01g09200|protein Protein kinase domain, putative e_value:0</td><td>0.114</td><td>(0.0485 0.4241)</td><td>0.0485</td><td>0.4241</td></tr>
<tr><td>3039</td><td>WT010_A16_CLUSTER_20897</td><td>gi|50898796|ref|XP_450186.1| putative phospholipase D beta 1 [Oryza sativa (japonica cultivar-group)] >gnl|BL_ORD_ID|1828448 gi|32526679|dbj|BAC79202.1| putative phospholipase D beta 1 [Oryza sativa (japonica cultivar-group)] e_value:0</td><td>11681.m03306|LOC_Os09g37100|protein C2 domain, putative e_value:0</td><td>0.204</td><td>(0.0937 0.4589)</td><td>0.0937</td><td>0.4589</td></tr>
<tr><td>3040</td><td>WT003_H03_CLUSTER_15750</td><td>gi|62734630|gb|AAX96739.1| Transposable element protein, putative [Oryza sativa (japonica cultivar-group)] e_value:8.45689e-101</td><td>11687.m00874|LOC_Os11g09310|protein Transposable element protein, putative e_value:1.7097e-102</td><td>0.22</td><td>(0.1551 0.7036)</td><td>0.1551</td><td>0.7036</td></tr>
<tr><td>3041</td><td>WT009_D18_CLUSTER_1776</td><td>gi|50929763|ref|XP_474409.1| OSJNBa0088H09.5 [Oryza sativa (japonica cultivar-group)] >gnl|BL_ORD_ID|1594591 gi|32488704|emb|CAE03447.1| OSJNBa0088H09.5 [Oryza sativa (japonica cultivar-group)] e_value:3.00151e-121</td><td>11670.m05818|LOC_Os04g58730|protein expressed protein e_value:1.77879e-142</td><td>0.254</td><td>(0.1796 0.7076)</td><td>0.1796</td><td>0.7076</td></tr>
<tr><td>3042</td><td>WT011_D15_CLUSTER_7180</td><td>gi|31433036|gb|AAP54596.1| unknown protein [Oryza sativa (japonica cultivar-group)] >gnl|BL_ORD_ID|2012627 gi|37536014|ref|NP_922309.1| unknown protein [Oryza sativa (japonica cultivar-group)] >gnl|BL_ORD_ID|2012627 gi|10140674|gb|AAG13509.1| unknow</td><td>11676.m03328|LOC_Os10g36950|protein expressed protein e_value:1.32377e-173</td><td>0.307</td><td>(0.1796 0.5849)</td><td>0.1796</td><td>0.5849</td></tr>
<tr><td>3043</td><td>WT008_A14_CLUSTER_3685</td><td>gi|31433064|gb|AAP54624.1| putative transcription factor [Oryza sativa (japonica cultivar-group)] >gnl|BL_ORD_ID|2012655 gi|37536070|ref|NP_922337.1| putative transcription factor [Oryza sativa (japonica cultivar-group)] >gnl|BL_ORD_ID|2012655 gi|13</td><td>11669.m00146|LOC_Os03g02240|protein hypothetical protein e_value:0</td><td>0.636</td><td>(0.4046 0.6363)</td><td>0.4046</td><td>0.6363</td></tr>
<tr><td>3044</td><td>WT010_G07_CLUSTER_24946</td><td>gi|50929571|ref|XP_474313.1| OSJNBb0004A17.11 [Oryza sativa (japonica cultivar-group)] >gnl|BL_ORD_ID|1594426 gi|32488732|emb|CAE03609.1| OSJNBb0004A17.11 [Oryza sativa (japonica cultivar-group)] e_value:1.03352e-168</td><td>11670.m05703|LOC_Os04g57690|protein expressed protein e_value:3.54404e-170</td><td>0.265</td><td>(0.1518 0.5738)</td><td>0.1518</td><td>0.5738</td></tr>
<tr><td>3045</td><td>SET2_C21_CLUSTER_27594</td><td>gi|50915794|ref|XP_468361.1| F-box family protein-like [Oryza sativa (japonica cultivar-group)] >gi|51964510|ref|XP_507040.1| PREDICTED OSJNBa0053L11.25 gene product [Oryza sativa (japonica cultivar-group)] >gi|47848539|dbj|BAD22391.1| F-box family</td><td>11668.m05613|LOC_Os02g56840|protein probable phloem-specific lectin [imported] – Arabidopsis thaliana</td><td>0.235</td><td>(0.1724 0.7352)</td><td>0.1724</td><td>0.7352</td></tr>
<tr><td>3046</td><td>WT011_F09_CLUSTER_27044</td><td>gi|50916020|ref|XP_468474.1| putative AT-hook protein 1 [Oryza sativa (japonica cultivar-group)] >gnl|BL_ORD_ID|1583586 gi|48717090|dbj|BAD22863.1| putative AT-hook protein 1 [Oryza sativa (japonica cultivar-group)] >gnl|BL_ORD_ID|1583586 gi|4871631</td><td>11668.m05730|LOC_Os02g57820|protein probable DNA-binding protein – garden pea e_value:1.67947e-154</td><td>0.22</td><td>(0.1200 0.5447)</td><td>0.12</td><td>0.5447</td></tr>
<tr><td>3047</td><td>WT002_I12_CLUSTER_22828</td><td>gi|21262150|emb|CAD32683.1| acyl-[acyl-carrier protein] thioesterase [Triticum aestivum] e_value:3.12752e-167</td><td>11681.m02998|LOC_Os09g32760|protein acyl-[acyl-carrier protein] thioesterase e_value:1.89588e-169</td><td>0.202</td><td>(0.0985 0.4886)</td><td>0.0985</td><td>0.4886</td></tr>
<tr><td>3048</td><td>WT007_D02_CLUSTER_31706</td><td>gi|52353666|gb|AAU44232.1| hypothetical protein [Oryza sativa (japonica cultivar-group)] e_value:1.52006e-148</td><td>11682.m00353|LOC_Os05g04180|protein Similar to protein T31J12.4 [imported] – Arabidopsis thaliana e_value:6.04407e-</td><td>0.381</td><td>(0.2877 0.7551)</td><td>0.2877</td><td>0.7551</td></tr>
<tr><td>3049</td><td>WT005_M04_CLUSTER_398</td><td>gi|38566636|gb|AAR24208.1| At2g38130 [Arabidopsis thaliana] >gnl|BL_ORD_ID|2276137 gi|3335361|gb|AAC27162.1| putative acetyltransferase [Arabidopsis thaliana] >gnl|BL_ORD_ID|2276137 gi|46931260|gb|AAT06434.1| At2g38130 [Arabidopsis thaliana] >gnl</td><td>11687.m02969|LOC_Os11g32280|protein N-acetyltransferase homolog F16M14.6 – Arabidopsis thaliana e_value:9.29289e-99</td><td>0.074</td><td>(0.0475 0.6387)</td><td>0.0475</td><td>0.6387</td></tr>
<tr><td>3050</td><td>WT013_A09_CLUSTER_13961</td><td>gi|55297199|dbj|BAD68873.1| putative somatic embryogenesis protein kinase 1 [Oryza sativa (japonica cultivar-group)] e_value:0</td><td>11680.m00357|LOC_Os06g04370|protein Leucine Rich Repeat, putative e_value:0</td><td>0.129</td><td>(0.0878 0.6814)</td><td>0.0878</td><td>0.6814</td></tr>
<tr><td>3051</td><td>WT007_F06_CLUSTER_18829</td><td>gi|50948043|ref|XP_483549.1| receptor protein kinase PERK1-like protein [Oryza sativa (japonica cultivar-group)] >gnl|BL_ORD_ID|1610514 gi|38175551|dbj|BAD01244.1| receptor protein kinase PERK1-like protein [Oryza sativa (japonica cultivar-</td><td>11674.m04292|LOC_Os08g42580|protein Similar to contains similarity to receptor protein kinase~gene_id:MIL23.20 e_value:0</td><td>0.29</td><td>(0.1521 0.5239)</td><td>0.1521</td><td>0.5239</td></tr>
<tr><td>3052</td><td>WT005_P02_CLUSTER_34635</td><td>gi|13236654|gb|AAK16176.1| translation initiation factor 5A [Oryza sativa (japonica cultivar-group)] >gnl|BL_ORD_ID|2252714 gi|50918889|ref|XP_469841.1| translation initiation factor 5A [Oryza sativa (japonica cultivar-group)] >gnl|BL_ORD_ID|2252714</td><td>11669.m05548|LOC_Os03g55150|protein translation initiation factor 5A e_value:1.61521e-80</td><td>0.076</td><td>(0.0824 1.0786)</td><td>0.0824</td><td>1.0786</td></tr>
<tr><td>3053</td><td>WT007_E17_CLUSTER_7957</td><td>gi|62733017|gb|AAU89191.2| expressed protein [Oryza sativa (japonica cultivar-group)] e_value:1.55525e-128</td><td>11686.m04231|LOC_Os12g42430|protein gene_id:MKA23.10 e_value:1.86556e-153</td><td>0.345</td><td>(0.2086 0.6039)</td><td>0.2086</td><td>0.6039</td></tr>
<tr><td>3054</td><td>WT002_N13_CLUSTER_3231</td><td>gi|34906654|ref|NP_914674.1| P0431G06.23 [Oryza sativa (japonica cultivar-group)] >gnl|BL_ORD_ID|1540892 gi|20161860|dbj|BAB90773.1| RING finger-like protein [Oryza sativa (japonica cultivar-group)] >gnl|BL_ORD_ID|1540892</td><td>11667.m07272|LOC_Os01g72480|protein hypothetical protein e_value:6.38909e-79</td><td>0.231</td><td>(0.1420 0.6136)</td><td>0.142</td><td>0.6136</td></tr>
<tr><td>3055</td><td>SET6_I10_CLUSTER_3231</td><td>gi|34906654|ref|NP_914674.1| P0431G06.23 [Oryza sativa (japonica cultivar-group)] >gi|20161860|dbj|BAB90773.1| RING finger-like protein [Oryza sativa (japonica cultivar-group)] >gi|15528816|dbj|BAB64711.1| RING finger-like protein [Oryza sativa (jap</td><td>11667.m07272|LOC_Os01g72480|protein hypothetical protein e_value:6.38909e-79</td><td>0.231</td><td>(0.1420 0.6136)</td><td>0.142</td><td>0.6136</td></tr>
<tr><td>3056</td><td>WT006_I22_CLUSTER_1181</td><td>gi|7489413|pir||T06214 probable b-keto acyl reductase (EC 1.1.-.-) – barley >gnl|BL_ORD_ID|319616 gi|2586127|gb|AAB82766.1| b-keto acyl reductase [Hordeum vulgare] e_value:1.69953e-180</td><td>11668.m03679|LOC_Os02g38440|protein probable b-keto acyl reductase (EC 1.1.-.-) – barley e_value:1.18885e-167</td><td>0.14</td><td>(0.0581 0.4148)</td><td>0.0581</td><td>0.4148</td></tr>
<tr><td>3057</td><td>SET6_A15_CLUSTER_1106</td><td>gi|34897094|ref|NP_909893.1| unknown protein [Oryza sativa (japonica cultivar-group)] >gi|12957724|gb|AAK09242.1| unknown protein [Oryza sativa (japonica cultivar-group)] e_value:5.48435e-102</td><td>11669.m05744|LOC_Os03g56900|protein expressed protein e_value:1.13637e-103</td><td>0.124</td><td>(0.1106 0.8895)</td><td>0.1106</td><td>0.8895</td></tr>
<tr><td>3058</td><td>WT012_A17_CLUSTER_25272</td><td>gi|55297407|dbj|BAD69260.1| phosphatidylinositol 3- and 4-kinase family-like [Oryza sativa (japonica cultivar-group)] e_value:0</td><td>11680.m01720|LOC_Os06g17290|protein At2g03890/T18C20.9 e_value:0</td><td>0.195</td><td>(0.0947 0.4866)</td><td>0.0947</td><td>0.4866</td></tr>
<tr><td>3059</td><td>WT009_B21_CLUSTER_33122</td><td>gi|50948275|ref|XP_483665.1| typical P-type R2R3 Myb protein [Oryza sativa (japonica cultivar-group)] >gnl|BL_ORD_ID|1611197 gi|42407806|dbj|BAD08950.1| typical P-type R2R3 Myb protein [Oryza sativa (japonica cultivar-group)] e_value:8.76877e-99</td><td>11674.m04396|LOC_Os08g43550|protein Myb-like DNA-binding domain, putative e_value:1.76157e-100</td><td>0.487</td><td>(0.1959 0.4023)</td><td>0.1959</td><td>0.4023</td></tr>
<tr><td>3060</td><td>WT002_G24_CLUSTER_33074</td><td>gi|54291541|dbj|BAD61401.1| mitogen-activated protein kinase 7-like [Oryza sativa (japonica cultivar-group)] e_value:0</td><td>11667.m04260|LOC_Os01g43910|protein Protein kinase domain, putative e_value:0</td><td>0.132</td><td>(0.1547 1.1707)</td><td>0.1547</td><td>1.1707</td></tr>
<tr><td>3061</td><td>SET4_H01_CLUSTER_8959</td><td>gi|53793525|dbj|BAD54686.1| unknown protein [Oryza sativa (japonica cultivar-group)] e_value:0</td><td>11681.m03610|LOC_Os09g40000|protein expressed protein</td><td>0.259</td><td>(0.1285 0.4956)</td><td>0.1285</td><td>0.4956</td></tr>
<tr><td>3062</td><td>WT004_I06_CLUSTER_19556</td><td>gi|52076694|dbj|BAD45607.1| putative UDP-glucuronyltransferase-I [Oryza sativa (japonica cultivar-group)] >gnl|BL_ORD_ID|379068 gi|52077030|dbj|BAD46063.1| putative UDP-glucuronyltransferase-I [Oryza sativa (japonica cultivar-group)] e_value:0</td><td>11680.m04678|LOC_Os06g47340|protein Glycosyltransferase family 43, putative e_value:0</td><td>0.148</td><td>(0.0983 0.6638)</td><td>0.0983</td><td>0.6638</td></tr>
<tr><td>3063</td><td>SET1_J18_CLUSTER_13911</td><td>gi|52077363|dbj|BAD46403.1| putative glutaredoxin [Oryza sativa (japonica cultivar-group)] >gi|51536174|dbj|BAD38347.1| putative glutaredoxin [Oryza sativa (japonica cultivar-group)] e_value:1.93657e-54</td><td>11680.m04417|LOC_Os06g44910|protein Glutaredoxin, putative e_value:3.10546e-56</td><td>0.184</td><td>(0.0995 0.5418)</td><td>0.0995</td><td>0.5418</td></tr>
<tr><td>3064</td><td>WT002_D21_CLUSTER_14565</td><td>gi|55771342|dbj|BAD72267.1| unknown protein [Oryza sativa (japonica cultivar-group)] e_value:1.09652e-33</td><td>11686.m03933|LOC_Os12g39710|protein expressed protein e_value:1.42002e-66</td><td>0.553</td><td>(0.5502 0.9944)</td><td>0.5502</td><td>0.9944</td></tr>
<tr><td>3065</td><td>WT002_M14_CLUSTER_32884</td><td>gi|50912077|ref|XP_467446.1| putative NADPH-thioredoxin reductase [Oryza sativa (japonica cultivar-group)] >gnl|BL_ORD_ID|1580589 gi|51964302|ref|XP_506936.1| PREDICTED OJ1479_B12.9 gene product [Oryza sativa (japonica cultivar-group)] >gnl|BL_ORD_I</td><td>11668.m04687|LOC_Os02g48290|protein thioredoxin-disulfide reductase e_value:5.14253e-174</td><td>0.138</td><td>(0.0486 0.3522)</td><td>0.0486</td><td>0.3522</td></tr>
<tr><td>3066</td><td>SET1_H06_CLUSTER_24688</td><td>gi|50926698|ref|XP_473296.1| OSJNBb0039L24.13 [Oryza sativa (japonica cultivar-group)] >gi|32489830|emb|CAE04574.1| OSJNBb0039L24.13 [Oryza sativa (japonica cultivar-group)] e_value:5.78811e-91</td><td>11670.m04374|LOC_Os04g45070|protein Remorin, C-terminal region, putative e_value:1.12139e-92</td><td>0.129</td><td>(0.0715 0.5546)</td><td>0.0715</td><td>0.5546</td></tr>
<tr><td>3067</td><td>WT005_N14_CLUSTER_33596</td><td>gi|50919385|ref|XP_470089.1| putative methylenetetrahydrofolate reductase [Oryza sativa (japonica cultivar-group)] >gnl|BL_ORD_ID|1588159 gi|40786561|gb|AAR89836.1| putative methylenetetrahydrofolate reductase [Oryza sativa (japonica cultivar-group)] e</td><td>11669.m06107|LOC_Os03g60090|protein putative methylenetetrahydrofolate reductase e_value:0</td><td>0.069</td><td>(0.0476 0.6942)</td><td>0.0476</td><td>0.6942</td></tr>
<tr><td>3068</td><td>WT003_J23_CLUSTER_31801</td><td>gi|34334010|gb|AAQ64632.1| cytosolic glutathione reductase [Triticum monococcum] e_value:0</td><td>11668.m05614|LOC_Os02g56850|protein glutathione-disulfide reductase e_value:0</td><td>0.101</td><td>(0.0632 0.6248)</td><td>0.0632</td><td>0.6248</td></tr>
<tr><td>3069</td><td>SET5_M11_CLUSTER_33564</td><td>gi|62733231|gb|AAX95348.1| SNF7 family, putative [Oryza sativa (japonica cultivar-group)] e_value:7.47349e-108</td><td>11687.m04483|LOC_Os11g47710|protein SNF7 family, putative e_value:1.41954e-109</td><td>0.098</td><td>(0.0562 0.5751)</td><td>0.0562</td><td>0.5751</td></tr>
</table>

PDF Complete
Your complimentary use period has ended. Thank you for using PDF Complete.
Click Here to upgrade to Unlimited Pages and Expanded Features

<table>
<tr><th></th><th></th><th></th><th></th><th></th><th></th><th></th><th></th></tr>
<tr><td>3070</td><td>WT008_L14_CLUSTER_32125</td><td>gi|34903044|ref|NP_912869.1| unnamed protein product [Oryza sativa (japonica cultivar-group)] >gnl|BL_ORD_ID|1752357
gi|55700873|tpe|CAH69246.1| TPA: class III peroxidase 3 precursor [Oryza sativa (japonica cultivar-group)] >gnl|BL_ORD_ID|1752357 gi</td><td>11667.m01585|LOC_Os01g15830|protein Peroxidase, putative
e_value:1.46847e-168</td><td>0.128</td><td>(0.0700 0.5465)</td><td>0.07</td><td>0.5465</td></tr>
<tr><td>3071</td><td>WT003_A15_CLUSTER_16513</td><td>gi|58532131|emb|CAH68542.2| OSJNBa0009P12.20 [Oryza sativa (japonica cultivar-group)] e_value:2.85951e-123</td><td>11668.m04515|LOC_Os02g46660|protein hypothetical protein
e_value:2.20606e-120</td><td>0.08</td><td>(0.0862 1.0772)</td><td>0.0862</td><td>1.0772</td></tr>
<tr><td>3072</td><td>WT002_G20_CLUSTER_3696</td><td>gi|34897922|ref|NP_910307.1| unknown protein [Oryza sativa (japonica cultivar-group)] >gnl|BL_ORD_ID|1752565
gi|7248400|dbj|BAA92723.1| unknown protein [Oryza sativa (japonica cultivar-group)] e_value:0</td><td>11680.m00144|LOC_Os06g02370|protein expressed protein
e_value:0</td><td>0.109</td><td>(0.0703 0.6449)</td><td>0.0703</td><td>0.6449</td></tr>
<tr><td>3073</td><td>WT002_E09_CLUSTER_34092</td><td>gi|50936275|ref|XP_477665.1| putative CLB1 protein (calcium-dependent lipid binding) protein [Oryza sativa (japonica cultivar-group)]
>gnl|BL_ORD_ID|1527356 gi|33354209|dbj|BAC81175.1| putative CLB1 protein (calcium-dependent lipid binding) protein [Or</td><td>11673.m02087|LOC_Os07g22640|protein C2 domain, putative
e_value:0</td><td>0.132</td><td>(0.0592 0.4487)</td><td>0.0592</td><td>0.4487</td></tr>
<tr><td>3074</td><td>SET5_N19_CLUSTER_34918</td><td>gi|32352142|dbj|BAC78564.1| hypothetical protein [Oryza sativa (japonica cultivar-group)] >gi|50293119|gb|AAT72926.1| 17.7 kDa low
temperature induced protein [Oryza sativa (japonica cultivar-group)] e_value:1.91894e-47</td><td>11669.m02373|LOC_Os03g23010|protein expressed protein
e_value:2.31332e-49</td><td>0.567</td><td>(0.1635 0.2882)</td><td>0.1635</td><td>0.2882</td></tr>
<tr><td>3075</td><td>SET1_N14_CLUSTER_34681</td><td>gi|50915240|ref|XP_468084.1| putative UMP/CMP kinase a [Oryza sativa (japonica cultivar-group)] >gi|47497455|dbj|BAD19510.1|
putative UMP/CMP kinase a [Oryza sativa (japonica cultivar-group)] e_value:1.76209e-105</td><td>11668.m05284|LOC_Os02g53790|protein UMP-CMP kinase
family e_value:2.72496e-107</td><td>0.1</td><td>(0.0457 0.4568)</td><td>0.0457</td><td>0.4568</td></tr>
<tr><td>3076</td><td>WT004_B22_CLUSTER_722</td><td>gi|50725177|dbj|BAD33903.1| putative serine/threonine protein kinase [Oryza sativa (japonica cultivar-group)] e_value:0</td><td>11681.m00442|LOC_Os09g06230|protein serine/threonine
protein kinase - Arabidopsis thaliana e_value:0</td><td>0.101</td><td>(0.0537 0.5299)</td><td>0.0537</td><td>0.5299</td></tr>
<tr><td>3077</td><td>WT008_D19_CLUSTER_32881</td><td>gi|53749226|gb|AAU90086.1| unknown protein [Oryza sativa (japonica cultivar-group)] e_value:2.4529e-126</td><td>11682.m03458|LOC_Os05g36910|protein AT5g06370/MHF15_11
e_value:5.56932e-128</td><td>0.204</td><td>(0.0892 0.4376)</td><td>0.0892</td><td>0.4376</td></tr>
<tr><td>3078</td><td>WT004_D09_CLUSTER_33103</td><td>gi|33668156|gb|AAQ24632.1| GPRP [Oryza sativa (indica cultivar-group)] e_value:3.40218e-68</td><td>11682.m00201|LOC_Os05g02780|protein expressed protein
e_value:7.32392e-70</td><td>0.168</td><td>(0.1974 1.1771)</td><td>0.1974</td><td>1.1771</td></tr>
<tr><td>3079</td><td>SET5_A05_CLUSTER_34972</td><td>gi|50920031|ref|XP_470376.1| putative late embryogenesis abundant protein [Oryza sativa (japonica cultivar-group)]
>gi|41469632|gb|AAS07355.1| putative late embryogenesis abundant protein [Oryza sativa (japonica cultivar-group)] e_value:1.35121e-</td><td>11669.m06400|LOC_Os03g62620|protein putative late
embryogenesis abundant protein e_value:3.4275e-151</td><td>0.193</td><td>(0.1151 0.5973)</td><td>0.1151</td><td>0.5973</td></tr>
<tr><td>3080</td><td>WT006_O01_CLUSTER_22453</td><td>gi|50938755|ref|XP_478905.1| chitin-inducible gibberellin-responsive protein [Oryza sativa (japonica cultivar-group)]
>gnl|BL_ORD_ID|1529808 gi|51963506|ref|XP_506430.1| PREDICTED OJ1127_E01.113 gene product [Oryza sativa (japonica cultivar-
group)] &gt</td><td>11673.m03845|LOC_Os07g39470|protein GRAS family
transcription factor, putative e_value:0</td><td>0.105</td><td>(0.0775 0.7411)</td><td>0.0775</td><td>0.7411</td></tr>
<tr><td>3081</td><td>WT006_G09_CLUSTER_34605</td><td>gi|50905597|ref|XP_464287.1| putative heme activated protein [Oryza sativa (japonica cultivar-group)] >gnl|BL_ORD_ID|1566339
gi|49388078|dbj|BAD25190.1| putative heme activated protein [Oryza sativa (japonica cultivar-group)] >gnl|BL_ORD_ID|1566339</td><td>11668.m00705|LOC_Os02g07450|protein CCAAT-box binding
factor HAP5 homolog e_value:5.68428e-124</td><td>0.13</td><td>(0.0743 0.5715)</td><td>0.0743</td><td>0.5715</td></tr>
<tr><td>3082</td><td>WT003__C04_CLUSTER_32552</td><td>gi|4158230|emb|CAA77236.1| amylogenin [Triticum aestivum] e_value:0</td><td>11670.m05573|LOC_Os04g56520|protein Reversibly glycosylated
polypeptide e_value:1.76731e-180</td><td>0.113</td><td>(0.0710 0.6304)</td><td>0.071</td><td>0.6304</td></tr>
<tr><td>3083</td><td>SET1_E13_CLUSTER_31823</td><td>gi|50919191|ref|XP_469992.1| putative oxidoreductase [Oryza sativa (japonica cultivar-group)] >gi|29150365|gb|AAO72374.1| putative
oxidoreductase [Oryza sativa (japonica cultivar-group)] e_value:1.68769e-142</td><td>11669.m05974|LOC_Os03g58890|protein putative
oxidoreductase e_value:4.65085e-144</td><td>0.143</td><td>(0.0891 0.6233)</td><td>0.0891</td><td>0.6233</td></tr>
<tr><td>3084</td><td>WT010_A09_CLUSTER_34397</td><td>gi|34901996|ref|NP_912344.1| unknown protein [Oryza sativa (japonica cultivar-group)] >gnl|BL_ORD_ID|1588800
gi|29893582|gb|AAP06836.1| unknown protein [Oryza sativa (japonica cultivar-group)] e_value:9.19236e-171</td><td>11669.m01585|LOC_Os03g15860|protein Mitochondrial carrier
protein, putative e_value:2.29442e-172</td><td>0.146</td><td>(0.0572 0.3921)</td><td>0.0572</td><td>0.3921</td></tr>
<tr><td>3085</td><td>WT004_F21_CLUSTER_26876</td><td>gi|52077261|dbj|BAD46303.1| putative calmodulin-binding heat-shock protein [Oryza sativa (japonica cultivar-group)] e_value:0</td><td>11680.m01434|LOC_Os06g14490|protein calmodulin-binding
heat-shock protein - common tobacco e_value:0</td><td>0.29</td><td>(0.1087 0.3752)</td><td>0.1087</td><td>0.3752</td></tr>
<tr><td>3086</td><td>WT009_L14_CLUSTER_8039</td><td>gi|52077150|dbj|BAD46196.1| putative fructose/tagatose bisphosphate aldolase [Oryza sativa (japonica cultivar-group)] e_value:0</td><td>11680.m01459|LOC_Os06g14740|protein ketose-bisphosphate
aldolases, putative e_value:0</td><td>0.18</td><td>(0.0721 0.3996)</td><td>0.0721</td><td>0.3996</td></tr>
<tr><td>3087</td><td>SET4_B06_CLUSTER_13428</td><td>gi|51963600|ref|XP_506504.1| PREDICTED P0524G08.101 gene product [Oryza sativa (japonica cultivar-group)]
>gi|50939521|ref|XP_479288.1| unknown protein [Oryza sativa (japonica cultivar-group)] >gi|23237891|dbj|BAC16465.1| unknown
protein [Oryza sati</td><td>11673.m04393|LOC_Os07g44650|protein Complex 1 protein (LYR
family), putative e_value:4.04853e-63</td><td>0.24</td><td>(0.0692 0.2887)</td><td>0.0692</td><td>0.2887</td></tr>
<tr><td>3088</td><td>WT004_M06_CLUSTER_32077</td><td>gi|50908769|ref|XP_465873.1| ubiquitin-associated (UBA)/TS-N domain-containing protein-like [Oryza sativa (japonica cultivar-group)]
>gnl|BL_ORD_ID|1576453 gi|48716187|dbj|BAD23227.1| ubiquitin-associated (UBA)/TS-N domain-containing protein-like [Oryz</td><td>11668.m02866|LOC_Os02g30180|protein UBA/TS-N domain,
putative e_value:0</td><td>0.1</td><td>(0.0670 0.6728)</td><td>0.067</td><td>0.6728</td></tr>
<tr><td>3089</td><td>SET4_K16_CLUSTER_32279</td><td>gi|34912132|ref|NP_917413.1| OSJNBb0024F06.18 [Oryza sativa (japonica cultivar-group)] >gi|21644683|dbj|BAC01240.1| unknown
protein [Oryza sativa (japonica cultivar-group)] >gi|14588680|dbj|BAB61845.1| unknown protein [Oryza sativa (japonica cultiva</td><td>11667.m03870|LOC_Os01g40280|protein hypothetical protein
e_value:0</td><td>0.142</td><td>(0.0697 0.4907)</td><td>0.0697</td><td>0.4907</td></tr>
<tr><td>3090</td><td>WT005_I07_CLUSTER_644</td><td>gi|50933509|ref|XP_476282.1| putative acyl-CoA oxidase [Oryza sativa (japonica cultivar-group)] >gnl|BL_ORD_ID|1752528
gi|24413971|dbj|BAC22222.1| putative acyl-CoA oxidase [Oryza sativa (japonica cultivar-group)] e_value:0</td><td>11680.m00040|LOC_Os06g01390|protein Acyl-CoA oxidase,
putative e_value:0</td><td>0.077</td><td>(0.0452 0.5855)</td><td>0.0452</td><td>0.5855</td></tr>
<tr><td>3091</td><td>WT003_A14_CLUSTER_34634</td><td>gi|50938451|ref|XP_478753.1| floral organ regulator 2 [Oryza sativa (japonica cultivar-group)] >gnl|BL_ORD_ID|1529481
gi|51963488|ref|XP_506417.1| PREDICTED OJ1019_E02.4 gene product [Oryza sativa (japonica cultivar-group)] >gnl|BL_ORD_ID|1529481</td><td>11673.m03703|LOC_Os07g38130|protein Leucine Rich Repeat,
putative e_value:5.58164e-152</td><td>0.399</td><td>(0.1437 0.3606)</td><td>0.1437</td><td>0.3606</td></tr>
<tr><td>3092</td><td>WT003_K08_CLUSTER_31129</td><td>gi|53749302|gb|AAU90161.1| putative ubiquitin ligase SINAT5 [Oryza sativa (japonica cultivar-group)] e_value:0</td><td>11682.m01376|LOC_Os05g14860|protein Seven in absentia
protein family, putative e_value:0</td><td>0.072</td><td>(0.0502 0.6937)</td><td>0.0502</td><td>0.6937</td></tr>
<tr><td>3093</td><td>WT003_F24_CLUSTER_751</td><td>gi|31433420|gb|AAP54933.1| putative proline oxidase [Oryza sativa (japonica cultivar-group)] >gnl|BL_ORD_ID|2012958
gi|37536688|ref|NP_922646.1| putative proline oxidase [Oryza sativa (japonica cultivar-group)] >gnl|BL_ORD_ID|2012958 gi|10140631|gb|</td><td>11676.m03696|LOC_Os10g40360|protein putative proline oxidase
e_value:0</td><td>0.344</td><td>(0.1097 0.3186)</td><td>0.1097</td><td>0.3186</td></tr>
<tr><td>3094</td><td>WT004_H18_CLUSTER_31737</td><td>gi|34910406|ref|NP_916550.1| putative vesicle transport v-SNARE protein [Oryza sativa (japonica cultivar-group)]
>gnl|BL_ORD_ID|2386838 gi|19571103|dbj|BAB86528.1| vesicle transport v-SNARE (vesicle soluble NSF attachment protein receptor)</td><td>11667.m03630|LOC_Os01g37980|protein Vesicle transport v-
SNARE protein, putative e_value:4.30787e-79</td><td>0.196</td><td>(0.2406 1.2246)</td><td>0.2406</td><td>1.2246</td></tr>
<tr><td>3095</td><td>WT008_O15_CLUSTER_33729</td><td>gi|37694731|gb|AAQ99276.1| NADP malic enzyme [Oryza sativa (japonica cultivar-group)] >gnl|BL_ORD_ID|2261230
gi|54287505|gb|AAV31249.1| NADP malic enzyme [Oryza sativa (japonica cultivar-group)] e_value:0</td><td>11682.m00917|LOC_Os05g09440|protein Malic enzyme, NAD
binding domain, putative e_value:0</td><td>0.094</td><td>(0.0548 0.5805)</td><td>0.0548</td><td>0.5805</td></tr>
<tr><td>3096</td><td>SET4_M06_CLUSTER_30065</td><td>gi|50948809|ref|XP_493762.1| putative C-terminal zinc-finger [Oryza sativa (japonica cultivar-group)] >gi|8570055|dbj|BAA96760.1|
putative C-terminal zinc-finger [Oryza sativa (japonica cultivar-group)] >gi|9757680|dbj|BAB08199.1| ESTs AU075348(C112</td><td>11682.m00664|LOC_Os05g07070|protein Zinc finger, C3HC4
type (RING finger), putative e_value:1.9541e-99</td><td>0.435</td><td>(0.4974 1.1440)</td><td>0.4974</td><td>1.144</td></tr>
<tr><td>3097</td><td>WT005_L21_CLUSTER_27462</td><td>gi|50947571|ref|XP_483313.1| putative AT-hook DNA-binding protein [Oryza sativa (japonica cultivar-group)] >gnl|BL_ORD_ID|1609613
gi|42408801|dbj|BAD10062.1| putative AT-hook DNA-binding protein [Oryza sativa (japonica cultivar-group)] e_value:1.4644e-</td><td>11674.m04027|LOC_Os08g40150|protein expressed protein
e_value:1.25515e-136</td><td>0.202</td><td>(0.1194 0.5902)</td><td>0.1194</td><td>0.5902</td></tr>
<tr><td>3098</td><td>WT008_M08_CLUSTER_31530</td><td>gi|50582717|gb|AAT78787.1| putative macronuclear development protein [Oryza sativa (japonica cultivar-group)] e_value:4.81793e-109</td><td>11669.m00658|LOC_Os03g06980|protein At1g20220/T20H2_3
e_value:2.03126e-148</td><td>0.107</td><td>(0.0472 0.4390)</td><td>0.0472</td><td>0.439</td></tr>
<tr><td>3099</td><td>SET5_G10_CLUSTER_34376</td><td>gi|50253195|dbj|BAD29452.1| flavonoid 7-O-methyltransferase-like [Oryza sativa (japonica cultivar-group)]
>gi|50252861|dbj|BAD29092.1| flavonoid 7-O-methyltransferase-like [Oryza sativa (japonica cultivar-group)] e_value:1.49601e-109</td><td>11681.m01599|LOC_Os09g17560|protein O-methyltransferase,
putative e_value:4.28998e-111</td><td>0.305</td><td>(0.3132 1.0256)</td><td>0.3132</td><td>1.0256</td></tr>
<tr><td>3100</td><td>SET4_K04_CLUSTER_26370</td><td>gi|55733872|gb|AAV59379.1| putative early-responsive to dehydration stress protein (ERD4) [Oryza sativa (japonica cultivar-group)]
>gi|50933001|ref|XP_476028.1| putative early-responsive to dehydration stress protein (ERD4) [Oryza sativa (japonica cult</td><td>11682.m04988|LOC_Os05g51630|protein expressed protein
e_value:0</td><td>0.153</td><td>(0.1126 0.7353)</td><td>0.1126</td><td>0.7353</td></tr>
<tr><td>3101</td><td>WT006_K21_CLUSTER_31857</td><td>gi|55792424|gb|AAV65332.1| HGA2 [Hordeum vulgare] e_value:0</td><td>11667.m00203|LOC_Os01g02930|protein hypothetical protein
e_value:1.22357e-144</td><td>0.602</td><td>(0.2833 0.4705)</td><td>0.2833</td><td>0.4705</td></tr>
<tr><td>3102</td><td>WT013_K15_CLUSTER_13863</td><td>gi|52353685|gb|AAU44251.1| putative aminotransferase [Oryza sativa (japonica cultivar-group)] >gnl|BL_ORD_ID|1243917
gi|52353616|gb|AAU44182.1| putative aminotransferase class-III [Oryza sativa (japonica cultivar-group)] e_value:0</td><td>11682.m03777|LOC_Os05g39770|protein aminotransferase,
putative e_value:0</td><td>0.085</td><td>(0.0444 0.5198)</td><td>0.0444</td><td>0.5198</td></tr>
<tr><td>3103</td><td>WT006_G06_CLUSTER_7778</td><td>gi|50948061|ref|XP_483558.1| putative VIP2 protein [Oryza sativa (japonica cultivar-group)] >gnl|BL_ORD_ID|1610524
gi|50725677|dbj|BAD33143.1| putative VIP2 protein [Oryza sativa (japonica cultivar-group)] e_value:0</td><td>11674.m04299|LOC_Os08g42640|protein Zinc finger, C3HC4
type (RING finger), putative e_value:0</td><td>0.184</td><td>(0.0905 0.4912)</td><td>0.0905</td><td>0.4912</td></tr>
<tr><td>3104</td><td>WT007_E08_CLUSTER_31732</td><td>gi|50932273|ref|XP_475664.1| hypothetical protein [Oryza sativa (japonica cultivar-group)] >gnl|BL_ORD_ID|1598096
gi|48475189|gb|AAT44258.1| hypothetical protein [Oryza sativa (japonica cultivar-group)] e_value:0</td><td>11682.m04109|LOC_Os05g43230|protein cysteine proteinase
(EC 3.4.22.-) [similarity] - Arabidopsis thaliana e_value:0</td><td>0.216</td><td>(0.1130 0.5230)</td><td>0.113</td><td>0.523</td></tr>
<tr><td>3105</td><td>WT012_E16_CLUSTER_24116</td><td>gi|6523083|emb|CAB62341.1| cytochrome P450-like protein [Arabidopsis thaliana] >gnl|BL_ORD_ID|1451133 gi|53828589|gb|AAU94404.1|
At3g48520 [Arabidopsis thaliana] >gnl|BL_ORD_ID|1451133 gi|51536434|gb|AAU05455.1| At3g48520 [Arabidopsis thaliana] ></td><td>11687.m02666|LOC_Os11g29290|protein Cytochrome P450
e_value:0</td><td>0.322</td><td>(0.1612 0.4999)</td><td>0.1612</td><td>0.4999</td></tr>
<tr><td>3106</td><td>WT005_K21_CLUSTER_4913</td><td>No hits found</td><td>11674.m02352|LOC_Os08g24200|protein hypothetical protein
e_value:1.36134e-10</td><td>0.574</td><td>(0.3846 0.6704)</td><td>0.3846</td><td>0.6704</td></tr>
<tr><td>3107</td><td>WT003_J14_CLUSTER_4860</td><td>gi|31430514|gb|AAP52416.1| putative calcium-binding protein [Oryza sativa (japonica cultivar-group)] >gnl|BL_ORD_ID|2013638
gi|37531654|ref|NP_920129.1| putative calcium-binding protein [Oryza sativa (japonica cultivar-group)] >gnl|BL_ORD_ID|2013638</td><td>11669.m01443|LOC_Os03g14590|protein Similar to EF-hand
containing protein e_value:9.7362e-110</td><td>0.217</td><td>(0.1753 0.8093)</td><td>0.1753</td><td>0.8093</td></tr>
<tr><td>3108</td><td>WT002_B01_CLUSTER_33792</td><td>gi|56784865|dbj|BAD82105.1| putative bg55 [Oryza sativa (japonica cultivar-group)] e_value:0</td><td>11667.m06796|LOC_Os01g67500|protein U-box domain, putative
e_value:0</td><td>0.17</td><td>(0.1048 0.6150)</td><td>0.1048</td><td>0.615</td></tr>
<tr><td>3109</td><td>WT007_H20_CLUSTER_32455</td><td>gi|30102766|gb|AAP21301.1| At5g39990 [Arabidopsis thaliana] >gnl|BL_ORD_ID|1231816 gi|10176991|dbj|BAB10223.1| glycosylation
enzyme-like protein [Arabidopsis thaliana] >gnl|BL_ORD_ID|1231816 gi|15242532|ref|NP_198815.1| glycosyltransferase family 14</td><td>11680.m03906|LOC_Os06g40060|protein glycosylation enzyme-
like protein e_value:0</td><td>0.163</td><td>(0.0600 0.3685)</td><td>0.06</td><td>0.3685</td></tr>
<tr><td>3110</td><td>SET5_K05_CLUSTER_18338</td><td>gi|50919917|ref|XP_470319.1| putative deoxycytidine deaminase [Oryza sativa (japonica cultivar-group)] >gi|40714681|gb|AAR88587.1|
putative deoxycytidine deaminase [Oryza sativa (japonica cultivar-group)] e_value:8.84572e-97</td><td>11669.m06299|LOC_Os03g61810|protein putative deoxycytidine
deaminase e_value:1.80615e-98</td><td>0.053</td><td>(0.0395 0.7516)</td><td>0.0395</td><td>0.7516</td></tr>
<tr><td>3111</td><td>WT010_L13_CLUSTER_31764</td><td>gi|13940165|emb|CAC37797.1| BAX inhibitor 1 [Hordeum vulgare subsp. vulgare] e_value:3.46016e-133</td><td>11668.m00256|LOC_Os02g03280|protein bax inhibitor-1 (bi-1)
(osbi-1) e_value:2.65513e-123</td><td>0.113</td><td>(0.0525 0.4623)</td><td>0.0525</td><td>0.4623</td></tr>
<tr><td>3112</td><td>WT005_G11_CLUSTER_33143</td><td>gi|37703720|gb|AAR01225.1| putative aminotransferase AGD2 [Oryza sativa (japonica cultivar-group)] e_value:0</td><td>11669.m01908|LOC_Os03g18810|protein probable transaminase
(EC 2.6.1.-) T16L1.170 [similarity] - Arabidopsis thaliana</td><td>0.118</td><td>(0.0585 0.4966)</td><td>0.0585</td><td>0.4966</td></tr>
</table>

PDF Complete
Your complimentary use period has ended. Thank you for using PDF Complete.
Click Here to upgrade to Unlimited Pages and Expanded Features

3113	SET3_A06_CLUSTER_4783	gi|218181|dbj|BAA14402.1| unnamed protein product [Oryza sativa (japonica cultivar-group)] >gi|67644|pir||KHRZOA oryzain (EC 3.4.22.-) alpha precursor - rice >gi|129231|sp|P25776|ORYA_ORYSA Oryzain alpha chain precursor e_value:0	11670.m05480|LOC_Os04g55650|protein Similar to oryzain alpha chain precursor (ec 3.4.22.-). [rice] e_value:0	0.222	(0.1080 0.4869)	0.108	0.4869
3114	WT002_J07_CLUSTER_33007	gi|1084470|pir||S43953 cold acclimation protein - wheat >gnl|BL_ORD_ID|2307534 gi|1169018|sp|P46524|CO410_WHEAT Dehydrin COR410 (Cold-induced COR410 protein) >gnl|BL_ORD_ID|2307534 gi|457389|gb|AAA20189.1| cold acclimation protein e_value:8.71411e-	11668.m04329|LOC_Os02g44870|protein probable lipase - maize e_value:2.23984e-99	0.24	(0.1223 0.5103)	0.1223	0.5103
3115	SET6_A07_CLUSTER_33007	gi|1084470|pir||S43953 cold acclimation protein - wheat >gi|1169018|sp|P46524|CO410_WHEAT Dehydrin COR410 (Cold-induced COR410 protein) >gi|457389|gb|AAA20189.1| cold acclimation protein e_value:9.13193e-147	11668.m04329|LOC_Os02g44870|protein probable lipase - maize e_value:2.23984e-99	0.24	(0.1223 0.5103)	0.1223	0.5103
3116	WT002_G22_CLUSTER_34550	gi|20067423|emb|CAC94005.1| glutathione transferase [Triticum aestivum] e_value:2.78582e-127	11676.m03516|LOC_Os10g38720|protein putative glutathione S-transferase e_value:7.88123e-72	0.544	(0.2919 0.5365)	0.2919	0.5365
3117	SET6_C16_CLUSTER_34550	gi|20067423|emb|CAC94005.1| glutathione transferase [Triticum aestivum] e_value:1.34794e-139	11676.m03516|LOC_Os10g38720|protein putative glutathione S-transferase e_value:2.99486e-71	0.578	(0.3038 0.5255)	0.3038	0.5255
3118	SET1_O05_CLUSTER_34510	gi|50898394|ref|XP_449985.1| unknown protein [Oryza sativa (japonica cultivar-group)] >gi|46806423|dbj|BAD17580.1| unknown protein [Oryza sativa (japonica cultivar-group)] e_value:1.98213e-06	11681.m00122|LOC_Os09g02180|protein hypothetical protein e_value:4.6382e-08	0.821	(0.3943 0.4801)	0.3943	0.4801
3119	WT006_L17_CLUSTER_33198	gi|11993905|gb|AAG42149.1| mitochondrial processing peptidase alpha-chain precursor [Dactylis glomerata] e_value:0	11667.m00916|LOC_Os01g09560|protein mitochondrial processing peptidase alpha-chain precursor e_value:0	0.209	(0.0980 0.4694)	0.098	0.4694
3120	WT004_B18_CLUSTER_34698	gi|21740743|emb|CAD40552.1| OSJNBa0072K14.5 [Oryza sativa (japonica cultivar-group)] >gnl|BL_ORD_ID|1522091 gi|50923903|ref|XP_472312.1| OSJNBa0072K14.5 [Oryza sativa (japonica cultivar-group)] e_value:0	11670.m03088|LOC_Os04g32330|protein 2-oxoglutarate dehydrogenase, E2 component, dihydrolipoamide succinyltransferase e_value:0	0.114	(0.0463 0.4073)	0.0463	0.4073
3121	WT008_C22_CLUSTER_22447	gi|56784446|dbj|BAD82539.1| unknown protein [Oryza sativa (japonica cultivar-group)] e_value:0	11667.m04797|LOC_Os01g49310|protein expressed protein	0.451	(0.2257 0.5004)	0.2257	0.5004
3122	WT013_E13_CLUSTER_33694	gi|33151125|gb|AAP97431.1| cysteine protease CP1 [Oryza sativa (japonica cultivar-group)] >gnl|BL_ORD_ID|2092803 gi|52353572|gb|AAU44138.1| cysteine proteinase CP1 [Oryza sativa (japonica cultivar-group)] >gnl|BL_ORD_ID|2092803 gi|14719319|gb|AAK731	11682.m00097|LOC_Os05g01810|protein Papain family cysteine protease, putative e_value:4.13219e-161	0.272	(0.1331 0.4889)	0.1331	0.4889
3123	WT003_G08_CLUSTER_10520	gi|30689295|ref|NP_194945.3| eukaryotic pantothenate kinase family protein [Arabidopsis thaliana] e_value:0	11681.m03221|LOC_Os09g36270|protein pantothenate kinase, putative e_value:0	0.123	(0.0658 0.5371)	0.0658	0.5371
3124	WT002_O22_CLUSTER_19089	gi|62701690|gb|AAX92763.1| transposon protein, putative, unclassified [Oryza sativa (japonica cultivar-group)] e_value:2.92054e-174	11686.m00476|LOC_Os12g05420|protein IQ calmodulin-binding motif, putative e_value:0	0.197	(0.1044 0.5301)	0.1044	0.5301
3125	SET6_A23_CLUSTER_24942	gi|50920357|ref|XP_470539.1| Unknown protein [Oryza sativa (japonica cultivar-group)] >gi|27436759|gb|AAO13478.1| Unknown protein [Oryza sativa (japonica cultivar-group)] >gi|24960740|gb|AAN65434.1| Unknown protein [Oryza sativa (japonica cultivar-g	11669.m00797|LOC_Os03g08270|protein Smr domain, putative e_value:0	0.214	(0.1057 0.4933)	0.1057	0.4933
3126	WT008_J22_CLUSTER_12250	gi|31249736|gb|AAP46228.1| putative galactose kinase [Oryza sativa (japonica cultivar-group)] >gnl|BL_ORD_ID|1999819 gi|50919609|ref|XP_470165.1| putative galactose kinase [Oryza sativa (japonica cultivar-group)] e_value:0	11669.m06288|LOC_Os03g61710|protein putative galactose kinase e_value:0	0.13	(0.0694 0.5344)	0.0694	0.5344
3127	WT005_E02_CLUSTER_12942	gi|52077361|dbj|BAD46401.1| putative villin 2 [Oryza sativa (japonica cultivar-group)] >gnl|BL_ORD_ID|252497 gi|51536172|dbj|BAD38345.1| putative villin 2 [Oryza sativa (japonica cultivar-group)] e_value:0	11680.m04415|LOC_Os06g44890|protein actin filament bundling protein P-115-ABP e_value:0	0.146	(0.0750 0.5132)	0.075	0.5132
3128	SET5_K04_CLUSTER_4153	gi|50928039|ref|XP_473547.1| OSJNBb0032E06.11 [Oryza sativa (japonica cultivar-group)] >gi|38344377|emb|CAE02252.2| OSJNBb0032E06.11 [Oryza sativa (japonica cultivar-group)] e_value:1.35504e-144	11670.m04716|LOC_Os04g48130|protein Rhomboid family, putative e_value:3.61182e-146	0.483	(0.1561 0.3235)	0.1561	0.3235
3129	WT006_F20_CLUSTER_3520	gi|56783969|dbj|BAD81406.1| putative protein kinase [Oryza sativa (japonica cultivar-group)] e_value:0	11667.m01547|LOC_Os01g15470|protein Protein kinase domain, putative e_value:0	0.22	(0.1306 0.5940)	0.1306	0.594
3130	WT005_E24_CLUSTER_34431	gi|50933567|ref|XP_476311.1| ethylene-forming-enzyme-like dioxygenase-like protein [Oryza sativa (japonica cultivar-group)] >gnl|BL_ORD_ID|1743511 gi|24413984|dbj|BAC22235.1| putative iron/ascorbate-dependent oxidoreductase [Oryza sativa (japonica cult	11680.m00756|LOC_Os06g08060|protein oxidoreductase, 2OG-Fe(II) oxygenase family, putative e_value:9.46773e-131	0.292	(0.1967 0.6735)	0.1967	0.6735
3131	SET6_K18_CLUSTER_34551	gi|50921199|ref|XP_470960.1| OSJNBa0094O15.3 [Oryza sativa (japonica cultivar-group)] >gi|21741573|emb|CAD39335.1| OSJNBa0094O15.3 [Oryza sativa (japonica cultivar-group)] e_value:1.17815e-162	11670.m00030|LOC_Os04g01300|protein expressed protein e_value:9.07571e-165	0.075	(0.0445 0.5953)	0.0445	0.5953
3132	SET3_N17_CLUSTER_32664	gi|50659030|gb|AAT80328.1| UDP-D-glucuronate decarboxylase [Hordeum vulgare] e_value:0	11673.m04724|LOC_Os07g47700|protein NAD dependent epimerase/dehydratase family, putative e_value:0	0.334	(0.1305 0.3901)	0.1305	0.3901
3133	SET5_L23_CLUSTER_10308	gi|34910204|ref|NP_916449.1| putative flavonol glucosyltransferase [Oryza sativa (japonica cultivar-group)] >gi|15624027|dbj|BAB68081.1| putative arbutin synthase [Oryza sativa (japonica cultivar-group)] e_value:1.20391e-177	11667.m05234|LOC_Os01g53330|protein UDP-glucoronosyl and UDP-glucosyl transferase e_value:3.73253e-179	0.522	(0.2260 0.4327)	0.226	0.4327
3134	WT009_O03_CLUSTER_32114	gi|23504741|emb|CAD29476.1| glutathione transferase F3 [Triticum aestivum] e_value:4.60112e-100	11667.m02615|LOC_Os01g27210|protein Glutathione S-transferase, C-terminal domain, putative e_value:8.69231e-75	0.46	(0.2832 0.6161)	0.2832	0.6161
3135	SET2_O09_CLUSTER_32114	gi|23504741|emb|CAD29476.1| glutathione transferase F3 [Triticum aestivum] e_value:1.5053e-124	11667.m02615|LOC_Os01g27210|protein Glutathione S-transferase, C-terminal domain, putative e_value:6.99419e-77	0.426	(0.2465 0.5789)	0.2465	0.5789
3136	WT010_C09_CLUSTER_31239	gi|50948135|ref|XP_483595.1| ankyrin-like protein [Oryza sativa (japonica cultivar-group)] >gnl|BL_ORD_ID|1610560 gi|42407837|dbj|BAD08980.1| ankyrin-like protein [Oryza sativa (japonica cultivar-group)] e_value:0	11674.m04335|LOC_Os08g42960|protein expressed protein e_value:0	0.073	(0.0651 0.8913)	0.0651	0.8913
3137	WT012_P06_CLUSTER_31516	gi|31432618|gb|AAP54226.1| putative RNA-binding protein [Oryza sativa (japonica cultivar-group)] >gnl|BL_ORD_ID|2012112 gi|37535274|ref|NP_921939.1| putative RNA-binding protein [Oryza sativa (japonica cultivar-group)] >gnl|BL_ORD_ID|2012112 gi|1071	11676.m02922|LOC_Os10g33230|protein putative RNA binding protein e_value:0	0.149	(0.0807 0.5430)	0.0807	0.543
3138	WT003_J07_CLUSTER_31287	gi|50911981|ref|XP_467398.1| uridine kinase-like protein [Oryza sativa (japonica cultivar-group)] >gnl|BL_ORD_ID|1580537 gi|41053166|dbj|BAD08108.1| uridine kinase-like protein [Oryza sativa (japonica cultivar-group)] e_value:0	11668.m04644|LOC_Os02g47860|protein Adenylate cyclase, putative e_value:0	0.145	(0.0641 0.4434)	0.0641	0.4434
3139	WT006_A01_CLUSTER_1699	gi|50901862|ref|XP_463364.1| protein phosphatase 2C-like protein [Oryza sativa (japonica cultivar-group)] e_value:0	11682.m04973|LOC_Os05g51510|protein Protein phosphatase 2C e_value:3.39679e-167	0.18	(0.1966 1.0921)	0.1966	1.0921
3140	WT002_G02_CLUSTER_12528	gi|34908820|ref|NP_915757.1| P0557A01.33 [Oryza sativa (japonica cultivar-group)] >gnl|BL_ORD_ID|1546953 gi|20160842|dbj|BAB89782.1| putative drought-inducible protein 1OS [Oryza sativa (japonica cultivar-group)] >gnl|BL_ORD_ID|1546953	11667.m05761|LOC_Os01g58080|protein expressed protein e_value:0	0.217	(0.0786 0.3620)	0.0786	0.362
3141	WT005_H19_CLUSTER_34999	gi|50910713|ref|XP_466845.1| putative phenylalanine ammonia-lyase [Oryza sativa (japonica cultivar-group)] >gnl|BL_ORD_ID|1582699 gi|48716548|dbj|BAD23151.1| putative phenylalanine ammonia-lyase [Oryza sativa (japonica cultivar-group)] e_value:0	11668.m03974|LOC_Os02g41650|protein phenylalanine ammonia-lyase e_value:0	0.073	(0.0327 0.4503)	0.0327	0.4503
3142	WT004_G17_CLUSTER_34999	gi|50910709|ref|XP_466843.1| putative phenylalanine ammonia-lyase [Oryza sativa (japonica cultivar-group)] >gnl|BL_ORD_ID|1582705 gi|48716546|dbj|BAD23149.1| putative phenylalanine ammonia-lyase [Oryza sativa (japonica cultivar-group)] >gnl|BL_ORD_I	11668.m03971|LOC_Os02g41630|protein phenylalanine ammonia-lyase e_value:0	0.171	(0.0550 0.3209)	0.055	0.3209
3143	WT011_C16_CLUSTER_22012	gi|50905629|ref|XP_464303.1| putative copper-exporting ATPase [Oryza sativa (japonica cultivar-group)] >gnl|BL_ORD_ID|1566357 gi|49387577|dbj|BAD25508.1| putative copper-exporting ATPase [Oryza sativa (japonica cultivar-group)] e_value:0	11668.m00723|LOC_Os02g07630|protein copper-translocating P-type ATPase, putative e_value:0	0.152	(0.0782 0.5139)	0.0782	0.5139
3144	WT007_I04_CLUSTER_34653	gi|50932469|ref|XP_475762.1| unknown protein [Oryza sativa (japonica cultivar-group)] >gnl|BL_ORD_ID|1598359 gi|48843834|gb|AAT47093.1| unknown protein [Oryza sativa (japonica cultivar-group)] e_value:0	11682.m04396|LOC_Os05g45830|protein expressed protein e_value:0	0.404	(0.2055 0.5080)	0.2055	0.508
3145	WT011_F13_CLUSTER_32590	gi|34910732|ref|NP_916713.1| P0022F10.12 [Oryza sativa (japonica cultivar-group)] e_value:0	11667.m05146|LOC_Os01g52500|protein Malic enzyme, N-terminal domain, putative e_value:0	0.138	(0.0561 0.4076)	0.0561	0.4076
3146	WT007_P22_CLUSTER_10801	gi|50947329|ref|XP_483192.1| zinc metalloproteinase-like [Oryza sativa (japonica cultivar-group)] >gnl|BL_ORD_ID|1609440 gi|42407752|dbj|BAD08898.1| zinc metalloproteinase-like [Oryza sativa (japonica cultivar-group)] e_value:0	11674.m03917|LOC_Os08g39150|protein expressed protein e_value:0	0.226	(0.1219 0.5396)	0.1219	0.5396
3147	SET1_O11_CLUSTER_26967	gi|50935787|ref|XP_477421.1| putative B12D protein [Oryza sativa (japonica cultivar-group)] >gi|34395050|dbj|BAC84633.1| putative B12D protein [Oryza sativa (japonica cultivar-group)] >gi|50508850|dbj|BAD31625.1| putative B12D protein [Oryza sativa	11673.m01698|LOC_Os07g17330|protein B12D protein e_value:1.05733e-38	0.442	(0.2108 0.4765)	0.2108	0.4765
3148	WT006_C15_CLUSTER_14963	gi|51964536|ref|XP_507053.1| PREDICTED OJ1202_E07.22 gene product [Oryza sativa (japonica cultivar-group)] >gnl|BL_ORD_ID|1583556 gi|50915930|ref|XP_468429.1| putative protein serine/threonine kinase [Oryza sativa (japonica cultivar-group)]	11668.m05686|LOC_Os02g57420|protein protein kinase e_value:0	0.068	(0.0664 0.9805)	0.0664	0.9805
3149	WT004_A20_CLUSTER_18645	gi|53792690|dbj|BAD53702.1| putative CPRD2 [Oryza sativa (japonica cultivar-group)] e_value:0	11680.m03455|LOC_Os06g35700|protein berberine bridge enzyme e_value:0	0.44	(0.1713 0.3896)	0.1713	0.3896
3150	SET4_E03_CLUSTER_30293	gi|50905451|ref|XP_464214.1| putative Avr9 elicitor response protein [Oryza sativa (japonica cultivar-group)] >gi|49388048|dbj|BAD25162.1| putative Avr9 elicitor response protein [Oryza sativa (japonica cultivar-group)] e_value:0	11668.m00635|LOC_Os02g06840|protein Galactosyltransferase, putative e_value:0	0.095	(0.0558 0.5872)	0.0558	0.5872
3151	WT005_E16_CLUSTER_12276	gi|51964394|ref|XP_506982.1| PREDICTED P0627E03.33 gene product [Oryza sativa (japonica cultivar-group)] >gnl|BL_ORD_ID|1582986 gi|50912923|ref|XP_467869.1| putative UDP-glucose glucosyltransferase1 [Oryza sativa (japonica cultivar-group)] >gnl|BL_O	11668.m05077|LOC_Os02g51930|protein UDP-glucoronosyl and UDP-glucosyl transferase e_value:0	0.531	(0.1584 0.2983)	0.1584	0.2983
3152	WT005_O14_CLUSTER_1758	gi|62733684|gb|AAX95795.1| invertase, putative [Oryza sativa (japonica cultivar-group)] e_value:0	11687.m00682|LOC_Os11g07440|protein invertase, putative	0.049	(0.0355 0.7274)	0.0355	0.7274
3153	SET1_O24_CLUSTER_2731	gi|50938775|ref|XP_478915.1| putative heterogeneous nuclear ribonucleoprotein A1 [Oryza sativa (japonica cultivar-group)] >gi|38175737|dbj|BAC55617.2| putative heterogeneous nuclear ribonucleoprotein A1 [Oryza sativa (japonica cultivar-group)] e_value:	11673.m03857|LOC_Os07g39560|protein RNA recognition motif. (a.k.a. RRM, RBD, or RNP domain), putative e_value:0	0.129	(0.0546 0.4228)	0.0546	0.4228
3154	WT007_P24_CLUSTER_29965	gi|7576892|gb|AAF64042.1| fatty acid alpha-oxidase [Oryza sativa] e_value:0	11686.m02534|LOC_Os12g26290|protein expressed protein	0.328	(0.0369 0.1125)	0.0369	0.1125
3155	WT011_J02_CLUSTER_16935	gi|50919595|ref|XP_470158.1| putative hydrolase [Oryza sativa] >gnl|BL_ORD_ID|2389125 gi|28372700|gb|AAO39884.1| putative hydrolase [Oryza sativa (japonica cultivar-group)] >gnl|BL_ORD_ID|2389125 gi|18855051|gb|AAL79743.1| putative hydrolase [Oryza	11669.m06252|LOC_Os03g61360|protein putative hydrolase e_value:1.25017e-140	0.272	(0.1871 0.6882)	0.1871	0.6882
3156	SET1_E24_CLUSTER_11919	gi|51451358|gb|AAU03113.1| putative o-methyltransferase ZRP4 [Oryza sativa (japonica cultivar-group)] >gi|46575943|gb|AAT01304.1| putative o-methyltransferase ZRP4 [Oryza sativa (japonica cultivar-group)] e_value:2.05011e-138	11682.m04183|LOC_Os05g43940|protein o-methyltransferase zrp4 (ec 2.1.1.-) (omt). [maize e_value:5.70167e-140	0.426	(0.2094 0.4917)	0.2094	0.4917

PDF Complete
Your complimentary use period has ended. Thank you for using PDF Complete.
Click Here to upgrade to Unlimited Pages and Expanded Features

<table>
<tr><th></th><th></th><th></th><th></th><th></th><th></th><th></th></tr>
<tr><td>3157</td><td>WT008_H06_CLUSTER_31931</td><td>gi|50510121|dbj|BAD30889.1| glucose/sorbosone dehydrogenases-like protein [Oryza sativa (japonica cultivar-group)] >gnl|BL_ORD_ID|233971 gi|50509091|dbj|BAD30151.1| glucose/sorbosone dehydrogenases-like protein [Oryza sativa (japonica cultivar-group)]</td><td>11673.m00894|LOC_Os07g09520|protein hipl1 protein precursor e_value:0</td><td>0.351</td><td>(0.1596 0.4549)</td><td>0.1596</td><td>0.4549</td></tr>
<tr><td>3158</td><td>SET5_A02_CLUSTER_33807</td><td>gi|18539471|gb|AAL74406.1| JIOsPR10 [Oryza sativa] >gi|16589076|gb|AAL27005.1| pathogen-related protein [Oryza sativa] e_value:3.6014e-66</td><td>11669.m01912|LOC_Os03g18850|protein pathogen-related protein e_value:5.47501e-68</td><td>0.454</td><td>(0.1232 0.2716)</td><td>0.1232</td><td>0.2716</td></tr>
<tr><td>3159</td><td>SET4_A15_CLUSTER_32678</td><td>gi|2920666|gb|AAC18566.1| 2,4-D inducible glutathione S-transferase [Glycine max] >gi|7447302|pir||T06239 probable glutathione transferase (EC 2.5.1.18), 2,4-D inducible - soybean e_value:3.32968e-61</td><td>11673.m00497|LOC_Os07g05800|protein probable glutathione transferase (EC 2.5.1.18), 2,4-D inducible - soybean e_value:3.71185e-73</td><td>0.183</td><td>(0.1907 1.0414)</td><td>0.1907</td><td>1.0414</td></tr>
<tr><td>3160</td><td>SET1_K11_CLUSTER_9493</td><td>gi|50948351|ref|XP_483703.1| hypothetical protein [Oryza sativa (japonica cultivar-group)] >gi|42408962|dbj|BAD10218.1| hypothetical protein [Oryza sativa (japonica cultivar-group)] >gi|50725532|dbj|BAD33001.1| hypothetical protein [Oryza sativa (ja</td><td>11674.m04428|LOC_Os08g43870|protein hypothetical protein e_value:1.55021e-77</td><td>0.313</td><td>(0.2145 0.6856)</td><td>0.2145</td><td>0.6856</td></tr>
<tr><td>3161</td><td>SET1_I16_CLUSTER_9202</td><td>gi|14164983|gb|AAK55326.1| thaumatin-like protein TLP8 [Hordeum vulgare] e_value:6.00467e-129</td><td>11669.m04584|LOC_Os03g45960|protein putative antifungal zeamatin-like protein e_value:8.1085e-108</td><td>0.465</td><td>(0.1453 0.3125)</td><td>0.1453</td><td>0.3125</td></tr>
<tr><td>3162</td><td>SET1_G24_CLUSTER_25515</td><td>gi|5360659|dbj|BAA82095.1| anthranilate synthase alpha 2 subunit [Oryza sativa (japonica cultivar-group)] e_value:0</td><td>11669.m01576|LOC_Os03g15780|protein anthranilate synthase component I e_value:0</td><td>0.157</td><td>(0.0673 0.4278)</td><td>0.0673</td><td>0.4278</td></tr>
<tr><td>3163</td><td>SET1_B13_CLUSTER_3371</td><td>gi|100588|pir||A36070 L-lactate dehydrogenase (EC 1.1.1.27) chain A - barley >gi|167067|gb|AAA62696.1| L-lactate dehydrogenase >gi|126033|sp|P22988|LDHA_HORVU L-lactate dehydrogenase A (LDH-A) e_value:0</td><td>11668.m00053|LOC_Os02g01510|protein L-lactate dehydrogenase e_value:4.66972e-168</td><td>0.182</td><td>(0.0866 0.4762)</td><td>0.0866</td><td>0.4762</td></tr>
<tr><td>3164</td><td>WT012_P01_CLUSTER_13864</td><td>gi|9755654|emb|CAC01807.1| putative protein [Arabidopsis thaliana] >gnl|BL_ORD_ID|1487609 gi|21280967|gb|AAM44955.1| unknown protein [Arabidopsis thaliana] >gnl|BL_ORD_ID|1487609 gi|13878129|gb|AAK44142.1| unknown protein [Arabidopsis thaliana] ></td><td>11669.m01521|LOC_Os03g15270|protein expressed protein e_value:1.31817e-170</td><td>0.285</td><td>(0.0997 0.3505)</td><td>0.0997</td><td>0.3505</td></tr>
<tr><td>3165</td><td>WT012_M08_CLUSTER_1022</td><td>gi|31430487|gb|AAP52396.1| putative wall-associated protein kinase [Oryza sativa (japonica cultivar-group)] >gnl|BL_ORD_ID|2008768 gi|37531614|ref|NP_920109.1| putative wall-associated protein kinase [Oryza sativa (japonica cultivar-group)] >gnl|BL_</td><td>11676.m00838|LOC_Os10g09620|protein Protein kinase domain, putative e_value:0</td><td>0.225</td><td>(0.3100 1.3801)</td><td>0.31</td><td>1.3801</td></tr>
<tr><td>3166</td><td>WT012_L14_CLUSTER_16711</td><td>gi|50931301|ref|XP_475178.1| unknown protein [Oryza sativa (japonica cultivar-group)] >gnl|BL_ORD_ID|1596399 gi|54287605|gb|AAV31349.1| unknown protein [Oryza sativa (japonica cultivar-group)] >gnl|BL_ORD_ID|1596399 gi|48926649|gb|AAT47438.1| unknow</td><td>11682.m03016|LOC_Os05g32320|protein folate/biopterin transporter e_value:0</td><td>0.195</td><td>(0.0767 0.3928)</td><td>0.0767</td><td>0.3928</td></tr>
<tr><td>3167</td><td>WT012_J22_CLUSTER_29094</td><td>gi|20514810|gb|AAM23255.1| Putative disease resistance protein [Oryza sativa (japonica cultivar-group)] e_value:0</td><td>11676.m00320|LOC_Os10g04110|protein NB-ARC domain, putative e_value:0</td><td>0.317</td><td>(0.3699 1.1654)</td><td>0.3699</td><td>1.1654</td></tr>
<tr><td>3168</td><td>WT012_G05_CLUSTER_29612</td><td>gi|50936677|ref|XP_477866.1| integral membrane protein-like [Oryza sativa (japonica cultivar-group)] >gnl|BL_ORD_ID|1526072 gi|34394242|dbj|BAC84694.1| integral membrane protein-like [Oryza sativa (japonica cultivar-group)] e_value:5.88203e-65</td><td>11673.m02444|LOC_Os07g26110|protein plant integral membrane protein TIGR01569, putative e_value:1.09025e-66</td><td>0.439</td><td>(0.1816 0.4138)</td><td>0.1816</td><td>0.4138</td></tr>
<tr><td>3169</td><td>WT012_E05_CLUSTER_8582</td><td>gi|50912843|ref|XP_467829.1| putative phosphoglycerate mutase [Oryza sativa (japonica cultivar-group)] >gnl|BL_ORD_ID|1582946 gi|46390222|dbj|BAD15653.1| putative phosphoglycerate mutase [Oryza sativa (japonica cultivar-group)] e_value:1.09656e-151</td><td>11668.m05039|LOC_Os02g51590|protein phosphoglycerate mutase 1 family, putative e_value:1.02614e-153</td><td>0.131</td><td>(0.1164 0.8913)</td><td>0.1164</td><td>0.8913</td></tr>
<tr><td>3170</td><td>WT011_A04_CLUSTER_22953</td><td>gi|50911529|ref|XP_467172.1| YbaK/prolyl-tRNA synthetase-like [Oryza sativa (japonica cultivar-group)] >gnl|BL_ORD_ID|1580272 gi|50251742|dbj|BAD27675.1| YbaK/prolyl-tRNA synthetase-like [Oryza sativa (japonica cultivar-group)] >gnl|BL_ORD_ID|158027</td><td>11668.m04270|LOC_Os02g44330|protein YbaK / prolyl-tRNA synthetases associated domain, putative e_value:1.86328e-107</td><td>0.202</td><td>(0.0762 0.3782)</td><td>0.0762</td><td>0.3782</td></tr>
<tr><td>3171</td><td>WT010_P09_CLUSTER_17167</td><td>gi|32490271|emb|CAE05560.1| OSJNBb0116K07.13 [Oryza sativa (japonica cultivar-group)] >gnl|BL_ORD_ID|1536962 gi|38345166|emb|CAE02948.2| OSJNBa0014K14.20 [Oryza sativa (japonica cultivar-group)] >gnl|BL_ORD_ID|1536962 gi|50926284|ref|XP_473089.1| OS</td><td>11670.m04112|LOC_Os04g42620|protein extensin-like protein e_value:0</td><td>0.557</td><td>(0.1996 0.3583)</td><td>0.1996</td><td>0.3583</td></tr>
<tr><td>3172</td><td>WT010_E07_CLUSTER_4711</td><td>gi|50902030|ref|XP_463448.1| P0512C01.22 [Oryza sativa (japonica cultivar-group)] >gnl|BL_ORD_ID|2302335 gi|20804680|dbj|BAB92368.1| phospholipase-like [Oryza sativa (japonica cultivar-group)] >gnl|BL_ORD_ID|2302335</td><td>11667.m05492|LOC_Os01g55650|protein Patatin-like phospholipase, putative e_value:0</td><td>0.251</td><td>(0.1135 0.4519)</td><td>0.1135</td><td>0.4519</td></tr>
<tr><td>3173</td><td>WT009_N15_CLUSTER_1559</td><td>gi|56201783|dbj|BAD73233.1| HEAT repeat-containing protein-like [Oryza sativa (japonica cultivar-group)] e_value:0</td><td>11667.m03121|LOC_Os01g33060|protein HEAT repeat, putative e_value:0</td><td>0.166</td><td>(0.0824 0.4957)</td><td>0.0824</td><td>0.4957</td></tr>
<tr><td>3174</td><td>WT009_I22_CLUSTER_21830</td><td>gi|50940185|ref|XP_479620.1| putative phosphoinositide-specific phospholipase C [Oryza sativa (japonica cultivar-group)] >gnl|BL_ORD_ID|1534745 gi|34394025|dbj|BAC84056.1| putative phosphoinositide-specific phospholipase C [Oryza sativa (japonica culti</td><td>11673.m04909|LOC_Os07g49330|protein Phosphatidylinositol-specific phospholipase C, X domain, putative e_value:0</td><td>0.234</td><td>(0.1387 0.5916)</td><td>0.1387</td><td>0.5916</td></tr>
<tr><td>3175</td><td>WT008__C03_CLUSTER_1559</td><td>gi|56201783|dbj|BAD73233.1| HEAT repeat-containing protein-like [Oryza sativa (japonica cultivar-group)] e_value:0</td><td>11667.m03121|LOC_Os01g33060|protein HEAT repeat, putative e_value:0</td><td>0.166</td><td>(0.0824 0.4957)</td><td>0.0824</td><td>0.4957</td></tr>
<tr><td>3176</td><td>WT005_O22_CLUSTER_16828</td><td>gi|50902190|ref|XP_463528.1| putative amino acid transport protein [Oryza sativa (japonica cultivar-group)] >gnl|BL_ORD_ID|1558781 gi|20161442|dbj|BAB90366.1| putative amino acid carrier [Oryza sativa (japonica cultivar-group)] e_value:0</td><td>11667.m06632|LOC_Os01g66010|protein Transmembrane amino acid transporter protein e_value:0</td><td>0.259</td><td>(0.0899 0.3470)</td><td>0.0899</td><td>0.347</td></tr>
<tr><td>3177</td><td>WT004_O07_CLUSTER_2196</td><td>gi|38346908|emb|CAE03880.2| OSJNBb0015N08.8 [Oryza sativa (japonica cultivar-group)] >gnl|BL_ORD_ID|1535337 gi|50928537|ref|XP_473796.1| OSJNBb0015N08.8 [Oryza sativa (japonica cultivar-group)] >gnl|BL_ORD_ID|1535337 gi|46394390|tpg|DAA05133.1| TPA:</td><td>11670.m05041|LOC_Os04g51560|protein WRKY DNA -binding domain, putative e_value:9.34956e-138</td><td>0.328</td><td>(0.1262 0.3843)</td><td>0.1262</td><td>0.3843</td></tr>
<tr><td>3178</td><td>WT004_M17_CLUSTER_17378</td><td>gi|50907653|ref|XP_465315.1| putative HGA1 [Oryza sativa (japonica cultivar-group)] >gnl|BL_ORD_ID|1569379 gi|46389948|dbj|BAD15800.1| putative HGA1 [Oryza sativa (japonica cultivar-group)] e_value:0</td><td>11668.m02166|LOC_Os02g22380|protein At3g18170 e_value:0</td><td>0.357</td><td>(0.1215 0.3398)</td><td>0.1215</td><td>0.3398</td></tr>
<tr><td>3179</td><td>WT004_L16_CLUSTER_7301</td><td>gi|34907082|ref|NP_914888.1| OSJNBa0052O12.6 [Oryza sativa (japonica cultivar-group)] e_value:4.16879e-107</td><td>11667.m07046|LOC_Os01g70330|protein Prefoldin subunit, putative e_value:3.42597e-132</td><td>0.361</td><td>(0.1722 0.4774)</td><td>0.1722</td><td>0.4774</td></tr>
<tr><td>3180</td><td>WT004_F18_CLUSTER_2699</td><td>gi|50252365|dbj|BAD28472.1| unknown protein [Oryza sativa (japonica cultivar-group)] e_value:0</td><td>11681.m01621|LOC_Os09g17760|protein expressed protein</td><td>0.13</td><td>(0.0696 0.5356)</td><td>0.0696</td><td>0.5356</td></tr>
<tr><td>3181</td><td>WT003_F05_CLUSTER_8734</td><td>gi|50906467|ref|XP_464722.1| unknown protein [Oryza sativa (japonica cultivar-group)] >gnl|BL_ORD_ID|1567272 gi|46805637|dbj|BAD17056.1| unknown protein [Oryza sativa (japonica cultivar-group)] >gnl|BL_ORD_ID|1567272 gi|46806542|dbj|BAD17655.1| unkn</td><td>11668.m01202|LOC_Os02g13060|protein hypothetical protein e_value:7.84102e-52</td><td>0.362</td><td>(0.2465 0.6810)</td><td>0.2465</td><td>0.681</td></tr>
<tr><td>3182</td><td>WT003_D14_CLUSTER_1334</td><td>gi|56202242|dbj|BAD73683.1| glutaredoxin-like [Oryza sativa (japonica cultivar-group)] >gnl|BL_ORD_ID|547547 gi|56202177|dbj|BAD73655.1| glutaredoxin-like [Oryza sativa (japonica cultivar-group)] e_value:1.83113e-42</td><td>11682.m04691|LOC_Os05g48930|protein Glutaredoxin, putative e_value:1.21775e-46</td><td>0.483</td><td>(0.1448 0.2999)</td><td>0.1448</td><td>0.2999</td></tr>
<tr><td>3183</td><td>SET5_K13_CLUSTER_13799</td><td>gi|66813790|ref|XP_641074.1| hypothetical protein DDB0206068 [Dictyostelium discoideum] >gi|60469107|gb|EAL67103.1| hypothetical protein DDB0206068 [Dictyostelium discoideum] e_value:1.55408e-07</td><td>11680.m02092|LOC_Os06g21400|protein hypothetical protein e_value:2.21357e-10</td><td>0.488</td><td>(0.2708 0.5552)</td><td>0.2708</td><td>0.5552</td></tr>
<tr><td>3184</td><td>SET4_O03_CLUSTER_31036</td><td>gi|51090448|dbj|BAD35400.1| putative 2'-hydroxyisoflavone reductase [Oryza sativa (japonica cultivar-group)] e_value:2.33354e-105</td><td>11680.m02648|LOC_Os06g27770|protein isoflavone reductase related protein e_value:5.03753e-107</td><td>0.158</td><td>(0.2965 1.8778)</td><td>0.2965</td><td>1.8778</td></tr>
<tr><td>3185</td><td>SET4__C14_CLUSTER_24080</td><td>gi|51979393|ref|XP_507543.1| PREDICTED OJ1695_H09.29 gene product [Oryza sativa (japonica cultivar-group)] >gi|51964486|ref|XP_507028.1| PREDICTED OJ1695_H09.29 gene product [Oryza sativa (japonica cultivar-group)] >gi|50915604|ref|XP_468266.1| puta</td><td>11668.m05453|LOC_Os02g55320|protein response regulator 9 e_value:0</td><td>0.277</td><td>(0.1525 0.5514)</td><td>0.1525</td><td>0.5514</td></tr>
<tr><td>3186</td><td>WT010_F07_CLUSTER_11973</td><td>gi|3169012|emb|CAA05637.1| putative calmodulin binding transporter protein [Hordeum vulgare subsp. vulgare] >gnl|BL_ORD_ID|1312146 gi|7489415|pir||T04424 probable calmodulin binding transport protein - barley e_value:0</td><td>11680.m03236|LOC_Os06g33570|protein probable calmodulin binding transport protein - barley e_value:0</td><td>0.192</td><td>(0.0990 0.5151)</td><td>0.099</td><td>0.5151</td></tr>
<tr><td>3187</td><td>WT013_D06_CLUSTER_32601</td><td>gi|50917433|ref|XP_469113.1| putative DNA-binding protein [Oryza sativa (japonica cultivar-group)] >gnl|BL_ORD_ID|1586096 gi|27764684|gb|AAO23109.1| putative DNA-binding protein [Oryza sativa (japonica cultivar-group)] e_value:0</td><td>11669.m04561|LOC_Os03g45730|protein Transposable element protein, putative e_value:0</td><td>0.413</td><td>(0.2726 0.6593)</td><td>0.2726</td><td>0.6593</td></tr>
<tr><td>3188</td><td>SET2_E03_CLUSTER_15949</td><td>gi|57900138|dbj|BAD88200.1| putative auxin response factor [Oryza sativa (japonica cultivar-group)] e_value:0</td><td>11667.m07039|LOC_Os01g70270|protein auxin response factor 2 e_value:0</td><td>0.2</td><td>(0.1133 0.5665)</td><td>0.1133</td><td>0.5665</td></tr>
<tr><td>3189</td><td>SET3_D01_CLUSTER_3056</td><td>gi|50947161|ref|XP_483108.1| C2 domain/GRAM domain-containing protein-like [Oryza sativa (japonica cultivar-group)] >gi|42408774|dbj|BAD10009.1| C2 domain/GRAM domain-containing protein-like [Oryza sativa (japonica cultivar-group)] e_value:0</td><td>11674.m03833|LOC_Os08g38440|protein C2 domain, putative e_value:0</td><td>0.239</td><td>(0.1076 0.4506)</td><td>0.1076</td><td>0.4506</td></tr>
<tr><td>3190</td><td>WT007_D01_CLUSTER_8896</td><td>gi|50899228|ref|XP_450402.1| BolA-like family protein / Fe-S metabolism associated domain-containing protein-like [Oryza sativa (japonica cultivar-group)] >gnl|BL_ORD_ID|1563672 gi|48717025|dbj|BAD23715.1| putative plastid protein SufE [Oryza sativa (j</td><td>11681.m00808|LOC_Os09g09790|protein Fe-S metabolism associated domain, putative e_value:1.95535e-150</td><td>0.309</td><td>(0.1278 0.4141)</td><td>0.1278</td><td>0.4141</td></tr>
<tr><td>3191</td><td>SET5_B08_CLUSTER_4189</td><td>gi|50928713|ref|XP_473884.1| OSJNBa0008M17.8 [Oryza sativa (japonica cultivar-group)] >gi|38344255|emb|CAE04335.2| OSJNBa0008M17.8 [Oryza sativa (japonica cultivar-group)] e_value:1.40378e-15</td><td>11674.m01000|LOC_Os08g10500|protein expressed protein e_value:9.81604e-19</td><td>0.433</td><td>(0.2288 0.5282)</td><td>0.2288</td><td>0.5282</td></tr>
<tr><td>3192</td><td>WT012_B11_CLUSTER_27047</td><td>gi|22022398|gb|AAL47687.1| glutathione-S-transferase Cla47 [Triticum aestivum] e_value:1.3528e-84</td><td>11676.m03515|LOC_Os10g38710|protein putative glutathione S-transferase e_value:1.58837e-82</td><td>0.538</td><td>(0.2421 0.4503)</td><td>0.2421</td><td>0.4503</td></tr>
<tr><td>3193</td><td>WT002_J11_CLUSTER_13787</td><td>gi|50252082|dbj|BAD28012.1| unknown protein [Oryza sativa (japonica cultivar-group)] e_value:0</td><td>11668.m00802|LOC_Os02g08330|protein expressed protein</td><td>0.364</td><td>(0.1426 0.3922)</td><td>0.1426</td><td>0.3922</td></tr>
<tr><td>3194</td><td>SET1__C22_CLUSTER_13320</td><td>gi|42558472|gb|AAS19930.1| allantoin permease [Phaseolus vulgaris] e_value:3.333e-162</td><td>11686.m03100|LOC_Os12g31850|protein allantoin permease e_value:0</td><td>0.09</td><td>(0.0529 0.5888)</td><td>0.0529</td><td>0.5888</td></tr>
<tr><td>3195</td><td>WT008_P11_CLUSTER_7007</td><td>gi|50947767|ref|XP_483411.1| putative auxin-induced protein [Oryza sativa (japonica cultivar-group)] >gnl|BL_ORD_ID|1609736 gi|42761399|dbj|BAD11564.1| putative auxin-induced protein [Oryza sativa (japonica cultivar-group)] >gnl|BL_ORD_ID|1609736 gi</td><td>11674.m04151|LOC_Os08g41280|protein hypothetical protein e_value:1.87611e-173</td><td>0.286</td><td>(0.1435 0.5021)</td><td>0.1435</td><td>0.5021</td></tr>
<tr><td>3196</td><td>WT013_A20_CLUSTER_26807</td><td>gi|50929141|ref|XP_474098.1| OSJNBa0033G05.21 [Oryza sativa (japonica cultivar-group)] >gnl|BL_ORD_ID|1594377 gi|38344897|emb|CAD41920.2| OSJNBa0033G05.21 [Oryza sativa (japonica cultivar-group)] >gnl|BL_ORD_ID|1594377 gi|32487348|emb|CAE03170.1| OS</td><td>11670.m05432|LOC_Os04g55220|protein Ca2+-dependent lipid-binding protein, putative e_value:0</td><td>0.13</td><td>(0.0725 0.5569)</td><td>0.0725</td><td>0.5569</td></tr>
<tr><td>3197</td><td>WT003__C22_CLUSTER_34997</td><td>gi|586082|sp|P37115|TCMO_PHAAU Trans-cinnamate 4-monooxygenase (Cinnamic acid 4-hydroxylase) (CA4H) (C4H) (P450C4H) (Cytochrome P450 73) >gnl|BL_ORD_ID|2377797 gi|169325|gb|AAA33755.1| cinnamate 4-hydroxylase [Vigna radiata var. radiata]</td><td>11667.m06018|LOC_Os01g60450|protein trans-cinnamic acid hydroxylase e_value:0</td><td>0.397</td><td>(0.0970 0.2447)</td><td>0.097</td><td>0.2447</td></tr>
</table>

PDF Complete
Your complimentary use period has ended. Thank you for using PDF Complete.
Click Here to upgrade to Unlimited Pages and Expanded Features

<table>
<tr><td>3198</td><td>WT006_I13_CLUSTER_10622</td><td>gi|53749382|gb|AAU90241.1| putative ferredoxin sulfite reductase [Oryza sativa (japonica cultivar-group)] >gnl|BL_ORD_ID|1285197 gi|53749386|gb|AAU90245.1| putative ferredoxin-sulfite reductase [Oryza sativa (japonica cultivar-group)] e_value:0</td><td>11682.m04070|LOC_Os05g42350|protein sulfite reductase, ferredoxin dependent e_value:0</td><td>0.062</td><td>(0.0380 0.6086)</td><td>0.038</td><td>0.6086</td></tr>
<tr><td>3199</td><td>SET4_D17_CLUSTER_5470</td><td>gi|31431852|gb|AAP53571.1| putative translation initiation factor 2B beta subunit [Oryza sativa (japonica cultivar-group)] >gi|37533964|ref|NP_921284.1| putative translation initiation factor 2B beta subunit [Oryza sativa (japonica cultivar-group)] &gt</td><td>11676.m02147|LOC_Os10g25320|protein Initiation factor 2 subunit family e_value:0</td><td>0.069</td><td>(0.0291 0.4210)</td><td>0.0291</td><td>0.421</td></tr>
<tr><td>3200</td><td>WT012_N23_CLUSTER_18146</td><td>gi|50878317|gb|AAT85092.1| putative aminotransferase [Oryza sativa (japonica cultivar-group)] e_value:0</td><td>11682.m04638|LOC_Os05g48450|protein branched-chain amino acid aminotransferase, putative e_value:0</td><td>0.124</td><td>(0.0749 0.6036)</td><td>0.0749</td><td>0.6036</td></tr>
<tr><td>3201</td><td>WT008_A11_CLUSTER_8806</td><td>gi|50919397|ref|XP_470095.1| putative protein kinase [Oryza sativa (japonica cultivar-group)] >gnl|BL_ORD_ID|1588164 gi|28876011|gb|AAO60020.1| putative protein kinase [Oryza sativa (japonica cultivar-group)] e_value:1.27612e-173</td><td>11669.m06113|LOC_Os03g60150|protein putative protein kinase e_value:1.57395e-167</td><td>0.075</td><td>(0.0477 0.6392)</td><td>0.0477</td><td>0.6392</td></tr>
<tr><td>3202</td><td>WT004_D16_CLUSTER_27347</td><td>gi|34908900|ref|NP_915797.1| ethylene-responsive element binding factor 3 [Oryza sativa (japonica cultivar-group)] >gnl|BL_ORD_ID|1546948 gi|20160965|dbj|BAB89900.1| ethylene responsive element binding factor3 [Oryza sativa (japonica cultivar-group)] &</td><td>11667.m05797|LOC_Os01g58420|protein AP2 domain, putative e_value:3.03079e-103</td><td>0.108</td><td>(0.0966 0.8923)</td><td>0.0966</td><td>0.8923</td></tr>
<tr><td>3203</td><td>WT009_K24_CLUSTER_8737</td><td>gi|51535597|dbj|BAD37540.1| putative U5 snRNP-specific 40 kDa protein [Oryza sativa (japonica cultivar-group)] >gnl|BL_ORD_ID|242593 gi|51535047|dbj|BAD37418.1| putative U5 snRNP-specific 40 kDa protein [Oryza sativa (japonica cultivar-group)] e_value:</td><td>11680.m04363|LOC_Os06g44370|protein probable splicing factor [imported] - Arabidopsis thaliana e_value:0</td><td>0.043</td><td>(0.0250 0.5797)</td><td>0.025</td><td>0.5797</td></tr>
<tr><td>3204</td><td>WT005_F05_CLUSTER_10807</td><td>gi|50929401|ref|XP_474228.1| OSJNBa0084K01.12 [Oryza sativa (japonica cultivar-group)] >gnl|BL_ORD_ID|1594269 gi|38346072|emb|CAE04840.2| OSJNBa0084K01.12 [Oryza sativa (japonica cultivar-group)] e_value:0</td><td>11670.m05595|LOC_Os04g56720|protein Regulator of chromosome condensation (RCC1), putative e_value:0</td><td>0.206</td><td>(0.0950 0.4614)</td><td>0.095</td><td>0.4614</td></tr>
<tr><td>3205</td><td>WT002_L11_CLUSTER_33492</td><td>gi|50928873|ref|XP_473964.1| OSJNBb0060E08.6 [Oryza sativa (japonica cultivar-group)] >gnl|BL_ORD_ID|1592593 gi|39545835|emb|CAE04743.3| OSJNBb0060E08.6 [Oryza sativa (japonica cultivar-group)] e_value:0</td><td>11674.m00850|LOC_Os08g09100|protein RNA recognition motif. (a.k.a. RRM, RBD, or RNP domain), putative e_value:0</td><td>0.159</td><td>(0.1190 0.7485)</td><td>0.119</td><td>0.7485</td></tr>
<tr><td>3206</td><td>WT004_J15_CLUSTER_17401</td><td>gi|50898486|ref|XP_450031.1| putative KIF4 [Oryza sativa (japonica cultivar-group)] >gnl|BL_ORD_ID|1563224 gi|46390030|dbj|BAD16507.1| putative KIF4 [Oryza sativa (japonica cultivar-group)] e_value:0</td><td>11681.m00171|LOC_Os09g02650|protein Kinesin motor domain, putative e_value:0</td><td>0.094</td><td>(0.0477 0.5056)</td><td>0.0477</td><td>0.5056</td></tr>
<tr><td>3207</td><td>WT003_H16_CLUSTER_11157</td><td>gi|9293909|dbj|BAB01812.1| sugar transporter protein [Arabidopsis thaliana] >gnl|BL_ORD_ID|1090008 gi|15230212|ref|NP_188513.1| mannitol transporter, putative [Arabidopsis thaliana] e_value:1.22572e-162</td><td>11669.m00952|LOC_Os03g10090|protein sugar transporter, putative e_value:0</td><td>0.229</td><td>(0.0732 0.3190)</td><td>0.0732</td><td>0.319</td></tr>
<tr><td>3208</td><td>WT013_D09_CLUSTER_7162</td><td>gi|34909910|ref|NP_916302.1| P0665A11.15 [Oryza sativa (japonica cultivar-group)] >gnl|BL_ORD_ID|1551221 gi|14209573|dbj|BAB56069.1| F-box family protein-like [Oryza sativa (japonica cultivar-group)] e_value:5.46043e-176</td><td>11667.m05576|LOC_Os01g56390|protein F-box domain, putative e_value:1.29075e-177</td><td>0.188</td><td>(0.0898 0.4771)</td><td>0.0898</td><td>0.4771</td></tr>
<tr><td>3209</td><td>WT013_D03_CLUSTER_7</td><td>gi|50936843|ref|XP_477949.1| putative 1,4-beta-D xylan xylanohydrolase [Oryza sativa (japonica cultivar-group)] >gnl|BL_ORD_ID|1526445 gi|38175736|dbj|BAC57375.2| putative 1,4-beta-D xylan xylanohydrolase [Oryza sativa (japonica cultivar-</td><td>11673.m02570|LOC_Os07g27320|protein Carbohydrate binding domain, putative e_value:0</td><td>0.175</td><td>(0.0984 0.5638)</td><td>0.0984</td><td>0.5638</td></tr>
<tr><td>3210</td><td>WT005_C21_CLUSTER_33931</td><td>gi|50910741|ref|XP_466859.1| phosphate translocator-like [Oryza sativa (japonica cultivar-group)] >gnl|BL_ORD_ID|1580666 gi|48717036|dbj|BAD23725.1| phosphate translocator-like [Oryza sativa (japonica cultivar-group)] e_value:1.16458e-170</td><td>11668.m03988|LOC_Os02g41780|protein At2g30460 e_value:2.92168e-172</td><td>0.043</td><td>(0.0500 1.1591)</td><td>0.05</td><td>1.1591</td></tr>
<tr><td>3211</td><td>WT003_D02_CLUSTER_31025</td><td>gi|51091329|dbj|BAD36064.1| unknown protein [Oryza sativa (japonica cultivar-group)] e_value:0</td><td>11668.m05481|LOC_Os02g55590|protein expressed protein</td><td>0.224</td><td>(0.1505 0.6708)</td><td>0.1505</td><td>0.6708</td></tr>
<tr><td>3212</td><td>WT002_M15_CLUSTER_15457</td><td>gi|50933625|ref|XP_476340.1| unknown protein [Oryza sativa (japonica cultivar-group)] >gnl|BL_ORD_ID|1517661 gi|51963230|ref|XP_506126.1| PREDICTED B1026C12.9 gene product [Oryza sativa (japonica cultivar-group)] >gnl|BL_ORD_ID|1517661 gi|50508913|d</td><td>11673.m00008|LOC_Os07g01060|protein expressed protein e_value:0</td><td>0.352</td><td>(0.1544 0.4388)</td><td>0.1544</td><td>0.4388</td></tr>
<tr><td>3213</td><td>WT003_D19_CLUSTER_7282</td><td>gi|56202374|dbj|BAD73736.1| putative heme A:farnesyltransferase [Oryza sativa (japonica cultivar-group)] >gnl|BL_ORD_ID|552193 gi|56201999|dbj|BAD73506.1| putative heme A:farnesyltransferase [Oryza sativa (japonica cultivar-group)] e_value:0</td><td>11667.m03254|LOC_Os01g34390|protein protoheme IX farnesyltransferase, putative e_value:0</td><td>0.2</td><td>(0.1015 0.5070)</td><td>0.1015</td><td>0.507</td></tr>
<tr><td>3214</td><td>SET5_E11_CLUSTER_4455</td><td>gi|50919883|ref|XP_470302.1| hypothetical protein [Oryza sativa (japonica cultivar-group)] >gi|19071623|gb|AAL84290.1| hypothetical protein [Oryza sativa (japonica cultivar-group)] e_value:4.46619e-31</td><td>11669.m00701|LOC_Os03g07380|protein hypothetical protein e_value:2.81631e-33</td><td>0.835</td><td>(0.2348 0.2812)</td><td>0.2348</td><td>0.2812</td></tr>
<tr><td>3215</td><td>WT002_I24_CLUSTER_34856</td><td>gi|54291083|dbj|BAD61759.1| putative ubiquitin [Oryza sativa (japonica cultivar-group)] >gnl|BL_ORD_ID|265744 gi|54290883|dbj|BAD61543.1| putative ubiquitin [Oryza sativa (japonica cultivar-group)] e_value:0</td><td>11680.m02292|LOC_Os06g23290|protein Phosphatidylinositol 3- and 4-kinase, putative e_value:0</td><td>0.159</td><td>(0.0837 0.5258)</td><td>0.0837</td><td>0.5258</td></tr>
<tr><td>3216</td><td>SET3_M24_CLUSTER_33974</td><td>gi|53982658|gb|AAV25637.1| putative GDP dissociation inhibitor [Oryza sativa (japonica cultivar-group)] >gi|51854409|gb|AAU10789.1| putative GDP dissociation inhibitor [Oryza sativa (japonica cultivar-group)] e_value:0</td><td>11682.m02195|LOC_Os05g23860|protein GDP dissociation inhibitor protein - rice e_value:0</td><td>0.04</td><td>(0.0282 0.7053)</td><td>0.0282</td><td>0.7053</td></tr>
<tr><td>3217</td><td>WT005_O01_CLUSTER_33739</td><td>gi|50910563|ref|XP_466770.1| putative timing of CAB expression 1 [Oryza sativa (japonica cultivar-group)] >gnl|BL_ORD_ID|1578641 gi|47847675|dbj|BAD21456.1| putative timing of CAB expression 1 [Oryza sativa (japonica cultivar-group)] >gnl|BL_ORD_ID|</td><td>11668.m03903|LOC_Os02g40510|protein Similar to pseudo-response regulator APRR1 [imported] - Arabidopsis thaliana</td><td>0.245</td><td>(0.1229 0.5018)</td><td>0.1229</td><td>0.5018</td></tr>
<tr><td>3218</td><td>WT005_H10_CLUSTER_31753</td><td>gi|29367133|gb|AAO72438.1| phosphate transporter HvPT4 [Hordeum vulgare subsp. vulgare] >gnl|BL_ORD_ID|1107508 gi|29367131|gb|AAO72437.1| phosphate transporter HvPT4 [Hordeum vulgare subsp. vulgare] e_value:0</td><td>11676.m02666|LOC_Os10g30790|protein phosphate:H+ symporter e_value:0</td><td>0.215</td><td>(0.0777 0.3618)</td><td>0.0777</td><td>0.3618</td></tr>
<tr><td>3219</td><td>WT011_G22_CLUSTER_34305</td><td>gi|854626|emb|CAA60505.1| peptidylprolyl isomerase [Triticum aestivum] >gnl|BL_ORD_ID|1711071 gi|1076772|pir||S55383 peptidylprolyl isomerase (EC 5.2.1.8) - wheat >gnl|BL_ORD_ID|1711071 gi|3023751|sp|Q43207|FKB7_WHEAT 70 kDa peptidylprolyl isomerase</td><td>11674.m04163|LOC_Os08g41390|protein peptidyl-prolyl cis-trans isomerase, FKBP-type, putative e_value:0</td><td>0.123</td><td>(0.0601 0.4902)</td><td>0.0601</td><td>0.4902</td></tr>
<tr><td>3220</td><td>SET5_K21_CLUSTER_34305</td><td>gi|854626|emb|CAA60505.1| peptidylprolyl isomerase [Triticum aestivum] >gi|1076772|pir||S55383 peptidylprolyl isomerase (EC 5.2.1.8) - wheat >gi|3023751|sp|Q43207|FKB7_WHEAT 70 kDa peptidylprolyl isomerase (Peptidyl-prolyl cis-trans isomerase) (PPIa</td><td>11674.m04163|LOC_Os08g41390|protein peptidyl-prolyl cis-trans isomerase, FKBP-type, putative e_value:0</td><td>0.128</td><td>(0.0609 0.4742)</td><td>0.0609</td><td>0.4742</td></tr>
<tr><td>3221</td><td>WT006_H08_CLUSTER_32445</td><td>gi|57900293|dbj|BAD87126.1| putative receptor-like protein kinase 1 [Oryza sativa (japonica cultivar-group)] e_value:0</td><td>11667.m07329|LOC_Os01g72990|protein Protein kinase domain, putative e_value:0</td><td>0.136</td><td>(0.1180 0.8656)</td><td>0.118</td><td>0.8656</td></tr>
<tr><td>3222</td><td>SET2_G17_CLUSTER_25670</td><td>gi|50938371|ref|XP_478713.1| unknown protein [Oryza sativa (japonica cultivar-group)] >gi|50508675|dbj|BAD31160.1| unknown protein [Oryza sativa (japonica cultivar-group)] >gi|34393368|dbj|BAC83377.1| unknown protein [Oryza sativa (japonica cultivar</td><td>11673.m03624|LOC_Os07g37400|protein F-box domain, putative e_value:6.57308e-56</td><td>0.3</td><td>(0.1709 0.5707)</td><td>0.1709</td><td>0.5707</td></tr>
<tr><td>3223</td><td>WT007_F24_CLUSTER_8557</td><td>gi|50917697|ref|XP_469245.1| expressed protein (with alternative splicing) [Oryza sativa (japonica cultivar-group)] >gnl|BL_ORD_ID|1539700 gi|40538945|gb|AAR87202.1| expressed protein (with alternative splicing) [Oryza sativa (japonica cultivar-</td><td>11669.m04711|LOC_Os03g47070|protein hypothetical protein e_value:0</td><td>0.22</td><td>(0.1073 0.4878)</td><td>0.1073</td><td>0.4878</td></tr>
<tr><td>3224</td><td>WT004_C10_CLUSTER_33464</td><td>gi|50938449|ref|XP_478752.1| putative calcium-dependent protein kinase [Oryza sativa (japonica cultivar-group)] >gnl|BL_ORD_ID|1529480 gi|34393291|dbj|BAC83205.1| putative calcium-dependent protein kinase [Oryza sativa (japonica cultivar-</td><td>11673.m03702|LOC_Os07g38120|protein Protein kinase domain, putative e_value:0</td><td>0.103</td><td>(0.0426 0.4119)</td><td>0.0426</td><td>0.4119</td></tr>
<tr><td>3225</td><td>SET1_G18_CLUSTER_32544</td><td>gi|51091397|dbj|BAD36140.1| unknown protein [Oryza sativa (japonica cultivar-group)] >gi|51091347|dbj|BAD36082.1| unknown protein [Oryza sativa (japonica cultivar-group)] e_value:2.87657e-57</td><td>11681.m02450|LOC_Os09g26780|protein ZIM motif, putative e_value:5.37139e-59</td><td>0.407</td><td>(0.2699 0.6639)</td><td>0.2699</td><td>0.6639</td></tr>
<tr><td>3226</td><td>SET3_I07_CLUSTER_34514</td><td>gi|50929037|ref|XP_474046.1| OSJNBb0034I13.13 [Oryza sativa (japonica cultivar-group)] >gi|39545747|emb|CAD41728.3| OSJNBb0034I13.13 [Oryza sativa (japonica cultivar-group)] e_value:2.09312e-112</td><td>11670.m05355|LOC_Os04g54480|protein transcription factor STGA1 - soybean e_value:5.64025e-131</td><td>0.276</td><td>(0.2126 0.7703)</td><td>0.2126</td><td>0.7703</td></tr>
<tr><td>3227</td><td>SET2_M14_CLUSTER_4301</td><td>gi|21689669|gb|AAM67456.1| unknown protein [Arabidopsis thaliana] >gi|20268764|gb|AAM14085.1| unknown protein [Arabidopsis thaliana] >gi|4678927|emb|CAB41318.1| hypothetical protein [Arabidopsis thaliana] >gi|15231095|ref|NP_190766.1| expressed p</td><td>11681.m03059|LOC_Os09g33780|protein hypothetical protein e_value:1.74906e-177</td><td>0.203</td><td>(0.1258 0.6199)</td><td>0.1258</td><td>0.6199</td></tr>
<tr><td>3228</td><td>WT003_D05_CLUSTER_7175</td><td>gi|50910059|ref|XP_466518.1| glycosyl transferase-like protein [Oryza sativa (japonica cultivar-group)] >gnl|BL_ORD_ID|1577388 gi|46805291|dbj|BAD16823.1| glycosyl transferase-like protein [Oryza sativa (japonica cultivar-group)] e_value:0</td><td>11668.m03648|LOC_Os02g38140|protein Glycosyltransferase family 17 e_value:0</td><td>0.059</td><td>(0.0420 0.7183)</td><td>0.042</td><td>0.7183</td></tr>
<tr><td>3229</td><td>WT012_H17_CLUSTER_16797</td><td>gi|50946879|ref|XP_482967.1| putative ubiquitin-specific protease [Oryza sativa (japonica cultivar-group)] >gnl|BL_ORD_ID|1609195 gi|42407867|dbj|BAD09009.1| putative ubiquitin-specific protease [Oryza sativa (japonica cultivar-group)] e_value:0</td><td>11674.m03717|LOC_Os08g37350|protein Ubiquitin carboxyl-terminal hydrolase, putative e_value:0</td><td>0.391</td><td>(0.2079 0.5320)</td><td>0.2079</td><td>0.532</td></tr>
<tr><td>3230</td><td>WT005_N06_CLUSTER_15555</td><td>gi|50924232|ref|XP_472476.1| OSJNBa0028I23.19 [Oryza sativa (japonica cultivar-group)] >gnl|BL_ORD_ID|1591453 gi|39546212|emb|CAE04637.3| OSJNBa0028I23.19 [Oryza sativa (japonica cultivar-group)] e_value:0</td><td>11670.m03318|LOC_Os04g34440|protein expressed protein e_value:0</td><td>0.187</td><td>(0.0972 0.5211)</td><td>0.0972</td><td>0.5211</td></tr>
<tr><td>3231</td><td>WT012_A19_CLUSTER_32921</td><td>gi|50918923|ref|XP_469858.1| putative protein phosphatase [Oryza sativa (japonica cultivar-group)] >gnl|BL_ORD_ID|1586240 gi|14488375|gb|AAK63942.1| putative protein phosphatase [Oryza sativa (japonica cultivar-group)] e_value:0</td><td>11669.m05566|LOC_Os03g55320|protein putative protein phosphatase e_value:0</td><td>0.183</td><td>(0.0842 0.4596)</td><td>0.0842</td><td>0.4596</td></tr>
<tr><td>3232</td><td>WT008_G19_CLUSTER_26279</td><td>gi|51091708|dbj|BAD36509.1| putative SMA-9 class B [Oryza sativa (japonica cultivar-group)] e_value:0</td><td>11681.m01200|LOC_Os09g13610|protein expressed protein</td><td>0.22</td><td>(0.1487 0.6753)</td><td>0.1487</td><td>0.6753</td></tr>
<tr><td>3233</td><td>WT008_B01_CLUSTER_20423</td><td>gi|52076926|dbj|BAD45937.1| putative protein phosphatase type-2C [Oryza sativa (japonica cultivar-group)] e_value:7.66404e-157</td><td>11681.m03455|LOC_Os09g38550|protein protein phosphatase 2C homolog F11C18.60 - Arabidopsis thaliana e_value:1.63766e-158</td><td>0.236</td><td>(0.1483 0.6279)</td><td>0.1483</td><td>0.6279</td></tr>
<tr><td>3234</td><td>WT011_B08_CLUSTER_32876</td><td>gi|51091339|dbj|BAD36074.1| putative chaperonin 21 precursor [Oryza sativa (japonica cultivar-group)] e_value:2.40299e-94</td><td>11681.m02445|LOC_Os09g26730|protein chaperonin, 10 kDa, putative e_value:6.33504e-96</td><td>0.35</td><td>(0.1486 0.4248)</td><td>0.1486</td><td>0.4248</td></tr>
<tr><td>3235</td><td>WT010_D21_CLUSTER_33730</td><td>gi|4680211|gb|AAD27574.1| hypothetical protein [Sorghum bicolor] e_value:4.98996e-54</td><td>11687.m03847|LOC_Os11g41140|protein Zinc finger, C3HC4 type (RING finger), putative e_value:8.59991e-112</td><td>0.178</td><td>(0.0825 0.4622)</td><td>0.0825</td><td>0.4622</td></tr>
<tr><td>3236</td><td>WT005_G21_CLUSTER_7561</td><td>gi|62732984|gb|AAX95103.1| expressed protein [Oryza sativa (japonica cultivar-group)] >gnl|BL_ORD_ID|135159 gi|62732983|gb|AAX95102.1| expressed protein [Oryza sativa (japonica cultivar-group)] e_value:9.98429e-168</td><td>11687.m00573|LOC_Os11g06410|protein expressed protein e_value:1.41156e-164</td><td>0.304</td><td>(0.1710 0.5635)</td><td>0.171</td><td>0.5635</td></tr>
<tr><td>3237</td><td>WT013_B09_CLUSTER_3542</td><td>gi|31712095|gb|AAP68399.1| unknown protein [Oryza sativa (japonica cultivar-group)] >gnl|BL_ORD_ID|2050005 gi|50917257|ref|XP_469025.1| putative Rhomboid family protein (with alternative splicing) [Oryza sativa (japonica cultivar-group)] e_value:0</td><td>11669.m04469|LOC_Os03g44830|protein hypothetical protein e_value:0</td><td>0.174</td><td>(0.0713 0.4106)</td><td>0.0713</td><td>0.4106</td></tr>
<tr><td>3238</td><td>SET3_G24_CLUSTER_6156</td><td>gi|53749250|gb|AAU90110.1| putative LRR-containing F-box protein [Oryza sativa (japonica cultivar-group)] e_value:0</td><td>11682.m03542|LOC_Os05g37690|protein Leucine Rich Repeat, putative e_value:0</td><td>0.16</td><td>(0.0727 0.4538)</td><td>0.0727</td><td>0.4538</td></tr>
<tr><td>3239</td><td>WT002_A20_CLUSTER_32814</td><td>gi|51979016|ref|XP_507385.1| PREDICTED P0453G03.28 gene product [Oryza sativa (japonica cultivar-group)] >gnl|BL_ORD_ID|1529774 gi|51963502|ref|XP_506427.1| PREDICTED P0453G03.28 gene product [Oryza sativa (japonica cultivar-group)] >gnl|BL_ORD_ID|1</td><td>11673.m03825|LOC_Os07g39280|protein expressed protein e_value:0</td><td>0.089</td><td>(0.0376 0.4208)</td><td>0.0376</td><td>0.4208</td></tr>
<tr><td>3240</td><td>WT004_D19_CLUSTER_32879</td><td>gi|34896156|ref|NP_909422.1| kinase interacting protein 1 -like [Oryza sativa (japonica cultivar-group)] >gnl|BL_ORD_ID|1751351 gi|13486675|dbj|BAB39912.1| contains EST AU062670(C30068)~similar to Arabidopsis thaliana chromosome 2, At2g30500~unknown pr</td><td>11667.m00673|LOC_Os01g07370|protein hypothetical protein e_value:0</td><td>0.212</td><td>(0.1395 0.6581)</td><td>0.1395</td><td>0.6581</td></tr>
<tr><td>3241</td><td>WT003_E02_CLUSTER_33402</td><td>gi|22165075|gb|AAM93692.1| putative PKCq-interacting protein [Oryza sativa (japonica cultivar-group)] >gnl|BL_ORD_ID|2269829 gi|31432897|gb|AAP54473.1| putative PKCq-interacting protein [Oryza sativa (japonica cultivar-group)] >gnl|BL_ORD_ID|2269829</td><td>11676.m03192|LOC_Os10g35720|protein putative PKCq-interacting protein e_value:0</td><td>0.147</td><td>(0.0786 0.5341)</td><td>0.0786</td><td>0.5341</td></tr>
</table>

PDF Complete
Your complimentary use period has ended. Thank you for using PDF Complete.
Click Here to upgrade to Unlimited Pages and Expanded Features

<table>
<tr><td>3242</td><td>WT007_H11_CLUSTER_31882</td><td>gi|7437361|pir||T02094 glucose-6-phosphate isomerase (EC 5.3.1.9) 1 - maize >gnl|BL_ORD_ID|2333000 gi|596023|gb|AAA82734.1| glucose-6 phosphate isomerase >gnl|BL_ORD_ID|2333000 gi|1346073|sp|P49105|G6PI_MAIZE Glucose-6-phosphate isomerase, cytosolic</td><td>11680.m01436|LOC_Os06g14510|protein glucose-6-phosphate isomerase e_value:0</td><td>0.122</td><td>(0.0581 0.4774)</td><td>0.0581</td><td>0.4774</td></tr>
<tr><td>3243</td><td>WT008__C17_CLUSTER_25737</td><td>gi|50912779|ref|XP_467797.1| kelch repeat-containing F-box-like [Oryza sativa (japonica cultivar-group)] >gnl|BL_ORD_ID|1582911 gi|46390943|dbj|BAD16457.1| kelch repeat-containing F-box-like [Oryza sativa (japonica cultivar-group)] e_value:0</td><td>11668.m05013|LOC_Os02g51350|protein Kelch motif, putative e_value:0</td><td>0.112</td><td>(0.0747 0.6645)</td><td>0.0747</td><td>0.6645</td></tr>
<tr><td>3244</td><td>WT008_L06_CLUSTER_32946</td><td>gi|51963858|ref|XP_506712.1| PREDICTED OSJNBa0081C13.10 gene product [Oryza sativa (japonica cultivar-group)] >gnl|BL_ORD_ID|1566308 gi|50905117|ref|XP_464047.1| Sec13p [Oryza sativa (japonica cultivar-group)] >gnl|BL_ORD_ID|1566308 gi|42409112|dbj|</td><td>11668.m00365|LOC_Os02g04320|protein expressed protein e_value:4.87608e-176</td><td>0.037</td><td>(0.0292 0.7894)</td><td>0.0292</td><td>0.7894</td></tr>
<tr><td>3245</td><td>WT005_M10_CLUSTER_29319</td><td>gi|17104799|gb|AAL34288.1| putative 2-nitropropane dioxygenase [Arabidopsis thaliana] >gnl|BL_ORD_ID|1207413 gi|13878127|gb|AAK44141.1| putative 2-nitropropane dioxygenase [Arabidopsis thaliana] >gnl|BL_ORD_ID|1207413 gi|9759401|dbj|BAB09856.1| 2-ni</td><td>11674.m03771|LOC_Os08g37870|protein oxidoreductase, 2-nitropropane dioxygenase family e_value:2.91111e-140</td><td>0.186</td><td>(0.0819 0.4398)</td><td>0.0819</td><td>0.4398</td></tr>
<tr><td>3246</td><td>SET4_B18_CLUSTER_26113</td><td>gi|21928059|gb|AAM78058.1| AT5g61030/maf19_30 [Arabidopsis thaliana] >gi|9759450|dbj|BAB10366.1| unnamed protein product [Arabidopsis thaliana] >gi|16974556|gb|AAL31194.1| AT5g61030/maf19_30 [Arabidopsis thaliana] >gi|15239505|ref|NP_200911.1| RN</td><td>11686.m03093|LOC_Os12g31800|protein RNA-binding protein precursor e_value:1.12093e-80</td><td>0.269</td><td>(0.2084 0.7752)</td><td>0.2084</td><td>0.7752</td></tr>
<tr><td>3247</td><td>SET1_P05_CLUSTER_23158</td><td>gi|21389677|gb|AAM48037.1| ADP-ribosylation factor-like protein [Arabidopsis thaliana] >gi|18252989|gb|AAL62421.1| ADP-RIBOSYLATION FACTOR -like protein [Arabidopsis thaliana] >gi|22331720|ref|NP_190556.2| ADP-ribosylation factor, putative [Arabidop</td><td>11669.m00980|LOC_Os03g10370|protein ADP-ribosylation factor-like protein e_value:4.48744e-102</td><td>0.019</td><td>(0.0094 0.4872)</td><td>0.0094</td><td>0.4872</td></tr>
<tr><td>3248</td><td>WT004_D12_CLUSTER_29366</td><td>gi|52077166|dbj|BAD46211.1| unknown protein [Oryza sativa (japonica cultivar-group)] e_value:0</td><td>11681.m03551|LOC_Os09g39460|protein hypothetical protein e_value:1.01681e-155</td><td>0.302</td><td>(0.1525 0.5043)</td><td>0.1525</td><td>0.5043</td></tr>
<tr><td>3249</td><td>WT011_A14_CLUSTER_34726</td><td>gi|50912411|ref|XP_467613.1| putative potassium transporter HAK2p [Oryza sativa (japonica cultivar-group)] >gnl|BL_ORD_ID|1580536 gi|51964336|ref|XP_506953.1| PREDICTED OSJNBa0072H09.37 gene product [Oryza sativa (japonica cultivar-group)] >gnl|BL_O</td><td>11668.m04844|LOC_Os02g49760|protein potassium uptake protein e_value:0</td><td>0.156</td><td>(0.0810 0.5196)</td><td>0.081</td><td>0.5196</td></tr>
<tr><td>3250</td><td>SET3_M02_CLUSTER_34185</td><td>gi|51854398|gb|AAU10778.1| unknown protein [Oryza sativa (japonica cultivar-group)] >gi|46391110|gb|AAS90637.1| unknown protein [Oryza sativa (japonica cultivar-group)] e_value:9.63825e-52</td><td>11682.m00427|LOC_Os05g04860|protein Agenet domain, putative e_value:1.77771e-53</td><td>0.761</td><td>(0.4903 0.6439)</td><td>0.4903</td><td>0.6439</td></tr>
<tr><td>3251</td><td>SET6_O07_CLUSTER_27871</td><td>gi|34914298|ref|NP_918496.1| putative FtsH protease [Oryza sativa (japonica cultivar-group)] >gi|20521392|dbj|BAB91903.1| cell division protein ftsH (ftsH)-like [Oryza sativa (japonica cultivar-group)] e_value:0</td><td>11667.m03763|LOC_Os01g39260|protein ATP-dependent metalloprotease FtsH, putative e_value:0</td><td>0.11</td><td>(0.0611 0.5535)</td><td>0.0611</td><td>0.5535</td></tr>
<tr><td>3252</td><td>WT012_D07_CLUSTER_26715</td><td>gi|34908388|ref|NP_915541.1| P0529E05.20 [Oryza sativa (japonica cultivar-group)] >gnl|BL_ORD_ID|1557158 gi|18461207|dbj|BAB84404.1| C2 domain-containing protein-like [Oryza sativa (japonica cultivar-group)] e_value:2.0509e-146</td><td>11667.m06355|LOC_Os01g63470|protein C2 domain, putative e_value:5.83816e-148</td><td>0.27</td><td>(0.1098 0.4066)</td><td>0.1098</td><td>0.4066</td></tr>
<tr><td>3253</td><td>SET1_G11_CLUSTER_21368</td><td>gi|50929409|ref|XP_474232.1| OSJNBa0084K01.16 [Oryza sativa (japonica cultivar-group)] >gi|38346076|emb|CAE04844.2| OSJNBa0084K01.16 [Oryza sativa (japonica cultivar-group)] >gi|47777378|gb|AAT38012.1| unknown protein [Oryza sativa (japonica</td><td>11682.m02816|LOC_Os05g30410|protein PHF5-like protein e_value:7.52549e-66</td><td></td><td>(0.0000 -1.0000)</td><td>0</td><td>-1</td></tr>
<tr><td>3254</td><td>WT010_I17_CLUSTER_34257</td><td>gi|51964578|ref|XP_507073.1| PREDICTED OSJNBb0060J21.31 gene product [Oryza sativa (japonica cultivar-group)] >gnl|BL_ORD_ID|1587268 gi|50919091|ref|XP_469942.1| putative vesicle trafficking protein [Oryza sativa (japonica cultivar-group)] >gnl|BL_O</td><td>11669.m05843|LOC_Os03g57760|protein putative vesicle trafficking protein e_value:7.985e-118</td><td>0.047</td><td>(0.0278 0.5932)</td><td>0.0278</td><td>0.5932</td></tr>
<tr><td>3255</td><td>WT008_G04_CLUSTER_13914</td><td>gi|50911140|ref|XP_467003.1| putative vesicle-associated membrane protein-associated protein [Oryza sativa (japonica cultivar-group)] >gnl|BL_ORD_ID|1579952 gi|49388105|dbj|BAD25238.1| putative vesicle-associated membrane protein-associated protein [Or</td><td>11670.m04388|LOC_Os04g45190|protein 27k vesicle-associated membrane protein-associated protein - curled-leaved tobacco e_value:1.04652e-111</td><td>0.166</td><td>(0.0894 0.5405)</td><td>0.0894</td><td>0.5405</td></tr>
<tr><td>3256</td><td>WT006_P19_CLUSTER_30077</td><td>gi|34897868|ref|NP_910280.1| putative extra-large G-protein [Oryza sativa (japonica cultivar-group)] >gnl|BL_ORD_ID|1752557 gi|7363278|dbj|BAA93022.1| putative extra-large G-protein [Oryza sativa (japonica cultivar-group)] e_value:0</td><td>11680.m00120|LOC_Os06g02130|protein G-protein alpha subunit, putative e_value:0</td><td>0.197</td><td>(0.1191 0.6054)</td><td>0.1191</td><td>0.6054</td></tr>
<tr><td>3257</td><td>SET4_I07_CLUSTER_18192</td><td>gi|34904256|ref|NP_913475.1| P0452F10.16 [Oryza sativa (japonica cultivar-group)] >gi|17385739|dbj|BAB78679.1| MpV17 transgene -like [Oryza sativa (japonica cultivar-group)] e_value:1.99574e-106</td><td>11667.m01253|LOC_Os01g12800|protein Mpv17 / PMP22 family, putative e_value:3.3626e-108</td><td>0.152</td><td>(0.0688 0.4518)</td><td>0.0688</td><td>0.4518</td></tr>
<tr><td>3258</td><td>WT002_F04_CLUSTER_11982</td><td>gi|31415923|gb|AAP50944.1| unknown protein [Oryza sativa (japonica cultivar-group)] >gnl|BL_ORD_ID|2005428 gi|50918997|ref|XP_469895.1| unknown protein [Oryza sativa (japonica cultivar-group)] e_value:1.62488e-95</td><td>11669.m05606|LOC_Os03g55670|protein hypothetical protein e_value:3.45801e-97</td><td>0.158</td><td>(0.1158 0.7330)</td><td>0.1158</td><td>0.733</td></tr>
<tr><td>3259</td><td>WT002_G17_CLUSTER_34555</td><td>gi|38345685|emb|CAE01908.2| OSJNBb0070J16.1 [Oryza sativa (japonica cultivar-group)] >gnl|BL_ORD_ID|1522631 gi|38345327|emb|CAE54544.1| OSJNBa0004N05.24 [Oryza sativa (japonica cultivar-group)] >gnl|BL_ORD_ID|1522631 gi|50926426|ref|XP_473160.1| OSJ</td><td>11670.m04191|LOC_Os04g43360|protein Glycosyl hydrolase family 1 e_value:0</td><td>0.198</td><td>(0.2997 1.5173)</td><td>0.2997</td><td>1.5173</td></tr>
<tr><td>3260</td><td>WT009_A02_CLUSTER_34866</td><td>gi|34907958|ref|NP_915326.1| putative NADH dehydrogenase [Oryza sativa (japonica cultivar-group)] e_value:0</td><td>11667.m06119|LOC_Os01g61410|protein Pyridine nucleotide-disulphide oxidoreductase, putative e_value:0</td><td>0.137</td><td>(0.0526 0.3828)</td><td>0.0526</td><td>0.3828</td></tr>
<tr><td>3261</td><td>WT008_M14_CLUSTER_34878</td><td>gi|37991913|gb|AAR06359.1| putative metacaspase, having alternative splicing products [Oryza sativa (japonica cultivar-group)] >gnl|BL_ORD_ID|2263187 gi|50920853|ref|XP_470787.1| putative metacaspase, having alternative splicing products [Oryza sativ</td><td>11669.m02759|LOC_Os03g27120|protein putative metacaspase, having alternative splicing products e_value:9.68603e-137</td><td>0.877</td><td>(0.2497 0.2847)</td><td>0.2497</td><td>0.2847</td></tr>
<tr><td>3262</td><td>WT006_H21_CLUSTER_33572</td><td>gi|31432124|gb|AAP53794.1| putative heat shock protein [Oryza sativa (japonica cultivar-group)] >gnl|BL_ORD_ID|2011322 gi|37534410|ref|NP_921507.1| putative heat shock protein [Oryza sativa (japonica cultivar-group)] e_value:7.85293e-78</td><td>11676.m02413|LOC_Os10g28360|protein ARD/ARD' family e_value:2.19537e-79</td><td>0.23</td><td>(0.1499 0.6519)</td><td>0.1499</td><td>0.6519</td></tr>
<tr><td>3263</td><td>WT010_G03_CLUSTER_33491</td><td>gi|18057163|gb|AAL58186.1| putative endoxyloglucan transferase [Oryza sativa (japonica cultivar-group)] >gnl|BL_ORD_ID|2066635 gi|31433676|gb|AAP55160.1| putative endoxyloglucan transferase [Oryza sativa (japonica cultivar-group)] >gnl|BL_ORD_ID|206</td><td>11676.m03951|LOC_Os10g42670|protein putative endoxyloglucan transferase e_value:2.10461e-107</td><td>0.535</td><td>(0.2704 0.5050)</td><td>0.2704</td><td>0.505</td></tr>
<tr><td>3264</td><td>WT004_O02_CLUSTER_12412</td><td>gi|6684442|gb|AAF23590.1| succinic semialdehyde dehydrogenase [Arabidopsis thaliana] >gnl|BL_ORD_ID|1917607 gi|15810165|gb|AAL07226.1| putative succinic semialdehyde dehydrogenase gabD [Arabidopsis thaliana] >gnl|BL_ORD_ID|1917607 gi|15219379|ref|NP</td><td>11668.m00737|LOC_Os02g07760|protein succinic semialdehyde dehydrogenase gabD, putative e_value:0</td><td>0.14</td><td>(0.0544 0.3888)</td><td>0.0544</td><td>0.3888</td></tr>
<tr><td>3265</td><td>WT007_I20_CLUSTER_33342</td><td>gi|50904709|ref|XP_463843.1| putative 4-aminobutyrate aminotransferase [Oryza sativa (japonica cultivar-group)] >gnl|BL_ORD_ID|1565662 gi|41052763|dbj|BAD07632.1| putative 4-aminobutyrate aminotransferase [Oryza sativa (japonica cultivar-</td><td>11670.m05137|LOC_Os04g52450|protein aminotransferase, class III e_value:0</td><td>0.202</td><td>(0.1412 0.7005)</td><td>0.1412</td><td>0.7005</td></tr>
<tr><td>3266</td><td>WT009_H03_CLUSTER_32331</td><td>gi|50920711|ref|XP_470716.1| putative polypyrimidine tract-binding protein [Oryza sativa] >gnl|BL_ORD_ID|1511144 gi|18921326|gb|AAL82531.1| putative polypyrimidine tract-binding protein [Oryza sativa] e_value:0</td><td>11669.m02637|LOC_Os03g25980|protein putative polypyrimidine tract-binding protein e_value:0</td><td>0.135</td><td>(0.0669 0.4957)</td><td>0.0669</td><td>0.4957</td></tr>
<tr><td>3267</td><td>WT005_O16_CLUSTER_34979</td><td>gi|1902903|dbj|BAA19467.1| phospholipase D [Oryza sativa (japonica cultivar-group)] >gnl|BL_ORD_ID|724330 gi|7489548|pir||T03402 probable phospholipase D (EC 3.1.4.4) - rice >gnl|BL_ORD_ID|724330 gi|1020415|dbj|BAA11136.1| phospholipase D [Oryza sat</td><td>11667.m00718|LOC_Os01g07760|protein Similar to phospholipase d alpha 1 precursor (ec 3.1.4.4) (pld alpha 1) (choline phosphatase 1) (phosphatidylcholine-hydrolyzing</td><td>0.099</td><td>(0.0569 0.5754)</td><td>0.0569</td><td>0.5754</td></tr>
<tr><td>3268</td><td>WT005_F16_CLUSTER_34080</td><td>gi|50919223|ref|XP_470008.1| putative helicase [Oryza sativa (japonica cultivar-group)] >gnl|BL_ORD_ID|1587484 gi|41469394|gb|AAS07217.1| putative helicase [Oryza sativa (japonica cultivar-group)] e_value:0</td><td>11669.m05993|LOC_Os03g59050|protein putative helicase e_value:0</td><td>0.106</td><td>(0.0729 0.6868)</td><td>0.0729</td><td>0.6868</td></tr>
<tr><td>3269</td><td>WT003_D17_CLUSTER_34334</td><td>gi|50909441|ref|XP_466209.1| ranBPM-like [Oryza sativa (japonica cultivar-group)] >gnl|BL_ORD_ID|1576328 gi|46389862|dbj|BAD15463.1| ranBPM-like [Oryza sativa (japonica cultivar-group)] e_value:0</td><td>11668.m03324|LOC_Os02g35070|protein SPRY domain, putative e_value:0</td><td>0.19</td><td>(0.0942 0.4960)</td><td>0.0942</td><td>0.496</td></tr>
<tr><td>3270</td><td>WT002_J14_CLUSTER_24302</td><td>gi|34907198|ref|NP_914946.1| putative HAK2 (K+ transporter) [Oryza sativa (japonica cultivar-group)] >gnl|BL_ORD_ID|1540867 gi|15408801|dbj|BAB64197.1| putative HAK2 [Oryza sativa (japonica cultivar-group)] e_value:0</td><td>11667.m07110|LOC_Os01g70940|protein potassium uptake protein e_value:0</td><td>0.157</td><td>(0.0764 0.4876)</td><td>0.0764</td><td>0.4876</td></tr>
<tr><td>3271</td><td>SET1_M15_CLUSTER_11247</td><td>gi|52353602|gb|AAU44168.1| putative rab1 small GTP-binding protein [Oryza sativa (japonica cultivar-group)] e_value:2.73678e-100</td><td>11682.m00058|LOC_Os05g01490|protein GTP-binding protein RAB1 - garden petunia e_value:1.69724e-103</td><td>0.098</td><td>(0.0442 0.4503)</td><td>0.0442</td><td>0.4503</td></tr>
<tr><td>3272</td><td>WT008_L13_CLUSTER_16066</td><td>gi|56784451|dbj|BAD82544.1| unknown protein [Oryza sativa (japonica cultivar-group)] e_value:1.61826e-135</td><td>11667.m04801|LOC_Os01g49350|protein expressed protein e_value:4.73818e-137</td><td>0.212</td><td>(0.1025 0.4834)</td><td>0.1025</td><td>0.4834</td></tr>
<tr><td>3273</td><td>SET4_K13_CLUSTER_25227</td><td>gi|50919593|ref|XP_470157.1| putative hydrolase [Oryza sativa] >gi|28372678|gb|AAO39862.1| putative hydrolase [Oryza sativa (japonica cultivar-group)] >gi|18855052|gb|AAL79744.1| putative hydrolase [Oryza sativa] e_value:2.58229e-163</td><td>11669.m06250|LOC_Os03g61340|protein putative hydrolase e_value:7.54184e-165</td><td>0.217</td><td>(0.0961 0.4427)</td><td>0.0961</td><td>0.4427</td></tr>
<tr><td>3274</td><td>WT004_D24_CLUSTER_1265</td><td>gi|50938485|ref|XP_478770.1| putative insulin degrading enzyme [Oryza sativa (japonica cultivar-group)] >gnl|BL_ORD_ID|1529500 gi|33146782|dbj|BAC79700.1| putative insulin degrading enzyme [Oryza sativa (japonica cultivar-group)] e_value:0</td><td>11673.m03717|LOC_Os07g38270|protein Insulinase (Peptidase family M16), putative e_value:0</td><td>0.161</td><td>(0.0841 0.5212)</td><td>0.0841</td><td>0.5212</td></tr>
<tr><td>3275</td><td>WT002_E11_CLUSTER_31550</td><td>gi|31432075|gb|AAP53760.1| putative acylaminoacyl-peptidase like protein [Oryza sativa (japonica cultivar-group)] >gnl|BL_ORD_ID|2011205 gi|37534342|ref|NP_921473.1| putative acylaminoacyl-peptidase like protein [Oryza sativa (japonica cultivar-</td><td>11676.m02376|LOC_Os10g28020|protein prolyl oligopeptidase family, putative e_value:0</td><td>0.177</td><td>(0.1005 0.5668)</td><td>0.1005</td><td>0.5668</td></tr>
<tr><td>3276</td><td>SET6_O09_CLUSTER_31550</td><td>gi|31432075|gb|AAP53760.1| putative acylaminoacyl-peptidase like protein [Oryza sativa (japonica cultivar-group)] >gi|37534342|ref|NP_921473.1| putative acylaminoacyl-peptidase like protein [Oryza sativa (japonica cultivar-group)] e_value:0</td><td>11676.m02376|LOC_Os10g28020|protein prolyl oligopeptidase family, putative e_value:0</td><td>0.163</td><td>(0.0994 0.6100)</td><td>0.0994</td><td>0.61</td></tr>
<tr><td>3277</td><td>WT005_B20_CLUSTER_8814</td><td>gi|55770539|ref|XP_550260.1| unknown protein [Oryza sativa (japonica cultivar-group)] >gnl|BL_ORD_ID|1753627 gi|55297509|dbj|BAD68311.1| unknown protein [Oryza sativa (japonica cultivar-group)] e_value:7.1554e-143</td><td>11667.m00676|LOC_Os01g07400|protein expressed protein e_value:1.79002e-144</td><td>0.273</td><td>(0.2235 0.8187)</td><td>0.2235</td><td>0.8187</td></tr>
<tr><td>3278</td><td>WT003_M23_CLUSTER_12730</td><td>gi|34898706|ref|NP_910699.1| hypothetical protein [Oryza sativa (japonica cultivar-group)] >gnl|BL_ORD_ID|1517639 gi|11862972|dbj|BAB19353.1| putative Na+-dependent neutral amino acid transporter [Oryza sativa (japonica cultivar-group)] e_value:0</td><td>11680.m01631|LOC_Os06g16420|protein Transmembrane amino acid transporter protein e_value:0</td><td>0.112</td><td>(0.0605 0.5388)</td><td>0.0605</td><td>0.5388</td></tr>
<tr><td>3279</td><td>SET2_H22_CLUSTER_27288</td><td>gi|50909853|ref|XP_466415.1| putative Oxygen-evolving enhancer protein 3-2, chloroplast precursor (OEE3) [Oryza sativa (japonica cultivar-group)] >gi|50253294|dbj|BAD29563.1| putative Oxygen-evolving enhancer protein 3-2, chloroplast precursor (OEE3) [</td><td>11668.m03509|LOC_Os02g36850|protein Oxygen evolving enhancer protein 3 (PsbQ) e_value:1.4905e-92</td><td>0.25</td><td>(0.1153 0.4606)</td><td>0.1153</td><td>0.4606</td></tr>
<tr><td>3280</td><td>WT012_O14_CLUSTER_14246</td><td>gi|56202174|dbj|BAD73652.1| C-terminal zinc-finger-like [Oryza sativa (japonica cultivar-group)] e_value:0</td><td>11667.m04625|LOC_Os01g47740|protein Zinc finger, C3HC4 type (RING finger), putative e_value:0</td><td>0.27</td><td>(0.1230 0.4553)</td><td>0.123</td><td>0.4553</td></tr>
<tr><td>3281</td><td>WT009_O07_CLUSTER_33683</td><td>gi|38345244|emb|CAD41088.2| OSJNBb0011N17.5 [Oryza sativa (japonica cultivar-group)] >gnl|BL_ORD_ID|1522071 gi|50925215|ref|XP_472909.1| OSJNBb0011N17.5 [Oryza sativa (japonica cultivar-group)] e_value:0</td><td>11670.m03871|LOC_Os04g40490|protein Cellulase (glycosyl hydrolase family 5), putative e_value:0</td><td>0.212</td><td>(0.1183 0.5572)</td><td>0.1183</td><td>0.5572</td></tr>
<tr><td>3282</td><td>WT009_L23_CLUSTER_27545</td><td>gi|50900022|ref|XP_450799.1| putative cysteine proteinase [Oryza sativa (japonica cultivar-group)] >gnl|BL_ORD_ID|1564107 gi|49388888|dbj|BAD26098.1| putative cysteine proteinase [Oryza sativa (japonica cultivar-group)] >gnl|BL_ORD_ID|1564107 gi|493</td><td>11681.m03494|LOC_Os09g38920|protein cysteine proteinase (EC 3.4.22.-) - rice e_value:2.19487e-144</td><td>0.223</td><td>(0.1746 0.7818)</td><td>0.1746</td><td>0.7818</td></tr>
</table>

PDF Complete
Your complimentary use period has ended. Thank you for using PDF Complete.
Click Here to upgrade to Unlimited Pages and Expanded Features

3283	WT002_M18_CLUSTER_1920	gi|2695710|emb|CAA04245.1| 4-hydroxyphenylpyruvate dioxygenase [Hordeum vulgare subsp. vulgare] >gnl|BL_ORD_ID|1321662 gi|7459541|pir||T04471 probable 4-hydroxyphenylpyruvate dioxygenase (EC 1.13.11.27) - barley >gnl|BL_ORD_ID|1321662 gi|3334222|sp|	11668.m00671|LOC_Os02g07160|protein 4-hydroxyphenylpyruvate dioxygenase e_value:0	0.286	(0.0971 0.3396)	0.0971	0.3396
3284	WT013_F18_CLUSTER_7900	gi|42742279|gb|AAS45242.1| Bx2-like protein [Hordeum lechleri] e_value:6.2392e-147	11687.m02508|LOC_Os11g27730|protein Cytochrome P450 e_value:1.88931e-147	0.326	(0.3672 1.1257)	0.3672	1.1257
3285	WT008_N17_CLUSTER_28998	gi|50948929|ref|XP_493822.1| ESTs AU031435(E61570),AU078245(E61570) correspond to a region of the predicted gene.~hypothetical protein [Oryza sativa (japonica cultivar-group)] >gnl|BL_ORD_ID|1732303 gi|6063553|dbj|BAA85413.1| ESTs AU031435(E61570),AU07	11669.m00317|LOC_Os03g03830|protein EF hand, putative e_value:0	0.23	(0.1001 0.4354)	0.1001	0.4354
3286	WT006_K06_CLUSTER_28488	gi|38568022|emb|CAE05207.3| OSJNBa0070C17.14 [Oryza sativa (japonica cultivar-group)] >gnl|BL_ORD_ID|1538878 gi|50928677|ref|XP_473866.1| OSJNBa0070C17.14 [Oryza sativa (japonica cultivar-group)] e_value:0	11670.m05118|LOC_Os04g52290|protein Similar to T8K14.9 [imported] - Arabidopsis thaliana e_value:0	0.173	(0.1038 0.5990)	0.1038	0.599
3287	SET1_D11_CLUSTER_14119	gi|1806261|emb|CAA41453.1| DNA-binding protein; bZIP type [Petroselinum crispum] >gi|2842757|sp|Q99090|CPRF2_PETCR Light-inducible protein CPRF-2 e_value:4.37683e-45	11686.m04062|LOC_Os12g40920|protein Similar to probable DNA binding protein - rice e_value:6.53904e-77	0.436	(0.2910 0.6676)	0.291	0.6676
3288	WT004_G18_CLUSTER_33901	gi|32352182|dbj|BAC78584.1| hypothetical protein [Oryza sativa (japonica cultivar-group)] >gnl|BL_ORD_ID|1043265 gi|51535179|dbj|BAD38152.1| putative RNA-binding protein [Oryza sativa (japonica cultivar-group)] e_value:3.99309e-133	11680.m01232|LOC_Os06g12580|protein expressed protein e_value:1.22566e-134	0.324	(0.1809 0.5577)	0.1809	0.5577
3289	WT002_K17_CLUSTER_21119	gi|31430070|gb|AAP52034.1| putative lipase-like protein [Oryza sativa (japonica cultivar-group)] >gnl|BL_ORD_ID|2007985 gi|37530890|ref|NP_919747.1| putative lipase-like protein [Oryza sativa (japonica cultivar-group)] >gnl|BL_ORD_ID|2007985 gi|1864	11676.m00373|LOC_Os10g04620|protein lipase, putative e_value:0	0.1	(0.0607 0.6067)	0.0607	0.6067
3290	WT006_B06_CLUSTER_34545	gi|42573319|ref|NP_974756.1| 2-oxoisovalerate dehydrogenase, putative / 3-methyl-2-oxobutanoate dehydrogenase, putative / branched-chain alpha-keto acid dehydrogenase E1 alpha subunit, putative [Arabidopsis thaliana] e_value:4.99505e-172	11686.m00784|LOC_Os12g08260|protein branched-chain alpha keto-acid dehydrogenase E1 alpha subunit-like protein e_value:0	0.15	(0.0982 0.6553)	0.0982	0.6553
3291	WT003_N02_CLUSTER_13257	gi|50253316|dbj|BAD29585.1| putative RCH2 protein [Oryza sativa (japonica cultivar-group)] >gnl|BL_ORD_ID|551224 gi|50251711|dbj|BAD27632.1| putative RCH2 protein [Oryza sativa (japonica cultivar-group)] e_value:0	11670.m03706|LOC_Os04g39030|protein POT family, putative e_value:0	0.343	(0.1832 0.5340)	0.1832	0.534
3292	WT010_I09_CLUSTER_32246	gi|50872461|gb|AAT85061.1| nitrate transporter, putative [Oryza sativa (japonica cultivar-group)] e_value:0	11686.m04409|LOC_Os12g44110|protein probable peptide transporter T17F3.11 [imported] - Arabidopsis thaliana e_value:0	0.316	(0.1988 0.6301)	0.1988	0.6301
3293	WT008_O07_CLUSTER_34012	gi|52076944|dbj|BAD45955.1| putative SV2 related protein [Oryza sativa (japonica cultivar-group)] e_value:0	11670.m05083|LOC_Os04g51970|protein At3g13050/MGH6_16 e_value:1.66441e-177	0.092	(0.2690 2.9393)	0.269	2.9393
3294	WT007_M18_CLUSTER_678	gi|50915656|ref|XP_468292.1| decapping protein 2-like [Oryza sativa (japonica cultivar-group)] >gnl|BL_ORD_ID|1583882 gi|47497392|dbj|BAD19430.1| decapping protein 2-like [Oryza sativa (japonica cultivar-group)] >gnl|BL_ORD_ID|1583882 gi|47497342|db	11668.m05547|LOC_Os02g56210|protein Dcp2, box A domain, putative e_value:5.22289e-166	0.105	(0.0596 0.5660)	0.0596	0.566
3295	WT013_G22_CLUSTER_33439	gi|50947295|ref|XP_483175.1| putative WRKY DNA-binding protein [Oryza sativa (japonica cultivar-group)] >gnl|BL_ORD_ID|1609422 gi|58042751|gb|AAW63719.1| WRKY30 [Oryza sativa (japonica cultivar-group)] >gnl|BL_ORD_ID|1609422 gi|42407341|dbj|BAD08802	11674.m03899|LOC_Os08g38990|protein probable wrky transcription factor 2 (wrky dna-binding protein 2) e_value:0	0.277	(0.2976 1.0746)	0.2976	1.0746
3296	SET3_L03_CLUSTER_30265	gi|50929557|ref|XP_474306.1| OSJNBb0004A17.4 [Oryza sativa (japonica cultivar-group)] >gi|32488725|emb|CAE03602.1| OSJNBb0004A17.4 [Oryza sativa (japonica cultivar-group)] e_value:0	11670.m05694|LOC_Os04g57600|protein Zinc finger C-x8-C-x5-C-x3-H type (and similar), putative e_value:0	0.302	(0.1647 0.5464)	0.1647	0.5464
3297	SET2_N19_CLUSTER_27232	gi|51536380|dbj|BAD37573.1| kelch repeat containing F-box protein-like [Oryza sativa (japonica cultivar-group)] >gi|51535613|dbj|BAD37556.1| kelch repeat containing F-box protein-like [Oryza sativa (japonica cultivar-group)] e_value:1.43672e-175	11680.m04376|LOC_Os06g44500|protein Kelch motif, putative e_value:3.54775e-177	0.202	(0.1013 0.5020)	0.1013	0.502
3298	SET2_M09_CLUSTER_9645	gi|50904807|ref|XP_463892.1| putative protein kinase [Oryza sativa (japonica cultivar-group)] >gi|41053247|dbj|BAD07615.1| putative protein kinase [Oryza sativa (japonica cultivar-group)] e_value:9.15974e-125	11668.m00172|LOC_Os02g02600|protein Protein kinase domain, putative e_value:1.80074e-125	0.33	(0.2612 0.7918)	0.2612	0.7918
3299	SET2_L22_CLUSTER_12960	gi|62738052|pdb|1TX6|J Chain J, Trypsin:bbi Complex >gi|62738051|pdb|1TX6|I Chain I, Trypsin:bbi Complex e_value:3.70936e-55	11667.m00250|LOC_Os01g03380|protein Bowman-Birk serine protease inhibitor family, putative e_value:2.93509e-29	0.779	(0.5807 0.7457)	0.5807	0.7457
3300	WT013_O10_CLUSTER_7236	gi|18410809|ref|NP_567055.1| glycoside hydrolase family 28 protein / polygalacturonase (pectinase) family protein [Arabidopsis thaliana] e_value:4.888e-157	11681.m02889|LOC_Os09g31270|protein hypothetical protein e_value:0	0.124	(0.0627 0.5055)	0.0627	0.5055
3301	WT012_L03_CLUSTER_10800	gi|50919845|ref|XP_470283.1| hypothetical protein [Oryza sativa (japonica cultivar-group)] >gnl|BL_ORD_ID|1539357 gi|19071620|gb|AAL84287.1| hypothetical protein [Oryza sativa (japonica cultivar-group)] e_value:2.84032e-115	11669.m00681|LOC_Os03g07190|protein expressed protein e_value:6.8182e-117	0.205	(0.0948 0.4635)	0.0948	0.4635
3302	WT012_B23_CLUSTER_24398	gi|62734369|gb|AAX96478.1| hypothetical protein [Oryza sativa (japonica cultivar-group)] >gnl|BL_ORD_ID|2111863 gi|62701924|gb|AAX92997.1| hypothetical protein LOC_Os11g06740 [Oryza sativa (japonica cultivar-group)] e_value:6.68422e-98	11687.m00608|LOC_Os11g06740|protein hypothetical protein e_value:1.45181e-99	0.106	(0.0577 0.5441)	0.0577	0.5441
3303	WT012__C05_CLUSTER_20017	gi|15795155|dbj|BAB03143.1| ankyrin-like protein [Arabidopsis thaliana] e_value:7.86483e-51	11686.m03183|LOC_Os12g32630|protein F-box protein family, putative e_value:1.46279e-162	0.271	(0.1552 0.5716)	0.1552	0.5716
3304	WT010_O02_CLUSTER_9622	gi|50946791|ref|XP_482923.1| putative SEC23 [Oryza sativa (japonica cultivar-group)] >gnl|BL_ORD_ID|1609149 gi|42408205|dbj|BAD09341.1| putative SEC23 [Oryza sativa (japonica cultivar-group)] e_value:0	11681.m02651|LOC_Os09g28610|protein Sec23/Sec24 trunk domain, putative e_value:0	0.154	(0.0781 0.5065)	0.0781	0.5065
3305	WT010_D03_CLUSTER_24774	gi|31430636|gb|AAP52524.1| hypothetical protein [Oryza sativa (japonica cultivar-group)] >gnl|BL_ORD_ID|2009193 gi|37531870|ref|NP_920237.1| hypothetical protein [Oryza sativa (japonica cultivar-group)] >gnl|BL_ORD_ID|2009193 gi|22725983|gb|AAN04983	11676.m00992|LOC_Os10g11060|protein expressed protein e_value:1.42429e-84	0.418	(0.2163 0.5171)	0.2163	0.5171
3306	WT009_I09_CLUSTER_13981	gi|53680379|gb|AAU89392.1| glutamine-dependent asparagine synthetase [Triticum aestivum] e_value:0	11680.m01528|LOC_Os06g15420|protein asparagine synthase (glutamine-hydrolyzing), putative e_value:0		(0.1750 -1.0000)	0.175	-1
3307	WT009_D08_CLUSTER_6647	gi|34898064|ref|NP_910378.1| Similar to Arabidopsis thaliana DNA chromosome 4, BAC clone F25I24 (AL049525) [Oryza sativa (japonica cultivar-group)] >gnl|BL_ORD_ID|1752590 gi|5734630|dbj|BAA83361.1| unknown protein [Oryza sativa (japonica cultivar-group	11680.m00298|LOC_Os06g03840|protein expressed protein e_value:6.31705e-165	0.279	(0.2018 0.7235)	0.2018	0.7235
3308	WT009__C19_CLUSTER_27969	gi|52076557|dbj|BAD45460.1| putative pectinesterase [Oryza sativa (japonica cultivar-group)] e_value:0	11673.m04739|LOC_Os07g47830|protein Pectinesterase, putative e_value:1.48323e-180	0.61	(0.3427 0.5619)	0.3427	0.5619
3309	WT008_P17_CLUSTER_23212	gi|50932253|ref|XP_475654.1| 'unknown protein, contains zip zinc transporter protein, PF02535' [Oryza sativa (japonica cultivar-group)] >gnl|BL_ORD_ID|1597181 gi|50080291|gb|AAT69626.1| 'unknown protein, contains zip zinc transporter pro	11682.m00681|LOC_Os05g07210|protein ZIP zinc/iron transport family e_value:1.22175e-180	0.201	(0.0852 0.4234)	0.0852	0.4234
3310	WT008_I19_CLUSTER_15623	gi|33333542|gb|AAQ11887.1| knotted 7 [Hordeum vulgare] e_value:4.44812e-162	11680.m04310|LOC_Os06g43860|protein KNOX2 domain, putative e_value:1.73493e-146	0.073	(0.0538 0.7355)	0.0538	0.7355
3311	WT007_P06_CLUSTER_18737	gi|40538972|gb|AAR87229.1| putaive subtilisin-like proteinase [Oryza sativa (japonica cultivar-group)] >gnl|BL_ORD_ID|2058582 gi|50582703|gb|AAT78773.1| putative serine protease [Oryza sativa (japonica cultivar-group)] e_value:0	11669.m04043|LOC_Os03g40830|protein putative serine protease e_value:0	0.21	(0.0794 0.3781)	0.0794	0.3781
3312	WT006_I03_CLUSTER_16290	gi|50942001|ref|XP_480528.1| putative CCAAT box binding factor/transcription factor Hap2a [Oryza sativa (japonica cultivar-group)] >gnl|BL_ORD_ID|1602958 gi|38637434|dbj|BAD03691.1| putative CCAAT box binding factor/transcription factor Hap2a [Oryza sa	11686.m04168|LOC_Os12g41880|protein transcription factor, putative e_value:4.68817e-85	0.209	(0.1401 0.6707)	0.1401	0.6707
3313	WT005_L05_CLUSTER_8771	gi|50911885|ref|XP_467350.1| putative cytochrome P450 [Oryza sativa (japonica cultivar-group)] >gnl|BL_ORD_ID|1580476 gi|41053128|dbj|BAD08071.1| putative cytochrome P450 [Oryza sativa (japonica cultivar-group)] >gnl|BL_ORD_ID|1580476 gi|41052704|db	11681.m02629|LOC_Os09g28390|protein Cytochrome P450 e_value:1.87761e-149	0.548	(0.3716 0.6776)	0.3716	0.6776
3314	WT005__C12_CLUSTER_30565	gi|53749356|gb|AAU90215.1| putative uracil phosphoribosyltransferase [Oryza sativa (japonica cultivar-group)] e_value:2.02854e-133	11682.m03598|LOC_Os05g38170|protein uracil phosphoribosyltransferase, putative e_value:5.18132e-135	0.238	(0.0907 0.3817)	0.0907	0.3817
3315	WT004_H22_CLUSTER_12	gi|50934693|ref|XP_476874.1| putative tryptophan synthase alpha chain [Oryza sativa (japonica cultivar-group)] >gnl|BL_ORD_ID|1522234 gi|51963304|ref|XP_506200.1| PREDICTED OJ1506_G02.1 gene product [Oryza sativa (japonica cultivar-group)] >gnl|BL_O	11673.m00779|LOC_Os07g08430|protein tryptophan synthase, alpha subunit, putative e_value:7.97782e-146	0.208	(0.1044 0.5026)	0.1044	0.5026
3316	WT004_B07_CLUSTER_1716	gi|38344940|emb|CAE03041.2| OSJNBa0084A10.16 [Oryza sativa (japonica cultivar-group)] >gnl|BL_ORD_ID|1521471 gi|50924390|ref|XP_472555.1| OSJNBa0084A10.16 [Oryza sativa (japonica cultivar-group)] e_value:0	11670.m03409|LOC_Os04g35290|protein Thioredoxin, putative e_value:0	0.14	(0.0633 0.4536)	0.0633	0.4536
3317	WT003_K04_CLUSTER_21133	gi|55417864|ref|XP_467517.2| putative chloroplast nucleoid DNA-binding protein cnd41 [Oryza sativa (japonica cultivar-group)] >gnl|BL_ORD_ID|1580615 gi|50912217|ref|XP_467516.1| putative chloroplast nucleoid DNA-binding protein cnd41 [Oryza sativa (jap	11680.m01950|LOC_Os06g20040|protein Eukaryotic aspartyl protease e_value:0	0.495	(0.2141 0.4328)	0.2141	0.4328
3318	WT002_L05_CLUSTER_15180	gi|50917329|ref|XP_469061.1| putative cationic amino acid transporter [Oryza sativa (japonica cultivar-group)] >gnl|BL_ORD_ID|1585063 gi|50582770|gb|AAT78840.1| putative amino acid permease [Oryza sativa (japonica cultivar-group)] >gnl|BL_ORD_ID|158	11686.m04275|LOC_Os12g42850|protein Amino acid permease e_value:0	0.232	(0.1530 0.6595)	0.153	0.6595
3319	WT002_B08_CLUSTER_13022	gi|55296497|dbj|BAD68693.1| WD-40 repeat family protein-like [Oryza sativa (japonica cultivar-group)] e_value:0	11667.m04481|LOC_Os01g46510|protein expressed protein	0.109	(0.0504 0.4607)	0.0504	0.4607
3320	SET3_P01_CLUSTER_29007	gi|22122921|gb|AAM92304.1| hypothetical protein [Oryza sativa (japonica cultivar-group)] e_value:0	11676.m03411|LOC_Os10g37730|protein hypothetical protein e_value:0	0.097	(0.0516 0.5304)	0.0516	0.5304
3321	SET3_O24_CLUSTER_149	gi|50940801|ref|XP_479928.1| putative zinc-finger protein Lsd1 [Oryza sativa (japonica cultivar-group)] >gi|29467531|dbj|BAC66720.1| putative zinc-finger protein Lsd1 [Oryza sativa (japonica cultivar-group)] e_value:1.7638e-39	11674.m00274|LOC_Os08g03610|protein LSD1 zinc finger, putative e_value:1.53326e-41	0.272	(0.2363 0.8703)	0.2363	0.8703
3322	WT012_E14_CLUSTER_15557	gi|55733792|gb|AAV59299.1| unknown protein [Oryza sativa (japonica cultivar-group)] >gnl|BL_ORD_ID|1741242 gi|50932357|ref|XP_475706.1| unknown protein [Oryza sativa (japonica cultivar-group)] e_value:4.15837e-177	11682.m04168|LOC_Os05g43790|protein expressed protein e_value:1.36093e-175	0.085	(0.0526 0.6160)	0.0526	0.616
3323	WT009_P06_CLUSTER_19778	gi|50946305|ref|XP_482680.1| putative dentin sialophosphoprotein precursor [Oryza sativa (japonica cultivar-group)] >gnl|BL_ORD_ID|1608494 gi|42409465|dbj|BAD09822.1| putative dentin sialophosphoprotein precursor [Oryza sativa (japonica cultivar-	11674.m03444|LOC_Os08g34760|protein hypothetical protein e_value:0	0.534	(0.2992 0.5600)	0.2992	0.56
3324	SET3_N05_CLUSTER_22446	gi|20455047|sp|Q42777|MCCA_SOYBN Methylcrotonoyl-CoA carboxylase alpha chain, mitochondrial precursor (3-Methylcrotonyl-CoA carboxylase 1) (MCCase alpha subunit) (3-methylcrotonyl-CoA:carbon dioxide ligase alpha subunit) e_value:0	11686.m04098|LOC_Os12g41250|protein methylcrotonyl-coa carboxylase alpha chain, mitochondrial precursor(ec 6.4.1.4) (3-methylcrotonyl-coa carboxylase 1) (mccase alphasubunit) (3-methylcrotonyl-coa:carbon dioxide ligase alpha subunit)	0.177	(0.0757 0.4278)	0.0757	0.4278
3325	WT009_F15_CLUSTER_28689	No hits found	11687.m03894|LOC_Os11g41600|protein expressed protein e_value:2.01237e-36	0.346	(0.2534 0.7325)	0.2534	0.7325

PDF Complete
Your complimentary use period has ended. Thank you for using PDF Complete.
Click Here to upgrade to Unlimited Pages and Expanded Features

3326	SET2_N05_CLUSTER_5587	gi|34896102|ref|NP_909395.1| B1189A09.36 [Oryza sativa (japonica cultivar-group)] e_value:2.02786e-123	11667.m00644|LOC_Os01g07100|protein hypothetical protein e_value:3.78119e-137	0.273	(0.1398 0.5129)	0.1398	0.5129
3327	WT006_C11_CLUSTER_33698	gi|51090410|dbj|BAD35332.1| putative transcriptional regulator [Oryza sativa (japonica cultivar-group)] e_value:0	11680.m02651|LOC_Os06g27800|protein expressed protein	0.136	(0.0835 0.6153)	0.0835	0.6153
3328	SET5_P13_CLUSTER_14722	gi|50911651|ref|XP_467233.1| unknown protein [Oryza sativa (japonica cultivar-group)] >gi|41052812|dbj|BAD07680.1| unknown protein [Oryza sativa (japonica cultivar-group)] e_value:9.80829e-14	11668.m04497|LOC_Os02g46480|protein hypothetical protein e_value:1.70267e-15	0.291	(0.2204 0.7585)	0.2204	0.7585
3329	SET5_G19_CLUSTER_31835	gi|11995457|gb|AAG43027.1| aldehyde dehydrogenase [Oryza sativa] >gi|51091407|dbj|BAD36150.1| aldehyde dehydrogenase [Oryza sativa (japonica cultivar-group)] e_value:0	11681.m02464|LOC_Os09g26880|protein aldehyde dehydrogenase e_value:0	0.162	(0.0637 0.3918)	0.0637	0.3918
3330	WT012_E03_CLUSTER_26901	gi|50929791|ref|XP_474423.1| OSJNBa0088H09.19 [Oryza sativa (japonica cultivar-group)] >gnl|BL_ORD_ID|1594610 gi|38345820|emb|CAD41923.2| OSJNBa0070M12.1 [Oryza sativa (japonica cultivar-group)] >gnl|BL_ORD_ID|1594610 gi|32488718|emb|CAE03461.1| OSJ	11670.m05835|LOC_Os04g58890|protein expressed protein e_value:3.17955e-97	0.34	(0.1698 0.4993)	0.1698	0.4993
3331	WT003_C09_CLUSTER_16471	gi|19021|emb|CAA28804.1| aleurain [Hordeum vulgare] >gnl|BL_ORD_ID|1653185 gi|113603|sp|P05167|ALEU_HORVU Thiol protease aleurain precursor e_value:0	11681.m02480|LOC_Os09g27030|protein oryzain gamma chain precursor (ec 3.4.22.-) e_value:0	0.178	(0.0856 0.4794)	0.0856	0.4794
3332	WT005_L02_CLUSTER_31730	gi|34912804|ref|NP_917749.1| P0501G01.11 [Oryza sativa (japonica cultivar-group)] >gnl|BL_ORD_ID|1543980 gi|12313677|dbj|BAB21082.1| unknown protein [Oryza sativa (japonica cultivar-group)] e_value:4.64048e-130	11667.m04209|LOC_Os01g43420|protein expressed protein e_value:9.48285e-132	0.226	(0.0964 0.4258)	0.0964	0.4258
3333	WT005_G07_CLUSTER_33475	gi|50355722|gb|AAT75247.1| expressed protein [Oryza sativa (japonica cultivar-group)] e_value:0	11669.m05809|LOC_Os03g57460|protein expressed protein	0.189	(0.0716 0.3790)	0.0716	0.379
3334	WT005_M07_CLUSTER_34190	gi|50904603|ref|XP_463790.1| unknown protein [Oryza sativa (japonica cultivar-group)] >gnl|BL_ORD_ID|1566059 gi|51963778|ref|XP_506672.1| PREDICTED B1370C05.31 gene product [Oryza sativa (japonica cultivar-group)] >gnl|BL_ORD_ID|1566059 gi|41053238|	11668.m00026|LOC_Os02g01240|protein hypothetical protein e_value:2.94999e-83	0.084	(0.0702 0.8401)	0.0702	0.8401
3335	SET2_F23_CLUSTER_34093	gi|3550485|emb|CAA11894.1| cp33Hv [Hordeum vulgare subsp. vulgare] >gi|7446339|pir||T05730 probable RNA-binding protein cp33 precursor - barley e_value:7.2693e-176	11673.m00565|LOC_Os07g06450|protein RNA recognition motif. (a.k.a. RRM, RBD, or RNP domain), putative e_value:2.59376e-	0.28	(0.1890 0.6756)	0.189	0.6756
3336	WT011_C20_CLUSTER_30074	gi|50937015|ref|XP_478035.1| plastid-lipid associated protein PAP/fibrillin family-like protein [Oryza sativa (japonica cultivar-group)] e_value:9.63495e-152	11673.m02721|LOC_Os07g28790|protein PAP_fibrillin, putative e_value:5.28271e-163	0.351	(0.1676 0.4781)	0.1676	0.4781
3337	WT003_I05_CLUSTER_25320	gi|50911413|ref|XP_467114.1| amino acid permease-like [Oryza sativa (japonica cultivar-group)] >gnl|BL_ORD_ID|1580209 gi|49388207|dbj|BAD25330.1| amino acid permease-like [Oryza sativa (japonica cultivar-group)] >gnl|BL_ORD_ID|1580209 gi|49388552|db	11668.m04213|LOC_Os02g43860|protein Amino acid permease e_value:0	0.147	(0.0615 0.4185)	0.0615	0.4185
3338	WT008_H09_CLUSTER_12245	gi|54291827|gb|AAV32195.1| unknown protein [Oryza sativa (japonica cultivar-group)] e_value:4.3128e-123	11682.m00526|LOC_Os05g05790|protein Double-stranded RNA binding motif, putative e_value:0	0.316	(0.1794 0.5684)	0.1794	0.5684
3339	WT002_G06_CLUSTER_31854	gi|51535412|dbj|BAD37282.1| putative salt tolerance protein 5 [Oryza sativa (japonica cultivar-group)] e_value:2.76109e-130	11680.m01226|LOC_Os06g12530|protein salt tolerance protein 5 e_value:7.86491e-132	0.233	(0.1083 0.4640)	0.1083	0.464
3340	WT005_P14_CLUSTER_26910	gi|50912151|ref|XP_467483.1| unknown protein [Oryza sativa (japonica cultivar-group)] >gnl|BL_ORD_ID|1608634 gi|51964310|ref|XP_506940.1| PREDICTED OJ2056_H01.18 gene product [Oryza sativa (japonica cultivar-group)] >gnl|BL_ORD_ID|1608634 gi|4573586	11668.m04722|LOC_Os02g48630|protein expressed protein e_value:0	0.119	(0.0516 0.4349)	0.0516	0.4349
3341	WT009_N22_CLUSTER_17413	gi|53792249|dbj|BAD52882.1| 30S ribosomal protein S1-like [Oryza sativa (japonica cultivar-group)] e_value:2.45641e-165	11667.m03911|LOC_Os01g40640|protein S1 RNA binding domain, putative e_value:8.54537e-167	0.333	(0.1859 0.5589)	0.1859	0.5589
3342	WT007_E06_CLUSTER_33629	gi|50915540|ref|XP_468234.1| hypothetical protein [Oryza sativa (japonica cultivar-group)] >gnl|BL_ORD_ID|1583355 gi|47497144|dbj|BAD19193.1| hypothetical protein [Oryza sativa (japonica cultivar-group)] >gnl|BL_ORD_ID|1583355	11668.m04688|LOC_Os02g48300|protein F-box domain, putative e_value:1.40771e-106	0.324	(0.4013 1.2393)	0.4013	1.2393
3343	WT002_G18_CLUSTER_6838	gi|56682917|gb|AAW21813.1| cysteine protease [Triticum aestivum] e_value:0	11668.m02542|LOC_Os02g27030|protein Papain family cysteine protease, putative e_value:0	0.188	(0.0943 0.5019)	0.0943	0.5019
3344	SET6_G07_CLUSTER_6838	gi|56682917|gb|AAW21813.1| cysteine protease [Triticum aestivum] e_value:0	11668.m02542|LOC_Os02g27030|protein Papain family cysteine protease, putative e_value:0	0.191	(0.0923 0.4840)	0.0923	0.484
3345	SET5_O06_CLUSTER_32579	gi|51535770|dbj|BAD37809.1| putative system A transporter isoform 2 [Oryza sativa (japonica cultivar-group)] e_value:0	11680.m04190|LOC_Os06g42720|protein Transmembrane amino acid transporter protein e_value:0	0.091	(0.0538 0.5927)	0.0538	0.5927
3346	WT004_A08_CLUSTER_11946	gi|50939993|ref|XP_479524.1| putative Serine/threonine Kinase [Oryza sativa (japonica cultivar-group)] >gnl|BL_ORD_ID|1534243 gi|33146431|dbj|BAC79539.1| putative Serine/threonine Kinase [Oryza sativa (japonica cultivar-group)] e_value:0	11673.m04768|LOC_Os07g48100|protein Protein kinase domain, putative e_value:0	0.11	(0.0887 0.8027)	0.0887	0.8027
3347	WT003_G01_CLUSTER_34413	gi|22135845|gb|AAM91108.1| At2g47250/T8I13.9 [Arabidopsis thaliana] >gnl|BL_ORD_ID|1122450 gi|28416499|gb|AAO42780.1| At2g47250/T8I13.9 [Arabidopsis thaliana] >gnl|BL_ORD_ID|1122450 gi|2275203|gb|AAB63825.1| putative pre-mRNA splicing factor RNA	11669.m02032|LOC_Os03g19960|protein pre-mrna splicing factor atp-dependent rna helicase, putative e_value:0	0.046	(0.0227 0.4940)	0.0227	0.494
3348	SET6_E16_CLUSTER_30886	gi|50929237|ref|XP_474146.1| OSJNBb0059K02.23 [Oryza sativa (japonica cultivar-group)] >gi|38345913|emb|CAE04513.2| OSJNBb0059K02.23 [Oryza sativa (japonica cultivar-group)] >gi|38345895|emb|CAE03539.2| OSJNBa0060D06.5 [Oryza sativa (japonica cultiv	11680.m00447|LOC_Os06g05160|protein probable sulfate transporter 3.4 e_value:0	0.319	(0.3340 1.0486)	0.334	1.0486
3349	WT003_K06_CLUSTER_33002	gi|50912529|ref|XP_467672.1| putative dihydropyrimidine dehydrogenase [Oryza sativa (japonica cultivar-group)] >gnl|BL_ORD_ID|1582782 gi|46390439|dbj|BAD15901.1| putative dihydropyrimidine dehydrogenase [Oryza sativa (japonica cultivar-group)] e_value:	11668.m04904|LOC_Os02g50350|protein Dihydroorotate dehydrogenase, putative e_value:0	0.086	(0.0426 0.4946)	0.0426	0.4946
3350	WT003_O22_CLUSTER_8519	gi|62733608|gb|AAX95725.1| Protease inhibitor/seed storage/LTP family, putative [Oryza sativa (japonica cultivar-group)] >gnl|BL_ORD_ID|2113807 gi|31432947|gb|AAP54518.1| putative lipid transfer protein [Oryza sativa (japonica cultivar-group)] >gnl|	11676.m03242|LOC_Os10g36170|protein putative lipid transfer protein e_value:2.03521e-29	1.226	(0.3478 0.2836)	0.3478	0.2836
3351	SET5_K15_CLUSTER_31766	gi|11544696|emb|CAC17628.1| putative phospholipid hydroperoxide glutathione peroxidase [Oryza sativa (japonica cultivar-group)] e_value:1.86827e-87	11669.m02466|LOC_Os03g24380|protein phospholipid hydroperoxide glutathione peroxidase, putative	0.077	(0.0471 0.6103)	0.0471	0.6103
3352	WT012_K05_CLUSTER_22275	gi|51535686|dbj|BAD37705.1| putative cytosine deaminase [Oryza sativa (japonica cultivar-group)] e_value:0	11680.m02818|LOC_Os06g29430|protein At1g68720 e_value:0	0.402	(0.2208 0.5491)	0.2208	0.5491
3353	WT003_F20_CLUSTER_22615	gi|51535066|dbj|BAD37655.1| unknown protein [Oryza sativa (japonica cultivar-group)] e_value:3.8186e-82	11680.m02748|LOC_Os06g28740|protein expressed protein e_value:4.10747e-84	0.092	(0.0520 0.5668)	0.052	0.5668
3354	SET5_I01_CLUSTER_26763	gi|50940399|ref|XP_479727.1| putative survival motor neuron domain containing 1; splicing factor 30, survival of motor neuron-related [Oryza sativa (japonica cultivar-group)] >gi|42408381|dbj|BAD09532.1| putative survival motor neuron domain containing	11674.m00091|LOC_Os08g01840|protein expressed protein e_value:1.753e-146	0.129	(0.0644 0.4991)	0.0644	0.4991
3355	WT003_F01_CLUSTER_32468	gi|6562306|emb|CAB62604.1| diacylglycerol kinase (ATDGK1) [Arabidopsis thaliana] >gnl|BL_ORD_ID|2378098 gi|10176726|dbj|BAB09956.1| diacylglycerol kinase ATDGK1 homolog [Arabidopsis thaliana] >gnl|BL_ORD_ID|2378098	11686.m03830|LOC_Os12g38780|protein diacylglycerol kinase 1 (ec 2.7.1.107) (diglyceride kinase 1)(dgk 1) (dag kinase 1)	0.168	(0.0843 0.5018)	0.0843	0.5018
3356	WT010_H23_CLUSTER_10491	gi|38345749|emb|CAE03477.2| OSJNBa0065O17.2 [Oryza sativa (japonica cultivar-group)] >gnl|BL_ORD_ID|1522636 gi|38345597|emb|CAD41650.2| OSJNBb0012E24.15 [Oryza sativa (japonica cultivar-group)] >gnl|BL_ORD_ID|1522636 gi|50927875|ref|XP_473465.1| OSJ	11670.m04587|LOC_Os04g47020|protein genetic modifier e_value:4.79748e-146	0.252	(0.1306 0.5178)	0.1306	0.5178
3357	SET3_P13_CLUSTER_31192	gi|54287592|gb|AAV31336.1| putative UDP-sulfoquinovose synthase [Oryza sativa (japonica cultivar-group)] e_value:0	11682.m02998|LOC_Os05g32140|protein udp-sulfoquinovose synthase, chloroplast precursor (ec 3.13.1.1)(sulfite:udp-glucose sulfotransferase) (sulfolipid biosynthesisprotein) e_value:0	0.084	(0.0525 0.6212)	0.0525	0.6212
3358	WT008_F03_CLUSTER_34930	gi|51538213|gb|AAU06081.1| auxin amidohydrolase [Triticum aestivum] e_value:0	11667.m03628|LOC_Os01g37960|protein amidohydrolase	0.128	(0.0606 0.4724)	0.0606	0.4724
3359	WT003_K20_CLUSTER_33621	gi|311764|emb|CAA80493.1| (1,3;1,4) beta glucanase [Triticum aestivum] >gnl|BL_ORD_ID|2367137 gi|486849|pir||S36235 licheninase (EC 3.2.1.73) precursor - wheat e_value:0	11682.m02891|LOC_Os05g31140|protein Glycosyl hydrolases family 17 e_value:2.58077e-165	0.254	(0.1049 0.4132)	0.1049	0.4132
3360	SET5_A23_CLUSTER_22187	gi|50934809|ref|XP_476932.1| transducin / WD-40 repeat protein-like [Oryza sativa (japonica cultivar-group)] >gi|34394605|dbj|BAC83907.1| transducin / WD-40 repeat protein-like [Oryza sativa (japonica cultivar-group)] e_value:1.22226e-179	11673.m00842|LOC_Os07g09000|protein expressed protein e_value:1.28464e-176	0.275	(0.1055 0.3841)	0.1055	0.3841
3361	SET5_D03_CLUSTER_25082	gi|50940445|ref|XP_479750.1| putative CRK1 protein [Oryza sativa (japonica cultivar-group)] >gi|42408357|dbj|BAD09509.1| putative CRK1 protein [Oryza sativa (japonica cultivar-group)] e_value:0	11674.m00112|LOC_Os08g02050|protein Protein kinase domain, putative e_value:0	0.129	(0.0705 0.5486)	0.0705	0.5486
3362	WT010_J07_CLUSTER_32431	gi|50251779|dbj|BAD27711.1| putative Citrate synthase, glyoxysomal precursor (GCS) [Oryza sativa (japonica cultivar-group)] e_value:0	11668.m01280|LOC_Os02g13840|protein citrate synthase I	0.13	(0.0622 0.4772)	0.0622	0.4772
3363	WT008_A17_CLUSTER_7895	gi|50912649|ref|XP_467732.1| putative DegP2 protease [Oryza sativa (japonica cultivar-group)] >gnl|BL_ORD_ID|1582844 gi|46390287|dbj|BAD15737.1| putative DegP2 protease [Oryza sativa (japonica cultivar-group)] e_value:0	11668.m04960|LOC_Os02g50880|protein Trypsin, putative e_value:0	0.146	(0.0600 0.4101)	0.06	0.4101
3364	SET1_K02_CLUSTER_7895	gi|50912649|ref|XP_467732.1| putative DegP2 protease [Oryza sativa (japonica cultivar-group)] >gi|46390287|dbj|BAD15737.1| putative DegP2 protease [Oryza sativa (japonica cultivar-group)] e_value:0	11668.m04960|LOC_Os02g50880|protein Trypsin, putative e_value:0	0.142	(0.0590 0.4147)	0.059	0.4147
3365	SET1_D10_CLUSTER_33078	gi|54290425|dbj|BAD61295.1| unknown protein [Oryza sativa (japonica cultivar-group)] e_value:7.9113e-117	11667.m04169|LOC_Os01g43070|protein expressed protein e_value:2.03011e-118	0.192	(0.1612 0.8397)	0.1612	0.8397
3366	WT006_K03_CLUSTER_8495	gi|34907956|ref|NP_915325.1| putative ABC transporter [Oryza sativa (japonica cultivar-group)] >gnl|BL_ORD_ID|1555579 gi|20160641|dbj|BAB89586.1| ABC transporter subunit-like [Oryza sativa (japonica cultivar-group)] >gnl|BL_ORD_ID|1555579 gi|1562406	11667.m06118|LOC_Os01g61400|protein FeS assembly protein SufB e_value:0	0.12	(0.0573 0.4762)	0.0573	0.4762
3367	SET4_N06_CLUSTER_5083	gi|50920707|ref|XP_470714.1| putative ribonucleoprotein [Oryza sativa] >gi|18921322|gb|AAL82527.1| putative ribonucleoprotein [Oryza sativa] e_value:7.9834e-91	11669.m02634|LOC_Os03g25960|protein putative ribonucleoprotein e_value:2.10767e-92	0.352	(0.1804 0.5123)	0.1804	0.5123
3368	WT010_P01_CLUSTER_34152	gi|55168078|gb|AAV43946.1| unknown protein [Oryza sativa (japonica cultivar-group)] e_value:0	11682.m02706|LOC_Os05g28860|protein probable 3''-5'' exonuclease [imported] - Arabidopsis thaliana e_value:0	0.194	(0.0935 0.4812)	0.0935	0.4812

PDF Complete
Your complimentary use period has ended. Thank you for using PDF Complete.
Click Here to upgrade to Unlimited Pages and Expanded Features

<table>
<tr><td>3369</td><td>WT006_H15_CLUSTER_13394</td><td>gi|30908921|gb|AAP37480.1| putative zinc finger transcription factor ZFP33 [Oryza sativa (japonica cultivar-group)] >gnl|BL_ORD_ID|1855546 gi|50934425|ref|XP_476740.1| putative zinc finger protein 216 [Oryza sativa (japonica cultivar-group)] >gnl|BL</td><td>11673.m00662|LOC_Os07g07350|protein AN1-like Zinc finger, putative e_value:9.64361e-78</td><td>0.111</td><td>(0.0759 0.6859)</td><td>0.0759</td><td>0.6859</td></tr>
<tr><td>3370</td><td>WT005_O08_CLUSTER_18102</td><td>gi|50838955|gb|AAT81716.1| putative glycine rich protein [Oryza sativa (japonica cultivar-group)] >gnl|BL_ORD_ID|1025951 gi|50872466|gb|AAT85066.1| hypothetical protein [Oryza sativa (japonica cultivar-group)] e_value:1.36533e-157</td><td>11669.m04834|LOC_Os03g48260|protein putative glycine rich protein e_value:4.29042e-159</td><td>0.324</td><td>(0.2207 0.6804)</td><td>0.2207</td><td>0.6804</td></tr>
<tr><td>3371</td><td>WT011_G10_CLUSTER_28766</td><td>gi|34899766|ref|NP_911229.1| putative leucine-rich repeat transmembrane protein kinase 1 [Oryza sativa (japonica cultivar-group)] >gnl|BL_ORD_ID|1528957 gi|24417188|dbj|BAC22547.1| putative leucine-rich repeat transmembrane protein kinase 1 [Oryza sati</td><td>11673.m03668|LOC_Os07g37810|protein Protein kinase domain, putative e_value:0</td><td>0.25</td><td>(0.1503 0.6001)</td><td>0.1503</td><td>0.6001</td></tr>
<tr><td>3372</td><td>WT003_M04_CLUSTER_15939</td><td>gi|34910488|ref|NP_916591.1| putative H+-transporting ATP synthase [Oryza sativa (japonica cultivar-group)] >gnl|BL_ORD_ID|1547843 gi|20146320|dbj|BAB89101.1| putative vacuolar ATPase B subunit [Oryza sativa (japonica cultivar-group)] >gnl|BL_ORD_ID</td><td>11667.m05020|LOC_Os01g51380|protein V-type ATPase, B subunit e_value:0</td><td>0.03</td><td>(0.0118 0.4012)</td><td>0.0118</td><td>0.4012</td></tr>
<tr><td>3373</td><td>WT010_J23_CLUSTER_33820</td><td>gi|50905775|ref|XP_464376.1| receptor protein kinase PERK1-like protein [Oryza sativa (japonica cultivar-group)] >gnl|BL_ORD_ID|1566694 gi|51963906|ref|XP_506736.1| PREDICTED OJ1115_B01.27 gene product [Oryza sativa (japonica cultivar-</td><td>11668.m00857|LOC_Os02g09350|protein Protein kinase domain, putative e_value:1.76166e-149</td><td>0.088</td><td>(0.0803 0.9117)</td><td>0.0803</td><td>0.9117</td></tr>
<tr><td>3374</td><td>WT007_A19_CLUSTER_33861</td><td>gi|34911376|ref|NP_917035.1| putative Ser/Thr protein phosphatase [Oryza sativa (japonica cultivar-group)] >gnl|BL_ORD_ID|1547202 gi|18565419|dbj|BAB84606.1| putative phosphoprotein phosphatase [Oryza sativa (japonica cultivar-group)] e_value:8.01015e-</td><td>11667.m04837|LOC_Os01g49690|protein Ser/Thr protein phosphatase family protein, putative e_value:1.89389e-180</td><td>0.027</td><td>(0.0144 0.5371)</td><td>0.0144</td><td>0.5371</td></tr>
<tr><td>3375</td><td>WT005_P19_CLUSTER_14446</td><td>gi|50915526|ref|XP_468227.1| putative tumor differentially expressed protein 1 [Oryza sativa (japonica cultivar-group)] >gnl|BL_ORD_ID|1583348 gi|51979391|ref|XP_507542.1| PREDICTED OJ1249_F12.26 gene product [Oryza sativa (japonica cultivar-group)] &g</td><td>11668.m05418|LOC_Os02g54990|protein TMS membrane protein/tumour differentially expressed protein (TDE) e_value:0</td><td>0.055</td><td>(0.0267 0.4871)</td><td>0.0267</td><td>0.4871</td></tr>
<tr><td>3376</td><td>SET4_A05_CLUSTER_33086</td><td>gi|50915364|ref|XP_468146.1| 26S proteasome regulatory particle triple-A ATPase subunit1 [Oryza sativa (japonica cultivar-group)] >gi|51091125|dbj|BAD35822.1| 26S protease regulatory subunit 7 [Oryza sativa (japonica cultivar-group)] >gi|51090788|db</td><td>11680.m00888|LOC_Os06g09290|protein 26s protease regulatory subunit 7 (26s proteasome subunit 7) (26sproteasome aaa-atpase subunit rpt1) (regulatory particle triple-aatpase subunit</td><td>0.01</td><td>(0.0185 1.7665)</td><td>0.0185</td><td>1.7665</td></tr>
<tr><td>3377</td><td>WT007_P18_CLUSTER_12437</td><td>gi|57164636|gb|AAW34261.1| DANA2 [Zea mays] e_value:3.33158e-97</td><td>11669.m06554|LOC_Os03g63860|protein expressed protein e_value:1.16118e-91</td><td>0.521</td><td>(0.1586 0.3045)</td><td>0.1586</td><td>0.3045</td></tr>
<tr><td>3378</td><td>SET2_H08_CLUSTER_2160</td><td>gi|50926604|ref|XP_473249.1| OSJNBa0038O10.21 [Oryza sativa (japonica cultivar-group)] >gi|38344449|emb|CAE05655.2| OSJNBa0038O10.21 [Oryza sativa (japonica cultivar-group)] e_value:1.60906e-61</td><td>11670.m04317|LOC_Os04g44550|protein CHCH domain, putative e_value:2.01404e-63</td><td>0.171</td><td>(0.0899 0.5268)</td><td>0.0899</td><td>0.5268</td></tr>
<tr><td>3379</td><td>SET5_E07_CLUSTER_3265</td><td>gi|31433718|gb|AAP55197.1| putative ankyrin protein [Oryza sativa (japonica cultivar-group)] >gi|37537216|ref|NP_922911.1| putative ankyrin protein [Oryza sativa (japonica cultivar-group)] >gi|32352144|dbj|BAC78565.1| ankyrin [Oryza sativa (japonica</td><td>11676.m04006|LOC_Os10g43040|protein putative ankyrin protein e_value:4.52067e-116</td><td>0.168</td><td>(0.1022 0.6101)</td><td>0.1022</td><td>0.6101</td></tr>
<tr><td>3380</td><td>WT008_K04_CLUSTER_34226</td><td>gi|50908833|ref|XP_465905.1| putative mitochondrial NADH:ubiquinone oxidoreductase 29 kDa subunit [Oryza sativa (japonica cultivar-group)] >gnl|BL_ORD_ID|1576485 gi|48716956|dbj|BAD23649.1| putative mitochondrial NADH:ubiquinone oxidoreductase 29 kDa s</td><td>11668.m02895|LOC_Os02g30460|protein expressed protein e_value:1.00105e-128</td><td>0.075</td><td>(0.0399 0.5313)</td><td>0.0399</td><td>0.5313</td></tr>
<tr><td>3381</td><td>WT010_O20_CLUSTER_29564</td><td>gi|50918593|ref|XP_469693.1| putative ATP sulfurylase [Oryza sativa (japonica cultivar-group)] >gnl|BL_ORD_ID|1538763 gi|30017582|gb|AAP13004.1| putative ATP sulfurylase [Oryza sativa (japonica cultivar-group)] e_value:0</td><td>11669.m05387|LOC_Os03g53230|protein putative ATP sulfurylase e_value:0</td><td>0.13</td><td>(0.0639 0.4912)</td><td>0.0639</td><td>0.4912</td></tr>
<tr><td>3382</td><td>SET5_L12_CLUSTER_34273</td><td>gi|50905045|ref|XP_464011.1| putative Ras-GTPase activating protein SH3 domain-binding protein 2 [Oryza sativa (japonica cultivar-group)] >gi|41052569|dbj|BAD07751.1| putative Ras-GTPase activating protein SH3 domain-binding protein 2 [Oryza sativa (ja</td><td>11668.m00320|LOC_Os02g03890|protein Nuclear transport factor 2 (NTF2) domain, putative e_value:0</td><td>0.175</td><td>(0.1118 0.6396)</td><td>0.1118</td><td>0.6396</td></tr>
<tr><td>3383</td><td>WT010_G05_CLUSTER_32450</td><td>gi|24111401|gb|AAN46835.1| At1g71710/F14O23_9 [Arabidopsis thaliana] >gnl|BL_ORD_ID|1522044 gi|18409892|ref|NP_565023.1| inositol polyphosphate 5-phosphatase, putative [Arabidopsis thaliana] e_value:1.97569e-168</td><td>11667.m05072|LOC_Os01g51890|protein Endonuclease/Exonuclease/phosphatase family, putative</td><td>0.254</td><td>(0.1393 0.5488)</td><td>0.1393</td><td>0.5488</td></tr>
<tr><td>3384</td><td>WT005_D06_CLUSTER_34744</td><td>gi|14317906|dbj|BAB59136.1| casein kinase II alpha [Triticum aestivum] e_value:0</td><td>11673.m00141|LOC_Os07g02350|protein Protein kinase domain e_value:0</td><td>0.021</td><td>(0.0122 0.5882)</td><td>0.0122</td><td>0.5882</td></tr>
<tr><td>3385</td><td>WT008_G16_CLUSTER_900</td><td>gi|34895194|ref|NP_908922.1| B1051E10.38 [Oryza sativa (japonica cultivar-group)] >gnl|BL_ORD_ID|1561083 gi|21104839|dbj|BAB93424.1| SEC15 (S. cerevisiae)-like protein [Oryza sativa (japonica cultivar-group)] >gnl|BL_ORD_ID|1561083</td><td>11667.m02433|LOC_Os01g24950|protein Exocyst complex subunit Sec15, putative e_value:0</td><td>0.067</td><td>(0.0446 0.6673)</td><td>0.0446</td><td>0.6673</td></tr>
<tr><td>3386</td><td>SET2_B14_CLUSTER_33772</td><td>gi|31415917|gb|AAP50938.1| unknown protein [Oryza sativa (japonica cultivar-group)] >gi|50921125|ref|XP_470923.1| unknown protein [Oryza sativa (japonica cultivar-group)] e_value:1.19452e-134</td><td>11669.m02933|LOC_Os03g29180|protein expressed protein e_value:2.24752e-136</td><td>0.184</td><td>(0.1030 0.5602)</td><td>0.103</td><td>0.5602</td></tr>
<tr><td>3387</td><td>WT003_C16_CLUSTER_30085</td><td>gi|50901856|ref|XP_463361.1| B1097D05.23 [Oryza sativa (japonica cultivar-group)] >gnl|BL_ORD_ID|1542144 gi|21902022|dbj|BAC05572.1| unknown protein [Oryza sativa (japonica cultivar-group)] e_value:0</td><td>11667.m03848|LOC_Os01g40070|protein expressed protein e_value:0</td><td>0.219</td><td>(0.0884 0.4045)</td><td>0.0884</td><td>0.4045</td></tr>
<tr><td>3388</td><td>WT006_F02_CLUSTER_18970</td><td>gi|50911847|ref|XP_467331.1| putative gamma-tocopherol methyltransferase [Oryza sativa (japonica cultivar-group)] >gnl|BL_ORD_ID|1580451 gi|51964278|ref|XP_506924.1| PREDICTED OJ1111_E07.25 gene product [Oryza sativa (japonica cultivar-group)] >gnl|</td><td>11668.m04583|LOC_Os02g47310|protein expressed protein e_value:1.96085e-161</td><td>0.196</td><td>(0.1231 0.6297)</td><td>0.1231</td><td>0.6297</td></tr>
<tr><td>3389</td><td>WT002_D18_CLUSTER_15589</td><td>gi|50948179|ref|XP_483617.1| unknown protein [Oryza sativa (japonica cultivar-group)] >gnl|BL_ORD_ID|1610586 gi|42408556|dbj|BAD09734.1| unknown protein [Oryza sativa (japonica cultivar-group)] >gnl|BL_ORD_ID|1610586 gi|42408079|dbj|BAD09220.1| unkn</td><td>11674.m04358|LOC_Os08g43180|protein expressed protein e_value:0</td><td>0.367</td><td>(0.1395 0.3805)</td><td>0.1395</td><td>0.3805</td></tr>
<tr><td>3390</td><td>WT011_F01_CLUSTER_32806</td><td>gi|31431195|gb|AAP53010.1| putative mevalonate kinase [Oryza sativa (japonica cultivar-group)] >gnl|BL_ORD_ID|2009998 gi|37532842|ref|NP_920723.1| putative mevalonate kinase [Oryza sativa (japonica cultivar-group)] >gnl|BL_ORD_ID|2009998 gi|16905216</td><td>11676.m01520|LOC_Os10g18220|protein putative mevalonate kinase e_value:3.84329e-171</td><td>0.189</td><td>(0.1100 0.5824)</td><td>0.11</td><td>0.5824</td></tr>
<tr><td>3391</td><td>SET2_G19_CLUSTER_24352</td><td>gi|34978689|gb|AAQ83587.1| putative zinc finger transcription factor ZFP38 [Oryza sativa (japonica cultivar-group)] >gi|51979526|ref|XP_507556.1| PREDICTED OSJNBb0060J21.18 gene product [Oryza sativa (japonica cultivar-group)] >gi|50919117|ref|XP_46</td><td>11669.m05859|LOC_Os03g57890|protein putative zinc finger protein e_value:2.01046e-70</td><td>0.15</td><td>(0.1292 0.8617)</td><td>0.1292</td><td>0.8617</td></tr>
<tr><td>3392</td><td>WT005_G15_CLUSTER_34260</td><td>gi|34897892|ref|NP_910292.1| methylthioadenosine/S-adenosyl homocysteine nucleosidase [Oryza sativa (japonica cultivar-group)] >gnl|BL_ORD_ID|1752560 gi|32352128|dbj|BAC78557.1| hypothetical protein [Oryza sativa (japonica cultivar-group)] >gnl|BL_O</td><td>11680.m00129|LOC_Os06g02220|protein Phosphorylase family e_value:6.51272e-123</td><td>0.161</td><td>(0.0837 0.5209)</td><td>0.0837</td><td>0.5209</td></tr>
<tr><td>3393</td><td>SET5_N17_CLUSTER_34815</td><td>gi|2654208|gb|AAB91471.1| heat shock 70 protein [Spinacia oleracea] >gi|2773050|gb|AAB96659.1| heat shock 70 protein [Spinacia oleracea] >gi|7441880|pir||T08899 dnaK-type molecular chaperone HSC70-9, chloroplast - spinach e_value:0</td><td>11686.m01378|LOC_Os12g14070|protein chaperone protein DnaK e_value:0</td><td>0.093</td><td>(0.0529 0.5672)</td><td>0.0529</td><td>0.5672</td></tr>
<tr><td>3394</td><td>SET1_F18_CLUSTER_33758</td><td>gi|20159729|gb|AAM11916.1| unknown cold induced protein [Deschampsia antarctica] e_value:4.08277e-58</td><td>11667.m05277|LOC_Os01g53730|protein expressed protein e_value:1.75909e-54</td><td>0.123</td><td>(0.1743 1.4182)</td><td>0.1743</td><td>1.4182</td></tr>
<tr><td>3395</td><td>WT009_F07_CLUSTER_33828</td><td>gi|34909932|ref|NP_916313.1| putative ubiquitin carboxyl-terminal hydrolase [Oryza sativa (japonica cultivar-group)] >gnl|BL_ORD_ID|1551976 gi|56785209|dbj|BAD82061.1| putative ubiquitin carboxyl-terminal hydrolase 7 [Oryza sativa (japonica cultivar-</td><td>11667.m05586|LOC_Os01g56490|protein Ubiquitin carboxyl-terminal hydrolase, putative e_value:0</td><td>0.214</td><td>(0.1427 0.6664)</td><td>0.1427</td><td>0.6664</td></tr>
<tr><td>3396</td><td>WT008_B06_CLUSTER_4103</td><td>gi|34912442|ref|NP_917568.1| P0681B11.25 [Oryza sativa (japonica cultivar-group)] e_value:0</td><td>11667.m04052|LOC_Os01g42030|protein ATPase, AAA family, putative e_value:0</td><td>0.104</td><td>(0.0511 0.4931)</td><td>0.0511</td><td>0.4931</td></tr>
<tr><td>3397</td><td>WT006_L13_CLUSTER_21592</td><td>gi|50912885|ref|XP_467850.1| putative Ribosomal RNA processing protein [Oryza sativa (japonica cultivar-group)] >gnl|BL_ORD_ID|1582967 gi|46805940|dbj|BAD17234.1| putative Ribosomal RNA processing protein [Oryza sativa (japonica cultivar-</td><td>11668.m05061|LOC_Os02g51780|protein RNB-like protein, putative e_value:0</td><td>0.292</td><td>(0.1429 0.4895)</td><td>0.1429</td><td>0.4895</td></tr>
<tr><td>3398</td><td>SET4_I06_CLUSTER_31496</td><td>gi|50915650|ref|XP_468289.1| putative quinone oxidoreductase [Oryza sativa (japonica cultivar-group)] >gi|51979438|ref|XP_507546.1| PREDICTED OJ1548_F12.23 gene product [Oryza sativa (japonica cultivar-group)] >gi|51964492|ref|XP_507031.1| PREDICTED</td><td>11668.m05543|LOC_Os02g56180|protein oxidoreductase, zinc-binding dehydrogenase family e_value:4.41563e-162</td><td>0.149</td><td>(0.0564 0.3794)</td><td>0.0564</td><td>0.3794</td></tr>
<tr><td>3399</td><td>WT007_I01_CLUSTER_33766</td><td>gi|22655416|gb|AAM98300.1| At3g58180/F9D24_90 [Arabidopsis thaliana] >gnl|BL_ORD_ID|1197074 gi|13926240|gb|AAK49594.1| AT3g58180/F9D24_90 [Arabidopsis thaliana] >gnl|BL_ORD_ID|1197074 gi|18410896|ref|NP_567062.1| PBS lyase HEAT-like repeat-</td><td>11686.m04305|LOC_Os12g43100|protein expressed protein e_value:6.80214e-149</td><td>0.107</td><td>(0.0488 0.4563)</td><td>0.0488</td><td>0.4563</td></tr>
<tr><td>3400</td><td>WT004_P17_CLUSTER_31986</td><td>gi|50726629|dbj|BAD34349.1| phosphatidylinositol 3- and 4-kinase family-like [Oryza sativa (japonica cultivar-group)] e_value:0</td><td>11681.m02789|LOC_Os09g29890|protein Phosphatidylinositol 3- and 4-kinase, putative e_value:0</td><td>0.176</td><td>(0.0852 0.4838)</td><td>0.0852</td><td>0.4838</td></tr>
<tr><td>3401</td><td>SET5_E14_CLUSTER_23010</td><td>gi|50252055|dbj|BAD27986.1| putative small nuclear ribonucleoprotein polypeptide F [Oryza sativa (japonica cultivar-group)] e_value:2.1634e-36</td><td>11668.m00774|LOC_Os02g08090|protein probable small nuclear ribonucleoprotein f (snrnp-f) (sm protein f)(sm-f) (smf) e_value:1.85534e-38</td><td>0.115</td><td>(0.0846 0.7373)</td><td>0.0846</td><td>0.7373</td></tr>
<tr><td>3402</td><td>WT011_E14_CLUSTER_20519</td><td>gi|53370697|gb|AAU89192.1| expressed protein [Oryza sativa (japonica cultivar-group)] e_value:0</td><td>11669.m04434|LOC_Os03g44530|protein expressed protein</td><td>0.253</td><td>(0.1065 0.4214)</td><td>0.1065</td><td>0.4214</td></tr>
<tr><td>3403</td><td>WT007_O12_CLUSTER_20850</td><td>gi|57899790|dbj|BAD87535.1| ALM beta-like [Oryza sativa (japonica cultivar-group)] e_value:1.63959e-116</td><td>11667.m06705|LOC_Os01g66730|protein expressed protein e_value:3.17215e-118</td><td>0.118</td><td>(0.0678 0.5741)</td><td>0.0678</td><td>0.5741</td></tr>
<tr><td>3404</td><td>WT005_J15_CLUSTER_6896</td><td>gi|34901780|ref|NP_912236.1| putative ATP synthase delta' chain, mitochondrial precursor [Oryza sativa (japonica cultivar-group)] >gnl|BL_ORD_ID|1525440 gi|24059902|dbj|BAC21366.1| putative ATP synthase delta' chain, mitochondrial precursor [</td><td>11673.m02982|LOC_Os07g31300|protein ATP synthase F1, epsilon subunit, putative e_value:5.35153e-89</td><td>0.423</td><td>(0.1247 0.2946)</td><td>0.1247</td><td>0.2946</td></tr>
<tr><td>3405</td><td>WT012_O18_CLUSTER_2799</td><td>gi|55168021|gb|AAV43889.1| unknown protein [Oryza sativa (japonica cultivar-group)] e_value:0</td><td>11682.m02683|LOC_Os05g28630|protein expressed protein</td><td>0.299</td><td>(0.2011 0.6719)</td><td>0.2011</td><td>0.6719</td></tr>
<tr><td>3406</td><td>WT004_L13_CLUSTER_21784</td><td>gi|34908962|ref|NP_915828.1| P0003D09.18 [Oryza sativa (japonica cultivar-group)] >gnl|BL_ORD_ID|1554686 gi|19570997|dbj|BAB86424.1| mucin-like protein [Oryza sativa (japonica cultivar-group)] e_value:5.42182e-171</td><td>11667.m05830|LOC_Os01g58750|protein expressed protein e_value:1.69821e-172</td><td>0.192</td><td>(0.0747 0.3898)</td><td>0.0747</td><td>0.3898</td></tr>
<tr><td>3407</td><td>WT007_K12_CLUSTER_33507</td><td>gi|31712074|gb|AAP68379.1| putative RNA-binding protein [Oryza sativa (japonica cultivar-group)] >gnl|BL_ORD_ID|2049985 gi|50917825|ref|XP_469309.1| putative RNA-binding protein [Oryza sativa (japonica cultivar-group)] e_value:8.24242e-152</td><td>11669.m04788|LOC_Os03g47800|protein Transposable element protein, putative e_value:2.09715e-153</td><td>0.142</td><td>(0.0917 0.6465)</td><td>0.0917</td><td>0.6465</td></tr>
<tr><td>3408</td><td>WT011_M02_CLUSTER_29662</td><td>gi|37718868|gb|AAR01739.1| putative LSTK-1-like kinase [Oryza sativa (japonica cultivar-group)] >gnl|BL_ORD_ID|2260638 gi|50917187|ref|XP_468990.1| putative LSTK-1-like kinase [Oryza sativa (japonica cultivar-group)] e_value:0</td><td>11686.m04088|LOC_Os12g41180|protein Protein kinase domain, putative e_value:0</td><td>0.095</td><td>(0.0519 0.5451)</td><td>0.0519</td><td>0.5451</td></tr>
<tr><td>3409</td><td>WT006_P12_CLUSTER_23676</td><td>gi|34903732|ref|NP_913213.1| unnamed protein product [Oryza sativa (japonica cultivar-group)] >gnl|BL_ORD_ID|1752026 gi|7340858|dbj|BAA92948.1| unknown protein [Oryza sativa (japonica cultivar-group)] e_value:0</td><td>11667.m01036|LOC_Os01g10680|protein expressed protein e_value:0</td><td>0.26</td><td>(0.1079 0.4153)</td><td>0.1079</td><td>0.4153</td></tr>
<tr><td>3410</td><td>WT010_I08_CLUSTER_15697</td><td>gi|57900170|dbj|BAD88255.1| putative mitochondrial processing peptidase [Oryza sativa (japonica cultivar-group)] e_value:0</td><td>11667.m07386|LOC_Os01g73550|protein Insulinase (Peptidase family M16), putative e_value:0</td><td>0.202</td><td>(0.1121 0.5547)</td><td>0.1121</td><td>0.5547</td></tr>
</table>

PDF Complete
Your complimentary use period has ended. Thank you for using PDF Complete.
Click Here to upgrade to Unlimited Pages and Expanded Features

3411	WT002_I14_CLUSTER_8763	gi|51963776|ref|XP_506668.1| PREDICTED OJ1595_D08.21 gene product [Oryza sativa (japonica cultivar-group)] >gnl|BL_ORD_ID|1564291 gi|50900358|ref|XP_450967.1| homeodomain leucine zipper protein [Oryza sativa (japonica cultivar-group)]	11681.m02743|LOC_Os09g29460|protein Homeobox associated leucine zipper, putative e_value:1.50913e-114	0.346	(0.1104 0.3195)	0.1104	0.3195
3412	WT013_M16_CLUSTER_34544	gi|50935357|ref|XP_477206.1| putative RNA and export factor binding protein [Oryza sativa (japonica cultivar-group)] >gnl|BL_ORD_ID|1523252 gi|33146678|dbj|BAC80073.1| putative RNA and export factor binding protein [Oryza sativa (japonica cultivar-	11687.m00002|LOC_Os11g01020|protein transcriptional coactivator-like protein e_value:1.82028e-43	0.339	(0.3930 1.1596)	0.393	1.1596
3413	WT010_P19_CLUSTER_18370	gi|51535592|dbj|BAD37536.1| protein kinase-like [Oryza sativa (japonica cultivar-group)] >gnl|BL_ORD_ID|250887 gi|51536357|dbj|BAD37488.1| protein kinase-like [Oryza sativa (japonica cultivar-group)] e_value:0	11680.m04246|LOC_Os06g43270|protein At4g32250/F10M6_110 e_value:0	0.103	(0.0836 0.8096)	0.0836	0.8096
3414	WT006_M16_CLUSTER_34048	gi|31433543|gb|AAP55041.1| putative nucleic acid binding protein [Oryza sativa (japonica cultivar-group)] >gnl|BL_ORD_ID|2013064 gi|37536904|ref|NP_922754.1| putative nucleic acid binding protein [Oryza sativa (japonica cultivar-group)] >gnl|BL_ORD_	11676.m03814|LOC_Os10g41440|protein putative nucleic acid binding protein e_value:0	0.167	(0.0739 0.4429)	0.0739	0.4429
3415	WT006_A20_CLUSTER_3599	gi|51536001|dbj|BAD38081.1| putative chromatin complex subunit A101 [Oryza sativa (japonica cultivar-group)] e_value:0	11669.m05166|LOC_Os03g51230|protein Transposable element protein, putative e_value:0	0.191	(0.1612 0.8438)	0.1612	0.8438
3416	WT008_F20_CLUSTER_34101	gi|38346055|emb|CAE02007.2| OJ000223_09.9 [Oryza sativa (japonica cultivar-group)] >gnl|BL_ORD_ID|1523159 gi|38345346|emb|CAE03157.2| OSJNBa0081L15.19 [Oryza sativa (japonica cultivar-group)] >gnl|BL_ORD_ID|1523159 gi|50925291|ref|XP_472943.1| OSJNB	11670.m03915|LOC_Os04g40880|protein ParA family ATPase, putative e_value:0	0.041	(0.0432 1.0505)	0.0432	1.0505
3417	WT005_G08_CLUSTER_33238	gi|23345038|gb|AAN17454.1| hypersensitive-induced reaction protein 4 [Hordeum vulgare subsp. vulgare] >gnl|BL_ORD_ID|2285602 gi|23345048|gb|AAN17465.1| hypersensitive-induced reaction protein 4 [Hordeum vulgare subsp. vulgare] e_value:1.23785e-157	11680.m00366|LOC_Os06g04460|protein hypersensitive-induced reaction protein 4 e_value:1.95335e-147	0.092	(0.0527 0.5730)	0.0527	0.573
3418	WT002_G05_CLUSTER_31909	gi|29367365|gb|AAO72555.1| PP2A regulatory subunit-like protein [Oryza sativa (japonica cultivar-group)] e_value:0	11686.m00355|LOC_Os12g04290|protein TAP42-like family	0.125	(0.0628 0.5008)	0.0628	0.5008
3419	WT006_C19_CLUSTER_34256	gi|31432560|gb|AAP54175.1| putative WD domain containing protein [Oryza sativa (japonica cultivar-group)] >gnl|BL_ORD_ID|2012062 gi|37535172|ref|NP_921888.1| putative WD domain containing protein [Oryza sativa (japonica cultivar-group)] >gnl|BL_ORD_	11676.m02869|LOC_Os10g32710|protein putative WD domain containing protein e_value:0	0.129	(0.0746 0.5784)	0.0746	0.5784
3420	SET2_B12_CLUSTER_27854	gi|50945861|ref|XP_482458.1| unknown protein [Oryza sativa (japonica cultivar-group)] >gi|37573093|dbj|BAC98663.1| unknown protein [Oryza sativa (japonica cultivar-group)] e_value:4.30254e-77	11667.m01552|LOC_Os01g15520|protein hypothetical protein e_value:7.80681e-80	0.242	(0.1613 0.6674)	0.1613	0.6674
3421	WT004_C06_CLUSTER_31812	gi|31432570|gb|AAP54185.1| putative amylase [Oryza sativa (japonica cultivar-group)] >gnl|BL_ORD_ID|2012071 gi|37535192|ref|NP_921898.1| putative amylase [Oryza sativa (japonica cultivar-group)] >gnl|BL_ORD_ID|2012071 gi|13489165|gb|AAK27799.1| puta	11669.m00417|LOC_Os03g04770|protein Glycosyl hydrolase family 14, putative e_value:0	0.259	(0.1239 0.4780)	0.1239	0.478
3422	WT003_L05_CLUSTER_34924	gi|50911156|ref|XP_467011.1| asparaginyl endopeptidase REP-2 [Oryza sativa (japonica cultivar-group)] >gnl|BL_ORD_ID|1579937 gi|26006022|dbj|BAC41387.1| asparaginyl endopeptidase REP-2 [Oryza sativa (japonica cultivar-group)] >gnl|BL_ORD_ID|1579937	11668.m04119|LOC_Os02g43010|protein Peptidase C13 family, putative e_value:0	0.102	(0.0737 0.7207)	0.0737	0.7207
3423	WT008_I03_CLUSTER_33248	gi|38605952|emb|CAD41666.3| OSJNBa0019K04.13 [Oryza sativa (japonica cultivar-group)] >gnl|BL_ORD_ID|1524312 gi|50928103|ref|XP_473579.1| OSJNBa0019K04.13 [Oryza sativa (japonica cultivar-group)] e_value:0	11670.m04753|LOC_Os04g48460|protein Cytochrome P450 e_value:0	0.308	(0.1427 0.4630)	0.1427	0.463
3424	WT003_B20_CLUSTER_34004	gi|38346050|emb|CAE02005.2| OJ000223_09.4 [Oryza sativa (japonica cultivar-group)] >gnl|BL_ORD_ID|1523154 gi|38345341|emb|CAE03152.2| OSJNBa0081L15.14 [Oryza sativa (japonica cultivar-group)] >gnl|BL_ORD_ID|1523154 gi|50925277|ref|XP_472938.1| OSJNB	11670.m03911|LOC_Os04g40840|protein histone acetyltransferase, ELP3 family e_value:0	0.036	(0.0171 0.4715)	0.0171	0.4715
3425	WT006_K15_CLUSTER_33814	gi|52076165|dbj|BAD46678.1| putative RNA helicase [Oryza sativa (japonica cultivar-group)] e_value:0	11681.m03128|LOC_Os09g34910|protein RNA helicase e_value:0	0.237	(0.1281 0.5401)	0.1281	0.5401
3426	WT013_M18_CLUSTER_19935	gi|24111419|gb|AAN46859.1| At2g32120/F22D22.13 [Arabidopsis thaliana] >gnl|BL_ORD_ID|1522052 gi|21703121|gb|AAM74502.1| At2g32120/F22D22.13 [Arabidopsis thaliana] >gnl|BL_ORD_ID|1522052 gi|4263707|gb|AAD15393.1| 70kD heat shock protein [Arabidopsis	11669.m01147|LOC_Os03g11910|protein 70kD heat shock protein e_value:0	0.09	(0.0686 0.7653)	0.0686	0.7653
3427	SET2_C09_CLUSTER_13083	gi|50939241|ref|XP_479148.1| ABC transporter permease protein-like protein [Oryza sativa (japonica cultivar-group)] >gi|23237921|dbj|BAC16494.1| ABC transporter permease protein-like protein [Oryza sativa (japonica cultivar-group)] e_value:4.29788e-	11673.m04137|LOC_Os07g42250|protein Strictosidine synthase e_value:1.12895e-148	0.377	(0.1816 0.4819)	0.1816	0.4819
3428	WT007_O01_CLUSTER_31710	gi|21742781|emb|CAD40535.1| OSJNBa0055C08.3 [Oryza sativa (japonica cultivar-group)] >gnl|BL_ORD_ID|1536053 gi|50923873|ref|XP_472297.1| OSJNBa0055C08.3 [Oryza sativa (japonica cultivar-group)] e_value:4.07197e-106	11686.m02692|LOC_Os12g27830|protein Similar to AB023037 11-beta-hydroxysteroid dehydrogenase e_value:8.20508e-170	0.141	(0.1013 0.7193)	0.1013	0.7193
3429	SET3_E08_CLUSTER_31710	gi|50923873|ref|XP_472297.1| OSJNBa0055C08.3 [Oryza sativa (japonica cultivar-group)] >gi|21742781|emb|CAD40535.1| OSJNBa0055C08.3 [Oryza sativa (japonica cultivar-group)] e_value:4.22171e-106	11686.m02692|LOC_Os12g27830|protein Similar to AB023037 11-beta-hydroxysteroid dehydrogenase e_value:8.20508e-170	0.137	(0.1013 0.7402)	0.1013	0.7402
3430	SET5_I18_CLUSTER_29002	gi|34904486|ref|NP_913590.1| putative heat shock protein 40 [Oryza sativa (japonica cultivar-group)] >gi|13486861|dbj|BAB40091.1| putative DnaJ-like protein [Oryza sativa (japonica cultivar-group)] >gi|11034711|dbj|BAB17212.1| putative DnaJ-like pro	11667.m01359|LOC_Os01g13760|protein DnaJ domain, putative e_value:1.53972e-152	0.19	(0.1283 0.6755)	0.1283	0.6755
3431	WT009_J24_CLUSTER_33414	gi|50919843|ref|XP_470282.1| putative embryo-specific protein [Oryza sativa (japonica cultivar-group)] >gnl|BL_ORD_ID|1539356 gi|19071621|gb|AAL84288.1| putative embryo-specific protein [Oryza sativa (japonica cultivar-group)] e_value:3.53704e-97	11669.m00680|LOC_Os03g07180|protein putative embryo-specific protein e_value:1.14573e-98	0.658	(0.2968 0.4512)	0.2968	0.4512
3432	WT004_A01_CLUSTER_33924	gi|22460|emb|CAA41854.1| rab28 [Zea mays] >gnl|BL_ORD_ID|985631 gi|100920|pir||S18545 rab28 protein - maize e_value:5.57572e-99	11669.m00589|LOC_Os03g06360|protein Seed maturation protein, putative e_value:2.22911e-97	0.412	(0.1803 0.4379)	0.1803	0.4379
3433	SET2_P15_CLUSTER_28361	gi|4105692|gb|AAD02496.1| embryo-specific protein [Oryza sativa subsp. indica] >gi|4105683|gb|AAD02495.1| unknown [Oryza sativa] e_value:7.49016e-123	11682.m04750|LOC_Os05g49440|protein expressed protein e_value:1.71588e-124	0.234	(0.0705 0.3011)	0.0705	0.3011
3434	WT007_A13_CLUSTER_11784	gi|10140710|gb|AAG13544.1| putative beta-tonoplast intrinsic protein [Oryza sativa (japonica cultivar-group)] >gnl|BL_ORD_ID|2281666 gi|31432819|gb|AAP54406.1| putative beta-tonoplast intrinsic protein [Oryza sativa (japonica cultivar-group)] >gnl|B	11676.m03116|LOC_Os10g35050|protein putative beta-tonoplast intrinsic protein e_value:4.80757e-134	0.196	(0.0592 0.3023)	0.0592	0.3023
3435	SET5_L04_CLUSTER_33011	gi|2130034|pir||S65779 gamma-thionin precursor - barley >gi|790641|gb|AAB01671.1| gamma-thionin e_value:2.87841e-34	11668.m01100|LOC_Os02g12060|protein proteinase inhibitor - rice e_value:3.84027e-07	0.817	(0.6566 0.8033)	0.6566	0.8033
3436	SET1_C18_CLUSTER_32874	gi|100844|pir||S11530 purothionin gamma - poulard wheat >gi|135784|sp|P20159|THG2_WHEAT Gamma-2 purothionin >gi|227203|prf||1616227B purothionin gamma2 e_value:4.3719e-24	11669.m00315|LOC_Os03g03810|protein proteinase inhibitor - rice e_value:1.51791e-11	1.11	(0.6416 0.5779)	0.6416	0.5779
3437	SET3_I03_CLUSTER_34085	gi|19013|emb|CAA44622.1| LEA B19.1 [Hordeum vulgare subsp. vulgare] >gi|542122|pir||S23749 embryonic abundant protein B19.1 - barley >gi|547817|sp|Q05190|L19A_HORVU Late embryogenesis abundant protein B19.1A (B19.1) e_value:5.57764e-45	11682.m02640|LOC_Os05g28210|protein embryonic abundant protein 1 e_value:2.72389e-42	0.276	(0.0741 0.2689)	0.0741	0.2689
3438	WT006_B03_CLUSTER_20478	gi|17221648|dbj|BAB78478.1| preproMP73 [Cucurbita maxima] e_value:6.63164e-74	11669.m00954|LOC_Os03g10110|protein Similar to preproMP73 e_value:0	0.278	(0.1941 0.6992)	0.1941	0.6992
3439	SET1_L14_CLUSTER_3616	gi|50928369|ref|XP_473712.1| OSJNBa0086O06.12 [Oryza sativa (japonica cultivar-group)] >gi|38346714|emb|CAE04864.2| OSJNBa0086O06.12 [Oryza sativa (japonica cultivar-group)] e_value:1.03101e-32	11670.m04919|LOC_Os04g49980|protein Late embryogenesis abundant (LEA) group 1, putative e_value:8.97244e-35	0.564	(0.1932 0.3424)	0.1932	0.3424
3440	WT008_P07_CLUSTER_31006	gi|50929593|ref|XP_474324.1| OSJNBa0018M05.11 [Oryza sativa (japonica cultivar-group)] >gnl|BL_ORD_ID|1594466 gi|38344920|emb|CAE03236.2| OSJNBa0018M05.11 [Oryza sativa (japonica cultivar-group)] e_value:1.06013e-116	11670.m05718|LOC_Os04g57810|protein expressed protein e_value:2.2226e-118	0.17	(0.1063 0.6236)	0.1063	0.6236
3441	WT008_O02_CLUSTER_34498	gi|31429900|gb|AAP51889.1| putative o-methyltransferase ZRP4 [Oryza sativa (japonica cultivar-group)] >gnl|BL_ORD_ID|2007840 gi|37530600|ref|NP_919602.1| putative o-methyltransferase ZRP4 [Oryza sativa (japonica cultivar-group)] >gnl|BL_ORD_ID|20078	11676.m00192|LOC_Os10g02840|protein O-methyltransferase, putative e_value:2.1344e-109	0.292	(0.2938 1.0056)	0.2938	1.0056
3442	SET1_C02_CLUSTER_2519	gi|123975|sp|P16347|IAAS_WHEAT Endogenous alpha-amylase/subtilisin inhibitor (WASI) >gi|225042|prf||1207200A alpha amylase inhibitor e_value:5.06862e-103	11670.m04307|LOC_Os04g44470|protein alpha-amylase/subtilisin inhibitor (rasi) e_value:1.52313e-64	0.607	(0.2481 0.4084)	0.2481	0.4084
3443	WT005_K20_CLUSTER_32691	gi|50924045|ref|XP_472383.1| OSJNBb0118P14.11 [Oryza sativa (japonica cultivar-group)] >gnl|BL_ORD_ID|1591437 gi|38569173|emb|CAE05363.3| OJ000315_02.8 [Oryza sativa (japonica cultivar-group)] >gnl|BL_ORD_ID|1591437 gi|38346151|emb|CAD40673.2| OSJNB	11670.m03178|LOC_Os04g33150|protein hypothetical protein e_value:1.3198e-155	0.302	(0.0884 0.2929)	0.0884	0.2929
3444	SET1_O23_CLUSTER_28558	gi|15451616|gb|AAK98740.1| Hypothetical protein with similarity to ankyrins and BRCA1-associated ring domain proteins [Oryza sativa] e_value:1.55829e-77	11669.m00471|LOC_Os03g05260|protein hypothetical protein e_value:1.77478e-47	0.591	(0.3379 0.5720)	0.3379	0.572
3445	SET1_M16_CLUSTER_1549	gi|50924790|ref|XP_472745.1| OSJNBa0036B21.25 [Oryza sativa (japonica cultivar-group)] >gi|21740911|emb|CAD40907.1| OSJNBa0036B21.25 [Oryza sativa (japonica cultivar-group)] >gi|32479938|emb|CAE01533.1| OSJNBa0072F16.2 [Oryza sativa (japonica cultiv	11670.m03661|LOC_Os04g38610|protein Ran-interacting Mob1 protein, putative e_value:6.56424e-100	0.126	(0.0608 0.4841)	0.0608	0.4841
3446	WT003_J12_CLUSTER_27651	gi|50918733|ref|XP_469763.1| putative stress-related protein [Oryza sativa (japonica cultivar-group)] >gnl|BL_ORD_ID|1586191 gi|40539010|gb|AAR87267.1| putative stress-related protein [Oryza sativa (japonica cultivar-group)] e_value:8.69917e-60	11669.m05464|LOC_Os03g53900|protein putative stress-related protein e_value:2.12054e-61	0.559	(0.1818 0.3252)	0.1818	0.3252
3447	SET1_I08_CLUSTER_20860	gi|50945233|ref|XP_482144.1| unknown protein [Oryza sativa (japonica cultivar-group)] >gi|40253896|dbj|BAD05830.1| unknown protein [Oryza sativa (japonica cultivar-group)] e_value:3.7727e-31	11674.m02907|LOC_Os08g29600|protein expressed protein e_value:8.896e-33	0.225	(0.0987 0.4378)	0.0987	0.4378
3448	WT004_M14_CLUSTER_32186	gi|25777824|gb|AAN75627.1| lipid transfer protein 1 precursor [Triticum aestivum] e_value:2.83081e-62	11687.m00146|LOC_Os11g02390|protein Protease inhibitor/seed storage/LTP family, putative e_value:2.10105e-36	0.522	(0.3515 0.6733)	0.3515	0.6733
3449	WT009_M06_CLUSTER_6181	gi|53370752|gb|AAU89247.1| hypothetical protein [Oryza sativa (japonica cultivar-group)] e_value:0	11669.m02949|LOC_Os03g29340|protein expressed protein	0.172	(0.1255 0.7310)	0.1255	0.731
3450	WT009_B07_CLUSTER_3964	gi|50915540|ref|XP_468234.1| hypothetical protein [Oryza sativa (japonica cultivar-group)] >gnl|BL_ORD_ID|1583355 gi|47497144|dbj|BAD19193.1| hypothetical protein [Oryza sativa (japonica cultivar-group)] >gnl|BL_ORD_ID|1583355	11668.m05425|LOC_Os02g55050|protein F-box domain, putative e_value:1.31241e-118	0.222	(0.4173 1.8781)	0.4173	1.8781
3451	WT007_P15_CLUSTER_12693	gi|56784749|dbj|BAD81898.1| beta-glucanase-like protein [Oryza sativa (japonica cultivar-group)] >gnl|BL_ORD_ID|803616 gi|56784619|dbj|BAD81666.1| beta-glucanase-like protein [Oryza sativa (japonica cultivar-group)] e_value:0	11667.m06603|LOC_Os01g65720|protein hypothetical protein e_value:0	0.117	(0.1115 0.9513)	0.1115	0.9513
3452	WT007_F16_CLUSTER_20417	gi|34900662|ref|NP_911677.1| putative UDP-glucosyltransferase [Oryza sativa (japonica cultivar-group)] >gnl|BL_ORD_ID|1523279 gi|23237887|dbj|BAC16461.1| putative UDP-glucosyltransferase [Oryza sativa (japonica cultivar-group)] e_value:2.56893e-164	11669.m06205|LOC_Os03g60960|protein putative UDP-glucosyltransferase e_value:1.09105e-162	0.573	(0.2535 0.4422)	0.2535	0.4422
3453	WT002_A10_CLUSTER_20227	gi|3135957|emb|CAA06856.1| wheatwin1 [Triticum aestivum] >gnl|BL_ORD_ID|987970 gi|7442146|pir||T06485 barwin homolog wheatwin1 precursor - wheat >gnl|BL_ORD_ID|987970 gi|34925030|sp|O64392|WHW1_WHEAT Wheatwin1 precursor (Pathogenesis-related protein	11687.m03508|LOC_Os11g37950|protein barwin e_value:2.10401e-70	0.166	(0.1113 0.6716)	0.1113	0.6716

PDF Complete
Your complimentary use period has ended. Thank you for using PDF Complete.
Click Here to upgrade to Unlimited Pages and Expanded Features

<table>
<tr><td>3454</td><td>WT002_C22_CLUSTER_4567</td><td>gi|31433545|gb|AAP55043.1| hypothetical protein [Oryza sativa (japonica cultivar-group)] >gnl|BL_ORD_ID|2013066 gi|37536908|ref|NP_922756.1| hypothetical protein [Oryza sativa (japonica cultivar-group)] >gnl|BL_ORD_ID|2013066 gi|12597883|gb|AAG60191</td><td>11676.m03816|LOC_Os10g41460|protein expressed protein e_value:1.56251e-132</td><td>0.232</td><td>(0.1001 0.4313)</td><td>0.1001</td><td>0.4313</td></tr>
<tr><td>3455</td><td>WT011_A06_CLUSTER_27833</td><td>gi|49065958|gb|AAT49064.1| ent-kaurene oxidase 1 [Hordeum vulgare subsp. vulgare] e_value:0</td><td>11680.m03628|LOC_Os06g37350|protein ent-kaurene oxidase CYP701A5 e_value:0</td><td>0.181</td><td>(0.1111 0.6149)</td><td>0.1111</td><td>0.6149</td></tr>
<tr><td>3456</td><td>WT009_P23_CLUSTER_23424</td><td>gi|50508168|dbj|BAD30974.1| putative formamidopyrimidine-DNA glycosylase [Oryza sativa (japonica cultivar-group)] >gnl|BL_ORD_ID|230942 gi|50508157|dbj|BAD30940.1| putative formamidopyrimidine-DNA glycosylase [Oryza sativa (japonica cultivar-group)] e_</td><td>11674.m02059|LOC_Os08g21330|protein putative glycosylase e_value:1.60412e-130</td><td>0.426</td><td>(0.3044 0.7152)</td><td>0.3044</td><td>0.7152</td></tr>
<tr><td>3457</td><td>WT009_O23_CLUSTER_34003</td><td>gi|50906511|ref|XP_464744.1| putative peroxisomal membrane protein 22 kDa [Oryza sativa (japonica cultivar-group)] >gnl|BL_ORD_ID|1566983 gi|49388530|dbj|BAD25652.1| putative peroxisomal membrane protein 22 kDa [Oryza sativa (japonica cultivar-group)]</td><td>11668.m01223|LOC_Os02g13270|protein Mpv17 / PMP22 family, putative e_value:3.99065e-102</td><td>0.139</td><td>(0.0582 0.4189)</td><td>0.0582</td><td>0.4189</td></tr>
<tr><td>3458</td><td>WT011_O14_CLUSTER_31512</td><td>gi|56201272|dbj|BAD72882.1| xylanase inhibitor TAXI-IV [Triticum aestivum] e_value:0</td><td>11667.m07127|LOC_Os01g71090|protein expressed protein e_value:7.87468e-122</td><td>0.795</td><td>(0.3502 0.4407)</td><td>0.3502</td><td>0.4407</td></tr>
<tr><td>3459</td><td>WT009_E12_CLUSTER_19835</td><td>gi|34898788|ref|NP_910740.1| unknown protein [Oryza sativa (japonica cultivar-group)] >gnl|BL_ORD_ID|1528235 gi|50508288|dbj|BAD32137.1| unknown protein [Oryza sativa (japonica cultivar-group)] >gnl|BL_ORD_ID|1528235 gi|22831058|dbj|BAC15921.1| unkn</td><td>11673.m03375|LOC_Os07g35050|protein F-box domain, putative e_value:1.03866e-50</td><td>0.642</td><td>(0.6459 1.0063)</td><td>0.6459</td><td>1.0063</td></tr>
<tr><td>3460</td><td>WT009_O10_CLUSTER_9404</td><td>gi|3080740|gb|AAC39445.1| pasticcino 1-D [Arabidopsis thaliana] e_value:0</td><td>11669.m02548|LOC_Os03g25140|protein pasticcino 1-A</td><td>0.164</td><td>(0.0862 0.5241)</td><td>0.0862</td><td>0.5241</td></tr>
<tr><td>3461</td><td>WT008_G03_CLUSTER_29572</td><td>gi|21555820|gb|AAM63941.1| unknown [Arabidopsis thaliana] >gnl|BL_ORD_ID|1217199 gi|22136760|gb|AAM91699.1| unknown protein [Arabidopsis thaliana] >gnl|BL_ORD_ID|1217199 gi|17979289|gb|AAL49870.1| unknown protein [Arabidopsis thaliana] >gnl|BL_OR</td><td>11686.m00778|LOC_Os12g08230|protein expressed protein e_value:3.85081e-69</td><td>0.174</td><td>(0.1043 0.6003)</td><td>0.1043</td><td>0.6003</td></tr>
<tr><td>3462</td><td>WT002_D19_CLUSTER_32055</td><td>gi|50919437|ref|XP_470115.1| putative multiple inositol polyphosphate phosphatase [Oryza sativa (japonica cultivar-group)] >gnl|BL_ORD_ID|1588189 gi|28876004|gb|AAO60013.1| putative multiple inositol polyphosphate phosphatase [Oryza sativa (japonica cu</td><td>11669.m06138|LOC_Os03g60370|protein putative multiple inositol polyphosphate phosphatase e_value:0</td><td>0.188</td><td>(0.0899 0.4777)</td><td>0.0899</td><td>0.4777</td></tr>
<tr><td>3463</td><td>SET2_E23_CLUSTER_13866</td><td>gi|34910450|ref|NP_916572.1| lysine decarboxylase-like protein [Oryza sativa (japonica cultivar-group)] >gi|20804658|dbj|BAB92347.1| lysine decarboxylase-like [Oryza sativa (japonica cultivar-group)] e_value:7.6643e-108</td><td>11667.m05001|LOC_Os01g51210|protein Possible lysine decarboxylase, putative e_value:1.47772e-109</td><td>0.088</td><td>(0.0647 0.7368)</td><td>0.0647</td><td>0.7368</td></tr>
<tr><td>3464</td><td>WT009_P07_CLUSTER_5773</td><td>gi|30023738|gb|AAP13402.1| At3g26710 [Arabidopsis thaliana] >gnl|BL_ORD_ID|1221658 gi|21592738|gb|AAM64687.1| unknown [Arabidopsis thaliana] >gnl|BL_ORD_ID|1221658 gi|22655152|gb|AAM98166.1| expressed protein [Arabidopsis thaliana] >gnl|BL_ORD_ID</td><td>11687.m02973|LOC_Os11g32320|protein At3g26710 e_value:1.55676e-102</td><td>0.162</td><td>(0.1134 0.7016)</td><td>0.1134</td><td>0.7016</td></tr>
<tr><td>3465</td><td>WT003_G17_CLUSTER_33361</td><td>gi|31433652|gb|AAP55136.1| unknown protein [Oryza sativa (japonica cultivar-group)] >gnl|BL_ORD_ID|2013159 gi|37537094|ref|NP_922849.1| unknown protein [Oryza sativa (japonica cultivar-group)] >gnl|BL_ORD_ID|2013159 gi|12643061|gb|AAK00450.1| unknow</td><td>11676.m03923|LOC_Os10g42420|protein expressed protein e_value:0</td><td>0.157</td><td>(0.0763 0.4853)</td><td>0.0763</td><td>0.4853</td></tr>
<tr><td>3466</td><td>WT010_A04_CLUSTER_9043</td><td>gi|29126338|gb|AAO66530.1| putative p21 C-terminal-binding protein (alternative splicing products) [Oryza sativa (japonica cultivar-group)] >gnl|BL_ORD_ID|2058548 gi|50920169|ref|XP_470445.1| putative p21 C-terminal-binding protein [Oryza sativa (japon</td><td>11669.m06531|LOC_Os03g63690|protein putative p21 C-terminal-binding protein (alternative splicing products)</td><td>0.199</td><td>(0.1349 0.6789)</td><td>0.1349</td><td>0.6789</td></tr>
<tr><td>3467</td><td>SET1_D17_CLUSTER_7824</td><td>gi|50906385|ref|XP_464681.1| putative senescence-associated protein [Oryza sativa (japonica cultivar-group)] >gi|46805859|dbj|BAD17193.1| putative senescence-associated protein [Oryza sativa (japonica cultivar-group)] e_value:1.66082e-137</td><td>11668.m01171|LOC_Os02g12750|protein probable senescence-associated protein 5 [imported] - Arabidopsis thaliana e_value:3.89605e-139</td><td>0.239</td><td>(0.0688 0.2875)</td><td>0.0688</td><td>0.2875</td></tr>
<tr><td>3468</td><td>WT011_J18_CLUSTER_7612</td><td>gi|55296998|dbj|BAD68473.1| putative root cap-specific protein [Oryza sativa (japonica cultivar-group)] >gnl|BL_ORD_ID|269231 gi|55296457|dbj|BAD68653.1| putative root cap-specific protein [Oryza sativa (japonica cultivar-group)] e_value:0</td><td>11680.m00383|LOC_Os06g04620|protein GDP-mannose 4,6-dehydratase e_value:0</td><td>0.163</td><td>(0.0502 0.3077)</td><td>0.0502</td><td>0.3077</td></tr>
<tr><td>3469</td><td>WT011_B14_CLUSTER_32498</td><td>gi|34902824|ref|NP_912759.1| unnamed protein product [Oryza sativa (japonica cultivar-group)] e_value:0</td><td>11667.m01485|LOC_Os01g14920|protein Transposable element protein, putative e_value:0</td><td>0.248</td><td>(0.1104 0.4455)</td><td>0.1104</td><td>0.4455</td></tr>
<tr><td>3470</td><td>WT005_B01_CLUSTER_24376</td><td>gi|34895400|ref|NP_909043.1| P0445D12.7 [Oryza sativa (japonica cultivar-group)] e_value:1.32498e-39</td><td>11667.m00230|LOC_Os01g03180|protein hypothetical protein e_value:3.37209e-41</td><td>0.575</td><td>(0.7320 1.2729)</td><td>0.732</td><td>1.2729</td></tr>
<tr><td>3471</td><td>WT004_A06_CLUSTER_3021</td><td>gi|50904417|ref|XP_463697.1| putative hexokinase [Oryza sativa (japonica cultivar-group)] e_value:0</td><td>11682.m02887|LOC_Os05g31110|protein hypothetical protein e_value:1.16343e-173</td><td>0.26</td><td>(0.2250 0.8644)</td><td>0.225</td><td>0.8644</td></tr>
<tr><td>3472</td><td>WT005_I19_CLUSTER_18089</td><td>gi|62732927|gb|AAX95046.1| expressed protein [Oryza sativa (japonica cultivar-group)] e_value:5.04878e-64</td><td>11687.m00939|LOC_Os11g09940|protein hypothetical protein e_value:4.63654e-65</td><td>0.734</td><td>(0.8845 1.2044)</td><td>0.8845</td><td>1.2044</td></tr>
<tr><td>3473</td><td>SET3_I01_CLUSTER_27125</td><td>gi|29126350|gb|AAO66542.1| expressed protein [Oryza sativa (japonica cultivar-group)] >gi|50920179|ref|XP_470450.1| unknown protein [Oryza sativa (japonica cultivar-group)] e_value:9.18375e-179</td><td>11669.m06537|LOC_Os03g63730|protein expressed protein e_value:2.54142e-180</td><td>0.206</td><td>(0.1514 0.7368)</td><td>0.1514</td><td>0.7368</td></tr>
<tr><td>3474</td><td>WT009_H13_CLUSTER_8160</td><td>gi|34899382|ref|NP_911037.1| unknown protein [Oryza sativa (japonica cultivar-group)] >gnl|BL_ORD_ID|1518572 gi|51963242|ref|XP_506138.1| PREDICTED P0585H11.111 gene product [Oryza sativa (japonica cultivar-group)] >gnl|BL_ORD_ID|1518572 gi|23617055</td><td>11673.m00075|LOC_Os07g01720|protein expressed protein e_value:2.53316e-145</td><td>0.517</td><td>(0.2893 0.5598)</td><td>0.2893</td><td>0.5598</td></tr>
<tr><td>3475</td><td>SET2_K02_CLUSTER_3174</td><td>gi|56783935|dbj|BAD81372.1| putative histidinol dehydrogenase precursor, chloroplast [Oryza sativa (japonica cultivar-group)] e_value:0</td><td>11667.m01297|LOC_Os01g13190|protein histidinol dehydrogenase e_value:0</td><td>0.052</td><td>(0.0300 0.5741)</td><td>0.03</td><td>0.5741</td></tr>
<tr><td>3476</td><td>WT006_G13_CLUSTER_1296</td><td>gi|38345389|emb|CAE03115.2| OSJNBa0067K08.18 [Oryza sativa (japonica cultivar-group)] >gnl|BL_ORD_ID|1522179 gi|50925987|ref|XP_473041.1| OSJNBa0067K08.18 [Oryza sativa (japonica cultivar-group)] e_value:2.85339e-171</td><td>11670.m04041|LOC_Os04g42010|protein La domain, putative e_value:9.12915e-173</td><td>0.294</td><td>(0.1585 0.5398)</td><td>0.1585</td><td>0.5398</td></tr>
<tr><td>3477</td><td>WT004_C19_CLUSTER_32265</td><td>gi|50922337|ref|XP_471529.1| OSJNBa0071G03.1 [Oryza sativa (japonica cultivar-group)] >gnl|BL_ORD_ID|1590258 gi|38346597|emb|CAD39788.2| OSJNBa0071G03.1 [Oryza sativa (japonica cultivar-group)] >gnl|BL_ORD_ID|1590258 gi|38344646|emb|CAE05079.2| OSJN</td><td>11670.m01963|LOC_Os04g21320|protein plant integral membrane protein TIGR01569, putative e_value:4.41952e-91</td><td>0.202</td><td>(0.0723 0.3583)</td><td>0.0723</td><td>0.3583</td></tr>
<tr><td>3478</td><td>WT003_M24_CLUSTER_11323</td><td>gi|34906372|ref|NP_914533.1| unnamed protein product [Oryza sativa (japonica cultivar-group)] >gnl|BL_ORD_ID|1752023 gi|9711835|dbj|BAB07934.1| NADP-dependent malic enzyme [Oryza sativa (japonica cultivar-group)] >gnl|BL_ORD_ID|1752023 gi|9558508|db</td><td>11667.m00888|LOC_Os01g09320|protein Malic enzyme, N-terminal domain, putative e_value:0</td><td>0.122</td><td>(0.0733 0.6014)</td><td>0.0733</td><td>0.6014</td></tr>
<tr><td>3479</td><td>WT002_A15_CLUSTER_7634</td><td>gi|38345795|emb|CAE03567.2| OSJNBa0085I10.12 [Oryza sativa (japonica cultivar-group)] >gnl|BL_ORD_ID|1522683 gi|50928645|ref|XP_473850.1| OSJNBa0085I10.12 [Oryza sativa (japonica cultivar-group)] e_value:4.09131e-83</td><td>11670.m05099|LOC_Os04g52110|protein Late embryogenesis abundant protein, putative e_value:1.23039e-84</td><td>0.4</td><td>(0.2005 0.5019)</td><td>0.2005</td><td>0.5019</td></tr>
<tr><td>3480</td><td>SET5_O13_CLUSTER_34872</td><td>gi|25989705|gb|AAN74637.1| LEA1 protein [Triticum aestivum] e_value:2.65914e-59</td><td>11682.m04475|LOC_Os05g46480|protein LEA protein - rice e_value:1.337e-61</td><td>0.491</td><td>(0.2023 0.4119)</td><td>0.2023</td><td>0.4119</td></tr>
<tr><td>3481</td><td>WT011_J07_CLUSTER_31627</td><td>gi|50252531|dbj|BAD28706.1| putative tRNA splicing protein [Oryza sativa (japonica cultivar-group)] e_value:0</td><td>11681.m01534|LOC_Os09g16910|protein cysteine desulfurase, mitochondrial precursor (ec 2.8.1.7) e_value:0</td><td>0.122</td><td>(0.0487 0.4002)</td><td>0.0487</td><td>0.4002</td></tr>
<tr><td>3482</td><td>SET1_F15_CLUSTER_11793</td><td>gi|34903232|ref|NP_912963.1| unnamed protein product [Oryza sativa (japonica cultivar-group)] >gi|6815105|dbj|BAA90391.1| hypothetical protein [Oryza sativa (japonica cultivar-group)] e_value:4.06546e-31</td><td>11667.m01672|LOC_Os01g16640|protein expressed protein e_value:2.75391e-37</td><td>0.039</td><td>(0.0273 0.7008)</td><td>0.0273</td><td>0.7008</td></tr>
<tr><td>3483</td><td>WT004_G15_CLUSTER_8641</td><td>gi|50932867|ref|XP_475961.1| putative late embryogenesis abundant protein [Oryza sativa (japonica cultivar-group)] >gnl|BL_ORD_ID|1599529 gi|48843797|gb|AAT47056.1| 'putative late embryogenesis abundant protein, LEA14-A' [Oryza sativa (japoni</td><td>11682.m04884|LOC_Os05g50710|protein Late embryogenesis abundant protein e_value:4.47367e-65</td><td>0.864</td><td>(0.1833 0.2122)</td><td>0.1833</td><td>0.2122</td></tr>
<tr><td>3484</td><td>WT003_I12_CLUSTER_2488</td><td>gi|51535466|dbj|BAD37363.1| unknown protein [Oryza sativa (japonica cultivar-group)] e_value:2.39276e-98</td><td>11680.m04275|LOC_Os06g43550|protein expressed protein e_value:5.11297e-100</td><td>0.234</td><td>(0.1678 0.7182)</td><td>0.1678</td><td>0.7182</td></tr>
<tr><td>3485</td><td>WT010_B15_CLUSTER_15923</td><td>gi|51090886|dbj|BAD35459.1| putative mitochondrial energy transfer protein [Oryza sativa (japonica cultivar-group)] e_value:0</td><td>11680.m03905|LOC_Os06g40050|protein brittle-1 protein, chloroplast precursor e_value:0</td><td>0.095</td><td>(0.0608 0.6396)</td><td>0.0608</td><td>0.6396</td></tr>
<tr><td>3486</td><td>SET5_F10_CLUSTER_25967</td><td>gi|20908090|gb|AAM26728.1| AT4g01940/T7B11_20 [Arabidopsis thaliana] >gi|15215670|gb|AAK91380.1| AT4g01940/T7B11_20 [Arabidopsis thaliana] >gi|18411785|ref|NP_567219.1| nitrogen fixation NifU-like family protein [Arabidopsis thaliana] >gi|1451743</td><td>11669.m02039|LOC_Os03g20010|protein Similar to NFU1 protein e_value:2.64549e-92</td><td>0.331</td><td>(0.1244 0.3760)</td><td>0.1244</td><td>0.376</td></tr>
<tr><td>3487</td><td>SET5_H01_CLUSTER_18533</td><td>gi|34914854|ref|NP_918774.1| P0416G11.10 [Oryza sativa (japonica cultivar-group)] >gi|13365982|dbj|BAB39260.1| NPL4 -like protein [Oryza sativa (japonica cultivar-group)] e_value:1.62628e-108</td><td>11667.m00071|LOC_Os01g01680|protein expressed protein e_value:9.38269e-36</td><td>0.619</td><td>(0.4663 0.7531)</td><td>0.4663</td><td>0.7531</td></tr>
</table>
