# Supplementary material for: Assessment of adaptive evolution between wheat and rice as deduced from full-length common wheat cDNA sequence data and expression patterns
Source: BMC Genomics. 2009 Jun 18;10:271. doi: 10.1186/1471-2164-10-271 (PMC2703658; doi:10.1186/1471-2164-10-271)
Supplement: Additional file 2 — Adaptational genes showing relatively faster evolution during diversification between wheat and rice. Adaptational genes showing relatively faster evolution are listed and their annotation is given with their Ka/Ks values. [file 1471-2164-10-271-S2.doc]

# Additional file 2

**Adaptational genes showing relatively faster evolution during diversification between wheat and rice**

Genes showing a *Ka/Ks* value higher than 0.5 are listed in the table.

| Tissue  (No. annotated genes/Total No. genes) | Gene function | Annotated gene | Expected function | *Ka* | *Ks* | *Ka/Ks* | Reference |
| --- | --- | --- | --- | --- | --- | --- | --- |
| Root  (6/16) | Transcription factor | C3HC4 zinc RING finger | Up-regulated in response to Cd stress. | 0.438 | 0.546 | 0.803 | (1) |
|  |  | WRKY | Constitutive expression of WRKY enhances disease resistance, and affects root growth. | 0.291 | 0.568 | 0.512 | (2) |
|  | Polysaccharide  -related | β-glucanase | Regulated by plant hormones, wounding, salicylic acid and fungal elicitors. | 0.199 | 0.371 | 0.535 | (3) |
|  | REDOX | Uclacyanin | Plant-specific mononuclear blue copper protein and involved in the plant defense. | 0.180 | 0.359 | 0.501 | (4) |
|  | ROS | Peroxydase (2) | PO improves tolerance against multiple stresses including salt and rice blast fungus. | 0.179 | 0.242 | 0.739 | (5), (6) |
|  | Nodulin | MtN21 | MtN21 control bacterial leaf blight resistance. | 0.210 | 0.383 | 0.549 | (7) |
| Spike at booting stage  (4/12) | Lipid related | Stearoyl-acyl-carrier protein desaturase | Protein(s) are actively modulated in the developing seeds. | 0.301 | 0.508 | 0.593 | (8) |
|  |  | GDSL-like lipase/acylhydrolase | Protein is active in the early stages of seed development. | 0.158 | 0.300 | 0.527 | (9) |
|  | REDOX in photosynthesis | CP12 | Protein assembles photosynthetic supramolecular complexes. | 0.156 | 0.285 | 0.547 | (10) |
|  | REDOX in respiration | Cytochrome oxidase B5 | High level of expression for COB5 gene is found in embryogenesis and male and female reproductive organs. | 0.700 | 1.076 | 0.651 | (11) |
| Spike at flowering  (8/15) | Nucleic acid binding protein | RNA binding protein | RBP has a role in flower and seed development. | 0.503 | 0.483 | 1.042 | (12) |
|  |  | Chloroplast nucleoid DNA binding protein | Histone-like protein that primarily organizes chloroplast DNA into nucleoid. | 0.282 | 0.498 | 0.565 | (13) |
|  | Pollen allergen | Pollen allergen related protein | Pollen allergen-encoding genes are expressed in pollen and ovaries. | 0.712 | 1.00 | 0.712 | (14), (15) |
|  |  |  | Pollen allergen Lol p IIA in perennial ryegrass. | 0.558 | 0.873 | 0.640 |  |
|  | Methylation | O-methyltransferase | OMT is induced in the microspore by cold treatment. | 0.361 | 0.506 | 0.712 | (16) |
|  | ROS | Glutathione S-transferase | GST is expressed in response to aluminum stress. | 0.312 | 0.512 | 0.608 | (17) |
|  | Polysaccharide  -related | Pectate lyase | PL is expressed under the control of developmental stage, hormonal and stress regulation. | 0.172 | 0.317 | 0.542 | (18) |
|  |  | Glucan endo-1,3-beta-D-glucosidase | GEBG is induced by stress. | 0.328 | 0.641 | 0.512 | (19) |
|  | Osmotic pressure-related | Osmotin | Osmotin gene is induced by abiotic stress and affects apotosis and cytoskeleton. | 0.251 | 0.422 | 0.595 | (20), (21) |
|  | Signal transduction | Receptor-like protein kinase | RPK plays a role during ovule development. | 0.180 | 0.306 | 0.589 | (22) |
|  | Proteinase  Inhibitor | Proteinase inhibitor | PI is expressed in the seed development. | 0.264 | 0.430 | 0.615 | (23) |
| Seed at DPA30**  (10/16) | REDOX | Thionin 1 | TH genes are specifically expressed at the seed and control defense against FHB fungi. | 0.642 | 0.578 | 1.11 | (24) |
|  |  | Thionin 2 |  | 0.657 | 0.803 | 0.817 |  |
|  | Polysaccharide-  related | Xylanase inhibitor TAXI-IV | XI-TAXI is induced by pathogens and wounding. | 0.350 | 0.441 | 0.795 | (25) |
|  |  | UDP-glucosyltransferase | UDP-GT is induced by pathogens and wounding, and detoxified mycotoxin of FHB. | 0.254 | 0.442 | 0.573 | (26), (27) |
|  | Lipid-related | Lipid transfer | LT operates on plant defense. | 0.352 | 0.673 | 0.522 | (28) |
|  | Stress response-related during seed maturation | Late embryo abundant protein I | LEAs are specifically expressed at the seed and up-regulated in the dry seed. | 0.183 | 0.212 | 0.864 | (29) |
|  |  | Late embryo abundant protein II |  | 0.297 | 0.451 | 0.658 |  |
|  |  | Stress-related protein | - | 0.182 | 0.325 | 0.559 | (30) |
|  | Hydrolase | Alpha-amylase | Amylase hydrolyzes starch in the seed. | 0.248 | 0.408 | 0.607 | (31) |
|  | Plasma membrane | Ankyrins | Ankyrins are components of plasma membrane and act for plant defence against stresses. | 0.338 | 0.572 | 0.591 | (32) |
| Seedling  with cold treatment | Lipid-related | Lipid transfer | LT operates on plant defense. | 0.264 | 0.444 | 0.596 | (33) |
| (4/9) | Polysaccharide  -related | Glucan endo-1,3-beta-D-glucosidase | GEBG is induced by stress. | 0.184 | 0.332 | 0.554 | (34) |
|  | Flavonoid synthesis | Chalcone isomerase | CHI is found in the nodules of *E. umbellata*. | 0.876 | 1.669 | 0.525 | (35) |
|  | Abiotic stress | Metallothionein | MT is induced by low temperature and water. deficit. | 0.200 | 0.393 | 0.509 | (36) |
| Desiccated seedling | Chaperon | DnaJ | DnaJ is expressed in a tissue-specific manner. | 0.252 | 0.473 | 0.532 | (37) |
| (4/9) | Stress-related | PrMC3 | PrMC3 is induced by cold treatment of the rice root. | 0.364 | 0.635 | 0.574 | (38) |
|  | Photosystem II | PsbQ | PsbQ is a member of PSII. | 0.272 | 0.484 | 0.562 | (39) |
|  | Ribosome-related | RALyase | RALyase recovers damaged ribosomes. | 0.302 | 0.474 | 0.637 | (40) |

Number of genes grouped into the category is given in parentheses.

** DPA: Days post anthesis.

**References**

Additional references presented in Additional file 1 are listed.

1. Minglin L, Yuxiu Z, Tuanyao C: **Identification of genes up-regulated in response to Cd exposure in *Brassica juncea* L**. *Gene* 2005, **363**:151-158.
2. Zhang Y, Shih DS: **Isolation of an osmotin-like protein gene from strawberry and analysis of the response of this gene to abiotic stresses.** *J Plant Physiol* 2007, **164**:68-77.
3. Simmons CR, Litts JC, Huang N, Rodriguez RL: **Structure of a rice beta-glucanase gene regulated by ethylene, cytokinin, wounding, salicylic acid and fungal elicitors.** *Plnat Mol Biol* 1992, **18**:33-45.
4. Nersissian AM, Immoos C, Hill MG, Hart PJ, Williams G, Herrmann RG, Valentine JS: **Uclacyanins, and plant cyanins are distinct subfamilies of phytocyanins: Plant-specific mononuclear blue copper proteins.** *Protein Sci* 1998, **7**:1915-1929.
5. Sasaki K, Iwai T, Hiraga S, Kuroda K, Seo S, Mitsuhara I, Miyasaka A, Iwano M, Ito H, Matsui H, Ohashi Y: **Ten rice peroxidases redundantly respond to multiple stresses including infection with rice blast fungus.** *Plant Cell Physiol* 2004, **45**:1442-1452.
6. Lu Z, Liu D, Liu S: **Two rice cytosolic ascorbate peroxydases differentially improve salt tolerance in transgenic Arabidopsis.** *Plant Cell Rep* 2007, **26**:1909-1917.
7. Kottapalli KR, Kottapalli P, Agrawal GK, Kikuchi S, Rakwal R: **Recessive bacterial leaf blight resistance in rice: complexity, challenges and strategy.** *Biochem Biophys Res Commun* 2007, **355**:295-301.
8. Salas JJ, Youssar L, Martinez-Force E, Garces R: **The biochemical characterization of a high-stearic acid sunflower mutant reveals the coordinated regulation of stearoyl-acyl carrier protein desaturases.** *Plant Physiol Biochem* 2008, **46**:109-116.
9. Clauss K, Baumert A, Nimtz M, Milkowski C, Strack D: **Role of a GDSL lipase-like protein as sinapine esterase in Brassicaceae.** *Plant J* 2008, **53**:802-813.
10. Marri L, Trost P, Trivelli X, Gonnelli L, Pupillo P, Sparla F: **Spontaneous assembly of photosynthetic supramolecular complexes as mediated by the intrinsically unstructured protein CP12.** *J Biol Chem* 2008, **283**:1831-1838.
11. Martsinkovskaya AI, Poghosyan ZP, Haralampidis K, Murphy DJ, Hatzopoulos P: **Temporal and spatial gene expression of cytochrome B5 during flower and fruit development in olives.** *Plant Mol Biol* 1999, **40**:79-90.
12. Fusaro AF, Bocca SN, Ramos RL, Barroco RM, Magioli C, Jorge, VC, Couthinho TC, Rangel-Lima CM, De Rycke R, Inze D, Engler G, Sachetto-Martins G: **AtGRP2, a cold-induced nucleo-cytoplasmic RNA-binding protein, has a role in flower and seed development.** *Planta* 2007, **225**:1339-1351.
13. Kobayashi T, Takahara M, Miyagishima SY, Kuroiwa H, Sasaki N, Ohta N, Matsuzaki M, Kuroiwa T: **Detection and localization of a chloroplast-encoded HU-like protein that organizes chloroplast nucleoids.** *Plant Cell* 2002, **14**:1579-1589.
14. Balzer HJ, Borisiuk L, Meyer HM, Matzk F, Baeumlein H: **A pollen allergen-encoding gene is expressed in wheat ovaries.** Plant Mol Biol 1996, **32**:435-445.
15. Jiang SY, Jasmin PX, Ting YY, Ramachandran S: **Genome-wide identification and molecular characterization of Ole_e_I, Allerg_1 and Allerg_2 domain-containing pollen-allergen-like genes in *Oryza sativa*.** *DNA Res* 2005, **12**:167-179.
16. Imin N, Kerim T, Weinman JJ, Rolfe BG: **Low temperature treatment at the young microspore stage induces protein changes in rice anther.** *Mol Cell Proteomics* 2006, **5**:274-292.
17. Ezaki B, Suzauki M, Motoda H, Kawamura M, Nakashima S, Matsumoto H: **Mechanism of gene expression of Arabidopsis glutathione S-transferase, AtGST1, and ATGST11 in response to aluminum stress.** *Plant Physiol* 2004, **134**:1672-1682.
18. Palusa SG, Golovkin M, Shin SB, Richardson DN, Reddy AS: **Organ-specific, developmental, hormonal and stress regulation of expression of putative pectate lyase genes in Arabidopsis.** *New Phytol* 2007, **174**:537-550.
19. Opassiri R, Pomthong B, Akiyama T, Nakphaichit M, Onkoksoong T, Ketudata Cairns M, Ketudat Cains Jr: A stress-induced rice (Oryza sativa L.) beta-glucosidase represents a new subfamily of glycosyl hydrolase family 5 containing a fascin-like domain. *Biochem J* 2007, **408**:241-249.
20. D’Angeli S, Altamura MM: **Osmotin induces cold protection in olive trees by affecting programmed cell death and cytoskeleton organization.** *Planta* 2007, **225**:1147-1163.
21. Zhang Y, Shih DS: **Isolation of an osmotin-like protein gene from strawberry and analysis of the response of this gene to abiotic stresses.** *J Plant Physiol* 2007, **164**:68-77.
22. Pillitteri LJ, Bermis SM, Shpak ED, Torii KU: **Haploinsufficiency after successive loss of signaling reveals a role for *ERECTA*-family gene in Arabidopsis ovule development.** *Development* 2007, **134**:3099-3109.
23. Arai S, Matsumoto I, Emori Y, Abe K: **Plant seed cystatins and their target enzymes of endonenous and exogenous origin.** *J Agric Food Chem* 2002, **50**:6612-6617.
24. Mackintosh CA, Lewis J, Radmer LE, Shin S, Heinen SJ, Smith LA, Wyckoff MN, Dill-Macky R, Evans CK, Kravchenko S, Baldridge GD, Zeyen RJ, Muehlbauer GJ: Overexpression of defense response genes in transgenic wheat enhances resistance to Fusarium head blight. Plant Cell Rep 2007, **26**:479-488.
25. Igawa T, Ochiai-Fukuda T, Takahashi-Ando N, Ohsato S, Shibata T, Yamaguchi I, Kimura M: **New TAXI-type xylanase inhibitor genes are inducible by pathogens and wounding in hexaploid wheat.** *Plant Cell Physiol* 2004, **45**:1347-1360.
26. Poppenberger B, Berthiller F, Lucyshyn D, Sieberer T, Schuhmacher R, Krska R, Kuchlerk, Goessl J, Luschnig C, Adam G: **Detoxification of the Fusarium mycotoxin deoxynivalenol by a UDP-glucosyltransferase from *Arabidopsis thaliana*.** *J Biol Chem* 2003, **278**:47905-47914.
27. Sepulveda-Jimenez G, Rueda-Benitez P, Porta H, Rocha-Sosa M: **UDP-glucosyl transferase gene induced by wounding, bacterial infiltration and oxidative stress.** *J Exp Bot* 2005, **56**:605-611.
28. Cheng CS, Samuel D, Liu YJ, Shyu JC, Lai SM, Liu KF, Lyu PC: **Binding mechanism of non specific lipid transfer proteins and their role in plant defense.** *Biochemistry* 2004, **43**:13628-13636.
29. Delsery M, Bies-Etheve N, Carles C, Hull G, Vicient C, Raynal M, Grellet F, Aspart L (2001) **Late embryogenesis abundant (LEA) protein gene regulation during Arabidopsis seed maturation.** *J Plant Physiol* 2001, **158**:419-427
30. Tai HH, Tai GCC, Beardmore T: **Dynamic histone acetylation of late embryonic genes during seed germination.** *Plant Mol Biol* 2005, **59:**909-925
31. Mrva K, Wallwork M, Mares DJ: **alpha-Amylase and programmed cell death in aleurone of ripening wheat grains.** *J Exp Bo*t 2006, **57**:877-885
32. Lu H, Liu Y, Greenberg JT: **Structure-function analysis of the plasma membrane-localized Arabidopsis defense component ACD6.** *Plant J* 2005 44:798-809
33. Cheng CS, Samuel D, Liu YJ, Shyu JC, Lai SM, Liu KF, Lyu PC: **Binding mechanism of non specific lipid transfer proteins and their role in plant defense.** *Biochemistry* 2004, **43**:13628-13636
34. Opassiri R, Pomthong B, Akiyama T, Nakphaichit M, Onkoksoong T, Ketudata Cairns M, Ketudat Cains Jr: **A stress-induced rice (Oryza sativa L.) beta-glucosidase represents a new subfamily of glycosyl hydrolase family 5 containing a fascin-like domain**. 2007, *Biochem J* **408**:241-249
35. Kim HB, Bae JH, Lim JD, Yu CY, An CS: **Expression of a functional type-I chalcone isomerase gene is localized to the infected cells of root nodules of *Elaeagnus umbellate*.** *Mol Cells* 2007, **23**:405-409
36. Wisniewski M, Bassett C, Norelli J, Macarisin D, Artlip T, Gasic K, Korbar S: **Expressed sequence tag analysis of the response of apple (Malus x domestica ‘Royal Gala’) to low temperature and water deficit.** *Physiol Plant* 2008, **133**:298-317
37. Frugis G, Mele G, Giannino D, Mariotti D: **MsJ1, an alfalfa DnaJ-like gene, is tissue specific and transcriptionally regulated during cell cycle.** *Plant Mol Biol* 1999, **40**:397-408
38. Lee D-G, Ahsan N, Lee S-H, Lee JJ, Bahk JD, Kang KY, Lee B-H: **Chilling stress-induced proteomic changes in rice root.** *J Plant Physiol* 2008, E-pub ahead. Doi:10.1016/j.physletb 2003.10.071
39. Balsera M, Menendez M, Saiz JL, de Las Rivas J, Andreu JM, Arellano JB: **Structural stability of the PsbQ protein of higher plant photosystem II.** *Biochemistry* 2004, **43**: 14171-14179
40. Sawasaki T, Nishihara M, Endo Y: **RIP and RALyase cleave the sarcin/ricin domain, a critical domain for ribosome function, during senescence of wheat coleoptiles.** *Biochem Biophys Res Commun* 2008, **370**: 561-565
